# Mining protein networks for synthetic genetic interactions

**DOI:** 10.1186/1471-2105-9-426

**Published:** 2008-10-09

**Authors:** Sri R Paladugu, Shan Zhao, Animesh Ray, Alpan Raval

**Affiliations:** 1 Keck Graduate Institute of Applied Life Sciences, 535 Watson Drive, Claremont, CA 91711, USA; 2 Virtual Endoscopy and Computer-Aided Diagnosis Laboratory, Department of Radiology, National Institutes of Health Clinical Center, Bethesda, MD 20892-1182, USA; 3 School of Mathematical Sciences, Claremont Graduate University, 710 N. College Avenue, Claremont, CA 91711, USA

## Abstract

**Background:**

The local connectivity and global position of a protein in a protein interaction network are known to correlate with some of its functional properties, including its essentiality or dispensability. It is therefore of interest to extend this observation and examine whether network properties of two proteins considered simultaneously can determine their joint dispensability, i.e., their propensity for synthetic sick/lethal interaction. Accordingly, we examine the predictive power of protein interaction networks for synthetic genetic interaction in *Saccharomyces cerevisiae*, an organism in which high confidence protein interaction networks are available and synthetic sick/lethal gene pairs have been extensively identified.

**Results:**

We design a support vector machine system that uses graph-theoretic properties of two proteins in a protein interaction network as input features for prediction of synthetic sick/lethal interactions. The system is trained on interacting and non-interacting gene pairs culled from large scale genetic screens as well as literature-curated data. We find that the method is capable of predicting synthetic genetic interactions with sensitivity and specificity both exceeding 85%. We further find that the prediction performance is reasonably robust with respect to errors in the protein interaction network and with respect to changes in the features of test datasets. Using the prediction system, we carried out novel predictions of synthetic sick/lethal gene pairs at a genome-wide scale. These pairs appear to have functional properties that are similar to those that characterize the known synthetic lethal gene pairs.

**Conclusion:**

Our analysis shows that protein interaction networks can be used to predict synthetic lethal interactions with accuracies on par with or exceeding that of other computational methods that use a variety of input features, including functional annotations. This indicates that protein interaction networks could plausibly be rich sources of information about epistatic effects among genes.

## Background

Successful prediction of gene function from disparate data sources is an important challenge in the post-genomic era. Methods to do so can illuminate new mechanisms for the emergence and organization of function at the genome level, and lead to the understanding of disease mechanisms or prediction of drug targets. Functional organization of genes is often dramatically revealed by their positions in biomolecular networks and the topological constraints that these positions entail. Much work has been done on using graph properties of protein interaction networks (PINs) to elucidate gene and protein function, particularly in the baker's yeast *Saccharomyces cerevisiae *for which high quality genome-scale interaction maps (or graphs) can be constructed. In these interaction graphs, nodes represent individual proteins and edges represent their physical binding. Several previous reports find interesting correlations between network properties and aspects of biological function [1-10]. For example, pairwise correlations have been found between protein degree/centrality, gene essentiality, and evolutionary rate [1-4]. Other methods attempt to uncover sets of genes implicated in a common function, i.e., functional modules, from network structure [5-9]. Yet other methods combine graph-theoretic data with other data sources, such as expression levels [11] or functional annotation of neighboring proteins in the interaction map [12-16], to further elucidate function.

One of the earliest observed correlations between a graph-theoretic property and a functional attribute is the well-known correlation between the degree (i.e., the number of interaction partners or "hubness") of a protein in a yeast PIN and the essentiality of the corresponding protein-coding gene [1,17] (an essential gene is one that produces a lethal phenotype when deleted). Correlations between essentiality and other graph properties, including various centrality measures, have also been reported in yeast [2-4] and other organisms such as the nematode worm *Caenorhabditis elegans *and fruit fly *Drosophila melanogaster *[4]. The principal observation emerging from these studies is that essential proteins tend to be over-represented among proteins with high degrees and high centralities in a PIN. This leads to the hypothesis that the indispensability of a given protein for cellular function is at least partially determined by both its local connectivity (quantified by degree) and its global position (quantified by other centrality measures) in a PIN [2,1].

Synthetic lethal interactions are important genetic interactions for understanding biological function and for potentially developing novel classes of drug targets [18]. Two genes are said to participate in a synthetic lethal interaction if simultaneous deletion mutations in both, but not in any one alone, cause lethality. Thus, the concept of synthetic lethality may be viewed as an extension of essentiality. Indeed, it has been recently proposed that the type of genetic redundancy conferred by synthetic lethality leads to redundancy being more prevalent among proteins that are highly connected and highly central in PINs [19]. This finding implies correlations between PIN graph properties and synthetic lethality similar to the correlations with essentiality of single genes. In fact, such correlations have also been reported earlier. Tong et.al. [20] observed that synthetic genetic interactions, including synthetic lethal (SL) and synthetic sick (SS, where simultaneous deletion of two genes causes growth retardation) interactions, are more prevalent between genes encoding proteins within the same protein complex (two or more proteins that form a clique) than between those encoding proteins across complexes or between proteins that are not part of any known complex. These authors also addressed the issue of using this correlation to predict protein-protein interactions from synthetic genetic interactions, and concluded that this predictive value is limited because few (approximately 1%) gene pairs encode proteins that are members of the same complex. They further find that the number of common neighbors between two genes in a synthetic genetic network correlates with the existence of a protein-protein interaction between the corresponding gene products. It is well understood that proteins encoded by genes having synthetic genetic interaction are enriched among proteins that lie in separate pathways as opposed to the same pathway (see, for example, [9,17]), thus further indicating a preference for protein network position among the products of synthetic gene pairs. In spite of these studies, it is an open question whether there is enough information in PINs alone for determining synthetic lethal interactions. The conceptual basis for this expectation is that all biological function is ultimately defined by the interaction of proteins with other proteins, DNA/RNA, or small molecules (metabolites). Since most protein-DNA/RNA or protein-metabolite interactions are directly or indirectly influenced by other protein-protein interactions, it is reasonable that the structure of the PIN might encode sufficient information for all other interaction networks. However, this encoding could be subtle and perhaps beyond simple linear statistical correlations envisaged in explicit analytical models.

Arguably the most systematic and accurate method to date for genome-wide prediction of synthetic sick or lethal (SSL) interactions was carried out by Wong et al. [21] in *Saccharomyces cerevisiae *using multiple input features, including protein interactions, gene expression, functional annotation, and sequence motifs. The method can be used to streamline the SSL discovery process wherein it would require one to experimentally test less than 20% of the pairs to discover SSL pairs with 80% success rate. But the method relies on disparate data sources (viz., expression data, subcellular localization, physical complexes) which are not readily available for organisms other than *S. cerevisiae*. Furthermore, the success of this method does not answer the question posed in the previous paragraph.

Here we study, in *S. cerevisiae*, the extent to which a SSL interaction between two genes can be predicted solely from the topological properties of the corresponding proteins in a PIN and from the knowledge of other SSL interactions that the genes in question participate in. Our motivation stems not only from the expectations described above, but also from the observation that the yeast protein interaction network formed by literature curated interactions [22], composed of ~3300 proteins and ~12000 interactions, is substantially larger than the size of the most recent synthetic genetic network formed by literature curated and high-throughput screen interactions (~1000 genes and ~7000 interactions [20,23,22]), thus potentially enabling the discovery of new SSL interactions. Strikingly, we find that the best overall accuracy for SSL prediction from protein interaction data is comparable or higher than that found via a combination of disparate inputs [21], thus confirming the predictive power of PINs for SSL interactions and opening up the possibility of predicting the SSL network for other organisms where genome-scale protein interaction networks have been found.

## Methods

### Protein Interaction Network (PIN) data

For computing network properties associated with protein-protein interactions in yeast, we used the literature curated protein interactions in *Saccharomyces cerevisiae *culled by Reguly et.al [22]. This network contains 3289 proteins and 11334 interactions.

### PIN graph-theoretic properties used for predicting SSLs

For use as inputs to a Support Vector Machine (SVM) classifier, we computed the following PIN graph-theoretic properties for each protein.

(a) Degree: the number of direct interactions with other proteins.

(b) Clustering coefficient [24]: the fraction of possible interactions among direct neighbors of a protein in the PIN.

(c) Closeness centrality [25]: Let *d*(*p*, *q*) be the shortest distance between proteins *p *and *q *in a PIN. Then the closeness centrality of protein *p *is defined as (*n *- 1)/Σ_*q *_*d*(*p*, *q*), where *n *is the total number of proteins in the PIN. It therefore measures the extent to which protein *p *is close to all other proteins in the PIN.

(d) Normalized betweenness centrality [26]: Let *σ*_*p*,*q *_be the number of shortest paths between proteins *p *and *q *in a PIN, and let *σ*_*p*,*q*_(*r*) be the number of shortest paths between *p *and *q *that pass through protein *r*. Then the betweenness centrality of *r *is defined as Σ*σ*_*p*,*q*_(*r*)/*σ*_*p*,*q*_, where the sum is taken over all distinct pairs *p *and *q*. We normalize this measure to lie between 0 and 1 by dividing the betweenness centrality by the total number of pairs in the network not including *r*: (*n *- 1)(*n *- 2), where *n *is the number of proteins in the PIN of interest. It essentially measures the fraction of network shortest paths that a given protein lies on.

(e) Eigenvector centrality [27]: Let *v *denote the (row or column) index of a particular protein in the adjacency matrix corresponding to a PIN. Then the eigenvector centrality of that protein is defined as the *v*^*th *^element of the principal eigenvector of the adjacency matrix. This principal eigenvector is normalized such that its largest entry is 1. This centrality is a measure for how well connected a protein is to other highly connected proteins in a network.

(f) Stress centrality: the absolute number of network shortest paths that pass through protein r.

(g) Bridging centrality [28]: The bridging centrality of a protein (*r*) is defined as the product of the betweenness centrality and the bridging coefficient of the protein. While the betweenness centrality measures the fraction of network shortest paths a given protein lies on, the bridging coefficient measures the extent to which a protein is lying between other densely connected proteins in a network. Let *d*_*r *_and *N*_*r *_represent the degree and the set of neighbors of a protein *r*. Then the bridging coefficient (BC) of the protein *r *is defined as

$$BC(r)=\frac{d_{r}^{-1}}{\sum_{i\in N_{r}}d_{i}^{-1}}.$$

(h) Information centrality [29]: Let **A **be the adjacency matrix of the PIN, **D **a diagonal matrix of the degrees of each protein, and **J **a matrix with all its elements equal to 1. Let **B **= **D **- **A **+ **J **and let **C **= **B**^-1^. This construction yields the information matrix **I **with elements *I*_*ij *_= (*C*_*ii *_+ *C*_*jj *_- *C*_*ij*_)^-1^. The information centrality *IC*(*i*) of protein *i *is then defined as a harmonic mean:

$$IC(i)=n{\left(\sum_{j}I_{ij}^{-1}\right)}^{-1}.$$

As recognized by [30], this measure essentially measures the mean lengths of paths ending at protein *i*. A similar interpretation was given by [31], who showed that information centrality is identical to current-flow closeness centrality.

(i) Current-flow betweenness centrality [31]: This centrality measure is a generalization of the standard betweenness centrality index that takes into account not just shortest paths but other paths as well. Its definition is obtained from the definition of betweenness centrality by replacing *σ*_*p*,*q*_(*r*)/*σ*_*p*,*q *_by *τ*_*p*,*q*_(*r*), the throughput through node *r *[31]. It is related to the distance traversed by a random walk along the network that ends at a particular protein node.

All of the above properties are properties of single nodes in a network. The first two are sensitive only to the local network structure around the node, while all the others are sensitive to the global network topology. Furthermore, the last two properties depend not only on shortest paths through the network, but on other paths as well.

In addition to the above 9 single-node properties, we also computed a set of two-node properties, namely, the inverse of shortest distance *d*(*p*, *q*) between proteins *p *and *q*, number of mutual neighbors between proteins *p *and *q*, and two indicator variables 2Hop S-S and 2Hop S-P, which exploit the fact that the known synthetic genetic network contains a large number of triangles [21]. 2Hop S-S takes a value 1 if the genes encoding the two proteins *p *and *q *share a synthetic lethal partner and 0 otherwise, whereas 2Hop S-P takes a value 1 if there exists a protein *r *such that *r *has physical interaction with protein *p *and the gene corresponding to protein *r *has a SSL interaction with protein *q *or vice versa. We computed properties (a)-(d) using the network analysis tool Pajek [32], (e) and (f) using the SNA package for the R statistical computing platform [33], (g) based on the formula given in [28], and (h) and (i) using our implementation of the algorithm given in [31].

### Synthetic genetic interaction data

Our primary data sources for training and testing the SVM classifier (described below) were the literature curated genetic interactions from [22] and large-scale genetic interaction screens of [20,23], filtered for SSL interactions. From these sources we extracted only those pairs of genes whose protein products were found in the literature curated protein interaction network of [22], resulting in a dataset of 4553 confirmed SSL pairs from [20,23], and 7020 pairs when combined with literature curated SSL interactions of [22]. We then excluded gene pairs whose protein products were localized to mitochondria – this was necessary because our initial results showed that it is difficult to distinguish synthetic sick mutants from yeast mutants where the slow growth is conferred by the absence of a single (as opposed to two) mitochondrial protein. This resulted in a dataset of 3962 pairs of confirmed SSL interactions from [20,23], and 6074 pairs of confirmed SSL interactions if we included pairs from the literature curated genetic interactions of [22]. In order to train the classifier, we also required a list of negatives, i.e., pairs of genes confirmed to be not partaking in a SSL interaction. We constructed non-SSL pairs by generating all pairwise combinations of the 227 baits used in the large scale genetic interaction screen of [20] with all other non-essential genes in yeast whose protein products were not localized to mitochondria and then removing from this dataset the SSL interactions confirmed by high throughput and other experimental methods. The resulting number of SSL pairs and non-SSL pairs that were obtained from literature curated and high throughput methods are presented in Table 1. It is important to bear in mind that some of the inferred non-SSL pairs may well be SSL because of errors in the high throughput screen. This explains the decrease in the number of non-SSL pairs upon inclusion of literature curated (LC) data. For the combined data from literature curated and genome wide screens, we also generated probability distributions of each PIN graph-theoretic property discussed above, separately for SSL and non-SSL pairs. Probability distributions were converted from histograms to smoothed probability density functions using Gaussian smoothing as implemented by the 'density' function in the R statistical computing platform.

**Table 1:** Statistics of known SSL pairs in yeast

	**Lethal/Sick**	**Non-Lethal/Non-Sick**
HTP	3962	400,869
HTP + LC	6074	400,473

The table lists number of SSL pairs and non-SSL pairs in high throughput (HTP) and combined (HTP + literature curated (LC)) data sets.

### Support vector machine classifier

**Figure 1:**
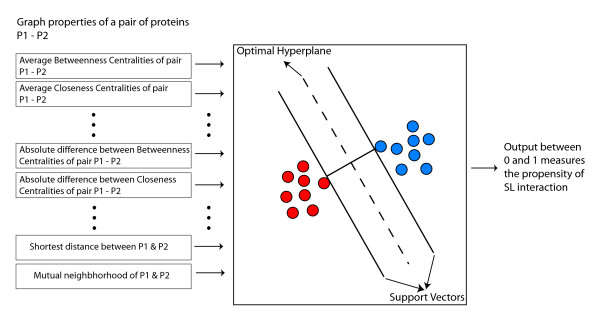
A schematic diagram representing the SVM classifier with various graph theoretic properties fed as inputs.

We use support vector machines (SVMs) to model correlations between PIN properties and the existence of a SSL interaction. Various graph-theoretical properties (local as well as global) of two proteins in a PIN are fed as inputs to the SVM classifier, which is schematically represented in Figure 1.

The output of the SVM classifier is a score that measures the propensity of the two corresponding genes to partake in a SSL interaction. High scores indicate higher propensities for SSL interactions, while low scores indicate higher propensities for lack of a SSL interaction. We found that SVMs had the highest overall accuracy as compared to other prediction systems that we experimented with, including naive Bayes classifiers and neural networks. In our study, we used libSVM, a publicly available integrated software for support vector classification [34]. The full prediction system has 22 inputs lumped into a single vector **x**: the averages and absolute differences of the 9 properties (a)-(i) for each protein pair, the shortest distance *d*(*p*, *q*) between the two proteins, number of mutual neighbors of the two proteins in PIN, and the two indicator variables 2Hop S-S and 2Hop S-P. The raw output SVM scores that were assigned to the protein pairs by the SVM classifier were mapped to posterior probabilities with a value ranging between 0 and 1 to measure propensity for a SSL interaction between the corresponding genes [35]. The SVMs were trained using a radial basis function kernel whose parameters were optimized by performing a five-fold cross-validation on the entire training set, as suggested [36]. The SVM classifier was trained on a randomly selected but a balanced subset of pairs of genes with and without SSL interaction, i.e., the training set comprised of two-thirds of the known synthetic lethal pairs and an equal number of known non-SSL pairs. The test set consisted of the remaining gene pairs (both known SSL and non-SSL) that were not used in training the SVM classifier.

Since the SVM output score takes a continuum of values between 0 and 1, we set a variable cutoff *κ *for deciding whether a pair of genes partakes in a SSL interaction. Pairs that scored above *κ *were predicted to be SSL, while pairs that scored below *κ *were predicted to be non-SSL. We then measured the sensitivity and specificity of the method on the test sets as a function of *κ *and generated ROC (Receiver Operating Characteristic) curves of sensitivity vs. 1-specificity to represent the overall accuracy of the method. Sensitivity is defined as the proportion of true positives that are classified correctly by the method and specificity is defined as the proportion of true negatives that are classified correctly by the method. The area under the ROC curve, a statistic commonly used to assess prediction accuracy (see, for example, [37]) was computed in a non-parametric manner using the trapezoidal approximation. In a similar manner, we found the positive predictive value (precision rate) of the method as a function of the threshold *κ*, where positive predictive value is defined as the ratio of correctly predicted positives to the total number of predicted positives.

### Cross validation studies

Ten-fold cross-validation studies of the performance of the SVM classifier were carried out by using balanced SSL and non-SSL training data for training but representative datasets for testing, as follows. Known SSL pairs were first divided randomly into 10 groups. An equal number of non-SSL pairs were randomly selected and each of these pairs was randomly assigned to one of the 10 groups. Thus, each group contained an equal number of SSL and non-SSL pairs. Nine such groups were combined for training the SVM classifier, which was subsequently tested on all SSL pairs from the withheld group and all non-SSL pairs from the withheld group as well as the remaining data. This was repeated 10 times with each group playing the role of the test group once.

### Prediction of novel SSL interactions

**Figure 2:**
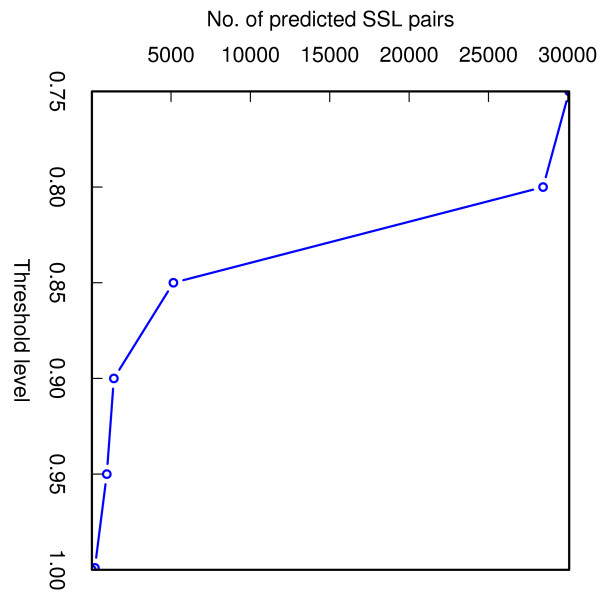
**Number of predicted novel SSL pairs at different levels of threshold**. At a threshold level of 0.75, the total number of predictions of the classifier equals the number of true SSL predictions that the data set is expected to contain.

Finally, we sought to identify novel SSL interactions. To this end, we first culled a data set consisting of gene pairs that were not tested for synthetic genetic interactions (obtained by generating all possible pairwise combinations of non essential genes in *S. cereviseae *and then removing known SSL and non-SSL pairs that were used in the assessment of cross-validation accuracy). To score the new set of gene pairs we retrained our classifier on the complete set of known SSL pairs and an equal number of randomly selected non-SSL pairs. The retrained classifier was then used to evaluate the propensity of each of the ≈1, 620, 000 gene pairs in the newly constructed data set to be SSL. This prediction task was repeated five times, each time training on a different set of randomly selected non-SSL pairs. The gene pairs that scored above the desired cutoff in all the five runs were reported as putative novel SSLs (the number of putative novel SSLs at different thresholds is shown in Figure 2). Based on results from high throughput genetic analysis studies, it has been estimated that the global SSL network of yeast will contain ≈200,000 interactions [38]. As there are ≈4500 non-essential genes in *S. cerevisiae*, we expect the newly constructed data set to proportionately contain ≈200,000 × 1,620,000/$\left(\begin{array}{c} 4500\\ 2 \end{array}\right)$ = 31,307 true SSL interactions, which turns out to be approximately equal to the number of novel SSL predictions that can be obtained at a SVM output threshold level of *κ *= 0.75 (Figure 2).

## Results and discussion

We used thirteen different graph-theoretic properties of proteins (see Methods section) in a PIN as putative predictors for the existence of a SSL interaction between the corresponding gene pair. This resulted in a maximum of 22 inputs to the SVM classifier: two values for each single node property, and one value each for the shortest distance, mutual protein neighborhood, 2Hop S-S and 2Hop S-P.

### Input feature distributions for SSLs and non-SSLs

**Figure 3:**
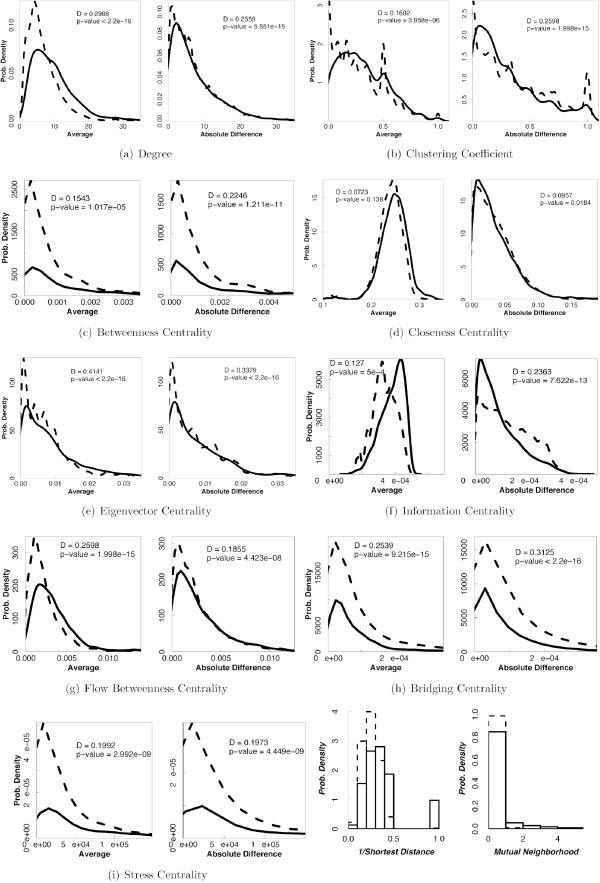
**Distributional differences in PIN graph-theoretic properties in SSL and non-SSL pairs**. The distributions of the average and difference of each property across two proteins in case of SSL pairs (solid curves) and non-SSL pairs (dashed curves) are displayed here. Numbers in each plot indicate the D-statistic associated with the Kolmogorov-Smirnov test for the difference between the two distributions and the corresponding P-value.

In order to assess the suitability of each of the graph-theoretic properties in distinguishing SSL pairs from non-SSL pairs we obtained the distributions of these properties across SSL pairs and non-SSL pairs. For each property, we plot the distribution of the average of that property over two genes in a pair, and the absolute difference of that property across the two genes. Most properties studied here show statistically significant but small distributional differences between SSL pairs and non-SSL pairs (see Figure 3). Properties that display the greatest distributional differences (as measured by the Kolmogorov-Smirnov statistic) are eigenvector centrality, degree and bridging centrality. Since shortest distance is technically infinite for two proteins that lie in two different components of the PIN, we used the inverse of shortest distance as input to the SVM classifier. From Table 2 one can infer that, when viewed as part of a PIN, SSL pairs as compared to non-SSL ones tend to have higher average degree, higher average closeness centrality, higher average information centrality and higher number of mutual neighbors.

**Table 2:** Network statistics of SSL vs. non-SSL pairs

**Gene pair characteristic**	***t *statistic**	**P-value**
Average Degree	21.1908	< 2.2 × 10^-16^
Average Closeness Centrality	22.9225	< 2.2 × 10^-16^
Average Information Centrality	53.1484	< 2.2 × 10^-16^
Mutual Neighborhood	18.2122	< 2.2 × 10^-16^

Difference in mean values of various graph properties between SSL and non-SSL pairs, assessed using an independent sample *t*-test.

### Accuracy of SSL predictions

**Figure 4:**
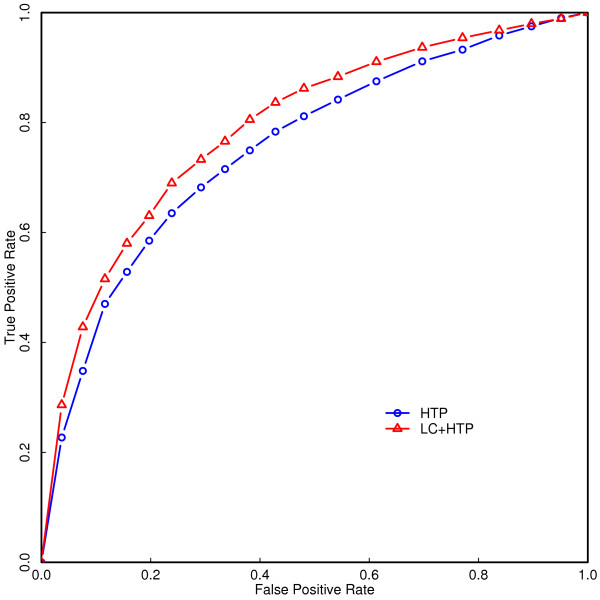
**ROC curves for SVM classifier trained on high throughput SSL data alone and combined data**. The combined data comprised of both high throughput and literature-curated SSL interactions. In both cases, the inputs to the SVM classifier included all the graph theoretic PIN properties (but not 2Hop features).

We first assessed the performance of the SVM classifier when only PIN properties were used and the "triangle-completing" 2Hop properties were excluded. This is because 2Hop properties are binary inputs whose prediction accuracy is extremely sensitive to the choice of test data set (described below). We randomly selected 2/3 of the known SSL pairs and an equal number of non-SSL pairs for training the SVM classifier and withheld the remaining pairs of genes for testing the accuracy of the method. We tested the method on the withheld data, resulting in the ROC curves for different training sets. As is clear from Figure 4, the overall performance of the predictor, as measured by the area under the ROC curve, shows slight improvement when interactions from literature curated data are included in the training set as opposed to using interactions from high throughput synthetic lethal screens alone, with overall accuracies of 0.7960 and 0.7550 respectively. We note that higher values of the SVM classifier output threshold, *κ*, (corresponding to a lower false positive rate) lead to higher specificity, while lower values of *κ *(corresponding to a lower false negative rate) lead to higher sensitivity.

### Addition of 2Hop features to the inputs improves the performance of the classifier

**Figure 5:**
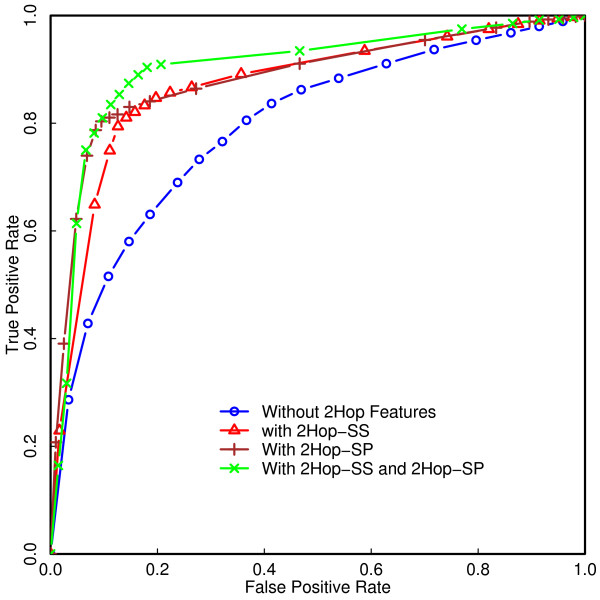
**Comparison of ROC curves before and after addition of 2Hop characteristics**. ROC curves for SVM classifiers trained on combined data (literature curated and high through put data) with and without using 2Hop characteristics as inputs.

When 2Hop features were included as additional inputs to the SVM classifier there was a significant improvement in the performance of the classifier on the randomly chosen test set (Figure 5), consistent with previously reported increases in accuracy when 2Hop features are included [21]. The best overall accuracy, as measured by the AUC, is about 90.4%, obtained with the SVM classifier trained on literature curated and high throughput data using all the PIN properties and 2Hop features.

### Comparison to other approaches

**Figure 6:**
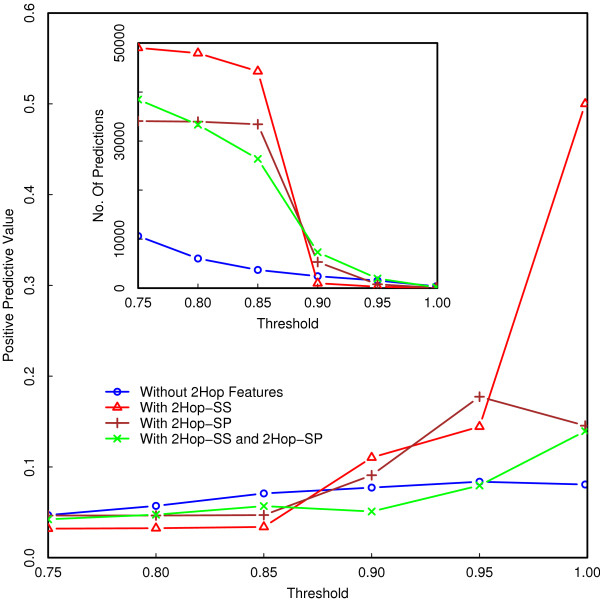
**Comparison of PPVs for various classifiers**. Positive predictive value for SVM classifiers trained on combined data with and without using 2Hop characteristics as inputs. At a threshold level of 0.999, the classifier trained with all graph theoretic protein properties and 2Hop SS as inputs has the highest positive predictive value. Addition of the extra indicator variable 2Hop SP as input results in a classifier with decreased positive predictive accuracy. The subplot in the figure shows the number of predicted pairs (true positives and false positives) at different levels of threshold.

We compared our results to an earlier study [21] where probabilistic decision trees were used to distinguish between SSL and non-SSL pairs. The accuracy of our method appears higher than that reported in [21] (the AUC was not explicitly computed there), where diverse genomic datasets and 2Hop properties are included as putative determinants of SSL interaction (see Additional file 1 for a detailed comparison with the results of [21]). Further, the accuracy obtained by our method when 2Hop properties are excluded is significantly higher than that found in [21] when 2Hop properties are excluded, as further discussed below (see also Supplementary Information). We note that accuracies measured by area under the ROC curve take into account both false positive and false negative errors. However, they do not account for the low *prevalence *of SSLs among all pairs of genes in a genome. We therefore also compute the positive predictive accuracies for our SVM classifiers as a function of the threshold level (Figure 6). It is interesting to note that addition of both 2Hop characteristics leads to a *decrease *in predictive accuracy at high thresholds, even though the AUC increases when both these features are included. The reason for this is that the increase in sensitivity after addition of both 2Hop characteristics is offset by a faster increase in false positive rate. The positive predictive values (PPV) of the predictors at a threshold level of 0.999 are listed in Table 3. Note that in order to assess the the fold-improvement in prediction ability, the PPV should be compared to the estimated prevalence of SSLs among all gene pairs in yeast (this prevalence is ≈200000/$\left(\begin{array}{c} 4500\\ 2 \end{array}\right)$ = 0.0198, as it is estimated that there are ≅ 200,000 SSL interactions and ≈4500 non-essential genes in *S. cerevisiae*) [38].

**Table 3:** Predictive power of the SVM classifiers

**Set of input features to SVM classifier**	**Positive predictive value**
All graph theoretic protein characteristics	0.08
All graph theoretic protein characteristics + 2Hop SS	0.50
All graph theoretic protein characteristics + 2Hop SP	0.15
All graph theoretic protein characteristics + 2Hop SS + 2Hop SP	0.14

Positive predictive values for classifiers trained with and without 2Hop characteristics at a threshold level of 0.999.

### Individual importance of predictor variables

**Figure 7:**
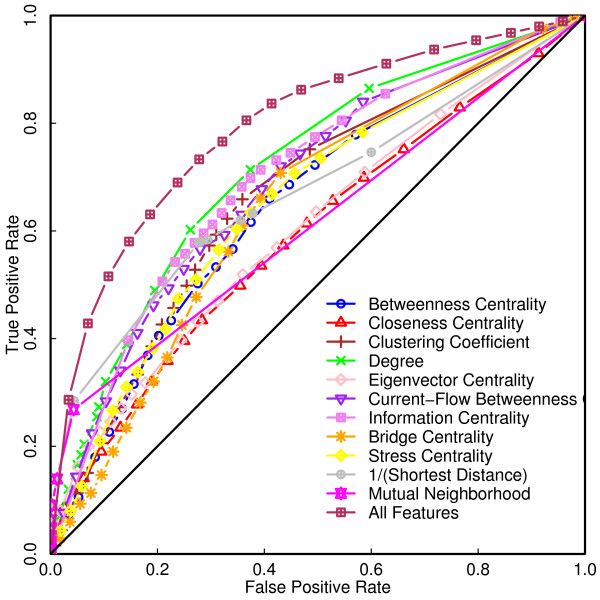
**Importance of individual predictor variables**. ROC curves for SVM classifiers trained on literature curated and high throughput data using individual predictor variables. The diagonal line indicates random prediction. The ROC curve for the SVM classifier trained using all the input features is also shown in the figure.

We investigated the importance of each of the individual predictor variables by training the SVM classifier on each of them separately (Figure 7). Classifiers trained using individual predictors perform better than random classifiers, although the classification performance when all the predictor variables are used is much better than any of the classifiers trained on individual predictor variables. Of all the PIN predictor variables studied, degree turns out to be the best performing individual predictor. The known importance of degree in characterizing gene essentiality therefore extends to SSL properties of gene pairs as well. Indeed, strong correlations between synthetic lethality and node degree have been reported earlier [39]. The second best predictor was information centrality, a hybrid measure which relates to both closeness centrality and random walk based eigen-centrality, each of which turned out to be significant predictor variables on their own. Also, the significant contribution of information centrality to SSL prediction may indicate that information propagation in a biological network does not always favor shortest paths. We further tested the individual importance of the 2Hop characteristics when used singly or jointly as predictor variables. Since these features always assign equal scores to all pairs for which "triangle completion" is possible and equal scores to all pairs for which this is not possible, these inputs lead to fixed specificity and sensitivity values. While it is possible to predict SSL pairs by triangle completion with reasonably high specificity and sensitivity on certain test sets (see Table 4), namely those that have a large number of SSL or protein interactions with other genes/proteins, the specificities and sensitivities will vary greatly as properties of the test set are changed (discussed below).

**Table 4:** Accuracy of prediction performance using 2Hop characteristics alone

**2Hop Characteristic**	**Threshold Level (T)**	**Sensitivity**	**Specificity**
2HopSS	0.000000 ≤ T ≤ 0.235290	1.000	0.000
	0.235290 < T ≤ 0.860423	0.723	0.886
	0.860423 < T ≤ 1.000000	0.000	1.000
2HopSP	0.000000 ≤ T ≤ 0.217713	1.000	0.000
	0.217713 < T ≤ 0.905607	0.755	0.922
	0.905607 < T ≤ 1.000000	0.000	1.000
2HopSS and 2HopSP	0.000000 ≤ T ≤ 0.138735	1.000	0.000
	0.138735 < T ≤ 0.840256	0.868	0.835
	0.840256 < T ≤ 0.840260	0.723	0.886
	0.840260 < T ≤ 0.840392	0.610	0.973
	0.860423 < T ≤ 1.000000	0.000	1.000

The table lists sensitivity and specificity values at different threshold levels in case of classifiers trained using 2Hop characteristics alone.

### Robustness of prediction with respect to choice of test data

We first performed ten-fold cross validation of the SVM classifier (Methods) with all inputs, and found less than 1% variation in classification accuracy as measured by area under the ROC curve (Table 5), thus confirming the robustness of the classification performance with respect to different choices of randomly constructed test sets.

**Table 5:** Area under the ROC curves for ten cross validation runs

**CrossValidation Run #**	**AUC**
Run1	0.913
Run2	0.901
Run3	0.916
Run4	0.900
Run5	0.905
Run6	0.912
Run7	0.906
Run8	0.896
Run9	0.899
Run10	0.908

The classifier was trained using all the graph-theoretic properties and the 2Hop characteristics.

Next, in order to further assess the role of the 2Hop properties in the prediction task, we designed a test set in which none of the genes had SSL interactions with other genes/proteins. Both 2Hop properties are identically zero for all pairs in this test set and these properties therefore lose predictive value on such a set. While this type of test set does not reflect the enhanced prevalence of triangles in SSL networks, we carried out this procedure in order to assess whether PIN properties by themselves would also significantly lose their predictive value when no triangles can be completed with known SSL interactions for a test pair. Table 6 shows that although there is some loss of accuracy, the accuracy of 70% is still considerably larger than the corresponding accuracy in Wong et al. [21] when 2Hop properties are not included.

**Table 6:** Effect of the exclusion of gene pairs with non-zero 2Hop properties

**Across 10 cross-validation runs**	**AUC – Before exclusion**	**AUC – After exclusion**
Average	0.797	0.697
Standard Deviation	0.038	0.005

Ten fold cross-validation was carried out with and without excluding gene pairs having non-zero 2Hop properties from training and test sets. In both the cases the classifiers were trained without using 2Hop characteristics.

### Robustness of prediction with respect to errors in the protein interaction network

Since our prediction method relies strongly on protein interaction data, it is important to assess the prediction quality with respect to errors in protein interaction data. Since we use high confidence protein interaction data (with a low false positive rate), we surmised that the dominant error in the protein interaction network could be attributed to missing interactions. We therefore added a predetermined number of new edges randomly to the original protein interaction network, retrained and reevaluated our SVM classifier. This task was repeated, each time adding a different number of random interactions (250, 500, 750, 1000) to the PIN. While adding more than 500 random interactions (representing approximately 5% of the number of existing protein interactions) significantly changes the numerical value of the propensity for SSL interaction assigned by the SVM, we found no detectable change in the ROC curves (see AUC values in Table 7). This suggests that random additional interactions contribute roughly equally to input features of SSL and non-SSL pairs, resulting in no significant change in overall discrimination ability.

**Table 7:** Robustness analysis with respect to addition of random edges to PIN

**Number of random edges added to PIN**	**P-value for difference in propensities**	**AUC**
250	0.7905	0.8692
500	0.1391	0.8724
750	< 2.2 × 10^-16^	0.8646
1000	< 2.2 × 10^-16^	0.8671

Paired sample *t*-test P-values for the difference in SSL propensities assigned to the test dataset by the SVM before and after adding random edges to the protein interaction network.

### Comparison of predicted SSL network with the known SSL network

Having achieved reasonably high cross-validation accuracy, we proceeded to find out if the predicted SSL network shared the same characteristics as the known SSL network (see Methods for construction of the network comprising of novel SSL predictions). Earlier studies have indicated that genes known to have SSL interaction tend to share similar Gene Ontology (GO) annotation, are enriched for common upstream regulators, and preferentially are part of the same protein complex [40,41]. Since none of these characteristics were used as input features for our prediction method (except, indirectly, participation in the same protein complex), we investigated whether our predicted SSLs were also enriched for these features at different threshold levels starting from 0.75 up to 1.000 (See Supplementary Information for the list of predictions at these threshold levels). As is evident from Table 8, predicted SSL interactions at each of the threshold levels appear to have properties similar to known SSL interactions. While the existence of a common upstream regulator among the predicted novel SSL pairs does not appear statistically significant beyond a threshold level of 0.85, this may be attributed to the small sample size at high thresholds. Odds ratios for all properties generally show an increasing trend as the threshold level is increased, showing that the SVM classifier preferentially selects gene pairs having the properties studied with higher accuracy at higher thresholds, even though the properties themselves are not used as input features for prediction. The simultaneous increase in the predictive accuracy of SSL prediction (as threshold level is increased) and enrichment for participation in the same protein complex (evinced by the increase in protein complex enrichment odds) is consistent with the dominance of "within-pathway" explanations for genetic interactions suggested by Kelley and Ideker [42].

**Table 8:** Comparison of the predicted SSL network to the known SSL network

Threshold Level (Number of pre dictions)	Gene-pair Characteristic	S∩C	Sonly	Conly	S'∩C'	Odds	P-value
Known SSL Pairs (6074)	Cellular Component	4467	1607	193007	207466	2.988	< 2.2 × 10^-16^
	Molecular Function	1464	4610	38865	361608	2.955	< 2.2 × 10^-16^
	Biological Process	4074	2000	137827	262646	3.882	< 2.2 × 10^-16^
	Protein Complex	197	5877	747	399726	17.937	< 2.2 × 10^-16^
	Upstream Regulator	220	5854	10327	390146	1.420	9 × 10^-7^
0.75 (30087)	Cellular Component	17967	12120	781640	799150	1.516	< 2.2 × 10^-16^
	Molecular Function	4115	25972	113100	1467690	2.056	< 2.2 × 10^-16^
	Biological Process	12966	17121	314562	1266228	3.048	< 2.2 × 10^-16^
	Protein Complex	363	29724	1339	1579451	14.405	< 2.2 × 10^-16^
	Upstream Regulator	1052	29035	47294	1533496	1.175	3.76 × 10^-7^
0.80 (28440)	Component	16942	11498	782665	799772	1.506	< 2.2 × 10^-16^
	Function	3826	24614	113389	1469048	2.014	< 2.2 × 10^-16^
	Process	12245	16195	315283	1267154	3.039	< 2.2 × 10^-16^
	Protein Complex	346	28094	1356	1581081	14.360	< 2.2 × 10^-16^
	Upstream Regulator	986	27454	47360	1535077	1.164	3.01 × 10^-6^
0.850 (5149)	Component	3799	1350	795808	809920	2.864	< 2.2 × 10^-16^
	Function	1309	3840	115906	1489822	4.382	< 2.2 × 10^-16^
	Process	3221	1928	324307	1281421	6.601	< 2.2 × 10^-16^
	Protein Complex	287	4862	1415	1604313	66.927	< 2.2 × 10^-16^
	Upstream Regulator	173	4976	48173	1557555	1.124	0.024
0.900 (1398)	Component	1184	214	798423	811056	5.602	< 2.2 × 10^-16^
	Function	508	890	116707	1492772	7.301	< 2.2 × 10^-16^
	Process	1060	338	326468	1283011	12.325	< 2.2 × 10^-16^
	Protein Complex	151	1247	1551	1607928	125.535	< 2.2 × 10^-16^
	Upstream Regulator	49	1349	48297	1561182	1.174	0.1525
0.950 (953)	Component	831	122	798776	811148	6.917	< 2.2 × 10^-16^
	Function	368	585	116847	1493077	8.038	< 2.2 × 10^-16^
	Process	776	177	326752	1283172	17.217	< 2.2 × 10^-16^
	Protein Complex	126	827	1576	1608348	155.485	< 2.2 × 10^-16^
	Upstream Regulator	29	924	48317	1561607	1.014	0.4958
0.999 (202)	Component	184	18	799423	811252	10.373	< 2.2 × 10^-16^
	Function	93	109	117122	1493553	10.880	< 2.2 × 10^-16^
	Process	179	23	327349	1283326	30.511	< 2.2 × 10^-16^
	Protein Complex	37	165	1665	1609010	216.702	< 2.2 × 10^-16^
	Upstream Regulator	5	197	48341	1562334	0.820	0.7274

Association between SSL interaction and gene-pair characteristic, following the framework of [20]. S ∩ C represents the number of gene pairs with both SSL interaction(S) and same gene-pair characteristic(C). Sonly represents the number of gene pairs with SSL interaction only. Conly represents the number of non-SSL gene pairs that share the same characteristic. S' ∩ C' represents the number of gene pairs that neither have SSL interaction nor share a specified characteristic. Odds represents the odds ratio of a SSL pair sharing a given characteristic to a non-SSL pair sharing the same characteristic. The P-value represents the statistical significance of overlap between SSL interaction and the specified characteristic as computed by Fisher's exact test. If a protein corresponding to a gene is not assigned to any of the known protein complexes then the gene and its interacting partner are considered to come from two different protein complexes. Similarly in the case of upstream regulators, if a gene does not have any known upstream regulator then the gene and its interacting partner are treated as if they don't share any common upstream regulator. The P-values are not significant in case of common upstream regulator due to the fact the number of predicted positives that share a common upstream regulator is very small in comparison to the total number of pairs that share a common upstream regulator, which is also reflected in the low odds ratio seen at different levels of threshold.

## Conclusion

Our results clearly demonstrate the informative value of protein interaction networks for SSL genetic interactions. We show that graph-theoretic properties of proteins in a protein interaction network serve as compelling and relatively robust determinants for the existence of synthetic lethality between their gene counterparts. When members of the gene pair in question have known SSL interactions with other genes, the predictive power for SSL interaction within that gene pair is greatly enhanced by the tendency of triangles to form in SSL networks (2Hop properties). However, even in the absence of known SSL interactions, we have shown that PINs by themselves can predict SSL interactions with significantly higher accuracy than previously found. Inclusion of PIN centralities in the development of meta-servers for SSL prediction is therefore likely to be very useful. Further, even though no functional properties are used as input features in our method, the method identifies gene pairs that are enriched for participation in common GO categories, in the same protein complex, and to a more limited extent, for having the same upstream regulator. These properties may be therefore viewed as further *predictions *of the method, even though they were earlier used as *inputs *for identifying SSLs [21]. This shows that PINs, even in the absence of qualifying data from gene regulatory and gene expression studies, may be more informative of gene function than normally envisioned.

## Authors' contributions

SP & SZ performed the analysis and wrote the paper. AlR & AnR conceived the project and wrote the paper.

## Supplementary Material

Additional file 1 **Supplementary Information**. This file contains analysis of the performance of our SSL prediction method as compared to the method used in [21]. It also contains novel SSLs predicted by our method at different threshold levels. Click here for file
