# Supplementary material for: Mining protein networks for synthetic genetic interactions
Source: BMC Bioinformatics. 2008 Oct 9;9:426. doi: 10.1186/1471-2105-9-426 (PMC2577120; doi:10.1186/1471-2105-9-426)
Supplement: Additional file 1 — Supplementary Information. This file contains analysis of the performance of our SSL prediction method as compared to the method used in [21]. It also contains novel SSLs predicted by our method at different threshold levels. [file 1471-2105-9-426-S1.pdf]

Supplementary Information

Classifier performance comparison.

Comparison of the classification performance between the method used in this paper and that of Wong et al. [2] can be done in two ways. One approach is to apply the classification method used by Wong et al. on the SSL and non-SSL datasets used in this paper, but this could not be done as the authors did not report the values for the input features. The other approach is to apply the classification method used in this paper on the SSL and non-SSL used by Wong et al. So the analysis was redone using the SSL and non-SSL pairs from [1] alone. Unfortunately, an exact comparison is not possible even in this case as the classification method used in this paper requires the proteins corresponding to the input SSL and non-SSL pairs be present in literature curated (LC) protein interaction network used in this paper (from which the input centrality measures were calculated). After removal of pairs whose corresponding proteins are not represented in the LC protein interaction network along with repeats (A-B needs to be treated same as B-A) and pairs whose corresponding ORFs were found to be dubious, the genetic interaction dataset was reduced to 2681 unique SSL pairs and 223663 unique non-SSL pairs. A 4-fold cross validation (as in [2]) was then carried out by using balanced SSL and non-SSL training data for training but representative datasets for testing (similar to 10-fold cross validation in this paper). In each cross validation run, the classifier was trained using the training data and tested on the withheld data resulting in 4 different ROC curves for as many training sets. To assess the importance of 2Hop characteristics 4-fold cross validation was carried before and after the inclusion of 2Hop characteristics as was done in [2]. The ROC curves are reported in Supplementary Figure 1.

The accuracy metric reported in [2] was the sensitivity of the method at a false positive rate of 18%. This sensitivity was found to be 80% in [2] when 2Hop features were included. Further, from the ROC curves reported in [2], we estimated the sensitivity at 18% without 2Hop features to be about 35%, the area under the ROC curve (AUC) with 2Hop features to be about 0.87, and the AUC without 2Hop features to be about 0.70. In comparison, even though our method uses a more limited feature set, it performs better under both metrics (sensitivity at 18% FPR as well as AUC) with and without 2Hop features included, as is clear from Supplementary Table 1.

Supplementary Table 1: Area under the ROC curves (AUC) and sensitivity at 18% False Positive Rate (S18FPR) across 4 cross validation runs. Four fold cross validation was carried out with and without using 2Hop characteristics.

No.	AUC - w/o 2Hop	AUC - with 2Hop	S18FPR - w/o 2Hop	S18FPR - with 2Hop
Run1	0.721	0.900	48.3%	85.7%
Run2	0.727	0.896	52.4%	86.6%
Run3	0.740	0.883	52.2%	84.3%
Run4	0.726	0.893	52.1%	85.7%

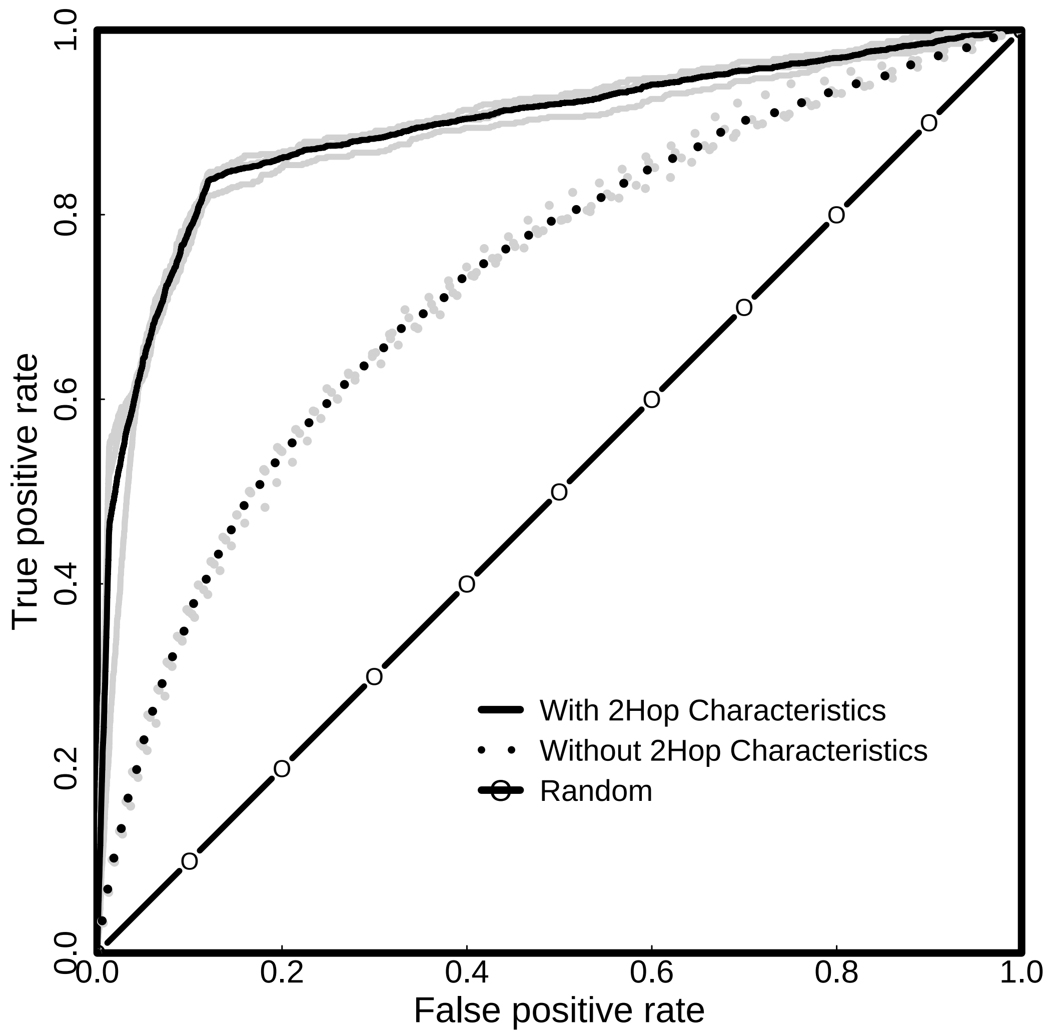

Supplementary Figure 1: Performance of the classifier in cross validation with and without using 2Hop Characteristics. The ROC curves for cross-validation runs (shown in grey) were overlaid by the vertical average curves.

References

[1] A.H.Y. Tong, G. Lesage, G.D. Bader, H Ding, H. Xu, X. Xin, J. Young, and etal. Global mapping of the yeast genetic interaction network. *Science*, 303:808–813, 2004.

[2] S. L. Wong, L. V. Zhang, A. H. Tong, Z. Li, D. S. Goldberg, O. D. King, G. Lesage, M. Vidal, B. Andrews, H. Bussey, C. Boone, and F. P. Roth. Combining biological networks to predict genetic interactions. *Proc Natl Acad Sci U S A*, 101(44):15682–15687, November 2004.

Predictions at different threshold levels

Since the predictions at each of the threshold levels were obtained by collecting the common predictions at that level of threshold across five different runs it is not possible to assign a unique score (propensity for being SSL) for each of the predictions. So we have reported the common predictions separately at each threshold level.

Predicted pairs at a threshold level of 0.75

Predictions at threshold level 0.75			
Gene1 of predicted pair		Gene2 of predicted pair	
**Systematic Name**	**Standard Name**	**Systematic Name**	**Standard Name**
YGL025C	PGD1	YJR032W	CPR7
YNL199C	GCR2	YGR059W	SPR3
YLR377C	FBP1	YDR028C	REG1
YDL116W	NUP84	YNL236W	SIN4
YNL054W	VAC7	YDR378C	LSM6
YKL038W	RGT1	YOR047C	STD1
YGR108W	CLB1	YDL155W	CLB3
YJL136C	RPS21B	YJL095W	BCK1
YMR095C	SNO1	YNL032W	SIW14
YLR435W	TSR2	YGL019W	CKB1
YHR111W	UBA4	YGR270W	YTA7
YBL102W	SFT2	YJR040W	GEF1
YBL008W	HIR1	YML115C	VAN1
YNR072W	HXT17	YLR180W	SAM1
YCR081W	SRB8	YMR311C	GLC8
YLR094C	GIS3	YPL137C	GIP3
YOR275C	RIM20	YLR320W	MMS22
YOR043W	WHI2	YDL077C	VAM6
YJL183W	MNN11	YKL119C	VPH2
YHR056C	RSC30	YPR119W	CLB2
YGR014W	MSB2	YKL041W	VPS24

Predictions at threshold level 0.75 continued...			
Gene1 of predicted pair		Gene2 of predicted pair	
Systematic Name	Standard Name	Systematic Name	Standard Name
YEL027W	CUP5	YHR039C-A	VMA10
YBR131W	CCZ1	YBR041W	FAT1
YFR031C-A	RPL2A	YHR081W	LRP1
YFR036W	CDC26	YNL107W	YAF9
YGL025C	PGD1	YBR289W	SNF5
YDR137W	RGP1	YOR038C	HIR2
YOR358W	HAP5	YAL002W	VPS8
YBL051C	PIN4	YDR183W	PLP1
YBL102W	SFT2	YNL291C	MID1
YMR109W	MYO5	YLR233C	EST1
YCR077C	PAT1	YGR188C	BUB1
YER161C	SPT2	YDR440W	DOT1
YMR080C	NAM7	YHR077C	NMD2
YLR043C	TRX1	YDR423C	CAD1
YDR456W	NHX1	YNL199C	GCR2
YPL057C	SUR1	YBR019C	GAL10
YDL161W	ENT1	YNR006W	VPS27
YLR288C	MEC3	YML016C	PPZ1
YLR018C	POM34	YDR207C	UME6
YGL003C	CDH1	YMR214W	SCJ1
YOR080W	DIA2	YDL047W	SIT4
YMR205C	PFK2	YKR020W	VPS51
YMR284W	YKU70	YER116C	SLX8
YKR001C	VPS1	YLR182W	SWI6
YHR108W	GGA2	YDL192W	ARF1
YBR009C	HHF1	YOR064C	YNG1
YOR270C	VPH1	YNL307C	MCK1
YNL054W	VAC7	YJR060W	CBF1
YPR072W	NOT5	YJR063W	RPA12

Predictions at threshold level 0.75 continued...			
Gene1 of predicted pair		Gene2 of predicted pair	
Systematic Name	Standard Name	Systematic Name	Standard Name
YLR292C	SEC72	YOR023C	AHC1
YDR443C	SSN2	YBR019C	GAL10
YDR507C	GIN4	YOR327C	SNC2
YJL187C	SWE1	YCR086W	CSM1
YLR360W	VPS38	YLR079W	SIC1
YJL154C	VPS35	YDL192W	ARF1
YGL163C	RAD54	YOR038C	HIR2
YLR234W	TOP3	YNL206C	RTT106
YBR258C	SHG1	YGL237C	HAP2
YPL178W	CBC2	YKL068W	NUP100
YGR071C	YGR071C	YGR188C	BUB1
YLR386W	VAC14	YER116C	SLX8
YBR042C	CST26	YDR358W	GGA1
YBL079W	NUP170	YKR024C	DBP7
YLR216C	CPR6	YFR036W	CDC26
YHR056C	RSC30	YKL113C	RAD27
YBR107C	IML3	YNL147W	LSM7
YPR120C	CLB5	YLR315W	NKP2
YBR173C	UMP1	YGL115W	SNF4
YBR042C	CST26	YDR137W	RGP1
YGR070W	ROM1	YOL081W	IRA2
YEL065W	SIT1	YEL060C	PRB1
YDR129C	SAC6	YDR310C	SUM1
YMR198W	CIK1	YPL180W	TCO89
YGL208W	SIP2	YJL187C	SWE1
YBL103C	RTG3	YER040W	GLN3
YOR270C	VPH1	YBR171W	SEC66
YGL033W	HOP2	YDL192W	ARF1
YOR337W	TEA1	YJL095W	BCK1

Predictions at threshold level 0.75 continued...			
Gene1 of predicted pair		Gene2 of predicted pair	
Systematic Name	Standard Name	Systematic Name	Standard Name
YJR063W	RPA12	YOR064C	YNG1
YGR261C	APL6	YJL053W	PEP8
YGL025C	PGD1	YOR327C	SNC2
YJL124C	LSM1	YBL047C	EDE1
YBR200W	BEM1	YBL021C	HAP3
YJL006C	CTK2	YLR182W	SWI6
YDR335W	MSN5	YDR318W	MCM21
YPL105C	YPL105C	YOR327C	SNC2
YOR080W	DIA2	YJR135C	MCM22
YDR320C	SWA2	YGR105W	VMA21
YDL020C	RPN4	YGR270W	YTA7
YPL024W	RMI1	YPR036W	VMA13
YBR274W	CHK1	YGR188C	BUB1
YFR031C-A	RPL2A	YAL002W	VPS8
YDR012W	RPL4B	YOR078W	BUD21
YDL122W	UBP1	YOR124C	UBP2
YOR127W	RGA1	YML001W	YPT7
YDR260C	SWM1	YGR135W	PRE9
YNL236W	SIN4	YER068W	MOT2
YKL126W	YPK1	YGL212W	VAM7
YOL018C	TLG2	YOR036W	PEP12
YDR383C	NKP1	YJL006C	CTK2
YNR052C	POP2	YLR315W	NKP2
YNL087W	TCB2	YER144C	UBP5
YER116C	SLX8	YPL037C	EGD1
YLR079W	SIC1	YNL116W	DMA2
YER044C	ERG28	YOR035C	SHE4
YOR290C	SNF2	YBR019C	GAL10
YDL223C	HBT1	YML071C	COG8

Predictions at threshold level 0.75 continued...			
Gene1 of predicted pair		Gene2 of predicted pair	
Systematic Name	Standard Name	Systematic Name	Standard Name
YPR017C	DSS4	YKR001C	VPS1
YPL042C	SSN3	YOR038C	HIR2
YNR072W	HXT17	YDL192W	ARF1
YCR020C-A	MAK31	YNL238W	KEX2
YIR003W	YIR003W	YPR119W	CLB2
YBR194W	SOY1	YJL183W	MNN11
YPL024W	RMI1	YDR358W	GGA1
YDL020C	RPN4	YAL002W	VPS8
YDR320C	SWA2	YCR066W	RAD18
YBR173C	UMP1	YOR368W	RAD17
YHL020C	OPI1	YNR032W	PPG1
YBR030W	YBR030W	YOL121C	RPS19A
YNL238W	KEX2	YHR108W	GGA2
YDL013W	SLX5	YDL155W	CLB3
YPL137C	GIP3	YER162C	RAD4
YPL050C	MNN9	YCR088W	ABP1
YER110C	KAP123	YPL246C	RBD2
YJL117W	PHO86	YPL089C	RLM1
YOL067C	RTG1	YAR014C	BUD14
YOR039W	CKB2	YCR031C	RPS14A
YML095C	RAD10	YDR318W	MCM21
YNL054W	VAC7	YNL248C	RPA49
YIL076W	SEC28	YDR320C	SWA2
YLR240W	VPS34	YMR202W	ERG2
YOR132W	VPS17	YPL234C	TFP3
YBR017C	KAP104	YKL057C	NUP120
YCL001W	RER1	YHR012W	VPS29
YDR310C	SUM1	YNR032W	PPG1
YMR224C	MRE11	YKR020W	VPS51

Predictions at threshold level 0.75 continued...			
Gene1 of predicted pair		Gene2 of predicted pair	
Systematic Name	Standard Name	Systematic Name	Standard Name
YNL087W	TCB2	YER040W	GLN3
YPL042C	SSN3	YPL057C	SUR1
YOR069W	VPS5	YJR032W	CPR7
YAL056W	GPB2	YPR119W	CLB2
YDL127W	PCL2	YDR289C	RTT103
YML103C	NUP188	YPL018W	CTF19
YIL053W	RHR2	YPL195W	APL5
YCL027W	FUS1	YNL058C	YNL058C
YBL078C	ATG8	YBR131W	CCZ1
YDL065C	PEX19	YGL153W	PEX14
YOL051W	GAL11	YBR010W	HHT1
YIL036W	CST6	YLR399C	BDF1
YER161C	SPT2	YDR310C	SUM1
YMR199W	CLN1	YPR119W	CLB2
YMR167W	MLH1	YPR119W	CLB2
YNL054W	VAC7	YMR055C	BUB2
YNL087W	TCB2	YJR084W	CSN12
YOL121C	RPS19A	YOR035C	SHE4
YBR111W-A	SUS1	YLR399C	BDF1
YLR386W	VAC14	YGL212W	VAM7
YMR284W	YKU70	YLR313C	SPH1
YGL025C	PGD1	YOR066W	MSA1
YOR109W	INP53	YDR207C	UME6
YNL032W	SIW14	YMR016C	SOK2
YGL133W	ITC1	YLR102C	APC9
YMR224C	MRE11	YOR312C	RPL20B
YHR039C-A	VMA10	YDL083C	RPS16B
YEL065W	SIT1	YMR133W	REC114
YPL105C	YPL105C	YNL051W	COG5

Predictions at threshold level 0.75 continued...			
Gene1 of predicted pair		Gene2 of predicted pair	
Systematic Name	Standard Name	Systematic Name	Standard Name
YDR378C	LSM6	YGL003C	CDH1
YGR241C	YAP1802	YKL135C	APL2
YMR273C	ZDS1	YML057W	CMP2
YMR172W	HOT1	YNL288W	CAF40
YKR020W	VPS51	YNL289W	PCL1
YJR032W	CPR7	YOL018C	TLG2
YKR099W	BAS1	YMR311C	GLC8
YGL163C	RAD54	YHR115C	DMA1
YDL155W	CLB3	YPL018W	CTF19
YML099C	ARG81	YDR207C	UME6
YGL212W	VAM7	YCL001W	RER1
YKL032C	IXR1	YNL116W	DMA2
YMR055C	BUB2	YPL152W	RRD2
YDR440W	DOT1	YGL003C	CDH1
YJR135C	MCM22	YER122C	GLO3
YNL032W	SIW14	YPL057C	SUR1
YDR225W	HTA1	YBL003C	HTA2
YBR107C	IML3	YLR233C	EST1
YKR035W-A	DID2	YEL060C	PRB1
YGL115W	SNF4	YOL091W	SPO21
YMR224C	MRE11	YKL126W	YPK1
YLR381W	CTF3	YNL166C	BNI5
YHR119W	SET1	YBR175W	SWD3
YBR030W	YBR030W	YMR152W	YIM1
YFR031C-A	RPL2A	YFR010W	UBP6
YER115C	SPR6	YMR273C	ZDS1
YCR020C-A	MAK31	YOR069W	VPS5
YLR337C	VRP1	YGL124C	MON1
YDL161W	ENT1	YJR125C	ENT3

Predictions at threshold level 0.75 continued...			
Gene1 of predicted pair		Gene2 of predicted pair	
Systematic Name	Standard Name	Systematic Name	Standard Name
YMR016C	SOK2	YLR370C	ARC18
YEL065W	SIT1	YFR036W	CDC26
YMR199W	CLN1	YBL087C	RPL23A
YMR116C	ASC1	YGL153W	PEX14
YDR080W	VPS41	YHR012W	VPS29
YDR156W	RPA14	YOR275C	RIM20
YDR004W	RAD57	YFR036W	CDC26
YML038C	YMD8	YDR153C	ENT5
YLR095C	IOC2	YDR310C	SUM1
YLR079W	SIC1	YOR195W	SLK19
YGR184C	UBR1	YDL013W	SLX5
YDR395W	SXM1	YKL068W	NUP100
YPR131C	NAT3	YDR192C	NUP42
YIL159W	BNR1	YOR337W	TEA1
YPL253C	VIK1	YHR115C	DMA1
YER114C	BOI2	YHR061C	GIC1
YDR320C	SWA2	YJR040W	GEF1
YLR352W	YLR352W	YMR038C	CCS1
YLR094C	GIS3	YGL003C	CDH1
YFL047W	RGD2	YPL246C	RBD2
YHR039C-A	VMA10	YDR207C	UME6
YJL006C	CTK2	YOR141C	ARP8
YGL078C	DBP3	YEL060C	PRB1
YGR020C	VMA7	YER162C	RAD4
YFR030W	MET10	YJL006C	CTK2
YLR079W	SIC1	YMR199W	CLN1
YER116C	SLX8	YLR093C	NYV1
YJR056C	YJR056C	YGL003C	CDH1
YPL046C	ELC1	YNL230C	ELA1

Predictions at threshold level 0.75 continued...			
Gene1 of predicted pair		Gene2 of predicted pair	
Systematic Name	Standard Name	Systematic Name	Standard Name
YLR423C	ATG17	YPR119W	CLB2
YMR125W	STO1	YML057W	CMP2
YDL020C	RPN4	YOR358W	HAP5
YDL192W	ARF1	YEL042W	GDA1
YHR052W	CIC1	YLR325C	RPL38
YOR195W	SLK19	YMR055C	BUB2
YPL105C	YPL105C	YLR309C	IMH1
YLR119W	SRN2	YNL058C	YNL058C
YJL138C	TIF2	YDR310C	SUM1
YOR127W	RGA1	YDR144C	MKC7
YIL103W	DPH1	YGR188C	BUB1
YDL116W	NUP84	YLR313C	SPH1
YNR052C	POP2	YMR038C	CCS1
YBR036C	CSG2	YMR230W	RPS10B
YDR385W	EFT2	YBL071W-A	KTI11
YMR095C	SNO1	YER040W	GLN3
YPR030W	CSR2	YDR358W	GGA1
YMR224C	MRE11	YGL252C	RTG2
YBR171W	SEC66	YHR200W	RPN10
YLR381W	CTF3	YJR060W	CBF1
YBR164C	ARL1	YEL022W	GEA2
YDR477W	SNF1	YBR171W	SEC66
YLR399C	BDF1	YML112W	CTK3
YFR031C-A	RPL2A	YFL023W	BUD27
YDR363W-A	SEM1	YGR188C	BUB1
YCR066W	RAD18	YAL015C	NTG1
YOR127W	RGA1	YML095C	RAD10
YBR057C	MUM2	YEL036C	ANP1
YOR290C	SNF2	YKL109W	HAP4

Predictions at threshold level 0.75 continued...			
Gene1 of predicted pair		Gene2 of predicted pair	
Systematic Name	Standard Name	Systematic Name	Standard Name
YBL051C	PIN4	YLR447C	VMA6
YKL101W	HSL1	YOL081W	IRA2
YBL058W	SHP1	YMR133W	REC114
YDR200C	VPS64	YKL114C	APN1
YOR112W	CEX1	YPL037C	EGD1
YBR030W	YBR030W	YHR039C-A	VMA10
YPR084W	YPR084W	YDR289C	RTT103
YOR039W	CKB2	YGR252W	GCN5
YHR157W	REC104	YGL213C	SKI8
YPL147W	PXA1	YPL145C	KES1
YOR094W	ARF3	YCL034W	LSB5
YPR018W	RLF2	YCR086W	CSM1
YEL065W	SIT1	YGR104C	SRB5
YPL129W	TAF14	YBL075C	SSA3
YGL212W	VAM7	YIL133C	RPL16A
YLR381W	CTF3	YPL057C	SUR1
YPR018W	RLF2	YDR181C	SAS4
YMR055C	BUB2	YBR036C	CSG2
YDL226C	GCS1	YKL114C	APN1
YDL116W	NUP84	YDR076W	RAD55
YCL037C	SRO9	YNL297C	MON2
YDR443C	SSN2	YNL076W	MKS1
YOR059C	YOR059C	YKL094W	YJU3
YBL058W	SHP1	YNL307C	MCK1
YJR060W	CBF1	YDL226C	GCS1
YPL022W	RAD1	YML095C	RAD10
YOL111C	MDY2	YMR214W	SCJ1
YMR055C	BUB2	YOR014W	RTS1
YPR040W	TIP41	YOR327C	SNC2

Predictions at threshold level 0.75 continued...			
Gene1 of predicted pair		Gene2 of predicted pair	
Systematic Name	Standard Name	Systematic Name	Standard Name
YGL200C	EMP24	YML012W	ERV25
YML095C	RAD10	YJR135C	MCM22
YER116C	SLX8	YHR010W	RPL27A
YIL036W	CST6	YDL006W	PTC1
YOL111C	MDY2	YDR261C	EXG2
YBL101C	ECM21	YBL025W	RRN10
YJR084W	CSN12	YDR310C	SUM1
YGL124C	MON1	YCR053W	THR4
YDR477W	SNF1	YER096W	SHC1
YOR140W	SFL1	YDR207C	UME6
YDR358W	GGA1	YMR214W	SCJ1
YDL002C	NHP10	YEL013W	VAC8
YPR018W	RLF2	YOR033C	EXO1
YHR060W	VMA22	YNR032W	PPG1
YKL048C	ELM1	YHR115C	DMA1
YOL126C	MDH2	YKL117W	SBA1
YDR101C	ARX1	YBR267W	REI1
YNL215W	IES2	YDR254W	CHL4
YPL037C	EGD1	YOR327C	SNC2
YGL019W	CKB1	YLR350W	ORM2
YKL110C	KTI12	YLR182W	SWI6
YBL058W	SHP1	YOR035C	SHE4
YLR043C	TRX1	YER042W	MXR1
YNL307C	MCK1	YGR270W	YTA7
YKL048C	ELM1	YKR035W-A	DID2
YHR039C-A	VMA10	YOR327C	SNC2
YHR014W	SPO13	YIL002C	INP51
YKL048C	ELM1	YML071C	COG8
YDR484W	VPS52	YOR069W	VPS5

Predictions at threshold level 0.75 continued...			
Gene1 of predicted pair		Gene2 of predicted pair	
Systematic Name	Standard Name	Systematic Name	Standard Name
YPL024W	RMI1	YML115C	VAN1
YHL031C	GOS1	YDR137W	RGP1
YDL160C	DHH1	YKR095W	MLP1
YOR337W	TEA1	YBR171W	SEC66
YGR249W	MGA1	YLR452C	SST2
YNL307C	MCK1	YOR043W	WHI2
YBR107C	IML3	YGL252C	RTG2
YPL037C	EGD1	YBL025W	RRN10
YPR051W	MAK3	YKL048C	ELM1
YPL042C	SSN3	YDL002C	NHP10
YNR072W	HXT17	YER044C	ERG28
YNL031C	HHT2	YBL002W	HTB2
YDR254W	CHL4	YJR060W	CBF1
YKR048C	NAP1	YBR010W	HHT1
YOR066W	MSA1	YNL041C	COG6
YMR199W	CLN1	YPL161C	BEM4
YGR071C	YGR071C	YHR167W	THP2
YMR224C	MRE11	YJL124C	LSM1
YER122C	GLO3	YKR030W	GMH1
YGL178W	MPT5	YBR171W	SEC66
YLR386W	VAC14	YEL060C	PRB1
YMR116C	ASC1	YJL117W	PHO86
YJL127C	SPT10	YLR410W	VIP1
YBL047C	EDE1	YJL006C	CTK2
YGL223C	COG1	YEL043W	YEL043W
YDL006W	PTC1	YOL018C	TLG2
YGR105W	VMA21	YER122C	GLO3
YGR019W	UGA1	YBR171W	SEC66
YNL032W	SIW14	YER162C	RAD4

Predictions at threshold level 0.75 continued...			
Gene1 of predicted pair		Gene2 of predicted pair	
Systematic Name	Standard Name	Systematic Name	Standard Name
YBL008W	HIR1	YJL176C	SWI3
YDL020C	RPN4	YNL206C	RTT106
YDR484W	VPS52	YOR066W	MSA1
YCR065W	HCM1	YOL081W	IRA2
YNL106C	INP52	YML103C	NUP188
YOR132W	VPS17	YHL002W	HSE1
YNL087W	TCB2	YEL060C	PRB1
YOR043W	WHI2	YOL081W	IRA2
YAL024C	LTE1	YPL137C	GIP3
YLL039C	UBI4	YPL213W	LEA1
YBR173C	UMP1	YPL137C	GIP3
YJR140C	HIR3	YDR363W-A	SEM1
YBR114W	RAD16	YNL107W	YAF9
YNL069C	RPL16B	YJL036W	SNX4
YMR199W	CLN1	YKL161C	YKL161C
YAL024C	LTE1	YPL037C	EGD1
YNL180C	RHO5	YPL240C	HSP82
YJL095W	BCK1	YPR119W	CLB2
YDR289C	RTT103	YLR254C	NDL1
YLR094C	GIS3	YBR019C	GAL10
YMR055C	BUB2	YLR315W	NKP2
YPR029C	APL4	YDR153C	ENT5
YNL127W	FAR11	YDR153C	ENT5
YLR234W	TOP3	YGL163C	RAD54
YDL223C	HBT1	YER040W	GLN3
YKL139W	CTK1	YPL022W	RAD1
YIL076W	SEC28	YOR043W	WHI2
YAL030W	SNC1	YPR032W	SRO7
YNL325C	FIG4	YBL025W	RRN10

Predictions at threshold level 0.75 continued...			
Gene1 of predicted pair		Gene2 of predicted pair	
Systematic Name	Standard Name	Systematic Name	Standard Name
YJL176C	SWI3	YGL215W	CLG1
YLR362W	STE11	YBL085W	BOI1
YPL042C	SSN3	YOL051W	GAL11
YPL152W	RRD2	YGL163C	RAD54
YBR036C	CSG2	YOL081W	IRA2
YPL105C	YPL105C	YJR060W	CBF1
YKR101W	SIR1	YDL042C	SIR2
YLR398C	SKI2	YMR214W	SCJ1
YHR178W	STB5	YNL107W	YAF9
YMR199W	CLN1	YNL229C	URE2
YOR337W	TEA1	YDR137W	RGP1
YBR264C	YPT10	YNL044W	YIP3
YDR310C	SUM1	YAL002W	VPS8
YPL046C	ELC1	YBR114W	RAD16
YPL129W	TAF14	YBR289W	SNF5
YGR184C	UBR1	YDR363W-A	SEM1
YAL024C	LTE1	YLR234W	TOP3
YGR108W	CLB1	YDR144C	MKC7
YNL117W	MLS1	YJL095W	BCK1
YPL161C	BEM4	YLR309C	IMH1
YMR224C	MRE11	YOL111C	MDY2
YMR109W	MYO5	YDR310C	SUM1
YLR441C	RPS1A	YPL161C	BEM4
YJL124C	LSM1	YNL016W	PUB1
YDL223C	HBT1	YDR261C	EXG2
YJR040W	GEF1	YNR032W	PPG1
YLR320W	MMS22	YMR152W	YIM1
YLR337C	VRP1	YDL155W	CLB3
YNL069C	RPL16B	YKR001C	VPS1

Predictions at threshold level 0.75 continued...			
Gene1 of predicted pair		Gene2 of predicted pair	
Systematic Name	Standard Name	Systematic Name	Standard Name
YGL178W	MPT5	YMR263W	SAP30
YDR483W	KRE2	YBR171W	SEC66
YPR131C	NAT3	YNL236W	SIN4
YKL139W	CTK1	YMR133W	REC114
YJL124C	LSM1	YCL037C	SRO9
YOR101W	RAS1	YNL079C	TPM1
YGL200C	EMP24	YFL048C	EMP47
YER162C	RAD4	YOL081W	IRA2
YGL094C	PAN2	YKL114C	APN1
YDR293C	SSD1	YJR032W	CPR7
YNL215W	IES2	YGL151W	NUT1
YMR054W	STV1	YEL051W	VMA8
YOR069W	VPS5	YHL020C	OPI1
YOL044W	PEX15	YGL153W	PEX14
YER144C	UBP5	YPL195W	APL5
YOR043W	WHI2	YGL115W	SNF4
YBR274W	CHK1	YGL250W	RMR1
YHR152W	SPO12	YKL032C	IXR1
YBR217W	ATG12	YCR053W	THR4
YDL077C	VAM6	YJR033C	RAV1
YOR124C	UBP2	YLR182W	SWI6
YKR014C	YPT52	YEL036C	ANP1
YBR010W	HHT1	YNL030W	HHF2
YCL001W	RER1	YEL053C	MAK10
YNR052C	POP2	YLR182W	SWI6
YOR132W	VPS17	YJR060W	CBF1
YDR174W	HMO1	YJR135C	MCM22
YMR095C	SNO1	YML115C	VAN1
YOR023C	AHC1	YDR310C	SUM1

Predictions at threshold level 0.75 continued...			
Gene1 of predicted pair		Gene2 of predicted pair	
Systematic Name	Standard Name	Systematic Name	Standard Name
YDR108W	GSG1	YKR001C	VPS1
YLR234W	TOP3	YBR200W	BEM1
YBR274W	CHK1	YNL234W	YNL234W
YEL065W	SIT1	YOR270C	VPH1
YFR040W	SAP155	YLR452C	SST2
YDR484W	VPS52	YDR207C	UME6
YLR093C	NYV1	YOR327C	SNC2
YCL067C	HMLALPHA2	YGR252W	GCN5
YDR477W	SNF1	YMR016C	SOK2
YOR043W	WHI2	YHR039C-A	VMA10
YBR228W	SLX1	YJL124C	LSM1
YKR035W-A	DID2	YLR452C	SST2
YGL212W	VAM7	YDL226C	GCS1
YDR477W	SNF1	YIL076W	SEC28
YNL307C	MCK1	YOR035C	SHE4
YDR320C	SWA2	YPL234C	TFP3
YLR309C	IMH1	YDR207C	UME6
YKR072C	SIS2	YJR090C	GRR1
YOR132W	VPS17	YMR004W	MVP1
YBL103C	RTG3	YBR171W	SEC66
YFL013C	IES1	YJL124C	LSM1
YPR030W	CSR2	YJL095W	BCK1
YKL126W	YPK1	YMR133W	REC114
YPL022W	RAD1	YJL006C	CTK2
YOR112W	CEX1	YOL108C	INO4
YHR060W	VMA22	YKR020W	VPS51
YLR090W	XDJ1	YFR031C-A	RPL2A
YGR054W	YGR054W	YOL067C	RTG1
YML115C	VAN1	YPR046W	MCM16

Predictions at threshold level 0.75 continued...			
Gene1 of predicted pair		Gene2 of predicted pair	
Systematic Name	Standard Name	Systematic Name	Standard Name
YKR072C	SIS2	YJR035W	RAD26
YJL154C	VPS35	YHL020C	OPI1
YPL037C	EGD1	YOL081W	IRA2
YDL184C	RPL41A	YOR035C	SHE4
YML115C	VAN1	YCR031C	RPS14A
YBR217W	ATG12	YJR084W	CSN12
YGL033W	HOP2	YER139C	YER139C
YMR216C	SKY1	YNL238W	KEX2
YDL020C	RPN4	YDL059C	RAD59
YJL124C	LSM1	YLR254C	NDL1
YNL325C	FIG4	YPR030W	CSR2
YNL087W	TCB2	YJL183W	MNN11
YDR108W	GSG1	YAR014C	BUD14
YOR132W	VPS17	YEL043W	YEL043W
YCR086W	CSM1	YGL031C	RPL24A
YER107C	GLE2	YMR223W	UBP8
YDR123C	INO2	YHL020C	OPI1
YOR096W	RPS7A	YOR078W	BUD21
YER110C	KAP123	YDR310C	SUM1
YOR127W	RGA1	YEL051W	VMA8
YPL152W	RRD2	YPL018W	CTF19
YLR360W	VPS38	YDR153C	ENT5
YLR447C	VMA6	YJL095W	BCK1
YMR224C	MRE11	YML103C	NUP188
YHR039C-A	VMA10	YPL137C	GIP3
YBL008W	HIR1	YHL025W	SNF6
YHR013C	ARD1	YBR181C	RPS6B
YDR137W	RGP1	YER044C	ERG28
YGR020C	VMA7	YPL195W	APL5

Predictions at threshold level 0.75 continued...			
Gene1 of predicted pair		Gene2 of predicted pair	
Systematic Name	Standard Name	Systematic Name	Standard Name
YHR111W	UBA4	YPL161C	BEM4
YLR006C	SSK1	YCR077C	PAT1
YDL020C	RPN4	YLR288C	MEC3
YNL117W	MLS1	YML028W	TSA1
YMR164C	MSS11	YPL178W	CBC2
YBL103C	RTG3	YNL076W	MKS1
YDR443C	SSN2	YBL025W	RRN10
YDR448W	ADA2	YFR034C	PHO4
YDR254W	CHL4	YOL081W	IRA2
YOR337W	TEA1	YPR036W	VMA13
YCL024W	KCC4	YKL101W	HSL1
YOR109W	INP53	YDR310C	SUM1
YGL133W	ITC1	YOR127W	RGA1
YBR200W	BEM1	YBR127C	VMA2
YGL175C	SAE2	YMR231W	PEP5
YGL115W	SNF4	YNL107W	YAF9
YDR245W	MNN10	YHR108W	GGA2
YLR399C	BDF1	YPR046W	MCM16
YNL054W	VAC7	YLR315W	NKP2
YOL086C	ADH1	YPR119W	CLB2
YJL172W	CPS1	YKR035W-A	DID2
YGL078C	DBP3	YPL195W	APL5
YFR036W	CDC26	YGL124C	MON1
YPL024W	RMI1	YCR053W	THR4
YPL253C	VIK1	YML112W	CTK3
YEL027W	CUP5	YNL238W	KEX2
YIL034C	CAP2	YLR429W	CRN1
YJL095W	BCK1	YLR398C	SKI2
YDR004W	RAD57	YKL068W	NUP100

Predictions at threshold level 0.75 continued...			
Gene1 of predicted pair		Gene2 of predicted pair	
**Systematic Name**	**Standard Name**	**Systematic Name**	**Standard Name**
YJR084W	CSN12	YCR053W	THR4
YGR249W	MGA1	YBR036C	CSG2
YJL154C	VPS35	YAR014C	BUD14
YOR290C	SNF2	YPL137C	GIP3
YMR026C	PEX12	YMR230W	RPS10B
YHR073W	OSH3	YJR024C	MDE1
YGL135W	RPL1B	YML071C	COG8
YDR108W	GSG1	YEL051W	VMA8
YDR171W	HSP42	YJR135C	MCM22
YPR052C	NHP6A	YDR448W	ADA2
YPR072W	NOT5	YGL094C	PAN2
YNL325C	FIG4	YBR019C	GAL10
YGL223C	COG1	YJL029C	VPS53
YJL164C	TPK1	YAL030W	SNC1
YER116C	SLX8	YJL172W	CPS1
YMR016C	SOK2	YPR046W	MCM16
YJL053W	PEP8	YDL226C	GCS1
YJL187C	SWE1	YDL226C	GCS1
YMR095C	SNO1	YBL058W	SHP1
YJR060W	CBF1	YDR183W	PLP1
YLR288C	MEC3	YNL297C	MON2
YDL002C	NHP10	YLR350W	ORM2
YER162C	RAD4	YLR309C	IMH1
YGR249W	MGA1	YML071C	COG8
YPL024W	RMI1	YBR108W	YBR108W
YBL103C	RTG3	YPR046W	MCM16
YDR358W	GGA1	YGL003C	CDH1
YHR134W	WSS1	YDR144C	MKC7
YJL078C	PRY3	YMR319C	FET4

Predictions at threshold level 0.75 continued...			
Gene1 of predicted pair		Gene2 of predicted pair	
Systematic Name	Standard Name	Systematic Name	Standard Name
YDR277C	MTH1	YPR119W	CLB2
YGR217W	CCH1	YDL006W	PTC1
YHR111W	UBA4	YJR135C	MCM22
YBR173C	UMP1	YDL047W	SIT4
YHR178W	STB5	YEL056W	HAT2
YBL103C	RTG3	YDL083C	RPS16B
YKR059W	TIF1	YJR140C	HIR3
YPL161C	BEM4	YDR358W	GGA1
YPL115C	BEM3	YLR386W	VAC14
YML115C	VAN1	YPL195W	APL5
YKR061W	KTR2	YML112W	CTK3
YKL006W	RPL14A	YDR440W	DOT1
YCR024C-A	PMP1	YPL246C	RBD2
YLR234W	TOP3	YBR195C	MSI1
YJR052W	RAD7	YOR028C	CIN5
YMR016C	SOK2	YML001W	YPT7
YDR254W	CHL4	YLR254C	NDL1
YBL058W	SHP1	YJL183W	MNN11
YPR036W	VMA13	YGL163C	RAD54
YKL126W	YPK1	YLR180W	SAM1
YEL065W	SIT1	YBR200W	BEM1
YKL139W	CTK1	YPL137C	GIP3
YGL212W	VAM7	YNL238W	KEX2
YDR156W	RPA14	YKR020W	VPS51
YKL010C	UFD4	YMR116C	ASC1
YLR371W	ROM2	YBR019C	GAL10
YLR337C	VRP1	YDR245W	MNN10
YLR090W	XDJ1	YHR060W	VMA22
YPL234C	TFP3	YER122C	GLO3

Predictions at threshold level 0.75 continued...			
Gene1 of predicted pair		Gene2 of predicted pair	
Systematic Name	Standard Name	Systematic Name	Standard Name
YDL020C	RPN4	YML115C	VAN1
YHR178W	STB5	YJR140C	HIR3
YNL041C	COG6	YDL077C	VAM6
YMR116C	ASC1	YGL175C	SAE2
YDR448W	ADA2	YOL051W	GAL11
YML016C	PPZ1	YJL124C	LSM1
YLR360W	VPS38	YOR106W	VAM3
YNL058C	YNL058C	YOR035C	SHE4
YEL013W	VAC8	YER122C	GLO3
YOL030W	GAS5	YJL124C	LSM1
YJR060W	CBF1	YPL234C	TFP3
YMR055C	BUB2	YJR005W	APL1
YJL036W	SNX4	YPL161C	BEM4
YHR012W	VPS29	YEL036C	ANP1
YDR155C	CPR1	YJL095W	BCK1
YOR358W	HAP5	YGR059W	SPR3
YGR249W	MGA1	YEL060C	PRB1
YDR443C	SSN2	YER139C	YER139C
YPR171W	BSP1	YBR126C	TPS1
YGL175C	SAE2	YDR207C	UME6
YLR234W	TOP3	YHR111W	UBA4
YNL199C	GCR2	YHR012W	VPS29
YBR215W	HPC2	YNL107W	YAF9
YHR081W	LRP1	YML071C	COG8
YIL076W	SEC28	YPL105C	YPL105C
YLR337C	VRP1	YGR270W	YTA7
YPR189W	SKI3	YLR398C	SKI2
YML038C	YMD8	YNL107W	YAF9
YIL076W	SEC28	YBR217W	ATG12

Predictions at threshold level 0.75 continued...			
Gene1 of predicted pair		Gene2 of predicted pair	
Systematic Name	Standard Name	Systematic Name	Standard Name
YGL163C	RAD54	YGL148W	ARO2
YPL105C	YPL105C	YEL053C	MAK10
YNL199C	GCR2	YPL246C	RBD2
YBR089C-A	NHP6B	YPL248C	GAL4
YKR099W	BAS1	YNL058C	YNL058C
YMR313C	TGL3	YMR152W	YIM1
YDR129C	SAC6	YJL029C	VPS53
YKL126W	YPK1	YKR035W-A	DID2
YLR386W	VAC14	YLR254C	NDL1
YJL006C	CTK2	YML112W	CTK3
YDL083C	RPS16B	YEL060C	PRB1
YDR379W	RGA2	YOR275C	RIM20
YBL051C	PIN4	YOR023C	AHC1
YNR052C	POP2	YDL155W	CLB3
YEL053C	MAK10	YBR280C	SAF1
YIR005W	IST3	YPL018W	CTF19
YNL243W	SLA2	YCR009C	RVS161
YIL076W	SEC28	YOR308C	SNU66
YPL042C	SSN3	YDR363W-A	SEM1
YBR290W	BSD2	YJR032W	CPR7
YOR275C	RIM20	YHR200W	RPN10
YDL020C	RPN4	YLR024C	UBR2
YLR452C	SST2	YGL115W	SNF4
YJR056C	YJR056C	YGL124C	MON1
YML001W	YPT7	YOR066W	MSA1
YHR108W	GGA2	YLR306W	UBC12
YHR060W	VMA22	YOR043W	WHI2
YFR036W	CDC26	YBR019C	GAL10
YPR084W	YPR084W	YMR004W	MVP1

Predictions at threshold level 0.75 continued...			
Gene1 of predicted pair		Gene2 of predicted pair	
Systematic Name	Standard Name	Systematic Name	Standard Name
YPL057C	SUR1	YCL037C	SRO9
YMR224C	MRE11	YML112W	CTK3
YIL076W	SEC28	YER040W	GLN3
YML001W	YPT7	YMR054W	STV1
YDL184C	RPL41A	YHR039C-A	VMA10
YGL078C	DBP3	YGL025C	PGD1
YBR173C	UMP1	YBR030W	YBR030W
YBL101C	ECM21	YMR089C	YTA12
YBR019C	GAL10	YMR214W	SCJ1
YNL201C	PSY2	YBR200W	BEM1
YCL037C	SRO9	YEL013W	VAC8
YOR140W	SFL1	YER049W	TPA1
YMR116C	ASC1	YBR200W	BEM1
YLR386W	VAC14	YJL190C	RPS22A
YGL237C	HAP2	YPL161C	BEM4
YHR156C	LIN1	YER122C	GLO3
YPR120C	CLB5	YGL148W	ARO2
YOR043W	WHI2	YGL163C	RAD54
YOL111C	MDY2	YGL115W	SNF4
YER116C	SLX8	YML071C	COG8
YBR194W	SOY1	YOR038C	HIR2
YIL159W	BNR1	YBL058W	SHP1
YPL001W	HAT1	YGL148W	ARO2
YMR116C	ASC1	YEL044W	IES6
YMR055C	BUB2	YML112W	CTK3
YIL076W	SEC28	YOR080W	DIA2
YFR030W	MET10	YNL225C	CNM67
YGL078C	DBP3	YHR200W	RPN10
YJL210W	PEX2	YLR191W	PEX13

Predictions at threshold level 0.75 continued...			
Gene1 of predicted pair		Gene2 of predicted pair	
Systematic Name	Standard Name	Systematic Name	Standard Name
YPR052C	NHP6A	YNL031C	HHT2
YAL024C	LTE1	YHR178W	STB5
YHR013C	ARD1	YDL155W	CLB3
YNL166C	BNI5	YIL008W	URM1
YIL159W	BNR1	YJL183W	MNN11
YKR020W	VPS51	YPL045W	VPS16
YIL076W	SEC28	YMR311C	GLC8
YOR337W	TEA1	YMR153W	NUP53
YBR274W	CHK1	YMR055C	BUB2
YKL126W	YPK1	YOL111C	MDY2
YFL023W	BUD27	YBR036C	CSG2
YIL159W	BNR1	YGR249W	MGA1
YLR234W	TOP3	YMR055C	BUB2
YNL054W	VAC7	YJL006C	CTK2
YGL033W	HOP2	YFR010W	UBP6
YGL060W	YBP2	YGL003C	CDH1
YGL212W	VAM7	YPL120W	VPS30
YLR399C	BDF1	YDR183W	PLP1
YLR381W	CTF3	YNL116W	DMA2
YDR225W	HTA1	YPL018W	CTF19
YCR020C-A	MAK31	YHR012W	VPS29
YHR013C	ARD1	YML028W	TSA1
YNL288W	CAF40	YOR023C	AHC1
YLL039C	UBI4	YHL002W	HSE1
YMR284W	YKU70	YBL019W	APN2
YPR049C	ATG11	YKL103C	LAP4
YBR108W	YBR108W	YOR066W	MSA1
YMR153W	NUP53	YLR094C	GIS3
YDR378C	LSM6	YOL081W	IRA2

Predictions at threshold level 0.75 continued...			
Gene1 of predicted pair		Gene2 of predicted pair	
Systematic Name	Standard Name	Systematic Name	Standard Name
YBL047C	EDE1	YHR158C	KEL1
YKL139W	CTK1	YGL025C	PGD1
YLR386W	VAC14	YHR039C-A	VMA10
YCR020C-A	MAK31	YPL037C	EGD1
YML095C	RAD10	YGR188C	BUB1
YPL253C	VIK1	YJL190C	RPS22A
YKR061W	KTR2	YBR107C	IML3
YGL237C	HAP2	YPL018W	CTF19
YDR103W	STE5	YGR152C	RSR1
YEL065W	SIT1	YPL161C	BEM4
YNL069C	RPL16B	YGR105W	VMA21
YPL203W	TPK2	YIL033C	BCY1
YMR004W	MVP1	YPL105C	YPL105C
YGL147C	RPL9A	YGL003C	CDH1
YHL031C	GOS1	YDR335W	MSN5
YNL248C	RPA49	YDR335W	MSN5
YLR410W	VIP1	YPL018W	CTF19
YOR076C	SKI7	YGR071C	YGR071C
YBR057C	MUM2	YBL047C	EDE1
YLR386W	VAC14	YJR060W	CBF1
YGL087C	MMS2	YLR360W	VPS38
YJR140C	HIR3	YNL199C	GCR2
YLR398C	SKI2	YBR280C	SAF1
YJL078C	PRY3	YJR106W	ECM27
YJR056C	YJR056C	YHR200W	RPN10
YDR202C	RAV2	YER162C	RAD4
YJR032W	CPR7	YDR358W	GGA1
YNL032W	SIW14	YMR153W	NUP53
YGR152C	RSR1	YDR310C	SUM1

Predictions at threshold level 0.75 continued...			
Gene1 of predicted pair		Gene2 of predicted pair	
Systematic Name	Standard Name	Systematic Name	Standard Name
YKR095W	MLP1	YMR273C	ZDS1
YIL076W	SEC28	YJR033C	RAV1
YHR134W	WSS1	YDR310C	SUM1
YFR031C-A	RPL2A	YNL069C	RPL16B
YJR063W	RPA12	YLR254C	NDL1
YJL124C	LSM1	YHR111W	UBA4
YDR484W	VPS52	YDL192W	ARF1
YJL036W	SNX4	YNL107W	YAF9
YNL264C	PDR17	YDR363W-A	SEM1
YJR125C	ENT3	YOR327C	SNC2
YDR261C	EXG2	YBL025W	RRN10
YNL079C	TPM1	YHL002W	HSE1
YBR082C	UBC4	YAL002W	VPS8
YNL215W	IES2	YLR399C	BDF1
YNL201C	PSY2	YLR399C	BDF1
YJL176C	SWI3	YNL106C	INP52
YMR231W	PEP5	YGL095C	VPS45
YBR126C	TPS1	YDR207C	UME6
YGR270W	YTA7	YGL253W	HXK2
YNL218W	MGS1	YOL090W	MSH2
YLR410W	VIP1	YBR082C	UBC4
YNL201C	PSY2	YLR079W	SIC1
YGL147C	RPL9A	YJR032W	CPR7
YDL047W	SIT4	YAL030W	SNC1
YDR137W	RGP1	YAL055W	PEX22
YNL094W	APP1	YBL106C	SRO77
YPR046W	MCM16	YOL067C	RTG1
YFR031C-A	RPL2A	YMR133W	REC114
YOR023C	AHC1	YDR318W	MCM21

Predictions at threshold level 0.75 continued...			
Gene1 of predicted pair		Gene2 of predicted pair	
Systematic Name	Standard Name	Systematic Name	Standard Name
YHR039C-A	VMA10	YLR309C	IMH1
YNL297C	MON2	YDR440W	DOT1
YPR029C	APL4	YKL135C	APL2
YIR005W	IST3	YEL013W	VAC8
YLR273C	PIG1	YLR258W	GSY2
YNL139C	RLR1	YKL139W	CTK1
YLR410W	VIP1	YPR046W	MCM16
YBL058W	SHP1	YBR019C	GAL10
YBL058W	SHP1	YLR192C	HCR1
YMR153W	NUP53	YMR230W	RPS10B
YPR036W	VMA13	YJL183W	MNN11
YLR410W	VIP1	YHR115C	DMA1
YMR004W	MVP1	YER122C	GLO3
YDR293C	SSD1	YCR077C	PAT1
YOR043W	WHI2	YGL124C	MON1
YDR202C	RAV2	YCL001W	RER1
YLR386W	VAC14	YOL067C	RTG1
YMR127C	SAS2	YHR012W	VPS29
YOL111C	MDY2	YJR032W	CPR7
YPL137C	GIP3	YOL081W	IRA2
YHR205W	SCH9	YHR030C	SLT2
YML016C	PPZ1	YDR378C	LSM6
YBR057C	MUM2	YPL253C	VIK1
YBR057C	MUM2	YHR013C	ARD1
YJL187C	SWE1	YGL031C	RPL24A
YNL031C	HHT2	YMR273C	ZDS1
YKR057W	RPS21A	YKL032C	IXR1
YHR111W	UBA4	YJL006C	CTK2
YBR065C	ECM2	YGR006W	PRP18

Predictions at threshold level 0.75 continued...			
Gene1 of predicted pair		Gene2 of predicted pair	
Systematic Name	Standard Name	Systematic Name	Standard Name
YER049W	TPA1	YDR207C	UME6
YPL256C	CLN2	YOR368W	RAD17
YLR313C	SPH1	YML028W	TSA1
YFR010W	UBP6	YLR370C	ARC18
YPR051W	MAK3	YGR105W	VMA21
YNR072W	HXT17	YLR320W	MMS22
YGL147C	RPL9A	YDR484W	VPS52
YGL135W	RPL1B	YAL055W	PEX22
YNL044W	YIP3	YJR033C	RAV1
YKR072C	SIS2	YBR195C	MSI1
YER139C	YER139C	YLR350W	ORM2
YDL160C	DHH1	YKL114C	APN1
YBR108W	YBR108W	YDL077C	VAM6
YMR153W	NUP53	YNR072W	HXT17
YOR001W	RRP6	YPL018W	CTF19
YBL102W	SFT2	YHL031C	GOS1
YLR315W	NKP2	YOR124C	UBP2
YLR119W	SRN2	YBR108W	YBR108W
YDR363W-A	SEM1	YFR036W	CDC26
YML097C	VPS9	YBR181C	RPS6B
YDR448W	ADA2	YDR216W	ADR1
YMR026C	PEX12	YGR249W	MGA1
YER096W	SHC1	YMR055C	BUB2
YBL102W	SFT2	YGL212W	VAM7
YBL021C	HAP3	YPL253C	VIK1
YDL184C	RPL41A	YPL195W	APL5
YJL060W	BNA3	YNL236W	SIN4
YDR137W	RGP1	YNL051W	COG5
YDR293C	SSD1	YIL038C	NOT3

Predictions at threshold level 0.75 continued...			
Gene1 of predicted pair		Gene2 of predicted pair	
Systematic Name	Standard Name	Systematic Name	Standard Name
YPL022W	RAD1	YPL106C	SSE1
YFR031C-A	RPL2A	YDL006W	PTC1
YCR086W	CSM1	YNL225C	CNM67
YLR090W	XDJ1	YOR035C	SHE4
YOR023C	AHC1	YCR086W	CSM1
YGL066W	SGF73	YLR102C	APC9
YDR129C	SAC6	YER122C	GLO3
YMR142C	RPL13B	YNL238W	KEX2
YJL176C	SWI3	YDR440W	DOT1
YGL174W	BUD13	YJR050W	ISY1
YDL013W	SLX5	YOR368W	RAD17
YKR001C	VPS1	YKR030W	GMH1
YJR033C	RAV1	YAL002W	VPS8
YML095C	RAD10	YJL006C	CTK2
YNL106C	INP52	YHR167W	THP2
YOR001W	RRP6	YDR289C	RTT103
YDR456W	NHX1	YNL079C	TPM1
YLR119W	SRN2	YOL111C	MDY2
YNL087W	TCB2	YOL121C	RPS19A
YJL136C	RPS21B	YGR135W	PRE9
YPL024W	RMI1	YLR386W	VAC14
YER144C	UBP5	YDL223C	HBT1
YBR171W	SEC66	YLR371W	ROM2
YML103C	NUP188	YHR115C	DMA1
YML071C	COG8	YMR152W	YIM1
YBR041W	FAT1	YKL048C	ELM1
YAL024C	LTE1	YNL293W	MSB3
YNL087W	TCB2	YJL172W	CPS1
YNL229C	URE2	YNL116W	DMA2

Predictions at threshold level 0.75 continued...			
Gene1 of predicted pair		Gene2 of predicted pair	
Systematic Name	Standard Name	Systematic Name	Standard Name
YKR035W-A	DID2	YMR152W	YIM1
YDR318W	MCM21	YGL252C	RTG2
YJL172W	CPS1	YPL195W	APL5
YNR052C	POP2	YNL307C	MCK1
YDR080W	VPS41	YML071C	COG8
YDR363W-A	SEM1	YGL003C	CDH1
YLR441C	RPS1A	YER044C	ERG28
YPL161C	BEM4	YMR089C	YTA12
YLR381W	CTF3	YDR260C	SWM1
YML103C	NUP188	YIL103W	DPH1
YLR119W	SRN2	YNL307C	MCK1
YNL058C	YNL058C	YDR261C	EXG2
YMR004W	MVP1	YDR080W	VPS41
YDR171W	HSP42	YPL018W	CTF19
YHR178W	STB5	YER110C	KAP123
YLR452C	SST2	YLR398C	SKI2
YBR270C	BIT2	YIL105C	SLM1
YHR200W	RPN10	YPR046W	MCM16
YDR004W	RAD57	YER161C	SPT2
YNL069C	RPL16B	YPR051W	MAK3
YGR241C	YAP1802	YKR020W	VPS51
YKL101W	HSL1	YGL003C	CDH1
YBL058W	SHP1	YBL103C	RTG3
YJL006C	CTK2	YGL003C	CDH1
YEL065W	SIT1	YNL325C	FIG4
YKR059W	TIF1	YML016C	PPZ1
YDL116W	NUP84	YMR125W	STO1
YGL174W	BUD13	YNL107W	YAF9
YDR293C	SSD1	YHR086W	NAM8

Predictions at threshold level 0.75 continued...			
Gene1 of predicted pair		Gene2 of predicted pair	
Systematic Name	Standard Name	Systematic Name	Standard Name
YPL024W	RMI1	YOR035C	SHE4
YOR327C	SNC2	YAR014C	BUD14
YBR171W	SEC66	YPL050C	MNN9
YPR049C	ATG11	YPR119W	CLB2
YJL006C	CTK2	YJL183W	MNN11
YLR320W	MMS22	YML071C	COG8
YJL060W	BNA3	YLR079W	SIC1
YMR039C	SUB1	YBR107C	IML3
YBR020W	GAL1	YHR200W	RPN10
YBL103C	RTG3	YPR036W	VMA13
YPL161C	BEM4	YLR371W	ROM2
YGR249W	MGA1	YKR099W	BAS1
YDR293C	SSD1	YOR109W	INP53
YJR024C	MDE1	YIL148W	RPL40A
YPL057C	SUR1	YDR137W	RGP1
YOR023C	AHC1	YPL248C	GAL4
YDR192C	NUP42	YER110C	KAP123
YNL264C	PDR17	YNL236W	SIN4
YLR079W	SIC1	YLR381W	CTF3
YPL158C	YPL158C	YHR158C	KEL1
YJL124C	LSM1	YGL151W	NUT1
YGL025C	PGD1	YJL095W	BCK1
YNL307C	MCK1	YPL161C	BEM4
YGR019W	UGA1	YHR114W	BZZ1
YOR080W	DIA2	YCR086W	CSM1
YML115C	VAN1	YML097C	VPS9
YLR452C	SST2	YML001W	YPT7
YPR051W	MAK3	YDR289C	RTT103
YMR142C	RPL13B	YML001W	YPT7

Predictions at threshold level 0.75 continued...			
Gene1 of predicted pair		Gene2 of predicted pair	
Systematic Name	Standard Name	Systematic Name	Standard Name
YMR016C	SOK2	YGL124C	MON1
YER044C	ERG28	YBL025W	RRN10
YKL126W	YPK1	YDL083C	RPS16B
YNL068C	FKH2	YIL008W	URM1
YMR133W	REC114	YHL022C	SPO11
YBR082C	UBC4	YBR181C	RPS6B
YPR018W	RLF2	YLR381W	CTF3
YML007W	YAP1	YER110C	KAP123
YGL212W	VAM7	YNL051W	COG5
YMR224C	MRE11	YJR035W	RAD26
YDL059C	RAD59	YML028W	TSA1
YNR072W	HXT17	YGL124C	MON1
YPR084W	YPR084W	YKR001C	VPS1
YLR381W	CTF3	YGL115W	SNF4
YLR452C	SST2	YFR036W	CDC26
YJL154C	VPS35	YNL127W	FAR11
YJL210W	PEX2	YDR142C	PEX7
YHR013C	ARD1	YOR014W	RTS1
YER162C	RAD4	YPR032W	SRO7
YOR080W	DIA2	YNL236W	SIN4
YJL006C	CTK2	YHR115C	DMA1
YJL127C	SPT10	YJR060W	CBF1
YPL057C	SUR1	YNL297C	MON2
YJL172W	CPS1	YJR056C	YJR056C
YOR279C	RFM1	YDR310C	SUM1
YPL253C	VIK1	YHR013C	ARD1
YPR018W	RLF2	YDR260C	SWM1
YGL025C	PGD1	YDL077C	VAM6
YDL077C	VAM6	YBR036C	CSG2

Predictions at threshold level 0.75 continued...			
Gene1 of predicted pair		Gene2 of predicted pair	
Systematic Name	Standard Name	Systematic Name	Standard Name
YLR093C	NYV1	YPL195W	APL5
YJR056C	YJR056C	YJR032W	CPR7
YDR507C	GIN4	YDR335W	MSN5
YGL009C	LEU1	YPR119W	CLB2
YLR234W	TOP3	YOL090W	MSH2
YDR174W	HMO1	YKL110C	KTI12
YPL253C	VIK1	YMR038C	CCS1
YOL147C	PEX11	YDL078C	MDH3
YNL201C	PSY2	YKR061W	KTR2
YNR072W	HXT17	YMR133W	REC114
YKL006W	RPL14A	YBL101C	ECM21
YMR039C	SUB1	YPL024W	RMI1
YGR072W	UPF3	YPL195W	APL5
YGR081C	SLX9	YPL022W	RAD1
YHR039C-A	VMA10	YLR093C	NYV1
YHR171W	ATG7	YNL223W	ATG4
YKR039W	GAP1	YCL018W	LEU2
YBR195C	MSI1	YGL148W	ARO2
YLR234W	TOP3	YOR023C	AHC1
YBR173C	UMP1	YNL238W	KEX2
YIL034C	CAP2	YCR077C	PAT1
YDR174W	HMO1	YNL054W	VAC7
YMR224C	MRE11	YDL223C	HBT1
YHR012W	VPS29	YOR208W	PTP2
YKR099W	BAS1	YPL195W	APL5
YKR020W	VPS51	YBR082C	UBC4
YER002W	NOP16	YKL009W	MRT4
YHR178W	STB5	YDR155C	CPR1
YBL008W	HIR1	YBR274W	CHK1

Predictions at threshold level 0.75 continued...			
Gene1 of predicted pair		Gene2 of predicted pair	
Systematic Name	Standard Name	Systematic Name	Standard Name
YOR195W	SLK19	YNL116W	DMA2
YER027C	GAL83	YGL179C	TOS3
YMR004W	MVP1	YHL020C	OPI1
YNL297C	MON2	YDR207C	UME6
YHR156C	LIN1	YEL060C	PRB1
YDR443C	SSN2	YMR199W	CLN1
YGL115W	SNF4	YDR261C	EXG2
YKR072C	SIS2	YFL023W	BUD27
YGL178W	MPT5	YLR452C	SST2
YKL048C	ELM1	YGR188C	BUB1
YBR200W	BEM1	YHR152W	SPO12
YDR176W	NGG1	YGL252C	RTG2
YDR335W	MSN5	YLR410W	VIP1
YPR051W	MAK3	YHR114W	BZZ1
YPR036W	VMA13	YGL124C	MON1
YML071C	COG8	YNL051W	COG5
YML001W	YPT7	YPR046W	MCM16
YLR095C	IOC2	YEL053C	MAK10
YFR010W	UBP6	YNL199C	GCR2
YIL159W	BNR1	YLR441C	RPS1A
YGL078C	DBP3	YOR035C	SHE4
YLR386W	VAC14	YDL083C	RPS16B
YDR202C	RAV2	YJR040W	GEF1
YFR031C-A	RPL2A	YDR202C	RAV2
YDR439W	LRS4	YJR060W	CBF1
YGL215W	CLG1	YLR131C	ACE2
YER116C	SLX8	YNL238W	KEX2
YER001W	MNN1	YOR035C	SHE4
YGL124C	MON1	YDL192W	ARF1

Predictions at threshold level 0.75 continued...			
**Gene1 of predicted pair**		**Gene2 of predicted pair**	
**Systematic Name**	**Standard Name**	**Systematic Name**	**Standard Name**
YLR135W	SLX4	YLR313C	SPH1
YNL238W	KEX2	YER040W	GLN3
YEL053C	MAK10	YMR133W	REC114
YCR066W	RAD18	YML071C	COG8
YGL163C	RAD54	YLR182W	SWI6
YOR080W	DIA2	YER139C	YER139C
YBR057C	MUM2	YNL291C	MID1
YCR077C	PAT1	YHR167W	THP2
YDR383C	NKP1	YLR386W	VAC14
YLR192C	HCR1	YJR032W	CPR7
YAL024C	LTE1	YKR061W	KTR2
YEL065W	SIT1	YBR030W	YBR030W
YER116C	SLX8	YBL103C	RTG3
YEL050C	RML2	YMR202W	ERG2
YLL039C	UBI4	YBR083W	TEC1
YGL212W	VAM7	YER139C	YER139C
YOR106W	VAM3	YJL095W	BCK1
YLR094C	GIS3	YOR327C	SNC2
YJL151C	SNA3	YPL246C	RBD2
YML012W	ERV25	YFL048C	EMP47
YDR477W	SNF1	YNL238W	KEX2
YMR016C	SOK2	YBR019C	GAL10
YLR292C	SEC72	YJR135C	MCM22
YDR293C	SSD1	YGL212W	VAM7
YFR010W	UBP6	YMR142C	RPL13B
YCL001W	RER1	YOL108C	INO4
YGR275W	RTT102	YDR073W	SNF11
YLR095C	IOC2	YDL083C	RPS16B
YLR313C	SPH1	YDL223C	HBT1

Predictions at threshold level 0.75 continued...			
Gene1 of predicted pair		Gene2 of predicted pair	
Systematic Name	Standard Name	Systematic Name	Standard Name
YJL083W	TAX4	YIL002C	INP51
YIL053W	RHR2	YLR119W	SRN2
YKL139W	CTK1	YDR378C	LSM6
YGR104C	SRB5	YLR425W	TUS1
YLR191W	PEX13	YDL065C	PEX19
YDR378C	LSM6	YLR425W	TUS1
YKL114C	APN1	YLR032W	RAD5
YKL117W	SBA1	YBL079W	NUP170
YNL288W	CAF40	YHL002W	HSE1
YMR192W	GYL1	YNL094W	APP1
YBR173C	UMP1	YBR171W	SEC66
YOR195W	SLK19	YNL248C	RPA49
YIL103W	DPH1	YHR115C	DMA1
YLL007C	YLL007C	YHR012W	VPS29
YKL048C	ELM1	YNL229C	URE2
YBL047C	EDE1	YKR048C	NAP1
YMR224C	MRE11	YMR311C	GLC8
YBR195C	MSI1	YER161C	SPT2
YJL095W	BCK1	YOR014W	RTS1
YOR080W	DIA2	YKL114C	APN1
YPL042C	SSN3	YJR060W	CBF1
YLR396C	VPS33	YPL195W	APL5
YML001W	YPT7	YGL003C	CDH1
YJL036W	SNX4	YGR020C	VMA7
YDL020C	RPN4	YKL048C	ELM1
YDR363W-A	SEM1	YDR264C	AKR1
YBR030W	YBR030W	YLR370C	ARC18
YGR104C	SRB5	YKL213C	DOA1
YFR031C-A	RPL2A	YPL120W	VPS30

Predictions at threshold level 0.75 continued...			
Gene1 of predicted pair		Gene2 of predicted pair	
Systematic Name	Standard Name	Systematic Name	Standard Name
YNR052C	POP2	YML102W	CAC2
YGL174W	BUD13	YPR101W	SNT309
YNL025C	SSN8	YOR001W	RRP6
YMR153W	NUP53	YDR137W	RGP1
YGR040W	KSS1	YGL178W	MPT5
YLR119W	SRN2	YBR173C	UMP1
YPL057C	SUR1	YMR311C	GLC8
YJL060W	BNA3	YJL006C	CTK2
YMR153W	NUP53	YLR093C	NYV1
YKL116C	PRR1	YBR036C	CSG2
YDR192C	NUP42	YBL047C	EDE1
YDR123C	INO2	YOL108C	INO4
YBR030W	YBR030W	YDR207C	UME6
YKL006W	RPL14A	YBL047C	EDE1
YJL154C	VPS35	YER096W	SHC1
YLR090W	XDJ1	YFR036W	CDC26
YCR066W	RAD18	YOR368W	RAD17
YPL057C	SUR1	YDL077C	VAM6
YLR119W	SRN2	YKL139W	CTK1
YLR360W	VPS38	YPL256C	CLN2
YOR008C	SLG1	YNL079C	TPM1
YPL024W	RMI1	YHR081W	LRP1
YLR288C	MEC3	YBR195C	MSI1
YPL042C	SSN3	YLR350W	ORM2
YMR026C	PEX12	YOR043W	WHI2
YKR072C	SIS2	YNL215W	IES2
YMR004W	MVP1	YOR109W	INP53
YIL140W	AXL2	YCR038C	BUD5
YFR032C-A	RPL29	YLR371W	ROM2

Predictions at threshold level 0.75 continued...			
Gene1 of predicted pair		Gene2 of predicted pair	
Systematic Name	Standard Name	Systematic Name	Standard Name
YFL013C	IES1	YBR194W	SOY1
YHR156C	LIN1	YKR035W-A	DID2
YOR112W	CEX1	YJL004C	SYS1
YGR159C	NSR1	YOR375C	GDH1
YBL047C	EDE1	YHL011C	PRS3
YHR039C-A	VMA10	YNL051W	COG5
YLR381W	CTF3	YDR310C	SUM1
YML016C	PPZ1	YGR252W	GCN5
YGR019W	UGA1	YLR381W	CTF3
YCR081W	SRB8	YPL253C	VIK1
YHL031C	GOS1	YNL127W	FAR11
YML102W	CAC2	YLR352W	YLR352W
YLR386W	VAC14	YKR099W	BAS1
YLR315W	NKP2	YDR289C	RTT103
YGL212W	VAM7	YNL079C	TPM1
YKL139W	CTK1	YBR036C	CSG2
YDR477W	SNF1	YER122C	GLO3
YPR066W	UBA3	YJR099W	YUH1
YFR036W	CDC26	YOL121C	RPS19A
YJL060W	BNA3	YPL022W	RAD1
YMR224C	MRE11	YDR192C	NUP42
YIL036W	CST6	YDR378C	LSM6
YDR225W	HTA1	YOR141C	ARP8
YHR171W	ATG7	YNR049C	MSO1
YDR176W	NGG1	YOL145C	CTR9
YML115C	VAN1	YFL048C	EMP47
YJL053W	PEP8	YJR040W	GEF1
YOR127W	RGA1	YNL297C	MON2
YMR139W	RIM11	YJL106W	IME2

Predictions at threshold level 0.75 continued...			
Gene1 of predicted pair		Gene2 of predicted pair	
Systematic Name	Standard Name	Systematic Name	Standard Name
YDR477W	SNF1	YHR205W	SCH9
YMR054W	STV1	YDR310C	SUM1
YML071C	COG8	YNR032W	PPG1
YGL151W	NUT1	YDR310C	SUM1
YLR119W	SRN2	YMR152W	YIM1
YLR410W	VIP1	YIL103W	DPH1
YOR127W	RGA1	YKR001C	VPS1
YPL096C-A	ERI1	YDR388W	RVS167
YDR254W	CHL4	YHR114W	BZZ1
YNL288W	CAF40	YOR127W	RGA1
YCL001W	RER1	YPL120W	VPS30
YGL071W	AFT1	YPL240C	HSP82
YGL115W	SNF4	YLR410W	VIP1
YDL160C	DHH1	YGL190C	CDC55
YOR066W	MSA1	YDL077C	VAM6
YOR080W	DIA2	YDR440W	DOT1
YFR010W	UBP6	YOR195W	SLK19
YJL095W	BCK1	YLR370C	ARC18
YOR043W	WHI2	YBL025W	RRN10
YBR126C	TPS1	YKL204W	EAP1
YNL166C	BNI5	YOR358W	HAP5
YOR035C	SHE4	YCR053W	THR4
YDR261C	EXG2	YDR358W	GGA1
YNL307C	MCK1	YDL013W	SLX5
YDL077C	VAM6	YMR311C	GLC8
YGL200C	EMP24	YKR039W	GAP1
YJL053W	PEP8	YJL204C	RCY1
YDR171W	HSP42	YDL226C	GCS1
YNL087W	TCB2	YDL192W	ARF1

Predictions at threshold level 0.75 continued...			
Gene1 of predicted pair		Gene2 of predicted pair	
Systematic Name	Standard Name	Systematic Name	Standard Name
YGL147C	RPL9A	YNL238W	KEX2
YPL050C	MNN9	YOR312C	RPL20B
YLR337C	VRP1	YNL107W	YAF9
YOR001W	RRP6	YOR308C	SNU66
YDR484W	VPS52	YDL226C	GCS1
YBR215W	HPC2	YGL163C	RAD54
YML028W	TSA1	YGR188C	BUB1
YKL048C	ELM1	YGL179C	TOS3
YJL151C	SNA3	YBR239C	YBR239C
YJR024C	MDE1	YLR079W	SIC1
YGR270W	YTA7	YLR410W	VIP1
YHL020C	OPI1	YOL018C	TLG2
YER115C	SPR6	YMR164C	MSS11
YPL161C	BEM4	YLR425W	TUS1
YGL163C	RAD54	YJL006C	CTK2
YIL076W	SEC28	YDR289C	RTT103
YKR035W-A	DID2	YEL053C	MAK10
YHR108W	GGA2	YDL137W	ARF2
YJR032W	CPR7	YDL077C	VAM6
YDL116W	NUP84	YGR270W	YTA7
YJL183W	MNN11	YHL002W	HSE1
YDR004W	RAD57	YHR039C-A	VMA10
YJL004C	SYS1	YDL077C	VAM6
YBR240C	THI2	YKL139W	CTK1
YOR270C	VPH1	YJR033C	RAV1
YDR477W	SNF1	YNL325C	FIG4
YLR441C	RPS1A	YPL195W	APL5
YHR156C	LIN1	YDR484W	VPS52
YLR410W	VIP1	YCR086W	CSM1

Predictions at threshold level 0.75 continued...			
Gene1 of predicted pair		Gene2 of predicted pair	
Systematic Name	Standard Name	Systematic Name	Standard Name
YDR484W	VPS52	YLR094C	GIS3
YDR004W	RAD57	YLR018C	POM34
YGR133W	PEX4	YHR013C	ARD1
YOR140W	SFL1	YNL199C	GCR2
YMR224C	MRE11	YMR231W	PEP5
YGL163C	RAD54	YGL031C	RPL24A
YOR270C	VPH1	YDR137W	RGP1
YMR026C	PEX12	YLR370C	ARC18
YBR127C	VMA2	YLR396C	VPS33
YGR261C	APL6	YAL002W	VPS8
YMR022W	UBC7	YNL051W	COG5
YJR024C	MDE1	YMR273C	ZDS1
YPL161C	BEM4	YOL067C	RTG1
YMR026C	PEX12	YDR477W	SNF1
YPL057C	SUR1	YIR005W	IST3
YJR140C	HIR3	YDR440W	DOT1
YOL121C	RPS19A	YPR046W	MCM16
YCR020C-A	MAK31	YGR261C	APL6
YNL288W	CAF40	YBR108W	YBR108W
YNL215W	IES2	YNL264C	PDR17
YDR225W	HTA1	YPR119W	CLB2
YHR200W	RPN10	YLR421C	RPN13
YDL020C	RPN4	YGR054W	YGR054W
YGR072W	UPF3	YOR127W	RGA1
YBR200W	BEM1	YDR155C	CPR1
YPL140C	MKK2	YOR208W	PTP2
YNL291C	MID1	YHR200W	RPN10
YGR249W	MGA1	YOR035C	SHE4
YDR004W	RAD57	YDL020C	RPN4

Predictions at threshold level 0.75 continued...			
Gene1 of predicted pair		Gene2 of predicted pair	
Systematic Name	Standard Name	Systematic Name	Standard Name
YGL133W	ITC1	YNL206C	RTT106
YMR311C	GLC8	YDR310C	SUM1
YKL126W	YPK1	YCR053W	THR4
YIL076W	SEC28	YDR261C	EXG2
YOR312C	RPL20B	YNL079C	TPM1
YBL058W	SHP1	YBR108W	YBR108W
YLR227C	ADY4	YNL225C	CNM67
YJR132W	NMD5	YPR119W	CLB2
YDL065C	PEX19	YLR452C	SST2
YIL034C	CAP2	YGL252C	RTG2
YNL069C	RPL16B	YOR132W	VPS17
YKR035W-A	DID2	YLR398C	SKI2
YKR072C	SIS2	YGL148W	ARO2
YMR004W	MVP1	YAL002W	VPS8
YOR337W	TEA1	YEL053C	MAK10
YOR327C	SNC2	YER122C	GLO3
YBR019C	GAL10	YJL095W	BCK1
YPL001W	HAT1	YKR001C	VPS1
YFR021W	ATG18	YJR025C	BNA1
YEL051W	VMA8	YAL002W	VPS8
YML102W	CAC2	YMR038C	CCS1
YOR112W	CEX1	YOR109W	INP53
YFR031C-A	RPL2A	YJR040W	GEF1
YDR439W	LRS4	YDR318W	MCM21
YPL161C	BEM4	YER122C	GLO3
YIL076W	SEC28	YEL013W	VAC8
YML016C	PPZ1	YHR111W	UBA4
YNL087W	TCB2	YER044C	ERG28
YLR064W	YLR064W	YMR253C	YMR253C

Predictions at threshold level 0.75 continued...			
Gene1 of predicted pair		Gene2 of predicted pair	
Systematic Name	Standard Name	Systematic Name	Standard Name
YIL053W	RHR2	YAR014C	BUD14
YNL297C	MON2	YDL192W	ARF1
YJL060W	BNA3	YMR202W	ERG2
YNL106C	INP52	YNL225C	CNM67
YBL102W	SFT2	YLR309C	IMH1
YBR108W	YBR108W	YGL124C	MON1
YNL291C	MID1	YNL107W	YAF9
YMR109W	MYO5	YPL161C	BEM4
YLR079W	SIC1	YFR036W	CDC26
YBR108W	YBR108W	YGR054W	YGR054W
YJR140C	HIR3	YGR270W	YTA7
YDL080C	THI3	YER139C	YER139C
YIL076W	SEC28	YCL001W	RER1
YDR477W	SNF1	YPR119W	CLB2
YIR025W	MND2	YDR260C	SWM1
YER161C	SPT2	YDR289C	RTT103
YHR108W	GGA2	YOL081W	IRA2
YIL156W	UBP7	YCR024C-A	PMP1
YLR119W	SRN2	YOR043W	WHI2
YGL161C	YIP5	YEL056W	HAT2
YBR041W	FAT1	YER044C	ERG28
YOR275C	RIM20	YPL089C	RLM1
YCR020C-A	MAK31	YKR001C	VPS1
YPL057C	SUR1	YER040W	GLN3
YDL184C	RPL41A	YGL212W	VAM7
YMR199W	CLN1	YMR052W	FAR3
YFR031C-A	RPL2A	YPR051W	MAK3
YGL078C	DBP3	YOR043W	WHI2
YHR185C	PFS1	YDL020C	RPN4

Predictions at threshold level 0.75 continued...			
Gene1 of predicted pair		Gene2 of predicted pair	
Systematic Name	Standard Name	Systematic Name	Standard Name
YKL101W	HSL1	YMR038C	CCS1
YDL020C	RPN4	YPR051W	MAK3
YDL184C	RPL41A	YDR484W	VPS52
YBL103C	RTG3	YOR066W	MSA1
YPR089W	YPR089W	YBR283C	SSH1
YJL127C	SPT10	YLR381W	CTF3
YMR055C	BUB2	YNL225C	CNM67
YKL025C	PAN3	YLR234W	TOP3
YMR250W	GAD1	YPR119W	CLB2
YOR132W	VPS17	YGR270W	YTA7
YDR245W	MNN10	YMR089C	YTA12
YER144C	UBP5	YBL103C	RTG3
YDR443C	SSN2	YMR224C	MRE11
YFR010W	UBP6	YKL116C	PRR1
YDR358W	GGA1	YDL192W	ARF1
YOR018W	ROD1	YMR243C	ZRC1
YMR202W	ERG2	YDR200C	VPS64
YDL013W	SLX5	YHR013C	ARD1
YDR484W	VPS52	YPR036W	VMA13
YLL007C	YLL007C	YDL155W	CLB3
YOR337W	TEA1	YPR046W	MCM16
YMR202W	ERG2	YDR254W	CHL4
YBR264C	YPT10	YER074W-A	YOS1
YMR224C	MRE11	YCR053W	THR4
YBR217W	ATG12	YER044C	ERG28
YNL166C	BNI5	YGL124C	MON1
YPR030W	CSR2	YMR311C	GLC8
YNL307C	MCK1	YNL116W	DMA2
YKL010C	UFD4	YGR135W	PRE9

Predictions at threshold level 0.75 continued...			
**Gene1 of predicted pair**		**Gene2 of predicted pair**	
**Systematic Name**	**Standard Name**	**Systematic Name**	**Standard Name**
YNL127W	FAR11	YKR030W	GMH1
YGR270W	YTA7	YKL110C	KTI12
YDL013W	SLX5	YOL090W	MSH2
YOR023C	AHC1	YGR252W	GCN5
YBL047C	EDE1	YNL229C	URE2
YER129W	SAK1	YPL256C	CLN2
YNL201C	PSY2	YDL101C	DUN1
YPR131C	NAT3	YPL018W	CTF19
YFR010W	UBP6	YFR036W	CDC26
YBR194W	SOY1	YKL213C	DOA1
YCR065W	HCM1	YMR055C	BUB2
YJL058C	BIT61	YDR318W	MCM21
YKR101W	SIR1	YBR009C	HHF1
YBL021C	HAP3	YEL053C	MAK10
YLR094C	GIS3	YOR035C	SHE4
YLR095C	IOC2	YBR173C	UMP1
YER107C	GLE2	YML103C	NUP188
YGL025C	PGD1	YDR207C	UME6
YHR039C-A	VMA10	YMR230W	RPS10B
YLR018C	POM34	YDR137W	RGP1
YLR090W	XDJ1	YDL223C	HBT1
YBL103C	RTG3	YMR152W	YIM1
YNL215W	IES2	YEL056W	HAT2
YBR126C	TPS1	YDL192W	ARF1
YER120W	SCS2	YMR054W	STV1
YDR448W	ADA2	YPL248C	GAL4
YPR120C	CLB5	YDR318W	MCM21
YHR205W	SCH9	YOR008C	SLG1
YLR410W	VIP1	YOL081W	IRA2

Predictions at threshold level 0.75 continued...			
Gene1 of predicted pair		Gene2 of predicted pair	
Systematic Name	Standard Name	Systematic Name	Standard Name
YDR439W	LRS4	YJL124C	LSM1
YJL004C	SYS1	YHR016C	YSC84
YBL008W	HIR1	YGR252W	GCN5
YBR131W	CCZ1	YMR231W	PEP5
YLR315W	NKP2	YCR077C	PAT1
YPL057C	SUR1	YMR055C	BUB2
YLR320W	MMS22	YFR036W	CDC26
YML103C	NUP188	YOL126C	MDH2
YPR051W	MAK3	YAR014C	BUD14
YLR292C	SEC72	YLR386W	VAC14
YLR094C	GIS3	YGL115W	SNF4
YGL178W	MPT5	YBR200W	BEM1
YDL065C	PEX19	YGR077C	PEX8
YLR090W	XDJ1	YPL037C	EGD1
YJL095W	BCK1	YNL090W	RHO2
YMR016C	SOK2	YMR230W	RPS10B
YDR363W-A	SEM1	YHR115C	DMA1
YDL161W	ENT1	YDL226C	GCS1
YPR029C	APL4	YDL006W	PTC1
YLR170C	APS1	YOR069W	VPS5
YJR024C	MDE1	YNL199C	GCR2
YPR189W	SKI3	YPR119W	CLB2
YML071C	COG8	YEL042W	GDA1
YMR055C	BUB2	YJR135C	MCM22
YIL105C	SLM1	YJL058C	BIT61
YDR293C	SSD1	YNL288W	CAF40
YPL105C	YPL105C	YEL051W	VMA8
YLR018C	POM34	YBR042C	CST26
YCR086W	CSM1	YLR233C	EST1

Predictions at threshold level 0.75 continued...			
Gene1 of predicted pair		Gene2 of predicted pair	
Systematic Name	Standard Name	Systematic Name	Standard Name
YFR010W	UBP6	YGL025C	PGD1
YMR224C	MRE11	YAR014C	BUD14
YDR293C	SSD1	YJL190C	RPS22A
YDR254W	CHL4	YGL124C	MON1
YNL238W	KEX2	YJR033C	RAV1
YBR200W	BEM1	YPL234C	TFP3
YPL024W	RMI1	YGL151W	NUT1
YOR337W	TEA1	YPL137C	GIP3
YLR093C	NYV1	YFL048C	EMP47
YMR026C	PEX12	YER122C	GLO3
YBR200W	BEM1	YDR310C	SUM1
YHR134W	WSS1	YGL175C	SAE2
YMR054W	STV1	YDR153C	ENT5
YPL234C	TFP3	YJL183W	MNN11
YNL215W	IES2	YCL037C	SRO9
YDR174W	HMO1	YNL116W	DMA2
YFL023W	BUD27	YKL048C	ELM1
YIL159W	BNR1	YBL051C	PIN4
YHL020C	OPI1	YDL077C	VAM6
YLR288C	MEC3	YJL138C	TIF2
YLR254C	NDL1	YGL252C	RTG2
YLR360W	VPS38	YOR109W	INP53
YOL145C	CTR9	YGL025C	PGD1
YJR063W	RPA12	YER068W	MOT2
YDR156W	RPA14	YOR032C	HMS1
YIL034C	CAP2	YGL212W	VAM7
YGL135W	RPL1B	YBR042C	CST26
YJL210W	PEX2	YAL055W	PEX22
YJL136C	RPS21B	YDL047W	SIT4

Predictions at threshold level 0.75 continued...			
Gene1 of predicted pair		Gene2 of predicted pair	
Systematic Name	Standard Name	Systematic Name	Standard Name
YNL248C	RPA49	YKL048C	ELM1
YBR260C	RGD1	YLR425W	TUS1
YLR192C	HCR1	YDL184C	RPL41A
YDR477W	SNF1	YBR260C	RGD1
YGL147C	RPL9A	YIL159W	BNR1
YDR484W	VPS52	YKR035W-A	DID2
YKR072C	SIS2	YOR368W	RAD17
YDR080W	VPS41	YPL057C	SUR1
YDR443C	SSN2	YGL124C	MON1
YDR227W	SIR4	YHR061C	GIC1
YFR010W	UBP6	YBR194W	SOY1
YCL037C	SRO9	YOR308C	SNU66
YDR293C	SSD1	YJL004C	SYS1
YPL246C	RBD2	YKR030W	GMH1
YPL049C	DIG1	YBR083W	TEC1
YCR024C-A	PMP1	YMR125W	STO1
YMR095C	SNO1	YDR358W	GGA1
YBR107C	IML3	YIR005W	IST3
YJR056C	YJR056C	YNL041C	COG6
YOR270C	VPH1	YOR035C	SHE4
YBR173C	UMP1	YOL111C	MDY2
YDR202C	RAV2	YDR137W	RGP1
YIL002C	INP51	YDL161W	ENT1
YPL246C	RBD2	YOR284W	HUA2
YPR119W	CLB2	YLR032W	RAD5
YGL200C	EMP24	YLR131C	ACE2
YGR188C	BUB1	YNL225C	CNM67
YGL016W	KAP122	YKL068W	NUP100
YER162C	RAD4	YDR358W	GGA1

Predictions at threshold level 0.75 continued...			
**Gene1 of predicted pair**		**Gene2 of predicted pair**	
**Systematic Name**	**Standard Name**	**Systematic Name**	**Standard Name**
YDL223C	HBT1	YJL095W	BCK1
YDR137W	RGP1	YDL077C	VAM6
YML109W	ZDS2	YNL030W	HHF2
YML001W	YPT7	YBR280C	SAF1
YDL002C	NHP10	YHR167W	THP2
YBR194W	SOY1	YBR200W	BEM1
YBR173C	UMP1	YOR035C	SHE4
YPL161C	BEM4	YFL033C	RIM15
YDR477W	SNF1	YPR036W	VMA13
YLR360W	VPS38	YDR207C	UME6
YOR332W	VMA4	YKL119C	VPH2
YMR224C	MRE11	YDL155W	CLB3
YNL288W	CAF40	YEL053C	MAK10
YCR020C-A	MAK31	YOR132W	VPS17
YPL022W	RAD1	YGL094C	PAN2
YLR292C	SEC72	YAL002W	VPS8
YPL152W	RRD2	YPR119W	CLB2
YER139C	YER139C	YLR180W	SAM1
YLR292C	SEC72	YJL095W	BCK1
YBL102W	SFT2	YDR108W	GSG1
YBR171W	SEC66	YPL037C	EGD1
YJR056C	YJR056C	YHR039C-A	VMA10
YGR054W	YGR054W	YJR135C	MCM22
YGL237C	HAP2	YMR055C	BUB2
YMR311C	GLC8	YLR370C	ARC18
YHR111W	UBA4	YJR032W	CPR7
YPR018W	RLF2	YOL110W	SHR5
YGL250W	RMR1	YPR046W	MCM16
YJL176C	SWI3	YCR077C	PAT1

Predictions at threshold level 0.75 continued...			
Gene1 of predicted pair		Gene2 of predicted pair	
Systematic Name	Standard Name	Systematic Name	Standard Name
YGL115W	SNF4	YDL047W	SIT4
YNL215W	IES2	YHL031C	GOS1
YER139C	YER139C	YDL192W	ARF1
YIL036W	CST6	YLR254C	NDL1
YOR033C	EXO1	YJL124C	LSM1
YML001W	YPT7	YMR230W	RPS10B
YDR477W	SNF1	YDL077C	VAM6
YDR383C	NKP1	YJR063W	RPA12
YKL006W	RPL14A	YBL072C	RPS8A
YFR031C-A	RPL2A	YER120W	SCS2
YLR410W	VIP1	YNL107W	YAF9
YER044C	ERG28	YEL053C	MAK10
YCR088W	ABP1	YOR284W	HUA2
YNL135C	FPR1	YDR155C	CPR1
YPL001W	HAT1	YDL002C	NHP10
YGL019W	CKB1	YPR119W	CLB2
YLL039C	UBI4	YDR440W	DOT1
YHL031C	GOS1	YBR215W	HPC2
YFR031C-A	RPL2A	YPR032W	SRO7
YFR010W	UBP6	YER161C	SPT2
YGR135W	PRE9	YOR035C	SHE4
YOR080W	DIA2	YGR105W	VMA21
YHR039C-A	VMA10	YKL080W	VMA5
YMR016C	SOK2	YOL081W	IRA2
YOR038C	HIR2	YLR350W	ORM2
YML103C	NUP188	YDR378C	LSM6
YGL212W	VAM7	YBL047C	EDE1
YIL033C	BCY1	YLR206W	ENT2
YOR001W	RRP6	YDR440W	DOT1

Predictions at threshold level 0.75 continued...			
Gene1 of predicted pair		Gene2 of predicted pair	
Systematic Name	Standard Name	Systematic Name	Standard Name
YPR084W	YPR084W	YOR080W	DIA2
YPL161C	BEM4	YJR032W	CPR7
YMR179W	SPT21	YGL003C	CDH1
YOR076C	SKI7	YPL024W	RMI1
YCR020C-A	MAK31	YOR109W	INP53
YDR156W	RPA14	YML117W	NAB6
YPL253C	VIK1	YOR014W	RTS1
YDL077C	VAM6	YKL119C	VPH2
YIR005W	IST3	YJR135C	MCM22
YNL041C	COG6	YLR309C	IMH1
YOR270C	VPH1	YER139C	YER139C
YLL039C	UBI4	YDR477W	SNF1
YMR172W	HOT1	YLR234W	TOP3
YHR134W	WSS1	YER110C	KAP123
YGL060W	YBP2	YJL058C	BIT61
YGL066W	SGF73	YBR107C	IML3
YEL065W	SIT1	YBR171W	SEC66
YCL037C	SRO9	YML071C	COG8
YLR094C	GIS3	YPR046W	MCM16
YDR443C	SSN2	YBR171W	SEC66
YML041C	VPS71	YGL094C	PAN2
YDR225W	HTA1	YLR399C	BDF1
YGR261C	APL6	YCL001W	RER1
YMR224C	MRE11	YOL090W	MSH2
YPR171W	BSP1	YHR016C	YSC84
YNL307C	MCK1	YKR035W-A	DID2
YJR040W	GEF1	YDL006W	PTC1
YPR131C	NAT3	YML095C	RAD10
YLR119W	SRN2	YNL087W	TCB2

Predictions at threshold level 0.75 continued...			
Gene1 of predicted pair		Gene2 of predicted pair	
Systematic Name	Standard Name	Systematic Name	Standard Name
YPL120W	VPS30	YNL107W	YAF9
YFR031C-A	RPL2A	YOR112W	CEX1
YML038C	YMD8	YEL051W	VMA8
YGL115W	SNF4	YJR135C	MCM22
YMR167W	MLH1	YNL082W	PMS1
YGR268C	HUA1	YKR030W	GMH1
YDR080W	VPS41	YEL053C	MAK10
YOR327C	SNC2	YDR310C	SUM1
YEL009C	GCN4	YNL025C	SSN8
YDR443C	SSN2	YMR016C	SOK2
YNL025C	SSN8	YNL199C	GCR2
YLR234W	TOP3	YDR155C	CPR1
YGL178W	MPT5	YER016W	BIM1
YLR079W	SIC1	YDR335W	MSN5
YGL208W	SIP2	YGL115W	SNF4
YDL192W	ARF1	YER040W	GLN3
YLR150W	STM1	YFR036W	CDC26
YPR131C	NAT3	YLR381W	CTF3
YML058W	SML1	YDR071C	PAA1
YMR139W	RIM11	YJR094C	IME1
YGR261C	APL6	YGL212W	VAM7
YNL058C	YNL058C	YOL081W	IRA2
YHR200W	RPN10	YLR093C	NYV1
YGR081C	SLX9	YJR090C	GRR1
YBL051C	PIN4	YDL155W	CLB3
YGL087C	MMS2	YFR010W	UBP6
YHR103W	SBE22	YFR021W	ATG18
YGL135W	RPL1B	YLR061W	RPL22A
YDR392W	SPT3	YER107C	GLE2

Predictions at threshold level 0.75 continued...			
Gene1 of predicted pair		Gene2 of predicted pair	
Systematic Name	Standard Name	Systematic Name	Standard Name
YJL036W	SNX4	YNL297C	MON2
YGL208W	SIP2	YDR028C	REG1
YLR337C	VRP1	YMR055C	BUB2
YOR080W	DIA2	YPL057C	SUR1
YJL176C	SWI3	YCL037C	SRO9
YOR043W	WHI2	YPL256C	CLN2
YLR119W	SRN2	YBR036C	CSG2
YER161C	SPT2	YGL148W	ARO2
YDR443C	SSN2	YKR035W-A	DID2
YBR274W	CHK1	YOL081W	IRA2
YNL166C	BNI5	YDR318W	MCM21
YDR295C	HDA2	YEL043W	YEL043W
YFR010W	UBP6	YLR180W	SAM1
YKL139W	CTK1	YLR399C	BDF1
YLR192C	HCR1	YIL076W	SEC28
YBR107C	IML3	YKL048C	ELM1
YER144C	UBP5	YHR200W	RPN10
YOR337W	TEA1	YLR441C	RPS1A
YPL057C	SUR1	YCR077C	PAT1
YLR234W	TOP3	YHR178W	STB5
YDL155W	CLB3	YPR046W	MCM16
YHR013C	ARD1	YKL041W	VPS24
YLR386W	VAC14	YKL101W	HSL1
YBR260C	RGD1	YNL079C	TPM1
YPR018W	RLF2	YML102W	CAC2
YIL036W	CST6	YPL253C	VIK1
YGL115W	SNF4	YFR036W	CDC26
YDR254W	CHL4	YLR370C	ARC18
YLR018C	POM34	YER110C	KAP123

Predictions at threshold level 0.75 continued...			
Gene1 of predicted pair		Gene2 of predicted pair	
Systematic Name	Standard Name	Systematic Name	Standard Name
YGL124C	MON1	YHR115C	DMA1
YCR065W	HCM1	YGL124C	MON1
YLR337C	VRP1	YLR370C	ARC18
YKL048C	ELM1	YOR275C	RIM20
YKL116C	PRR1	YMR142C	RPL13B
YEL053C	MAK10	YHL020C	OPI1
YGL178W	MPT5	YLR182W	SWI6
YDR289C	RTT103	YJL190C	RPS22A
YOR290C	SNF2	YEL060C	PRB1
YMR224C	MRE11	YGL078C	DBP3
YPR051W	MAK3	YBR171W	SEC66
YPR018W	RLF2	YPL018W	CTF19
YHR111W	UBA4	YNL289W	PCL1
YIL159W	BNR1	YMR224C	MRE11
YER114C	BOI2	YER118C	SHO1
YOR195W	SLK19	YML016C	PPZ1
YJR135C	MCM22	YGL151W	NUT1
YMR199W	CLN1	YGL175C	SAE2
YDR225W	HTA1	YBL002W	HTB2
YPL037C	EGD1	YBR019C	GAL10
YLR083C	EMP70	YGR020C	VMA7
YBR131W	CCZ1	YOL018C	TLG2
YGL078C	DBP3	YKL048C	ELM1
YBR194W	SOY1	YIL036W	CST6
YHR200W	RPN10	YGR135W	PRE9
YBR194W	SOY1	YLR350W	ORM2
YJL036W	SNX4	YOR106W	VAM3
YBR030W	YBR030W	YNL307C	MCK1
YOR109W	INP53	YNL127W	FAR11

Predictions at threshold level 0.75 continued...			
Gene1 of predicted pair		Gene2 of predicted pair	
Systematic Name	Standard Name	Systematic Name	Standard Name
YIL159W	BNR1	YJL095W	BCK1
YPL234C	TFP3	YLR309C	IMH1
YHR052W	CIC1	YCR086W	CSM1
YDR443C	SSN2	YER096W	SHC1
YKR035W-A	DID2	YHR081W	LRP1
YLR360W	VPS38	YDR245W	MNN10
YNL166C	BNI5	YGL253W	HXK2
YDR379W	RGA2	YGR152C	RSR1
YKL048C	ELM1	YDL083C	RPS16B
YPL001W	HAT1	YCR086W	CSM1
YER087C-B	SBH1	YOR085W	OST3
YNL201C	PSY2	YGR270W	YTA7
YDR004W	RAD57	YDR254W	CHL4
YGL066W	SGF73	YPR036W	VMA13
YDR264C	AKR1	YNL116W	DMA2
YDL020C	RPN4	YBL051C	PIN4
YJL154C	VPS35	YLR396C	VPS33
YKL110C	KTI12	YOL054W	PSH1
YPL049C	DIG1	YHR084W	STE12
YLR079W	SIC1	YHR111W	UBA4
YJL177W	RPL17B	YPL240C	HSP82
YNL253W	TEX1	YCL011C	GBP2
YBR173C	UMP1	YHR156C	LIN1
YJL154C	VPS35	YNL238W	KEX2
YLL007C	YLL007C	YJR032W	CPR7
YGL054C	ERV14	YAL002W	VPS8
YDR335W	MSN5	YER161C	SPT2
YJL157C	FAR1	YGL003C	CDH1
YBR019C	GAL10	YMR311C	GLC8

Predictions at threshold level 0.75 continued...			
Gene1 of predicted pair		Gene2 of predicted pair	
Systematic Name	Standard Name	Systematic Name	Standard Name
YOR066W	MSA1	YDR358W	GGA1
YOR066W	MSA1	YFR036W	CDC26
YKR001C	VPS1	YPL112C	PEX25
YDL155W	CLB3	YCR077C	PAT1
YKR020W	VPS51	YDR309C	GIC2
YKR001C	VPS1	YPR032W	SRO7
YOR195W	SLK19	YNL054W	VAC7
YGL223C	COG1	YPL120W	VPS30
YDR245W	MNN10	YHR167W	THP2
YMR026C	PEX12	YKR099W	BAS1
YLR337C	VRP1	YBR082C	UBC4
YDR137W	RGP1	YER139C	YER139C
YBR057C	MUM2	YFR036W	CDC26
YNL325C	FIG4	YOL081W	IRA2
YBL047C	EDE1	YAL002W	VPS8
YLR102C	APC9	YJL095W	BCK1
YOR039W	CKB2	YJL138C	TIF2
YNL291C	MID1	YNL307C	MCK1
YNL307C	MCK1	YFR036W	CDC26
YPL253C	VIK1	YAL002W	VPS8
YPL253C	VIK1	YGR054W	YGR054W
YER096W	SHC1	YPL161C	BEM4
YBR030W	YBR030W	YML071C	COG8
YCR024C-A	PMP1	YBL079W	NUP170
YPL037C	EGD1	YJL095W	BCK1
YAL024C	LTE1	YMR055C	BUB2
YMR179W	SPT21	YML016C	PPZ1
YMR224C	MRE11	YBL103C	RTG3
YHR156C	LIN1	YBR108W	YBR108W

Predictions at threshold level 0.75 continued...			
Gene1 of predicted pair		Gene2 of predicted pair	
Systematic Name	Standard Name	Systematic Name	Standard Name
YKL139W	CTK1	YPL152W	RRD2
YFR032C-A	RPL29	YBR131W	CCZ1
YBR173C	UMP1	YLR032W	RAD5
YDR443C	SSN2	YPL253C	VIK1
YDR245W	MNN10	YOR141C	ARP8
YDR439W	LRS4	YPL018W	CTF19
YHL027W	RIM101	YLR370C	ARC18
YPL057C	SUR1	YGL124C	MON1
YLR079W	SIC1	YHR115C	DMA1
YFR036W	CDC26	YLR370C	ARC18
YML001W	YPT7	YEL060C	PRB1
YHR060W	VMA22	YOL121C	RPS19A
YKR001C	VPS1	YDR310C	SUM1
YPR120C	CLB5	YBR036C	CSG2
YLR234W	TOP3	YJR090C	GRR1
YDR484W	VPS52	YLR398C	SKI2
YNL032W	SIW14	YHL011C	PRS3
YLR381W	CTF3	YNL147W	LSM7
YPL024W	RMI1	YOL121C	RPS19A
YMR205C	PFK2	YLR371W	ROM2
YMR133W	REC114	YGL003C	CDH1
YNR052C	POP2	YJL124C	LSM1
YJL176C	SWI3	YDR137W	RGP1
YJL183W	MNN11	YGL253W	HXK2
YLR093C	NYV1	YNR007C	ATG3
YGL223C	COG1	YAR014C	BUD14
YDR289C	RTT103	YEL056W	HAT2
YDL116W	NUP84	YOR195W	SLK19
YGL147C	RPL9A	YBR108W	YBR108W

Predictions at threshold level 0.75 continued...			
Gene1 of predicted pair		Gene2 of predicted pair	
Systematic Name	Standard Name	Systematic Name	Standard Name
YBR200W	BEM1	YDR183W	PLP1
YBR107C	IML3	YBR114W	RAD16
YLL007C	YLL007C	YDR289C	RTT103
YNL238W	KEX2	YLR306W	UBC12
YDR245W	MNN10	YDR310C	SUM1
YGR270W	YTA7	YJL006C	CTK2
YPR171W	BSP1	YKL204W	EAP1
YJL157C	FAR1	YDL047W	SIT4
YDR254W	CHL4	YJL183W	MNN11
YGL223C	COG1	YDL192W	ARF1
YPL037C	EGD1	YPR046W	MCM16
YDL006W	PTC1	YOL108C	INO4
YOR109W	INP53	YHR039C-A	VMA10
YER044C	ERG28	YJL183W	MNN11
YJR040W	GEF1	YHR060W	VMA22
YHR013C	ARD1	YGL163C	RAD54
YKR072C	SIS2	YDR146C	SWI5
YMR172W	HOT1	YJR135C	MCM22
YDR261C	EXG2	YLR370C	ARC18
YPL256C	CLN2	YBL087C	RPL23A
YGR072W	UPF3	YGR071C	YGR071C
YMR224C	MRE11	YIR002C	MPH1
YDL116W	NUP84	YAL024C	LTE1
YDR265W	PEX10	YNL214W	PEX17
YHR060W	VMA22	YPL120W	VPS30
YIL159W	BNR1	YEL060C	PRB1
YPR046W	MCM16	YAL002W	VPS8
YGL033W	HOP2	YBL103C	RTG3
YJR056C	YJR056C	YDR358W	GGA1

Predictions at threshold level 0.75 continued...			
Gene1 of predicted pair		Gene2 of predicted pair	
Systematic Name	Standard Name	Systematic Name	Standard Name
YBL008W	HIR1	YDR318W	MCM21
YBL102W	SFT2	YCL037C	SRO9
YML007W	YAP1	YGL208W	SIP2
YNL117W	MLS1	YCR086W	CSM1
YLR360W	VPS38	YPR051W	MAK3
YBR171W	SEC66	YOR066W	MSA1
YJR060W	CBF1	YDR318W	MCM21
YDR137W	RGP1	YEL042W	GDA1
YLR337C	VRP1	YKR014C	YPT52
YDR129C	SAC6	YBL101C	ECM21
YOR197W	MCA1	YPL112C	PEX25
YBR108W	YBR108W	YLR381W	CTF3
YPL096C-A	ERI1	YLR370C	ARC18
YDL116W	NUP84	YNR052C	POP2
YLR447C	VMA6	YGR054W	YGR054W
YGL161C	YIP5	YOR213C	SAS5
YLR018C	POM34	YMR153W	NUP53
YFR031C-A	RPL2A	YLR094C	GIS3
YLR006C	SSK1	YDR379W	RGA2
YCR088W	ABP1	YKR001C	VPS1
YBR173C	UMP1	YOR043W	WHI2
YGR249W	MGA1	YDR137W	RGP1
YFR010W	UBP6	YBR228W	SLX1
YDL127W	PCL2	YFR034C	PHO4
YKL139W	CTK1	YDR363W-A	SEM1
YGL198W	YIP4	YNR049C	MSO1
YML071C	COG8	YJL095W	BCK1
YJR035W	RAD26	YDR207C	UME6
YOR043W	WHI2	YNL079C	TPM1

Predictions at threshold level 0.75 continued...			
Gene1 of predicted pair		Gene2 of predicted pair	
Systematic Name	Standard Name	Systematic Name	Standard Name
YKL048C	ELM1	YGL019W	CKB1
YPL137C	GIP3	YPR030W	CSR2
YBL103C	RTG3	YOL121C	RPS19A
YGR268C	HUA1	YHR016C	YSC84
YBR171W	SEC66	YNL107W	YAF9
YGR020C	VMA7	YPL120W	VPS30
YLR370C	ARC18	YDR310C	SUM1
YML016C	PPZ1	YMR109W	MYO5
YDR080W	VPS41	YJR040W	GEF1
YIL076W	SEC28	YLR094C	GIS3
YJL029C	VPS53	YGL198W	YIP4
YJL124C	LSM1	YEL056W	HAT2
YMR224C	MRE11	YIL036W	CST6
YFR010W	UBP6	YEL053C	MAK10
YPR051W	MAK3	YJL190C	RPS22A
YNL139C	RLR1	YBR111W-A	SUS1
YLR381W	CTF3	YNL248C	RPA49
YMR004W	MVP1	YJR060W	CBF1
YPL253C	VIK1	YDR363W-A	SEM1
YGL212W	VAM7	YDR261C	EXG2
YML115C	VAN1	YNL238W	KEX2
YPR131C	NAT3	YMR199W	CLN1
YGR108W	CLB1	YDR254W	CHL4
YPR018W	RLF2	YDL155W	CLB3
YML001W	YPT7	YNL051W	COG5
YLR337C	VRP1	YGL153W	PEX14
YIL007C	NAS2	YDL240W	LRG1
YNL297C	MON2	YML038C	YMD8
YKL116C	PRR1	YLR398C	SKI2

Predictions at threshold level 0.75 continued...			
Gene1 of predicted pair		Gene2 of predicted pair	
Systematic Name	Standard Name	Systematic Name	Standard Name
YHR060W	VMA22	YCL001W	RER1
YOR033C	EXO1	YDR207C	UME6
YOL126C	MDH2	YER115C	SPR6
YPL057C	SUR1	YGL163C	RAD54
YKL025C	PAN3	YPL115C	BEM3
YLR292C	SEC72	YDR318W	MCM21
YPR089W	YPR089W	YMR022W	UBC7
YNR072W	HXT17	YDR261C	EXG2
YHR134W	WSS1	YLR233C	EST1
YCL037C	SRO9	YIR005W	IST3
YOR312C	RPL20B	YEL036C	ANP1
YBR200W	BEM1	YDR261C	EXG2
YMR224C	MRE11	YGL153W	PEX14
YDL101C	DUN1	YML028W	TSA1
YDR146C	SWI5	YHR108W	GGA2
YPL024W	RMI1	YER144C	UBP5
YLR095C	IOC2	YGR249W	MGA1
YCR066W	RAD18	YER162C	RAD4
YDL020C	RPN4	YJR135C	MCM22
YDR320C	SWA2	YHR039C-A	VMA10
YKR001C	VPS1	YGL115W	SNF4
YNL032W	SIW14	YMR133W	REC114
YLR083C	EMP70	YOR127W	RGA1
YJR059W	PTK2	YMR125W	STO1
YCL073C	YCL073C	YER098W	UBP9
YLR093C	NYV1	YAL030W	SNC1
YNL044W	YIP3	YNL293W	MSB3
YDR293C	SSD1	YIL008W	URM1
YLR094C	GIS3	YMR152W	YIM1

Predictions at threshold level 0.75 continued...			
Gene1 of predicted pair		Gene2 of predicted pair	
Systematic Name	Standard Name	Systematic Name	Standard Name
YER114C	BOI2	YHR012W	VPS29
YDR200C	VPS64	YDL047W	SIT4
YBL103C	RTG3	YGL252C	RTG2
YLR192C	HCR1	YMR311C	GLC8
YKL025C	PAN3	YPL022W	RAD1
YPR051W	MAK3	YCR086W	CSM1
YJR032W	CPR7	YPR119W	CLB2
YMR214W	SCJ1	YDR207C	UME6
YBR173C	UMP1	YER139C	YER139C
YFL013C	IES1	YDR289C	RTT103
YGL066W	SGF73	YML095C	RAD10
YPL024W	RMI1	YDL077C	VAM6
YEL013W	VAC8	YHR167W	THP2
YLR288C	MEC3	YNL215W	IES2
YDR477W	SNF1	YNL087W	TCB2
YCR066W	RAD18	YMR055C	BUB2
YLR234W	TOP3	YPL161C	BEM4
YDR254W	CHL4	YHR200W	RPN10
YPL042C	SSN3	YPR072W	NOT5
YGR019W	UGA1	YOR127W	RGA1
YFL013C	IES1	YFR010W	UBP6
YCL037C	SRO9	YAR014C	BUD14
YGR270W	YTA7	YGR188C	BUB1
YMR109W	MYO5	YPR046W	MCM16
YGR184C	UBR1	YGL163C	RAD54
YOR197W	MCA1	YIL033C	BCY1
YGL212W	VAM7	YNR032W	PPG1
YGR252W	GCN5	YLR425W	TUS1
YMR198W	CIK1	YPL234C	TFP3

Predictions at threshold level 0.75 continued...			
Gene1 of predicted pair		Gene2 of predicted pair	
Systematic Name	Standard Name	Systematic Name	Standard Name
YIL008W	URM1	YGR135W	PRE9
YOR106W	VAM3	YFL033C	RIM15
YOR001W	RRP6	YCL011C	GBP2
YMR224C	MRE11	YJR021C	REC107
YER001W	MNN1	YHL031C	GOS1
YDR335W	MSN5	YDR363W-A	SEM1
YPR131C	NAT3	YLR370C	ARC18
YGR105W	VMA21	YPR032W	SRO7
YER144C	UBP5	YER162C	RAD4
YPR131C	NAT3	YML103C	NUP188
YBR111W-A	SUS1	YDR216W	ADR1
YDR004W	RAD57	YBL079W	NUP170
YMR116C	ASC1	YPL050C	MNN9
YFR036W	CDC26	YMR311C	GLC8
YNL201C	PSY2	YNL106C	INP52
YPL129W	TAF14	YJR060W	CBF1
YGR261C	APL6	YOR327C	SNC2
YPR084W	YPR084W	YJR060W	CBF1
YOR001W	RRP6	YCL037C	SRO9
YIL008W	URM1	YJR032W	CPR7
YNL206C	RTT106	YDR440W	DOT1
YJR106W	ECM27	YPR149W	NCE102
YOL031C	SIL1	YPR119W	CLB2
YPR089W	YPR089W	YDR310C	SUM1
YMR311C	GLC8	YLR425W	TUS1
YBL102W	SFT2	YML071C	COG8
YDR456W	NHX1	YLR371W	ROM2
YML115C	VAN1	YLR447C	VMA6
YMR142C	RPL13B	YPL161C	BEM4

Predictions at threshold level 0.75 continued...			
Gene1 of predicted pair		Gene2 of predicted pair	
Systematic Name	Standard Name	Systematic Name	Standard Name
YPL001W	HAT1	YKL032C	IXR1
YEL065W	SIT1	YMR095C	SNO1
YAL024C	LTE1	YML028W	TSA1
YDR245W	MNN10	YLR182W	SWI6
YBR200W	BEM1	YMR179W	SPT21
YKL010C	UFD4	YDR378C	LSM6
YLR381W	CTF3	YOL091W	SPO21
YKR001C	VPS1	YGR105W	VMA21
YCR020C-A	MAK31	YNL051W	COG5
YDR320C	SWA2	YEL051W	VMA8
YOR332W	VMA4	YJR033C	RAV1
YPL161C	BEM4	YBR280C	SAF1
YBR107C	IML3	YJL183W	MNN11
YOR195W	SLK19	YER116C	SLX8
YJR090C	GRR1	YJL190C	RPS22A
YER116C	SLX8	YKL068W	NUP100
YKL010C	UFD4	YOL081W	IRA2
YFR036W	CDC26	YPR119W	CLB2
YHR134W	WSS1	YML028W	TSA1
YNL069C	RPL16B	YPR032W	SRO7
YBR200W	BEM1	YHR200W	RPN10
YDR137W	RGP1	YOR043W	WHI2
YOR327C	SNC2	YBL025W	RRN10
YBR030W	YBR030W	YHR060W	VMA22
YNL041C	COG6	YNL238W	KEX2
YHL031C	GOS1	YPL234C	TFP3
YBL008W	HIR1	YDR254W	CHL4
YMR153W	NUP53	YML071C	COG8
YNL248C	RPA49	YGR188C	BUB1

Predictions at threshold level 0.75 continued...			
Gene1 of predicted pair		Gene2 of predicted pair	
Systematic Name	Standard Name	Systematic Name	Standard Name
YPL057C	SUR1	YOR308C	SNU66
YHR156C	LIN1	YNL325C	FIG4
YMR153W	NUP53	YKL048C	ELM1
YCR077C	PAT1	YBL072C	RPS8A
YPL045W	VPS16	YPL195W	APL5
YJR060W	CBF1	YNL107W	YAF9
YOR332W	VMA4	YGR105W	VMA21
YJL006C	CTK2	YCR077C	PAT1
YDL116W	NUP84	YGL031C	RPL24A
YLR362W	STE11	YER124C	DSE1
YER144C	UBP5	YLR371W	ROM2
YGR081C	SLX9	YBL079W	NUP170
YLR320W	MMS22	YDL192W	ARF1
YIL036W	CST6	YLR410W	VIP1
YCR065W	HCM1	YDR108W	GSG1
YKL032C	IXR1	YLR370C	ARC18
YOR290C	SNF2	YHR013C	ARD1
YOR290C	SNF2	YDL192W	ARF1
YHL031C	GOS1	YDL002C	NHP10
YFR036W	CDC26	YDL077C	VAM6
YCL032W	STE50	YDR309C	GIC2
YBR194W	SOY1	YDR245W	MNN10
YLR018C	POM34	YGR081C	SLX9
YHR073W	OSH3	YFL047W	RGD2
YDL002C	NHP10	YNL107W	YAF9
YOR023C	AHC1	YGL151W	NUT1
YPL001W	HAT1	YOR368W	RAD17
YHR012W	VPS29	YER162C	RAD4
YKR072C	SIS2	YJL187C	SWE1

Predictions at threshold level 0.75 continued...			
Gene1 of predicted pair		Gene2 of predicted pair	
Systematic Name	Standard Name	Systematic Name	Standard Name
YNL307C	MCK1	YOL090W	MSH2
YLR083C	EMP70	YDR080W	VPS41
YNL025C	SSN8	YLR350W	ORM2
YPL137C	GIP3	YER122C	GLO3
YLL007C	YLL007C	YBR114W	RAD16
YPL129W	TAF14	YMR127C	SAS2
YKL048C	ELM1	YDR289C	RTT103
YCL035C	GRX1	YML007W	YAP1
YMR224C	MRE11	YBL047C	EDE1
YDL059C	RAD59	YNL307C	MCK1
YDR080W	VPS41	YPL195W	APL5
YBL103C	RTG3	YNL238W	KEX2
YNL087W	TCB2	YBR019C	GAL10
YDR443C	SSN2	YPL195W	APL5
YGR261C	APL6	YNL291C	MID1
YNL051W	COG5	YDR207C	UME6
YJL190C	RPS22A	YBR114W	RAD16
YLR410W	VIP1	YDR207C	UME6
YCR077C	PAT1	YEL056W	HAT2
YOR043W	WHI2	YOL121C	RPS19A
YEL056W	HAT2	YBR036C	CSG2
YOR106W	VAM3	YGR209C	TRX2
YLR018C	POM34	YML057W	CMP2
YOL126C	MDH2	YNL199C	GCR2
YOL108C	INO4	YKR030W	GMH1
YOR083W	WHI5	YLR370C	ARC18
YGR104C	SRB5	YFR010W	UBP6
YOR212W	STE4	YDR309C	GIC2
YPL001W	HAT1	YBR009C	HHF1

Predictions at threshold level 0.75 continued...			
Gene1 of predicted pair		Gene2 of predicted pair	
Systematic Name	Standard Name	Systematic Name	Standard Name
YMR198W	CIK1	YKL041W	VPS24
YJL154C	VPS35	YDR320C	SWA2
YOR014W	RTS1	YPR119W	CLB2
YJR032W	CPR7	YDR310C	SUM1
YNL068C	FKH2	YNL206C	RTT106
YEL053C	MAK10	YAL002W	VPS8
YKL073W	LHS1	YMR214W	SCJ1
YGL054C	ERV14	YDL077C	VAM6
YGL003C	CDH1	YKL114C	APN1
YGL163C	RAD54	YPL256C	CLN2
YLR371W	ROM2	YMR230W	RPS10B
YPL042C	SSN3	YLR386W	VAC14
YLR102C	APC9	YHR200W	RPN10
YJR140C	HIR3	YML016C	PPZ1
YER001W	MNN1	YJL183W	MNN11
YMR127C	SAS2	YOR023C	AHC1
YPR036W	VMA13	YER044C	ERG28
YOR132W	VPS17	YPL161C	BEM4
YAL024C	LTE1	YJL154C	VPS35
YOR023C	AHC1	YMR055C	BUB2
YLR386W	VAC14	YAL010C	MDM10
YDR137W	RGP1	YER040W	GLN3
YDR293C	SSD1	YGR261C	APL6
YMR109W	MYO5	YHR012W	VPS29
YJR040W	GEF1	YKR020W	VPS51
YGL054C	ERV14	YOR327C	SNC2
YKR014C	YPT52	YNL049C	SFB2
YDR443C	SSN2	YDR456W	NHX1
YML115C	VAN1	YLR425W	TUS1

Predictions at threshold level 0.75 continued...			
Gene1 of predicted pair		Gene2 of predicted pair	
Systematic Name	Standard Name	Systematic Name	Standard Name
YDL116W	NUP84	YLR182W	SWI6
YLR083C	EMP70	YOR112W	CEX1
YDL161W	ENT1	YDR323C	PEP7
YPR028W	YOP1	YNR049C	MSO1
YLR090W	XDJ1	YEL053C	MAK10
YDR183W	PLP1	YDR310C	SUM1
YER144C	UBP5	YLR094C	GIS3
YFR031C-A	RPL2A	YOR270C	VPH1
YOR106W	VAM3	YEL053C	MAK10
YDR320C	SWA2	YNL107W	YAF9
YOR337W	TEA1	YNL041C	COG6
YHR073W	OSH3	YKL068W	NUP100
YLR240W	VPS34	YPL120W	VPS30
YGL200C	EMP24	YNL049C	SFB2
YML115C	VAN1	YEL036C	ANP1
YBR228W	SLX1	YGL031C	RPL24A
YLR083C	EMP70	YML001W	YPT7
YDL149W	ATG9	YML059C	NTE1
YMR311C	GLC8	YER040W	GLN3
YJL172W	CPS1	YDR358W	GGA1
YOR080W	DIA2	YOR308C	SNU66
YMR319C	FET4	YOR071C	THI71
YPL253C	VIK1	YJL006C	CTK2
YHR134W	WSS1	YMR109W	MYO5
YLR320W	MMS22	YOR327C	SNC2
YBL021C	HAP3	YLR233C	EST1
YLR456W	YLR456W	YIR009W	MSL1
YDL042C	SIR2	YBR009C	HHF1
YPL024W	RMI1	YFR036W	CDC26

Predictions at threshold level 0.75 continued...			
**Gene1 of predicted pair**		**Gene2 of predicted pair**	
**Systematic Name**	**Standard Name**	**Systematic Name**	**Standard Name**
YBR173C	UMP1	YPL057C	SUR1
YMR054W	STV1	YML071C	COG8
YLR386W	VAC14	YML095C	RAD10
YIR005W	IST3	YGL252C	RTG2
YIR009W	MSL1	YPR101W	SNT309
YOR066W	MSA1	YBR036C	CSG2
YHR056C	RSC30	YHR191C	CTF8
YML095C	RAD10	YJR060W	CBF1
YBR278W	DPB3	YOR304W	ISW2
YLR079W	SIC1	YPL022W	RAD1
YJL004C	SYS1	YKR030W	GMH1
YGL237C	HAP2	YAL002W	VPS8
YBR200W	BEM1	YJL136C	RPS21B
YBL102W	SFT2	YDR202C	RAV2
YLR288C	MEC3	YMR199W	CLN1
YDR260C	SWM1	YDR254W	CHL4
YOR191W	RIS1	YBR228W	SLX1
YBR217W	ATG12	YNL087W	TCB2
YDR507C	GIN4	YDR363W-A	SEM1
YGL212W	VAM7	YMR230W	RPS10B
YDR293C	SSD1	YHR060W	VMA22
YPL024W	RMI1	YPL253C	VIK1
YGL237C	HAP2	YJR032W	CPR7
YGR241C	YAP1802	YPL045W	VPS16
YPR084W	YPR084W	YMR038C	CCS1
YGL147C	RPL9A	YDL223C	HBT1
YGL212W	VAM7	YGR020C	VMA7
YJL004C	SYS1	YDR310C	SUM1
YNL248C	RPA49	YLR254C	NDL1

Predictions at threshold level 0.75 continued...			
Gene1 of predicted pair		Gene2 of predicted pair	
Systematic Name	Standard Name	Systematic Name	Standard Name
YDL020C	RPN4	YPR046W	MCM16
YFR010W	UBP6	YHL031C	GOS1
YKR020W	VPS51	YNL079C	TPM1
YIL159W	BNR1	YKR048C	NAP1
YBL047C	EDE1	YBL072C	RPS8A
YBR280C	SAF1	YDR310C	SUM1
YHL002W	HSE1	YLR425W	TUS1
YGR249W	MGA1	YER162C	RAD4
YLR381W	CTF3	YGL237C	HAP2
YDR097C	MSH6	YNL076W	MKS1
YFR031C-A	RPL2A	YDL083C	RPS16B
YGL252C	RTG2	YOL081W	IRA2
YPL057C	SUR1	YHR039C-A	VMA10
YNL069C	RPL16B	YGL223C	COG1
YNL215W	IES2	YDL006W	PTC1
YOR132W	VPS17	YGL253W	HXK2
YMR004W	MVP1	YEL043W	YEL043W
YOL145C	CTR9	YPL057C	SUR1
YKL048C	ELM1	YHR039C-A	VMA10
YBR173C	UMP1	YHR039C-A	VMA10
YLR216C	CPR6	YDR293C	SSD1
YBR057C	MUM2	YMR038C	CCS1
YJR106W	ECM27	YDR105C	TMS1
YHR086W	NAM8	YDR378C	LSM6
YKR072C	SIS2	YNL016W	PUB1
YDL020C	RPN4	YDR207C	UME6
YLR090W	XDJ1	YLR386W	VAC14
YIL076W	SEC28	YOR270C	VPH1
YGL078C	DBP3	YLR398C	SKI2

Predictions at threshold level 0.75 continued...			
Gene1 of predicted pair		Gene2 of predicted pair	
Systematic Name	Standard Name	Systematic Name	Standard Name
YMR133W	REC114	YJL183W	MNN11
YHR200W	RPN10	YLR452C	SST2
YNL243W	SLA2	YJL187C	SWE1
YPL106C	SSE1	YJR032W	CPR7
YDR477W	SNF1	YLR094C	GIS3
YOR076C	SKI7	YPL057C	SUR1
YPL253C	VIK1	YDL013W	SLX5
YDR443C	SSN2	YKL139W	CTK1
YNL032W	SIW14	YNL307C	MCK1
YGL163C	RAD54	YPR046W	MCM16
YIL053W	RHR2	YGL025C	PGD1
YMR142C	RPL13B	YER044C	ERG28
YDR076W	RAD55	YLR032W	RAD5
YMR091C	NPL6	YLL026W	HSP104
YPR036W	VMA13	YPR119W	CLB2
YPR030W	CSR2	YMR230W	RPS10B
YJR063W	RPA12	YLR410W	VIP1
YNL307C	MCK1	YDR260C	SWM1
YFL023W	BUD27	YPR046W	MCM16
YGL060W	YBP2	YKL114C	APN1
YNL206C	RTT106	YNL147W	LSM7
YLR288C	MEC3	YDR146C	SWI5
YJL172W	CPS1	YMR311C	GLC8
YMR026C	PEX12	YPR046W	MCM16
YER110C	KAP123	YKL048C	ELM1
YLR216C	CPR6	YER114C	BOI2
YDR293C	SSD1	YDR202C	RAV2
YDL116W	NUP84	YMR284W	YKU70
YLR093C	NYV1	YJL183W	MNN11

Predictions at threshold level 0.75 continued...			
Gene1 of predicted pair		Gene2 of predicted pair	
Systematic Name	Standard Name	Systematic Name	Standard Name
YGL212W	VAM7	YJR084W	CSN12
YDR507C	GIN4	YPR040W	TIP41
YDR254W	CHL4	YPL018W	CTF19
YDR484W	VPS52	YCR053W	THR4
YLL039C	UBI4	YOL044W	PEX15
YLR191W	PEX13	YPR173C	VPS4
YJL124C	LSM1	YIR005W	IST3
YMR224C	MRE11	YLR093C	NYV1
YDR176W	NGG1	YPR101W	SNT309
YNL106C	INP52	YCL014W	BUD3
YDL184C	RPL41A	YLR371W	ROM2
YEL009C	GCN4	YDR146C	SWI5
YMR055C	BUB2	YKL110C	KTI12
YDR192C	NUP42	YKR095W	MLP1
YBL101C	ECM21	YLR370C	ARC18
YDL020C	RPN4	YLR292C	SEC72
YJL124C	LSM1	YPL220W	RPL1A
YDL052C	SLC1	YBR041W	FAT1
YLR396C	VPS33	YGL198W	YIP4
YMR153W	NUP53	YHR060W	VMA22
YNL307C	MCK1	YLR254C	NDL1
YBR274W	CHK1	YJL138C	TIF2
YHR119W	SET1	YLR015W	BRE2
YFR036W	CDC26	YML028W	TSA1
YDR146C	SWI5	YOR140W	SFL1
YPL024W	RMI1	YLR180W	SAM1
YOR033C	EXO1	YMR231W	PEP5
YBL008W	HIR1	YBR278W	DPB3
YLR410W	VIP1	YGL252C	RTG2

Predictions at threshold level 0.75 continued...			
Gene1 of predicted pair		Gene2 of predicted pair	
Systematic Name	Standard Name	Systematic Name	Standard Name
YDL184C	RPL41A	YPL220W	RPL1A
YGL060W	YBP2	YMR198W	CIK1
YER129W	SAK1	YDR028C	REG1
YLR386W	VAC14	YGL237C	HAP2
YDR443C	SSN2	YMR311C	GLC8
YPR046W	MCM16	YDR183W	PLP1
YKL048C	ELM1	YJR135C	MCM22
YHR079C	IRE1	YCR053W	THR4
YPL022W	RAD1	YPR119W	CLB2
YJL058C	BIT61	YJR035W	RAD26
YKL139W	CTK1	YKR035W-A	DID2
YMR199W	CLN1	YMR231W	PEP5
YBR108W	YBR108W	YGR188C	BUB1
YDR108W	GSG1	YGR071C	YGR071C
YNL201C	PSY2	YHR111W	UBA4
YIL076W	SEC28	YOR106W	VAM3
YHR073W	OSH3	YIL148W	RPL40A
YDR395W	SXM1	YML103C	NUP188
YHL031C	GOS1	YEL013W	VAC8
YGR108W	CLB1	YML016C	PPZ1
YGR014W	MSB2	YLR398C	SKI2
YOR304W	ISW2	YMR042W	ARG80
YMR216C	SKY1	YCL037C	SRO9
YLR234W	TOP3	YJR035W	RAD26
YCR084C	TUP1	YBR009C	HHF1
YIL076W	SEC28	YOR109W	INP53
YLR360W	VPS38	YPL234C	TFP3
YLR441C	RPS1A	YML071C	COG8
YLR079W	SIC1	YIL148W	RPL40A

Predictions at threshold level 0.75 continued...			
Gene1 of predicted pair		Gene2 of predicted pair	
Systematic Name	Standard Name	Systematic Name	Standard Name
YPL253C	VIK1	YHR114W	BZZ1
YKL139W	CTK1	YLR381W	CTF3
YER110C	KAP123	YMR125W	STO1
YDR223W	CRF1	YJL141C	YAK1
YBL047C	EDE1	YJL183W	MNN11
YOR358W	HAP5	YPR046W	MCM16
YBL051C	PIN4	YDR309C	GIC2
YJR032W	CPR7	YPR030W	CSR2
YAL024C	LTE1	YJR140C	HIR3
YDR261C	EXG2	YCR053W	THR4
YDR245W	MNN10	YER139C	YER139C
YGR252W	GCN5	YNL079C	TPM1
YKL025C	PAN3	YGR019W	UGA1
YPL137C	GIP3	YJL183W	MNN11
YKL048C	ELM1	YPR120C	CLB5
YNL201C	PSY2	YML016C	PPZ1
YJL124C	LSM1	YER122C	GLO3
YKL116C	PRR1	YER144C	UBP5
YDL116W	NUP84	YBR290W	BSD2
YJL172W	CPS1	YMR214W	SCJ1
YLR360W	VPS38	YJR032W	CPR7
YPL042C	SSN3	YPL161C	BEM4
YHR039C-A	VMA10	YER120W	SCS2
YPL024W	RMI1	YBR019C	GAL10
YLR441C	RPS1A	YER122C	GLO3
YER110C	KAP123	YNL031C	HHT2
YOR112W	CEX1	YNL107W	YAF9
YBR195C	MSI1	YDR076W	RAD55
YIL159W	BNR1	YLR094C	GIS3

Predictions at threshold level 0.75 continued...			
Gene1 of predicted pair		Gene2 of predicted pair	
Systematic Name	Standard Name	Systematic Name	Standard Name
YBR030W	YBR030W	YBR200W	BEM1
YKR082W	NUP133	YER105C	NUP157
YOR033C	EXO1	YDL013W	SLX5
YJR040W	GEF1	YOR327C	SNC2
YLR292C	SEC72	YOR132W	VPS17
YIL053W	RHR2	YBL103C	RTG3
YLR360W	VPS38	YDR456W	NHX1
YFR010W	UBP6	YPL079W	RPL21B
YLL007C	YLL007C	YLR381W	CTF3
YDR477W	SNF1	YDR422C	SIP1
YDR101C	ARX1	YLR325C	RPL38
YNL094W	APP1	YPR154W	PIN3
YGL115W	SNF4	YBL025W	RRN10
YLR079W	SIC1	YJL006C	CTK2
YOR212W	STE4	YGR152C	RSR1
YMR167W	MLH1	YKL101W	HSL1
YIL159W	BNR1	YFR010W	UBP6
YBR030W	YBR030W	YPR030W	CSR2
YLR150W	STM1	YNL307C	MCK1
YHR156C	LIN1	YBL025W	RRN10
YDL223C	HBT1	YNL238W	KEX2
YCL063W	VAC17	YFR021W	ATG18
YBR200W	BEM1	YOR035C	SHE4
YOR106W	VAM3	YPR032W	SRO7
YNL093W	YPT53	YNL044W	YIP3
YNL041C	COG6	YBL025W	RRN10
YMR311C	GLC8	YBR036C	CSG2
YPL256C	CLN2	YER027C	GAL83
YLR360W	VPS38	YOR140W	SFL1

Predictions at threshold level 0.75 continued...			
Gene1 of predicted pair		Gene2 of predicted pair	
Systematic Name	Standard Name	Systematic Name	Standard Name
YLR079W	SIC1	YOR312C	RPL20B
YER144C	UBP5	YLR093C	NYV1
YBR126C	TPS1	YOL081W	IRA2
YDL155W	CLB3	YDR183W	PLP1
YMR172W	HOT1	YDR318W	MCM21
YDL002C	NHP10	YNL199C	GCR2
YDR385W	EFT2	YGL200C	EMP24
YBR083W	TEC1	YNR006W	VPS27
YOL018C	TLG2	YNL051W	COG5
YGL033W	HOP2	YKL139W	CTK1
YLR043C	TRX1	YGL212W	VAM7
YML115C	VAN1	YBL101C	ECM21
YMR142C	RPL13B	YDR310C	SUM1
YGL115W	SNF4	YGR105W	VMA21
YCL001W	RER1	YPL161C	BEM4
YBR200W	BEM1	YJL124C	LSM1
YIL053W	RHR2	YPR030W	CSR2
YNL225C	CNM67	YHR115C	DMA1
YPL256C	CLN2	YPR119W	CLB2
YNL116W	DMA2	YLR425W	TUS1
YLR150W	STM1	YGL246C	RAI1
YOL067C	RTG1	YHL002W	HSE1
YNL218W	MGS1	YHR167W	THP2
YJL154C	VPS35	YJL053W	PEP8
YHR156C	LIN1	YKL048C	ELM1
YPL057C	SUR1	YJL183W	MNN11
YDR080W	VPS41	YGL223C	COG1
YBR126C	TPS1	YNL032W	SIW14
YPR036W	VMA13	YGL003C	CDH1

Predictions at threshold level 0.75 continued...			
Gene1 of predicted pair		Gene2 of predicted pair	
Systematic Name	Standard Name	Systematic Name	Standard Name
YNL041C	COG6	YBR036C	CSG2
YPL079W	RPL21B	YHR077C	NMD2
YPL024W	RMI1	YBL058W	SHP1
YMR224C	MRE11	YDL013W	SLX5
YNL068C	FKH2	YGL066W	SGF73
YJL183W	MNN11	YOR327C	SNC2
YKL160W	ELF1	YER139C	YER139C
YLR150W	STM1	YGL163C	RAD54
YBL058W	SHP1	YOR290C	SNF2
YMR153W	NUP53	YJR032W	CPR7
YER047C	SAP1	YPR119W	CLB2
YJR024C	MDE1	YMR125W	STO1
YFR040W	SAP155	YPL256C	CLN2
YNL068C	FKH2	YMR199W	CLN1
YDR108W	GSG1	YGL115W	SNF4
YDR245W	MNN10	YGL153W	PEX14
YMR224C	MRE11	YJL183W	MNN11
YPL022W	RAD1	YLR032W	RAD5
YPR051W	MAK3	YGL223C	COG1
YLR360W	VPS38	YJL004C	SYS1
YOR109W	INP53	YEL053C	MAK10
YNL069C	RPL16B	YMR055C	BUB2
YKR001C	VPS1	YPL195W	APL5
YMR198W	CIK1	YDL047W	SIT4
YKR061W	KTR2	YHR013C	ARD1
YNL215W	IES2	YOR038C	HIR2
YPL161C	BEM4	YNL229C	URE2
YOR140W	SFL1	YER096W	SHC1
YOR109W	INP53	YDL006W	PTC1

Predictions at threshold level 0.75 continued...			
Gene1 of predicted pair		Gene2 of predicted pair	
Systematic Name	Standard Name	Systematic Name	Standard Name
YGL033W	HOP2	YDL184C	RPL41A
YJR059W	PTK2	YER115C	SPR6
YML016C	PPZ1	YIL008W	URM1
YJR140C	HIR3	YEL013W	VAC8
YPL037C	EGD1	YMR054W	STV1
YNL054W	VAC7	YJR135C	MCM22
YOL148C	SPT20	YBR089C-A	NHP6B
YBR173C	UMP1	YMR311C	GLC8
YMR004W	MVP1	YJR032W	CPR7
YKL129C	MYO3	YLR370C	ARC18
YBL103C	RTG3	YLR452C	SST2
YOR132W	VPS17	YGR054W	YGR054W
YPL234C	TFP3	YJR033C	RAV1
YGR072W	UPF3	YOR069W	VPS5
YOR127W	RGA1	YOR109W	INP53
YMR153W	NUP53	YLR441C	RPS1A
YGR135W	PRE9	YGR188C	BUB1
YLR083C	EMP70	YEL043W	YEL043W
YGR072W	UPF3	YJR060W	CBF1
YDR378C	LSM6	YDR363W-A	SEM1
YPR018W	RLF2	YMR199W	CLN1
YPR072W	NOT5	YJL164C	TPK1
YNR052C	POP2	YGR270W	YTA7
YNL054W	VAC7	YNL307C	MCK1
YGR239C	PEX21	YIL160C	POT1
YLR441C	RPS1A	YML001W	YPT7
YLR370C	ARC18	YKL041W	VPS24
YHR200W	RPN10	YDL077C	VAM6
YGR054W	YGR054W	YBR114W	RAD16

Predictions at threshold level 0.75 continued...			
**Gene1 of predicted pair**		**Gene2 of predicted pair**	
**Systematic Name**	**Standard Name**	**Systematic Name**	**Standard Name**
YDR443C	SSN2	YDR484W	VPS52
YLR192C	HCR1	YER044C	ERG28
YLR360W	VPS38	YGL054C	ERV14
YOR275C	RIM20	YGL163C	RAD54
YOR337W	TEA1	YKR099W	BAS1
YLR061W	RPL22A	YBR082C	UBC4
YGL212W	VAM7	YHR060W	VMA22
YKL006W	RPL14A	YKL032C	IXR1
YPL240C	HSP82	YJR086W	STE18
YJL183W	MNN11	YLR425W	TUS1
YBR084C-A	RPL19A	YDR379W	RGA2
YKL126W	YPK1	YNL076W	MKS1
YPR046W	MCM16	YCR053W	THR4
YML058W	SML1	YLR079W	SIC1
YPL253C	VIK1	YDR289C	RTT103
YMR142C	RPL13B	YFL023W	BUD27
YDR146C	SWI5	YBR200W	BEM1
YDR289C	RTT103	YMR038C	CCS1
YLR292C	SEC72	YHL002W	HSE1
YHR041C	SRB2	YBR089C-A	NHP6B
YMR016C	SOK2	YHR060W	VMA22
YNR052C	POP2	YHR115C	DMA1
YIL053W	RHR2	YDR477W	SNF1
YOR080W	DIA2	YGL175C	SAE2
YHR167W	THP2	YOR124C	UBP2
YIL053W	RHR2	YDR484W	VPS52
YOR043W	WHI2	YMR214W	SCJ1
YNL087W	TCB2	YDR261C	EXG2
YBL058W	SHP1	YGL003C	CDH1

Predictions at threshold level 0.75 continued...			
Gene1 of predicted pair		Gene2 of predicted pair	
Systematic Name	Standard Name	Systematic Name	Standard Name
YBL047C	EDE1	YLR370C	ARC18
YBR127C	VMA2	YPL234C	TFP3
YFR021W	ATG18	YLR431C	ATG23
YBL101C	ECM21	YKL041W	VPS24
YHR200W	RPN10	YNL107W	YAF9
YHR134W	WSS1	YJL183W	MNN11
YHR161C	YAP1801	YDR358W	GGA1
YBR171W	SEC66	YER162C	RAD4
YOR106W	VAM3	YLR396C	VPS33
YDR076W	RAD55	YHR010W	RPL27A
YHR012W	VPS29	YKL119C	VPH2
YDR260C	SWM1	YGL003C	CDH1
YPR185W	ATG13	YPR119W	CLB2
YLR079W	SIC1	YDR171W	HSP42
YER044C	ERG28	YCR053W	THR4
YDL160C	DHH1	YDR448W	ADA2
YOR001W	RRP6	YPR120C	CLB5
YFR031C-A	RPL2A	YGL124C	MON1
YIL159W	BNR1	YPR040W	TIP41
YHR111W	UBA4	YIR005W	IST3
YHR144C	DCD1	YPL022W	RAD1
YLR418C	CDC73	YHR056C	RSC30
YER110C	KAP123	YDR183W	PLP1
YDR225W	HTA1	YBR010W	HHT1
YAL024C	LTE1	YGL252C	RTG2
YBL008W	HIR1	YPL253C	VIK1
YLR381W	CTF3	YDR144C	MKC7
YGL133W	ITC1	YNL229C	URE2
YNL243W	SLA2	YNL271C	BNI1

Predictions at threshold level 0.75 continued...			
Gene1 of predicted pair		Gene2 of predicted pair	
Systematic Name	Standard Name	Systematic Name	Standard Name
YPL105C	YPL105C	YDR207C	UME6
YJL053W	PEP8	YGL095C	VPS45
YDL160C	DHH1	YNL147W	LSM7
YDR080W	VPS41	YPL234C	TFP3
YPL129W	TAF14	YBR112C	CYC8
YLR370C	ARC18	YER040W	GLN3
YLR093C	NYV1	YJL095W	BCK1
YHL031C	GOS1	YDL077C	VAM6
YDR225W	HTA1	YBR289W	SNF5
YPL120W	VPS30	YPR032W	SRO7
YJR063W	RPA12	YFR036W	CDC26
YNL098C	RAS2	YIL033C	BCY1
YNL087W	TCB2	YNL041C	COG6
YIL076W	SEC28	YNL107W	YAF9
YER144C	UBP5	YML001W	YPT7
YBR108W	YBR108W	YHR114W	BZZ1
YDR146C	SWI5	YOR106W	VAM3
YML115C	VAN1	YMR038C	CCS1
YHR111W	UBA4	YIL008W	URM1
YLL039C	UBI4	YLR425W	TUS1
YLR094C	GIS3	YJR084W	CSN12
YML058W	SML1	YIL066C	RNR3
YPR036W	VMA13	YJL095W	BCK1
YHR167W	THP2	YAL002W	VPS8
YPL018W	CTF19	YMR038C	CCS1
YHL002W	HSE1	YAR014C	BUD14
YBR030W	YBR030W	YOR290C	SNF2
YOR001W	RRP6	YER041W	YEN1
YNL288W	CAF40	YHR134W	WSS1

Predictions at threshold level 0.75 continued...			
Gene1 of predicted pair		Gene2 of predicted pair	
Systematic Name	Standard Name	Systematic Name	Standard Name
YGR072W	UPF3	YDR202C	RAV2
YDR477W	SNF1	YNL032W	SIW14
YNR052C	POP2	YBL079W	NUP170
YDR378C	LSM6	YGL200C	EMP24
YJL053W	PEP8	YMR054W	STV1
YMR055C	BUB2	YGR270W	YTA7
YDR443C	SSN2	YPL037C	EGD1
YKR072C	SIS2	YDR293C	SSD1
YGR135W	PRE9	YKL041W	VPS24
YER116C	SLX8	YKR095W	MLP1
YJL154C	VPS35	YER120W	SCS2
YBR171W	SEC66	YLR233C	EST1
YEL060C	PRB1	YLR180W	SAM1
YNL032W	SIW14	YER044C	ERG28
YDL160C	DHH1	YDR378C	LSM6
YGL253W	HXK2	YDR310C	SUM1
YJR056C	YJR056C	YDL192W	ARF1
YML038C	YMD8	YGL054C	ERV14
YFR010W	UBP6	YDR289C	RTT103
YBR030W	YBR030W	YDR310C	SUM1
YOR043W	WHI2	YDL192W	ARF1
YDR264C	AKR1	YDL047W	SIT4
YLR192C	HCR1	YLR360W	VPS38
YNL243W	SLA2	YAL030W	SNC1
YJR040W	GEF1	YPL105C	YPL105C
YDL160C	DHH1	YJL187C	SWE1
YHR079C	IRE1	YFL031W	HAC1
YKL101W	HSL1	YDR310C	SUM1
YER139C	YER139C	YCR053W	THR4

Predictions at threshold level 0.75 continued...			
**Gene1 of predicted pair**		**Gene2 of predicted pair**	
**Systematic Name**	**Standard Name**	**Systematic Name**	**Standard Name**
YGL212W	VAM7	YLR320W	MMS22
YPL137C	GIP3	YOL121C	RPS19A
YIL076W	SEC28	YER162C	RAD4
YMR095C	SNO1	YGL003C	CDH1
YAL024C	LTE1	YHR060W	VMA22
YBL058W	SHP1	YJR032W	CPR7
YGL115W	SNF4	YMR214W	SCJ1
YMR026C	PEX12	YGR077C	PEX8
YDR244W	PEX5	YAL055W	PEX22
YMR199W	CLN1	YLR352W	YLR352W
YGL190C	CDC55	YAL011W	SWC3
YDL020C	RPN4	YKL119C	VPH2
YMR016C	SOK2	YFR036W	CDC26
YOR145C	PNO1	YPL240C	HSP82
YOR035C	SHE4	YER139C	YER139C
YDR129C	SAC6	YML071C	COG8
YOR140W	SFL1	YJL095W	BCK1
YOR106W	VAM3	YGL124C	MON1
YJL060W	BNA3	YMR198W	CIK1
YPR018W	RLF2	YGR188C	BUB1
YMR055C	BUB2	YGL163C	RAD54
YMR199W	CLN1	YJR090C	GRR1
YDR245W	MNN10	YDL006W	PTC1
YPL037C	EGD1	YOR066W	MSA1
YGL208W	SIP2	YPR119W	CLB2
YGL066W	SGF73	YOR290C	SNF2
YJL176C	SWI3	YJR140C	HIR3
YEL065W	SIT1	YJL095W	BCK1
YPL161C	BEM4	YHR039C-A	VMA10

Predictions at threshold level 0.75 continued...			
Gene1 of predicted pair		Gene2 of predicted pair	
Systematic Name	Standard Name	Systematic Name	Standard Name
YOR195W	SLK19	YOR080W	DIA2
YNL236W	SIN4	YPL018W	CTF19
YJR021C	REC107	YGL090W	LIF1
YMR198W	CIK1	YPL106C	SSE1
YOR327C	SNC2	YMR311C	GLC8
YDR379W	RGA2	YIL008W	URM1
YER107C	GLE2	YDR485C	VPS72
YJL006C	CTK2	YPR119W	CLB2
YFR021W	ATG18	YML059C	NTE1
YBR057C	MUM2	YLR150W	STM1
YPL022W	RAD1	YNL107W	YAF9
YOR069W	VPS5	YER122C	GLO3
YDL006W	PTC1	YBL101C	ECM21
YLR381W	CTF3	YML112W	CTK3
YNR052C	POP2	YPR120C	CLB5
YDR004W	RAD57	YML028W	TSA1
YGR019W	UGA1	YLR292C	SEC72
YCR077C	PAT1	YNL079C	TPM1
YBL103C	RTG3	YOL081W	IRA2
YDR363W-A	SEM1	YDR071C	PAA1
YOR005C	DNL4	YLR265C	NEJ1
YGR184C	UBR1	YDL155W	CLB3
YNL243W	SLA2	YNL238W	KEX2
YMR139W	RIM11	YML099C	ARG81
YNL032W	SIW14	YOR327C	SNC2
YER087C-B	SBH1	YOR026W	BUB3
YOL111C	MDY2	YHR039C-A	VMA10
YNL264C	PDR17	YBR194W	SOY1
YPR017C	DSS4	YML001W	YPT7

Predictions at threshold level 0.75 continued...			
Gene1 of predicted pair		Gene2 of predicted pair	
Systematic Name	Standard Name	Systematic Name	Standard Name
YPL042C	SSN3	YJL124C	LSM1
YAL024C	LTE1	YGR152C	RSR1
YNL079C	TPM1	YER049W	TPA1
YKR035W-A	DID2	YER040W	GLN3
YDR329C	PEX3	YLR191W	PEX13
YMR224C	MRE11	YGR270W	YTA7
YLR441C	RPS1A	YOR078W	BUD21
YBR084C-A	RPL19A	YGL163C	RAD54
YNL309W	STB1	YDL047W	SIT4
YPL213W	LEA1	YDL155W	CLB3
YML109W	ZDS2	YMR032W	HOF1
YMR016C	SOK2	YIL038C	NOT3
YPL037C	EGD1	YJR060W	CBF1
YMR133W	REC114	YMR311C	GLC8
YJL172W	CPS1	YPL037C	EGD1
YLR058C	SHM2	YBR200W	BEM1
YIL034C	CAP2	YIL133C	RPL16A
YOR033C	EXO1	YLR352W	YLR352W
YEL043W	YEL043W	YOL108C	INO4
YGL033W	HOP2	YNR072W	HXT17
YJL124C	LSM1	YBL079W	NUP170
YMR004W	MVP1	YEL051W	VMA8
YDR484W	VPS52	YHR012W	VPS29
YDL160C	DHH1	YDR507C	GIN4
YLR093C	NYV1	YER040W	GLN3
YNL069C	RPL16B	YJR040W	GEF1
YLR095C	IOC2	YBR126C	TPS1
YKL025C	PAN3	YGL094C	PAN2
YKR048C	NAP1	YKL101W	HSL1

Predictions at threshold level 0.75 continued...			
**Gene1 of predicted pair**		**Gene2 of predicted pair**	
**Systematic Name**	**Standard Name**	**Systematic Name**	**Standard Name**
YJR032W	CPR7	YJR033C	RAV1
YOR112W	CEX1	YPL120W	VPS30
YHR098C	SFB3	YNL233W	BNI4
YOR101W	RAS1	YGL025C	PGD1
YER068W	MOT2	YGL094C	PAN2
YBL102W	SFT2	YPL037C	EGD1
YHL006C	SHU1	YLR376C	PSY3
YDR156W	RPA14	YFR036W	CDC26
YBR258C	SHG1	YHR115C	DMA1
YFR031C-A	RPL2A	YEL053C	MAK10
YML103C	NUP188	YMR125W	STO1
YPR017C	DSS4	YHR012W	VPS29
YMR224C	MRE11	YKL101W	HSL1
YJL117W	PHO86	YMR038C	CCS1
YBL102W	SFT2	YOR327C	SNC2
YGR072W	UPF3	YNR032W	PPG1
YNL227C	JJJ1	YBR119W	MUD1
YGR019W	UGA1	YMR109W	MYO5
YAL024C	LTE1	YNL054W	VAC7
YPL115C	BEM3	YLR234W	TOP3
YNL069C	RPL16B	YHR039C-A	VMA10
YMR231W	PEP5	YOR368W	RAD17
YKL139W	CTK1	YOR308C	SNU66
YLR064W	YLR064W	YCL034W	LSB5
YBR057C	MUM2	YPR120C	CLB5
YNL106C	INP52	YGL031C	RPL24A
YDL020C	RPN4	YGL163C	RAD54
YLR216C	CPR6	YAR014C	BUD14
YJL138C	TIF2	YOL081W	IRA2

Predictions at threshold level 0.75 continued...			
Gene1 of predicted pair		Gene2 of predicted pair	
Systematic Name	Standard Name	Systematic Name	Standard Name
YIL156W	UBP7	YKL129C	MYO3
YML115C	VAN1	YMR205C	PFK2
YHR178W	STB5	YDL002C	NHP10
YER116C	SLX8	YHR060W	VMA22
YML071C	COG8	YBR181C	RPS6B
YPR084W	YPR084W	YOL108C	INO4
YML115C	VAN1	YKL080W	VMA5
YER087C-B	SBH1	YGR152C	RSR1
YMR172W	HOT1	YBR107C	IML3
YBR171W	SEC66	YKL048C	ELM1
YFR031C-A	RPL2A	YLR180W	SAM1
YMR198W	CIK1	YGR014W	MSB2
YPR051W	MAK3	YHR012W	VPS29
YGR252W	GCN5	YIL038C	NOT3
YOR106W	VAM3	YLR371W	ROM2
YLR095C	IOC2	YNR072W	HXT17
YOR132W	VPS17	YOR327C	SNC2
YMR109W	MYO5	YJL183W	MNN11
YMR199W	CLN1	YHR205W	SCH9
YEL065W	SIT1	YNL236W	SIN4
YNL215W	IES2	YOR001W	RRP6
YMR080C	NAM7	YGR071C	YGR071C
YLR337C	VRP1	YBR108W	YBR108W
YOR127W	RGA1	YPL253C	VIK1
YJL127C	SPT10	YPR120C	CLB5
YLR360W	VPS38	YCL001W	RER1
YKL025C	PAN3	YLR386W	VAC14
YLR135W	SLX4	YCL037C	SRO9
YOR127W	RGA1	YJL095W	BCK1

Predictions at threshold level 0.75 continued...			
Gene1 of predicted pair		Gene2 of predicted pair	
Systematic Name	Standard Name	Systematic Name	Standard Name
YJR032W	CPR7	YML071C	COG8
YMR080C	NAM7	YJL176C	SWI3
YBR131W	CCZ1	YER122C	GLO3
YIL076W	SEC28	YBL025W	RRN10
YPR036W	VMA13	YEL053C	MAK10
YLR090W	XDJ1	YML071C	COG8
YBL103C	RTG3	YOR043W	WHI2
YDL020C	RPN4	YER116C	SLX8
YOL051W	GAL11	YPL248C	GAL4
YGL237C	HAP2	YDR289C	RTT103
YER144C	UBP5	YNL238W	KEX2
YNL291C	MID1	YNR032W	PPG1
YOR080W	DIA2	YGL115W	SNF4
YBL103C	RTG3	YEL053C	MAK10
YBL021C	HAP3	YGL237C	HAP2
YFR031C-A	RPL2A	YJL183W	MNN11
YGL223C	COG1	YNR049C	MSO1
YJL095W	BCK1	YAL002W	VPS8
YPL042C	SSN3	YNL107W	YAF9
YBR126C	TPS1	YNR072W	HXT17
YLR441C	RPS1A	YOL121C	RPS19A
YNL325C	FIG4	YPL137C	GIP3
YMR224C	MRE11	YJR090C	GRR1
YMR153W	NUP53	YOL111C	MDY2
YMR224C	MRE11	YPL256C	CLN2
YPL183C	YPL183C	YBR061C	TRM7
YBL008W	HIR1	YDR363W-A	SEM1
YPL161C	BEM4	YGR188C	BUB1
YPR120C	CLB5	YOR038C	HIR2

Predictions at threshold level 0.75 continued...			
Gene1 of predicted pair		Gene2 of predicted pair	
Systematic Name	Standard Name	Systematic Name	Standard Name
YPR018W	RLF2	YOL051W	GAL11
YPL253C	VIK1	YLR399C	BDF1
YDR225W	HTA1	YBR111W-A	SUS1
YGL178W	MPT5	YGR252W	GCN5
YBR290W	BSD2	YPL246C	RBD2
YDL223C	HBT1	YNR072W	HXT17
YGL025C	PGD1	YNL058C	YNL058C
YMR202W	ERG2	YGL003C	CDH1
YEL043W	YEL043W	YNL107W	YAF9
YJL176C	SWI3	YOR243C	PUS7
YHR098C	SFB3	YGL054C	ERV14
YMR116C	ASC1	YEL036C	ANP1
YJL176C	SWI3	YDR385W	EFT2
YEL053C	MAK10	YEL051W	VMA8
YOR112W	CEX1	YCL001W	RER1
YER114C	BOI2	YHR115C	DMA1
YMR095C	SNO1	YKL048C	ELM1
YJR063W	RPA12	YPR120C	CLB5
YHR206W	SKN7	YOR208W	PTP2
YOR127W	RGA1	YNL107W	YAF9
YEL027W	CUP5	YEL051W	VMA8
YOR337W	TEA1	YBL103C	RTG3
YDR080W	VPS41	YLR093C	NYV1
YBL047C	EDE1	YOL081W	IRA2
YGL066W	SGF73	YKL048C	ELM1
YFR032C-A	RPL29	YPL089C	RLM1
YGR249W	MGA1	YIL033C	BCY1
YGR105W	VMA21	YLR131C	ACE2
YFR034C	PHO4	YNL289W	PCL1

Predictions at threshold level 0.75 continued...			
Gene1 of predicted pair		Gene2 of predicted pair	
Systematic Name	Standard Name	Systematic Name	Standard Name
YPL129W	TAF14	YNL236W	SIN4
YMR164C	MSS11	YMR273C	ZDS1
YML112W	CTK3	YAR014C	BUD14
YFL013C	IES1	YCL037C	SRO9
YDL185W	TFP1	YMR054W	STV1
YGL100W	SEH1	YOR368W	RAD17
YIL076W	SEC28	YDL077C	VAM6
YDR076W	RAD55	YKL114C	APN1
YPR131C	NAT3	YML102W	CAC2
YDR293C	SSD1	YGR105W	VMA21
YNL206C	RTT106	YKR048C	NAP1
YGR188C	BUB1	YNL107W	YAF9
YPL022W	RAD1	YLR370C	ARC18
YMR199W	CLN1	YMR273C	ZDS1
YBR030W	YBR030W	YML001W	YPT7
YFR010W	UBP6	YCL037C	SRO9
YDR477W	SNF1	YDL229W	SSB1
YNR031C	SSK2	YPR119W	CLB2
YNL087W	TCB2	YER162C	RAD4
YGL175C	SAE2	YBR082C	UBC4
YDR080W	VPS41	YJR060W	CBF1
YMR016C	SOK2	YJL172W	CPS1
YOR308C	SNU66	YML097C	VPS9
YOR066W	MSA1	YBR280C	SAF1
YMR224C	MRE11	YAL009W	SPO7
YMR038C	CCS1	YKL114C	APN1
YML103C	NUP188	YKL068W	NUP100
YLR254C	NDL1	YOL081W	IRA2
YBR009C	HHF1	YDR071C	PAA1

Predictions at threshold level 0.75 continued...			
Gene1 of predicted pair		Gene2 of predicted pair	
Systematic Name	Standard Name	Systematic Name	Standard Name
YLL039C	UBI4	YJL183W	MNN11
YLR148W	PEP3	YML001W	YPT7
YDL005C	MED2	YPL248C	GAL4
YOR043W	WHI2	YLR452C	SST2
YHR013C	ARD1	YJR135C	MCM22
YGR104C	SRB5	YOR038C	HIR2
YMR133W	REC114	YGL124C	MON1
YAL024C	LTE1	YPL024W	RMI1
YCR088W	ABP1	YGL095C	VPS45
YGR270W	YTA7	YPL018W	CTF19
YPL161C	BEM4	YBL101C	ECM21
YDR293C	SSD1	YEL053C	MAK10
YDR129C	SAC6	YPL050C	MNN9
YNL016W	PUB1	YGL175C	SAE2
YPL057C	SUR1	YDR335W	MSN5
YFR030W	MET10	YOL054W	PSH1
YER162C	RAD4	YCR053W	THR4
YNL215W	IES2	YOR080W	DIA2
YDR260C	SWM1	YHR200W	RPN10
YER139C	YER139C	YGL124C	MON1
YLR441C	RPS1A	YJL095W	BCK1
YCL037C	SRO9	YOL108C	INO4
YML115C	VAN1	YHR039C-A	VMA10
YOR069W	VPS5	YDR137W	RGP1
YNL243W	SLA2	YLR332W	MID2
YNL051W	COG5	YKR030W	GMH1
YLR079W	SIC1	YGR270W	YTA7
YOR106W	VAM3	YML028W	TSA1
YMR153W	NUP53	YER040W	GLN3

Predictions at threshold level 0.75 continued...			
Gene1 of predicted pair		Gene2 of predicted pair	
Systematic Name	Standard Name	Systematic Name	Standard Name
YML115C	VAN1	YMR311C	GLC8
YLR313C	SPH1	YGL031C	RPL24A
YNL032W	SIW14	YDL223C	HBT1
YOR023C	AHC1	YMR109W	MYO5
YPL018W	CTF19	YHR115C	DMA1
YDR110W	FOB1	YPR101W	SNT309
YML013W	UBX2	YDR310C	SUM1
YMR198W	CIK1	YKR001C	VPS1
YOL121C	RPS19A	YLR370C	ARC18
YDR484W	VPS52	YLR320W	MMS22
YBR112C	CYC8	YGL035C	MIG1
YBR228W	SLX1	YML028W	TSA1
YOR127W	RGA1	YOR069W	VPS5
YPR084W	YPR084W	YDL077C	VAM6
YLR094C	GIS3	YNL041C	COG6
YKR099W	BAS1	YPR046W	MCM16
YLR079W	SIC1	YJR059W	PTK2
YMR004W	MVP1	YML038C	YMD8
YOR080W	DIA2	YMR179W	SPT21
YDL013W	SLX5	YJR035W	RAD26
YIR009W	MSL1	YGL031C	RPL24A
YDR310C	SUM1	YLR182W	SWI6
YDR484W	VPS52	YGL212W	VAM7
YBR030W	YBR030W	YMR016C	SOK2
YGR072W	UPF3	YPL105C	YPL105C
YER143W	DDI1	YNR006W	VPS27
YEL027W	CUP5	YGR105W	VMA21
YJR040W	GEF1	YAL002W	VPS8
YPL042C	SSN3	YOL145C	CTR9

Predictions at threshold level 0.75 continued...			
**Gene1 of predicted pair**		**Gene2 of predicted pair**	
**Systematic Name**	**Standard Name**	**Systematic Name**	**Standard Name**
YDR137W	RGP1	YLR131C	ACE2
YHL031C	GOS1	YNR032W	PPG1
YNL069C	RPL16B	YOR069W	VPS5
YMR205C	PFK2	YBR041W	FAT1
YLR083C	EMP70	YGR105W	VMA21
YLL039C	UBI4	YPL248C	GAL4
YHL020C	OPI1	YMR054W	STV1
YLR386W	VAC14	YKR061W	KTR2
YEL065W	SIT1	YKL116C	PRR1
YGR081C	SLX9	YML102W	CAC2
YDR443C	SSN2	YDR245W	MNN10
YHR052W	CIC1	YER002W	NOP16
YLR410W	VIP1	YGL175C	SAE2
YML016C	PPZ1	YBR260C	RGD1
YMR026C	PEX12	YOR035C	SHE4
YBR274W	CHK1	YLR032W	RAD5
YBR173C	UMP1	YOL121C	RPS19A
YCR077C	PAT1	YPR046W	MCM16
YFR031C-A	RPL2A	YNL076W	MKS1
YEL053C	MAK10	YLR320W	MMS22
YCL032W	STE50	YJR094C	IME1
YEL065W	SIT1	YJL172W	CPS1
YLR095C	IOC2	YLR370C	ARC18
YLR083C	EMP70	YMR216C	SKY1
YPL037C	EGD1	YHR039C-A	VMA10
YMR016C	SOK2	YDR310C	SUM1
YOR043W	WHI2	YDL083C	RPS16B
YBR173C	UMP1	YMR133W	REC114
YMR055C	BUB2	YOL081W	IRA2

Predictions at threshold level 0.75 continued...			
Gene1 of predicted pair		Gene2 of predicted pair	
Systematic Name	Standard Name	Systematic Name	Standard Name
YHR200W	RPN10	YOR043W	WHI2
YDR320C	SWA2	YOR109W	INP53
YMR198W	CIK1	YOL091W	SPO21
YIL053W	RHR2	YMR224C	MRE11
YHR081W	LRP1	YDR310C	SUM1
YHR156C	LIN1	YBR217W	ATG12
YEL056W	HAT2	YDR310C	SUM1
YKR020W	VPS51	YOL108C	INO4
YDL223C	HBT1	YGR135W	PRE9
YKR057W	RPS21A	YDR264C	AKR1
YFL023W	BUD27	YLR370C	ARC18
YKR001C	VPS1	YKL080W	VMA5
YFR031C-A	RPL2A	YJR060W	CBF1
YJR056C	YJR056C	YOL081W	IRA2
YOR132W	VPS17	YDR080W	VPS41
YDR108W	GSG1	YMR054W	STV1
YPL022W	RAD1	YNL236W	SIN4
YFR010W	UBP6	YDR137W	RGP1
YNL201C	PSY2	YGL252C	RTG2
YBR215W	HPC2	YJR060W	CBF1
YLR216C	CPR6	YHR200W	RPN10
YML001W	YPT7	YDR358W	GGA1
YGL087C	MMS2	YMR055C	BUB2
YJL176C	SWI3	YJL006C	CTK2
YNL201C	PSY2	YAL024C	LTE1
YMR068W	AVO2	YIL105C	SLM1
YPR171W	BSP1	YDR310C	SUM1
YFR040W	SAP155	YOR035C	SHE4
YLL007C	YLL007C	YPL253C	VIK1

Predictions at threshold level 0.75 continued...			
Gene1 of predicted pair		Gene2 of predicted pair	
Systematic Name	Standard Name	Systematic Name	Standard Name
YOR290C	SNF2	YNL076W	MKS1
YGR252W	GCN5	YPR120C	CLB5
YJL036W	SNX4	YNL041C	COG6
YPR017C	DSS4	YPL161C	BEM4
YGL147C	RPL9A	YOR290C	SNF2
YKR020W	VPS51	YFR036W	CDC26
YOR039W	CKB2	YNL031C	HHT2
YHR178W	STB5	YJR032W	CPR7
YAL024C	LTE1	YCR053W	THR4
YLR234W	TOP3	YHR114W	BZZ1
YBL051C	PIN4	YPR051W	MAK3
YPL057C	SUR1	YGL115W	SNF4
YMR016C	SOK2	YER139C	YER139C
YDR507C	GIN4	YPL161C	BEM4
YPR018W	RLF2	YNL307C	MCK1
YLR119W	SRN2	YCR053W	THR4
YEL065W	SIT1	YGL147C	RPL9A
YHR039C-A	VMA10	YDR358W	GGA1
YIL103W	DPH1	YPL018W	CTF19
YNL215W	IES2	YCR077C	PAT1
YDR320C	SWA2	YEL053C	MAK10
YDL047W	SIT4	YMR038C	CCS1
YMR204C	INP1	YBR168W	PEX32
YOR106W	VAM3	YPL195W	APL5
YKR020W	VPS51	YEL036C	ANP1
YLR234W	TOP3	YBR108W	YBR108W
YKR035W-A	DID2	YHR200W	RPN10
YKL006W	RPL14A	YKR020W	VPS51
YNL218W	MGS1	YJL176C	SWI3

Predictions at threshold level 0.75 continued...			
Gene1 of predicted pair		Gene2 of predicted pair	
Systematic Name	Standard Name	Systematic Name	Standard Name
YNL087W	TCB2	YGL013C	PDR1
YDL116W	NUP84	YMR179W	SPT21
YIL036W	CST6	YLR381W	CTF3
YML112W	CTK3	YLR182W	SWI6
YOR290C	SNF2	YLR399C	BDF1
YCR024C-A	PMP1	YPL178W	CBC2
YNL201C	PSY2	YIR005W	IST3
YBL021C	HAP3	YHR012W	VPS29
YMR153W	NUP53	YJR056C	YJR056C
YLR150W	STM1	YLR032W	RAD5
YNR072W	HXT17	YDR207C	UME6
YHR012W	VPS29	YNL238W	KEX2
YNL238W	KEX2	YLR093C	NYV1
YDR293C	SSD1	YDR289C	RTT103
YEL009C	GCN4	YGL025C	PGD1
YJR056C	YJR056C	YGL115W	SNF4
YGL212W	VAM7	YGL252C	RTG2
YLR452C	SST2	YMR052W	FAR3
YCR097W	HMRA1	YCL067C	HMLALPHA2
YBR200W	BEM1	YJR005W	APL1
YHR200W	RPN10	YLR357W	RSC2
YLR148W	PEP3	YOR106W	VAM3
YOR195W	SLK19	YPL057C	SUR1
YDR146C	SWI5	YOL081W	IRA2
YDL135C	RDI1	YLR371W	ROM2
YER116C	SLX8	YNL076W	MKS1
YMR199W	CLN1	YJL157C	FAR1
YBR108W	YBR108W	YNR072W	HXT17
YDL184C	RPL41A	YMR016C	SOK2

Predictions at threshold level 0.75 continued...			
Gene1 of predicted pair		Gene2 of predicted pair	
Systematic Name	Standard Name	Systematic Name	Standard Name
YBR107C	IML3	YGR270W	YTA7
YKR014C	YPT52	YML071C	COG8
YOR033C	EXO1	YLR135W	SLX4
YDL116W	NUP84	YKL110C	KTI12
YMR199W	CLN1	YDL047W	SIT4
YOR337W	TEA1	YDR207C	UME6
YOR212W	STE4	YJR086W	STE18
YGR252W	GCN5	YDR227W	SIR4
YCR077C	PAT1	YOR368W	RAD17
YGL087C	MMS2	YER110C	KAP123
YDR383C	NKP1	YGL163C	RAD54
YPL240C	HSP82	YNL086W	YNL086W
YBR030W	YBR030W	YPL057C	SUR1
YDL155W	CLB3	YLR425W	TUS1
YBL058W	SHP1	YGL115W	SNF4
YMR153W	NUP53	YER162C	RAD4
YML115C	VAN1	YDR440W	DOT1
YKR101W	SIR1	YFR036W	CDC26
YML038C	YMD8	YAR014C	BUD14
YGL147C	RPL9A	YMR311C	GLC8
YGL066W	SGF73	YHR108W	GGA2
YLR398C	SKI2	YDR207C	UME6
YLR006C	SSK1	YNL293W	MSB3
YNL087W	TCB2	YNL307C	MCK1
YCR034W	FEN1	YBL008W	HIR1
YBR239C	YBR239C	YHR016C	YSC84
YPL256C	CLN2	YHR158C	KEL1
YLR079W	SIC1	YHR152W	SPO12
YDR156W	RPA14	YLR386W	VAC14

Predictions at threshold level 0.75 continued...			
Gene1 of predicted pair		Gene2 of predicted pair	
Systematic Name	Standard Name	Systematic Name	Standard Name
YBL103C	RTG3	YLR371W	ROM2
YNL079C	TPM1	YGR135W	PRE9
YGL078C	DBP3	YBR280C	SAF1
YPL129W	TAF14	YIR009W	MSL1
YLR090W	XDJ1	YGL115W	SNF4
YGL135W	RPL1B	YDR137W	RGP1
YDR137W	RGP1	YLR180W	SAM1
YNL248C	RPA49	YNL307C	MCK1
YLR337C	VRP1	YDR289C	RTT103
YNL289W	PCL1	YKR048C	NAP1
YDR004W	RAD57	YLR288C	MEC3
YNL293W	MSB3	YNR049C	MSO1
YIL036W	CST6	YPR120C	CLB5
YMR199W	CLN1	YOR106W	VAM3
YJR032W	CPR7	YGL003C	CDH1
YGL174W	BUD13	YJL124C	LSM1
YKR048C	NAP1	YGL124C	MON1
YIL076W	SEC28	YGR270W	YTA7
YHR134W	WSS1	YNL107W	YAF9
YPL019C	VTC3	YER072W	VTC1
YFR010W	UBP6	YOR368W	RAD17
YKR057W	RPS21A	YIL008W	URM1
YIL076W	SEC28	YDR335W	MSN5
YDL020C	RPN4	YMR199W	CLN1
YDR202C	RAV2	YKR030W	GMH1
YJL004C	SYS1	YDR207C	UME6
YGL212W	VAM7	YLR094C	GIS3
YLR119W	SRN2	YMR016C	SOK2
YDL116W	NUP84	YDR378C	LSM6

Predictions at threshold level 0.75 continued...			
Gene1 of predicted pair		Gene2 of predicted pair	
Systematic Name	Standard Name	Systematic Name	Standard Name
YKL025C	PAN3	YML058W	SML1
YMR016C	SOK2	YPR030W	CSR2
YPR131C	NAT3	YDL226C	GCS1
YKL116C	PRR1	YMR153W	NUP53
YBR057C	MUM2	YPL050C	MNN9
YAL024C	LTE1	YDR293C	SSD1
YOR270C	VPH1	YGL115W	SNF4
YKL116C	PRR1	YOR043W	WHI2
YBR009C	HHF1	YBR010W	HHT1
YDR480W	DIG2	YDL159W	STE7
YMR016C	SOK2	YNL041C	COG6
YKL048C	ELM1	YLR320W	MMS22
YDR108W	GSG1	YHL020C	OPI1
YFR036W	CDC26	YDR310C	SUM1
YPL180W	TCO89	YGL200C	EMP24
YBR057C	MUM2	YNL307C	MCK1
YDL184C	RPL41A	YEL053C	MAK10
YGR133W	PEX4	YJL095W	BCK1
YOR290C	SNF2	YLR441C	RPS1A
YER162C	RAD4	YLR370C	ARC18
YAL010C	MDM10	YOL081W	IRA2
YBL051C	PIN4	YBL021C	HAP3
YJL176C	SWI3	YKL109W	HAP4
YAL020C	ATS1	YJL187C	SWE1
YHL031C	GOS1	YER161C	SPT2
YDR176W	NGG1	YOL051W	GAL11
YDR174W	HMO1	YDR183W	PLP1
YCR020C-A	MAK31	YDR108W	GSG1
YGL087C	MMS2	YGL163C	RAD54

Predictions at threshold level 0.75 continued...			
Gene1 of predicted pair		Gene2 of predicted pair	
Systematic Name	Standard Name	Systematic Name	Standard Name
YGR249W	MGA1	YPR046W	MCM16
YER044C	ERG28	YDR358W	GGA1
YKL139W	CTK1	YJL124C	LSM1
YNL288W	CAF40	YDL155W	CLB3
YDR004W	RAD57	YPL018W	CTF19
YGL190C	CDC55	YER027C	GAL83
YDR443C	SSN2	YBR260C	RGD1
YOR195W	SLK19	YPL106C	SSE1
YLR148W	PEP3	YBR131W	CCZ1
YGL066W	SGF73	YDL155W	CLB3
YFR010W	UBP6	YEL013W	VAC8
YHR200W	RPN10	YML001W	YPT7
YFR030W	MET10	YIL103W	DPH1
YMR004W	MVP1	YML001W	YPT7
YKL139W	CTK1	YJL006C	CTK2
YDR245W	MNN10	YPL256C	CLN2
YGR100W	MDR1	YPR028W	YOP1
YNL079C	TPM1	YOL081W	IRA2
YMR243C	ZRC1	YPL058C	PDR12
YPR084W	YPR084W	YAL002W	VPS8
YGR133W	PEX4	YOR035C	SHE4
YPR051W	MAK3	YGL253W	HXK2
YJL006C	CTK2	YOL081W	IRA2
YLR176C	RFX1	YLR399C	BDF1
YJR032W	CPR7	YKL101W	HSL1
YKR035W-A	DID2	YLR094C	GIS3
YDR080W	VPS41	YAR014C	BUD14
YOR132W	VPS17	YCR086W	CSM1
YDR363W-A	SEM1	YNL107W	YAF9

Predictions at threshold level 0.75 continued...			
Gene1 of predicted pair		Gene2 of predicted pair	
Systematic Name	Standard Name	Systematic Name	Standard Name
YGR072W	UPF3	YNL291C	MID1
YDL184C	RPL41A	YMR230W	RPS10B
YDR379W	RGA2	YNL229C	URE2
YJR024C	MDE1	YKL068W	NUP100
YPL024W	RMI1	YGL025C	PGD1
YHR013C	ARD1	YBR082C	UBC4
YJR032W	CPR7	YER139C	YER139C
YHR156C	LIN1	YJR056C	YJR056C
YDR146C	SWI5	YGR089W	NNF2
YDR202C	RAV2	YNL051W	COG5
YPL240C	HSP82	YGR294W	PAU12
YNL135C	FPR1	YHR012W	VPS29
YIR005W	IST3	YHR167W	THP2
YDL184C	RPL41A	YER139C	YER139C
YGR249W	MGA1	YMR142C	RPL13B
YCL037C	SRO9	YHR167W	THP2
YNL289W	PCL1	YJL095W	BCK1
YNL025C	SSN8	YDR440W	DOT1
YFL013C	IES1	YKL139W	CTK1
YDR439W	LRS4	YDR335W	MSN5
YJL204C	RCY1	YAL030W	SNC1
YAL024C	LTE1	YHR115C	DMA1
YPL161C	BEM4	YCR077C	PAT1
YGL115W	SNF4	YNL041C	COG6
YPL024W	RMI1	YMR224C	MRE11
YMR198W	CIK1	YJR135C	MCM22
YBL071W-A	KTI11	YHR187W	IKI1
YDR440W	DOT1	YOR141C	ARP8
YEL044W	IES6	YBL047C	EDE1

Predictions at threshold level 0.75 continued...			
Gene1 of predicted pair		Gene2 of predicted pair	
Systematic Name	Standard Name	Systematic Name	Standard Name
YHL031C	GOS1	YJL124C	LSM1
YDR174W	HMO1	YGL252C	RTG2
YHR060W	VMA22	YGR105W	VMA21
YNL068C	FKH2	YPL161C	BEM4
YFR010W	UBP6	YKR099W	BAS1
YFR031C-A	RPL2A	YOR066W	MSA1
YBR200W	BEM1	YPL256C	CLN2
YMR153W	NUP53	YDR207C	UME6
YMR199W	CLN1	YPL178W	CBC2
YBR200W	BEM1	YFL023W	BUD27
YPL037C	EGD1	YER040W	GLN3
YLR150W	STM1	YKL101W	HSL1
YBR173C	UMP1	YOR327C	SNC2
YBR065C	ECM2	YPL213W	LEA1
YAL024C	LTE1	YMR133W	REC114
YBR107C	IML3	YDR144C	MKC7
YLR090W	XDJ1	YNR072W	HXT17
YBR289W	SNF5	YNL030W	HHF2
YDL065C	PEX19	YGR209C	TRX2
YJL117W	PHO86	YDL006W	PTC1
YMR224C	MRE11	YOL081W	IRA2
YLL039C	UBI4	YDL161W	ENT1
YNL127W	FAR11	YAR014C	BUD14
YDL083C	RPS16B	YPR046W	MCM16
YNL054W	VAC7	YIL103W	DPH1
YJL154C	VPS35	YMR127C	SAS2
YOR358W	HAP5	YBR114W	RAD16
YKR020W	VPS51	YLR396C	VPS33
YGL033W	HOP2	YKR035W-A	DID2

Predictions at threshold level 0.75 continued...			
**Gene1 of predicted pair**		**Gene2 of predicted pair**	
**Systematic Name**	**Standard Name**	**Systematic Name**	**Standard Name**
YPL024W	RMI1	YMR016C	SOK2
YPL178W	CBC2	YBL079W	NUP170
YDR293C	SSD1	YLR182W	SWI6
YML058W	SML1	YLR234W	TOP3
YLL007C	YLL007C	YDR144C	MKC7
YMR224C	MRE11	YOR270C	VPH1
YGL078C	DBP3	YKL116C	PRR1
YPR046W	MCM16	YDL192W	ARF1
YLR079W	SIC1	YML095C	RAD10
YER096W	SHC1	YGL031C	RPL24A
YML117W	NAB6	YBR200W	BEM1
YJL095W	BCK1	YOL067C	RTG1
YLR006C	SSK1	YMR052W	FAR3
YER144C	UBP5	YOR066W	MSA1
YDR477W	SNF1	YLR093C	NYV1
YFR010W	UBP6	YMR230W	RPS10B
YMR224C	MRE11	YDR137W	RGP1
YNL248C	RPA49	YGL252C	RTG2
YDL160C	DHH1	YMR224C	MRE11
YMR224C	MRE11	YPL161C	BEM4
YOL067C	RTG1	YHR114W	BZZ1
YDR195W	REF2	YOR179C	SYC1
YNR023W	SNF12	YPL240C	HSP82
YER139C	YER139C	YDR310C	SUM1
YIL148W	RPL40A	YGR059W	SPR3
YNL297C	MON2	YJR084W	CSN12
YBR217W	ATG12	YOR270C	VPH1
YKL139W	CTK1	YAR014C	BUD14
YDR129C	SAC6	YPL161C	BEM4

Predictions at threshold level 0.75 continued...			
Gene1 of predicted pair		Gene2 of predicted pair	
Systematic Name	Standard Name	Systematic Name	Standard Name
YBL058W	SHP1	YNL076W	MKS1
YIL036W	CST6	YHR111W	UBA4
YIL008W	URM1	YBL087C	RPL23A
YMR179W	SPT21	YDR254W	CHL4
YLR234W	TOP3	YLR032W	RAD5
YNL032W	SIW14	YML071C	COG8
YOL090W	MSH2	YGR258C	RAD2
YOR106W	VAM3	YMR216C	SKY1
YDL020C	RPN4	YGR108W	CLB1
YDR156W	RPA14	YFR032C-A	RPL29
YHR200W	RPN10	YGR232W	NAS6
YKL010C	UFD4	YJL176C	SWI3
YOR112W	CEX1	YAL002W	VPS8
YHL031C	GOS1	YNL199C	GCR2
YPL115C	BEM3	YHR111W	UBA4
YIL036W	CST6	YML097C	VPS9
YHR111W	UBA4	YPR120C	CLB5
YOR308C	SNU66	YNL107W	YAF9
YBR274W	CHK1	YOR043W	WHI2
YMR139W	RIM11	YMR042W	ARG80
YOR358W	HAP5	YGL016W	KAP122
YDL160C	DHH1	YNL145W	MFA2
YPL213W	LEA1	YNL079C	TPM1
YIL036W	CST6	YFR030W	MET10
YJL036W	SNX4	YGR105W	VMA21
YER139C	YER139C	YDR207C	UME6
YMR284W	YKU70	YDR227W	SIR4
YLR381W	CTF3	YPL161C	BEM4
YOL148C	SPT20	YDR216W	ADR1

Predictions at threshold level 0.75 continued...			
Gene1 of predicted pair		Gene2 of predicted pair	
Systematic Name	Standard Name	Systematic Name	Standard Name
YPR120C	CLB5	YNL225C	CNM67
YNR023W	SNF12	YGL025C	PGD1
YDR174W	HMO1	YML016C	PPZ1
YDR146C	SWI5	YMR055C	BUB2
YPR017C	DSS4	YCR066W	RAD18
YKR099W	BAS1	YGL115W	SNF4
YNL069C	RPL16B	YHL020C	OPI1
YEL065W	SIT1	YFR010W	UBP6
YNL297C	MON2	YPL037C	EGD1
YGR129W	SYF2	YPR101W	SNT309
YPL057C	SUR1	YER044C	ERG28
YAL024C	LTE1	YOL081W	IRA2
YJL183W	MNN11	YEL060C	PRB1
YDR261C	EXG2	YDL077C	VAM6
YMR039C	SUB1	YDR310C	SUM1
YOR112W	CEX1	YNL238W	KEX2
YGL105W	ARC1	YPR119W	CLB2
YDL020C	RPN4	YEL053C	MAK10
YKL126W	YPK1	YOL081W	IRA2
YKR099W	BAS1	YEL053C	MAK10
YDR108W	GSG1	YDR137W	RGP1
YDL161W	ENT1	YMR231W	PEP5
YGR249W	MGA1	YHR156C	LIN1
YJL058C	BIT61	YJR135C	MCM22
YGR249W	MGA1	YLR370C	ARC18
YLR119W	SRN2	YLR452C	SST2
YLR090W	XDJ1	YNL238W	KEX2
YPR018W	RLF2	YPR120C	CLB5
YFR010W	UBP6	YMR055C	BUB2

Predictions at threshold level 0.75 continued...			
Gene1 of predicted pair		Gene2 of predicted pair	
Systematic Name	Standard Name	Systematic Name	Standard Name
YMR199W	CLN1	YER049W	TPA1
YOR069W	VPS5	YHR060W	VMA22
YGL175C	SAE2	YLR352W	YLR352W
YML117W	NAB6	YOR275C	RIM20
YNL218W	MGS1	YHR134W	WSS1
YBL051C	PIN4	YNL116W	DMA2
YDR320C	SWA2	YGL212W	VAM7
YPL129W	TAF14	YOR001W	RRP6
YDR176W	NGG1	YNL107W	YAF9
YLR410W	VIP1	YDR289C	RTT103
YJR135C	MCM22	YHL002W	HSE1
YNL201C	PSY2	YFR036W	CDC26
YGR104C	SRB5	YER049W	TPA1
YGR270W	YTA7	YPR120C	CLB5
YMR092C	AIP1	YDR129C	SAC6
YCL037C	SRO9	YCR066W	RAD18
YDR477W	SNF1	YNR072W	HXT17
YGL025C	PGD1	YLR370C	ARC18
YNL025C	SSN8	YCR081W	SRB8
YDR318W	MCM21	YDR289C	RTT103
YBL058W	SHP1	YLR370C	ARC18
YLR135W	SLX4	YOR080W	DIA2
YJL004C	SYS1	YNL107W	YAF9
YGL133W	ITC1	YDR507C	GIN4
YDR108W	GSG1	YHR060W	VMA22
YIL159W	BNR1	YJL172W	CPS1
YDL013W	SLX5	YCR086W	CSM1
YKR099W	BAS1	YBR280C	SAF1
YJL060W	BNA3	YBR215W	HPC2

Predictions at threshold level 0.75 continued...			
**Gene1 of predicted pair**		**Gene2 of predicted pair**	
**Systematic Name**	**Standard Name**	**Systematic Name**	**Standard Name**
YGR054W	YGR054W	YJR090C	GRR1
YML115C	VAN1	YML012W	ERV25
YOL018C	TLG2	YDR153C	ENT5
YPR084W	YPR084W	YGL212W	VAM7
YBR171W	SEC66	YBL025W	RRN10
YLR234W	TOP3	YNL117W	MLS1
YJL124C	LSM1	YML112W	CTK3
YLL007C	YLL007C	YJR135C	MCM22
YHR060W	VMA22	YBL025W	RRN10
YBR171W	SEC66	YEL036C	ANP1
YGL115W	SNF4	YBR280C	SAF1
YLR292C	SEC72	YEL053C	MAK10
YGL060W	YBP2	YBR107C	IML3
YLR381W	CTF3	YLR032W	RAD5
YKR014C	YPT52	YOR035C	SHE4
YPL256C	CLN2	YHR115C	DMA1
YLR386W	VAC14	YPL161C	BEM4
YML115C	VAN1	YJR084W	CSN12
YNL016W	PUB1	YJR035W	RAD26
YGL147C	RPL9A	YER116C	SLX8
YKL213C	DOA1	YMR038C	CCS1
YOR132W	VPS17	YNR032W	PPG1
YGR249W	MGA1	YLR094C	GIS3
YIR005W	IST3	YFR036W	CDC26
YPR040W	TIP41	YPL152W	RRD2
YNL215W	IES2	YHR039C-A	VMA10
YLR399C	BDF1	YKL110C	KTI12
YMR080C	NAM7	YOR076C	SKI7
YOR127W	RGA1	YOR275C	RIM20

Predictions at threshold level 0.75 continued...			
Gene1 of predicted pair		Gene2 of predicted pair	
Systematic Name	Standard Name	Systematic Name	Standard Name
YDL047W	SIT4	YKR030W	GMH1
YHR013C	ARD1	YMR038C	CCS1
YBL079W	NUP170	YML057W	CMP2
YDR383C	NKP1	YLR254C	NDL1
YER087C-B	SBH1	YDL006W	PTC1
YIL076W	SEC28	YNL307C	MCK1
YDL083C	RPS16B	YDL077C	VAM6
YKR099W	BAS1	YBR036C	CSG2
YGL226C-A	OST5	YEL036C	ANP1
YDR260C	SWM1	YPL152W	RRD2
YDR443C	SSN2	YOR270C	VPH1
YAL024C	LTE1	YMR039C	SUB1
YGL025C	PGD1	YNL079C	TPM1
YLL007C	YLL007C	YGL124C	MON1
YJR024C	MDE1	YGL016W	KAP122
YBR126C	TPS1	YOR035C	SHE4
YHR012W	VPS29	YOR327C	SNC2
YGR104C	SRB5	YDR378C	LSM6
YDL116W	NUP84	YHR013C	ARD1
YLR381W	CTF3	YDR363W-A	SEM1
YGL147C	RPL9A	YOR270C	VPH1
YEL053C	MAK10	YLR370C	ARC18
YKR072C	SIS2	YGL031C	RPL24A
YDR254W	CHL4	YPL152W	RRD2
YLR095C	IOC2	YHR060W	VMA22
YMR172W	HOT1	YCR086W	CSM1
YHR205W	SCH9	YER096W	SHC1
YHR157W	REC104	YMR133W	REC114
YDR174W	HMO1	YOL054W	PSH1

Predictions at threshold level 0.75 continued...			
Gene1 of predicted pair		Gene2 of predicted pair	
Systematic Name	Standard Name	Systematic Name	Standard Name
YOR312C	RPL20B	YKL041W	VPS24
YNL264C	PDR17	YJR035W	RAD26
YHR134W	WSS1	YDR254W	CHL4
YMR055C	BUB2	YDL006W	PTC1
YJL154C	VPS35	YKL119C	VPH2
YDR293C	SSD1	YLR360W	VPS38
YNL215W	IES2	YDL002C	NHP10
YOR109W	INP53	YJR058C	APS2
YER110C	KAP123	YKR024C	DBP7
YML016C	PPZ1	YDR200C	VPS64
YPR131C	NAT3	YLR018C	POM34
YDR507C	GIN4	YCL024W	KCC4
YKL048C	ELM1	YGL124C	MON1
YOR337W	TEA1	YNL058C	YNL058C
YOR141C	ARP8	YOL081W	IRA2
YOR368W	RAD17	YGR188C	BUB1
YHR013C	ARD1	YJL006C	CTK2
YPL022W	RAD1	YDR207C	UME6
YPL046C	ELC1	YGL208W	SIP2
YGR249W	MGA1	YNL041C	COG6
YOR109W	INP53	YER120W	SCS2
YLR192C	HCR1	YBR171W	SEC66
YJL151C	SNA3	YLR144C	ACF2
YER179W	DMC1	YLR219W	MSC3
YPR120C	CLB5	YDR207C	UME6
YLR206W	ENT2	YNR006W	VPS27
YMR311C	GLC8	YEL060C	PRB1
YLR094C	GIS3	YMR311C	GLC8
YBL051C	PIN4	YNL107W	YAF9

Predictions at threshold level 0.75 continued...			
Gene1 of predicted pair		Gene2 of predicted pair	
Systematic Name	Standard Name	Systematic Name	Standard Name
YHR178W	STB5	YBR215W	HPC2
YJR040W	GEF1	YPR032W	SRO7
YDL192W	ARF1	YBR280C	SAF1
YBR057C	MUM2	YMR116C	ASC1
YLR240W	VPS34	YGL095C	VPS45
YGL163C	RAD54	YLR370C	ARC18
YPL024W	RMI1	YMR038C	CCS1
YNL069C	RPL16B	YER122C	GLO3
YPR036W	VMA13	YOR066W	MSA1
YKR048C	NAP1	YJL187C	SWE1
YLR093C	NYV1	YEL060C	PRB1
YPR018W	RLF2	YPL256C	CLN2
YDR245W	MNN10	YDR137W	RGP1
YKL116C	PRR1	YDR207C	UME6
YHR178W	STB5	YCR077C	PAT1
YGL147C	RPL9A	YMR214W	SCJ1
YHR108W	GGA2	YDR153C	ENT5
YJR084W	CSN12	YLR180W	SAM1
YHL006C	SHU1	YDR078C	SHU2
YJL053W	PEP8	YOL018C	TLG2
YGL212W	VAM7	YEL036C	ANP1
YMR116C	ASC1	YDR440W	DOT1
YLR292C	SEC72	YGR108W	CLB1
YGR108W	CLB1	YHR111W	UBA4
YLR079W	SIC1	YMR125W	STO1
YNL248C	RPA49	YHR013C	ARD1
YLR090W	XDJ1	YLR180W	SAM1
YDR309C	GIC2	YPL256C	CLN2
YGL163C	RAD54	YKL114C	APN1

Predictions at threshold level 0.75 continued...			
Gene1 of predicted pair		Gene2 of predicted pair	
Systematic Name	Standard Name	Systematic Name	Standard Name
YMR125W	STO1	YKL068W	NUP100
YPR084W	YPR084W	YEL043W	YEL043W
YDR293C	SSD1	YMR216C	SKY1
YLR234W	TOP3	YPR046W	MCM16
YDR137W	RGP1	YJL095W	BCK1
YBR217W	ATG12	YOR035C	SHE4
YNL093W	YPT53	YGL198W	YIP4
YDL080C	THI3	YML097C	VPS9
YLR006C	SSK1	YIL034C	CAP2
YBR278W	DPB3	YDL002C	NHP10
YLR292C	SEC72	YGL237C	HAP2
YGR014W	MSB2	YDL006W	PTC1
YDR004W	RAD57	YBR215W	HPC2
YJL124C	LSM1	YPL152W	RRD2
YFR040W	SAP155	YJR032W	CPR7
YOR124C	UBP2	YLR350W	ORM2
YLR384C	IKI3	YHR187W	IKI1
YML115C	VAN1	YMR133W	REC114
YDL065C	PEX19	YLR309C	IMH1
YDR293C	SSD1	YOR069W	VPS5
YDR293C	SSD1	YDR309C	GIC2
YFR010W	UBP6	YGR135W	PRE9
YKL116C	PRR1	YBR019C	GAL10
YPL253C	VIK1	YPR046W	MCM16
YOR127W	RGA1	YHL031C	GOS1
YBR030W	YBR030W	YLR386W	VAC14
YKR099W	BAS1	YDR310C	SUM1
YGL147C	RPL9A	YDL184C	RPL41A
YOL051W	GAL11	YLR399C	BDF1

Predictions at threshold level 0.75 continued...			
Gene1 of predicted pair		Gene2 of predicted pair	
Systematic Name	Standard Name	Systematic Name	Standard Name
YPL152W	RRD2	YIL103W	DPH1
YOR109W	INP53	YOL018C	TLG2
YML115C	VAN1	YCR053W	THR4
YDL154W	MSH5	YGL249W	ZIP2
YFL033C	RIM15	YDR310C	SUM1
YLR079W	SIC1	YER110C	KAP123
YOR195W	SLK19	YKL101W	HSL1
YPL042C	SSN3	YHR115C	DMA1
YGR159C	NSR1	YDR440W	DOT1
YLR360W	VPS38	YMR054W	STV1
YDL223C	HBT1	YJR032W	CPR7
YPL115C	BEM3	YJL183W	MNN11
YNL069C	RPL16B	YDR310C	SUM1
YDR245W	MNN10	YHR012W	VPS29
YDR080W	VPS41	YNL297C	MON2
YIL034C	CAP2	YBR200W	BEM1
YEL065W	SIT1	YER040W	GLN3
YLR360W	VPS38	YBR127C	VMA2
YML095C	RAD10	YDL059C	RAD59
YMR153W	NUP53	YCR053W	THR4
YKR020W	VPS51	YEL043W	YEL043W
YJL154C	VPS35	YJR135C	MCM22
YHR013C	ARD1	YNL107W	YAF9
YBL103C	RTG3	YPR119W	CLB2
YNL025C	SSN8	YCL037C	SRO9
YLR337C	VRP1	YML071C	COG8
YPL106C	SSE1	YLR370C	ARC18
YNL307C	MCK1	YGL100W	SEH1
YKL048C	ELM1	YJL006C	CTK2

Predictions at threshold level 0.75 continued...			
Gene1 of predicted pair		Gene2 of predicted pair	
Systematic Name	Standard Name	Systematic Name	Standard Name
YHL031C	GOS1	YDR378C	LSM6
YJL095W	BCK1	YBL072C	RPS8A
YNL291C	MID1	YHL031C	GOS1
YLR102C	APC9	YKL032C	IXR1
YJR056C	YJR056C	YDR310C	SUM1
YMR201C	RAD14	YGR258C	RAD2
YPL024W	RMI1	YJR140C	HIR3
YBR217W	ATG12	YMR133W	REC114
YPL129W	TAF14	YBR111W-A	SUS1
YEL065W	SIT1	YOR327C	SNC2
YLR288C	MEC3	YER032W	FIR1
YMR224C	MRE11	YKL006W	RPL14A
YKL126W	YPK1	YDR358W	GGA1
YDR440W	DOT1	YHL002W	HSE1
YEL043W	YEL043W	YER120W	SCS2
YBR107C	IML3	YLR452C	SST2
YML016C	PPZ1	YDR318W	MCM21
YHR060W	VMA22	YOL081W	IRA2
YOR358W	HAP5	YHR111W	UBA4
YDR129C	SAC6	YKL041W	VPS24
YDR379W	RGA2	YDL047W	SIT4
YMR026C	PEX12	YHR039C-A	VMA10
YLR148W	PEP3	YGL198W	YIP4
YDR507C	GIN4	YJL136C	RPS21B
YIR005W	IST3	YOR038C	HIR2
YGL033W	HOP2	YGL212W	VAM7
YOR132W	VPS17	YDL006W	PTC1
YDR076W	RAD55	YLR352W	YLR352W
YOR127W	RGA1	YBR108W	YBR108W

Predictions at threshold level 0.75 continued...			
Gene1 of predicted pair		Gene2 of predicted pair	
Systematic Name	Standard Name	Systematic Name	Standard Name
YIL036W	CST6	YML112W	CTK3
YJR040W	GEF1	YGR105W	VMA21
YOL054W	PSH1	YGL252C	RTG2
YJL176C	SWI3	YOR035C	SHE4
YOR132W	VPS17	YDR289C	RTT103
YPR018W	RLF2	YBR195C	MSI1
YLR216C	CPR6	YNL135C	FPR1
YOR033C	EXO1	YPR120C	CLB5
YLR309C	IMH1	YDR153C	ENT5
YDR225W	HTA1	YMR055C	BUB2
YGL190C	CDC55	YPR135W	CTF4
YBR200W	BEM1	YHR060W	VMA22
YOR059C	YOR059C	YMR313C	TGL3
YNL201C	PSY2	YIL103W	DPH1
YCR081W	SRB8	YLR079W	SIC1
YOR290C	SNF2	YMR152W	YIM1
YKR020W	VPS51	YHL020C	OPI1
YMR152W	YIM1	YMR230W	RPS10B
YCR066W	RAD18	YPL234C	TFP3
YPR084W	YPR084W	YOL018C	TLG2
YLR320W	MMS22	YMR230W	RPS10B
YMR133W	REC114	YMR152W	YIM1
YOR270C	VPH1	YNR072W	HXT17
YKR020W	VPS51	YEL053C	MAK10
YMR109W	MYO5	YGR054W	YGR054W
YOR195W	SLK19	YPL152W	RRD2
YDL184C	RPL41A	YKL126W	YPK1
YNL236W	SIN4	YHR108W	GGA2
YHR134W	WSS1	YJL187C	SWE1

Predictions at threshold level 0.75 continued...			
**Gene1 of predicted pair**		**Gene2 of predicted pair**	
**Systematic Name**	**Standard Name**	**Systematic Name**	**Standard Name**
YHR178W	STB5	YGL148W	ARO2
YOR043W	WHI2	YNL041C	COG6
YDR245W	MNN10	YJR035W	RAD26
YOR001W	RRP6	YHL031C	GOS1
YKL139W	CTK1	YLR410W	VIP1
YGL208W	SIP2	YPL256C	CLN2
YOR270C	VPH1	YPR030W	CSR2
YBR107C	IML3	YDL226C	GCS1
YBL008W	HIR1	YGL115W	SNF4
YLR234W	TOP3	YBL079W	NUP170
YNR072W	HXT17	YOL121C	RPS19A
YOR080W	DIA2	YER122C	GLO3
YJL172W	CPS1	YHR081W	LRP1
YFR008W	FAR7	YMR029C	FAR8
YMR039C	SUB1	YDL002C	NHP10
YKR001C	VPS1	YEL053C	MAK10
YBL087C	RPL23A	YGL031C	RPL24A
YNL054W	VAC7	YGL252C	RTG2
YIR005W	IST3	YLR182W	SWI6
YLR083C	EMP70	YNR032W	PPG1
YEL044W	IES6	YDR245W	MNN10
YGL078C	DBP3	YBR171W	SEC66
YDR443C	SSN2	YNL288W	CAF40
YBR084C-A	RPL19A	YHR200W	RPN10
YGR019W	UGA1	YLR360W	VPS38
YFR031C-A	RPL2A	YOR035C	SHE4
YJL183W	MNN11	YCR053W	THR4
YNL264C	PDR17	YJL183W	MNN11
YNL025C	SSN8	YDR137W	RGP1

Predictions at threshold level 0.75 continued...			
**Gene1 of predicted pair**		**Gene2 of predicted pair**	
**Systematic Name**	**Standard Name**	**Systematic Name**	**Standard Name**
YBR107C	IML3	YMR055C	BUB2
YLR410W	VIP1	YJL006C	CTK2
YAL020C	ATS1	YBR200W	BEM1
YJR140C	HIR3	YOR124C	UBP2
YBR019C	GAL10	YGL124C	MON1
YDR245W	MNN10	YJL187C	SWE1
YBR173C	UMP1	YGR184C	UBR1
YOL030W	GAS5	YMR038C	CCS1
YNL079C	TPM1	YHR158C	KEL1
YGL178W	MPT5	YMR312W	ELP6
YMR133W	REC114	YBR036C	CSG2
YGR072W	UPF3	YHR060W	VMA22
YDL101C	DUN1	YHR134W	WSS1
YKL117W	SBA1	YKR024C	DBP7
YIL053W	RHR2	YDL184C	RPL41A
YPL129W	TAF14	YGL252C	RTG2
YDR108W	GSG1	YNL044W	YIP3
YPL161C	BEM4	YNL076W	MKS1
YMR142C	RPL13B	YBR171W	SEC66
YDR358W	GGA1	YER040W	GLN3
YMR179W	SPT21	YKL101W	HSL1
YLR018C	POM34	YOR358W	HAP5
YDL184C	RPL41A	YBR280C	SAF1
YCL027W	FUS1	YMR032W	HOF1
YNL243W	SLA2	YPR135W	CTF4
YDR293C	SSD1	YPR051W	MAK3
YOR231W	MKK1	YDL006W	PTC1
YML115C	VAN1	YDR456W	NHX1
YMR038C	CCS1	YDR207C	UME6

Predictions at threshold level 0.75 continued...			
Gene1 of predicted pair		Gene2 of predicted pair	
Systematic Name	Standard Name	Systematic Name	Standard Name
YLR360W	VPS38	YGR020C	VMA7
YPL037C	EGD1	YML001W	YPT7
YOR312C	RPL20B	YJR035W	RAD26
YER044C	ERG28	YOR066W	MSA1
YPL137C	GIP3	YJR032W	CPR7
YBR280C	SAF1	YOL081W	IRA2
YMR198W	CIK1	YLR386W	VAC14
YMR198W	CIK1	YKL032C	IXR1
YOR106W	VAM3	YDL192W	ARF1
YPR017C	DSS4	YOL112W	MSB4
YJL095W	BCK1	YER040W	GLN3
YBR082C	UBC4	YDR207C	UME6
YJL029C	VPS53	YJL164C	TPK1
YEL065W	SIT1	YDR207C	UME6
YJR032W	CPR7	YEL042W	GDA1
YLR447C	VMA6	YOL018C	TLG2
YMR038C	CCS1	YLR032W	RAD5
YPL024W	RMI1	YMR133W	REC114
YLL039C	UBI4	YJR099W	YUH1
YHR111W	UBA4	YDR309C	GIC2
YML115C	VAN1	YBL103C	RTG3
YJL154C	VPS35	YOR106W	VAM3
YBR107C	IML3	YJR060W	CBF1
YBR127C	VMA2	YGR105W	VMA21
YLR288C	MEC3	YMR038C	CCS1
YPR084W	YPR084W	YDR363W-A	SEM1
YHR111W	UBA4	YKL101W	HSL1
YLL007C	YLL007C	YBR171W	SEC66
YIL008W	URM1	YGR188C	BUB1

Predictions at threshold level 0.75 continued...			
Gene1 of predicted pair		Gene2 of predicted pair	
Systematic Name	Standard Name	Systematic Name	Standard Name
YPR046W	MCM16	YGL124C	MON1
YML103C	NUP188	YGL252C	RTG2
YDR103W	STE5	YLR362W	STE11
YMR192W	GYL1	YPL249C	GYP5
YOR132W	VPS17	YDR484W	VPS52
YMR172W	HOT1	YOR023C	AHC1
YGR105W	VMA21	YNL107W	YAF9
YKL126W	YPK1	YML071C	COG8
YFR010W	UBP6	YGL100W	SEH1
YPL018W	CTF19	YBR114W	RAD16
YLR447C	VMA6	YER122C	GLO3
YDL116W	NUP84	YOR023C	AHC1
YJL058C	BIT61	YPR046W	MCM16
YDR507C	GIN4	YHR111W	UBA4
YMR116C	ASC1	YJL006C	CTK2
YBR200W	BEM1	YLR447C	VMA6
YFR010W	UBP6	YNL325C	FIG4
YDR507C	GIN4	YCL032W	STE50
YML016C	PPZ1	YPL253C	VIK1
YHR081W	LRP1	YOL081W	IRA2
YEL065W	SIT1	YLR320W	MMS22
YDR477W	SNF1	YOR035C	SHE4
YJR035W	RAD26	YLR182W	SWI6
YGL178W	MPT5	YDR334W	SWR1
YPL057C	SUR1	YER161C	SPT2
YLR095C	IOC2	YJR056C	YJR056C
YDL006W	PTC1	YML097C	VPS9
YDR155C	CPR1	YLR233C	EST1
YDR254W	CHL4	YAL002W	VPS8

Predictions at threshold level 0.75 continued...			
Gene1 of predicted pair		Gene2 of predicted pair	
Systematic Name	Standard Name	Systematic Name	Standard Name
YBR010W	HHT1	YOR141C	ARP8
YGL033W	HOP2	YIL076W	SEC28
YPL042C	SSN3	YMR202W	ERG2
YFR010W	UBP6	YJR032W	CPR7
YBL058W	SHP1	YGL212W	VAM7
YGL115W	SNF4	YJR060W	CBF1
YPL024W	RMI1	YBL103C	RTG3
YGR054W	YGR054W	YDR289C	RTT103
YNL238W	KEX2	YBR280C	SAF1
YHR012W	VPS29	YER049W	TPA1
YKL116C	PRR1	YNL238W	KEX2
YMR172W	HOT1	YMR127C	SAS2
YGL190C	CDC55	YKL113C	RAD27
YDR335W	MSN5	YGR270W	YTA7
YAL024C	LTE1	YBR195C	MSI1
YLR386W	VAC14	YDR469W	SDC1
YOR132W	VPS17	YDL192W	ARF1
YHR111W	UBA4	YLR254C	NDL1
YER044C	ERG28	YPL161C	BEM4
YEL050C	RML2	YPL018W	CTF19
YJL124C	LSM1	YER139C	YER139C
YPL057C	SUR1	YMR214W	SCJ1
YML115C	VAN1	YBR036C	CSG2
YLR234W	TOP3	YML115C	VAN1
YER144C	UBP5	YNL307C	MCK1
YJL172W	CPS1	YLR093C	NYV1
YBR194W	SOY1	YJR135C	MCM22
YML109W	ZDS2	YER124C	DSE1
YGL163C	RAD54	YFR036W	CDC26

Predictions at threshold level 0.75 continued...			
Gene1 of predicted pair		Gene2 of predicted pair	
Systematic Name	Standard Name	Systematic Name	Standard Name
YKR014C	YPT52	YBR181C	RPS6B
YFR031C-A	RPL2A	YCL037C	SRO9
YMR026C	PEX12	YFR036W	CDC26
YML115C	VAN1	YKR001C	VPS1
YMR095C	SNO1	YGL033W	HOP2
YBL058W	SHP1	YER040W	GLN3
YGR249W	MGA1	YPL161C	BEM4
YGL051W	MST27	YAR033W	MST28
YOR132W	VPS17	YMR216C	SKY1
YCL037C	SRO9	YOL018C	TLG2
YLR370C	ARC18	YER122C	GLO3
YEL009C	GCN4	YBR010W	HHT1
YHR134W	WSS1	YGL237C	HAP2
YHR200W	RPN10	YKR030W	GMH1
YNL032W	SIW14	YPL161C	BEM4
YML109W	ZDS2	YCR077C	PAT1
YNL058C	YNL058C	YJL183W	MNN11
YFR032C-A	RPL29	YMR205C	PFK2
YGR252W	GCN5	YDR216W	ADR1
YBR215W	HPC2	YGL175C	SAE2
YGR241C	YAP1802	YNL238W	KEX2
YLR292C	SEC72	YLR233C	EST1
YPL129W	TAF14	YGR275W	RTT102
YDR477W	SNF1	YNL076W	MKS1
YJL117W	PHO86	YJL183W	MNN11
YDL083C	RPS16B	YER040W	GLN3
YDR379W	RGA2	YNL116W	DMA2
YGL212W	VAM7	YDL077C	VAM6
YNL139C	RLR1	YER063W	THO1

Predictions at threshold level 0.75 continued...			
Gene1 of predicted pair		Gene2 of predicted pair	
Systematic Name	Standard Name	Systematic Name	Standard Name
YHR060W	VMA22	YGL054C	ERV14
YHR012W	VPS29	YJR135C	MCM22
YDR496C	PUF6	YOL041C	NOP12
YOR033C	EXO1	YMR055C	BUB2
YGL003C	CDH1	YBR019C	GAL10
YMR152W	YIM1	YBR280C	SAF1
YDR439W	LRS4	YGL252C	RTG2
YJL176C	SWI3	YBR009C	HHF1
YBR084C-A	RPL19A	YPL256C	CLN2
YNL106C	INP52	YGL212W	VAM7
YGR252W	GCN5	YDR440W	DOT1
YCL001W	RER1	YNL127W	FAR11
YHR039C-A	VMA10	YNL041C	COG6
YFL023W	BUD27	YKR035W-A	DID2
YML115C	VAN1	YNL307C	MCK1
YMR199W	CLN1	YKL101W	HSL1
YER001W	MNN1	YML071C	COG8
YGR249W	MGA1	YLR398C	SKI2
YFL033C	RIM15	YHL020C	OPI1
YML109W	ZDS2	YOR043W	WHI2
YPR084W	YPR084W	YAR014C	BUD14
YBL008W	HIR1	YJL124C	LSM1
YNR072W	HXT17	YNL076W	MKS1
YMR198W	CIK1	YLR032W	RAD5
YOR290C	SNF2	YPR046W	MCM16
YDL184C	RPL41A	YER144C	UBP5
YHR013C	ARD1	YLR032W	RAD5
YJR058C	APS2	YOR327C	SNC2
YMR230W	RPS10B	YOL081W	IRA2

Predictions at threshold level 0.75 continued...			
Gene1 of predicted pair		Gene2 of predicted pair	
Systematic Name	Standard Name	Systematic Name	Standard Name
YGR108W	CLB1	YIL008W	URM1
YFR040W	SAP155	YPL234C	TFP3
YHL031C	GOS1	YMR054W	STV1
YGR019W	UGA1	YGR054W	YGR054W
YNL206C	RTT106	YHR115C	DMA1
YBR107C	IML3	YJR090C	GRR1
YPR051W	MAK3	YJR090C	GRR1
YDR103W	STE5	YDL159W	STE7
YLR182W	SWI6	YNL225C	CNM67
YNR052C	POP2	YER116C	SLX8
YHR039C-A	VMA10	YER122C	GLO3
YLR090W	XDJ1	YBR200W	BEM1
YOL090W	MSH2	YJR035W	RAD26
YIL159W	BNR1	YML115C	VAN1
YOR069W	VPS5	YPL120W	VPS30
YAL014C	SYN8	YHL031C	GOS1
YKL048C	ELM1	YHR200W	RPN10
YEL053C	MAK10	YJR135C	MCM22
YGL212W	VAM7	YMR133W	REC114
YJL060W	BNA3	YPR046W	MCM16
YMR039C	SUB1	YCR077C	PAT1
YBR171W	SEC66	YHL002W	HSE1
YKL048C	ELM1	YJL190C	RPS22A
YKL032C	IXR1	YBL101C	ECM21
YMR224C	MRE11	YBR107C	IML3
YML112W	CTK3	YNL225C	CNM67
YKR057W	RPS21A	YNL116W	DMA2
YKR061W	KTR2	YLR399C	BDF1
YBR073W	RDH54	YBR107C	IML3

Predictions at threshold level 0.75 continued...			
Gene1 of predicted pair		Gene2 of predicted pair	
Systematic Name	Standard Name	Systematic Name	Standard Name
YPL019C	VTC3	YOR270C	VPH1
YOR066W	MSA1	YOR327C	SNC2
YBR200W	BEM1	YBL025W	RRN10
YDR004W	RAD57	YEL056W	HAT2
YJR010C-A	SPC1	YDL006W	PTC1
YLR083C	EMP70	YOL108C	INO4
YHL031C	GOS1	YOL108C	INO4
YBR133C	HSL7	YKL101W	HSL1
YGR108W	CLB1	YGL003C	CDH1
YCL037C	SRO9	YKL160W	ELF1
YIR005W	IST3	YNL225C	CNM67
YNL106C	INP52	YPL152W	RRD2
YJL176C	SWI3	YHL025W	SNF6
YOR035C	SHE4	YDL192W	ARF1
YMR055C	BUB2	YDR254W	CHL4
YDR108W	GSG1	YDR310C	SUM1
YGL066W	SGF73	YDR216W	ADR1
YPL240C	HSP82	YJR136C	YJR136C
YDR443C	SSN2	YNL236W	SIN4
YGR184C	UBR1	YNL230C	ELA1
YKL213C	DOA1	YAL002W	VPS8
YDR378C	LSM6	YBL101C	ECM21
YDR477W	SNF1	YDR310C	SUM1
YMR004W	MVP1	YPL120W	VPS30
YNL094W	APP1	YOR284W	HUA2
YLR362W	STE11	YLR452C	SST2
YPL158C	YPL158C	YOL081W	IRA2
YDR192C	NUP42	YHR052W	CIC1
YGR072W	UPF3	YHR039C-A	VMA10

Predictions at threshold level 0.75 continued...			
Gene1 of predicted pair		Gene2 of predicted pair	
Systematic Name	Standard Name	Systematic Name	Standard Name
YLR386W	VAC14	YHR152W	SPO12
YLR315W	NKP2	YAR014C	BUD14
YIL008W	URM1	YHR114W	BZZ1
YNL041C	COG6	YNL051W	COG5
YOR327C	SNC2	YDR153C	ENT5
YHR060W	VMA22	YDR261C	EXG2
YKL025C	PAN3	YGL087C	MMS2
YGR268C	HUA1	YLR144C	ACF2
YIL053W	RHR2	YER040W	GLN3
YPR036W	VMA13	YBR019C	GAL10
YLR192C	HCR1	YKR035W-A	DID2
YER116C	SLX8	YGL212W	VAM7
YER116C	SLX8	YDL083C	RPS16B
YPL161C	BEM4	YJR033C	RAV1
YDR484W	VPS52	YGL003C	CDH1
YHR012W	VPS29	YGL124C	MON1
YKL126W	YPK1	YPL137C	GIP3
YHR152W	SPO12	YKR048C	NAP1
YCR002C	CDC10	YNL078W	NIS1
YFL013C	IES1	YDR363W-A	SEM1
YDR358W	GGA1	YER139C	YER139C
YPL137C	GIP3	YJL095W	BCK1
YIL076W	SEC28	YJL095W	BCK1
YDR293C	SSD1	YPR072W	NOT5
YNL206C	RTT106	YML028W	TSA1
YNL288W	CAF40	YLR381W	CTF3
YCL067C	HMLALPHA2	YMR042W	ARG80
YJR060W	CBF1	YHR039C-A	VMA10
YFR030W	MET10	YAR014C	BUD14

Predictions at threshold level 0.75 continued...			
**Gene1 of predicted pair**		**Gene2 of predicted pair**	
**Systematic Name**	**Standard Name**	**Systematic Name**	**Standard Name**
YHR134W	WSS1	YBR171W	SEC66
YGL115W	SNF4	YEL043W	YEL043W
YNL032W	SIW14	YBR171W	SEC66
YDR484W	VPS52	YEL060C	PRB1
YOR076C	SKI7	YDR083W	RRP8
YNL229C	URE2	YBL087C	RPL23A
YHR060W	VMA22	YDL077C	VAM6
YIL076W	SEC28	YKL160W	ELF1
YDR477W	SNF1	YFR036W	CDC26
YOL030W	GAS5	YNL016W	PUB1
YHR200W	RPN10	YML028W	TSA1
YNL288W	CAF40	YEL056W	HAT2
YGL212W	VAM7	YOR036W	PEP12
YIL036W	CST6	YGR188C	BUB1
YDR156W	RPA14	YML062C	MFT1
YKL139W	CTK1	YDR183W	PLP1
YJL058C	BIT61	YPL018W	CTF19
YDR225W	HTA1	YBR133C	HSL7
YJR040W	GEF1	YOL018C	TLG2
YPR131C	NAT3	YMR231W	PEP5
YBR200W	BEM1	YNL289W	PCL1
YLR192C	HCR1	YER116C	SLX8
YJR056C	YJR056C	YOR035C	SHE4
YDL184C	RPL41A	YPL161C	BEM4
YML115C	VAN1	YHR205W	SCH9
YBR111W-A	SUS1	YNL031C	HHT2
YOR270C	VPH1	YER072W	VTC1
YIL076W	SEC28	YNL325C	FIG4
YFR021W	ATG18	YER162C	RAD4

Predictions at threshold level 0.75 continued...			
Gene1 of predicted pair		Gene2 of predicted pair	
Systematic Name	Standard Name	Systematic Name	Standard Name
YDR293C	SSD1	YJR090C	GRR1
YNL087W	TCB2	YKR099W	BAS1
YGR105W	VMA21	YNR032W	PPG1
YOL111C	MDY2	YEL060C	PRB1
YDR443C	SSN2	YDR137W	RGP1
YDR078C	SHU2	YLR376C	PSY3
YGL212W	VAM7	YAR014C	BUD14
YHR156C	LIN1	YDL083C	RPS16B
YDR477W	SNF1	YMR142C	RPL13B
YLR119W	SRN2	YDL192W	ARF1
YDR443C	SSN2	YGR104C	SRB5
YPL129W	TAF14	YDR146C	SWI5
YFR010W	UBP6	YOR080W	DIA2
YDL020C	RPN4	YLR233C	EST1
YOR270C	VPH1	YDR207C	UME6
YOR127W	RGA1	YGR270W	YTA7
YPR036W	VMA13	YKL101W	HSL1
YMR039C	SUB1	YJL006C	CTK2
YDL002C	NHP10	YML097C	VPS9
YJL172W	CPS1	YOL121C	RPS19A
YBL103C	RTG3	YKL048C	ELM1
YLR396C	VPS33	YOR036W	PEP12
YOR069W	VPS5	YLR447C	VMA6
YDR477W	SNF1	YPL253C	VIK1
YGL115W	SNF4	YOR066W	MSA1
YDR310C	SUM1	YOL081W	IRA2
YDL116W	NUP84	YNL054W	VAC7
YOR112W	CEX1	YGL212W	VAM7
YLR410W	VIP1	YJL140W	RPB4

Predictions at threshold level 0.75 continued...			
Gene1 of predicted pair		Gene2 of predicted pair	
Systematic Name	Standard Name	Systematic Name	Standard Name
YJR059W	PTK2	YNL199C	GCR2
YBL047C	EDE1	YGL163C	RAD54
YNL106C	INP52	YDL226C	GCS1
YDL020C	RPN4	YGR072W	UPF3
YLL039C	UBI4	YOR035C	SHE4
YDL013W	SLX5	YFR036W	CDC26
YGR270W	YTA7	YEL053C	MAK10
YNL215W	IES2	YKL048C	ELM1
YDL020C	RPN4	YGR188C	BUB1
YLR095C	IOC2	YDL184C	RPL41A
YHR098C	SFB3	YML041C	VPS71
YPR101W	SNT309	YIR005W	IST3
YKR035W-A	DID2	YCR053W	THR4
YPR017C	DSS4	YDR080W	VPS41
YKL110C	KTI12	YGR188C	BUB1
YDR484W	VPS52	YNL041C	COG6
YBR017C	KAP104	YKL068W	NUP100
YBR200W	BEM1	YDR507C	GIN4
YLR234W	TOP3	YGL003C	CDH1
YGR249W	MGA1	YOR270C	VPH1
YER144C	UBP5	YDL083C	RPS16B
YOL112W	MSB4	YNR049C	MSO1
YPL129W	TAF14	YOL148C	SPT20
YHR206W	SKN7	YER118C	SHO1
YDR146C	SWI5	YPL161C	BEM4
YER087C-B	SBH1	YGR100W	MDR1
YDR225W	HTA1	YJR135C	MCM22
YBL058W	SHP1	YOR043W	WHI2
YPR046W	MCM16	YNL225C	CNM67

Predictions at threshold level 0.75 continued...			
Gene1 of predicted pair		Gene2 of predicted pair	
Systematic Name	Standard Name	Systematic Name	Standard Name
YHL020C	OPI1	YHR012W	VPS29
YLR090W	XDJ1	YIL159W	BNR1
YDR076W	RAD55	YLR370C	ARC18
YPR017C	DSS4	YDR310C	SUM1
YGL223C	COG1	YJR035W	RAD26
YER075C	PTP3	YOR231W	MKK1
YLR216C	CPR6	YNL069C	RPL16B
YPL115C	BEM3	YGR019W	UGA1
YDR225W	HTA1	YER110C	KAP123
YPR084W	YPR084W	YER161C	SPT2
YLR119W	SRN2	YBR019C	GAL10
YGR014W	MSB2	YPL180W	TCO89
YMR004W	MVP1	YOR106W	VAM3
YBR200W	BEM1	YLR371W	ROM2
YML115C	VAN1	YBR082C	UBC4
YKL116C	PRR1	YBR280C	SAF1
YNL127W	FAR11	YNR032W	PPG1
YDR202C	RAV2	YOL108C	INO4
YGL078C	DBP3	YKR035W-A	DID2
YER116C	SLX8	YBR171W	SEC66
YPL024W	RMI1	YBL025W	RRN10
YPL152W	RRD2	YNL116W	DMA2
YIL076W	SEC28	YLR182W	SWI6
YDR254W	CHL4	YJR032W	CPR7
YBR030W	YBR030W	YPL195W	APL5
YLR386W	VAC14	YBR258C	SHG1
YLR362W	STE11	YBL075C	SSA3
YDR156W	RPA14	YGR188C	BUB1
YEL043W	YEL043W	YDL006W	PTC1

Predictions at threshold level 0.75 continued...			
Gene1 of predicted pair		Gene2 of predicted pair	
Systematic Name	Standard Name	Systematic Name	Standard Name
YKL116C	PRR1	YKL048C	ELM1
YJR040W	GEF1	YKR001C	VPS1
YBR108W	YBR108W	YNL107W	YAF9
YOR083W	WHI5	YPL022W	RAD1
YMR054W	STV1	YER122C	GLO3
YJL006C	CTK2	YDR289C	RTT103
YBR084C-A	RPL19A	YBL087C	RPL23A
YGL147C	RPL9A	YNL041C	COG6
YHR200W	RPN10	YHR039C-A	VMA10
YGL237C	HAP2	YMR109W	MYO5
YOR270C	VPH1	YPR119W	CLB2
YDR171W	HSP42	YPR046W	MCM16
YGL147C	RPL9A	YDR207C	UME6
YLR079W	SIC1	YDR254W	CHL4
YEL009C	GCN4	YPR072W	NOT5
YLR102C	APC9	YHR115C	DMA1
YML001W	YPT7	YML071C	COG8
YLR095C	IOC2	YOL121C	RPS19A
YGR261C	APL6	YPL057C	SUR1
YNL106C	INP52	YDR080W	VPS41
YGL043W	DST1	YGL178W	MPT5
YOR023C	AHC1	YDR144C	MKC7
YGR067C	YGR067C	YPL240C	HSP82
YMR172W	HOT1	YPR046W	MCM16
YHR108W	GGA2	YOR124C	UBP2
YDL227C	HO	YML088W	UFO1
YJL058C	BIT61	YDR200C	VPS64
YMR026C	PEX12	YHR060W	VMA22
YGL025C	PGD1	YDL083C	RPS16B

Predictions at threshold level 0.75 continued...			
Gene1 of predicted pair		Gene2 of predicted pair	
Systematic Name	Standard Name	Systematic Name	Standard Name
YPL234C	TFP3	YKL119C	VPH2
YCR081W	SRB8	YKL139W	CTK1
YAL014C	SYN8	YOR106W	VAM3
YGL006W	PMC1	YLR093C	NYV1
YOR327C	SNC2	YLR309C	IMH1
YDR289C	RTT103	YNL225C	CNM67
YOR327C	SNC2	YNL127W	FAR11
YBR278W	DPB3	YJL124C	LSM1
YNL329C	PEX6	YMR089C	YTA12
YMR311C	GLC8	YLR398C	SKI2
YGL078C	DBP3	YFL023W	BUD27
YPR046W	MCM16	YLR180W	SAM1
YDR443C	SSN2	YEL053C	MAK10
YLR240W	VPS34	YDR129C	SAC6
YLR435W	TSR2	YOL081W	IRA2
YGL178W	MPT5	YBL061C	SKT5
YHR098C	SFB3	YBL061C	SKT5
YCR020C-A	MAK31	YDR320C	SWA2
YPR036W	VMA13	YFR036W	CDC26
YOR035C	SHE4	YPR119W	CLB2
YDR443C	SSN2	YNL238W	KEX2
YDR156W	RPA14	YGR217W	CCH1
YNL135C	FPR1	YGL252C	RTG2
YIL159W	BNR1	YGL115W	SNF4
YLR292C	SEC72	YPL018W	CTF19
YKL139W	CTK1	YPL248C	GAL4
YDR245W	MNN10	YLR425W	TUS1
YER096W	SHC1	YJL095W	BCK1
YDR335W	MSN5	YHR013C	ARD1

Predictions at threshold level 0.75 continued...			
Gene1 of predicted pair		Gene2 of predicted pair	
Systematic Name	Standard Name	Systematic Name	Standard Name
YLR079W	SIC1	YHR205W	SCH9
YGR275W	RTT102	YLR357W	RSC2
YGR133W	PEX4	YBL047C	EDE1
YLR441C	RPS1A	YOR066W	MSA1
YER110C	KAP123	YHR114W	BZZ1
YIL053W	RHR2	YHR081W	LRP1
YNL291C	MID1	YER116C	SLX8
YPL042C	SSN3	YBR194W	SOY1
YHR039C-A	VMA10	YLR398C	SKI2
YLR095C	IOC2	YEL060C	PRB1
YOR023C	AHC1	YGR054W	YGR054W
YGR239C	PEX21	YLR191W	PEX13
YBL021C	HAP3	YKL109W	HAP4
YBR195C	MSI1	YHR039C-A	VMA10
YDR108W	GSG1	YPL161C	BEM4
YIL072W	HOP1	YOR351C	MEK1
YHL031C	GOS1	YLR396C	VPS33
YNL325C	FIG4	YOR270C	VPH1
YPR084W	YPR084W	YML038C	YMD8
YPR029C	APL4	YOR036W	PEP12
YPL129W	TAF14	YKL109W	HAP4
YDR163W	CWC15	YJR050W	ISY1
YHR012W	VPS29	YJR090C	GRR1
YJR040W	GEF1	YDR207C	UME6
YIL036W	CST6	YJL124C	LSM1
YCR088W	ABP1	YDR310C	SUM1
YJL172W	CPS1	YNR072W	HXT17
YLR386W	VAC14	YOL121C	RPS19A
YLR095C	IOC2	YBR019C	GAL10

Predictions at threshold level 0.75 continued...			
Gene1 of predicted pair		Gene2 of predicted pair	
Systematic Name	Standard Name	Systematic Name	Standard Name
YNL106C	INP52	YJR060W	CBF1
YER044C	ERG28	YMR133W	REC114
YDR254W	CHL4	YPL161C	BEM4
YPL024W	RMI1	YER122C	GLO3
YGR249W	MGA1	YBL025W	RRN10
YOR043W	WHI2	YOR035C	SHE4
YLR083C	EMP70	YKR030W	GMH1
YOR270C	VPH1	YLR180W	SAM1
YDR507C	GIN4	YDR309C	GIC2
YDR076W	RAD55	YPR119W	CLB2
YPL129W	TAF14	YGR252W	GCN5
YDR146C	SWI5	YHR205W	SCH9
YNL288W	CAF40	YBL051C	PIN4
YGR108W	CLB1	YDL226C	GCS1
YKL007W	CAP1	YPR171W	BSP1
YOR109W	INP53	YER162C	RAD4
YER001W	MNN1	YGL223C	COG1
YGL147C	RPL9A	YLR192C	HCR1
YLL039C	UBI4	YPL050C	MNN9
YJL154C	VPS35	YOR327C	SNC2
YBL102W	SFT2	YDR153C	ENT5
YDR174W	HMO1	YKR061W	KTR2
YIL053W	RHR2	YOR337W	TEA1
YGL087C	MMS2	YPL161C	BEM4
YGL066W	SGF73	YJL140W	RPB4
YER087C-B	SBH1	YDL232W	OST4
YLR386W	VAC14	YER044C	ERG28
YDR108W	GSG1	YNL297C	MON2
YCR031C	RPS14A	YGR188C	BUB1

Predictions at threshold level 0.75 continued...			
Gene1 of predicted pair		Gene2 of predicted pair	
Systematic Name	Standard Name	Systematic Name	Standard Name
YGR261C	APL6	YNL051W	COG5
YDR174W	HMO1	YLR135W	SLX4
YGL100W	SEH1	YPL240C	HSP82
YLR148W	PEP3	YBL078C	ATG8
YLR061W	RPL22A	YGR133W	PEX4
YIL034C	CAP2	YJR090C	GRR1
YGL033W	HOP2	YBR019C	GAL10
YPR046W	MCM16	YDR358W	GGA1
YNL238W	KEX2	YOL121C	RPS19A
YHR098C	SFB3	YER016W	BIM1
YDR139C	RUB1	YLR128W	DCN1
YMR224C	MRE11	YML058W	SML1
YDR443C	SSN2	YGL003C	CDH1
YLR273C	PIG1	YIL045W	PIG2
YOR001W	RRP6	YOR038C	HIR2
YLR079W	SIC1	YBR215W	HPC2
YEL009C	GCN4	YPL042C	SSN3
YMR038C	CCS1	YLR350W	ORM2
YJL127C	SPT10	YOR001W	RRP6
YDR144C	MKC7	YPR046W	MCM16
YLR095C	IOC2	YLR386W	VAC14
YPR036W	VMA13	YPL161C	BEM4
YNL054W	VAC7	YNL107W	YAF9
YML013W	UBX2	YPR089W	YPR089W
YGR081C	SLX9	YMR038C	CCS1
YGL135W	RPL1B	YMR198W	CIK1
YEL050C	RML2	YBR200W	BEM1
YML115C	VAN1	YMR016C	SOK2
YPL115C	BEM3	YJL187C	SWE1

Predictions at threshold level 0.75 continued...			
Gene1 of predicted pair		Gene2 of predicted pair	
Systematic Name	Standard Name	Systematic Name	Standard Name
YNL238W	KEX2	YMR152W	YIM1
YJL036W	SNX4	YHR039C-A	VMA10
YLR192C	HCR1	YKR014C	YPT52
YPL057C	SUR1	YDR261C	EXG2
YMR142C	RPL13B	YDL184C	RPL41A
YBR215W	HPC2	YGL003C	CDH1
YJL124C	LSM1	YOL054W	PSH1
YMR004W	MVP1	YNR032W	PPG1
YLR360W	VPS38	YML115C	VAN1
YGL147C	RPL9A	YPL195W	APL5
YOR080W	DIA2	YLR182W	SWI6
YPL161C	BEM4	YDR469W	SDC1
YBR215W	HPC2	YLR370C	ARC18
YMR153W	NUP53	YHR081W	LRP1
YNR052C	POP2	YML016C	PPZ1
YDL116W	NUP84	YJR035W	RAD26
YER116C	SLX8	YKR099W	BAS1
YMR133W	REC114	YOR327C	SNC2
YEL009C	GCN4	YLR176C	RFX1
YOR023C	AHC1	YLR233C	EST1
YNL087W	TCB2	YLR441C	RPS1A
YKL126W	YPK1	YOR043W	WHI2
YBR200W	BEM1	YJR084W	CSN12
YNR032W	PPG1	YKR030W	GMH1
YDR293C	SSD1	YGL151W	NUT1
YMR224C	MRE11	YPR036W	VMA13
YDR108W	GSG1	YJL004C	SYS1
YLR170C	APS1	YOR036W	PEP12
YBR173C	UMP1	YBR019C	GAL10

Predictions at threshold level 0.75 continued...			
Gene1 of predicted pair		Gene2 of predicted pair	
Systematic Name	Standard Name	Systematic Name	Standard Name
YBL047C	EDE1	YPR120C	CLB5
YPR018W	RLF2	YGR081C	SLX9
YML103C	NUP188	YPL246C	RBD2
YMR109W	MYO5	YHR016C	YSC84
YHR012W	VPS29	YOL018C	TLG2
YPL129W	TAF14	YER161C	SPT2
YJR040W	GEF1	YGL115W	SNF4
YNL297C	MON2	YNR032W	PPG1
YGL170C	SPO74	YOL091W	SPO21
YNL041C	COG6	YDR153C	ENT5
YNL291C	MID1	YPL105C	YPL105C
YPR036W	VMA13	YLR371W	ROM2
YDR108W	GSG1	YDL006W	PTC1
YNL236W	SIN4	YLR306W	UBC12
YHR060W	VMA22	YEL043W	YEL043W
YML103C	NUP188	YKL117W	SBA1
YEL065W	SIT1	YER116C	SLX8
YDR004W	RAD57	YDL154W	MSH5
YLR240W	VPS34	YDR323C	PEP7
YDR202C	RAV2	YPL037C	EGD1
YJR040W	GEF1	YMR054W	STV1
YHR111W	UBA4	YGR152C	RSR1
YDR108W	GSG1	YCR066W	RAD18
YJR140C	HIR3	YBR089C-A	NHP6B
YGL237C	HAP2	YBR114W	RAD16
YNR052C	POP2	YPL022W	RAD1
YHR171W	ATG7	YOR327C	SNC2
YML001W	YPT7	YLR180W	SAM1
YLR292C	SEC72	YLR381W	CTF3

Predictions at threshold level 0.75 continued...			
Gene1 of predicted pair		Gene2 of predicted pair	
Systematic Name	Standard Name	Systematic Name	Standard Name
YNL288W	CAF40	YLR234W	TOP3
YHL031C	GOS1	YAL030W	SNC1
YDR378C	LSM6	YJL095W	BCK1
YDR146C	SWI5	YDR310C	SUM1
YGL163C	RAD54	YLR315W	NKP2
YKL007W	CAP1	YNL243W	SLA2
YOR337W	TEA1	YER162C	RAD4
YEL027W	CUP5	YLR447C	VMA6
YHR081W	LRP1	YMR152W	YIM1
YGL003C	CDH1	YLR182W	SWI6
YDR192C	NUP42	YLR131C	ACE2
YNL229C	URE2	YDL047W	SIT4
YLR191W	PEX13	YDR265W	PEX10
YJL124C	LSM1	YJL006C	CTK2
YOR033C	EXO1	YML028W	TSA1
YPR084W	YPR084W	YJR140C	HIR3
YOR106W	VAM3	YNR032W	PPG1
YBR019C	GAL10	YBL025W	RRN10
YFR010W	UBP6	YDL223C	HBT1
YHR073W	OSH3	YMR153W	NUP53
YDL116W	NUP84	YHR134W	WSS1
YDR378C	LSM6	YML112W	CTK3
YLR240W	VPS34	YLR360W	VPS38
YDR443C	SSN2	YIL159W	BNR1
YEL009C	GCN4	YGL252C	RTG2
YBR173C	UMP1	YER044C	ERG28
YKR095W	MLP1	YDR076W	RAD55
YGL147C	RPL9A	YLR386W	VAC14
YBR290W	BSD2	YKL068W	NUP100

Predictions at threshold level 0.75 continued...			
Gene1 of predicted pair		Gene2 of predicted pair	
Systematic Name	Standard Name	Systematic Name	Standard Name
YER116C	SLX8	YER040W	GLN3
YCR077C	PAT1	YGR135W	PRE9
YGR249W	MGA1	YLR192C	HCR1
YMR224C	MRE11	YMR179W	SPT21
YGR020C	VMA7	YJL095W	BCK1
YKR048C	NAP1	YNL078W	NIS1
YGL087C	MMS2	YJR032W	CPR7
YMR055C	BUB2	YDL013W	SLX5
YHR178W	STB5	YGL013C	PDR1
YDR293C	SSD1	YJL095W	BCK1
YNL087W	TCB2	YCR053W	THR4
YNL236W	SIN4	YDL226C	GCS1
YLR240W	VPS34	YLR396C	VPS33
YBR108W	YBR108W	YMR152W	YIM1
YEL065W	SIT1	YDR358W	GGA1
YFR024C-A	LSB3	YJL004C	SYS1
YPR131C	NAT3	YBR215W	HPC2
YOR066W	MSA1	YLR371W	ROM2
YKR099W	BAS1	YOR035C	SHE4
YOR106W	VAM3	YHR115C	DMA1
YLR090W	XDJ1	YMR142C	RPL13B
YLR399C	BDF1	YBR107C	IML3
YER042W	MXR1	YGR209C	TRX2
YDR496C	PUF6	YKL130C	SHE2
YOR327C	SNC2	YDL047W	SIT4
YPR084W	YPR084W	YHL020C	OPI1
YML115C	VAN1	YDR260C	SWM1
YNL291C	MID1	YJR033C	RAV1
YLR337C	VRP1	YKL006W	RPL14A

Predictions at threshold level 0.75 continued...			
Gene1 of predicted pair		Gene2 of predicted pair	
Systematic Name	Standard Name	Systematic Name	Standard Name
YMR039C	SUB1	YBR215W	HPC2
YBR030W	YBR030W	YER116C	SLX8
YOR083W	WHI5	YPR119W	CLB2
YNL199C	GCR2	YDR363W-A	SEM1
YLR433C	CNA1	YGR006W	PRP18
YPR084W	YPR084W	YLR360W	VPS38
YLR292C	SEC72	YJR090C	GRR1
YGL025C	PGD1	YNR072W	HXT17
YDR171W	HSP42	YDR318W	MCM21
YLR234W	TOP3	YJL138C	TIF2
YNL307C	MCK1	YIR005W	IST3
YEL043W	YEL043W	YLR309C	IMH1
YMR224C	MRE11	YML071C	COG8
YNL041C	COG6	YKR014C	YPT52
YLR095C	IOC2	YHR081W	LRP1
YEL043W	YEL043W	YML071C	COG8
YDR079C-A	TFB5	YPL240C	HSP82
YKL139W	CTK1	YCL011C	GBP2
YKR099W	BAS1	YNL076W	MKS1
YOR080W	DIA2	YPL018W	CTF19
YOR127W	RGA1	YDL047W	SIT4
YMR205C	PFK2	YBR131W	CCZ1
YDL020C	RPN4	YJR035W	RAD26
YPL240C	HSP82	YGR296W	YRF1-3
YIL159W	BNR1	YKR099W	BAS1
YDL184C	RPL41A	YFR036W	CDC26
YCL027W	FUS1	YKL092C	BUD2
YLR337C	VRP1	YNL079C	TPM1
YLR441C	RPS1A	YER139C	YER139C

Predictions at threshold level 0.75 continued...			
Gene1 of predicted pair		Gene2 of predicted pair	
Systematic Name	Standard Name	Systematic Name	Standard Name
YNL218W	MGS1	YBR228W	SLX1
YKR020W	VPS51	YHR039C-A	VMA10
YOL126C	MDH2	YMR164C	MSS11
YMR284W	YKU70	YOL030W	GAS5
YHR205W	SCH9	YNL271C	BNI1
YOR243C	PUS7	YGR071C	YGR071C
YDR245W	MNN10	YBL072C	RPS8A
YCR020C-A	MAK31	YNL107W	YAF9
YLR360W	VPS38	YAL002W	VPS8
YBR030W	YBR030W	YJL183W	MNN11
YGR054W	YGR054W	YAR014C	BUD14
YBL003C	HTA2	YNL107W	YAF9
YGL054C	ERV14	YKR030W	GMH1
YJR032W	CPR7	YNL076W	MKS1
YCR077C	PAT1	YBL101C	ECM21
YDR146C	SWI5	YML016C	PPZ1
YKR001C	VPS1	YJR060W	CBF1
YKL139W	CTK1	YML001W	YPT7
YFR010W	UBP6	YDR477W	SNF1
YBR108W	YBR108W	YPL161C	BEM4
YLR452C	SST2	YJL006C	CTK2
YDL005C	MED2	YNL236W	SIN4
YDL116W	NUP84	YML103C	NUP188
YHR178W	STB5	YGL237C	HAP2
YJR060W	CBF1	YER122C	GLO3
YIL076W	SEC28	YPR046W	MCM16
YHR039C-A	VMA10	YDL192W	ARF1
YKL139W	CTK1	YMR089C	YTA12
YPL115C	BEM3	YDR293C	SSD1

Predictions at threshold level 0.75 continued...			
Gene1 of predicted pair		Gene2 of predicted pair	
Systematic Name	Standard Name	Systematic Name	Standard Name
YGL087C	MMS2	YGR184C	UBR1
YEL050C	RML2	YDR318W	MCM21
YNL044W	YIP3	YJR032W	CPR7
YOR127W	RGA1	YHR111W	UBA4
YCR020C-A	MAK31	YJR033C	RAV1
YKL116C	PRR1	YMR230W	RPS10B
YBR231C	SWC5	YGL094C	PAN2
YJR040W	GEF1	YPL120W	VPS30
YNL325C	FIG4	YDL077C	VAM6
YDR144C	MKC7	YDR310C	SUM1
YBR200W	BEM1	YGL115W	SNF4
YPL105C	YPL105C	YNR032W	PPG1
YGL025C	PGD1	YPL248C	GAL4
YFR031C-A	RPL2A	YJL004C	SYS1
YNL236W	SIN4	YNL107W	YAF9
YDL116W	NUP84	YKR061W	KTR2
YML016C	PPZ1	YDR335W	MSN5
YMR127C	SAS2	YNL166C	BNI5
YPR131C	NAT3	YDR171W	HSP42
YDR245W	MNN10	YKL041W	VPS24
YMR109W	MYO5	YOR247W	SRL1
YNL025C	SSN8	YKL213C	DOA1
YLR083C	EMP70	YFR031C-A	RPL2A
YHR111W	UBA4	YOR327C	SNC2
YOR112W	CEX1	YML071C	COG8
YKL139W	CTK1	YMR016C	SOK2
YOR132W	VPS17	YBR108W	YBR108W
YGR104C	SRB5	YGL151W	NUT1
YGL212W	VAM7	YGL200C	EMP24

Predictions at threshold level 0.75 continued...			
Gene1 of predicted pair		Gene2 of predicted pair	
Systematic Name	Standard Name	Systematic Name	Standard Name
YHR013C	ARD1	YKL160W	ELF1
YML012W	ERV25	YNL238W	KEX2
YHL025W	SNF6	YLR357W	RSC2
YER052C	HOM3	YNL135C	FPR1
YLR398C	SKI2	YLR180W	SAM1
YLR315W	NKP2	YHR167W	THP2
YOR337W	TEA1	YMR026C	PEX12
YMR026C	PEX12	YOL121C	RPS19A
YNL199C	GCR2	YLR350W	ORM2
YHR013C	ARD1	YIR005W	IST3
YOR156C	NFI1	YDR469W	SDC1
YER120W	SCS2	YML071C	COG8
YPL253C	VIK1	YJR060W	CBF1
YEL053C	MAK10	YDL077C	VAM6
YML115C	VAN1	YKL119C	VPH2
YNL087W	TCB2	YMR152W	YIM1
YBR171W	SEC66	YBL047C	EDE1
YJL172W	CPS1	YDL192W	ARF1
YDR477W	SNF1	YOR106W	VAM3
YKR001C	VPS1	YMR204C	INP1
YLR456W	YLR456W	YPR119W	CLB2
YGR135W	PRE9	YAR014C	BUD14
YAL016W	TPD3	YDL047W	SIT4
YLR381W	CTF3	YJR063W	RPA12
YDR137W	RGP1	YOL111C	MDY2
YJL176C	SWI3	YHR134W	WSS1
YNL054W	VAC7	YDR335W	MSN5
YGL223C	COG1	YEL053C	MAK10
YLR095C	IOC2	YPL161C	BEM4

Predictions at threshold level 0.75 continued...			
Gene1 of predicted pair		Gene2 of predicted pair	
Systematic Name	Standard Name	Systematic Name	Standard Name
YEL065W	SIT1	YMR142C	RPL13B
YPR084W	YPR084W	YBL102W	SFT2
YMR142C	RPL13B	YDR207C	UME6
YCR065W	HCM1	YNL041C	COG6
YLR216C	CPR6	YGL124C	MON1
YIL034C	CAP2	YGL087C	MMS2
YOR127W	RGA1	YHR012W	VPS29
YPL161C	BEM4	YCR086W	CSM1
YGR019W	UGA1	YPL018W	CTF19
YGR104C	SRB5	YGL066W	SGF73
YNL087W	TCB2	YPL161C	BEM4
YMR192W	GYL1	YOR284W	HUA2
YKL135C	APL2	YDR358W	GGA1
YKR099W	BAS1	YOR327C	SNC2
YBR107C	IML3	YOL054W	PSH1
YLR386W	VAC14	YDR335W	MSN5
YJL053W	PEP8	YPL037C	EGD1
YOR080W	DIA2	YDR363W-A	SEM1
YBR215W	HPC2	YCR077C	PAT1
YIL053W	RHR2	YNL325C	FIG4
YBR217W	ATG12	YNL325C	FIG4
YML112W	CTK3	YGL252C	RTG2
YGR105W	VMA21	YDR358W	GGA1
YEL065W	SIT1	YDL083C	RPS16B
YOR112W	CEX1	YNL041C	COG6
YHR013C	ARD1	YKR014C	YPT52
YER019C-A	SBH2	YML019W	OST6
YIL034C	CAP2	YDL155W	CLB3
YLR119W	SRN2	YMR153W	NUP53

Predictions at threshold level 0.75 continued...			
Gene1 of predicted pair		Gene2 of predicted pair	
Systematic Name	Standard Name	Systematic Name	Standard Name
YNL325C	FIG4	YNR072W	HXT17
YOR337W	TEA1	YBR019C	GAL10
YBR131W	CCZ1	YBL101C	ECM21
YPL037C	EGD1	YLR398C	SKI2
YNL032W	SIW14	YMR273C	ZDS1
YKR001C	VPS1	YML001W	YPT7
YJL183W	MNN11	YNL076W	MKS1
YOL051W	GAL11	YDR363W-A	SEM1
YNL243W	SLA2	YKL135C	APL2
YOR290C	SNF2	YGL212W	VAM7
YDR448W	ADA2	YPL042C	SSN3
YLR301W	YLR301W	YPL240C	HSP82
YJL176C	SWI3	YPL079W	RPL21B
YGL087C	MMS2	YHL002W	HSE1
YPL253C	VIK1	YGL115W	SNF4
YMR116C	ASC1	YKL032C	IXR1
YOR069W	VPS5	YPL037C	EGD1
YMR095C	SNO1	YOR035C	SHE4
YML001W	YPT7	YJL095W	BCK1
YLR371W	ROM2	YPL195W	APL5
YPR051W	MAK3	YHR060W	VMA22
YLR192C	HCR1	YKL116C	PRR1
YER161C	SPT2	YOR038C	HIR2
YHR206W	SKN7	YJR074W	MOG1
YEL065W	SIT1	YDR477W	SNF1
YDR155C	CPR1	YNL107W	YAF9
YDR171W	HSP42	YJR005W	APL1
YJL154C	VPS35	YHR134W	WSS1
YDL223C	HBT1	YNL076W	MKS1

Predictions at threshold level 0.75 continued...			
Gene1 of predicted pair		Gene2 of predicted pair	
Systematic Name	Standard Name	Systematic Name	Standard Name
YKL048C	ELM1	YEL060C	PRB1
YGL147C	RPL9A	YNL058C	YNL058C
YGR105W	VMA21	YKR030W	GMH1
YOR270C	VPH1	YKL080W	VMA5
YHR161C	YAP1801	YNR049C	MSO1
YLR360W	VPS38	YKL048C	ELM1
YDL223C	HBT1	YNL079C	TPM1
YGL200C	EMP24	YKL041W	VPS24
YDR469W	SDC1	YOR308C	SNU66
YIL159W	BNR1	YNL032W	SIW14
YJL089W	SIP4	YMR280C	CAT8
YPL115C	BEM3	YLR006C	SSK1
YHR200W	RPN10	YNL116W	DMA2
YDR129C	SAC6	YDR389W	SAC7
YKL025C	PAN3	YLR381W	CTF3
YBR195C	MSI1	YGL163C	RAD54
YPL022W	RAD1	YML102W	CAC2
YJL053W	PEP8	YKR001C	VPS1
YGL200C	EMP24	YBL072C	RPS8A
YBR200W	BEM1	YDR171W	HSP42
YPR018W	RLF2	YPL024W	RMI1
YLR337C	VRP1	YHR111W	UBA4
YLR234W	TOP3	YPR051W	MAK3
YGL212W	VAM7	YAL030W	SNC1
YAL010C	MDM10	YLR254C	NDL1
YEL009C	GCN4	YBR111W-A	SUS1
YHR111W	UBA4	YGR054W	YGR054W
YHR098C	SFB3	YJR075W	HOC1
YBR200W	BEM1	YML016C	PPZ1

Predictions at threshold level 0.75 continued...			
Gene1 of predicted pair		Gene2 of predicted pair	
Systematic Name	Standard Name	Systematic Name	Standard Name
YBL051C	PIN4	YER110C	KAP123
YPR036W	VMA13	YDL083C	RPS16B
YHL027W	RIM101	YDR310C	SUM1
YGL078C	DBP3	YDR477W	SNF1
YML117W	NAB6	YBR131W	CCZ1
YLR288C	MEC3	YDR076W	RAD55
YDR440W	DOT1	YNL030W	HHF2
YPR030W	CSR2	YJL183W	MNN11
YNL309W	STB1	YLR452C	SST2
YNL291C	MID1	YGL100W	SEH1
YPL018W	CTF19	YOL091W	SPO21
YLR234W	TOP3	YGR188C	BUB1
YPL253C	VIK1	YKL048C	ELM1
YLR441C	RPS1A	YLR452C	SST2
YOR195W	SLK19	YLR032W	RAD5
YPL001W	HAT1	YGL161C	YIP5
YER116C	SLX8	YOR327C	SNC2
YIL034C	CAP2	YBL021C	HAP3
YGR270W	YTA7	YNL107W	YAF9
YKL048C	ELM1	YLR425W	TUS1
YAL024C	LTE1	YDR137W	RGP1
YGR249W	MGA1	YJL172W	CPS1
YBR173C	UMP1	YJL095W	BCK1
YPR046W	MCM16	YGL151W	NUT1
YKL116C	PRR1	YJL172W	CPS1
YJL136C	RPS21B	YHR200W	RPN10
YOR035C	SHE4	YOR327C	SNC2
YGL253W	HXK2	YEL056W	HAT2
YKL048C	ELM1	YMR133W	REC114

Predictions at threshold level 0.75 continued...			
Gene1 of predicted pair		Gene2 of predicted pair	
Systematic Name	Standard Name	Systematic Name	Standard Name
YFR010W	UBP6	YOR327C	SNC2
YAL055W	PEX22	YKL041W	VPS24
YOR337W	TEA1	YBR280C	SAF1
YLR192C	HCR1	YMR142C	RPL13B
YMR216C	SKY1	YGR105W	VMA21
YNL201C	PSY2	YCR086W	CSM1
YNL307C	MCK1	YER122C	GLO3
YNL307C	MCK1	YJR084W	CSN12
YCR002C	CDC10	YKL101W	HSL1
YNL206C	RTT106	YML016C	PPZ1
YER107C	GLE2	YOL012C	HTZ1
YDR320C	SWA2	YEL042W	GDA1
YNL201C	PSY2	YDL116W	NUP84
YLR386W	VAC14	YOL081W	IRA2
YJR032W	CPR7	YPR046W	MCM16
YGL212W	VAM7	YGL115W	SNF4
YLR094C	GIS3	YMR230W	RPS10B
YOR066W	MSA1	YJR032W	CPR7
YBR200W	BEM1	YNR072W	HXT17
YDR137W	RGP1	YLR315W	NKP2
YLR090W	XDJ1	YLR119W	SRN2
YLR337C	VRP1	YPR046W	MCM16
YOL147C	PEX11	YDL065C	PEX19
YDR484W	VPS52	YLR180W	SAM1
YKL025C	PAN3	YNL166C	BNI5
YBR171W	SEC66	YPR120C	CLB5
YMR016C	SOK2	YBR171W	SEC66
YPL024W	RMI1	YML058W	SML1
YKL025C	PAN3	YPL253C	VIK1

Predictions at threshold level 0.75 continued...			
Gene1 of predicted pair		Gene2 of predicted pair	
Systematic Name	Standard Name	Systematic Name	Standard Name
YLL022C	HIF1	YBR009C	HHF1
YEL065W	SIT1	YIL053W	RHR2
YPL161C	BEM4	YNL090W	RHO2
YGL161C	YIP5	YDR289C	RTT103
YJL036W	SNX4	YHR012W	VPS29
YJL006C	CTK2	YGL252C	RTG2
YNL297C	MON2	YNL051W	COG5
YHR134W	WSS1	YBL079W	NUP170
YBL078C	ATG8	YLR396C	VPS33
YDR320C	SWA2	YOR127W	RGA1
YOR132W	VPS17	YDR207C	UME6
YMR179W	SPT21	YLR410W	VIP1
YDL185W	TFP1	YJR033C	RAV1
YPR018W	RLF2	YDL013W	SLX5
YNL291C	MID1	YNL041C	COG6
YHL031C	GOS1	YHR060W	VMA22
YOR358W	HAP5	YJL095W	BCK1
YOR001W	RRP6	YJR135C	MCM22
YIL036W	CST6	YAR014C	BUD14
YMR055C	BUB2	YER161C	SPT2
YMR224C	MRE11	YKL110C	KTI12
YDR245W	MNN10	YBR041W	FAT1
YDR443C	SSN2	YBR173C	UMP1
YDR192C	NUP42	YGL163C	RAD54
YLR370C	ARC18	YNL107W	YAF9
YIL156W	UBP7	YKL117W	SBA1
YCL067C	HMLALPHA2	YCR084C	TUP1
YOR195W	SLK19	YBL047C	EDE1
YFR036W	CDC26	YKL041W	VPS24

Predictions at threshold level 0.75 continued...			
Gene1 of predicted pair		Gene2 of predicted pair	
Systematic Name	Standard Name	Systematic Name	Standard Name
YER107C	GLE2	YKR082W	NUP133
YML094W	GIM5	YHR056C	RSC30
YJL183W	MNN11	YEL036C	ANP1
YOL044W	PEX15	YGR077C	PEX8
YMR016C	SOK2	YBR200W	BEM1
YER049W	TPA1	YOR014W	RTS1
YGL115W	SNF4	YPL195W	APL5
YMR142C	RPL13B	YOL121C	RPS19A
YMR273C	ZDS1	YCR053W	THR4
YDR137W	RGP1	YOR308C	SNU66
YER001W	MNN1	YLR370C	ARC18
YLL026W	HSP104	YLR357W	RSC2
YMR198W	CIK1	YKR020W	VPS51
YNL032W	SIW14	YNL087W	TCB2
YNL087W	TCB2	YDR207C	UME6
YGL223C	COG1	YJR040W	GEF1
YPL129W	TAF14	YBL003C	HTA2
YBR020W	GAL1	YGR135W	PRE9
YPR051W	MAK3	YMR055C	BUB2
YMR116C	ASC1	YDR245W	MNN10
YPL057C	SUR1	YOL111C	MDY2
YER144C	UBP5	YJL095W	BCK1
YEL053C	MAK10	YOL018C	TLG2
YBL025W	RRN10	YDR310C	SUM1
YNL215W	IES2	YGL200C	EMP24
YNL230C	ELA1	YCL014W	BUD3
YNL206C	RTT106	YDR363W-A	SEM1
YJR032W	CPR7	YJL183W	MNN11
YCR065W	HCM1	YDL047W	SIT4

Predictions at threshold level 0.75 continued...			
Gene1 of predicted pair		Gene2 of predicted pair	
Systematic Name	Standard Name	Systematic Name	Standard Name
YLR292C	SEC72	YMR109W	MYO5
YKL116C	PRR1	YMR133W	REC114
YJL053W	PEP8	YPL161C	BEM4
YLR095C	IOC2	YBL103C	RTG3
YGL003C	CDH1	YOR327C	SNC2
YML016C	PPZ1	YNL079C	TPM1
YKR057W	RPS21A	YNL229C	URE2
YBR171W	SEC66	YDR183W	PLP1
YLR441C	RPS1A	YHR060W	VMA22
YPL057C	SUR1	YHR115C	DMA1
YJL172W	CPS1	YPL137C	GIP3
YOR001W	RRP6	YMR038C	CCS1
YLR288C	MEC3	YDR207C	UME6
YDR335W	MSN5	YBL079W	NUP170
YMR224C	MRE11	YJL187C	SWE1
YOR332W	VMA4	YJL095W	BCK1
YPR018W	RLF2	YHR010W	RPL27A
YDR480W	DIG2	YBL016W	FUS3
YIL053W	RHR2	YPL137C	GIP3
YBR200W	BEM1	YJR060W	CBF1
YLR148W	PEP3	YPL195W	APL5
YDR225W	HTA1	YNL206C	RTT106
YMR224C	MRE11	YNL058C	YNL058C
YDR103W	STE5	YDR254W	CHL4
YBR127C	VMA2	YEL051W	VMA8
YKR035W-A	DID2	YPL137C	GIP3
YPL161C	BEM4	YPL256C	CLN2
YGR020C	VMA7	YEL043W	YEL043W
YML012W	ERV25	YML067C	ERV41

Predictions at threshold level 0.75 continued...			
Gene1 of predicted pair		Gene2 of predicted pair	
Systematic Name	Standard Name	Systematic Name	Standard Name
YLR093C	NYV1	YOR035C	SHE4
YAL010C	MDM10	YOL054W	PSH1
YMR109W	MYO5	YKL204W	EAP1
YGL212W	VAM7	YLR370C	ARC18
YGR270W	YTA7	YJR032W	CPR7
YDR484W	VPS52	YLR093C	NYV1
YOR304W	ISW2	YJR094C	IME1
YOR127W	RGA1	YJL136C	RPS21B
YKL139W	CTK1	YHR013C	ARD1
YEL009C	GCN4	YGR275W	RTT102
YPR184W	GDB1	YPR119W	CLB2
YNL206C	RTT106	YBR200W	BEM1
YCR084C	TUP1	YPR068C	HOS1
YNL288W	CAF40	YCR086W	CSM1
YKL007W	CAP1	YKR048C	NAP1
YLR370C	ARC18	YHL002W	HSE1
YLR386W	VAC14	YMR016C	SOK2
YBL102W	SFT2	YIL076W	SEC28
YGR104C	SRB5	YEL013W	VAC8
YGL212W	VAM7	YEL013W	VAC8
YNR072W	HXT17	YHR060W	VMA22
YCR086W	CSM1	YAL002W	VPS8
YNL107W	YAF9	YPR032W	SRO7
YGL025C	PGD1	YLR452C	SST2
YDR320C	SWA2	YNR032W	PPG1
YBR289W	SNF5	YLR357W	RSC2
YOR270C	VPH1	YLR093C	NYV1
YDR507C	GIN4	YGL163C	RAD54
YFL023W	BUD27	YPL057C	SUR1

Predictions at threshold level 0.75 continued...			
Gene1 of predicted pair		Gene2 of predicted pair	
Systematic Name	Standard Name	Systematic Name	Standard Name
YFR010W	UBP6	YHR077C	NMD2
YGL100W	SEH1	YKL113C	RAD27
YOR078W	BUD21	YBR048W	RPS11B
YDL155W	CLB3	YML028W	TSA1
YNL243W	SLA2	YLR262C	YPT6
YNR052C	POP2	YLR381W	CTF3
YBL102W	SFT2	YML001W	YPT7
YDL161W	ENT1	YLR396C	VPS33
YOR038C	HIR2	YDR440W	DOT1
YDL013W	SLX5	YGL163C	RAD54
YEL053C	MAK10	YGL115W	SNF4
YBL103C	RTG3	YER044C	ERG28
YOR275C	RIM20	YHL027W	RIM101
YGL025C	PGD1	YLR180W	SAM1
YBR173C	UMP1	YEL060C	PRB1
YCL001W	RER1	YMR054W	STV1
YPL253C	VIK1	YEL036C	ANP1
YER144C	UBP5	YMR152W	YIM1
YNL325C	FIG4	YOR035C	SHE4
YLR386W	VAC14	YMR152W	YIM1
YCR066W	RAD18	YCL001W	RER1
YEL050C	RML2	YOR080W	DIA2
YER116C	SLX8	YFL023W	BUD27
YDL116W	NUP84	YFR030W	MET10
YLR083C	EMP70	YDL077C	VAM6
YIL076W	SEC28	YOR038C	HIR2
YMR172W	HOT1	YAR014C	BUD14
YCR086W	CSM1	YGL124C	MON1
YOR026W	BUB3	YNL243W	SLA2

Predictions at threshold level 0.75 continued...			
Gene1 of predicted pair		Gene2 of predicted pair	
Systematic Name	Standard Name	Systematic Name	Standard Name
YDL080C	THI3	YHR200W	RPN10
YLR313C	SPH1	YMR038C	CCS1
YNL243W	SLA2	YKR030W	GMH1
YKL139W	CTK1	YNR052C	POP2
YOR358W	HAP5	YMR273C	ZDS1
YJL095W	BCK1	YBR082C	UBC4
YJR056C	YJR056C	YPL195W	APL5
YGR054W	YGR054W	YEL053C	MAK10
YML001W	YPT7	YLR309C	IMH1
YCR020C-A	MAK31	YPR032W	SRO7
YML016C	PPZ1	YDR144C	MKC7
YJR032W	CPR7	YAL002W	VPS8
YNL325C	FIG4	YLR398C	SKI2
YBR108W	YBR108W	YGL115W	SNF4
YLR292C	SEC72	YJL190C	RPS22A
YBR274W	CHK1	YOR038C	HIR2
YCR084C	TUP1	YBR010W	HHT1
YDR244W	PEX5	YDR142C	PEX7
YDR137W	RGP1	YOR035C	SHE4
YOR290C	SNF2	YDL223C	HBT1
YLR360W	VPS38	YHR039C-A	VMA10
YDR137W	RGP1	YLR094C	GIS3
YJL006C	CTK2	YGR135W	PRE9
YMR026C	PEX12	YCR053W	THR4
YDR108W	GSG1	YGL174W	BUD13
YIR005W	IST3	YJR050W	ISY1
YOR132W	VPS17	YGR105W	VMA21
YDR207C	UME6	YAL002W	VPS8
YEL050C	RML2	YJR060W	CBF1

Predictions at threshold level 0.75 continued...			
Gene1 of predicted pair		Gene2 of predicted pair	
Systematic Name	Standard Name	Systematic Name	Standard Name
YKR035W-A	DID2	YMR230W	RPS10B
YFR010W	UBP6	YKL101W	HSL1
YER144C	UBP5	YFL023W	BUD27
YGL033W	HOP2	YBR200W	BEM1
YKR099W	BAS1	YNR072W	HXT17
YOR231W	MKK1	YLR313C	SPH1
YJR032W	CPR7	YLR350W	ORM2
YPR030W	CSR2	YPL195W	APL5
YMR016C	SOK2	YEL060C	PRB1
YOR358W	HAP5	YML057W	CMP2
YLR399C	BDF1	YNL225C	CNM67
YBR215W	HPC2	YDL226C	GCS1
YNL291C	MID1	YER162C	RAD4
YOR304W	ISW2	YLR182W	SWI6
YGL163C	RAD54	YNL225C	CNM67
YPL115C	BEM3	YMR055C	BUB2
YOR312C	RPL20B	YDR440W	DOT1
YBR173C	UMP1	YNL076W	MKS1
YKL116C	PRR1	YOR035C	SHE4
YLR360W	VPS38	YHR013C	ARD1
YBR107C	IML3	YHR200W	RPN10
YLR337C	VRP1	YML115C	VAN1
YBR173C	UMP1	YOL081W	IRA2
YOR039W	CKB2	YDL006W	PTC1
YDR144C	MKC7	YGL253W	HXK2
YEL053C	MAK10	YDL083C	RPS16B
YLR452C	SST2	YPR119W	CLB2
YOR109W	INP53	YML071C	COG8
YDL065C	PEX19	YPL112C	PEX25

Predictions at threshold level 0.75 continued...			
Gene1 of predicted pair		Gene2 of predicted pair	
Systematic Name	Standard Name	Systematic Name	Standard Name
YBR200W	BEM1	YNL058C	YNL058C
YOR127W	RGA1	YGL115W	SNF4
YLR148W	PEP3	YDL077C	VAM6
YNL291C	MID1	YKL101W	HSL1
YOR337W	TEA1	YFL023W	BUD27
YER116C	SLX8	YPL256C	CLN2
YAL024C	LTE1	YBR107C	IML3
YGL200C	EMP24	YLR398C	SKI2
YCL032W	STE50	YLR362W	STE11
YDL006W	PTC1	YPL195W	APL5
YPR131C	NAT3	YDR254W	CHL4
YAL024C	LTE1	YNL032W	SIW14
YLR090W	XDJ1	YER122C	GLO3
YBL025W	RRN10	YEL060C	PRB1
YJL006C	CTK2	YNL225C	CNM67
YPR052C	NHP6A	YGL178W	MPT5
YDR101C	ARX1	YER002W	NOP16
YJL176C	SWI3	YBR289W	SNF5
YER144C	UBP5	YLR452C	SST2
YOR337W	TEA1	YKL139W	CTK1
YDR261C	EXG2	YDR310C	SUM1
YJL122W	ALB1	YHR052W	CIC1
YLR094C	GIS3	YER162C	RAD4
YCR086W	CSM1	YJR090C	GRR1
YJL124C	LSM1	YDL226C	GCS1
YLR102C	APC9	YDR363W-A	SEM1
YLR381W	CTF3	YBR107C	IML3
YNL307C	MCK1	YER162C	RAD4
YMR153W	NUP53	YOL081W	IRA2

Predictions at threshold level 0.75 continued...			
Gene1 of predicted pair		Gene2 of predicted pair	
Systematic Name	Standard Name	Systematic Name	Standard Name
YDR137W	RGP1	YDR335W	MSN5
YBR108W	YBR108W	YPR051W	MAK3
YGR192C	TDH3	YPR119W	CLB2
YMR142C	RPL13B	YHR039C-A	VMA10
YLR192C	HCR1	YML001W	YPT7
YLR095C	IOC2	YJL183W	MNN11
YKL032C	IXR1	YKR048C	NAP1
YML016C	PPZ1	YOR023C	AHC1
YBR215W	HPC2	YDR363W-A	SEM1
YPR046W	MCM16	YER040W	GLN3
YMR198W	CIK1	YML071C	COG8
YNL307C	MCK1	YJL183W	MNN11
YPR036W	VMA13	YDR137W	RGP1
YJL128C	PBS2	YDL006W	PTC1
YMR167W	MLH1	YLR350W	ORM2
YPL253C	VIK1	YLR410W	VIP1
YDR254W	CHL4	YJR090C	GRR1
YBR020W	GAL1	YCL011C	GBP2
YOL121C	RPS19A	YGL003C	CDH1
YDR245W	MNN10	YOR038C	HIR2
YBR217W	ATG12	YMR142C	RPL13B
YDR443C	SSN2	YPL161C	BEM4
YMR224C	MRE11	YMR038C	CCS1
YGL124C	MON1	YJL190C	RPS22A
YJR032W	CPR7	YFR036W	CDC26
YGR184C	UBR1	YDR207C	UME6
YPL024W	RMI1	YGL115W	SNF4
YHR060W	VMA22	YDR358W	GGA1
YHR200W	RPN10	YJR060W	CBF1

Predictions at threshold level 0.75 continued...			
Gene1 of predicted pair		Gene2 of predicted pair	
Systematic Name	Standard Name	Systematic Name	Standard Name
YDL020C	RPN4	YNL106C	INP52
YGL200C	EMP24	YPL161C	BEM4
YFR031C-A	RPL2A	YMR004W	MVP1
YER144C	UBP5	YOL121C	RPS19A
YGR108W	CLB1	YPR046W	MCM16
YPL195W	APL5	YLR180W	SAM1
YOR083W	WHI5	YNL107W	YAF9
YLR061W	RPL22A	YKR014C	YPT52
YGR020C	VMA7	YDL185W	TFP1
YDR335W	MSN5	YOR368W	RAD17
YPL253C	VIK1	YJR090C	GRR1
YFL013C	IES1	YHR167W	THP2
YNL206C	RTT106	YPL001W	HAT1
YOR066W	MSA1	YDR207C	UME6
YLR094C	GIS3	YHR081W	LRP1
YIL076W	SEC28	YML115C	VAN1
YGR249W	MGA1	YEL053C	MAK10
YPL022W	RAD1	YCR086W	CSM1
YKL006W	RPL14A	YCR077C	PAT1
YER143W	DDI1	YOR327C	SNC2
YBR228W	SLX1	YGL175C	SAE2
YGR020C	VMA7	YHR039C-A	VMA10
YLR079W	SIC1	YOL126C	MDH2
YOR312C	RPL20B	YPR120C	CLB5
YDL116W	NUP84	YJR060W	CBF1
YNL291C	MID1	YCR086W	CSM1
YPR018W	RLF2	YGL163C	RAD54
YNL032W	SIW14	YNL076W	MKS1
YKL007W	CAP1	YDR310C	SUM1

Predictions at threshold level 0.75 continued...			
Gene1 of predicted pair		Gene2 of predicted pair	
Systematic Name	Standard Name	Systematic Name	Standard Name
YBR108W	YBR108W	YDR254W	CHL4
YNL215W	IES2	YPL129W	TAF14
YDR108W	GSG1	YEL042W	GDA1
YOL151W	GRE2	YPR119W	CLB2
YNL054W	VAC7	YLR410W	VIP1
YKR061W	KTR2	YGL252C	RTG2
YER107C	GLE2	YKL057C	NUP120
YFL026W	STE2	YNR006W	VPS27
YPL024W	RMI1	YGR071C	YGR071C
YOR101W	RAS1	YIL033C	BCY1
YBL102W	SFT2	YEL043W	YEL043W
YGL153W	PEX14	YLR370C	ARC18
YOR023C	AHC1	YJL095W	BCK1
YNL079C	TPM1	YJL183W	MNN11
YOR198C	BFR1	YPR119W	CLB2
YFR031C-A	RPL2A	YMR142C	RPL13B
YHR200W	RPN10	YDR310C	SUM1
YLR399C	BDF1	YFR030W	MET10
YLR148W	PEP3	YKR020W	VPS51
YDR448W	ADA2	YGL025C	PGD1
YNL054W	VAC7	YLR381W	CTF3
YML016C	PPZ1	YJR032W	CPR7
YGR019W	UGA1	YLR386W	VAC14
YEL050C	RML2	YPL253C	VIK1
YJL006C	CTK2	YDL226C	GCS1
YHR012W	VPS29	YNL079C	TPM1
YDL020C	RPN4	YMR231W	PEP5
YOR083W	WHI5	YBR200W	BEM1
YLR234W	TOP3	YDR076W	RAD55

Predictions at threshold level 0.75 continued...			
Gene1 of predicted pair		Gene2 of predicted pair	
Systematic Name	Standard Name	Systematic Name	Standard Name
YBR173C	UMP1	YER144C	UBP5
YBR030W	YBR030W	YBL103C	RTG3
YHR084W	STE12	YLR441C	RPS1A
YDR156W	RPA14	YML115C	VAN1
YGL078C	DBP3	YLR370C	ARC18
YFL013C	IES1	YKL160W	ELF1
YLR386W	VAC14	YDR507C	GIN4
YBR258C	SHG1	YGL003C	CDH1
YOR127W	RGA1	YCR086W	CSM1
YMR224C	MRE11	YML102W	CAC2
YGR252W	GCN5	YNL107W	YAF9
YKR035W-A	DID2	YMR214W	SCJ1
YLR386W	VAC14	YPL057C	SUR1
YGL087C	MMS2	YBR107C	IML3
YNL215W	IES2	YER118C	SHO1
YOR109W	INP53	YGL124C	MON1
YHR013C	ARD1	YGL252C	RTG2
YDL020C	RPN4	YKR030W	GMH1
YDR254W	CHL4	YML112W	CTK3
YEL065W	SIT1	YGL066W	SGF73
YNL068C	FKH2	YOR195W	SLK19
YBR200W	BEM1	YDL077C	VAM6
YOR195W	SLK19	YMR179W	SPT21
YOR368W	RAD17	YLR350W	ORM2
YOL051W	GAL11	YNL031C	HHT2
YKL126W	YPK1	YLR370C	ARC18
YNL325C	FIG4	YLR320W	MMS22
YAL024C	LTE1	YFR031C-A	RPL2A
YPR046W	MCM16	YHL002W	HSE1

Predictions at threshold level 0.75 continued...			
Gene1 of predicted pair		Gene2 of predicted pair	
Systematic Name	Standard Name	Systematic Name	Standard Name
YKL048C	ELM1	YIL008W	URM1
YBL021C	HAP3	YMR055C	BUB2
YOR275C	RIM20	YHR108W	GGA2
YKL139W	CTK1	YNL058C	YNL058C
YHL031C	GOS1	YGR270W	YTA7
YDL020C	RPN4	YHR010W	RPL27A
YLR119W	SRN2	YGL115W	SNF4
YOR156C	NFI1	YNL297C	MON2
YCR088W	ABP1	YKL204W	EAP1
YCR065W	HCM1	YBR036C	CSG2
YIL076W	SEC28	YEL043W	YEL043W
YPR120C	CLB5	YIL103W	DPH1
YPL129W	TAF14	YDR073W	SNF11
YBR030W	YBR030W	YMR230W	RPS10B
YDL155W	CLB3	YOL081W	IRA2
YNL041C	COG6	YOR035C	SHE4
YJL058C	BIT61	YPR119W	CLB2
YMR152W	YIM1	YGL124C	MON1
YAL014C	SYN8	YDR080W	VPS41
YPR046W	MCM16	YGL003C	CDH1
YPL022W	RAD1	YOL030W	GAS5
YNL215W	IES2	YDR363W-A	SEM1
YJR040W	GEF1	YJR033C	RAV1
YGL163C	RAD54	YJR135C	MCM22
YLR109W	AHP1	YIL008W	URM1
YDR439W	LRS4	YLR182W	SWI6
YGR135W	PRE9	YPL089C	RLM1
YOR023C	AHC1	YDL155W	CLB3
YBL017C	PEP1	YJL029C	VPS53

Predictions at threshold level 0.75 continued...			
Gene1 of predicted pair		Gene2 of predicted pair	
Systematic Name	Standard Name	Systematic Name	Standard Name
YLR435W	TSR2	YJL138C	TIF2
YNL206C	RTT106	YGR188C	BUB1
YDR293C	SSD1	YKL048C	ELM1
YER044C	ERG28	YBR036C	CSG2
YBR108W	YBR108W	YDL223C	HBT1
YLR079W	SIC1	YLR131C	ACE2
YJL172W	CPS1	YPR036W	VMA13
YGL147C	RPL9A	YPL137C	GIP3
YPR018W	RLF2	YBR010W	HHT1
YKL006W	RPL14A	YGL200C	EMP24
YPL140C	MKK2	YPR119W	CLB2
YOR195W	SLK19	YPR046W	MCM16
YMR172W	HOT1	YPR051W	MAK3
YBR030W	YBR030W	YLR371W	ROM2
YGL115W	SNF4	YJR032W	CPR7
YHR178W	STB5	YLR447C	VMA6
YNL201C	PSY2	YDR289C	RTT103
YLR079W	SIC1	YOR080W	DIA2
YNL307C	MCK1	YER040W	GLN3
YDL116W	NUP84	YKR024C	DBP7
YPR040W	TIP41	YDR309C	GIC2
YJL154C	VPS35	YCR066W	RAD18
YGR239C	PEX21	YOL044W	PEX15
YFL023W	BUD27	YMR311C	GLC8
YGR209C	TRX2	YDR423C	CAD1
YOR132W	VPS17	YGR188C	BUB1
YJR056C	YJR056C	YDL083C	RPS16B
YPL115C	BEM3	YDR289C	RTT103
YEL053C	MAK10	YPL120W	VPS30

Predictions at threshold level 0.75 continued...			
Gene1 of predicted pair		Gene2 of predicted pair	
Systematic Name	Standard Name	Systematic Name	Standard Name
YDR318W	MCM21	YOL067C	RTG1
YEL065W	SIT1	YLR192C	HCR1
YPR052C	NHP6A	YIL038C	NOT3
YNL288W	CAF40	YPR072W	NOT5
YML103C	NUP188	YML057W	CMP2
YNL025C	SSN8	YGR104C	SRB5
YNL218W	MGS1	YNR052C	POP2
YLL039C	UBI4	YKR020W	VPS51
YPR084W	YPR084W	YML071C	COG8
YGR105W	VMA21	YOR327C	SNC2
YAR014C	BUD14	YGR188C	BUB1
YPR036W	VMA13	YOL111C	MDY2
YDL116W	NUP84	YMR199W	CLN1
YDR260C	SWM1	YMR038C	CCS1
YGL212W	VAM7	YKR035W-A	DID2
YPL018W	CTF19	YJL183W	MNN11
YBR030W	YBR030W	YLR093C	NYV1
YMR199W	CLN1	YHR152W	SPO12
YLR288C	MEC3	YDL013W	SLX5
YOL111C	MDY2	YMR311C	GLC8
YBL058W	SHP1	YDR207C	UME6
YFR031C-A	RPL2A	YJR032W	CPR7
YNR052C	POP2	YGR134W	CAF130
YGL060W	YBP2	YGL115W	SNF4
YGL033W	HOP2	YNL076W	MKS1
YLL010C	PSR1	YOR043W	WHI2
YDR289C	RTT103	YHR115C	DMA1
YKL116C	PRR1	YNL307C	MCK1
YOR358W	HAP5	YCR086W	CSM1

Predictions at threshold level 0.75 continued...			
Gene1 of predicted pair		Gene2 of predicted pair	
Systematic Name	Standard Name	Systematic Name	Standard Name
YLR337C	VRP1	YJL176C	SWI3
YDR137W	RGP1	YIR005W	IST3
YDR245W	MNN10	YER049W	TPA1
YHR060W	VMA22	YMR214W	SCJ1
YJR040W	GEF1	YHR039C-A	VMA10
YGL212W	VAM7	YDR310C	SUM1
YBR030W	YBR030W	YNL041C	COG6
YBR019C	GAL10	YDR207C	UME6
YHR039C-A	VMA10	YJL004C	SYS1
YML115C	VAN1	YMR142C	RPL13B
YBR171W	SEC66	YER122C	GLO3
YDR264C	AKR1	YHR115C	DMA1
YMR224C	MRE11	YCR066W	RAD18
YDR146C	SWI5	YLR371W	ROM2
YLR441C	RPS1A	YER162C	RAD4
YNL032W	SIW14	YEL053C	MAK10
YML071C	COG8	YGL003C	CDH1
YPL213W	LEA1	YGR135W	PRE9
YBR194W	SOY1	YLR398C	SKI2
YNR031C	SSK2	YCR073C	SSK22
YIL076W	SEC28	YOR124C	UBP2
YNL054W	VAC7	YGL163C	RAD54
YMR202W	ERG2	YDL226C	GCS1
YDR202C	RAV2	YGR105W	VMA21
YPL057C	SUR1	YOR035C	SHE4
YDR073W	SNF11	YKL109W	HAP4
YDR378C	LSM6	YNL225C	CNM67
YLR234W	TOP3	YNL135C	FPR1
YBL051C	PIN4	YPR046W	MCM16

Predictions at threshold level 0.75 continued...			
Gene1 of predicted pair		Gene2 of predicted pair	
Systematic Name	Standard Name	Systematic Name	Standard Name
YHL031C	GOS1	YMR216C	SKY1
YDR171W	HSP42	YJR060W	CBF1
YLR119W	SRN2	YJR056C	YJR056C
YNL291C	MID1	YLR309C	IMH1
YLR234W	TOP3	YLR313C	SPH1
YFR010W	UBP6	YPR119W	CLB2
YKR057W	RPS21A	YDL047W	SIT4
YBL072C	RPS8A	YEL036C	ANP1
YBR126C	TPS1	YMR109W	MYO5
YER162C	RAD4	YAL002W	VPS8
YKL025C	PAN3	YJL095W	BCK1
YGR071C	YGR071C	YPL079W	RPL21B
YMR004W	MVP1	YAR014C	BUD14
YER144C	UBP5	YJR056C	YJR056C
YMR273C	ZDS1	YHL011C	PRS3
YOR337W	TEA1	YML071C	COG8
YLR061W	RPL22A	YML115C	VAN1
YMR231W	PEP5	YPL256C	CLN2
YNL291C	MID1	YKR001C	VPS1
YML016C	PPZ1	YGL237C	HAP2
YGL087C	MMS2	YJR090C	GRR1
YPL253C	VIK1	YIR005W	IST3
YNL243W	SLA2	YOR069W	VPS5
YMR055C	BUB2	YEL053C	MAK10
YOR109W	INP53	YOL108C	INO4
YKL126W	YPK1	YOR327C	SNC2
YHR134W	WSS1	YIL008W	URM1
YML103C	NUP188	YJL124C	LSM1
YGL223C	COG1	YCR066W	RAD18

Predictions at threshold level 0.75 continued...			
Gene1 of predicted pair		Gene2 of predicted pair	
Systematic Name	Standard Name	Systematic Name	Standard Name
YNL243W	SLA2	YPR046W	MCM16
YCR034W	FEN1	YHR195W	NVJ1
YNL297C	MON2	YOR368W	RAD17
YPL024W	RMI1	YBR195C	MSI1
YML117W	NAB6	YDR245W	MNN10
YGL174W	BUD13	YIR005W	IST3
YDR484W	VPS52	YJR056C	YJR056C
YFR010W	UBP6	YML001W	YPT7
YJR060W	CBF1	YJR005W	APL1
YDR484W	VPS52	YPR030W	CSR2
YPL045W	VPS16	YDR323C	PEP7
YLL039C	UBI4	YEL036C	ANP1
YMR205C	PFK2	YBR082C	UBC4
YMR039C	SUB1	YCR086W	CSM1
YDR439W	LRS4	YBR107C	IML3
YNL236W	SIN4	YNL079C	TPM1
YAL024C	LTE1	YER040W	GLN3
YKL139W	CTK1	YMR153W	NUP53
YKL025C	PAN3	YLR447C	VMA6
YGL078C	DBP3	YML001W	YPT7
YFR031C-A	RPL2A	YMR216C	SKY1
YKR001C	VPS1	YNR032W	PPG1
YBR057C	MUM2	YHR200W	RPN10
YER110C	KAP123	YDR144C	MKC7
YAL024C	LTE1	YIL103W	DPH1
YER161C	SPT2	YOR308C	SNU66
YOR127W	RGA1	YER120W	SCS2
YMR201C	RAD14	YJR052W	RAD7
YGL078C	DBP3	YDR137W	RGP1

Predictions at threshold level 0.75 continued...			
Gene1 of predicted pair		Gene2 of predicted pair	
Systematic Name	Standard Name	Systematic Name	Standard Name
YDL160C	DHH1	YML109W	ZDS2
YNL058C	YNL058C	YLR452C	SST2
YOR337W	TEA1	YPL195W	APL5
YJL124C	LSM1	YPL018W	CTF19
YKR059W	TIF1	YCR031C	RPS14A
YPL253C	VIK1	YBR260C	RGD1
YDR363W-A	SEM1	YKL041W	VPS24
YAL024C	LTE1	YDL184C	RPL41A
YLR433C	CNA1	YBR114W	RAD16
YGR081C	SLX9	YDR076W	RAD55
YLR357W	RSC2	YOR064C	YNG1
YMR109W	MYO5	YOR035C	SHE4
YPL161C	BEM4	YGR135W	PRE9
YPL161C	BEM4	YHR114W	BZZ1
YER044C	ERG28	YNL041C	COG6
YLR095C	IOC2	YDL077C	VAM6
YLR398C	SKI2	YBR036C	CSG2
YPL057C	SUR1	YLR180W	SAM1
YOR080W	DIA2	YDR385W	EFT2
YKL117W	SBA1	YPL256C	CLN2
YDR443C	SSN2	YFL023W	BUD27
YMR133W	REC114	YDR207C	UME6
YDL020C	RPN4	YLR447C	VMA6
YIL034C	CAP2	YNL107W	YAF9
YCL001W	RER1	YAR014C	BUD14
YJL172W	CPS1	YPL057C	SUR1
YBR057C	MUM2	YMR089C	YTA12
YHR060W	VMA22	YER040W	GLN3
YGL178W	MPT5	YOL051W	GAL11

Predictions at threshold level 0.75 continued...			
Gene1 of predicted pair		Gene2 of predicted pair	
Systematic Name	Standard Name	Systematic Name	Standard Name
YLR303W	MET17	YLR377C	FBP1
YDL083C	RPS16B	YLR371W	ROM2
YKR099W	BAS1	YHR200W	RPN10
YPR029C	APL4	YPR119W	CLB2
YPL129W	TAF14	YKL129C	MYO3
YLR234W	TOP3	YNL107W	YAF9
YML038C	YMD8	YNL051W	COG5
YML103C	NUP188	YJR060W	CBF1
YGR108W	CLB1	YKL048C	ELM1
YBR130C	SHE3	YKL185W	ASH1
YPR036W	VMA13	YOL081W	IRA2
YGR241C	YAP1802	YOR035C	SHE4
YEL053C	MAK10	YDR310C	SUM1
YDL020C	RPN4	YKL041W	VPS24
YGL147C	RPL9A	YOR035C	SHE4
YJL154C	VPS35	YKR001C	VPS1
YGL151W	NUT1	YGL148W	ARO2
YLR148W	PEP3	YDR080W	VPS41
YDR202C	RAV2	YDL006W	PTC1
YMR142C	RPL13B	YER116C	SLX8
YLR386W	VAC14	YLR452C	SST2
YGL115W	SNF4	YOR035C	SHE4
YKL139W	CTK1	YLR398C	SKI2
YPR036W	VMA13	YER139C	YER139C
YJL210W	PEX2	YGL153W	PEX14
YFR024C-A	LSB3	YPL246C	RBD2
YKR101W	SIR1	YMR273C	ZDS1
YMR198W	CIK1	YDR200C	VPS64
YIL159W	BNR1	YBR126C	TPS1

Predictions at threshold level 0.75 continued...			
Gene1 of predicted pair		Gene2 of predicted pair	
Systematic Name	Standard Name	Systematic Name	Standard Name
YLR102C	APC9	YBR195C	MSI1
YHR156C	LIN1	YPR046W	MCM16
YER161C	SPT2	YCR086W	CSM1
YBR270C	BIT2	YJL058C	BIT61
YPR084W	YPR084W	YEL013W	VAC8
YBR228W	SLX1	YNR052C	POP2
YOR284W	HUA2	YMR253C	YMR253C
YBR030W	YBR030W	YDL184C	RPL41A
YMR095C	SNO1	YNL087W	TCB2
YDL127W	PCL2	YJL124C	LSM1
YDR320C	SWA2	YPL195W	APL5
YLR360W	VPS38	YIL008W	URM1
YBL047C	EDE1	YDR440W	DOT1
YNL087W	TCB2	YBR200W	BEM1
YJL190C	RPS22A	YAR014C	BUD14
YHR178W	STB5	YML115C	VAN1
YDR425W	SNX41	YJL036W	SNX4
YDR137W	RGP1	YLR452C	SST2
YOL111C	MDY2	YER139C	YER139C
YLR288C	MEC3	YJR084W	CSN12
YKL139W	CTK1	YNL107W	YAF9
YNL079C	TPM1	YDR207C	UME6
YGL223C	COG1	YJR060W	CBF1
YMR016C	SOK2	YDR261C	EXG2
YHL031C	GOS1	YJR032W	CPR7
YGL066W	SGF73	YDR207C	UME6
YML115C	VAN1	YHL002W	HSE1
YDR379W	RGA2	YDR264C	AKR1
YNL054W	VAC7	YOL054W	PSH1

Predictions at threshold level 0.75 continued...			
Gene1 of predicted pair		Gene2 of predicted pair	
Systematic Name	Standard Name	Systematic Name	Standard Name
YJR033C	RAV1	YER122C	GLO3
YCR077C	PAT1	YOL081W	IRA2
YHR156C	LIN1	YDR310C	SUM1
YJL124C	LSM1	YCR077C	PAT1
YGR241C	YAP1802	YJL154C	VPS35
YDR490C	PKH1	YPR119W	CLB2
YBR200W	BEM1	YBR131W	CCZ1
YGR184C	UBR1	YOR368W	RAD17
YJR135C	MCM22	YML112W	CTK3
YLR441C	RPS1A	YLR371W	ROM2
YGL060W	YBP2	YNL107W	YAF9
YPR131C	NAT3	YMR055C	BUB2
YDR448W	ADA2	YIL038C	NOT3
YDL184C	RPL41A	YBR019C	GAL10
YPL037C	EGD1	YLR320W	MMS22
YDR320C	SWA2	YGR020C	VMA7
YOL082W	ATG19	YKL103C	LAP4
YEL022W	GEA2	YER122C	GLO3
YJL089W	SIP4	YER027C	GAL83
YAL010C	MDM10	YFR030W	MET10
YNR052C	POP2	YOR368W	RAD17
YKL139W	CTK1	YDL192W	ARF1
YNL291C	MID1	YOR195W	SLK19
YMR219W	ESC1	YPR119W	CLB2
YKL139W	CTK1	YHR167W	THP2
YCR077C	PAT1	YMR038C	CCS1
YBR200W	BEM1	YBL087C	RPL23A
YOR270C	VPH1	YOL111C	MDY2
YDR448W	ADA2	YJL140W	RPB4

Predictions at threshold level 0.75 continued...			
Gene1 of predicted pair		Gene2 of predicted pair	
Systematic Name	Standard Name	Systematic Name	Standard Name
YMR172W	HOT1	YGR188C	BUB1
YPL024W	RMI1	YGL148W	ARO2
YOR191W	RIS1	YJR140C	HIR3
YBR215W	HPC2	YGR270W	YTA7
YER162C	RAD4	YGL003C	CDH1
YHL022C	SPO11	YGL003C	CDH1
YNL243W	SLA2	YOL018C	TLG2
YNL291C	MID1	YGL054C	ERV14
YDR137W	RGP1	YPL195W	APL5
YMR179W	SPT21	YDR378C	LSM6
YBL027W	RPL19B	YMR055C	BUB2
YOR112W	CEX1	YDR207C	UME6
YMR026C	PEX12	YMR016C	SOK2
YDR363W-A	SEM1	YJR135C	MCM22
YOR132W	VPS17	YGL054C	ERV14
YIL076W	SEC28	YNR072W	HXT17
YLR079W	SIC1	YLR410W	VIP1
YHR056C	RSC30	YLR200W	YKE2
YML117W	NAB6	YBR041W	FAT1
YPL161C	BEM4	YHL020C	OPI1
YLR095C	IOC2	YJR032W	CPR7
YHR134W	WSS1	YBR108W	YBR108W
YER162C	RAD4	YJL183W	MNN11
YGL133W	ITC1	YMR199W	CLN1
YGR135W	PRE9	YBR082C	UBC4
YLR360W	VPS38	YPL045W	VPS16
YMR055C	BUB2	YJL095W	BCK1
YLR337C	VRP1	YIL130W	ASG1
YGL212W	VAM7	YBL025W	RRN10

Predictions at threshold level 0.75 continued...			
Gene1 of predicted pair		Gene2 of predicted pair	
Systematic Name	Standard Name	Systematic Name	Standard Name
YHR111W	UBA4	YPL152W	RRD2
YOR195W	SLK19	YDR335W	MSN5
YKL007W	CAP1	YIR003W	YIR003W
YDR484W	VPS52	YKR020W	VPS51
YNR072W	HXT17	YMR311C	GLC8
YIL053W	RHR2	YKR035W-A	DID2
YDR378C	LSM6	YDL047W	SIT4
YNL307C	MCK1	YDR363W-A	SEM1
YMR142C	RPL13B	YML071C	COG8
YPL022W	RAD1	YHR200W	RPN10
YDR261C	EXG2	YDL192W	ARF1
YDR383C	NKP1	YCR086W	CSM1
YCR065W	HCM1	YNL107W	YAF9
YNL297C	MON2	YML028W	TSA1
YGL133W	ITC1	YKR057W	RPS21A
YFL023W	BUD27	YBR215W	HPC2
YGR261C	APL6	YER120W	SCS2
YGL115W	SNF4	YOL121C	RPS19A
YMR095C	SNO1	YLR371W	ROM2
YPL195W	APL5	YJR033C	RAV1
YJR135C	MCM22	YBR114W	RAD16
YLR119W	SRN2	YPL195W	APL5
YLL007C	YLL007C	YLR292C	SEC72
YGL250W	RMR1	YOR368W	RAD17
YJR040W	GEF1	YOL108C	INO4
YFR031C-A	RPL2A	YNL238W	KEX2
YDR202C	RAV2	YCL037C	SRO9
YHR134W	WSS1	YJL190C	RPS22A
YPL240C	HSP82	YER074W-A	YOS1

Predictions at threshold level 0.75 continued...			
Gene1 of predicted pair		Gene2 of predicted pair	
Systematic Name	Standard Name	Systematic Name	Standard Name
YKL048C	ELM1	YBR114W	RAD16
YOR106W	VAM3	YKR001C	VPS1
YBR084C-A	RPL19A	YNL116W	DMA2
YFR010W	UBP6	YGL212W	VAM7
YOR275C	RIM20	YPR173C	VPS4
YER116C	SLX8	YOL090W	MSH2
YCR031C	RPS14A	YOR368W	RAD17
YFR010W	UBP6	YKL048C	ELM1
YJL124C	LSM1	YCR086W	CSM1
YFR010W	UBP6	YAR014C	BUD14
YBR030W	YBR030W	YBR171W	SEC66
YBL058W	SHP1	YML001W	YPT7
YJR140C	HIR3	YOR039W	CKB2
YJL154C	VPS35	YGR270W	YTA7
YPL057C	SUR1	YPL018W	CTF19
YJL060W	BNA3	YLR370C	ARC18
YGL003C	CDH1	YNL076W	MKS1
YPR173C	VPS4	YOL018C	TLG2
YGR108W	CLB1	YJL190C	RPS22A
YIL076W	SEC28	YCR066W	RAD18
YNL087W	TCB2	YPL137C	GIP3
YKR057W	RPS21A	YDR379W	RGA2
YBL025W	RRN10	YDL077C	VAM6
YDR245W	MNN10	YLR320W	MMS22
YDR080W	VPS41	YER122C	GLO3
YOR132W	VPS17	YPL253C	VIK1
YNL243W	SLA2	YGR200C	ELP2
YMR133W	REC114	YNL076W	MKS1
YMR216C	SKY1	YML071C	COG8

Predictions at threshold level 0.75 continued...			
Gene1 of predicted pair		Gene2 of predicted pair	
Systematic Name	Standard Name	Systematic Name	Standard Name
YPL057C	SUR1	YLR032W	RAD5
YOR270C	VPH1	YFR036W	CDC26
YJL004C	SYS1	YGL124C	MON1
YDL116W	NUP84	YBR215W	HPC2
YLR083C	EMP70	YCL001W	RER1
YLR234W	TOP3	YJL124C	LSM1
YDR335W	MSN5	YNL116W	DMA2
YPR036W	VMA13	YOR035C	SHE4
YGR040W	KSS1	YDR480W	DIG2
YMR026C	PEX12	YGL115W	SNF4
YPR131C	NAT3	YLR234W	TOP3
YML115C	VAN1	YPL096C-A	ERI1
YFR032C-A	RPL29	YBR041W	FAT1
YLR119W	SRN2	YMR230W	RPS10B
YKL032C	IXR1	YDR289C	RTT103
YBL008W	HIR1	YPL001W	HAT1
YIR005W	IST3	YIL103W	DPH1
YPL234C	TFP3	YDR153C	ENT5
YER174C	GRX4	YFR030W	MET10
YEL044W	IES6	YEL036C	ANP1
YPL024W	RMI1	YGR249W	MGA1
YCL001W	RER1	YEL051W	VMA8
YMR092C	AIP1	YOR035C	SHE4
YHR193C	EGD2	YPL037C	EGD1
YDR245W	MNN10	YKL161C	YKL161C
YMR016C	SOK2	YDR358W	GGA1
YGR105W	VMA21	YDR207C	UME6
YPL057C	SUR1	YDR254W	CHL4
YNL206C	RTT106	YNL031C	HHT2

Predictions at threshold level 0.75 continued...			
Gene1 of predicted pair		Gene2 of predicted pair	
Systematic Name	Standard Name	Systematic Name	Standard Name
YOR270C	VPH1	YJR056C	YJR056C
YLR360W	VPS38	YPL253C	VIK1
YJR040W	GEF1	YML038C	YMD8
YDR477W	SNF1	YMR311C	GLC8
YMR198W	CIK1	YGL161C	YIP5
YEL044W	IES6	YOR312C	RPL20B
YGR241C	YAP1802	YMR231W	PEP5
YGR261C	APL6	YEL043W	YEL043W
YML115C	VAN1	YDR245W	MNN10
YJR032W	CPR7	YAR014C	BUD14
YBR200W	BEM1	YPR040W	TIP41
YHR178W	STB5	YMR109W	MYO5
YLR150W	STM1	YPL018W	CTF19
YDR244W	PEX5	YIL160C	POT1
YMR026C	PEX12	YOR290C	SNF2
YIL034C	CAP2	YLR337C	VRP1
YER102W	RPS8B	YBL072C	RPS8A
YLR248W	RCK2	YMR243C	ZRC1
YDR153C	ENT5	YKR030W	GMH1
YOL145C	CTR9	YGL003C	CDH1
YDR443C	SSN2	YPR046W	MCM16
YJL154C	VPS35	YHR205W	SCH9
YML028W	TSA1	YOR014W	RTS1
YLR095C	IOC2	YKR099W	BAS1
YFR031C-A	RPL2A	YML071C	COG8
YGL087C	MMS2	YDR254W	CHL4
YNL325C	FIG4	YPL037C	EGD1
YNL041C	COG6	YJR033C	RAV1
YLR090W	XDJ1	YMR095C	SNO1

Predictions at threshold level 0.75 continued...			
Gene1 of predicted pair		Gene2 of predicted pair	
Systematic Name	Standard Name	Systematic Name	Standard Name
YMR159C	ATG16	YPL149W	ATG5
YBL088C	TEL1	YDL059C	RAD59
YNL079C	TPM1	YMR311C	GLC8
YBR278W	DPB3	YEL056W	HAT2
YKR014C	YPT52	YAL002W	VPS8
YHR060W	VMA22	YMR152W	YIM1
YDL116W	NUP84	YDR439W	LRS4
YNL215W	IES2	YDR378C	LSM6
YDL223C	HBT1	YOL081W	IRA2
YBR107C	IML3	YFR036W	CDC26
YNL201C	PSY2	YOR195W	SLK19
YHR013C	ARD1	YPL152W	RRD2
YKL025C	PAN3	YJL190C	RPS22A
YPL161C	BEM4	YGL003C	CDH1
YKL139W	CTK1	YLR182W	SWI6
YBR030W	YBR030W	YLR094C	GIS3
YKL048C	ELM1	YJL183W	MNN11
YOR106W	VAM3	YPL256C	CLN2
YNL215W	IES2	YMR055C	BUB2
YMR055C	BUB2	YJL183W	MNN11
YJR059W	PTK2	YPL178W	CBC2
YDR439W	LRS4	YFR036W	CDC26
YKR072C	SIS2	YBL079W	NUP170
YMR016C	SOK2	YLR180W	SAM1
YDR443C	SSN2	YGR252W	GCN5
YER139C	YER139C	YML097C	VPS9
YJL154C	VPS35	YCR086W	CSM1
YCL027W	FUS1	YNL293W	MSB3
YPL024W	RMI1	YOR038C	HIR2

Predictions at threshold level 0.75 continued...			
Gene1 of predicted pair		Gene2 of predicted pair	
Systematic Name	Standard Name	Systematic Name	Standard Name
YOR127W	RGA1	YKL048C	ELM1
YOR112W	CEX1	YEL043W	YEL043W
YMR164C	MSS11	YGR059W	SPR3
YJR140C	HIR3	YBR274W	CHK1
YGL087C	MMS2	YCR086W	CSM1
YDL083C	RPS16B	YPL195W	APL5
YMR095C	SNO1	YER139C	YER139C
YPR046W	MCM16	YBR114W	RAD16
YIL159W	BNR1	YKR020W	VPS51
YLR398C	SKI2	YDR310C	SUM1
YMR224C	MRE11	YHR010W	RPL27A
YBR127C	VMA2	YAL030W	SNC1
YBL103C	RTG3	YMR133W	REC114
YMR133W	REC114	YPL137C	GIP3
YJL060W	BNA3	YJR005W	APL1
YEL065W	SIT1	YDL223C	HBT1
YKR035W-A	DID2	YGL115W	SNF4
YLR370C	ARC18	YMR214W	SCJ1
YOR115C	TRS33	YEL022W	GEA2
YJL154C	VPS35	YDR108W	GSG1
YOR127W	RGA1	YER162C	RAD4
YFR010W	UBP6	YDR310C	SUM1
YDR004W	RAD57	YOR368W	RAD17
YBR108W	YBR108W	YLR441C	RPS1A
YOR127W	RGA1	YGL031C	RPL24A
YMR250W	GAD1	YJL124C	LSM1
YDR293C	SSD1	YBR114W	RAD16
YER116C	SLX8	YER122C	GLO3
YPL240C	HSP82	YER180C-A	SLO1

Predictions at threshold level 0.75 continued...			
Gene1 of predicted pair		Gene2 of predicted pair	
Systematic Name	Standard Name	Systematic Name	Standard Name
YML103C	NUP188	YLR399C	BDF1
YNL293W	MSB3	YPL256C	CLN2
YBR171W	SEC66	YCL001W	RER1
YNL069C	RPL16B	YML038C	YMD8
YDL160C	DHH1	YMR273C	ZDS1
YPR018W	RLF2	YBR057C	MUM2
YBR217W	ATG12	YOL081W	IRA2
YJR058C	APS2	YOR036W	PEP12
YDL160C	DHH1	YDR071C	PAA1
YFR036W	CDC26	YHR115C	DMA1
YOR043W	WHI2	YDR310C	SUM1
YBR020W	GAL1	YPL248C	GAL4
YGR249W	MGA1	YHR200W	RPN10
YBR171W	SEC66	YHR108W	GGA2
YNL236W	SIN4	YBR089C-A	NHP6B
YKL048C	ELM1	YPL018W	CTF19
YBR217W	ATG12	YNL238W	KEX2
YKL139W	CTK1	YOR035C	SHE4
YOR023C	AHC1	YAL002W	VPS8
YEL051W	VMA8	YOL108C	INO4
YML001W	YPT7	YNR032W	PPG1
YDL116W	NUP84	YMR224C	MRE11
YOR337W	TEA1	YOL111C	MDY2
YBL051C	PIN4	YAR014C	BUD14
YOR032C	HMS1	YCR077C	PAT1
YPL018W	CTF19	YOL054W	PSH1
YPL256C	CLN2	YLR352W	YLR352W
YHR013C	ARD1	YNL236W	SIN4
YLR399C	BDF1	YIR005W	IST3

Predictions at threshold level 0.75 continued...			
Gene1 of predicted pair		Gene2 of predicted pair	
Systematic Name	Standard Name	Systematic Name	Standard Name
YDL184C	RPL41A	YNL238W	KEX2
YJR024C	MDE1	YCR024C-A	PMP1
YDR108W	GSG1	YER162C	RAD4
YPL050C	MNN9	YNL049C	SFB2
YKR001C	VPS1	YPL234C	TFP3
YJR063W	RPA12	YBR010W	HHT1
YGL212W	VAM7	YGL223C	COG1
YGR270W	YTA7	YNL225C	CNM67
YDR254W	CHL4	YPR120C	CLB5
YHR200W	RPN10	YJR035W	RAD26
YMR133W	REC114	YML001W	YPT7
YKL117W	SBA1	YFL047W	RGD2
YDR448W	ADA2	YBR289W	SNF5
YLR418C	CDC73	YBR089C-A	NHP6B
YPL037C	EGD1	YMR133W	REC114
YGL174W	BUD13	YLR182W	SWI6
YML013W	UBX2	YBR283C	SSH1
YLL039C	UBI4	YDL190C	UFD2
YOR127W	RGA1	YMR055C	BUB2
YBR217W	ATG12	YGL003C	CDH1
YDR183W	PLP1	YAR014C	BUD14
YIL076W	SEC28	YNL076W	MKS1
YGL147C	RPL9A	YEL060C	PRB1
YBR173C	UMP1	YCR066W	RAD18
YFR010W	UBP6	YGR184C	UBR1
YJR032W	CPR7	YDR318W	MCM21
YIL159W	BNR1	YDR358W	GGA1
YML095C	RAD10	YKL048C	ELM1
YBL103C	RTG3	YLR093C	NYV1

Predictions at threshold level 0.75 continued...			
Gene1 of predicted pair		Gene2 of predicted pair	
Systematic Name	Standard Name	Systematic Name	Standard Name
YHR134W	WSS1	YDR318W	MCM21
YOR023C	AHC1	YJR090C	GRR1
YCR066W	RAD18	YGL115W	SNF4
YLR360W	VPS38	YLR399C	BDF1
YHR200W	RPN10	YOR327C	SNC2
YOR195W	SLK19	YLR386W	VAC14
YJL154C	VPS35	YNL079C	TPM1
YDR171W	HSP42	YPR119W	CLB2
YBR057C	MUM2	YLR370C	ARC18
YBR107C	IML3	YLR370C	ARC18
YBR215W	HPC2	YPR046W	MCM16
YGL174W	BUD13	YAL002W	VPS8
YER120W	SCS2	YAL002W	VPS8
YLR320W	MMS22	YOL081W	IRA2
YPR131C	NAT3	YOR080W	DIA2
YFR024C-A	LSB3	YHR114W	BZZ1
YPR051W	MAK3	YGR054W	YGR054W
YLR135W	SLX4	YPL057C	SUR1
YDR137W	RGP1	YLR370C	ARC18
YBR217W	ATG12	YDL223C	HBT1
YBL047C	EDE1	YBR082C	UBC4
YGL163C	RAD54	YCR077C	PAT1
YJR135C	MCM22	YKL114C	APN1
YGR129W	SYF2	YPL213W	LEA1
YGL087C	MMS2	YPL018W	CTF19
YEL043W	YEL043W	YDL077C	VAM6
YDL020C	RPN4	YLR337C	VRP1
YNL218W	MGS1	YOL030W	GAS5
YLR386W	VAC14	YJL124C	LSM1

Predictions at threshold level 0.75 continued...			
Gene1 of predicted pair		Gene2 of predicted pair	
Systematic Name	Standard Name	Systematic Name	Standard Name
YDL149W	ATG9	YER162C	RAD4
YCR081W	SRB8	YPL256C	CLN2
YDR156W	RPA14	YPL152W	RRD2
YJR135C	MCM22	YLR032W	RAD5
YDL083C	RPS16B	YBR019C	GAL10
YHR161C	YAP1801	YBL106C	SRO77
YNL087W	TCB2	YBR171W	SEC66
YER116C	SLX8	YJL183W	MNN11
YDR146C	SWI5	YNL199C	GCR2
YMR109W	MYO5	YGL124C	MON1
YDR174W	HMO1	YDR318W	MCM21
YHR200W	RPN10	YPL152W	RRD2
YDR202C	RAV2	YPL234C	TFP3
YOR270C	VPH1	YBR127C	VMA2
YGL163C	RAD54	YNL107W	YAF9
YPR141C	KAR3	YIL033C	BCY1
YDR378C	LSM6	YPL152W	RRD2
YNR072W	HXT17	YNL041C	COG6
YJL176C	SWI3	YDR245W	MNN10
YLR288C	MEC3	YCR066W	RAD18
YMR004W	MVP1	YPR051W	MAK3
YJR040W	GEF1	YLR309C	IMH1
YKR020W	VPS51	YNL127W	FAR11
YDR004W	RAD57	YNL076W	MKS1
YMR246W	FAA4	YKL094W	YJU3
YCR065W	HCM1	YGL003C	CDH1
YBR200W	BEM1	YEL027W	CUP5
YNL041C	COG6	YMR152W	YIM1
YKL135C	APL2	YNR006W	VPS27

Predictions at threshold level 0.75 continued...			
Gene1 of predicted pair		Gene2 of predicted pair	
Systematic Name	Standard Name	Systematic Name	Standard Name
YIL034C	CAP2	YDR293C	SSD1
YDR225W	HTA1	YNL031C	HHT2
YOR033C	EXO1	YPR131C	NAT3
YLR079W	SIC1	YPL246C	RBD2
YKR095W	MLP1	YAL009W	SPO7
YNL238W	KEX2	YEL051W	VMA8
YHR108W	GGA2	YLR425W	TUS1
YOR069W	VPS5	YDL192W	ARF1
YML095C	RAD10	YBR107C	IML3
YGL115W	SNF4	YCR053W	THR4
YIL053W	RHR2	YLR452C	SST2
YPL042C	SSN3	YIR005W	IST3
YLR441C	RPS1A	YGL115W	SNF4
YOR332W	VMA4	YIL002C	INP51
YMR055C	BUB2	YDR171W	HSP42
YOR080W	DIA2	YLR410W	VIP1
YNL218W	MGS1	YLR410W	VIP1
YKR020W	VPS51	YPR173C	VPS4
YLL039C	UBI4	YKL006W	RPL14A
YHR061C	GIC1	YBL085W	BOI1
YLR093C	NYV1	YOL018C	TLG2
YPR131C	NAT3	YGL163C	RAD54
YDR245W	MNN10	YDL226C	GCS1
YLR083C	EMP70	YCR066W	RAD18
YKL110C	KTI12	YFR036W	CDC26
YGR261C	APL6	YPL195W	APL5
YOR127W	RGA1	YEL043W	YEL043W
YPR036W	VMA13	YEL051W	VMA8
YNL215W	IES2	YLR182W	SWI6

Predictions at threshold level 0.75 continued...			
Gene1 of predicted pair		Gene2 of predicted pair	
Systematic Name	Standard Name	Systematic Name	Standard Name
YOR270C	VPH1	YHR081W	LRP1
YGL066W	SGF73	YBR200W	BEM1
YJR090C	GRR1	YOR368W	RAD17
YER144C	UBP5	YLR320W	MMS22
YMR224C	MRE11	YDR295C	HDA2
YKL048C	ELM1	YIL103W	DPH1
YNL166C	BNI5	YJR090C	GRR1
YDR469W	SDC1	YDR318W	MCM21
YDR146C	SWI5	YEL056W	HAT2
YBR173C	UMP1	YER040W	GLN3
YDR156W	RPA14	YNL215W	IES2
YOR195W	SLK19	YDR254W	CHL4
YLR337C	VRP1	YPL018W	CTF19
YGL087C	MMS2	YLR447C	VMA6
YFR010W	UBP6	YOR043W	WHI2
YML115C	VAN1	YML067C	ERV41
YMR116C	ASC1	YLR313C	SPH1
YLR079W	SIC1	YGR059W	SPR3
YFR010W	UBP6	YPL152W	RRD2
YMR125W	STO1	YFL047W	RGD2
YDR004W	RAD57	YPL001W	HAT1
YJL176C	SWI3	YGL200C	EMP24
YLR288C	MEC3	YLR032W	RAD5
YNL199C	GCR2	YDR207C	UME6
YKR020W	VPS51	YNL238W	KEX2
YHR134W	WSS1	YLR447C	VMA6
YKR099W	BAS1	YDR207C	UME6
YHR119W	SET1	YOL145C	CTR9
YOR066W	MSA1	YPL195W	APL5

Predictions at threshold level 0.75 continued...			
Gene1 of predicted pair		Gene2 of predicted pair	
Systematic Name	Standard Name	Systematic Name	Standard Name
YDR443C	SSN2	YGR134W	CAF130
YPL106C	SSE1	YLR357W	RSC2
YDR108W	GSG1	YGL054C	ERV14
YGR108W	CLB1	YBR114W	RAD16
YJL060W	BNA3	YPR119W	CLB2
YDR071C	PAA1	YDR469W	SDC1
YLR386W	VAC14	YAR014C	BUD14
YOR275C	RIM20	YLR371W	ROM2
YPL057C	SUR1	YFR036W	CDC26
YBR171W	SEC66	YLR180W	SAM1
YBL051C	PIN4	YJL095W	BCK1
YKR001C	VPS1	YAL002W	VPS8
YOL110W	SHR5	YLR246W	ERF2
YEL065W	SIT1	YPL195W	APL5
YPL024W	RMI1	YPR046W	MCM16
YMR198W	CIK1	YDR310C	SUM1
YHR060W	VMA22	YNL238W	KEX2
YCL037C	SRO9	YDR363W-A	SEM1
YGR184C	UBR1	YDR076W	RAD55
YPR036W	VMA13	YBR171W	SEC66
YLR452C	SST2	YMR152W	YIM1
YPR051W	MAK3	YDR254W	CHL4
YPR120C	CLB5	YPL018W	CTF19
YDR156W	RPA14	YKL110C	KTI12
YPL178W	CBC2	YMR273C	ZDS1
YMR004W	MVP1	YDR153C	ENT5
YNL325C	FIG4	YEL060C	PRB1
YDR320C	SWA2	YDL137W	ARF2
YER040W	GLN3	YMR230W	RPS10B

Predictions at threshold level 0.75 continued...			
**Gene1 of predicted pair**		**Gene2 of predicted pair**	
**Systematic Name**	**Standard Name**	**Systematic Name**	**Standard Name**
YDR261C	EXG2	YJR032W	CPR7
YDR443C	SSN2	YOR106W	VAM3
YLL007C	YLL007C	YBL051C	PIN4
YJR125C	ENT3	YEL013W	VAC8
YDR318W	MCM21	YDR183W	PLP1
YOR033C	EXO1	YNR052C	POP2
YGR019W	UGA1	YOR023C	AHC1
YIL036W	CST6	YGL200C	EMP24
YER139C	YER139C	YLR182W	SWI6
YDL083C	RPS16B	YER139C	YER139C
YKR072C	SIS2	YDR207C	UME6
YIL053W	RHR2	YPL024W	RMI1
YGL163C	RAD54	YGL252C	RTG2
YLR018C	POM34	YPL246C	RBD2
YNL238W	KEX2	YPR046W	MCM16
YML115C	VAN1	YKR020W	VPS51
YMR198W	CIK1	YOR035C	SHE4
YGL174W	BUD13	YNL041C	COG6
YLR119W	SRN2	YLR386W	VAC14
YPL045W	VPS16	YPR028W	YOP1
YBR171W	SEC66	YNL293W	MSB3
YLR320W	MMS22	YLR093C	NYV1
YGL025C	PGD1	YDR358W	GGA1
YGL147C	RPL9A	YJR084W	CSN12
YOR109W	INP53	YJL004C	SYS1
YIL034C	CAP2	YJL183W	MNN11
YMR276W	DSK2	YHR108W	GGA2
YBR200W	BEM1	YNL248C	RPA49
YER096W	SHC1	YMR311C	GLC8

Predictions at threshold level 0.75 continued...			
Gene1 of predicted pair		Gene2 of predicted pair	
Systematic Name	Standard Name	Systematic Name	Standard Name
YDR363W-A	SEM1	YJL095W	BCK1
YOR043W	WHI2	YEL056W	HAT2
YMR026C	PEX12	YDR265W	PEX10
YNL079C	TPM1	YBL072C	RPS8A
YGL066W	SGF73	YNL236W	SIN4
YOR033C	EXO1	YMR106C	YKU80
YDR363W-A	SEM1	YKL213C	DOA1
YDR443C	SSN2	YER068W	MOT2
YBL003C	HTA2	YBR009C	HHF1
YER143W	DDI1	YDL047W	SIT4
YJL183W	MNN11	YJL095W	BCK1
YDR245W	MNN10	YDR378C	LSM6
YKR099W	BAS1	YER122C	GLO3
YMR127C	SAS2	YML102W	CAC2
YKL048C	ELM1	YIL038C	NOT3
YDL002C	NHP10	YPL057C	SUR1
YLR079W	SIC1	YKR001C	VPS1
YBR111W-A	SUS1	YLR055C	SPT8
YNL106C	INP52	YJL004C	SYS1
YBL047C	EDE1	YKL101W	HSL1
YLR119W	SRN2	YFR036W	CDC26
YDR383C	NKP1	YIL103W	DPH1
YDR261C	EXG2	YOR327C	SNC2
YER162C	RAD4	YOL121C	RPS19A
YEL065W	SIT1	YDL184C	RPL41A
YLR324W	PEX30	YBR168W	PEX32
YMR202W	ERG2	YMR055C	BUB2
YOL111C	MDY2	YNL076W	MKS1
YNL325C	FIG4	YNL076W	MKS1

Predictions at threshold level 0.75 continued...			
Gene1 of predicted pair		Gene2 of predicted pair	
Systematic Name	Standard Name	Systematic Name	Standard Name
YOR080W	DIA2	YDR358W	GGA1
YDL184C	RPL41A	YCR053W	THR4
YFR031C-A	RPL2A	YOR290C	SNF2
YJR099W	YUH1	YNR006W	VPS27
YPL259C	APM1	YAL030W	SNC1
YHR134W	WSS1	YDR289C	RTT103
YLR083C	EMP70	YJL036W	SNX4
YPL086C	ELP3	YBR089C-A	NHP6B
YMR016C	SOK2	YML071C	COG8
YPL253C	VIK1	YDR207C	UME6
YPL115C	BEM3	YGL151W	NUT1
YJL053W	PEP8	YBR127C	VMA2
YMR153W	NUP53	YLR452C	SST2
YMR116C	ASC1	YML028W	TSA1
YLR452C	SST2	YLR182W	SWI6
YNL106C	INP52	YKL139W	CTK1
YBR171W	SEC66	YLR452C	SST2
YLR135W	SLX4	YJL124C	LSM1
YLL022C	HIF1	YEL056W	HAT2
YML028W	TSA1	YGL031C	RPL24A
YML016C	PPZ1	YGL019W	CKB1
YGR270W	YTA7	YML112W	CTK3
YNL288W	CAF40	YGL124C	MON1
YHR152W	SPO12	YGR135W	PRE9
YGR104C	SRB5	YPL161C	BEM4
YPR072W	NOT5	YPL203W	TPK2
YOR127W	RGA1	YLR447C	VMA6
YBL102W	SFT2	YOL018C	TLG2
YDL006W	PTC1	YPL259C	APM1

Predictions at threshold level 0.75 continued...			
Gene1 of predicted pair		Gene2 of predicted pair	
Systematic Name	Standard Name	Systematic Name	Standard Name
YLR102C	APC9	YBR200W	BEM1
YLR452C	SST2	YJL095W	BCK1
YOR059C	YOR059C	YKR046C	PET10
YAL055W	PEX22	YLR131C	ACE2
YLR410W	VIP1	YOL054W	PSH1
YOR008C	SLG1	YER096W	SHC1
YOR059C	YOR059C	YKL140W	TGL1
YDR293C	SSD1	YDR389W	SAC7
YJL138C	TIF2	YKL101W	HSL1
YPL042C	SSN3	YLR381W	CTF3
YIL034C	CAP2	YGR019W	UGA1
YDR217C	RAD9	YGL190C	CDC55
YOR368W	RAD17	YNL082W	PMS1
YNL054W	VAC7	YPL018W	CTF19
YJR056C	YJR056C	YKR035W-A	DID2
YLR371W	ROM2	YPL089C	RLM1
YMR312W	ELP6	YHR187W	IKI1
YFR010W	UBP6	YNL041C	COG6
YBR108W	YBR108W	YLR398C	SKI2
YMR198W	CIK1	YMR109W	MYO5
YOR312C	RPL20B	YFR036W	CDC26
YAL024C	LTE1	YBR171W	SEC66
YPR030W	CSR2	YBR280C	SAF1
YBR200W	BEM1	YKL041W	VPS24
YJL053W	PEP8	YER162C	RAD4
YDR244W	PEX5	YDL065C	PEX19
YNL199C	GCR2	YKL160W	ELF1
YLR292C	SEC72	YLR301W	YLR301W
YGL124C	MON1	YDL077C	VAM6

Predictions at threshold level 0.75 continued...			
Gene1 of predicted pair		Gene2 of predicted pair	
Systematic Name	Standard Name	Systematic Name	Standard Name
YDR202C	RAV2	YER122C	GLO3
YOL067C	RTG1	YAL002W	VPS8
YKL139W	CTK1	YER122C	GLO3
YBR200W	BEM1	YLR180W	SAM1
YJR060W	CBF1	YIL103W	DPH1
YER114C	BOI2	YNL307C	MCK1
YOR290C	SNF2	YLR410W	VIP1
YFR031C-A	RPL2A	YDL077C	VAM6
YMR039C	SUB1	YLR452C	SST2
YBL102W	SFT2	YGL115W	SNF4
YDR363W-A	SEM1	YDR440W	DOT1
YIL053W	RHR2	YHR060W	VMA22
YMR016C	SOK2	YEL053C	MAK10
YFR031C-A	RPL2A	YGR249W	MGA1
YDR293C	SSD1	YHR111W	UBA4
YNL054W	VAC7	YDR254W	CHL4
YDR385W	EFT2	YAL055W	PEX22
YPL120W	VPS30	YJR033C	RAV1
YAL024C	LTE1	YNL307C	MCK1
YBR107C	IML3	YGL124C	MON1
YMR224C	MRE11	YMR016C	SOK2
YML109W	ZDS2	YNL031C	HHT2
YOL043C	NTG2	YCR066W	RAD18
YHR060W	VMA22	YPR032W	SRO7
YIL076W	SEC28	YKL048C	ELM1
YLR386W	VAC14	YOR358W	HAP5
YJR060W	CBF1	YGL003C	CDH1
YJL004C	SYS1	YDL226C	GCS1
YNL032W	SIW14	YBR019C	GAL10

Predictions at threshold level 0.75 continued...			
Gene1 of predicted pair		Gene2 of predicted pair	
Systematic Name	Standard Name	Systematic Name	Standard Name
YBL008W	HIR1	YMR055C	BUB2
YKR072C	SIS2	YOR043W	WHI2
YGL190C	CDC55	YDR422C	SIP1
YEL065W	SIT1	YGL078C	DBP3
YBL088C	TEL1	YIL038C	NOT3
YOR275C	RIM20	YDL047W	SIT4
YOR290C	SNF2	YBR010W	HHT1
YOR023C	AHC1	YPL253C	VIK1
YML001W	YPT7	YNL041C	COG6
YMR224C	MRE11	YIL103W	DPH1
YJL036W	SNX4	YLR309C	IMH1
YLL007C	YLL007C	YEL056W	HAT2
YBL021C	HAP3	YMR109W	MYO5
YBR057C	MUM2	YBL072C	RPS8A
YBL103C	RTG3	YMR214W	SCJ1
YER144C	UBP5	YPR046W	MCM16
YOR080W	DIA2	YEL013W	VAC8
YPL161C	BEM4	YPR030W	CSR2
YHR013C	ARD1	YIL103W	DPH1
YPR052C	NHP6A	YKL160W	ELF1
YGR249W	MGA1	YJR056C	YJR056C
YAL024C	LTE1	YGL066W	SGF73
YPR036W	VMA13	YLR094C	GIS3
YPR120C	CLB5	YDL047W	SIT4
YJL124C	LSM1	YDR254W	CHL4
YCR084C	TUP1	YNL167C	SKO1
YBL079W	NUP170	YJL187C	SWE1
YHR161C	YAP1801	YDL161W	ENT1
YKL048C	ELM1	YPR046W	MCM16

Predictions at threshold level 0.75 continued...			
Gene1 of predicted pair		Gene2 of predicted pair	
Systematic Name	Standard Name	Systematic Name	Standard Name
YAL051W	OAF1	YDR310C	SUM1
YDR245W	MNN10	YLR370C	ARC18
YGR261C	APL6	YDR108W	GSG1
YOL145C	CTR9	YDR071C	PAA1
YOL111C	MDY2	YPL195W	APL5
YMR153W	NUP53	YKL126W	YPK1
YIL076W	SEC28	YDR207C	UME6
YPR046W	MCM16	YPR119W	CLB2
YMR199W	CLN1	YLR182W	SWI6
YLR386W	VAC14	YHL002W	HSE1
YBR173C	UMP1	YER162C	RAD4
YDR443C	SSN2	YDR207C	UME6
YBR200W	BEM1	YKL114C	APN1
YGR104C	SRB5	YDL005C	MED2
YKL139W	CTK1	YDL223C	HBT1
YFR010W	UBP6	YLR398C	SKI2
YLR309C	IMH1	YER122C	GLO3
YLR083C	EMP70	YNL051W	COG5
YNR072W	HXT17	YML071C	COG8
YHR012W	VPS29	YDL006W	PTC1
YOR109W	INP53	YGL115W	SNF4
YMR179W	SPT21	YLR381W	CTF3
YDL192W	ARF1	YDR207C	UME6
YHR200W	RPN10	YER139C	YER139C
YBL052C	SAS3	YBR089C-A	NHP6B
YDR448W	ADA2	YKL109W	HAP4
YOR109W	INP53	YAL030W	SNC1
YAL024C	LTE1	YHR061C	GIC1
YLR079W	SIC1	YMR153W	NUP53

Predictions at threshold level 0.75 continued...			
Gene1 of predicted pair		Gene2 of predicted pair	
Systematic Name	Standard Name	Systematic Name	Standard Name
YPL096C-A	ERI1	YMR089C	YTA12
YMR095C	SNO1	YJR032W	CPR7
YJL138C	TIF2	YCR031C	RPS14A
YKL139W	CTK1	YER139C	YER139C
YNL058C	YNL058C	YNL076W	MKS1
YDL020C	RPN4	YML016C	PPZ1
YMR284W	YKU70	YOL090W	MSH2
YGL033W	HOP2	YHR039C-A	VMA10
YDL155W	CLB3	YGR188C	BUB1
YDR448W	ADA2	YOL148C	SPT20
YDL223C	HBT1	YJR084W	CSN12
YOL051W	GAL11	YGL094C	PAN2
YGL003C	CDH1	YNL107W	YAF9
YKL025C	PAN3	YKL114C	APN1
YBL085W	BOI1	YER158C	YER158C
YJL124C	LSM1	YJL187C	SWE1
YBR200W	BEM1	YNL199C	GCR2
YHR152W	SPO12	YDR264C	AKR1
YGR252W	GCN5	YDL006W	PTC1
YPR046W	MCM16	YER139C	YER139C
YBR290W	BSD2	YLR360W	VPS38
YNR052C	POP2	YDR439W	LRS4
YOR127W	RGA1	YKL101W	HSL1
YPL096C-A	ERI1	YKL079W	SMY1
YJL029C	VPS53	YPL045W	VPS16
YIL008W	URM1	YEL056W	HAT2
YGR184C	UBR1	YLR219W	MSC3
YLR095C	IOC2	YLR441C	RPS1A
YPL057C	SUR1	YDR363W-A	SEM1

Predictions at threshold level 0.75 continued...			
Gene1 of predicted pair		Gene2 of predicted pair	
Systematic Name	Standard Name	Systematic Name	Standard Name
YOR109W	INP53	YPL259C	APM1
YDR156W	RPA14	YCR077C	PAT1
YLR006C	SSK1	YHR111W	UBA4
YMR198W	CIK1	YMR089C	YTA12
YDR245W	MNN10	YBR260C	RGD1
YBR111W-A	SUS1	YGR252W	GCN5
YAL024C	LTE1	YJR135C	MCM22
YGR135W	PRE9	YBL072C	RPS8A
YDR080W	VPS41	YOL018C	TLG2
YJL183W	MNN11	YNL107W	YAF9
YBR200W	BEM1	YPL037C	EGD1
YKL116C	PRR1	YNL087W	TCB2
YLR381W	CTF3	YPR051W	MAK3
YGL003C	CDH1	YOR035C	SHE4
YKL139W	CTK1	YNL041C	COG6
YOR290C	SNF2	YBL025W	RRN10
YFR021W	ATG18	YLR094C	GIS3
YDL127W	PCL2	YGL161C	YIP5
YDR156W	RPA14	YIL103W	DPH1
YFR031C-A	RPL2A	YOL121C	RPS19A
YKL101W	HSL1	YBL087C	RPL23A
YJL053W	PEP8	YGL115W	SNF4
YGR097W	ASK10	YPR119W	CLB2
YPR101W	SNT309	YDR163W	CWC15
YPR018W	RLF2	YDR004W	RAD57
YHR060W	VMA22	YOL018C	TLG2
YOR109W	INP53	YHR060W	VMA22
YOR140W	SFL1	YOR014W	RTS1
YER116C	SLX8	YER162C	RAD4

Predictions at threshold level 0.75 continued...			
Gene1 of predicted pair		Gene2 of predicted pair	
Systematic Name	Standard Name	Systematic Name	Standard Name
YDR439W	LRS4	YOL054W	PSH1
YDR293C	SSD1	YDR155C	CPR1
YML117W	NAB6	YPL161C	BEM4
YLR320W	MMS22	YBR036C	CSG2
YPR052C	NHP6A	YGL019W	CKB1
YFR032C-A	RPL29	YMR038C	CCS1
YOR195W	SLK19	YMR038C	CCS1
YKL214C	YRA2	YNL004W	HRB1
YPL120W	VPS30	YDR310C	SUM1
YML062C	MFT1	YMR273C	ZDS1
YJL124C	LSM1	YNL307C	MCK1
YGR270W	YTA7	YHR114W	BZZ1
YAL024C	LTE1	YGL115W	SNF4
YHR108W	GGA2	YMR038C	CCS1
YMR153W	NUP53	YPL195W	APL5
YDR142C	PEX7	YGL205W	POX1
YER044C	ERG28	YNL238W	KEX2
YLR360W	VPS38	YML001W	YPT7
YDR129C	SAC6	YHL002W	HSE1
YGL078C	DBP3	YKL139W	CTK1
YML001W	YPT7	YHR081W	LRP1
YPR046W	MCM16	YJR090C	GRR1
YIR003W	YIR003W	YMR109W	MYO5
YNL325C	FIG4	YMR230W	RPS10B
YLR410W	VIP1	YGL003C	CDH1
YFR031C-A	RPL2A	YGL003C	CDH1
YJL136C	RPS21B	YGL031C	RPL24A
YLR083C	EMP70	YBL102W	SFT2
YHR134W	WSS1	YDR207C	UME6

Predictions at threshold level 0.75 continued...			
Gene1 of predicted pair		Gene2 of predicted pair	
Systematic Name	Standard Name	Systematic Name	Standard Name
YMR231W	PEP5	YDR207C	UME6
YHR073W	OSH3	YKL117W	SBA1
YDR130C	FIN1	YPR119W	CLB2
YLR216C	CPR6	YDR318W	MCM21
YBR278W	DPB3	YHR039C-A	VMA10
YDL223C	HBT1	YGL200C	EMP24
YCL037C	SRO9	YGR270W	YTA7
YIL071C	PCI8	YKR026C	GCN3
YMR095C	SNO1	YBL103C	RTG3
YBL003C	HTA2	YKR048C	NAP1
YDR383C	NKP1	YNL107W	YAF9
YDR363W-A	SEM1	YOR368W	RAD17
YJL053W	PEP8	YMR004W	MVP1
YDR378C	LSM6	YLR182W	SWI6
YDL232W	OST4	YJR010C-A	SPC1
YBL008W	HIR1	YJL140W	RPB4
YMR089C	YTA12	YDR207C	UME6
YBR194W	SOY1	YNL107W	YAF9
YKL048C	ELM1	YMR214W	SCJ1
YJL036W	SNX4	YPL234C	TFP3
YHL002W	HSE1	YOL081W	IRA2
YNL243W	SLA2	YMR224C	MRE11
YLR240W	VPS34	YMR231W	PEP5
YMR224C	MRE11	YNL016W	PUB1
YLR090W	XDJ1	YBR126C	TPS1
YNL215W	IES2	YOR043W	WHI2
YDR469W	SDC1	YDR440W	DOT1
YLR320W	MMS22	YMR038C	CCS1
YLL007C	YLL007C	YAR014C	BUD14

Predictions at threshold level 0.75 continued...			
Gene1 of predicted pair		Gene2 of predicted pair	
Systematic Name	Standard Name	Systematic Name	Standard Name
YGL054C	ERV14	YJR033C	RAV1
YGL003C	CDH1	YEL060C	PRB1
YDR245W	MNN10	YGL163C	RAD54
YJL095W	BCK1	YHR158C	KEL1
YEL009C	GCN4	YCR084C	TUP1
YNL238W	KEX2	YER162C	RAD4
YGR108W	CLB1	YJR135C	MCM22
YPR036W	VMA13	YML071C	COG8
YPR018W	RLF2	YKL101W	HSL1
YDL155W	CLB3	YBR283C	SSH1
YDR293C	SSD1	YMR054W	STV1
YOR195W	SLK19	YBR107C	IML3
YHR156C	LIN1	YFR036W	CDC26
YGL115W	SNF4	YER122C	GLO3
YCR020W-B	HTL1	YPR120C	CLB5
YPL240C	HSP82	YML132W	COS3
YCR066W	RAD18	YJL004C	SYS1
YHR098C	SFB3	YJL168C	SET2
YMR153W	NUP53	YPL256C	CLN2
YDL116W	NUP84	YDR101C	ARX1
YIL159W	BNR1	YER139C	YER139C
YLR337C	VRP1	YBR200W	BEM1
YDR004W	RAD57	YGR081C	SLX9
YLR119W	SRN2	YER040W	GLN3
YOL051W	GAL11	YGR135W	PRE9
YDR293C	SSD1	YDR335W	MSN5
YML013W	UBX2	YMR022W	UBC7
YOR080W	DIA2	YBR215W	HPC2
YOL072W	THP1	YBR111W-A	SUS1

Predictions at threshold level 0.75 continued...			
Gene1 of predicted pair		Gene2 of predicted pair	
Systematic Name	Standard Name	Systematic Name	Standard Name
YBL047C	EDE1	YHR111W	UBA4
YLR095C	IOC2	YIL053W	RHR2
YML001W	YPT7	YER122C	GLO3
YNL049C	SFB2	YEL036C	ANP1
YPL037C	EGD1	YNL051W	COG5
YBL079W	NUP170	YDR076W	RAD55
YMR055C	BUB2	YLR182W	SWI6
YML016C	PPZ1	YEL053C	MAK10
YNL068C	FKH2	YHR152W	SPO12
YNL016W	PUB1	YBR082C	UBC4
YGL174W	BUD13	YDR289C	RTT103
YIL053W	RHR2	YHR039C-A	VMA10
YJL036W	SNX4	YLR423C	ATG17
YDR202C	RAV2	YNL297C	MON2
YHR064C	SSZ1	YGR285C	ZUO1
YLR315W	NKP2	YJL006C	CTK2
YDR383C	NKP1	YNL215W	IES2
YKL126W	YPK1	YNL058C	YNL058C
YMR172W	HOT1	YHR012W	VPS29
YOR001W	RRP6	YCR086W	CSM1
YMR224C	MRE11	YER162C	RAD4
YMR004W	MVP1	YJR040W	GEF1
YAR014C	BUD14	YNL107W	YAF9
YDR192C	NUP42	YMR273C	ZDS1
YMR198W	CIK1	YKL048C	ELM1
YNL201C	PSY2	YNL248C	RPA49
YPL024W	RMI1	YBR217W	ATG12
YLR386W	VAC14	YJR032W	CPR7
YLR079W	SIC1	YDR183W	PLP1

Predictions at threshold level 0.75 continued...			
Gene1 of predicted pair		Gene2 of predicted pair	
Systematic Name	Standard Name	Systematic Name	Standard Name
YBL103C	RTG3	YFL023W	BUD27
YLR018C	POM34	YER115C	SPR6
YMR127C	SAS2	YJL190C	RPS22A
YHL002W	HSE1	YBR114W	RAD16
YOR069W	VPS5	YAL030W	SNC1
YFR031C-A	RPL2A	YLR192C	HCR1
YLR399C	BDF1	YLR182W	SWI6
YDR320C	SWA2	YGL115W	SNF4
YFR010W	UBP6	YAL002W	VPS8
YKL032C	IXR1	YNL229C	URE2
YGL178W	MPT5	YDR388W	RVS167
YGL178W	MPT5	YHR030C	SLT2
YLR148W	PEP3	YDR323C	PEP7
YKR028W	SAP190	YDL047W	SIT4
YPL096C-A	ERI1	YCR009C	RVS161
YBR200W	BEM1	YEL053C	MAK10
YCR066W	RAD18	YPR119W	CLB2
YPL137C	GIP3	YBL025W	RRN10
YPL042C	SSN3	YJR135C	MCM22
YDL113C	ATG20	YLR191W	PEX13
YAL024C	LTE1	YPL152W	RRD2
YNL079C	TPM1	YPL256C	CLN2
YPL129W	TAF14	YMR037C	MSN2
YNL243W	SLA2	YPR029C	APL4
YPR018W	RLF2	YDL116W	NUP84
YLR234W	TOP3	YOR038C	HIR2
YGR105W	VMA21	YJL004C	SYS1
YDL192W	ARF1	YLR180W	SAM1
YDL160C	DHH1	YMR199W	CLN1

Predictions at threshold level 0.75 continued...			
Gene1 of predicted pair		Gene2 of predicted pair	
Systematic Name	Standard Name	Systematic Name	Standard Name
YHR156C	LIN1	YDL223C	HBT1
YKL010C	UFD4	YKL041W	VPS24
YBR194W	SOY1	YAL002W	VPS8
YPL024W	RMI1	YML028W	TSA1
YLR381W	CTF3	YIR005W	IST3
YJR063W	RPA12	YIL103W	DPH1
YPR046W	MCM16	YDR076W	RAD55
YLR079W	SIC1	YDR318W	MCM21
YOR069W	VPS5	YEL042W	GDA1
YDR264C	AKR1	YGL031C	RPL24A
YHR134W	WSS1	YMR284W	YKU70
YBR200W	BEM1	YKR020W	VPS51
YPR072W	NOT5	YNL030W	HHF2
YLR234W	TOP3	YPL253C	VIK1
YOR270C	VPH1	YEL053C	MAK10
YPR036W	VMA13	YEL060C	PRB1
YLR410W	VIP1	YDL226C	GCS1
YGR104C	SRB5	YBR194W	SOY1
YBL051C	PIN4	YJR090C	GRR1
YPL213W	LEA1	YDR163W	CWC15
YKR020W	VPS51	YAL002W	VPS8
YLR119W	SRN2	YLR441C	RPS1A
YPL050C	MNN9	YGR135W	PRE9
YGR108W	CLB1	YPL161C	BEM4
YDR155C	CPR1	YGR188C	BUB1
YNL076W	MKS1	YGL252C	RTG2
YDR129C	SAC6	YOR008C	SLG1
YGL223C	COG1	YKR020W	VPS51
YBL051C	PIN4	YEL053C	MAK10

Predictions at threshold level 0.75 continued...			
**Gene1 of predicted pair**		**Gene2 of predicted pair**	
**Systematic Name**	**Standard Name**	**Systematic Name**	**Standard Name**
YOR035C	SHE4	YHR114W	BZZ1
YBR173C	UMP1	YDL192W	ARF1
YFR010W	UBP6	YPR036W	VMA13
YAL009W	SPO7	YDR207C	UME6
YIL053W	RHR2	YDL223C	HBT1
YDR363W-A	SEM1	YOR038C	HIR2
YMR055C	BUB2	YHR039C-A	VMA10
YHR150W	PEX28	YBR168W	PEX32
YOR275C	RIM20	YDR264C	AKR1
YDR329C	PEX3	YGL153W	PEX14
YLR452C	SST2	YLR093C	NYV1
YDR225W	HTA1	YFR036W	CDC26
YBR200W	BEM1	YJL117W	PHO86
YLR079W	SIC1	YGL019W	CKB1
YOR132W	VPS17	YPL195W	APL5
YKL025C	PAN3	YNL288W	CAF40
YKR001C	VPS1	YEL043W	YEL043W
YHL031C	GOS1	YML071C	COG8
YOR083W	WHI5	YGR104C	SRB5
YOR001W	RRP6	YDL155W	CLB3
YBR127C	VMA2	YBL047C	EDE1
YKR001C	VPS1	YJL095W	BCK1
YLR381W	CTF3	YDR183W	PLP1
YDR448W	ADA2	YDR477W	SNF1
YCR077C	PAT1	YLR182W	SWI6
YBR194W	SOY1	YDR289C	RTT103
YNL107W	YAF9	YPR119W	CLB2
YLR192C	HCR1	YLR452C	SST2
YOR132W	VPS17	YOR036W	PEP12

Predictions at threshold level 0.75 continued...			
Gene1 of predicted pair		Gene2 of predicted pair	
Systematic Name	Standard Name	Systematic Name	Standard Name
YLR093C	NYV1	YJR125C	ENT3
YOR337W	TEA1	YKL116C	PRR1
YDR183W	PLP1	YJL095W	BCK1
YOR080W	DIA2	YJL006C	CTK2
YPL161C	BEM4	YOL108C	INO4
YKL048C	ELM1	YLR315W	NKP2
YNL307C	MCK1	YMR311C	GLC8
YJL154C	VPS35	YOL108C	INO4
YMR055C	BUB2	YDL226C	GCS1
YKR020W	VPS51	YMR022W	UBC7
YHR111W	UBA4	YKR020W	VPS51
YJR063W	RPA12	YNL030W	HHF2
YER044C	ERG28	YML001W	YPT7
YOR132W	VPS17	YOR035C	SHE4
YML001W	YPT7	YDL077C	VAM6
YMR055C	BUB2	YFR030W	MET10
YDR477W	SNF1	YER049W	TPA1
YGR054W	YGR054W	YJL190C	RPS22A
YPL115C	BEM3	YLR292C	SEC72
YJR056C	YJR056C	YER044C	ERG28
YLR083C	EMP70	YER162C	RAD4
YGR270W	YTA7	YJL095W	BCK1
YLR352W	YLR352W	YDR207C	UME6
YLR337C	VRP1	YGR188C	BUB1
YDL083C	RPS16B	YBR280C	SAF1
YHR073W	OSH3	YBL079W	NUP170
YBR200W	BEM1	YLR370C	ARC18
YGL174W	BUD13	YGR071C	YGR071C
YLR119W	SRN2	YLR192C	HCR1

Predictions at threshold level 0.75 continued...			
Gene1 of predicted pair		Gene2 of predicted pair	
Systematic Name	Standard Name	Systematic Name	Standard Name
YFR031C-A	RPL2A	YNL325C	FIG4
YMR095C	SNO1	YLR119W	SRN2
YMR016C	SOK2	YDR137W	RGP1
YPL115C	BEM3	YDR254W	CHL4
YNR058W	BIO3	YDL149W	ATG9
YCR065W	HCM1	YIR009W	MSL1
YBL078C	ATG8	YML001W	YPT7
YNR023W	SNF12	YDR073W	SNF11
YJL157C	FAR1	YGR152C	RSR1
YDR289C	RTT103	YEL013W	VAC8
YDR318W	MCM21	YMR038C	CCS1
YEL065W	SIT1	YOR035C	SHE4
YIL034C	CAP2	YDL226C	GCS1
YKR099W	BAS1	YBL025W	RRN10
YNL307C	MCK1	YLR182W	SWI6
YLR292C	SEC72	YBR114W	RAD16
YBL067C	UBP13	YDR207C	UME6
YNL054W	VAC7	YPL253C	VIK1
YLR441C	RPS1A	YLR180W	SAM1
YJL183W	MNN11	YLR180W	SAM1
YLR386W	VAC14	YPR036W	VMA13
YKL010C	UFD4	YBL072C	RPS8A
YLR399C	BDF1	YMR055C	BUB2
YNL248C	RPA49	YNL107W	YAF9
YAL024C	LTE1	YNL041C	COG6
YMR224C	MRE11	YLR370C	ARC18
YPL227C	ALG5	YMR214W	SCJ1
YLR234W	TOP3	YBR171W	SEC66
YIL159W	BNR1	YFL023W	BUD27

Predictions at threshold level 0.75 continued...			
Gene1 of predicted pair		Gene2 of predicted pair	
Systematic Name	Standard Name	Systematic Name	Standard Name
YML095C	RAD10	YNL229C	URE2
YDR174W	HMO1	YLR410W	VIP1
YPL256C	CLN2	YEL036C	ANP1
YFR031C-A	RPL2A	YBL103C	RTG3
YBL021C	HAP3	YDR155C	CPR1
YDL185W	TFP1	YEL051W	VMA8
YDR320C	SWA2	YGL054C	ERV14
YPR036W	VMA13	YGL212W	VAM7
YIL034C	CAP2	YHR178W	STB5
YKL007W	CAP1	YNL293W	MSB3
YJR032W	CPR7	YPL195W	APL5
YMR202W	ERG2	YPL018W	CTF19
YFR034C	PHO4	YDR363W-A	SEM1
YMR026C	PEX12	YJR032W	CPR7
YPL120W	VPS30	YOL018C	TLG2
YDR320C	SWA2	YJL004C	SYS1
YNL106C	INP52	YOR284W	HUA2
YDL042C	SIR2	YML109W	ZDS2
YLL007C	YLL007C	YJL095W	BCK1
YJR135C	MCM22	YOR141C	ARP8
YKL117W	SBA1	YGR059W	SPR3
YHR200W	RPN10	YPL018W	CTF19
YKL025C	PAN3	YHL002W	HSE1
YCR086W	CSM1	YAR014C	BUD14
YGL078C	DBP3	YNL076W	MKS1
YPL018W	CTF19	YNL116W	DMA2
YKL116C	PRR1	YLR320W	MMS22
YPR084W	YPR084W	YOR327C	SNC2
YDL160C	DHH1	YLR079W	SIC1

Predictions at threshold level 0.75 continued...			
Gene1 of predicted pair		Gene2 of predicted pair	
Systematic Name	Standard Name	Systematic Name	Standard Name
YGL054C	ERV14	YDL006W	PTC1
YDR097C	MSH6	YFR010W	UBP6
YDR439W	LRS4	YPR120C	CLB5
YMR133W	REC114	YOL121C	RPS19A
YML117W	NAB6	YJL183W	MNN11
YOR069W	VPS5	YDR358W	GGA1
YMR095C	SNO1	YNL307C	MCK1
YER110C	KAP123	YGL151W	NUT1
YPR036W	VMA13	YMR230W	RPS10B
YOR195W	SLK19	YKR057W	RPS21A
YBR290W	BSD2	YMR109W	MYO5
YPR120C	CLB5	YDR310C	SUM1
YGL033W	HOP2	YOL111C	MDY2
YBL103C	RTG3	YNR072W	HXT17
YEL053C	MAK10	YER122C	GLO3
YLR447C	VMA6	YDR155C	CPR1
YER144C	UBP5	YNL058C	YNL058C
YCR077C	PAT1	YNL147W	LSM7
YBR171W	SEC66	YPL195W	APL5
YLR386W	VAC14	YMR214W	SCJ1
YHR205W	SCH9	YMR311C	GLC8
YPL057C	SUR1	YBR107C	IML3
YDR443C	SSN2	YLR095C	IOC2
YIL159W	BNR1	YPR046W	MCM16
YDR358W	GGA1	YMR152W	YIM1
YGL133W	ITC1	YDL047W	SIT4
YMR231W	PEP5	YLR352W	YLR352W
YOL126C	MDH2	YJR059W	PTK2
YGR249W	MGA1	YKL116C	PRR1

Predictions at threshold level 0.75 continued...			
Gene1 of predicted pair		Gene2 of predicted pair	
Systematic Name	Standard Name	Systematic Name	Standard Name
YPR029C	APL4	YHR108W	GGA2
YMR022W	UBC7	YDL047W	SIT4
YOR109W	INP53	YAL002W	VPS8
YDR174W	HMO1	YOR141C	ARP8
YDL154W	MSH5	YHR079C-A	SAE3
YHL020C	OPI1	YKR030W	GMH1
YOR290C	SNF2	YLR306W	UBC12
YDR443C	SSN2	YLR398C	SKI2
YOR033C	EXO1	YJL187C	SWE1
YNL199C	GCR2	YDR440W	DOT1
YML016C	PPZ1	YLR410W	VIP1
YDR108W	GSG1	YNL127W	FAR11
YBR112C	CYC8	YOR140W	SFL1
YER144C	UBP5	YLR441C	RPS1A
YMR116C	ASC1	YKL048C	ELM1
YDL020C	RPN4	YDR137W	RGP1
YCR077C	PAT1	YLR370C	ARC18
YDR004W	RAD57	YML103C	NUP188
YNL248C	RPA49	YJR063W	RPA12
YOR290C	SNF2	YLR093C	NYV1
YOR195W	SLK19	YJL136C	RPS21B
YMR172W	HOT1	YDR293C	SSD1
YDR137W	RGP1	YER122C	GLO3
YIL159W	BNR1	YDL083C	RPS16B
YGR019W	UGA1	YOL067C	RTG1
YHR134W	WSS1	YDL013W	SLX5
YPL234C	TFP3	YPL120W	VPS30
YPL161C	BEM4	YGR054W	YGR054W
YKR048C	NAP1	YDL047W	SIT4

Predictions at threshold level 0.75 continued...			
Gene1 of predicted pair		Gene2 of predicted pair	
Systematic Name	Standard Name	Systematic Name	Standard Name
YOR132W	VPS17	YHR114W	BZZ1
YLR119W	SRN2	YEL053C	MAK10
YJL124C	LSM1	YJR063W	RPA12
YPL018W	CTF19	YAR014C	BUD14
YPL161C	BEM4	YBR036C	CSG2
YAL010C	MDM10	YHR115C	DMA1
YLR452C	SST2	YPL137C	GIP3
YLR192C	HCR1	YDR358W	GGA1
YDL042C	SIR2	YDR440W	DOT1
YGR072W	UPF3	YER162C	RAD4
YAL024C	LTE1	YJL095W	BCK1
YBR290W	BSD2	YJL190C	RPS22A
YGL115W	SNF4	YAL002W	VPS8
YJL136C	RPS21B	YBL047C	EDE1
YNL215W	IES2	YLR410W	VIP1
YLL039C	UBI4	YKL032C	IXR1
YGL054C	ERV14	YAR014C	BUD14
YOL111C	MDY2	YOR327C	SNC2
YHR178W	STB5	YER139C	YER139C
YDR202C	RAV2	YJR033C	RAV1
YJL127C	SPT10	YDL226C	GCS1
YNL229C	URE2	YGL163C	RAD54
YDR202C	RAV2	YMR054W	STV1
YER173W	RAD24	YGL100W	SEH1
YBR217W	ATG12	YML001W	YPT7
YNL106C	INP52	YDR378C	LSM6
YOR270C	VPH1	YGL025C	PGD1
YGR097W	ASK10	YNL025C	SSN8
YDR289C	RTT103	YOR141C	ARP8

Predictions at threshold level 0.75 continued...			
Gene1 of predicted pair		Gene2 of predicted pair	
Systematic Name	Standard Name	Systematic Name	Standard Name
YDL077C	VAM6	YNL127W	FAR11
YIR003W	YIR003W	YOR284W	HUA2
YDL020C	RPN4	YJR140C	HIR3
YLR079W	SIC1	YLR425W	TUS1
YPR120C	CLB5	YLR254C	NDL1
YBR173C	UMP1	YMR214W	SCJ1
YCL067C	HMLALPHA2	YBR112C	CYC8
YDR318W	MCM21	YLR370C	ARC18
YPL024W	RMI1	YNL032W	SIW14
YOR069W	VPS5	YER162C	RAD4
YDR137W	RGP1	YBR171W	SEC66
YDR174W	HMO1	YBR228W	SLX1
YKL126W	YPK1	YNL238W	KEX2
YMR055C	BUB2	YJR084W	CSN12
YKL007W	CAP1	YIL034C	CAP2
YHL022C	SPO11	YGL213C	SKI8
YPL024W	RMI1	YDL002C	NHP10
YPR046W	MCM16	YDR318W	MCM21
YHR156C	LIN1	YJL172W	CPS1
YDR443C	SSN2	YLR094C	GIS3
YDL184C	RPL41A	YML001W	YPT7
YFR010W	UBP6	YBR108W	YBR108W
YDR443C	SSN2	YDL223C	HBT1
YBR217W	ATG12	YPL037C	EGD1
YDR443C	SSN2	YBL075C	SSA3
YBR131W	CCZ1	YJL095W	BCK1
YGL237C	HAP2	YCR086W	CSM1
YER044C	ERG28	YPR046W	MCM16
YPL161C	BEM4	YMR054W	STV1

Predictions at threshold level 0.75 continued...			
Gene1 of predicted pair		Gene2 of predicted pair	
Systematic Name	Standard Name	Systematic Name	Standard Name
YPL024W	RMI1	YPR120C	CLB5
YLR337C	VRP1	YDR440W	DOT1
YDR477W	SNF1	YMR133W	REC114
YHR156C	LIN1	YNL076W	MKS1
YLR234W	TOP3	YNL291C	MID1
YMR142C	RPL13B	YLR371W	ROM2
YGR249W	MGA1	YDL223C	HBT1
YPL105C	YPL105C	YER122C	GLO3
YCR020C-A	MAK31	YOR327C	SNC2
YBL103C	RTG3	YJR084W	CSN12
YER054C	GIP2	YIL045W	PIG2
YIL076W	SEC28	YOR327C	SNC2
YLL039C	UBI4	YJR125C	ENT3
YLR093C	NYV1	YBR019C	GAL10
YNL087W	TCB2	YJR056C	YJR056C
YLR016C	PML1	YHR152W	SPO12
YDR174W	HMO1	YDR378C	LSM6
YPR017C	DSS4	YHL031C	GOS1
YGL250W	RMR1	YGR188C	BUB1
YCL032W	STE50	YER118C	SHO1
YPL120W	VPS30	YPR119W	CLB2
YOR001W	RRP6	YBR009C	HHF1
YJL036W	SNX4	YEL051W	VMA8
YHR073W	OSH3	YGR059W	SPR3
YNL201C	PSY2	YML103C	NUP188
YGL223C	COG1	YGL200C	EMP24
YBR200W	BEM1	YDL223C	HBT1
YLR170C	APS1	YAL030W	SNC1
YOR080W	DIA2	YOL030W	GAS5

Predictions at threshold level 0.75 continued...			
Gene1 of predicted pair		Gene2 of predicted pair	
Systematic Name	Standard Name	Systematic Name	Standard Name
YMR109W	MYO5	YER122C	GLO3
YPL195W	APL5	YLR370C	ARC18
YLR292C	SEC72	YBR200W	BEM1
YML102W	CAC2	YDR076W	RAD55
YPR131C	NAT3	YKL068W	NUP100
YKL048C	ELM1	YDR318W	MCM21
YLR090W	XDJ1	YIL076W	SEC28
YLR386W	VAC14	YGR270W	YTA7
YGR105W	VMA21	YML071C	COG8
YLR192C	HCR1	YDR129C	SAC6
YPL045W	VPS16	YGL124C	MON1
YPR051W	MAK3	YEL053C	MAK10
YHR111W	UBA4	YLR182W	SWI6
YDR477W	SNF1	YER116C	SLX8
YBL058W	SHP1	YDR484W	VPS52
YJL183W	MNN11	YKL080W	VMA5
YDL116W	NUP84	YER115C	SPR6
YFL023W	BUD27	YMR133W	REC114
YJR140C	HIR3	YOL081W	IRA2
YMR153W	NUP53	YMR133W	REC114
YLR381W	CTF3	YML095C	RAD10
YJL172W	CPS1	YDR137W	RGP1
YLR083C	EMP70	YPR032W	SRO7
YER044C	ERG28	YMR230W	RPS10B
YLR192C	HCR1	YOR290C	SNF2
YJL004C	SYS1	YER122C	GLO3
YOR304W	ISW2	YMR139W	RIM11
YML013W	UBX2	YGR188C	BUB1
YDR289C	RTT103	YPL018W	CTF19

Predictions at threshold level 0.75 continued...			
Gene1 of predicted pair		Gene2 of predicted pair	
Systematic Name	Standard Name	Systematic Name	Standard Name
YGR270W	YTA7	YDR183W	PLP1
YJL053W	PEP8	YLR309C	IMH1
YHR134W	WSS1	YPR051W	MAK3
YDR363W-A	SEM1	YCR077C	PAT1
YOR290C	SNF2	YMR133W	REC114
YPL161C	BEM4	YDR183W	PLP1
YOR043W	WHI2	YLR180W	SAM1
YGL135W	RPL1B	YOR080W	DIA2
YNL087W	TCB2	YMR133W	REC114
YFR010W	UBP6	YDL192W	ARF1
YBL101C	ECM21	YOL081W	IRA2
YBR041W	FAT1	YJL052W	TDH1
YOR358W	HAP5	YDR289C	RTT103
YOR290C	SNF2	YDL083C	RPS16B
YMR231W	PEP5	YHR010W	RPL27A
YBR173C	UMP1	YMR055C	BUB2
YCR063W	BUD31	YPR101W	SNT309
YLR119W	SRN2	YDL223C	HBT1
YGR072W	UPF3	YGL212W	VAM7
YJL036W	SNX4	YNR032W	PPG1
YBR108W	YBR108W	YJR090C	GRR1
YPR018W	RLF2	YDR254W	CHL4
YML117W	NAB6	YLR371W	ROM2
YNL243W	SLA2	YNL094W	APP1
YML115C	VAN1	YOR270C	VPH1
YML103C	NUP188	YDR289C	RTT103
YLR090W	XDJ1	YPL024W	RMI1
YDR076W	RAD55	YDR207C	UME6
YDR293C	SSD1	YNL293W	MSB3

Predictions at threshold level 0.75 continued...			
Gene1 of predicted pair		Gene2 of predicted pair	
Systematic Name	Standard Name	Systematic Name	Standard Name
YPR189W	SKI3	YGL213C	SKI8
YIR002C	MPH1	YPR119W	CLB2
YDL155W	CLB3	YNL107W	YAF9
YBL058W	SHP1	YFR010W	UBP6
YPL253C	VIK1	YML028W	TSA1
YBR030W	YBR030W	YGL003C	CDH1
YDR137W	RGP1	YNL044W	YIP3
YMR224C	MRE11	YNL206C	RTT106
YBR019C	GAL10	YDL192W	ARF1
YPL042C	SSN3	YGL003C	CDH1
YGL190C	CDC55	YML094W	GIM5
YHR098C	SFB3	YLR039C	RIC1
YGL174W	BUD13	YEL013W	VAC8
YIL076W	SEC28	YML071C	COG8
YDL135C	RDI1	YPL112C	PEX25
YBR131W	CCZ1	YLR371W	ROM2
YHL031C	GOS1	YML038C	YMD8
YDR507C	GIN4	YDR422C	SIP1
YDR195W	REF2	YAR014C	BUD14
YBL047C	EDE1	YEL036C	ANP1
YMR039C	SUB1	YHR178W	STB5
YNL238W	KEX2	YKL119C	VPH2
YDR245W	MNN10	YPL158C	YPL158C
YDR358W	GGA1	YER122C	GLO3
YDR261C	EXG2	YBR036C	CSG2
YLR095C	IOC2	YLR371W	ROM2
YMR216C	SKY1	YER162C	RAD4
YLR090W	XDJ1	YBL025W	RRN10
YGR261C	APL6	YDR153C	ENT5

Predictions at threshold level 0.75 continued...			
Gene1 of predicted pair		Gene2 of predicted pair	
Systematic Name	Standard Name	Systematic Name	Standard Name
YDL160C	DHH1	YER027C	GAL83
YJL036W	SNX4	YOL018C	TLG2
YPL105C	YPL105C	YER162C	RAD4
YPL161C	BEM4	YIL008W	URM1
YGL087C	MMS2	YDR289C	RTT103
YLR119W	SRN2	YMR224C	MRE11
YJL030W	MAD2	YIL033C	BCY1
YAL024C	LTE1	YDR289C	RTT103
YLR083C	EMP70	YDL006W	PTC1
YHR111W	UBA4	YDR318W	MCM21
YLR441C	RPS1A	YLR398C	SKI2
YER087C-B	SBH1	YNL044W	YIP3
YAL024C	LTE1	YDR156W	RPA14
YGR097W	ASK10	YGR104C	SRB5
YBL047C	EDE1	YKR020W	VPS51
YPL024W	RMI1	YKL101W	HSL1
YOR127W	RGA1	YOR327C	SNC2
YBR217W	ATG12	YFL023W	BUD27
YOR127W	RGA1	YJR040W	GEF1
YOL126C	MDH2	YER110C	KAP123
YNR052C	POP2	YML028W	TSA1
YIL159W	BNR1	YPL195W	APL5
YNL243W	SLA2	YOR032C	HMS1
YDL184C	RPL41A	YNR072W	HXT17
YLR095C	IOC2	YHR200W	RPN10
YNL218W	MGS1	YNL106C	INP52
YMR039C	SUB1	YLR234W	TOP3
YOR195W	SLK19	YPL253C	VIK1
YAL024C	LTE1	YOR327C	SNC2

Predictions at threshold level 0.75 continued...			
Gene1 of predicted pair		Gene2 of predicted pair	
Systematic Name	Standard Name	Systematic Name	Standard Name
YDR293C	SSD1	YGR072W	UPF3
YEL050C	RML2	YPR120C	CLB5
YPL161C	BEM4	YDR207C	UME6
YPR051W	MAK3	YDL155W	CLB3
YKL129C	MYO3	YBR260C	RGD1
YOR141C	ARP8	YOL091W	SPO21
YBL102W	SFT2	YNL127W	FAR11
YOR035C	SHE4	YJL095W	BCK1
YJR140C	HIR3	YPR120C	CLB5
YMR026C	PEX12	YMR152W	YIM1
YJR135C	MCM22	YNL107W	YAF9
YMR172W	HOT1	YMR109W	MYO5
YBR173C	UMP1	YPR119W	CLB2
YOR275C	RIM20	YBR082C	UBC4
YBL021C	HAP3	YDR289C	RTT103
YGL033W	HOP2	YDL083C	RPS16B
YBR108W	YBR108W	YBL025W	RRN10
YHR111W	UBA4	YJL183W	MNN11
YNL199C	GCR2	YLR371W	ROM2
YOR080W	DIA2	YDR245W	MNN10
YDR183W	PLP1	YAL002W	VPS8
YKL032C	IXR1	YNL079C	TPM1
YLR234W	TOP3	YJL183W	MNN11
YBR200W	BEM1	YER124C	DSE1
YOR033C	EXO1	YGL163C	RAD54
YFR031C-A	RPL2A	YKL116C	PRR1
YNL031C	HHT2	YJL140W	RPB4
YBR173C	UMP1	YPL037C	EGD1
YOR308C	SNU66	YER122C	GLO3

Predictions at threshold level 0.75 continued...			
Gene1 of predicted pair		Gene2 of predicted pair	
Systematic Name	Standard Name	Systematic Name	Standard Name
YPR017C	DSS4	YPR032W	SRO7
YHR081W	LRP1	YMR311C	GLC8
YLR399C	BDF1	YBR010W	HHT1
YDR289C	RTT103	YBR114W	RAD16
YGL147C	RPL9A	YJL183W	MNN11
YJL124C	LSM1	YDR335W	MSN5
YHL031C	GOS1	YJR033C	RAV1
YMR095C	SNO1	YGR249W	MGA1
YOR375C	GDH1	YPL161C	BEM4
YFR031C-A	RPL2A	YEL060C	PRB1
YPL001W	HAT1	YDR310C	SUM1
YKL007W	CAP1	YPR119W	CLB2
YKR014C	YPT52	YML097C	VPS9
YPL022W	RAD1	YJR005W	APL1
YMR004W	MVP1	YHR039C-A	VMA10
YDR443C	SSN2	YGL147C	RPL9A
YGR270W	YTA7	YGL163C	RAD54
YNL236W	SIN4	YJL006C	CTK2
YLR061W	RPL22A	YMR198W	CIK1
YLR447C	VMA6	YHR111W	UBA4
YJR056C	YJR056C	YPL037C	EGD1
YFR031C-A	RPL2A	YGL147C	RPL9A
YER115C	SPR6	YKR024C	DBP7
YGL115W	SNF4	YPL234C	TFP3
YPL161C	BEM4	YBL025W	RRN10
YPR051W	MAK3	YOL108C	INO4
YJR063W	RPA12	YGR270W	YTA7
YDL160C	DHH1	YGL003C	CDH1
YHR156C	LIN1	YER144C	UBP5

Predictions at threshold level 0.75 continued...			
Gene1 of predicted pair		Gene2 of predicted pair	
Systematic Name	Standard Name	Systematic Name	Standard Name
YFR031C-A	RPL2A	YPL161C	BEM4
YKL116C	PRR1	YDR484W	VPS52
YKR072C	SIS2	YML095C	RAD10
YMR095C	SNO1	YOR043W	WHI2
YJL124C	LSM1	YNL225C	CNM67
YNL058C	YNL058C	YBR019C	GAL10
YKL110C	KTI12	YDR183W	PLP1
YKL048C	ELM1	YOR327C	SNC2
YBR131W	CCZ1	YJL183W	MNN11
YBL087C	RPL23A	YDL047W	SIT4
YNL206C	RTT106	YBR009C	HHF1
YOR039W	CKB2	YMR167W	MLH1
YNL218W	MGS1	YDL101C	DUN1
YMR016C	SOK2	YOL111C	MDY2
YHR010W	RPL27A	YMR038C	CCS1
YNL058C	YNL058C	YEL053C	MAK10
YIL036W	CST6	YLR398C	SKI2
YGR040W	KSS1	YBL016W	FUS3
YOR080W	DIA2	YCL037C	SRO9
YDR146C	SWI5	YPL256C	CLN2
YHL020C	OPI1	YGL054C	ERV14
YPR051W	MAK3	YER162C	RAD4
YGL178W	MPT5	YML041C	VPS71
YMR224C	MRE11	YDR358W	GGA1
YOR035C	SHE4	YER040W	GLN3
YJL154C	VPS35	YGR188C	BUB1
YDL223C	HBT1	YEL053C	MAK10
YOR043W	WHI2	YHR081W	LRP1
YOR337W	TEA1	YOR290C	SNF2

Predictions at threshold level 0.75 continued...			
Gene1 of predicted pair		Gene2 of predicted pair	
Systematic Name	Standard Name	Systematic Name	Standard Name
YPL047W	SGF11	YER107C	GLE2
YGR270W	YTA7	YOL067C	RTG1
YOR290C	SNF2	YEL053C	MAK10
YOL006C	TOP1	YGL095C	VPS45
YLR396C	VPS33	YDR323C	PEP7
YKL025C	PAN3	YDL020C	RPN4
YNR072W	HXT17	YPL161C	BEM4
YLR371W	ROM2	YLR324W	PEX30
YGL087C	MMS2	YGR054W	YGR054W
YKL025C	PAN3	YOL067C	RTG1
YCR020W-B	HTL1	YGR275W	RTT102
YKR059W	TIF1	YJL138C	TIF2
YDL116W	NUP84	YIL103W	DPH1
YPR017C	DSS4	YNL051W	COG5
YDR448W	ADA2	YOR038C	HIR2
YGL033W	HOP2	YML115C	VAN1
YIL053W	RHR2	YBR108W	YBR108W
YJR060W	CBF1	YGR135W	PRE9
YPL057C	SUR1	YMR038C	CCS1
YMR127C	SAS2	YGR054W	YGR054W
YNL215W	IES2	YBR195C	MSI1
YIL076W	SEC28	YJR040W	GEF1
YOR132W	VPS17	YKL119C	VPH2
YPR084W	YPR084W	YJR040W	GEF1
YPR131C	NAT3	YDL059C	RAD59
YOL145C	CTR9	YNL031C	HHT2
YDL002C	NHP10	YDR310C	SUM1
YAL024C	LTE1	YBR108W	YBR108W
YDR395W	SXM1	YNL069C	RPL16B

Predictions at threshold level 0.75 continued...			
Gene1 of predicted pair		Gene2 of predicted pair	
Systematic Name	Standard Name	Systematic Name	Standard Name
YJR056C	YJR056C	YMR230W	RPS10B
YMR026C	PEX12	YGL078C	DBP3
YJL036W	SNX4	YAR014C	BUD14
YMR116C	ASC1	YPL213W	LEA1
YBR107C	IML3	YLR315W	NKP2
YFL023W	BUD27	YER162C	RAD4
YAL024C	LTE1	YPL001W	HAT1
YEL053C	MAK10	YOL121C	RPS19A
YOR337W	TEA1	YER139C	YER139C
YNL032W	SIW14	YPL037C	EGD1
YBR181C	RPS6B	YAL002W	VPS8
YMR172W	HOT1	YBL051C	PIN4
YDR484W	VPS52	YML071C	COG8
YHR060W	VMA22	YHL020C	OPI1
YBR260C	RGD1	YHR012W	VPS29
YKL160W	ELF1	YER122C	GLO3
YNL206C	RTT106	YNL116W	DMA2
YDR443C	SSN2	YLR079W	SIC1
YBR278W	DPB3	YDR310C	SUM1
YCR002C	CDC10	YBL085W	BOI1
YBR030W	YBR030W	YGL115W	SNF4
YBR228W	SLX1	YLR135W	SLX4
YPL018W	CTF19	YML112W	CTK3
YDL042C	SIR2	YNL206C	RTT106
YDL223C	HBT1	YBL067C	UBP13
YDR293C	SSD1	YPR032W	SRO7
YKL139W	CTK1	YOR195W	SLK19
YFR010W	UBP6	YNL058C	YNL058C
YHL031C	GOS1	YPL105C	YPL105C

Predictions at threshold level 0.75 continued...			
Gene1 of predicted pair		Gene2 of predicted pair	
Systematic Name	Standard Name	Systematic Name	Standard Name
YNL288W	CAF40	YLR233C	EST1
YNR072W	HXT17	YLR094C	GIS3
YPR036W	VMA13	YOR043W	WHI2
YJR135C	MCM22	YJR005W	APL1
YOR195W	SLK19	YPL022W	RAD1
YGR104C	SRB5	YNL248C	RPA49
YNL243W	SLA2	YLR268W	SEC22
YDR137W	RGP1	YMR152W	YIM1
YMR172W	HOT1	YEL053C	MAK10
YHR060W	VMA22	YOL108C	INO4
YKL139W	CTK1	YLR370C	ARC18
YLR386W	VAC14	YBR280C	SAF1
YNL116W	DMA2	YNL225C	CNM67
YIL034C	CAP2	YJL154C	VPS35
YPL042C	SSN3	YGL115W	SNF4
YFR031C-A	RPL2A	YDR137W	RGP1
YOR043W	WHI2	YJR032W	CPR7
YPL057C	SUR1	YMR152W	YIM1
YLR371W	ROM2	YLR398C	SKI2
YNL180C	RHO5	YFL047W	RGD2
YGL237C	HAP2	YGL253W	HXK2
YER087C-B	SBH1	YLR292C	SEC72
YML095C	RAD10	YDR155C	CPR1
YPL240C	HSP82	YER102W	RPS8B
YLR234W	TOP3	YDR310C	SUM1
YJR060W	CBF1	YNL051W	COG5
YNL087W	TCB2	YBL103C	RTG3
YNL025C	SSN8	YEL013W	VAC8
YGR261C	APL6	YBL102W	SFT2

Predictions at threshold level 0.75 continued...			
Gene1 of predicted pair		Gene2 of predicted pair	
Systematic Name	Standard Name	Systematic Name	Standard Name
YPL161C	BEM4	YML001W	YPT7
YDR293C	SSD1	YGL252C	RTG2
YDR146C	SWI5	YNL236W	SIN4
YLR288C	MEC3	YOR038C	HIR2
YBL088C	TEL1	YLR079W	SIC1
YGL006W	PMC1	YLR043C	TRX1
YGL115W	SNF4	YER162C	RAD4
YFL013C	IES1	YPR119W	CLB2
YGR217W	CCH1	YJL183W	MNN11
YJR140C	HIR3	YHR039C-A	VMA10
YDR277C	MTH1	YDL138W	RGT2
YNL025C	SSN8	YFL013C	IES1
YNL238W	KEX2	YLR371W	ROM2
YML103C	NUP188	YNR052C	POP2
YGR019W	UGA1	YDL155W	CLB3
YJL127C	SPT10	YDL155W	CLB3
YMR080C	NAM7	YOR243C	PUS7
YLR234W	TOP3	YJR032W	CPR7
YBL047C	EDE1	YCR077C	PAT1
YLR337C	VRP1	YBL101C	ECM21
YDR456W	NHX1	YPL161C	BEM4
YDR245W	MNN10	YHR200W	RPN10
YMR142C	RPL13B	YEL053C	MAK10
YJL124C	LSM1	YEL013W	VAC8
YLR095C	IOC2	YKL126W	YPK1
YDL160C	DHH1	YPR120C	CLB5
YNL215W	IES2	YML103C	NUP188
YEL065W	SIT1	YOR066W	MSA1
YPL115C	BEM3	YGR152C	RSR1

Predictions at threshold level 0.75 continued...			
Gene1 of predicted pair		Gene2 of predicted pair	
Systematic Name	Standard Name	Systematic Name	Standard Name
YLR381W	CTF3	YJR090C	GRR1
YJR135C	MCM22	YDR310C	SUM1
YOR033C	EXO1	YNL016W	PUB1
YKR020W	VPS51	YPL120W	VPS30
YPR036W	VMA13	YLR180W	SAM1
YNL069C	RPL16B	YNL127W	FAR11
YJR040W	GEF1	YGL054C	ERV14
YBR289W	SNF5	YBR009C	HHF1
YDR484W	VPS52	YER162C	RAD4
YML016C	PPZ1	YFR036W	CDC26
YDR225W	HTA1	YPL253C	VIK1
YDR004W	RAD57	YPR046W	MCM16
YOR312C	RPL20B	YOR014W	RTS1
YFL026W	STE2	YDR358W	GGA1
YJL136C	RPS21B	YDR264C	AKR1
YDR202C	RAV2	YPL161C	BEM4
YLR006C	SSK1	YJL187C	SWE1
YML095C	RAD10	YKL101W	HSL1
YNL264C	PDR17	YKL041W	VPS24
YGL078C	DBP3	YKL126W	YPK1
YKL032C	IXR1	YGL200C	EMP24
YIR005W	IST3	YER122C	GLO3
YGR184C	UBR1	YPL253C	VIK1
YDR137W	RGP1	YGR071C	YGR071C
YOR066W	MSA1	YJL183W	MNN11
YNL309W	STB1	YLR182W	SWI6
YDR443C	SSN2	YJL154C	VPS35
YMR198W	CIK1	YIL036W	CST6
YBR217W	ATG12	YML071C	COG8

Predictions at threshold level 0.75 continued...			
Gene1 of predicted pair		Gene2 of predicted pair	
Systematic Name	Standard Name	Systematic Name	Standard Name
YKL025C	PAN3	YLR360W	VPS38
YNL243W	SLA2	YJL030W	MAD2
YOR109W	INP53	YOR036W	PEP12
YML057W	CMP2	YPR119W	CLB2
YML071C	COG8	YLR398C	SKI2
YHR161C	YAP1801	YJL029C	VPS53
YKR099W	BAS1	YLR180W	SAM1
YMR198W	CIK1	YPR036W	VMA13
YLR234W	TOP3	YCR066W	RAD18
YLR429W	CRN1	YPR120C	CLB5
YDR378C	LSM6	YNL116W	DMA2
YLR292C	SEC72	YDR144C	MKC7
YNL069C	RPL16B	YHL031C	GOS1
YJR058C	APS2	YDR358W	GGA1
YJL127C	SPT10	YBR200W	BEM1
YJR090C	GRR1	YMR038C	CCS1
YER116C	SLX8	YCR053W	THR4
YDL002C	NHP10	YPR120C	CLB5
YJR063W	RPA12	YPL152W	RRD2
YNL238W	KEX2	YJL183W	MNN11
YML095C	RAD10	YDR379W	RGA2
YIL053W	RHR2	YDR310C	SUM1
YML071C	COG8	YKR030W	GMH1
YPL195W	APL5	YNL107W	YAF9
YDR289C	RTT103	YER139C	YER139C
YDR227W	SIR4	YBR010W	HHT1
YDR261C	EXG2	YDR207C	UME6
YNL031C	HHT2	YNL030W	HHF2
YNL291C	MID1	YAR014C	BUD14

Predictions at threshold level 0.75 continued...			
**Gene1 of predicted pair**		**Gene2 of predicted pair**	
**Systematic Name**	**Standard Name**	**Systematic Name**	**Standard Name**
YGL253W	HXK2	YAR014C	BUD14
YMR319C	FET4	YFL044C	OTU1
YPR017C	DSS4	YGR020C	VMA7
YPR120C	CLB5	YDR183W	PLP1
YPL253C	VIK1	YBR114W	RAD16
YNL106C	INP52	YFR030W	MET10
YDR484W	VPS52	YER144C	UBP5
YAL024C	LTE1	YLR452C	SST2
YJR060W	CBF1	YNR032W	PPG1
YLR148W	PEP3	YLR093C	NYV1
YGR019W	UGA1	YKL048C	ELM1
YFR040W	SAP155	YHL027W	RIM101
YBR010W	HHT1	YNL107W	YAF9
YPL253C	VIK1	YAL010C	MDM10
YPL037C	EGD1	YNL107W	YAF9
YMR198W	CIK1	YLR452C	SST2
YOR312C	RPL20B	YBL101C	ECM21
YDR144C	MKC7	YDR289C	RTT103
YGL087C	MMS2	YJR035W	RAD26
YPR084W	YPR084W	YDR320C	SWA2
YKR001C	VPS1	YDL006W	PTC1
YGL100W	SEH1	YNL273W	TOF1
YGR261C	APL6	YPR119W	CLB2
YHR073W	OSH3	YMR164C	MSS11
YLR079W	SIC1	YNL107W	YAF9
YNL218W	MGS1	YDR207C	UME6
YLR337C	VRP1	YBL047C	EDE1
YBL058W	SHP1	YKR099W	BAS1
YNL223W	ATG4	YLR093C	NYV1

Predictions at threshold level 0.75 continued...			
**Gene1 of predicted pair**		**Gene2 of predicted pair**	
**Systematic Name**	**Standard Name**	**Systematic Name**	**Standard Name**
YNL106C	INP52	YPR120C	CLB5
YML038C	YMD8	YER162C	RAD4
YOL111C	MDY2	YMR133W	REC114
YDL184C	RPL41A	YER044C	ERG28
YBL047C	EDE1	YHR108W	GGA2
YOR127W	RGA1	YBR114W	RAD16
YDR456W	NHX1	YDR207C	UME6
YMR205C	PFK2	YPL089C	RLM1
YDR192C	NUP42	YJR090C	GRR1
YKR020W	VPS51	YJR060W	CBF1
YKL139W	CTK1	YJR135C	MCM22
YPL195W	APL5	YBR036C	CSG2
YNL307C	MCK1	YHR039C-A	VMA10
YBR200W	BEM1	YNL166C	BNI5
YBR107C	IML3	YJR135C	MCM22
YNL243W	SLA2	YKL079W	SMY1
YMR133W	REC114	YBR280C	SAF1
YDR137W	RGP1	YJR032W	CPR7
YNL243W	SLA2	YCR077C	PAT1
YBR108W	YBR108W	YPL246C	RBD2
YKL110C	KTI12	YDR289C	RTT103
YDL116W	NUP84	YBR228W	SLX1
YJR010C-A	SPC1	YER019C-A	SBH2
YLR301W	YLR301W	YBR171W	SEC66
YEL065W	SIT1	YMR153W	NUP53
YBR108W	YBR108W	YDL192W	ARF1
YHR084W	STE12	YER109C	FLO8
YHR012W	VPS29	YOL067C	RTG1
YMR216C	SKY1	YGL115W	SNF4

Predictions at threshold level 0.75 continued...			
Gene1 of predicted pair		Gene2 of predicted pair	
Systematic Name	Standard Name	Systematic Name	Standard Name
YIL076W	SEC28	YNL127W	FAR11
YBR200W	BEM1	YLR381W	CTF3
YNL166C	BNI5	YAL002W	VPS8
YML095C	RAD10	YPR046W	MCM16
YDR155C	CPR1	YHR115C	DMA1
YBR108W	YBR108W	YHR012W	VPS29
YPL253C	VIK1	YJL095W	BCK1
YBR173C	UMP1	YKR099W	BAS1
YDL020C	RPN4	YPR036W	VMA13
YHR203C	RPS4B	YBL072C	RPS8A
YKL025C	PAN3	YDR183W	PLP1
YMR026C	PEX12	YPL057C	SUR1
YBL088C	TEL1	YGL090W	LIF1
YLR135W	SLX4	YGL175C	SAE2
YDR485C	VPS72	YBR089C-A	NHP6B
YDR200C	VPS64	YDL226C	GCS1
YDR335W	MSN5	YJR060W	CBF1
YOR033C	EXO1	YBR215W	HPC2
YKL126W	YPK1	YJL183W	MNN11
YPL024W	RMI1	YBR030W	YBR030W
YFR010W	UBP6	YML115C	VAN1
YBL025W	RRN10	YMR230W	RPS10B
YPR171W	BSP1	YJL095W	BCK1
YDR144C	MKC7	YGL124C	MON1
YMR127C	SAS2	YPL248C	GAL4
YGR104C	SRB5	YJL140W	RPB4
YCR081W	SRB8	YDR477W	SNF1
YLR170C	APS1	YOR035C	SHE4
YMR055C	BUB2	YGL148W	ARO2

Predictions at threshold level 0.75 continued...			
**Gene1 of predicted pair**		**Gene2 of predicted pair**	
**Systematic Name**	**Standard Name**	**Systematic Name**	**Standard Name**
YPR084W	YPR084W	YHL031C	GOS1
YNL199C	GCR2	YGL016W	KAP122
YML016C	PPZ1	YAL002W	VPS8
YOR197W	MCA1	YLL017W	YLL017W
YDR202C	RAV2	YLR447C	VMA6
YGR104C	SRB5	YCR081W	SRB8
YLR452C	SST2	YNL076W	MKS1
YJR063W	RPA12	YCR086W	CSM1
YBR107C	IML3	YGL253W	HXK2
YNL166C	BNI5	YNL078W	NIS1
YGR104C	SRB5	YDL106C	PHO2
YFR010W	UBP6	YNL082W	PMS1
YKR099W	BAS1	YDR358W	GGA1
YNL236W	SIN4	YGL003C	CDH1
YHR134W	WSS1	YPL161C	BEM4
YCL037C	SRO9	YOR327C	SNC2
YBR126C	TPS1	YDR261C	EXG2
YLR240W	VPS34	YOR035C	SHE4
YMR199W	CLN1	YOR275C	RIM20
YDR080W	VPS41	YNL107W	YAF9
YNL307C	MCK1	YBR036C	CSG2
YDR323C	PEP7	YDL226C	GCS1
YOR033C	EXO1	YMR116C	ASC1
YDR108W	GSG1	YLR447C	VMA6
YML016C	PPZ1	YPL152W	RRD2
YNL068C	FKH2	YOR275C	RIM20
YJR135C	MCM22	YMR038C	CCS1
YDR335W	MSN5	YDR076W	RAD55
YHR200W	RPN10	YKL101W	HSL1

Predictions at threshold level 0.75 continued...			
Gene1 of predicted pair		Gene2 of predicted pair	
Systematic Name	Standard Name	Systematic Name	Standard Name
YFL023W	BUD27	YOR327C	SNC2
YPL022W	RAD1	YBL079W	NUP170
YNL215W	IES2	YKL160W	ELF1
YMR224C	MRE11	YHR134W	WSS1
YLR095C	IOC2	YBL025W	RRN10
YLR079W	SIC1	YNL054W	VAC7
YJL095W	BCK1	YOL081W	IRA2
YDR484W	VPS52	YCR088W	ABP1
YHR060W	VMA22	YJR084W	CSN12
YPL105C	YPL105C	YGR105W	VMA21
YGR249W	MGA1	YNL307C	MCK1
YDR245W	MNN10	YPR120C	CLB5
YLR386W	VAC14	YOR290C	SNF2
YIL159W	BNR1	YBR280C	SAF1
YOR127W	RGA1	YKR048C	NAP1
YML016C	PPZ1	YPL158C	YPL158C
YLR191W	PEX13	YKL002W	DID4
YNL243W	SLA2	YMR202W	ERG2
YML103C	NUP188	YNL225C	CNM67
YDL116W	NUP84	YPR120C	CLB5
YGL223C	COG1	YNL107W	YAF9
YOR358W	HAP5	YEL053C	MAK10
YBR030W	YBR030W	YML115C	VAN1
YKR001C	VPS1	YMR054W	STV1
YOR076C	SKI7	YNR052C	POP2
YKL007W	CAP1	YNL069C	RPL16B
YDR079C-A	TFB5	YNL301C	RPL18B
YLR090W	XDJ1	YOR066W	MSA1
YER161C	SPT2	YAL002W	VPS8

Predictions at threshold level 0.75 continued...			
Gene1 of predicted pair		Gene2 of predicted pair	
Systematic Name	Standard Name	Systematic Name	Standard Name
YHR012W	VPS29	YPL195W	APL5
YKL007W	CAP1	YPL050C	MNN9
YKL191W	DPH2	YIL103W	DPH1
YDR146C	SWI5	YDL155W	CLB3
YPR046W	MCM16	YDL077C	VAM6
YJR063W	RPA12	YAR014C	BUD14
YDL013W	SLX5	YDR318W	MCM21
YBR009C	HHF1	YEL056W	HAT2
YKR072C	SIS2	YPL022W	RAD1
YLR064W	YLR064W	YJL004C	SYS1
YPR084W	YPR084W	YGL174W	BUD13
YNL166C	BNI5	YHL002W	HSE1
YLR288C	MEC3	YBR036C	CSG2
YBR042C	CST26	YAL055W	PEX22
YJL053W	PEP8	YPR032W	SRO7
YDL223C	HBT1	YMR089C	YTA12
YKL139W	CTK1	YNL199C	GCR2
YMR032W	HOF1	YER118C	SHO1
YGL178W	MPT5	YLL021W	SPA2
YDR484W	VPS52	YGL025C	PGD1
YDR174W	HMO1	YML103C	NUP188
YDR289C	RTT103	YAL002W	VPS8
YHR178W	STB5	YNL166C	BNI5
YPL057C	SUR1	YHR081W	LRP1
YKR059W	TIF1	YOL045W	PSK2
YDL013W	SLX5	YBL079W	NUP170
YBR171W	SEC66	YDL223C	HBT1
YBR112C	CYC8	YGR252W	GCN5
YML028W	TSA1	YKL114C	APN1

Predictions at threshold level 0.75 continued...			
Gene1 of predicted pair		Gene2 of predicted pair	
Systematic Name	Standard Name	Systematic Name	Standard Name
YMR091C	NPL6	YHL025W	SNF6
YDR080W	VPS41	YNR032W	PPG1
YIL159W	BNR1	YJR084W	CSN12
YNL093W	YPT53	YBR264C	YPT10
YHR039C-A	VMA10	YNL107W	YAF9
YBR215W	HPC2	YJL006C	CTK2
YDR108W	GSG1	YEL043W	YEL043W
YCR063W	BUD31	YGL174W	BUD13
YPR120C	CLB5	YJL157C	FAR1
YDR293C	SSD1	YNL135C	FPR1
YBR274W	CHK1	YEL056W	HAT2
YGR072W	UPF3	YPL079W	RPL21B
YGL078C	DBP3	YBR019C	GAL10
YLR079W	SIC1	YNR052C	POP2
YER116C	SLX8	YDR207C	UME6
YPL057C	SUR1	YKL160W	ELF1
YDR004W	RAD57	YDR192C	NUP42
YLR094C	GIS3	YDR207C	UME6
YLR109W	AHP1	YPR119W	CLB2
YGR071C	YGR071C	YER122C	GLO3
YFL023W	BUD27	YML028W	TSA1
YDR245W	MNN10	YOR014W	RTS1
YNL225C	CNM67	YOR177C	MPC54
YER116C	SLX8	YNL248C	RPA49
YHR086W	NAM8	YMR133W	REC114
YEL065W	SIT1	YPL057C	SUR1
YDR443C	SSN2	YDL184C	RPL41A
YHR111W	UBA4	YFR036W	CDC26
YPL161C	BEM4	YJL117W	PHO86

Predictions at threshold level 0.75 continued...			
Gene1 of predicted pair		Gene2 of predicted pair	
Systematic Name	Standard Name	Systematic Name	Standard Name
YJL176C	SWI3	YHL002W	HSE1
YDR293C	SSD1	YBR107C	IML3
YMR004W	MVP1	YHR060W	VMA22
YDR183W	PLP1	YHL002W	HSE1
YMR104C	YPK2	YOL100W	PKH2
YGL025C	PGD1	YKL048C	ELM1
YJL029C	VPS53	YER122C	GLO3
YDR146C	SWI5	YOR008C	SLG1
YGR239C	PEX21	YDL065C	PEX19
YHL031C	GOS1	YPL057C	SUR1
YDR484W	VPS52	YNR072W	HXT17
YMR052W	FAR3	YDL047W	SIT4
YGL133W	ITC1	YGL031C	RPL24A
YDL116W	NUP84	YOR080W	DIA2
YHR039C-A	VMA10	YPL120W	VPS30
YOR033C	EXO1	YDR156W	RPA14
YLR240W	VPS34	YKR020W	VPS51
YBL008W	HIR1	YNL031C	HHT2
YDR335W	MSN5	YLR350W	ORM2
YOL018C	TLG2	YEL042W	GDA1
YDL160C	DHH1	YGR054W	YGR054W
YBR030W	YBR030W	YPR046W	MCM16
YGL163C	RAD54	YPL018W	CTF19
YIL076W	SEC28	YFL023W	BUD27
YCR086W	CSM1	YHR115C	DMA1
YNL206C	RTT106	YBL047C	EDE1
YOR038C	HIR2	YNL107W	YAF9
YHR134W	WSS1	YOL067C	RTG1
YNR052C	POP2	YPL253C	VIK1

Predictions at threshold level 0.75 continued...			
Gene1 of predicted pair		Gene2 of predicted pair	
Systematic Name	Standard Name	Systematic Name	Standard Name
YDL190C	UFD2	YPR119W	CLB2
YJR135C	MCM22	YJR035W	RAD26
YOR358W	HAP5	YJL190C	RPS22A
YDR155C	CPR1	YHL002W	HSE1
YNL166C	BNI5	YEL056W	HAT2
YPL137C	GIP3	YFR036W	CDC26
YGL066W	SGF73	YLR410W	VIP1
YDR335W	MSN5	YAL002W	VPS8
YBR280C	SAF1	YEL060C	PRB1
YOR069W	VPS5	YGR020C	VMA7
YLR018C	POM34	YML115C	VAN1
YDL077C	VAM6	YOL081W	IRA2
YLR447C	VMA6	YJR032W	CPR7
YDR484W	VPS52	YMR133W	REC114
YPL024W	RMI1	YBR036C	CSG2
YNL307C	MCK1	YNL076W	MKS1
YOR039W	CKB2	YGL019W	CKB1
YPL129W	TAF14	YDR477W	SNF1
YOR290C	SNF2	YJR063W	RPA12
YAL014C	SYN8	YLR093C	NYV1
YPR131C	NAT3	YML028W	TSA1
YLR018C	POM34	YGR059W	SPR3
YOL111C	MDY2	YDL083C	RPS16B
YJL176C	SWI3	YBL025W	RRN10
YJR135C	MCM22	YHR115C	DMA1
YDR443C	SSN2	YKL048C	ELM1
YPR131C	NAT3	YBR107C	IML3
YGL003C	CDH1	YLR131C	ACE2
YOR127W	RGA1	YDR318W	MCM21

Predictions at threshold level 0.75 continued...			
Gene1 of predicted pair		Gene2 of predicted pair	
Systematic Name	Standard Name	Systematic Name	Standard Name
YDL223C	HBT1	YPL195W	APL5
YOR032C	HMS1	YML062C	MFT1
YGR019W	UGA1	YGL124C	MON1
YLR292C	SEC72	YLR234W	TOP3
YDL116W	NUP84	YJR135C	MCM22
YOR054C	VHS3	YKL113C	RAD27
YLR079W	SIC1	YBR194W	SOY1
YOR043W	WHI2	YML001W	YPT7
YGR184C	UBR1	YPL248C	GAL4
YBR084C-A	RPL19A	YHR115C	DMA1
YJL117W	PHO86	YBL101C	ECM21
YMR224C	MRE11	YGL212W	VAM7
YCR020C-A	MAK31	YMR216C	SKY1
YLR337C	VRP1	YBR107C	IML3
YOR132W	VPS17	YLR309C	IMH1
YDL047W	SIT4	YOL091W	SPO21
YHR178W	STB5	YOL081W	IRA2
YNL054W	VAC7	YKL048C	ELM1
YOR039W	CKB2	YIL008W	URM1
YDR440W	DOT1	YOL081W	IRA2
YER144C	UBP5	YGL124C	MON1
YOR368W	RAD17	YLR032W	RAD5
YDR293C	SSD1	YLR309C	IMH1
YER139C	YER139C	YMR038C	CCS1
YBR171W	SEC66	YPL018W	CTF19
YDR004W	RAD57	YDR289C	RTT103
YOR351C	MEK1	YLR263W	RED1
YEL065W	SIT1	YBL103C	RTG3
YLR360W	VPS38	YHR134W	WSS1

Predictions at threshold level 0.75 continued...			
Gene1 of predicted pair		Gene2 of predicted pair	
Systematic Name	Standard Name	Systematic Name	Standard Name
YML095C	RAD10	YML028W	TSA1
YJL136C	RPS21B	YHR152W	SPO12
YLR090W	XDJ1	YOL081W	IRA2
YDL223C	HBT1	YER122C	GLO3
YDL059C	RAD59	YKR001C	VPS1
YJL183W	MNN11	YEL056W	HAT2
YKR057W	RPS21A	YHR111W	UBA4
YGR135W	PRE9	YHL002W	HSE1
YDR293C	SSD1	YCR066W	RAD18
YKR099W	BAS1	YDL083C	RPS16B
YDR382W	RPP2B	YDL184C	RPL41A
YOR337W	TEA1	YLR386W	VAC14
YIL159W	BNR1	YNL076W	MKS1
YLR452C	SST2	YMR133W	REC114
YNR052C	POP2	YOL030W	GAS5
YDR289C	RTT103	YGL031C	RPL24A
YML016C	PPZ1	YDR363W-A	SEM1
YDR265W	PEX10	YPR119W	CLB2
YNR072W	HXT17	YDL083C	RPS16B
YFR010W	UBP6	YOL051W	GAL11
YHR073W	OSH3	YER115C	SPR6
YDR146C	SWI5	YBR036C	CSG2
YAL020C	ATS1	YDR309C	GIC2
YLR360W	VPS38	YPL105C	YPL105C
YHR156C	LIN1	YKL139W	CTK1
YLR381W	CTF3	YPL106C	SSE1
YMR017W	SPO20	YOR036W	PEP12
YIL034C	CAP2	YJL187C	SWE1
YAL024C	LTE1	YLR399C	BDF1

Predictions at threshold level 0.75 continued...			
Gene1 of predicted pair		Gene2 of predicted pair	
Systematic Name	Standard Name	Systematic Name	Standard Name
YLR386W	VAC14	YLR381W	CTF3
YMR116C	ASC1	YLR425W	TUS1
YLR386W	VAC14	YLR399C	BDF1
YOL030W	GAS5	YBL079W	NUP170
YIL053W	RHR2	YBL058W	SHP1
YNL215W	IES2	YOR195W	SLK19
YLR094C	GIS3	YER122C	GLO3
YGR056W	RSC1	YBR089C-A	NHP6B
YDR335W	MSN5	YOR308C	SNU66
YMR039C	SUB1	YNL215W	IES2
YOR290C	SNF2	YNR023W	SNF12
YJR056C	YJR056C	YDL077C	VAM6
YPR017C	DSS4	YJL004C	SYS1
YHR111W	UBA4	YPL256C	CLN2
YER162C	RAD4	YER139C	YER139C
YMR032W	HOF1	YHR061C	GIC1
YLR058C	SHM2	YPL018W	CTF19
YOR270C	VPH1	YPL037C	EGD1
YAL024C	LTE1	YLR320W	MMS22
YOR327C	SNC2	YHR115C	DMA1
YBR084C-A	RPL19A	YKL048C	ELM1
YOR035C	SHE4	YBR082C	UBC4
YKR020W	VPS51	YOR312C	RPL20B
YPL022W	RAD1	YBR082C	UBC4
YBR057C	MUM2	YOR195W	SLK19
YIL034C	CAP2	YMR172W	HOT1
YPR036W	VMA13	YML001W	YPT7
YKL139W	CTK1	YJR084W	CSN12
YCL001W	RER1	YML071C	COG8

Predictions at threshold level 0.75 continued...			
Gene1 of predicted pair		Gene2 of predicted pair	
Systematic Name	Standard Name	Systematic Name	Standard Name
YPR052C	NHP6A	YOR039W	CKB2
YML016C	PPZ1	YGL250W	RMR1
YGL200C	EMP24	YJL006C	CTK2
YHR111W	UBA4	YNL293W	MSB3
YNL291C	MID1	YDR202C	RAV2
YMR039C	SUB1	YEL056W	HAT2
YMR231W	PEP5	YPL045W	VPS16
YBR171W	SEC66	YER044C	ERG28
YPL042C	SSN3	YKL213C	DOA1
YBR173C	UMP1	YOR290C	SNF2
YDL101C	DUN1	YLR313C	SPH1
YOR290C	SNF2	YKL048C	ELM1
YGR019W	UGA1	YGL237C	HAP2
YHR039C-A	VMA10	YKR030W	GMH1
YDR293C	SSD1	YBL088C	TEL1
YHR200W	RPN10	YBR082C	UBC4
YBR217W	ATG12	YHR081W	LRP1
YLR090W	XDJ1	YGL078C	DBP3
YGL078C	DBP3	YNL307C	MCK1
YPR017C	DSS4	YDR293C	SSD1
YBR188C	NTC20	YIR009W	MSL1
YPR018W	RLF2	YOR195W	SLK19
YPL018W	CTF19	YPR119W	CLB2
YKR001C	VPS1	YJR035W	RAD26
YGR072W	UPF3	YDR320C	SWA2
YBL051C	PIN4	YLR233C	EST1
YOR023C	AHC1	YDR155C	CPR1
YCR086W	CSM1	YGL148W	ARO2
YPR029C	APL4	YAL030W	SNC1

Predictions at threshold level 0.75 continued...			
Gene1 of predicted pair		Gene2 of predicted pair	
Systematic Name	Standard Name	Systematic Name	Standard Name
YAL024C	LTE1	YBR173C	UMP1
YOR195W	SLK19	YPL256C	CLN2
YGL190C	CDC55	YLR200W	YKE2
YPL256C	CLN2	YLR131C	ACE2
YPL037C	EGD1	YMR311C	GLC8
YDL116W	NUP84	YDR174W	HMO1
YMR198W	CIK1	YHR012W	VPS29
YHR060W	VMA22	YFR036W	CDC26
YLR386W	VAC14	YER110C	KAP123
YIL103W	DPH1	YFR036W	CDC26
YOR337W	TEA1	YML115C	VAN1
YPR084W	YPR084W	YOR124C	UBP2
YFR030W	MET10	YML112W	CTK3
YLL039C	UBI4	YOR312C	RPL20B
YDR439W	LRS4	YGR188C	BUB1
YKL139W	CTK1	YOR124C	UBP2
YBR057C	MUM2	YMR055C	BUB2
YJL154C	VPS35	YBL051C	PIN4
YDR439W	LRS4	YJL006C	CTK2
YDR244W	PEX5	YGR077C	PEX8
YGL178W	MPT5	YPR119W	CLB2
YOR195W	SLK19	YJL127C	SPT10
YCR086W	CSM1	YLR032W	RAD5
YBR131W	CCZ1	YDL077C	VAM6
YDL006W	PTC1	YNL051W	COG5
YNL032W	SIW14	YGL124C	MON1
YNL044W	YIP3	YCL001W	RER1
YPR051W	MAK3	YGR020C	VMA7
YKL139W	CTK1	YLR315W	NKP2

Predictions at threshold level 0.75 continued...			
Gene1 of predicted pair		Gene2 of predicted pair	
Systematic Name	Standard Name	Systematic Name	Standard Name
YHR012W	VPS29	YDR310C	SUM1
YNL068C	FKH2	YJL136C	RPS21B
YPL253C	VIK1	YPR120C	CLB5
YMR054W	STV1	YAR014C	BUD14
YMR095C	SNO1	YLR320W	MMS22
YGL025C	PGD1	YDR261C	EXG2
YLR452C	SST2	YNL041C	COG6
YBR036C	CSG2	YER122C	GLO3
YDL083C	RPS16B	YPL137C	GIP3
YGL115W	SNF4	YDR422C	SIP1
YKL116C	PRR1	YHR039C-A	VMA10
YDR363W-A	SEM1	YIL008W	URM1
YDR137W	RGP1	YJR033C	RAV1
YDR289C	RTT103	YJR090C	GRR1
YOR080W	DIA2	YBR082C	UBC4
YIL148W	RPL40A	YOR358W	HAP5
YMR095C	SNO1	YBR019C	GAL10
YGR184C	UBR1	YPR036W	VMA13
YLR410W	VIP1	YGL163C	RAD54
YBR215W	HPC2	YGR252W	GCN5
YMR095C	SNO1	YLR192C	HCR1
YHL031C	GOS1	YAL002W	VPS8
YDR385W	EFT2	YDR137W	RGP1
YEL005C	VAB2	YEL013W	VAC8
YLR119W	SRN2	YHR039C-A	VMA10
YHR178W	STB5	YHR114W	BZZ1
YLR233C	EST1	YAL002W	VPS8
YHR098C	SFB3	YOL012C	HTZ1
YDR155C	CPR1	YGL003C	CDH1

Predictions at threshold level 0.75 continued...			
Gene1 of predicted pair		Gene2 of predicted pair	
Systematic Name	Standard Name	Systematic Name	Standard Name
YDL226C	GCS1	YDL137W	ARF2
YOL111C	MDY2	YOR066W	MSA1
YLR079W	SIC1	YBL079W	NUP170
YDL184C	RPL41A	YJL095W	BCK1
YIL036W	CST6	YDR363W-A	SEM1
YBR200W	BEM1	YMR038C	CCS1
YNL307C	MCK1	YGL003C	CDH1
YDL116W	NUP84	YHR073W	OSH3
YPL024W	RMI1	YLR234W	TOP3
YMR198W	CIK1	YJL095W	BCK1
YCL037C	SRO9	YEL060C	PRB1
YGR133W	PEX4	YKR014C	YPT52
YLR148W	PEP3	YHR161C	YAP1801
YDR477W	SNF1	YLR386W	VAC14
YCR077C	PAT1	YGL148W	ARO2
YBL021C	HAP3	YDR254W	CHL4
YOR132W	VPS17	YHR111W	UBA4
YPL037C	EGD1	YER139C	YER139C
YPR028W	YOP1	YGL198W	YIP4
YPR051W	MAK3	YGR188C	BUB1
YCR024C-A	PMP1	YGL016W	KAP122
YOR195W	SLK19	YDR264C	AKR1
YKR072C	SIS2	YMR055C	BUB2
YPR051W	MAK3	YCL037C	SRO9
YHR152W	SPO12	YHR200W	RPN10
YFR030W	MET10	YGR262C	BUD32
YBR200W	BEM1	YBR215W	HPC2
YIL053W	RHR2	YLR370C	ARC18
YJR040W	GEF1	YNL238W	KEX2

Predictions at threshold level 0.75 continued...			
Gene1 of predicted pair		Gene2 of predicted pair	
Systematic Name	Standard Name	Systematic Name	Standard Name
YDR261C	EXG2	YJR084W	CSN12
YOL126C	MDH2	YPL256C	CLN2
YMR026C	PEX12	YDL223C	HBT1
YFL023W	BUD27	YNL076W	MKS1
YOR270C	VPH1	YDL223C	HBT1
YHR156C	LIN1	YJL183W	MNN11
YKL032C	IXR1	YLR425W	TUS1
YNL238W	KEX2	YNL127W	FAR11
YDR378C	LSM6	YLR410W	VIP1
YJR032W	CPR7	YCR053W	THR4
YOR106W	VAM3	YPL234C	TFP3
YMR198W	CIK1	YMR038C	CCS1
YHR060W	VMA22	YDR207C	UME6
YDL155W	CLB3	YHR114W	BZZ1
YDR318W	MCM21	YNL116W	DMA2
YDL065C	PEX19	YOL044W	PEX15
YPR040W	TIP41	YLR425W	TUS1
YMR109W	MYO5	YJR090C	GRR1
YOR043W	WHI2	YLR398C	SKI2
YER075C	PTP3	YER089C	PTC2
YLR090W	XDJ1	YPL057C	SUR1
YOR327C	SNC2	YKR030W	GMH1
YMR004W	MVP1	YNL127W	FAR11
YMR116C	ASC1	YDL223C	HBT1
YOR033C	EXO1	YNL076W	MKS1
YLR381W	CTF3	YGR135W	PRE9
YMR152W	YIM1	YDL077C	VAM6
YBR200W	BEM1	YNL229C	URE2
YFR031C-A	RPL2A	YLR370C	ARC18

Predictions at threshold level 0.75 continued...			
Gene1 of predicted pair		Gene2 of predicted pair	
Systematic Name	Standard Name	Systematic Name	Standard Name
YLR018C	POM34	YGL215W	CLG1
YMR224C	MRE11	YNL291C	MID1
YHR111W	UBA4	YHR200W	RPN10
YFR024C-A	LSB3	YPR119W	CLB2
YKR093W	PTR2	YPR149W	NCE102
YLR135W	SLX4	YBR073W	RDH54
YEL065W	SIT1	YNL058C	YNL058C
YHR039C-A	VMA10	YPR032W	SRO7
YER118C	SHO1	YER040W	GLN3
YNL199C	GCR2	YOR014W	RTS1
YPR084W	YPR084W	YNL291C	MID1
YJR032W	CPR7	YNL051W	COG5
YDL116W	NUP84	YKL139W	CTK1
YGL163C	RAD54	YOR014W	RTS1
YIL076W	SEC28	YKR020W	VPS51
YLR079W	SIC1	YLR371W	ROM2
YMR016C	SOK2	YPL037C	EGD1
YGL212W	VAM7	YPL037C	EGD1
YGR249W	MGA1	YHR060W	VMA22
YFR031C-A	RPL2A	YNL307C	MCK1
YGL087C	MMS2	YEL053C	MAK10
YNL054W	VAC7	YJL124C	LSM1
YNR052C	POP2	YML112W	CTK3
YMR080C	NAM7	YNL106C	INP52
YJR052W	RAD7	YBR114W	RAD16
YJL176C	SWI3	YKL032C	IXR1
YDR174W	HMO1	YFR036W	CDC26
YJR135C	MCM22	YDR318W	MCM21
YDL020C	RPN4	YPL234C	TFP3

Predictions at threshold level 0.75 continued...			
Gene1 of predicted pair		Gene2 of predicted pair	
Systematic Name	Standard Name	Systematic Name	Standard Name
YCR002C	CDC10	YDR309C	GIC2
YPL042C	SSN3	YOR308C	SNU66
YHR156C	LIN1	YLR452C	SST2
YBR194W	SOY1	YKL114C	APN1
YPL057C	SUR1	YHR167W	THP2
YLR360W	VPS38	YBL051C	PIN4
YPL037C	EGD1	YMR214W	SCJ1
YML115C	VAN1	YLR180W	SAM1
YDL080C	THI3	YNL107W	YAF9
YNL087W	TCB2	YKL126W	YPK1
YPL137C	GIP3	YLR180W	SAM1
YER116C	SLX8	YPL253C	VIK1
YPL253C	VIK1	YDR200C	VPS64
YHR039C-A	VMA10	YNL076W	MKS1
YML071C	COG8	YDR207C	UME6
YLR192C	HCR1	YLR093C	NYV1
YDR129C	SAC6	YDL161W	ENT1
YOR018W	ROD1	YLR248W	RCK2
YPR084W	YPR084W	YLR182W	SWI6
YOR106W	VAM3	YKR030W	GMH1
YLR313C	SPH1	YDL013W	SLX5
YER110C	KAP123	YBR107C	IML3
YKR099W	BAS1	YNL041C	COG6
YDR443C	SSN2	YER044C	ERG28
YDL013W	SLX5	YBR082C	UBC4
YMR216C	SKY1	YEL053C	MAK10
YPR018W	RLF2	YOR101W	RAS1
YIL076W	SEC28	YHR167W	THP2
YMR070W	MOT3	YOR035C	SHE4

Predictions at threshold level 0.75 continued...			
Gene1 of predicted pair		Gene2 of predicted pair	
Systematic Name	Standard Name	Systematic Name	Standard Name
YGL115W	SNF4	YLR320W	MMS22
YDR080W	VPS41	YER120W	SCS2
YAL024C	LTE1	YDR363W-A	SEM1
YFL003C	MSH4	YPR119W	CLB2
YBR228W	SLX1	YMR116C	ASC1
YMR054W	STV1	YNR032W	PPG1
YOR106W	VAM3	YAR014C	BUD14
YLR386W	VAC14	YGL253W	HXK2
YKL139W	CTK1	YPL253C	VIK1
YNL291C	MID1	YNL051W	COG5
YLR360W	VPS38	YBR200W	BEM1
YJL172W	CPS1	YOR290C	SNF2
YLR381W	CTF3	YML028W	TSA1
YOL067C	RTG1	YNL107W	YAF9
YGL175C	SAE2	YOL090W	MSH2
YGL087C	MMS2	YDL013W	SLX5
YIL156W	UBP7	YKL068W	NUP100
YML109W	ZDS2	YDR309C	GIC2
YGR100W	MDR1	YNL293W	MSB3
YMR004W	MVP1	YNL238W	KEX2
YGR104C	SRB5	YNR052C	POP2
YDR363W-A	SEM1	YJL183W	MNN11
YDR254W	CHL4	YGL151W	NUT1
YEL050C	RML2	YNL107W	YAF9
YGR072W	UPF3	YFR010W	UBP6
YGR071C	YGR071C	YNL041C	COG6
YDR484W	VPS52	YJL172W	CPS1
YGL087C	MMS2	YDR076W	RAD55
YPL178W	CBC2	YFL047W	RGD2

Predictions at threshold level 0.75 continued...			
Gene1 of predicted pair		Gene2 of predicted pair	
Systematic Name	Standard Name	Systematic Name	Standard Name
YDR261C	EXG2	YNL076W	MKS1
YGR105W	VMA21	YAL055W	PEX22
YHR081W	LRP1	YMR214W	SCJ1
YKL214C	YRA2	YBR111W-A	SUS1
YDR496C	PUF6	YHR052W	CIC1
YBL058W	SHP1	YKL139W	CTK1
YMR142C	RPL13B	YJR084W	CSN12
YLR381W	CTF3	YOR023C	AHC1
YFR031C-A	RPL2A	YPL105C	YPL105C
YGL066W	SGF73	YBL047C	EDE1
YMR231W	PEP5	YDL226C	GCS1
YDR156W	RPA14	YDR335W	MSN5
YPL057C	SUR1	YGR188C	BUB1
YJR032W	CPR7	YOL081W	IRA2
YJL154C	VPS35	YJR040W	GEF1
YPL161C	BEM4	YER162C	RAD4
YIL076W	SEC28	YNL032W	SIW14
YOR290C	SNF2	YGL003C	CDH1
YLR288C	MEC3	YBR274W	CHK1
YFL023W	BUD27	YHR081W	LRP1
YER116C	SLX8	YLR381W	CTF3
YML016C	PPZ1	YNL147W	LSM7
YKR020W	VPS51	YNL051W	COG5
YLR292C	SEC72	YGL151W	NUT1
YLR119W	SRN2	YPL137C	GIP3
YML071C	COG8	YPR032W	SRO7
YBL025W	RRN10	YLR398C	SKI2
YML121W	GTR1	YKR007W	MEH1
YNL243W	SLA2	YHR108W	GGA2

Predictions at threshold level 0.75 continued...			
Gene1 of predicted pair		Gene2 of predicted pair	
Systematic Name	Standard Name	Systematic Name	Standard Name
YPL253C	VIK1	YJR063W	RPA12
YNL215W	IES2	YPL018W	CTF19
YGR105W	VMA21	YJR033C	RAV1
YGL066W	SGF73	YOR275C	RIM20
YKR061W	KTR2	YMR055C	BUB2
YLR119W	SRN2	YNR072W	HXT17
YJR056C	YJR056C	YDR137W	RGP1
YDR443C	SSN2	YMR230W	RPS10B
YHR060W	VMA22	YEL053C	MAK10
YJR140C	HIR3	YOR308C	SNU66
YDR484W	VPS52	YOR043W	WHI2
YLR102C	APC9	YNL116W	DMA2
YPL115C	BEM3	YLR447C	VMA6
YDR378C	LSM6	YDR183W	PLP1
YMR016C	SOK2	YBR280C	SAF1
YER044C	ERG28	YJR032W	CPR7
YNL288W	CAF40	YGR108W	CLB1
YBR030W	YBR030W	YJL172W	CPS1
YDR245W	MNN10	YDR207C	UME6
YLR093C	NYV1	YMR214W	SCJ1
YKL116C	PRR1	YOR290C	SNF2
YML038C	YMD8	YNR032W	PPG1
YKL213C	DOA1	YER122C	GLO3
YLR337C	VRP1	YGR108W	CLB1
YGL237C	HAP2	YDR183W	PLP1
YDR334W	SWR1	YGL190C	CDC55
YJR140C	HIR3	YGR252W	GCN5
YDR293C	SSD1	YDR310C	SUM1
YJL154C	VPS35	YNL291C	MID1

Predictions at threshold level 0.75 continued...			
Gene1 of predicted pair		Gene2 of predicted pair	
Systematic Name	Standard Name	Systematic Name	Standard Name
YJL124C	LSM1	YHR115C	DMA1
YJL137C	GLG2	YIL045W	PIG2
YNL236W	SIN4	YLR370C	ARC18
YMR109W	MYO5	YBL016W	FUS3
YDR004W	RAD57	YMR224C	MRE11
YDR080W	VPS41	YOR036W	PEP12
YIL008W	URM1	YAL002W	VPS8
YLR216C	CPR6	YJR135C	MCM22
YGL115W	SNF4	YNL051W	COG5
YBR127C	VMA2	YKR020W	VPS51
YER087C-B	SBH1	YPL047W	SGF11
YPL024W	RMI1	YBR215W	HPC2
YDR378C	LSM6	YJR060W	CBF1
YDR363W-A	SEM1	YOR312C	RPL20B
YGL174W	BUD13	YDR378C	LSM6
YDL184C	RPL41A	YBR171W	SEC66
YPL057C	SUR1	YOR043W	WHI2
YGR071C	YGR071C	YHR077C	NMD2
YOR043W	WHI2	YJL183W	MNN11
YNL032W	SIW14	YMR214W	SCJ1
YBR108W	YBR108W	YPL037C	EGD1
YLR119W	SRN2	YOR066W	MSA1
YFR031C-A	RPL2A	YKR020W	VPS51
YJR070C	LIA1	YEL022W	GEA2
YBR274W	CHK1	YPR120C	CLB5
YDR183W	PLP1	YML112W	CTK3
YPR029C	APL4	YOR327C	SNC2
YER001W	MNN1	YNL079C	TPM1
YGL087C	MMS2	YLR150W	STM1

Predictions at threshold level 0.75 continued...			
Gene1 of predicted pair		Gene2 of predicted pair	
Systematic Name	Standard Name	Systematic Name	Standard Name
YPR131C	NAT3	YJR135C	MCM22
YGL078C	DBP3	YLR371W	ROM2
YNL069C	RPL16B	YIL076W	SEC28
YMR026C	PEX12	YBR217W	ATG12
YOR132W	VPS17	YHR060W	VMA22
YBL058W	SHP1	YGL124C	MON1
YER001W	MNN1	YFR036W	CDC26
YPL018W	CTF19	YNL225C	CNM67
YJL176C	SWI3	YDR129C	SAC6
YER116C	SLX8	YMR133W	REC114
YHL031C	GOS1	YGL095C	VPS45
YOR290C	SNF2	YLR398C	SKI2
YGL033W	HOP2	YLR180W	SAM1
YJL127C	SPT10	YPL018W	CTF19
YBR217W	ATG12	YDL077C	VAM6
YMR039C	SUB1	YDR254W	CHL4
YBR171W	SEC66	YDL006W	PTC1
YGR104C	SRB5	YJR140C	HIR3
YGR217W	CCH1	YBR041W	FAT1
YBR030W	YBR030W	YNR072W	HXT17
YGL078C	DBP3	YOR290C	SNF2
YPR032W	SRO7	YAL002W	VPS8
YLR386W	VAC14	YER122C	GLO3
YMR039C	SUB1	YGL163C	RAD54
YDR293C	SSD1	YML001W	YPT7
YKL119C	VPH2	YKL041W	VPS24
YCL037C	SRO9	YER162C	RAD4
YIL036W	CST6	YDR289C	RTT103
YDR137W	RGP1	YHR167W	THP2

Predictions at threshold level 0.75 continued...			
Gene1 of predicted pair		Gene2 of predicted pair	
Systematic Name	Standard Name	Systematic Name	Standard Name
YDR225W	HTA1	YPL129W	TAF14
YNL201C	PSY2	YAR014C	BUD14
YDR477W	SNF1	YPR030W	CSR2
YLR079W	SIC1	YDR245W	MNN10
YIL034C	CAP2	YDR183W	PLP1
YDR358W	GGA1	YBL025W	RRN10
YGL200C	EMP24	YOR141C	ARP8
YGR135W	PRE9	YHR115C	DMA1
YDL006W	PTC1	YDR310C	SUM1
YER116C	SLX8	YBR215W	HPC2
YIL034C	CAP2	YMR055C	BUB2
YBR215W	HPC2	YNL236W	SIN4
YPL024W	RMI1	YOR014W	RTS1
YJR040W	GEF1	YPL234C	TFP3
YMR109W	MYO5	YER118C	SHO1
YPR051W	MAK3	YKR030W	GMH1
YCL037C	SRO9	YOR038C	HIR2
YER040W	GLN3	YOL081W	IRA2
YER139C	YER139C	YOL081W	IRA2
YDL116W	NUP84	YER116C	SLX8
YJL172W	CPS1	YDL077C	VAM6
YGL078C	DBP3	YHR060W	VMA22
YOL111C	MDY2	YGL124C	MON1
YAL024C	LTE1	YGL003C	CDH1
YAL024C	LTE1	YDR358W	GGA1
YCR039C	MATALPHA2	YPL240C	HSP82
YGR108W	CLB1	YOR141C	ARP8
YGR249W	MGA1	YBR217W	ATG12
YDR318W	MCM21	YHR115C	DMA1

Predictions at threshold level 0.75 continued...			
Gene1 of predicted pair		Gene2 of predicted pair	
Systematic Name	Standard Name	Systematic Name	Standard Name
YHR079C	IRE1	YOL031C	SIL1
YJR140C	HIR3	YML115C	VAN1
YHR012W	VPS29	YGL252C	RTG2
YIL008W	URM1	YNL079C	TPM1
YPL024W	RMI1	YKR099W	BAS1
YBR258C	SHG1	YDR155C	CPR1
YPR030W	CSR2	YDL192W	ARF1
YCR081W	SRB8	YCR084C	TUP1
YNL248C	RPA49	YJL140W	RPB4
YDL116W	NUP84	YPR131C	NAT3
YMR080C	NAM7	YJR140C	HIR3
YBL103C	RTG3	YJL183W	MNN11
YOL091W	SPO21	YNL107W	YAF9
YLR234W	TOP3	YGL148W	ARO2
YLR119W	SRN2	YPL057C	SUR1
YDR378C	LSM6	YPL213W	LEA1
YGL033W	HOP2	YPL037C	EGD1
YNL325C	FIG4	YOR066W	MSA1
YER044C	ERG28	YKR035W-A	DID2
YDR320C	SWA2	YPR119W	CLB2
YPR017C	DSS4	YPL120W	VPS30
YGR133W	PEX4	YJR084W	CSN12
YIL159W	BNR1	YGL124C	MON1
YNL206C	RTT106	YMR179W	SPT21
YJL124C	LSM1	YBR036C	CSG2
YLR102C	APC9	YNL229C	URE2
YJR140C	HIR3	YGL174W	BUD13
YOR195W	SLK19	YOR101W	RAS1
YLL007C	YLL007C	YJL190C	RPS22A

Predictions at threshold level 0.75 continued...			
Gene1 of predicted pair		Gene2 of predicted pair	
Systematic Name	Standard Name	Systematic Name	Standard Name
YGR217W	CCH1	YMR038C	CCS1
YOR270C	VPH1	YPL161C	BEM4
YER028C	MIG3	YPR119W	CLB2
YLR360W	VPS38	YBR171W	SEC66
YBL102W	SFT2	YHR012W	VPS29
YLR216C	CPR6	YCR077C	PAT1
YFR031C-A	RPL2A	YMR214W	SCJ1
YLR386W	VAC14	YPL152W	RRD2
YHR200W	RPN10	YGL124C	MON1
YKL139W	CTK1	YLR093C	NYV1
YGR019W	UGA1	YGR188C	BUB1
YMR139W	RIM11	YDR207C	UME6
YDR395W	SXM1	YER110C	KAP123
YMR004W	MVP1	YLR309C	IMH1
YPL001W	HAT1	YBR195C	MSI1
YBL058W	SHP1	YML115C	VAN1
YPL161C	BEM4	YDR318W	MCM21
YML103C	NUP188	YAR014C	BUD14
YML115C	VAN1	YER044C	ERG28
YIL156W	UBP7	YML103C	NUP188
YNL289W	PCL1	YOR327C	SNC2
YMR224C	MRE11	YNR072W	HXT17
YLR360W	VPS38	YKR030W	GMH1
YOR033C	EXO1	YHR134W	WSS1
YDR358W	GGA1	YJR125C	ENT3
YOR276W	CAF20	YOL045W	PSK2
YBR030W	YBR030W	YER040W	GLN3
YPR131C	NAT3	YKL114C	APN1
YLR360W	VPS38	YGL253W	HXK2

Predictions at threshold level 0.75 continued...			
Gene1 of predicted pair		Gene2 of predicted pair	
Systematic Name	Standard Name	Systematic Name	Standard Name
YHR152W	SPO12	YPL161C	BEM4
YNL307C	MCK1	YBR019C	GAL10
YPL253C	VIK1	YFR030W	MET10
YPR017C	DSS4	YDR202C	RAV2
YMR273C	ZDS1	YPL203W	TPK2
YDR156W	RPA14	YML103C	NUP188
YKL126W	YPK1	YBL047C	EDE1
YKR072C	SIS2	YOR038C	HIR2
YPL046C	ELC1	YGL115W	SNF4
YHR167W	THP2	YNL107W	YAF9
YKL213C	DOA1	YHL002W	HSE1
YJL176C	SWI3	YKR014C	YPT52
YPR102C	RPL11A	YGR100W	MDR1
YFL023W	BUD27	YPR030W	CSR2
YOL145C	CTR9	YDR155C	CPR1
YKL139W	CTK1	YOR080W	DIA2
YGL054C	ERV14	YEL051W	VMA8
YFR031C-A	RPL2A	YDR320C	SWA2
YMR273C	ZDS1	YGL016W	KAP122
YHR160C	PEX18	YNL214W	PEX17
YGR089W	NNF2	YCR086W	CSM1
YNL201C	PSY2	YLR386W	VAC14
YHR156C	LIN1	YJR084W	CSN12
YFL023W	BUD27	YBR280C	SAF1
YDR383C	NKP1	YJR060W	CBF1
YNL076W	MKS1	YEL060C	PRB1
YIR005W	IST3	YNL107W	YAF9
YDL223C	HBT1	YHR081W	LRP1
YMR198W	CIK1	YPL001W	HAT1

Predictions at threshold level 0.75 continued...			
Gene1 of predicted pair		Gene2 of predicted pair	
Systematic Name	Standard Name	Systematic Name	Standard Name
YBL008W	HIR1	YCR077C	PAT1
YER179W	DMC1	YNL106C	INP52
YKL116C	PRR1	YJR084W	CSN12
YOR132W	VPS17	YJR090C	GRR1
YDL160C	DHH1	YGR134W	CAF130
YLR360W	VPS38	YDR080W	VPS41
YMR039C	SUB1	YNL236W	SIN4
YNL236W	SIN4	YDR318W	MCM21
YEL065W	SIT1	YLR452C	SST2
YDR181C	SAS4	YMR127C	SAS2
YML103C	NUP188	YGR270W	YTA7
YBR217W	ATG12	YPL195W	APL5
YLR119W	SRN2	YDL077C	VAM6
YHR156C	LIN1	YNL041C	COG6
YBL058W	SHP1	YCR053W	THR4
YBR107C	IML3	YJR005W	APL1
YLR337C	VRP1	YHR013C	ARD1
YMR142C	RPL13B	YKR035W-A	DID2
YDR108W	GSG1	YDL192W	ARF1
YJL176C	SWI3	YLR360W	VPS38
YHR178W	STB5	YBR200W	BEM1
YPL161C	BEM4	YGL163C	RAD54
YBR057C	MUM2	YLR381W	CTF3
YDL047W	SIT4	YKL114C	APN1
YGL115W	SNF4	YDL192W	ARF1
YPL024W	RMI1	YKL139W	CTK1
YBR171W	SEC66	YJL183W	MNN11
YPL240C	HSP82	YBR089C-A	NHP6B
YPR120C	CLB5	YOR368W	RAD17

Predictions at threshold level 0.75 continued...			
Gene1 of predicted pair		Gene2 of predicted pair	
Systematic Name	Standard Name	Systematic Name	Standard Name
YLR192C	HCR1	YOL081W	IRA2
YDR383C	NKP1	YPL253C	VIK1
YKL010C	UFD4	YML115C	VAN1
YDR129C	SAC6	YLR371W	ROM2
YMR004W	MVP1	YJL004C	SYS1
YDR477W	SNF1	YGL003C	CDH1
YDL020C	RPN4	YJR032W	CPR7
YPR018W	RLF2	YLR102C	APC9
YGL163C	RAD54	YOR368W	RAD17
YPR084W	YPR084W	YDR207C	UME6
YBL008W	HIR1	YDL042C	SIR2
YLR192C	HCR1	YMR198W	CIK1
YLR234W	TOP3	YFR036W	CDC26
YLR083C	EMP70	YHL020C	OPI1
YJR090C	GRR1	YAL002W	VPS8
YCL067C	HMLALPHA2	YCR039C	MATALPHA2
YDR379W	RGA2	YJL095W	BCK1
YDL013W	SLX5	YDR207C	UME6
YPL024W	RMI1	YJL183W	MNN11
YBL058W	SHP1	YPR030W	CSR2
YLR102C	APC9	YIL008W	URM1
YPR084W	YPR084W	YOR112W	CEX1
YKL068W	NUP100	YDR076W	RAD55
YMR199W	CLN1	YER027C	GAL83
YJL006C	CTK2	YKL041W	VPS24
YMR179W	SPT21	YPL253C	VIK1
YNL201C	PSY2	YLR254C	NDL1
YOR080W	DIA2	YDL002C	NHP10
YJL154C	VPS35	YCR088W	ABP1

Predictions at threshold level 0.75 continued...			
Gene1 of predicted pair		Gene2 of predicted pair	
Systematic Name	Standard Name	Systematic Name	Standard Name
YIL076W	SEC28	YMR214W	SCJ1
YNL079C	TPM1	YGL019W	CKB1
YDL116W	NUP84	YPL042C	SSN3
YJL183W	MNN11	YMR230W	RPS10B
YLR381W	CTF3	YDR155C	CPR1
YBR173C	UMP1	YPL022W	RAD1
YER116C	SLX8	YNL087W	TCB2
YMR004W	MVP1	YOL018C	TLG2
YOR109W	INP53	YJR033C	RAV1
YMR124W	YMR124W	YEL043W	YEL043W
YKL139W	CTK1	YLR180W	SAM1
YOR106W	VAM3	YNR007C	ATG3
YMR202W	ERG2	YGL115W	SNF4
YGL078C	DBP3	YDL184C	RPL41A
YPL046C	ELC1	YER162C	RAD4
YDR254W	CHL4	YPR119W	CLB2
YBL017C	PEP1	YJR125C	ENT3
YDL184C	RPL41A	YKR099W	BAS1
YDR443C	SSN2	YER162C	RAD4
YJR032W	CPR7	YLR398C	SKI2
YMR142C	RPL13B	YGL115W	SNF4
YJL004C	SYS1	YNL127W	FAR11
YLR234W	TOP3	YBR036C	CSG2
YER179W	DMC1	YBR073W	RDH54
YPL024W	RMI1	YER139C	YER139C
YGL025C	PGD1	YPL161C	BEM4
YBR289W	SNF5	YKL109W	HAP4
YKL139W	CTK1	YHR200W	RPN10
YMR153W	NUP53	YGL003C	CDH1

Predictions at threshold level 0.75 continued...			
**Gene1 of predicted pair**		**Gene2 of predicted pair**	
**Systematic Name**	**Standard Name**	**Systematic Name**	**Standard Name**
YKL126W	YPK1	YER122C	GLO3
YEL053C	MAK10	YDR261C	EXG2
YOR127W	RGA1	YGR054W	YGR054W
YBR009C	HHF1	YPL248C	GAL4
YBR127C	VMA2	YJL183W	MNN11
YHR160C	PEX18	YJL210W	PEX2
YLR095C	IOC2	YKR035W-A	DID2
YEL053C	MAK10	YJR032W	CPR7
YPL046C	ELC1	YOR028C	CIN5
YEL027W	CUP5	YKR001C	VPS1
YPL037C	EGD1	YKR035W-A	DID2
YOR127W	RGA1	YCL037C	SRO9
YJL058C	BIT61	YPL180W	TCO89
YOR106W	VAM3	YGL198W	YIP4
YLR315W	NKP2	YDR440W	DOT1
YNL051W	COG5	YNL107W	YAF9
YKL139W	CTK1	YOR001W	RRP6
YGR184C	UBR1	YCL014W	BUD3
YIL159W	BNR1	YNL058C	YNL058C
YJL036W	SNX4	YOR109W	INP53
YOR195W	SLK19	YPR120C	CLB5
YDL180W	YDL180W	YMR004W	MVP1
YNL289W	PCL1	YNL116W	DMA2
YER144C	UBP5	YDR207C	UME6
YDL155W	CLB3	YOL067C	RTG1
YGR252W	GCN5	YHR200W	RPN10
YOR083W	WHI5	YJR005W	APL1
YMR179W	SPT21	YDR200C	VPS64
YBR009C	HHF1	YOR141C	ARP8

Predictions at threshold level 0.75 continued...			
**Gene1 of predicted pair**		**Gene2 of predicted pair**	
**Systematic Name**	**Standard Name**	**Systematic Name**	**Standard Name**
YOR001W	RRP6	YER139C	YER139C
YDR320C	SWA2	YJR031C	GEA1
YDR176W	NGG1	YJL140W	RPB4
YLR337C	VRP1	YOR308C	SNU66
YOR035C	SHE4	YDR207C	UME6
YJL176C	SWI3	YER161C	SPT2
YBL008W	HIR1	YDL002C	NHP10
YOR069W	VPS5	YNL107W	YAF9
YML016C	PPZ1	YBR107C	IML3
YPL253C	VIK1	YPL152W	RRD2
YLR386W	VAC14	YER162C	RAD4
YFL023W	BUD27	YOR035C	SHE4
YMR116C	ASC1	YMR205C	PFK2
YBR030W	YBR030W	YKR035W-A	DID2
YOR083W	WHI5	YPR120C	CLB5
YLR360W	VPS38	YGL200C	EMP24
YDL184C	RPL41A	YER040W	GLN3
YKR020W	VPS51	YNL107W	YAF9
YDL006W	PTC1	YGR105W	VMA21
YLR093C	NYV1	YOL081W	IRA2
YOR140W	SFL1	YCR084C	TUP1
YDL020C	RPN4	YMR224C	MRE11
YNL147W	LSM7	YOL081W	IRA2
YDR176W	NGG1	YLR182W	SWI6
YGL200C	EMP24	YOR035C	SHE4
YHR178W	STB5	YBR274W	CHK1
YPL246C	RBD2	YKL068W	NUP100
YOR001W	RRP6	YKL213C	DOA1
YGR104C	SRB5	YBR089C-A	NHP6B

Predictions at threshold level 0.75 continued...			
Gene1 of predicted pair		Gene2 of predicted pair	
Systematic Name	Standard Name	Systematic Name	Standard Name
YNL218W	MGS1	YMR284W	YKU70
YHR060W	VMA22	YAL002W	VPS8
YPL046C	ELC1	YER059W	PCL6
YOR106W	VAM3	YOL018C	TLG2
YMR273C	ZDS1	YBR009C	HHF1
YDR383C	NKP1	YNL248C	RPA49
YBR200W	BEM1	YJL187C	SWE1
YGR019W	UGA1	YHR012W	VPS29
YOR337W	TEA1	YDR477W	SNF1
YGL212W	VAM7	YER040W	GLN3
YKR035W-A	DID2	YPL195W	APL5
YPL024W	RMI1	YBR278W	DPB3
YGL054C	ERV14	YML067C	ERV41
YDL155W	CLB3	YGL124C	MON1
YNL325C	FIG4	YGL212W	VAM7
YEL065W	SIT1	YFR031C-A	RPL2A
YDR137W	RGP1	YGL124C	MON1
YER116C	SLX8	YDR076W	RAD55
YBR127C	VMA2	YKL080W	VMA5
YDL116W	NUP84	YML016C	PPZ1
YLR083C	EMP70	YDR202C	RAV2
YGL054C	ERV14	YJR060W	CBF1
YDR363W-A	SEM1	YDL226C	GCS1
YLR095C	IOC2	YOR337W	TEA1
YNL325C	FIG4	YDL184C	RPL41A
YIL053W	RHR2	YOR043W	WHI2
YPL152W	RRD2	YPR046W	MCM16
YOR080W	DIA2	YLR350W	ORM2
YMR284W	YKU70	YPL022W	RAD1

Predictions at threshold level 0.75 continued...			
Gene1 of predicted pair		Gene2 of predicted pair	
Systematic Name	Standard Name	Systematic Name	Standard Name
YBR215W	HPC2	YBR195C	MSI1
YDL116W	NUP84	YLR135W	SLX4
YHR084W	STE12	YDR103W	STE5
YLR386W	VAC14	YDR289C	RTT103
YNL087W	TCB2	YPR046W	MCM16
YGL078C	DBP3	YOR270C	VPH1
YER115C	SPR6	YNL199C	GCR2
YER144C	UBP5	YFR036W	CDC26
YLR018C	POM34	YLR079W	SIC1
YKR001C	VPS1	YPL161C	BEM4
YKL032C	IXR1	YOL081W	IRA2
YGR104C	SRB5	YLR399C	BDF1
YLR386W	VAC14	YJL172W	CPS1
YPL195W	APL5	YNR032W	PPG1
YJR059W	PTK2	YMR153W	NUP53
YGL175C	SAE2	YJR090C	GRR1
YMR004W	MVP1	YER120W	SCS2
YPL022W	RAD1	YDR363W-A	SEM1
YIL034C	CAP2	YDR318W	MCM21
YJR063W	RPA12	YBR289W	SNF5
YPL042C	SSN3	YFR010W	UBP6
YEL044W	IES6	YDL223C	HBT1
YIL008W	URM1	YOL067C	RTG1
YML115C	VAN1	YLR032W	RAD5
YBL103C	RTG3	YOL108C	INO4
YER162C	RAD4	YPR119W	CLB2
YHR012W	VPS29	YKR030W	GMH1
YML095C	RAD10	YPR119W	CLB2
YGR104C	SRB5	YPL042C	SSN3

Predictions at threshold level 0.75 continued...			
Gene1 of predicted pair		Gene2 of predicted pair	
Systematic Name	Standard Name	Systematic Name	Standard Name
YDR202C	RAV2	YPL195W	APL5
YPL022W	RAD1	YJL058C	BIT61
YLR006C	SSK1	YNR031C	SSK2
YLR447C	VMA6	YER072W	VTC1
YFR010W	UBP6	YNL087W	TCB2
YLR441C	RPS1A	YDL083C	RPS16B
YER096W	SHC1	YPL256C	CLN2
YDL184C	RPL41A	YNL041C	COG6
YKL116C	PRR1	YGL115W	SNF4
YOR066W	MSA1	YMR230W	RPS10B
YHR115C	DMA1	YGL252C	RTG2
YJR056C	YJR056C	YMR133W	REC114
YER114C	BOI2	YBL085W	BOI1
YOR106W	VAM3	YPR028W	YOP1
YPR018W	RLF2	YLR032W	RAD5
YGR104C	SRB5	YHL031C	GOS1
YLR360W	VPS38	YHR060W	VMA22
YJL154C	VPS35	YDR153C	ENT5
YBR195C	MSI1	YNL183C	NPR1
YJR056C	YJR056C	YBR036C	CSG2
YNL291C	MID1	YDR153C	ENT5
YEL065W	SIT1	YBR036C	CSG2
YOR145C	PNO1	YHR081W	LRP1
YIL076W	SEC28	YNL087W	TCB2
YER161C	SPT2	YMR038C	CCS1
YIL034C	CAP2	YKL213C	DOA1
YLR119W	SRN2	YNR006W	VPS27
YJL060W	BNA3	YGL003C	CDH1
YMR224C	MRE11	YGL003C	CDH1

Predictions at threshold level 0.75 continued...			
Gene1 of predicted pair		Gene2 of predicted pair	
Systematic Name	Standard Name	Systematic Name	Standard Name
YLR386W	VAC14	YJR135C	MCM22
YPR051W	MAK3	YMR054W	STV1
YHR200W	RPN10	YFL007W	BLM10
YJL187C	SWE1	YOL090W	MSH2
YLR288C	MEC3	YOR039W	CKB2
YOR001W	RRP6	YLR350W	ORM2
YKL032C	IXR1	YBL072C	RPS8A
YAL024C	LTE1	YNL107W	YAF9
YNL307C	MCK1	YER044C	ERG28
YDR202C	RAV2	YKR001C	VPS1
YML071C	COG8	YER040W	GLN3
YKL010C	UFD4	YGL200C	EMP24
YNL087W	TCB2	YNL076W	MKS1
YGL163C	RAD54	YIL103W	DPH1
YJL053W	PEP8	YOR106W	VAM3
YDL185W	TFP1	YKL080W	VMA5
YLR150W	STM1	YPR120C	CLB5
YGR135W	PRE9	YGL031C	RPL24A
YER116C	SLX8	YMR231W	PEP5
YDL122W	UBP1	YPR119W	CLB2
YDR137W	RGP1	YPR030W	CSR2
YDR225W	HTA1	YKL101W	HSL1
YLR148W	PEP3	YOR036W	PEP12
YNL236W	SIN4	YOR141C	ARP8
YBL008W	HIR1	YPR046W	MCM16
YER162C	RAD4	YMR230W	RPS10B
YJR106W	ECM27	YOR071C	THI71
YPL115C	BEM3	YPL256C	CLN2
YDR146C	SWI5	YOR038C	HIR2

Predictions at threshold level 0.75 continued...			
Gene1 of predicted pair		Gene2 of predicted pair	
Systematic Name	Standard Name	Systematic Name	Standard Name
YKL048C	ELM1	YGL252C	RTG2
YPL057C	SUR1	YDL083C	RPS16B
YMR172W	HOT1	YOR132W	VPS17
YPR101W	SNT309	YBR114W	RAD16
YPL115C	BEM3	YGL253W	HXK2
YIL159W	BNR1	YFR036W	CDC26
YPL045W	VPS16	YAL002W	VPS8
YDR137W	RGP1	YDL223C	HBT1
YLR288C	MEC3	YDL002C	NHP10
YGL025C	PGD1	YHR200W	RPN10
YDL155W	CLB3	YDR155C	CPR1
YNL145W	MFA2	YKR026C	GCN3
YBR041W	FAT1	YJL183W	MNN11
YDR507C	GIN4	YBL087C	RPL23A
YNL243W	SLA2	YIL002C	INP51
YER144C	UBP5	YOL111C	MDY2
YBR194W	SOY1	YEL013W	VAC8
YKL010C	UFD4	YLR425W	TUS1
YDR443C	SSN2	YOR014W	RTS1
YBR217W	ATG12	YGL115W	SNF4
YML016C	PPZ1	YHR108W	GGA2
YLR119W	SRN2	YKR035W-A	DID2
YBR200W	BEM1	YOR212W	STE4
YNL020C	ARK1	YCR088W	ABP1
YJL190C	RPS22A	YAL002W	VPS8
YML038C	YMD8	YEL053C	MAK10
YKL185W	ASH1	YFR001W	LOC1
YLR386W	VAC14	YDR264C	AKR1
YMR142C	RPL13B	YDL192W	ARF1

Predictions at threshold level 0.75 continued...			
Gene1 of predicted pair		Gene2 of predicted pair	
Systematic Name	Standard Name	Systematic Name	Standard Name
YPL115C	BEM3	YJR135C	MCM22
YFR031C-A	RPL2A	YHL031C	GOS1
YOR112W	CEX1	YDL006W	PTC1
YPL253C	VIK1	YLR182W	SWI6
YLR386W	VAC14	YOR043W	WHI2
YKR061W	KTR2	YDR289C	RTT103
YNL068C	FKH2	YHR115C	DMA1
YGL078C	DBP3	YOL111C	MDY2
YDR469W	SDC1	YNL107W	YAF9
YBR217W	ATG12	YBR036C	CSG2
YPL234C	TFP3	YNL051W	COG5
YPL246C	RBD2	YML057W	CMP2
YDR261C	EXG2	YER122C	GLO3
YDR108W	GSG1	YMR216C	SKY1
YDL223C	HBT1	YOR312C	RPL20B
YJL053W	PEP8	YHR060W	VMA22
YKL032C	IXR1	YHR111W	UBA4
YGR217W	CCH1	YBR131W	CCZ1
YHR134W	WSS1	YLR386W	VAC14
YPL057C	SUR1	YDR310C	SUM1
YDL184C	RPL41A	YLR441C	RPS1A
YDL077C	VAM6	YAL002W	VPS8
YPL256C	CLN2	YJL187C	SWE1
YJL172W	CPS1	YEL060C	PRB1
YNL041C	COG6	YHR081W	LRP1
YBR171W	SEC66	YOR358W	HAP5
YBR112C	CYC8	YNL167C	SKO1
YLL007C	YLL007C	YGR108W	CLB1
YNL069C	RPL16B	YJL004C	SYS1

Predictions at threshold level 0.75 continued...			
Gene1 of predicted pair		Gene2 of predicted pair	
Systematic Name	Standard Name	Systematic Name	Standard Name
YKR020W	VPS51	YAL030W	SNC1
YOL090W	MSH2	YOR368W	RAD17
YLR079W	SIC1	YGL252C	RTG2
YLR337C	VRP1	YPL234C	TFP3
YFR036W	CDC26	YBL025W	RRN10
YMR116C	ASC1	YOL030W	GAS5
YKR061W	KTR2	YLR254C	NDL1
YJL129C	TRK1	YGR262C	BUD32
YFR031C-A	RPL2A	YDL184C	RPL41A
YKR001C	VPS1	YOL018C	TLG2
YGR014W	MSB2	YMR089C	YTA12
YJR135C	MCM22	YDR183W	PLP1
YJR063W	RPA12	YJR135C	MCM22
YBR217W	ATG12	YDR207C	UME6
YIL034C	CAP2	YDL020C	RPN4
YHR200W	RPN10	YIL008W	URM1
YMR224C	MRE11	YOR368W	RAD17
YKR001C	VPS1	YKR020W	VPS51
YPR017C	DSS4	YER120W	SCS2
YGL078C	DBP3	YOR066W	MSA1
YJL124C	LSM1	YKL048C	ELM1
YHR156C	LIN1	YML115C	VAN1
YKL048C	ELM1	YER162C	RAD4
YNR072W	HXT17	YPL195W	APL5
YDR439W	LRS4	YKL048C	ELM1
YFR010W	UBP6	YMR153W	NUP53
YHR156C	LIN1	YOR035C	SHE4
YMR224C	MRE11	YER044C	ERG28
YOR106W	VAM3	YDR207C	UME6

Predictions at threshold level 0.75 continued...			
**Gene1 of predicted pair**		**Gene2 of predicted pair**	
**Systematic Name**	**Standard Name**	**Systematic Name**	**Standard Name**
YPR051W	MAK3	YNL127W	FAR11
YHR200W	RPN10	YMR311C	GLC8
YKL032C	IXR1	YKR001C	VPS1
YDL059C	RAD59	YHR010W	RPL27A
YMR055C	BUB2	YJR060W	CBF1
YOR127W	RGA1	YPL256C	CLN2
YLR386W	VAC14	YNL079C	TPM1
YER116C	SLX8	YOR043W	WHI2
YML103C	NUP188	YML112W	CTK3
YDR076W	RAD55	YML028W	TSA1
YOR195W	SLK19	YGL163C	RAD54
YDR383C	NKP1	YKL048C	ELM1
YLR371W	ROM2	YER040W	GLN3
YOR106W	VAM3	YGL054C	ERV14
YKR057W	RPS21A	YOR275C	RIM20
YPR084W	YPR084W	YLR309C	IMH1
YGL078C	DBP3	YML115C	VAN1
YDR507C	GIN4	YDR379W	RGA2
YGL054C	ERV14	YLR309C	IMH1
YMR179W	SPT21	YJL124C	LSM1
YLR018C	POM34	YER116C	SLX8
YPR084W	YPR084W	YFL013C	IES1
YML016C	PPZ1	YPL018W	CTF19
YGR159C	NSR1	YPL161C	BEM4
YDR477W	SNF1	YJL095W	BCK1
YER016W	BIM1	YGL094C	PAN2
YKR101W	SIR1	YNL031C	HHT2
YIL053W	RHR2	YDR137W	RGP1
YCR020C-A	MAK31	YOR112W	CEX1

Predictions at threshold level 0.75 continued...			
Gene1 of predicted pair		Gene2 of predicted pair	
Systematic Name	Standard Name	Systematic Name	Standard Name
YFL023W	BUD27	YER122C	GLO3
YNR052C	POP2	YDR289C	RTT103
YOR140W	SFL1	YEL036C	ANP1
YNL079C	TPM1	YOL110W	SHR5
YER116C	SLX8	YCR086W	CSM1
YDR310C	SUM1	YPR032W	SRO7
YPR018W	RLF2	YFR010W	UBP6
YOR001W	RRP6	YDL047W	SIT4
YML115C	VAN1	YNL032W	SIW14
YOR080W	DIA2	YMR038C	CCS1
YDL020C	RPN4	YBR108W	YBR108W
YNR052C	POP2	YJL141C	YAK1
YOR127W	RGA1	YJR060W	CBF1
YBR107C	IML3	YHR013C	ARD1
YDL006W	PTC1	YHR158C	KEL1
YOR337W	TEA1	YER144C	UBP5
YBR173C	UMP1	YLR452C	SST2
YDL160C	DHH1	YJL124C	LSM1
YNL032W	SIW14	YHR081W	LRP1
YOR109W	INP53	YFL033C	RIM15
YLR386W	VAC14	YNL041C	COG6
YDR383C	NKP1	YOR195W	SLK19
YBL079W	NUP170	YJR090C	GRR1
YDR156W	RPA14	YGL252C	RTG2
YML062C	MFT1	YOL145C	CTR9
YPL234C	TFP3	YML001W	YPT7
YDR439W	LRS4	YAR014C	BUD14
YHR203C	RPS4B	YER102W	RPS8B
YBR289W	SNF5	YNL236W	SIN4

Predictions at threshold level 0.75 continued...			
Gene1 of predicted pair		Gene2 of predicted pair	
Systematic Name	Standard Name	Systematic Name	Standard Name
YLR234W	TOP3	YJR140C	HIR3
YDR477W	SNF1	YCR084C	TUP1
YER144C	UBP5	YER044C	ERG28
YPR032W	SRO7	YDR207C	UME6
YGR217W	CCH1	YJL117W	PHO86
YBR173C	UMP1	YLR320W	MMS22
YLR288C	MEC3	YJR140C	HIR3
YKL116C	PRR1	YBR108W	YBR108W
YLR381W	CTF3	YBL021C	HAP3
YBR108W	YBR108W	YOR284W	HUA2
YLR360W	VPS38	YGL095C	VPS45
YDL184C	RPL41A	YEL060C	PRB1
YER144C	UBP5	YDR310C	SUM1
YDR192C	NUP42	YML103C	NUP188
YDR108W	GSG1	YJR040W	GEF1
YGL003C	CDH1	YLR180W	SAM1
YKR026C	GCN3	YNL147W	LSM7
YHR012W	VPS29	YDR207C	UME6
YLR148W	PEP3	YKR001C	VPS1
YEL051W	VMA8	YNL127W	FAR11
YMR224C	MRE11	YBR173C	UMP1
YER122C	GLO3	YLR180W	SAM1
YDL223C	HBT1	YJL183W	MNN11
YDR456W	NHX1	YER049W	TPA1
YLR206W	ENT2	YIL079C	AIR1
YKL025C	PAN3	YLL007C	YLL007C
YDL160C	DHH1	YKR059W	TIF1
YGL115W	SNF4	YJR084W	CSN12
YGL147C	RPL9A	YMR133W	REC114

Predictions at threshold level 0.75 continued...			
Gene1 of predicted pair		Gene2 of predicted pair	
Systematic Name	Standard Name	Systematic Name	Standard Name
YGR188C	BUB1	YDR310C	SUM1
YER162C	RAD4	YNL127W	FAR11
YBR108W	YBR108W	YML001W	YPT7
YHR039C-A	VMA10	YPL195W	APL5
YHR013C	ARD1	YAL002W	VPS8
YBL051C	PIN4	YML095C	RAD10
YER116C	SLX8	YPL195W	APL5
YBR131W	CCZ1	YOR275C	RIM20
YJL095W	BCK1	YEL060C	PRB1
YGR104C	SRB5	YFR034C	PHO4
YOR039W	CKB2	YBR200W	BEM1
YOR270C	VPH1	YJR084W	CSN12
YDR490C	PKH1	YKL126W	YPK1
YPR017C	DSS4	YOR132W	VPS17
YIL076W	SEC28	YCR077C	PAT1
YOL054W	PSH1	YLR182W	SWI6
YDR225W	HTA1	YBR107C	IML3
YMR052W	FAR3	YPR119W	CLB2
YNL068C	FKH2	YDR264C	AKR1
YHR081W	LRP1	YBR036C	CSG2
YOR096W	RPS7A	YBL072C	RPS8A
YEL065W	SIT1	YLR386W	VAC14
YGL163C	RAD54	YDR183W	PLP1
YDR129C	SAC6	YLR425W	TUS1
YNL127W	FAR11	YNL107W	YAF9
YBR127C	VMA2	YDR323C	PEP7
YLR148W	PEP3	YGR241C	YAP1802
YDL013W	SLX5	YPR119W	CLB2
YDR439W	LRS4	YLR315W	NKP2

Predictions at threshold level 0.75 continued...			
Gene1 of predicted pair		Gene2 of predicted pair	
Systematic Name	Standard Name	Systematic Name	Standard Name
YOR195W	SLK19	YDL013W	SLX5
YGL025C	PGD1	YJL183W	MNN11
YNL068C	FKH2	YOR127W	RGA1
YMR133W	REC114	YOL081W	IRA2
YDR225W	HTA1	YDR448W	ADA2
YGR019W	UGA1	YEL056W	HAT2
YGR270W	YTA7	YDL155W	CLB3
YGR249W	MGA1	YPR036W	VMA13
YKL010C	UFD4	YKL006W	RPL14A
YPL161C	BEM4	YML071C	COG8
YDR156W	RPA14	YOR195W	SLK19
YBR108W	YBR108W	YPL018W	CTF19
YPR084W	YPR084W	YDR310C	SUM1
YGL147C	RPL9A	YOL111C	MDY2
YER122C	GLO3	YMR214W	SCJ1
YLR170C	APS1	YJR058C	APS2
YOR132W	VPS17	YAL030W	SNC1
YIL159W	BNR1	YMR230W	RPS10B
YNL215W	IES2	YPL057C	SUR1
YPL152W	RRD2	YHR115C	DMA1
YOR337W	TEA1	YMR133W	REC114
YDL083C	RPS16B	YDR310C	SUM1
YLR386W	VAC14	YBR171W	SEC66
YHR052W	CIC1	YPR119W	CLB2
YIL148W	RPL40A	YPL256C	CLN2
YPR051W	MAK3	YOL067C	RTG1
YLR360W	VPS38	YIL076W	SEC28
YMR026C	PEX12	YJL095W	BCK1
YMR224C	MRE11	YNL087W	TCB2

Predictions at threshold level 0.75 continued...			
Gene1 of predicted pair		Gene2 of predicted pair	
Systematic Name	Standard Name	Systematic Name	Standard Name
YNL291C	MID1	YDL006W	PTC1
YJR084W	CSN12	YBR181C	RPS6B
YDR153C	ENT5	YER122C	GLO3
YER116C	SLX8	YBL079W	NUP170
YMR055C	BUB2	YLR370C	ARC18
YGR108W	CLB1	YEL050C	RML2
YEL056W	HAT2	YHR114W	BZZ1
YOR078W	BUD21	YDR025W	RPS11A
YDL160C	DHH1	YPL024W	RMI1
YDR443C	SSN2	YHR200W	RPN10
YLR079W	SIC1	YJL095W	BCK1
YJR060W	CBF1	YOL081W	IRA2
YPL057C	SUR1	YOR014W	RTS1
YNL031C	HHT2	YOR141C	ARP8
YKL160W	ELF1	YJR060W	CBF1
YPL024W	RMI1	YHR156C	LIN1
YLR288C	MEC3	YHR039C-A	VMA10
YFR036W	CDC26	YEL060C	PRB1
YML001W	YPT7	YBL025W	RRN10
YKL057C	NUP120	YER105C	NUP157
YGR261C	APL6	YOR112W	CEX1
YJR060W	CBF1	YJR135C	MCM22
YDR202C	RAV2	YGL054C	ERV14
YPR051W	MAK3	YDR183W	PLP1
YJL154C	VPS35	YLR360W	VPS38
YDR318W	MCM21	YBR114W	RAD16
YML012W	ERV25	YJL004C	SYS1
YEL009C	GCN4	YOL051W	GAL11
YNL201C	PSY2	YKL139W	CTK1

Predictions at threshold level 0.75 continued...			
Gene1 of predicted pair		Gene2 of predicted pair	
Systematic Name	Standard Name	Systematic Name	Standard Name
YGL147C	RPL9A	YDR477W	SNF1
YMR139W	RIM11	YPR119W	CLB2
YPR052C	NHP6A	YKL139W	CTK1
YMR224C	MRE11	YDR076W	RAD55
YLR090W	XDJ1	YMR311C	GLC8
YKL181W	PRS1	YOR043W	WHI2
YJL183W	MNN11	YER040W	GLN3
YLR191W	PEX13	YAR042W	SWH1
YGL215W	CLG1	YDR137W	RGP1
YHR156C	LIN1	YLR441C	RPS1A
YMR153W	NUP53	YEL060C	PRB1
YJL127C	SPT10	YDR200C	VPS64
YAL024C	LTE1	YPR119W	CLB2
YJL124C	LSM1	YPL057C	SUR1
YHR016C	YSC84	YKR030W	GMH1
YLR441C	RPS1A	YDR137W	RGP1
YDL185W	TFP1	YOL081W	IRA2
YDR363W-A	SEM1	YJL140W	RPB4
YPL253C	VIK1	YNL248C	RPA49
YMR127C	SAS2	YJR032W	CPR7
YBR258C	SHG1	YPL161C	BEM4
YMR016C	SOK2	YMR311C	GLC8
YFR010W	UBP6	YLR032W	RAD5
YLR441C	RPS1A	YJL183W	MNN11
YBR200W	BEM1	YGL025C	PGD1
YOR312C	RPL20B	YBL025W	RRN10
YNL307C	MCK1	YPL195W	APL5
YMR153W	NUP53	YML007W	YAP1
YML071C	COG8	YOR035C	SHE4

Predictions at threshold level 0.75 continued...			
Gene1 of predicted pair		Gene2 of predicted pair	
Systematic Name	Standard Name	Systematic Name	Standard Name
YNL330C	RPD3	YBR089C-A	NHP6B
YLR095C	IOC2	YLR094C	GIS3
YJR060W	CBF1	YFR030W	MET10
YEL027W	CUP5	YDL185W	TFP1
YPR171W	BSP1	YPL050C	MNN9
YDR293C	SSD1	YAL002W	VPS8
YBR200W	BEM1	YEL036C	ANP1
YDR310C	SUM1	YLR350W	ORM2
YOR337W	TEA1	YDR310C	SUM1
YOR001W	RRP6	YDR245W	MNN10
YNL215W	IES2	YGL161C	YIP5
YMR199W	CLN1	YLR452C	SST2
YJL006C	CTK2	YCR086W	CSM1
YEL065W	SIT1	YER139C	YER139C
YDR507C	GIN4	YKL032C	IXR1
YGL174W	BUD13	YBR036C	CSG2
YNL069C	RPL16B	YHR060W	VMA22
YLR288C	MEC3	YCR077C	PAT1
YBR173C	UMP1	YMR153W	NUP53
YDL020C	RPN4	YMR172W	HOT1
YAL024C	LTE1	YMR142C	RPL13B
YFR010W	UBP6	YDL077C	VAM6
YDR443C	SSN2	YPL057C	SUR1
YEL053C	MAK10	YOL108C	INO4
YPL137C	GIP3	YBR280C	SAF1
YDL155W	CLB3	YOR014W	RTS1
YBL058W	SHP1	YDL192W	ARF1
YBR264C	YPT10	YKR014C	YPT52
YGR252W	GCN5	YOR038C	HIR2

Predictions at threshold level 0.75 continued...			
Gene1 of predicted pair		Gene2 of predicted pair	
Systematic Name	Standard Name	Systematic Name	Standard Name
YBL021C	HAP3	YGL253W	HXK2
YER116C	SLX8	YOL111C	MDY2
YLL039C	UBI4	YGR135W	PRE9
YHR178W	STB5	YER161C	SPT2
YML016C	PPZ1	YGL252C	RTG2
YOR069W	VPS5	YCL037C	SRO9
YGL178W	MPT5	YER068W	MOT2
YBR126C	TPS1	YKL126W	YPK1
YNR072W	HXT17	YKL048C	ELM1
YMR224C	MRE11	YPR030W	CSR2
YLR315W	NKP2	YGR188C	BUB1
YBR274W	CHK1	YDL059C	RAD59
YNL054W	VAC7	YPR120C	CLB5
YPR018W	RLF2	YGL087C	MMS2
YNL297C	MON2	YJR033C	RAV1
YBR030W	YBR030W	YNL076W	MKS1
YHR060W	VMA22	YGL003C	CDH1
YAL014C	SYN8	YJL204C	RCY1
YLR452C	SST2	YER139C	YER139C
YGR135W	PRE9	YFR036W	CDC26
YNR072W	HXT17	YDR310C	SUM1
YDL185W	TFP1	YPR119W	CLB2
YLR386W	VAC14	YOL111C	MDY2
YMR224C	MRE11	YMR153W	NUP53
YPL057C	SUR1	YGL237C	HAP2
YHR111W	UBA4	YOL081W	IRA2
YIL053W	RHR2	YMR142C	RPL13B
YDR335W	MSN5	YKL068W	NUP100
YOR039W	CKB2	YNL030W	HHF2

Predictions at threshold level 0.75 continued...			
Gene1 of predicted pair		Gene2 of predicted pair	
Systematic Name	Standard Name	Systematic Name	Standard Name
YBR111W-A	SUS1	YAR002W	NUP60
YMR198W	CIK1	YDR076W	RAD55
YPL001W	HAT1	YDR289C	RTT103
YJL006C	CTK2	YDR183W	PLP1
YEL053C	MAK10	YDL192W	ARF1
YJL172W	CPS1	YML001W	YPT7
YKR057W	RPS21A	YKR048C	NAP1
YDL184C	RPL41A	YOL121C	RPS19A
YLR192C	HCR1	YDR310C	SUM1
YJL124C	LSM1	YNL116W	DMA2
YJL176C	SWI3	YGR105W	VMA21
YDR080W	VPS41	YHR039C-A	VMA10
YHR073W	OSH3	YML103C	NUP188
YML112W	CTK3	YNL116W	DMA2
YNL243W	SLA2	YOR106W	VAM3
YIL156W	UBP7	YLR079W	SIC1
YJL124C	LSM1	YHR013C	ARD1
YDR484W	VPS52	YHR039C-A	VMA10
YBR093C	PHO5	YMR198W	CIK1
YBR020W	GAL1	YJR060W	CBF1
YDR443C	SSN2	YDR477W	SNF1
YOR132W	VPS17	YIL008W	URM1
YNL238W	KEX2	YJR125C	ENT3
YLR288C	MEC3	YML013W	UBX2
YKL139W	CTK1	YHR060W	VMA22
YJL151C	SNA3	YNL094W	APP1
YDR363W-A	SEM1	YPR046W	MCM16
YMR198W	CIK1	YNL236W	SIN4
YLR018C	POM34	YKL041W	VPS24

Predictions at threshold level 0.75 continued...			
Gene1 of predicted pair		Gene2 of predicted pair	
Systematic Name	Standard Name	Systematic Name	Standard Name
YOR039W	CKB2	YLR399C	BDF1
YPL129W	TAF14	YOR140W	SFL1
YOR066W	MSA1	YCR053W	THR4
YLR192C	HCR1	YGL025C	PGD1
YDR378C	LSM6	YHR115C	DMA1
YHR012W	VPS29	YPR032W	SRO7
YMR070W	MOT3	YBR127C	VMA2
YIL008W	URM1	YDR318W	MCM21
YLL007C	YLL007C	YGR019W	UGA1
YLR337C	VRP1	YLR061W	RPL22A
YLR090W	XDJ1	YEL060C	PRB1
YMR172W	HOT1	YLR292C	SEC72
YDR137W	RGP1	YKL080W	VMA5
YGR054W	YGR054W	YDR310C	SUM1
YLR119W	SRN2	YBR171W	SEC66
YDR443C	SSN2	YER049W	TPA1
YMR142C	RPL13B	YKL048C	ELM1
YIL076W	SEC28	YOL108C	INO4
YLR119W	SRN2	YOL081W	IRA2
YBR200W	BEM1	YMR214W	SCJ1
YGL066W	SGF73	YMR199W	CLN1
YMR095C	SNO1	YER122C	GLO3
YGL163C	RAD54	YDR207C	UME6
YJR140C	HIR3	YER161C	SPT2
YMR224C	MRE11	YMR230W	RPS10B
YNL094W	APP1	YLR429W	CRN1
YMR224C	MRE11	YPL057C	SUR1
YDR443C	SSN2	YFR031C-A	RPL2A
YDR319C	YDR319C	YDL186W	YDL186W

Predictions at threshold level 0.75 continued...			
Gene1 of predicted pair		Gene2 of predicted pair	
Systematic Name	Standard Name	Systematic Name	Standard Name
YDR192C	NUP42	YER116C	SLX8
YGR072W	UPF3	YDR080W	VPS41
YPR017C	DSS4	YNL127W	FAR11
YHR060W	VMA22	YLR452C	SST2
YCL029C	BIK1	YGL190C	CDC55
YOR312C	RPL20B	YOR368W	RAD17
YNL069C	RPL16B	YPL120W	VPS30
YOR008C	SLG1	YER049W	TPA1
YPR120C	CLB5	YPL152W	RRD2
YPR051W	MAK3	YJL095W	BCK1
YLR192C	HCR1	YGR133W	PEX4
YFR030W	MET10	YJR135C	MCM22
YBR030W	YBR030W	YER044C	ERG28
YKL007W	CAP1	YOR208W	PTP2
YGL087C	MMS2	YMR038C	CCS1
YHR156C	LIN1	YBR171W	SEC66
YJL154C	VPS35	YPL037C	EGD1
YOR270C	VPH1	YPL057C	SUR1
YML117W	NAB6	YKL048C	ELM1
YBL102W	SFT2	YEL051W	VMA8
YBR290W	BSD2	YGL124C	MON1
YKL139W	CTK1	YBR200W	BEM1
YJR056C	YJR056C	YPR030W	CSR2
YNL201C	PSY2	YPR046W	MCM16
YPL161C	BEM4	YKL080W	VMA5
YOR337W	TEA1	YNL087W	TCB2
YIL053W	RHR2	YER116C	SLX8
YOR033C	EXO1	YLR313C	SPH1
YGR019W	UGA1	YDR155C	CPR1

Predictions at threshold level 0.75 continued...			
Gene1 of predicted pair		Gene2 of predicted pair	
Systematic Name	Standard Name	Systematic Name	Standard Name
YOR332W	VMA4	YBR200W	BEM1
YLR240W	VPS34	YJL053W	PEP8
YPL129W	TAF14	YGL246C	RAI1
YMR116C	ASC1	YGL212W	VAM7
YOR023C	AHC1	YDR183W	PLP1
YAL024C	LTE1	YAL002W	VPS8
YER110C	KAP123	YPL256C	CLN2
YMR224C	MRE11	YHR178W	STB5
YLR090W	XDJ1	YHR081W	LRP1
YGL066W	SGF73	YBL087C	RPL23A
YPR018W	RLF2	YLR288C	MEC3
YHR060W	VMA22	YNL051W	COG5
YNL032W	SIW14	YPR030W	CSR2
YMR055C	BUB2	YHL002W	HSE1
YDR004W	RAD57	YJR140C	HIR3
YDR171W	HSP42	YNL236W	SIN4
YDL154W	MSH5	YPL178W	CBC2
YDR080W	VPS41	YDL077C	VAM6
YHR013C	ARD1	YHR115C	DMA1
YBR200W	BEM1	YCR088W	ABP1
YLR399C	BDF1	YIL103W	DPH1
YDR080W	VPS41	YMR054W	STV1
YML038C	YMD8	YPR032W	SRO7
YIL034C	CAP2	YBR290W	BSD2
YJL053W	PEP8	YPL120W	VPS30
YHR012W	VPS29	YER122C	GLO3
YOL126C	MDH2	YCR024C-A	PMP1
YPR084W	YPR084W	YOR127W	RGA1
YDR496C	PUF6	YDR101C	ARX1

Predictions at threshold level 0.75 continued...			
**Gene1 of predicted pair**		**Gene2 of predicted pair**	
**Systematic Name**	**Standard Name**	**Systematic Name**	**Standard Name**
YOR195W	SLK19	YIL033C	BCY1
YGL147C	RPL9A	YLR119W	SRN2
YLR079W	SIC1	YHR013C	ARD1
YGL147C	RPL9A	YKL126W	YPK1
YPL037C	EGD1	YDR261C	EXG2
YBL051C	PIN4	YDR254W	CHL4
YGR249W	MGA1	YDL192W	ARF1
YJL124C	LSM1	YGR188C	BUB1
YER044C	ERG28	YGL115W	SNF4
YFL013C	IES1	YNL107W	YAF9
YDL006W	PTC1	YKR030W	GMH1
YDR318W	MCM21	YBR036C	CSG2
YBR171W	SEC66	YPR046W	MCM16
YGL078C	DBP3	YGR159C	NSR1
YDR507C	GIN4	YKR057W	RPS21A
YPL024W	RMI1	YLR452C	SST2
YDR137W	RGP1	YKR035W-A	DID2
YNL025C	SSN8	YNL107W	YAF9
YJL154C	VPS35	YLR386W	VAC14
YIL034C	CAP2	YIL008W	URM1
YBL021C	HAP3	YBR107C	IML3
YMR109W	MYO5	YGL253W	HXK2
YBR171W	SEC66	YPR119W	CLB2
YDR293C	SSD1	YGR108W	CLB1
YOL111C	MDY2	YEL053C	MAK10
YLR410W	VIP1	YJL187C	SWE1
YAL056W	GPB2	YJL106W	IME2
YEL051W	VMA8	YKL080W	VMA5
YNL291C	MID1	YLR032W	RAD5

Predictions at threshold level 0.75 continued...			
Gene1 of predicted pair		Gene2 of predicted pair	
Systematic Name	Standard Name	Systematic Name	Standard Name
YNL058C	YNL058C	YMR230W	RPS10B
YNL206C	RTT106	YBR010W	HHT1
YLL007C	YLL007C	YMR172W	HOT1
YDR293C	SSD1	YPL180W	TCO89
YNL199C	GCR2	YBR260C	RGD1
YMR199W	CLN1	YHR010W	RPL27A
YEL053C	MAK10	YNL107W	YAF9
YMR055C	BUB2	YER162C	RAD4
YER144C	UBP5	YGL115W	SNF4
YPL115C	BEM3	YGL124C	MON1
YOR043W	WHI2	YDR318W	MCM21
YNL293W	MSB3	YDR310C	SUM1
YNL215W	IES2	YDR289C	RTT103
YDR129C	SAC6	YOR036W	PEP12
YKL025C	PAN3	YJL183W	MNN11
YMR198W	CIK1	YDR171W	HSP42
YIR002C	MPH1	YGL163C	RAD54
YPL152W	RRD2	YJL187C	SWE1
YNL238W	KEX2	YOR035C	SHE4
YCR020C-A	MAK31	YNL291C	MID1
YDL160C	DHH1	YGR252W	GCN5
YDR171W	HSP42	YGL003C	CDH1
YLR058C	SHM2	YMR055C	BUB2
YPL037C	EGD1	YKL048C	ELM1
YJL004C	SYS1	YLR144C	ACF2
YLR148W	PEP3	YLR240W	VPS34
YHR039C-A	VMA10	YLR370C	ARC18
YBR017C	KAP104	YDR335W	MSN5
YMR224C	MRE11	YAL002W	VPS8

Predictions at threshold level 0.75 continued...			
Gene1 of predicted pair		Gene2 of predicted pair	
Systematic Name	Standard Name	Systematic Name	Standard Name
YDR080W	VPS41	YMR231W	PEP5
YDL116W	NUP84	YKR072C	SIS2
YFR010W	UBP6	YBR280C	SAF1
YLR320W	MMS22	YBR019C	GAL10
YDR225W	HTA1	YPL248C	GAL4
YOR033C	EXO1	YFR010W	UBP6
YIL053W	RHR2	YLR386W	VAC14
YDR293C	SSD1	YKL119C	VPH2
YHR200W	RPN10	YGL163C	RAD54
YPR046W	MCM16	YML028W	TSA1
YOR290C	SNF2	YGL115W	SNF4
YPL024W	RMI1	YLR370C	ARC18
YNL307C	MCK1	YOR327C	SNC2
YCR081W	SRB8	YNL079C	TPM1
YGR108W	CLB1	YPL018W	CTF19
YPL045W	VPS16	YDL226C	GCS1
YHR156C	LIN1	YOL111C	MDY2
YBR083W	TEC1	YDL159W	STE7
YGL115W	SNF4	YPL106C	SSE1
YBR200W	BEM1	YGR134W	CAF130
YHR200W	RPN10	YMR214W	SCJ1
YPL037C	EGD1	YNL127W	FAR11
YOR127W	RGA1	YML109W	ZDS2
YPL024W	RMI1	YOR043W	WHI2
YLR423C	ATG17	YPR185W	ATG13
YGR081C	SLX9	YGL175C	SAE2
YGL060W	YBP2	YPL022W	RAD1
YBR215W	HPC2	YKL114C	APN1
YOR043W	WHI2	YPR120C	CLB5

Predictions at threshold level 0.75 continued...			
Gene1 of predicted pair		Gene2 of predicted pair	
Systematic Name	Standard Name	Systematic Name	Standard Name
YBR217W	ATG12	YMR230W	RPS10B
YER144C	UBP5	YOR327C	SNC2
YLR094C	GIS3	YER040W	GLN3
YOR195W	SLK19	YDL047W	SIT4
YIL076W	SEC28	YGL003C	CDH1
YOR270C	VPH1	YER162C	RAD4
YDR245W	MNN10	YPL253C	VIK1
YLR320W	MMS22	YOL121C	RPS19A
YML115C	VAN1	YJL095W	BCK1
YDR108W	GSG1	YGR188C	BUB1
YOR096W	RPS7A	YDR378C	LSM6
YMR199W	CLN1	YJL095W	BCK1
YPL137C	GIP3	YOR327C	SNC2
YLR234W	TOP3	YJR084W	CSN12
YDR378C	LSM6	YKR020W	VPS51
YBR173C	UMP1	YJR056C	YJR056C
YFL023W	BUD27	YML001W	YPT7
YBR173C	UMP1	YDL184C	RPL41A
YMR142C	RPL13B	YLR441C	RPS1A
YAL042W	ERV46	YPR119W	CLB2
YDR456W	NHX1	YOR014W	RTS1
YBR108W	YBR108W	YER122C	GLO3
YPR036W	VMA13	YKR099W	BAS1
YIL103W	DPH1	YLR182W	SWI6
YDL116W	NUP84	YMR038C	CCS1
YLR079W	SIC1	YOR008C	SLG1
YGR209C	TRX2	YML028W	TSA1
YMR070W	MOT3	YDL161W	ENT1
YPL057C	SUR1	YEL013W	VAC8

Predictions at threshold level 0.75 continued...			
Gene1 of predicted pair		Gene2 of predicted pair	
Systematic Name	Standard Name	Systematic Name	Standard Name
YER161C	SPT2	YHR167W	THP2
YOR109W	INP53	YEL043W	YEL043W
YER001W	MNN1	YGL200C	EMP24
YBL102W	SFT2	YPL195W	APL5
YGL078C	DBP3	YER122C	GLO3
YHR012W	VPS29	YPR046W	MCM16
YGR097W	ASK10	YPL129W	TAF14
YBL058W	SHP1	YOR327C	SNC2
YNL309W	STB1	YMR052W	FAR3
YNL069C	RPL16B	YER114C	BOI2
YCR077C	PAT1	YNL107W	YAF9
YHR200W	RPN10	YPR120C	CLB5
YCR020C-A	MAK31	YJR040W	GEF1
YOR358W	HAP5	YJR032W	CPR7
YML038C	YMD8	YJL004C	SYS1
YGR262C	BUD32	YML056C	IMD4
YGL078C	DBP3	YDR358W	GGA1
YKL048C	ELM1	YCR086W	CSM1
YOR069W	VPS5	YNL051W	COG5
YPR046W	MCM16	YML071C	COG8
YBR030W	YBR030W	YCR053W	THR4
YIL034C	CAP2	YLR360W	VPS38
YBR173C	UMP1	YMR142C	RPL13B
YJL176C	SWI3	YOR312C	RPL20B
YDL059C	RAD59	YBL047C	EDE1
YBR108W	YBR108W	YJL190C	RPS22A
YJL124C	LSM1	YJR135C	MCM22
YPL256C	CLN2	YGR059W	SPR3
YNL016W	PUB1	YLR410W	VIP1

Predictions at threshold level 0.75 continued...			
Gene1 of predicted pair		Gene2 of predicted pair	
Systematic Name	Standard Name	Systematic Name	Standard Name
YGL095C	VPS45	YDL226C	GCS1
YEL013W	VAC8	YLR182W	SWI6
YBL008W	HIR1	YMR179W	SPT21
YHR156C	LIN1	YOL121C	RPS19A
YKL116C	PRR1	YCR053W	THR4
YLR102C	APC9	YDR260C	SWM1
YLR094C	GIS3	YER139C	YER139C
YBL021C	HAP3	YHR187W	IKI1
YLL039C	UBI4	YMR116C	ASC1
YJL154C	VPS35	YKR030W	GMH1
YGL066W	SGF73	YHR200W	RPN10
YMR142C	RPL13B	YJL095W	BCK1
YNL166C	BNI5	YJL095W	BCK1
YNL041C	COG6	YAR014C	BUD14
YPL137C	GIP3	YMR311C	GLC8
YLR337C	VRP1	YGR133W	PEX4
YGL115W	SNF4	YPL018W	CTF19
YKL007W	CAP1	YCR077C	PAT1
YHL031C	GOS1	YGL223C	COG1
YLR386W	VAC14	YER144C	UBP5
YDR245W	MNN10	YLR350W	ORM2
YKL048C	ELM1	YNL238W	KEX2
YLR119W	SRN2	YLR370C	ARC18
YLR058C	SHM2	YML095C	RAD10
YCL001W	RER1	YHR039C-A	VMA10
YGR184C	UBR1	YFR036W	CDC26
YLR058C	SHM2	YDR254W	CHL4
YHL030W	ECM29	YPR119W	CLB2
YPL253C	VIK1	YNL199C	GCR2

Predictions at threshold level 0.75 continued...			
Gene1 of predicted pair		Gene2 of predicted pair	
Systematic Name	Standard Name	Systematic Name	Standard Name
YOL051W	GAL11	YBR289W	SNF5
YOR112W	CEX1	YHL031C	GOS1
YDR443C	SSN2	YGL033W	HOP2
YLR381W	CTF3	YJR005W	APL1
YJR032W	CPR7	YDR289C	RTT103
YBL088C	TEL1	YDR253C	MET32
YNL058C	YNL058C	YLR370C	ARC18
YMR153W	NUP53	YBR171W	SEC66
YGL213C	SKI8	YLR398C	SKI2
YDR443C	SSN2	YMR026C	PEX12
YBL051C	PIN4	YPR040W	TIP41
YAL009W	SPO7	YMR165C	PAH1
YLR360W	VPS38	YNL079C	TPM1
YNL243W	SLA2	YDR245W	MNN10
YLR386W	VAC14	YLR094C	GIS3
YPL042C	SSN3	YPL106C	SSE1
YMR004W	MVP1	YDR207C	UME6
YPL057C	SUR1	YLR093C	NYV1
YLR360W	VPS38	YER049W	TPA1
YCR063W	BUD31	YJR050W	ISY1
YDR477W	SNF1	YDL223C	HBT1
YBR111W-A	SUS1	YHR167W	THP2
YMR172W	HOT1	YBR114W	RAD16
YMR202W	ERG2	YBR107C	IML3
YLR234W	TOP3	YMR198W	CIK1
YBL003C	HTA2	YGL241W	KAP114
YDR137W	RGP1	YGR105W	VMA21
YBR126C	TPS1	YGL033W	HOP2
YPL057C	SUR1	YPR046W	MCM16

Predictions at threshold level 0.75 continued...			
Gene1 of predicted pair		Gene2 of predicted pair	
Systematic Name	Standard Name	Systematic Name	Standard Name
YMR109W	MYO5	YGR270W	YTA7
YBL051C	PIN4	YMR109W	MYO5
YMR055C	BUB2	YML028W	TSA1
YBR112C	CYC8	YHL027W	RIM101
YJL137C	GLG2	YLR273C	PIG1
YMR273C	ZDS1	YGR059W	SPR3
YPL115C	BEM3	YGL038C	OCH1
YHL031C	GOS1	YEL053C	MAK10
YDR108W	GSG1	YNR032W	PPG1
YKL116C	PRR1	YJL183W	MNN11
YDR225W	HTA1	YCL010C	SGF29
YCL037C	SRO9	YER161C	SPT2
YOL090W	MSH2	YMR038C	CCS1
YDR254W	CHL4	YJL095W	BCK1
YKR072C	SIS2	YBR036C	CSG2
YOR195W	SLK19	YPL018W	CTF19
YML112W	CTK3	YHR115C	DMA1
YLR452C	SST2	YOR035C	SHE4
YFL047W	RGD2	YGR059W	SPR3
YGR239C	PEX21	YAR042W	SWH1
YJL124C	LSM1	YMR038C	CCS1
YPR084W	YPR084W	YHR012W	VPS29
YDR363W-A	SEM1	YNL229C	URE2
YBR107C	IML3	YDL013W	SLX5
YGR105W	VMA21	YOL018C	TLG2
YIL034C	CAP2	YHR114W	BZZ1
YNR052C	POP2	YGL175C	SAE2
YMR224C	MRE11	YGR184C	UBR1
YMR116C	ASC1	YLR320W	MMS22

Predictions at threshold level 0.75 continued...			
Gene1 of predicted pair		Gene2 of predicted pair	
Systematic Name	Standard Name	Systematic Name	Standard Name
YER116C	SLX8	YBR280C	SAF1
YHR156C	LIN1	YMR311C	GLC8
YER019C-A	SBH2	YGL226C-A	OST5
YGR081C	SLX9	YOR368W	RAD17
YDR004W	RAD57	YNL291C	MID1
YCR086W	CSM1	YDR207C	UME6
YKL126W	YPK1	YNL307C	MCK1
YBL051C	PIN4	YHR115C	DMA1
YIL034C	CAP2	YMR127C	SAS2
YDR318W	MCM21	YOL054W	PSH1
YDL116W	NUP84	YLR410W	VIP1
YPR017C	DSS4	YGL054C	ERV14
YPL024W	RMI1	YJR032W	CPR7
YGR261C	APL6	YJL004C	SYS1
YHR081W	LRP1	YJL183W	MNN11
YPL050C	MNN9	YPR154W	PIN3
YDR137W	RGP1	YGR270W	YTA7
YNL215W	IES2	YER122C	GLO3
YOR066W	MSA1	YDR310C	SUM1
YBR107C	IML3	YOR358W	HAP5
YIR005W	IST3	YHR115C	DMA1
YLR192C	HCR1	YMR133W	REC114
YNL054W	VAC7	YIR005W	IST3
YOR290C	SNF2	YCR053W	THR4
YML115C	VAN1	YGR188C	BUB1
YBL017C	PEP1	YHR108W	GGA2
YBR200W	BEM1	YPL195W	APL5
YBR073W	RDH54	YDR254W	CHL4
YHR013C	ARD1	YOL054W	PSH1

Predictions at threshold level 0.75 continued...			
Gene1 of predicted pair		Gene2 of predicted pair	
Systematic Name	Standard Name	Systematic Name	Standard Name
YKR035W-A	DID2	YOR327C	SNC2
YBR131W	CCZ1	YMR038C	CCS1
YNL087W	TCB2	YDL077C	VAM6
YML001W	YPT7	YOL018C	TLG2
YDL013W	SLX5	YJR135C	MCM22
YBR171W	SEC66	YJL095W	BCK1
YDR443C	SSN2	YAL024C	LTE1
YNL058C	YNL058C	YGL003C	CDH1
YHR060W	VMA22	YER122C	GLO3
YDL116W	NUP84	YLR079W	SIC1
YIL034C	CAP2	YDR144C	MKC7
YNL218W	MGS1	YMR038C	CCS1
YOR332W	VMA4	YPR119W	CLB2
YPR051W	MAK3	YBR107C	IML3
YJL172W	CPS1	YJL095W	BCK1
YOR112W	CEX1	YNR032W	PPG1
YJR031C	GEA1	YER122C	GLO3
YOR327C	SNC2	YBR280C	SAF1
YMR224C	MRE11	YML115C	VAN1
YJL029C	VPS53	YDL047W	SIT4
YGR108W	CLB1	YNL107W	YAF9
YLR248W	RCK2	YPR119W	CLB2
YGR249W	MGA1	YBR200W	BEM1
YKR001C	VPS1	YJR033C	RAV1
YLR371W	ROM2	YBR036C	CSG2
YLR381W	CTF3	YPL253C	VIK1
YPL057C	SUR1	YNL107W	YAF9
YGL215W	CLG1	YDR358W	GGA1
YMR198W	CIK1	YHR013C	ARD1

Predictions at threshold level 0.75 continued...			
Gene1 of predicted pair		Gene2 of predicted pair	
Systematic Name	Standard Name	Systematic Name	Standard Name
YML115C	VAN1	YJR056C	YJR056C
YNL087W	TCB2	YPL037C	EGD1
YOR275C	RIM20	YPL256C	CLN2
YOR132W	VPS17	YML038C	YMD8
YBR108W	YBR108W	YMR055C	BUB2
YPL161C	BEM4	YLR182W	SWI6
YOL126C	MDH2	YKL068W	NUP100
YDR383C	NKP1	YGR188C	BUB1
YGL147C	RPL9A	YMR095C	SNO1
YKR072C	SIS2	YDL059C	RAD59
YOR337W	TEA1	YER040W	GLN3
YPL195W	APL5	YBR019C	GAL10
YDR245W	MNN10	YCL037C	SRO9
YPL161C	BEM4	YOR014W	RTS1
YDR443C	SSN2	YEL060C	PRB1
YHR156C	LIN1	YPR036W	VMA13
YDL116W	NUP84	YMR080C	NAM7
YDR254W	CHL4	YOR014W	RTS1
YKL181W	PRS1	YHR030C	SLT2
YMR179W	SPT21	YDL155W	CLB3
YJL154C	VPS35	YDR207C	UME6
YNL236W	SIN4	YPR119W	CLB2
YOR106W	VAM3	YDR440W	DOT1
YLR102C	APC9	YGR135W	PRE9
YGR081C	SLX9	YDR207C	UME6
YIL159W	BNR1	YHR081W	LRP1
YER162C	RAD4	YOL018C	TLG2
YPL129W	TAF14	YMR201C	RAD14
YNR052C	POP2	YKL048C	ELM1

Predictions at threshold level 0.75 continued...			
Gene1 of predicted pair		Gene2 of predicted pair	
Systematic Name	Standard Name	Systematic Name	Standard Name
YBR194W	SOY1	YIR005W	IST3
YHR134W	WSS1	YNR052C	POP2
YPL001W	HAT1	YJL124C	LSM1
YFR010W	UBP6	YJL124C	LSM1
YBL047C	EDE1	YDL006W	PTC1
YNL209W	SSB2	YPL240C	HSP82
YNL106C	INP52	YLR254C	NDL1
YDR378C	LSM6	YBL047C	EDE1
YLR381W	CTF3	YLR254C	NDL1
YDR477W	SNF1	YER040W	GLN3
YJL127C	SPT10	YDR318W	MCM21
YJL172W	CPS1	YMR133W	REC114
YDR254W	CHL4	YJR005W	APL1
YBR274W	CHK1	YCR077C	PAT1
YAL024C	LTE1	YPL256C	CLN2
YLR090W	XDJ1	YHR156C	LIN1
YDR378C	LSM6	YER122C	GLO3
YDR443C	SSN2	YMR133W	REC114
YGL033W	HOP2	YPL057C	SUR1
YNL218W	MGS1	YJL124C	LSM1
YJL176C	SWI3	YJL095W	BCK1
YJR056C	YJR056C	YOR327C	SNC2
YGR072W	UPF3	YPL057C	SUR1
YBL102W	SFT2	YJL053W	PEP8
YLR320W	MMS22	YNL238W	KEX2
YGL133W	ITC1	YDR264C	AKR1
YER110C	KAP123	YML057W	CMP2
YLR386W	VAC14	YMR311C	GLC8
YMR153W	NUP53	YHR039C-A	VMA10

Predictions at threshold level 0.75 continued...			
Gene1 of predicted pair		Gene2 of predicted pair	
Systematic Name	Standard Name	Systematic Name	Standard Name
YBR009C	HHF1	YKR048C	NAP1
YKL007W	CAP1	YER114C	BOI2
YDR156W	RPA14	YLR381W	CTF3
YNL291C	MID1	YER122C	GLO3
YDL155W	CLB3	YMR022W	UBC7
YBL102W	SFT2	YHR060W	VMA22
YJL172W	CPS1	YKR099W	BAS1
YJR092W	BUD4	YPR122W	AXL1
YEL065W	SIT1	YNR072W	HXT17
YJL172W	CPS1	YBL025W	RRN10
YOL054W	PSH1	YNL225C	CNM67
YBR057C	MUM2	YEL044W	IES6
YNL297C	MON2	YHR039C-A	VMA10
YGL147C	RPL9A	YBR019C	GAL10
YEL009C	GCN4	YDR448W	ADA2
YLR381W	CTF3	YHR012W	VPS29
YGR261C	APL6	YLR093C	NYV1
YER143W	DDI1	YPR032W	SRO7
YOR270C	VPH1	YLR441C	RPS1A
YNL044W	YIP3	YJL029C	VPS53
YDR254W	CHL4	YLR315W	NKP2
YGL115W	SNF4	YNR032W	PPG1
YFR031C-A	RPL2A	YNL051W	COG5
YKL116C	PRR1	YLR370C	ARC18
YOR106W	VAM3	YDR202C	RAV2
YGL200C	EMP24	YPL213W	LEA1
YDL155W	CLB3	YOR141C	ARP8
YNL106C	INP52	YPL079W	RPL21B
YGL175C	SAE2	YJL187C	SWE1

Predictions at threshold level 0.75 continued...			
Gene1 of predicted pair		Gene2 of predicted pair	
Systematic Name	Standard Name	Systematic Name	Standard Name
YHL031C	GOS1	YKL213C	DOA1
YDR443C	SSN2	YHR081W	LRP1
YGR054W	YGR054W	YDL155W	CLB3
YJL140W	RPB4	YOR064C	YNG1
YDR309C	GIC2	YJL095W	BCK1
YOR127W	RGA1	YPL018W	CTF19
YDR439W	LRS4	YNL225C	CNM67
YLR337C	VRP1	YML097C	VPS9
YKL129C	MYO3	YHR061C	GIC1
YDL184C	RPL41A	YKR035W-A	DID2
YDR176W	NGG1	YKR048C	NAP1
YER116C	SLX8	YPR046W	MCM16
YAL024C	LTE1	YEL056W	HAT2
YIL053W	RHR2	YOR270C	VPH1
YEL009C	GCN4	YOL148C	SPT20
YAL024C	LTE1	YLR119W	SRN2
YLR360W	VPS38	YDR477W	SNF1
YMR202W	ERG2	YJR060W	CBF1
YOR270C	VPH1	YEL027W	CUP5
YMR224C	MRE11	YGL175C	SAE2
YMR127C	SAS2	YJL095W	BCK1
YKR001C	VPS1	YDL226C	GCS1
YML115C	VAN1	YDL184C	RPL41A
YDR335W	MSN5	YPL161C	BEM4
YMR205C	PFK2	YBL101C	ECM21
YHR039C-A	VMA10	YDR261C	EXG2
YJL176C	SWI3	YNL329C	PEX6
YFR010W	UBP6	YPL248C	GAL4
YJR063W	RPA12	YBR009C	HHF1

Predictions at threshold level 0.75 continued...			
Gene1 of predicted pair		Gene2 of predicted pair	
Systematic Name	Standard Name	Systematic Name	Standard Name
YNL087W	TCB2	YER139C	YER139C
YDL155W	CLB3	YNL079C	TPM1
YGL147C	RPL9A	YNL032W	SIW14
YLR386W	VAC14	YMR133W	REC114
YLR447C	VMA6	YNL238W	KEX2
YEL059C-A	SOM1	YMR035W	IMP2
YDL020C	RPN4	YOR023C	AHC1
YGL078C	DBP3	YPL057C	SUR1
YLR079W	SIC1	YMR273C	ZDS1
YDL013W	SLX5	YPL018W	CTF19
YBR200W	BEM1	YKL032C	IXR1
YML016C	PPZ1	YDL155W	CLB3
YKL126W	YPK1	YLR093C	NYV1
YBR171W	SEC66	YLR093C	NYV1
YLR447C	VMA6	YDR144C	MKC7
YDR245W	MNN10	YOR367W	SCP1
YLR399C	BDF1	YNL116W	DMA2
YDR245W	MNN10	YBL067C	UBP13
YDR071C	PAA1	YER068W	MOT2
YPL022W	RAD1	YJR090C	GRR1
YDR174W	HMO1	YBR073W	RDH54
YFR010W	UBP6	YMR167W	MLH1
YJL060W	BNA3	YJR135C	MCM22
YDL125C	HNT1	YDL047W	SIT4
YDR363W-A	SEM1	YGR232W	NAS6
YMR224C	MRE11	YGR275W	RTT102
YMR216C	SKY1	YPL120W	VPS30
YBL058W	SHP1	YPL195W	APL5
YIL159W	BNR1	YLR093C	NYV1

Predictions at threshold level 0.75 continued...			
Gene1 of predicted pair		Gene2 of predicted pair	
Systematic Name	Standard Name	Systematic Name	Standard Name
YLR337C	VRP1	YGL212W	VAM7
YHR200W	RPN10	YER162C	RAD4
YKL048C	ELM1	YMR311C	GLC8
YBR171W	SEC66	YMR214W	SCJ1
YBL103C	RTG3	YFR036W	CDC26
YKL160W	ELF1	YGR135W	PRE9
YGL078C	DBP3	YGL033W	HOP2
YKL025C	PAN3	YBR274W	CHK1
YLR018C	POM34	YGL016W	KAP122
YJR060W	CBF1	YPR046W	MCM16
YER161C	SPT2	YLR315W	NKP2
YOR132W	VPS17	YPR046W	MCM16
YDL223C	HBT1	YFR036W	CDC26
YMR095C	SNO1	YPR030W	CSR2
YLR095C	IOC2	YMR142C	RPL13B
YJL183W	MNN11	YER122C	GLO3
YBR173C	UMP1	YDR335W	MSN5
YIL076W	SEC28	YOR069W	VPS5
YDR477W	SNF1	YBR108W	YBR108W
YIL076W	SEC28	YLR309C	IMH1
YPL152W	RRD2	YOL081W	IRA2
YDR443C	SSN2	YLR093C	NYV1
YLR441C	RPS1A	YGL003C	CDH1
YNL058C	YNL058C	YML001W	YPT7
YBR133C	HSL7	YJL187C	SWE1
YLR192C	HCR1	YGL115W	SNF4
YGR184C	UBR1	YJR135C	MCM22
YLR337C	VRP1	YKL032C	IXR1
YKR072C	SIS2	YBR278W	DPB3

Predictions at threshold level 0.75 continued...			
Gene1 of predicted pair		Gene2 of predicted pair	
Systematic Name	Standard Name	Systematic Name	Standard Name
YJL127C	SPT10	YDR335W	MSN5
YOR038C	HIR2	YFR036W	CDC26
YML016C	PPZ1	YIL103W	DPH1
YNL041C	COG6	YMR311C	GLC8
YOR083W	WHI5	YOR195W	SLK19
YNL032W	SIW14	YER040W	GLN3
YDR320C	SWA2	YNL238W	KEX2
YNL229C	URE2	YJL095W	BCK1
YKL116C	PRR1	YGL124C	MON1
YNL264C	PDR17	YGL200C	EMP24
YJR056C	YJR056C	YEL060C	PRB1
YJL053W	PEP8	YHL020C	OPI1
YLR206W	ENT2	YJR125C	ENT3
YNL236W	SIN4	YGL094C	PAN2
YOR270C	VPH1	YMR054W	STV1
YNR052C	POP2	YNL248C	RPA49
YPR051W	MAK3	YAL002W	VPS8
YDL006W	PTC1	YDL077C	VAM6
YBR128C	ATG14	YPL120W	VPS30
YLR441C	RPS1A	YBR036C	CSG2
YCR020C-A	MAK31	YPR051W	MAK3
YDL232W	OST4	YIL162W	SUC2
YML103C	NUP188	YBR200W	BEM1
YPL161C	BEM4	YDL240W	LRG1
YGL200C	EMP24	YLR425W	TUS1
YPL050C	MNN9	YGL200C	EMP24
YDR174W	HMO1	YHR111W	UBA4
YEL043W	YEL043W	YAR014C	BUD14
YPL161C	BEM4	YOR327C	SNC2

Predictions at threshold level 0.75 continued...			
Gene1 of predicted pair		Gene2 of predicted pair	
Systematic Name	Standard Name	Systematic Name	Standard Name
YBR173C	UMP1	YPL253C	VIK1
YHR108W	GGA2	YHL011C	PRS3
YLR337C	VRP1	YGL135W	RPL1B
YKL139W	CTK1	YIR005W	IST3
YHR012W	VPS29	YMR311C	GLC8
YOR195W	SLK19	YLR410W	VIP1
YDL160C	DHH1	YCR077C	PAT1
YDR103W	STE5	YBR107C	IML3
YBR173C	UMP1	YER116C	SLX8
YBR274W	CHK1	YBR036C	CSG2
YDL020C	RPN4	YER110C	KAP123
YML062C	MFT1	YHR081W	LRP1
YMR026C	PEX12	YDR261C	EXG2
YOR195W	SLK19	YDR171W	HSP42
YFR036W	CDC26	YLR182W	SWI6
YPL018W	CTF19	YJR090C	GRR1
YAL024C	LTE1	YNL106C	INP52
YPL161C	BEM4	YJR060W	CBF1
YMR194W	RPL36A	YGL246C	RAI1
YPL096C-A	ERI1	YOR312C	RPL20B
YMR026C	PEX12	YFR010W	UBP6
YGL087C	MMS2	YDR183W	PLP1
YLR441C	RPS1A	YKR035W-A	DID2
YMR109W	MYO5	YJL190C	RPS22A
YMR224C	MRE11	YNL238W	KEX2
YLR381W	CTF3	YOR014W	RTS1
YCL001W	RER1	YJL004C	SYS1
YDR080W	VPS41	YML001W	YPT7
YBR107C	IML3	YML028W	TSA1

Predictions at threshold level 0.75 continued...			
Gene1 of predicted pair		Gene2 of predicted pair	
Systematic Name	Standard Name	Systematic Name	Standard Name
YGR019W	UGA1	YNL107W	YAF9
YFL013C	IES1	YMR038C	CCS1
YNL135C	FPR1	YNL023C	FAP1
YKL117W	SBA1	YKL068W	NUP100
YKR001C	VPS1	YDR289C	RTT103
YFL033C	RIM15	YLR309C	IMH1
YDL232W	OST4	YEL036C	ANP1
YMR016C	SOK2	YDL083C	RPS16B
YBR171W	SEC66	YGR270W	YTA7
YPL018W	CTF19	YGL253W	HXK2
YHR134W	WSS1	YHR077C	NMD2
YDR108W	GSG1	YKR030W	GMH1
YHR039C-A	VMA10	YOL108C	INO4
YBR030W	YBR030W	YOL081W	IRA2
YLR216C	CPR6	YKL117W	SBA1
YLL039C	UBI4	YDL137W	ARF2
YML115C	VAN1	YBR171W	SEC66
YOR096W	RPS7A	YPL081W	RPS9A
YDR137W	RGP1	YML001W	YPT7
YDR176W	NGG1	YDR469W	SDC1
YKL135C	APL2	YOR036W	PEP12
YBR111W-A	SUS1	YOR001W	RRP6
YBR126C	TPS1	YPL037C	EGD1
YML038C	YMD8	YPL234C	TFP3
YBR171W	SEC66	YKL161C	YKL161C
YCR065W	HCM1	YOR308C	SNU66
YHR012W	VPS29	YBR114W	RAD16
YOR001W	RRP6	YOR141C	ARP8
YBR290W	BSD2	YMR127C	SAS2

Predictions at threshold level 0.75 continued...			
**Gene1 of predicted pair**		**Gene2 of predicted pair**	
**Systematic Name**	**Standard Name**	**Systematic Name**	**Standard Name**
YIL036W	CST6	YIR005W	IST3
YJR060W	CBF1	YJR033C	RAV1
YGL223C	COG1	YAL002W	VPS8
YJL176C	SWI3	YLR131C	ACE2
YGL060W	YBP2	YPL018W	CTF19
YER120W	SCS2	YNL051W	COG5
YOR112W	CEX1	YHR039C-A	VMA10
YBR030W	YBR030W	YLR320W	MMS22
YLR094C	GIS3	YLR370C	ARC18
YLR079W	SIC1	YOR368W	RAD17
YHR060W	VMA22	YLR309C	IMH1
YGL066W	SGF73	YKR048C	NAP1
YBR200W	BEM1	YOL121C	RPS19A
YBR030W	YBR030W	YGL124C	MON1
YBR030W	YBR030W	YOR270C	VPH1
YDL116W	NUP84	YBL079W	NUP170
YIL076W	SEC28	YER139C	YER139C
YMR198W	CIK1	YER114C	BOI2
YLR095C	IOC2	YMR230W	RPS10B
YGL147C	RPL9A	YMR016C	SOK2
YOR337W	TEA1	YOL081W	IRA2
YOR127W	RGA1	YNL229C	URE2
YDL116W	NUP84	YFL047W	RGD2
YDR477W	SNF1	YLR371W	ROM2
YLR452C	SST2	YDR261C	EXG2
YLR386W	VAC14	YGL019W	CKB1
YIL005W	EPS1	YPL106C	SSE1
YHR134W	WSS1	YMR038C	CCS1
YGR261C	APL6	YBL078C	ATG8

Predictions at threshold level 0.75 continued...			
Gene1 of predicted pair		Gene2 of predicted pair	
Systematic Name	Standard Name	Systematic Name	Standard Name
YDR195W	REF2	YER032W	FIR1
YCR088W	ABP1	YDR323C	PEP7
YBL058W	SHP1	YPL137C	GIP3
YLR360W	VPS38	YGL223C	COG1
YLR432W	IMD3	YML056C	IMD4
YGL035C	MIG1	YPR119W	CLB2
YGR108W	CLB1	YCR086W	CSM1
YPL057C	SUR1	YPL037C	EGD1
YMR198W	CIK1	YJL183W	MNN11
YOR083W	WHI5	YDL226C	GCS1
YDL020C	RPN4	YGR104C	SRB5
YML062C	MFT1	YER063W	THO1
YPL253C	VIK1	YNL307C	MCK1
YLR192C	HCR1	YDL192W	ARF1
YKL094W	YJU3	YKL140W	TGL1
YMR055C	BUB2	YLR447C	VMA6
YPL042C	SSN3	YDR477W	SNF1
YPL037C	EGD1	YHL020C	OPI1
YDL192W	ARF1	YNL076W	MKS1
YLR192C	HCR1	YBL047C	EDE1
YLR292C	SEC72	YKL048C	ELM1
YLL026W	HSP104	YGR275W	RTT102
YML071C	COG8	YOR327C	SNC2
YJR058C	APS2	YPL259C	APM1
YNL041C	COG6	YOL121C	RPS19A
YDR080W	VPS41	YDL192W	ARF1
YOR191W	RIS1	YGR071C	YGR071C
YOL018C	TLG2	YAR014C	BUD14
YBR195C	MSI1	YFR036W	CDC26

Predictions at threshold level 0.75 continued...			
Gene1 of predicted pair		Gene2 of predicted pair	
Systematic Name	Standard Name	Systematic Name	Standard Name
YBR057C	MUM2	YNL079C	TPM1
YPL024W	RMI1	YDL059C	RAD59
YMR127C	SAS2	YOR213C	SAS5
YMR026C	PEX12	YJL183W	MNN11
YKR072C	SIS2	YPR120C	CLB5
YBL106C	SRO77	YOR327C	SNC2
YAL010C	MDM10	YLR315W	NKP2
YDR363W-A	SEM1	YDL047W	SIT4
YCR065W	HCM1	YER096W	SHC1
YGL066W	SGF73	YLR234W	TOP3
YDR335W	MSN5	YKL048C	ELM1
YDL116W	NUP84	YDR289C	RTT103
YNL325C	FIG4	YER040W	GLN3
YIL053W	RHR2	YIL159W	BNR1
YIL148W	RPL40A	YPL178W	CBC2
YER161C	SPT2	YLR182W	SWI6
YBL103C	RTG3	YBR036C	CSG2
YDR439W	LRS4	YKR061W	KTR2
YDL006W	PTC1	YNR032W	PPG1
YDL184C	RPL41A	YLR320W	MMS22
YGR072W	UPF3	YHL031C	GOS1
YML095C	RAD10	YHR115C	DMA1
YLR452C	SST2	YPL018W	CTF19
YMR153W	NUP53	YNL199C	GCR2
YGL078C	DBP3	YOL081W	IRA2
YNR052C	POP2	YOL081W	IRA2
YDR101C	ARX1	YOR312C	RPL20B
YJR059W	PTK2	YML057W	CMP2
YLR386W	VAC14	YNL116W	DMA2

Predictions at threshold level 0.75 continued...			
Gene1 of predicted pair		Gene2 of predicted pair	
Systematic Name	Standard Name	Systematic Name	Standard Name
YOR275C	RIM20	YDL155W	CLB3
YOL091W	SPO21	YDL226C	GCS1
YNR052C	POP2	YPR046W	MCM16
YLR386W	VAC14	YNL238W	KEX2
YPL042C	SSN3	YLR079W	SIC1
YPR046W	MCM16	YPL195W	APL5
YDR378C	LSM6	YDL155W	CLB3
YGL161C	YIP5	YPR119W	CLB2
YBR017C	KAP104	YBL079W	NUP170
YJL095W	BCK1	YGR188C	BUB1
YGL237C	HAP2	YGL003C	CDH1
YHR178W	STB5	YDR144C	MKC7
YDR108W	GSG1	YDR469W	SDC1
YLR192C	HCR1	YDR207C	UME6
YDL020C	RPN4	YDR076W	RAD55
YKL139W	CTK1	YNL236W	SIN4
YPR036W	VMA13	YKL080W	VMA5
YLR094C	GIS3	YML059C	NTE1
YDR097C	MSH6	YOR368W	RAD17
YBR107C	IML3	YDR289C	RTT103
YLR093C	NYV1	YMR152W	YIM1
YOR358W	HAP5	YDR254W	CHL4
YNL229C	URE2	YHR115C	DMA1
YML012W	ERV25	YNL041C	COG6
YBR108W	YBR108W	YPR046W	MCM16
YDR261C	EXG2	YMR152W	YIM1
YIL159W	BNR1	YER162C	RAD4
YDL047W	SIT4	YNL116W	DMA2
YHR079C	IRE1	YOR036W	PEP12

Predictions at threshold level 0.75 continued...			
Gene1 of predicted pair		Gene2 of predicted pair	
Systematic Name	Standard Name	Systematic Name	Standard Name
YFL033C	RIM15	YNL238W	KEX2
YML103C	NUP188	YLR386W	VAC14
YDR108W	GSG1	YNL051W	COG5
YOR358W	HAP5	YJL183W	MNN11
YKR099W	BAS1	YFR036W	CDC26
YMR224C	MRE11	YNL107W	YAF9
YKL006W	RPL14A	YLR425W	TUS1
YNL291C	MID1	YGR105W	VMA21
YMR016C	SOK2	YNL307C	MCK1
YNL201C	PSY2	YPL253C	VIK1
YDL101C	DUN1	YMR224C	MRE11
YJL176C	SWI3	YPL129W	TAF14
YER044C	ERG28	YER139C	YER139C
YGL078C	DBP3	YNL087W	TCB2
YHR205W	SCH9	YER111C	SWI4
YLR043C	TRX1	YML028W	TSA1
YLR234W	TOP3	YCR086W	CSM1
YDR004W	RAD57	YAL024C	LTE1
YKL139W	CTK1	YJR032W	CPR7
YFR010W	UBP6	YLR094C	GIS3
YLR452C	SST2	YHR039C-A	VMA10
YJL187C	SWE1	YGL019W	CKB1
YJL172W	CPS1	YER122C	GLO3
YMR095C	SNO1	YLR398C	SKI2
YML115C	VAN1	YDR484W	VPS52
YJR060W	CBF1	YAL002W	VPS8
YDR358W	GGA1	YGL124C	MON1
YBL103C	RTG3	YOR327C	SNC2
YLR441C	RPS1A	YER040W	GLN3

Predictions at threshold level 0.75 continued...			
Gene1 of predicted pair		Gene2 of predicted pair	
Systematic Name	Standard Name	Systematic Name	Standard Name
YNL069C	RPL16B	YJL053W	PEP8
YFL023W	BUD27	YOL081W	IRA2
YPL161C	BEM4	YML038C	YMD8
YBR173C	UMP1	YLR180W	SAM1
YDL184C	RPL41A	YDR137W	RGP1
YNL058C	YNL058C	YLR093C	NYV1
YGR261C	APL6	YOR069W	VPS5
YMR224C	MRE11	YBL025W	RRN10
YLR095C	IOC2	YNL325C	FIG4
YBR217W	ATG12	YNR007C	ATG3
YDR137W	RGP1	YGL003C	CDH1
YGL115W	SNF4	YHR012W	VPS29
YDR443C	SSN2	YOR140W	SFL1
YIL053W	RHR2	YPR046W	MCM16
YER116C	SLX8	YMR199W	CLN1
YOR001W	RRP6	YBR010W	HHT1
YMR142C	RPL13B	YPR046W	MCM16
YHR081W	LRP1	YBR280C	SAF1
YBR030W	YBR030W	YHR200W	RPN10
YJL176C	SWI3	YLR370C	ARC18
YJR056C	YJR056C	YJR084W	CSN12
YNL025C	SSN8	YLR315W	NKP2
YLR043C	TRX1	YPL195W	APL5
YER059W	PCL6	YOR028C	CIN5
YKL048C	ELM1	YMR152W	YIM1
YKR001C	VPS1	YNL238W	KEX2
YMR142C	RPL13B	YKL126W	YPK1
YIL156W	UBP7	YMR199W	CLN1
YPL057C	SUR1	YER139C	YER139C

Predictions at threshold level 0.75 continued...			
Gene1 of predicted pair		Gene2 of predicted pair	
Systematic Name	Standard Name	Systematic Name	Standard Name
YMR026C	PEX12	YDR358W	GGA1
YMR205C	PFK2	YJL095W	BCK1
YJL176C	SWI3	YLR192C	HCR1
YMR224C	MRE11	YDR477W	SNF1
YOR112W	CEX1	YDR080W	VPS41
YML103C	NUP188	YNL248C	RPA49
YDR318W	MCM21	YLR254C	NDL1
YLR396C	VPS33	YDL226C	GCS1
YPL057C	SUR1	YLR094C	GIS3
YLR360W	VPS38	YLR309C	IMH1
YDR202C	RAV2	YOR109W	INP53
YKL139W	CTK1	YNL248C	RPA49
YMR224C	MRE11	YMR026C	PEX12
YML095C	RAD10	YPL018W	CTF19
YDR335W	MSN5	YNL107W	YAF9
YMR199W	CLN1	YBR260C	RGD1
YNL218W	MGS1	YER116C	SLX8
YML016C	PPZ1	YKL161C	YKL161C
YEL022W	GEA2	YDL226C	GCS1
YLR452C	SST2	YLR371W	ROM2
YAL024C	LTE1	YKL116C	PRR1
YML097C	VPS9	YAL002W	VPS8
YNL025C	SSN8	YGR270W	YTA7
YGR159C	NSR1	YDR245W	MNN10
YJR056C	YJR056C	YLR371W	ROM2
YKL101W	HSL1	YNL116W	DMA2
YNL087W	TCB2	YPL057C	SUR1
YMR109W	MYO5	YDR318W	MCM21
YDR378C	LSM6	YLR315W	NKP2

Predictions at threshold level 0.75 continued...			
Gene1 of predicted pair		Gene2 of predicted pair	
Systematic Name	Standard Name	Systematic Name	Standard Name
YKL117W	SBA1	YMR125W	STO1
YBL072C	RPS8A	YKL041W	VPS24
YOR078W	BUD21	YHR203C	RPS4B
YNL288W	CAF40	YHR114W	BZZ1
YBR173C	UMP1	YMR152W	YIM1
YNL068C	FKH2	YLR102C	APC9
YBR173C	UMP1	YMR038C	CCS1
YJR140C	HIR3	YMR038C	CCS1
YIR005W	IST3	YOR308C	SNU66
YHL031C	GOS1	YER122C	GLO3
YDR129C	SAC6	YBL067C	UBP13
YOR078W	BUD21	YGR100W	MDR1
YLR090W	XDJ1	YLR094C	GIS3
YHL020C	OPI1	YGR105W	VMA21
YOR112W	CEX1	YEL051W	VMA8
YBR200W	BEM1	YKR099W	BAS1
YLR090W	XDJ1	YBR108W	YBR108W
YLR079W	SIC1	YDR456W	NHX1
YGL078C	DBP3	YLR094C	GIS3
YNL325C	FIG4	YER139C	YER139C
YDR264C	AKR1	YKL178C	STE3
YPL024W	RMI1	YGR184C	UBR1
YGL200C	EMP24	YCR077C	PAT1
YKL139W	CTK1	YBR171W	SEC66
YOR195W	SLK19	YKL110C	KTI12
YDR293C	SSD1	YLR120C	YPS1
YER116C	SLX8	YBR107C	IML3
YNL215W	IES2	YOR308C	SNU66
YNL214W	PEX17	YGR077C	PEX8

Predictions at threshold level 0.75 continued...			
Gene1 of predicted pair		Gene2 of predicted pair	
Systematic Name	Standard Name	Systematic Name	Standard Name
YDR293C	SSD1	YAR014C	BUD14
YMR142C	RPL13B	YMR230W	RPS10B
YBR041W	FAT1	YKR020W	VPS51
YDR293C	SSD1	YGL223C	COG1
YLR192C	HCR1	YBR200W	BEM1
YLR095C	IOC2	YOL111C	MDY2
YFR031C-A	RPL2A	YGR105W	VMA21
YEL065W	SIT1	YPL024W	RMI1
YJR056C	YJR056C	YER139C	YER139C
YJR032W	CPR7	YPL018W	CTF19
YBR009C	HHF1	YJL140W	RPB4
YKL139W	CTK1	YER044C	ERG28
YOR178C	GAC1	YLR258W	GSY2
YDR097C	MSH6	YNL291C	MID1
YEL015W	EDC3	YDR378C	LSM6
YEL013W	VAC8	YGR262C	BUD32
YGL133W	ITC1	YKR048C	NAP1
YGR249W	MGA1	YHR039C-A	VMA10
YCR077C	PAT1	YGL031C	RPL24A
YML016C	PPZ1	YJL138C	TIF2
YCL037C	SRO9	YDR153C	ENT5
YBL025W	RRN10	YDL192W	ARF1
YNL307C	MCK1	YDL155W	CLB3
YGL066W	SGF73	YDR507C	GIN4
YLR386W	VAC14	YML016C	PPZ1
YER161C	SPT2	YEL056W	HAT2
YHR084W	STE12	YPL042C	SSN3
YDL184C	RPL41A	YLR452C	SST2
YHR161C	YAP1801	YOR036W	PEP12

Predictions at threshold level 0.75 continued...			
Gene1 of predicted pair		Gene2 of predicted pair	
Systematic Name	Standard Name	Systematic Name	Standard Name
YEL065W	SIT1	YIL159W	BNR1
YDL020C	RPN4	YJR090C	GRR1
YDL116W	NUP84	YDR318W	MCM21
YLR435W	TSR2	YGL250W	RMR1
YNL106C	INP52	YPL057C	SUR1
YGR100W	MDR1	YJL029C	VPS53
YOR109W	INP53	YHR012W	VPS29
YNL106C	INP52	YLR410W	VIP1
YGL124C	MON1	YBR280C	SAF1
YFR031C-A	RPL2A	YDR207C	UME6
YML100W	TSL1	YMR261C	TPS3
YLR109W	AHP1	YHR111W	UBA4
YCR020C-A	MAK31	YNL069C	RPL16B
YEL009C	GCN4	YKL109W	HAP4
YAL010C	MDM10	YHR111W	UBA4
YBL003C	HTA2	YNL031C	HHT2
YNL206C	RTT106	YBR084C-A	RPL19A
YPL234C	TFP3	YAL002W	VPS8
YLR234W	TOP3	YLR150W	STM1
YNL215W	IES2	YCR086W	CSM1
YJL124C	LSM1	YGL003C	CDH1
YMR199W	CLN1	YKL117W	SBA1
YDR439W	LRS4	YNL107W	YAF9
YNR072W	HXT17	YPR030W	CSR2
YBR200W	BEM1	YLR410W	VIP1
YPL024W	RMI1	YDR146C	SWI5
YOR195W	SLK19	YML112W	CTK3
YNL032W	SIW14	YEL060C	PRB1
YOR375C	GDH1	YFR036W	CDC26

Predictions at threshold level 0.75 continued...			
Gene1 of predicted pair		Gene2 of predicted pair	
Systematic Name	Standard Name	Systematic Name	Standard Name
YFL023W	BUD27	YMR038C	CCS1
YDR293C	SSD1	YDR318W	MCM21
YGL066W	SGF73	YOL148C	SPT20
YPR120C	CLB5	YHL011C	PRS3
YGL025C	PGD1	YDR335W	MSN5
YHR178W	STB5	YGR270W	YTA7
YOL145C	CTR9	YOR106W	VAM3
YDR254W	CHL4	YGR188C	BUB1
YML001W	YPT7	YPL137C	GIP3
YIL053W	RHR2	YLR398C	SKI2
YLR381W	CTF3	YGL151W	NUT1
YER116C	SLX8	YDR484W	VPS52
YJL095W	BCK1	YMR311C	GLC8
YML001W	YPT7	YGL198W	YIP4
YJR140C	HIR3	YGL151W	NUT1
YDR156W	RPA14	YLR315W	NKP2
YBL025W	RRN10	YBR280C	SAF1
YHR134W	WSS1	YBR107C	IML3
YHR060W	VMA22	YER162C	RAD4
YNL309W	STB1	YMR199W	CLN1
YDR129C	SAC6	YKL048C	ELM1
YLR102C	APC9	YBR084C-A	RPL19A
YNL199C	GCR2	YER139C	YER139C
YNL307C	MCK1	YJR060W	CBF1
YCR020W-B	HTL1	YHR056C	RSC30
YLR090W	XDJ1	YPL137C	GIP3
YBL102W	SFT2	YMR216C	SKY1
YBL008W	HIR1	YOL081W	IRA2
YGL033W	HOP2	YOL081W	IRA2

Predictions at threshold level 0.75 continued...			
Gene1 of predicted pair		Gene2 of predicted pair	
Systematic Name	Standard Name	Systematic Name	Standard Name
YGL058W	RAD6	YHR056C	RSC30
YLR381W	CTF3	YKL101W	HSL1
YDL020C	RPN4	YDR310C	SUM1
YNL068C	FKH2	YKL048C	ELM1
YMR224C	MRE11	YKR035W-A	DID2
YLR288C	MEC3	YEL056W	HAT2
YKR014C	YPT52	YER074W-A	YOS1
YGR135W	PRE9	YNL107W	YAF9
YBR036C	CSG2	YBR161W	CSH1
YKL139W	CTK1	YLR432W	IMD3
YBL078C	ATG8	YPL195W	APL5
YIL159W	BNR1	YMR214W	SCJ1
YJL172W	CPS1	YHR200W	RPN10
YIL002C	INP51	YJL004C	SYS1
YCL001W	RER1	YML067C	ERV41
YBL102W	SFT2	YDR080W	VPS41
YJR135C	MCM22	YOL067C	RTG1
YMR026C	PEX12	YDR310C	SUM1
YGR270W	YTA7	YOR038C	HIR2
YOR080W	DIA2	YJR005W	APL1
YGR105W	VMA21	YKL041W	VPS24
YAL024C	LTE1	YLR192C	HCR1
YGR108W	CLB1	YDR378C	LSM6
YNL076W	MKS1	YLR180W	SAM1
YDR004W	RAD57	YLR381W	CTF3
YLR240W	VPS34	YOR106W	VAM3
YMR026C	PEX12	YDL184C	RPL41A
YNL032W	SIW14	YJL183W	MNN11
YOR080W	DIA2	YJL058C	BIT61

Predictions at threshold level 0.75 continued...			
Gene1 of predicted pair		Gene2 of predicted pair	
Systematic Name	Standard Name	Systematic Name	Standard Name
YMR311C	GLC8	YLR180W	SAM1
YGR072W	UPF3	YLR360W	VPS38
YDR253C	MET32	YML095C	RAD10
YMR142C	RPL13B	YOR066W	MSA1
YNL243W	SLA2	YOR109W	INP53
YIL053W	RHR2	YER144C	UBP5
YDR080W	VPS41	YPL161C	BEM4
YNL215W	IES2	YJL006C	CTK2
YHL011C	PRS3	YLR425W	TUS1
YIL159W	BNR1	YPR030W	CSR2
YLR386W	VAC14	YKR057W	RPS21A
YJL136C	RPS21B	YIL008W	URM1
YPR084W	YPR084W	YNL238W	KEX2
YMR095C	SNO1	YBR217W	ATG12
YBR290W	BSD2	YML103C	NUP188
YDR448W	ADA2	YOR039W	CKB2
YGR249W	MGA1	YNR072W	HXT17
YOR112W	CEX1	YMR054W	STV1
YPL037C	EGD1	YCL001W	RER1
YOR132W	VPS17	YHL020C	OPI1
YLR216C	CPR6	YLR381W	CTF3
YLR234W	TOP3	YKR095W	MLP1
YDR293C	SSD1	YGR020C	VMA7
YGR019W	UGA1	YLR447C	VMA6
YLR371W	ROM2	YLR180W	SAM1
YNL291C	MID1	YOR132W	VPS17
YPL018W	CTF19	YHL002W	HSE1
YGR104C	SRB5	YBR215W	HPC2
YBL102W	SFT2	YHL020C	OPI1

Predictions at threshold level 0.75 continued...			
Gene1 of predicted pair		Gene2 of predicted pair	
Systematic Name	Standard Name	Systematic Name	Standard Name
YMR026C	PEX12	YLR371W	ROM2
YMR016C	SOK2	YDL077C	VAM6
YLR441C	RPS1A	YMR311C	GLC8
YLR216C	CPR6	YBR107C	IML3
YML095C	RAD10	YGL175C	SAE2
YLR119W	SRN2	YNL325C	FIG4
YNL106C	INP52	YCR077C	PAT1
YBR131W	CCZ1	YKR001C	VPS1
YMR231W	PEP5	YML028W	TSA1
YCL010C	SGF29	YBR111W-A	SUS1
YKR072C	SIS2	YOL090W	MSH2
YHR052W	CIC1	YOL041C	NOP12
YDL077C	VAM6	YGL198W	YIP4
YCR081W	SRB8	YER068W	MOT2
YIL076W	SEC28	YHL020C	OPI1
YDR202C	RAV2	YMR216C	SKY1
YNL288W	CAF40	YBR200W	BEM1
YKL048C	ELM1	YOL081W	IRA2
YBR194W	SOY1	YDR137W	RGP1
YFR010W	UBP6	YMR038C	CCS1
YMR127C	SAS2	YOR038C	HIR2
YLL007C	YLL007C	YNL166C	BNI5
YLR381W	CTF3	YDR289C	RTT103
YDR174W	HMO1	YKL139W	CTK1
YLR079W	SIC1	YOR358W	HAP5
YOR038C	HIR2	YGL151W	NUT1
YDL185W	TFP1	YDR318W	MCM21
YPL253C	VIK1	YDR076W	RAD55
YEL065W	SIT1	YHR200W	RPN10

Predictions at threshold level 0.75 continued...			
Gene1 of predicted pair		Gene2 of predicted pair	
Systematic Name	Standard Name	Systematic Name	Standard Name
YJL060W	BNA3	YNL107W	YAF9
YGL223C	COG1	YML012W	ERV25
YFR010W	UBP6	YNL107W	YAF9
YBL047C	EDE1	YOR312C	RPL20B
YMR039C	SUB1	YGL151W	NUT1
YOR332W	VMA4	YKL041W	VPS24
YPL161C	BEM4	YNL127W	FAR11
YML115C	VAN1	YOR312C	RPL20B
YKR057W	RPS21A	YHR200W	RPN10
YGL033W	HOP2	YDR261C	EXG2
YDL223C	HBT1	YOL111C	MDY2
YKL007W	CAP1	YDR309C	GIC2
YBR200W	BEM1	YJL095W	BCK1
YML103C	NUP188	YNL054W	VAC7
YDR137W	RGP1	YDR469W	SDC1
YNL291C	MID1	YNL127W	FAR11
YDR192C	NUP42	YDR101C	ARX1
YPL024W	RMI1	YHR077C	NMD2
YFR031C-A	RPL2A	YOR109W	INP53
YLR192C	HCR1	YNL032W	SIW14
YNL069C	RPL16B	YMR216C	SKY1
YDR320C	SWA2	YBL017C	PEP1
YOL145C	CTR9	YBR010W	HHT1
YIL076W	SEC28	YDR358W	GGA1
YBR131W	CCZ1	YJL117W	PHO86
YDR080W	VPS41	YKR020W	VPS51
YAL024C	LTE1	YGL161C	YIP5
YDR378C	LSM6	YGR188C	BUB1
YLR192C	HCR1	YNL325C	FIG4

Predictions at threshold level 0.75 continued...			
Gene1 of predicted pair		Gene2 of predicted pair	
Systematic Name	Standard Name	Systematic Name	Standard Name
YNL325C	FIG4	YBL103C	RTG3
YMR224C	MRE11	YOR195W	SLK19
YBR108W	YBR108W	YPL137C	GIP3
YGR040W	KSS1	YHR084W	STE12
YGL115W	SNF4	YPR030W	CSR2
YNL206C	RTT106	YPR119W	CLB2
YEL051W	VMA8	YOL018C	TLG2
YNL041C	COG6	YER122C	GLO3
YER116C	SLX8	YJR084W	CSN12
YDR379W	RGA2	YKL032C	IXR1
YBL058W	SHP1	YLR306W	UBC12
YCR024C-A	PMP1	YKL117W	SBA1
YDR320C	SWA2	YMR216C	SKY1
YOR332W	VMA4	YHR039C-A	VMA10
YJL053W	PEP8	YDR108W	GSG1
YKL025C	PAN3	YHR114W	BZZ1
YOR033C	EXO1	YNL082W	PMS1
YBR200W	BEM1	YBR107C	IML3
YMR016C	SOK2	YKR035W-A	DID2
YGR108W	CLB1	YDR318W	MCM21
YER122C	GLO3	YAL002W	VPS8
YFR010W	UBP6	YJR060W	CBF1
YOR141C	ARP8	YDL226C	GCS1
YPR120C	CLB5	YKL101W	HSL1
YJR106W	ECM27	YFL044C	OTU1
YIL076W	SEC28	YJR140C	HIR3
YBR131W	CCZ1	YNL041C	COG6
YLR441C	RPS1A	YKR099W	BAS1
YHR134W	WSS1	YHR012W	VPS29

Predictions at threshold level 0.75 continued...			
Gene1 of predicted pair		Gene2 of predicted pair	
Systematic Name	Standard Name	Systematic Name	Standard Name
YOR156C	NFI1	YDR155C	CPR1
YHR160C	PEX18	YDL065C	PEX19
YPL106C	SSE1	YPR046W	MCM16
YMR198W	CIK1	YBR082C	UBC4
YNL127W	FAR11	YER122C	GLO3
YFR032C-A	RPL29	YDL006W	PTC1
YOR096W	RPS7A	YER102W	RPS8B
YOR083W	WHI5	YDR335W	MSN5
YNL032W	SIW14	YML001W	YPT7
YPL129W	TAF14	YMR109W	MYO5
YPL024W	RMI1	YDR318W	MCM21
YGL135W	RPL1B	YBR181C	RPS6B
YPL024W	RMI1	YHR200W	RPN10
YBR195C	MSI1	YCR077C	PAT1
YMR026C	PEX12	YPL137C	GIP3
YLR337C	VRP1	YBL051C	PIN4
YJL136C	RPS21B	YGL163C	RAD54
YBL102W	SFT2	YOR127W	RGA1
YML115C	VAN1	YER162C	RAD4
YML103C	NUP188	YFR030W	MET10
YNL166C	BNI5	YDR289C	RTT103
YLR058C	SHM2	YNL236W	SIN4
YJL128C	PBS2	YER118C	SHO1
YLR337C	VRP1	YBR057C	MUM2
YER122C	GLO3	YLR182W	SWI6
YGR072W	UPF3	YML071C	COG8
YDR443C	SSN2	YNL058C	YNL058C
YOR083W	WHI5	YGL003C	CDH1
YKR072C	SIS2	YJR140C	HIR3

Predictions at threshold level 0.75 continued...			
Gene1 of predicted pair		Gene2 of predicted pair	
Systematic Name	Standard Name	Systematic Name	Standard Name
YBR041W	FAT1	YDL006W	PTC1
YFR031C-A	RPL2A	YOL081W	IRA2
YDR289C	RTT103	YOR308C	SNU66
YJL172W	CPS1	YOL111C	MDY2
YHR152W	SPO12	YNL229C	URE2
YDL116W	NUP84	YER110C	KAP123
YAL024C	LTE1	YLR182W	SWI6
YPR120C	CLB5	YJR060W	CBF1
YMR231W	PEP5	YDR076W	RAD55
YPL050C	MNN9	YMR089C	YTA12
YNL253W	TEX1	YNL004W	HRB1
YFR032C-A	RPL29	YGR217W	CCH1
YMR055C	BUB2	YPL106C	SSE1
YGL003C	CDH1	YJR035W	RAD26
YML001W	YPT7	YAL002W	VPS8
YDR225W	HTA1	YLR032W	RAD5
YCR020C-A	MAK31	YJL004C	SYS1
YLR371W	ROM2	YMR038C	CCS1
YDR137W	RGP1	YHR081W	LRP1
YLR337C	VRP1	YPL213W	LEA1
YCR086W	CSM1	YGL253W	HXK2
YOL030W	GAS5	YJL187C	SWE1
YMR231W	PEP5	YGL198W	YIP4
YDR101C	ARX1	YOL041C	NOP12
YJL176C	SWI3	YBL101C	ECM21
YNL238W	KEX2	YCR053W	THR4
YER107C	GLE2	YLR055C	SPT8
YEL050C	RML2	YDL226C	GCS1
YBR200W	BEM1	YDR264C	AKR1

Predictions at threshold level 0.75 continued...			
**Gene1 of predicted pair**		**Gene2 of predicted pair**	
**Systematic Name**	**Standard Name**	**Systematic Name**	**Standard Name**
YJR135C	MCM22	YGL252C	RTG2
YML071C	COG8	YBR036C	CSG2
YLR090W	XDJ1	YOL111C	MDY2
YGR020C	VMA7	YGL054C	ERV14
YJL210W	PEX2	YDL065C	PEX19
YNL238W	KEX2	YLR398C	SKI2
YNL041C	COG6	YNR032W	PPG1
YLR135W	SLX4	YOL090W	MSH2
YOL054W	PSH1	YOL081W	IRA2
YCR053W	THR4	YDR207C	UME6
YMR255W	GFD1	YBR017C	KAP104
YKL048C	ELM1	YOL111C	MDY2
YPR017C	DSS4	YNR032W	PPG1
YOR275C	RIM20	YIL008W	URM1
YPL037C	EGD1	YDR153C	ENT5
YFR030W	MET10	YDR183W	PLP1
YFR031C-A	RPL2A	YBR171W	SEC66
YLR131C	ACE2	YKL041W	VPS24
YCL037C	SRO9	YOL045W	PSK2
YLR441C	RPS1A	YHR039C-A	VMA10
YFL023W	BUD27	YGL175C	SAE2
YIL159W	BNR1	YMR152W	YIM1
YPL161C	BEM4	YLR370C	ARC18
YLR360W	VPS38	YNR032W	PPG1
YDR496C	PUF6	YKL185W	ASH1
YJL154C	VPS35	YJL036W	SNX4
YLR079W	SIC1	YDR289C	RTT103
YNL325C	FIG4	YJR056C	YJR056C
YLR148W	PEP3	YLR043C	TRX1

Predictions at threshold level 0.75 continued...			
Gene1 of predicted pair		Gene2 of predicted pair	
Systematic Name	Standard Name	Systematic Name	Standard Name
YMR216C	SKY1	YJR040W	GEF1
YLR095C	IOC2	YHR039C-A	VMA10
YOR270C	VPH1	YNL058C	YNL058C
YER162C	RAD4	YOR035C	SHE4
YBL027W	RPL19B	YER096W	SHC1
YBR200W	BEM1	YDR207C	UME6
YBR194W	SOY1	YML028W	TSA1
YNR052C	POP2	YNL016W	PUB1
YDR225W	HTA1	YPL057C	SUR1
YKR020W	VPS51	YDR323C	PEP7
YOR038C	HIR2	YDR310C	SUM1
YNL288W	CAF40	YBR171W	SEC66
YOR043W	WHI2	YEL060C	PRB1
YBR200W	BEM1	YOL081W	IRA2
YDL223C	HBT1	YHR200W	RPN10
YPR030W	CSR2	YER040W	GLN3
YLR447C	VMA6	YGL124C	MON1
YHR156C	LIN1	YML071C	COG8
YLR441C	RPS1A	YNR072W	HXT17
YJL151C	SNA3	YGR268C	HUA1
YPL105C	YPL105C	YDL006W	PTC1
YJL058C	BIT61	YNL229C	URE2
YLR370C	ARC18	YJR035W	RAD26
YPL115C	BEM3	YMR172W	HOT1
YHR012W	VPS29	YDR153C	ENT5
YDL002C	NHP10	YDR440W	DOT1
YDR137W	RGP1	YNL041C	COG6
YER161C	SPT2	YER139C	YER139C
YBR171W	SEC66	YHR012W	VPS29

Predictions at threshold level 0.75 continued...			
Gene1 of predicted pair		Gene2 of predicted pair	
Systematic Name	Standard Name	Systematic Name	Standard Name
YER116C	SLX8	YNL058C	YNL058C
YDL005C	MED2	YNL079C	TPM1
YDR335W	MSN5	YHR167W	THP2
YDR142C	PEX7	YNL214W	PEX17
YMR016C	SOK2	YOR327C	SNC2
YNL314W	DAL82	YIR023W	DAL81
YBL008W	HIR1	YGL148W	ARO2
YLR386W	VAC14	YKL032C	IXR1
YOR001W	RRP6	YDR378C	LSM6
YKR020W	VPS51	YDL192W	ARF1
YGL135W	RPL1B	YML097C	VPS9
YBR126C	TPS1	YHR156C	LIN1
YPR084W	YPR084W	YML001W	YPT7
YOR275C	RIM20	YBR283C	SSH1
YER110C	KAP123	YJR135C	MCM22
YKL160W	ELF1	YOR308C	SNU66
YHR178W	STB5	YGR188C	BUB1
YDR156W	RPA14	YLR079W	SIC1
YAL020C	ATS1	YBL051C	PIN4
YLR381W	CTF3	YFR030W	MET10
YEL065W	SIT1	YHR013C	ARD1
YIL053W	RHR2	YDR358W	GGA1
YMR026C	PEX12	YDL192W	ARF1
YAL024C	LTE1	YLR180W	SAM1
YMR224C	MRE11	YJL095W	BCK1
YNL243W	SLA2	YBR200W	BEM1
YLR337C	VRP1	YPL253C	VIK1
YMR080C	NAM7	YLR135W	SLX4
YDR261C	EXG2	YER139C	YER139C

Predictions at threshold level 0.75 continued...			
Gene1 of predicted pair		Gene2 of predicted pair	
Systematic Name	Standard Name	Systematic Name	Standard Name
YBR041W	FAT1	YGR135W	PRE9
YPL180W	TCO89	YLR398C	SKI2
YOR076C	SKI7	YOR191W	RIS1
YMR179W	SPT21	YGR188C	BUB1
YJR060W	CBF1	YOL108C	INO4
YDR137W	RGP1	YNL297C	MON2
YDR358W	GGA1	YAL055W	PEX22
YHR111W	UBA4	YGL124C	MON1
YDR108W	GSG1	YOR109W	INP53
YPL001W	HAT1	YMR055C	BUB2
YNL041C	COG6	YMR214W	SCJ1
YOR106W	VAM3	YPR051W	MAK3
YPL057C	SUR1	YML001W	YPT7
YAL024C	LTE1	YHR111W	UBA4
YDL223C	HBT1	YOR066W	MSA1
YPR084W	YPR084W	YJL154C	VPS35
YJR040W	GEF1	YEL053C	MAK10
YDR439W	LRS4	YPL152W	RRD2
YLR386W	VAC14	YGL151W	NUT1
YNL297C	MON2	YOR109W	INP53
YOR039W	CKB2	YNL206C	RTT106
YPL106C	SSE1	YDL047W	SIT4
YGR072W	UPF3	YKR001C	VPS1
YMR216C	SKY1	YPL037C	EGD1
YNL058C	YNL058C	YMR152W	YIM1
YKL160W	ELF1	YLR410W	VIP1
YML001W	YPT7	YJR033C	RAV1
YBR084C-A	RPL19A	YJL095W	BCK1
YNL243W	SLA2	YLR330W	CHS5

Predictions at threshold level 0.75 continued...			
Gene1 of predicted pair		Gene2 of predicted pair	
Systematic Name	Standard Name	Systematic Name	Standard Name
YJR059W	PTK2	YER110C	KAP123
YOR290C	SNF2	YDR469W	SDC1
YPR017C	DSS4	YEL043W	YEL043W
YLR090W	XDJ1	YKL126W	YPK1
YPL058C	PDR12	YDR423C	CAD1
YOR080W	DIA2	YDR318W	MCM21
YBL008W	HIR1	YPR120C	CLB5
YBR171W	SEC66	YCR053W	THR4
YER120W	SCS2	YPR032W	SRO7
YPL024W	RMI1	YPR030W	CSR2
YLR102C	APC9	YGL031C	RPL24A
YKL025C	PAN3	YML016C	PPZ1
YIL076W	SEC28	YPL161C	BEM4
YBR215W	HPC2	YMR055C	BUB2
YNL297C	MON2	YPL234C	TFP3
YDR477W	SNF1	YML071C	COG8
YLR315W	NKP2	YLR350W	ORM2
YDL013W	SLX5	YGL031C	RPL24A
YPL042C	SSN3	YMR037C	MSN2
YHR060W	VMA22	YBR280C	SAF1
YMR092C	AIP1	YDR389W	SAC7
YLR083C	EMP70	YPR051W	MAK3
YBR171W	SEC66	YHR114W	BZZ1
YMR202W	ERG2	YJL095W	BCK1
YMR199W	CLN1	YBL047C	EDE1
YOR191W	RIS1	YOR243C	PUS7
YGL175C	SAE2	YML028W	TSA1
YEL043W	YEL043W	YOR327C	SNC2
YDL059C	RAD59	YGL175C	SAE2

Predictions at threshold level 0.75 continued...			
Gene1 of predicted pair		Gene2 of predicted pair	
Systematic Name	Standard Name	Systematic Name	Standard Name
YOR127W	RGA1	YPR051W	MAK3
YAL024C	LTE1	YFR030W	MET10
YML095C	RAD10	YBR084C-A	RPL19A
YBR290W	BSD2	YMR273C	ZDS1
YDR076W	RAD55	YJR035W	RAD26
YFL023W	BUD27	YEL053C	MAK10
YNL069C	RPL16B	YDL077C	VAM6
YDR245W	MNN10	YJL006C	CTK2
YGL087C	MMS2	YLR381W	CTF3
YKR099W	BAS1	YLR093C	NYV1
YBR127C	VMA2	YNL238W	KEX2
YPL042C	SSN3	YGR252W	GCN5
YBL058W	SHP1	YHR081W	LRP1
YML095C	RAD10	YMR201C	RAD14
YPL195W	APL5	YMR230W	RPS10B
YMR224C	MRE11	YMR214W	SCJ1
YJR056C	YJR056C	YML001W	YPT7
YDR108W	GSG1	YJR060W	CBF1
YMR199W	CLN1	YFL023W	BUD27
YGL066W	SGF73	YPR046W	MCM16
YGR239C	PEX21	YNL214W	PEX17
YOR132W	VPS17	YER122C	GLO3
YBL088C	TEL1	YML095C	RAD10
YLR083C	EMP70	YJR060W	CBF1
YJL095W	BCK1	YDL077C	VAM6
YMR095C	SNO1	YMR230W	RPS10B
YBR010W	HHT1	YLR357W	RSC2
YGR104C	SRB5	YGR270W	YTA7
YJL154C	VPS35	YCL037C	SRO9

Predictions at threshold level 0.75 continued...			
Gene1 of predicted pair		Gene2 of predicted pair	
Systematic Name	Standard Name	Systematic Name	Standard Name
YMR224C	MRE11	YML062C	MFT1
YDL002C	NHP10	YOR213C	SAS5
YGL054C	ERV14	YHR012W	VPS29
YJR140C	HIR3	YPL057C	SUR1
YDR260C	SWM1	YCR086W	CSM1
YER096W	SHC1	YOR014W	RTS1
YDL161W	ENT1	YCR088W	ABP1
YPR030W	CSR2	YDR207C	UME6
YDR383C	NKP1	YMR224C	MRE11
YDR137W	RGP1	YJL183W	MNN11
YJL006C	CTK2	YNL079C	TPM1
YNL069C	RPL16B	YHR012W	VPS29
YJL124C	LSM1	YOR141C	ARP8
YDR507C	GIN4	YHR200W	RPN10
YDL116W	NUP84	YGL175C	SAE2
YOR358W	HAP5	YKL109W	HAP4
YKR001C	VPS1	YDL192W	ARF1
YLR095C	IOC2	YBR171W	SEC66
YBR019C	GAL10	YBR280C	SAF1
YDL020C	RPN4	YML095C	RAD10
YEL065W	SIT1	YLR095C	IOC2
YJR032W	CPR7	YMR152W	YIM1
YDL101C	DUN1	YGL175C	SAE2
YEL065W	SIT1	YML001W	YPT7
YHR157W	REC104	YJR021C	REC107
YIL002C	INP51	YPR173C	VPS4
YML115C	VAN1	YBL025W	RRN10
YPL024W	RMI1	YLR320W	MMS22
YMR153W	NUP53	YKL117W	SBA1

Predictions at threshold level 0.75 continued...			
Gene1 of predicted pair		Gene2 of predicted pair	
Systematic Name	Standard Name	Systematic Name	Standard Name
YML001W	YPT7	YMR231W	PEP5
YDR174W	HMO1	YBR200W	BEM1
YML095C	RAD10	YPL161C	BEM4
YEL044W	IES6	YPL050C	MNN9
YDL180W	YDL180W	YDR319C	YDR319C
YPR084W	YPR084W	YMR054W	STV1
YOR337W	TEA1	YMR152W	YIM1
YKL160W	ELF1	YNL107W	YAF9
YMR095C	SNO1	YOR337W	TEA1
YMR026C	PEX12	YBR173C	UMP1
YBR108W	YBR108W	YGR268C	HUA1
YPL240C	HSP82	YPR118W	MRI1
YDR477W	SNF1	YNL199C	GCR2
YGL066W	SGF73	YLR032W	RAD5
YGR133W	PEX4	YML097C	VPS9
YLR083C	EMP70	YDR153C	ENT5
YJL006C	CTK2	YFR036W	CDC26
YJR032W	CPR7	YMR311C	GLC8
YER116C	SLX8	YGL003C	CDH1
YFR031C-A	RPL2A	YNL058C	YNL058C
YGL066W	SGF73	YOR014W	RTS1
YHR115C	DMA1	YGL031C	RPL24A
YNL058C	YNL058C	YJR084W	CSN12
YDL020C	RPN4	YGL253W	HXK2
YBR107C	IML3	YGL163C	RAD54
YNL291C	MID1	YDL077C	VAM6
YLR234W	TOP3	YDL059C	RAD59
YLL039C	UBI4	YER143W	DDI1
YJR140C	HIR3	YPL001W	HAT1

Predictions at threshold level 0.75 continued...			
**Gene1 of predicted pair**		**Gene2 of predicted pair**	
**Systematic Name**	**Standard Name**	**Systematic Name**	**Standard Name**
YMR224C	MRE11	YGL031C	RPL24A
YGL033W	HOP2	YFL023W	BUD27
YBL047C	EDE1	YLR425W	TUS1
YDR108W	GSG1	YOR308C	SNU66
YGR104C	SRB5	YBR260C	RGD1
YER179W	DMC1	YPL121C	MEI5
YER116C	SLX8	YBR073W	RDH54
YJL095W	BCK1	YKL161C	YKL161C
YOR038C	HIR2	YOR308C	SNU66
YPL037C	EGD1	YAR014C	BUD14
YJR135C	MCM22	YOL091W	SPO21
YER110C	KAP123	YJR032W	CPR7
YGL147C	RPL9A	YGL025C	PGD1
YOR083W	WHI5	YJR035W	RAD26
YHR081W	LRP1	YGL124C	MON1
YDR245W	MNN10	YKR020W	VPS51
YBR107C	IML3	YDR260C	SWM1
YOR033C	EXO1	YML062C	MFT1
YCR066W	RAD18	YKL114C	APN1
YNL238W	KEX2	YER122C	GLO3
YGL147C	RPL9A	YPL024W	RMI1
YLR360W	VPS38	YDL161W	ENT1
YNL031C	HHT2	YJR063W	RPA12
YNL058C	YNL058C	YGL124C	MON1
YJL128C	PBS2	YCR073C	SSK22
YDR176W	NGG1	YKL139W	CTK1
YMR054W	STV1	YJR032W	CPR7
YAL024C	LTE1	YGL212W	VAM7
YLR083C	EMP70	YPL195W	APL5

Predictions at threshold level 0.75 continued...			
Gene1 of predicted pair		Gene2 of predicted pair	
Systematic Name	Standard Name	Systematic Name	Standard Name
YGL060W	YBP2	YMR202W	ERG2
YKL139W	CTK1	YLR306W	UBC12
YPL105C	YPL105C	YML038C	YMD8
YPR051W	MAK3	YPR032W	SRO7
YBL072C	RPS8A	YHL002W	HSE1
YHR111W	UBA4	YBR114W	RAD16
YKR020W	VPS51	YGL054C	ERV14
YJL036W	SNX4	YPL195W	APL5
YPR040W	TIP41	YDL047W	SIT4
YKR072C	SIS2	YCR077C	PAT1
YNL051W	COG5	YPR032W	SRO7
YPL096C-A	ERI1	YGR135W	PRE9
YGL212W	VAM7	YOL121C	RPS19A
YLR192C	HCR1	YJL095W	BCK1
YPR017C	DSS4	YNL291C	MID1
YPR017C	DSS4	YGR105W	VMA21
YLR018C	POM34	YNL199C	GCR2
YML058W	SML1	YDR253C	MET32
YDR146C	SWI5	YOR275C	RIM20
YAR014C	BUD14	YLR182W	SWI6
YMR142C	RPL13B	YNR072W	HXT17
YKL139W	CTK1	YGL124C	MON1
YOR312C	RPL20B	YLR182W	SWI6
YIL159W	BNR1	YNR072W	HXT17
YHR178W	STB5	YDL155W	CLB3
YHR200W	RPN10	YML071C	COG8
YDL160C	DHH1	YML058W	SML1
YLR410W	VIP1	YJR035W	RAD26
YGL212W	VAM7	YJR040W	GEF1

Predictions at threshold level 0.75 continued...			
Gene1 of predicted pair		Gene2 of predicted pair	
Systematic Name	Standard Name	Systematic Name	Standard Name
YPR051W	MAK3	YBR114W	RAD16
YGL237C	HAP2	YKL048C	ELM1
YPL024W	RMI1	YKL048C	ELM1
YER174C	GRX4	YJL129C	TRK1
YMR224C	MRE11	YDR310C	SUM1
YKR020W	VPS51	YPL089C	RLM1
YKL126W	YPK1	YLR371W	ROM2
YMR133W	REC114	YPR030W	CSR2
YHR052W	CIC1	YDR312W	SSF2
YEL053C	MAK10	YER040W	GLN3
YPL024W	RMI1	YLR094C	GIS3
YJR056C	YJR056C	YLR320W	MMS22
YDR137W	RGP1	YDL083C	RPS16B
YNL218W	MGS1	YBL079W	NUP170
YKL048C	ELM1	YFR030W	MET10
YAL024C	LTE1	YKR035W-A	DID2
YIL053W	RHR2	YGL147C	RPL9A
YDR202C	RAV2	YEL053C	MAK10
YBR290W	BSD2	YIL156W	UBP7
YDR254W	CHL4	YJR063W	RPA12
YLR079W	SIC1	YLR254C	NDL1
YMR216C	SKY1	YDR153C	ENT5
YNL229C	URE2	YER040W	GLN3
YLR095C	IOC2	YER044C	ERG28
YDL216C	RRI1	YOL117W	RRI2
YDR392W	SPT3	YBR111W-A	SUS1
YMR068W	AVO2	YNL229C	URE2
YFR010W	UBP6	YDR318W	MCM21
YGR184C	UBR1	YNL307C	MCK1

Predictions at threshold level 0.75 continued...			
Gene1 of predicted pair		Gene2 of predicted pair	
Systematic Name	Standard Name	Systematic Name	Standard Name
YMR127C	SAS2	YBR107C	IML3
YKL139W	CTK1	YOL111C	MDY2
YNR049C	MSO1	YPR032W	SRO7
YDL020C	RPN4	YPL018W	CTF19
YBR217W	ATG12	YPR036W	VMA13
YKR035W-A	DID2	YJL095W	BCK1
YJL154C	VPS35	YDL161W	ENT1
YLR337C	VRP1	YHR200W	RPN10
YIL103W	DPH1	YCR086W	CSM1
YOR358W	HAP5	YGR188C	BUB1
YMR032W	HOF1	YBL085W	BOI1
YDR378C	LSM6	YPR046W	MCM16
YFR010W	UBP6	YPL037C	EGD1
YOR026W	BUB3	YHR205W	SCH9
YKL048C	ELM1	YDL194W	SNF3
YNL025C	SSN8	YJL124C	LSM1
YGR270W	YTA7	YHR167W	THP2
YAL024C	LTE1	YER144C	UBP5
YAL015C	NTG1	YKL114C	APN1
YDR245W	MNN10	YBR215W	HPC2
YOR337W	TEA1	YMR139W	RIM11
YLR288C	MEC3	YOR368W	RAD17
YNL087W	TCB2	YOR043W	WHI2
YBR194W	SOY1	YJR060W	CBF1
YML001W	YPT7	YLR371W	ROM2
YNL218W	MGS1	YOR243C	PUS7
YDR329C	PEX3	YJL210W	PEX2
YOL062C	APM4	YPL259C	APM1
YJR059W	PTK2	YGR059W	SPR3

Predictions at threshold level 0.75 continued...			
Gene1 of predicted pair		Gene2 of predicted pair	
Systematic Name	Standard Name	Systematic Name	Standard Name
YLR181C	VTA1	YPR173C	VPS4
YJL060W	BNA3	YML095C	RAD10
YMR199W	CLN1	YGR135W	PRE9
YLR337C	VRP1	YLR447C	VMA6
YDR507C	GIN4	YGL208W	SIP2
YAL024C	LTE1	YER139C	YER139C
YOR083W	WHI5	YMR198W	CIK1
YDR245W	MNN10	YEL013W	VAC8
YGR072W	UPF3	YMR054W	STV1
YGR104C	SRB5	YBL075C	SSA3
YKR020W	VPS51	YOL018C	TLG2
YAL024C	LTE1	YHR039C-A	VMA10
YPR036W	VMA13	YNR072W	HXT17
YNL032W	SIW14	YGL115W	SNF4
YNR023W	SNF12	YER161C	SPT2
YAL024C	LTE1	YDL223C	HBT1
YOR112W	CEX1	YMR216C	SKY1
YHL031C	GOS1	YOR036W	PEP12
YLR234W	TOP3	YJR135C	MCM22
YDR363W-A	SEM1	YDR469W	SDC1
YBL058W	SHP1	YJL095W	BCK1
YHR052W	CIC1	YKR057W	RPS21A
YOR106W	VAM3	YER042W	MXR1
YMR224C	MRE11	YGR081C	SLX9
YER116C	SLX8	YLR398C	SKI2
YKL006W	RPL14A	YJL006C	CTK2
YDL077C	VAM6	YNR032W	PPG1
YBR200W	BEM1	YKL129C	MYO3
YCR065W	HCM1	YPL042C	SSN3

Predictions at threshold level 0.75 continued...			
Gene1 of predicted pair		Gene2 of predicted pair	
Systematic Name	Standard Name	Systematic Name	Standard Name
YMR127C	SAS2	YHR111W	UBA4
YNL288W	CAF40	YOL067C	RTG1
YDR477W	SNF1	YBR217W	ATG12
YPL240C	HSP82	YER105C	NUP157
YAL051W	OAF1	YMR198W	CIK1
YHR178W	STB5	YOL067C	RTG1
YEL050C	RML2	YOR001W	RRP6
YHR079C	IRE1	YGL223C	COG1
YMR172W	HOT1	YJL154C	VPS35
YNL054W	VAC7	YNL116W	DMA2
YJL053W	PEP8	YDR310C	SUM1
YHR081W	LRP1	YBR019C	GAL10
YPR131C	NAT3	YJR005W	APL1
YMR224C	MRE11	YHR156C	LIN1
YDR440W	DOT1	YEL013W	VAC8
YOR290C	SNF2	YOR064C	YNG1
YML058W	SML1	YGL115W	SNF4
YFR031C-A	RPL2A	YNR032W	PPG1
YBR126C	TPS1	YFL033C	RIM15
YJL036W	SNX4	YMR054W	STV1
YDL020C	RPN4	YHR012W	VPS29
YNL243W	SLA2	YNL153C	GIM3
YBR215W	HPC2	YPL256C	CLN2
YGL178W	MPT5	YLR418C	CDC73
YNR052C	POP2	YJL164C	TPK1
YPR052C	NHP6A	YDL160C	DHH1
YDR477W	SNF1	YCR053W	THR4
YOL090W	MSH2	YNL076W	MKS1
YHL031C	GOS1	YCL037C	SRO9

Predictions at threshold level 0.75 continued...			
**Gene1 of predicted pair**		**Gene2 of predicted pair**	
**Systematic Name**	**Standard Name**	**Systematic Name**	**Standard Name**
YMR039C	SUB1	YML016C	PPZ1
YLR094C	GIS3	YBL025W	RRN10
YKL032C	IXR1	YDR264C	AKR1
YMR016C	SOK2	YNL076W	MKS1
YMR198W	CIK1	YGR133W	PEX4
YKR001C	VPS1	YER120W	SCS2
YHR081W	LRP1	YPL195W	APL5
YBR107C	IML3	YJL095W	BCK1
YDR202C	RAV2	YNL127W	FAR11
YLR386W	VAC14	YPR030W	CSR2
YJL095W	BCK1	YEL036C	ANP1
YAL024C	LTE1	YKL139W	CTK1
YNL107W	YAF9	YNL225C	CNM67
YJL006C	CTK2	YNL107W	YAF9
YPR051W	MAK3	YFL033C	RIM15
YOR195W	SLK19	YDR207C	UME6
YPR084W	YPR084W	YPL195W	APL5
YNL264C	PDR17	YGR014W	MSB2
YOR127W	RGA1	YML071C	COG8
YIR003W	YIR003W	YDR245W	MNN10
YPR036W	VMA13	YCR053W	THR4
YFR010W	UBP6	YMR016C	SOK2
YMR172W	HOT1	YGL253W	HXK2
YLR079W	SIC1	YMR164C	MSS11
YOR033C	EXO1	YLR234W	TOP3
YPL115C	BEM3	YOR127W	RGA1
YDR261C	EXG2	YMR214W	SCJ1
YNR072W	HXT17	YHR081W	LRP1
YMR116C	ASC1	YLR410W	VIP1

Predictions at threshold level 0.75 continued...			
Gene1 of predicted pair		Gene2 of predicted pair	
Systematic Name	Standard Name	Systematic Name	Standard Name
YLR093C	NYV1	YDR207C	UME6
YFR036W	CDC26	YNL225C	CNM67
YNL297C	MON2	YEL053C	MAK10
YBL102W	SFT2	YER162C	RAD4
YFL023W	BUD27	YML102W	CAC2
YCR088W	ABP1	YPL045W	VPS16
YMR054W	STV1	YOL108C	INO4
YGL246C	RAI1	YIL133C	RPL16A
YBL071W-A	KTI11	YOL121C	RPS19A
YPR171W	BSP1	YMR109W	MYO5
YDR245W	MNN10	YDL002C	NHP10
YLR425W	TUS1	YDR207C	UME6
YLR452C	SST2	YJR032W	CPR7
YBR171W	SEC66	YBR019C	GAL10
YOR290C	SNF2	YLR180W	SAM1
YBR171W	SEC66	YDR358W	GGA1
YDR137W	RGP1	YAL002W	VPS8
YIL053W	RHR2	YGL078C	DBP3
YHR060W	VMA22	YER139C	YER139C
YPL115C	BEM3	YOR132W	VPS17
YML115C	VAN1	YLR094C	GIS3
YDR448W	ADA2	YBL002W	HTB2
YML115C	VAN1	YER144C	UBP5
YJR063W	RPA12	YDR318W	MCM21
YOR083W	WHI5	YBR107C	IML3
YOR124C	UBP2	YML099C	ARG81
YDR254W	CHL4	YLR429W	CRN1
YDR129C	SAC6	YJR032W	CPR7
YKR020W	VPS51	YER120W	SCS2

Predictions at threshold level 0.75 continued...			
Gene1 of predicted pair		Gene2 of predicted pair	
Systematic Name	Standard Name	Systematic Name	Standard Name
YNL218W	MGS1	YOR191W	RIS1
YDR207C	UME6	YKR030W	GMH1
YPL106C	SSE1	YDR318W	MCM21
YLR370C	ARC18	YLR425W	TUS1
YIL156W	UBP7	YBL079W	NUP170
YFL023W	BUD27	YLR398C	SKI2
YBR217W	ATG12	YDR137W	RGP1
YPR084W	YPR084W	YDL002C	NHP10
YOR290C	SNF2	YLR370C	ARC18
YGR249W	MGA1	YOR066W	MSA1
YNL201C	PSY2	YLR410W	VIP1
YGR249W	MGA1	YBR108W	YBR108W
YMR246W	FAA4	YMR313C	TGL3
YMR273C	ZDS1	YPL161C	BEM4
YDL083C	RPS16B	YER122C	GLO3
YPL115C	BEM3	YEL056W	HAT2
YPL096C-A	ERI1	YGL212W	VAM7
YOR156C	NFI1	YGL237C	HAP2
YGL025C	PGD1	YML071C	COG8
YIL008W	URM1	YJL190C	RPS22A
YOR191W	RIS1	YHR077C	NMD2
YML103C	NUP188	YNL199C	GCR2
YGR020C	VMA7	YAR014C	BUD14
YDR484W	VPS52	YOL018C	TLG2
YIL034C	CAP2	YDR155C	CPR1
YJL124C	LSM1	YML028W	TSA1
YLR234W	TOP3	YOR039W	CKB2
YPL195W	APL5	YOR327C	SNC2
YOL121C	RPS19A	YMR214W	SCJ1

Predictions at threshold level 0.75 continued...			
Gene1 of predicted pair		Gene2 of predicted pair	
Systematic Name	Standard Name	Systematic Name	Standard Name
YJL183W	MNN11	YLR398C	SKI2
YOL117W	RRI2	YJR084W	CSN12
YMR198W	CIK1	YBR194W	SOY1
YML115C	VAN1	YKR099W	BAS1
YNL243W	SLA2	YML094W	GIM5
YGR261C	APL6	YNL238W	KEX2
YJR135C	MCM22	YDL226C	GCS1
YFR010W	UBP6	YHR013C	ARD1
YNL243W	SLA2	YJL148W	RPA34
YPL024W	RMI1	YML071C	COG8
YML058W	SML1	YPR120C	CLB5
YDR443C	SSN2	YOR008C	SLG1
YLR234W	TOP3	YML013W	UBX2
YHR161C	YAP1801	YOR035C	SHE4
YDR443C	SSN2	YMR142C	RPL13B
YMR004W	MVP1	YGR020C	VMA7
YJR106W	ECM27	YMR319C	FET4
YKL116C	PRR1	YMR311C	GLC8
YMR095C	SNO1	YJR084W	CSN12
YKL080W	VMA5	YJL095W	BCK1
YLR083C	EMP70	YNL069C	RPL16B
YKR095W	MLP1	YKL068W	NUP100
YGR100W	MDR1	YHR203C	RPS4B
YHR200W	RPN10	YKR048C	NAP1
YDL184C	RPL41A	YMR153W	NUP53
YDR071C	PAA1	YJL140W	RPB4
YDL175C	AIR2	YNL147W	LSM7
YHL020C	OPI1	YER120W	SCS2
YNL291C	MID1	YJR060W	CBF1

Predictions at threshold level 0.75 continued...			
**Gene1 of predicted pair**		**Gene2 of predicted pair**	
**Systematic Name**	**Standard Name**	**Systematic Name**	**Standard Name**
YJL057C	IKS1	YPR119W	CLB2
YOR083W	WHI5	YDR254W	CHL4
YGR184C	UBR1	YLR032W	RAD5
YHR039C-A	VMA10	YER040W	GLN3
YBL008W	HIR1	YLR320W	MMS22
YFL013C	IES1	YLR315W	NKP2
YNL079C	TPM1	YBL067C	UBP13
YJR059W	PTK2	YIL148W	RPL40A
YGL250W	RMR1	YLR350W	ORM2
YOR023C	AHC1	YEL056W	HAT2
YGR072W	UPF3	YPR032W	SRO7
YJL060W	BNA3	YLR058C	SHM2
YNR072W	HXT17	YLR371W	ROM2
YOR132W	VPS17	YHR012W	VPS29
YLR192C	HCR1	YMR214W	SCJ1
YKL126W	YPK1	YPR046W	MCM16
YKR035W-A	DID2	YBR019C	GAL10
YLR135W	SLX4	YGR071C	YGR071C
YNL325C	FIG4	YLR370C	ARC18
YBL058W	SHP1	YBR030W	YBR030W
YMR224C	MRE11	YER116C	SLX8
YMR153W	NUP53	YDL077C	VAM6
YIL076W	SEC28	YDL184C	RPL41A
YMR038C	CCS1	YOR014W	RTS1
YLR234W	TOP3	YMR199W	CLN1
YDR439W	LRS4	YJR063W	RPA12
YDR110W	FOB1	YBR119W	MUD1
YGR054W	YGR054W	YGR188C	BUB1
YML001W	YPT7	YDR261C	EXG2

Predictions at threshold level 0.75 continued...			
Gene1 of predicted pair		Gene2 of predicted pair	
Systematic Name	Standard Name	Systematic Name	Standard Name
YLR399C	BDF1	YML074C	FPR3
YMR224C	MRE11	YMR284W	YKU70
YMR152W	YIM1	YOR327C	SNC2
YDL192W	ARF1	YDL077C	VAM6
YLR234W	TOP3	YBL021C	HAP3
YDR137W	RGP1	YOL081W	IRA2
YEL027W	CUP5	YJL095W	BCK1
YNL325C	FIG4	YKR035W-A	DID2
YDR004W	RAD57	YLR234W	TOP3
YDR310C	SUM1	YKR030W	GMH1
YER116C	SLX8	YPL137C	GIP3
YJL127C	SPT10	YDR378C	LSM6
YBR107C	IML3	YDR183W	PLP1
YOR080W	DIA2	YBR200W	BEM1
YLR386W	VAC14	YNL307C	MCK1
YPL178W	CBC2	YKR024C	DBP7
YPL042C	SSN3	YLR315W	NKP2
YKL139W	CTK1	YHR108W	GGA2
YDR137W	RGP1	YHR060W	VMA22
YDR225W	HTA1	YEL056W	HAT2
YJL060W	BNA3	YDR254W	CHL4
YDR293C	SSD1	YGL237C	HAP2
YAL051W	OAF1	YDR076W	RAD55
YBR200W	BEM1	YDR254W	CHL4
YAL024C	LTE1	YKL126W	YPK1
YPL057C	SUR1	YAL002W	VPS8
YJL172W	CPS1	YDR261C	EXG2
YNL307C	MCK1	YBR280C	SAF1
YOR195W	SLK19	YLR058C	SHM2

Predictions at threshold level 0.75 continued...			
**Gene1 of predicted pair**		**Gene2 of predicted pair**	
**Systematic Name**	**Standard Name**	**Systematic Name**	**Standard Name**
YNL248C	RPA49	YOL054W	PSH1
YNL288W	CAF40	YDR183W	PLP1
YER107C	GLE2	YPR119W	CLB2
YHL031C	GOS1	YOR038C	HIR2
YDR363W-A	SEM1	YDR318W	MCM21
YJL036W	SNX4	YDR153C	ENT5
YFR010W	UBP6	YDL047W	SIT4
YLL007C	YLL007C	YDR293C	SSD1
YOR368W	RAD17	YJR035W	RAD26
YFR010W	UBP6	YBL103C	RTG3
YPL213W	LEA1	YJL006C	CTK2
YOL051W	GAL11	YCR084C	TUP1
YPL057C	SUR1	YNL238W	KEX2
YGL124C	MON1	YDL226C	GCS1
YPL037C	EGD1	YJR084W	CSN12
YBR200W	BEM1	YGL253W	HXK2
YKL048C	ELM1	YDR310C	SUM1
YJL183W	MNN11	YBL072C	RPS8A
YDR254W	CHL4	YOR141C	ARP8
YNL107W	YAF9	YHR115C	DMA1
YGR142W	BTN2	YER074W-A	YOS1
YKR001C	VPS1	YBR283C	SSH1
YPL234C	TFP3	YNL107W	YAF9
YBL102W	SFT2	YCR066W	RAD18
YDL223C	HBT1	YKL048C	ELM1
YOR127W	RGA1	YDR289C	RTT103
YOR195W	SLK19	YKR061W	KTR2
YIL053W	RHR2	YGL115W	SNF4
YBR215W	HPC2	YLR320W	MMS22

Predictions at threshold level 0.75 continued...			
Gene1 of predicted pair		Gene2 of predicted pair	
Systematic Name	Standard Name	Systematic Name	Standard Name
YLR083C	EMP70	YOR106W	VAM3
YPL129W	TAF14	YEL044W	IES6
YBR173C	UMP1	YPL195W	APL5
YJL154C	VPS35	YEL036C	ANP1
YDR293C	SSD1	YER162C	RAD4
YNL087W	TCB2	YOL081W	IRA2
YGR055W	MUP1	YPL240C	HSP82
YDR137W	RGP1	YPL161C	BEM4
YGR249W	MGA1	YMR230W	RPS10B
YIL034C	CAP2	YLR292C	SEC72
YPR040W	TIP41	YPL161C	BEM4
YBR111W-A	SUS1	YPL248C	GAL4
YBL021C	HAP3	YOR358W	HAP5
YAL024C	LTE1	YDL002C	NHP10
YCL037C	SRO9	YPL195W	APL5
YOR023C	AHC1	YPR051W	MAK3
YIL159W	BNR1	YLR386W	VAC14
YNL297C	MON2	YNL238W	KEX2
YOR275C	RIM20	YJL095W	BCK1
YER044C	ERG28	YDL192W	ARF1
YBR030W	YBR030W	YMR133W	REC114
YJR084W	CSN12	YML097C	VPS9
YDR335W	MSN5	YER105C	NUP157
YJL157C	FAR1	YJL187C	SWE1
YDL184C	RPL41A	YBR036C	CSG2
YDR293C	SSD1	YDL077C	VAM6
YDR144C	MKC7	YJL095W	BCK1
YPL178W	CBC2	YOR358W	HAP5
YLR192C	HCR1	YDR261C	EXG2

Predictions at threshold level 0.75 continued...			
**Gene1 of predicted pair**		**Gene2 of predicted pair**	
**Systematic Name**	**Standard Name**	**Systematic Name**	**Standard Name**
YMR224C	MRE11	YDL077C	VAM6
YJR140C	HIR3	YNL107W	YAF9
YDR283C	GCN2	YPR119W	CLB2
YMR153W	NUP53	YER139C	YER139C
YGL200C	EMP24	YJL095W	BCK1
YEL009C	GCN4	YBL008W	HIR1
YDL020C	RPN4	YDR293C	SSD1
YGL163C	RAD54	YDR289C	RTT103
YFR010W	UBP6	YNR072W	HXT17
YLR441C	RPS1A	YDR261C	EXG2
YLR386W	VAC14	YML115C	VAN1
YBR031W	RPL4A	YGL246C	RAI1
YPL121C	MEI5	YHR079C-A	SAE3
YMR199W	CLN1	YOR008C	SLG1
YGL163C	RAD54	YBR036C	CSG2
YOR327C	SNC2	YOL081W	IRA2
YKL010C	UFD4	YBL047C	EDE1
YGL115W	SNF4	YDL006W	PTC1
YMR039C	SUB1	YPR120C	CLB5
YPR084W	YPR084W	YDR245W	MNN10
YER116C	SLX8	YHR081W	LRP1
YGL215W	CLG1	YKL041W	VPS24
YGL147C	RPL9A	YPL037C	EGD1
YNL069C	RPL16B	YOR327C	SNC2
YOR112W	CEX1	YGL223C	COG1
YKR057W	RPS21A	YJL095W	BCK1
YGL148W	ARO2	YDR310C	SUM1
YML095C	RAD10	YHR200W	RPN10
YLR292C	SEC72	YOR127W	RGA1

Predictions at threshold level 0.75 continued...			
Gene1 of predicted pair		Gene2 of predicted pair	
Systematic Name	Standard Name	Systematic Name	Standard Name
YJL124C	LSM1	YDL002C	NHP10
YPL253C	VIK1	YMR109W	MYO5
YKL126W	YPK1	YPL057C	SUR1
YGL078C	DBP3	YNL032W	SIW14
YBR108W	YBR108W	YJR135C	MCM22
YPL253C	VIK1	YDR378C	LSM6
YLR371W	ROM2	YMR214W	SCJ1
YKR061W	KTR2	YPL152W	RRD2
YDL184C	RPL41A	YBL103C	RTG3
YDR378C	LSM6	YGR135W	PRE9
YKR001C	VPS1	YOR014W	RTS1
YJL058C	BIT61	YBR215W	HPC2
YGL025C	PGD1	YPR046W	MCM16
YMR179W	SPT21	YLR429W	CRN1
YBR107C	IML3	YJR032W	CPR7
YJR125C	ENT3	YNR006W	VPS27
YLR360W	VPS38	YDL077C	VAM6
YDL154W	MSH5	YPR119W	CLB2
YDR156W	RPA14	YNL106C	INP52
YNL243W	SLA2	YIL034C	CAP2
YOR043W	WHI2	YEL053C	MAK10
YMR172W	HOT1	YHR178W	STB5
YDR477W	SNF1	YPL195W	APL5
YLR288C	MEC3	YLR435W	TSR2
YLR381W	CTF3	YGL003C	CDH1
YNL106C	INP52	YJR063W	RPA12
YHR178W	STB5	YIL008W	URM1
YGR108W	CLB1	YDR310C	SUM1
YLR254C	NDL1	YML112W	CTK3

Predictions at threshold level 0.75 continued...			
Gene1 of predicted pair		Gene2 of predicted pair	
Systematic Name	Standard Name	Systematic Name	Standard Name
YOR038C	HIR2	YOR368W	RAD17
YOR337W	TEA1	YMR230W	RPS10B
YLR292C	SEC72	YGR188C	BUB1
YFR043C	IRC6	YLR248W	RCK2
YGL212W	VAM7	YLR398C	SKI2
YFL003C	MSH4	YDL154W	MSH5
YPR046W	MCM16	YLR032W	RAD5
YBL019W	APN2	YMR038C	CCS1
YLL026W	HSP104	YNL041C	COG6
YBR131W	CCZ1	YMR004W	MVP1
YGL151W	NUT1	YGL124C	MON1
YIL159W	BNR1	YBR030W	YBR030W
YNL032W	SIW14	YDR310C	SUM1
YMR142C	RPL13B	YER162C	RAD4
YDL101C	DUN1	YNL016W	PUB1
YKL139W	CTK1	YDR318W	MCM21
YNL054W	VAC7	YLR182W	SWI6
YNL087W	TCB2	YGL115W	SNF4
YML103C	NUP188	YJR059W	PTK2
YPL129W	TAF14	YOL018C	TLG2
YPL001W	HAT1	YDR076W	RAD55
YGL147C	RPL9A	YER139C	YER139C
YPL120W	VPS30	YER122C	GLO3
YDR202C	RAV2	YNL107W	YAF9
YBL102W	SFT2	YOR106W	VAM3
YDR507C	GIN4	YHR152W	SPO12
YHR012W	VPS29	YBR283C	SSH1
YPL115C	BEM3	YGL087C	MMS2
YBR194W	SOY1	YHL031C	GOS1

Predictions at threshold level 0.75 continued...			
Gene1 of predicted pair		Gene2 of predicted pair	
Systematic Name	Standard Name	Systematic Name	Standard Name
YDR146C	SWI5	YOL051W	GAL11
YJL124C	LSM1	YOR368W	RAD17
YPL137C	GIP3	YDL077C	VAM6
YBL047C	EDE1	YKR001C	VPS1
YNL041C	COG6	YOL018C	TLG2
YNL206C	RTT106	YKL101W	HSL1
YBR173C	UMP1	YGL003C	CDH1
YJR058C	APS2	YOR035C	SHE4
YHR081W	LRP1	YJL095W	BCK1
YGL087C	MMS2	YGR270W	YTA7
YIL034C	CAP2	YBR114W	RAD16
YLR234W	TOP3	YJL190C	RPS22A
YBR057C	MUM2	YOR035C	SHE4
YJL176C	SWI3	YOL081W	IRA2
YMR142C	RPL13B	YBR280C	SAF1
YCR084C	TUP1	YKL181W	PRS1
YMR016C	SOK2	YOR066W	MSA1
YLR429W	CRN1	YPL018W	CTF19
YNL201C	PSY2	YNL215W	IES2
YBR228W	SLX1	YPL079W	RPL21B
YDR477W	SNF1	YML062C	MFT1
YDL020C	RPN4	YNL291C	MID1
YDR192C	NUP42	YBL079W	NUP170
YCR020C-A	MAK31	YMR004W	MVP1
YDR320C	SWA2	YOL018C	TLG2
YMR199W	CLN1	YOR358W	HAP5
YHL031C	GOS1	YJR060W	CBF1
YDR507C	GIN4	YBR267W	REI1
YNL223W	ATG4	YPR119W	CLB2

Predictions at threshold level 0.75 continued...			
Gene1 of predicted pair		Gene2 of predicted pair	
Systematic Name	Standard Name	Systematic Name	Standard Name
YLR292C	SEC72	YLR360W	VPS38
YKR072C	SIS2	YBR082C	UBC4
YGL147C	RPL9A	YHR060W	VMA22
YPR018W	RLF2	YDR227W	SIR4
YBL067C	UBP13	YLR425W	TUS1
YDR146C	SWI5	YOR014W	RTS1
YBL051C	PIN4	YMR127C	SAS2
YPL161C	BEM4	YJR090C	GRR1
YDL223C	HBT1	YBL047C	EDE1
YJL124C	LSM1	YDR200C	VPS64
YER087C-B	SBH1	YLR055C	SPT8
YMR202W	ERG2	YJL006C	CTK2
YBR228W	SLX1	YBR082C	UBC4
YOR080W	DIA2	YNL107W	YAF9
YEL042W	GDA1	YDR310C	SUM1
YBL079W	NUP170	YKL068W	NUP100
YPL037C	EGD1	YMR230W	RPS10B
YDR335W	MSN5	YFR030W	MET10
YMR026C	PEX12	YDL083C	RPS16B
YNL307C	MCK1	YNL041C	COG6
YLR119W	SRN2	YLR320W	MMS22
YDR507C	GIN4	YER027C	GAL83
YBR274W	CHK1	YBR195C	MSI1
YMR004W	MVP1	YNL051W	COG5
YOR195W	SLK19	YDL226C	GCS1
YBR108W	YBR108W	YLR447C	VMA6
YPL253C	VIK1	YNL225C	CNM67
YNL325C	FIG4	YOR290C	SNF2
YLR360W	VPS38	YNL051W	COG5

Predictions at threshold level 0.75 continued...			
Gene1 of predicted pair		Gene2 of predicted pair	
Systematic Name	Standard Name	Systematic Name	Standard Name
YDR080W	VPS41	YDR323C	PEP7
YBR173C	UMP1	YBL103C	RTG3
YBL102W	SFT2	YNL238W	KEX2
YDL184C	RPL41A	YDL077C	VAM6
YDR378C	LSM6	YNL107W	YAF9
YJR035W	RAD26	YOR014W	RTS1
YKL160W	ELF1	YNL236W	SIN4
YLR170C	APS1	YPR029C	APL4
YAL024C	LTE1	YGR270W	YTA7
YDL020C	RPN4	YBR290W	BSD2
YDR440W	DOT1	YNL107W	YAF9
YHR039C-A	VMA10	YNL238W	KEX2
YBR036C	CSG2	YMR214W	SCJ1
YDL005C	MED2	YKR048C	NAP1
YMR026C	PEX12	YKR035W-A	DID2
YER158C	YER158C	YAR014C	BUD14
YBR030W	YBR030W	YER162C	RAD4
YEL050C	RML2	YDR200C	VPS64
YML115C	VAN1	YDL192W	ARF1
YCR034W	FEN1	YMR167W	MLH1
YPL046C	ELC1	YJR052W	RAD7
YMR311C	GLC8	YOR014W	RTS1
YBL021C	HAP3	YKL048C	ELM1
YDR137W	RGP1	YKL160W	ELF1
YKR020W	VPS51	YBL072C	RPS8A
YGL087C	MMS2	YJR135C	MCM22
YPR036W	VMA13	YHR081W	LRP1
YGL003C	CDH1	YDL226C	GCS1
YPL042C	SSN3	YEL050C	RML2

Predictions at threshold level 0.75 continued...			
Gene1 of predicted pair		Gene2 of predicted pair	
Systematic Name	Standard Name	Systematic Name	Standard Name
YPL057C	SUR1	YHR200W	RPN10
YJR056C	YJR056C	YNL238W	KEX2
YDR156W	RPA14	YNL307C	MCK1
YPR051W	MAK3	YEL056W	HAT2
YMR127C	SAS2	YJR135C	MCM22
YPR017C	DSS4	YCL037C	SRO9
YKL116C	PRR1	YER139C	YER139C
YNL297C	MON2	YMR054W	STV1
YKR099W	BAS1	YLR452C	SST2
YPL022W	RAD1	YJL124C	LSM1
YGL147C	RPL9A	YJL172W	CPS1
YNL329C	PEX6	YBL101C	ECM21
YDR469W	SDC1	YJL140W	RPB4
YLR090W	XDJ1	YER144C	UBP5
YBR126C	TPS1	YJL172W	CPS1
YGR270W	YTA7	YIR005W	IST3
YDR153C	ENT5	YDL137W	ARF2
YML028W	TSA1	YBR082C	UBC4
YER144C	UBP5	YMR133W	REC114
YER116C	SLX8	YPL057C	SUR1
YJL183W	MNN11	YMR214W	SCJ1
YEL027W	CUP5	YJL183W	MNN11
YDL002C	NHP10	YGL151W	NUT1
YCR020C-A	MAK31	YKR020W	VPS51
YPL105C	YPL105C	YJL004C	SYS1
YBL088C	TEL1	YJL106W	IME2
YGL174W	BUD13	YDR469W	SDC1
YHR061C	GIC1	YCR086W	CSM1
YGR270W	YTA7	YFR036W	CDC26

Predictions at threshold level 0.75 continued...			
Gene1 of predicted pair		Gene2 of predicted pair	
Systematic Name	Standard Name	Systematic Name	Standard Name
YLR079W	SIC1	YJL124C	LSM1
YKL025C	PAN3	YBR200W	BEM1
YCR020C-A	MAK31	YBL102W	SFT2
YJR099W	YUH1	YLR306W	UBC12
YLR079W	SIC1	YKL139W	CTK1
YLR386W	VAC14	YDR155C	CPR1
YOR047C	STD1	YDR477W	SNF1
YML102W	CAC2	YGL163C	RAD54
YCR020C-A	MAK31	YEL051W	VMA8
YDL184C	RPL41A	YDR358W	GGA1
YOR337W	TEA1	YEL060C	PRB1
YIL076W	SEC28	YHR200W	RPN10
YLR058C	SHM2	YBR107C	IML3
YLR324W	PEX30	YMR204C	INP1
YLR381W	CTF3	YJL183W	MNN11
YBR108W	YBR108W	YLR370C	ARC18
YMR205C	PFK2	YDR245W	MNN10
YHR012W	VPS29	YCR086W	CSM1
YBR131W	CCZ1	YPL161C	BEM4
YER143W	DDI1	YOL018C	TLG2
YLR094C	GIS3	YLR093C	NYV1
YOL030W	GAS5	YJR035W	RAD26
YJL154C	VPS35	YDL155W	CLB3
YBR173C	UMP1	YMR230W	RPS10B
YDR329C	PEX3	YDR265W	PEX10
YKL116C	PRR1	YGL003C	CDH1
YLR370C	ARC18	YDL226C	GCS1
YMR198W	CIK1	YDR254W	CHL4
YGL060W	YBP2	YPL106C	SSE1

Predictions at threshold level 0.75 continued...			
Gene1 of predicted pair		Gene2 of predicted pair	
Systematic Name	Standard Name	Systematic Name	Standard Name
YOR043W	WHI2	YDL006W	PTC1
YEL044W	IES6	YMR089C	YTA12
YKR001C	VPS1	YHR039C-A	VMA10
YOL111C	MDY2	YDL077C	VAM6
YBL103C	RTG3	YIL133C	RPL16A
YBR057C	MUM2	YPL057C	SUR1
YHR012W	VPS29	YGR188C	BUB1
YML095C	RAD10	YBL087C	RPL23A
YGL025C	PGD1	YER122C	GLO3
YPR084W	YPR084W	YNL025C	SSN8
YDR137W	RGP1	YDR207C	UME6
YBR019C	GAL10	YER122C	GLO3
YBL008W	HIR1	YDR225W	HTA1
YLR399C	BDF1	YDR289C	RTT103
YLR095C	IOC2	YDR137W	RGP1
YOR080W	DIA2	YML095C	RAD10
YNL025C	SSN8	YER139C	YER139C
YPL057C	SUR1	YBR036C	CSG2
YDL223C	HBT1	YNL307C	MCK1
YPL022W	RAD1	YLR381W	CTF3
YJL176C	SWI3	YHR167W	THP2
YBR107C	IML3	YCR086W	CSM1
YDL175C	AIR2	YDR378C	LSM6
YLR452C	SST2	YOL081W	IRA2
YLR371W	ROM2	YOR014W	RTS1
YFR031C-A	RPL2A	YHR060W	VMA22
YLR234W	TOP3	YER162C	RAD4
YDL052C	SLC1	YOR059C	YOR059C
YOR083W	WHI5	YML095C	RAD10

Predictions at threshold level 0.75 continued...			
Gene1 of predicted pair		Gene2 of predicted pair	
Systematic Name	Standard Name	Systematic Name	Standard Name
YDL006W	PTC1	YDL192W	ARF1
YML012W	ERV25	YKR014C	YPT52
YPR131C	NAT3	YGL003C	CDH1
YGR188C	BUB1	YPR119W	CLB2
YOR195W	SLK19	YDR260C	SWM1
YLL039C	UBI4	YJL006C	CTK2
YGL135W	RPL1B	YKR014C	YPT52
YDR448W	ADA2	YNL107W	YAF9
YKL048C	ELM1	YDR254W	CHL4
YIL151C	YIL151C	YPL240C	HSP82
YPL024W	RMI1	YDL155W	CLB3
YJL149W	YJL149W	YML088W	UFO1
YMR055C	BUB2	YGL253W	HXK2
YNL297C	MON2	YPL161C	BEM4
YIL053W	RHR2	YMR153W	NUP53
YKL002W	DID4	YKL041W	VPS24
YLR090W	XDJ1	YLR093C	NYV1
YNL016W	PUB1	YCR086W	CSM1
YMR004W	MVP1	YDL077C	VAM6
YLR399C	BDF1	YOL054W	PSH1
YPL213W	LEA1	YJL183W	MNN11
YDR004W	RAD57	YDR146C	SWI5
YMR192W	GYL1	YBR108W	YBR108W
YKL116C	PRR1	YPR046W	MCM16
YKL032C	IXR1	YIL008W	URM1
YMR198W	CIK1	YOR141C	ARP8
YDR137W	RGP1	YNL307C	MCK1
YLR119W	SRN2	YER139C	YER139C
YBR290W	BSD2	YMR055C	BUB2

Predictions at threshold level 0.75 continued...			
Gene1 of predicted pair		Gene2 of predicted pair	
Systematic Name	Standard Name	Systematic Name	Standard Name
YKL117W	SBA1	YML057W	CMP2
YMR026C	PEX12	YMR142C	RPL13B
YPL024W	RMI1	YGL066W	SGF73
YFR031C-A	RPL2A	YOR132W	VPS17
YLR337C	VRP1	YAR014C	BUD14
YHR081W	LRP1	YOL121C	RPS19A
YLR410W	VIP1	YLR306W	UBC12
YIL148W	RPL40A	YNL199C	GCR2
YGR188C	BUB1	YDR207C	UME6
YLR090W	XDJ1	YOL121C	RPS19A
YER144C	UBP5	YCR053W	THR4
YJL004C	SYS1	YNL051W	COG5
YMR224C	MRE11	YHR200W	RPN10
YBR200W	BEM1	YMR199W	CLN1
YFR010W	UBP6	YPL253C	VIK1
YMR038C	CCS1	YML028W	TSA1
YDR004W	RAD57	YMR039C	SUB1
YDR176W	NGG1	YPL248C	GAL4
YGL212W	VAM7	YJR033C	RAV1
YER040W	GLN3	YDR207C	UME6
YNL215W	IES2	YOR213C	SAS5
YJL154C	VPS35	YOR035C	SHE4
YOR069W	VPS5	YPL161C	BEM4
YJL124C	LSM1	YOR043W	WHI2
YPR051W	MAK3	YDR153C	ENT5
YLR381W	CTF3	YOR358W	HAP5
YBR108W	YBR108W	YKL126W	YPK1
YLR447C	VMA6	YKR020W	VPS51
YPR036W	VMA13	YKL048C	ELM1

Predictions at threshold level 0.75 continued...			
Gene1 of predicted pair		Gene2 of predicted pair	
Systematic Name	Standard Name	Systematic Name	Standard Name
YOL051W	GAL11	YNR023W	SNF12
YKL010C	UFD4	YPL161C	BEM4
YMR199W	CLN1	YGR059W	SPR3
YLR337C	VRP1	YER139C	YER139C
YJL183W	MNN11	YBL025W	RRN10
YLR234W	TOP3	YMR167W	MLH1
YBR112C	CYC8	YCR084C	TUP1
YOR195W	SLK19	YBL087C	RPL23A
YGR261C	APL6	YHR039C-A	VMA10
YJL036W	SNX4	YOL108C	INO4
YNL025C	SSN8	YER122C	GLO3
YMR198W	CIK1	YBL047C	EDE1
YER161C	SPT2	YER122C	GLO3
YGR105W	VMA21	YPL120W	VPS30
YOR080W	DIA2	YJL187C	SWE1
YDR146C	SWI5	YBR260C	RGD1
YBR200W	BEM1	YER044C	ERG28
YBL079W	NUP170	YGL016W	KAP122
YHR157W	REC104	YHL022C	SPO11
YJR084W	CSN12	YJL095W	BCK1
YPR120C	CLB5	YGL252C	RTG2
YDR293C	SSD1	YNL044W	YIP3
YMR202W	ERG2	YDR318W	MCM21
YPL253C	VIK1	YLR371W	ROM2
YLL007C	YLL007C	YER110C	KAP123
YOR076C	SKI7	YLR398C	SKI2
YBR107C	IML3	YAR014C	BUD14
YPL115C	BEM3	YBR171W	SEC66
YER044C	ERG28	YLR180W	SAM1

Predictions at threshold level 0.75 continued...			
Gene1 of predicted pair		Gene2 of predicted pair	
Systematic Name	Standard Name	Systematic Name	Standard Name
YNR052C	POP2	YKL110C	KTI12
YKR020W	VPS51	YHR115C	DMA1
YPR036W	VMA13	YLR320W	MMS22
YMR133W	REC114	YDL192W	ARF1
YLR337C	VRP1	YEL036C	ANP1
YMR016C	SOK2	YNR072W	HXT17
YDR245W	MNN10	YPL089C	RLM1
YNL201C	PSY2	YLR381W	CTF3
YIL034C	CAP2	YDR389W	SAC7
YML115C	VAN1	YFR036W	CDC26
YJR059W	PTK2	YMR273C	ZDS1
YLR386W	VAC14	YOR035C	SHE4
YHR084W	STE12	YBL016W	FUS3
YDL101C	DUN1	YBL088C	TEL1
YGL250W	RMR1	YGL019W	CKB1
YCR024C-A	PMP1	YMR273C	ZDS1
YLR119W	SRN2	YMR133W	REC114
YDR378C	LSM6	YJL183W	MNN11
YMR109W	MYO5	YCR086W	CSM1
YMR198W	CIK1	YJL006C	CTK2
YHR134W	WSS1	YGL253W	HXK2
YJR033C	RAV1	YDR207C	UME6
YOR213C	SAS5	YER139C	YER139C
YPL220W	RPL1A	YKR026C	GCN3
YDR363W-A	SEM1	YNL236W	SIN4
YHR039C-A	VMA10	YGL124C	MON1
YLR288C	MEC3	YGR081C	SLX9
YFR010W	UBP6	YLR182W	SWI6
YCL037C	SRO9	YEL053C	MAK10

Predictions at threshold level 0.75 continued...			
Gene1 of predicted pair		Gene2 of predicted pair	
Systematic Name	Standard Name	Systematic Name	Standard Name
YLR192C	HCR1	YHR081W	LRP1
YGR020C	VMA7	YJR060W	CBF1
YBR171W	SEC66	YCR086W	CSM1
YJR140C	HIR3	YJR032W	CPR7
YOR069W	VPS5	YDR202C	RAV2
YKL032C	IXR1	YKL041W	VPS24
YCR024C-A	PMP1	YML057W	CMP2
YOR132W	VPS17	YLR381W	CTF3
YBR171W	SEC66	YNL041C	COG6
YBR108W	YBR108W	YHR200W	RPN10
YPL234C	TFP3	YNL041C	COG6
YLR170C	APS1	YHL019C	APM2
YMR224C	MRE11	YER122C	GLO3
YGL237C	HAP2	YEL053C	MAK10
YHR134W	WSS1	YOL030W	GAS5
YCR020C-A	MAK31	YJL053W	PEP8
YKL048C	ELM1	YER122C	GLO3
YLR288C	MEC3	YGL148W	ARO2
YBR200W	BEM1	YMR311C	GLC8
YLR079W	SIC1	YKR061W	KTR2
YGL190C	CDC55	YPL152W	RRD2
YKR059W	TIF1	YGL019W	CKB1
YKR099W	BAS1	YER040W	GLN3
YBR131W	CCZ1	YGR135W	PRE9
YER114C	BOI2	YDR309C	GIC2
YOR112W	CEX1	YCR066W	RAD18
YPL024W	RMI1	YNL076W	MKS1
YMR142C	RPL13B	YHR200W	RPN10
YOR332W	VMA4	YDR137W	RGP1

Predictions at threshold level 0.75 continued...			
Gene1 of predicted pair		Gene2 of predicted pair	
Systematic Name	Standard Name	Systematic Name	Standard Name
YGL095C	VPS45	YJL164C	TPK1
YKR099W	BAS1	YDL192W	ARF1
YFL026W	STE2	YOR109W	INP53
YAR014C	BUD14	YDR207C	UME6
YML115C	VAN1	YEL060C	PRB1
YCL037C	SRO9	YER120W	SCS2
YGR275W	RTT102	YOR290C	SNF2
YOL018C	TLG2	YDR207C	UME6
YJL154C	VPS35	YGR261C	APL6
YHR052W	CIC1	YER105C	NUP157
YFR036W	CDC26	YML071C	COG8
YDR329C	PEX3	YDR142C	PEX7
YMR224C	MRE11	YKR095W	MLP1
YDR080W	VPS41	YGL124C	MON1
YHR156C	LIN1	YER044C	ERG28
YBR278W	DPB3	YBR274W	CHK1
YLR337C	VRP1	YKL126W	YPK1
YFR031C-A	RPL2A	YPL234C	TFP3
YKL101W	HSL1	YGL031C	RPL24A
YHR013C	ARD1	YPR046W	MCM16
YHR156C	LIN1	YPL195W	APL5
YKL139W	CTK1	YNL225C	CNM67
YBR126C	TPS1	YMR152W	YIM1
YNL248C	RPA49	YGR270W	YTA7
YJL154C	VPS35	YBR260C	RGD1
YDR004W	RAD57	YJR090C	GRR1
YPL037C	EGD1	YEL053C	MAK10
YLR192C	HCR1	YLR398C	SKI2
YMR016C	SOK2	YPL161C	BEM4

Predictions at threshold level 0.75 continued...			
Gene1 of predicted pair		Gene2 of predicted pair	
Systematic Name	Standard Name	Systematic Name	Standard Name
YOR106W	VAM3	YER096W	SHC1
YMR095C	SNO1	YPL037C	EGD1
YLR064W	YLR064W	YHR114W	BZZ1
YHR111W	UBA4	YNL079C	TPM1
YHR060W	VMA22	YNL076W	MKS1
YGL066W	SGF73	YJR135C	MCM22
YBR290W	BSD2	YAL002W	VPS8
YJL124C	LSM1	YOL090W	MSH2
YHR178W	STB5	YPL253C	VIK1
YBL058W	SHP1	YDR477W	SNF1
YNL058C	YNL058C	YOR066W	MSA1
YDR174W	HMO1	YHR013C	ARD1
YDR254W	CHL4	YDR318W	MCM21
YGL060W	YBP2	YPR131C	NAT3
YPR084W	YPR084W	YPL037C	EGD1
YOL111C	MDY2	YPL137C	GIP3
YGL115W	SNF4	YOR327C	SNC2
YEL065W	SIT1	YOR337W	TEA1
YBR173C	UMP1	YKR035W-A	DID2
YJR056C	YJR056C	YBL085W	BOI1
YBR108W	YBR108W	YMR230W	RPS10B
YNL215W	IES2	YPL253C	VIK1
YGL212W	VAM7	YNL076W	MKS1
YOR358W	HAP5	YPL246C	RBD2
YMR026C	PEX12	YEL053C	MAK10
YJR056C	YJR056C	YMR152W	YIM1
YFR010W	UBP6	YHR060W	VMA22
YBL088C	TEL1	YPR072W	NOT5
YDR443C	SSN2	YKR099W	BAS1

Predictions at threshold level 0.75 continued...			
**Gene1 of predicted pair**		**Gene2 of predicted pair**	
**Systematic Name**	**Standard Name**	**Systematic Name**	**Standard Name**
YPR120C	CLB5	YLR425W	TUS1
YFR031C-A	RPL2A	YEL051W	VMA8
YLR313C	SPH1	YLR362W	STE11
YER162C	RAD4	YGL124C	MON1
YOR195W	SLK19	YMR202W	ERG2
YBL078C	ATG8	YEL013W	VAC8
YMR205C	PFK2	YPL161C	BEM4
YDR309C	GIC2	YGR152C	RSR1
YAR014C	BUD14	YKR030W	GMH1
YHR012W	VPS29	YLR371W	ROM2
YDR108W	GSG1	YHR012W	VPS29
YGL133W	ITC1	YDR363W-A	SEM1
YOR080W	DIA2	YGL031C	RPL24A
YPL120W	VPS30	YNL051W	COG5
YGL178W	MPT5	YNL271C	BNI1
YPR017C	DSS4	YEL053C	MAK10
YDR137W	RGP1	YDL192W	ARF1
YNL218W	MGS1	YLR313C	SPH1
YGR081C	SLX9	YFL023W	BUD27
YNL243W	SLA2	YLR200W	YKE2
YJL124C	LSM1	YDR076W	RAD55
YBR200W	BEM1	YLR399C	BDF1
YJR005W	APL1	YDL226C	GCS1
YNL069C	RPL16B	YOR106W	VAM3
YGR105W	VMA21	YLR309C	IMH1
YDR176W	NGG1	YOR023C	AHC1
YBL051C	PIN4	YIL008W	URM1
YGR104C	SRB5	YBR200W	BEM1
YDR335W	MSN5	YNL236W	SIN4

Predictions at threshold level 0.75 continued...			
**Gene1 of predicted pair**		**Gene2 of predicted pair**	
**Systematic Name**	**Standard Name**	**Systematic Name**	**Standard Name**
YBR031W	RPL4A	YOR078W	BUD21
YKR035W-A	DID2	YLR180W	SAM1
YOR327C	SNC2	YGL124C	MON1
YMR016C	SOK2	YLR093C	NYV1
YBR260C	RGD1	YHR114W	BZZ1
YER087C-B	SBH1	YJR010C-A	SPC1
YOL111C	MDY2	YLR180W	SAM1
YJR135C	MCM22	YCR086W	CSM1
YMR055C	BUB2	YJL187C	SWE1
YER115C	SPR6	YCR024C-A	PMP1
YBL047C	EDE1	YOR043W	WHI2
YMR319C	FET4	YDR105C	TMS1
YCL037C	SRO9	YHL020C	OPI1
YLR095C	IOC2	YNL058C	YNL058C
YNR072W	HXT17	YDL077C	VAM6
YOR270C	VPH1	YGL124C	MON1
YFL026W	STE2	YJR125C	ENT3
YLR135W	SLX4	YMR038C	CCS1
YJL151C	SNA3	YBR108W	YBR108W
YLR083C	EMP70	YCR020C-A	MAK31
YGL174W	BUD13	YER161C	SPT2
YOR327C	SNC2	YLR370C	ARC18
YDR358W	GGA1	YDL077C	VAM6
YKL110C	KTI12	YCR086W	CSM1
YHR111W	UBA4	YDR155C	CPR1
YLR216C	CPR6	YBL103C	RTG3
YMR054W	STV1	YKL119C	VPH2
YOR195W	SLK19	YLR381W	CTF3
YNL058C	YNL058C	YPL195W	APL5

Predictions at threshold level 0.75 continued...			
Gene1 of predicted pair		Gene2 of predicted pair	
Systematic Name	Standard Name	Systematic Name	Standard Name
YER162C	RAD4	YNL076W	MKS1
YDR129C	SAC6	YDR323C	PEP7
YMR127C	SAS2	YGR252W	GCN5
YGR270W	YTA7	YDR144C	MKC7
YDR363W-A	SEM1	YDL155W	CLB3
YCR066W	RAD18	YPL120W	VPS30
YGR108W	CLB1	YOL091W	SPO21
YOR096W	RPS7A	YBR189W	RPS9B
YPL018W	CTF19	YOR141C	ARP8
YGL156W	AMS1	YKL103C	LAP4
YBR290W	BSD2	YER115C	SPR6
YHR161C	YAP1801	YKR001C	VPS1
YKL116C	PRR1	YMR152W	YIM1
YKL214C	YRA2	YOR001W	RRP6
YMR095C	SNO1	YML071C	COG8
YIL076W	SEC28	YDL083C	RPS16B
YOR008C	SLG1	YJL095W	BCK1
YDR129C	SAC6	YEL036C	ANP1
YER011W	TIR1	YPL178W	CBC2
YHR200W	RPN10	YLR032W	RAD5
YOR112W	CEX1	YHL020C	OPI1
YAL024C	LTE1	YFR036W	CDC26
YLR180W	SAM1	YDR207C	UME6
YAL024C	LTE1	YDR383C	NKP1
YPR017C	DSS4	YDL077C	VAM6
YDL160C	DHH1	YOR368W	RAD17
YAL024C	LTE1	YMR179W	SPT21
YER143W	DDI1	YNR049C	MSO1
YGR104C	SRB5	YNL215W	IES2

Predictions at threshold level 0.75 continued...			
Gene1 of predicted pair		Gene2 of predicted pair	
Systematic Name	Standard Name	Systematic Name	Standard Name
YOR101W	RAS1	YDL005C	MED2
YJL036W	SNX4	YDR080W	VPS41
YLR043C	TRX1	YIL008W	URM1
YJL187C	SWE1	YJL095W	BCK1
YAL024C	LTE1	YMR311C	GLC8
YOR337W	TEA1	YJR084W	CSN12
YPL120W	VPS30	YOR327C	SNC2
YJR063W	RPA12	YOL081W	IRA2
YDR443C	SSN2	YML071C	COG8
YOR039W	CKB2	YLR435W	TSR2
YNL041C	COG6	YPL120W	VPS30
YDR183W	PLP1	YHR115C	DMA1
YGL212W	VAM7	YLR452C	SST2
YCR053W	THR4	YBR280C	SAF1
YBR171W	SEC66	YOR035C	SHE4
YJL124C	LSM1	YKR001C	VPS1
YBL051C	PIN4	YKL048C	ELM1
YMR055C	BUB2	YJL138C	TIF2
YMR311C	GLC8	YMR230W	RPS10B
YFR010W	UBP6	YOR035C	SHE4
YGR249W	MGA1	YDR261C	EXG2
YHR098C	SFB3	YOR036W	PEP12
YKR035W-A	DID2	YJR032W	CPR7
YGR252W	GCN5	YNL236W	SIN4
YGL250W	RMR1	YPL018W	CTF19
YJR063W	RPA12	YDR183W	PLP1
YAL024C	LTE1	YPL253C	VIK1
YJL172W	CPS1	YER139C	YER139C
YGL033W	HOP2	YDR207C	UME6

Predictions at threshold level 0.75 continued...			
**Gene1 of predicted pair**		**Gene2 of predicted pair**	
**Systematic Name**	**Standard Name**	**Systematic Name**	**Standard Name**
YEL065W	SIT1	YBR108W	YBR108W
YGR072W	UPF3	YOL108C	INO4
YIL034C	CAP2	YLR233C	EST1
YBL058W	SHP1	YDL077C	VAM6
YLR254C	NDL1	YNL116W	DMA2
YJR024C	MDE1	YMR164C	MSS11
YCR065W	HCM1	YOR080W	DIA2
YOR023C	AHC1	YHL002W	HSE1
YCL001W	RER1	YAL002W	VPS8
YNL206C	RTT106	YDR227W	SIR4
YLR192C	HCR1	YBR036C	CSG2
YAR014C	BUD14	YPR119W	CLB2
YKR020W	VPS51	YJR032W	CPR7
YGL135W	RPL1B	YGR133W	PEX4
YLR386W	VAC14	YCR086W	CSM1
YIL076W	SEC28	YPL234C	TFP3
YMR116C	ASC1	YML115C	VAN1
YJL095W	BCK1	YJL190C	RPS22A
YOR039W	CKB2	YGL250W	RMR1
YIL103W	DPH1	YAR014C	BUD14
YGL147C	RPL9A	YIL076W	SEC28
YKL116C	PRR1	YGL212W	VAM7
YDL192W	ARF1	YLR370C	ARC18
YKR072C	SIS2	YLR313C	SPH1
YBR194W	SOY1	YOR001W	RRP6
YPL253C	VIK1	YDR318W	MCM21
YOR080W	DIA2	YDR378C	LSM6
YGL087C	MMS2	YGL253W	HXK2
YKL048C	ELM1	YGR270W	YTA7

Predictions at threshold level 0.75 continued...			
Gene1 of predicted pair		Gene2 of predicted pair	
Systematic Name	Standard Name	Systematic Name	Standard Name
YDR176W	NGG1	YLR357W	RSC2
YBR171W	SEC66	YLR447C	VMA6
YHR086W	NAM8	YBR119W	MUD1
YIL159W	BNR1	YDL192W	ARF1
YCR081W	SRB8	YOR014W	RTS1
YGL095C	VPS45	YOR327C	SNC2
YDR320C	SWA2	YHL031C	GOS1
YNL325C	FIG4	YDR137W	RGP1
YOR066W	MSA1	YMR311C	GLC8
YKL032C	IXR1	YJL183W	MNN11
YOR066W	MSA1	YDR261C	EXG2
YLR360W	VPS38	YNL107W	YAF9
YMR142C	RPL13B	YDR358W	GGA1
YLR119W	SRN2	YKR099W	BAS1
YBR217W	ATG12	YMR016C	SOK2
YLR150W	STM1	YJR135C	MCM22
YOR106W	VAM3	YHR060W	VMA22
YJR005W	APL1	YPR046W	MCM16
YLR135W	SLX4	YDL013W	SLX5
YLR119W	SRN2	YPR036W	VMA13
YHR111W	UBA4	YHR114W	BZZ1
YFR043C	IRC6	YMR243C	ZRC1
YJL095W	BCK1	YDR310C	SUM1
YDR144C	MKC7	YDR183W	PLP1
YLR234W	TOP3	YOR127W	RGA1
YPR072W	NOT5	YLR357W	RSC2
YBL008W	HIR1	YCR086W	CSM1
YNL215W	IES2	YML112W	CTK3
YLR119W	SRN2	YMR311C	GLC8

Predictions at threshold level 0.75 continued...			
Gene1 of predicted pair		Gene2 of predicted pair	
Systematic Name	Standard Name	Systematic Name	Standard Name
YJL020C	BBC1	YGL095C	VPS45
YJR063W	RPA12	YNL116W	DMA2
YGL066W	SGF73	YDL013W	SLX5
YOR033C	EXO1	YCR077C	PAT1
YPL057C	SUR1	YPL195W	APL5
YBR173C	UMP1	YGL025C	PGD1
YNL288W	CAF40	YLR360W	VPS38
YDR484W	VPS52	YBL103C	RTG3
YBR217W	ATG12	YDL083C	RPS16B
YGL087C	MMS2	YJL124C	LSM1
YLL007C	YLL007C	YJL154C	VPS35
YLR109W	AHP1	YER042W	MXR1
YJL183W	MNN11	YJL190C	RPS22A
YLR095C	IOC2	YML115C	VAN1
YCL067C	HMLALPHA2	YPL240C	HSP82
YLR090W	XDJ1	YBL103C	RTG3
YML103C	NUP188	YKL048C	ELM1
YMR106C	YKU80	YML028W	TSA1
YFR031C-A	RPL2A	YPR046W	MCM16
YDR469W	SDC1	YDR155C	CPR1
YPR046W	MCM16	YDR155C	CPR1
YFR010W	UBP6	YML028W	TSA1
YCR034W	FEN1	YGL250W	RMR1
YDR477W	SNF1	YPL256C	CLN2
YOR127W	RGA1	YDL006W	PTC1
YJL029C	VPS53	YGL095C	VPS45
YHL031C	GOS1	YNL041C	COG6
YHR013C	ARD1	YDR183W	PLP1
YER116C	SLX8	YLR410W	VIP1

Predictions at threshold level 0.75 continued...			
Gene1 of predicted pair		Gene2 of predicted pair	
Systematic Name	Standard Name	Systematic Name	Standard Name
YNL307C	MCK1	YML071C	COG8
YPL240C	HSP82	YER100W	UBC6
YDR225W	HTA1	YNR072W	HXT17
YNL206C	RTT106	YEL056W	HAT2
YPL161C	BEM4	YDL192W	ARF1
YBR260C	RGD1	YJL095W	BCK1
YHR052W	CIC1	YLR150W	STM1
YPL042C	SSN3	YJL127C	SPT10
YKL110C	KTI12	YBL071W-A	KTI11
YHR160C	PEX18	YLR191W	PEX13
YIL159W	BNR1	YDR137W	RGP1
YPL129W	TAF14	YNL031C	HHT2
YPL050C	MNN9	YOR035C	SHE4
YHL031C	GOS1	YJL004C	SYS1
YDR004W	RAD57	YMR231W	PEP5
YDR318W	MCM21	YGL003C	CDH1
YIL036W	CST6	YNL307C	MCK1
YEL053C	MAK10	YEL056W	HAT2
YBL008W	HIR1	YMR167W	MLH1
YGR249W	MGA1	YMR214W	SCJ1
YPR072W	NOT5	YDR071C	PAA1
YNL307C	MCK1	YML028W	TSA1
YDR245W	MNN10	YDR456W	NHX1
YHR039C-A	VMA10	YOR038C	HIR2
YBR200W	BEM1	YBL103C	RTG3
YLR292C	SEC72	YMR055C	BUB2
YDR293C	SSD1	YOR127W	RGA1
YLR192C	HCR1	YLR370C	ARC18
YNL307C	MCK1	YOL111C	MDY2

Predictions at threshold level 0.75 continued...			
Gene1 of predicted pair		Gene2 of predicted pair	
Systematic Name	Standard Name	Systematic Name	Standard Name
YGL178W	MPT5	YCR009C	RVS161
YLR093C	NYV1	YML071C	COG8
YPL253C	VIK1	YGR270W	YTA7
YPR120C	CLB5	YKL161C	YKL161C
YOR132W	VPS17	YPL037C	EGD1
YOL044W	PEX15	YAL055W	PEX22
YPL057C	SUR1	YLR315W	NKP2
YPR029C	APL4	YJR125C	ENT3
YGL212W	VAM7	YCL037C	SRO9
YNL291C	MID1	YPR120C	CLB5
YDR225W	HTA1	YLR381W	CTF3
YFR031C-A	RPL2A	YGL223C	COG1
YMR080C	NAM7	YNL218W	MGS1
YDR309C	GIC2	YOR327C	SNC2
YJR135C	MCM22	YJR032W	CPR7
YJL183W	MNN11	YLR233C	EST1
YCR077C	PAT1	YLR350W	ORM2
YMR224C	MRE11	YBR228W	SLX1
YER116C	SLX8	YHR013C	ARD1
YLR337C	VRP1	YPL050C	MNN9
YDL184C	RPL41A	YBR200W	BEM1
YNR072W	HXT17	YOR043W	WHI2
YBL071W-A	KTI11	YIL103W	DPH1
YOL111C	MDY2	YKR035W-A	DID2
YOR043W	WHI2	YDR469W	SDC1
YJL127C	SPT10	YJL124C	LSM1
YAL024C	LTE1	YDR335W	MSN5
YPL018W	CTF19	YKL114C	APN1
YLR119W	SRN2	YPR046W	MCM16

Predictions at threshold level 0.75 continued...			
**Gene1 of predicted pair**		**Gene2 of predicted pair**	
**Systematic Name**	**Standard Name**	**Systematic Name**	**Standard Name**
YDR080W	VPS41	YHR060W	VMA22
YBR107C	IML3	YDR254W	CHL4
YIL156W	UBP7	YNL199C	GCR2
YJR060W	CBF1	YHR012W	VPS29
YDL083C	RPS16B	YHR081W	LRP1
YHR156C	LIN1	YLR398C	SKI2
YBR042C	CST26	YGL215W	CLG1
YLR254C	NDL1	YAR014C	BUD14
YER114C	BOI2	YBR200W	BEM1
YJL172W	CPS1	YNL041C	COG6
YOR043W	WHI2	YHR158C	KEL1
YJL176C	SWI3	YGR275W	RTT102
YBR194W	SOY1	YPL018W	CTF19
YDR443C	SSN2	YPL042C	SSN3
YOR043W	WHI2	YBR280C	SAF1
YCR066W	RAD18	YHR012W	VPS29
YLR079W	SIC1	YPR046W	MCM16
YKL025C	PAN3	YBL021C	HAP3
YNL054W	VAC7	YGR188C	BUB1
YDR295C	HDA2	YDR318W	MCM21
YPL024W	RMI1	YMR055C	BUB2
YOR290C	SNF2	YML001W	YPT7
YLR095C	IOC2	YPL037C	EGD1
YLL007C	YLL007C	YOL067C	RTG1
YKR099W	BAS1	YLR370C	ARC18
YBR127C	VMA2	YKL041W	VPS24
YIL053W	RHR2	YML071C	COG8
YDR379W	RGA2	YHR111W	UBA4
YFR036W	CDC26	YGL003C	CDH1

Predictions at threshold level 0.75 continued...			
Gene1 of predicted pair		Gene2 of predicted pair	
Systematic Name	Standard Name	Systematic Name	Standard Name
YCR086W	CSM1	YEL056W	HAT2
YER144C	UBP5	YOR035C	SHE4
YBR111W-A	SUS1	YNL030W	HHF2
YMR224C	MRE11	YMR199W	CLN1
YGL033W	HOP2	YMR153W	NUP53
YNL069C	RPL16B	YKR030W	GMH1
YLR094C	GIS3	YDR358W	GGA1
YDR245W	MNN10	YGL200C	EMP24
YKL025C	PAN3	YPL161C	BEM4
YKL139W	CTK1	YKR099W	BAS1
YFR010W	UBP6	YDR261C	EXG2
YPL057C	SUR1	YHR077C	NMD2
YMR080C	NAM7	YBR228W	SLX1
YJL154C	VPS35	YEL043W	YEL043W
YFR010W	UBP6	YGL163C	RAD54
YDR108W	GSG1	YBR131W	CCZ1
YPR046W	MCM16	YMR214W	SCJ1
YFR010W	UBP6	YBR020W	GAL1
YPL112C	PEX25	YAL055W	PEX22
YOR014W	RTS1	YLR182W	SWI6
YKL139W	CTK1	YBR107C	IML3
YOR023C	AHC1	YJR032W	CPR7
YGL133W	ITC1	YKL048C	ELM1
YGL174W	BUD13	YGR270W	YTA7
YNL289W	PCL1	YHR115C	DMA1
YHL027W	RIM101	YPR173C	VPS4
YPL137C	GIP3	YBR019C	GAL10
YPL129W	TAF14	YOR156C	NFI1
YPL024W	RMI1	YER044C	ERG28

Predictions at threshold level 0.75 continued...			
Gene1 of predicted pair		Gene2 of predicted pair	
Systematic Name	Standard Name	Systematic Name	Standard Name
YLR234W	TOP3	YBR114W	RAD16
YNL325C	FIG4	YNL238W	KEX2
YER144C	UBP5	YOL081W	IRA2
YGL033W	HOP2	YJR084W	CSN12
YLR288C	MEC3	YER161C	SPT2
YJL060W	BNA3	YJR060W	CBF1
YGL147C	RPL9A	YLR320W	MMS22
YDR378C	LSM6	YKL110C	KTI12
YGR108W	CLB1	YHL002W	HSE1
YDR318W	MCM21	YNL107W	YAF9
YBR107C	IML3	YHL002W	HSE1
YDR264C	AKR1	YJR086W	STE18
YCL067C	HMLALPHA2	YDL005C	MED2
YNL215W	IES2	YAL002W	VPS8
YGR104C	SRB5	YDR477W	SNF1
YDL020C	RPN4	YGR081C	SLX9
YJL053W	PEP8	YAR014C	BUD14
YEL053C	MAK10	YLR180W	SAM1
YBR194W	SOY1	YMR038C	CCS1
YNL031C	HHT2	YOR064C	YNG1
YGL095C	VPS45	YOR036W	PEP12
YPL240C	HSP82	YJL184W	GON7
YDR254W	CHL4	YLR233C	EST1
YDR108W	GSG1	YDR153C	ENT5
YMR055C	BUB2	YDR200C	VPS64
YOR112W	CEX1	YPL234C	TFP3
YOR043W	WHI2	YNL076W	MKS1
YDL020C	RPN4	YNL307C	MCK1
YOR043W	WHI2	YBR019C	GAL10

Predictions at threshold level 0.75 continued...			
Gene1 of predicted pair		Gene2 of predicted pair	
Systematic Name	Standard Name	Systematic Name	Standard Name
YGR019W	UGA1	YDR293C	SSD1
YDL116W	NUP84	YGL163C	RAD54
YHL027W	RIM101	YJL095W	BCK1
YMR284W	YKU70	YOR080W	DIA2
YML097C	VPS9	YMR089C	YTA12
YPL057C	SUR1	YGL003C	CDH1
YDR213W	UPC2	YMR070W	MOT3
YDR358W	GGA1	YOL081W	IRA2
YML117W	NAB6	YBR082C	UBC4
YJL136C	RPS21B	YKR048C	NAP1
YDR484W	VPS52	YML001W	YPT7
YMR224C	MRE11	YDR260C	SWM1
YNL041C	COG6	YLR398C	SKI2
YDL042C	SIR2	YLR399C	BDF1
YGR270W	YTA7	YLR350W	ORM2
YDR080W	VPS41	YCR066W	RAD18
YGR054W	YGR054W	YGL253W	HXK2
YGL025C	PGD1	YBR171W	SEC66
YKL129C	MYO3	YOR035C	SHE4
YMR026C	PEX12	YBR280C	SAF1
YDL116W	NUP84	YHR111W	UBA4
YPR017C	DSS4	YCL001W	RER1
YPR036W	VMA13	YOL121C	RPS19A
YBR173C	UMP1	YDL223C	HBT1
YBL102W	SFT2	YLR360W	VPS38
YLR386W	VAC14	YFR036W	CDC26
YHR156C	LIN1	YMR214W	SCJ1
YDR080W	VPS41	YPR032W	SRO7
YOR038C	HIR2	YER139C	YER139C

Predictions at threshold level 0.75 continued...			
Gene1 of predicted pair		Gene2 of predicted pair	
Systematic Name	Standard Name	Systematic Name	Standard Name
YLR192C	HCR1	YBR082C	UBC4
YOR290C	SNF2	YKR035W-A	DID2
YNL243W	SLA2	YDL006W	PTC1
YHL031C	GOS1	YEL036C	ANP1
YEL065W	SIT1	YJR084W	CSN12
YLR192C	HCR1	YBR280C	SAF1
YNL307C	MCK1	YLR032W	RAD5
YHR134W	WSS1	YHR167W	THP2
YDR329C	PEX3	YDL065C	PEX19
YER161C	SPT2	YHR039C-A	VMA10
YLR315W	NKP2	YNL107W	YAF9
YNR072W	HXT17	YJL183W	MNN11
YLR309C	IMH1	YNR032W	PPG1
YJR056C	YJR056C	YOR043W	WHI2
YLR095C	IOC2	YER144C	UBP5
YLR337C	VRP1	YLR292C	SEC72
YOR337W	TEA1	YPR030W	CSR2
YGL124C	MON1	YER122C	GLO3
YML038C	YMD8	YHR012W	VPS29
YLR353W	BUD8	YOR301W	RAX1
YLR119W	SRN2	YGL078C	DBP3
YNL087W	TCB2	YOR327C	SNC2
YDR363W-A	SEM1	YHR167W	THP2
YNL079C	TPM1	YPR119W	CLB2
YIL053W	RHR2	YBR173C	UMP1
YJL176C	SWI3	YBR082C	UBC4
YFR036W	CDC26	YNL076W	MKS1
YNL215W	IES2	YDR335W	MSN5
YMR224C	MRE11	YOR213C	SAS5

Predictions at threshold level 0.75 continued...			
Gene1 of predicted pair		Gene2 of predicted pair	
Systematic Name	Standard Name	Systematic Name	Standard Name
YHR073W	OSH3	YPL178W	CBC2
YLR320W	MMS22	YDR261C	EXG2
YDR108W	GSG1	YKL119C	VPH2
YER116C	SLX8	YDR318W	MCM21
YMR152W	YIM1	YNL076W	MKS1
YML038C	YMD8	YJR033C	RAV1
YDR378C	LSM6	YKR026C	GCN3
YJL124C	LSM1	YBR215W	HPC2
YHL020C	OPI1	YFL031W	HAC1
YNL218W	MGS1	YOR076C	SKI7
YER096W	SHC1	YLR425W	TUS1
YKL160W	ELF1	YLR306W	UBC12
YMR038C	CCS1	YGL031C	RPL24A
YJL176C	SWI3	YGR072W	UPF3
YKL025C	PAN3	YJR032W	CPR7
YGL147C	RPL9A	YLR370C	ARC18
YGR108W	CLB1	YLR429W	CRN1
YIL053W	RHR2	YEL060C	PRB1
YBR215W	HPC2	YPR119W	CLB2
YDR443C	SSN2	YOR066W	MSA1
YDL077C	VAM6	YLR309C	IMH1
YPL158C	YPL158C	YJL095W	BCK1
YGL087C	MMS2	YLR032W	RAD5
YBL051C	PIN4	YGR270W	YTA7
YDR071C	PAA1	YNL107W	YAF9
YPR084W	YPR084W	YCR077C	PAT1
YEL053C	MAK10	YEL043W	YEL043W
YDR080W	VPS41	YKR001C	VPS1
YBR200W	BEM1	YPL057C	SUR1

Predictions at threshold level 0.75 continued...			
Gene1 of predicted pair		Gene2 of predicted pair	
Systematic Name	Standard Name	Systematic Name	Standard Name
YDR443C	SSN2	YOL051W	GAL11
YML095C	RAD10	YJL190C	RPS22A
YGL060W	YBP2	YLR381W	CTF3
YLR150W	STM1	YHR200W	RPN10
YOR109W	INP53	YPR032W	SRO7
YJR059W	PTK2	YMR199W	CLN1
YDL101C	DUN1	YCR086W	CSM1
YDR245W	MNN10	YOR275C	RIM20
YLR094C	GIS3	YJR032W	CPR7
YPL195W	APL5	YPL120W	VPS30
YMR095C	SNO1	YHR060W	VMA22
YMR109W	MYO5	YHL027W	RIM101
YOL126C	MDH2	YML057W	CMP2
YNR052C	POP2	YDR378C	LSM6
YKL048C	ELM1	YDR144C	MKC7
YLR094C	GIS3	YOR066W	MSA1
YNL051W	COG5	YAL002W	VPS8
YMR055C	BUB2	YKL101W	HSL1
YIL050W	PCL7	YPL219W	PCL8
YLR381W	CTF3	YKR061W	KTR2
YPL024W	RMI1	YBR194W	SOY1
YBR260C	RGD1	YOR035C	SHE4
YNL031C	HHT2	YEL056W	HAT2
YKL048C	ELM1	YLR410W	VIP1
YFL023W	BUD27	YER044C	ERG28
YPL022W	RAD1	YER116C	SLX8
YMR224C	MRE11	YDL192W	ARF1
YKL139W	CTK1	YNL116W	DMA2
YBR111W-A	SUS1	YOL148C	SPT20

Predictions at threshold level 0.75 continued...			
Gene1 of predicted pair		Gene2 of predicted pair	
Systematic Name	Standard Name	Systematic Name	Standard Name
YGL066W	SGF73	YPL161C	BEM4
YBR107C	IML3	YJL190C	RPS22A
YGR104C	SRB5	YKL139W	CTK1
YNL325C	FIG4	YDR484W	VPS52
YJR032W	CPR7	YDR183W	PLP1
YNL215W	IES2	YBR215W	HPC2
YOR145C	PNO1	YKL143W	LTV1
YJL176C	SWI3	YBR010W	HHT1
YDL116W	NUP84	YOR358W	HAP5
YMR224C	MRE11	YFR036W	CDC26
YPR131C	NAT3	YDR207C	UME6
YBR200W	BEM1	YER162C	RAD4
YML103C	NUP188	YBR017C	KAP104
YKR069W	MET1	YKL129C	MYO3
YNL291C	MID1	YPR051W	MAK3
YOR253W	NAT5	YHR013C	ARD1
YBR057C	MUM2	YGL212W	VAM7
YGL066W	SGF73	YGR252W	GCN5
YBR041W	FAT1	YBL101C	ECM21
YDR146C	SWI5	YFR034C	PHO4
YDR108W	GSG1	YAL002W	VPS8
YLR095C	IOC2	YGL115W	SNF4
YDL116W	NUP84	YNL307C	MCK1
YEL053C	MAK10	YDL006W	PTC1
YEL050C	RML2	YDR378C	LSM6
YIL076W	SEC28	YDR080W	VPS41
YHR156C	LIN1	YOL081W	IRA2
YDR156W	RPA14	YKL048C	ELM1
YDR137W	RGP1	YPL234C	TFP3

Predictions at threshold level 0.75 continued...			
Gene1 of predicted pair		Gene2 of predicted pair	
Systematic Name	Standard Name	Systematic Name	Standard Name
YNL307C	MCK1	YOR312C	RPL20B
YDL122W	UBP1	YJR099W	YUH1
YBL008W	HIR1	YGR108W	CLB1
YDR456W	NHX1	YLR425W	TUS1
YGL147C	RPL9A	YMR230W	RPS10B
YMR116C	ASC1	YBL047C	EDE1
YLR083C	EMP70	YLR309C	IMH1
YDR320C	SWA2	YKR001C	VPS1
YNL288W	CAF40	YDR310C	SUM1
YKL032C	IXR1	YCR077C	PAT1
YGL161C	YIP5	YML097C	VPS9
YOR141C	ARP8	YLR182W	SWI6
YPL256C	CLN2	YJR090C	GRR1
YML095C	RAD10	YKR048C	NAP1
YNL243W	SLA2	YBL061C	SKT5
YKR035W-A	DID2	YJR084W	CSN12
YER179W	DMC1	YNL230C	ELA1
YDR293C	SSD1	YPL037C	EGD1
YML115C	VAN1	YGL200C	EMP24
YLR288C	MEC3	YDR289C	RTT103
YHR052W	CIC1	YGL246C	RAI1
YBL051C	PIN4	YMR055C	BUB2
YDR309C	GIC2	YOL112W	MSB4
YDR484W	VPS52	YHR060W	VMA22
YIL159W	BNR1	YDL184C	RPL41A
YCR065W	HCM1	YPR119W	CLB2
YMR224C	MRE11	YNL248C	RPA49
YGR252W	GCN5	YHR108W	GGA2
YDR137W	RGP1	YPL037C	EGD1

Predictions at threshold level 0.75 continued...			
Gene1 of predicted pair		Gene2 of predicted pair	
Systematic Name	Standard Name	Systematic Name	Standard Name
YOR127W	RGA1	YNL206C	RTT106
YBL058W	SHP1	YBR280C	SAF1
YLR192C	HCR1	YJL183W	MNN11
YIL034C	CAP2	YBR108W	YBR108W
YDR117C	TMA64	YHR203C	RPS4B
YOR083W	WHI5	YMR055C	BUB2
YJL053W	PEP8	YDL161W	ENT1
YER162C	RAD4	YJL095W	BCK1
YJR060W	CBF1	YMR054W	STV1
YLR135W	SLX4	YML028W	TSA1
YLR315W	NKP2	YER139C	YER139C
YNL047C	SLM2	YNL229C	URE2
YHR060W	VMA22	YHR012W	VPS29
YML016C	PPZ1	YJR060W	CBF1
YKL139W	CTK1	YMR214W	SCJ1
YLR090W	XDJ1	YLR398C	SKI2
YDR443C	SSN2	YPL024W	RMI1
YJR056C	YJR056C	YPR046W	MCM16
YDR004W	RAD57	YMR055C	BUB2
YPL140C	MKK2	YJL095W	BCK1
YHR108W	GGA2	YHR158C	KEL1
YLR119W	SRN2	YKL116C	PRR1
YNL206C	RTT106	YGR252W	GCN5
YDR443C	SSN2	YNL307C	MCK1
YPR036W	VMA13	YDR261C	EXG2
YLR148W	PEP3	YAL002W	VPS8
YJL053W	PEP8	YNR032W	PPG1
YDR484W	VPS52	YOR327C	SNC2
YPL248C	GAL4	YKL109W	HAP4

Predictions at threshold level 0.75 continued...			
Gene1 of predicted pair		Gene2 of predicted pair	
Systematic Name	Standard Name	Systematic Name	Standard Name
YDR071C	PAA1	YGR135W	PRE9
YMR127C	SAS2	YGR188C	BUB1
YIL053W	RHR2	YNL087W	TCB2
YDR309C	GIC2	YBL085W	BOI1
YDR176W	NGG1	YNL236W	SIN4
YNL215W	IES2	YNL199C	GCR2
YPL253C	VIK1	YBR107C	IML3
YFR010W	UBP6	YGL141W	HUL5
YFR010W	UBP6	YNL032W	SIW14
YBR108W	YBR108W	YKR035W-A	DID2
YGL163C	RAD54	YJR035W	RAD26
YOR141C	ARP8	YNL107W	YAF9
YNL032W	SIW14	YKL126W	YPK1
YGR105W	VMA21	YJR032W	CPR7
YHR134W	WSS1	YJR135C	MCM22
YDR477W	SNF1	YBR010W	HHT1
YKR020W	VPS51	YCR077C	PAT1
YMR127C	SAS2	YPR051W	MAK3
YCL001W	RER1	YDL077C	VAM6
YNL032W	SIW14	YMR152W	YIM1
YDR293C	SSD1	YPL018W	CTF19
YGL054C	ERV14	YNL107W	YAF9
YDR392W	SPT3	YGL178W	MPT5
YKR072C	SIS2	YEL056W	HAT2
YOR127W	RGA1	YGL054C	ERV14
YOR083W	WHI5	YDR171W	HSP42
YMR095C	SNO1	YGL212W	VAM7
YNL206C	RTT106	YKL074C	MUD2
YDR137W	RGP1	YDR440W	DOT1

Predictions at threshold level 0.75 continued...			
Gene1 of predicted pair		Gene2 of predicted pair	
Systematic Name	Standard Name	Systematic Name	Standard Name
YDR379W	RGA2	YKR048C	NAP1
YGL124C	MON1	YMR311C	GLC8
YBR217W	ATG12	YLL042C	ATG10
YGR252W	GCN5	YKL160W	ELF1
YOR197W	MCA1	YNL094W	APP1
YDR293C	SSD1	YJL183W	MNN11
YDR202C	RAV2	YPL105C	YPL105C
YOR069W	VPS5	YNL127W	FAR11
YHR060W	VMA22	YEL051W	VMA8
YPL037C	EGD1	YEL043W	YEL043W
YNL307C	MCK1	YNL082W	PMS1
YDR335W	MSN5	YDR183W	PLP1
YMR224C	MRE11	YGL115W	SNF4
YDR320C	SWA2	YGL223C	COG1
YGL033W	HOP2	YLR371W	ROM2
YDL116W	NUP84	YNL199C	GCR2
YGL175C	SAE2	YGL163C	RAD54
YIL159W	BNR1	YGL003C	CDH1
YHR111W	UBA4	YDR310C	SUM1
YDR383C	NKP1	YDR183W	PLP1
YMR142C	RPL13B	YBR200W	BEM1
YGL200C	EMP24	YBL047C	EDE1
YDR477W	SNF1	YGL212W	VAM7
YGR204W	ADE3	YEL046C	GLY1
YKL161C	YKL161C	YHR158C	KEL1
YBR020W	GAL1	YNL004W	HRB1
YNL116W	DMA2	YOL081W	IRA2
YGL060W	YBP2	YOR080W	DIA2
YMR224C	MRE11	YGL033W	HOP2

Predictions at threshold level 0.75 continued...			
Gene1 of predicted pair		Gene2 of predicted pair	
Systematic Name	Standard Name	Systematic Name	Standard Name
YFL033C	RIM15	YOL018C	TLG2
YKL116C	PRR1	YER122C	GLO3
YJL154C	VPS35	YBL102W	SFT2
YGR019W	UGA1	YEL053C	MAK10
YHR060W	VMA22	YNL127W	FAR11
YDR144C	MKC7	YNL107W	YAF9
YGL147C	RPL9A	YDL192W	ARF1
YML103C	NUP188	YKR095W	MLP1
YDR363W-A	SEM1	YGL115W	SNF4
YJL095W	BCK1	YCR053W	THR4
YKL126W	YPK1	YJR032W	CPR7
YOR140W	SFL1	YLR425W	TUS1
YOR109W	INP53	YPL161C	BEM4
YBL103C	RTG3	YPL161C	BEM4
YGL153W	PEX14	YJL006C	CTK2
YJR032W	CPR7	YBR036C	CSG2
YBR108W	YBR108W	YJR056C	YJR056C
YDR477W	SNF1	YFL023W	BUD27
YHR167W	THP2	YHR077C	NMD2
YBL008W	HIR1	YOL091W	SPO21
YAL024C	LTE1	YHR158C	KEL1
YPL096C-A	ERI1	YDL223C	HBT1
YLR216C	CPR6	YNL307C	MCK1
YEL043W	YEL043W	YPL120W	VPS30
YCR084C	TUP1	YNL236W	SIN4
YBR173C	UMP1	YKL048C	ELM1
YDR443C	SSN2	YML001W	YPT7
YDR383C	NKP1	YNL116W	DMA2
YLR362W	STE11	YDR309C	GIC2

Predictions at threshold level 0.75 continued...			
Gene1 of predicted pair		Gene2 of predicted pair	
Systematic Name	Standard Name	Systematic Name	Standard Name
YAL024C	LTE1	YGL163C	RAD54
YHR079C	IRE1	YOR023C	AHC1
YGL223C	COG1	YHR060W	VMA22
YOR337W	TEA1	YBR200W	BEM1
YMR095C	SNO1	YDR477W	SNF1
YPL057C	SUR1	YKR035W-A	DID2
YER116C	SLX8	YKL101W	HSL1
YDL002C	NHP10	YAL002W	VPS8
YLR371W	ROM2	YER162C	RAD4
YKL204W	EAP1	YDR310C	SUM1
YPR120C	CLB5	YGL003C	CDH1
YER116C	SLX8	YJR090C	GRR1
YLR381W	CTF3	YNL307C	MCK1
YKR072C	SIS2	YNR052C	POP2
YLR119W	SRN2	YOR327C	SNC2
YBR194W	SOY1	YLR381W	CTF3
YPL024W	RMI1	YER040W	GLN3
YDR080W	VPS41	YDL006W	PTC1
YDR245W	MNN10	YGL212W	VAM7
YIL076W	SEC28	YEL051W	VMA8
YNL288W	CAF40	YBR107C	IML3
YJL154C	VPS35	YPL161C	BEM4
YOR001W	RRP6	YDR335W	MSN5
YLR381W	CTF3	YGL252C	RTG2
YDL020C	RPN4	YBR215W	HPC2
YML016C	PPZ1	YJR135C	MCM22
YDL116W	NUP84	YMR164C	MSS11
YBR108W	YBR108W	YNL166C	BNI5
YAL024C	LTE1	YJL183W	MNN11

Predictions at threshold level 0.75 continued...			
Gene1 of predicted pair		Gene2 of predicted pair	
Systematic Name	Standard Name	Systematic Name	Standard Name
YJR084W	CSN12	YLR032W	RAD5
YIL034C	CAP2	YAR014C	BUD14
YDR289C	RTT103	YOL081W	IRA2
YGR241C	YAP1802	YNR006W	VPS27
YDR254W	CHL4	YLR182W	SWI6
YGL174W	BUD13	YBR215W	HPC2
YLR192C	HCR1	YKR020W	VPS51
YNL032W	SIW14	YOL121C	RPS19A
YLR386W	VAC14	YDL184C	RPL41A
YGL054C	ERV14	YPL234C	TFP3
YBL102W	SFT2	YKR020W	VPS51
YDR484W	VPS52	YPL195W	APL5
YEL053C	MAK10	YGL253W	HXK2
YGL066W	SGF73	YNL307C	MCK1
YDR378C	LSM6	YDL226C	GCS1
YNL297C	MON2	YJR040W	GEF1
YJL110C	GZF3	YPR119W	CLB2
YBR050C	REG2	YER027C	GAL83
YGL133W	ITC1	YBL087C	RPL23A
YHL031C	GOS1	YPL050C	MNN9
YOR290C	SNF2	YKL126W	YPK1
YGL124C	MON1	YBR036C	CSG2
YOR290C	SNF2	YPL161C	BEM4
YIL034C	CAP2	YPL253C	VIK1
YOR270C	VPH1	YPL137C	GIP3
YAL024C	LTE1	YBR036C	CSG2
YMR224C	MRE11	YOR043W	WHI2
YBR171W	SEC66	YFR036W	CDC26
YPL178W	CBC2	YHR079C-A	SAE3

Predictions at threshold level 0.75 continued...			
Gene1 of predicted pair		Gene2 of predicted pair	
Systematic Name	Standard Name	Systematic Name	Standard Name
YBR290W	BSD2	YLR337C	VRP1
YHR200W	RPN10	YCR053W	THR4
YNL199C	GCR2	YMR125W	STO1
YGL115W	SNF4	YDL083C	RPS16B
YBR217W	ATG12	YPL161C	BEM4
YGL212W	VAM7	YPR046W	MCM16
YLR337C	VRP1	YGR135W	PRE9
YIL034C	CAP2	YPL018W	CTF19
YPL253C	VIK1	YER049W	TPA1
YFR031C-A	RPL2A	YOR043W	WHI2
YFR031C-A	RPL2A	YJL154C	VPS35
YOR337W	TEA1	YNL076W	MKS1
YGR270W	YTA7	YDR155C	CPR1
YDR439W	LRS4	YHR013C	ARD1
YHL031C	GOS1	YMR038C	CCS1
YMR273C	ZDS1	YFL047W	RGD2
YJL053W	PEP8	YKR030W	GMH1
YNL238W	KEX2	YJL140W	RPB4
YDR448W	ADA2	YDR469W	SDC1
YML071C	COG8	YMR214W	SCJ1
YOR132W	VPS17	YGL115W	SNF4
YLR064W	YLR064W	YPL246C	RBD2
YDR363W-A	SEM1	YER139C	YER139C
YDR108W	GSG1	YER120W	SCS2
YOL067C	RTG1	YBR114W	RAD16
YDR293C	SSD1	YKR020W	VPS51
YDR335W	MSN5	YPR046W	MCM16
YLR386W	VAC14	YOR066W	MSA1
YGR108W	CLB1	YDL047W	SIT4

Predictions at threshold level 0.75 continued...			
Gene1 of predicted pair		Gene2 of predicted pair	
Systematic Name	Standard Name	Systematic Name	Standard Name
YPL057C	SUR1	YDR289C	RTT103
YDL080C	THI3	YOR213C	SAS5
YKR048C	NAP1	YLR182W	SWI6
YOR033C	EXO1	YMR199W	CLN1
YFR010W	UBP6	YKL126W	YPK1
YGR270W	YTA7	YAL002W	VPS8
YOL126C	MDH2	YBL079W	NUP170
YLL007C	YLL007C	YML095C	RAD10
YBR108W	YBR108W	YDR484W	VPS52
YLR018C	POM34	YBL079W	NUP170
YFR031C-A	RPL2A	YDL192W	ARF1
YHR144C	DCD1	YMR201C	RAD14
YDR363W-A	SEM1	YBL087C	RPL23A
YJR063W	RPA12	YDR071C	PAA1
YLR396C	VPS33	YPL045W	VPS16
YJR090C	GRR1	YGL252C	RTG2
YLR093C	NYV1	YBR036C	CSG2
YGL095C	VPS45	YDR323C	PEP7
YPL256C	CLN2	YGL031C	RPL24A
YGR020C	VMA7	YDR153C	ENT5
YBR042C	CST26	YGR105W	VMA21
YDR363W-A	SEM1	YDR260C	SWM1
YOR076C	SKI7	YOR145C	PNO1
YBR194W	SOY1	YDL226C	GCS1
YHR013C	ARD1	YNL238W	KEX2
YML071C	COG8	YCR053W	THR4
YOR023C	AHC1	YPL161C	BEM4
YHR134W	WSS1	YGL151W	NUT1
YDL184C	RPL41A	YPR030W	CSR2

Predictions at threshold level 0.75 continued...			
Gene1 of predicted pair		Gene2 of predicted pair	
Systematic Name	Standard Name	Systematic Name	Standard Name
YJL036W	SNX4	YJR060W	CBF1
YHR156C	LIN1	YHR134W	WSS1
YNL107W	YAF9	YOL081W	IRA2
YBR215W	HPC2	YDR335W	MSN5
YEL060C	PRB1	YMR230W	RPS10B
YBR131W	CCZ1	YGR105W	VMA21
YLR079W	SIC1	YML057W	CMP2
YNL032W	SIW14	YDL192W	ARF1
YGL178W	MPT5	YDL056W	MBP1
YPL253C	VIK1	YAR014C	BUD14
YLR234W	TOP3	YML103C	NUP188
YMR127C	SAS2	YFR036W	CDC26
YBR278W	DPB3	YBR036C	CSG2
YML103C	NUP188	YDR254W	CHL4
YHR205W	SCH9	YPL161C	BEM4
YOR035C	SHE4	YJR084W	CSN12
YLR371W	ROM2	YEL036C	ANP1
YBR107C	IML3	YFR030W	MET10
YDL101C	DUN1	YCR066W	RAD18
YPR018W	RLF2	YPR131C	NAT3
YOR127W	RGA1	YOR112W	CEX1
YJL136C	RPS21B	YML095C	RAD10
YOR035C	SHE4	YBR181C	RPS6B
YOR337W	TEA1	YER116C	SLX8
YMR216C	SKY1	YDL077C	VAM6
YDL184C	RPL41A	YOL111C	MDY2
YPL037C	EGD1	YPL105C	YPL105C
YER001W	MNN1	YMR089C	YTA12
YDR379W	RGA2	YGL038C	OCH1

Predictions at threshold level 0.75 continued...			
Gene1 of predicted pair		Gene2 of predicted pair	
Systematic Name	Standard Name	Systematic Name	Standard Name
YKL006W	RPL14A	YJL095W	BCK1
YJR032W	CPR7	YJL190C	RPS22A
YNR064C	YNR064C	YOR344C	TYE7
YBR111W-A	SUS1	YMR273C	ZDS1
YHR156C	LIN1	YMR230W	RPS10B
YKL135C	APL2	YJR005W	APL1
YKR048C	NAP1	YPL256C	CLN2
YMR142C	RPL13B	YJL172W	CPS1
YDR174W	HMO1	YCR086W	CSM1
YLR435W	TSR2	YKL101W	HSL1
YPL137C	GIP3	YMR214W	SCJ1
YMR026C	PEX12	YNL032W	SIW14
YMR216C	SKY1	YOL108C	INO4
YGL078C	DBP3	YPL037C	EGD1
YOR069W	VPS5	YKR030W	GMH1
YOR112W	CEX1	YDR153C	ENT5
YLR234W	TOP3	YBR194W	SOY1
YJL124C	LSM1	YFR030W	MET10
YLR079W	SIC1	YPL152W	RRD2
YFR010W	UBP6	YLR386W	VAC14
YCR065W	HCM1	YNL236W	SIN4
YNL116W	DMA2	YLR182W	SWI6
YKL126W	YPK1	YHR200W	RPN10
YKL139W	CTK1	YDR261C	EXG2
YLR381W	CTF3	YJR135C	MCM22
YGR241C	YAP1802	YLR206W	ENT2
YBR274W	CHK1	YCR086W	CSM1
YNL291C	MID1	YGL163C	RAD54
YGL124C	MON1	YNL076W	MKS1

Predictions at threshold level 0.75 continued...			
Gene1 of predicted pair		Gene2 of predicted pair	
Systematic Name	Standard Name	Systematic Name	Standard Name
YMR133W	REC114	YNL238W	KEX2
YNL215W	IES2	YPL152W	RRD2
YNL325C	FIG4	YPR036W	VMA13
YML103C	NUP188	YOR358W	HAP5
YDR260C	SWM1	YLR032W	RAD5
YGL212W	VAM7	YOL108C	INO4
YLR441C	RPS1A	YNL076W	MKS1
YML115C	VAN1	YGL025C	PGD1
YGL178W	MPT5	YBR231C	SWC5
YDR477W	SNF1	YDL184C	RPL41A
YJR056C	YJR056C	YBR019C	GAL10
YPL057C	SUR1	YDR207C	UME6
YGL060W	YBP2	YJR035W	RAD26
YMR198W	CIK1	YML095C	RAD10
YJL172W	CPS1	YOR066W	MSA1
YPR017C	DSS4	YPR051W	MAK3
YPL018W	CTF19	YHR114W	BZZ1
YLR386W	VAC14	YFL023W	BUD27
YNL069C	RPL16B	YOL108C	INO4
YBR057C	MUM2	YGL200C	EMP24
YDR137W	RGP1	YCL037C	SRO9
YNL032W	SIW14	YDL083C	RPS16B
YGR019W	UGA1	YDR310C	SUM1
YKR035W-A	DID2	YDL077C	VAM6
YOR001W	RRP6	YER063W	THO1
YGL133W	ITC1	YHR200W	RPN10
YFL013C	IES1	YOR001W	RRP6
YNL106C	INP52	YBR228W	SLX1
YEL050C	RML2	YDR254W	CHL4

Predictions at threshold level 0.75 continued...			
Gene1 of predicted pair		Gene2 of predicted pair	
Systematic Name	Standard Name	Systematic Name	Standard Name
YBR057C	MUM2	YER001W	MNN1
YBL058W	SHP1	YPR046W	MCM16
YOR337W	TEA1	YFR010W	UBP6
YHR013C	ARD1	YLR410W	VIP1
YJR140C	HIR3	YCR031C	RPS14A
YIL159W	BNR1	YLR371W	ROM2
YPL022W	RAD1	YOL090W	MSH2
YMR216C	SKY1	YHL020C	OPI1
YHR039C-A	VMA10	YBR036C	CSG2
YLR090W	XDJ1	YNL076W	MKS1
YLR292C	SEC72	YPL253C	VIK1
YJR063W	RPA12	YLR357W	RSC2
YER144C	UBP5	YER139C	YER139C
YPL152W	RRD2	YJL006C	CTK2
YDL020C	RPN4	YGR135W	PRE9
YMR004W	MVP1	YJR033C	RAV1
YBL058W	SHP1	YEL060C	PRB1
YCR086W	CSM1	YMR038C	CCS1
YMR026C	PEX12	YAL055W	PEX22
YLR192C	HCR1	YLR180W	SAM1
YML071C	COG8	YLR180W	SAM1
YJL154C	VPS35	YLR371W	ROM2
YLR090W	XDJ1	YOR043W	WHI2
YHR119W	SET1	YBR279W	PAF1
YDL083C	RPS16B	YOR066W	MSA1
YBR131W	CCZ1	YMR054W	STV1
YLR337C	VRP1	YGR019W	UGA1
YER110C	KAP123	YPL018W	CTF19
YJR135C	MCM22	YJL183W	MNN11

Predictions at threshold level 0.75 continued...			
Gene1 of predicted pair		Gene2 of predicted pair	
Systematic Name	Standard Name	Systematic Name	Standard Name
YML115C	VAN1	YPR119W	CLB2
YGR054W	YGR054W	YJL095W	BCK1
YMR198W	CIK1	YOR080W	DIA2
YML109W	ZDS2	YHR061C	GIC1
YBR030W	YBR030W	YDR261C	EXG2
YDR004W	RAD57	YFL003C	MSH4
YOR069W	VPS5	YER120W	SCS2
YMR004W	MVP1	YGL054C	ERV14
YPR084W	YPR084W	YPL234C	TFP3
YJR135C	MCM22	YIL103W	DPH1
YPR141C	KAR3	YHR056C	RSC30
YOR005C	DNL4	YGL090W	LIF1
YDR146C	SWI5	YER096W	SHC1
YLR102C	APC9	YGL163C	RAD54
YOR127W	RGA1	YMR199W	CLN1
YPL057C	SUR1	YML071C	COG8
YJL154C	VPS35	YPR032W	SRO7
YDR507C	GIN4	YDR264C	AKR1
YOL090W	MSH2	YML028W	TSA1
YBR108W	YBR108W	YJL095W	BCK1
YEL053C	MAK10	YCR086W	CSM1
YGL212W	VAM7	YMR152W	YIM1
YJL172W	CPS1	YGL003C	CDH1
YOR069W	VPS5	YGR105W	VMA21
YAL021C	CCR4	YHR056C	RSC30
YLR386W	VAC14	YDR137W	RGP1
YDR335W	MSN5	YKL160W	ELF1
YJL030W	MAD2	YGL190C	CDC55
YBL103C	RTG3	YDL223C	HBT1

Predictions at threshold level 0.75 continued...			
Gene1 of predicted pair		Gene2 of predicted pair	
Systematic Name	Standard Name	Systematic Name	Standard Name
YOL121C	RPS19A	YGL124C	MON1
YJL124C	LSM1	YLR362W	STE11
YML103C	NUP188	YGR188C	BUB1
YNL248C	RPA49	YFR036W	CDC26
YDR378C	LSM6	YGL115W	SNF4
YCL063W	VAC17	YEL013W	VAC8
YKR009C	FOX2	YOL147C	PEX11
YDR293C	SSD1	YIL076W	SEC28
YLR292C	SEC72	YDL155W	CLB3
YNL307C	MCK1	YMR230W	RPS10B
YKR020W	VPS51	YGL115W	SNF4
YAL024C	LTE1	YDR318W	MCM21
YMR004W	MVP1	YHR012W	VPS29
YJR056C	YJR056C	YDR207C	UME6
YLR018C	POM34	YDR358W	GGA1
YLR292C	SEC72	YPR051W	MAK3
YER116C	SLX8	YOL030W	GAS5
YHR171W	ATG7	YLL042C	ATG10
YPL195W	APL5	YOL108C	INO4
YMR153W	NUP53	YBL079W	NUP170
YOR127W	RGA1	YBR171W	SEC66
YLR399C	BDF1	YFR036W	CDC26
YIL053W	RHR2	YLR371W	ROM2
YPL057C	SUR1	YDR076W	RAD55
YKL117W	SBA1	YNL199C	GCR2
YJL029C	VPS53	YAL030W	SNC1
YGL087C	MMS2	YKR001C	VPS1
YGR135W	PRE9	YOL054W	PSH1
YDR496C	PUF6	YFR001W	LOC1

Predictions at threshold level 0.75 continued...			
Gene1 of predicted pair		Gene2 of predicted pair	
Systematic Name	Standard Name	Systematic Name	Standard Name
YMR313C	TGL3	YKL140W	TGL1
YDL020C	RPN4	YDR225W	HTA1
YGR020C	VMA7	YJR040W	GEF1
YNR072W	HXT17	YCR053W	THR4
YNL307C	MCK1	YPL037C	EGD1
YJL172W	CPS1	YLR094C	GIS3
YJL154C	VPS35	YDR183W	PLP1
YHL031C	GOS1	YPL120W	VPS30
YKR061W	KTR2	YFR036W	CDC26
YIR009W	MSL1	YPR119W	CLB2
YHR012W	VPS29	YLR309C	IMH1
YLR119W	SRN2	YHR060W	VMA22
YLR452C	SST2	YDR207C	UME6
YHR134W	WSS1	YDL155W	CLB3
YGL226C-A	OST5	YML019W	OST6
YOR023C	AHC1	YGL237C	HAP2
YPL246C	RBD2	YJL004C	SYS1
YBR131W	CCZ1	YLR320W	MMS22
YDR443C	SSN2	YDR146C	SWI5
YGL147C	RPL9A	YPR046W	MCM16
YBR200W	BEM1	YGL003C	CDH1
YGR072W	UPF3	YEL043W	YEL043W
YOR069W	VPS5	YHR012W	VPS29
YDL116W	NUP84	YGL100W	SEH1
YEL065W	SIT1	YHR039C-A	VMA10
YNL288W	CAF40	YBL021C	HAP3
YFL023W	BUD27	YJR056C	YJR056C
YKR020W	VPS51	YMR231W	PEP5
YGR108W	CLB1	YNL166C	BNI5

Predictions at threshold level 0.75 continued...			
Gene1 of predicted pair		Gene2 of predicted pair	
Systematic Name	Standard Name	Systematic Name	Standard Name
YLR006C	SSK1	YOR127W	RGA1
YGR249W	MGA1	YER144C	UBP5
YBR200W	BEM1	YDR245W	MNN10
YMR016C	SOK2	YLR371W	ROM2
YLR182W	SWI6	YLR350W	ORM2
YLR192C	HCR1	YGL003C	CDH1
YKR099W	BAS1	YJR032W	CPR7
YLR386W	VAC14	YGL115W	SNF4
YNL325C	FIG4	YDL223C	HBT1
YLR234W	TOP3	YGR270W	YTA7
YPL129W	TAF14	YGR270W	YTA7
YNL106C	INP52	YER096W	SHC1
YLR234W	TOP3	YOR132W	VPS17
YML103C	NUP188	YMR153W	NUP53
YHR200W	RPN10	YDL192W	ARF1
YGL174W	BUD13	YKL160W	ELF1
YBR057C	MUM2	YGR188C	BUB1
YDL059C	RAD59	YBR215W	HPC2
YOR078W	BUD21	YBL072C	RPS8A
YGL060W	YBP2	YLR079W	SIC1
YBL103C	RTG3	YDR358W	GGA1
YGL134W	PCL10	YPL219W	PCL8
YGL124C	MON1	YGL253W	HXK2
YJL149W	YJL149W	YDR139C	RUB1
YLR083C	EMP70	YNL127W	FAR11
YHR073W	OSH3	YMR125W	STO1
YMR022W	UBC7	YPR119W	CLB2
YDR383C	NKP1	YDR439W	LRS4
YML103C	NUP188	YPL178W	CBC2

Predictions at threshold level 0.75 continued...			
Gene1 of predicted pair		Gene2 of predicted pair	
Systematic Name	Standard Name	Systematic Name	Standard Name
YML071C	COG8	YDR153C	ENT5
YGR249W	MGA1	YBR171W	SEC66
YPL042C	SSN3	YDL226C	GCS1
YCR081W	SRB8	YJL154C	VPS35
YIL148W	RPL40A	YCR024C-A	PMP1
YHL031C	GOS1	YEL042W	GDA1
YHR052W	CIC1	YKL009W	MRT4
YNL041C	COG6	YOR308C	SNU66
YNL044W	YIP3	YNL041C	COG6
YMR224C	MRE11	YBR280C	SAF1
YDR004W	RAD57	YPL022W	RAD1
YBR108W	YBR108W	YHR111W	UBA4
YOR132W	VPS17	YJR040W	GEF1
YGL115W	SNF4	YEL060C	PRB1
YJL172W	CPS1	YMR152W	YIM1
YMR284W	YKU70	YNL016W	PUB1
YDR135C	YCF1	YGL181W	GTS1
YDR443C	SSN2	YHR039C-A	VMA10
YCR088W	ABP1	YJL187C	SWE1
YNL325C	FIG4	YMR016C	SOK2
YOR156C	NFI1	YNL078W	NIS1
YER115C	SPR6	YMR125W	STO1
YPL024W	RMI1	YOR368W	RAD17
YGR104C	SRB5	YDR289C	RTT103
YJR056C	YJR056C	YPR036W	VMA13
YIL034C	CAP2	YLR234W	TOP3
YDR156W	RPA14	YMR205C	PFK2
YLR090W	XDJ1	YKR099W	BAS1
YMR179W	SPT21	YJR060W	CBF1

Predictions at threshold level 0.75 continued...			
Gene1 of predicted pair		Gene2 of predicted pair	
Systematic Name	Standard Name	Systematic Name	Standard Name
YBR200W	BEM1	YHR081W	LRP1
YDR378C	LSM6	YCR077C	PAT1
YOR270C	VPH1	YLR094C	GIS3
YMR224C	MRE11	YNL106C	INP52
YPR120C	CLB5	YNL107W	YAF9
YPR030W	CSR2	YER162C	RAD4
YOR043W	WHI2	YBR195C	MSI1
YML115C	VAN1	YML028W	TSA1
YER116C	SLX8	YMR311C	GLC8
YDR293C	SSD1	YBL103C	RTG3
YMR199W	CLN1	YHL011C	PRS3
YLR386W	VAC14	YDL192W	ARF1
YOR069W	VPS5	YJR033C	RAV1
YDR156W	RPA14	YGR135W	PRE9
YPL115C	BEM3	YPR051W	MAK3
YDR440W	DOT1	YHR115C	DMA1
YNL199C	GCR2	YER122C	GLO3
YDR146C	SWI5	YOR290C	SNF2
YKR020W	VPS51	YML001W	YPT7
YHR060W	VMA22	YOL111C	MDY2
YDR137W	RGP1	YEL027W	CUP5
YKL007W	CAP1	YKL213C	DOA1
YNL051W	COG5	YKL119C	VPH2
YNR052C	POP2	YNL107W	YAF9
YGL078C	DBP3	YBR200W	BEM1
YFR024C-A	LSB3	YBR108W	YBR108W
YDL020C	RPN4	YKL139W	CTK1
YIR025W	MND2	YGL003C	CDH1
YJL176C	SWI3	YDR378C	LSM6

Predictions at threshold level 0.75 continued...			
Gene1 of predicted pair		Gene2 of predicted pair	
Systematic Name	Standard Name	Systematic Name	Standard Name
YCL001W	RER1	YNL107W	YAF9
YDL116W	NUP84	YKL048C	ELM1
YBR290W	BSD2	YHR114W	BZZ1
YGR135W	PRE9	YBL101C	ECM21
YCR031C	RPS14A	YGL019W	CKB1
YLR360W	VPS38	YDR318W	MCM21
YPL042C	SSN3	YNL297C	MON2
YER068W	MOT2	YLR357W	RSC2
YLR192C	HCR1	YLR371W	ROM2
YMR004W	MVP1	YKR030W	GMH1
YFR036W	CDC26	YKL101W	HSL1
YDL184C	RPL41A	YJR032W	CPR7
YGR003W	CUL3	YLR306W	UBC12
YGR135W	PRE9	YKL101W	HSL1
YJL053W	PEP8	YJR060W	CBF1
YLR360W	VPS38	YMR311C	GLC8
YBR126C	TPS1	YKL139W	CTK1
YBR200W	BEM1	YLR320W	MMS22
YDL116W	NUP84	YBR200W	BEM1
YPL137C	GIP3	YHR081W	LRP1
YAL024C	LTE1	YOR035C	SHE4
YNL087W	TCB2	YHR060W	VMA22
YNL166C	BNI5	YPR046W	MCM16
YGL003C	CDH1	YMR311C	GLC8
YPR154W	PIN3	YHR016C	YSC84
YHR129C	ARP1	YHR098C	SFB3
YLR119W	SRN2	YMR026C	PEX12
YLL007C	YLL007C	YOR023C	AHC1
YKL139W	CTK1	YOL051W	GAL11

Predictions at threshold level 0.75 continued...			
**Gene1 of predicted pair**		**Gene2 of predicted pair**	
**Systematic Name**	**Standard Name**	**Systematic Name**	**Standard Name**
YBR009C	HHF1	YER068W	MOT2
YOR112W	CEX1	YDR310C	SUM1
YPL125W	KAP120	YBL079W	NUP170
YCR020C-A	MAK31	YJL154C	VPS35
YDR318W	MCM21	YPR119W	CLB2
YDR130C	FIN1	YCR086W	CSM1
YLR337C	VRP1	YOR213C	SAS5
YJL154C	VPS35	YMR004W	MVP1
YDR183W	PLP1	YNL225C	CNM67
YMR142C	RPL13B	YOR035C	SHE4
YML038C	YMD8	YPL120W	VPS30
YNL264C	PDR17	YPL180W	TCO89
YLR362W	STE11	YJL128C	PBS2
YOR035C	SHE4	YAL002W	VPS8
YLR386W	VAC14	YIR005W	IST3
YPL161C	BEM4	YMR311C	GLC8
YER016W	BIM1	YHR056C	RSC30
YDR244W	PEX5	YAR042W	SWH1
YDR477W	SNF1	YLR180W	SAM1
YDR129C	SAC6	YOR312C	RPL20B
YBR131W	CCZ1	YNL044W	YIP3
YMR224C	MRE11	YDR289C	RTT103
YDR484W	VPS52	YPL037C	EGD1
YOR069W	VPS5	YPL234C	TFP3
YGL115W	SNF4	YDL155W	CLB3
YNL087W	TCB2	YEL053C	MAK10
YLR370C	ARC18	YBR036C	CSG2
YDL116W	NUP84	YCR086W	CSM1
YLR386W	VAC14	YPL137C	GIP3

Predictions at threshold level 0.75 continued...			
Gene1 of predicted pair		Gene2 of predicted pair	
Systematic Name	Standard Name	Systematic Name	Standard Name
YER110C	KAP123	YGR270W	YTA7
YGL066W	SGF73	YKL101W	HSL1
YBR200W	BEM1	YGR270W	YTA7
YBR200W	BEM1	YEL056W	HAT2
YGR249W	MGA1	YDR207C	UME6
YHR060W	VMA22	YJL004C	SYS1
YNL094W	APP1	YKL129C	MYO3
YOR001W	RRP6	YKL160W	ELF1
YGR217W	CCH1	YBR082C	UBC4
YOR290C	SNF2	YJL140W	RPB4
YEL065W	SIT1	YDR443C	SSN2
YEL051W	VMA8	YDL077C	VAM6
YBR200W	BEM1	YDL006W	PTC1
YBR200W	BEM1	YHR013C	ARD1
YLR234W	TOP3	YCR077C	PAT1
YJR060W	CBF1	YGR105W	VMA21
YDL020C	RPN4	YBR107C	IML3
YDR137W	RGP1	YBL025W	RRN10
YER116C	SLX8	YDL184C	RPL41A
YOR197W	MCA1	YHR161C	YAP1801
YDL020C	RPN4	YMR127C	SAS2
YLR083C	EMP70	YOR109W	INP53
YPL161C	BEM4	YOR066W	MSA1
YDL013W	SLX5	YDR076W	RAD55
YNL288W	CAF40	YLR447C	VMA6
YKL139W	CTK1	YGL115W	SNF4
YOL051W	GAL11	YHR200W	RPN10
YMR026C	PEX12	YNL325C	FIG4
YBL102W	SFT2	YPL105C	YPL105C

Predictions at threshold level 0.75 continued...			
Gene1 of predicted pair		Gene2 of predicted pair	
Systematic Name	Standard Name	Systematic Name	Standard Name
YNL032W	SIW14	YER122C	GLO3
YGL178W	MPT5	YGR229C	SMI1
YDL083C	RPS16B	YMR152W	YIM1
YBR108W	YBR108W	YPL253C	VIK1
YPL240C	HSP82	YDR545W	YRF1-1
YDL116W	NUP84	YNL248C	RPA49
YLR337C	VRP1	YHR134W	WSS1
YDL116W	NUP84	YPL253C	VIK1
YLR216C	CPR6	YOR195W	SLK19
YPR018W	RLF2	YLR150W	STM1
YAL020C	ATS1	YKR020W	VPS51
YLR102C	APC9	YBL047C	EDE1
YDR202C	RAV2	YGL115W	SNF4
YPR036W	VMA13	YGR105W	VMA21
YPL022W	RAD1	YLR320W	MMS22
YNL291C	MID1	YOR127W	RGA1
YFR030W	MET10	YLR182W	SWI6
YIL159W	BNR1	YBR200W	BEM1
YDR320C	SWA2	YKR020W	VPS51
YGR249W	MGA1	YBR280C	SAF1
YMR127C	SAS2	YAL002W	VPS8
YMR224C	MRE11	YMR152W	YIM1
YBR030W	YBR030W	YFL023W	BUD27
YPL253C	VIK1	YOL054W	PSH1
YDR253C	MET32	YPR120C	CLB5
YBR019C	GAL10	YBR036C	CSG2
YCR097W	HMRA1	YPL240C	HSP82
YLR452C	SST2	YCR053W	THR4
YHL025W	SNF6	YOR038C	HIR2

Predictions at threshold level 0.75 continued...			
Gene1 of predicted pair		Gene2 of predicted pair	
Systematic Name	Standard Name	Systematic Name	Standard Name
YDR383C	NKP1	YPL152W	RRD2
YGL212W	VAM7	YKL041W	VPS24
YPR036W	VMA13	YLR452C	SST2
YDR507C	GIN4	YBL047C	EDE1
YDR260C	SWM1	YDR318W	MCM21
YLR410W	VIP1	YDR200C	VPS64
YBL058W	SHP1	YER144C	UBP5
YIL036W	CST6	YOL054W	PSH1
YML062C	MFT1	YCL011C	GBP2
YCL010C	SGF29	YPL248C	GAL4
YDR137W	RGP1	YML071C	COG8
YPR089W	YPR089W	YKR001C	VPS1
YOR156C	NFI1	YOL145C	CTR9
YGR105W	VMA21	YDR310C	SUM1
YBR019C	GAL10	YCR053W	THR4
YMR039C	SUB1	YJR060W	CBF1
YLR441C	RPS1A	YGL025C	PGD1
YBR173C	UMP1	YJR084W	CSN12
YHR178W	STB5	YPL001W	HAT1
YDR225W	HTA1	YOR023C	AHC1
YDR103W	STE5	YJL157C	FAR1
YLR386W	VAC14	YOR156C	NFI1
YKL048C	ELM1	YOL054W	PSH1
YBR171W	SEC66	YDR310C	SUM1
YLR447C	VMA6	YGR270W	YTA7
YOL136C	PFK27	YGR209C	TRX2
YPL234C	TFP3	YJL095W	BCK1
YPR052C	NHP6A	YBR240C	THI2
YGL054C	ERV14	YER122C	GLO3

Predictions at threshold level 0.75 continued...			
Gene1 of predicted pair		Gene2 of predicted pair	
Systematic Name	Standard Name	Systematic Name	Standard Name
YMR153W	NUP53	YGL016W	KAP122
YGL175C	SAE2	YOR368W	RAD17
YLR090W	XDJ1	YOR290C	SNF2
YDR260C	SWM1	YML028W	TSA1
YCR086W	CSM1	YLR254C	NDL1
YIL159W	BNR1	YDR309C	GIC2
YPR120C	CLB5	YHR158C	KEL1
YOR290C	SNF2	YMR214W	SCJ1
YLR288C	MEC3	YNL291C	MID1
YGR249W	MGA1	YPL137C	GIP3
YNL297C	MON2	YEL043W	YEL043W
YHL031C	GOS1	YDR440W	DOT1
YBR215W	HPC2	YHR167W	THP2
YJL124C	LSM1	YHR039C-A	VMA10
YDL223C	HBT1	YLR093C	NYV1
YOR124C	UBP2	YNL107W	YAF9
YIL053W	RHR2	YPL057C	SUR1
YBL047C	EDE1	YHR115C	DMA1
YCR031C	RPS14A	YDR310C	SUM1
YFR031C-A	RPL2A	YBR217W	ATG12
YLR381W	CTF3	YLR447C	VMA6
YER116C	SLX8	YLR320W	MMS22
YDR254W	CHL4	YOL054W	PSH1
YKR020W	VPS51	YKR030W	GMH1
YBL002W	HTB2	YNL030W	HHF2
YDR254W	CHL4	YPL106C	SSE1
YDR363W-A	SEM1	YJR060W	CBF1
YBR126C	TPS1	YMR142C	RPL13B
YGL066W	SGF73	YNL238W	KEX2

Predictions at threshold level 0.75 continued...			
Gene1 of predicted pair		Gene2 of predicted pair	
Systematic Name	Standard Name	Systematic Name	Standard Name
YJL053W	PEP8	YPL234C	TFP3
YMR004W	MVP1	YPL037C	EGD1
YLR337C	VRP1	YMR116C	ASC1
YJL154C	VPS35	YPL234C	TFP3
YPL106C	SSE1	YGL003C	CDH1
YLR399C	BDF1	YJL006C	CTK2
YBR215W	HPC2	YJR090C	GRR1
YJR032W	CPR7	YLR180W	SAM1
YHR178W	STB5	YDR310C	SUM1
YER110C	KAP123	YOL067C	RTG1
YPL024W	RMI1	YLR398C	SKI2
YHR114W	BZZ1	YKR030W	GMH1
YBL051C	PIN4	YGL237C	HAP2
YGR104C	SRB5	YCL037C	SRO9
YOR327C	SNC2	YJR033C	RAV1
YBR126C	TPS1	YPL161C	BEM4
YDR146C	SWI5	YGR252W	GCN5
YGL115W	SNF4	YNL230C	ELA1
YML115C	VAN1	YOR327C	SNC2
YDR146C	SWI5	YMR311C	GLC8
YOR195W	SLK19	YLR182W	SWI6
YBR195C	MSI1	YPR119W	CLB2
YDR477W	SNF1	YJR090C	GRR1
YDR137W	RGP1	YJR135C	MCM22
YFL023W	BUD27	YNL238W	KEX2
YLR360W	VPS38	YDL006W	PTC1
YBR107C	IML3	YGL003C	CDH1
YIL008W	URM1	YDL047W	SIT4
YPR051W	MAK3	YNL238W	KEX2

Predictions at threshold level 0.75 continued...			
Gene1 of predicted pair		Gene2 of predicted pair	
Systematic Name	Standard Name	Systematic Name	Standard Name
YDR071C	PAA1	YOR368W	RAD17
YLR381W	CTF3	YLR315W	NKP2
YBL021C	HAP3	YDR144C	MKC7
YDR156W	RPA14	YLR399C	BDF1
YNL325C	FIG4	YMR133W	REC114
YDR183W	PLP1	YGL124C	MON1
YDL042C	SIR2	YDR110W	FOB1
YDR293C	SSD1	YOL018C	TLG2
YLR441C	RPS1A	YBR019C	GAL10
YML067C	ERV41	YLR093C	NYV1
YOR337W	TEA1	YKL048C	ELM1
YKR001C	VPS1	YNL041C	COG6
YCR020C-A	MAK31	YHR039C-A	VMA10
YCL037C	SRO9	YNL107W	YAF9
YMR116C	ASC1	YCR077C	PAT1
YGR275W	RTT102	YBR289W	SNF5
YFR010W	UBP6	YLR234W	TOP3
YBR215W	HPC2	YGL151W	NUT1
YER116C	SLX8	YBR082C	UBC4
YKL139W	CTK1	YOR038C	HIR2
YDR080W	VPS41	YOR109W	INP53
YGL087C	MMS2	YGL237C	HAP2
YHR134W	WSS1	YLR135W	SLX4
YGR100W	MDR1	YNR049C	MSO1
YDR395W	SXM1	YDR335W	MSN5
YPR017C	DSS4	YER162C	RAD4
YAL024C	LTE1	YIL159W	BNR1
YHR039C-A	VMA10	YAL002W	VPS8
YBR057C	MUM2	YKR020W	VPS51

Predictions at threshold level 0.75 continued...			
Gene1 of predicted pair		Gene2 of predicted pair	
Systematic Name	Standard Name	Systematic Name	Standard Name
YJL176C	SWI3	YPL057C	SUR1
YNR052C	POP2	YLR386W	VAC14
YOR106W	VAM3	YGR105W	VMA21
YPL213W	LEA1	YIR005W	IST3
YPL120W	VPS30	YAR014C	BUD14
YNL307C	MCK1	YAR014C	BUD14
YML115C	VAN1	YMR214W	SCJ1
YPL137C	GIP3	YGL003C	CDH1
YLR398C	SKI2	YKL041W	VPS24
YPL161C	BEM4	YJL183W	MNN11
YOR106W	VAM3	YGL212W	VAM7
YPL203W	TPK2	YER068W	MOT2
YDR439W	LRS4	YIR005W	IST3
YAL024C	LTE1	YOR308C	SNU66
YDR293C	SSD1	YHR114W	BZZ1
YLR119W	SRN2	YHR081W	LRP1
YGL087C	MMS2	YDR225W	HTA1
YGL033W	HOP2	YPL161C	BEM4
YPR046W	MCM16	YDR310C	SUM1
YGR104C	SRB5	YIR005W	IST3
YDL020C	RPN4	YDR289C	RTT103
YFR036W	CDC26	YML112W	CTK3
YLR083C	EMP70	YEL051W	VMA8
YJL036W	SNX4	YPL037C	EGD1
YIL034C	CAP2	YNL288W	CAF40
YMR095C	SNO1	YKL139W	CTK1
YDL116W	NUP84	YNL218W	MGS1
YJL172W	CPS1	YJL183W	MNN11
YDL223C	HBT1	YPL050C	MNN9

Predictions at threshold level 0.75 continued...			
Gene1 of predicted pair		Gene2 of predicted pair	
Systematic Name	Standard Name	Systematic Name	Standard Name
YBR200W	BEM1	YDR378C	LSM6
YDL020C	RPN4	YFL023W	BUD27
YER118C	SHO1	YLR398C	SKI2
YOR275C	RIM20	YPL161C	BEM4
YNL032W	SIW14	YBL103C	RTG3
YMR133W	REC114	YPR046W	MCM16
YKL116C	PRR1	YIL076W	SEC28
YIL034C	CAP2	YEL053C	MAK10
YDR225W	HTA1	YJR060W	CBF1
YBR200W	BEM1	YKL119C	VPH2
YPL198W	RPL7B	YER102W	RPS8B
YKR035W-A	DID2	YML001W	YPT7
YDR200C	VPS64	YPR119W	CLB2
YLR148W	PEP3	YBR127C	VMA2
YNL325C	FIG4	YLR371W	ROM2
YML103C	NUP188	YGR059W	SPR3
YMR224C	MRE11	YLR094C	GIS3
YOR106W	VAM3	YOR008C	SLG1
YMR142C	RPL13B	YBL103C	RTG3
YBR217W	ATG12	YHR060W	VMA22
YHL020C	OPI1	YHR039C-A	VMA10
YML016C	PPZ1	YBR171W	SEC66
YGL135W	RPL1B	YHR013C	ARD1
YGL087C	MMS2	YOR127W	RGA1
YLR043C	TRX1	YKR001C	VPS1
YLR254C	NDL1	YNL225C	CNM67
YDR004W	RAD57	YGL151W	NUT1
YDR507C	GIN4	YGL031C	RPL24A
YHL011C	PRS3	YHR158C	KEL1

Predictions at threshold level 0.75 continued...			
Gene1 of predicted pair		Gene2 of predicted pair	
Systematic Name	Standard Name	Systematic Name	Standard Name
YPR032W	SRO7	YKR030W	GMH1
YPR046W	MCM16	YLR233C	EST1
YDR443C	SSN2	YDR310C	SUM1
YGR249W	MGA1	YLR371W	ROM2
YER044C	ERG28	YHR200W	RPN10
YIL159W	BNR1	YHR111W	UBA4
YOR043W	WHI2	YPR030W	CSR2
YML012W	ERV25	YNL049C	SFB2
YLR337C	VRP1	YBR171W	SEC66
YNL106C	INP52	YDR318W	MCM21
YFL023W	BUD27	YOL111C	MDY2
YOR337W	TEA1	YGL078C	DBP3
YPR084W	YPR084W	YGR105W	VMA21
YDR448W	ADA2	YNR052C	POP2
YLR083C	EMP70	YHR039C-A	VMA10
YLR292C	SEC72	YDR155C	CPR1
YGL153W	PEX14	YKR020W	VPS51
YML115C	VAN1	YHR013C	ARD1
YCR020C-A	MAK31	YJR060W	CBF1
YDR448W	ADA2	YNL206C	RTT106
YBR280C	SAF1	YLR180W	SAM1
YDL239C	ADY3	YOR177C	MPC54
YHR171W	ATG7	YPL149W	ATG5
YDL002C	NHP10	YER139C	YER139C
YBL051C	PIN4	YEL056W	HAT2
YLR079W	SIC1	YKL117W	SBA1
YIL159W	BNR1	YKL139W	CTK1
YDR103W	STE5	YBL016W	FUS3
YHR039C-A	VMA10	YEL056W	HAT2

Predictions at threshold level 0.75 continued...			
Gene1 of predicted pair		Gene2 of predicted pair	
Systematic Name	Standard Name	Systematic Name	Standard Name
YEL065W	SIT1	YGL124C	MON1
YMR153W	NUP53	YDR358W	GGA1
YGL163C	RAD54	YMR231W	PEP5
YGL250W	RMR1	YJR032W	CPR7
YFR010W	UBP6	YOL111C	MDY2
YEL065W	SIT1	YKR035W-A	DID2
YLR064W	YLR064W	YNL238W	KEX2
YOL054W	PSH1	YNL107W	YAF9
YMR095C	SNO1	YLR386W	VAC14
YPL037C	EGD1	YLR094C	GIS3
YBR200W	BEM1	YPL161C	BEM4
YIL007C	NAS2	YLR324W	PEX30
YLR138W	NHA1	YML132W	COS3
YJL052W	TDH1	YPR119W	CLB2
YMR055C	BUB2	YNL079C	TPM1
YPR029C	APL4	YPL259C	APM1
YDR144C	MKC7	YJR090C	GRR1
YGL147C	RPL9A	YKR099W	BAS1
YPR084W	YPR084W	YER122C	GLO3
YLR083C	EMP70	YLR360W	VPS38
YBR107C	IML3	YOR141C	ARP8
YOR043W	WHI2	YMR230W	RPS10B
YGL250W	RMR1	YJR135C	MCM22
YML001W	YPT7	YDR153C	ENT5
YIL159W	BNR1	YOR035C	SHE4
YPL256C	CLN2	YLR182W	SWI6
YJR140C	HIR3	YOR080W	DIA2
YBL103C	RTG3	YBL025W	RRN10
YER116C	SLX8	YER139C	YER139C

Predictions at threshold level 0.75 continued...			
Gene1 of predicted pair		Gene2 of predicted pair	
Systematic Name	Standard Name	Systematic Name	Standard Name
YPL161C	BEM4	YCR053W	THR4
YML109W	ZDS2	YKR095W	MLP1
YML115C	VAN1	YJL006C	CTK2
YER122C	GLO3	YPR032W	SRO7
YOL111C	MDY2	YLR398C	SKI2
YLL039C	UBI4	YDR378C	LSM6
YAL024C	LTE1	YPL018W	CTF19
YBR082C	UBC4	YJR035W	RAD26
YFL023W	BUD27	YJL095W	BCK1
YGR249W	MGA1	YML115C	VAN1
YMR004W	MVP1	YMR054W	STV1
YDR076W	RAD55	YDR310C	SUM1
YDR382W	RPP2B	YDR378C	LSM6
YGR104C	SRB5	YER068W	MOT2
YPR084W	YPR084W	YIR005W	IST3
YDR129C	SAC6	YGR133W	PEX4
YJL124C	LSM1	YPR119W	CLB2
YHR039C-A	VMA10	YGL148W	ARO2
YNL206C	RTT106	YNL229C	URE2
YAR014C	BUD14	YNL225C	CNM67
YLR371W	ROM2	YMR152W	YIM1
YNL069C	RPL16B	YDR202C	RAV2
YBL106C	SRO77	YNL090W	RHO2
YBL008W	HIR1	YNL206C	RTT106
YDR382W	RPP2B	YNL145W	MFA2
YLR320W	MMS22	YBL101C	ECM21
YGL147C	RPL9A	YLR371W	ROM2
YNL206C	RTT106	YML102W	CAC2
YLR360W	VPS38	YDL226C	GCS1

Predictions at threshold level 0.75 continued...			
Gene1 of predicted pair		Gene2 of predicted pair	
Systematic Name	Standard Name	Systematic Name	Standard Name
YKL126W	YPK1	YDR261C	EXG2
YKL139W	CTK1	YBL101C	ECM21
YMR199W	CLN1	YHR200W	RPN10
YPL129W	TAF14	YOR189W	IES4
YLR410W	VIP1	YMR038C	CCS1
YPL115C	BEM3	YPR046W	MCM16
YLR093C	NYV1	YDL077C	VAM6
YEL053C	MAK10	YGL054C	ERV14
YPL024W	RMI1	YLR192C	HCR1
YDR254W	CHL4	YJL006C	CTK2
YOR375C	GDH1	YJL095W	BCK1
YGR249W	MGA1	YDR358W	GGA1
YLR102C	APC9	YGL003C	CDH1
YOR069W	VPS5	YKL119C	VPH2
YDL127W	PCL2	YGR233C	PHO81
YLR441C	RPS1A	YPL057C	SUR1
YDR335W	MSN5	YJL157C	FAR1
YLR192C	HCR1	YJL172W	CPS1
YLR079W	SIC1	YMR198W	CIK1
YGL135W	RPL1B	YBR082C	UBC4
YKR099W	BAS1	YOR043W	WHI2
YPL137C	GIP3	YBR036C	CSG2
YMR224C	MRE11	YLR394W	CST9
YDR378C	LSM6	YOR320C	GNT1
YBR274W	CHK1	YBR215W	HPC2
YJR099W	YUH1	YOR124C	UBP2
YFL023W	BUD27	YKL126W	YPK1
YLR083C	EMP70	YGL054C	ERV14
YLR064W	YLR064W	YLR144C	ACF2

Predictions at threshold level 0.75 continued...			
**Gene1 of predicted pair**		**Gene2 of predicted pair**	
**Systematic Name**	**Standard Name**	**Systematic Name**	**Standard Name**
YDL006W	PTC1	YAL002W	VPS8
YFR031C-A	RPL2A	YER040W	GLN3
YNL106C	INP52	YFR036W	CDC26
YNL201C	PSY2	YOR141C	ARP8
YPL022W	RAD1	YJR060W	CBF1
YNR052C	POP2	YJR035W	RAD26
YLL007C	YLL007C	YAL002W	VPS8
YGL033W	HOP2	YNL325C	FIG4
YDL020C	RPN4	YGL175C	SAE2
YBR107C	IML3	YGR188C	BUB1
YGR104C	SRB5	YCR077C	PAT1
YBR171W	SEC66	YGL151W	NUT1
YLR320W	MMS22	YDR310C	SUM1
YMR004W	MVP1	YNL044W	YIP3
YKR048C	NAP1	YOR327C	SNC2
YBR171W	SEC66	YGR252W	GCN5
YPR018W	RLF2	YDR207C	UME6
YLR292C	SEC72	YBR107C	IML3
YCR081W	SRB8	YBR260C	RGD1
YHR200W	RPN10	YLR370C	ARC18
YDR443C	SSN2	YGR249W	MGA1
YGL054C	ERV14	YPL120W	VPS30
YHR111W	UBA4	YJR060W	CBF1
YKL126W	YPK1	YDL223C	HBT1
YHR152W	SPO12	YJL095W	BCK1
YLR337C	VRP1	YMR127C	SAS2
YLR288C	MEC3	YPR120C	CLB5
YOR308C	SNU66	YOR124C	UBP2
YIL053W	RHR2	YER122C	GLO3

Predictions at threshold level 0.75 continued...			
Gene1 of predicted pair		Gene2 of predicted pair	
Systematic Name	Standard Name	Systematic Name	Standard Name
YDR108W	GSG1	YCL001W	RER1
YIL053W	RHR2	YJR032W	CPR7
YBR216C	YBP1	YML007W	YAP1
YMR017W	SPO20	YNR049C	MSO1
YOR290C	SNF2	YHR108W	GGA2
YDR320C	SWA2	YDR137W	RGP1
YGL223C	COG1	YNL041C	COG6
YMR095C	SNO1	YLR180W	SAM1
YOR195W	SLK19	YKL114C	APN1
YLR018C	POM34	YDL059C	RAD59
YKL116C	PRR1	YER040W	GLN3
YOR106W	VAM3	YLR309C	IMH1
YFR010W	UBP6	YIL076W	SEC28
YLR399C	BDF1	YPR072W	NOT5
YMR199W	CLN1	YKR048C	NAP1
YKR061W	KTR2	YIR005W	IST3
YNR072W	HXT17	YBR019C	GAL10
YNL307C	MCK1	YGR188C	BUB1
YDR080W	VPS41	YCL001W	RER1
YHR098C	SFB3	YGR229C	SMI1
YOR076C	SKI7	YJR140C	HIR3
YDR443C	SSN2	YER122C	GLO3
YDR289C	RTT103	YHR114W	BZZ1
YKL006W	RPL14A	YKL041W	VPS24
YPR051W	MAK3	YDR318W	MCM21
YJR056C	YJR056C	YOR066W	MSA1
YFR040W	SAP155	YJL095W	BCK1
YPL115C	BEM3	YMR273C	ZDS1
YNL297C	MON2	YLR032W	RAD5

Predictions at threshold level 0.75 continued...			
Gene1 of predicted pair		Gene2 of predicted pair	
Systematic Name	Standard Name	Systematic Name	Standard Name
YLR090W	XDJ1	YDR477W	SNF1
YJR135C	MCM22	YJL190C	RPS22A
YHL031C	GOS1	YER162C	RAD4
YGL212W	VAM7	YLR309C	IMH1
YGL087C	MMS2	YEL056W	HAT2
YCR020C-A	MAK31	YCR066W	RAD18
YDL184C	RPL41A	YOR290C	SNF2
YNL307C	MCK1	YPL018W	CTF19
YOR076C	SKI7	YHR167W	THP2
YPL137C	GIP3	YMR230W	RPS10B
YOR127W	RGA1	YBL021C	HAP3
YBR108W	YBR108W	YKR099W	BAS1
YDR310C	SUM1	YMR230W	RPS10B
YBL078C	ATG8	YHL031C	GOS1
YKR020W	VPS51	YJL004C	SYS1
YLR337C	VRP1	YFR036W	CDC26
YPR066W	UBA3	YDR139C	RUB1
YHL031C	GOS1	YJL036W	SNX4
YPR131C	NAT3	YHR010W	RPL27A
YMR054W	STV1	YNL051W	COG5
YGL200C	EMP24	YKR014C	YPT52
YEL013W	VAC8	YAL002W	VPS8
YOR035C	SHE4	YJL183W	MNN11
YOR069W	VPS5	YJR040W	GEF1
YNL325C	FIG4	YBR280C	SAF1
YMR199W	CLN1	YGL190C	CDC55
YER116C	SLX8	YMR153W	NUP53
YPL249C-A	RPL36B	YCR009C	RVS161
YBL008W	HIR1	YNL236W	SIN4

Predictions at threshold level 0.75 continued...			
Gene1 of predicted pair		Gene2 of predicted pair	
Systematic Name	Standard Name	Systematic Name	Standard Name
YBR126C	TPS1	YLR320W	MMS22
YOR043W	WHI2	YMR152W	YIM1
YDR320C	SWA2	YNR006W	VPS27
YDL185W	TFP1	YNL107W	YAF9
YMR026C	PEX12	YDR142C	PEX7
YPL037C	EGD1	YFL033C	RIM15
YPR046W	MCM16	YJR035W	RAD26
YML058W	SML1	YGL094C	PAN2
YNL215W	IES2	YGR270W	YTA7
YPL253C	VIK1	YDR155C	CPR1
YDR358W	GGA1	YLR398C	SKI2
YLR320W	MMS22	YGR188C	BUB1
YCR065W	HCM1	YJL006C	CTK2
YNL253W	TEX1	YBR020W	GAL1
YBR171W	SEC66	YDL083C	RPS16B
YPL022W	RAD1	YOR368W	RAD17
YKL116C	PRR1	YBR171W	SEC66
YMR172W	HOT1	YGR054W	YGR054W
YPL096C-A	ERI1	YER111C	SWI4
YKL139W	CTK1	YKL110C	KTI12
YNL087W	TCB2	YFR036W	CDC26
YDR004W	RAD57	YPL178W	CBC2
YDL184C	RPL41A	YDL083C	RPS16B
YPL022W	RAD1	YGL163C	RAD54
YHR178W	STB5	YAL002W	VPS8
YOR127W	RGA1	YJR135C	MCM22
YOL051W	GAL11	YER068W	MOT2
YPL001W	HAT1	YCR077C	PAT1
YOR290C	SNF2	YLR371W	ROM2

Predictions at threshold level 0.75 continued...			
Gene1 of predicted pair		Gene2 of predicted pair	
Systematic Name	Standard Name	Systematic Name	Standard Name
YOR076C	SKI7	YHR077C	NMD2
YAL024C	LTE1	YNL076W	MKS1
YOR039W	CKB2	YNL079C	TPM1
YJL036W	SNX4	YKR030W	GMH1
YNL248C	RPA49	YPR120C	CLB5
YMR224C	MRE11	YDL083C	RPS16B
YNL106C	INP52	YLR079W	SIC1
YHR178W	STB5	YBL021C	HAP3
YDL083C	RPS16B	YGL124C	MON1
YFR036W	CDC26	YOL054W	PSH1
YDL082W	RPL13A	YGL246C	RAI1
YDR004W	RAD57	YKL101W	HSL1
YDR378C	LSM6	YNL079C	TPM1
YBL071W-A	KTI11	YKL191W	DPH2
YML071C	COG8	YPL120W	VPS30
YML016C	PPZ1	YKL101W	HSL1
YLR094C	GIS3	YOL081W	IRA2
YDL116W	NUP84	YDL059C	RAD59
YBL047C	EDE1	YHL002W	HSE1
YJL036W	SNX4	YDL077C	VAM6
YDR254W	CHL4	YNL236W	SIN4
YGL078C	DBP3	YIL076W	SEC28
YNL058C	YNL058C	YER122C	GLO3
YOR243C	PUS7	YCL037C	SRO9
YML095C	RAD10	YML102W	CAC2
YJL138C	TIF2	YGL019W	CKB1
YDR137W	RGP1	YFR036W	CDC26
YNL090W	RHO2	YDL240W	LRG1
YER115C	SPR6	YMR199W	CLN1

Predictions at threshold level 0.75 continued...			
Gene1 of predicted pair		Gene2 of predicted pair	
Systematic Name	Standard Name	Systematic Name	Standard Name
YLR337C	VRP1	YPL096C-A	ERI1
YOR035C	SHE4	YLR180W	SAM1
YIL156W	UBP7	YOL126C	MDH2
YKL048C	ELM1	YDL077C	VAM6
YLR079W	SIC1	YKL213C	DOA1
YLR309C	IMH1	YAR014C	BUD14
YER144C	UBP5	YEL060C	PRB1
YAL051W	OAF1	YEL056W	HAT2
YKR057W	RPS21A	YKL048C	ELM1
YKL048C	ELM1	YLR370C	ARC18
YLR234W	TOP3	YNL297C	MON2
YDL116W	NUP84	YDL013W	SLX5
YER118C	SHO1	YJL183W	MNN11
YDR383C	NKP1	YOL054W	PSH1
YOR109W	INP53	YCR066W	RAD18
YDR137W	RGP1	YJR084W	CSN12
YNL044W	YIP3	YKR020W	VPS51
YNL325C	FIG4	YBR036C	CSG2
YGR108W	CLB1	YBL051C	PIN4
YPR051W	MAK3	YKR020W	VPS51
YLR095C	IOC2	YMR311C	GLC8
YNL032W	SIW14	YLR370C	ARC18
YKR048C	NAP1	YGL031C	RPL24A
YBR108W	YBR108W	YJL183W	MNN11
YJR032W	CPR7	YHR081W	LRP1
YGR200C	ELP2	YBL071W-A	KTI11
YNL215W	IES2	YER161C	SPT2
YNL291C	MID1	YJR135C	MCM22
YKL116C	PRR1	YNL058C	YNL058C

Predictions at threshold level 0.75 continued...			
Gene1 of predicted pair		Gene2 of predicted pair	
Systematic Name	Standard Name	Systematic Name	Standard Name
YDR477W	SNF1	YDR358W	GGA1
YOR337W	TEA1	YPL161C	BEM4
YPL253C	VIK1	YNL107W	YAF9
YEL065W	SIT1	YHR060W	VMA22
YCR097W	HMRA1	YCR039C	MATALPHA2
YMR095C	SNO1	YOL081W	IRA2
YLR119W	SRN2	YBR217W	ATG12
YIL076W	SEC28	YKR099W	BAS1
YMR152W	YIM1	YER139C	YER139C
YPL001W	HAT1	YBR274W	CHK1
YPR120C	CLB5	YOL091W	SPO21
YNL106C	INP52	YML016C	PPZ1
YGL153W	PEX14	YJL095W	BCK1
YML117W	NAB6	YMR038C	CCS1
YPL115C	BEM3	YCR086W	CSM1
YOR066W	MSA1	YBR019C	GAL10
YCL001W	RER1	YNL238W	KEX2
YOR197W	MCA1	YBL106C	SRO77
YIL076W	SEC28	YEL053C	MAK10
YLR360W	VPS38	YMR199W	CLN1
YDR363W-A	SEM1	YGR252W	GCN5
YPR017C	DSS4	YER122C	GLO3
YDR443C	SSN2	YGL115W	SNF4
YLR360W	VPS38	YER162C	RAD4
YMR199W	CLN1	YEL036C	ANP1
YGL147C	RPL9A	YER122C	GLO3
YNL068C	FKH2	YNL229C	URE2
YLR135W	SLX4	YCR086W	CSM1
YDR245W	MNN10	YPL213W	LEA1

Predictions at threshold level 0.75 continued...			
Gene1 of predicted pair		Gene2 of predicted pair	
Systematic Name	Standard Name	Systematic Name	Standard Name
YGL133W	ITC1	YIL008W	URM1
YER068W	MOT2	YGR134W	CAF130
YER110C	KAP123	YBR171W	SEC66
YGL212W	VAM7	YDR080W	VPS41
YMR055C	BUB2	YPR046W	MCM16
YNL325C	FIG4	YNL307C	MCK1
YNL297C	MON2	YGR105W	VMA21
YBR200W	BEM1	YDR200C	VPS64
YMR224C	MRE11	YHR115C	DMA1
YGR020C	VMA7	YOL018C	TLG2
YKL032C	IXR1	YDL155W	CLB3
YGL078C	DBP3	YDL077C	VAM6
YDR146C	SWI5	YDR207C	UME6
YNL107W	YAF9	YLR182W	SWI6
YOR270C	VPH1	YMR152W	YIM1
YOL121C	RPS19A	YMR311C	GLC8
YPL042C	SSN3	YOR080W	DIA2
YMR164C	MSS11	YKL068W	NUP100
YMR231W	PEP5	YLR093C	NYV1
YOR127W	RGA1	YJL183W	MNN11
YHR012W	VPS29	YJL095W	BCK1
YBL072C	RPS8A	YOL081W	IRA2
YER144C	UBP5	YJR084W	CSN12
YDR103W	STE5	YCL032W	STE50
YLR386W	VAC14	YBR200W	BEM1
YFL048C	EMP47	YEL036C	ANP1
YLR234W	TOP3	YNL016W	PUB1
YBL103C	RTG3	YOL067C	RTG1
YJL078C	PRY3	YFL044C	OTU1

Predictions at threshold level 0.75 continued...			
**Gene1 of predicted pair**		**Gene2 of predicted pair**	
**Systematic Name**	**Standard Name**	**Systematic Name**	**Standard Name**
YKR072C	SIS2	YPL001W	HAT1
YMR199W	CLN1	YMR311C	GLC8
YOR001W	RRP6	YLR182W	SWI6
YIL159W	BNR1	YPL037C	EGD1
YBR108W	YBR108W	YER139C	YER139C
YHR161C	YAP1801	YAL030W	SNC1
YLR234W	TOP3	YMR038C	CCS1
YJL036W	SNX4	YPR049C	ATG11
YKL139W	CTK1	YNL325C	FIG4
YOR275C	RIM20	YKR048C	NAP1
YPL253C	VIK1	YOL067C	RTG1
YDL116W	NUP84	YMR198W	CIK1
YMR224C	MRE11	YLR441C	RPS1A
YER044C	ERG28	YML071C	COG8
YLR079W	SIC1	YJR063W	RPA12
YAL014C	SYN8	YOR036W	PEP12
YNL106C	INP52	YPL018W	CTF19
YMR224C	MRE11	YMR055C	BUB2
YPL253C	VIK1	YHR111W	UBA4
YBL008W	HIR1	YGL003C	CDH1
YDL006W	PTC1	YBR082C	UBC4
YLR006C	SSK1	YGL038C	OCH1
YPR072W	NOT5	YOR064C	YNG1
YFR036W	CDC26	YLR398C	SKI2
YLR360W	VPS38	YOR035C	SHE4
YHR178W	STB5	YMR055C	BUB2
YHR013C	ARD1	YBL025W	RRN10
YDR071C	PAA1	YKL114C	APN1
YMR224C	MRE11	YHR111W	UBA4

Predictions at threshold level 0.75 continued...			
Gene1 of predicted pair		Gene2 of predicted pair	
Systematic Name	Standard Name	Systematic Name	Standard Name
YIL036W	CST6	YGR270W	YTA7
YKL048C	ELM1	YJL117W	PHO86
YCL037C	SRO9	YKR020W	VPS51
YBR108W	YBR108W	YHL002W	HSE1
YHR084W	STE12	YHR052W	CIC1
YML112W	CTK3	YOL054W	PSH1
YPR072W	NOT5	YGR134W	CAF130
YBL051C	PIN4	YOL067C	RTG1
YGR104C	SRB5	YBR289W	SNF5
YPR084W	YPR084W	YOR069W	VPS5
YOR035C	SHE4	YMR089C	YTA12
YDR363W-A	SEM1	YNL116W	DMA2
YKL007W	CAP1	YCR088W	ABP1
YOR112W	CEX1	YPL105C	YPL105C
YDR225W	HTA1	YDR227W	SIR4
YFR031C-A	RPL2A	YDR080W	VPS41
YOR144C	ELG1	YHR056C	RSC30
YBR065C	ECM2	YBR188C	NTC20
YPL115C	BEM3	YLR381W	CTF3
YBR194W	SOY1	YMR202W	ERG2
YNL054W	VAC7	YOL081W	IRA2
YDL101C	DUN1	YER116C	SLX8
YOR066W	MSA1	YJR084W	CSN12
YGR105W	VMA21	YDL192W	ARF1
YLR182W	SWI6	YOL081W	IRA2
YLR357W	RSC2	YNL107W	YAF9
YDL065C	PEX19	YAL055W	PEX22
YKL007W	CAP1	YLR006C	SSK1
YHR111W	UBA4	YJR063W	RPA12

Predictions at threshold level 0.75 continued...			
Gene1 of predicted pair		Gene2 of predicted pair	
Systematic Name	Standard Name	Systematic Name	Standard Name
YOR043W	WHI2	YLR094C	GIS3
YLR447C	VMA6	YEL051W	VMA8
YIL159W	BNR1	YOR043W	WHI2
YMR095C	SNO1	YNL333W	SNZ2
YHL027W	RIM101	YMR032W	HOF1
YOR106W	VAM3	YAL002W	VPS8
YJL006C	CTK2	YJR035W	RAD26
YNL297C	MON2	YKL119C	VPH2
YMR133W	REC114	YJL095W	BCK1
YOR375C	GDH1	YOR141C	ARP8
YAL024C	LTE1	YOR066W	MSA1
YDR440W	DOT1	YHR167W	THP2
YOL018C	TLG2	YNL107W	YAF9
YPL253C	VIK1	YLR032W	RAD5
YKR095W	MLP1	YJR090C	GRR1
YDL059C	RAD59	YMR231W	PEP5
YKR059W	TIF1	YOL081W	IRA2
YNL032W	SIW14	YBR200W	BEM1
YOL062C	APM4	YJR005W	APL1
YBL051C	PIN4	YDR507C	GIN4
YCR034W	FEN1	YGL019W	CKB1
YGR072W	UPF3	YEL051W	VMA8
YIL159W	BNR1	YDR261C	EXG2
YOR290C	SNF2	YGL025C	PGD1
YOR043W	WHI2	YMR133W	REC114
YLR325C	RPL38	YIL133C	RPL16A
YLR234W	TOP3	YML062C	MFT1
YPR131C	NAT3	YFL023W	BUD27
YGR241C	YAP1802	YJR035W	RAD26

Predictions at threshold level 0.75 continued...			
Gene1 of predicted pair		Gene2 of predicted pair	
Systematic Name	Standard Name	Systematic Name	Standard Name
YJL154C	VPS35	YJL004C	SYS1
YMR142C	RPL13B	YMR311C	GLC8
YBR173C	UMP1	YPL161C	BEM4
YPL161C	BEM4	YKL101W	HSL1
YDL116W	NUP84	YJL127C	SPT10
YLR095C	IOC2	YPL024W	RMI1
YDR004W	RAD57	YDL013W	SLX5
YFR036W	CDC26	YLR093C	NYV1
YLR452C	SST2	YOR327C	SNC2
YER110C	KAP123	YAL002W	VPS8
YMR016C	SOK2	YJR032W	CPR7
YMR004W	MVP1	YFL033C	RIM15
YDR318W	MCM21	YGL124C	MON1
YEL051W	VMA8	YPL120W	VPS30
YMR109W	MYO5	YGR192C	TDH3
YER087C-B	SBH1	YGL244W	RTF1
YEL050C	RML2	YGL115W	SNF4
YBR173C	UMP1	YBR108W	YBR108W
YLR399C	BDF1	YGR270W	YTA7
YJR090C	GRR1	YDR139C	RUB1
YNL291C	MID1	YHR060W	VMA22
YNL325C	FIG4	YHR039C-A	VMA10
YDR293C	SSD1	YKR030W	GMH1
YOR327C	SNC2	YNL076W	MKS1
YDR004W	RAD57	YOR038C	HIR2
YMR029C	FAR8	YMR052W	FAR3
YDR245W	MNN10	YHL011C	PRS3
YDR225W	HTA1	YOL148C	SPT20
YNL041C	COG6	YPR032W	SRO7

Predictions at threshold level 0.75 continued...			
Gene1 of predicted pair		Gene2 of predicted pair	
Systematic Name	Standard Name	Systematic Name	Standard Name
YGL163C	RAD54	YFR030W	MET10
YIR003W	YIR003W	YNL020C	ARK1
YLR119W	SRN2	YBR280C	SAF1
YDR108W	GSG1	YMR004W	MVP1
YOR195W	SLK19	YDR378C	LSM6
YKL048C	ELM1	YDL047W	SIT4
YOR001W	RRP6	YNL236W	SIN4
YGR019W	UGA1	YPL253C	VIK1
YBR200W	BEM1	YNL041C	COG6
YBR108W	YBR108W	YKL048C	ELM1
YDR174W	HMO1	YDR439W	LRS4
YCL037C	SRO9	YJR060W	CBF1
YPL137C	GIP3	YLR370C	ARC18
YCL037C	SRO9	YDR378C	LSM6
YGL212W	VAM7	YJR032W	CPR7
YNL236W	SIN4	YGL151W	NUT1
YLR181C	VTA1	YKL002W	DID4
YMR026C	PEX12	YLR320W	MMS22
YIL159W	BNR1	YJR032W	CPR7
YDR225W	HTA1	YOR008C	SLG1
YGL200C	EMP24	YLR370C	ARC18
YEL053C	MAK10	YJL190C	RPS22A
YMR055C	BUB2	YDR289C	RTT103
YNL297C	MON2	YNL041C	COG6
YGL033W	HOP2	YDR477W	SNF1
YCR020C-A	MAK31	YAR014C	BUD14
YJR099W	YUH1	YLR128W	DCN1
YHR039C-A	VMA10	YGL163C	RAD54
YDR156W	RPA14	YMR038C	CCS1

Predictions at threshold level 0.75 continued...			
Gene1 of predicted pair		Gene2 of predicted pair	
Systematic Name	Standard Name	Systematic Name	Standard Name
YNR072W	HXT17	YMR214W	SCJ1
YIL034C	CAP2	YHR012W	VPS29
YML103C	NUP188	YLR079W	SIC1
YER162C	RAD4	YLR398C	SKI2
YOR337W	TEA1	YNL238W	KEX2
YER116C	SLX8	YMR230W	RPS10B
YMR142C	RPL13B	YPL057C	SUR1
YDR225W	HTA1	YDR176W	NGG1
YJR024C	MDE1	YPL256C	CLN2
YMR026C	PEX12	YOL081W	IRA2
YPR171W	BSP1	YHL002W	HSE1
YER096W	SHC1	YBR260C	RGD1
YCR034W	FEN1	YLR435W	TSR2
YDR137W	RGP1	YCR053W	THR4
YHR119W	SET1	YDR469W	SDC1
YKR001C	VPS1	YOR035C	SHE4
YGL237C	HAP2	YKL109W	HAP4
YGL163C	RAD54	YAR014C	BUD14
YKL126W	YPK1	YEL060C	PRB1
YML112W	CTK3	YOL081W	IRA2
YPL037C	EGD1	YHR081W	LRP1
YIL053W	RHR2	YNL041C	COG6
YJR140C	HIR3	YDR245W	MNN10
YKL048C	ELM1	YDR264C	AKR1
YMR127C	SAS2	YDL155W	CLB3
YNL214W	PEX17	YAL055W	PEX22
YDR174W	HMO1	YLR254C	NDL1
YOR080W	DIA2	YDR137W	RGP1
YER116C	SLX8	YLR094C	GIS3

Predictions at threshold level 0.75 continued...			
Gene1 of predicted pair		Gene2 of predicted pair	
Systematic Name	Standard Name	Systematic Name	Standard Name
YHR134W	WSS1	YPL018W	CTF19
YEL053C	MAK10	YHR039C-A	VMA10
YOL067C	RTG1	YDR310C	SUM1
YLR386W	VAC14	YNR072W	HXT17
YKR099W	BAS1	YPR030W	CSR2
YLR315W	NKP2	YOL054W	PSH1
YOR039W	CKB2	YBR009C	HHF1
YMR095C	SNO1	YKL116C	PRR1
YHR200W	RPN10	YNL238W	KEX2
YNR052C	POP2	YGR188C	BUB1
YLR119W	SRN2	YER044C	ERG28
YLR234W	TOP3	YLR350W	ORM2
YLR357W	RSC2	YNL030W	HHF2
YJR032W	CPR7	YOL121C	RPS19A
YBR217W	ATG12	YDR484W	VPS52
YBR042C	CST26	YGL200C	EMP24
YJL095W	BCK1	YGL253W	HXK2
YFR010W	UBP6	YHR039C-A	VMA10
YPL161C	BEM4	YDR155C	CPR1
YPL234C	TFP3	YOL108C	INO4
YKR061W	KTR2	YJR060W	CBF1
YLR386W	VAC14	YGL252C	RTG2
YNL127W	FAR11	YNL051W	COG5
YBR217W	ATG12	YER139C	YER139C
YHL011C	PRS3	YOL081W	IRA2
YDL192W	ARF1	YMR311C	GLC8
YLR288C	MEC3	YOR363C	PIP2
YGL134W	PCL10	YPL240C	HSP82
YGL060W	YBP2	YOR083W	WHI5

Predictions at threshold level 0.75 continued...			
Gene1 of predicted pair		Gene2 of predicted pair	
Systematic Name	Standard Name	Systematic Name	Standard Name
YLR090W	XDJ1	YJL183W	MNN11
YDR153C	ENT5	YOL108C	INO4
YOR035C	SHE4	YDL077C	VAM6
YNL225C	CNM67	YOL081W	IRA2
YPR084W	YPR084W	YCR020C-A	MAK31
YHR041C	SRB2	YNR023W	SNF12
YJL060W	BNA3	YDR171W	HSP42
YIL159W	BNR1	YDL077C	VAM6
YDR080W	VPS41	YDR310C	SUM1
YKL048C	ELM1	YJR032W	CPR7
YFR010W	UBP6	YJR140C	HIR3
YMR095C	SNO1	YPL137C	GIP3
YNL032W	SIW14	YFR036W	CDC26
YIL034C	CAP2	YJL095W	BCK1
YOR109W	INP53	YNR006W	VPS27
YDR137W	RGP1	YBR280C	SAF1
YDR456W	NHX1	YJL095W	BCK1
YMR294W	JNM1	YGL190C	CDC55
YGR105W	VMA21	YKL119C	VPH2
YHR178W	STB5	YDR254W	CHL4
YDR004W	RAD57	YBL008W	HIR1
YMR026C	PEX12	YNL041C	COG6
YMR276W	DSK2	YJR099W	YUH1
YMR142C	RPL13B	YPR030W	CSR2
YHR012W	VPS29	YJL004C	SYS1
YHR200W	RPN10	YMR230W	RPS10B
YBR258C	SHG1	YPL057C	SUR1
YDL155W	CLB3	YBL101C	ECM21
YFR040W	SAP155	YER122C	GLO3

Predictions at threshold level 0.75 continued...			
Gene1 of predicted pair		Gene2 of predicted pair	
Systematic Name	Standard Name	Systematic Name	Standard Name
YMR216C	SKY1	YPR032W	SRO7
YBR171W	SEC66	YPL161C	BEM4
YEL009C	GCN4	YLR399C	BDF1
YNL201C	PSY2	YOL081W	IRA2
YPR018W	RLF2	YGR252W	GCN5
YBR215W	HPC2	YBR036C	CSG2
YNL291C	MID1	YML028W	TSA1
YMR026C	PEX12	YER162C	RAD4
YFR010W	UBP6	YER139C	YER139C
YLR254C	NDL1	YLR182W	SWI6
YPL234C	TFP3	YAR014C	BUD14
YIL008W	URM1	YAR014C	BUD14
YOR270C	VPH1	YOL081W	IRA2
YHR060W	VMA22	YLR398C	SKI2
YAL024C	LTE1	YGR135W	PRE9
YOR275C	RIM20	YJL117W	PHO86
YMR109W	MYO5	YDR254W	CHL4
YIL008W	URM1	YDR183W	PLP1
YLR381W	CTF3	YGL124C	MON1
YMR224C	MRE11	YBR019C	GAL10
YOR195W	SLK19	YDR076W	RAD55
YDR379W	RGA2	YPL161C	BEM4
YML016C	PPZ1	YOL081W	IRA2
YNL307C	MCK1	YOL121C	RPS19A
YIL036W	CST6	YPL152W	RRD2
YGL033W	HOP2	YLR441C	RPS1A
YGR133W	PEX4	YAL055W	PEX22
YOR270C	VPH1	YLR371W	ROM2
YPR046W	MCM16	YJR084W	CSN12

Predictions at threshold level 0.75 continued...			
Gene1 of predicted pair		Gene2 of predicted pair	
Systematic Name	Standard Name	Systematic Name	Standard Name
YJR135C	MCM22	YPR119W	CLB2
YLR095C	IOC2	YOR043W	WHI2
YKR001C	VPS1	YJR058C	APS2
YOL121C	RPS19A	YJR084W	CSN12
YDL020C	RPN4	YNL238W	KEX2
YPL253C	VIK1	YGL003C	CDH1
YJL176C	SWI3	YBL047C	EDE1
YDL135C	RDI1	YDL240W	LRG1
YHR119W	SET1	YLR288C	MEC3
YDR202C	RAV2	YNL238W	KEX2
YIL159W	BNR1	YNL307C	MCK1
YAL024C	LTE1	YKR072C	SIS2
YOR127W	RGA1	YDR155C	CPR1
YJL124C	LSM1	YDR318W	MCM21
YJR032W	CPR7	YGL151W	NUT1
YLR234W	TOP3	YIL008W	URM1
YHR206W	SKN7	YNL027W	CRZ1
YFL023W	BUD27	YDL083C	RPS16B
YNL106C	INP52	YMR055C	BUB2
YER110C	KAP123	YJL190C	RPS22A
YPL042C	SSN3	YBR107C	IML3
YPL096C-A	ERI1	YHR030C	SLT2
YHR039C-A	VMA10	YAR014C	BUD14
YNL215W	IES2	YKL139W	CTK1
YER001W	MNN1	YNL051W	COG5
YMR224C	MRE11	YGL161C	YIP5
YOR275C	RIM20	YHR012W	VPS29
YGR135W	PRE9	YEL036C	ANP1
YLR429W	CRN1	YDL226C	GCS1

Predictions at threshold level 0.75 continued...			
Gene1 of predicted pair		Gene2 of predicted pair	
Systematic Name	Standard Name	Systematic Name	Standard Name
YMR153W	NUP53	YPL137C	GIP3
YDL006W	PTC1	YOR036W	PEP12
YMR016C	SOK2	YLR441C	RPS1A
YNL206C	RTT106	YHR111W	UBA4
YDR379W	RGA2	YGL163C	RAD54
YDR443C	SSN2	YMR153W	NUP53
YDR323C	PEP7	YOR036W	PEP12
YJL124C	LSM1	YDR310C	SUM1
YFL023W	BUD27	YNL307C	MCK1
YKL080W	VMA5	YKL041W	VPS24
YNL307C	MCK1	YJR135C	MCM22
YDL101C	DUN1	YMR055C	BUB2
YPL105C	YPL105C	YML001W	YPT7
YBR171W	SEC66	YIL008W	URM1
YHR039C-A	VMA10	YPR030W	CSR2
YDL116W	NUP84	YLR381W	CTF3
YDL020C	RPN4	YPL079W	RPL21B
YOR127W	RGA1	YBR084C-A	RPL19A
YDR254W	CHL4	YNL147W	LSM7
YBR274W	CHK1	YLR320W	MMS22
YGR159C	NSR1	YOR141C	ARP8
YPL037C	EGD1	YER044C	ERG28
YGR104C	SRB5	YDL002C	NHP10
YGR104C	SRB5	YDR363W-A	SEM1
YDR129C	SAC6	YKR020W	VPS51
YBR217W	ATG12	YMR152W	YIM1
YPL057C	SUR1	YOR124C	UBP2
YHR010W	RPL27A	YOR368W	RAD17
YOR001W	RRP6	YOR124C	UBP2

Predictions at threshold level 0.75 continued...			
Gene1 of predicted pair		Gene2 of predicted pair	
Systematic Name	Standard Name	Systematic Name	Standard Name
YGR020C	VMA7	YML001W	YPT7
YDR156W	RPA14	YJL095W	BCK1
YLR192C	HCR1	YLR386W	VAC14
YNL238W	KEX2	YPR030W	CSR2
YGR249W	MGA1	YMR152W	YIM1
YDR439W	LRS4	YGL163C	RAD54
YLR094C	GIS3	YCR053W	THR4
YDL161W	ENT1	YJL029C	VPS53
YJL172W	CPS1	YBR019C	GAL10
YOR001W	RRP6	YPL178W	CBC2
YAL024C	LTE1	YHR081W	LRP1
YJR083C	ACF4	YOR284W	HUA2
YJL124C	LSM1	YDL155W	CLB3
YKL048C	ELM1	YIR005W	IST3
YGL163C	RAD54	YJL095W	BCK1
YFL031W	HAC1	YDR207C	UME6
YML088W	UFO1	YDR139C	RUB1
YGL147C	RPL9A	YGL212W	VAM7
YER139C	YER139C	YJR084W	CSN12
YPL253C	VIK1	YJL183W	MNN11
YMR038C	CCS1	YNL107W	YAF9
YPL129W	TAF14	YNL030W	HHF2
YBR108W	YBR108W	YMR016C	SOK2
YMR167W	MLH1	YNL076W	MKS1
YGR108W	CLB1	YGR054W	YGR054W
YEL009C	GCN4	YMR091C	NPL6
YLR337C	VRP1	YKR020W	VPS51
YMR016C	SOK2	YGL003C	CDH1
YPL161C	BEM4	YJR084W	CSN12

Predictions at threshold level 0.75 continued...			
Gene1 of predicted pair		Gene2 of predicted pair	
Systematic Name	Standard Name	Systematic Name	Standard Name
YDR334W	SWR1	YBR089C-A	NHP6B
YMR231W	PEP5	YDL077C	VAM6
YLR234W	TOP3	YOL081W	IRA2
YLR453C	RIF2	YER098W	UBP9
YDR137W	RGP1	YMR054W	STV1
YCR066W	RAD18	YER122C	GLO3
YAL056W	GPB2	YOR107W	RGS2
YNL068C	FKH2	YDL047W	SIT4
YPL018W	CTF19	YEL056W	HAT2
YGL212W	VAM7	YDR153C	ENT5
YML016C	PPZ1	YLR429W	CRN1
YDL006W	PTC1	YDR153C	ENT5
YJL136C	RPS21B	YDR379W	RGA2
YCR065W	HCM1	YER114C	BOI2
YIL159W	BNR1	YBR019C	GAL10
YBL008W	HIR1	YKR072C	SIS2
YHR060W	VMA22	YML001W	YPT7
YNL307C	MCK1	YPR120C	CLB5
YOR290C	SNF2	YNR072W	HXT17
YLR386W	VAC14	YNL225C	CNM67
YNL288W	CAF40	YGR134W	CAF130
YML016C	PPZ1	YOR358W	HAP5
YCR077C	PAT1	YAL002W	VPS8
YJL176C	SWI3	YBR057C	MUM2
YDR477W	SNF1	YLR370C	ARC18
YML001W	YPT7	YCR053W	THR4
YMR224C	MRE11	YDR439W	LRS4
YOR132W	VPS17	YBR171W	SEC66
YLR288C	MEC3	YER162C	RAD4

Predictions at threshold level 0.75 continued...			
Gene1 of predicted pair		Gene2 of predicted pair	
Systematic Name	Standard Name	Systematic Name	Standard Name
YLR234W	TOP3	YGR184C	UBR1
YDR422C	SIP1	YJR090C	GRR1
YOR275C	RIM20	YKL041W	VPS24
YKL116C	PRR1	YBL103C	RTG3
YER110C	KAP123	YGR054W	YGR054W
YBL008W	HIR1	YJR032W	CPR7
YER116C	SLX8	YOR290C	SNF2
YJL210W	PEX2	YGR077C	PEX8
YMR273C	ZDS1	YPL256C	CLN2
YOR035C	SHE4	YLR370C	ARC18
YCR081W	SRB8	YLR371W	ROM2
YBR171W	SEC66	YLR398C	SKI2
YML094W	GIM5	YEL022W	GEA2
YBL102W	SFT2	YOR069W	VPS5
YNL264C	PDR17	YGL025C	PGD1
YEL027W	CUP5	YHR060W	VMA22
YHR134W	WSS1	YHR111W	UBA4
YMR026C	PEX12	YBL103C	RTG3
YKR020W	VPS51	YER122C	GLO3
YPL234C	TFP3	YPR032W	SRO7
YDR108W	GSG1	YPR051W	MAK3
YIL162W	SUC2	YEL036C	ANP1
YGR249W	MGA1	YNL238W	KEX2
YDR103W	STE5	YDR309C	GIC2
YMR199W	CLN1	YPL158C	YPL158C
YNL248C	RPA49	YNL116W	DMA2
YLR094C	GIS3	YMR214W	SCJ1
YGL025C	PGD1	YOL121C	RPS19A
YPR131C	NAT3	YPL106C	SSE1

Predictions at threshold level 0.75 continued...			
Gene1 of predicted pair		Gene2 of predicted pair	
Systematic Name	Standard Name	Systematic Name	Standard Name
YLR386W	VAC14	YOR270C	VPH1
YNL025C	SSN8	YBL075C	SSA3
YMR142C	RPL13B	YFR036W	CDC26
YGL095C	VPS45	YDL047W	SIT4
YGL087C	MMS2	YJL190C	RPS22A
YBR290W	BSD2	YDR289C	RTT103
YMR253C	YMR253C	YHR114W	BZZ1
YDR448W	ADA2	YBL003C	HTA2
YBR171W	SEC66	YPL137C	GIP3
YKL204W	EAP1	YKL213C	DOA1
YOR106W	VAM3	YGL223C	COG1
YBR200W	BEM1	YJL058C	BIT61
YJL053W	PEP8	YPL195W	APL5
YCR053W	THR4	YEL060C	PRB1
YMR142C	RPL13B	YOL081W	IRA2
YDL116W	NUP84	YNL016W	PUB1
YMR199W	CLN1	YJL136C	RPS21B
YBR127C	VMA2	YGL095C	VPS45
YOR043W	WHI2	YER122C	GLO3
YDL002C	NHP10	YCR086W	CSM1
YML115C	VAN1	YDR137W	RGP1
YOR141C	ARP8	YAR014C	BUD14
YPR018W	RLF2	YKL160W	ELF1
YPR120C	CLB5	YPL158C	YPL158C
YOR312C	RPL20B	YDR310C	SUM1
YER129W	SAK1	YMR199W	CLN1
YML117W	NAB6	YLR320W	MMS22
YDR245W	MNN10	YMR109W	MYO5
YBR200W	BEM1	YDL226C	GCS1

Predictions at threshold level 0.75 continued...			
Gene1 of predicted pair		Gene2 of predicted pair	
Systematic Name	Standard Name	Systematic Name	Standard Name
YNL288W	CAF40	YBR114W	RAD16
YKL139W	CTK1	YAL002W	VPS8
YLR360W	VPS38	YJL053W	PEP8
YMR153W	NUP53	YGL124C	MON1
YNL106C	INP52	YNL215W	IES2
YHR086W	NAM8	YPR101W	SNT309
YDR202C	RAV2	YPR036W	VMA13
YDR443C	SSN2	YOR290C	SNF2
YNL307C	MCK1	YMR167W	MLH1
YIL076W	SEC28	YGL124C	MON1
YJL124C	LSM1	YOL081W	IRA2
YPL161C	BEM4	YPL234C	TFP3
YFL031W	HAC1	YCR053W	THR4
YKR020W	VPS51	YMR038C	CCS1
YML103C	NUP188	YCR024C-A	PMP1
YOR332W	VMA4	YML115C	VAN1
YOR066W	MSA1	YJL095W	BCK1
YPR029C	APL4	YOR132W	VPS17
YMR172W	HOT1	YHR111W	UBA4
YHR060W	VMA22	YNL041C	COG6
YPL037C	EGD1	YOL111C	MDY2
YLR386W	VAC14	YDL006W	PTC1
YML102W	CAC2	YJR090C	GRR1
YMR199W	CLN1	YHR158C	KEL1
YOR132W	VPS17	YDR310C	SUM1
YMR133W	REC114	YEL060C	PRB1
YDR108W	GSG1	YPR032W	SRO7
YOR127W	RGA1	YDR363W-A	SEM1
YPL022W	RAD1	YMR199W	CLN1

Predictions at threshold level 0.75 continued...			
Gene1 of predicted pair		Gene2 of predicted pair	
Systematic Name	Standard Name	Systematic Name	Standard Name
YHR200W	RPN10	YMR276W	DSK2
YNL307C	MCK1	YOR014W	RTS1
YBR200W	BEM1	YOR001W	RRP6
YML016C	PPZ1	YNL307C	MCK1
YIL034C	CAP2	YPL050C	MNN9
YBR217W	ATG12	YNL041C	COG6
YLR191W	PEX13	YIL160C	POT1
YNL238W	KEX2	YMR038C	CCS1
YOR195W	SLK19	YKR048C	NAP1
YGL115W	SNF4	YOL108C	INO4
YNL025C	SSN8	YDR245W	MNN10
YMR127C	SAS2	YPL253C	VIK1
YNL201C	PSY2	YPR120C	CLB5
YOR132W	VPS17	YOR112W	CEX1
YOR337W	TEA1	YNL307C	MCK1
YDR260C	SWM1	YPR119W	CLB2
YHR134W	WSS1	YGR188C	BUB1
YLR119W	SRN2	YGL003C	CDH1
YJL029C	VPS53	YPR028W	YOP1
YDR137W	RGP1	YDR310C	SUM1
YNL069C	RPL16B	YGR020C	VMA7
YOR332W	VMA4	YMR070W	MOT3
YKL025C	PAN3	YPL018W	CTF19
YMR253C	YMR253C	YPR119W	CLB2
YOR023C	AHC1	YGL253W	HXK2
YHR200W	RPN10	YAR014C	BUD14
YKR014C	YPT52	YPR028W	YOP1
YLR337C	VRP1	YCR086W	CSM1
YMR224C	MRE11	YLR150W	STM1

Predictions at threshold level 0.75 continued...			
Gene1 of predicted pair		Gene2 of predicted pair	
Systematic Name	Standard Name	Systematic Name	Standard Name
YIL148W	RPL40A	YMR125W	STO1
YFR031C-A	RPL2A	YML038C	YMD8
YOR290C	SNF2	YNL041C	COG6
YMR172W	HOT1	YJL095W	BCK1
YOL126C	MDH2	YFL047W	RGD2
YDR146C	SWI5	YLR425W	TUS1
YFR010W	UBP6	YBR030W	YBR030W
YMR224C	MRE11	YLR410W	VIP1
YFL013C	IES1	YIL076W	SEC28
YJL124C	LSM1	YGL175C	SAE2
YKL032C	IXR1	YPL161C	BEM4
YER044C	ERG28	YOR043W	WHI2
YPL115C	BEM3	YJR032W	CPR7
YHL020C	OPI1	YEL051W	VMA8
YER110C	KAP123	YNL166C	BNI5
YHR160C	PEX18	YGR077C	PEX8
YBR030W	YBR030W	YBR280C	SAF1
YNL215W	IES2	YJL183W	MNN11
YBR194W	SOY1	YDR363W-A	SEM1
YLR386W	VAC14	YMR142C	RPL13B
YFR010W	UBP6	YKR030W	GMH1
YJR056C	YJR056C	YMR214W	SCJ1
YNL297C	MON2	YHR115C	DMA1
YGR104C	SRB5	YGR135W	PRE9
YHR039C-A	VMA10	YJL095W	BCK1
YDR477W	SNF1	YDR207C	UME6
YLR360W	VPS38	YNL166C	BNI5
YDL020C	RPN4	YNL107W	YAF9
YBR108W	YBR108W	YNL307C	MCK1

Predictions at threshold level 0.75 continued...			
Gene1 of predicted pair		Gene2 of predicted pair	
Systematic Name	Standard Name	Systematic Name	Standard Name
YMR216C	SKY1	YKR001C	VPS1
YDR101C	ARX1	YBR017C	KAP104
YNL236W	SIN4	YJR005W	APL1
YNL087W	TCB2	YDR358W	GGA1
YCR065W	HCM1	YML016C	PPZ1
YGL033W	HOP2	YBR173C	UMP1
YIR005W	IST3	YOR124C	UBP2
YJR084W	CSN12	YGR188C	BUB1
YPL256C	CLN2	YHL011C	PRS3
YOR064C	YNG1	YNL030W	HHF2
YHR156C	LIN1	YLR320W	MMS22
YPL024W	RMI1	YEL053C	MAK10
YDL160C	DHH1	YPL256C	CLN2
YPL115C	BEM3	YJR090C	GRR1
YNL201C	PSY2	YNL054W	VAC7
YHR111W	UBA4	YJL190C	RPS22A
YDR448W	ADA2	YDR146C	SWI5
YGR072W	UPF3	YNL107W	YAF9
YPR051W	MAK3	YGL115W	SNF4
YML095C	RAD10	YGR135W	PRE9
YPR017C	DSS4	YML038C	YMD8
YNL307C	MCK1	YMR133W	REC114
YKL126W	YPK1	YPR030W	CSR2
YHR156C	LIN1	YER162C	RAD4
YER162C	RAD4	YDL077C	VAM6
YDR477W	SNF1	YPL248C	GAL4
YBR173C	UMP1	YLR398C	SKI2
YML016C	PPZ1	YGL115W	SNF4
YHR200W	RPN10	YDR469W	SDC1

Predictions at threshold level 0.75 continued...			
Gene1 of predicted pair		Gene2 of predicted pair	
Systematic Name	Standard Name	Systematic Name	Standard Name
YDR254W	CHL4	YHR115C	DMA1
YBR057C	MUM2	YGL163C	RAD54
YDR443C	SSN2	YDR358W	GGA1
YDR004W	RAD57	YPL057C	SUR1
YLR095C	IOC2	YPL137C	GIP3
YFR010W	UBP6	YMR214W	SCJ1
YBR290W	BSD2	YER110C	KAP123
YIL076W	SEC28	YNR032W	PPG1
YKR099W	BAS1	YLR398C	SKI2
YNL291C	MID1	YGL115W	SNF4
YMR224C	MRE11	YOR014W	RTS1
YMR026C	PEX12	YBR108W	YBR108W
YOR270C	VPH1	YKL126W	YPK1
YPL057C	SUR1	YKL048C	ELM1
YLR234W	TOP3	YPL057C	SUR1
YDL005C	MED2	YLR182W	SWI6
YMR026C	PEX12	YOL044W	PEX15
YKL007W	CAP1	YAL024C	LTE1
YCL001W	RER1	YER162C	RAD4
YMR142C	RPL13B	YDR484W	VPS52
YNL291C	MID1	YOR109W	INP53
YEL060C	PRB1	YDR310C	SUM1
YOR023C	AHC1	YBL021C	HAP3
YLR315W	NKP2	YOL081W	IRA2
YLR381W	CTF3	YEL056W	HAT2
YDR156W	RPA14	YMR224C	MRE11
YGR252W	GCN5	YOL148C	SPT20
YNL307C	MCK1	YKR001C	VPS1
YDR192C	NUP42	YBR017C	KAP104

Predictions at threshold level 0.75 continued...			
Gene1 of predicted pair		Gene2 of predicted pair	
Systematic Name	Standard Name	Systematic Name	Standard Name
YDL101C	DUN1	YOL090W	MSH2
YNL288W	CAF40	YNR052C	POP2
YMR016C	SOK2	YLR320W	MMS22
YKL116C	PRR1	YER044C	ERG28
YPR051W	MAK3	YML038C	YMD8
YNL041C	COG6	YJL095W	BCK1
YOR112W	CEX1	YEL053C	MAK10
YOL030W	GAS5	YBR082C	UBC4
YDR329C	PEX3	YOL147C	PEX11
YPL024W	RMI1	YOL111C	MDY2
YHR060W	VMA22	YDL083C	RPS16B
YDR108W	GSG1	YJL036W	SNX4
YOR001W	RRP6	YCR077C	PAT1
YHR134W	WSS1	YNL016W	PUB1
YFR010W	UBP6	YOR124C	UBP2
YHL020C	OPI1	YNL051W	COG5
YBR217W	ATG12	YLR386W	VAC14
YNL116W	DMA2	YGL031C	RPL24A
YMR055C	BUB2	YCR086W	CSM1
YNL238W	KEX2	YBR036C	CSG2
YLR090W	XDJ1	YLR320W	MMS22
YIL034C	CAP2	YLR216C	CPR6
YOR368W	RAD17	YNL107W	YAF9
YBL008W	HIR1	YLR079W	SIC1
YNL032W	SIW14	YER139C	YER139C
YMR198W	CIK1	YPR119W	CLB2
YNL087W	TCB2	YJL095W	BCK1
YER115C	SPR6	YPL178W	CBC2
YIR009W	MSL1	YDR184C	ATC1

Predictions at threshold level 0.75 continued...			
Gene1 of predicted pair		Gene2 of predicted pair	
Systematic Name	Standard Name	Systematic Name	Standard Name
YFL013C	IES1	YOR124C	UBP2
YBL008W	HIR1	YNL107W	YAF9
YBL058W	SHP1	YBR036C	CSG2
YDR484W	VPS52	YOL111C	MDY2
YMR198W	CIK1	YLR058C	SHM2
YFL023W	BUD27	YPL256C	CLN2
YEL053C	MAK10	YGR188C	BUB1
YLR377C	FBP1	YNR065C	YNR065C
YOR069W	VPS5	YKR020W	VPS51
YLR079W	SIC1	YGL115W	SNF4
YDL083C	RPS16B	YFR036W	CDC26
YLR119W	SRN2	YMR142C	RPL13B
YEL065W	SIT1	YOL121C	RPS19A
YBR171W	SEC66	YPL227C	ALG5
YFR010W	UBP6	YLR371W	ROM2
YHR084W	STE12	YDL159W	STE7
YJR135C	MCM22	YLR370C	ARC18
YLR452C	SST2	YDR289C	RTT103
YHR056C	RSC30	YCL061C	MRC1
YOR127W	RGA1	YCL001W	RER1
YMR055C	BUB2	YPR119W	CLB2
YDR289C	RTT103	YML112W	CTK3
YGR188C	BUB1	YAL002W	VPS8
YGR252W	GCN5	YEL056W	HAT2
YBR200W	BEM1	YFR036W	CDC26
YFL013C	IES1	YAL002W	VPS8
YJR135C	MCM22	YJL095W	BCK1
YOL111C	MDY2	YJL183W	MNN11
YDR335W	MSN5	YCR077C	PAT1

Predictions at threshold level 0.75 continued...			
Gene1 of predicted pair		Gene2 of predicted pair	
Systematic Name	Standard Name	Systematic Name	Standard Name
YDR225W	HTA1	YDL155W	CLB3
YDR320C	SWA2	YOL108C	INO4
YPL234C	TFP3	YMR054W	STV1
YGL078C	DBP3	YJL172W	CPS1
YHR156C	LIN1	YOR327C	SNC2
YJL136C	RPS21B	YOR275C	RIM20
YOR033C	EXO1	YDR076W	RAD55
YJL154C	VPS35	YKL048C	ELM1
YNL106C	INP52	YCR088W	ABP1
YLR399C	BDF1	YCR086W	CSM1
YDR457W	TOM1	YLL039C	UBI4
YLR119W	SRN2	YKL126W	YPK1
YML115C	VAN1	YMR230W	RPS10B
YFR010W	UBP6	YGR232W	NAS6
YHR012W	VPS29	YJL183W	MNN11
YGR105W	VMA21	YJL183W	MNN11
YPL024W	RMI1	YOR337W	TEA1
YLR058C	SHM2	YDL226C	GCS1
YLR148W	PEP3	YDL161W	ENT1
YMR224C	MRE11	YDL002C	NHP10
YPL086C	ELP3	YHR187W	IKI1
YPR018W	RLF2	YLR352W	YLR352W
YEL053C	MAK10	YHR012W	VPS29
YDR245W	MNN10	YPL161C	BEM4
YPR051W	MAK3	YNL107W	YAF9
YJL183W	MNN11	YDR207C	UME6
YLR360W	VPS38	YJR084W	CSN12
YMR153W	NUP53	YBR036C	CSG2
YBL102W	SFT2	YOR112W	CEX1

Predictions at threshold level 0.75 continued...			
Gene1 of predicted pair		Gene2 of predicted pair	
Systematic Name	Standard Name	Systematic Name	Standard Name
YOR270C	VPH1	YBR036C	CSG2
YPL195W	APL5	YNL076W	MKS1
YLR095C	IOC2	YDR477W	SNF1
YCR077C	PAT1	YEL013W	VAC8
YBL058W	SHP1	YDL083C	RPS16B
YPR046W	MCM16	YLR370C	ARC18
YCL037C	SRO9	YKL213C	DOA1
YHL020C	OPI1	YML071C	COG8
YLR191W	PEX13	YKL041W	VPS24
YIL002C	INP51	YMR070W	MOT3
YOR026W	BUB3	YGL190C	CDC55
YOR023C	AHC1	YPL018W	CTF19
YCR065W	HCM1	YBR200W	BEM1
YBR030W	YBR030W	YDR137W	RGP1
YKL126W	YPK1	YER044C	ERG28
YDL192W	ARF1	YLR398C	SKI2
YBR030W	YBR030W	YLR441C	RPS1A
YIL053W	RHR2	YBL025W	RRN10
YLR452C	SST2	YBR280C	SAF1
YDR443C	SSN2	YFL028C	CAF16
YLR447C	VMA6	YGR188C	BUB1
YOR106W	VAM3	YDR310C	SUM1
YEL027W	CUP5	YPL234C	TFP3
YHR010W	RPL27A	YJL190C	RPS22A
YGR105W	VMA21	YEL051W	VMA8
YLR360W	VPS38	YOR112W	CEX1
YMR224C	MRE11	YNL225C	CNM67
YNL032W	SIW14	YNL238W	KEX2
YLR094C	GIS3	YHR039C-A	VMA10

Predictions at threshold level 0.75 continued...			
Gene1 of predicted pair		Gene2 of predicted pair	
Systematic Name	Standard Name	Systematic Name	Standard Name
YLR102C	APC9	YFR036W	CDC26
YNL106C	INP52	YKL048C	ELM1
YJL140W	RPB4	YNL030W	HHF2
YNR052C	POP2	YDR318W	MCM21
YNL199C	GCR2	YGR270W	YTA7
YBR108W	YBR108W	YLR180W	SAM1
YDL101C	DUN1	YNR052C	POP2
YPL194W	DDC1	YGL100W	SEH1
YKR046C	PET10	YMR152W	YIM1
YDR443C	SSN2	YCR053W	THR4
YLR381W	CTF3	YPR046W	MCM16
YEL056W	HAT2	YAL002W	VPS8
YDR200C	VPS64	YJR035W	RAD26
YHR200W	RPN10	YMR133W	REC114
YIL076W	SEC28	YER144C	UBP5
YLR398C	SKI2	YMR230W	RPS10B
YML013W	UBX2	YLR032W	RAD5
YOR358W	HAP5	YHR012W	VPS29
YDR129C	SAC6	YDR440W	DOT1
YOR140W	SFL1	YNL236W	SIN4
YLR095C	IOC2	YDR358W	GGA1
YAL024C	LTE1	YJL006C	CTK2
YKL139W	CTK1	YDL002C	NHP10
YIR005W	IST3	YDR318W	MCM21
YLR429W	CRN1	YOL091W	SPO21
YPR051W	MAK3	YHR039C-A	VMA10
YKL101W	HSL1	YDL047W	SIT4
YOL126C	MDH2	YMR125W	STO1
YER144C	UBP5	YGL003C	CDH1

Predictions at threshold level 0.75 continued...			
Gene1 of predicted pair		Gene2 of predicted pair	
Systematic Name	Standard Name	Systematic Name	Standard Name
YLR090W	XDJ1	YJL172W	CPS1
YLR234W	TOP3	YKL114C	APN1
YDR477W	SNF1	YDR261C	EXG2
YPL024W	RMI1	YPL018W	CTF19
YLR399C	BDF1	YPL018W	CTF19
YBR217W	ATG12	YLR370C	ARC18
YPR046W	MCM16	YDR200C	VPS64
YNL106C	INP52	YOL081W	IRA2
YPL024W	RMI1	YKR035W-A	DID2
YAL024C	LTE1	YNL215W	IES2
YPR131C	NAT3	YBR200W	BEM1
YLR191W	PEX13	YOL044W	PEX15
YPL042C	SSN3	YCR077C	PAT1
YLR095C	IOC2	YER139C	YER139C
YPL042C	SSN3	YMR198W	CIK1
YKL048C	ELM1	YDR130C	FIN1
YDL185W	TFP1	YLR452C	SST2
YIL008W	URM1	YJR135C	MCM22
YJR032W	CPR7	YER040W	GLN3
YLR452C	SST2	YER162C	RAD4
YKR046C	PET10	YKL140W	TGL1
YDR254W	CHL4	YGR270W	YTA7
YDR383C	NKP1	YOL081W	IRA2
YAR042W	SWH1	YHR195W	NVJ1
YGL163C	RAD54	YEL056W	HAT2
YJR024C	MDE1	YER110C	KAP123
YLR093C	NYV1	YDR358W	GGA1
YPR072W	NOT5	YBR009C	HHF1
YFR030W	MET10	YDR289C	RTT103

Predictions at threshold level 0.75 continued...			
Gene1 of predicted pair		Gene2 of predicted pair	
Systematic Name	Standard Name	Systematic Name	Standard Name
YBR200W	BEM1	YNL236W	SIN4
YDR245W	MNN10	YER096W	SHC1
YER110C	KAP123	YDL155W	CLB3
YGL147C	RPL9A	YJR056C	YJR056C
YOR332W	VMA4	YNL238W	KEX2
YDR484W	VPS52	YBR036C	CSG2
YNL087W	TCB2	YLR093C	NYV1
YOR290C	SNF2	YOL081W	IRA2
YDR379W	RGA2	YGL031C	RPL24A
YIL076W	SEC28	YDR245W	MNN10
YPL024W	RMI1	YGL087C	MMS2
YKL126W	YPK1	YML001W	YPT7
YDR137W	RGP1	YNR072W	HXT17
YHR082C	KSP1	YPR119W	CLB2
YHL031C	GOS1	YNL051W	COG5
YDR261C	EXG2	YER162C	RAD4
YGR019W	UGA1	YIL008W	URM1
YML095C	RAD10	YOL090W	MSH2
YBL019W	APN2	YCR066W	RAD18
YPR120C	CLB5	YOR014W	RTS1
YKL025C	PAN3	YOR358W	HAP5
YCR086W	CSM1	YPL018W	CTF19
YLR381W	CTF3	YER110C	KAP123
YLR337C	VRP1	YDL223C	HBT1
YLR386W	VAC14	YGL163C	RAD54
YDR383C	NKP1	YAR014C	BUD14
YBR019C	GAL10	YDR310C	SUM1
YLR288C	MEC3	YGL003C	CDH1
YLR006C	SSK1	YCR073C	SSK22

Predictions at threshold level 0.75 continued...			
Gene1 of predicted pair		Gene2 of predicted pair	
Systematic Name	Standard Name	Systematic Name	Standard Name
YEL065W	SIT1	YDR484W	VPS52
YBR030W	YBR030W	YFR036W	CDC26
YHL031C	GOS1	YFL048C	EMP47
YBR057C	MUM2	YDR363W-A	SEM1
YLR182W	SWI6	YGL252C	RTG2
YLR399C	BDF1	YDR227W	SIR4
YFR040W	SAP155	YDL047W	SIT4
YJR140C	HIR3	YLR435W	TSR2
YNL248C	RPA49	YDR183W	PLP1
YJL095W	BCK1	YDR207C	UME6
YKL048C	ELM1	YLR398C	SKI2
YBR173C	UMP1	YDR207C	UME6
YHR134W	WSS1	YOL090W	MSH2
YCL001W	RER1	YKR030W	GMH1
YKL116C	PRR1	YDL184C	RPL41A
YER144C	UBP5	YLR398C	SKI2
YJL154C	VPS35	YGL054C	ERV14
YOL111C	MDY2	YOL081W	IRA2
YKL139W	CTK1	YNL253W	TEX1
YEL009C	GCN4	YDR176W	NGG1
YEL053C	MAK10	YMR311C	GLC8
YGR104C	SRB5	YGR252W	GCN5
YDL155W	CLB3	YKL041W	VPS24
YBL103C	RTG3	YDL077C	VAM6
YNL021W	HDA1	YPR119W	CLB2
YMR026C	PEX12	YDR137W	RGP1
YDR137W	RGP1	YNL238W	KEX2
YLR360W	VPS38	YLR425W	TUS1
YPL037C	EGD1	YPL120W	VPS30

Predictions at threshold level 0.75 continued...			
Gene1 of predicted pair		Gene2 of predicted pair	
Systematic Name	Standard Name	Systematic Name	Standard Name
YDR080W	VPS41	YAL002W	VPS8
YMR284W	YKU70	YLR410W	VIP1
YCR081W	SRB8	YJL095W	BCK1
YGL174W	BUD13	YCR077C	PAT1
YPL180W	TCO89	YJL183W	MNN11
YBR171W	SEC66	YKR014C	YPT52
YGL223C	COG1	YML071C	COG8
YLR441C	RPS1A	YLR320W	MMS22
YJR005W	APL1	YDR200C	VPS64
YLR447C	VMA6	YPL018W	CTF19
YOR035C	SHE4	YMR230W	RPS10B
YHL031C	GOS1	YJR040W	GEF1
YDR245W	MNN10	YOR375C	GDH1
YHR156C	LIN1	YHR039C-A	VMA10
YHL031C	GOS1	YDR310C	SUM1
YFL023W	BUD27	YML071C	COG8
YNL054W	VAC7	YBR107C	IML3
YJR056C	YJR056C	YML071C	COG8
YEL065W	SIT1	YDR137W	RGP1
YHR114W	BZZ1	YAR014C	BUD14
YLR435W	TSR2	YMR167W	MLH1
YGL115W	SNF4	YLR398C	SKI2
YKR099W	BAS1	YBR019C	GAL10
YDR378C	LSM6	YGL252C	RTG2
YKL160W	ELF1	YAL002W	VPS8
YMR224C	MRE11	YLR315W	NKP2
YCR077C	PAT1	YKL041W	VPS24
YDL116W	NUP84	YIL036W	CST6
YBR200W	BEM1	YPR120C	CLB5

Predictions at threshold level 0.75 continued...			
Gene1 of predicted pair		Gene2 of predicted pair	
Systematic Name	Standard Name	Systematic Name	Standard Name
YDR382W	RPP2B	YKR026C	GCN3
YDR202C	RAV2	YGL212W	VAM7
YLR119W	SRN2	YOR337W	TEA1
YKR020W	VPS51	YKL041W	VPS24
YER139C	YER139C	YMR311C	GLC8
YKL032C	IXR1	YHR115C	DMA1
YGR252W	GCN5	YBL002W	HTB2
YOR043W	WHI2	YDR358W	GGA1
YNL069C	RPL16B	YCR066W	RAD18
YDL020C	RPN4	YLR386W	VAC14
YMR224C	MRE11	YKR072C	SIS2
YBR171W	SEC66	YBR107C	IML3
YML016C	PPZ1	YOR275C	RIM20
YKL126W	YPK1	YOR066W	MSA1
YKR072C	SIS2	YGL163C	RAD54
YPR007C	REC8	YPR119W	CLB2
YBR228W	SLX1	YHR077C	NMD2
YER162C	RAD4	YBR036C	CSG2
YGL135W	RPL1B	YLR018C	POM34
YBR126C	TPS1	YER139C	YER139C
YML016C	PPZ1	YMR055C	BUB2
YML088W	UFO1	YJL204C	RCY1
YJL004C	SYS1	YNR032W	PPG1
YAL024C	LTE1	YGL151W	NUT1
YJL124C	LSM1	YKL213C	DOA1
YDR101C	ARX1	YKR048C	NAP1
YMR116C	ASC1	YGL200C	EMP24
YML071C	COG8	YDL192W	ARF1
YER116C	SLX8	YML013W	UBX2

Predictions at threshold level 0.75 continued...			
Gene1 of predicted pair		Gene2 of predicted pair	
Systematic Name	Standard Name	Systematic Name	Standard Name
YOR106W	VAM3	YER120W	SCS2
YGL115W	SNF4	YHR039C-A	VMA10
YLR192C	HCR1	YDL223C	HBT1
YMR142C	RPL13B	YJL183W	MNN11
YPL256C	CLN2	YOL081W	IRA2
YMR142C	RPL13B	YNL076W	MKS1
YKL116C	PRR1	YJL095W	BCK1
YMR202W	ERG2	YER044C	ERG28
YMR116C	ASC1	YMR038C	CCS1
YGL178W	MPT5	YDR485C	VPS72
YLR094C	GIS3	YDL149W	ATG9
YHR161C	YAP1801	YLR396C	VPS33
YIL034C	CAP2	YKL204W	EAP1
YHL020C	OPI1	YER162C	RAD4
YDR174W	HMO1	YDR289C	RTT103
YLR018C	POM34	YMR273C	ZDS1
YDR484W	VPS52	YJL204C	RCY1
YMR198W	CIK1	YJR035W	RAD26
YDR080W	VPS41	YPL037C	EGD1
YPL024W	RMI1	YDR261C	EXG2
YBL058W	SHP1	YOL121C	RPS19A
YGR078C	PAC10	YHR056C	RSC30
YPR036W	VMA13	YMR214W	SCJ1
YMR153W	NUP53	YBR108W	YBR108W
YJR040W	GEF1	YER122C	GLO3
YNL087W	TCB2	YOL111C	MDY2
YPR017C	DSS4	YGR072W	UPF3
YBR126C	TPS1	YBR030W	YBR030W
YDL155W	CLB3	YDR440W	DOT1

Predictions at threshold level 0.75 continued...			
Gene1 of predicted pair		Gene2 of predicted pair	
Systematic Name	Standard Name	Systematic Name	Standard Name
YBR107C	IML3	YDR171W	HSP42
YLR337C	VRP1	YOR127W	RGA1
YMR016C	SOK2	YJR084W	CSN12
YJL154C	VPS35	YGL253W	HXK2
YPR046W	MCM16	YJL183W	MNN11
YCR020C-A	MAK31	YAL002W	VPS8
YPL253C	VIK1	YLR254C	NDL1
YNL288W	CAF40	YPL042C	SSN3
YDR382W	RPP2B	YPL220W	RPL1A
YJR135C	MCM22	YAR014C	BUD14
YOR001W	RRP6	YDR137W	RGP1
YDR129C	SAC6	YAL002W	VPS8
YDL116W	NUP84	YCR024C-A	PMP1
YMR142C	RPL13B	YER040W	GLN3
YJL176C	SWI3	YJR084W	CSN12
YJL060W	BNA3	YDL226C	GCS1
YMR055C	BUB2	YFR036W	CDC26
YER120W	SCS2	YLR309C	IMH1
YBR260C	RGD1	YNL090W	RHO2
YBR215W	HPC2	YCL037C	SRO9
YJL029C	VPS53	YDL226C	GCS1
YGR020C	VMA7	YDR310C	SUM1
YBL102W	SFT2	YHR039C-A	VMA10
YBL078C	ATG8	YNL223W	ATG4
YNL288W	CAF40	YAL002W	VPS8
YLR254C	NDL1	YPL038W	MET31
YEL050C	RML2	YPL106C	SSE1
YBR126C	TPS1	YMR311C	GLC8
YDR245W	MNN10	YFL048C	EMP47

Predictions at threshold level 0.75 continued...			
Gene1 of predicted pair		Gene2 of predicted pair	
Systematic Name	Standard Name	Systematic Name	Standard Name
YJL154C	VPS35	YPL253C	VIK1
YDR293C	SSD1	YNL166C	BNI5
YNL238W	KEX2	YGL003C	CDH1
YLR360W	VPS38	YGL151W	NUT1
YIL159W	BNR1	YDR310C	SUM1
YOL145C	CTR9	YDR440W	DOT1
YJL172W	CPS1	YER162C	RAD4
YDR004W	RAD57	YMR198W	CIK1
YJL154C	VPS35	YNL297C	MON2
YNL031C	HHT2	YDR227W	SIR4
YOR195W	SLK19	YNL307C	MCK1
YPR030W	CSR2	YER139C	YER139C
YKL139W	CTK1	YJR140C	HIR3
YBR171W	SEC66	YML071C	COG8
YPR052C	NHP6A	YBR215W	HPC2
YPL057C	SUR1	YLR371W	ROM2
YER162C	RAD4	YMR152W	YIM1
YLR385C	SWC7	YLR399C	BDF1
YNL215W	IES2	YBR036C	CSG2
YHR060W	VMA22	YKL041W	VPS24
YOR076C	SKI7	YPR189W	SKI3
YIR005W	IST3	YNL116W	DMA2
YKL048C	ELM1	YGL115W	SNF4
YBR107C	IML3	YEL053C	MAK10
YDL160C	DHH1	YJR090C	GRR1
YKR061W	KTR2	YDR335W	MSN5
YJL172W	CPS1	YLR180W	SAM1
YBR019C	GAL10	YLR370C	ARC18
YNL079C	TPM1	YLR371W	ROM2

Predictions at threshold level 0.75 continued...			
Gene1 of predicted pair		Gene2 of predicted pair	
Systematic Name	Standard Name	Systematic Name	Standard Name
YBR200W	BEM1	YCR086W	CSM1
YEL065W	SIT1	YOL081W	IRA2
YDR484W	VPS52	YKL048C	ELM1
YCR020C-A	MAK31	YDL006W	PTC1
YGL133W	ITC1	YGR135W	PRE9
YOR375C	GDH1	YDR440W	DOT1
YKL160W	ELF1	YLR182W	SWI6
YLR337C	VRP1	YOL067C	RTG1
YPR051W	MAK3	YHL002W	HSE1
YDR289C	RTT103	YLR350W	ORM2
YCR092C	MSH3	YOL090W	MSH2
YKL006W	RPL14A	YGR135W	PRE9
YGL212W	VAM7	YKR048C	NAP1
YKL139W	CTK1	YOR270C	VPH1
YJR060W	CBF1	YLR309C	IMH1
YMR055C	BUB2	YDR260C	SWM1
YOR275C	RIM20	YBL087C	RPL23A
YBR126C	TPS1	YMR224C	MRE11
YGL003C	CDH1	YDL047W	SIT4
YPL105C	YPL105C	YPL120W	VPS30
YLR234W	TOP3	YBR057C	MUM2
YNL243W	SLA2	YLR337C	VRP1
YML115C	VAN1	YOR275C	RIM20
YDR363W-A	SEM1	YLR421C	RPN13
YDR358W	GGA1	YMR311C	GLC8
YDR507C	GIN4	YHR061C	GIC1
YKL025C	PAN3	YGL253W	HXK2
YPR018W	RLF2	YDL059C	RAD59
YOR290C	SNF2	YKL160W	ELF1

Predictions at threshold level 0.75 continued...			
Gene1 of predicted pair		Gene2 of predicted pair	
Systematic Name	Standard Name	Systematic Name	Standard Name
YMR273C	ZDS1	YKR024C	DBP7
YMR004W	MVP1	YNL107W	YAF9
YDR477W	SNF1	YOL081W	IRA2
YMR080C	NAM7	YHR134W	WSS1
YLR452C	SST2	YJR084W	CSN12
YML013W	UBX2	YMR055C	BUB2
YOR358W	HAP5	YBL079W	NUP170
YNL199C	GCR2	YNL107W	YAF9
YPL161C	BEM4	YHR061C	GIC1
YKL139W	CTK1	YDR254W	CHL4
YOL111C	MDY2	YER040W	GLN3
YDR320C	SWA2	YDR310C	SUM1
YPL022W	RAD1	YNL016W	PUB1
YJL176C	SWI3	YGL153W	PEX14
YBR171W	SEC66	YHR111W	UBA4
YDR320C	SWA2	YMR004W	MVP1
YFR010W	UBP6	YPL195W	APL5
YML001W	YPT7	YLR093C	NYV1
YKL116C	PRR1	YPR030W	CSR2
YGL078C	DBP3	YMR153W	NUP53
YKR099W	BAS1	YHR039C-A	VMA10
YBR073W	RDH54	YPL018W	CTF19
YKR001C	VPS1	YOR312C	RPL20B
YHR060W	VMA22	YPR046W	MCM16
YKL006W	RPL14A	YPL161C	BEM4
YOR127W	RGA1	YHR060W	VMA22
YGL174W	BUD13	YOR043W	WHI2
YPL259C	APM1	YOR327C	SNC2
YHR098C	SFB3	YPR135W	CTF4

Predictions at threshold level 0.75 continued...			
Gene1 of predicted pair		Gene2 of predicted pair	
Systematic Name	Standard Name	Systematic Name	Standard Name
YNL058C	YNL058C	YHR081W	LRP1
YGL038C	OCH1	YMR052W	FAR3
YPL086C	ELP3	YBR111W-A	SUS1
YDL020C	RPN4	YHR039C-A	VMA10
YPL240C	HSP82	YDL246C	SOR2
YMR202W	ERG2	YJL187C	SWE1
YIL034C	CAP2	YPR046W	MCM16
YGL078C	DBP3	YMR016C	SOK2
YMR038C	CCS1	YER122C	GLO3
YKL139W	CTK1	YMR038C	CCS1
YNL199C	GCR2	YDR289C	RTT103
YLR429W	CRN1	YOR141C	ARP8
YEL044W	IES6	YOR035C	SHE4
YIL103W	DPH1	YOL081W	IRA2
YEL005C	VAB2	YHR195W	NVJ1
YDL113C	ATG20	YDR142C	PEX7
YKR099W	BAS1	YMR152W	YIM1
YHL031C	GOS1	YGL212W	VAM7
YOR106W	VAM3	YKR020W	VPS51
YLR083C	EMP70	YNL107W	YAF9
YDR202C	RAV2	YHR060W	VMA22
YOR023C	AHC1	YHR111W	UBA4
YLR360W	VPS38	YCR086W	CSM1
YJR060W	CBF1	YEL051W	VMA8
YPL119C	DBP1	YPR119W	CLB2
YLR423C	ATG17	YNL090W	RHO2
YIL133C	RPL16A	YGL252C	RTG2
YMR198W	CIK1	YPL018W	CTF19
YGR252W	GCN5	YGL252C	RTG2

Predictions at threshold level 0.75 continued...			
Gene1 of predicted pair		Gene2 of predicted pair	
Systematic Name	Standard Name	Systematic Name	Standard Name
YHR200W	RPN10	YML097C	VPS9
YPL024W	RMI1	YDL192W	ARF1
YMR142C	RPL13B	YPL137C	GIP3
YGL174W	BUD13	YDL002C	NHP10
YIL034C	CAP2	YOL067C	RTG1
YOR109W	INP53	YPL105C	YPL105C
YDR507C	GIN4	YJL187C	SWE1
YML016C	PPZ1	YLR425W	TUS1
YOL145C	CTR9	YJL140W	RPB4
YJL154C	VPS35	YDR080W	VPS41
YBR200W	BEM1	YKL080W	VMA5
YBR017C	KAP104	YGL100W	SEH1
YDR103W	STE5	YOR212W	STE4
YDR363W-A	SEM1	YGL163C	RAD54
YPL037C	EGD1	YNL041C	COG6
YPL024W	RMI1	YFL023W	BUD27
YMR152W	YIM1	YJR084W	CSN12
YLR094C	GIS3	YPL195W	APL5
YIL008W	URM1	YGL253W	HXK2
YJR043C	POL32	YGL100W	SEH1
YDR137W	RGP1	YGL025C	PGD1
YLR386W	VAC14	YBL087C	RPL23A
YMR055C	BUB2	YGR054W	YGR054W
YJL117W	PHO86	YLR371W	ROM2
YLR083C	EMP70	YGR261C	APL6
YBR200W	BEM1	YDL047W	SIT4
YDR192C	NUP42	YGR081C	SLX9
YIL034C	CAP2	YOR132W	VPS17
YFR032C-A	RPL29	YKL048C	ELM1

Predictions at threshold level 0.75 continued...			
Gene1 of predicted pair		Gene2 of predicted pair	
Systematic Name	Standard Name	Systematic Name	Standard Name
YLR371W	ROM2	YLR093C	NYV1
YBR084C-A	RPL19A	YHR152W	SPO12
YBR030W	YBR030W	YLR398C	SKI2
YGR249W	MGA1	YGL003C	CDH1
YGL066W	SGF73	YGR135W	PRE9
YER107C	GLE2	YML041C	VPS71
YLR119W	SRN2	YLR371W	ROM2
YMR224C	MRE11	YLR032W	RAD5
YJL124C	LSM1	YFR036W	CDC26
YNR072W	HXT17	YFR036W	CDC26
YNL068C	FKH2	YKR057W	RPS21A
YPR141C	KAR3	YGL190C	CDC55
YCR081W	SRB8	YLR425W	TUS1
YLR360W	VPS38	YJL029C	VPS53
YGR217W	CCH1	YMR205C	PFK2
YBR031W	RPL4A	YER102W	RPS8B
YHR156C	LIN1	YNL058C	YNL058C
YLR234W	TOP3	YHR013C	ARD1
YLR410W	VIP1	YHR108W	GGA2
YLR240W	VPS34	YJL029C	VPS53
YBR171W	SEC66	YDL056W	MBP1
YAL024C	LTE1	YHL022C	SPO11
YDR335W	MSN5	YIL103W	DPH1
YNL069C	RPL16B	YER162C	RAD4
YBL058W	SHP1	YNL058C	YNL058C
YNL325C	FIG4	YOR327C	SNC2
YDR383C	NKP1	YLR381W	CTF3
YLR079W	SIC1	YDR378C	LSM6
YHR060W	VMA22	YGL115W	SNF4

Predictions at threshold level 0.75 continued...			
Gene1 of predicted pair		Gene2 of predicted pair	
Systematic Name	Standard Name	Systematic Name	Standard Name
YPL042C	SSN3	YBR215W	HPC2
YER116C	SLX8	YEL060C	PRB1
YJL187C	SWE1	YML028W	TSA1
YDL065C	PEX19	YGL205W	POX1
YNL307C	MCK1	YGL124C	MON1
YDR261C	EXG2	YBR280C	SAF1
YPL152W	RRD2	YDR318W	MCM21
YGR217W	CCH1	YPL089C	RLM1
YBR107C	IML3	YDR155C	CPR1
YLR386W	VAC14	YHR013C	ARD1
YLR090W	XDJ1	YMR214W	SCJ1
YER110C	KAP123	YGR059W	SPR3
YMR025W	CSI1	YJR084W	CSN12
YLR313C	SPH1	YLR410W	VIP1
YKR001C	VPS1	YER162C	RAD4
YDR477W	SNF1	YPR046W	MCM16
YDR176W	NGG1	YBR089C-A	NHP6B
YPL037C	EGD1	YFR036W	CDC26
YLR337C	VRP1	YDR183W	PLP1
YDR245W	MNN10	YCR077C	PAT1
YPL213W	LEA1	YCR077C	PAT1
YHL031C	GOS1	YMR004W	MVP1
YNR072W	HXT17	YNL238W	KEX2
YDR477W	SNF1	YMR153W	NUP53
YHR012W	VPS29	YAR014C	BUD14
YIL076W	SEC28	YMR004W	MVP1
YBL071W-A	KTI11	YPL101W	ELP4
YGR270W	YTA7	YGL252C	RTG2
YMR055C	BUB2	YHR013C	ARD1

Predictions at threshold level 0.75 continued...			
Gene1 of predicted pair		Gene2 of predicted pair	
Systematic Name	Standard Name	Systematic Name	Standard Name
YGR135W	PRE9	YPL256C	CLN2
YMR026C	PEX12	YIL076W	SEC28
YOL051W	GAL11	YLR357W	RSC2
YGR019W	UGA1	YBL051C	PIN4
YLR410W	VIP1	YAR014C	BUD14
YLR399C	BDF1	YFR034C	PHO4
YBL047C	EDE1	YKL048C	ELM1
YDR382W	RPP2B	YNL147W	LSM7
YML115C	VAN1	YMR199W	CLN1
YIR003W	YIR003W	YPL050C	MNN9
YHR081W	LRP1	YER040W	GLN3
YDL226C	GCS1	YOR036W	PEP12
YFR031C-A	RPL2A	YNL107W	YAF9
YKL068W	NUP100	YJR090C	GRR1
YJR090C	GRR1	YNL107W	YAF9
YDR254W	CHL4	YJR035W	RAD26
YOR039W	CKB2	YJL187C	SWE1
YPR029C	APL4	YKR001C	VPS1
YER116C	SLX8	YOR066W	MSA1
YBR107C	IML3	YDR310C	SUM1
YPR036W	VMA13	YNL076W	MKS1
YIL053W	RHR2	YGR249W	MGA1
YGL078C	DBP3	YDR207C	UME6
YMR116C	ASC1	YKL139W	CTK1
YDR156W	RPA14	YHR200W	RPN10
YDR137W	RGP1	YOL018C	TLG2
YOR106W	VAM3	YML071C	COG8
YMR109W	MYO5	YDR289C	RTT103
YAL024C	LTE1	YDL083C	RPS16B

Predictions at threshold level 0.75 continued...			
Gene1 of predicted pair		Gene2 of predicted pair	
Systematic Name	Standard Name	Systematic Name	Standard Name
YNL032W	SIW14	YOR270C	VPH1
YBR194W	SOY1	YER122C	GLO3
YOR230W	WTM1	YOR229W	WTM2
YPR046W	MCM16	YNL076W	MKS1
YBL102W	SFT2	YMR004W	MVP1
YGR173W	RBG2	YDR152W	GIR2
YLR381W	CTF3	YGR054W	YGR054W
YNL238W	KEX2	YMR311C	GLC8
YNL069C	RPL16B	YCL001W	RER1
YDR245W	MNN10	YOR124C	UBP2
YLR288C	MEC3	YHR010W	RPL27A
YHR200W	RPN10	YEL060C	PRB1
YJL172W	CPS1	YCR053W	THR4
YML115C	VAN1	YMR153W	NUP53
YLL007C	YLL007C	YGL253W	HXK2
YPL022W	RAD1	YPL256C	CLN2
YCL001W	RER1	YPL234C	TFP3
YIL053W	RHR2	YBR126C	TPS1
YML117W	NAB6	YGR135W	PRE9
YKR099W	BAS1	YLR094C	GIS3
YNL079C	TPM1	YDR440W	DOT1
YJL172W	CPS1	YFL023W	BUD27
YDR137W	RGP1	YLR371W	ROM2
YGL212W	VAM7	YDR207C	UME6
YBR290W	BSD2	YCR086W	CSM1
YOR109W	INP53	YNL238W	KEX2
YLL039C	UBI4	YBL072C	RPS8A
YBL058W	SHP1	YLR386W	VAC14
YBR274W	CHK1	YKL114C	APN1

Predictions at threshold level 0.75 continued...			
Gene1 of predicted pair		Gene2 of predicted pair	
Systematic Name	Standard Name	Systematic Name	Standard Name
YJR035W	RAD26	YKL054C	DEF1
YGL212W	VAM7	YGL095C	VPS45
YPL213W	LEA1	YLR425W	TUS1
YNL041C	COG6	YDR358W	GGA1
YMR026C	PEX12	YGL124C	MON1
YEL050C	RML2	YJR135C	MCM22
YKL139W	CTK1	YDL077C	VAM6
YPR120C	CLB5	YCR077C	PAT1
YKL139W	CTK1	YGL252C	RTG2
YLR090W	XDJ1	YER040W	GLN3
YEL053C	MAK10	YBR019C	GAL10
YGR072W	UPF3	YDL006W	PTC1
YER116C	SLX8	YEL053C	MAK10
YBL103C	RTG3	YDL192W	ARF1
YBL008W	HIR1	YLR350W	ORM2
YEL013W	VAC8	YPR185W	ATG13
YLR320W	MMS22	YDL006W	PTC1
YLR386W	VAC14	YPL253C	VIK1
YNL058C	YNL058C	YDL077C	VAM6
YDR477W	SNF1	YBL103C	RTG3
YHR039C-A	VMA10	YOR066W	MSA1
YHR167W	THP2	YLR350W	ORM2
YNL215W	IES2	YFR030W	MET10
YOL044W	PEX15	YNL214W	PEX17
YBR200W	BEM1	YML112W	CTK3
YGL163C	RAD54	YIR005W	IST3
YFR010W	UBP6	YKL139W	CTK1
YDR477W	SNF1	YMR152W	YIM1
YBR108W	YBR108W	YBL103C	RTG3

Predictions at threshold level 0.75 continued...			
Gene1 of predicted pair		Gene2 of predicted pair	
Systematic Name	Standard Name	Systematic Name	Standard Name
YPR036W	VMA13	YER005W	YND1
YDR293C	SSD1	YEL056W	HAT2
YGR270W	YTA7	YOL054W	PSH1
YCR066W	RAD18	YHL020C	OPI1
YMR109W	MYO5	YDR155C	CPR1
YPL057C	SUR1	YNL076W	MKS1
YOR080W	DIA2	YPR119W	CLB2
YMR142C	RPL13B	YOR270C	VPH1
YBR215W	HPC2	YOL081W	IRA2
YBL021C	HAP3	YDR310C	SUM1
YMR199W	CLN1	YDR379W	RGA2
YGR081C	SLX9	YBR215W	HPC2
YBR030W	YBR030W	YNL238W	KEX2
YGL025C	PGD1	YML001W	YPT7
YML016C	PPZ1	YGL003C	CDH1
YLR447C	VMA6	YAL002W	VPS8
YOR106W	VAM3	YDR469W	SDC1
YBR215W	HPC2	YDR171W	HSP42
YAL024C	LTE1	YMR198W	CIK1
YJL004C	SYS1	YJR033C	RAV1
YCR088W	ABP1	YKL213C	DOA1
YDR484W	VPS52	YJL029C	VPS53
YNL206C	RTT106	YBR107C	IML3
YOR096W	RPS7A	YLR441C	RPS1A
YPL050C	MNN9	YPL112C	PEX25
YLL007C	YLL007C	YHR111W	UBA4
YNL025C	SSN8	YKL139W	CTK1
YKR101W	SIR1	YBR010W	HHT1
YDR129C	SAC6	YJR084W	CSN12

Predictions at threshold level 0.75 continued...			
Gene1 of predicted pair		Gene2 of predicted pair	
Systematic Name	Standard Name	Systematic Name	Standard Name
YNL069C	RPL16B	YLR309C	IMH1
YJL154C	VPS35	YOR014W	RTS1
YML016C	PPZ1	YDR183W	PLP1
YOR337W	TEA1	YPL037C	EGD1
YFL047W	RGD2	YGL016W	KAP122
YFR036W	CDC26	YOR368W	RAD17
YHR013C	ARD1	YDR289C	RTT103
YNL058C	YNL058C	YPR046W	MCM16
YBR057C	MUM2	YGR135W	PRE9
YPL037C	EGD1	YHR012W	VPS29
YHR134W	WSS1	YOR023C	AHC1
YLR083C	EMP70	YHR060W	VMA22
YDR363W-A	SEM1	YPL161C	BEM4
YNL297C	MON2	YHL020C	OPI1
YDR156W	RPA14	YPL018W	CTF19
YPL057C	SUR1	YEL053C	MAK10
YML038C	YMD8	YER120W	SCS2
YLR109W	AHP1	YOL151W	GRE2
YGR270W	YTA7	YDR310C	SUM1
YLR447C	VMA6	YOL067C	RTG1
YDR225W	HTA1	YLR234W	TOP3
YGL237C	HAP2	YNL107W	YAF9
YKR099W	BAS1	YMR214W	SCJ1
YOR083W	WHI5	YBR194W	SOY1
YGR261C	APL6	YDL006W	PTC1
YEL051W	VMA8	YNL107W	YAF9
YBR084C-A	RPL19A	YHR111W	UBA4
YHR200W	RPN10	YDL047W	SIT4
YER144C	UBP5	YDL077C	VAM6

Predictions at threshold level 0.75 continued...			
Gene1 of predicted pair		Gene2 of predicted pair	
Systematic Name	Standard Name	Systematic Name	Standard Name
YGR159C	NSR1	YLR410W	VIP1
YFR010W	UBP6	YDR484W	VPS52
YCL037C	SRO9	YML038C	YMD8
YML016C	PPZ1	YPR120C	CLB5
YPL042C	SSN3	YKL160W	ELF1
YGR108W	CLB1	YDR289C	RTT103
YLR148W	PEP3	YMR231W	PEP5
YNL041C	COG6	YER040W	GLN3
YCL010C	SGF29	YDR216W	ADR1
YMR224C	MRE11	YLR182W	SWI6
YNL127W	FAR11	YAL002W	VPS8
YCR086W	CSM1	YDR183W	PLP1
YJL053W	PEP8	YHR161C	YAP1801
YNL032W	SIW14	YLR180W	SAM1
YGR020C	VMA7	YER122C	GLO3
YPR030W	CSR2	YCR053W	THR4
YNL106C	INP52	YHR111W	UBA4
YLR447C	VMA6	YKL080W	VMA5
YOR132W	VPS17	YNL127W	FAR11
YOR337W	TEA1	YKL126W	YPK1
YDR378C	LSM6	YOL091W	SPO21
YHR111W	UBA4	YBL087C	RPL23A
YMR224C	MRE11	YPL195W	APL5
YGL078C	DBP3	YLR452C	SST2
YNL076W	MKS1	YDR310C	SUM1
YNL032W	SIW14	YNL041C	COG6
YIR025W	MND2	YFR036W	CDC26
YAL024C	LTE1	YBR278W	DPB3
YHR156C	LIN1	YER116C	SLX8

Predictions at threshold level 0.75 continued...			
Gene1 of predicted pair		Gene2 of predicted pair	
Systematic Name	Standard Name	Systematic Name	Standard Name
YDL006W	PTC1	YPL089C	RLM1
YOR195W	SLK19	YOR127W	RGA1
YGL006W	PMC1	YBL078C	ATG8
YNL041C	COG6	YEL036C	ANP1
YDR176W	NGG1	YCL010C	SGF29
YIL159W	BNR1	YLR425W	TUS1
YGL250W	RMR1	YKL101W	HSL1
YKR095W	MLP1	YBL079W	NUP170
YNL325C	FIG4	YKL126W	YPK1
YNL058C	YNL058C	YNL238W	KEX2
YER144C	UBP5	YPL161C	BEM4
YDR310C	SUM1	YOR014W	RTS1
YDR004W	RAD57	YKR072C	SIS2
YDR484W	VPS52	YNL058C	YNL058C
YMR199W	CLN1	YDR076W	RAD55
YGL178W	MPT5	YPL101W	ELP4
YLR396C	VPS33	YAL002W	VPS8
YDR484W	VPS52	YLR370C	ARC18
YNL291C	MID1	YMR038C	CCS1
YGL066W	SGF73	YPL248C	GAL4
YNL117W	MLS1	YLR032W	RAD5
YGR108W	CLB1	YBR200W	BEM1
YER116C	SLX8	YLR370C	ARC18
YLR170C	APS1	YOR132W	VPS17
YBR194W	SOY1	YOR124C	UBP2
YLR119W	SRN2	YDL083C	RPS16B
YMR026C	PEX12	YKL048C	ELM1
YMR216C	SKY1	YCR066W	RAD18
YDL155W	CLB3	YGL253W	HXK2

Predictions at threshold level 0.75 continued...			
Gene1 of predicted pair		Gene2 of predicted pair	
Systematic Name	Standard Name	Systematic Name	Standard Name
YPR017C	DSS4	YOR112W	CEX1
YMR284W	YKU70	YLR135W	SLX4
YML038C	YMD8	YML001W	YPT7
YGL100W	SEH1	YKR082W	NUP133
YPL256C	CLN2	YLR425W	TUS1
YHR157W	REC104	YER044C-A	MEI4
YBR290W	BSD2	YOR358W	HAP5
YLR043C	TRX1	YDR080W	VPS41
YOR270C	VPH1	YHR200W	RPN10
YPL037C	EGD1	YEL060C	PRB1
YNR052C	POP2	YBR089C-A	NHP6B
YCR086W	CSM1	YHR114W	BZZ1
YPL018W	CTF19	YGL151W	NUT1
YJL154C	VPS35	YER122C	GLO3
YHR205W	SCH9	YPL253C	VIK1
YGR270W	YTA7	YGR054W	YGR054W
YNL106C	INP52	YOR195W	SLK19
YPL253C	VIK1	YHR200W	RPN10
YDR146C	SWI5	YBL047C	EDE1
YDL083C	RPS16B	YML071C	COG8
YBR200W	BEM1	YJR035W	RAD26
YFR031C-A	RPL2A	YMR230W	RPS10B
YDL239C	ADY3	YNL225C	CNM67
YBR200W	BEM1	YER040W	GLN3
YMR224C	MRE11	YMR142C	RPL13B
YBR278W	DPB3	YOR043W	WHI2
YJL029C	VPS53	YER143W	DDI1
YGL066W	SGF73	YKL160W	ELF1
YML115C	VAN1	YLR398C	SKI2

Predictions at threshold level 0.75 continued...			
Gene1 of predicted pair		Gene2 of predicted pair	
Systematic Name	Standard Name	Systematic Name	Standard Name
YGR089W	NNF2	YHL010C	YHL010C
YNL264C	PDR17	YLR398C	SKI2
YHR156C	LIN1	YGL025C	PGD1
YGL025C	PGD1	YLR398C	SKI2
YHR073W	OSH3	YOL126C	MDH2
YBR108W	YBR108W	YLR452C	SST2
YIL159W	BNR1	YPL161C	BEM4
YLR095C	IOC2	YLR119W	SRN2
YML062C	MFT1	YNL004W	HRB1
YHR200W	RPN10	YPL256C	CLN2
YGR261C	APL6	YGL115W	SNF4
YML095C	RAD10	YGL163C	RAD54
YOR014W	RTS1	YLR425W	TUS1
YOR270C	VPH1	YNL041C	COG6
YML016C	PPZ1	YFR030W	MET10
YOR035C	SHE4	YML097C	VPS9
YDR443C	SSN2	YLR192C	HCR1
YBR215W	HPC2	YBR107C	IML3
YDL127W	PCL2	YEL056W	HAT2
YNL297C	MON2	YAR014C	BUD14
YBR215W	HPC2	YDR378C	LSM6
YGL066W	SGF73	YPR120C	CLB5
YPR046W	MCM16	YOL091W	SPO21
YDL160C	DHH1	YMR125W	STO1
YAL024C	LTE1	YCR086W	CSM1
YOR212W	STE4	YCL032W	STE50
YER144C	UBP5	YDL192W	ARF1
YIL076W	SEC28	YAL002W	VPS8
YLR447C	VMA6	YKL041W	VPS24

Predictions at threshold level 0.75 continued...			
Gene1 of predicted pair		Gene2 of predicted pair	
Systematic Name	Standard Name	Systematic Name	Standard Name
YER107C	GLE2	YBR231C	SWC5
YNL068C	FKH2	YLR386W	VAC14
YKL110C	KTI12	YLR254C	NDL1
YGR020C	VMA7	YHL020C	OPI1
YNL218W	MGS1	YLR234W	TOP3
YDR129C	SAC6	YHR013C	ARD1
YDR484W	VPS52	YDR137W	RGP1
YOR043W	WHI2	YLR425W	TUS1
YML117W	NAB6	YJL095W	BCK1
YHR060W	VMA22	YHR039C-A	VMA10
YGR104C	SRB5	YER161C	SPT2
YPL096C-A	ERI1	YJL148W	RPA34
YLR399C	BDF1	YHR013C	ARD1
YBR200W	BEM1	YNL225C	CNM67
YDR484W	VPS52	YMR152W	YIM1
YKR020W	VPS51	YDL006W	PTC1
YDR146C	SWI5	YDL006W	PTC1
YAL024C	LTE1	YBL103C	RTG3
YGR249W	MGA1	YBL103C	RTG3
YBR108W	YBR108W	YOR270C	VPH1
YLR381W	CTF3	YPR119W	CLB2
YJR024C	MDE1	YMR153W	NUP53
YDR318W	MCM21	YJR035W	RAD26
YLR119W	SRN2	YLR398C	SKI2
YCR081W	SRB8	YDR146C	SWI5
YMR172W	HOT1	YIL008W	URM1
YPL042C	SSN3	YNR052C	POP2
YNL058C	YNL058C	YDL192W	ARF1
YGR241C	YAP1802	YFL026W	STE2

Predictions at threshold level 0.75 continued...			
Gene1 of predicted pair		Gene2 of predicted pair	
Systematic Name	Standard Name	Systematic Name	Standard Name
YGL095C	VPS45	YOR035C	SHE4
YDR004W	RAD57	YER162C	RAD4
YHR178W	STB5	YMR127C	SAS2
YDR004W	RAD57	YOL090W	MSH2
YBR111W-A	SUS1	YBR009C	HHF1
YGL078C	DBP3	YPL161C	BEM4
YBR131W	CCZ1	YNL051W	COG5
YOR106W	VAM3	YDL077C	VAM6
YBR200W	BEM1	YFR030W	MET10
YHR134W	WSS1	YHR178W	STB5
YMR055C	BUB2	YGR188C	BUB1
YJL124C	LSM1	YPR120C	CLB5
YCR081W	SRB8	YOL051W	GAL11
YDR080W	VPS41	YKR030W	GMH1
YCL037C	SRO9	YPR032W	SRO7
YMR216C	SKY1	YNR032W	PPG1
YNR072W	HXT17	YJR084W	CSN12
YPL037C	EGD1	YDR252W	BTT1
YJR084W	CSN12	YEL060C	PRB1
YDR507C	GIN4	YJL095W	BCK1
YNL041C	COG6	YNL076W	MKS1
YDR293C	SSD1	YJL053W	PEP8
YKL025C	PAN3	YOR127W	RGA1
YPR017C	DSS4	YML071C	COG8
YER016W	BIM1	YBR089C-A	NHP6B
YPL042C	SSN3	YMR038C	CCS1
YDR202C	RAV2	YJR032W	CPR7
YHR013C	ARD1	YJR084W	CSN12
YGR239C	PEX21	YDR142C	PEX7

Predictions at threshold level 0.75 continued...			
Gene1 of predicted pair		Gene2 of predicted pair	
Systematic Name	Standard Name	Systematic Name	Standard Name
YLR254C	NDL1	YOR141C	ARP8
YNL206C	RTT106	YMR199W	CLN1
YDL047W	SIT4	YLR182W	SWI6
YCR020C-A	MAK31	YNL297C	MON2
YJR032W	CPR7	YBR114W	RAD16
YLR381W	CTF3	YJL006C	CTK2
YEL065W	SIT1	YLR180W	SAM1
YKL139W	CTK1	YNL054W	VAC7
YEL043W	YEL043W	YGR105W	VMA21
YGR072W	UPF3	YOR109W	INP53
YOR023C	AHC1	YEL053C	MAK10
YBL079W	NUP170	YGL163C	RAD54
YNL230C	ELA1	YLR219W	MSC3
YKL048C	ELM1	YLR094C	GIS3
YHL031C	GOS1	YHL020C	OPI1
YBR112C	CYC8	YGL025C	PGD1
YBR200W	BEM1	YOR290C	SNF2
YKR072C	SIS2	YHR178W	STB5
YJL138C	TIF2	YPR119W	CLB2
YPL022W	RAD1	YGR135W	PRE9
YDR293C	SSD1	YPL195W	APL5
YNL307C	MCK1	YLR180W	SAM1
YLR381W	CTF3	YHR114W	BZZ1
YKR020W	VPS51	YBL025W	RRN10
YBR217W	ATG12	YOR327C	SNC2
YGL237C	HAP2	YDR440W	DOT1
YLR360W	VPS38	YHR012W	VPS29
YCL001W	RER1	YGL115W	SNF4
YER054C	GIP2	YPR119W	CLB2

Predictions at threshold level 0.75 continued...			
Gene1 of predicted pair		Gene2 of predicted pair	
Systematic Name	Standard Name	Systematic Name	Standard Name
YDL184C	RPL41A	YLR094C	GIS3
YDR309C	GIC2	YLR425W	TUS1
YMR095C	SNO1	YBR171W	SEC66
YCR034W	FEN1	YJL138C	TIF2
YDR293C	SSD1	YBR171W	SEC66
YLR094C	GIS3	YLR320W	MMS22
YLR371W	ROM2	YML071C	COG8
YKL048C	ELM1	YBL101C	ECM21
YFR031C-A	RPL2A	YLR360W	VPS38
YIL036W	CST6	YJL183W	MNN11
YDL020C	RPN4	YGR020C	VMA7
YIL053W	RHR2	YML001W	YPT7
YDL101C	DUN1	YOR080W	DIA2
YBR084C-A	RPL19A	YIL008W	URM1
YIL036W	CST6	YJR060W	CBF1
YER087C-B	SBH1	YLR418C	CDC73
YBL008W	HIR1	YBR200W	BEM1
YBL008W	HIR1	YOR368W	RAD17
YML103C	NUP188	YOR195W	SLK19
YLR396C	VPS33	YGL095C	VPS45
YML006C	GIS4	YGL115W	SNF4
YLR090W	XDJ1	YMR153W	NUP53
YPR030W	CSR2	YLR370C	ARC18
YOR112W	CEX1	YML001W	YPT7
YPR036W	VMA13	YER122C	GLO3
YEL065W	SIT1	YOR043W	WHI2
YBL102W	SFT2	YDR310C	SUM1
YOR195W	SLK19	YNL206C	RTT106
YLR288C	MEC3	YCR031C	RPS14A

Predictions at threshold level 0.75 continued...			
Gene1 of predicted pair		Gene2 of predicted pair	
Systematic Name	Standard Name	Systematic Name	Standard Name
YJL154C	VPS35	YLR292C	SEC72
YNL206C	RTT106	YIL008W	URM1
YLR061W	RPL22A	YHR013C	ARD1
YDR507C	GIN4	YNL116W	DMA2
YLR238W	FAR10	YMR052W	FAR3
YDR507C	GIN4	YNL078W	NIS1
YJR140C	HIR3	YOR243C	PUS7
YGR261C	APL6	YJL036W	SNX4
YDL013W	SLX5	YPR046W	MCM16
YMR055C	BUB2	YJR032W	CPR7
YPL161C	BEM4	YLR180W	SAM1
YMR080C	NAM7	YPL079W	RPL21B
YNL307C	MCK1	YBL025W	RRN10
YFR036W	CDC26	YLR180W	SAM1
YKL025C	PAN3	YBR107C	IML3
YNL069C	RPL16B	YNR032W	PPG1
YDR484W	VPS52	YLR452C	SST2
YDL223C	HBT1	YOL121C	RPS19A
YML103C	NUP188	YFL047W	RGD2
YIR005W	IST3	YAR014C	BUD14
YBR107C	IML3	YPL018W	CTF19
YJL127C	SPT10	YNL107W	YAF9
YNL325C	FIG4	YCR053W	THR4
YMR026C	PEX12	YFL023W	BUD27
YML016C	PPZ1	YLR182W	SWI6
YMR095C	SNO1	YDR207C	UME6
YLR425W	TUS1	YEL036C	ANP1
YHL020C	OPI1	YER122C	GLO3
YML115C	VAN1	YER049W	TPA1

Predictions at threshold level 0.75 continued...			
Gene1 of predicted pair		Gene2 of predicted pair	
Systematic Name	Standard Name	Systematic Name	Standard Name
YDR174W	HMO1	YPR120C	CLB5
YPR120C	CLB5	YOL054W	PSH1
YBL103C	RTG3	YGL115W	SNF4
YHR134W	WSS1	YMR055C	BUB2
YBL058W	SHP1	YLR094C	GIS3
YHR134W	WSS1	YMR127C	SAS2
YJL154C	VPS35	YPL256C	CLN2
YML067C	ERV41	YKR014C	YPT52
YFR010W	UBP6	YMR198W	CIK1
YDL042C	SIR2	YGR252W	GCN5
YMR026C	PEX12	YLR191W	PEX13
YLR288C	MEC3	YGL019W	CKB1
YNL054W	VAC7	YBR200W	BEM1
YDL116W	NUP84	YLR429W	CRN1
YLR337C	VRP1	YOR332W	VMA4
YDR207C	UME6	YMR230W	RPS10B
YKL126W	YPK1	YBR036C	CSG2
YDR477W	SNF1	YER144C	UBP5
YLR425W	TUS1	YHR115C	DMA1
YFR032C-A	RPL29	YML115C	VAN1
YMR142C	RPL13B	YLR398C	SKI2
YDR289C	RTT103	YNL147W	LSM7
YIL034C	CAP2	YJR135C	MCM22
YNL264C	PDR17	YER118C	SHO1
YDL223C	HBT1	YMR311C	GLC8
YEL065W	SIT1	YKL139W	CTK1
YDR309C	GIC2	YJL157C	FAR1
YBR107C	IML3	YMR038C	CCS1
YPL234C	TFP3	YHR039C-A	VMA10

Predictions at threshold level 0.75 continued...			
Gene1 of predicted pair		Gene2 of predicted pair	
Systematic Name	Standard Name	Systematic Name	Standard Name
YJR056C	YJR056C	YLR452C	SST2
YBR217W	ATG12	YLR398C	SKI2
YPL161C	BEM4	YKL041W	VPS24
YKL025C	PAN3	YBR171W	SEC66
YHR013C	ARD1	YLR306W	UBC12
YDR443C	SSN2	YGL025C	PGD1
YHR114W	BZZ1	YGR188C	BUB1
YJL204C	RCY1	YGL249W	ZIP2
YAL024C	LTE1	YOR043W	WHI2
YPL240C	HSP82	YLR419W	YLR419W
YKL032C	IXR1	YGL031C	RPL24A
YNL215W	IES2	YLR234W	TOP3
YNL243W	SLA2	YLR206W	ENT2
YGL076C	RPL7A	YGL246C	RAI1
YLR337C	VRP1	YJL095W	BCK1
YMR172W	HOT1	YLR386W	VAC14
YDR202C	RAV2	YHR039C-A	VMA10
YIL076W	SEC28	YJR056C	YJR056C
YFR032C-A	RPL29	YJL183W	MNN11
YHR079C	IRE1	YHR098C	SFB3
YDR507C	GIN4	YJL157C	FAR1
YER001W	MNN1	YEL036C	ANP1
YLR254C	NDL1	YDR183W	PLP1
YKL007W	CAP1	YNL307C	MCK1
YCR077C	PAT1	YJL095W	BCK1
YGL078C	DBP3	YJR084W	CSN12
YCR066W	RAD18	YNL051W	COG5
YAL024C	LTE1	YLR216C	CPR6
YAL014C	SYN8	YJR125C	ENT3

Predictions at threshold level 0.75 continued...			
Gene1 of predicted pair		Gene2 of predicted pair	
Systematic Name	Standard Name	Systematic Name	Standard Name
YMR224C	MRE11	YFL023W	BUD27
YNL025C	SSN8	YIL076W	SEC28
YOR195W	SLK19	YJR063W	RPA12
YLR386W	VAC14	YNL325C	FIG4
YPL256C	CLN2	YDR310C	SUM1
YNL243W	SLA2	YMR022W	UBC7
YPL140C	MKK2	YDL159W	STE7
YLL007C	YLL007C	YDR318W	MCM21
YKR061W	KTR2	YLR410W	VIP1
YKL139W	CTK1	YKL041W	VPS24
YML115C	VAN1	YKL126W	YPK1
YKL160W	ELF1	YIR005W	IST3
YGR108W	CLB1	YLR079W	SIC1
YMR224C	MRE11	YFR030W	MET10
YPR046W	MCM16	YJL095W	BCK1
YOR132W	VPS17	YLR386W	VAC14
YOL111C	MDY2	YMR152W	YIM1
YJL058C	BIT61	YDR254W	CHL4
YHL031C	GOS1	YEL051W	VMA8
YBR108W	YBR108W	YOL081W	IRA2
YMR004W	MVP1	YGL212W	VAM7
YNL166C	BNI5	YDR310C	SUM1
YHR010W	RPL27A	YJR090C	GRR1
YLL039C	UBI4	YMR276W	DSK2
YMR039C	SUB1	YOL145C	CTR9
YCR077C	PAT1	YCR086W	CSM1
YOR290C	SNF2	YNL058C	YNL058C
YBR215W	HPC2	YOR368W	RAD17
YHR052W	CIC1	YGL189C	RPS26A

Predictions at threshold level 0.75 continued...			
Gene1 of predicted pair		Gene2 of predicted pair	
Systematic Name	Standard Name	Systematic Name	Standard Name
YPR018W	RLF2	YDL042C	SIR2
YGL237C	HAP2	YPR046W	MCM16
YBR215W	HPC2	YDR076W	RAD55
YNL206C	RTT106	YCR084C	TUP1
YMR039C	SUB1	YJL124C	LSM1
YKL048C	ELM1	YBR019C	GAL10
YHR013C	ARD1	YGR135W	PRE9
YKL048C	ELM1	YNL076W	MKS1
YDL155W	CLB3	YMR038C	CCS1
YOR080W	DIA2	YOL090W	MSH2
YJL124C	LSM1	YNL147W	LSM7
YLR381W	CTF3	YIL103W	DPH1
YML095C	RAD10	YDL047W	SIT4
YLR386W	VAC14	YJR063W	RPA12
YLR119W	SRN2	YGL212W	VAM7
YGL163C	RAD54	YMR038C	CCS1
YMR199W	CLN1	YER110C	KAP123
YMR092C	AIP1	YIL034C	CAP2
YGR004W	PEX31	YLR324W	PEX30
YJR084W	CSN12	YMR230W	RPS10B
YNL206C	RTT106	YDR289C	RTT103
YBR093C	PHO5	YNL107W	YAF9
YPR046W	MCM16	YEL056W	HAT2
YHR152W	SPO12	YBL047C	EDE1
YNL166C	BNI5	YAR014C	BUD14
YIR003W	YIR003W	YIL034C	CAP2
YGL178W	MPT5	YBR089C-A	NHP6B
YJR060W	CBF1	YHR115C	DMA1
YNR072W	HXT17	YER162C	RAD4

Predictions at threshold level 0.75 continued...			
Gene1 of predicted pair		Gene2 of predicted pair	
Systematic Name	Standard Name	Systematic Name	Standard Name
YNL087W	TCB2	YNL238W	KEX2
YDR335W	MSN5	YMR038C	CCS1
YLR079W	SIC1	YOR039W	CKB2
YGR261C	APL6	YHR060W	VMA22
YGR071C	YGR071C	YDR318W	MCM21
YLR360W	VPS38	YHR161C	YAP1801
YAL024C	LTE1	YLR381W	CTF3
YIL034C	CAP2	YLR447C	VMA6
YGR014W	MSB2	YJL183W	MNN11
YGR249W	MGA1	YBR019C	GAL10
YLL007C	YLL007C	YHR178W	STB5
YGL174W	BUD13	YKL074C	MUD2
YGR072W	UPF3	YPL234C	TFP3
YMR055C	BUB2	YDR144C	MKC7
YJR052W	RAD7	YGL115W	SNF4
YKL213C	DOA1	YLR182W	SWI6
YHR205W	SCH9	YOR027W	STI1
YPL024W	RMI1	YGL003C	CDH1
YKL048C	ELM1	YDL192W	ARF1
YMR179W	SPT21	YMR038C	CCS1
YBL003C	HTA2	YPL248C	GAL4
YBR131W	CCZ1	YML071C	COG8
YHR134W	WSS1	YBL021C	HAP3
YCR077C	PAT1	YOR308C	SNU66
YKL139W	CTK1	YHR111W	UBA4
YKR020W	VPS51	YDR153C	ENT5
YER116C	SLX8	YBR036C	CSG2
YPR102C	RPL11A	YER102W	RPS8B
YHR134W	WSS1	YCL037C	SRO9

Predictions at threshold level 0.75 continued...			
Gene1 of predicted pair		Gene2 of predicted pair	
Systematic Name	Standard Name	Systematic Name	Standard Name
YJL036W	SNX4	YMR216C	SKY1
YMR038C	CCS1	YAL002W	VPS8
YKL139W	CTK1	YMR055C	BUB2
YLR371W	ROM2	YLR370C	ARC18
YKL007W	CAP1	YHR111W	UBA4
YPR046W	MCM16	YDR207C	UME6
YGL087C	MMS2	YDR155C	CPR1
YMR231W	PEP5	YPR028W	YOP1
YKR020W	VPS51	YJR033C	RAV1
YMR224C	MRE11	YLR425W	TUS1
YNL215W	IES2	YNR052C	POP2
YMR055C	BUB2	YHR200W	RPN10
YGL190C	CDC55	YNL250W	RAD50
YNL051W	COG5	YNR032W	PPG1
YDL101C	DUN1	YDR207C	UME6
YAL024C	LTE1	YLR410W	VIP1
YPR046W	MCM16	YLR398C	SKI2
YNL025C	SSN8	YIR005W	IST3
YMR199W	CLN1	YLR371W	ROM2
YNL264C	PDR17	YNL107W	YAF9
YJL176C	SWI3	YFR010W	UBP6
YLR079W	SIC1	YLR370C	ARC18
YGL033W	HOP2	YOR290C	SNF2
YLR192C	HCR1	YPL195W	APL5
YAL024C	LTE1	YDL192W	ARF1
YGL124C	MON1	YHR114W	BZZ1
YBR127C	VMA2	YPR119W	CLB2
YKL032C	IXR1	YPR046W	MCM16
YML095C	RAD10	YJR032W	CPR7

Predictions at threshold level 0.75 continued...			
Gene1 of predicted pair		Gene2 of predicted pair	
Systematic Name	Standard Name	Systematic Name	Standard Name
YEL050C	RML2	YDL047W	SIT4
YGL200C	EMP24	YML097C	VPS9
YDR295C	HDA2	YPR179C	HDA3
YLR386W	VAC14	YGR135W	PRE9
YDR363W-A	SEM1	YPR120C	CLB5
YJL089W	SIP4	YPL248C	GAL4
YNL041C	COG6	YJR084W	CSN12
YDR477W	SNF1	YML115C	VAN1
YGL215W	CLG1	YOR080W	DIA2
YOR066W	MSA1	YPR046W	MCM16
YDR105C	TMS1	YPR149W	NCE102
YNL032W	SIW14	YJR056C	YJR056C
YOR106W	VAM3	YER049W	TPA1
YHR187W	IKI1	YDL047W	SIT4
YBR215W	HPC2	YLR182W	SWI6
YGL200C	EMP24	YCL001W	RER1
YOR066W	MSA1	YMR214W	SCJ1
YDR293C	SSD1	YOR358W	HAP5
YGR070W	ROM1	YLR425W	TUS1
YGR054W	YGR054W	YHR012W	VPS29
YMR026C	PEX12	YPL161C	BEM4
YLR337C	VRP1	YJL190C	RPS22A
YDR293C	SSD1	YOR023C	AHC1
YNL025C	SSN8	YHR167W	THP2
YMR055C	BUB2	YCR077C	PAT1
YER044C-A	MEI4	YMR133W	REC114
YNL201C	PSY2	YJR135C	MCM22
YHL020C	OPI1	YJR033C	RAV1
YPL024W	RMI1	YJR084W	CSN12

Predictions at threshold level 0.75 continued...			
Gene1 of predicted pair		Gene2 of predicted pair	
Systematic Name	Standard Name	Systematic Name	Standard Name
YGR108W	CLB1	YGL115W	SNF4
YLR386W	VAC14	YPR051W	MAK3
YDR142C	PEX7	YGL153W	PEX14
YPL024W	RMI1	YCL037C	SRO9
YLR083C	EMP70	YPL234C	TFP3
YBR111W-A	SUS1	YBR010W	HHT1
YDR103W	STE5	YDR335W	MSN5
YHR156C	LIN1	YNL032W	SIW14
YJL124C	LSM1	YJR060W	CBF1
YIL159W	BNR1	YMR016C	SOK2
YNL307C	MCK1	YOR066W	MSA1
YCR020C-A	MAK31	YGR020C	VMA7
YDR469W	SDC1	YHR115C	DMA1
YBR107C	IML3	YKL114C	APN1
YGR241C	YAP1802	YDR358W	GGA1
YPL256C	CLN2	YJL095W	BCK1
YMR224C	MRE11	YGR249W	MGA1
YDR137W	RGP1	YER161C	SPT2
YGR129W	SYF2	YCR063W	BUD31
YDR477W	SNF1	YNL041C	COG6
YDR383C	NKP1	YKL139W	CTK1
YGL124C	MON1	YNL116W	DMA2
YLR192C	HCR1	YML071C	COG8
YGR081C	SLX9	YGL163C	RAD54
YGL025C	PGD1	YKR048C	NAP1
YLR248W	RCK2	YGL158W	RCK1
YMR243C	ZRC1	YGL158W	RCK1
YGL078C	DBP3	YFR010W	UBP6
YGL178W	MPT5	YLR330W	CHS5

Predictions at threshold level 0.75 continued...			
Gene1 of predicted pair		Gene2 of predicted pair	
Systematic Name	Standard Name	Systematic Name	Standard Name
YGL078C	DBP3	YHR039C-A	VMA10
YPL161C	BEM4	YER040W	GLN3
YOR043W	WHI2	YOR038C	HIR2
YOL018C	TLG2	YDL047W	SIT4
YOR069W	VPS5	YPL105C	YPL105C
YKL116C	PRR1	YDR310C	SUM1
YDR335W	MSN5	YOR141C	ARP8
YLR234W	TOP3	YOR014W	RTS1
YML071C	COG8	YAR014C	BUD14
YKL116C	PRR1	YNL032W	SIW14
YHL031C	GOS1	YKR001C	VPS1
YGR261C	APL6	YCR066W	RAD18
YDR363W-A	SEM1	YOR141C	ARP8
YDR108W	GSG1	YNL238W	KEX2
YDL184C	RPL41A	YHR081W	LRP1
YPR154W	PIN3	YBL106C	SRO77
YGL025C	PGD1	YER162C	RAD4
YLR192C	HCR1	YNL307C	MCK1
YEL009C	GCN4	YOR290C	SNF2
YOR290C	SNF2	YNL236W	SIN4
YPR017C	DSS4	YDR207C	UME6
YBR045C	GIP1	YDR103W	STE5
YHR160C	PEX18	YOL044W	PEX15
YLR360W	VPS38	YDR144C	MKC7
YDR440W	DOT1	YLR182W	SWI6
YBR215W	HPC2	YJR005W	APL1
YNL087W	TCB2	YOR270C	VPH1
YNL044W	YIP3	YER122C	GLO3
YDR146C	SWI5	YPL253C	VIK1

Predictions at threshold level 0.75 continued...			
Gene1 of predicted pair		Gene2 of predicted pair	
Systematic Name	Standard Name	Systematic Name	Standard Name
YDR293C	SSD1	YBL102W	SFT2
YPL037C	EGD1	YLR370C	ARC18
YFL013C	IES1	YGL174W	BUD13
YNL106C	INP52	YBL027W	RPL19B
YKL139W	CTK1	YER068W	MOT2
YIL008W	URM1	YJL187C	SWE1
YDR261C	EXG2	YOL121C	RPS19A
YGR270W	YTA7	YGL151W	NUT1
YGR020C	VMA7	YLR447C	VMA6
YER001W	MNN1	YFL048C	EMP47
YOR112W	CEX1	YMR004W	MVP1
YHR073W	OSH3	YOR358W	HAP5
YNL229C	URE2	YDR264C	AKR1
YDR264C	AKR1	YDR183W	PLP1
YKR020W	VPS51	YEL051W	VMA8
YML095C	RAD10	YDR076W	RAD55
YGL066W	SGF73	YLR386W	VAC14
YCL037C	SRO9	YHR039C-A	VMA10
YDR293C	SSD1	YER120W	SCS2
YJL127C	SPT10	YBR171W	SEC66
YMR004W	MVP1	YDR319C	YDR319C
YBR171W	SEC66	YPL158C	YPL158C
YMR224C	MRE11	YKL116C	PRR1
YHR012W	VPS29	YNR032W	PPG1
YJL006C	CTK2	YPL018W	CTF19
YNL041C	COG6	YEL060C	PRB1
YJR033C	RAV1	YDR310C	SUM1
YGR105W	VMA21	YMR054W	STV1
YDL013W	SLX5	YPR120C	CLB5

Predictions at threshold level 0.75 continued...			
Gene1 of predicted pair		Gene2 of predicted pair	
Systematic Name	Standard Name	Systematic Name	Standard Name
YDL116W	NUP84	YFL023W	BUD27
YAL024C	LTE1	YBR274W	CHK1
YGR129W	SYF2	YGL174W	BUD13
YMR199W	CLN1	YGL208W	SIP2
YLL039C	UBI4	YBL101C	ECM21
YLR079W	SIC1	YKR024C	DBP7
YGR104C	SRB5	YBR009C	HHF1
YMR095C	SNO1	YPL161C	BEM4
YOR033C	EXO1	YOL090W	MSH2
YGL013C	PDR1	YPR119W	CLB2
YOL111C	MDY2	YPR046W	MCM16
YBL106C	SRO77	YGL198W	YIP4
YOR109W	INP53	YAR014C	BUD14
YJL172W	CPS1	YLR441C	RPS1A
YFR031C-A	RPL2A	YER162C	RAD4
YER139C	YER139C	YMR230W	RPS10B
YKL048C	ELM1	YJR084W	CSN12
YBL103C	RTG3	YPL195W	APL5
YDR383C	NKP1	YNR052C	POP2
YIL076W	SEC28	YOR112W	CEX1
YJL176C	SWI3	YNL079C	TPM1
YDR156W	RPA14	YDR439W	LRS4
YHR039C-A	VMA10	YOR368W	RAD17
YLR452C	SST2	YDR310C	SUM1
YPR120C	CLB5	YGR188C	BUB1
YNL025C	SSN8	YDR378C	LSM6
YNL047C	SLM2	YJL058C	BIT61
YEL065W	SIT1	YGL003C	CDH1
YBL051C	PIN4	YJL187C	SWE1

Predictions at threshold level 0.75 continued...			
Gene1 of predicted pair		Gene2 of predicted pair	
Systematic Name	Standard Name	Systematic Name	Standard Name
YMR133W	REC114	YDR358W	GGA1
YDL077C	VAM6	YEL042W	GDA1
YCL001W	RER1	YGL054C	ERV14
YGR020C	VMA7	YNL297C	MON2
YNL051W	COG5	YDR153C	ENT5
YBR290W	BSD2	YJR135C	MCM22
YLR410W	VIP1	YDL047W	SIT4
YKL139W	CTK1	YCR086W	CSM1
YBR194W	SOY1	YIL076W	SEC28
YDR156W	RPA14	YOR141C	ARP8
YNL025C	SSN8	YKL160W	ELF1
YNL106C	INP52	YDR439W	LRS4
YIL008W	URM1	YDR289C	RTT103
YHR010W	RPL27A	YGL246C	RAI1
YLR447C	VMA6	YGR105W	VMA21
YBR215W	HPC2	YER139C	YER139C
YKR035W-A	DID2	YOR035C	SHE4
YMR116C	ASC1	YBL025W	RRN10
YLR337C	VRP1	YOR023C	AHC1
YDR293C	SSD1	YER110C	KAP123
YJR040W	GEF1	YCL001W	RER1
YLR452C	SST2	YDL083C	RPS16B
YDR245W	MNN10	YOR308C	SNU66
YLR192C	HCR1	YMR230W	RPS10B
YOR195W	SLK19	YDR439W	LRS4
YEL027W	CUP5	YKL119C	VPH2
YDR129C	SAC6	YDL155W	CLB3
YMR039C	SUB1	YKR072C	SIS2
YJR140C	HIR3	YCL037C	SRO9

Predictions at threshold level 0.75 continued...			
Gene1 of predicted pair		Gene2 of predicted pair	
Systematic Name	Standard Name	Systematic Name	Standard Name
YKL048C	ELM1	YNL225C	CNM67
YMR039C	SUB1	YNL107W	YAF9
YGL178W	MPT5	YPL031C	PHO85
YBR073W	RDH54	YPR046W	MCM16
YGL087C	MMS2	YHR012W	VPS29
YPL240C	HSP82	YMR165C	PAH1
YPR072W	NOT5	YNL236W	SIN4
YML115C	VAN1	YOR035C	SHE4
YMR246W	FAA4	YOR059C	YOR059C
YML038C	YMD8	YEL043W	YEL043W
YMR224C	MRE11	YIL076W	SEC28
YOR195W	SLK19	YGR188C	BUB1
YKL139W	CTK1	YPR120C	CLB5
YPR084W	YPR084W	YEL051W	VMA8
YMR039C	SUB1	YDR146C	SWI5
YHR111W	UBA4	YIL103W	DPH1
YGR241C	YAP1802	YDL161W	ENT1
YMR026C	PEX12	YLR441C	RPS1A
YDR225W	HTA1	YOR368W	RAD17
YDL116W	NUP84	YNL107W	YAF9
YKL032C	IXR1	YHL002W	HSE1
YML115C	VAN1	YER139C	YER139C
YKL010C	UFD4	YGR184C	UBR1
YML115C	VAN1	YNL076W	MKS1
YDR477W	SNF1	YBL025W	RRN10
YML001W	YPT7	YEL051W	VMA8
YLR441C	RPS1A	YEL060C	PRB1
YGL161C	YIP5	YDR076W	RAD55
YPR120C	CLB5	YGL175C	SAE2

Predictions at threshold level 0.75 continued...			
Gene1 of predicted pair		Gene2 of predicted pair	
Systematic Name	Standard Name	Systematic Name	Standard Name
YGL200C	EMP24	YOR327C	SNC2
YDR477W	SNF1	YJL172W	CPS1
YOR141C	ARP8	YNL225C	CNM67
YHR060W	VMA22	YGL124C	MON1
YPL105C	YPL105C	YPR032W	SRO7
YBL058W	SHP1	YEL053C	MAK10
YCR020C-A	MAK31	YHL020C	OPI1
YLR006C	SSK1	YGR152C	RSR1
YNL238W	KEX2	YJR032W	CPR7
YDR443C	SSN2	YOR043W	WHI2
YGR108W	CLB1	YOL067C	RTG1
YGR135W	PRE9	YLR370C	ARC18
YPL050C	MNN9	YFR036W	CDC26
YNL025C	SSN8	YOR038C	HIR2
YMR055C	BUB2	YHR115C	DMA1
YML103C	NUP188	YIL036W	CST6
YIR009W	MSL1	YKR099W	BAS1
YHR156C	LIN1	YFL023W	BUD27
YJL124C	LSM1	YLR399C	BDF1
YDR507C	GIN4	YLR425W	TUS1
YMR004W	MVP1	YDL186W	YDL186W
YKL010C	UFD4	YLL039C	UBI4
YPL042C	SSN3	YBR112C	CYC8
YPL042C	SSN3	YLR410W	VIP1
YHR134W	WSS1	YHR114W	BZZ1
YJL154C	VPS35	YPL120W	VPS30
YMR164C	MSS11	YMR199W	CLN1
YER087C-B	SBH1	YDR392W	SPT3
YHL031C	GOS1	YOR327C	SNC2

Predictions at threshold level 0.75 continued...			
Gene1 of predicted pair		Gene2 of predicted pair	
Systematic Name	Standard Name	Systematic Name	Standard Name
YPR017C	DSS4	YDR108W	GSG1
YLR386W	VAC14	YER040W	GLN3
YLR441C	RPS1A	YNL307C	MCK1
YNL329C	PEX6	YDR440W	DOT1
YAL024C	LTE1	YML016C	PPZ1
YDL006W	PTC1	YDR358W	GGA1
YMR216C	SKY1	YJL004C	SYS1
YIL103W	DPH1	YML112W	CTK3
YKL116C	PRR1	YPL161C	BEM4
YNR072W	HXT17	YMR152W	YIM1
YKR001C	VPS1	YCL001W	RER1
YDR202C	RAV2	YEL043W	YEL043W
YFL003C	MSH4	YGL249W	ZIP2
YJL154C	VPS35	YML038C	YMD8
YMR172W	HOT1	YGR108W	CLB1
YPR017C	DSS4	YJR033C	RAV1
YNL243W	SLA2	YJR058C	APS2
YML012W	ERV25	YLR093C	NYV1
YER110C	KAP123	YBR009C	HHF1
YLR095C	IOC2	YOR290C	SNF2
YCL037C	SRO9	YPL079W	RPL21B
YJL210W	PEX2	YDR244W	PEX5
YAL024C	LTE1	YER162C	RAD4
YGL003C	CDH1	YER122C	GLO3
YDR422C	SIP1	YGL179C	TOS3
YPL106C	SSE1	YJL006C	CTK2
YCR081W	SRB8	YER096W	SHC1
YLR399C	BDF1	YNL031C	HHT2
YNL238W	KEX2	YPL195W	APL5

Predictions at threshold level 0.75 continued...			
Gene1 of predicted pair		Gene2 of predicted pair	
Systematic Name	Standard Name	Systematic Name	Standard Name
YPR046W	MCM16	YER122C	GLO3
YER144C	UBP5	YBR280C	SAF1
YHR205W	SCH9	YBR260C	RGD1
YDR004W	RAD57	YHR013C	ARD1
YDL020C	RPN4	YBR228W	SLX1
YNL243W	SLA2	YPL031C	PHO85
YHR098C	SFB3	YFL031W	HAC1
YIL036W	CST6	YGL252C	RTG2
YLR386W	VAC14	YLR102C	APC9
YDL020C	RPN4	YGL237C	HAP2
YLR095C	IOC2	YOR066W	MSA1
YOL136C	PFK27	YLR043C	TRX1
YMR224C	MRE11	YGL163C	RAD54
YFR024C-A	LSB3	YMR253C	YMR253C
YEL051W	VMA8	YLR309C	IMH1
YIL076W	SEC28	YBR171W	SEC66
YGL178W	MPT5	YPR072W	NOT5
YEL053C	MAK10	YAR014C	BUD14
YKL178C	STE3	YJR086W	STE18
YLR095C	IOC2	YOL081W	IRA2
YLR399C	BDF1	YNL236W	SIN4
YDR378C	LSM6	YDR254W	CHL4
YMR004W	MVP1	YDR137W	RGP1
YOR033C	EXO1	YKL114C	APN1
YLR320W	MMS22	YBR280C	SAF1
YOR035C	SHE4	YOR036W	PEP12
YPR084W	YPR084W	YGL223C	COG1
YDR174W	HMO1	YJL124C	LSM1
YML103C	NUP188	YOR141C	ARP8

Predictions at threshold level 0.75 continued...			
Gene1 of predicted pair		Gene2 of predicted pair	
Systematic Name	Standard Name	Systematic Name	Standard Name
YNL243W	SLA2	YMR109W	MYO5
YHR056C	RSC30	YKR082W	NUP133
YDR293C	SSD1	YMR127C	SAS2
YBR290W	BSD2	YOR023C	AHC1
YPR051W	MAK3	YDL006W	PTC1
YNL054W	VAC7	YAR014C	BUD14
YPL037C	EGD1	YJL004C	SYS1
YHR079C	IRE1	YNR049C	MSO1
YPL115C	BEM3	YAR014C	BUD14
YMR199W	CLN1	YNL079C	TPM1
YDR245W	MNN10	YNL107W	YAF9
YFR032C-A	RPL29	YLR320W	MMS22
YGR261C	APL6	YJR033C	RAV1
YBR010W	HHT1	YDR216W	ADR1
YOR332W	VMA4	YJL183W	MNN11
YGL124C	MON1	YNL107W	YAF9
YOR109W	INP53	YOR327C	SNC2
YCR081W	SRB8	YPL161C	BEM4
YOR132W	VPS17	YDR358W	GGA1
YMR026C	PEX12	YMR133W	REC114
YDR254W	CHL4	YDR310C	SUM1
YOR035C	SHE4	YMR214W	SCJ1
YGL025C	PGD1	YPR120C	CLB5
YGR019W	UGA1	YPR046W	MCM16
YOR132W	VPS17	YAR014C	BUD14
YGR159C	NSR1	YKL041W	VPS24
YDR176W	NGG1	YOL148C	SPT20
YBR057C	MUM2	YKL139W	CTK1
YLR452C	SST2	YML071C	COG8

Predictions at threshold level 0.75 continued...			
Gene1 of predicted pair		Gene2 of predicted pair	
Systematic Name	Standard Name	Systematic Name	Standard Name
YLR386W	VAC14	YLR180W	SAM1
YOR035C	SHE4	YOL081W	IRA2
YDR320C	SWA2	YPR051W	MAK3
YMR016C	SOK2	YJR056C	YJR056C
YDL080C	THI3	YLR337C	VRP1
YKL139W	CTK1	YML115C	VAN1
YBR042C	CST26	YOR080W	DIA2
YMR109W	MYO5	YHL002W	HSE1
YKL032C	IXR1	YKL101W	HSL1
YGL115W	SNF4	YPR046W	MCM16
YBR171W	SEC66	YAL002W	VPS8
YPR051W	MAK3	YML071C	COG8
YDR137W	RGP1	YLR398C	SKI2
YDR293C	SSD1	YHL031C	GOS1
YML001W	YPT7	YMR214W	SCJ1
YKL032C	IXR1	YDR076W	RAD55
YFR010W	UBP6	YMR133W	REC114
YIL053W	RHR2	YBR200W	BEM1
YOR332W	VMA4	YMR054W	STV1
YPL024W	RMI1	YCR031C	RPS14A
YGL078C	DBP3	YER116C	SLX8
YMR133W	REC114	YNL041C	COG6
YLL007C	YLL007C	YEL053C	MAK10
YJL176C	SWI3	YLR357W	RSC2
YGL066W	SGF73	YNL229C	URE2
YLR150W	STM1	YBR107C	IML3
YJL154C	VPS35	YDL006W	PTC1
YHR161C	YAP1801	YPL045W	VPS16
YBL008W	HIR1	YDL047W	SIT4

Predictions at threshold level 0.75 continued...			
Gene1 of predicted pair		Gene2 of predicted pair	
Systematic Name	Standard Name	Systematic Name	Standard Name
YLR320W	MMS22	YDL077C	VAM6
YLR234W	TOP3	YAR014C	BUD14
YBR270C	BIT2	YPR119W	CLB2
YBL008W	HIR1	YBR089C-A	NHP6B
YBR073W	RDH54	YNL248C	RPA49
YOR039W	CKB2	YKL048C	ELM1
YHR200W	RPN10	YER122C	GLO3
YDR080W	VPS41	YEL013W	VAC8
YPL115C	BEM3	YOR358W	HAP5
YDR477W	SNF1	YHR039C-A	VMA10
YLR102C	APC9	YDR379W	RGA2
YGL153W	PEX14	YBL101C	ECM21
YDR254W	CHL4	YDL047W	SIT4
YMR319C	FET4	YPR149W	NCE102
YKR020W	VPS51	YOR036W	PEP12
YMR029C	FAR8	YPR119W	CLB2
YLR360W	VPS38	YGR188C	BUB1
YJR140C	HIR3	YDR310C	SUM1
YJL136C	RPS21B	YBR084C-A	RPL19A
YMR198W	CIK1	YML016C	PPZ1
YFR036W	CDC26	YPL018W	CTF19
YDR378C	LSM6	YJR135C	MCM22
YDL005C	MED2	YNL031C	HHT2
YFR010W	UBP6	YJL172W	CPS1
YHR012W	VPS29	YOR014W	RTS1
YKL010C	UFD4	YGL153W	PEX14
YBR173C	UMP1	YLR371W	ROM2
YDL116W	NUP84	YLR315W	NKP2
YKL092C	BUD2	YGR152C	RSR1

Predictions at threshold level 0.75 continued...			
**Gene1 of predicted pair**		**Gene2 of predicted pair**	
**Systematic Name**	**Standard Name**	**Systematic Name**	**Standard Name**
YEL056W	HAT2	YOL067C	RTG1
YLR018C	POM34	YKL068W	NUP100
YMR004W	MVP1	YPL234C	TFP3
YLR371W	ROM2	YPR046W	MCM16
YGL033W	HOP2	YLR093C	NYV1
YLR083C	EMP70	YGL223C	COG1
YHR012W	VPS29	YDR183W	PLP1
YPR154W	PIN3	YHR114W	BZZ1
YNL325C	FIG4	YBR108W	YBR108W
YPL213W	LEA1	YKL041W	VPS24
YLR083C	EMP70	YGL212W	VAM7
YLR234W	TOP3	YGR108W	CLB1
YDR153C	ENT5	YPR032W	SRO7
YLL039C	UBI4	YGL153W	PEX14
YLR337C	VRP1	YMR032W	HOF1
YOL121C	RPS19A	YDR358W	GGA1
YML058W	SML1	YKL139W	CTK1
YAL051W	OAF1	YOR363C	PIP2
YIL002C	INP51	YDR358W	GGA1
YNL291C	MID1	YEL053C	MAK10
YPL024W	RMI1	YLR381W	CTF3
YML071C	COG8	YBR019C	GAL10
YIL076W	SEC28	YNL058C	YNL058C
YGR270W	YTA7	YPR046W	MCM16
YGL147C	RPL9A	YGL033W	HOP2
YER116C	SLX8	YLR441C	RPS1A
YDL161W	ENT1	YKR020W	VPS51
YLR371W	ROM2	YFR036W	CDC26
YBR030W	YBR030W	YMR311C	GLC8

Predictions at threshold level 0.75 continued...			
Gene1 of predicted pair		Gene2 of predicted pair	
**Systematic Name**	**Standard Name**	**Systematic Name**	**Standard Name**
YML095C	RAD10	YNL116W	DMA2
YGL178W	MPT5	YNL236W	SIN4
YBR107C	IML3	YGL115W	SNF4
YNL215W	IES2	YOL054W	PSH1
YKL139W	CTK1	YGL163C	RAD54
YML095C	RAD10	YDR363W-A	SEM1
YDR080W	VPS41	YNL127W	FAR11
YEL043W	YEL043W	YNL051W	COG5
YOR069W	VPS5	YPL195W	APL5
YML001W	YPT7	YJL004C	SYS1
YDR283C	GCN2	YKR026C	GCN3
YDR129C	SAC6	YBR181C	RPS6B
YHR156C	LIN1	YBR280C	SAF1
YLR095C	IOC2	YER122C	GLO3
YPL240C	HSP82	YHL050C	YHL050C
YFR010W	UBP6	YIR005W	IST3
YDR137W	RGP1	YKL041W	VPS24
YML071C	COG8	YKL119C	VPH2
YNL218W	MGS1	YBR082C	UBC4
YHR178W	STB5	YBL051C	PIN4
YPL234C	TFP3	YDL077C	VAM6
YNL025C	SSN8	YPL248C	GAL4
YBR131W	CCZ1	YML001W	YPT7
YDR192C	NUP42	YDR076W	RAD55
YMR224C	MRE11	YEL060C	PRB1
YML001W	YPT7	YPL045W	VPS16
YOR195W	SLK19	YGL031C	RPL24A
YGL237C	HAP2	YBR171W	SEC66
YER044C	ERG28	YPL137C	GIP3

Predictions at threshold level 0.75 continued...			
Gene1 of predicted pair		Gene2 of predicted pair	
Systematic Name	Standard Name	Systematic Name	Standard Name
YPL174C	NIP100	YIL033C	BCY1
YHR111W	UBA4	YPR046W	MCM16
YHR205W	SCH9	YEL036C	ANP1
YJR135C	MCM22	YNL116W	DMA2
YKL139W	CTK1	YOL081W	IRA2
YBL102W	SFT2	YJL036W	SNX4
YNL307C	MCK1	YLR398C	SKI2
YMR116C	ASC1	YLR135W	SLX4
YPR032W	SRO7	YOL108C	INO4
YOR069W	VPS5	YPL259C	APM1
YOR127W	RGA1	YAL002W	VPS8
YNL069C	RPL16B	YPL105C	YPL105C
YMR095C	SNO1	YMR153W	NUP53
YKL214C	YRA2	YNL253W	TEX1
YDR146C	SWI5	YER161C	SPT2
YLR191W	PEX13	YDR142C	PEX7
YCL027W	FUS1	YER118C	SHO1
YKR024C	DBP7	YML057W	CMP2
YBR200W	BEM1	YBL051C	PIN4
YPL042C	SSN3	YPR046W	MCM16
YER116C	SLX8	YDL059C	RAD59
YER116C	SLX8	YDL155W	CLB3
YNL087W	TCB2	YOR035C	SHE4
YNL069C	RPL16B	YOL018C	TLG2
YPR131C	NAT3	YMR224C	MRE11
YBR194W	SOY1	YGR014W	MSB2
YNL291C	MID1	YPL195W	APL5
YGL212W	VAM7	YNL307C	MCK1
YGL054C	ERV14	YML001W	YPT7

Predictions at threshold level 0.75 continued...			
Gene1 of predicted pair		Gene2 of predicted pair	
Systematic Name	Standard Name	Systematic Name	Standard Name
YNL243W	SLA2	YLL021W	SPA2
YMR224C	MRE11	YGR188C	BUB1
YER110C	KAP123	YGL016W	KAP122
YPR040W	TIP41	YKL048C	ELM1
YGL163C	RAD54	YNL116W	DMA2
YOR080W	DIA2	YPR120C	CLB5
YJR056C	YJR056C	YJL183W	MNN11
YBL051C	PIN4	YOR358W	HAP5
YLR396C	VPS33	YMR231W	PEP5
YDR004W	RAD57	YKR095W	MLP1
YDR383C	NKP1	YFR030W	MET10
YDR484W	VPS52	YOR036W	PEP12
YMR095C	SNO1	YBL025W	RRN10
YAL020C	ATS1	YDR507C	GIN4
YNL041C	COG6	YKR030W	GMH1
YDR264C	AKR1	YKR048C	NAP1
YDR245W	MNN10	YJL183W	MNN11
YGL237C	HAP2	YGR188C	BUB1
YLR018C	POM34	YIL148W	RPL40A
YBR107C	IML3	YDR363W-A	SEM1
YHR134W	WSS1	YKL048C	ELM1
YLR337C	VRP1	YER001W	MNN1
YCR066W	RAD18	YJR060W	CBF1
YGL066W	SGF73	YDR264C	AKR1
YGL147C	RPL9A	YOR066W	MSA1
YMR055C	BUB2	YOR038C	HIR2
YHL020C	OPI1	YPL234C	TFP3
YKL116C	PRR1	YOL111C	MDY2
YBR200W	BEM1	YAR014C	BUD14

Predictions at threshold level 0.75 continued...			
Gene1 of predicted pair		Gene2 of predicted pair	
Systematic Name	Standard Name	Systematic Name	Standard Name
YBR289W	SNF5	YBR010W	HHT1
YLR433C	CNA1	YIR009W	MSL1
YMR055C	BUB2	YLR233C	EST1
YDR320C	SWA2	YFL026W	STE2
YDR422C	SIP1	YPL256C	CLN2
YDR245W	MNN10	YOR368W	RAD17
YNL243W	SLA2	YBR164C	ARL1
YKL135C	APL2	YDL006W	PTC1
YGL163C	RAD54	YGL151W	NUT1
YNL041C	COG6	YBR280C	SAF1
YGL115W	SNF4	YER027C	GAL83
YPR167C	MET16	YLR093C	NYV1
YOR035C	SHE4	YBR036C	CSG2
YJL124C	LSM1	YML088W	UFO1
YDR289C	RTT103	YOL067C	RTG1
YJL140W	RPB4	YNL107W	YAF9
YDR129C	SAC6	YKL032C	IXR1
YDR484W	VPS52	YFL023W	BUD27
YMR055C	BUB2	YDL155W	CLB3
YDL020C	RPN4	YHR111W	UBA4
YDR202C	RAV2	YCR066W	RAD18
YMR199W	CLN1	YML028W	TSA1
YIR005W	IST3	YML112W	CTK3
YCR081W	SRB8	YEL036C	ANP1
YNL069C	RPL16B	YCR077C	PAT1
YLR386W	VAC14	YMR153W	NUP53
YOR112W	CEX1	YNL297C	MON2
YHR167W	THP2	YLR182W	SWI6
YGL200C	EMP24	YER118C	SHO1

Predictions at threshold level 0.75 continued...			
Gene1 of predicted pair		Gene2 of predicted pair	
Systematic Name	Standard Name	Systematic Name	Standard Name
YIL053W	RHR2	YHR200W	RPN10
YIL034C	CAP2	YPL161C	BEM4
YPL057C	SUR1	YBL025W	RRN10
YPR036W	VMA13	YNL238W	KEX2
YBL047C	EDE1	YOR368W	RAD17
YLR102C	APC9	YNL206C	RTT106
YPL112C	PEX25	YPR119W	CLB2
YFR010W	UBP6	YLR320W	MMS22
YOR080W	DIA2	YHR167W	THP2
YJL172W	CPS1	YFR036W	CDC26
YDR004W	RAD57	YGR188C	BUB1
YPL024W	RMI1	YDR245W	MNN10
YEL015W	EDC3	YJR140C	HIR3
YPL180W	TCO89	YML097C	VPS9
YGL078C	DBP3	YJL095W	BCK1
YPL042C	SSN3	YOR156C	NFI1
YGR184C	UBR1	YBR107C	IML3
YOR368W	RAD17	YBR036C	CSG2
YLL007C	YLL007C	YKL048C	ELM1
YPL024W	RMI1	YPL161C	BEM4
YLR386W	VAC14	YDR310C	SUM1
YKL139W	CTK1	YOR141C	ARP8
YPL129W	TAF14	YNR023W	SNF12
YMR004W	MVP1	YKR020W	VPS51
YNL058C	YNL058C	YFR036W	CDC26
YBR107C	IML3	YHR111W	UBA4
YBR274W	CHK1	YGL163C	RAD54
YOR270C	VPH1	YLR452C	SST2
YPL253C	VIK1	YOL081W	IRA2

Predictions at threshold level 0.75 continued...			
Gene1 of predicted pair		Gene2 of predicted pair	
Systematic Name	Standard Name	Systematic Name	Standard Name
YGL054C	ERV14	YDR207C	UME6
YBL051C	PIN4	YPL253C	VIK1
YGR020C	VMA7	YCL001W	RER1
YPR018W	RLF2	YJR135C	MCM22
YPL057C	SUR1	YGR071C	YGR071C
YKR035W-A	DID2	YER122C	GLO3
YPL024W	RMI1	YKL126W	YPK1
YGR241C	YAP1802	YCR088W	ABP1
YML038C	YMD8	YLR309C	IMH1
YDR484W	VPS52	YBR019C	GAL10
YDL101C	DUN1	YML095C	RAD10
YDR144C	MKC7	YAR014C	BUD14
YGR097W	ASK10	YPL248C	GAL4
YJR032W	CPR7	YNL107W	YAF9
YPR017C	DSS4	YNL107W	YAF9
YHR205W	SCH9	YOR014W	RTS1
YDL116W	NUP84	YPL246C	RBD2
YBL047C	EDE1	YKL161C	YKL161C
YGR261C	APL6	YPL105C	YPL105C
YML071C	COG8	YNL107W	YAF9
YCL037C	SRO9	YDR207C	UME6
YPL001W	HAT1	YNL031C	HHT2
YBR041W	FAT1	YPL161C	BEM4
YOR071C	THI71	YPR149W	NCE102
YNL288W	CAF40	YDR254W	CHL4
YLR095C	IOC2	YGL147C	RPL9A
YKR020W	VPS51	YPL213W	LEA1
YNL032W	SIW14	YMR311C	GLC8
YOR358W	HAP5	YDR318W	MCM21

Predictions at threshold level 0.75 continued...			
Gene1 of predicted pair		Gene2 of predicted pair	
Systematic Name	Standard Name	Systematic Name	Standard Name
YGR249W	MGA1	YPL195W	APL5
YBR274W	CHK1	YJR032W	CPR7
YLR240W	VPS34	YCR088W	ABP1
YJL106W	IME2	YJR094C	IME1
YDR378C	LSM6	YMR038C	CCS1
YGL223C	COG1	YDL006W	PTC1
YOR080W	DIA2	YJR060W	CBF1
YHR156C	LIN1	YLR180W	SAM1
YLR386W	VAC14	YDR358W	GGA1
YOR038C	HIR2	YGL148W	ARO2
YGL033W	HOP2	YNL041C	COG6
YER162C	RAD4	YER122C	GLO3
YJL006C	CTK2	YNL116W	DMA2
YOR080W	DIA2	YLR370C	ARC18
YGR019W	UGA1	YBR108W	YBR108W
YHR081W	LRP1	YER122C	GLO3
YOR005C	DNL4	YCR014C	POL4
YNL087W	TCB2	YOR290C	SNF2
YPR084W	YPR084W	YCL037C	SRO9
YLR079W	SIC1	YML112W	CTK3
YBL102W	SFT2	YAL002W	VPS8
YDR129C	SAC6	YBR171W	SEC66
YLR079W	SIC1	YMR179W	SPT21
YNL206C	RTT106	YOR275C	RIM20
YDL244W	THI13	YPL240C	HSP82
YML095C	RAD10	YHR010W	RPL27A
YLR090W	XDJ1	YBR030W	YBR030W
YBR217W	ATG12	YJL095W	BCK1
YML115C	VAN1	YER040W	GLN3

Predictions at threshold level 0.75 continued...			
Gene1 of predicted pair		Gene2 of predicted pair	
Systematic Name	Standard Name	Systematic Name	Standard Name
YLR360W	VPS38	YDR254W	CHL4
YNL094W	APP1	YKR030W	GMH1
YDR363W-A	SEM1	YAL002W	VPS8
YBR131W	CCZ1	YPL089C	RLM1
YKR061W	KTR2	YJR135C	MCM22
YFR024C-A	LSB3	YKR030W	GMH1
YPL024W	RMI1	YGL163C	RAD54
YER019C-A	SBH2	YDL006W	PTC1
YPL096C-A	ERI1	YJL095W	BCK1
YMR172W	HOT1	YOL067C	RTG1
YJL176C	SWI3	YBL072C	RPS8A
YML038C	YMD8	YDL186W	YDL186W
YMR179W	SPT21	YJR135C	MCM22
YBR107C	IML3	YAL002W	VPS8
YLR386W	VAC14	YAL002W	VPS8
YGL147C	RPL9A	YER044C	ERG28
YEL044W	IES6	YBL072C	RPS8A
YNL058C	YNL058C	YER139C	YER139C
YPL022W	RAD1	YLR410W	VIP1
YJR024C	MDE1	YJR059W	PTK2
YGL078C	DBP3	YMR133W	REC114
YLR320W	MMS22	YPR030W	CSR2
YDR004W	RAD57	YJL124C	LSM1
YMR224C	MRE11	YNL307C	MCK1
YLR079W	SIC1	YDL047W	SIT4
YBR217W	ATG12	YER122C	GLO3
YNL166C	BNI5	YPL018W	CTF19
YBR200W	BEM1	YMR230W	RPS10B
YMR054W	STV1	YPL120W	VPS30

Predictions at threshold level 0.75 continued...			
Gene1 of predicted pair		Gene2 of predicted pair	
Systematic Name	Standard Name	Systematic Name	Standard Name
YGL212W	VAM7	YER162C	RAD4
YGR249W	MGA1	YDR477W	SNF1
YDR477W	SNF1	YEL053C	MAK10
YDR395W	SXM1	YBL079W	NUP170
YGL161C	YIP5	YDR310C	SUM1
YDR254W	CHL4	YOL067C	RTG1
YBR131W	CCZ1	YDL192W	ARF1
YMR202W	ERG2	YDR507C	GIN4
YDR289C	RTT103	YDR155C	CPR1
YLR090W	XDJ1	YBR217W	ATG12
YNL116W	DMA2	YGL252C	RTG2
YDR137W	RGP1	YEL013W	VAC8
YBL017C	PEP1	YKR020W	VPS51
YGL135W	RPL1B	YLR360W	VPS38
YLR410W	VIP1	YFR036W	CDC26
YML115C	VAN1	YBR041W	FAT1
YBR171W	SEC66	YEL053C	MAK10
YGL060W	YBP2	YJL006C	CTK2
YPL042C	SSN3	YJL089W	SIP4
YLR399C	BDF1	YOR141C	ARP8
YGL250W	RMR1	YDR310C	SUM1
YDL184C	RPL41A	YPR036W	VMA13
YOR035C	SHE4	YBL072C	RPS8A
YOR337W	TEA1	YDR261C	EXG2
YGR105W	VMA21	YKL080W	VMA5
YDL020C	RPN4	YIL008W	URM1
YNL307C	MCK1	YGR135W	PRE9
YBR200W	BEM1	YLR425W	TUS1
YLR447C	VMA6	YPL234C	TFP3

Predictions at threshold level 0.75 continued...			
Gene1 of predicted pair		Gene2 of predicted pair	
Systematic Name	Standard Name	Systematic Name	Standard Name
YDR309C	GIC2	YNL116W	DMA2
YNL243W	SLA2	YNL020C	ARK1
YGR072W	UPF3	YDR153C	ENT5
YKL116C	PRR1	YER162C	RAD4
YDL116W	NUP84	YKL117W	SBA1
YEL009C	GCN4	YOR298C-A	MBF1
YOR127W	RGA1	YOL018C	TLG2
YOR337W	TEA1	YLR370C	ARC18
YLR370C	ARC18	YPR119W	CLB2
YBR215W	HPC2	YDR440W	DOT1
YPL045W	VPS16	YGL198W	YIP4
YKR072C	SIS2	YOR080W	DIA2
YGR108W	CLB1	YEL056W	HAT2
YOR132W	VPS17	YJR058C	APS2
YDR440W	DOT1	YDR155C	CPR1
YOR358W	HAP5	YGL253W	HXK2
YPR046W	MCM16	YOR035C	SHE4
YDL116W	NUP84	YJL006C	CTK2
YOR069W	VPS5	YDL006W	PTC1
YGL223C	COG1	YML001W	YPT7
YBR217W	ATG12	YPL137C	GIP3
YPL057C	SUR1	YHR013C	ARD1
YNL199C	GCR2	YKL068W	NUP100
YGR217W	CCH1	YGR135W	PRE9
YFR010W	UBP6	YNL117W	MLS1
YPL042C	SSN3	YDR289C	RTT103
YGR270W	YTA7	YHR115C	DMA1
YBR194W	SOY1	YLR058C	SHM2
YOR270C	VPH1	YDL192W	ARF1

Predictions at threshold level 0.75 continued...			
Gene1 of predicted pair		Gene2 of predicted pair	
Systematic Name	Standard Name	Systematic Name	Standard Name
YFL023W	BUD27	YPR036W	VMA13
YGL241W	KAP114	YBR010W	HHT1
YKL101W	HSL1	YGR188C	BUB1
YBL087C	RPL23A	YHR115C	DMA1
YAR014C	BUD14	YER122C	GLO3
YPL161C	BEM4	YDR289C	RTT103
YNL259C	ATX1	YDR270W	CCC2
YML095C	RAD10	YJR090C	GRR1
YOR001W	RRP6	YGL174W	BUD13
YPL253C	VIK1	YPL018W	CTF19
YBR217W	ATG12	YNL076W	MKS1
YER110C	KAP123	YPR051W	MAK3
YMR055C	BUB2	YOR043W	WHI2
YJR084W	CSN12	YER040W	GLN3
YMR224C	MRE11	YHR013C	ARD1
YBL058W	SHP1	YER162C	RAD4
YCR065W	HCM1	YGL031C	RPL24A
YBR108W	YBR108W	YOL111C	MDY2
YER120W	SCS2	YDR310C	SUM1
YLR320W	MMS22	YHR039C-A	VMA10
YGR135W	PRE9	YDR318W	MCM21
YML001W	YPT7	YNL107W	YAF9
YIL076W	SEC28	YLR441C	RPS1A
YLR337C	VRP1	YLR234W	TOP3
YBR228W	SLX1	YCL037C	SRO9
YGR249W	MGA1	YER139C	YER139C
YOR033C	EXO1	YGL175C	SAE2
YIL053W	RHR2	YMR133W	REC114
YNL307C	MCK1	YFR030W	MET10

Predictions at threshold level 0.75 continued...			
Gene1 of predicted pair		Gene2 of predicted pair	
Systematic Name	Standard Name	Systematic Name	Standard Name
YCR086W	CSM1	YBR036C	CSG2
YLR337C	VRP1	YBR114W	RAD16
YPL057C	SUR1	YOR038C	HIR2
YML115C	VAN1	YOL081W	IRA2
YGL095C	VPS45	YAL030W	SNC1
YBR057C	MUM2	YLR032W	RAD5
YJL187C	SWE1	YDR207C	UME6
YGR072W	UPF3	YLR135W	SLX4
YGR270W	YTA7	YCR077C	PAT1
YOR127W	RGA1	YMR054W	STV1
YDR080W	VPS41	YJR033C	RAV1
YIL159W	BNR1	YJR056C	YJR056C
YGL078C	DBP3	YJR056C	YJR056C
YOR145C	PNO1	YOR078W	BUD21
YKR001C	VPS1	YOR368W	RAD17
YDL116W	NUP84	YDR156W	RPA14
YLR399C	BDF1	YNL248C	RPA49
YML103C	NUP188	YKL110C	KTI12
YMR198W	CIK1	YAL002W	VPS8
YPR101W	SNT309	YJR050W	ISY1
YKR072C	SIS2	YMR038C	CCS1
YDR225W	HTA1	YMR153W	NUP53
YLR234W	TOP3	YML016C	PPZ1
YIL008W	URM1	YGL163C	RAD54
YOR069W	VPS5	YDR310C	SUM1
YNL291C	MID1	YPL161C	BEM4
YMR224C	MRE11	YKR001C	VPS1
YLR452C	SST2	YER122C	GLO3
YDR176W	NGG1	YBL005W	PDR3

Predictions at threshold level 0.75 continued...			
Gene1 of predicted pair		Gene2 of predicted pair	
Systematic Name	Standard Name	Systematic Name	Standard Name
YGL147C	RPL9A	YDL083C	RPS16B
YFR010W	UBP6	YBR200W	BEM1
YBL019W	APN2	YMR055C	BUB2
YGR261C	APL6	YER162C	RAD4
YDR484W	VPS52	YJR084W	CSN12
YDR097C	MSH6	YGL100W	SEH1
YMR224C	MRE11	YMR116C	ASC1
YBR030W	YBR030W	YJR084W	CSN12
YNL329C	PEX6	YKL041W	VPS24
YPL105C	YPL105C	YKR030W	GMH1
YGR261C	APL6	YLR309C	IMH1
YDR335W	MSN5	YMR055C	BUB2
YAL020C	ATS1	YKL048C	ELM1
YJL154C	VPS35	YIL008W	URM1
YLR094C	GIS3	YJL095W	BCK1
YDR202C	RAV2	YDL192W	ARF1
YPL105C	YPL105C	YKR020W	VPS51
YKL139W	CTK1	YDR310C	SUM1
YBR290W	BSD2	YJL095W	BCK1
YKR072C	SIS2	YHR010W	RPL27A
YOR132W	VPS17	YJL190C	RPS22A
YNL016W	PUB1	YMR038C	CCS1
YNL025C	SSN8	YNL236W	SIN4
YKL032C	IXR1	YDR310C	SUM1
YFL023W	BUD27	YGL124C	MON1
YGR129W	SYF2	YKL074C	MUD2
YKL007W	CAP1	YLR216C	CPR6
YNL248C	RPA49	YDR289C	RTT103
YGL253W	HXK2	YAL002W	VPS8

Predictions at threshold level 0.75 continued...			
Gene1 of predicted pair		Gene2 of predicted pair	
Systematic Name	Standard Name	Systematic Name	Standard Name
YDR477W	SNF1	YDR456W	NHX1
YPL050C	MNN9	YNL041C	COG6
YJR135C	MCM22	YOR014W	RTS1
YGR072W	UPF3	YBL102W	SFT2
YOR069W	VPS5	YCR066W	RAD18
YMR142C	RPL13B	YHR081W	LRP1
YJL124C	LSM1	YDR289C	RTT103
YNL135C	FPR1	YBL103C	RTG3
YOR156C	NFI1	YPL057C	SUR1
YML095C	RAD10	YGL031C	RPL24A
YMR153W	NUP53	YBL103C	RTG3
YNL307C	MCK1	YJL095W	BCK1
YBL102W	SFT2	YDR207C	UME6
YLR234W	TOP3	YGL019W	CKB1
YPL246C	RBD2	YHR016C	YSC84
YLR399C	BDF1	YNL107W	YAF9
YNL215W	IES2	YGL163C	RAD54
YCR034W	FEN1	YML016C	PPZ1
YBR173C	UMP1	YHR081W	LRP1
YPL050C	MNN9	YMR109W	MYO5
YML071C	COG8	YER139C	YER139C
YDR165W	TRM82	YDL201W	TRM8
YGL135W	RPL1B	YML115C	VAN1
YHR060W	VMA22	YPL234C	TFP3
YDR137W	RGP1	YOR124C	UBP2
YMR224C	MRE11	YLR288C	MEC3
YKL025C	PAN3	YEL053C	MAK10
YGR072W	UPF3	YOR327C	SNC2
YKR099W	BAS1	YKL048C	ELM1

Predictions at threshold level 0.75 continued...			
Gene1 of predicted pair		Gene2 of predicted pair	
Systematic Name	Standard Name	Systematic Name	Standard Name
YLR192C	HCR1	YCR053W	THR4
YCR020C-A	MAK31	YMR054W	STV1
YER162C	RAD4	YLR180W	SAM1
YJR135C	MCM22	YHR114W	BZZ1
YLR119W	SRN2	YNL032W	SIW14
YDR309C	GIC2	YDR227W	SIR4
YLR083C	EMP70	YER122C	GLO3
YMR142C	RPL13B	YDL077C	VAM6
YPL240C	HSP82	YJL103C	GSM1
YBL087C	RPL23A	YNL116W	DMA2
YJR140C	HIR3	YCR077C	PAT1
YKL130C	SHE2	YKL185W	ASH1
YDL213C	NOP6	YOR078W	BUD21
YBR200W	BEM1	YMR109W	MYO5
YML016C	PPZ1	YPL256C	CLN2
YKL048C	ELM1	YHR060W	VMA22
YDR155C	CPR1	YPL018W	CTF19
YPL129W	TAF14	YLR176C	RFX1
YDR320C	SWA2	YDL226C	GCS1
YLR095C	IOC2	YOR270C	VPH1
YGR270W	YTA7	YKL160W	ELF1
YIL159W	BNR1	YEL053C	MAK10
YJR059W	PTK2	YMR164C	MSS11
YPL195W	APL5	YEL013W	VAC8
YLR386W	VAC14	YKR035W-A	DID2
YDR080W	VPS41	YGR105W	VMA21
YGL054C	ERV14	YGR105W	VMA21
YDR260C	SWM1	YPL018W	CTF19
YBL007C	SLA1	YGL095C	VPS45

Predictions at threshold level 0.75 continued...			
Gene1 of predicted pair		Gene2 of predicted pair	
Systematic Name	Standard Name	Systematic Name	Standard Name
YML095C	RAD10	YLR352W	YLR352W
YBR200W	BEM1	YBR171W	SEC66
YGL087C	MMS2	YNL166C	BNI5
YFL023W	BUD27	YPL137C	GIP3
YLR313C	SPH1	YBR082C	UBC4
YBR138C	YBR138C	YGR223C	HSV2
YKR048C	NAP1	YLR425W	TUS1
YCR077C	PAT1	YGR152C	RSR1
YBR127C	VMA2	YMR054W	STV1
YPL256C	CLN2	YKL101W	HSL1
YAL024C	LTE1	YHR200W	RPN10
YOR290C	SNF2	YML071C	COG8
YMR133W	REC114	YBL025W	RRN10
YJR135C	MCM22	YDR289C	RTT103
YNL032W	SIW14	YHR200W	RPN10
YBR215W	HPC2	YCR086W	CSM1
YPR018W	RLF2	YMR038C	CCS1
YDR202C	RAV2	YDR207C	UME6
YER162C	RAD4	YLR093C	NYV1
YGL223C	COG1	YEL036C	ANP1
YHR013C	ARD1	YAR014C	BUD14
YKL126W	YPK1	YOL121C	RPS19A
YNL307C	MCK1	YEL053C	MAK10
YAL024C	LTE1	YOL111C	MDY2
YNL325C	FIG4	YPL057C	SUR1
YGL087C	MMS2	YAR014C	BUD14
YLR386W	VAC14	YNL076W	MKS1
YGL066W	SGF73	YDR254W	CHL4
YGL115W	SNF4	YHR081W	LRP1

Predictions at threshold level 0.75 continued...			
**Gene1 of predicted pair**		**Gene2 of predicted pair**	
**Systematic Name**	**Standard Name**	**Systematic Name**	**Standard Name**
YGR152C	RSR1	YNL090W	RHO2
YLR386W	VAC14	YDR261C	EXG2
YDR358W	GGA1	YOR035C	SHE4
YEL053C	MAK10	YLR309C	IMH1
YLR090W	XDJ1	YDR358W	GGA1
YDR320C	SWA2	YML038C	YMD8
YHR134W	WSS1	YOR080W	DIA2
YNL004W	HRB1	YCL011C	GBP2
YDR363W-A	SEM1	YJR035W	RAD26
YBR107C	IML3	YDL155W	CLB3
YLR090W	XDJ1	YDR137W	RGP1
YOR368W	RAD17	YKL114C	APN1
YDR004W	RAD57	YBR195C	MSI1
YKL025C	PAN3	YIL034C	CAP2
YJR135C	MCM22	YGR135W	PRE9
YPR051W	MAK3	YJR135C	MCM22
YOR069W	VPS5	YOR106W	VAM3
YOR195W	SLK19	YJR005W	APL1
YOL111C	MDY2	YJR084W	CSN12
YGL237C	HAP2	YJL190C	RPS22A
YJR140C	HIR3	YMR055C	BUB2
YNL206C	RTT106	YOR141C	ARP8
YKL160W	ELF1	YHR167W	THP2
YER116C	SLX8	YAL009W	SPO7
YJL036W	SNX4	YPR119W	CLB2
YGL161C	YIP5	YNL107W	YAF9
YGL033W	HOP2	YER040W	GLN3
YEL053C	MAK10	YJL004C	SYS1
YHR152W	SPO12	YDL047W	SIT4

Predictions at threshold level 0.75 continued...			
Gene1 of predicted pair		Gene2 of predicted pair	
Systematic Name	Standard Name	Systematic Name	Standard Name
YGR239C	PEX21	YGL153W	PEX14
YDR293C	SSD1	YPR120C	CLB5
YOR337W	TEA1	YHR039C-A	VMA10
YHR200W	RPN10	YPL137C	GIP3
YLR452C	SST2	YPR046W	MCM16
YBL051C	PIN4	YML016C	PPZ1
YLR192C	HCR1	YLR441C	RPS1A
YGL066W	SGF73	YDR379W	RGA2
YIL053W	RHR2	YMR230W	RPS10B
YMR116C	ASC1	YBL079W	NUP170
YPR084W	YPR084W	YPL057C	SUR1
YJL124C	LSM1	YGL031C	RPL24A
YMR127C	SAS2	YDR183W	PLP1
YLR095C	IOC2	YNL307C	MCK1
YMR224C	MRE11	YEL053C	MAK10
YPL024W	RMI1	YPL001W	HAT1
YOR106W	VAM3	YDL006W	PTC1
YHL031C	GOS1	YDR080W	VPS41
YOR270C	VPH1	YML071C	COG8
YDR260C	SWM1	YKL101W	HSL1
YGR133W	PEX4	YML071C	COG8
YGL151W	NUT1	YAL002W	VPS8
YLR119W	SRN2	YEL060C	PRB1
YGL147C	RPL9A	YBR171W	SEC66
YDR293C	SSD1	YNL238W	KEX2
YPL137C	GIP3	YDR207C	UME6
YIL034C	CAP2	YAL002W	VPS8
YFR036W	CDC26	YER122C	GLO3
YGL087C	MMS2	YMR109W	MYO5

Predictions at threshold level 0.75 continued...			
Gene1 of predicted pair		Gene2 of predicted pair	
Systematic Name	Standard Name	Systematic Name	Standard Name
YCL037C	SRO9	YNR032W	PPG1
YBR127C	VMA2	YEL027W	CUP5
YMR055C	BUB2	YHR012W	VPS29
YLR386W	VAC14	YOR023C	AHC1
YGR108W	CLB1	YDR363W-A	SEM1
YNL054W	VAC7	YHR115C	DMA1
YHR200W	RPN10	YJR084W	CSN12
YML001W	YPT7	YHR012W	VPS29
YGL124C	MON1	YGR188C	BUB1
YLL042C	ATG10	YPL149W	ATG5
YBL047C	EDE1	YDL047W	SIT4
YBR260C	RGD1	YOR014W	RTS1
YLR192C	HCR1	YKL139W	CTK1
YIR009W	MSL1	YGR188C	BUB1
YKR057W	RPS21A	YBL087C	RPL23A
YNL243W	SLA2	YMR294W	JNM1
YNL288W	CAF40	YIL008W	URM1
YCR086W	CSM1	YDR289C	RTT103
YLR102C	APC9	YKR048C	NAP1
YPL120W	VPS30	YKR030W	GMH1
YJR032W	CPR7	YGR188C	BUB1
YKL181W	PRS1	YPR119W	CLB2
YDR225W	HTA1	YGL163C	RAD54
YPR135W	CTF4	YBR089C-A	NHP6B
YEL044W	IES6	YGL212W	VAM7
YFR010W	UBP6	YDR076W	RAD55
YNL297C	MON2	YAL002W	VPS8
YOR290C	SNF2	YFR034C	PHO4
YLR360W	VPS38	YER120W	SCS2

Predictions at threshold level 0.75 continued...			
Gene1 of predicted pair		Gene2 of predicted pair	
Systematic Name	Standard Name	Systematic Name	Standard Name
YDR253C	MET32	YGR285C	ZUO1
YNL058C	YNL058C	YER040W	GLN3
YIL053W	RHR2	YLR320W	MMS22
YMR026C	PEX12	YOR270C	VPH1
YGL087C	MMS2	YDR310C	SUM1
YOR106W	VAM3	YHR039C-A	VMA10
YJR059W	PTK2	YKL117W	SBA1
YLR150W	STM1	YHR013C	ARD1
YPL022W	RAD1	YDR318W	MCM21
YGR104C	SRB5	YDR440W	DOT1
YHR060W	VMA22	YHR081W	LRP1
YLR095C	IOC2	YDL223C	HBT1
YPL195W	APL5	YCR053W	THR4
YIR005W	IST3	YPR046W	MCM16
YJL036W	SNX4	YAL030W	SNC1
YPL042C	SSN3	YOL091W	SPO21
YMR109W	MYO5	YKL213C	DOA1
YDR092W	UBC13	YLR032W	RAD5
YMR153W	NUP53	YNL238W	KEX2
YKL161C	YKL161C	YLR425W	TUS1
YJL124C	LSM1	YOR312C	RPL20B
YER001W	MNN1	YKL041W	VPS24
YIL159W	BNR1	YMR026C	PEX12
YDR379W	RGA2	YBL087C	RPL23A
YBR228W	SLX1	YBR073W	RDH54
YLR119W	SRN2	YPL037C	EGD1
YCR020C-A	MAK31	YPL161C	BEM4
YMR273C	ZDS1	YHR061C	GIC1
YFR036W	CDC26	YOR327C	SNC2

Predictions at threshold level 0.75 continued...			
Gene1 of predicted pair		Gene2 of predicted pair	
Systematic Name	Standard Name	Systematic Name	Standard Name
YGL153W	PEX14	YGL200C	EMP24
YCL037C	SRO9	YEL051W	VMA8
YBL047C	EDE1	YLR182W	SWI6
YMR153W	NUP53	YNL076W	MKS1
YPL234C	TFP3	YOR327C	SNC2
YDL184C	RPL41A	YPR046W	MCM16
YHR178W	STB5	YOR038C	HIR2
YGL163C	RAD54	YDR076W	RAD55
YKR048C	NAP1	YNL116W	DMA2
YAL024C	LTE1	YOR368W	RAD17
YBR030W	YBR030W	YOR327C	SNC2
YOR132W	VPS17	YMR054W	STV1
YGR159C	NSR1	YGL200C	EMP24
YNL031C	HHT2	YDR071C	PAA1
YBL103C	RTG3	YNL058C	YNL058C
YPL161C	BEM4	YBL087C	RPL23A
YER116C	SLX8	YKR035W-A	DID2
YPR017C	DSS4	YEL051W	VMA8
YAL024C	LTE1	YEL060C	PRB1
YDR144C	MKC7	YBR114W	RAD16
YMR198W	CIK1	YJL127C	SPT10
YER087C-B	SBH1	YBR279W	PAF1
YER044C	ERG28	YMR311C	GLC8
YBR107C	IML3	YOL081W	IRA2
YGL124C	MON1	YEL060C	PRB1
YAL024C	LTE1	YKL048C	ELM1
YCR097W	HMRA1	YCR084C	TUP1
YPL037C	EGD1	YNR072W	HXT17
YNL243W	SLA2	YPL024W	RMI1

Predictions at threshold level 0.75 continued...			
Gene1 of predicted pair		Gene2 of predicted pair	
Systematic Name	Standard Name	Systematic Name	Standard Name
YGL033W	HOP2	YLR192C	HCR1
YDR123C	INO2	YPL248C	GAL4
YAL024C	LTE1	YJR063W	RPA12
YBL008W	HIR1	YDR378C	LSM6
YPL050C	MNN9	YNL079C	TPM1
YDR142C	PEX7	YDL065C	PEX19
YLR095C	IOC2	YDR207C	UME6
YGL175C	SAE2	YJR035W	RAD26
YER110C	KAP123	YGL124C	MON1
YKL139W	CTK1	YLR320W	MMS22
YJR035W	RAD26	YNL107W	YAF9
YOR112W	CEX1	YOL018C	TLG2
YKL025C	PAN3	YOR132W	VPS17
YLR119W	SRN2	YJR032W	CPR7
YAL024C	LTE1	YPL195W	APL5
YBR278W	DPB3	YOR038C	HIR2
YDR244W	PEX5	YOL044W	PEX15
YIL034C	CAP2	YHL002W	HSE1
YBR171W	SEC66	YDR207C	UME6
YCL010C	SGF29	YGR252W	GCN5
YMR055C	BUB2	YDR183W	PLP1
YNR072W	HXT17	YDR358W	GGA1
YGR249W	MGA1	YCR053W	THR4
YPL022W	RAD1	YGL031C	RPL24A
YDR440W	DOT1	YKL041W	VPS24
YDL155W	CLB3	YLR131C	ACE2
YHR161C	YAP1801	YOR327C	SNC2
YPL256C	CLN2	YNL116W	DMA2
YPL042C	SSN3	YDR318W	MCM21

Predictions at threshold level 0.75 continued...			
Gene1 of predicted pair		Gene2 of predicted pair	
Systematic Name	Standard Name	Systematic Name	Standard Name
YDR289C	RTT103	YOR368W	RAD17
YDL116W	NUP84	YML112W	CTK3
YBR200W	BEM1	YMR032W	HOF1
YDR254W	CHL4	YLR032W	RAD5
YJR084W	CSN12	YOR368W	RAD17
YDL116W	NUP84	YPL178W	CBC2
YPL161C	BEM4	YHL002W	HSE1
YGR105W	VMA21	YAL002W	VPS8
YDR101C	ARX1	YKL068W	NUP100
YDL160C	DHH1	YML095C	RAD10
YPL018W	CTF19	YJR035W	RAD26
YIL008W	URM1	YPL018W	CTF19
YBR171W	SEC66	YGR188C	BUB1
YBR215W	HPC2	YDR200C	VPS64
YPR017C	DSS4	YOL018C	TLG2
YDR105C	TMS1	YFL044C	OTU1
YKL048C	ELM1	YJL157C	FAR1
YPL115C	BEM3	YDL155W	CLB3
YHR185C	PFS1	YIL007C	NAS2
YDL005C	MED2	YCR084C	TUP1
YDR293C	SSD1	YDR183W	PLP1
YLR360W	VPS38	YOR036W	PEP12
YPR084W	YPR084W	YKL213C	DOA1
YJR084W	CSN12	YMR214W	SCJ1
YER116C	SLX8	YLR150W	STM1
YJL176C	SWI3	YKL041W	VPS24
YJR090C	GRR1	YER027C	GAL83
YDR254W	CHL4	YEL056W	HAT2
YFL003C	MSH4	YLR394W	CST9

Predictions at threshold level 0.75 continued...			
Gene1 of predicted pair		Gene2 of predicted pair	
Systematic Name	Standard Name	Systematic Name	Standard Name
YOL121C	RPS19A	YLR093C	NYV1
YGR019W	UGA1	YHR178W	STB5
YOR132W	VPS17	YKR030W	GMH1
YFR031C-A	RPL2A	YPR017C	DSS4
YOR001W	RRP6	YLR429W	CRN1
YCR086W	CSM1	YGR188C	BUB1
YPL022W	RAD1	YKR030W	GMH1
YMR224C	MRE11	YNL116W	DMA2
YNL116W	DMA2	YHR115C	DMA1
YJR024C	MDE1	YOR358W	HAP5
YOR106W	VAM3	YNL079C	TPM1
YPL195W	APL5	YMR214W	SCJ1
YOL090W	MSH2	YGL031C	RPL24A
YER087C-B	SBH1	YBR200W	BEM1
YDL184C	RPL41A	YNL307C	MCK1
YER116C	SLX8	YPL161C	BEM4
YCR002C	CDC10	YDR507C	GIN4
YMR004W	MVP1	YER162C	RAD4
YDL160C	DHH1	YDR253C	MET32
YER044C	ERG28	YDL077C	VAM6
YMR153W	NUP53	YHR200W	RPN10
YGL124C	MON1	YMR230W	RPS10B
YPL120W	VPS30	YDR207C	UME6
YLR292C	SEC72	YML095C	RAD10
YJR084W	CSN12	YDL077C	VAM6
YHR060W	VMA22	YER120W	SCS2
YOL147C	PEX11	YNL117W	MLS1
YDL223C	HBT1	YOR035C	SHE4
YDR245W	MNN10	YKL126W	YPK1

Predictions at threshold level 0.75 continued...			
**Gene1 of predicted pair**		**Gene2 of predicted pair**	
**Systematic Name**	**Standard Name**	**Systematic Name**	**Standard Name**
YDR358W	GGA1	YBR280C	SAF1
YGL078C	DBP3	YGL003C	CDH1
YLR181C	VTA1	YKR035W-A	DID2
YNL032W	SIW14	YLR398C	SKI2
YHR205W	SCH9	YDR388W	RVS167
YPR167C	MET16	YML028W	TSA1
YGR217W	CCH1	YKR020W	VPS51
YOR035C	SHE4	YKL041W	VPS24
YPR052C	NHP6A	YGR275W	RTT102
YJL190C	RPS22A	YGR188C	BUB1
YKL032C	IXR1	YDR440W	DOT1
YDR156W	RPA14	YNR052C	POP2
YMR224C	MRE11	YJL172W	CPS1
YLR079W	SIC1	YBL047C	EDE1
YDR153C	ENT5	YDR207C	UME6
YDL006W	PTC1	YEL051W	VMA8
YJL127C	SPT10	YLR429W	CRN1
YPL152W	RRD2	YJR135C	MCM22
YDL116W	NUP84	YJR063W	RPA12
YMR153W	NUP53	YMR016C	SOK2
YOR290C	SNF2	YJR032W	CPR7
YOR083W	WHI5	YJR135C	MCM22
YNL087W	TCB2	YDR137W	RGP1
YJR106W	ECM27	YKR093W	PTR2
YGL190C	CDC55	YJL187C	SWE1
YBR200W	BEM1	YNL307C	MCK1
YKL139W	CTK1	YGL212W	VAM7
YBR093C	PHO5	YOR368W	RAD17
YGL212W	VAM7	YCR053W	THR4

Predictions at threshold level 0.75 continued...			
Gene1 of predicted pair		Gene2 of predicted pair	
Systematic Name	Standard Name	Systematic Name	Standard Name
YLR399C	BDF1	YPL152W	RRD2
YNL291C	MID1	YJL095W	BCK1
YCR086W	CSM1	YNL116W	DMA2
YMR016C	SOK2	YJL183W	MNN11
YLR309C	IMH1	YKR030W	GMH1
YHR111W	UBA4	YOR275C	RIM20
YMR172W	HOT1	YAL002W	VPS8
YCL037C	SRO9	YJL004C	SYS1
YER120W	SCS2	YEL051W	VMA8
YFR010W	UBP6	YDR245W	MNN10
YGL060W	YBP2	YML095C	RAD10
YEL043W	YEL043W	YEL051W	VMA8
YOR298C-A	MBF1	YPR119W	CLB2
YLR079W	SIC1	YAR014C	BUD14
YLR337C	VRP1	YPR051W	MAK3
YJL006C	CTK2	YLR425W	TUS1
YGR104C	SRB5	YJL124C	LSM1
YLR191W	PEX13	YDR244W	PEX5
YGL060W	YBP2	YJR005W	APL1
YBL067C	UBP13	YOR035C	SHE4
YOR023C	AHC1	YJR135C	MCM22
YGR270W	YTA7	YHR012W	VPS29
YHR016C	YSC84	YPL249C	GYP5
YMR133W	REC114	YDL083C	RPS16B
YPL129W	TAF14	YHL025W	SNF6
YDR378C	LSM6	YKL048C	ELM1
YMR311C	GLC8	YCR053W	THR4
YJR005W	APL1	YPL018W	CTF19
YMR055C	BUB2	YGL019W	CKB1

Predictions at threshold level 0.75 continued...			
Gene1 of predicted pair		Gene2 of predicted pair	
Systematic Name	Standard Name	Systematic Name	Standard Name
YEL065W	SIT1	YJR032W	CPR7
YMR153W	NUP53	YOR043W	WHI2
YBR215W	HPC2	YML102W	CAC2
YGR241C	YAP1802	YBL047C	EDE1
YFL023W	BUD27	YDR207C	UME6
YNR052C	POP2	YIL036W	CST6
YHR060W	VMA22	YDL006W	PTC1
YGL087C	MMS2	YDR363W-A	SEM1
YOL145C	CTR9	YNL107W	YAF9
YNL243W	SLA2	YNL098C	RAS2
YKL006W	RPL14A	YOL081W	IRA2
YDR335W	MSN5	YOL054W	PSH1
YGR252W	GCN5	YDR469W	SDC1
YPR018W	RLF2	YDL005C	MED2
YMR198W	CIK1	YML097C	VPS9
YGL078C	DBP3	YPL137C	GIP3
YDR004W	RAD57	YPR036W	VMA13
YKL025C	PAN3	YDR318W	MCM21
YOR337W	TEA1	YGL212W	VAM7
YBR200W	BEM1	YBR114W	RAD16
YDR156W	RPA14	YNL054W	VAC7
YOR043W	WHI2	YFR036W	CDC26
YBR108W	YBR108W	YIL008W	URM1
YHR111W	UBA4	YJR090C	GRR1
YKR061W	KTR2	YJL006C	CTK2
YKL135C	APL2	YJR058C	APS2
YNR064C	YNR064C	YOL108C	INO4
YPL022W	RAD1	YMR231W	PEP5
YBR171W	SEC66	YDR335W	MSN5

Predictions at threshold level 0.75 continued...			
Gene1 of predicted pair		Gene2 of predicted pair	
Systematic Name	Standard Name	Systematic Name	Standard Name
YER044C	ERG28	YLR398C	SKI2
YNL087W	TCB2	YKR035W-A	DID2
YIL156W	UBP7	YMR164C	MSS11
YLR234W	TOP3	YAL015C	NTG1
YMR153W	NUP53	YDL083C	RPS16B
YNL058C	YNL058C	YML071C	COG8
YGL178W	MPT5	YAL040C	CLN3
YDR363W-A	SEM1	YLR410W	VIP1
YGL161C	YIP5	YKR001C	VPS1
YOR080W	DIA2	YDR289C	RTT103
YPL253C	VIK1	YKL101W	HSL1
YLR410W	VIP1	YGL031C	RPL24A
YKL032C	IXR1	YJL006C	CTK2
YMR026C	PEX12	YHR200W	RPN10
YBL008W	HIR1	YOR080W	DIA2
YHL031C	GOS1	YGR020C	VMA7
YGR252W	GCN5	YJL095W	BCK1
YDL002C	NHP10	YOR308C	SNU66
YLR360W	VPS38	YPL161C	BEM4
YMR224C	MRE11	YNL117W	MLS1
YGL133W	ITC1	YHR152W	SPO12
YOR001W	RRP6	YMR125W	STO1
YDR156W	RPA14	YMR055C	BUB2
YNL041C	COG6	YML071C	COG8
YPL037C	EGD1	YKR030W	GMH1
YLR337C	VRP1	YDR155C	CPR1
YIL036W	CST6	YHR115C	DMA1
YMR153W	NUP53	YLR180W	SAM1
YPL115C	BEM3	YPL018W	CTF19

Predictions at threshold level 0.75 continued...			
Gene1 of predicted pair		Gene2 of predicted pair	
Systematic Name	Standard Name	Systematic Name	Standard Name
YDR379W	RGA2	YJL187C	SWE1
YEL043W	YEL043W	YJL004C	SYS1
YLR371W	ROM2	YER049W	TPA1
YKR020W	VPS51	YAR014C	BUD14
YFL023W	BUD27	YDL077C	VAM6
YNL325C	FIG4	YKL048C	ELM1
YDR174W	HMO1	YEL027W	CUP5
YOR080W	DIA2	YNL016W	PUB1
YGR072W	UPF3	YNL041C	COG6
YPL161C	BEM4	YMR038C	CCS1
YDR101C	ARX1	YER110C	KAP123
YIR009W	MSL1	YCR077C	PAT1
YER110C	KAP123	YHR111W	UBA4
YOR290C	SNF2	YER044C	ERG28
YPL240C	HSP82	YGL096W	TOS8
YDL101C	DUN1	YOL030W	GAS5
YOR156C	NFI1	YLR357W	RSC2
YJL154C	VPS35	YOR140W	SFL1
YFR036W	CDC26	YMR038C	CCS1
YKL160W	ELF1	YCR077C	PAT1
YGL200C	EMP24	YOL081W	IRA2
YHR200W	RPN10	YGL115W	SNF4
YFL013C	IES1	YHL031C	GOS1
YDR363W-A	SEM1	YKL101W	HSL1
YHR114W	BZZ1	YHL002W	HSE1
YPL024W	RMI1	YDR137W	RGP1
YLR362W	STE11	YDL159W	STE7
YHL031C	GOS1	YDR245W	MNN10
YDR004W	RAD57	YML013W	UBX2

Predictions at threshold level 0.75 continued...			
Gene1 of predicted pair		Gene2 of predicted pair	
Systematic Name	Standard Name	Systematic Name	Standard Name
YPR120C	CLB5	YDL155W	CLB3
YMR198W	CIK1	YHL027W	RIM101
YER110C	KAP123	YLR399C	BDF1
YOR038C	HIR2	YLR182W	SWI6
YDR079C-A	TFB5	YGL009C	LEU1
YPR029C	APL4	YOR069W	VPS5
YLR094C	GIS3	YNL076W	MKS1
YKL048C	ELM1	YNL289W	PCL1
YPL112C	PEX25	YLR371W	ROM2
YIL076W	SEC28	YER161C	SPT2
YKL139W	CTK1	YBR215W	HPC2
YLR381W	CTF3	YEL053C	MAK10
YNL291C	MID1	YPL037C	EGD1
YMR091C	NPL6	YLR357W	RSC2
YMR080C	NAM7	YBL079W	NUP170
YOL013C	HRD1	YMR022W	UBC7
YLR234W	TOP3	YOR043W	WHI2
YPR131C	NAT3	YOR195W	SLK19
YKL006W	RPL14A	YJL183W	MNN11
YOR212W	STE4	YDR183W	PLP1
YOR337W	TEA1	YLR192C	HCR1
YJR021C	REC107	YMR133W	REC114
YDL020C	RPN4	YJR060W	CBF1
YOR106W	VAM3	YJR060W	CBF1
YDL002C	NHP10	YOR124C	UBP2
YIL034C	CAP2	YOR358W	HAP5
YMR153W	NUP53	YER115C	SPR6
YMR172W	HOT1	YBR108W	YBR108W
YLR234W	TOP3	YMR231W	PEP5

Predictions at threshold level 0.75 continued...			
Gene1 of predicted pair		Gene2 of predicted pair	
Systematic Name	Standard Name	Systematic Name	Standard Name
YCL032W	STE50	YCR086W	CSM1
YGR133W	PEX4	YKR020W	VPS51
YNL243W	SLA2	YNL250W	RAD50
YHR156C	LIN1	YDL192W	ARF1
YJL078C	PRY3	YPR149W	NCE102
YLR090W	XDJ1	YPR030W	CSR2
YJR056C	YJR056C	YPL137C	GIP3
YKL048C	ELM1	YHR012W	VPS29
YPL022W	RAD1	YDL226C	GCS1
YMR109W	MYO5	YGL151W	NUT1
YFR010W	UBP6	YDR335W	MSN5
YCL037C	SRO9	YPL120W	VPS30
YOL061W	PRS5	YOR043W	WHI2
YML013W	UBX2	YER162C	RAD4
YMR179W	SPT21	YDL047W	SIT4
YEL065W	SIT1	YPR046W	MCM16
YGL003C	CDH1	YLR370C	ARC18
YBR194W	SOY1	YCR077C	PAT1
YLR320W	MMS22	YJL183W	MNN11
YPL105C	YPL105C	YEL043W	YEL043W
YFR036W	CDC26	YBR036C	CSG2
YLR192C	HCR1	YKL126W	YPK1
YFR021W	ATG18	YPR119W	CLB2
YPL096C-A	ERI1	YEL036C	ANP1
YMR109W	MYO5	YAL002W	VPS8
YHR039C-A	VMA10	YDR153C	ENT5
YLR360W	VPS38	YPL018W	CTF19
YLR360W	VPS38	YOR008C	SLG1
YDR261C	EXG2	YFR036W	CDC26

Predictions at threshold level 0.75 continued...			
Gene1 of predicted pair		Gene2 of predicted pair	
Systematic Name	Standard Name	Systematic Name	Standard Name
YML071C	COG8	YJR084W	CSN12
YBL103C	RTG3	YER139C	YER139C
YNL106C	INP52	YNL054W	VAC7
YPR171W	BSP1	YKR030W	GMH1
YIL156W	UBP7	YJR059W	PTK2
YHR178W	STB5	YKL048C	ELM1
YGR054W	YGR054W	YHL002W	HSE1
YOR080W	DIA2	YCR077C	PAT1
YFR036W	CDC26	YDR440W	DOT1
YIL159W	BNR1	YLR191W	PEX13
YCL001W	RER1	YLR309C	IMH1
YML103C	NUP188	YGL163C	RAD54
YGL025C	PGD1	YNL307C	MCK1
YOR368W	RAD17	YDR207C	UME6
YJL187C	SWE1	YBR082C	UBC4
YOR127W	RGA1	YEL053C	MAK10
YOR106W	VAM3	YMR204C	INP1
YGL223C	COG1	YML067C	ERV41
YLL007C	YLL007C	YLR234W	TOP3
YBR107C	IML3	YIL008W	URM1
YLR192C	HCR1	YDR137W	RGP1
YDR225W	HTA1	YNL030W	HHF2
YBL078C	ATG8	YNR007C	ATG3
YDR477W	SNF1	YJR056C	YJR056C
YLR221C	RSA3	YKR024C	DBP7
YMR202W	ERG2	YJR135C	MCM22
YGR108W	CLB1	YMR109W	MYO5
YMR199W	CLN1	YDR264C	AKR1
YJL154C	VPS35	YBR200W	BEM1

Predictions at threshold level 0.75 continued...			
Gene1 of predicted pair		Gene2 of predicted pair	
Systematic Name	Standard Name	Systematic Name	Standard Name
YDR108W	GSG1	YJR135C	MCM22
YML117W	NAB6	YGR217W	CCH1
YJL124C	LSM1	YIL103W	DPH1
YJL138C	TIF2	YLR350W	ORM2
YFR024C-A	LSB3	YGR268C	HUA1
YMR016C	SOK2	YDR207C	UME6
YER087C-B	SBH1	YGL226C-A	OST5
YFR031C-A	RPL2A	YNR072W	HXT17
YMR016C	SOK2	YKR099W	BAS1
YPL018W	CTF19	YNL147W	LSM7
YNL206C	RTT106	YDR264C	AKR1
YNL106C	INP52	YHR108W	GGA2
YDR156W	RPA14	YML016C	PPZ1
YKL025C	PAN3	YGL237C	HAP2
YDR192C	NUP42	YDR207C	UME6
YDR484W	VPS52	YJR032W	CPR7
YMR142C	RPL13B	YDR137W	RGP1
YML038C	YMD8	YPL195W	APL5
YKL116C	PRR1	YLR371W	ROM2
YBR200W	BEM1	YIL008W	URM1
YPL022W	RAD1	YJL187C	SWE1
YDR392W	SPT3	YBR089C-A	NHP6B
YGL212W	VAM7	YKR020W	VPS51
YOR001W	RRP6	YML016C	PPZ1
YNL288W	CAF40	YMR109W	MYO5
YLR170C	APS1	YKL135C	APL2
YML103C	NUP188	YLR182W	SWI6
YBR171W	SEC66	YEL056W	HAT2
YMR199W	CLN1	YGL163C	RAD54

Predictions at threshold level 0.75 continued...			
Gene1 of predicted pair		Gene2 of predicted pair	
Systematic Name	Standard Name	Systematic Name	Standard Name
YLR386W	VAC14	YBR084C-A	RPL19A
YKL025C	PAN3	YPR046W	MCM16
YDL042C	SIR2	YDR227W	SIR4
YER116C	SLX8	YER044C	ERG28
YER114C	BOI2	YGR014W	MSB2
YEL065W	SIT1	YBR280C	SAF1
YPL057C	SUR1	YKL101W	HSL1
YNL288W	CAF40	YPL253C	VIK1
YOR106W	VAM3	YJR040W	GEF1
YLR398C	SKI2	YER040W	GLN3
YDR146C	SWI5	YBR215W	HPC2
YNL206C	RTT106	YDL185W	TFP1
YLR360W	VPS38	YOR069W	VPS5
YPL018W	CTF19	YGL252C	RTG2
YLR381W	CTF3	YNL236W	SIN4
YLR292C	SEC72	YHR134W	WSS1
YLR192C	HCR1	YHR060W	VMA22
YMR224C	MRE11	YJR084W	CSN12
YDL223C	HBT1	YNL041C	COG6
YFR010W	UBP6	YOR066W	MSA1
YLR441C	RPS1A	YOR035C	SHE4
YNL215W	IES2	YLR398C	SKI2
YJL154C	VPS35	YNL199C	GCR2
YJL172W	CPS1	YPL161C	BEM4
YDR137W	RGP1	YNL107W	YAF9
YCR038C	BUD5	YPR119W	CLB2
YHR111W	UBA4	YAL002W	VPS8
YLR144C	ACF2	YOR284W	HUA2
YJL172W	CPS1	YGL115W	SNF4

Predictions at threshold level 0.75 continued...			
Gene1 of predicted pair		Gene2 of predicted pair	
Systematic Name	Standard Name	Systematic Name	Standard Name
YOR140W	SFL1	YNL079C	TPM1
YDR130C	FIN1	YLR254C	NDL1
YOR290C	SNF2	YNL107W	YAF9
YDR439W	LRS4	YDR378C	LSM6
YJR031C	GEA1	YDL137W	ARF2
YGL087C	MMS2	YHR178W	STB5
YPL161C	BEM4	YHR012W	VPS29
YER144C	UBP5	YPR030W	CSR2
YMR026C	PEX12	YJR056C	YJR056C
YHR134W	WSS1	YBR200W	BEM1
YGL087C	MMS2	YGR188C	BUB1
YER110C	KAP123	YPL253C	VIK1
YMR224C	MRE11	YML095C	RAD10
YLR360W	VPS38	YMR231W	PEP5
YDL135C	RDI1	YLR324W	PEX30
YLR150W	STM1	YDR254W	CHL4
YOR106W	VAM3	YHL020C	OPI1
YDL223C	HBT1	YLR320W	MMS22
YOL111C	MDY2	YBR019C	GAL10
YGR129W	SYF2	YBR065C	ECM2
YOR337W	TEA1	YLR371W	ROM2
YPL018W	CTF19	YDL226C	GCS1
YDR484W	VPS52	YNL238W	KEX2
YHR200W	RPN10	YDL006W	PTC1
YNL166C	BNI5	YGR188C	BUB1
YBL103C	RTG3	YCR053W	THR4
YBR112C	CYC8	YPR068C	HOS1
YMR224C	MRE11	YPL022W	RAD1
YDR378C	LSM6	YPR120C	CLB5

Predictions at threshold level 0.75 continued...			
Gene1 of predicted pair		Gene2 of predicted pair	
Systematic Name	Standard Name	Systematic Name	Standard Name
YDL155W	CLB3	YKL101W	HSL1
YFR010W	UBP6	YCR086W	CSM1
YBR217W	ATG12	YLR180W	SAM1
YDL116W	NUP84	YPL152W	RRD2
YOR270C	VPH1	YKR099W	BAS1
YGL043W	DST1	YHR056C	RSC30
YBL058W	SHP1	YOL081W	IRA2
YNL253W	TEX1	YHR167W	THP2
YNL333W	SNZ2	YMR096W	SNZ1
YDL125C	HNT1	YNL236W	SIN4
YDR080W	VPS41	YPR173C	VPS4
YKR035W-A	DID2	YLR371W	ROM2
YEL050C	RML2	YGL003C	CDH1
YGL033W	HOP2	YDR484W	VPS52
YDL002C	NHP10	YHR200W	RPN10
YDL077C	VAM6	YLR398C	SKI2
YJR010C-A	SPC1	YOR085W	OST3
YNL199C	GCR2	YMR038C	CCS1
YML038C	YMD8	YKR030W	GMH1
YDR285W	ZIP1	YLR394W	CST9
YKL139W	CTK1	YLR254C	NDL1
YDR245W	MNN10	YDR289C	RTT103
YLR381W	CTF3	YKL048C	ELM1
YDR484W	VPS52	YHR200W	RPN10
YOR035C	SHE4	YBR280C	SAF1
YLR058C	SHM2	YGL003C	CDH1
YIL053W	RHR2	YGL033W	HOP2
YDR137W	RGP1	YGL200C	EMP24
YAL024C	LTE1	YNR052C	POP2

Predictions at threshold level 0.75 continued...			
Gene1 of predicted pair		Gene2 of predicted pair	
Systematic Name	Standard Name	Systematic Name	Standard Name
YKL116C	PRR1	YNL076W	MKS1
YHR128W	FUR1	YPL240C	HSP82
YDL192W	ARF1	YDR310C	SUM1
YLR083C	EMP70	YPL037C	EGD1
YJR135C	MCM22	YGL003C	CDH1
YPL057C	SUR1	YDR440W	DOT1
YNL215W	IES2	YGR014W	MSB2
YMR255W	GFD1	YIL033C	BCY1
YER114C	BOI2	YNL116W	DMA2
YMR199W	CLN1	YDR245W	MNN10
YER001W	MNN1	YEL044W	IES6
YKR001C	VPS1	YPL105C	YPL105C
YLL007C	YLL007C	YHR134W	WSS1
YPR017C	DSS4	YKR020W	VPS51
YHR152W	SPO12	YNL116W	DMA2
YHR084W	STE12	YLR362W	STE11
YHR060W	VMA22	YLR320W	MMS22
YBR215W	HPC2	YPL106C	SSE1
YHR039C-A	VMA10	YBL025W	RRN10
YNL058C	YNL058C	YNR072W	HXT17
YNL243W	SLA2	YNR051C	BRE5
YDR202C	RAV2	YNL044W	YIP3
YDR363W-A	SEM1	YLR182W	SWI6
YDR183W	PLP1	YLR182W	SWI6
YFR010W	UBP6	YLR192C	HCR1
YAL020C	ATS1	YLR425W	TUS1
YBR200W	BEM1	YBR084C-A	RPL19A
YMR153W	NUP53	YNL058C	YNL058C
YJL154C	VPS35	YML001W	YPT7

Predictions at threshold level 0.75 continued...			
Gene1 of predicted pair		Gene2 of predicted pair	
Systematic Name	Standard Name	Systematic Name	Standard Name
YBR200W	BEM1	YOR140W	SFL1
YPR017C	DSS4	YOR127W	RGA1
YOR127W	RGA1	YGR105W	VMA21
YML103C	NUP188	YMR273C	ZDS1
YDR335W	MSN5	YML112W	CTK3
YPL161C	BEM4	YBL085W	BOI1
YLR083C	EMP70	YGL115W	SNF4
YBR030W	YBR030W	YNL087W	TCB2
YMR224C	MRE11	YOR337W	TEA1
YGL066W	SGF73	YHR013C	ARD1
YBL047C	EDE1	YDR264C	AKR1
YNL206C	RTT106	YJL141C	YAK1
YMR198W	CIK1	YBR171W	SEC66
YHR056C	RSC30	YKL057C	NUP120
YMR089C	YTA12	YEL036C	ANP1
YHR178W	STB5	YOR358W	HAP5
YCL001W	RER1	YDR310C	SUM1
YLR090W	XDJ1	YMR224C	MRE11
YEL053C	MAK10	YLR371W	ROM2
YPL042C	SSN3	YEL013W	VAC8
YFR010W	UBP6	YBR171W	SEC66
YCR020C-A	MAK31	YDR293C	SSD1
YCR024C-A	PMP1	YKR024C	DBP7
YJL183W	MNN11	YKL041W	VPS24
YMR224C	MRE11	YGL200C	EMP24
YOR106W	VAM3	YER122C	GLO3
YFR031C-A	RPL2A	YJR033C	RAV1
YDR507C	GIN4	YGR135W	PRE9
YFL023W	BUD27	YML095C	RAD10

Predictions at threshold level 0.75 continued...			
Gene1 of predicted pair		Gene2 of predicted pair	
Systematic Name	Standard Name	Systematic Name	Standard Name
YLR320W	MMS22	YPL089C	RLM1
YNL218W	MGS1	YMR116C	ASC1
YMR311C	GLC8	YPR119W	CLB2
YGR104C	SRB5	YBR020W	GAL1
YML112W	CTK3	YNL107W	YAF9
YHL031C	GOS1	YML001W	YPT7
YJR140C	HIR3	YEL056W	HAT2
YOR106W	VAM3	YEL051W	VMA8
YDR439W	LRS4	YOL081W	IRA2
YER144C	UBP5	YBR019C	GAL10
YPL195W	APL5	YDR207C	UME6
YDR358W	GGA1	YLR206W	ENT2
YJL122W	ALB1	YDR101C	ARX1
YJL176C	SWI3	YML097C	VPS9
YBL008W	HIR1	YML016C	PPZ1
YBR215W	HPC2	YEL013W	VAC8
YLR360W	VPS38	YDL155W	CLB3
YER116C	SLX8	YBR108W	YBR108W
YJL154C	VPS35	YBR108W	YBR108W
YPR017C	DSS4	YNR049C	MSO1
YGL147C	RPL9A	YOL121C	RPS19A
YNL069C	RPL16B	YBL102W	SFT2
YNL238W	KEX2	YHR081W	LRP1
YDR146C	SWI5	YEL036C	ANP1
YGL078C	DBP3	YDR484W	VPS52
YPL195W	APL5	YDR153C	ENT5
YML115C	VAN1	YAL002W	VPS8
YBL047C	EDE1	YPL256C	CLN2
YKR059W	TIF1	YGR188C	BUB1

Predictions at threshold level 0.75 continued...			
**Gene1 of predicted pair**		**Gene2 of predicted pair**	
**Systematic Name**	**Standard Name**	**Systematic Name**	**Standard Name**
YIL133C	RPL16A	YOR208W	PTP2
YNL041C	COG6	YNL127W	FAR11
YLL039C	UBI4	YGL200C	EMP24
YIL148W	RPL40A	YGL016W	KAP122
YNL238W	KEX2	YDR207C	UME6
YFR031C-A	RPL2A	YER144C	UBP5
YMR231W	PEP5	YOR035C	SHE4
YLR441C	RPS1A	YKL048C	ELM1
YNL215W	IES2	YIL036W	CST6
YLR360W	VPS38	YMR127C	SAS2
YMR095C	SNO1	YLR094C	GIS3
YFL013C	IES1	YDR137W	RGP1
YFR031C-A	RPL2A	YNL032W	SIW14
YJR094C	IME1	YDR207C	UME6
YDR225W	HTA1	YNL215W	IES2
YBL102W	SFT2	YGL223C	COG1
YAL024C	LTE1	YGL038C	OCH1
YMR004W	MVP1	YOR327C	SNC2
YER044C	ERG28	YGL003C	CDH1
YGL066W	SGF73	YPL256C	CLN2
YLR006C	SSK1	YDR293C	SSD1
YDL020C	RPN4	YMR198W	CIK1
YGR285C	ZUO1	YPL038W	MET31
YER116C	SLX8	YLR032W	RAD5
YOR043W	WHI2	YER040W	GLN3
YER120W	SCS2	YML001W	YPT7
YLR090W	XDJ1	YKR035W-A	DID2
YDR129C	SAC6	YCR077C	PAT1
YDR225W	HTA1	YDR318W	MCM21

Predictions at threshold level 0.75 continued...			
Gene1 of predicted pair		Gene2 of predicted pair	
Systematic Name	Standard Name	Systematic Name	Standard Name
YOR008C	SLG1	YEL036C	ANP1
YDR477W	SNF1	YJR084W	CSN12
YLR233C	EST1	YGR188C	BUB1
YGL135W	RPL1B	YDR129C	SAC6
YPL022W	RAD1	YLR352W	YLR352W
YCR077C	PAT1	YOR038C	HIR2
YHR156C	LIN1	YNL307C	MCK1
YKR035W-A	DID2	YER139C	YER139C
YKL139W	CTK1	YDL083C	RPS16B
YNL107W	YAF9	YAL002W	VPS8
YMR214W	SCJ1	YLR180W	SAM1
YHR010W	RPL27A	YML028W	TSA1
YGR019W	UGA1	YMR127C	SAS2
YPR036W	VMA13	YBL025W	RRN10
YGL163C	RAD54	YCR086W	CSM1
YKR101W	SIR1	YPR119W	CLB2
YDL006W	PTC1	YER118C	SHO1
YKL025C	PAN3	YGL151W	NUT1
YDR363W-A	SEM1	YER161C	SPT2
YDR108W	GSG1	YPL037C	EGD1
YIL132C	CSM2	YHL006C	SHU1
YOR270C	VPH1	YGR020C	VMA7
YHR108W	GGA2	YJL095W	BCK1
YML115C	VAN1	YAL055W	PEX22
YNL087W	TCB2	YMR214W	SCJ1
YHR161C	YAP1801	YGL095C	VPS45
YBR173C	UMP1	YKL126W	YPK1
YLR288C	MEC3	YLR320W	MMS22
YNL297C	MON2	YMR038C	CCS1

Predictions at threshold level 0.75 continued...			
Gene1 of predicted pair		Gene2 of predicted pair	
Systematic Name	Standard Name	Systematic Name	Standard Name
YDL020C	RPN4	YOR243C	PUS7
YBR194W	SOY1	YDL006W	PTC1
YOR327C	SNC2	YMR214W	SCJ1
YNL291C	MID1	YPR046W	MCM16
YGL033W	HOP2	YJL095W	BCK1
YGL100W	SEH1	YMR048W	CSM3
YGR108W	CLB1	YAR014C	BUD14
YJL058C	BIT61	YJL006C	CTK2
YGL212W	VAM7	YBR171W	SEC66
YOR127W	RGA1	YDR379W	RGA2
YGL212W	VAM7	YJL004C	SYS1
YJR084W	CSN12	YLR398C	SKI2
YKR020W	VPS51	YJL117W	PHO86
YCL027W	FUS1	YPR119W	CLB2
YNL044W	YIP3	YFL048C	EMP47
YOL121C	RPS19A	YML071C	COG8
YKL213C	DOA1	YOR124C	UBP2
YGR104C	SRB5	YPL129W	TAF14
YDR108W	GSG1	YOR327C	SNC2
YML012W	ERV25	YOR327C	SNC2
YHR039C-A	VMA10	YKL041W	VPS24
YPR030W	CSR2	YPR046W	MCM16
YNL307C	MCK1	YDL192W	ARF1
YPL057C	SUR1	YJR032W	CPR7
YBR108W	YBR108W	YEL060C	PRB1
YER139C	YER139C	YOR327C	SNC2
YMR042W	ARG80	YDR207C	UME6
YKR048C	NAP1	YHR115C	DMA1
YPR051W	MAK3	YPR046W	MCM16

Predictions at threshold level 0.75 continued...			
Gene1 of predicted pair		Gene2 of predicted pair	
Systematic Name	Standard Name	Systematic Name	Standard Name
YCR053W	THR4	YBR036C	CSG2
YOR039W	CKB2	YML074C	FPR3
YGR019W	UGA1	YBR114W	RAD16
YMR109W	MYO5	YKL048C	ELM1
YLR381W	CTF3	YHR111W	UBA4
YMR224C	MRE11	YFR010W	UBP6
YHR205W	SCH9	YER049W	TPA1
YML115C	VAN1	YDL077C	VAM6
YPR120C	CLB5	YKL114C	APN1
YER087C-B	SBH1	YGL058W	RAD6
YGR270W	YTA7	YIL103W	DPH1
YMR016C	SOK2	YER040W	GLN3
YLR399C	BDF1	YAR014C	BUD14
YLR337C	VRP1	YNL166C	BNI5
YBR217W	ATG12	YPR030W	CSR2
YGL078C	DBP3	YER144C	UBP5
YOR358W	HAP5	YMR055C	BUB2
YBR200W	BEM1	YLR094C	GIS3
YJL053W	PEP8	YOR036W	PEP12
YJR140C	HIR3	YGR071C	YGR071C
YLR386W	VAC14	YKL048C	ELM1
YGR019W	UGA1	YBL021C	HAP3
YGR270W	YTA7	YMR038C	CCS1
YNL243W	SLA2	YOR132W	VPS17
YGL147C	RPL9A	YKL116C	PRR1
YDR004W	RAD57	YPL024W	RMI1
YMR284W	YKU70	YDL013W	SLX5
YLR083C	EMP70	YJR033C	RAV1
YCL037C	SRO9	YGR071C	YGR071C

Predictions at threshold level 0.75 continued...			
Gene1 of predicted pair		Gene2 of predicted pair	
Systematic Name	Standard Name	Systematic Name	Standard Name
YML115C	VAN1	YPL089C	RLM1
YNR072W	HXT17	YEL053C	MAK10
YBR200W	BEM1	YFR040W	SAP155
YLR441C	RPS1A	YOR043W	WHI2
YOR132W	VPS17	YOL108C	INO4
YBL058W	SHP1	YDR310C	SUM1
YMR026C	PEX12	YDR207C	UME6
YDL006W	PTC1	YAL030W	SNC1
YNL106C	INP52	YGR072W	UPF3
YKL139W	CTK1	YKL048C	ELM1
YLR043C	TRX1	YOR106W	VAM3
YDR335W	MSN5	YDR378C	LSM6
YGL078C	DBP3	YML071C	COG8
YKR001C	VPS1	YJL004C	SYS1
YIL076W	SEC28	YLR452C	SST2
YFR010W	UBP6	YGL115W	SNF4
YKR095W	MLP1	YDR207C	UME6
YOR195W	SLK19	YCR086W	CSM1
YHR178W	STB5	YGL163C	RAD54
YJL078C	PRY3	YOR071C	THI71
YDR137W	RGP1	YBR127C	VMA2
YJR056C	YJR056C	YKR099W	BAS1
YGR217W	CCH1	YHR200W	RPN10
YLR386W	VAC14	YGL003C	CDH1
YOR008C	SLG1	YNL030W	HHF2
YLR090W	XDJ1	YER116C	SLX8
YDR080W	VPS41	YGL054C	ERV14
YJL089W	SIP4	YPR119W	CLB2
YLR095C	IOC2	YFR036W	CDC26

Predictions at threshold level 0.75 continued...			
Gene1 of predicted pair		Gene2 of predicted pair	
Systematic Name	Standard Name	Systematic Name	Standard Name
YDL020C	RPN4	YEL056W	HAT2
YOR337W	TEA1	YJR094C	IME1
YML115C	VAN1	YBL047C	EDE1
YLR079W	SIC1	YER049W	TPA1
YML115C	VAN1	YNR072W	HXT17
YDR225W	HTA1	YMR038C	CCS1
YDR379W	RGA2	YHR200W	RPN10
YMR026C	PEX12	YNL214W	PEX17
YNL153C	GIM3	YHR056C	RSC30
YBR200W	BEM1	YBR260C	RGD1
YKL032C	IXR1	YDL047W	SIT4
YBL051C	PIN4	YHR114W	BZZ1
YNL206C	RTT106	YGL163C	RAD54
YJL060W	BNA3	YOR080W	DIA2
YGL212W	VAM7	YDL223C	HBT1
YOR001W	RRP6	YNL107W	YAF9
YDR383C	NKP1	YDR156W	RPA14
YDR156W	RPA14	YBR200W	BEM1
YCR020C-A	MAK31	YCL001W	RER1
YGR108W	CLB1	YOR001W	RRP6
YPR040W	TIP41	YHR115C	DMA1
YPR171W	BSP1	YDR245W	MNN10
YLR148W	PEP3	YJL029C	VPS53
YMR205C	PFK2	YDL006W	PTC1
YPL195W	APL5	YDL192W	ARF1
YER096W	SHC1	YEL036C	ANP1
YDR245W	MNN10	YCR088W	ABP1
YDR245W	MNN10	YBR131W	CCZ1
YHR013C	ARD1	YFR030W	MET10

Predictions at threshold level 0.75 continued...			
**Gene1 of predicted pair**		**Gene2 of predicted pair**	
**Systematic Name**	**Standard Name**	**Systematic Name**	**Standard Name**
YML038C	YMD8	YML071C	COG8
YDR156W	RPA14	YKL139W	CTK1
YML038C	YMD8	YDL077C	VAM6
YPL240C	HSP82	YBL071W-A	KTI11
YLR320W	MMS22	YJR084W	CSN12
YFR031C-A	RPL2A	YGL054C	ERV14
YAL024C	LTE1	YLR095C	IOC2
YOR033C	EXO1	YML102W	CAC2
YHR156C	LIN1	YDR358W	GGA1
YBR107C	IML3	YLR032W	RAD5
YOR275C	RIM20	YDL006W	PTC1
YMR026C	PEX12	YLR094C	GIS3
YML095C	RAD10	YLR447C	VMA6
YER161C	SPT2	YIR005W	IST3
YBR188C	NTC20	YPL213W	LEA1
YDL116W	NUP84	YFR036W	CDC26
YPL161C	BEM4	YGR152C	RSR1
YLR192C	HCR1	YLR094C	GIS3
YBL019W	APN2	YOL043C	NTG2
YPL001W	HAT1	YBR036C	CSG2
YKL048C	ELM1	YHR013C	ARD1
YPL161C	BEM4	YDR310C	SUM1
YDR335W	MSN5	YLR254C	NDL1
YMR199W	CLN1	YER096W	SHC1
YKR057W	RPS21A	YHR115C	DMA1
YAL024C	LTE1	YIL076W	SEC28
YJL127C	SPT10	YLR182W	SWI6
YDL002C	NHP10	YEL056W	HAT2
YML001W	YPT7	YNL127W	FAR11

Predictions at threshold level 0.75 continued...			
**Gene1 of predicted pair**		**Gene2 of predicted pair**	
**Systematic Name**	**Standard Name**	**Systematic Name**	**Standard Name**
YFR031C-A	RPL2A	YML001W	YPT7
YJR033C	RAV1	YLR309C	IMH1
YKL139W	CTK1	YDR440W	DOT1
YIL076W	SEC28	YER122C	GLO3
YCR088W	ABP1	YLR396C	VPS33
YDL020C	RPN4	YKL101W	HSL1
YNL248C	RPA49	YDR378C	LSM6
YMR224C	MRE11	YBR171W	SEC66
YKL213C	DOA1	YNL107W	YAF9
YJL095W	BCK1	YEL056W	HAT2
YER144C	UBP5	YKR035W-A	DID2
YPL024W	RMI1	YMR230W	RPS10B
YBR107C	IML3	YNL307C	MCK1
YNL288W	CAF40	YDR318W	MCM21
YIL156W	UBP7	YLR064W	YLR064W
YDL190C	UFD2	YMR276W	DSK2
YOR358W	HAP5	YEL056W	HAT2
YLR090W	XDJ1	YDR310C	SUM1
YPL024W	RMI1	YCR086W	CSM1
YGL147C	RPL9A	YOR327C	SNC2
YMR133W	REC114	YDR261C	EXG2
YGL212W	VAM7	YGL054C	ERV14
YKL007W	CAP1	YGL124C	MON1
YOR195W	SLK19	YDR200C	VPS64
YDR389W	SAC7	YLR429W	CRN1
YCL010C	SGF29	YPR072W	NOT5
YDR484W	VPS52	YJL095W	BCK1
YDR004W	RAD57	YNL297C	MON2
YML095C	RAD10	YOR368W	RAD17

Predictions at threshold level 0.75 continued...			
Gene1 of predicted pair		Gene2 of predicted pair	
Systematic Name	Standard Name	Systematic Name	Standard Name
YDL083C	RPS16B	YOR327C	SNC2
YLR386W	VAC14	YJL183W	MNN11
YDR146C	SWI5	YPR111W	DBF20
YNR032W	PPG1	YAL002W	VPS8
YHR039C-A	VMA10	YEL060C	PRB1
YKL139W	CTK1	YOL054W	PSH1
YGL163C	RAD54	YGR188C	BUB1
YBR194W	SOY1	YPR046W	MCM16
YMR172W	HOT1	YDR183W	PLP1
YPL024W	RMI1	YMR152W	YIM1
YKL139W	CTK1	YML006C	GIS4
YLR192C	HCR1	YNR072W	HXT17
YMR026C	PEX12	YPL037C	EGD1
YEL009C	GCN4	YER161C	SPT2
YNL243W	SLA2	YKL113C	RAD27
YDR507C	GIN4	YNL229C	URE2
YDL116W	NUP84	YJL124C	LSM1
YFR040W	SAP155	YMR109W	MYO5
YOR327C	SNC2	YER040W	GLN3
YNR052C	POP2	YPL152W	RRD2
YNL079C	TPM1	YOR014W	RTS1
YCL001W	RER1	YKR020W	VPS51
YLR429W	CRN1	YDL047W	SIT4
YGL200C	EMP24	YNL041C	COG6
YER116C	SLX8	YDR335W	MSN5
YGL147C	RPL9A	YMR152W	YIM1
YPR120C	CLB5	YJL095W	BCK1
YJL183W	MNN11	YFL048C	EMP47
YML013W	UBX2	YKR001C	VPS1

Predictions at threshold level 0.75 continued...			
Gene1 of predicted pair		Gene2 of predicted pair	
Systematic Name	Standard Name	Systematic Name	Standard Name
YER122C	GLO3	YMR230W	RPS10B
YBR057C	MUM2	YOL054W	PSH1
YLR079W	SIC1	YPL178W	CBC2
YER115C	SPR6	YFL047W	RGD2
YOR270C	VPH1	YNL076W	MKS1
YNL307C	MCK1	YML112W	CTK3
YPL115C	BEM3	YGR108W	CLB1
YCR066W	RAD18	YLR309C	IMH1
YGL003C	CDH1	YBR280C	SAF1
YDR293C	SSD1	YDL056W	MBP1
YMR068W	AVO2	YBR270C	BIT2
YDR144C	MKC7	YDR318W	MCM21
YCR038C	BUD5	YPR122W	AXL1
YJL124C	LSM1	YGL115W	SNF4
YHR178W	STB5	YJL124C	LSM1
YIL103W	DPH1	YGL252C	RTG2
YDR293C	SSD1	YJL036W	SNX4
YOL018C	TLG2	YAL002W	VPS8
YPL024W	RMI1	YDR225W	HTA1
YJL172W	CPS1	YLR370C	ARC18
YJR090C	GRR1	YDR207C	UME6
YIL113W	SDP1	YOR208W	PTP2
YBR273C	UBX7	YPR119W	CLB2
YPR036W	VMA13	YMR133W	REC114
YPL024W	RMI1	YPL137C	GIP3
YMR199W	CLN1	YOR014W	RTS1
YGR108W	CLB1	YMR198W	CIK1
YDR176W	NGG1	YGR252W	GCN5
YDR293C	SSD1	YDR254W	CHL4

Predictions at threshold level 0.75 continued...			
Gene1 of predicted pair		Gene2 of predicted pair	
Systematic Name	Standard Name	Systematic Name	Standard Name
YBR126C	TPS1	YNL325C	FIG4
YLR386W	VAC14	YEL056W	HAT2
YOR043W	WHI2	YDR207C	UME6
YPL129W	TAF14	YBR009C	HHF1
YCL037C	SRO9	YCR077C	PAT1
YLR119W	SRN2	YFR010W	UBP6
YDR156W	RPA14	YLR234W	TOP3
YDR245W	MNN10	YBL101C	ECM21
YPL234C	TFP3	YPL195W	APL5
YNL087W	TCB2	YMR016C	SOK2
YDR293C	SSD1	YKR001C	VPS1
YNL264C	PDR17	YMR089C	YTA12
YHR178W	STB5	YBR278W	DPB3
YBL047C	EDE1	YGR135W	PRE9
YLR192C	HCR1	YEL060C	PRB1
YAL024C	LTE1	YPL161C	BEM4
YPR018W	RLF2	YPL253C	VIK1
YPR131C	NAT3	YLR288C	MEC3
YJR140C	HIR3	YLR135W	SLX4
YNL288W	CAF40	YJR135C	MCM22
YPR052C	NHP6A	YGR252W	GCN5
YGR184C	UBR1	YKL101W	HSL1
YCL037C	SRO9	YDL192W	ARF1
YCR086W	CSM1	YOL090W	MSH2
YGL115W	SNF4	YOL018C	TLG2
YMR055C	BUB2	YOL067C	RTG1
YLR360W	VPS38	YCL037C	SRO9
YDL155W	CLB3	YLR032W	RAD5
YKL006W	RPL14A	YDL155W	CLB3

Predictions at threshold level 0.75 continued...			
Gene1 of predicted pair		Gene2 of predicted pair	
**Systematic Name**	**Standard Name**	**Systematic Name**	**Standard Name**
YGR054W	YGR054W	YPR046W	MCM16
YLR371W	ROM2	YBR280C	SAF1
YLR035C	MLH2	YMR167W	MLH1
YMR202W	ERG2	YLR425W	TUS1
YGL078C	DBP3	YMR311C	GLC8
YNL145W	MFA2	YCR077C	PAT1
YPR030W	CSR2	YDR310C	SUM1
YJR040W	GEF1	YER162C	RAD4
YMR039C	SUB1	YDL185W	TFP1
YPR017C	DSS4	YAR014C	BUD14
YDL192W	ARF1	YMR214W	SCJ1
YHR013C	ARD1	YFR036W	CDC26
YLR337C	VRP1	YDR318W	MCM21
YLR095C	IOC2	YMR133W	REC114
YDR101C	ARX1	YHR052W	CIC1
YML109W	ZDS2	YPR119W	CLB2
YHR039C-A	VMA10	YOL018C	TLG2
YBL021C	HAP3	YAL002W	VPS8
YJR033C	RAV1	YPR032W	SRO7
YDR108W	GSG1	YOL108C	INO4
YNL078W	NIS1	YCL024W	KCC4
YIL036W	CST6	YKL110C	KTI12
YFR031C-A	RPL2A	YHR039C-A	VMA10
YFR040W	SAP155	YJL183W	MNN11
YCL037C	SRO9	YER139C	YER139C
YLR371W	ROM2	YCR053W	THR4
YLR090W	XDJ1	YPR046W	MCM16
YIR005W	IST3	YMR038C	CCS1
YFL033C	RIM15	YKR030W	GMH1

Predictions at threshold level 0.75 continued...			
Gene1 of predicted pair		Gene2 of predicted pair	
Systematic Name	Standard Name	Systematic Name	Standard Name
YML006C	GIS4	YJR090C	GRR1
YKL116C	PRR1	YDR358W	GGA1
YBR171W	SEC66	YJL190C	RPS22A
YDR443C	SSN2	YDL077C	VAM6
YOR066W	MSA1	YDL192W	ARF1
YHR156C	LIN1	YJL095W	BCK1
YNL297C	MON2	YJL004C	SYS1
YKR072C	SIS2	YER161C	SPT2
YBR108W	YBR108W	YER040W	GLN3
YDR004W	RAD57	YPL256C	CLN2
YLR083C	EMP70	YDR293C	SSD1
YGL147C	RPL9A	YCR053W	THR4
YPR052C	NHP6A	YGR104C	SRB5
YIL076W	SEC28	YDR484W	VPS52
YMR172W	HOT1	YGL124C	MON1
YGL200C	EMP24	YOR312C	RPL20B
YFR024C-A	LSB3	YHR016C	YSC84
YLR306W	UBC12	YDR139C	RUB1
YLR216C	CPR6	YMR055C	BUB2
YNL297C	MON2	YKR030W	GMH1
YGL033W	HOP2	YHR200W	RPN10
YNL215W	IES2	YKL213C	DOA1
YKL101W	HSL1	YLR032W	RAD5
YMR216C	SKY1	YPL195W	APL5
YIL036W	CST6	YDR318W	MCM21
YJR052W	RAD7	YNL230C	ELA1
YDR176W	NGG1	YGL066W	SGF73
YNL025C	SSN8	YDR289C	RTT103
YOR023C	AHC1	YAR014C	BUD14

Predictions at threshold level 0.75 continued...			
**Gene1 of predicted pair**		**Gene2 of predicted pair**	
**Systematic Name**	**Standard Name**	**Systematic Name**	**Standard Name**
YDL059C	RAD59	YLR182W	SWI6
YML115C	VAN1	YOR066W	MSA1
YPL253C	VIK1	YNL236W	SIN4
YNL206C	RTT106	YLR381W	CTF3
YER144C	UBP5	YPL037C	EGD1
YFL023W	BUD27	YHR010W	RPL27A
YMR116C	ASC1	YDL006W	PTC1
YNL291C	MID1	YDR254W	CHL4
YNL243W	SLA2	YDR388W	RVS167
YLR090W	XDJ1	YHR039C-A	VMA10
YDR225W	HTA1	YKL068W	NUP100
YLR360W	VPS38	YNL297C	MON2
YNL206C	RTT106	YML095C	RAD10
YDL184C	RPL41A	YMR214W	SCJ1
YPL129W	TAF14	YPR072W	NOT5
YOR308C	SNU66	YLR350W	ORM2
YFL013C	IES1	YPL057C	SUR1
YJR063W	RPA12	YKL110C	KTI12
YNL307C	MCK1	YGL252C	RTG2
YFR030W	MET10	YOL081W	IRA2
YBR200W	BEM1	YIR005W	IST3
YAL024C	LTE1	YKR099W	BAS1
YMR055C	BUB2	YHR111W	UBA4
YEL065W	SIT1	YHR081W	LRP1
YOL030W	GAS5	YCR086W	CSM1
YLR119W	SRN2	YDR477W	SNF1
YER044C-A	MEI4	YHR086W	NAM8
YGL215W	CLG1	YGR105W	VMA21
YOR106W	VAM3	YBR260C	RGD1

Predictions at threshold level 0.75 continued...			
Gene1 of predicted pair		Gene2 of predicted pair	
Systematic Name	Standard Name	Systematic Name	Standard Name
YGR217W	CCH1	YDR245W	MNN10
YNL054W	VAC7	YPL152W	RRD2
YER096W	SHC1	YDR207C	UME6
YGL223C	COG1	YPL234C	TFP3
YPL161C	BEM4	YPR046W	MCM16
YOR132W	VPS17	YDR320C	SWA2
YPR101W	SNT309	YDR378C	LSM6
YGL025C	PGD1	YBR019C	GAL10
YJL172W	CPS1	YKL048C	ELM1
YGL060W	YBP2	YJL127C	SPT10
YDR318W	MCM21	YKL101W	HSL1
YBL047C	EDE1	YGL031C	RPL24A
YPL234C	TFP3	YEL051W	VMA8
YGR040W	KSS1	YDR103W	STE5
YML103C	NUP188	YDR183W	PLP1
YMR142C	RPL13B	YJR032W	CPR7
YGR135W	PRE9	YOL081W	IRA2
YLR410W	VIP1	YPL152W	RRD2
YPL246C	RBD2	YGL016W	KAP122
YNL068C	FKH2	YDR379W	RGA2
YNL307C	MCK1	YPL152W	RRD2
YAL024C	LTE1	YGL078C	DBP3
YPR046W	MCM16	YBR036C	CSG2
YFR010W	UBP6	YLR350W	ORM2
YPR131C	NAT3	YBR194W	SOY1
YDR146C	SWI5	YBR274W	CHK1
YLR234W	TOP3	YLR320W	MMS22
YMR224C	MRE11	YCR086W	CSM1
YDR156W	RPA14	YML112W	CTK3

Predictions at threshold level 0.75 continued...			
Gene1 of predicted pair		Gene2 of predicted pair	
Systematic Name	Standard Name	Systematic Name	Standard Name
YGL237C	HAP2	YJL095W	BCK1
YHR134W	WSS1	YBR114W	RAD16
YKR099W	BAS1	YER162C	RAD4
YMR172W	HOT1	YPL253C	VIK1
YLR192C	HCR1	YDR484W	VPS52
YMR026C	PEX12	YPR030W	CSR2
YML102W	CAC2	YDR207C	UME6
YDR469W	SDC1	YGR188C	BUB1
YLR320W	MMS22	YEL060C	PRB1
YMR142C	RPL13B	YLR452C	SST2
YER118C	SHO1	YML097C	VPS9
YOR290C	SNF2	YDR071C	PAA1
YPL161C	BEM4	YAL002W	VPS8
YOR337W	TEA1	YMR311C	GLC8
YPR046W	MCM16	YML112W	CTK3
YHR108W	GGA2	YJL140W	RPB4
YML016C	PPZ1	YDL047W	SIT4
YPR017C	DSS4	YNL041C	COG6
YFR031C-A	RPL2A	YBL058W	SHP1
YLR192C	HCR1	YBR030W	YBR030W
YOR127W	RGA1	YJR090C	GRR1
YMR172W	HOT1	YLR447C	VMA6
YNL218W	MGS1	YJR035W	RAD26
YIR003W	YIR003W	YKL213C	DOA1
YPL057C	SUR1	YJR084W	CSN12
YDR146C	SWI5	YBR171W	SEC66
YNL248C	RPA49	YBR107C	IML3
YHR161C	YAP1801	YHR108W	GGA2
YDL083C	RPS16B	YER162C	RAD4

Predictions at threshold level 0.75 continued...			
**Gene1 of predicted pair**		**Gene2 of predicted pair**	
**Systematic Name**	**Standard Name**	**Systematic Name**	**Standard Name**
YDL184C	RPL41A	YHR200W	RPN10
YBR188C	NTC20	YGL174W	BUD13
YJL154C	VPS35	YMR231W	PEP5
YOR290C	SNF2	YNL030W	HHF2
YNL107W	YAF9	YDR207C	UME6
YJR063W	RPA12	YML112W	CTK3
YOR106W	VAM3	YMR054W	STV1
YBR217W	ATG12	YER116C	SLX8
YLR079W	SIC1	YDL226C	GCS1
YBL058W	SHP1	YDR137W	RGP1
YIL076W	SEC28	YBR019C	GAL10
YNL069C	RPL16B	YNL291C	MID1
YOR127W	RGA1	YJL157C	FAR1
YHL027W	RIM101	YPL234C	TFP3
YMR311C	GLC8	YNL076W	MKS1
YGL078C	DBP3	YBL025W	RRN10
YGR081C	SLX9	YDL059C	RAD59
YMR016C	SOK2	YKL126W	YPK1
YDR293C	SSD1	YOL108C	INO4
YBL017C	PEP1	YHR161C	YAP1801
YDL059C	RAD59	YMR038C	CCS1
YNL094W	APP1	YGR136W	LSB1
YGR072W	UPF3	YOR112W	CEX1
YMR039C	SUB1	YBL008W	HIR1
YHR081W	LRP1	YGL003C	CDH1
YAL024C	LTE1	YHR013C	ARD1
YDR225W	HTA1	YMR284W	YKU70
YLR386W	VAC14	YHR012W	VPS29
YCR020C-A	MAK31	YML038C	YMD8

Predictions at threshold level 0.75 continued...			
Gene1 of predicted pair		Gene2 of predicted pair	
Systematic Name	Standard Name	Systematic Name	Standard Name
YNL238W	KEX2	YPR032W	SRO7
YNL329C	PEX6	YKR020W	VPS51
YBR171W	SEC66	YAR014C	BUD14
YNL206C	RTT106	YJL095W	BCK1
YIL159W	BNR1	YGL078C	DBP3
YIL159W	BNR1	YER118C	SHO1
YML095C	RAD10	YGR270W	YTA7
YFL013C	IES1	YOR038C	HIR2
YFL023W	BUD27	YGL003C	CDH1
YMR216C	SKY1	YMR054W	STV1
YLR079W	SIC1	YLR386W	VAC14
YLR090W	XDJ1	YLR192C	HCR1
YNL215W	IES2	YPL180W	TCO89
YOR290C	SNF2	YFL023W	BUD27
YOR080W	DIA2	YMR202W	ERG2
YOL018C	TLG2	YKL119C	VPH2
YBR083W	TEC1	YDR358W	GGA1
YDR363W-A	SEM1	YAR014C	BUD14
YNL044W	YIP3	YGR105W	VMA21
YJR056C	YJR056C	YCR053W	THR4
YHR013C	ARD1	YMR089C	YTA12
YPL195W	APL5	YOL018C	TLG2
YIL076W	SEC28	YGL174W	BUD13
YML001W	YPT7	YER162C	RAD4
YLL039C	UBI4	YNR006W	VPS27
YLR447C	VMA6	YIL008W	URM1
YOR270C	VPH1	YKR035W-A	DID2
YGL033W	HOP2	YBR036C	CSG2
YPL024W	RMI1	YNL307C	MCK1

Predictions at threshold level 0.75 continued...			
Gene1 of predicted pair		Gene2 of predicted pair	
Systematic Name	Standard Name	Systematic Name	Standard Name
YDR146C	SWI5	YOR368W	RAD17
YPL042C	SSN3	YOR124C	UBP2
YNL054W	VAC7	YNL225C	CNM67
YAL020C	ATS1	YNL116W	DMA2
YLR216C	CPR6	YDR254W	CHL4
YGL033W	HOP2	YDL223C	HBT1
YFR024C-A	LSB3	YLR144C	ACF2
YPL024W	RMI1	YJR135C	MCM22
YAL024C	LTE1	YNL058C	YNL058C
YOR109W	INP53	YPL234C	TFP3
YML103C	NUP188	YDR318W	MCM21
YMR026C	PEX12	YML001W	YPT7
YNL297C	MON2	YJR060W	CBF1
YMR026C	PEX12	YNR072W	HXT17
YMR055C	BUB2	YGL151W	NUT1
YPR017C	DSS4	YNL293W	MSB3
YDR129C	SAC6	YJL006C	CTK2
YML115C	VAN1	YBR280C	SAF1
YPL096C-A	ERI1	YBL007C	SLA1
YLR337C	VRP1	YHR016C	YSC84
YMR153W	NUP53	YPL037C	EGD1
YMR224C	MRE11	YGL066W	SGF73
YMR153W	NUP53	YGL025C	PGD1
YPL024W	RMI1	YDR289C	RTT103
YDL155W	CLB3	YDR318W	MCM21
YPL253C	VIK1	YBR171W	SEC66
YML071C	COG8	YDR310C	SUM1
YGL087C	MMS2	YBL051C	PIN4
YLR064W	YLR064W	YKR030W	GMH1

Predictions at threshold level 0.75 continued...			
**Gene1 of predicted pair**		**Gene2 of predicted pair**	
**Systematic Name**	**Standard Name**	**Systematic Name**	**Standard Name**
YDR108W	GSG1	YBR036C	CSG2
YDL116W	NUP84	YDL155W	CLB3
YDR004W	RAD57	YCR077C	PAT1
YDL020C	RPN4	YOR368W	RAD17
YAL024C	LTE1	YLR386W	VAC14
YMR216C	SKY1	YLR309C	IMH1
YMR109W	MYO5	YOR358W	HAP5
YIL036W	CST6	YBR107C	IML3
YLR337C	VRP1	YDR293C	SSD1
YDR004W	RAD57	YBR274W	CHK1
YDR293C	SSD1	YGL003C	CDH1
YDR171W	HSP42	YJR035W	RAD26
YBL078C	ATG8	YGL212W	VAM7
YOR358W	HAP5	YPL018W	CTF19
YKR072C	SIS2	YDR076W	RAD55
YPL042C	SSN3	YDL005C	MED2
YCR031C	RPS14A	YOL081W	IRA2
YML103C	NUP188	YMR164C	MSS11
YHR012W	VPS29	YHR114W	BZZ1
YEL065W	SIT1	YHR156C	LIN1
YGL133W	ITC1	YJL095W	BCK1
YLR170C	APS1	YOR109W	INP53
YLR441C	RPS1A	YDR207C	UME6
YPL129W	TAF14	YJR140C	HIR3
YFR030W	MET10	YLR254C	NDL1
YBR057C	MUM2	YDL223C	HBT1
YNL166C	BNI5	YJL190C	RPS22A
YJR084W	CSN12	YNL076W	MKS1
YNL199C	GCR2	YPL256C	CLN2

Predictions at threshold level 0.75 continued...			
Gene1 of predicted pair		Gene2 of predicted pair	
Systematic Name	Standard Name	Systematic Name	Standard Name
YMR284W	YKU70	YCR066W	RAD18
YPL240C	HSP82	YEL022W	GEA2
YJL154C	VPS35	YDR245W	MNN10
YNL069C	RPL16B	YDR207C	UME6
YMR198W	CIK1	YDR378C	LSM6
YBR073W	RDH54	YDL013W	SLX5
YDR129C	SAC6	YMR198W	CIK1
YPR051W	MAK3	YPL018W	CTF19
YLR360W	VPS38	YBR108W	YBR108W
YJL151C	SNA3	YLR064W	YLR064W
YNL058C	YNL058C	YBL025W	RRN10
YOR270C	VPH1	YPL234C	TFP3
YER144C	UBP5	YER040W	GLN3
YGR054W	YGR054W	YPR163C	TIF3
YNL307C	MCK1	YLR452C	SST2
YLR102C	APC9	YPL256C	CLN2
YPL037C	EGD1	YDL083C	RPS16B
YIL076W	SEC28	YOR066W	MSA1
YPR052C	NHP6A	YLR357W	RSC2
YBR215W	HPC2	YAL002W	VPS8
YDR293C	SSD1	YHR178W	STB5
YOR043W	WHI2	YCR086W	CSM1
YGL223C	COG1	YDR153C	ENT5
YOR290C	SNF2	YJR084W	CSN12
YLR119W	SRN2	YFL023W	BUD27
YDL155W	CLB3	YHL002W	HSE1
YBL102W	SFT2	YPL120W	VPS30
YFR032C-A	RPL29	YJL117W	PHO86
YDL077C	VAM6	YNL076W	MKS1

Predictions at threshold level 0.75 continued...			
Gene1 of predicted pair		Gene2 of predicted pair	
Systematic Name	Standard Name	Systematic Name	Standard Name
YNL069C	RPL16B	YML001W	YPT7
YLR386W	VAC14	YMR055C	BUB2
YKL025C	PAN3	YBR108W	YBR108W
YPR017C	DSS4	YDL006W	PTC1
YOR275C	RIM20	YHR158C	KEL1
YFL023W	BUD27	YEL060C	PRB1
YLR313C	SPH1	YJR035W	RAD26
YHR161C	YAP1801	YJR125C	ENT3
YDR174W	HMO1	YIL103W	DPH1
YGR261C	APL6	YNL297C	MON2
YGL212W	VAM7	YJL095W	BCK1
YML016C	PPZ1	YJL006C	CTK2
YPL057C	SUR1	YDL192W	ARF1
YJL172W	CPS1	YLR452C	SST2
YBR009C	HHF1	YDR440W	DOT1
YLR234W	TOP3	YBR215W	HPC2
YBR200W	BEM1	YKL110C	KTI12
YOL111C	MDY2	YDL192W	ARF1
YPL253C	VIK1	YNL079C	TPM1
YML071C	COG8	YDL077C	VAM6
YDR443C	SSN2	YOR337W	TEA1
YFL033C	RIM15	YPR032W	SRO7
YKR072C	SIS2	YLR352W	YLR352W
YBR171W	SEC66	YOL111C	MDY2
YMR026C	PEX12	YBR030W	YBR030W
YHR149C	SKG6	YPL240C	HSP82
YFR031C-A	RPL2A	YGR020C	VMA7
YDR293C	SSD1	YDL155W	CLB3
YKL126W	YPK1	YJR056C	YJR056C

Predictions at threshold level 0.75 continued...			
Gene1 of predicted pair		Gene2 of predicted pair	
Systematic Name	Standard Name	Systematic Name	Standard Name
YDR363W-A	SEM1	YML097C	VPS9
YPR084W	YPR084W	YPL161C	BEM4
YKR035W-A	DID2	YPL161C	BEM4
YPL161C	BEM4	YKR020W	VPS51
YFL023W	BUD27	YGL115W	SNF4
YKL006W	RPL14A	YDR245W	MNN10
YJL095W	BCK1	YNL116W	DMA2
YLR371W	ROM2	YBL025W	RRN10
YML115C	VAN1	YJL117W	PHO86
YPR120C	CLB5	YCR086W	CSM1
YNL248C	RPA49	YLR182W	SWI6
YLR360W	VPS38	YMR109W	MYO5
YJR056C	YJR056C	YNL307C	MCK1
YNL041C	COG6	YPL137C	GIP3
YDL155W	CLB3	YJR090C	GRR1
YNL044W	YIP3	YPR028W	YOP1
YNL248C	RPA49	YGL163C	RAD54
YLR371W	ROM2	YDL077C	VAM6
YFL033C	RIM15	YML001W	YPT7
YNR052C	POP2	YDR335W	MSN5
YER120W	SCS2	YPL195W	APL5
YPL024W	RMI1	YOR195W	SLK19
YJL183W	MNN11	YOL081W	IRA2
YBR200W	BEM1	YNL293W	MSB3
YGL237C	HAP2	YDR155C	CPR1
YLL039C	UBI4	YKL041W	VPS24
YER143W	DDI1	YOR036W	PEP12
YJR084W	CSN12	YOL081W	IRA2
YMR116C	ASC1	YBL101C	ECM21

Predictions at threshold level 0.75 continued...			
Gene1 of predicted pair		Gene2 of predicted pair	
Systematic Name	Standard Name	Systematic Name	Standard Name
YPL037C	EGD1	YDR310C	SUM1
YPR036W	VMA13	YMR054W	STV1
YGR108W	CLB1	YEL053C	MAK10
YDR156W	RPA14	YNL116W	DMA2
YBR215W	HPC2	YJR135C	MCM22
YLR095C	IOC2	YKL116C	PRR1
YOR337W	TEA1	YMR042W	ARG80
YKR072C	SIS2	YER116C	SLX8
YGL115W	SNF4	YML001W	YPT7
YPR030W	CSR2	YOR035C	SHE4
YBR107C	IML3	YPR120C	CLB5
YNL186W	UBP10	YPR119W	CLB2
YDR144C	MKC7	YEL056W	HAT2
YBR171W	SEC66	YOR043W	WHI2
YGL066W	SGF73	YMR055C	BUB2
YDL223C	HBT1	YML001W	YPT7
YGL124C	MON1	YOL081W	IRA2
YDL020C	RPN4	YMR055C	BUB2
YDR137W	RGP1	YGR188C	BUB1
YNL215W	IES2	YDR146C	SWI5
YJL036W	SNX4	YPR051W	MAK3
YOR080W	DIA2	YDR254W	CHL4
YDR378C	LSM6	YDR289C	RTT103
YLL039C	UBI4	YDL155W	CLB3
YLR360W	VPS38	YCR088W	ABP1
YNL094W	APP1	YCR088W	ABP1
YNL215W	IES2	YKL041W	VPS24
YER116C	SLX8	YOR368W	RAD17
YDL013W	SLX5	YJL187C	SWE1

Predictions at threshold level 0.75 continued...			
Gene1 of predicted pair		Gene2 of predicted pair	
Systematic Name	Standard Name	Systematic Name	Standard Name
YCL032W	STE50	YDL159W	STE7
YKR072C	SIS2	YLR135W	SLX4
YGR249W	MGA1	YOR327C	SNC2
YJL095W	BCK1	YMR089C	YTA12
YNL243W	SLA2	YML062C	MFT1
YMR109W	MYO5	YDR183W	PLP1
YDR265W	PEX10	YOL044W	PEX15
YMR202W	ERG2	YOR327C	SNC2
YLR441C	RPS1A	YOL081W	IRA2
YDR156W	RPA14	YNL225C	CNM67
YNL025C	SSN8	YDR477W	SNF1
YER087C-B	SBH1	YML019W	OST6
YLR288C	MEC3	YPL256C	CLN2
YOR033C	EXO1	YGL100W	SEH1
YBL058W	SHP1	YMR152W	YIM1
YCR066W	RAD18	YKR020W	VPS51
YOR290C	SNF2	YHR039C-A	VMA10
YCR086W	CSM1	YDR155C	CPR1
YLR360W	VPS38	YDR323C	PEP7
YOR106W	VAM3	YGR020C	VMA7
YGL003C	CDH1	YGL124C	MON1
YJR140C	HIR3	YOR043W	WHI2
YLR135W	SLX4	YPL079W	RPL21B
YOR112W	CEX1	YJL036W	SNX4
YNL238W	KEX2	YNL051W	COG5
YHR178W	STB5	YOR368W	RAD17
YKL139W	CTK1	YKR030W	GMH1
YNL106C	INP52	YNL307C	MCK1
YLR386W	VAC14	YNL248C	RPA49

Predictions at threshold level 0.75 continued...			
Gene1 of predicted pair		Gene2 of predicted pair	
Systematic Name	Standard Name	Systematic Name	Standard Name
YDR383C	NKP1	YML103C	NUP188
YBR215W	HPC2	YMR038C	CCS1
YBL103C	RTG3	YHR081W	LRP1
YEL053C	MAK10	YER120W	SCS2
YLR315W	NKP2	YIL103W	DPH1
YLR079W	SIC1	YKL048C	ELM1
YCL037C	SRO9	YDR440W	DOT1
YBR289W	SNF5	YOR038C	HIR2
YPR051W	MAK3	YMR109W	MYO5
YLR399C	BDF1	YDR254W	CHL4
YFR031C-A	RPL2A	YJL053W	PEP8
YER144C	UBP5	YHR060W	VMA22
YBL102W	SFT2	YGR020C	VMA7
YJL095W	BCK1	YMR214W	SCJ1
YJR040W	GEF1	YEL051W	VMA8
YLR119W	SRN2	YBR200W	BEM1
YPR030W	CSR2	YBL025W	RRN10
YGR108W	CLB1	YAL002W	VPS8
YMR055C	BUB2	YDR363W-A	SEM1
YML012W	ERV25	YKR039W	GAP1
YLL007C	YLL007C	YLR447C	VMA6
YOR358W	HAP5	YGR054W	YGR054W
YBR108W	YBR108W	YNL058C	YNL058C
YBR171W	SEC66	YNL166C	BNI5
YBR057C	MUM2	YML028W	TSA1
YCR066W	RAD18	YGL054C	ERV14
YOR083W	WHI5	YPL018W	CTF19
YOR195W	SLK19	YNR052C	POP2
YLR090W	XDJ1	YNL325C	FIG4

Predictions at threshold level 0.75 continued...			
Gene1 of predicted pair		Gene2 of predicted pair	
Systematic Name	Standard Name	Systematic Name	Standard Name
YLR135W	SLX4	YGL031C	RPL24A
YMR224C	MRE11	YKL032C	IXR1
YLR234W	TOP3	YHL002W	HSE1
YMR116C	ASC1	YGR135W	PRE9
YPL180W	TCO89	YDR363W-A	SEM1
YML071C	COG8	YMR230W	RPS10B
YOR127W	RGA1	YNL238W	KEX2
YDR484W	VPS52	YOL121C	RPS19A
YDL020C	RPN4	YGL003C	CDH1
YHL020C	OPI1	YDR153C	ENT5
YDR225W	HTA1	YNL291C	MID1
YJR084W	CSN12	YBR280C	SAF1
YLL007C	YLL007C	YJL183W	MNN11
YKL007W	CAP1	YGR152C	RSR1
YKL020C	SPT23	YPR119W	CLB2
YNL248C	RPA49	YNL225C	CNM67
YJL154C	VPS35	YEL053C	MAK10
YKL010C	UFD4	YBL101C	ECM21
YDR443C	SSN2	YNL079C	TPM1
YLR094C	GIS3	YJL183W	MNN11
YBR041W	FAT1	YKR046C	PET10
YLR381W	CTF3	YBR215W	HPC2
YOL148C	SPT20	YPL248C	GAL4
YBR107C	IML3	YOL067C	RTG1
YMR199W	CLN1	YOL081W	IRA2
YKL007W	CAP1	YKL204W	EAP1
YNR058W	BIO3	YJR025C	BNA1
YMR199W	CLN1	YMR038C	CCS1
YBR171W	SEC66	YBR280C	SAF1

Predictions at threshold level 0.75 continued...			
Gene1 of predicted pair		Gene2 of predicted pair	
Systematic Name	Standard Name	Systematic Name	Standard Name
YDL002C	NHP10	YOR141C	ARP8
YPL105C	YPL105C	YML071C	COG8
YER110C	KAP123	YNL107W	YAF9
YEL065W	SIT1	YDL077C	VAM6
YLR410W	VIP1	YLR315W	NKP2
YBR107C	IML3	YGR135W	PRE9
YBL008W	HIR1	YKL101W	HSL1
YGL033W	HOP2	YJL172W	CPS1
YCL037C	SRO9	YDR289C	RTT103
YDL020C	RPN4	YML102W	CAC2
YMR095C	SNO1	YMR152W	YIM1
YBR273C	UBX7	YJL048C	UBX6
YLL039C	UBI4	YBL047C	EDE1
YLR119W	SRN2	YBR030W	YBR030W
YER116C	SLX8	YDL192W	ARF1
YJL176C	SWI3	YOR290C	SNF2
YFL023W	BUD27	YDR076W	RAD55
YER087C-B	SBH1	YAR003W	SWD1
YNL215W	IES2	YDR245W	MNN10
YIL076W	SEC28	YPL057C	SUR1
YIL076W	SEC28	YOL018C	TLG2
YMR133W	REC114	YER040W	GLN3
YNL058C	YNL058C	YMR311C	GLC8
YMR153W	NUP53	YDR310C	SUM1
YDL154W	MSH5	YMR167W	MLH1
YHR056C	RSC30	YLR357W	RSC2
YKL110C	KTI12	YFR030W	MET10
YFR031C-A	RPL2A	YLR441C	RPS1A
YKR099W	BAS1	YHR081W	LRP1

Predictions at threshold level 0.75 continued...			
Gene1 of predicted pair		Gene2 of predicted pair	
Systematic Name	Standard Name	Systematic Name	Standard Name
YDR289C	RTT103	YNL116W	DMA2
YDL020C	RPN4	YBL021C	HAP3
YBR171W	SEC66	YKR035W-A	DID2
YHR013C	ARD1	YJL140W	RPB4
YLR381W	CTF3	YJL124C	LSM1
YLR337C	VRP1	YEL056W	HAT2
YMR224C	MRE11	YML001W	YPT7
YMR142C	RPL13B	YGL003C	CDH1
YOL121C	RPS19A	YDL192W	ARF1
YPL253C	VIK1	YGL237C	HAP2
YDL020C	RPN4	YPL057C	SUR1
YGL100W	SEH1	YKL068W	NUP100
YGL124C	MON1	YDR310C	SUM1
YKL032C	IXR1	YBL047C	EDE1
YOR001W	RRP6	YGR270W	YTA7
YAL024C	LTE1	YER116C	SLX8
YPR084W	YPR084W	YNR032W	PPG1
YNL307C	MCK1	YNR072W	HXT17
YHR178W	STB5	YBR108W	YBR108W
YNL025C	SSN8	YDR363W-A	SEM1
YDR142C	PEX7	YLL061W	MMP1
YMR216C	SKY1	YHR012W	VPS29
YBR057C	MUM2	YGR184C	UBR1
YDR254W	CHL4	YGR135W	PRE9
YDR080W	VPS41	YNL051W	COG5
YJL124C	LSM1	YDL059C	RAD59
YNL051W	COG5	YEL042W	GDA1
YMR095C	SNO1	YNR072W	HXT17
YBR278W	DPB3	YCR077C	PAT1

Predictions at threshold level 0.75 continued...			
Gene1 of predicted pair		Gene2 of predicted pair	
Systematic Name	Standard Name	Systematic Name	Standard Name
YPL161C	BEM4	YDL083C	RPS16B
YNR072W	HXT17	YMR038C	CCS1
YNL288W	CAF40	YPL161C	BEM4
YGR217W	CCH1	YKL048C	ELM1
YMR198W	CIK1	YLR398C	SKI2
YMR055C	BUB2	YPL161C	BEM4
YPL089C	RLM1	YJL183W	MNN11
YFR010W	UBP6	YOL081W	IRA2
YBR278W	DPB3	YBR215W	HPC2
YEL036C	ANP1	YDR207C	UME6
YDL223C	HBT1	YBR019C	GAL10
YLR058C	SHM2	YPL106C	SSE1
YJR056C	YJR056C	YBR171W	SEC66
YBL051C	PIN4	YJL183W	MNN11
YJL176C	SWI3	YPL161C	BEM4
YHR073W	OSH3	YMR273C	ZDS1
YBL058W	SHP1	YBR171W	SEC66
YHR060W	VMA22	YBR036C	CSG2
YDR146C	SWI5	YCR086W	CSM1
YGL178W	MPT5	YGL003C	CDH1
YPR084W	YPR084W	YPR017C	DSS4
YLR292C	SEC72	YJR032W	CPR7
YNL215W	IES2	YLR079W	SIC1
YGR054W	YGR054W	YNL107W	YAF9
YLR234W	TOP3	YHR200W	RPN10
YGL060W	YBP2	YNL236W	SIN4
YPL037C	EGD1	YDL006W	PTC1
YAR042W	SWH1	YDL065C	PEX19
YDR443C	SSN2	YBR108W	YBR108W

Predictions at threshold level 0.75 continued...			
Gene1 of predicted pair		Gene2 of predicted pair	
Systematic Name	Standard Name	Systematic Name	Standard Name
YER161C	SPT2	YBR009C	HHF1
YGL019W	CKB1	YOL081W	IRA2
YFR030W	MET10	YPL018W	CTF19
YIL156W	UBP7	YPL178W	CBC2
YDR389W	SAC7	YBR200W	BEM1
YDL080C	THI3	YMR224C	MRE11
YKL126W	YPK1	YDR207C	UME6
YGL190C	CDC55	YNL271C	BNI1
YLR386W	VAC14	YDL223C	HBT1
YGL190C	CDC55	YGL208W	SIP2
YOL111C	MDY2	YOR035C	SHE4
YDR129C	SAC6	YHL027W	RIM101
YDR085C	AFR1	YBL085W	BOI1
YCR081W	SRB8	YGL025C	PGD1
YMR125W	STO1	YBL079W	NUP170
YPR017C	DSS4	YOL108C	INO4
YGL147C	RPL9A	YLR180W	SAM1
YJL154C	VPS35	YOR069W	VPS5
YBR127C	VMA2	YHR060W	VMA22
YJL060W	BNA3	YPL018W	CTF19
YOR337W	TEA1	YJL183W	MNN11
YNL041C	COG6	YJR135C	MCM22
YPL024W	RMI1	YBR173C	UMP1
YMR179W	SPT21	YGL115W	SNF4
YLR083C	EMP70	YJR040W	GEF1
YLL007C	YLL007C	YDL020C	RPN4
YJL124C	LSM1	YDR363W-A	SEM1
YBL102W	SFT2	YPR051W	MAK3
YIL076W	SEC28	YDL002C	NHP10

Predictions at threshold level 0.75 continued...			
**Gene1 of predicted pair**		**Gene2 of predicted pair**	
**Systematic Name**	**Standard Name**	**Systematic Name**	**Standard Name**
YMR095C	SNO1	YGL115W	SNF4
YKR099W	BAS1	YDL223C	HBT1
YPL115C	BEM3	YBL051C	PIN4
YBR111W-A	SUS1	YDR159W	SAC3
YMR116C	ASC1	YBR041W	FAT1
YLR094C	GIS3	YDL077C	VAM6
YDR293C	SSD1	YML071C	COG8
YGR249W	MGA1	YPL203W	TPK2
YJL154C	VPS35	YMR216C	SKY1
YGR072W	UPF3	YOR106W	VAM3
YDL223C	HBT1	YDL192W	ARF1
YLR337C	VRP1	YLR192C	HCR1
YJL154C	VPS35	YLR309C	IMH1
YKL025C	PAN3	YLR337C	VRP1
YOR127W	RGA1	YKR030W	GMH1
YCL001W	RER1	YPL195W	APL5
YBL051C	PIN4	YHR012W	VPS29
YLR095C	IOC2	YOR035C	SHE4
YFR010W	UBP6	YCR077C	PAT1
YPL090C	RPS6A	YOR078W	BUD21
YKL139W	CTK1	YFR030W	MET10
YMR172W	HOT1	YPL161C	BEM4
YMR224C	MRE11	YBR030W	YBR030W
YML115C	VAN1	YER116C	SLX8
YLR119W	SRN2	YJL172W	CPS1
YER115C	SPR6	YBL079W	NUP170
YDR261C	EXG2	YGL003C	CDH1
YJR060W	CBF1	YPR032W	SRO7
YOR337W	TEA1	YNL032W	SIW14

Predictions at threshold level 0.75 continued...			
Gene1 of predicted pair		Gene2 of predicted pair	
Systematic Name	Standard Name	Systematic Name	Standard Name
YMR273C	ZDS1	YPL246C	RBD2
YLR371W	ROM2	YEL060C	PRB1
YFR024C-A	LSB3	YOR284W	HUA2
YDR108W	GSG1	YFL033C	RIM15
YHR086W	NAM8	YJR021C	REC107
YIL076W	SEC28	YPR032W	SRO7
YPL195W	APL5	YDL077C	VAM6
YFL066C	YFL066C	YNL147W	LSM7
YDR202C	RAV2	YOL018C	TLG2
YGL003C	CDH1	YCR053W	THR4
YDR484W	VPS52	YPR028W	YOP1
YOR156C	NFI1	YNL107W	YAF9
YDR293C	SSD1	YJR135C	MCM22
YHR013C	ARD1	YLR315W	NKP2
YMR125W	STO1	YPL256C	CLN2
YGR275W	RTT102	YOR156C	NFI1
YOR080W	DIA2	YGL200C	EMP24
YJL172W	CPS1	YOL081W	IRA2
YOR270C	VPH1	YDR310C	SUM1
YDR440W	DOT1	YBR010W	HHT1
YGR003W	CUL3	YJL204C	RCY1
YDR293C	SSD1	YBL051C	PIN4
YNL054W	VAC7	YKL110C	KTI12
YHR156C	LIN1	YGL115W	SNF4
YPL115C	BEM3	YMR127C	SAS2
YNL069C	RPL16B	YDL006W	PTC1
YLR119W	SRN2	YGL124C	MON1
YLR398C	SKI2	YOL081W	IRA2
YLR381W	CTF3	YGL250W	RMR1

Predictions at threshold level 0.75 continued...			
Gene1 of predicted pair		Gene2 of predicted pair	
Systematic Name	Standard Name	Systematic Name	Standard Name
YER044C	ERG28	YLR371W	ROM2
YPR018W	RLF2	YLR234W	TOP3
YBR258C	SHG1	YOL145C	CTR9
YHR039C-A	VMA10	YBR280C	SAF1
YAL024C	LTE1	YPR046W	MCM16
YMR199W	CLN1	YML095C	RAD10
YHR119W	SET1	YBR258C	SHG1
YML016C	PPZ1	YLR350W	ORM2
YGL115W	SNF4	YGL054C	ERV14
YHR200W	RPN10	YJR135C	MCM22
YGL200C	EMP24	YAL055W	PEX22
YKR072C	SIS2	YGL175C	SAE2
YLR093C	NYV1	YER139C	YER139C
YHR156C	LIN1	YGL212W	VAM7
YGL246C	RAI1	YDR025W	RPS11A
YGL025C	PGD1	YPR030W	CSR2
YER116C	SLX8	YDR310C	SUM1
YJL154C	VPS35	YMR054W	STV1
YDR260C	SWM1	YOL054W	PSH1
YOR195W	SLK19	YLR315W	NKP2
YNL199C	GCR2	YIR005W	IST3
YPL161C	BEM4	YBR082C	UBC4
YLL007C	YLL007C	YJR090C	GRR1
YIL053W	RHR2	YBR036C	CSG2
YNL054W	VAC7	YML016C	PPZ1
YHR098C	SFB3	YBR231C	SWC5
YBL046W	PSY4	YPR040W	TIP41
YDR174W	HMO1	YLR182W	SWI6
YDL116W	NUP84	YJR059W	PTK2

Predictions at threshold level 0.75 continued...			
Gene1 of predicted pair		Gene2 of predicted pair	
Systematic Name	Standard Name	Systematic Name	Standard Name
YGL003C	CDH1	YLR398C	SKI2
YER162C	RAD4	YPL120W	VPS30
YFL033C	RIM15	YJR060W	CBF1
YJL053W	PEP8	YML001W	YPT7
YBR030W	YBR030W	YPL137C	GIP3
YDR309C	GIC2	YKR048C	NAP1
YBR019C	GAL10	YNL076W	MKS1
YHR134W	WSS1	YOR132W	VPS17
YHR205W	SCH9	YGR063C	SPT4
YLR313C	SPH1	YGL175C	SAE2
YLL039C	UBI4	YHR108W	GGA2
YIL036W	CST6	YJL006C	CTK2
YER179W	DMC1	YLR044C	PDC1
YBR200W	BEM1	YBR019C	GAL10
YDL013W	SLX5	YGR188C	BUB1
YDR439W	LRS4	YPL253C	VIK1
YOR106W	VAM3	YGL237C	HAP2
YLR043C	TRX1	YPL057C	SUR1
YDR507C	GIN4	YDL047W	SIT4
YOR033C	EXO1	YJR090C	GRR1
YCR020C-A	MAK31	YIL076W	SEC28
YML115C	VAN1	YER001W	MNN1
YDR477W	SNF1	YPL057C	SUR1
YDR202C	RAV2	YDL077C	VAM6
YGL078C	DBP3	YGR249W	MGA1
YLR150W	STM1	YMR038C	CCS1
YML013W	UBX2	YNL297C	MON2
YJL176C	SWI3	YLR425W	TUS1
YDR156W	RPA14	YDR183W	PLP1

Predictions at threshold level 0.75 continued...			
**Gene1 of predicted pair**		**Gene2 of predicted pair**	
**Systematic Name**	**Standard Name**	**Systematic Name**	**Standard Name**
YPR131C	NAT3	YGR081C	SLX9
YOR127W	RGA1	YKL032C	IXR1
YLR234W	TOP3	YFL023W	BUD27
YBR173C	UMP1	YLR192C	HCR1
YBR108W	YBR108W	YDR183W	PLP1
YIL159W	BNR1	YPL024W	RMI1
YIL076W	SEC28	YJR060W	CBF1
YGR020C	VMA7	YDL077C	VAM6
YLR360W	VPS38	YDR183W	PLP1
YIL159W	BNR1	YML071C	COG8
YNL288W	CAF40	YML016C	PPZ1
YGL180W	ATG1	YPR185W	ATG13
YML016C	PPZ1	YGR188C	BUB1
YIL076W	SEC28	YDR137W	RGP1
YML038C	YMD8	YNL127W	FAR11
YGL190C	CDC55	YKL114C	APN1
YJL154C	VPS35	YDR456W	NHX1
YHR013C	ARD1	YPR120C	CLB5
YER120W	SCS2	YNR032W	PPG1
YGL147C	RPL9A	YDR310C	SUM1
YBR173C	UMP1	YLR234W	TOP3
YGR104C	SRB5	YGL174W	BUD13
YDL223C	HBT1	YLR425W	TUS1
YOR132W	VPS17	YKR001C	VPS1
YHR060W	VMA22	YPL161C	BEM4
YOR001W	RRP6	YGL115W	SNF4
YNR006W	VPS27	YLR128W	DCN1
YMR054W	STV1	YER162C	RAD4
YBR258C	SHG1	YOR156C	NFI1

Predictions at threshold level 0.75 continued...			
Gene1 of predicted pair		Gene2 of predicted pair	
Systematic Name	Standard Name	Systematic Name	Standard Name
YEL043W	YEL043W	YNR032W	PPG1
YDR202C	RAV2	YDR080W	VPS41
YJR040W	GEF1	YHR012W	VPS29
YMR133W	REC114	YJR032W	CPR7
YBR290W	BSD2	YDL155W	CLB3
YHR200W	RPN10	YNL229C	URE2
YLR441C	RPS1A	YMR133W	REC114
YIL130W	ASG1	YDL156W	YDL156W
YKL048C	ELM1	YDL006W	PTC1
YBR138C	YBR138C	YMR022W	UBC7
YOR109W	INP53	YPL037C	EGD1
YML001W	YPT7	YPR030W	CSR2
YKL135C	APL2	YOR069W	VPS5
YER115C	SPR6	YML109W	ZDS2
YMR133W	REC114	YMR214W	SCJ1
YMR055C	BUB2	YHR114W	BZZ1
YBR036C	CSG2	YDR310C	SUM1
YML103C	NUP188	YMR199W	CLN1
YML103C	NUP188	YJR090C	GRR1
YDL184C	RPL41A	YFL023W	BUD27
YLR320W	MMS22	YOR035C	SHE4
YML058W	SML1	YBL088C	TEL1
YBL103C	RTG3	YHR039C-A	VMA10
YPR049C	ATG11	YGL180W	ATG1
YOR290C	SNF2	YPL057C	SUR1
YJR135C	MCM22	YBR036C	CSG2
YOR069W	VPS5	YJR060W	CBF1
YIL036W	CST6	YNL116W	DMA2
YGL223C	COG1	YOR327C	SNC2

Predictions at threshold level 0.75 continued...			
Gene1 of predicted pair		Gene2 of predicted pair	
Systematic Name	Standard Name	Systematic Name	Standard Name
YDL184C	RPL41A	YHR060W	VMA22
YER114C	BOI2	YGL124C	MON1
YDL116W	NUP84	YLR386W	VAC14
YLR315W	NKP2	YAL002W	VPS8
YHR200W	RPN10	YHR081W	LRP1
YNL307C	MCK1	YMR055C	BUB2
YJL060W	BNA3	YJL058C	BIT61
YGL115W	SNF4	YDL226C	GCS1
YPR046W	MCM16	YDL047W	SIT4
YER116C	SLX8	YNL297C	MON2
YBR131W	CCZ1	YLR043C	TRX1
YFL013C	IES1	YPL129W	TAF14
YDL227C	HO	YLL013C	PUF3
YNL297C	MON2	YER120W	SCS2
YGR135W	PRE9	YJL183W	MNN11
YML058W	SML1	YKL114C	APN1
YNL325C	FIG4	YOR043W	WHI2
YLR386W	VAC14	YDR378C	LSM6
YEL051W	VMA8	YER122C	GLO3
YOR096W	RPS7A	YDR025W	RPS11A
YPL161C	BEM4	YER120W	SCS2
YFR010W	UBP6	YDR378C	LSM6
YEL065W	SIT1	YMR214W	SCJ1
YLR315W	NKP2	YMR038C	CCS1
YJR010C-A	SPC1	YML019W	OST6
YDR004W	RAD57	YCR086W	CSM1
YBR030W	YBR030W	YNL058C	YNL058C
YFL031W	HAC1	YOR036W	PEP12
YGR019W	UGA1	YPR051W	MAK3

Predictions at threshold level 0.75 continued...			
Gene1 of predicted pair		Gene2 of predicted pair	
Systematic Name	Standard Name	Systematic Name	Standard Name
YGR072W	UPF3	YAR014C	BUD14
YPL105C	YPL105C	YGL054C	ERV14
YMR198W	CIK1	YGR188C	BUB1
YOR112W	CEX1	YOR106W	VAM3
YBL102W	SFT2	YER122C	GLO3
YBR171W	SEC66	YNR072W	HXT17
YJR032W	CPR7	YEL060C	PRB1
YBR215W	HPC2	YER122C	GLO3
YEL065W	SIT1	YNL307C	MCK1
YBR215W	HPC2	YGL148W	ARO2
YNL215W	IES2	YHR167W	THP2
YGL031C	RPL24A	YDR207C	UME6
YPR101W	SNT309	YPL213W	LEA1
YOR195W	SLK19	YBR200W	BEM1
YDR004W	RAD57	YML102W	CAC2
YJR140C	HIR3	YER122C	GLO3
YOR270C	VPH1	YCR053W	THR4
YML103C	NUP188	YDR439W	LRS4
YJL127C	SPT10	YPL161C	BEM4
YHR134W	WSS1	YPL057C	SUR1
YNL166C	BNI5	YMR055C	BUB2
YGR104C	SRB5	YHR108W	GGA2
YJR140C	HIR3	YLR350W	ORM2
YMR198W	CIK1	YMR202W	ERG2
YFR010W	UBP6	YPR120C	CLB5
YDL020C	RPN4	YPL161C	BEM4
YMR026C	PEX12	YNL238W	KEX2
YDR137W	RGP1	YMR311C	GLC8
YGL161C	YIP5	YJL124C	LSM1

Predictions at threshold level 0.75 continued...			
Gene1 of predicted pair		Gene2 of predicted pair	
Systematic Name	Standard Name	Systematic Name	Standard Name
YNL325C	FIG4	YER144C	UBP5
YDR363W-A	SEM1	YOR308C	SNU66
YDR293C	SSD1	YGR152C	RSR1
YBR158W	AMN1	YLR309C	IMH1
YDR144C	MKC7	YOL067C	RTG1
YGL212W	VAM7	YMR311C	GLC8
YKL110C	KTI12	YGL252C	RTG2
YHR119W	SET1	YGR166W	KRE11
YPR120C	CLB5	YOL081W	IRA2
YPL115C	BEM3	YDR379W	RGA2
YGR135W	PRE9	YLR425W	TUS1
YLR386W	VAC14	YLR233C	EST1
YBL102W	SFT2	YER120W	SCS2
YMR224C	MRE11	YOR066W	MSA1
YLR018C	POM34	YKR095W	MLP1
YJR084W	CSN12	YDR207C	UME6
YDR004W	RAD57	YJR084W	CSN12
YNL069C	RPL16B	YNL107W	YAF9
YNL199C	GCR2	YNL079C	TPM1
YDR071C	PAA1	YBR010W	HHT1
YGR108W	CLB1	YMR055C	BUB2
YML103C	NUP188	YKL139W	CTK1
YOR033C	EXO1	YMR224C	MRE11
YOR127W	RGA1	YLR233C	EST1
YHR111W	UBA4	YEL056W	HAT2
YBR107C	IML3	YGR054W	YGR054W
YKR035W-A	DID2	YDR261C	EXG2
YIL005W	EPS1	YHR200W	RPN10
YGL025C	PGD1	YDL005C	MED2

Predictions at threshold level 0.75 continued...			
Gene1 of predicted pair		Gene2 of predicted pair	
Systematic Name	Standard Name	Systematic Name	Standard Name
YJL004C	SYS1	YOR327C	SNC2
YOR197W	MCA1	YPR154W	PIN3
YLR447C	VMA6	YPR046W	MCM16
YNL325C	FIG4	YML001W	YPT7
YMR224C	MRE11	YOL054W	PSH1
YPL024W	RMI1	YBR107C	IML3
YGL253W	HXK2	YLR233C	EST1
YER139C	YER139C	YLR398C	SKI2
YER059W	PCL6	YPL219W	PCL8
YJL176C	SWI3	YAL002W	VPS8
YGR184C	UBR1	YDR335W	MSN5
YLR148W	PEP3	YLR360W	VPS38
YML095C	RAD10	YIL008W	URM1
YHR205W	SCH9	YGR078C	PAC10
YPR131C	NAT3	YLR079W	SIC1
YFR010W	UBP6	YHR156C	LIN1
YNL031C	HHT2	YBR009C	HHF1
YOR109W	INP53	YDL077C	VAM6
YNL291C	MID1	YDR320C	SWA2
YML016C	PPZ1	YGR270W	YTA7
YJR031C	GEA1	YDL226C	GCS1
YGL174W	BUD13	YDR440W	DOT1
YLR234W	TOP3	YLR381W	CTF3
YGR108W	CLB1	YPL253C	VIK1
YBR200W	BEM1	YDR137W	RGP1
YGL147C	RPL9A	YFR010W	UBP6
YAL024C	LTE1	YML001W	YPT7
YDR176W	NGG1	YBL003C	HTA2
YBR041W	FAT1	YBR082C	UBC4

Predictions at threshold level 0.75 continued...			
Gene1 of predicted pair		Gene2 of predicted pair	
Systematic Name	Standard Name	Systematic Name	Standard Name
YGR072W	UPF3	YDR108W	GSG1
YER162C	RAD4	YNL051W	COG5
YOR337W	TEA1	YFR036W	CDC26
YFL023W	BUD27	YMR230W	RPS10B
YKL048C	ELM1	YPL152W	RRD2
YGR184C	UBR1	YHR013C	ARD1
YGL025C	PGD1	YNL076W	MKS1
YOL062C	APM4	YJR058C	APS2
YDR254W	CHL4	YIR005W	IST3
YGL115W	SNF4	YDR153C	ENT5
YJL117W	PHO86	YGR135W	PRE9
YBR057C	MUM2	YML115C	VAN1
YOR132W	VPS17	YBR114W	RAD16
YBR057C	MUM2	YDR260C	SWM1
YBR108W	YBR108W	YMR127C	SAS2
YLR043C	TRX1	YLR396C	VPS33
YDR443C	SSN2	YCR084C	TUP1
YDL184C	RPL41A	YKR026C	GCN3
YLR058C	SHM2	YDR171W	HSP42
YDR202C	RAV2	YKL119C	VPH2
YPL256C	CLN2	YKL161C	YKL161C
YGR072W	UPF3	YJL036W	SNX4
YHR161C	YAP1801	YMR017W	SPO20
YDL116W	NUP84	YHR010W	RPL27A
YNL106C	INP52	YGR270W	YTA7
YNL106C	INP52	YOR141C	ARP8
YMR312W	ELP6	YBL071W-A	KTI11
YDR477W	SNF1	YKR035W-A	DID2
YGL212W	VAM7	YOR208W	PTP2

Predictions at threshold level 0.75 continued...			
Gene1 of predicted pair		Gene2 of predicted pair	
Systematic Name	Standard Name	Systematic Name	Standard Name
YBR019C	GAL10	YEL060C	PRB1
YOR197W	MCA1	YLR206W	ENT2
YDR320C	SWA2	YJR125C	ENT3
YKR020W	VPS51	YDR310C	SUM1
YML115C	VAN1	YHR200W	RPN10
YOR290C	SNF2	YHR081W	LRP1
YMR055C	BUB2	YDR155C	CPR1
YBR108W	YBR108W	YNL238W	KEX2
YKL139W	CTK1	YBR019C	GAL10
YBR114W	RAD16	YDR310C	SUM1
YOR270C	VPH1	YNL238W	KEX2
YEL056W	HAT2	YNL107W	YAF9
YMR224C	MRE11	YML097C	VPS9
YHR052W	CIC1	YIL133C	RPL16A
YEL009C	GCN4	YHR079C	IRE1
YHL020C	OPI1	YGL115W	SNF4
YNL032W	SIW14	YDL184C	RPL41A
YMR142C	RPL13B	YOL111C	MDY2
YDR469W	SDC1	YER122C	GLO3
YKR059W	TIF1	YLR234W	TOP3
YNL215W	IES2	YHR111W	UBA4
YBR217W	ATG12	YPL149W	ATG5
YBR195C	MSI1	YGL151W	NUT1
YPR167C	MET16	YGR209C	TRX2
YLR079W	SIC1	YJR005W	APL1
YBR057C	MUM2	YJL095W	BCK1
YOR033C	EXO1	YBR082C	UBC4
YDR320C	SWA2	YNL127W	FAR11
YKR001C	VPS1	YNL127W	FAR11

Predictions at threshold level 0.75 continued...			
Gene1 of predicted pair		Gene2 of predicted pair	
Systematic Name	Standard Name	Systematic Name	Standard Name
YIL076W	SEC28	YEL060C	PRB1
YJR083C	ACF4	YLR144C	ACF2
YNL139C	RLR1	YNL004W	HRB1
YNL025C	SSN8	YLR182W	SWI6
YGL033W	HOP2	YGR249W	MGA1
YFL023W	BUD27	YHR039C-A	VMA10
YBL058W	SHP1	YNL032W	SIW14
YNL032W	SIW14	YBR108W	YBR108W
YLR441C	RPS1A	YDL223C	HBT1
YBR200W	BEM1	YEL043W	YEL043W
YLR216C	CPR6	YHR013C	ARD1
YIL159W	BNR1	YLR192C	HCR1
YMR224C	MRE11	YLR352W	YLR352W
YPL152W	RRD2	YOR141C	ARP8
YBR217W	ATG12	YNR072W	HXT17
YGL153W	PEX14	YNL079C	TPM1
YML038C	YMD8	YDR207C	UME6
YJR140C	HIR3	YDR378C	LSM6
YFR010W	UBP6	YDR254W	CHL4
YKL010C	UFD4	YFR010W	UBP6
YMR224C	MRE11	YDR254W	CHL4
YOR337W	TEA1	YNR072W	HXT17
YDR080W	VPS41	YML038C	YMD8
YLR119W	SRN2	YJL095W	BCK1
YAL024C	LTE1	YEL053C	MAK10
YBR200W	BEM1	YDR363W-A	SEM1
YLR093C	NYV1	YEL036C	ANP1
YLR079W	SIC1	YFL047W	RGD2
YPL240C	HSP82	YHR071W	PCL5

Predictions at threshold level 0.75 continued...			
Gene1 of predicted pair		Gene2 of predicted pair	
Systematic Name	Standard Name	Systematic Name	Standard Name
YPL140C	MKK2	YLR313C	SPH1
YIL076W	SEC28	YBL103C	RTG3
YML074C	FPR3	YGL019W	CKB1
YGL147C	RPL9A	YML115C	VAN1
YNL044W	YIP3	YDL192W	ARF1
YJR056C	YJR056C	YGL212W	VAM7
YEL050C	RML2	YLR429W	CRN1
YNL206C	RTT106	YNL107W	YAF9
YGR072W	UPF3	YIL076W	SEC28
YLR083C	EMP70	YPR084W	YPR084W
YDR245W	MNN10	YPL057C	SUR1
YJR140C	HIR3	YFR021W	ATG18
YDR156W	RPA14	YAR014C	BUD14
YNL218W	MGS1	YOR033C	EXO1
YDL223C	HBT1	YEL036C	ANP1
YDL223C	HBT1	YER139C	YER139C
YLR095C	IOC2	YPR036W	VMA13
YDR320C	SWA2	YKR030W	GMH1
YPR051W	MAK3	YPL105C	YPL105C
YMR224C	MRE11	YJL176C	SWI3
YJL204C	RCY1	YNR049C	MSO1
YNL291C	MID1	YJL004C	SYS1
YOR043W	WHI2	YLR320W	MMS22
YDL020C	RPN4	YGL087C	MMS2
YOR132W	VPS17	YOR023C	AHC1
YPL096C-A	ERI1	YGR229C	SMI1
YFR031C-A	RPL2A	YPR030W	CSR2
YCR020C-A	MAK31	YPL105C	YPL105C
YGL212W	VAM7	YNL058C	YNL058C

Predictions at threshold level 0.75 continued...			
Gene1 of predicted pair		Gene2 of predicted pair	
Systematic Name	Standard Name	Systematic Name	Standard Name
YDR004W	RAD57	YLR352W	YLR352W
YLR095C	IOC2	YML071C	COG8
YHR216W	IMD2	YGR262C	BUD32
YLR315W	NKP2	YPL018W	CTF19
YLR360W	VPS38	YKR014C	YPT52
YOR127W	RGA1	YGL003C	CDH1
YOR337W	TEA1	YOR304W	ISW2
YPL129W	TAF14	YNL027W	CRZ1
YDR181C	SAS4	YOR213C	SAS5
YDR484W	VPS52	YDL083C	RPS16B
YDR289C	RTT103	YDR183W	PLP1
YGR020C	VMA7	YMR054W	STV1
YKL139W	CTK1	YDR358W	GGA1
YEL009C	GCN4	YDL005C	MED2
YML016C	PPZ1	YIR005W	IST3
YDR254W	CHL4	YLR410W	VIP1
YDR440W	DOT1	YLR425W	TUS1
YDR422C	SIP1	YPR119W	CLB2
YBR200W	BEM1	YLR120C	YPS1
YBR107C	IML3	YPR119W	CLB2
YGL190C	CDC55	YPL256C	CLN2
YBR215W	HPC2	YDR137W	RGP1
YDR004W	RAD57	YHR178W	STB5
YBR200W	BEM1	YJL172W	CPS1
YPL253C	VIK1	YER096W	SHC1
YMR142C	RPL13B	YEL060C	PRB1
YBR200W	BEM1	YHL031C	GOS1
YGL147C	RPL9A	YBR036C	CSG2
YLR234W	TOP3	YML095C	RAD10

Predictions at threshold level 0.75 continued...			
Gene1 of predicted pair		Gene2 of predicted pair	
Systematic Name	Standard Name	Systematic Name	Standard Name
YHR205W	SCH9	YGL086W	MAD1
YBR173C	UMP1	YJR032W	CPR7
YNL041C	COG6	YMR054W	STV1
YLR292C	SEC72	YAR014C	BUD14
YKL139W	CTK1	YHL031C	GOS1
YLR337C	VRP1	YBL021C	HAP3
YGL178W	MPT5	YPR120C	CLB5
YKL116C	PRR1	YDL077C	VAM6
YDL020C	RPN4	YOR132W	VPS17
YGL174W	BUD13	YOR124C	UBP2
YBL051C	PIN4	YGL124C	MON1
YBR019C	GAL10	YLR398C	SKI2
YNL291C	MID1	YML038C	YMD8
YNL215W	IES2	YOR124C	UBP2
YLR360W	VPS38	YGR054W	YGR054W
YGR104C	SRB5	YNL107W	YAF9
YDL216C	RRI1	YMR025W	CSI1
YOR106W	VAM3	YDR153C	ENT5
YDR254W	CHL4	YLR452C	SST2
YDR335W	MSN5	YBR107C	IML3
YPL024W	RMI1	YJR056C	YJR056C
YDR383C	NKP1	YIL036W	CST6
YDR174W	HMO1	YAR014C	BUD14
YJL053W	PEP8	YER120W	SCS2
YMR153W	NUP53	YLR398C	SKI2
YJR040W	GEF1	YPL195W	APL5
YNL288W	CAF40	YNL166C	BNI5
YPR084W	YPR084W	YNL069C	RPL16B
YDL002C	NHP10	YLR315W	NKP2

Predictions at threshold level 0.75 continued...			
Gene1 of predicted pair		Gene2 of predicted pair	
Systematic Name	Standard Name	Systematic Name	Standard Name
YEL053C	MAK10	YOR066W	MSA1
YPR046W	MCM16	YOR014W	RTS1
YGL115W	SNF4	YAR014C	BUD14
YDR245W	MNN10	YIR005W	IST3
YDL042C	SIR2	YHR061C	GIC1
YPL253C	VIK1	YNL166C	BNI5
YOR032C	HMS1	YLR234W	TOP3
YPL195W	APL5	YAR014C	BUD14
YAL024C	LTE1	YLR254C	NDL1
YKL116C	PRR1	YMR016C	SOK2
YDR183W	PLP1	YGL151W	NUT1
YNL068C	FKH2	YBL087C	RPL23A
YOR112W	CEX1	YPR051W	MAK3
YPL129W	TAF14	YLR357W	RSC2
YDR183W	PLP1	YHR114W	BZZ1
YOR290C	SNF2	YOL111C	MDY2
YCL037C	SRO9	YHR060W	VMA22
YNL215W	IES2	YHR200W	RPN10
YDL116W	NUP84	YMR153W	NUP53
YIL036W	CST6	YNL225C	CNM67
YMR202W	ERG2	YNL107W	YAF9
YML013W	UBX2	YOR368W	RAD17
YPL115C	BEM3	YDR309C	GIC2
YNL297C	MON2	YGR188C	BUB1
YPR120C	CLB5	YNL236W	SIN4
YBR217W	ATG12	YOL121C	RPS19A
YEL050C	RML2	YMR198W	CIK1
YDL223C	HBT1	YDR207C	UME6
YDR318W	MCM21	YDL226C	GCS1

Predictions at threshold level 0.75 continued...			
Gene1 of predicted pair		Gene2 of predicted pair	
Systematic Name	Standard Name	Systematic Name	Standard Name
YPR101W	SNT309	YGR006W	PRP18
YNL032W	SIW14	YPL137C	GIP3
YBL008W	HIR1	YOR043W	WHI2
YDR108W	GSG1	YDR080W	VPS41
YGL066W	SGF73	YMR198W	CIK1
YJR059W	PTK2	YGL016W	KAP122
YER001W	MNN1	YPL050C	MNN9
YMR153W	NUP53	YGL115W	SNF4
YOR043W	WHI2	YOR308C	SNU66
YLR234W	TOP3	YBR278W	DPB3
YMR172W	HOT1	YOR127W	RGA1
YMR054W	STV1	YPL195W	APL5
YOL126C	MDH2	YGR059W	SPR3
YMR216C	SKY1	YPL234C	TFP3
YLL007C	YLL007C	YGR054W	YGR054W
YGL174W	BUD13	YPL213W	LEA1
YHR039C-A	VMA10	YMR152W	YIM1
YDR318W	MCM21	YJL095W	BCK1
YFR010W	UBP6	YOL121C	RPS19A
YPR046W	MCM16	YMR038C	CCS1
YOR069W	VPS5	YGL054C	ERV14
YOR127W	RGA1	YHR114W	BZZ1
YGL115W	SNF4	YJR033C	RAV1
YDR477W	SNF1	YER139C	YER139C
YML115C	VAN1	YDL155W	CLB3
YBR127C	VMA2	YMR231W	PEP5
YNL054W	VAC7	YHR111W	UBA4
YGL223C	COG1	YFL031W	HAC1
YDR108W	GSG1	YOR043W	WHI2

Predictions at threshold level 0.75 continued...			
Gene1 of predicted pair		Gene2 of predicted pair	
Systematic Name	Standard Name	Systematic Name	Standard Name
YGL161C	YIP5	YKR014C	YPT52
YPL105C	YPL105C	YGL115W	SNF4
YMR179W	SPT21	YDL226C	GCS1
YDR289C	RTT103	YDR310C	SUM1
YLR292C	SEC72	YGL124C	MON1
YNL325C	FIG4	YPL195W	APL5
YGR249W	MGA1	YKL126W	YPK1
YPL120W	VPS30	YDL077C	VAM6
YAL056W	GPB2	YOR371C	GPB1
YFR031C-A	RPL2A	YBR108W	YBR108W
YBR108W	YBR108W	YBR107C	IML3
YGL115W	SNF4	YLR093C	NYV1
YPR036W	VMA13	YPR120C	CLB5
YNL215W	IES2	YFR010W	UBP6
YKL126W	YPK1	YLR452C	SST2
YKL007W	CAP1	YBL103C	RTG3
YLR061W	RPL22A	YLR192C	HCR1
YBR274W	CHK1	YGL094C	PAN2
YBL058W	SHP1	YMR016C	SOK2
YGL060W	YBP2	YML007W	YAP1
YLR337C	VRP1	YEL044W	IES6
YNL058C	YNL058C	YDR358W	GGA1
YNL307C	MCK1	YIL103W	DPH1
YDL083C	RPS16B	YJR032W	CPR7
YBR200W	BEM1	YJR063W	RPA12
YHR134W	WSS1	YHL002W	HSE1
YPL024W	RMI1	YHR039C-A	VMA10
YLR148W	PEP3	YPR028W	YOP1
YLR150W	STM1	YDL013W	SLX5

Predictions at threshold level 0.75 continued...			
Gene1 of predicted pair		Gene2 of predicted pair	
Systematic Name	Standard Name	Systematic Name	Standard Name
YKR020W	VPS51	YML097C	VPS9
YBL047C	EDE1	YOR275C	RIM20
YBR194W	SOY1	YMR055C	BUB2
YNL329C	PEX6	YBL025W	RRN10
YMR224C	MRE11	YPL018W	CTF19
YOL148C	SPT20	YNL107W	YAF9
YBR019C	GAL10	YOL081W	IRA2
YMR224C	MRE11	YPR046W	MCM16
YDR146C	SWI5	YCR077C	PAT1
YJL095W	BCK1	YML028W	TSA1
YFR010W	UBP6	YBL025W	RRN10
YGR270W	YTA7	YLR182W	SWI6
YDR320C	SWA2	YAR014C	BUD14
YBR200W	BEM1	YKR061W	KTR2
YBR258C	SHG1	YOR106W	VAM3
YMR164C	MSS11	YPL256C	CLN2
YNL041C	COG6	YGR105W	VMA21
YGR104C	SRB5	YOL145C	CTR9
YOR069W	VPS5	YEL053C	MAK10
YNL069C	RPL16B	YPL195W	APL5
YHR012W	VPS29	YJL190C	RPS22A
YOR106W	VAM3	YOR109W	INP53
YMR224C	MRE11	YLR386W	VAC14
YKR014C	YPT52	YBL106C	SRO77
YOL018C	TLG2	YNR032W	PPG1
YLL039C	UBI4	YLR370C	ARC18
YDR320C	SWA2	YER162C	RAD4
YOR195W	SLK19	YDR289C	RTT103
YLR441C	RPS1A	YMR152W	YIM1

Predictions at threshold level 0.75 continued...			
Gene1 of predicted pair		Gene2 of predicted pair	
**Systematic Name**	**Standard Name**	**Systematic Name**	**Standard Name**
YPL024W	RMI1	YPL057C	SUR1
YKL139W	CTK1	YCR077C	PAT1
YDR309C	GIC2	YNL293W	MSB3
YBR173C	UMP1	YDL077C	VAM6
YPL161C	BEM4	YPL120W	VPS30
YHR203C	RPS4B	YBR189W	RPS9B
YJL058C	BIT61	YBR107C	IML3
YMR026C	PEX12	YER040W	GLN3
YOR076C	SKI7	YOR001W	RRP6
YKR061W	KTR2	YOR141C	ARP8
YGL025C	PGD1	YNL031C	HHT2
YLR061W	RPL22A	YLR360W	VPS38
YJR099W	YUH1	YDR358W	GGA1
YDL106C	PHO2	YDR146C	SWI5
YNL041C	COG6	YNL107W	YAF9
YDL020C	RPN4	YLR182W	SWI6
YIL156W	UBP7	YIL148W	RPL40A
YEL050C	RML2	YOR195W	SLK19
YJL124C	LSM1	YER161C	SPT2
YLR090W	XDJ1	YBR036C	CSG2
YDL116W	NUP84	YLL026W	HSP104
YDR448W	ADA2	YBR111W-A	SUS1
YDL216C	RRI1	YJR084W	CSN12
YKL116C	PRR1	YOL081W	IRA2
YML071C	COG8	YJL183W	MNN11
YDR439W	LRS4	YDR289C	RTT103
YKL048C	ELM1	YGL003C	CDH1
YJL172W	CPS1	YPR030W	CSR2
YPL042C	SSN3	YOR106W	VAM3

Predictions at threshold level 0.75 continued...			
Gene1 of predicted pair		Gene2 of predicted pair	
Systematic Name	Standard Name	Systematic Name	Standard Name
YNL215W	IES2	YPR046W	MCM16
YHR013C	ARD1	YOR035C	SHE4
YBR278W	DPB3	YDR146C	SWI5
YPL161C	BEM4	YJL004C	SYS1
YJL187C	SWE1	YOR327C	SNC2
YKR072C	SIS2	YHR134W	WSS1
YBR030W	YBR030W	YKL048C	ELM1
YEL009C	GCN4	YDR225W	HTA1
YNR052C	POP2	YNL206C	RTT106
YKR059W	TIF1	YBR215W	HPC2
YJL154C	VPS35	YDL226C	GCS1
YGL175C	SAE2	YCR086W	CSM1
YCR020C-A	MAK31	YKR030W	GMH1
YGL174W	BUD13	YCL037C	SRO9
YPL115C	BEM3	YHR061C	GIC1
YDR448W	ADA2	YBR010W	HHT1
YPL089C	RLM1	YBL101C	ECM21
YDR477W	SNF1	YDL083C	RPS16B
YPR017C	DSS4	YLR360W	VPS38
YDR129C	SAC6	YMR116C	ASC1
YPR046W	MCM16	YPL018W	CTF19
YEL065W	SIT1	YPR030W	CSR2
YMR224C	MRE11	YKL048C	ELM1
YGL054C	ERV14	YDR310C	SUM1
YJR040W	GEF1	YNL051W	COG5
YLL007C	YLL007C	YGL087C	MMS2
YNL307C	MCK1	YPR030W	CSR2
YEL065W	SIT1	YJL183W	MNN11
YPL037C	EGD1	YGL054C	ERV14

Predictions at threshold level 0.75 continued...			
Gene1 of predicted pair		Gene2 of predicted pair	
Systematic Name	Standard Name	Systematic Name	Standard Name
YDR004W	RAD57	YGL100W	SEH1
YLR079W	SIC1	YMR202W	ERG2
YNL054W	VAC7	YDR183W	PLP1
YMR153W	NUP53	YMR125W	STO1
YJR032W	CPR7	YLR371W	ROM2
YFR036W	CDC26	YJL183W	MNN11
YLR061W	RPL22A	YBL047C	EDE1
YJR032W	CPR7	YGL253W	HXK2
YAL024C	LTE1	YER044C	ERG28
YNL041C	COG6	YLR180W	SAM1
YBR171W	SEC66	YHR039C-A	VMA10
YOR270C	VPH1	YOR066W	MSA1
YBR107C	IML3	YDL185W	TFP1
YGL003C	CDH1	YGR188C	BUB1
YLR083C	EMP70	YML038C	YMD8
YOR284W	HUA2	YKR030W	GMH1
YPL120W	VPS30	YOL108C	INO4
YBR172C	SMY2	YKL074C	MUD2
YMR142C	RPL13B	YNL041C	COG6
YDR320C	SWA2	YCL001W	RER1
YPR084W	YPR084W	YDR137W	RGP1
YAL024C	LTE1	YGL148W	ARO2
YDR448W	ADA2	YNL030W	HHF2
YPL018W	CTF19	YAL002W	VPS8
YDR261C	EXG2	YPL195W	APL5
YHL025W	SNF6	YER161C	SPT2
YNL297C	MON2	YER122C	GLO3
YNL032W	SIW14	YPR046W	MCM16
YOR132W	VPS17	YBL051C	PIN4

Predictions at threshold level 0.75 continued...			
Gene1 of predicted pair		Gene2 of predicted pair	
Systematic Name	Standard Name	Systematic Name	Standard Name
YNL218W	MGS1	YLR135W	SLX4
YML016C	PPZ1	YJL095W	BCK1
YGR249W	MGA1	YDL083C	RPS16B
YIR005W	IST3	YCR077C	PAT1
YMR152W	YIM1	YJL183W	MNN11
YBL008W	HIR1	YEL056W	HAT2
YCL037C	SRO9	YGR105W	VMA21
YGR270W	YTA7	YOR124C	UBP2
YOR127W	RGA1	YBL047C	EDE1
YBL021C	HAP3	YHR111W	UBA4
YOR043W	WHI2	YMR037C	MSN2
YNL201C	PSY2	YDR383C	NKP1
YJL154C	VPS35	YOR127W	RGA1
YFR024C-A	LSB3	YPR171W	BSP1
YHR098C	SFB3	YNL107W	YAF9
YIR009W	MSL1	YBR114W	RAD16
YGR105W	VMA21	YOL108C	INO4
YDR245W	MNN10	YNL199C	GCR2
YDR309C	GIC2	YCR086W	CSM1
YIL076W	SEC28	YJL036W	SNX4
YMR199W	CLN1	YBR084C-A	RPL19A
YOR014W	RTS1	YEL036C	ANP1
YKL130C	SHE2	YBR130C	SHE3
YPR154W	PIN3	YPR119W	CLB2
YPR084W	YPR084W	YBR215W	HPC2
YEL015W	EDC3	YBL066C	SEF1
YGL134W	PCL10	YIL050W	PCL7
YDR484W	VPS52	YOL081W	IRA2
YBR127C	VMA2	YNL259C	ATX1

Predictions at threshold level 0.75 continued...			
Gene1 of predicted pair		Gene2 of predicted pair	
Systematic Name	Standard Name	Systematic Name	Standard Name
YMR153W	NUP53	YML001W	YPT7
YBR200W	BEM1	YER096W	SHC1
YOR023C	AHC1	YBR114W	RAD16
YOR083W	WHI5	YDR318W	MCM21
YJL154C	VPS35	YMR109W	MYO5
YKL197C	PEX1	YNL329C	PEX6
YBR036C	CSG2	YER040W	GLN3
YGR100W	MDR1	YNL044W	YIP3
YMR070W	MOT3	YBL047C	EDE1
YNL218W	MGS1	YPL057C	SUR1
YGR249W	MGA1	YFR036W	CDC26
YJR032W	CPR7	YGL019W	CKB1
YPR173C	VPS4	YJL004C	SYS1
YNL058C	YNL058C	YLR180W	SAM1
YPL246C	RBD2	YMR253C	YMR253C
YPR029C	APL4	YDL192W	ARF1
YPL001W	HAT1	YGL163C	RAD54
YMR216C	SKY1	YGR020C	VMA7
YGL135W	RPL1B	YLR192C	HCR1
YGL223C	COG1	YCR053W	THR4
YMR179W	SPT21	YML028W	TSA1
YHR039C-A	VMA10	YGL151W	NUT1
YPR051W	MAK3	YGL054C	ERV14
YDL184C	RPL41A	YJL124C	LSM1
YOR270C	VPH1	YLR320W	MMS22
YOR270C	VPH1	YFL023W	BUD27
YER042W	MXR1	YML028W	TSA1
YGL200C	EMP24	YDR363W-A	SEM1
YLR095C	IOC2	YHR156C	LIN1

Predictions at threshold level 0.75 continued...			
Gene1 of predicted pair		Gene2 of predicted pair	
Systematic Name	Standard Name	Systematic Name	Standard Name
YJL053W	PEP8	YMR231W	PEP5
YJR056C	YJR056C	YHR060W	VMA22
YBL103C	RTG3	YDR310C	SUM1
YPL152W	RRD2	YLR182W	SWI6
YML115C	VAN1	YOL121C	RPS19A
YDR137W	RGP1	YMR214W	SCJ1
YMR026C	PEX12	YGR239C	PEX21
YML013W	UBX2	YOR275C	RIM20
YNL238W	KEX2	YDR153C	ENT5
YBL101C	ECM21	YBL072C	RPS8A
YOR270C	VPH1	YML001W	YPT7
YPL050C	MNN9	YEL036C	ANP1
YML112W	CTK3	YOR141C	ARP8
YDR004W	RAD57	YFR010W	UBP6
YJR032W	CPR7	YBR019C	GAL10
YAL024C	LTE1	YDL020C	RPN4
YDR293C	SSD1	YNL297C	MON2
YKL025C	PAN3	YHR178W	STB5
YGL066W	SGF73	YDL047W	SIT4
YOR080W	DIA2	YKL041W	VPS24
YPL178W	CBC2	YGR059W	SPR3
YKL007W	CAP1	YJL187C	SWE1
YFR031C-A	RPL2A	YBR173C	UMP1
YFR010W	UBP6	YOR290C	SNF2
YMR179W	SPT21	YGR184C	UBR1
YPL057C	SUR1	YPL161C	BEM4
YBR200W	BEM1	YOR327C	SNC2
YMR199W	CLN1	YHR111W	UBA4
YGL147C	RPL9A	YPL161C	BEM4

Predictions at threshold level 0.75 continued...			
Gene1 of predicted pair		Gene2 of predicted pair	
Systematic Name	Standard Name	Systematic Name	Standard Name
YNL025C	SSN8	YHL031C	GOS1
YNL069C	RPL16B	YPL234C	TFP3
YLR360W	VPS38	YMR198W	CIK1
YJL036W	SNX4	YJL004C	SYS1
YKR001C	VPS1	YGL054C	ERV14
YKL139W	CTK1	YOR312C	RPL20B
YNL069C	RPL16B	YEL053C	MAK10
YOR132W	VPS17	YOR127W	RGA1
YGR270W	YTA7	YEL013W	VAC8
YNR072W	HXT17	YJL095W	BCK1
YGR188C	BUB1	YGL252C	RTG2
YDR108W	GSG1	YGR020C	VMA7
YLR058C	SHM2	YJL058C	BIT61
YDR335W	MSN5	YOL081W	IRA2
YPR154W	PIN3	YPL249C	GYP5
YOR327C	SNC2	YLR425W	TUS1
YOR066W	MSA1	YPL137C	GIP3
YIL034C	CAP2	YER114C	BOI2
YLR079W	SIC1	YJR035W	RAD26
YIL076W	SEC28	YIR005W	IST3
YIL053W	RHR2	YOR066W	MSA1
YGR261C	APL6	YPL037C	EGD1
YJR140C	HIR3	YCR086W	CSM1
YML115C	VAN1	YGL212W	VAM7
YEL009C	GCN4	YHL025W	SNF6
YLR360W	VPS38	YGR270W	YTA7
YNL243W	SLA2	YPL240C	HSP82
YGR261C	APL6	YOR132W	VPS17
YGR270W	YTA7	YIL008W	URM1

Predictions at threshold level 0.75 continued...			
Gene1 of predicted pair		Gene2 of predicted pair	
Systematic Name	Standard Name	Systematic Name	Standard Name
YGL249W	ZIP2	YPR119W	CLB2
YLR410W	VIP1	YNL225C	CNM67
YLR170C	APS1	YDL192W	ARF1
YOR039W	CKB2	YMR055C	BUB2
YOR001W	RRP6	YDR363W-A	SEM1
YHR178W	STB5	YPL161C	BEM4
YDL002C	NHP10	YDR363W-A	SEM1
YKL214C	YRA2	YML062C	MFT1
YOR270C	VPH1	YDL185W	TFP1
YFL023W	BUD27	YLR093C	NYV1
YMR311C	GLC8	YAR014C	BUD14
YGR019W	UGA1	YNL288W	CAF40
YNL325C	FIG4	YJR032W	CPR7
YBL102W	SFT2	YFL033C	RIM15
YKR035W-A	DID2	YML071C	COG8
YHR016C	YSC84	YHR114W	BZZ1
YNL106C	INP52	YLR399C	BDF1
YLR360W	VPS38	YDR146C	SWI5
YIL159W	BNR1	YMR032W	HOF1
YNL032W	SIW14	YJR084W	CSN12
YCL029C	BIK1	YIL033C	BCY1
YDL035C	GPR1	YGL121C	GPG1
YGL115W	SNF4	YNL076W	MKS1
YDL006W	PTC1	YLR309C	IMH1
YJL004C	SYS1	YOL018C	TLG2
YEL065W	SIT1	YER144C	UBP5
YPR018W	RLF2	YMR231W	PEP5
YNL307C	MCK1	YJR035W	RAD26
YPR084W	YPR084W	YGR072W	UPF3

Predictions at threshold level 0.75 continued...			
**Gene1 of predicted pair**		**Gene2 of predicted pair**	
**Systematic Name**	**Standard Name**	**Systematic Name**	**Standard Name**
YPR036W	VMA13	YHR060W	VMA22
YMR284W	YKU70	YBL079W	NUP170
YMR026C	PEX12	YML071C	COG8
YOL054W	PSH1	YGR188C	BUB1
YPL057C	SUR1	YHR060W	VMA22
YML016C	PPZ1	YDR155C	CPR1
YDR076W	RAD55	YMR038C	CCS1
YNL025C	SSN8	YBR215W	HPC2
YBR108W	YBR108W	YOR043W	WHI2
YOR141C	ARP8	YNL059C	ARP5
YMR164C	MSS11	YFL047W	RGD2
YDR443C	SSN2	YEL036C	ANP1
YJR040W	GEF1	YNL107W	YAF9
YDR320C	SWA2	YPL105C	YPL105C
YPL096C-A	ERI1	YPL240C	HSP82
YKL048C	ELM1	YLR182W	SWI6
YKR020W	VPS51	YOR327C	SNC2
YDR310C	SUM1	YLR180W	SAM1
YPL246C	RBD2	YHR114W	BZZ1
YDR378C	LSM6	YLR254C	NDL1
YIL148W	RPL40A	YKL117W	SBA1
YGL200C	EMP24	YHL002W	HSE1
YJL053W	PEP8	YEL053C	MAK10
YHR178W	STB5	YHR200W	RPN10
YKR048C	NAP1	YJL095W	BCK1
YLR381W	CTF3	YDL047W	SIT4
YHR205W	SCH9	YJR075W	HOC1
YGL033W	HOP2	YMR214W	SCJ1
YKR099W	BAS1	YBR171W	SEC66

Predictions at threshold level 0.75 continued...			
Gene1 of predicted pair		Gene2 of predicted pair	
Systematic Name	Standard Name	Systematic Name	Standard Name
YGR135W	PRE9	YJL140W	RPB4
YNL329C	PEX6	YOR312C	RPL20B
YGL078C	DBP3	YNL041C	COG6
YGR270W	YTA7	YPL161C	BEM4
YDL223C	HBT1	YPL037C	EGD1
YDL083C	RPS16B	YDL192W	ARF1
YPR051W	MAK3	YOL018C	TLG2
YMR198W	CIK1	YLR381W	CTF3
YKL116C	PRR1	YDR137W	RGP1
YLR018C	POM34	YGR105W	VMA21
YGR056W	RSC1	YHR056C	RSC30
YBL019W	APN2	YAL015C	NTG1
YDR155C	CPR1	YGL253W	HXK2
YNL291C	MID1	YMR216C	SKY1
YNL087W	TCB2	YDR310C	SUM1
YNL139C	RLR1	YMR273C	ZDS1
YHR178W	STB5	YHR039C-A	VMA10
YBR108W	YBR108W	YLR371W	ROM2
YBL103C	RTG3	YNL041C	COG6
YOR033C	EXO1	YLR410W	VIP1
YGL174W	BUD13	YER122C	GLO3
YNL106C	INP52	YPR046W	MCM16
YNL243W	SLA2	YOR332W	VMA4
YBR171W	SEC66	YOL121C	RPS19A
YMR198W	CIK1	YER122C	GLO3
YNL307C	MCK1	YEL060C	PRB1
YER120W	SCS2	YOR327C	SNC2
YNR072W	HXT17	YBL025W	RRN10
YIL053W	RHR2	YDL192W	ARF1

Predictions at threshold level 0.75 continued...			
Gene1 of predicted pair		Gene2 of predicted pair	
Systematic Name	Standard Name	Systematic Name	Standard Name
YBR200W	BEM1	YJL006C	CTK2
YMR016C	SOK2	YER162C	RAD4
YPL234C	TFP3	YOL018C	TLG2
YBR126C	TPS1	YEL060C	PRB1
YBL008W	HIR1	YJL127C	SPT10
YMR142C	RPL13B	YNL325C	FIG4
YPR051W	MAK3	YPL195W	APL5
YBR280C	SAF1	YER040W	GLN3
YML102W	CAC2	YMR231W	PEP5
YDR484W	VPS52	YPL045W	VPS16
YLL039C	UBI4	YJL157C	FAR1
YAL024C	LTE1	YNL248C	RPA49
YMR153W	NUP53	YFL023W	BUD27
YBR274W	CHK1	YGL019W	CKB1
YBL058W	SHP1	YGL078C	DBP3
YGR261C	APL6	YDR202C	RAV2
YML095C	RAD10	YBR171W	SEC66
YDR443C	SSN2	YLR425W	TUS1
YPL253C	VIK1	YOR358W	HAP5
YHR060W	VMA22	YMR311C	GLC8
YLR095C	IOC2	YNL087W	TCB2
YKL025C	PAN3	YMR172W	HOT1
YGL054C	ERV14	YAL007C	ERP2
YGL147C	RPL9A	YHR200W	RPN10
YBR195C	MSI1	YBR036C	CSG2
YNL201C	PSY2	YGL163C	RAD54
YNL218W	MGS1	YNL016W	PUB1
YHR200W	RPN10	YJL183W	MNN11
YHR200W	RPN10	YPR030W	CSR2

Predictions at threshold level 0.75 continued...			
Gene1 of predicted pair		Gene2 of predicted pair	
Systematic Name	Standard Name	Systematic Name	Standard Name
YKL139W	CTK1	YJL095W	BCK1
YGR072W	UPF3	YOL018C	TLG2
YMR109W	MYO5	YGR188C	BUB1
YIL036W	CST6	YPR046W	MCM16
YHL031C	GOS1	YPR032W	SRO7
YDR245W	MNN10	YER122C	GLO3
YDR358W	GGA1	YLR180W	SAM1
YHR178W	STB5	YGL161C	YIP5
YGL163C	RAD54	YJR090C	GRR1
YGL060W	YBP2	YDR171W	HSP42
YML016C	PPZ1	YEL056W	HAT2
YBR127C	VMA2	YLR447C	VMA6
YNL106C	INP52	YKL110C	KTI12
YNL307C	MCK1	YLR410W	VIP1
YLR381W	CTF3	YBR171W	SEC66
YBR215W	HPC2	YPR120C	CLB5
YPL178W	CBC2	YML057W	CMP2
YGL060W	YBP2	YPL042C	SSN3
YCL010C	SGF29	YGL252C	RTG2
YLR386W	VAC14	YDR318W	MCM21
YBL025W	RRN10	YBR036C	CSG2
YER001W	MNN1	YDL223C	HBT1
YLR315W	NKP2	YOR308C	SNU66
YNL068C	FKH2	YNL116W	DMA2
YOR047C	STD1	YGL252C	RTG2
YAL024C	LTE1	YCR065W	HCM1
YBR194W	SOY1	YDR200C	VPS64
YBR030W	YBR030W	YKL126W	YPK1
YLR452C	SST2	YMR230W	RPS10B

Predictions at threshold level 0.75 continued...			
Gene1 of predicted pair		Gene2 of predicted pair	
Systematic Name	Standard Name	Systematic Name	Standard Name
YGR072W	UPF3	YHR134W	WSS1
YLR337C	VRP1	YGR054W	YGR054W
YDR254W	CHL4	YGL250W	RMR1
YNL016W	PUB1	YBL079W	NUP170
YKR014C	YPT52	YOR327C	SNC2
YMR095C	SNO1	YJL095W	BCK1
YOR109W	INP53	YMR054W	STV1
YOR035C	SHE4	YNL076W	MKS1
YGL060W	YBP2	YJL060W	BNA3
YOR127W	RGA1	YDR254W	CHL4
YNL307C	MCK1	YOR368W	RAD17
YBR107C	IML3	YLR447C	VMA6
YNL054W	VAC7	YDR289C	RTT103
YMR231W	PEP5	YPL195W	APL5
YPL161C	BEM4	YJL190C	RPS22A
YNL215W	IES2	YOR141C	ARP8
YBL008W	HIR1	YOR290C	SNF2
YDR378C	LSM6	YKL041W	VPS24
YFR031C-A	RPL2A	YLR093C	NYV1
YGR252W	GCN5	YPL158C	YPL158C
YBL008W	HIR1	YDR310C	SUM1
YMR153W	NUP53	YMR273C	ZDS1
YDR293C	SSD1	YBL021C	HAP3
YCR020C-A	MAK31	YNL041C	COG6
YHL031C	GOS1	YLR093C	NYV1
YNL291C	MID1	YBR107C	IML3
YHR081W	LRP1	YLR398C	SKI2
YDL116W	NUP84	YOL081W	IRA2
YNL236W	SIN4	YLR410W	VIP1

Predictions at threshold level 0.75 continued...			
Gene1 of predicted pair		Gene2 of predicted pair	
Systematic Name	Standard Name	Systematic Name	Standard Name
YPL037C	EGD1	YPR030W	CSR2
YOR270C	VPH1	YOR327C	SNC2
YDR477W	SNF1	YOR008C	SLG1
YBR194W	SOY1	YJL006C	CTK2
YJL006C	CTK2	YOL054W	PSH1
YBL058W	SHP1	YDL223C	HBT1
YJL136C	RPS21B	YKL032C	IXR1
YJR135C	MCM22	YDR076W	RAD55
YER162C	RAD4	YBR280C	SAF1
YDL116W	NUP84	YBR017C	KAP104
YML115C	VAN1	YEL044W	IES6
YPL161C	BEM4	YMR214W	SCJ1
YPR120C	CLB5	YDR071C	PAA1
YKL139W	CTK1	YPL018W	CTF19
YBL016W	FUS3	YBR083W	TEC1
YER116C	SLX8	YOL081W	IRA2
YDL077C	VAM6	YNL107W	YAF9
YBL067C	UBP13	YMR089C	YTA12
YPR046W	MCM16	YAR014C	BUD14
YKL139W	CTK1	YHR115C	DMA1
YPR089W	YPR089W	YHR012W	VPS29
YDL077C	VAM6	YAR014C	BUD14
YHR039C-A	VMA10	YDR289C	RTT103
YGR104C	SRB5	YOR308C	SNU66
YOR001W	RRP6	YPL057C	SUR1
YIL034C	CAP2	YPL256C	CLN2
YBR200W	BEM1	YOR043W	WHI2
YFR008W	FAR7	YNL127W	FAR11
YCL037C	SRO9	YKR001C	VPS1

Predictions at threshold level 0.75 continued...			
Gene1 of predicted pair		Gene2 of predicted pair	
Systematic Name	Standard Name	Systematic Name	Standard Name
YPR036W	VMA13	YDL013W	SLX5
YDR484W	VPS52	YNL076W	MKS1
YNL087W	TCB2	YLR371W	ROM2
YPL024W	RMI1	YNL297C	MON2
YPL161C	BEM4	YLR398C	SKI2
YDL223C	HBT1	YDR310C	SUM1
YDR144C	MKC7	YPL018W	CTF19
YGL094C	PAN2	YLR085C	ARP6
YDL185W	TFP1	YPL018W	CTF19
YLR216C	CPR6	YPR046W	MCM16
YIL148W	RPL40A	YBL079W	NUP170
YGL200C	EMP24	YJL183W	MNN11
YPL259C	APM1	YOR036W	PEP12
YOR112W	CEX1	YGR020C	VMA7
YPL161C	BEM4	YGL124C	MON1
YBR107C	IML3	YEL056W	HAT2
YGL087C	MMS2	YBR200W	BEM1
YMR039C	SUB1	YLR288C	MEC3
YPL115C	BEM3	YNL166C	BNI5
YOL030W	GAS5	YLR313C	SPH1
YOR275C	RIM20	YOL081W	IRA2
YIL076W	SEC28	YMR153W	NUP53
YKR061W	KTR2	YDR318W	MCM21
YKL213C	DOA1	YER139C	YER139C
YGL223C	COG1	YCL037C	SRO9
YBR020W	GAL1	YDR363W-A	SEM1
YLL026W	HSP104	YJR032W	CPR7
YJL124C	LSM1	YKL110C	KTI12
YMR172W	HOT1	YHL002W	HSE1

Predictions at threshold level 0.75 continued...			
Gene1 of predicted pair		Gene2 of predicted pair	
Systematic Name	Standard Name	Systematic Name	Standard Name
YMR055C	BUB2	YDR318W	MCM21
YLR079W	SIC1	YBR200W	BEM1
YNL025C	SSN8	YFR010W	UBP6
YHR200W	RPN10	YFR036W	CDC26
YNL032W	SIW14	YLR093C	NYV1
YBR171W	SEC66	YMR311C	GLC8
YER144C	UBP5	YPL057C	SUR1
YLR337C	VRP1	YBL067C	UBP13
YDL226C	GCS1	YNL107W	YAF9
YCR077C	PAT1	YDR310C	SUM1
YKR020W	VPS51	YKR048C	NAP1
YKL139W	CTK1	YOR290C	SNF2
YOL145C	CTR9	YPL161C	BEM4
YOL018C	TLG2	YNL127W	FAR11
YBR215W	HPC2	YHR010W	RPL27A
YDR137W	RGP1	YLR093C	NYV1
YNR052C	POP2	YIL103W	DPH1
YGL087C	MMS2	YBR171W	SEC66
YDR477W	SNF1	YMR140W	SIP5
YPR017C	DSS4	YGL115W	SNF4
YHR134W	WSS1	YLR381W	CTF3
YBR173C	UMP1	YLR094C	GIS3
YMR116C	ASC1	YJL183W	MNN11
YOR332W	VMA4	YDR202C	RAV2
YDR448W	ADA2	YGL252C	RTG2
YCR081W	SRB8	YOR140W	SFL1
YGL158W	RCK1	YPL058C	PDR12
YOR043W	WHI2	YGL003C	CDH1
YDR358W	GGA1	YLR128W	DCN1

Predictions at threshold level 0.75 continued...			
Gene1 of predicted pair		Gene2 of predicted pair	
Systematic Name	Standard Name	Systematic Name	Standard Name
YDL013W	SLX5	YKL101W	HSL1
YOR327C	SNC2	YBR036C	CSG2
YHL027W	RIM101	YJL183W	MNN11
YLR452C	SST2	YNL107W	YAF9
YDL116W	NUP84	YDR207C	UME6
YDR477W	SNF1	YOR270C	VPH1
YGL147C	RPL9A	YBR200W	BEM1
YGL066W	SGF73	YLR150W	STM1
YNL264C	PDR17	YOR101W	RAS1
YMR153W	NUP53	YDR261C	EXG2
YKL139W	CTK1	YNL238W	KEX2
YPR046W	MCM16	YNL116W	DMA2
YOR270C	VPH1	YPL195W	APL5
YML071C	COG8	YGL124C	MON1
YML001W	YPT7	YAR014C	BUD14
YFR010W	UBP6	YNL291C	MID1
YGR003W	CUL3	YPR066W	UBA3
YMR216C	SKY1	YKR020W	VPS51
YDR207C	UME6	YOL081W	IRA2
YML103C	NUP188	YGR081C	SLX9
YDR383C	NKP1	YFR036W	CDC26
YMR152W	YIM1	YER122C	GLO3
YFR036W	CDC26	YMR214W	SCJ1
YDL155W	CLB3	YOL091W	SPO21
YML112W	CTK3	YPR119W	CLB2
YBR215W	HPC2	YCR031C	RPS14A
YBR200W	BEM1	YER122C	GLO3
YMR224C	MRE11	YNL079C	TPM1
YFL023W	BUD27	YOR066W	MSA1

Predictions at threshold level 0.75 continued...			
Gene1 of predicted pair		Gene2 of predicted pair	
Systematic Name	Standard Name	Systematic Name	Standard Name
YBR200W	BEM1	YDL056W	MBP1
YGL066W	SGF73	YJL095W	BCK1
YLR386W	VAC14	YJL006C	CTK2
YOR080W	DIA2	YBR107C	IML3
YLR384C	IKI3	YBL071W-A	KTI11
YAL024C	LTE1	YMR038C	CCS1
YIL156W	UBP7	YFL047W	RGD2
YKR048C	NAP1	YIL133C	RPL16A
YOR069W	VPS5	YMR054W	STV1
YDL192W	ARF1	YOL081W	IRA2
YML095C	RAD10	YLR370C	ARC18
YDR318W	MCM21	YGL151W	NUT1
YBL072C	RPS8A	YLR370C	ARC18
YJL036W	SNX4	YAL002W	VPS8
YOR140W	SFL1	YGL025C	PGD1
YER161C	SPT2	YLR350W	ORM2
YJL172W	CPS1	YBL103C	RTG3
YNL031C	HHT2	YER068W	MOT2
YOR109W	INP53	YDL192W	ARF1
YBR200W	BEM1	YDR144C	MKC7
YGR241C	YAP1802	YJL029C	VPS53
YGR020C	VMA7	YER120W	SCS2
YKR061W	KTR2	YLR315W	NKP2
YMR224C	MRE11	YKR099W	BAS1
YOR371C	GPB1	YGL121C	GPG1
YLR079W	SIC1	YOL091W	SPO21
YBL051C	PIN4	YNL166C	BNI5
YDR296W	MHR1	YPL248C	GAL4
YNL218W	MGS1	YPL022W	RAD1

Predictions at threshold level 0.75 continued...			
Gene1 of predicted pair		Gene2 of predicted pair	
Systematic Name	Standard Name	Systematic Name	Standard Name
YNL069C	RPL16B	YOR127W	RGA1
YML103C	NUP188	YPL152W	RRD2
YGL212W	VAM7	YPL050C	MNN9
YMR125W	STO1	YPL246C	RBD2
YGR020C	VMA7	YCL037C	SRO9
YLR399C	BDF1	YJR060W	CBF1
YLR058C	SHM2	YJR135C	MCM22
YML095C	RAD10	YMR109W	MYO5
YLR083C	EMP70	YJL154C	VPS35
YOL111C	MDY2	YOL121C	RPS19A
YCR081W	SRB8	YDR245W	MNN10
YBR228W	SLX1	YJR035W	RAD26
YLR119W	SRN2	YDR484W	VPS52
YNL288W	CAF40	YKL048C	ELM1
YOR132W	VPS17	YEL042W	GDA1
YKL139W	CTK1	YOR043W	WHI2
YNL307C	MCK1	YDR207C	UME6
YFR036W	CDC26	YER139C	YER139C
YHR156C	LIN1	YPL137C	GIP3
YCR088W	ABP1	YOR367W	SCP1
YAL024C	LTE1	YIR005W	IST3
YCL001W	RER1	YJR033C	RAV1
YLR320W	MMS22	YMR133W	REC114
YER162C	RAD4	YKR030W	GMH1
YGL135W	RPL1B	YGL215W	CLG1
YPL022W	RAD1	YJR052W	RAD7
YKR057W	RPS21A	YPL161C	BEM4
YNL016W	PUB1	YGL031C	RPL24A
YNL201C	PSY2	YFR030W	MET10

Predictions at threshold level 0.75 continued...			
**Gene1 of predicted pair**		**Gene2 of predicted pair**	
**Systematic Name**	**Standard Name**	**Systematic Name**	**Standard Name**
YML001W	YPT7	YNL238W	KEX2
YML095C	RAD10	YDL155W	CLB3
YOR076C	SKI7	YNL106C	INP52
YHR200W	RPN10	YAL002W	VPS8
YLR386W	VAC14	YCR053W	THR4
YMR142C	RPL13B	YER122C	GLO3
YHR160C	PEX18	YGL153W	PEX14
YGL115W	SNF4	YLR370C	ARC18
YNR052C	POP2	YAR014C	BUD14
YDR477W	SNF1	YLR441C	RPS1A
YHR060W	VMA22	YML038C	YMD8
YDR443C	SSN2	YJL172W	CPS1
YPR017C	DSS4	YJL053W	PEP8
YGL212W	VAM7	YNL041C	COG6
YFR031C-A	RPL2A	YDL223C	HBT1
YLR452C	SST2	YNL147W	LSM7
YAL051W	OAF1	YJL124C	LSM1
YDR484W	VPS52	YPL137C	GIP3
YCL001W	RER1	YEL043W	YEL043W
YHR178W	STB5	YAR014C	BUD14
YNL288W	CAF40	YGL151W	NUT1
YEL009C	GCN4	YPL129W	TAF14
YKL116C	PRR1	YLR180W	SAM1
YDL160C	DHH1	YBR089C-A	NHP6B
YBR131W	CCZ1	YBR082C	UBC4
YJL154C	VPS35	YGR105W	VMA21
YPL037C	EGD1	YPL234C	TFP3
YOR109W	INP53	YNL107W	YAF9
YOR337W	TEA1	YLR320W	MMS22

Predictions at threshold level 0.75 continued...			
Gene1 of predicted pair		Gene2 of predicted pair	
Systematic Name	Standard Name	Systematic Name	Standard Name
YPL246C	RBD2	YPL256C	CLN2
YML115C	VAN1	YBR181C	RPS6B
YDR253C	MET32	YPL022W	RAD1
YLR435W	TSR2	YDR310C	SUM1
YDR129C	SAC6	YGL095C	VPS45
YML071C	COG8	YNL076W	MKS1
YLR441C	RPS1A	YPR030W	CSR2
YER109C	FLO8	YBR083W	TEC1
YMR199W	CLN1	YML057W	CMP2
YNL087W	TCB2	YOR066W	MSA1
YOR290C	SNF2	YPR036W	VMA13
YNL215W	IES2	YLR254C	NDL1
YIL034C	CAP2	YDR309C	GIC2
YLR441C	RPS1A	YHR200W	RPN10
YOR290C	SNF2	YPL195W	APL5
YCR020C-A	MAK31	YGL115W	SNF4
YIL076W	SEC28	YKR035W-A	DID2
YLR292C	SEC72	YLR447C	VMA6
YBR171W	SEC66	YLR425W	TUS1
YDR174W	HMO1	YMR224C	MRE11
YGR270W	YTA7	YJR060W	CBF1
YGL016W	KAP122	YKR024C	DBP7
YMR026C	PEX12	YLR452C	SST2
YLR234W	TOP3	YKL068W	NUP100
YAL024C	LTE1	YPR120C	CLB5
YCL037C	SRO9	YGL054C	ERV14
YML058W	SML1	YGL175C	SAE2
YDR129C	SAC6	YJL183W	MNN11
YGL153W	PEX14	YDL155W	CLB3

Predictions at threshold level 0.75 continued...			
Gene1 of predicted pair		Gene2 of predicted pair	
Systematic Name	Standard Name	Systematic Name	Standard Name
YIL072W	HOP1	YLR263W	RED1
YPL137C	GIP3	YGL124C	MON1
YEL050C	RML2	YDL155W	CLB3
YCR066W	RAD18	YMR038C	CCS1
YBL027W	RPL19B	YIR009W	MSL1
YGR249W	MGA1	YOL111C	MDY2
YKL032C	IXR1	YMR055C	BUB2
YCR020C-A	MAK31	YEL053C	MAK10
YEL051W	VMA8	YPR032W	SRO7
YLR360W	VPS38	YEL051W	VMA8
YJR140C	HIR3	YGL019W	CKB1
YLR386W	VAC14	YPR120C	CLB5
YHR178W	STB5	YBR036C	CSG2
YGR072W	UPF3	YCR066W	RAD18
YPL195W	APL5	YAL002W	VPS8
YNL106C	INP52	YDR183W	PLP1
YDR225W	HTA1	YPR120C	CLB5
YFR030W	MET10	YCR086W	CSM1
YLR079W	SIC1	YDL059C	RAD59
YLR360W	VPS38	YJR135C	MCM22
YPL105C	YPL105C	YNL041C	COG6
YJL095W	BCK1	YKL119C	VPH2
YKR072C	SIS2	YML102W	CAC2
YDR378C	LSM6	YOR308C	SNU66
YMR179W	SPT21	YNL107W	YAF9
YER144C	UBP5	YNL041C	COG6
YDR192C	NUP42	YMR255W	GFD1
YMR216C	SKY1	YDR310C	SUM1
YNL291C	MID1	YOR368W	RAD17

Predictions at threshold level 0.75 continued...			
Gene1 of predicted pair		Gene2 of predicted pair	
Systematic Name	Standard Name	Systematic Name	Standard Name
YBR194W	SOY1	YLR370C	ARC18
YPL256C	CLN2	YKL068W	NUP100
YLR386W	VAC14	YLR410W	VIP1
YFL026W	STE2	YHR108W	GGA2
YGL078C	DBP3	YDL083C	RPS16B
YKL116C	PRR1	YOR066W	MSA1
YLR150W	STM1	YML028W	TSA1
YPL161C	BEM4	YHR081W	LRP1
YNL329C	PEX6	YOL044W	PEX15
YOR109W	INP53	YNL051W	COG5
YAL024C	LTE1	YMR230W	RPS10B
YLR288C	MEC3	YML095C	RAD10
YLR018C	POM34	YIL156W	UBP7
YDR507C	GIN4	YKR048C	NAP1
YHR060W	VMA22	YAR014C	BUD14
YOR078W	BUD21	YHR052W	CIC1
YGL087C	MMS2	YPR046W	MCM16
YPL115C	BEM3	YBR108W	YBR108W
YBL016W	FUS3	YLR452C	SST2
YDR318W	MCM21	YOR308C	SNU66
YGR249W	MGA1	YNL325C	FIG4
YLR381W	CTF3	YDL226C	GCS1
YGR188C	BUB1	YOL081W	IRA2
YLR452C	SST2	YHR158C	KEL1
YOR127W	RGA1	YDR202C	RAV2
YGL223C	COG1	YJL004C	SYS1
YOR026W	BUB3	YIL033C	BCY1
YGR059W	SPR3	YKR024C	DBP7
YPL161C	BEM4	YDR144C	MKC7

Predictions at threshold level 0.75 continued...			
Gene1 of predicted pair		Gene2 of predicted pair	
Systematic Name	Standard Name	Systematic Name	Standard Name
YGL124C	MON1	YIL133C	RPL16A
YER044C	ERG28	YNL076W	MKS1
YNL201C	PSY2	YBR107C	IML3
YDL223C	HBT1	YLR398C	SKI2
YAL024C	LTE1	YJR060W	CBF1
YBR019C	GAL10	YJL183W	MNN11
YLR381W	CTF3	YPL152W	RRD2
YPR017C	DSS4	YPR028W	YOP1
YER001W	MNN1	YJL095W	BCK1
YLR234W	TOP3	YDR260C	SWM1
YNL206C	RTT106	YJL136C	RPS21B
YLR135W	SLX4	YNL016W	PUB1
YML062C	MFT1	YBR111W-A	SUS1
YPL057C	SUR1	YOL121C	RPS19A
YOR076C	SKI7	YGR072W	UPF3
YNL041C	COG6	YEL042W	GDA1
YPL019C	VTC3	YFL004W	VTC2
YNL325C	FIG4	YLR093C	NYV1
YBL058W	SHP1	YPR036W	VMA13
YGL033W	HOP2	YGL115W	SNF4
YLR447C	VMA6	YGL253W	HXK2
YNL206C	RTT106	YER110C	KAP123
YKL074C	MUD2	YNL091W	NST1
YNR072W	HXT17	YKR035W-A	DID2
YGR249W	MGA1	YJR032W	CPR7
YFL023W	BUD27	YPL161C	BEM4
YOR001W	RRP6	YNL297C	MON2
YGR188C	BUB1	YOR014W	RTS1
YOR290C	SNF2	YER139C	YER139C

Predictions at threshold level 0.75 continued...			
Gene1 of predicted pair		Gene2 of predicted pair	
Systematic Name	Standard Name	Systematic Name	Standard Name
YLR192C	HCR1	YOR035C	SHE4
YMR095C	SNO1	YPL195W	APL5
YER044C	ERG28	YER162C	RAD4
YDR484W	VPS52	YOR035C	SHE4
YBR010W	HHT1	YLR182W	SWI6
YBR194W	SOY1	YER139C	YER139C
YMR142C	RPL13B	YMR153W	NUP53
YFR010W	UBP6	YKL213C	DOA1
YCR034W	FEN1	YOL081W	IRA2
YMR202W	ERG2	YLR370C	ARC18
YHL031C	GOS1	YDR289C	RTT103
YDR080W	VPS41	YGR020C	VMA7
YMR039C	SUB1	YGL148W	ARO2
YGR135W	PRE9	YJR035W	RAD26
YBR126C	TPS1	YOL111C	MDY2
YOR106W	VAM3	YNL041C	COG6
YLR018C	POM34	YKL117W	SBA1
YIL053W	RHR2	YJR084W	CSN12
YPL115C	BEM3	YBR290W	BSD2
YGR270W	YTA7	YDR289C	RTT103
YLR315W	NKP2	YDR318W	MCM21
YFR031C-A	RPL2A	YKR035W-A	DID2
YBR200W	BEM1	YPL089C	RLM1
YJL106W	IME2	YPR119W	CLB2
YMR133W	REC114	YHR039C-A	VMA10
YHR195W	NVJ1	YEL013W	VAC8
YMR127C	SAS2	YLR399C	BDF1
YIL008W	URM1	YGL019W	CKB1
YMR205C	PFK2	YGR135W	PRE9

Predictions at threshold level 0.75 continued...			
Gene1 of predicted pair		Gene2 of predicted pair	
Systematic Name	Standard Name	Systematic Name	Standard Name
YMR224C	MRE11	YNL041C	COG6
YEL050C	RML2	YBR107C	IML3
YFR031C-A	RPL2A	YGR072W	UPF3
YGL090W	LIF1	YCR014C	POL4
YBR171W	SEC66	YMR133W	REC114
YDR329C	PEX3	YHR160C	PEX18
YDR496C	PUF6	YKL009W	MRT4
YGL087C	MMS2	YLR386W	VAC14
YER162C	RAD4	YDR153C	ENT5
YDR129C	SAC6	YLR429W	CRN1
YAR014C	BUD14	YOL081W	IRA2
YOR112W	CEX1	YKR030W	GMH1
YBR131W	CCZ1	YPL045W	VPS16
YDL002C	NHP10	YHR039C-A	VMA10
YLR083C	EMP70	YML071C	COG8
YPL253C	VIK1	YLR150W	STM1
YER116C	SLX8	YMR152W	YIM1
YDR142C	PEX7	YOL044W	PEX15
YDL154W	MSH5	YLR234W	TOP3
YCR020C-A	MAK31	YLR309C	IMH1
YGR249W	MGA1	YLR180W	SAM1
YLR337C	VRP1	YGL253W	HXK2
YNL087W	TCB2	YJR032W	CPR7
YGL200C	EMP24	YGR135W	PRE9
YLR192C	HCR1	YOL111C	MDY2
YLR058C	SHM2	YJL006C	CTK2
YMR216C	SKY1	YOR327C	SNC2
YOR127W	RGA1	YHR152W	SPO12
YLR381W	CTF3	YDR378C	LSM6

Predictions at threshold level 0.75 continued...			
Gene1 of predicted pair		Gene2 of predicted pair	
Systematic Name	Standard Name	Systematic Name	Standard Name
YDR383C	NKP1	YMR055C	BUB2
YPL024W	RMI1	YHR134W	WSS1
YNL291C	MID1	YER120W	SCS2
YBL102W	SFT2	YJR060W	CBF1
YLR386W	VAC14	YBR036C	CSG2
YPL125W	KAP120	YKL068W	NUP100
YJR005W	APL1	YGL003C	CDH1
YPL120W	VPS30	YAL002W	VPS8
YNL032W	SIW14	YDR207C	UME6
YGL043W	DST1	YGL094C	PAN2
YFL023W	BUD27	YBR171W	SEC66
YGL054C	ERV14	YNL051W	COG5
YOR308C	SNU66	YER139C	YER139C
YEL043W	YEL043W	YER162C	RAD4
YOR191W	RIS1	YCL037C	SRO9
YAL024C	LTE1	YLR441C	RPS1A
YOR080W	DIA2	YKL160W	ELF1
YDL083C	RPS16B	YPR030W	CSR2
YGL223C	COG1	YDL077C	VAM6
YKR099W	BAS1	YNL238W	KEX2
YLR386W	VAC14	YPL018W	CTF19
YKL007W	CAP1	YDR293C	SSD1
YOL018C	TLG2	YLR309C	IMH1
YER040W	GLN3	YLR180W	SAM1
YFL013C	IES1	YDR378C	LSM6
YKR001C	VPS1	YHR060W	VMA22
YOR141C	ARP8	YNL116W	DMA2
YBR200W	BEM1	YGL019W	CKB1
YLR234W	TOP3	YNR052C	POP2

Predictions at threshold level 0.75 continued...			
Gene1 of predicted pair		Gene2 of predicted pair	
Systematic Name	Standard Name	Systematic Name	Standard Name
YDR137W	RGP1	YNL076W	MKS1
YPR084W	YPR084W	YHR039C-A	VMA10
YBR217W	ATG12	YKL126W	YPK1
YKL025C	PAN3	YLR233C	EST1
YML038C	YMD8	YMR054W	STV1
YDR358W	GGA1	YJL183W	MNN11
YPL140C	MKK2	YIL113W	SDP1
YFL033C	RIM15	YOR327C	SNC2
YDR457W	TOM1	YPR119W	CLB2
YKL110C	KTI12	YPR046W	MCM16
YJL036W	SNX4	YML071C	COG8
YFR036W	CDC26	YJL164C	TPK1
YDR108W	GSG1	YOR069W	VPS5
YHR111W	UBA4	YGL252C	RTG2
YCR020C-A	MAK31	YGL223C	COG1
YKL139W	CTK1	YDR245W	MNN10
YFR030W	MET10	YHR115C	DMA1
YLR191W	PEX13	YNL214W	PEX17
YOR076C	SKI7	YJL176C	SWI3
YGR252W	GCN5	YKR048C	NAP1
YPL161C	BEM4	YGL054C	ERV14
YOR270C	VPH1	YMR133W	REC114
YMR273C	ZDS1	YBR010W	HHT1
YCR020C-A	MAK31	YNL127W	FAR11
YAL024C	LTE1	YDL013W	SLX5
YBR217W	ATG12	YBR200W	BEM1
YFL013C	IES1	YGR270W	YTA7
YGR261C	APL6	YEL053C	MAK10
YDL116W	NUP84	YML102W	CAC2

Predictions at threshold level 0.75 continued...			
Gene1 of predicted pair		Gene2 of predicted pair	
Systematic Name	Standard Name	Systematic Name	Standard Name
YJL127C	SPT10	YBR107C	IML3
YGL208W	SIP2	YGL179C	TOS3
YEL053C	MAK10	YMR152W	YIM1
YDL020C	RPN4	YOR332W	VMA4
YGR241C	YAP1802	YDR320C	SWA2
YNL199C	GCR2	YLR315W	NKP2
YDR108W	GSG1	YHR039C-A	VMA10
YAL024C	LTE1	YML103C	NUP188
YIL076W	SEC28	YER120W	SCS2
YCR053W	THR4	YER040W	GLN3
YJL176C	SWI3	YML115C	VAN1
YGL078C	DBP3	YLR093C	NYV1
YGL115W	SNF4	YPL137C	GIP3
YLR360W	VPS38	YML097C	VPS9
YPL115C	BEM3	YER110C	KAP123
YGL003C	CDH1	YJL183W	MNN11
YDR443C	SSN2	YDL083C	RPS16B
YJR135C	MCM22	YDR155C	CPR1
YHR178W	STB5	YPL018W	CTF19
YIR017C	MET28	YDR318W	MCM21
YDR156W	RPA14	YPR120C	CLB5
YGR019W	UGA1	YOR358W	HAP5
YPL037C	EGD1	YML038C	YMD8
YPR120C	CLB5	YLR410W	VIP1
YPL042C	SSN3	YDR335W	MSN5
YFL013C	IES1	YLR182W	SWI6
YBR057C	MUM2	YPL152W	RRD2
YIL076W	SEC28	YCL037C	SRO9
YFR031C-A	RPL2A	YNL291C	MID1

Predictions at threshold level 0.75 continued...			
Gene1 of predicted pair		Gene2 of predicted pair	
Systematic Name	Standard Name	Systematic Name	Standard Name
YML095C	RAD10	YCR086W	CSM1
YER059W	PCL6	YNL230C	ELA1
YJL095W	BCK1	YHR114W	BZZ1
YMR224C	MRE11	YBL088C	TEL1
YNL087W	TCB2	YLR094C	GIS3
YPL152W	RRD2	YDR183W	PLP1
YLR360W	VPS38	YPL195W	APL5
YLR135W	SLX4	YPL022W	RAD1
YKL139W	CTK1	YER116C	SLX8
YOR368W	RAD17	YNL076W	MKS1
YPR084W	YPR084W	YKL160W	ELF1
YMR095C	SNO1	YOL121C	RPS19A
YPL161C	BEM4	YMR152W	YIM1
YKL048C	ELM1	YDR261C	EXG2
YDR254W	CHL4	YOL091W	SPO21
YMR224C	MRE11	YNL076W	MKS1
YBR194W	SOY1	YML095C	RAD10
YMR216C	SKY1	YER122C	GLO3
YOR083W	WHI5	YLR058C	SHM2
YPR072W	NOT5	YJL140W	RPB4
YHR187W	IKI1	YPL101W	ELP4
YMR095C	SNO1	YKL126W	YPK1
YAL024C	LTE1	YGR188C	BUB1
YLR119W	SRN2	YDR261C	EXG2
YNR052C	POP2	YMR055C	BUB2
YDR174W	HMO1	YLR386W	VAC14
YKR072C	SIS2	YJL124C	LSM1
YML103C	NUP188	YHR111W	UBA4
YGL025C	PGD1	YGR252W	GCN5

Predictions at threshold level 0.75 continued...			
Gene1 of predicted pair		Gene2 of predicted pair	
Systematic Name	Standard Name	Systematic Name	Standard Name
YJL095W	BCK1	YER122C	GLO3
YPL024W	RMI1	YHR167W	THP2
YLR216C	CPR6	YFR010W	UBP6
YGL163C	RAD54	YOL081W	IRA2
YJL124C	LSM1	YDR378C	LSM6
YPL129W	TAF14	YGL025C	PGD1
YBR173C	UMP1	YBL025W	RRN10
YOR023C	AHC1	YLR447C	VMA6
YML115C	VAN1	YLR320W	MMS22
YER120W	SCS2	YDR153C	ENT5
YGL003C	CDH1	YDR310C	SUM1
YLR399C	BDF1	YPL248C	GAL4
YDL192W	ARF1	YFL048C	EMP47
YDR245W	MNN10	YNL079C	TPM1
YDR335W	MSN5	YLR032W	RAD5
YDR378C	LSM6	YLR370C	ARC18
YKL126W	YPK1	YJR084W	CSN12
YGL033W	HOP2	YMR230W	RPS10B
YHR200W	RPN10	YJR032W	CPR7
YOR195W	SLK19	YFR036W	CDC26
YLR447C	VMA6	YDL192W	ARF1
YPL105C	YPL105C	YPL234C	TFP3
YKL020C	SPT23	YOR124C	UBP2
YDR261C	EXG2	YER040W	GLN3
YDR004W	RAD57	YER116C	SLX8
YJL006C	CTK2	YKL114C	APN1
YGL066W	SGF73	YOR195W	SLK19
YLR234W	TOP3	YBR082C	UBC4
YBR200W	BEM1	YGL163C	RAD54

Predictions at threshold level 0.75 continued...			
Gene1 of predicted pair		Gene2 of predicted pair	
Systematic Name	Standard Name	Systematic Name	Standard Name
YAL024C	LTE1	YNL225C	CNM67
YPL024W	RMI1	YDR156W	RPA14
YHR134W	WSS1	YGR071C	YGR071C
YKR020W	VPS51	YGR105W	VMA21
YDR202C	RAV2	YPR032W	SRO7
YIL050W	PCL7	YGR233C	PHO81
YHR013C	ARD1	YDR076W	RAD55
YGR104C	SRB5	YPL256C	CLN2
YBR171W	SEC66	YDL192W	ARF1
YLR120C	YPS1	YPL161C	BEM4
YDL223C	HBT1	YLR180W	SAM1
YJR063W	RPA12	YJR060W	CBF1
YOR275C	RIM20	YPR119W	CLB2
YKR099W	BAS1	YGL003C	CDH1
YMR224C	MRE11	YJR056C	YJR056C
YBR111W-A	SUS1	YPR072W	NOT5
YBR019C	GAL10	YOR327C	SNC2
YPL057C	SUR1	YLR398C	SKI2
YOR358W	HAP5	YLR447C	VMA6
YJR032W	CPR7	YOR035C	SHE4
YMR095C	SNO1	YLR452C	SST2
YHR081W	LRP1	YDR207C	UME6
YOL091W	SPO21	YNL225C	CNM67
YDL080C	THI3	YBR240C	THI2
YGL212W	VAM7	YLR396C	VPS33
YOR290C	SNF2	YLR320W	MMS22
YIL103W	DPH1	YLR254C	NDL1
YEL054C	RPL12A	YGL246C	RAI1
YLR360W	VPS38	YHR205W	SCH9

Predictions at threshold level 0.75 continued...			
**Gene1 of predicted pair**		**Gene2 of predicted pair**	
**Systematic Name**	**Standard Name**	**Systematic Name**	**Standard Name**
YCR081W	SRB8	YOR106W	VAM3
YPL057C	SUR1	YOL081W	IRA2
YKR035W-A	DID2	YLR320W	MMS22
YPL057C	SUR1	YGR270W	YTA7
YLR254C	NDL1	YOL054W	PSH1
YMR152W	YIM1	YPL195W	APL5
YIL076W	SEC28	YNL199C	GCR2
YPR051W	MAK3	YGR270W	YTA7
YBR290W	BSD2	YJL183W	MNN11
YBR171W	SEC66	YHR158C	KEL1
YNL325C	FIG4	YMR311C	GLC8
YMR055C	BUB2	YJR063W	RPA12
YML001W	YPT7	YOL108C	INO4
YBL102W	SFT2	YGR105W	VMA21
YIL053W	RHR2	YOR035C	SHE4
YPL079W	RPL21B	YHR167W	THP2
YJR032W	CPR7	YOL067C	RTG1
YCR053W	THR4	YLR398C	SKI2
YGL115W	SNF4	YDR318W	MCM21
YNL242W	ATG2	YLR431C	ATG23
YLR083C	EMP70	YKR020W	VPS51
YOR023C	AHC1	YIL008W	URM1
YMR224C	MRE11	YDR261C	EXG2
YIL005W	EPS1	YBR170C	NPL4
YJL006C	CTK2	YDR318W	MCM21
YER116C	SLX8	YDR358W	GGA1
YHR111W	UBA4	YOL067C	RTG1
YMR095C	SNO1	YEL060C	PRB1
YPR084W	YPR084W	YNL041C	COG6

Predictions at threshold level 0.75 continued...			
Gene1 of predicted pair		Gene2 of predicted pair	
Systematic Name	Standard Name	Systematic Name	Standard Name
YBR200W	BEM1	YGR020C	VMA7
YOR106W	VAM3	YNL051W	COG5
YMR199W	CLN1	YGR252W	GCN5
YLR292C	SEC72	YBL021C	HAP3
YNL087W	TCB2	YDL223C	HBT1
YOR080W	DIA2	YLR315W	NKP2
YDR254W	CHL4	YDL185W	TFP1
YHR200W	RPN10	YPL089C	RLM1
YNL307C	MCK1	YDL077C	VAM6
YPL057C	SUR1	YER162C	RAD4
YBR171W	SEC66	YJR084W	CSN12
YLR094C	GIS3	YDL083C	RPS16B
YGL135W	RPL1B	YAL002W	VPS8
YML038C	YMD8	YGR105W	VMA21
YGR020C	VMA7	YBR127C	VMA2
YDL020C	RPN4	YPL022W	RAD1
YPL018W	CTF19	YDR310C	SUM1
YBR126C	TPS1	YBL103C	RTG3
YHR086W	NAM8	YPL213W	LEA1
YLR216C	CPR6	YPL152W	RRD2
YDR484W	VPS52	YAL030W	SNC1
YNL264C	PDR17	YMR198W	CIK1
YBR217W	ATG12	YBR108W	YBR108W
YDR439W	LRS4	YLR254C	NDL1
YKL110C	KTI12	YDR318W	MCM21
YLR079W	SIC1	YBR107C	IML3
YBR171W	SEC66	YCR077C	PAT1
YDR378C	LSM6	YNL236W	SIN4
YOR033C	EXO1	YOR032C	HMS1

Predictions at threshold level 0.75 continued...			
Gene1 of predicted pair		Gene2 of predicted pair	
Systematic Name	Standard Name	Systematic Name	Standard Name
YLR447C	VMA6	YML071C	COG8
YBL021C	HAP3	YGR270W	YTA7
YML012W	ERV25	YEL036C	ANP1
YDR156W	RPA14	YJR060W	CBF1
YPR120C	CLB5	YDR076W	RAD55
YKR061W	KTR2	YAR014C	BUD14
YPR084W	YPR084W	YCR066W	RAD18
YIL034C	CAP2	YGR270W	YTA7
YDR335W	MSN5	YDR289C	RTT103
YGL133W	ITC1	YHR111W	UBA4
YNL032W	SIW14	YMR230W	RPS10B
YOR080W	DIA2	YDL013W	SLX5
YDL116W	NUP84	YBR107C	IML3
YPR167C	MET16	YOR106W	VAM3
YGR241C	YAP1802	YKR001C	VPS1
YMR109W	MYO5	YHR111W	UBA4
YPL115C	BEM3	YHR178W	STB5
YLR386W	VAC14	YFR030W	MET10
YNL307C	MCK1	YOL054W	PSH1
YPL086C	ELP3	YBL071W-A	KTI11
YPR036W	VMA13	YPL234C	TFP3
YOR368W	RAD17	YGL148W	ARO2
YDL116W	NUP84	YOL090W	MSH2
YLR320W	MMS22	YLR370C	ARC18
YMR216C	SKY1	YNL107W	YAF9
YNL307C	MCK1	YDR289C	RTT103
YBR030W	YBR030W	YJR032W	CPR7
YML115C	VAN1	YGR133W	PEX4
YBR200W	BEM1	YDR309C	GIC2

Predictions at threshold level 0.75 continued...			
Gene1 of predicted pair		Gene2 of predicted pair	
Systematic Name	Standard Name	Systematic Name	Standard Name
YFR031C-A	RPL2A	YKR030W	GMH1
YNL264C	PDR17	YOL051W	GAL11
YPL050C	MNN9	YJL183W	MNN11
YHR060W	VMA22	YLR093C	NYV1
YMR224C	MRE11	YBR073W	RDH54
YNL127W	FAR11	YLR309C	IMH1
YMR026C	PEX12	YJR084W	CSN12
YLR381W	CTF3	YDR318W	MCM21
YNL087W	TCB2	YLR452C	SST2
YKL126W	YPK1	YMR089C	YTA12
YJR033C	RAV1	YAR014C	BUD14
YBR290W	BSD2	YGR188C	BUB1
YMR153W	NUP53	YOR290C	SNF2
YEL050C	RML2	YOR141C	ARP8
YGL212W	VAM7	YML001W	YPT7
YBR171W	SEC66	YLR320W	MMS22
YOR127W	RGA1	YOR358W	HAP5
YGL066W	SGF73	YNL206C	RTT106
YBR171W	SEC66	YMR109W	MYO5
YGR104C	SRB5	YOR290C	SNF2
YFR031C-A	RPL2A	YDR108W	GSG1
YMR179W	SPT21	YPL018W	CTF19
YLR079W	SIC1	YER096W	SHC1
YJR024C	MDE1	YFL047W	RGD2
YNL058C	YNL058C	YLR371W	ROM2
YML103C	NUP188	YJR135C	MCM22
YKR046C	PET10	YKL094W	YJU3
YOR195W	SLK19	YGL003C	CDH1
YDR378C	LSM6	YNL307C	MCK1

Predictions at threshold level 0.75 continued...			
Gene1 of predicted pair		Gene2 of predicted pair	
Systematic Name	Standard Name	Systematic Name	Standard Name
YER144C	UBP5	YKR099W	BAS1
YPL240C	HSP82	YGL076C	RPL7A
YMR199W	CLN1	YML102W	CAC2
YML016C	PPZ1	YKL048C	ELM1
YDR202C	RAV2	YJL004C	SYS1
YML095C	RAD10	YHR152W	SPO12
YIL076W	SEC28	YGL212W	VAM7
YIL036W	CST6	YDR183W	PLP1
YMR202W	ERG2	YKR020W	VPS51
YMR095C	SNO1	YMR016C	SOK2
YMR116C	ASC1	YBR082C	UBC4
YML115C	VAN1	YGL003C	CDH1
YNR052C	POP2	YCR086W	CSM1
YEL027W	CUP5	YKL041W	VPS24
YOL111C	MDY2	YOR043W	WHI2
YLR234W	TOP3	YJL095W	BCK1
YPL057C	SUR1	YLR350W	ORM2
YNL166C	BNI5	YJL183W	MNN11
YLR337C	VRP1	YCR077C	PAT1
YMR198W	CIK1	YJR032W	CPR7
YBR057C	MUM2	YOR312C	RPL20B
YPL042C	SSN3	YER122C	GLO3
YGR261C	APL6	YKR020W	VPS51
YOL051W	GAL11	YGR252W	GCN5
YNL291C	MID1	YOL018C	TLG2
YJL183W	MNN11	YLR370C	ARC18
YBR108W	YBR108W	YLR320W	MMS22
YMR095C	SNO1	YER044C	ERG28
YNL307C	MCK1	YDR358W	GGA1

Predictions at threshold level 0.75 continued...			
Gene1 of predicted pair		Gene2 of predicted pair	
Systematic Name	Standard Name	Systematic Name	Standard Name
YNL291C	MID1	YNL076W	MKS1
YHR013C	ARD1	YLR182W	SWI6
YPL024W	RMI1	YGL078C	DBP3
YMR026C	PEX12	YDR244W	PEX5
YPR029C	APL4	YNR006W	VPS27
YHR205W	SCH9	YDR456W	NHX1
YHR052W	CIC1	YKL068W	NUP100
YLR192C	HCR1	YML115C	VAN1
YNR052C	POP2	YDR183W	PLP1
YDR484W	VPS52	YPL161C	BEM4
YMR179W	SPT21	YPR036W	VMA13
YIL045W	PIG2	YMR071C	TVP18
YGL200C	EMP24	YDR358W	GGA1
YIL156W	UBP7	YKR024C	DBP7
YJL187C	SWE1	YLR425W	TUS1
YLR119W	SRN2	YOL121C	RPS19A
YKR020W	VPS51	YJL204C	RCY1
YHR034C	PIH1	YCR060W	TAH1
YML095C	RAD10	YBR215W	HPC2
YMR055C	BUB2	YIL008W	URM1
YHR156C	LIN1	YLR093C	NYV1
YGR071C	YGR071C	YDR469W	SDC1
YDR378C	LSM6	YHL002W	HSE1
YOR127W	RGA1	YDR309C	GIC2
YPL018W	CTF19	YNL107W	YAF9
YDR318W	MCM21	YDR310C	SUM1
YPR046W	MCM16	YCR086W	CSM1
YGL212W	VAM7	YJR060W	CBF1
YLR079W	SIC1	YPL161C	BEM4

Predictions at threshold level 0.75 continued...			
Gene1 of predicted pair		Gene2 of predicted pair	
Systematic Name	Standard Name	Systematic Name	Standard Name
YNL264C	PDR17	YIL036W	CST6
YLR313C	SPH1	YPR119W	CLB2
YDL125C	HNT1	YMR039C	SUB1
YLR435W	TSR2	YOR368W	RAD17
YFR010W	UBP6	YMR152W	YIM1
YGR261C	APL6	YLR043C	TRX1
YNL215W	IES2	YDR318W	MCM21
YNL094W	APP1	YPL249C	GYP5
YFR031C-A	RPL2A	YGL078C	DBP3
YDR320C	SWA2	YLR309C	IMH1
YNL058C	YNL058C	YER044C	ERG28
YHR200W	RPN10	YPL195W	APL5
YPL022W	RAD1	YLR313C	SPH1
YDR378C	LSM6	YNL199C	GCR2
YOR195W	SLK19	YGR270W	YTA7
YBL008W	HIR1	YHR039C-A	VMA10
YHR156C	LIN1	YHR081W	LRP1
YKL160W	ELF1	YOR124C	UBP2
YDR358W	GGA1	YNL076W	MKS1
YDR395W	SXM1	YDL075W	RPL31A
YNL288W	CAF40	YGL237C	HAP2
YKR099W	BAS1	YER139C	YER139C
YGR268C	HUA1	YOR284W	HUA2
YHR156C	LIN1	YOR043W	WHI2
YEL053C	MAK10	YDR153C	ENT5
YGL147C	RPL9A	YHR039C-A	VMA10
YBL008W	HIR1	YJR060W	CBF1
YNL307C	MCK1	YAL010C	MDM10
YNL087W	TCB2	YML071C	COG8

Predictions at threshold level 0.75 continued...			
Gene1 of predicted pair		Gene2 of predicted pair	
Systematic Name	Standard Name	Systematic Name	Standard Name
YLR083C	EMP70	YNL238W	KEX2
YBR283C	SSH1	YMR022W	UBC7
YKL213C	DOA1	YOR038C	HIR2
YBL058W	SHP1	YKL048C	ELM1
YJR090C	GRR1	YHL002W	HSE1
YDR174W	HMO1	YER116C	SLX8
YDL006W	PTC1	YER122C	GLO3
YJR135C	MCM22	YML028W	TSA1
YNL325C	FIG4	YHR081W	LRP1
YMR224C	MRE11	YJR135C	MCM22
YFR010W	UBP6	YDL184C	RPL41A
YBR194W	SOY1	YJL058C	BIT61
YGL115W	SNF4	YDR358W	GGA1
YCR081W	SRB8	YER049W	TPA1
YBR278W	DPB3	YER161C	SPT2
YCL037C	SRO9	YMR054W	STV1
YOR367W	SCP1	YJL187C	SWE1
YBR194W	SOY1	YGL003C	CDH1
YBR127C	VMA2	YCR088W	ABP1
YNL325C	FIG4	YDL083C	RPS16B
YDR080W	VPS41	YPR051W	MAK3
YHR200W	RPN10	YBL101C	ECM21
YJL176C	SWI3	YMR198W	CIK1
YLR447C	VMA6	YJL183W	MNN11
YPR046W	MCM16	YGL252C	RTG2
YNL106C	INP52	YJL124C	LSM1
YBR215W	HPC2	YML028W	TSA1
YOR212W	STE4	YDR335W	MSN5
YPL022W	RAD1	YGL003C	CDH1

Predictions at threshold level 0.75 continued...			
Gene1 of predicted pair		Gene2 of predicted pair	
Systematic Name	Standard Name	Systematic Name	Standard Name
YIR017C	MET28	YJR060W	CBF1
YMR153W	NUP53	YJR084W	CSN12
YBR108W	YBR108W	YPL195W	APL5
YGR020C	VMA7	YJR033C	RAV1
YPL120W	VPS30	YLR309C	IMH1
YGR104C	SRB5	YMR109W	MYO5
YLR192C	HCR1	YFL023W	BUD27
YER049W	TPA1	YEL036C	ANP1
YPR018W	RLF2	YJL095W	BCK1
YDL065C	PEX19	YMR204C	INP1
YOR035C	SHE4	YLR425W	TUS1
YER116C	SLX8	YNL307C	MCK1
YJL190C	RPS22A	YLR233C	EST1
YGL033W	HOP2	YLR370C	ARC18
YDR293C	SSD1	YCR086W	CSM1
YOR290C	SNF2	YNL307C	MCK1
YPR052C	NHP6A	YPL042C	SSN3
YDR289C	RTT103	YLR233C	EST1
YOR358W	HAP5	YMR125W	STO1
YNL297C	MON2	YMR055C	BUB2
YPR052C	NHP6A	YBL008W	HIR1
YLR192C	HCR1	YPL037C	EGD1
YHR178W	STB5	YOR023C	AHC1
YOR109W	INP53	YCL001W	RER1
YAL051W	OAF1	YPR119W	CLB2
YKR084C	HBS1	YNL001W	DOM34
YBL003C	HTA2	YBL002W	HTB2
YFL023W	BUD27	YMR152W	YIM1
YPR084W	YPR084W	YDR440W	DOT1

Predictions at threshold level 0.75 continued...			
Gene1 of predicted pair		Gene2 of predicted pair	
Systematic Name	Standard Name	Systematic Name	Standard Name
YBL047C	EDE1	YOR035C	SHE4
YLR337C	VRP1	YBR181C	RPS6B
YLR234W	TOP3	YKL048C	ELM1
YDL116W	NUP84	YGR188C	BUB1
YDL056W	MBP1	YDR335W	MSN5
YKL110C	KTI12	YPL152W	RRD2
YNL243W	SLA2	YOR367W	SCP1
YGL223C	COG1	YLR093C	NYV1
YBR108W	YBR108W	YDR144C	MKC7
YPL022W	RAD1	YDR171W	HSP42
YEL065W	SIT1	YAL024C	LTE1
YOR109W	INP53	YJR060W	CBF1
YMR055C	BUB2	YGL124C	MON1
YPL115C	BEM3	YBR114W	RAD16
YEL009C	GCN4	YOL018C	TLG2
YOR043W	WHI2	YBR036C	CSG2
YOR069W	VPS5	YPR032W	SRO7
YLR410W	VIP1	YLR182W	SWI6
YDR004W	RAD57	YOR195W	SLK19
YGL078C	DBP3	YKR099W	BAS1
YER116C	SLX8	YJR056C	YJR056C
YOL086C	ADH1	YOL151W	GRE2
YGL003C	CDH1	YBL025W	RRN10
YHL031C	GOS1	YGL174W	BUD13
YGL003C	CDH1	YDR207C	UME6
YNL288W	CAF40	YNL107W	YAF9
YLR094C	GIS3	YDR310C	SUM1
YMR224C	MRE11	YJR060W	CBF1
YMR127C	SAS2	YEL053C	MAK10

Predictions at threshold level 0.75 continued...			
Gene1 of predicted pair		Gene2 of predicted pair	
Systematic Name	Standard Name	Systematic Name	Standard Name
YDR456W	NHX1	YPL256C	CLN2
YPL161C	BEM4	YPR120C	CLB5
YEL051W	VMA8	YAR014C	BUD14
YJR033C	RAV1	YKL119C	VPH2
YOR132W	VPS17	YJL004C	SYS1
YMR038C	CCS1	YBR082C	UBC4
YGL212W	VAM7	YPR173C	VPS4
YHR161C	YAP1801	YCR088W	ABP1
YPL256C	CLN2	YMR038C	CCS1
YGL066W	SGF73	YFR010W	UBP6
YNL025C	SSN8	YGL025C	PGD1
YGR249W	MGA1	YPL057C	SUR1
YER162C	RAD4	YDR310C	SUM1
YJR084W	CSN12	YBR036C	CSG2
YNL032W	SIW14	YOL081W	IRA2
YLR192C	HCR1	YDR477W	SNF1
YJL154C	VPS35	YGR054W	YGR054W
YLR079W	SIC1	YNL248C	RPA49
YHR081W	LRP1	YLR093C	NYV1
YOR023C	AHC1	YJL183W	MNN11
YNL248C	RPA49	YOR141C	ARP8
YMR142C	RPL13B	YMR133W	REC114
YOL043C	NTG2	YKL114C	APN1
YDR144C	MKC7	YAL002W	VPS8
YPR017C	DSS4	YOR109W	INP53
YBR126C	TPS1	YBL058W	SHP1
YLR079W	SIC1	YDR200C	VPS64
YJL136C	RPS21B	YHR111W	UBA4
YPR051W	MAK3	YDR207C	UME6

Predictions at threshold level 0.75 continued...			
Gene1 of predicted pair		Gene2 of predicted pair	
Systematic Name	Standard Name	Systematic Name	Standard Name
YKR099W	BAS1	YJR084W	CSN12
YGL033W	HOP2	YOR035C	SHE4
YNL058C	YNL058C	YGL115W	SNF4
YMR095C	SNO1	YDR261C	EXG2
YGL087C	MMS2	YHR200W	RPN10
YOR127W	RGA1	YDL077C	VAM6
YPL253C	VIK1	YDL047W	SIT4
YIL076W	SEC28	YJR084W	CSN12
YGL133W	ITC1	YNL116W	DMA2
YDR363W-A	SEM1	YKR030W	GMH1
YBR127C	VMA2	YKR001C	VPS1
YDR245W	MNN10	YBR171W	SEC66
YIL076W	SEC28	YOL081W	IRA2
YIL076W	SEC28	YLR350W	ORM2
YKL139W	CTK1	YDL184C	RPL41A
YNL325C	FIG4	YPL161C	BEM4
YKL007W	CAP1	YMR055C	BUB2
YLR399C	BDF1	YKR048C	NAP1
YPR131C	NAT3	YMR038C	CCS1
YDR245W	MNN10	YDR440W	DOT1
YOR039W	CKB2	YLR350W	ORM2
YBR094W	PBY1	YGL100W	SEH1
YGL060W	YBP2	YBR200W	BEM1
YML016C	PPZ1	YOR043W	WHI2
YMR016C	SOK2	YOR043W	WHI2
YLR381W	CTF3	YJR032W	CPR7
YIL036W	CST6	YHR013C	ARD1
YLR360W	VPS38	YJL095W	BCK1
YKR072C	SIS2	YDR310C	SUM1

Predictions at threshold level 0.75 continued...			
Gene1 of predicted pair		Gene2 of predicted pair	
Systematic Name	Standard Name	Systematic Name	Standard Name
YNL215W	IES2	YML016C	PPZ1
YJR091C	JSN1	YLR370C	ARC18
YAR042W	SWH1	YOL044W	PEX15
YKR001C	VPS1	YLR064W	YLR064W
YDR156W	RPA14	YDL006W	PTC1
YNL291C	MID1	YPL234C	TFP3
YPL001W	HAT1	YHR039C-A	VMA10
YML001W	YPT7	YDR310C	SUM1
YLR095C	IOC2	YGL212W	VAM7
YEL065W	SIT1	YNL041C	COG6
YDL083C	RPS16B	YDR358W	GGA1
YNL218W	MGS1	YGL175C	SAE2
YDL161W	ENT1	YPL045W	VPS16
YDR192C	NUP42	YPR119W	CLB2
YKR059W	TIF1	YLR320W	MMS22
YML103C	NUP188	YNL116W	DMA2
YGR209C	TRX2	YDL138W	RGT2
YDL155W	CLB3	YEL056W	HAT2
YLR396C	VPS33	YGL124C	MON1
YNL106C	INP52	YJR135C	MCM22
YOR069W	VPS5	YGL212W	VAM7
YBR173C	UMP1	YNL032W	SIW14
YPL106C	SSE1	YPL018W	CTF19
YDR254W	CHL4	YAR014C	BUD14
YOR191W	RIS1	YLR135W	SLX4
YBR173C	UMP1	YDR310C	SUM1
YJR032W	CPR7	YDL192W	ARF1
YPL253C	VIK1	YML095C	RAD10
YIL140W	AXL2	YGL054C	ERV14

Predictions at threshold level 0.75 continued...			
Gene1 of predicted pair		Gene2 of predicted pair	
Systematic Name	Standard Name	Systematic Name	Standard Name
YNL307C	MCK1	YMR214W	SCJ1
YMR153W	NUP53	YKR035W-A	DID2
YNL291C	MID1	YDR207C	UME6
YOR008C	SLG1	YOR014W	RTS1
YKL048C	ELM1	YBL087C	RPL23A
YNL041C	COG6	YFR036W	CDC26
YPL121C	MEI5	YPR119W	CLB2
YDR071C	PAA1	YLR357W	RSC2
YPL057C	SUR1	YCR053W	THR4
YDL226C	GCS1	YJR035W	RAD26
YHR161C	YAP1801	YBR127C	VMA2
YGL124C	MON1	YGL252C	RTG2
YNL079C	TPM1	YFR036W	CDC26
YDR448W	ADA2	YBR009C	HHF1
YNL031C	HHT2	YBR010W	HHT1
YFL023W	BUD27	YLR320W	MMS22
YJL029C	VPS53	YOR035C	SHE4
YBL106C	SRO77	YLR423C	ATG17
YLR381W	CTF3	YKL110C	KTI12
YGL153W	PEX14	YGR077C	PEX8
YLR216C	CPR6	YPL018W	CTF19
YNL215W	IES2	YDR137W	RGP1
YDL083C	RPS16B	YOR035C	SHE4
YNR072W	HXT17	YBR280C	SAF1
YGL025C	PGD1	YNL238W	KEX2
YNL025C	SSN8	YMR038C	CCS1
YKL139W	CTK1	YBR217W	ATG12
YDR225W	HTA1	YDR335W	MSN5
YHL019C	APM2	YKL135C	APL2

Predictions at threshold level 0.75 continued...			
Gene1 of predicted pair		Gene2 of predicted pair	
Systematic Name	Standard Name	Systematic Name	Standard Name
YGL174W	BUD13	YOR038C	HIR2
YIL002C	INP51	YBL047C	EDE1
YGL253W	HXK2	YOL067C	RTG1
YOR195W	SLK19	YHR111W	UBA4
YGL087C	MMS2	YGR108W	CLB1
YDL184C	RPL41A	YDR207C	UME6
YPL057C	SUR1	YPR030W	CSR2
YKL068W	NUP100	YKR024C	DBP7
YER122C	GLO3	YDR207C	UME6
YLR441C	RPS1A	YGL124C	MON1
YKL126W	YPK1	YLR441C	RPS1A
YDR254W	CHL4	YKL101W	HSL1
YGL151W	NUT1	YGR188C	BUB1
YJR056C	YJR056C	YMR311C	GLC8
YDR439W	LRS4	YOR141C	ARP8
YER110C	KAP123	YBL079W	NUP170
YER116C	SLX8	YDL223C	HBT1
YIL036W	CST6	YKL041W	VPS24
YJL154C	VPS35	YJL095W	BCK1
YMR055C	BUB2	YOL054W	PSH1
YOR066W	MSA1	YER040W	GLN3
YER129W	SAK1	YGL190C	CDC55
YOR275C	RIM20	YOR043W	WHI2
YBR200W	BEM1	YMR133W	REC114
YKL116C	PRR1	YGL025C	PGD1
YNL199C	GCR2	YFL047W	RGD2
YDR335W	MSN5	YKL110C	KTI12
YBR107C	IML3	YDL047W	SIT4
YJL053W	PEP8	YHR012W	VPS29

Predictions at threshold level 0.75 continued...			
Gene1 of predicted pair		Gene2 of predicted pair	
Systematic Name	Standard Name	Systematic Name	Standard Name
YBR171W	SEC66	YBR036C	CSG2
YEL050C	RML2	YJL127C	SPT10
YBR057C	MUM2	YKL101W	HSL1
YDR293C	SSD1	YBR200W	BEM1
YHL002W	HSE1	YGR188C	BUB1
YEL053C	MAK10	YPL137C	GIP3
YER162C	RAD4	YJR033C	RAV1
YLR234W	TOP3	YHR012W	VPS29
YDR293C	SSD1	YNR032W	PPG1
YJR083C	ACF4	YDR507C	GIN4
YCR020C-A	MAK31	YPL234C	TFP3
YGR019W	UGA1	YCR086W	CSM1
YJR005W	APL1	YPL259C	APM1
YER162C	RAD4	YDL192W	ARF1
YDR001C	NTH1	YPR119W	CLB2
YLR360W	VPS38	YML038C	YMD8
YMR223W	UBP8	YBR111W-A	SUS1
YHR178W	STB5	YPR051W	MAK3
YDL223C	HBT1	YPR046W	MCM16
YMR016C	SOK2	YGL212W	VAM7
YOR043W	WHI2	YOR066W	MSA1
YPR018W	RLF2	YGL175C	SAE2
YPL024W	RMI1	YDL083C	RPS16B
YML103C	NUP188	YGL016W	KAP122
YKR020W	VPS51	YHL002W	HSE1
YNL325C	FIG4	YER116C	SLX8
YML115C	VAN1	YML071C	COG8
YBR114W	RAD16	YLR233C	EST1
YNL027W	CRZ1	YJL164C	TPK1

Predictions at threshold level 0.75 continued...			
Gene1 of predicted pair		Gene2 of predicted pair	
Systematic Name	Standard Name	Systematic Name	Standard Name
YBR171W	SEC66	YMR230W	RPS10B
YJR005W	APL1	YLR370C	ARC18
YBL025W	RRN10	YER040W	GLN3
YOR043W	WHI2	YPR046W	MCM16
YLR090W	XDJ1	YBL058W	SHP1
YER116C	SLX8	YBL025W	RRN10
YDL006W	PTC1	YHL011C	PRS3
YJL176C	SWI3	YOR080W	DIA2
YJL036W	SNX4	YKR020W	VPS51
YMR054W	STV1	YNL127W	FAR11
YMR199W	CLN1	YDR207C	UME6
YOL121C	RPS19A	YMR230W	RPS10B
YHR205W	SCH9	YLR425W	TUS1
YLR180W	SAM1	YMR230W	RPS10B
YOR083W	WHI5	YCR065W	HCM1
YAL024C	LTE1	YJL172W	CPS1
YIL076W	SEC28	YMR230W	RPS10B
YDL006W	PTC1	YLR371W	ROM2
YKL048C	ELM1	YPL089C	RLM1
YMR039C	SUB1	YLR381W	CTF3
YGL060W	YBP2	YPR046W	MCM16
YBR200W	BEM1	YMR202W	ERG2
YDL020C	RPN4	YMR109W	MYO5
YLL039C	UBI4	YDR129C	SAC6
YBR057C	MUM2	YPL018W	CTF19
YCL001W	RER1	YGR105W	VMA21
YHR073W	OSH3	YPL246C	RBD2
YHR178W	STB5	YBR114W	RAD16
YKL025C	PAN3	YKL048C	ELM1

Predictions at threshold level 0.75 continued...			
Gene1 of predicted pair		Gene2 of predicted pair	
Systematic Name	Standard Name	Systematic Name	Standard Name
YCR066W	RAD18	YOL018C	TLG2
YGR270W	YTA7	YHL002W	HSE1
YHR178W	STB5	YLR320W	MMS22
YBR200W	BEM1	YLR233C	EST1
YDL002C	NHP10	YDR289C	RTT103
YDR254W	CHL4	YGR054W	YGR054W
YLR320W	MMS22	YER122C	GLO3
YGL025C	PGD1	YJL140W	RPB4
YFR009W	GCN20	YJL189W	RPL39
YCL001W	RER1	YHL020C	OPI1
YNR072W	HXT17	YHR200W	RPN10
YIL034C	CAP2	YER110C	KAP123
YOR033C	EXO1	YPL256C	CLN2
YLR083C	EMP70	YNL297C	MON2
YNL068C	FKH2	YDR507C	GIN4
YNL079C	TPM1	YEL036C	ANP1
YOR195W	SLK19	YOR368W	RAD17
YNL201C	PSY2	YPL152W	RRD2
YFR030W	MET10	YGR188C	BUB1
YLR452C	SST2	YER040W	GLN3
YCR020C-A	MAK31	YER162C	RAD4
YBR215W	HPC2	YOR038C	HIR2
YEL053C	MAK10	YPL018W	CTF19
YLR422W	YLR422W	YNR064C	YNR064C
YKR072C	SIS2	YDL002C	NHP10
YAL016W	TPD3	YPR040W	TIP41
YHR134W	WSS1	YJR035W	RAD26
YGR081C	SLX9	YOR001W	RRP6
YGL250W	RMR1	YOL081W	IRA2

Predictions at threshold level 0.75 continued...			
Gene1 of predicted pair		Gene2 of predicted pair	
Systematic Name	Standard Name	Systematic Name	Standard Name
YBR194W	SOY1	YER118C	SHO1
YJL172W	CPS1	YKL126W	YPK1
YJL058C	BIT61	YPL106C	SSE1
YPL096C-A	ERI1	YEL044W	IES6
YPL037C	EGD1	YOL121C	RPS19A
YGR261C	APL6	YHR012W	VPS29
YLR452C	SST2	YDR318W	MCM21
YML115C	VAN1	YML016C	PPZ1
YHR178W	STB5	YML016C	PPZ1
YER144C	UBP5	YKL048C	ELM1
YAL010C	MDM10	YIL103W	DPH1
YKL161C	YKL161C	YOL081W	IRA2
YHR152W	SPO12	YOR275C	RIM20
YBR031W	RPL4A	YHR203C	RPS4B
YMR054W	STV1	YKR030W	GMH1
YLR234W	TOP3	YOL043C	NTG2
YNL243W	SLA2	YJR125C	ENT3
YBR108W	YBR108W	YMR311C	GLC8
YNR072W	HXT17	YLR370C	ARC18
YCL037C	SRO9	YAL002W	VPS8
YPL018W	CTF19	YGL003C	CDH1
YDR174W	HMO1	YPL022W	RAD1
YHR134W	WSS1	YNL248C	RPA49
YDL002C	NHP10	YCL037C	SRO9
YBR200W	BEM1	YDR484W	VPS52
YOL121C	RPS19A	YER122C	GLO3
YMR224C	MRE11	YKL139W	CTK1
YKL007W	CAP1	YDL226C	GCS1
YDR129C	SAC6	YBR127C	VMA2

Predictions at threshold level 0.75 continued...			
Gene1 of predicted pair		Gene2 of predicted pair	
Systematic Name	Standard Name	Systematic Name	Standard Name
YPR018W	RLF2	YPL022W	RAD1
YOL044W	PEX15	YJL095W	BCK1
YNL094W	APP1	YHR114W	BZZ1
YGL060W	YBP2	YJR060W	CBF1
YPL129W	TAF14	YLR182W	SWI6
YDR129C	SAC6	YKR001C	VPS1
YIR005W	IST3	YCR086W	CSM1
YBR260C	RGD1	YDR207C	UME6
YDR225W	HTA1	YGL241W	KAP114
YBL051C	PIN4	YDR155C	CPR1
YDR142C	PEX7	YDR265W	PEX10
YGL212W	VAM7	YOL111C	MDY2
YKL116C	PRR1	YDL083C	RPS16B
YKL025C	PAN3	YGR188C	BUB1
YGR081C	SLX9	YML095C	RAD10
YBR108W	YBR108W	YBR036C	CSG2
YJL127C	SPT10	YJR135C	MCM22
YEL065W	SIT1	YMR038C	CCS1
YNL106C	INP52	YCR086W	CSM1
YHR016C	YSC84	YOR284W	HUA2
YLR170C	APS1	YKR001C	VPS1
YAL024C	LTE1	YDR254W	CHL4
YMR224C	MRE11	YOR080W	DIA2
YOR141C	ARP8	YOL054W	PSH1
YDR244W	PEX5	YGL153W	PEX14
YML001W	YPT7	YLR370C	ARC18
YKL126W	YPK1	YKR099W	BAS1
YGL147C	RPL9A	YHR156C	LIN1
YBL021C	HAP3	YBR171W	SEC66

Predictions at threshold level 0.75 continued...			
Gene1 of predicted pair		Gene2 of predicted pair	
Systematic Name	Standard Name	Systematic Name	Standard Name
YML095C	RAD10	YPL106C	SSE1
YML109W	ZDS2	YCL032W	STE50
YPL024W	RMI1	YFR010W	UBP6
YER096W	SHC1	YHR012W	VPS29
YJL154C	VPS35	YDR310C	SUM1
YLR181C	VTA1	YDR486C	VPS60
YHR012W	VPS29	YPL256C	CLN2
YMR004W	MVP1	YPL195W	APL5
YNL166C	BNI5	YGL151W	NUT1
YNL032W	SIW14	YHR060W	VMA22
YKL139W	CTK1	YHR081W	LRP1
YDR156W	RPA14	YHR115C	DMA1
YOR112W	CEX1	YGR105W	VMA21
YDL116W	NUP84	YOR033C	EXO1
YPL057C	SUR1	YPL137C	GIP3
YHR030C	SLT2	YER099C	PRS2
YIL076W	SEC28	YNL238W	KEX2
YDR265W	PEX10	YDL065C	PEX19
YJL183W	MNN11	YBL101C	ECM21
YLR079W	SIC1	YFR034C	PHO4
YBR195C	MSI1	YEL056W	HAT2
YBR200W	BEM1	YOR106W	VAM3
YDL101C	DUN1	YDL013W	SLX5
YJL006C	CTK2	YLR254C	NDL1
YER144C	UBP5	YDR358W	GGA1
YBR127C	VMA2	YOR036W	PEP12
YHR171W	ATG7	YHR161C	YAP1801
YBR194W	SOY1	YNL199C	GCR2
YPL129W	TAF14	YNL059C	ARP5

Predictions at threshold level 0.75 continued...			
Gene1 of predicted pair		Gene2 of predicted pair	
Systematic Name	Standard Name	Systematic Name	Standard Name
YFL013C	IES1	YKL213C	DOA1
YLR148W	PEP3	YPL045W	VPS16
YDR202C	RAV2	YML038C	YMD8
YLR058C	SHM2	YLR370C	ARC18
YJL127C	SPT10	YGL003C	CDH1
YLR093C	NYV1	YER122C	GLO3
YNL032W	SIW14	YBR036C	CSG2
YCR065W	HCM1	YLR079W	SIC1
YMR039C	SUB1	YPL018W	CTF19
YAL051W	OAF1	YPL001W	HAT1
YCR020C-A	MAK31	YHL031C	GOS1
YLR386W	VAC14	YPL195W	APL5
YHR114W	BZZ1	YNL107W	YAF9
YLR148W	PEP3	YGL124C	MON1
YLR083C	EMP70	YPR017C	DSS4
YOR332W	VMA4	YHR060W	VMA22
YDL184C	RPL41A	YER122C	GLO3
YLR292C	SEC72	YCL001W	RER1
YML115C	VAN1	YGL215W	CLG1
YML016C	PPZ1	YBL047C	EDE1
YDR265W	PEX10	YGR077C	PEX8
YGR040W	KSS1	YBR083W	TEC1
YOR066W	MSA1	YLR398C	SKI2
YLR441C	RPS1A	YNL058C	YNL058C
YPR018W	RLF2	YOR014W	RTS1
YML001W	YPT7	YPL120W	VPS30
YCL037C	SRO9	YNL238W	KEX2
YPL024W	RMI1	YBR171W	SEC66
YDR318W	MCM21	YGR188C	BUB1

Predictions at threshold level 0.75 continued...			
Gene1 of predicted pair		Gene2 of predicted pair	
Systematic Name	Standard Name	Systematic Name	Standard Name
YOR132W	VPS17	YJL095W	BCK1
YBR171W	SEC66	YLR182W	SWI6
YGL223C	COG1	YLR309C	IMH1
YLL007C	YLL007C	YHL002W	HSE1
YNL215W	IES2	YDR440W	DOT1
YMR216C	SKY1	YNL041C	COG6
YKL139W	CTK1	YER161C	SPT2
YFR024C-A	LSB3	YNL094W	APP1
YDL116W	NUP84	YNL106C	INP52
YER144C	UBP5	YKL126W	YPK1
YPL157W	TGS1	YPR119W	CLB2
YKR001C	VPS1	YPR120C	CLB5
YIL159W	BNR1	YHR200W	RPN10
YDR245W	MNN10	YKL160W	ELF1
YNL291C	MID1	YFL033C	RIM15
YNL215W	IES2	YDR310C	SUM1
YBR108W	YBR108W	YDR207C	UME6
YGR283C	YGR283C	YMR310C	YMR310C
YLR095C	IOC2	YGL003C	CDH1
YJR032W	CPR7	YOR368W	RAD17
YBL003C	HTA2	YGR275W	RTT102
YFL023W	BUD27	YLR352W	YLR352W
YPL115C	BEM3	YHR114W	BZZ1
YDR363W-A	SEM1	YLR306W	UBC12
YHR134W	WSS1	YDR183W	PLP1
YDL059C	RAD59	YML102W	CAC2
YOR195W	SLK19	YPL161C	BEM4
YKL010C	UFD4	YLR370C	ARC18
YBR030W	YBR030W	YBR019C	GAL10

Predictions at threshold level 0.75 continued...			
Gene1 of predicted pair		Gene2 of predicted pair	
Systematic Name	Standard Name	Systematic Name	Standard Name
YOR141C	ARP8	YKL041W	VPS24
YBR030W	YBR030W	YMR214W	SCJ1
YDR153C	ENT5	YNL107W	YAF9
YKR061W	KTR2	YHR111W	UBA4
YNL291C	MID1	YCL037C	SRO9
YLR234W	TOP3	YMR106C	YKU80
YIL159W	BNR1	YOL121C	RPS19A
YOR195W	SLK19	YGL115W	SNF4
YBL102W	SFT2	YGL054C	ERV14
YGR019W	UGA1	YGR270W	YTA7
YHR084W	STE12	YBR127C	VMA2
YOR001W	RRP6	YDL226C	GCS1
YLR429W	CRN1	YOR035C	SHE4
YGL087C	MMS2	YNL107W	YAF9
YLR095C	IOC2	YCR053W	THR4
YML016C	PPZ1	YCR031C	RPS14A
YHR200W	RPN10	YJL117W	PHO86
YPR084W	YPR084W	YLR350W	ORM2
YBL008W	HIR1	YKR059W	TIF1
YPL037C	EGD1	YLR452C	SST2
YJR135C	MCM22	YJR090C	GRR1
YOR127W	RGA1	YPL037C	EGD1
YOR069W	VPS5	YOL018C	TLG2
YPL037C	EGD1	YJL183W	MNN11
YGL223C	COG1	YDR310C	SUM1
YLR381W	CTF3	YMR202W	ERG2
YPL018W	CTF19	YLR182W	SWI6
YOR001W	RRP6	YPL253C	VIK1
YBR126C	TPS1	YNL238W	KEX2

Predictions at threshold level 0.75 continued...			
Gene1 of predicted pair		Gene2 of predicted pair	
Systematic Name	Standard Name	Systematic Name	Standard Name
YER144C	UBP5	YOR290C	SNF2
YDR443C	SSN2	YJL183W	MNN11
YER116C	SLX8	YPR036W	VMA13
YGL016W	KAP122	YML057W	CMP2
YBR158W	AMN1	YGL003C	CDH1
YGR054W	YGR054W	YIL008W	URM1
YLR119W	SRN2	YML071C	COG8
YHR060W	VMA22	YLR180W	SAM1
YML062C	MFT1	YHR167W	THP2
YDR156W	RPA14	YJL183W	MNN11
YLR095C	IOC2	YNL041C	COG6
YLR079W	SIC1	YJL058C	BIT61
YHR079C	IRE1	YDR448W	ADA2
YNL307C	MCK1	YMR038C	CCS1
YHR200W	RPN10	YDR261C	EXG2
YOR337W	TEA1	YOR066W	MSA1
YML115C	VAN1	YBR260C	RGD1
YPL018W	CTF19	YJL095W	BCK1
YLR234W	TOP3	YDR289C	RTT103
YML103C	NUP188	YIR005W	IST3
YDR108W	GSG1	YGR105W	VMA21
YGR133W	PEX4	YBR181C	RPS6B
YER116C	SLX8	YPL018W	CTF19
YGL174W	BUD13	YDR363W-A	SEM1
YNL325C	FIG4	YDR261C	EXG2
YCL001W	RER1	YOR327C	SNC2
YNL248C	RPA49	YJL006C	CTK2
YGR054W	YGR054W	YGL124C	MON1
YHR161C	YAP1801	YNL238W	KEX2

Predictions at threshold level 0.75 continued...			
Gene1 of predicted pair		Gene2 of predicted pair	
Systematic Name	Standard Name	Systematic Name	Standard Name
YBL008W	HIR1	YMR198W	CIK1
YBR215W	HPC2	YNL199C	GCR2
YLR234W	TOP3	YGR054W	YGR054W
YGL147C	RPL9A	YEL053C	MAK10
YGL241W	KAP114	YBR009C	HHF1
YJR140C	HIR3	YBR215W	HPC2
YDR383C	NKP1	YDR174W	HMO1
YPR049C	ATG11	YOL082W	ATG19
YJL060W	BNA3	YOR195W	SLK19
YML115C	VAN1	YNL058C	YNL058C
YNL289W	PCL1	YLR258W	GSY2
YHR081W	LRP1	YFR036W	CDC26
YGR108W	CLB1	YJR060W	CBF1
YLR119W	SRN2	YDR137W	RGP1
YDL083C	RPS16B	YDR261C	EXG2
YDR484W	VPS52	YHR161C	YAP1801
YNL201C	PSY2	YDR254W	CHL4
YGL025C	PGD1	YGL003C	CDH1
YDL116W	NUP84	YML028W	TSA1
YPL050C	MNN9	YNL238W	KEX2
YPR084W	YPR084W	YOR132W	VPS17
YLR119W	SRN2	YPR030W	CSR2
YDR363W-A	SEM1	YOR124C	UBP2
YOR106W	VAM3	YHR012W	VPS29
YMR068W	AVO2	YNL135C	FPR1
YMR224C	MRE11	YLR398C	SKI2
YMR224C	MRE11	YBR057C	MUM2
YOR358W	HAP5	YNL107W	YAF9
YJL006C	CTK2	YHL002W	HSE1

Predictions at threshold level 0.75 continued...			
Gene1 of predicted pair		Gene2 of predicted pair	
Systematic Name	Standard Name	Systematic Name	Standard Name
YPL042C	SSN3	YNL199C	GCR2
YGR252W	GCN5	YPL256C	CLN2
YMR202W	ERG2	YPL161C	BEM4
YLR315W	NKP2	YIR005W	IST3
YLR234W	TOP3	YER161C	SPT2
YHR134W	WSS1	YCR086W	CSM1
YPL037C	EGD1	YNL076W	MKS1
YPL178W	CBC2	YPL256C	CLN2
YGL151W	NUT1	YOL067C	RTG1
YFR010W	UBP6	YGL124C	MON1
YBR107C	IML3	YKL101W	HSL1
YOR140W	SFL1	YOR008C	SLG1
YLR090W	XDJ1	YCR053W	THR4
YNL288W	CAF40	YJL183W	MNN11
YDR004W	RAD57	YGL175C	SAE2
YDR378C	LSM6	YEL013W	VAC8
YOR290C	SNF2	YLR176C	RFX1
YOR337W	TEA1	YOL121C	RPS19A
YGL147C	RPL9A	YOL081W	IRA2
YPL037C	EGD1	YLR180W	SAM1
YDR244W	PEX5	YDL078C	MDH3
YBR217W	ATG12	YPR046W	MCM16
YNL243W	SLA2	YIL084C	SDS3
YGL078C	DBP3	YBR108W	YBR108W
YCR020C-A	MAK31	YDR202C	RAV2
YMR116C	ASC1	YFR036W	CDC26
YMR224C	MRE11	YER040W	GLN3
YNR072W	HXT17	YPR046W	MCM16
YLR386W	VAC14	YPR046W	MCM16

Predictions at threshold level 0.75 continued...			
Gene1 of predicted pair		Gene2 of predicted pair	
Systematic Name	Standard Name	Systematic Name	Standard Name
YDR378C	LSM6	YOR124C	UBP2
YGR104C	SRB5	YMR199W	CLN1
YDR108W	GSG1	YML001W	YPT7
YPL256C	CLN2	YGR152C	RSR1
YMR025W	CSI1	YOL117W	RRI2
YGR072W	UPF3	YNL051W	COG5
YER110C	KAP123	YPR046W	MCM16
YBR217W	ATG12	YKR035W-A	DID2
YDL101C	DUN1	YAL015C	NTG1
YMR224C	MRE11	YOL121C	RPS19A
YMR022W	UBC7	YDR310C	SUM1
YDL077C	VAM6	YLR370C	ARC18
YLR320W	MMS22	YNL041C	COG6
YGL212W	VAM7	YPL161C	BEM4
YDR379W	RGA2	YKL101W	HSL1
YCR065W	HCM1	YLR370C	ARC18
YLR094C	GIS3	YJR025C	BNA1
YDL192W	ARF1	YMR230W	RPS10B
YOR141C	ARP8	YGL252C	RTG2
YKR099W	BAS1	YPL137C	GIP3
YNR052C	POP2	YGR252W	GCN5
YBR126C	TPS1	YNL087W	TCB2
YMR016C	SOK2	YJL095W	BCK1
YLR234W	TOP3	YLR452C	SST2
YNL145W	MFA2	YNL147W	LSM7
YLR292C	SEC72	YML016C	PPZ1
YMR133W	REC114	YLR398C	SKI2
YIL034C	CAP2	YGL253W	HXK2
YLR095C	IOC2	YLR452C	SST2

Predictions at threshold level 0.75 continued...			
Gene1 of predicted pair		Gene2 of predicted pair	
Systematic Name	Standard Name	Systematic Name	Standard Name
YBR084C-A	RPL19A	YDR363W-A	SEM1
YDR137W	RGP1	YNL199C	GCR2
YDR293C	SSD1	YMR004W	MVP1
YBR239C	YBR239C	YGR268C	HUA1
YGL134W	PCL10	YLR258W	GSY2
YLR192C	HCR1	YKL048C	ELM1
YFR010W	UBP6	YMR311C	GLC8
YAL024C	LTE1	YMR153W	NUP53
YDR318W	MCM21	YLR182W	SWI6
YER162C	RAD4	YER040W	GLN3
YDR004W	RAD57	YGL087C	MMS2
YIL159W	BNR1	YBL025W	RRN10
YHR039C-A	VMA10	YER139C	YER139C
YNL058C	YNL058C	YJR032W	CPR7
YJL124C	LSM1	YGL163C	RAD54
YPL105C	YPL105C	YDR153C	ENT5
YBR194W	SOY1	YJR140C	HIR3
YKR059W	TIF1	YDR310C	SUM1
YNL259C	ATX1	YBL047C	EDE1
YBL058W	SHP1	YPL037C	EGD1
YPL037C	EGD1	YBR036C	CSG2
YHR161C	YAP1801	YDR323C	PEP7
YER162C	RAD4	YGR188C	BUB1
YNL069C	RPL16B	YDR320C	SWA2
YPL180W	TCO89	YDL006W	PTC1
YBR200W	BEM1	YHR039C-A	VMA10
YIL076W	SEC28	YOL111C	MDY2
YDR389W	SAC7	YPL161C	BEM4
YHR156C	LIN1	YPL057C	SUR1

Predictions at threshold level 0.75 continued...			
Gene1 of predicted pair		Gene2 of predicted pair	
Systematic Name	Standard Name	Systematic Name	Standard Name
YIL053W	RHR2	YML115C	VAN1
YGL087C	MMS2	YGR019W	UGA1
YMR198W	CIK1	YGL003C	CDH1
YAL024C	LTE1	YMR016C	SOK2
YCL037C	SRO9	YNL127W	FAR11
YBL058W	SHP1	YLR371W	ROM2
YHL031C	GOS1	YCL001W	RER1
YDR379W	RGA2	YBL047C	EDE1
YOL043C	NTG2	YMR055C	BUB2
YBR041W	FAT1	YMR313C	TGL3
YIL076W	SEC28	YLR370C	ARC18
YKL048C	ELM1	YPL137C	GIP3
YMR004W	MVP1	YPR032W	SRO7
YGR261C	APL6	YMR004W	MVP1
YPL022W	RAD1	YJR035W	RAD26
YMR243C	ZRC1	YDL047W	SIT4
YOR043W	WHI2	YCR077C	PAT1
YPR030W	CSR2	YFR036W	CDC26
YKL129C	MYO3	YHR114W	BZZ1
YOR008C	SLG1	YHR012W	VPS29
YBR215W	HPC2	YOR043W	WHI2
YAL024C	LTE1	YNL238W	KEX2
YMR224C	MRE11	YNL054W	VAC7
YMR106C	YKU80	YLR233C	EST1
YML115C	VAN1	YBR127C	VMA2
YDR174W	HMO1	YBR107C	IML3
YKL135C	APL2	YOR035C	SHE4
YBR194W	SOY1	YJR035W	RAD26
YAL024C	LTE1	YLR337C	VRP1

Predictions at threshold level 0.75 continued...			
Gene1 of predicted pair		Gene2 of predicted pair	
Systematic Name	Standard Name	Systematic Name	Standard Name
YBR200W	BEM1	YKL048C	ELM1
YLR381W	CTF3	YOR141C	ARP8
YEL065W	SIT1	YKR099W	BAS1
YNL307C	MCK1	YJR063W	RPA12
YHR178W	STB5	YOR043W	WHI2
YOR127W	RGA1	YMR109W	MYO5
YPL037C	EGD1	YGR105W	VMA21
YJL154C	VPS35	YOR023C	AHC1
YCR039C	MATALPHA2	YCR084C	TUP1
YMR055C	BUB2	YGL003C	CDH1
YAL051W	OAF1	YKR001C	VPS1
YMR273C	ZDS1	YDR440W	DOT1
YPR036W	VMA13	YLR093C	NYV1
YMR016C	SOK2	YHR039C-A	VMA10
YGR108W	CLB1	YBR107C	IML3
YNL307C	MCK1	YDR261C	EXG2
YPR018W	RLF2	YPL057C	SUR1
YCR020C-A	MAK31	YER122C	GLO3
YBR030W	YBR030W	YDR358W	GGA1
YNR032W	PPG1	YNL107W	YAF9
YMR008C	PLB1	YMR127C	SAS2
YPL152W	RRD2	YGL252C	RTG2
YHR171W	ATG7	YAL030W	SNC1
YDL116W	NUP84	YPR046W	MCM16
YKL048C	ELM1	YDR183W	PLP1
YDR129C	SAC6	YML115C	VAN1
YDR477W	SNF1	YDR484W	VPS52
YFL033C	RIM15	YEL043W	YEL043W
YGL078C	DBP3	YLR441C	RPS1A

Predictions at threshold level 0.75 continued...			
Gene1 of predicted pair		Gene2 of predicted pair	
Systematic Name	Standard Name	Systematic Name	Standard Name
YOR358W	HAP5	YIL008W	URM1
YMR224C	MRE11	YNL325C	FIG4
YDR146C	SWI5	YGL163C	RAD54
YOR127W	RGA1	YDR310C	SUM1
YJR005W	APL1	YJR035W	RAD26
YKR001C	VPS1	YAR014C	BUD14
YLL007C	YLL007C	YDR254W	CHL4
YOR008C	SLG1	YLR425W	TUS1
YIL076W	SEC28	YGL025C	PGD1
YGR249W	MGA1	YLR320W	MMS22
YPL129W	TAF14	YBR200W	BEM1
YDR293C	SSD1	YBR119W	MUD1
YGL060W	YBP2	YMR055C	BUB2
YOR106W	VAM3	YLR093C	NYV1
YGR209C	TRX2	YLR309C	IMH1
YDL223C	HBT1	YLR094C	GIS3
YIL053W	RHR2	YLR094C	GIS3
YDR383C	NKP1	YHR115C	DMA1
YNL288W	CAF40	YDR289C	RTT103
YPR018W	RLF2	YBR215W	HPC2
YFR010W	UBP6	YPL022W	RAD1
YJR032W	CPR7	YHR114W	BZZ1
YGR135W	PRE9	YLR371W	ROM2
YNL087W	TCB2	YER122C	GLO3
YLR381W	CTF3	YML016C	PPZ1
YDR363W-A	SEM1	YEL013W	VAC8
YLR452C	SST2	YLR320W	MMS22
YDR443C	SSN2	YER116C	SLX8
YMR127C	SAS2	YGL253W	HXK2

Predictions at threshold level 0.75 continued...			
Gene1 of predicted pair		Gene2 of predicted pair	
Systematic Name	Standard Name	Systematic Name	Standard Name
YNL291C	MID1	YHR012W	VPS29
YMR026C	PEX12	YML115C	VAN1
YMR153W	NUP53	YNL307C	MCK1
YKL032C	IXR1	YKL048C	ELM1
YOR101W	RAS1	YPL018W	CTF19
YOR043W	WHI2	YDR261C	EXG2
YBL103C	RTG3	YML071C	COG8
YHR205W	SCH9	YLR371W	ROM2
YOL121C	RPS19A	YJL095W	BCK1
YNL325C	FIG4	YMR153W	NUP53
YNL297C	MON2	YPR032W	SRO7
YPL037C	EGD1	YGL124C	MON1
YER110C	KAP123	YOR358W	HAP5
YKL139W	CTK1	YER040W	GLN3
YNL297C	MON2	YCR066W	RAD18
YPR167C	MET16	YLR043C	TRX1
YHR039C-A	VMA10	YNR032W	PPG1
YEL065W	SIT1	YML115C	VAN1
YJR024C	MDE1	YPL246C	RBD2
YJL124C	LSM1	YOR320C	GNT1
YOR156C	NFI1	YHR115C	DMA1
YGL190C	CDC55	YML124C	TUB3
YPL037C	EGD1	YML071C	COG8
YKR020W	VPS51	YLR425W	TUS1
YEL065W	SIT1	YGL025C	PGD1
YPL022W	RAD1	YGL175C	SAE2
YPL057C	SUR1	YLR452C	SST2
YBR194W	SOY1	YGR270W	YTA7
YPL213W	LEA1	YJR050W	ISY1

Predictions at threshold level 0.75 continued...			
Gene1 of predicted pair		Gene2 of predicted pair	
Systematic Name	Standard Name	Systematic Name	Standard Name
YBR171W	SEC66	YLR094C	GIS3
YMR116C	ASC1	YDR207C	UME6
YBL008W	HIR1	YPL018W	CTF19
YOR038C	HIR2	YCR086W	CSM1
YBL079W	NUP170	YGR059W	SPR3
YML115C	VAN1	YML001W	YPT7
YLR370C	ARC18	YLR180W	SAM1
YNL236W	SIN4	YNL238W	KEX2
YOL147C	PEX11	YDR244W	PEX5
YFR032C-A	RPL29	YBR082C	UBC4
YPR084W	YPR084W	YGR270W	YTA7
YNL016W	PUB1	YDR207C	UME6
YML038C	YMD8	YFL033C	RIM15
YER161C	SPT2	YKL160W	ELF1
YOR127W	RGA1	YBR107C	IML3
YDL059C	RAD59	YPR120C	CLB5
YLR320W	MMS22	YCR053W	THR4
YJL172W	CPS1	YLR320W	MMS22
YMR127C	SAS2	YOL067C	RTG1
YJR040W	GEF1	YDL077C	VAM6
YOR080W	DIA2	YPL022W	RAD1
YNL291C	MID1	YIL076W	SEC28
YDR004W	RAD57	YOR033C	EXO1
YBR217W	ATG12	YEL060C	PRB1
YOR270C	VPH1	YBR280C	SAF1
YDL101C	DUN1	YGL031C	RPL24A
YPL161C	BEM4	YJL006C	CTK2
YHR013C	ARD1	YDR318W	MCM21
YBL003C	HTA2	YDR254W	CHL4

Predictions at threshold level 0.75 continued...			
Gene1 of predicted pair		Gene2 of predicted pair	
Systematic Name	Standard Name	Systematic Name	Standard Name
YPL152W	RRD2	YIR005W	IST3
YAL024C	LTE1	YDR310C	SUM1
YLR006C	SSK1	YDR310C	SUM1
YBL103C	RTG3	YLR320W	MMS22
YDR378C	LSM6	YGR270W	YTA7
YMR095C	SNO1	YMR026C	PEX12
YGL135W	RPL1B	YKR020W	VPS51
YML103C	NUP188	YPR046W	MCM16
YDL006W	PTC1	YNL107W	YAF9
YMR142C	RPL13B	YER139C	YER139C
YOR270C	VPH1	YER122C	GLO3
YDR245W	MNN10	YKL032C	IXR1
YKR001C	VPS1	YLR093C	NYV1
YIL008W	URM1	YJL095W	BCK1
YNL238W	KEX2	YBL025W	RRN10
YBR200W	BEM1	YGR135W	PRE9
YHL027W	RIM101	YJR032W	CPR7
YHR115C	DMA1	YOL081W	IRA2
YEL056W	HAT2	YAR014C	BUD14
YHR111W	UBA4	YHL002W	HSE1
YPL161C	BEM4	YDL077C	VAM6
YOR069W	VPS5	YDR080W	VPS41
YOR127W	RGA1	YGL237C	HAP2
YHL031C	GOS1	YNL238W	KEX2
YLR058C	SHM2	YPR046W	MCM16
YIL159W	BNR1	YKL048C	ELM1
YJR090C	GRR1	YBR114W	RAD16
YOR212W	STE4	YFL026W	STE2
YDR293C	SSD1	YOL067C	RTG1

Predictions at threshold level 0.75 continued...			
Gene1 of predicted pair		Gene2 of predicted pair	
Systematic Name	Standard Name	Systematic Name	Standard Name
YML115C	VAN1	YJL172W	CPS1
YLR292C	SEC72	YDR293C	SSD1
YDL101C	DUN1	YBL019W	APN2
YLR399C	BDF1	YLR410W	VIP1
YLR018C	POM34	YMR125W	STO1
YJR060W	CBF1	YDL006W	PTC1
YPR120C	CLB5	YJR135C	MCM22
YPL057C	SUR1	YDR469W	SDC1
YCR066W	RAD18	YHR056C	RSC30
YIL076W	SEC28	YDL006W	PTC1
YMR311C	GLC8	YBR280C	SAF1
YOR106W	VAM3	YPL037C	EGD1
YKR020W	VPS51	YLR320W	MMS22
YPL037C	EGD1	YPL161C	BEM4
YDR289C	RTT103	YLR182W	SWI6
YOL018C	TLG2	YDL077C	VAM6
YOR195W	SLK19	YLR370C	ARC18
YNL044W	YIP3	YKL119C	VPH2
YIL159W	BNR1	YHR156C	LIN1
YOL121C	RPS19A	YER139C	YER139C
YDR477W	SNF1	YGL124C	MON1
YDR443C	SSN2	YLR452C	SST2
YOR290C	SNF2	YHL025W	SNF6
YBL027W	RPL19B	YDR289C	RTT103
YIR009W	MSL1	YMR125W	STO1
YBR283C	SSH1	YDR310C	SUM1
YBR126C	TPS1	YER144C	UBP5
YDR484W	VPS52	YDL161W	ENT1
YPL234C	TFP3	YDR207C	UME6

Predictions at threshold level 0.75 continued...			
Gene1 of predicted pair		Gene2 of predicted pair	
Systematic Name	Standard Name	Systematic Name	Standard Name
YKL101W	HSL1	YDR207C	UME6
YPR051W	MAK3	YNL297C	MON2
YHR134W	WSS1	YJL124C	LSM1
YOR140W	SFL1	YOR106W	VAM3
YNL201C	PSY2	YLR315W	NKP2
YFL026W	STE2	YPL187W	MF(ALPHA)1
YBL047C	EDE1	YDR310C	SUM1
YOR358W	HAP5	YDL155W	CLB3
YOR035C	SHE4	YEL060C	PRB1
YCR081W	SRB8	YML115C	VAN1
YNL291C	MID1	YLR150W	STM1
YOR270C	VPH1	YEL060C	PRB1
YDR443C	SSN2	YLR119W	SRN2
YBR194W	SOY1	YBR215W	HPC2
YJR125C	ENT3	YHR108W	GGA2
YMR054W	STV1	YOR327C	SNC2
YNL325C	FIG4	YLR441C	RPS1A
YER110C	KAP123	YDR335W	MSN5
YPL152W	RRD2	YOL054W	PSH1
YPL253C	VIK1	YDR456W	NHX1
YBR278W	DPB3	YOR368W	RAD17
YBL103C	RTG3	YER162C	RAD4
YOR132W	VPS17	YER120W	SCS2
YPL024W	RMI1	YOL081W	IRA2
YFR010W	UBP6	YBR019C	GAL10
YDR318W	MCM21	YOL081W	IRA2
YFR031C-A	RPL2A	YLR320W	MMS22
YGR241C	YAP1802	YOR036W	PEP12
YLR399C	BDF1	YOL081W	IRA2

Predictions at threshold level 0.75 continued...			
Gene1 of predicted pair		Gene2 of predicted pair	
Systematic Name	Standard Name	Systematic Name	Standard Name
YDL077C	VAM6	YDR310C	SUM1
YDR076W	RAD55	YKL101W	HSL1
YIL076W	SEC28	YCR053W	THR4
YHR111W	UBA4	YAR014C	BUD14
YGL078C	DBP3	YDR310C	SUM1
YBR267W	REI1	YKR048C	NAP1
YML115C	VAN1	YBR019C	GAL10
YDL184C	RPL41A	YNL087W	TCB2
YOR195W	SLK19	YPR036W	VMA13
YDR320C	SWA2	YML071C	COG8
YMR116C	ASC1	YDL155W	CLB3
YOR127W	RGA1	YGL253W	HXK2
YNL307C	MCK1	YMR152W	YIM1
YMR231W	PEP5	YDR323C	PEP7
YML016C	PPZ1	YJL183W	MNN11
YHR073W	OSH3	YMR199W	CLN1
YOL061W	PRS5	YER099C	PRS2
YPL161C	BEM4	YFR036W	CDC26
YOR038C	HIR2	YEL013W	VAC8
YBR173C	UMP1	YMR284W	YKU70
YGL147C	RPL9A	YBR173C	UMP1
YIL162W	SUC2	YOR085W	OST3
YCL001W	RER1	YDR207C	UME6
YBR290W	BSD2	YDR318W	MCM21
YOR127W	RGA1	YPR046W	MCM16
YCR081W	SRB8	YNL236W	SIN4
YBL103C	RTG3	YBR019C	GAL10
YGL115W	SNF4	YDR200C	VPS64
YMR026C	PEX12	YDR484W	VPS52

Predictions at threshold level 0.75 continued...			
Gene1 of predicted pair		Gene2 of predicted pair	
Systematic Name	Standard Name	Systematic Name	Standard Name
YNL291C	MID1	YFR036W	CDC26
YOR076C	SKI7	YFR010W	UBP6
YPL137C	GIP3	YPL195W	APL5
YDL020C	RPN4	YPL253C	VIK1
YPL213W	LEA1	YBL101C	ECM21
YPL115C	BEM3	YNL107W	YAF9
YJR060W	CBF1	YLR254C	NDL1
YNL166C	BNI5	YLR447C	VMA6
YDL077C	VAM6	YPR032W	SRO7
YML016C	PPZ1	YLR254C	NDL1
YER087C-B	SBH1	YMR223W	UBP8
YGR261C	APL6	YMR216C	SKY1
YNL079C	TPM1	YOR035C	SHE4
YMR231W	PEP5	YJR090C	GRR1
YHR010W	RPL27A	YLR352W	YLR352W
YIL076W	SEC28	YPL120W	VPS30
YNL307C	MCK1	YHR200W	RPN10
YBR200W	BEM1	YLR093C	NYV1
YDR137W	RGP1	YER162C	RAD4
YDR329C	PEX3	YAL055W	PEX22
YOR132W	VPS17	YEL053C	MAK10
YOR127W	RGA1	YHL002W	HSE1
YBR126C	TPS1	YML001W	YPT7
YPL256C	CLN2	YHR010W	RPL27A
YMR284W	YKU70	YGL163C	RAD54
YIL053W	RHR2	YER139C	YER139C
YMR284W	YKU70	YNR052C	POP2
YOR112W	CEX1	YER122C	GLO3
YJL036W	SNX4	YPR032W	SRO7

Predictions at threshold level 0.75 continued...			
Gene1 of predicted pair		Gene2 of predicted pair	
Systematic Name	Standard Name	Systematic Name	Standard Name
YGR270W	YTA7	YLR233C	EST1
YML109W	ZDS2	YCR086W	CSM1
YMR202W	ERG2	YPR046W	MCM16
YMR109W	MYO5	YDR144C	MKC7
YLR192C	HCR1	YER144C	UBP5
YLR043C	TRX1	YLR093C	NYV1
YMR004W	MVP1	YKL119C	VPH2
YLR309C	IMH1	YOL108C	INO4
YDL083C	RPS16B	YLR180W	SAM1
YDR076W	RAD55	YJR090C	GRR1
YML115C	VAN1	YLR370C	ARC18
YNL041C	COG6	YDR207C	UME6
YMR095C	SNO1	YLR093C	NYV1
YOR069W	VPS5	YLR309C	IMH1
YFL013C	IES1	YER139C	YER139C
YKR020W	VPS51	YBR181C	RPS6B
YBR200W	BEM1	YNL079C	TPM1
YNL041C	COG6	YPL195W	APL5
YJR032W	CPR7	YLR233C	EST1
YML038C	YMD8	YER122C	GLO3
YDR484W	VPS52	YPR046W	MCM16
YBR108W	YBR108W	YBR280C	SAF1
YOR327C	SNC2	YFL048C	EMP47
YKL048C	ELM1	YML112W	CTK3
YER144C	UBP5	YGL212W	VAM7
YNL135C	FPR1	YOR208W	PTP2
YHR178W	STB5	YJL183W	MNN11
YFL013C	IES1	YDR440W	DOT1
YKR020W	VPS51	YEL042W	GDA1

Predictions at threshold level 0.75 continued...			
Gene1 of predicted pair		Gene2 of predicted pair	
Systematic Name	Standard Name	Systematic Name	Standard Name
YGR072W	UPF3	YHR167W	THP2
YBR171W	SEC66	YOR327C	SNC2
YHR084W	STE12	YMR164C	MSS11
YNL021W	HDA1	YPR179C	HDA3
YMR026C	PEX12	YJL172W	CPS1
YNL044W	YIP3	YLR447C	VMA6
YPR017C	DSS4	YPL195W	APL5
YMR016C	SOK2	YJL164C	TPK1
YDL002C	NHP10	YGR270W	YTA7
YPR051W	MAK3	YHR111W	UBA4
YOR132W	VPS17	YJL053W	PEP8
YKL139W	CTK1	YNL076W	MKS1
YHR156C	LIN1	YLR386W	VAC14
YGR135W	PRE9	YLR182W	SWI6
YGR019W	UGA1	YDR183W	PLP1
YIL036W	CST6	YMR089C	YTA12
YDR440W	DOT1	YER122C	GLO3
YDR225W	HTA1	YGR184C	UBR1
YDL020C	RPN4	YDR183W	PLP1
YHR134W	WSS1	YJR140C	HIR3
YLR102C	APC9	YML095C	RAD10
YBR108W	YBR108W	YFR036W	CDC26
YIL132C	CSM2	YLR376C	PSY3
YGL163C	RAD54	YKL101W	HSL1
YOR270C	VPH1	YJL183W	MNN11
YBR200W	BEM1	YBR280C	SAF1
YGL190C	CDC55	YPL269W	KAR9
YMR026C	PEX12	YER139C	YER139C
YEL051W	VMA8	YNR032W	PPG1

Predictions at threshold level 0.75 continued...			
Gene1 of predicted pair		Gene2 of predicted pair	
Systematic Name	Standard Name	Systematic Name	Standard Name
YER144C	UBP5	YLR370C	ARC18
YNL297C	MON2	YCR086W	CSM1
YGL133W	ITC1	YOR304W	ISW2
YPL024W	RMI1	YNL106C	INP52
YDR318W	MCM21	YJL183W	MNN11
YJR032W	CPR7	YJR090C	GRR1
YCR020C-A	MAK31	YGR105W	VMA21
YLR288C	MEC3	YFL023W	BUD27
YJR135C	MCM22	YLR233C	EST1
YJL004C	SYS1	YDR153C	ENT5
YDL002C	NHP10	YOR368W	RAD17
YLR192C	HCR1	YLR320W	MMS22
YKL126W	YPK1	YGL124C	MON1
YNR072W	HXT17	YOR066W	MSA1
YBR057C	MUM2	YDL013W	SLX5
YLR320W	MMS22	YPR046W	MCM16
YDR137W	RGP1	YDR289C	RTT103
YMR095C	SNO1	YMR096W	SNZ1
YMR179W	SPT21	YOL091W	SPO21
YGL237C	HAP2	YJL183W	MNN11
YML095C	RAD10	YGL003C	CDH1
YDR383C	NKP1	YML016C	PPZ1
YML001W	YPT7	YDL083C	RPS16B
YEL043W	YEL043W	YJR060W	CBF1
YDR318W	MCM21	YLR032W	RAD5
YIL053W	RHR2	YBR171W	SEC66
YMR142C	RPL13B	YOR290C	SNF2
YEL065W	SIT1	YDR310C	SUM1
YLR018C	POM34	YGL163C	RAD54

Predictions at threshold level 0.75 continued...			
Gene1 of predicted pair		Gene2 of predicted pair	
Systematic Name	Standard Name	Systematic Name	Standard Name
YML109W	ZDS2	YBR009C	HHF1
YHL031C	GOS1	YKL119C	VPH2
YDR484W	VPS52	YER040W	GLN3
YPR046W	MCM16	YDR289C	RTT103
YMR095C	SNO1	YPR036W	VMA13
YFR010W	UBP6	YHR167W	THP2
YKL116C	PRR1	YMR214W	SCJ1
YHR039C-A	VMA10	YDL077C	VAM6
YFR031C-A	RPL2A	YJL036W	SNX4
YDR440W	DOT1	YJL095W	BCK1
YMR095C	SNO1	YER162C	RAD4
YML115C	VAN1	YJL183W	MNN11
YDR192C	NUP42	YOR185C	GSP2
YOR332W	VMA4	YGR020C	VMA7
YPR046W	MCM16	YBL025W	RRN10
YJR060W	CBF1	YDR153C	ENT5
YKL025C	PAN3	YDR289C	RTT103
YML115C	VAN1	YBR274W	CHK1
YPR029C	APL4	YOR035C	SHE4
YIL053W	RHR2	YOL111C	MDY2
YMR224C	MRE11	YDR335W	MSN5
YDL101C	DUN1	YMR038C	CCS1
YGR020C	VMA7	YJL183W	MNN11
YOR138C	RUP1	YOR124C	UBP2
YLR058C	SHM2	YBR215W	HPC2
YGR270W	YTA7	YDR318W	MCM21
YDR225W	HTA1	YBR173C	UMP1
YAL024C	LTE1	YOL121C	RPS19A
YPL001W	HAT1	YGR252W	GCN5

Predictions at threshold level 0.75 continued...			
Gene1 of predicted pair		Gene2 of predicted pair	
Systematic Name	Standard Name	Systematic Name	Standard Name
YLR079W	SIC1	YOR106W	VAM3
YER161C	SPT2	YKL213C	DOA1
YMR142C	RPL13B	YDL223C	HBT1
YER116C	SLX8	YJR032W	CPR7
YDR484W	VPS52	YBR127C	VMA2
YKL048C	ELM1	YKL110C	KTI12
YNL106C	INP52	YGL124C	MON1
YNR023W	SNF12	YBR289W	SNF5
YHR111W	UBA4	YJL095W	BCK1
YMR095C	SNO1	YML001W	YPT7
YOR033C	EXO1	YDL101C	DUN1
YBR065C	ECM2	YIR009W	MSL1
YBL051C	PIN4	YLR425W	TUS1
YOR368W	RAD17	YDR310C	SUM1
YLR080W	EMP46	YEL036C	ANP1
YIL076W	SEC28	YPL195W	APL5
YIL036W	CST6	YNL054W	VAC7
YDR440W	DOT1	YMR089C	YTA12
YFR031C-A	RPL2A	YLR309C	IMH1
YMR199W	CLN1	YDR456W	NHX1
YLR386W	VAC14	YEL053C	MAK10
YEL065W	SIT1	YKL160W	ELF1
YGL121C	GPG1	YJL106W	IME2
YKR072C	SIS2	YBR215W	HPC2
YPR052C	NHP6A	YDR176W	NGG1
YFR031C-A	RPL2A	YDR293C	SSD1
YBL016W	FUS3	YDL159W	STE7
YBR108W	YBR108W	YAL002W	VPS8
YCR020C-A	MAK31	YDR153C	ENT5

Predictions at threshold level 0.75 continued...			
Gene1 of predicted pair		Gene2 of predicted pair	
Systematic Name	Standard Name	Systematic Name	Standard Name
YML095C	RAD10	YGL253W	HXK2
YLR216C	CPR6	YGR188C	BUB1
YKR101W	SIR1	YCL027W	FUS1
YKL139W	CTK1	YML071C	COG8
YMR026C	PEX12	YPR036W	VMA13
YBL047C	EDE1	YML097C	VPS9
YNL206C	RTT106	YPL256C	CLN2
YLR234W	TOP3	YBR274W	CHK1
YKL139W	CTK1	YDR335W	MSN5
YIL053W	RHR2	YKL116C	PRR1
YBR171W	SEC66	YHR060W	VMA22
YHR134W	WSS1	YLR313C	SPH1
YDL116W	NUP84	YDR004W	RAD57
YLR176C	RFX1	YCR084C	TUP1
YGL133W	ITC1	YKL032C	IXR1
YDR103W	STE5	YBR200W	BEM1
YIL076W	SEC28	YNL051W	COG5
YFR031C-A	RPL2A	YGL025C	PGD1
YJR060W	CBF1	YDL155W	CLB3
YDR174W	HMO1	YDL013W	SLX5
YNL032W	SIW14	YBR280C	SAF1
YDR129C	SAC6	YDL223C	HBT1
YLR102C	APC9	YOR127W	RGA1
YMR095C	SNO1	YHR039C-A	VMA10
YKL116C	PRR1	YOR270C	VPH1
YGR261C	APL6	YPL234C	TFP3
YDR192C	NUP42	YLR234W	TOP3
YGL087C	MMS2	YOR358W	HAP5
YOR195W	SLK19	YDL155W	CLB3

Predictions at threshold level 0.75 continued...			
Gene1 of predicted pair		Gene2 of predicted pair	
Systematic Name	Standard Name	Systematic Name	Standard Name
YNL215W	IES2	YEL013W	VAC8
YDL180W	YDL180W	YDL186W	YDL186W
YPL049C	DIG1	YGR040W	KSS1
YAL024C	LTE1	YIR009W	MSL1
YIL159W	BNR1	YKR035W-A	DID2
YKL139W	CTK1	YKR020W	VPS51
YKL126W	YPK1	YPL037C	EGD1
YDR383C	NKP1	YLR399C	BDF1
YBL103C	RTG3	YMR230W	RPS10B
YGL147C	RPL9A	YKL139W	CTK1
YBR194W	SOY1	YLR182W	SWI6
YLR090W	XDJ1	YDR207C	UME6
YBL088C	TEL1	YER068W	MOT2
YMR095C	SNO1	YMR224C	MRE11
YPR018W	RLF2	YKR072C	SIS2
YJL172W	CPS1	YDR207C	UME6
YDR264C	AKR1	YGR135W	PRE9
YOR033C	EXO1	YDL059C	RAD59
YDR254W	CHL4	YNL116W	DMA2
YNL087W	TCB2	YGL212W	VAM7
YMR026C	PEX12	YDL077C	VAM6
YLR452C	SST2	YDL047W	SIT4
YPL105C	YPL105C	YER120W	SCS2
YDR144C	MKC7	YCR086W	CSM1
YOR337W	TEA1	YIL076W	SEC28
YFR010W	UBP6	YHR081W	LRP1
YBL102W	SFT2	YOR132W	VPS17
YPR084W	YPR084W	YOR109W	INP53
YIL159W	BNR1	YNL116W	DMA2

Predictions at threshold level 0.75 continued...			
Gene1 of predicted pair		Gene2 of predicted pair	
Systematic Name	Standard Name	Systematic Name	Standard Name
YNR072W	HXT17	YEL060C	PRB1
YJL172W	CPS1	YOR043W	WHI2
YPR119W	CLB2	YDR131C	YDR131C
YLR095C	IOC2	YLR398C	SKI2
YDR137W	RGP1	YDR358W	GGA1
YHR152W	SPO12	YKL101W	HSL1
YBR200W	BEM1	YMR055C	BUB2
YJL124C	LSM1	YKL032C	IXR1
YFR031C-A	RPL2A	YDR310C	SUM1
YCR020C-A	MAK31	YNR032W	PPG1
YLR135W	SLX4	YJR035W	RAD26
YAL024C	LTE1	YER122C	GLO3
YPL042C	SSN3	YAL002W	VPS8
YPR018W	RLF2	YOR368W	RAD17
YOR059C	YOR059C	YMR152W	YIM1
YHR060W	VMA22	YJR032W	CPR7
YNL291C	MID1	YCL001W	RER1
YJR060W	CBF1	YNL041C	COG6
YHR206W	SKN7	YDL056W	MBP1
YJL154C	VPS35	YNR032W	PPG1
YGR020C	VMA7	YKR020W	VPS51
YGL115W	SNF4	YDR207C	UME6
YBR200W	BEM1	YER144C	UBP5
YPR046W	MCM16	YLR182W	SWI6
YNL307C	MCK1	YLR093C	NYV1
YDR378C	LSM6	YOR141C	ARP8
YDR443C	SSN2	YBL103C	RTG3
YHR195W	NVJ1	YPR185W	ATG13
YNL264C	PDR17	YDL005C	MED2

Predictions at threshold level 0.75 continued...			
Gene1 of predicted pair		Gene2 of predicted pair	
Systematic Name	Standard Name	Systematic Name	Standard Name
YPL253C	VIK1	YMR311C	GLC8
YBR108W	YBR108W	YHR060W	VMA22
YLR360W	VPS38	YDR202C	RAV2
YNL078W	NIS1	YKL101W	HSL1
YNL215W	IES2	YMR038C	CCS1
YLR079W	SIC1	YDR207C	UME6
YML016C	PPZ1	YNL248C	RPA49
YHR052W	CIC1	YJL047C	RTT101
YEL050C	RML2	YNL236W	SIN4
YOR212W	STE4	YJL157C	FAR1
YBR194W	SOY1	YCL037C	SRO9
YGR019W	UGA1	YDR144C	MKC7
YDR293C	SSD1	YOR112W	CEX1
YLR386W	VAC14	YHR200W	RPN10
YHR012W	VPS29	YGR105W	VMA21
YLR398C	SKI2	YER122C	GLO3
YJL136C	RPS21B	YKL101W	HSL1
YEL053C	MAK10	YML001W	YPT7
YKL110C	KTI12	YNL107W	YAF9
YGR104C	SRB5	YOL018C	TLG2
YBR200W	BEM1	YKR057W	RPS21A
YKL139W	CTK1	YGR270W	YTA7
YLR360W	VPS38	YLR447C	VMA6
YDL006W	PTC1	YGL019W	CKB1
YMR205C	PFK2	YHR200W	RPN10
YPR173C	VPS4	YGL124C	MON1
YMR198W	CIK1	YDR289C	RTT103
YNL209W	SSB2	YDR028C	REG1
YMR153W	NUP53	YMR214W	SCJ1

Predictions at threshold level 0.75 continued...			
Gene1 of predicted pair		Gene2 of predicted pair	
Systematic Name	Standard Name	Systematic Name	Standard Name
YDR176W	NGG1	YDR216W	ADR1
YGL223C	COG1	YHR039C-A	VMA10
YPL037C	EGD1	YGL115W	SNF4
YGR249W	MGA1	YDR484W	VPS52
YLR090W	XDJ1	YDL077C	VAM6
YAL024C	LTE1	YJR032W	CPR7
YJL127C	SPT10	YGL115W	SNF4
YFR036W	CDC26	YEL036C	ANP1
YLR337C	VRP1	YKL041W	VPS24
YHR060W	VMA22	YDR310C	SUM1
YAL024C	LTE1	YGR238C	KEL2
YEL060C	PRB1	YOL081W	IRA2
YBL079W	NUP170	YCR086W	CSM1
YKL135C	APL2	YOR109W	INP53
YNL206C	RTT106	YBL087C	RPL23A
YDR171W	HSP42	YNL107W	YAF9
YMR091C	NPL6	YCR020W-B	HTL1
YBL102W	SFT2	YJR033C	RAV1
YER116C	SLX8	YDL077C	VAM6
YGL147C	RPL9A	YER144C	UBP5
YKL007W	CAP1	YDR245W	MNN10
YML103C	NUP188	YKR061W	KTR2
YDL184C	RPL41A	YOR066W	MSA1
YEL056W	HAT2	YBR114W	RAD16
YJR135C	MCM22	YOL081W	IRA2
YGL147C	RPL9A	YLR441C	RPS1A
YPL234C	TFP3	YHR012W	VPS29
YBR108W	YBR108W	YGL003C	CDH1
YJL080C	SCP160	YFL026W	STE2

Predictions at threshold level 0.75 continued...			
Gene1 of predicted pair		Gene2 of predicted pair	
Systematic Name	Standard Name	Systematic Name	Standard Name
YIL034C	CAP2	YGR080W	TWF1
YFR032C-A	RPL29	YBR200W	BEM1
YBL051C	PIN4	YDR318W	MCM21
YJL197W	UBP12	YFL026W	STE2
YGR241C	YAP1802	YJL053W	PEP8
YLR192C	HCR1	YKR099W	BAS1
YDL116W	NUP84	YJR024C	MDE1
YDR254W	CHL4	YDR155C	CPR1
YJR063W	RPA12	YGL163C	RAD54
YER129W	SAK1	YJL187C	SWE1
YLR079W	SIC1	YLR429W	CRN1
YOL051W	GAL11	YKR048C	NAP1
YGL163C	RAD54	YLR032W	RAD5
YNL201C	PSY2	YLR182W	SWI6
YGL174W	BUD13	YJR135C	MCM22
YBL058W	SHP1	YMR230W	RPS10B
YMR172W	HOT1	YEL056W	HAT2
YLR313C	SPH1	YDR207C	UME6
YBR194W	SOY1	YGL200C	EMP24
YMR055C	BUB2	YNL236W	SIN4
YGL134W	PCL10	YPL031C	PHO85
YJR032W	CPR7	YGL124C	MON1
YNL032W	SIW14	YJL095W	BCK1
YGR229C	SMI1	YHR205W	SCH9
YNL264C	PDR17	YBL078C	ATG8
YNL106C	INP52	YKR061W	KTR2
YBL008W	HIR1	YLR435W	TSR2
YOR023C	AHC1	YGR270W	YTA7
YPR084W	YPR084W	YNL127W	FAR11

Predictions at threshold level 0.75 continued...			
Gene1 of predicted pair		Gene2 of predicted pair	
Systematic Name	Standard Name	Systematic Name	Standard Name
YDR176W	NGG1	YGL025C	PGD1
YKL160W	ELF1	YEL013W	VAC8
YOR290C	SNF2	YMR038C	CCS1
YEL053C	MAK10	YHL002W	HSE1
YBR173C	UMP1	YML001W	YPT7
YPL152W	RRD2	YGR188C	BUB1
YEL053C	MAK10	YBL025W	RRN10
YLR399C	BDF1	YGL252C	RTG2
YBL008W	HIR1	YPR119W	CLB2
YNL054W	VAC7	YLR399C	BDF1
YCL067C	HMLALPHA2	YNL330C	RPD3
YGR020C	VMA7	YNL041C	COG6
YEL051W	VMA8	YDR207C	UME6
YIL036W	CST6	YML016C	PPZ1
YOR039W	CKB2	YBR274W	CHK1
YPL253C	VIK1	YFR036W	CDC26
YOR008C	SLG1	YNL199C	GCR2
YOR080W	DIA2	YER161C	SPT2
YPL234C	TFP3	YJR032W	CPR7
YPR018W	RLF2	YNL206C	RTT106
YBR108W	YBR108W	YGL025C	PGD1
YPR052C	NHP6A	YJR140C	HIR3
YDR183W	PLP1	YEL056W	HAT2
YGL151W	NUT1	YJL190C	RPS22A
YOR035C	SHE4	YDL226C	GCS1
YDR310C	SUM1	YMR214W	SCJ1
YKL116C	PRR1	YPL195W	APL5
YGR261C	APL6	YJR060W	CBF1
YMR054W	STV1	YLR309C	IMH1

Predictions at threshold level 0.75 continued...			
Gene1 of predicted pair		Gene2 of predicted pair	
Systematic Name	Standard Name	Systematic Name	Standard Name
YLR288C	MEC3	YLR352W	YLR352W
YHR013C	ARD1	YNL225C	CNM67
YDR080W	VPS41	YHL020C	OPI1
YLR176C	RFX1	YGL025C	PGD1
YDR176W	NGG1	YPR072W	NOT5
YGL078C	DBP3	YHR081W	LRP1
YGL212W	VAM7	YDR358W	GGA1
YDR156W	RPA14	YBR041W	FAT1
YDR137W	RGP1	YLR320W	MMS22
YER087C-B	SBH1	YDL074C	BRE1
YER110C	KAP123	YNL030W	HHF2
YGR261C	APL6	YPL161C	BEM4
YNL230C	ELA1	YOR028C	CIN5
YFR010W	UBP6	YDR260C	SWM1
YGR070W	ROM1	YJL095W	BCK1
YOR337W	TEA1	YBL058W	SHP1
YML071C	COG8	YJR033C	RAV1
YLR119W	SRN2	YPR173C	VPS4
YMR091C	NPL6	YPL106C	SSE1
YOR080W	DIA2	YMR055C	BUB2
YPL115C	BEM3	YML109W	ZDS2
YGL147C	RPL9A	YMR026C	PEX12
YJL172W	CPS1	YLR371W	ROM2
YPR051W	MAK3	YEL051W	VMA8
YBL008W	HIR1	YDL155W	CLB3
YNR072W	HXT17	YML001W	YPT7
YML016C	PPZ1	YLR452C	SST2
YDR443C	SSN2	YHR060W	VMA22
YJR060W	CBF1	YKR030W	GMH1

Predictions at threshold level 0.75 continued...			
Gene1 of predicted pair		Gene2 of predicted pair	
Systematic Name	Standard Name	Systematic Name	Standard Name
YHL020C	OPI1	YAR014C	BUD14
YBL103C	RTG3	YLR441C	RPS1A
YHR156C	LIN1	YDR477W	SNF1
YOR337W	TEA1	YDL077C	VAM6
YMR142C	RPL13B	YCR053W	THR4
YOR195W	SLK19	YLR150W	STM1
YKL160W	ELF1	YDR440W	DOT1
YDL083C	RPS16B	YBL025W	RRN10
YNL044W	YIP3	YKR001C	VPS1
YKL048C	ELM1	YER068W	MOT2
YDR383C	NKP1	YML112W	CTK3
YGL147C	RPL9A	YKR035W-A	DID2
YML016C	PPZ1	YPR046W	MCM16
YMR109W	MYO5	YJR032W	CPR7
YHR012W	VPS29	YDL192W	ARF1
YGL212W	VAM7	YOL081W	IRA2
YPR030W	CSR2	YMR214W	SCJ1
YPR052C	NHP6A	YOL145C	CTR9
YMR205C	PFK2	YKL048C	ELM1
YGL003C	CDH1	YDL077C	VAM6
YER114C	BOI2	YLR362W	STE11
YLR371W	ROM2	YOR035C	SHE4
YOR043W	WHI2	YER162C	RAD4
YBR065C	ECM2	YGL174W	BUD13
YOR033C	EXO1	YNL291C	MID1
YER143W	DDI1	YAL030W	SNC1
YNL307C	MCK1	YDR076W	RAD55
YDL223C	HBT1	YKR020W	VPS51
YNL215W	IES2	YLR381W	CTF3

Predictions at threshold level 0.75 continued...			
**Gene1 of predicted pair**		**Gene2 of predicted pair**	
**Systematic Name**	**Standard Name**	**Systematic Name**	**Standard Name**
YJL095W	BCK1	YOR141C	ARP8
YDL116W	NUP84	YKL068W	NUP100
YGL054C	ERV14	YPL195W	APL5
YLR090W	XDJ1	YEL065W	SIT1
YGL212W	VAM7	YOR035C	SHE4
YDR448W	ADA2	YNL236W	SIN4
YPR051W	MAK3	YOR327C	SNC2
YBR200W	BEM1	YLR182W	SWI6
YMR172W	HOT1	YDR289C	RTT103
YBL058W	SHP1	YMR214W	SCJ1
YER044C	ERG28	YDR207C	UME6
YKL139W	CTK1	YBR108W	YBR108W
YAL024C	LTE1	YLR370C	ARC18
YNL248C	RPA49	YLR315W	NKP2
YPL024W	RMI1	YGL212W	VAM7
YLR360W	VPS38	YLR386W	VAC14
YBR171W	SEC66	YOR275C	RIM20
YBL008W	HIR1	YLR381W	CTF3
YMR016C	SOK2	YOR035C	SHE4
YOR312C	RPL20B	YJL095W	BCK1
YOR270C	VPH1	YDL083C	RPS16B
YBR107C	IML3	YJR035W	RAD26
YBR173C	UMP1	YNR072W	HXT17
YGL033W	HOP2	YMR142C	RPL13B
YCR063W	BUD31	YDR163W	CWC15
YGR188C	BUB1	YLR182W	SWI6
YHR098C	SFB3	YLR085C	ARP6
YDL059C	RAD59	YKL068W	NUP100
YBL025W	RRN10	YMR214W	SCJ1

Predictions at threshold level 0.75 continued...			
Gene1 of predicted pair		Gene2 of predicted pair	
Systematic Name	Standard Name	Systematic Name	Standard Name
YGR071C	YGR071C	YOR308C	SNU66
YDL185W	TFP1	YHR039C-A	VMA10
YOR066W	MSA1	YLR180W	SAM1
YNL106C	INP52	YBR107C	IML3
YGL078C	DBP3	YHR156C	LIN1
YBL008W	HIR1	YDR289C	RTT103
YGR072W	UPF3	YKR020W	VPS51
YHR111W	UBA4	YLR425W	TUS1
YBR239C	YBR239C	YLR064W	YLR064W
YLR079W	SIC1	YNL236W	SIN4
YGR020C	VMA7	YCR066W	RAD18
YBR173C	UMP1	YKL114C	APN1
YGL151W	NUT1	YJL095W	BCK1
YGL163C	RAD54	YML028W	TSA1
YLR094C	GIS3	YLR180W	SAM1
YLR447C	VMA6	YDL185W	TFP1
YDR443C	SSN2	YBR200W	BEM1
YNL032W	SIW14	YOR035C	SHE4
YKR061W	KTR2	YAL010C	MDM10
YLR094C	GIS3	YEL060C	PRB1
YDR443C	SSN2	YOL121C	RPS19A
YHR205W	SCH9	YDL074C	BRE1
YOR035C	SHE4	YEL036C	ANP1
YKL101W	HSL1	YML028W	TSA1
YBR194W	SOY1	YOR195W	SLK19
YKL006W	RPL14A	YLR370C	ARC18
YDR260C	SWM1	YAR014C	BUD14
YFR010W	UBP6	YDL013W	SLX5
YLR234W	TOP3	YMR284W	YKU70

Predictions at threshold level 0.75 continued...			
Gene1 of predicted pair		Gene2 of predicted pair	
Systematic Name	Standard Name	Systematic Name	Standard Name
YDR422C	SIP1	YJL187C	SWE1
YLR399C	BDF1	YNL030W	HHF2
YLR095C	IOC2	YPR046W	MCM16
YOR127W	RGA1	YGR188C	BUB1
YJL095W	BCK1	YHL002W	HSE1
YPL161C	BEM4	YBR019C	GAL10
YGL178W	MPT5	YLR085C	ARP6
YLR192C	HCR1	YJR084W	CSN12
YDR363W-A	SEM1	YGR135W	PRE9
YPR030W	CSR2	YLR180W	SAM1
YNL307C	MCK1	YHR013C	ARD1
YBR009C	HHF1	YDR227W	SIR4
YHR200W	RPN10	YDR076W	RAD55
YIL053W	RHR2	YMR311C	GLC8
YBR127C	VMA2	YDL161W	ENT1
YJR032W	CPR7	YER122C	GLO3
YOR195W	SLK19	YBR084C-A	RPL19A
YPR084W	YPR084W	YDR202C	RAV2
YCR053W	THR4	YER122C	GLO3
YKL139W	CTK1	YKR061W	KTR2
YGL078C	DBP3	YPR030W	CSR2
YLR288C	MEC3	YCR086W	CSM1
YFR010W	UBP6	YGR071C	YGR071C
YNL079C	TPM1	YKL161C	YKL161C
YPL042C	SSN3	YGL025C	PGD1
YAL024C	LTE1	YOL054W	PSH1
YGL054C	ERV14	YNL238W	KEX2
YBR126C	TPS1	YCR088W	ABP1
YJR033C	RAV1	YKR030W	GMH1

Predictions at threshold level 0.75 continued...			
Gene1 of predicted pair		Gene2 of predicted pair	
Systematic Name	Standard Name	Systematic Name	Standard Name
YLR234W	TOP3	YCR031C	RPS14A
YDL160C	DHH1	YPR163C	TIF3
YMR026C	PEX12	YGL153W	PEX14
YKR057W	RPS21A	YGR135W	PRE9
YBL058W	SHP1	YOR270C	VPH1
YLR095C	IOC2	YBR030W	YBR030W
YDR245W	MNN10	YBR082C	UBC4
YDR137W	RGP1	YKR020W	VPS51
YKL139W	CTK1	YEL053C	MAK10
YNL032W	SIW14	YBL025W	RRN10
YLR320W	MMS22	YMR214W	SCJ1
YDR443C	SSN2	YGL212W	VAM7
YGR275W	RTT102	YNR023W	SNF12
YDL101C	DUN1	YDR477W	SNF1
YGR270W	YTA7	YBR114W	RAD16
YBR200W	BEM1	YPL137C	GIP3
YLR018C	POM34	YPL256C	CLN2
YMR026C	PEX12	YEL060C	PRB1
YPL024W	RMI1	YNL215W	IES2
YDL184C	RPL41A	YLR180W	SAM1
YPL024W	RMI1	YER162C	RAD4
YPR120C	CLB5	YHR115C	DMA1
YER059W	PCL6	YIL050W	PCL7
YJL151C	SNA3	YHR016C	YSC84
YIL008W	URM1	YGL031C	RPL24A
YGL237C	HAP2	YIL008W	URM1
YBL103C	RTG3	YMR311C	GLC8
YMR202W	ERG2	YPR173C	VPS4
YMR116C	ASC1	YJL095W	BCK1

Predictions at threshold level 0.75 continued...			
Gene1 of predicted pair		Gene2 of predicted pair	
Systematic Name	Standard Name	Systematic Name	Standard Name
YMR273C	ZDS1	YNL199C	GCR2
YEL043W	YEL043W	YGR089W	NNF2
YBR260C	RGD1	YLR371W	ROM2
YOR106W	VAM3	YDR155C	CPR1
YLR234W	TOP3	YPL001W	HAT1
YBR289W	SNF5	YOR064C	YNG1
YPL115C	BEM3	YGR270W	YTA7
YIL007C	NAS2	YPL161C	BEM4
YKL126W	YPK1	YPL195W	APL5
YKL010C	UFD4	YHR200W	RPN10
YGL147C	RPL9A	YFL023W	BUD27
YDR156W	RPA14	YOL054W	PSH1
YFR030W	MET10	YFR036W	CDC26
YKL116C	PRR1	YNR072W	HXT17
YGR261C	APL6	YOR109W	INP53
YJL187C	SWE1	YNL116W	DMA2
YLR362W	STE11	YBL016W	FUS3
YMR038C	CCS1	YGR188C	BUB1
YDR320C	SWA2	YHR060W	VMA22
YLR350W	ORM2	YAL002W	VPS8
YMR095C	SNO1	YLR441C	RPS1A
YFR010W	UBP6	YLR441C	RPS1A
YGR249W	MGA1	YDL184C	RPL41A
YGL223C	COG1	YGL115W	SNF4
YHR111W	UBA4	YNL225C	CNM67
YPR036W	VMA13	YPR046W	MCM16
YLR386W	VAC14	YPL037C	EGD1
YDR261C	EXG2	YPR046W	MCM16
YGR184C	UBR1	YDR318W	MCM21

Predictions at threshold level 0.75 continued...			
Gene1 of predicted pair		Gene2 of predicted pair	
Systematic Name	Standard Name	Systematic Name	Standard Name
YPR173C	VPS4	YKL002W	DID4
YDR383C	NKP1	YHR111W	UBA4
YOR112W	CEX1	YPL195W	APL5
YBR274W	CHK1	YDR289C	RTT103
YBR126C	TPS1	YMR016C	SOK2
YJL058C	BIT61	YJR060W	CBF1
YJL210W	PEX2	YOL044W	PEX15
YLR381W	CTF3	YMR038C	CCS1
YBR084C-A	RPL19A	YPL161C	BEM4
YDR443C	SSN2	YNR072W	HXT17
YER120W	SCS2	YGR105W	VMA21
YER107C	GLE2	YER016W	BIM1
YKL048C	ELM1	YFR036W	CDC26
YOR337W	TEA1	YHR200W	RPN10
YBL103C	RTG3	YGL212W	VAM7
YGR249W	MGA1	YGL212W	VAM7
YBL079W	NUP170	YGL175C	SAE2
YHR056C	RSC30	YDL047W	SIT4
YEL043W	YEL043W	YNL041C	COG6
YDL052C	SLC1	YKL094W	YJU3
YOL111C	MDY2	YLR370C	ARC18
YNL069C	RPL16B	YML071C	COG8
YNL229C	URE2	YIL008W	URM1
YGR014W	MSB2	YGL200C	EMP24
YER118C	SHO1	YHL002W	HSE1
YIL053W	RHR2	YNL307C	MCK1
YHR200W	RPN10	YOR014W	RTS1
YOR023C	AHC1	YER110C	KAP123
YMR095C	SNO1	YFL023W	BUD27

Predictions at threshold level 0.75 continued...			
Gene1 of predicted pair		Gene2 of predicted pair	
Systematic Name	Standard Name	Systematic Name	Standard Name
YDL223C	HBT1	YOR043W	WHI2
YDL101C	DUN1	YKR072C	SIS2
YMR133W	REC114	YOR035C	SHE4
YHR012W	VPS29	YIL133C	RPL16A
YLR396C	VPS33	YOR035C	SHE4
YDR293C	SSD1	YEL051W	VMA8
YLR360W	VPS38	YPL037C	EGD1
YDR477W	SNF1	YER162C	RAD4
YDR329C	PEX3	YOL044W	PEX15
YAL024C	LTE1	YJR056C	YJR056C
YNL116W	DMA2	YGR188C	BUB1
YKL126W	YPK1	YMR230W	RPS10B
YLR288C	MEC3	YGL163C	RAD54
YLR386W	VAC14	YHR115C	DMA1
YMR164C	MSS11	YNL199C	GCR2
YGL033W	HOP2	YBL025W	RRN10
YJR060W	CBF1	YPL018W	CTF19
YNL166C	BNI5	YDL155W	CLB3
YGL237C	HAP2	YEL056W	HAT2
YDR329C	PEX3	YDR244W	PEX5
YMR127C	SAS2	YER110C	KAP123
YIL148W	RPL40A	YMR164C	MSS11
YAL024C	LTE1	YLR398C	SKI2
YMR216C	SKY1	YOL018C	TLG2
YNL215W	IES2	YBR274W	CHK1
YGR261C	APL6	YGR105W	VMA21
YBR274W	CHK1	YER161C	SPT2
YIL159W	BNR1	YOR327C	SNC2
YGL038C	OCH1	YML071C	COG8

Predictions at threshold level 0.75 continued...			
Gene1 of predicted pair		Gene2 of predicted pair	
Systematic Name	Standard Name	Systematic Name	Standard Name
YBR194W	SOY1	YPL057C	SUR1
YDR103W	STE5	YLR313C	SPH1
YGR019W	UGA1	YGL253W	HXK2
YNL238W	KEX2	YNR032W	PPG1
YIL076W	SEC28	YKR001C	VPS1
YNL325C	FIG4	YEL053C	MAK10
YJL124C	LSM1	YNL199C	GCR2
YLR410W	VIP1	YKR048C	NAP1
YGR040W	KSS1	YLR452C	SST2
YMR153W	NUP53	YOL121C	RPS19A
YFR031C-A	RPL2A	YGL115W	SNF4
YGR020C	VMA7	YEL051W	VMA8
YGL025C	PGD1	YER040W	GLN3
YKL110C	KTI12	YHR115C	DMA1
YBR057C	MUM2	YDR440W	DOT1
YML103C	NUP188	YKR024C	DBP7
YBL058W	SHP1	YNL238W	KEX2
YLR240W	VPS34	YDL226C	GCS1
YOR112W	CEX1	YOR327C	SNC2
YLL039C	UBI4	YPR154W	PIN3
YKL006W	RPL14A	YGL153W	PEX14
YBR194W	SOY1	YML097C	VPS9
YGL237C	HAP2	YDR254W	CHL4
YOR191W	RIS1	YPR119W	CLB2
YPR131C	NAT3	YMR198W	CIK1
YCR066W	RAD18	YNL107W	YAF9
YJL029C	VPS53	YKR020W	VPS51
YGR004W	PEX31	YDR479C	PEX29
YBR107C	IML3	YJR063W	RPA12

Predictions at threshold level 0.75 continued...			
Gene1 of predicted pair		Gene2 of predicted pair	
Systematic Name	Standard Name	Systematic Name	Standard Name
YER139C	YER139C	YEL060C	PRB1
YDL013W	SLX5	YDR254W	CHL4
YOR156C	NFI1	YOR106W	VAM3
YDL006W	PTC1	YJL187C	SWE1
YOL061W	PRS5	YBL068W	PRS4
YDR080W	VPS41	YNL147W	LSM7
YGL190C	CDC55	YPL240C	HSP82
YGL115W	SNF4	YMR054W	STV1
YOR112W	CEX1	YPR032W	SRO7
YOR080W	DIA2	YHL031C	GOS1
YOL051W	GAL11	YGL025C	PGD1
YHL031C	GOS1	YPL195W	APL5
YJL053W	PEP8	YOR112W	CEX1
YGL212W	VAM7	YBR019C	GAL10
YNL238W	KEX2	YEL036C	ANP1
YNL291C	MID1	YNL082W	PMS1
YOR195W	SLK19	YHR115C	DMA1
YMR142C	RPL13B	YMR152W	YIM1
YHR060W	VMA22	YOR035C	SHE4
YGL078C	DBP3	YPR046W	MCM16
YHR156C	LIN1	YER040W	GLN3
YDL155W	CLB3	YJR032W	CPR7
YJL124C	LSM1	YKR026C	GCN3
YOL121C	RPS19A	YPL195W	APL5
YLR094C	GIS3	YML001W	YPT7
YPL152W	RRD2	YGR135W	PRE9
YKL116C	PRR1	YKL126W	YPK1
YGL163C	RAD54	YAL009W	SPO7
YGL060W	YBP2	YDL047W	SIT4

Predictions at threshold level 0.75 continued...			
Gene1 of predicted pair		Gene2 of predicted pair	
Systematic Name	Standard Name	Systematic Name	Standard Name
YFL023W	BUD27	YOL121C	RPS19A
YML115C	VAN1	YKL048C	ELM1
YDL155W	CLB3	YJR135C	MCM22
YBR030W	YBR030W	YGL212W	VAM7
YGL033W	HOP2	YBR171W	SEC66
YJR084W	CSN12	YAL002W	VPS8
YGR020C	VMA7	YNL238W	KEX2
YDR146C	SWI5	YHR039C-A	VMA10
YDR080W	VPS41	YNL041C	COG6
YDR335W	MSN5	YOR124C	UBP2
YNL297C	MON2	YDR155C	CPR1
YEL009C	GCN4	YGL066W	SGF73
YOR191W	RIS1	YJL176C	SWI3
YKL213C	DOA1	YCR077C	PAT1
YBR030W	YBR030W	YBR036C	CSG2
YFR010W	UBP6	YGL174W	BUD13
YGL133W	ITC1	YGL066W	SGF73
YJL004C	SYS1	YEL051W	VMA8
YPL161C	BEM4	YDR440W	DOT1
YOR083W	WHI5	YJL058C	BIT61
YBL051C	PIN4	YHL002W	HSE1
YDR108W	GSG1	YJR033C	RAV1
YLR079W	SIC1	YIL103W	DPH1
YER144C	UBP5	YOR043W	WHI2
YIR005W	IST3	YJL006C	CTK2
YJR063W	RPA12	YPR046W	MCM16
YGL147C	RPL9A	YDR261C	EXG2
YHR178W	STB5	YEL053C	MAK10
YLR083C	EMP70	YAR014C	BUD14

Predictions at threshold level 0.75 continued...			
Gene1 of predicted pair		Gene2 of predicted pair	
Systematic Name	Standard Name	Systematic Name	Standard Name
YKL010C	UFD4	YDR129C	SAC6
YDL020C	RPN4	YDR155C	CPR1
YPL042C	SSN3	YIL076W	SEC28
YPL256C	CLN2	YHR108W	GGA2
YDR379W	RGA2	YHR115C	DMA1
YHR167W	THP2	YMR038C	CCS1
YGL054C	ERV14	YML071C	COG8
YDL160C	DHH1	YDR422C	SIP1
YPR036W	VMA13	YBR036C	CSG2
YJR084W	CSN12	YDL192W	ARF1
YDR057W	YOS9	YDR144C	MKC7
YLR135W	SLX4	YBR082C	UBC4
YKL025C	PAN3	YGR270W	YTA7
YGR241C	YAP1802	YLR396C	VPS33
YNL206C	RTT106	YNL030W	HHF2
YLR337C	VRP1	YHR178W	STB5
YLR109W	AHP1	YLR043C	TRX1
YMR224C	MRE11	YHR060W	VMA22
YIL002C	INP51	YGL212W	VAM7
YNL206C	RTT106	YKL032C	IXR1
YJL004C	SYS1	YPL120W	VPS30
YPL022W	RAD1	YDR200C	VPS64
YPL045W	VPS16	YDL077C	VAM6
YDR004W	RAD57	YGL163C	RAD54
YDR383C	NKP1	YLR315W	NKP2
YMR152W	YIM1	YBL025W	RRN10
YMR116C	ASC1	YHL002W	HSE1
YEL015W	EDC3	YER032W	FIR1
YJL124C	LSM1	YDR183W	PLP1

Predictions at threshold level 0.75 continued...			
Gene1 of predicted pair		Gene2 of predicted pair	
Systematic Name	Standard Name	Systematic Name	Standard Name
YML100W	TSL1	YFL033C	RIM15
YPL018W	CTF19	YML028W	TSA1
YMR153W	NUP53	YPL246C	RBD2
YBR173C	UMP1	YBR217W	ATG12
YML102W	CAC2	YOR368W	RAD17
YJL124C	LSM1	YLR350W	ORM2
YMR095C	SNO1	YNL238W	KEX2
YKL139W	CTK1	YNL307C	MCK1
YPR018W	RLF2	YDR318W	MCM21
YPL042C	SSN3	YDL047W	SIT4
YDL020C	RPN4	YOR127W	RGA1
YKR099W	BAS1	YDL077C	VAM6
YDL020C	RPN4	YLR360W	VPS38
YDL161W	ENT1	YHR108W	GGA2
YPR028W	YOP1	YDL077C	VAM6
YHR039C-A	VMA10	YER162C	RAD4
YDL002C	NHP10	YKL213C	DOA1
YOL145C	CTR9	YGL237C	HAP2
YLR288C	MEC3	YHR178W	STB5
YHR200W	RPN10	YDL083C	RPS16B
YNL243W	SLA2	YGR078C	PAC10
YMR224C	MRE11	YBR036C	CSG2
YLR429W	CRN1	YDR200C	VPS64
YBR019C	GAL10	YLR180W	SAM1
YOR195W	SLK19	YML095C	RAD10
YIL159W	BNR1	YOR270C	VPH1
YFL013C	IES1	YPL042C	SSN3
YNL078W	NIS1	YHR114W	BZZ1
YDR293C	SSD1	YEL043W	YEL043W

Predictions at threshold level 0.75 continued...			
Gene1 of predicted pair		Gene2 of predicted pair	
Systematic Name	Standard Name	Systematic Name	Standard Name
YEL065W	SIT1	YER162C	RAD4
YDL223C	HBT1	YHR039C-A	VMA10
YER044C	ERG28	YLR094C	GIS3
YJL187C	SWE1	YGR152C	RSR1
YJL172W	CPS1	YNL307C	MCK1
YPL115C	BEM3	YLR360W	VPS38
YMR004W	MVP1	YGL115W	SNF4
YOR132W	VPS17	YMR109W	MYO5
YGL078C	DBP3	YNL325C	FIG4
YLR234W	TOP3	YDR146C	SWI5
YDR254W	CHL4	YKL114C	APN1
YLR083C	EMP70	YOR069W	VPS5
YKR035W-A	DID2	YNL041C	COG6
YLR102C	APC9	YDL047W	SIT4
YKL139W	CTK1	YKL126W	YPK1
YNL079C	TPM1	YLR370C	ARC18
YLR090W	XDJ1	YOR337W	TEA1
YPL042C	SSN3	YDR137W	RGP1
YAL016W	TPD3	YGL190C	CDC55
YBR108W	YBR108W	YMR133W	REC114
YBR131W	CCZ1	YNL297C	MON2
YBR279W	PAF1	YGL178W	MPT5
YOL111C	MDY2	YML001W	YPT7
YDL020C	RPN4	YBR020W	GAL1
YBL021C	HAP3	YGR188C	BUB1
YER116C	SLX8	YHR200W	RPN10
YOL145C	CTR9	YHR200W	RPN10
YDL020C	RPN4	YHR114W	BZZ1
YML103C	NUP188	YDR076W	RAD55

Predictions at threshold level 0.75 continued...			
Gene1 of predicted pair		Gene2 of predicted pair	
Systematic Name	Standard Name	Systematic Name	Standard Name
YGR020C	VMA7	YML071C	COG8
YOR106W	VAM3	YJL004C	SYS1
YEL043W	YEL043W	YJR033C	RAV1
YLR096W	KIN2	YPR032W	SRO7
YBL085W	BOI1	YGR152C	RSR1
YJL154C	VPS35	YHR161C	YAP1801
YGR249W	MGA1	YER122C	GLO3
YPL024W	RMI1	YOR290C	SNF2
YGL124C	MON1	YER040W	GLN3
YGL087C	MMS2	YMR224C	MRE11
YIL103W	DPH1	YPR046W	MCM16
YMR153W	NUP53	YFR036W	CDC26
YLR079W	SIC1	YDR146C	SWI5
YER139C	YER139C	YBR280C	SAF1
YBL047C	EDE1	YBL087C	RPL23A
YMR199W	CLN1	YHR115C	DMA1
YLR337C	VRP1	YKL048C	ELM1
YCR066W	RAD18	YDL006W	PTC1
YOR368W	RAD17	YOR014W	RTS1
YJL172W	CPS1	YOR035C	SHE4
YDR477W	SNF1	YER027C	GAL83
YNL106C	INP52	YHR134W	WSS1
YDL020C	RPN4	YKL160W	ELF1
YLR079W	SIC1	YCR086W	CSM1
YGR072W	UPF3	YHR077C	NMD2
YOR132W	VPS17	YGL124C	MON1
YNR072W	HXT17	YOR035C	SHE4
YNL058C	YNL058C	YNL307C	MCK1
YIL076W	SEC28	YKL126W	YPK1

Predictions at threshold level 0.75 continued...			
Gene1 of predicted pair		Gene2 of predicted pair	
Systematic Name	Standard Name	Systematic Name	Standard Name
YGR275W	RTT102	YHR056C	RSC30
YNR072W	HXT17	YOL111C	MDY2
YOR127W	RGA1	YMR216C	SKY1
YPL253C	VIK1	YDL155W	CLB3
YNL041C	COG6	YGR188C	BUB1
YJL183W	MNN11	YBR082C	UBC4
YDR146C	SWI5	YGL148W	ARO2
YNL243W	SLA2	YPR173C	VPS4
YMR055C	BUB2	YGL250W	RMR1
YPL050C	MNN9	YKL213C	DOA1
YJR060W	CBF1	YDL077C	VAM6
YIL053W	RHR2	YKL126W	YPK1
YJR060W	CBF1	YOL018C	TLG2
YIL076W	SEC28	YJL172W	CPS1
YMR276W	DSK2	YPL248C	GAL4
YKR035W-A	DID2	YLR370C	ARC18
YNL238W	KEX2	YDL006W	PTC1
YDL116W	NUP84	YLR234W	TOP3
YMR142C	RPL13B	YMR016C	SOK2
YJL004C	SYS1	YLR309C	IMH1
YGR081C	SLX9	YKR095W	MLP1
YFR030W	MET10	YGL252C	RTG2
YER044C	ERG28	YPL195W	APL5
YML102W	CAC2	YPR119W	CLB2
YLR090W	XDJ1	YMR230W	RPS10B
YOR039W	CKB2	YJR032W	CPR7
YGR019W	UGA1	YHR134W	WSS1
YPR084W	YPR084W	YOR038C	HIR2
YPR017C	DSS4	YHR060W	VMA22

Predictions at threshold level 0.75 continued...			
Gene1 of predicted pair		Gene2 of predicted pair	
Systematic Name	Standard Name	Systematic Name	Standard Name
YLR435W	TSR2	YLR350W	ORM2
YLR315W	NKP2	YJR135C	MCM22
YPL037C	EGD1	YKR001C	VPS1
YDR137W	RGP1	YMR038C	CCS1
YMR039C	SUB1	YNL206C	RTT106
YNL325C	FIG4	YMR152W	YIM1
YNL079C	TPM1	YKL041W	VPS24
YMR039C	SUB1	YDR318W	MCM21
YPL037C	EGD1	YJR032W	CPR7
YOL051W	GAL11	YOR101W	RAS1
YML063W	RPS1B	YHR084W	STE12
YBR173C	UMP1	YPR036W	VMA13
YBL047C	EDE1	YJR035W	RAD26
YBL058W	SHP1	YDR261C	EXG2
YPR119W	CLB2	YNL197C	WHI3
YDR383C	NKP1	YOR141C	ARP8
YHR039C-A	VMA10	YCR077C	PAT1
YML115C	VAN1	YPL256C	CLN2
YGL033W	HOP2	YNL032W	SIW14
YLR288C	MEC3	YDL059C	RAD59
YIL156W	UBP7	YER115C	SPR6
YLR410W	VIP1	YDR183W	PLP1
YNL051W	COG5	YER122C	GLO3
YHR013C	ARD1	YPL018W	CTF19
YNL215W	IES2	YJL124C	LSM1
YGL060W	YBP2	YDR254W	CHL4
YFR031C-A	RPL2A	YNL297C	MON2
YMR284W	YKU70	YDR207C	UME6
YOR270C	VPH1	YBR019C	GAL10

Predictions at threshold level 0.75 continued...			
Gene1 of predicted pair		Gene2 of predicted pair	
Systematic Name	Standard Name	Systematic Name	Standard Name
YJL006C	CTK2	YPR046W	MCM16
YJL053W	PEP8	YLR396C	VPS33
YDR129C	SAC6	YBR200W	BEM1
YHR052W	CIC1	YLR435W	TSR2
YGR020C	VMA7	YGL223C	COG1
YIL148W	RPL40A	YMR273C	ZDS1
YGL163C	RAD54	YOR141C	ARP8
YHR084W	STE12	YHR030C	SLT2
YMR179W	SPT21	YPR046W	MCM16
YJL078C	PRY3	YDR105C	TMS1
YBL103C	RTG3	YDR137W	RGP1
YNL291C	MID1	YML115C	VAN1
YBR228W	SLX1	YLR410W	VIP1
YKR061W	KTR2	YPR046W	MCM16
YMR055C	BUB2	YBR114W	RAD16
YCR002C	CDC10	YJL187C	SWE1
YNL291C	MID1	YDR108W	GSG1
YMR127C	SAS2	YKL160W	ELF1
YPR052C	NHP6A	YDR225W	HTA1
YDR176W	NGG1	YBR010W	HHT1
YPL105C	YPL105C	YKL074C	MUD2
YGL100W	SEH1	YNL250W	RAD50
YGL025C	PGD1	YBR010W	HHT1
YBR108W	YBR108W	YLR144C	ACF2
YER115C	SPR6	YOR358W	HAP5
YKL007W	CAP1	YGL212W	VAM7
YMR199W	CLN1	YMR125W	STO1
YBR030W	YBR030W	YOR043W	WHI2
YMR153W	NUP53	YBR200W	BEM1

Predictions at threshold level 0.75 continued...			
Gene1 of predicted pair		Gene2 of predicted pair	
Systematic Name	Standard Name	Systematic Name	Standard Name
YPL018W	CTF19	YLR233C	EST1
YOR312C	RPL20B	YOR035C	SHE4
YMR142C	RPL13B	YJR056C	YJR056C
YDR477W	SNF1	YGL035C	MIG1
YOR106W	VAM3	YDR080W	VPS41
YKL129C	MYO3	YHR016C	YSC84
YGL115W	SNF4	YOR141C	ARP8
YGR261C	APL6	YML071C	COG8
YKL204W	EAP1	YJL095W	BCK1
YBR217W	ATG12	YNL058C	YNL058C
YPR101W	SNT309	YOR308C	SNU66
YKR048C	NAP1	YGL252C	RTG2
YEL053C	MAK10	YMR214W	SCJ1
YJL176C	SWI3	YDL155W	CLB3
YDR004W	RAD57	YDR225W	HTA1
YNL087W	TCB2	YPR030W	CSR2
YHR200W	RPN10	YBR019C	GAL10
YBR030W	YBR030W	YER139C	YER139C
YDL184C	RPL41A	YOL081W	IRA2
YNL106C	INP52	YNR052C	POP2
YMR224C	MRE11	YBR274W	CHK1
YCL010C	SGF29	YOL148C	SPT20
YOR270C	VPH1	YER040W	GLN3
YLR320W	MMS22	YCR031C	RPS14A
YPR084W	YPR084W	YGR261C	APL6
YPL024W	RMI1	YBR280C	SAF1
YBR194W	SOY1	YDR254W	CHL4
YAL024C	LTE1	YBR019C	GAL10
YOL030W	GAS5	YOL090W	MSH2

Predictions at threshold level 0.75 continued...			
Gene1 of predicted pair		Gene2 of predicted pair	
Systematic Name	Standard Name	Systematic Name	Standard Name
YLR350W	ORM2	YOL081W	IRA2
YGL033W	HOP2	YBR280C	SAF1
YMR127C	SAS2	YDR318W	MCM21
YIL076W	SEC28	YJL124C	LSM1
YER120W	SCS2	YDL077C	VAM6
YIL076W	SEC28	YHR081W	LRP1
YBL103C	RTG3	YKR035W-A	DID2
YPL253C	VIK1	YJR135C	MCM22
YGR072W	UPF3	YDR310C	SUM1
YDR156W	RPA14	YLR182W	SWI6
YOR195W	SLK19	YGL252C	RTG2
YDR318W	MCM21	YPL018W	CTF19
YGR270W	YTA7	YER139C	YER139C
YNL218W	MGS1	YPL024W	RMI1
YDR440W	DOT1	YMR038C	CCS1
YGL078C	DBP3	YER044C	ERG28
YPL246C	RBD2	YKR024C	DBP7
YJL127C	SPT10	YML016C	PPZ1
YLR288C	MEC3	YMR055C	BUB2
YNL238W	KEX2	YFR036W	CDC26
YOR327C	SNC2	YLR180W	SAM1
YGL237C	HAP2	YGR270W	YTA7
YOR001W	RRP6	YEL013W	VAC8
YIL159W	BNR1	YMR142C	RPL13B
YLR337C	VRP1	YOR312C	RPL20B
YBL103C	RTG3	YKL126W	YPK1
YPL152W	RRD2	YCR086W	CSM1
YKR072C	SIS2	YMR284W	YKU70
YKL110C	KTI12	YJR060W	CBF1

Predictions at threshold level 0.75 continued...			
Gene1 of predicted pair		Gene2 of predicted pair	
Systematic Name	Standard Name	Systematic Name	Standard Name
YMR004W	MVP1	YOL108C	INO4
YAL020C	ATS1	YNL289W	PCL1
YDR335W	MSN5	YPR120C	CLB5
YPL161C	BEM4	YLR094C	GIS3
YMR284W	YKU70	YCR086W	CSM1
YOR195W	SLK19	YHR152W	SPO12
YDR156W	RPA14	YNL248C	RPA49
YDR507C	GIN4	YIL008W	URM1
YFL023W	BUD27	YNL058C	YNL058C
YDR174W	HMO1	YHR134W	WSS1
YDL192W	ARF1	YEL060C	PRB1
YJL154C	VPS35	YBR171W	SEC66
YMR095C	SNO1	YJL172W	CPS1
YJR140C	HIR3	YAL002W	VPS8
YPL057C	SUR1	YOR327C	SNC2
YLR192C	HCR1	YER122C	GLO3
YPL042C	SSN3	YER139C	YER139C
YLR234W	TOP3	YOR368W	RAD17
YHR119W	SET1	YPL138C	SPP1
YGR072W	UPF3	YNL127W	FAR11
YNL058C	YNL058C	YKL048C	ELM1
YLR386W	VAC14	YIL008W	URM1
YGL087C	MMS2	YDR293C	SSD1
YCL037C	SRO9	YNL199C	GCR2
YML115C	VAN1	YOL111C	MDY2
YMR004W	MVP1	YNL041C	COG6
YJR056C	YJR056C	YLR093C	NYV1
YGR261C	APL6	YCL037C	SRO9
YLR095C	IOC2	YMR095C	SNO1

Predictions at threshold level 0.75 continued...			
Gene1 of predicted pair		Gene2 of predicted pair	
Systematic Name	Standard Name	Systematic Name	Standard Name
YER044C	ERG28	YDL083C	RPS16B
YBR215W	HPC2	YLR352W	YLR352W
YKR020W	VPS51	YGL095C	VPS45
YLR234W	TOP3	YDL155W	CLB3
YDL127W	PCL2	YPL001W	HAT1
YLR313C	SPH1	YNL016W	PUB1
YJL089W	SIP4	YGL115W	SNF4
YBL047C	EDE1	YKL041W	VPS24
YKL139W	CTK1	YJR063W	RPA12
YPR131C	NAT3	YLR058C	SHM2
YDR254W	CHL4	YML028W	TSA1
YGL087C	MMS2	YBR108W	YBR108W
YDL020C	RPN4	YHL002W	HSE1
YLR399C	BDF1	YLR254C	NDL1
YBR173C	UMP1	YDL083C	RPS16B
YNL166C	BNI5	YJR032W	CPR7
YPL089C	RLM1	YJL095W	BCK1
YDR477W	SNF1	YNL307C	MCK1
YEL043W	YEL043W	YNL127W	FAR11
YGL087C	MMS2	YDL059C	RAD59
YMR016C	SOK2	YBL025W	RRN10
YDR358W	GGA1	YOR036W	PEP12
YNL032W	SIW14	YNR072W	HXT17
YHR114W	BZZ1	YAL002W	VPS8
YIL159W	BNR1	YER116C	SLX8
YML001W	YPT7	YLR398C	SKI2
YNL238W	KEX2	YBR019C	GAL10
YDL006W	PTC1	YKL041W	VPS24
YDR080W	VPS41	YMR216C	SKY1

Predictions at threshold level 0.75 continued...			
**Gene1 of predicted pair**		**Gene2 of predicted pair**	
**Systematic Name**	**Standard Name**	**Systematic Name**	**Standard Name**
YGR270W	YTA7	YNL116W	DMA2
YGL223C	COG1	YKR014C	YPT52
YEL065W	SIT1	YNL032W	SIW14
YBR112C	CYC8	YDL005C	MED2
YLR206W	ENT2	YGR152C	RSR1
YOR195W	SLK19	YHR200W	RPN10
YLR386W	VAC14	YNL206C	RTT106
YML071C	COG8	YER122C	GLO3
YBL027W	RPL19B	YCR077C	PAT1
YLR320W	MMS22	YLR180W	SAM1
YOR083W	WHI5	YMR202W	ERG2
YML016C	PPZ1	YBL021C	HAP3
YEL053C	MAK10	YNL076W	MKS1
YGL115W	SNF4	YOR028C	CIN5
YNR072W	HXT17	YER040W	GLN3
YPL057C	SUR1	YKR001C	VPS1
YNL238W	KEX2	YKL041W	VPS24
YER139C	YER139C	YEL013W	VAC8
YPR036W	VMA13	YGR020C	VMA7
YER162C	RAD4	YOR368W	RAD17
YKL073W	LHS1	YBR138C	YBR138C
YLR191W	PEX13	YLR181C	VTA1
YLR095C	IOC2	YIL159W	BNR1
YGL033W	HOP2	YDL077C	VAM6
YDL013W	SLX5	YMR038C	CCS1
YNL218W	MGS1	YMR224C	MRE11
YCR086W	CSM1	YHL002W	HSE1
YNL087W	TCB2	YPL195W	APL5
YKR001C	VPS1	YMR022W	UBC7

Predictions at threshold level 0.75 continued...			
Gene1 of predicted pair		Gene2 of predicted pair	
Systematic Name	Standard Name	Systematic Name	Standard Name
YMR179W	SPT21	YDR318W	MCM21
YDR378C	LSM6	YNL145W	MFA2
YDR477W	SNF1	YPL137C	GIP3
YOR001W	RRP6	YOL091W	SPO21
YHR111W	UBA4	YNL229C	URE2
YLR292C	SEC72	YDR289C	RTT103
YER052C	HOM3	YNL023C	FAP1
YAL024C	LTE1	YLR315W	NKP2
YKL116C	PRR1	YEL053C	MAK10
YJR060W	CBF1	YLR370C	ARC18
YOR191W	RIS1	YPL079W	RPL21B
YPL115C	BEM3	YHL002W	HSE1
YKL139W	CTK1	YPL037C	EGD1
YPR017C	DSS4	YJL154C	VPS35
YOR290C	SNF2	YPR072W	NOT5
YNR052C	POP2	YKR061W	KTR2
YMR054W	STV1	YOL018C	TLG2
YFR010W	UBP6	YPL057C	SUR1
YPL253C	VIK1	YGL163C	RAD54
YKL139W	CTK1	YDL047W	SIT4
YDR382W	RPP2B	YJL124C	LSM1
YJR060W	CBF1	YNL127W	FAR11
YLR095C	IOC2	YOR327C	SNC2
YML097C	VPS9	YKL041W	VPS24
YMR153W	NUP53	YER110C	KAP123
YHR156C	LIN1	YNL238W	KEX2
YPL178W	CBC2	YDL047W	SIT4
YNL243W	SLA2	YPL086C	ELP3
YDR174W	HMO1	YNL215W	IES2

Predictions at threshold level 0.75 continued...			
Gene1 of predicted pair		Gene2 of predicted pair	
Systematic Name	Standard Name	Systematic Name	Standard Name
YDR227W	SIR4	YMR106C	YKU80
YGL133W	ITC1	YNL068C	FKH2
YGR159C	NSR1	YIL079C	AIR1
YOR023C	AHC1	YGR188C	BUB1
YDR443C	SSN2	YFR010W	UBP6
YBL008W	HIR1	YGR188C	BUB1
YCR020W-B	HTL1	YNL041C	COG6
YOR106W	VAM3	YCL001W	RER1
YBR200W	BEM1	YGR152C	RSR1
YLR148W	PEP3	YDR484W	VPS52
YHL031C	GOS1	YOR124C	UBP2
YLR018C	POM34	YDR385W	EFT2
YHR084W	STE12	YHL007C	STE20
YNL243W	SLA2	YHR016C	YSC84
YBR108W	YBR108W	YLR094C	GIS3
YGL060W	YBP2	YBR215W	HPC2
YLR192C	HCR1	YBL103C	RTG3
YKR001C	VPS1	YDL155W	CLB3
YOR304W	ISW2	YDR207C	UME6
YNL076W	MKS1	YOL081W	IRA2
YBL103C	RTG3	YOL111C	MDY2
YER116C	SLX8	YML102W	CAC2
YMR172W	HOT1	YBR171W	SEC66
YDR310C	SUM1	YER040W	GLN3
YKL068W	NUP100	YDR207C	UME6
YNL291C	MID1	YPL120W	VPS30
YMR224C	MRE11	YDR207C	UME6
YIL053W	RHR2	YDL083C	RPS16B
YPL046C	ELC1	YMR201C	RAD14

Predictions at threshold level 0.75 continued...			
Gene1 of predicted pair		Gene2 of predicted pair	
Systematic Name	Standard Name	Systematic Name	Standard Name
YLR148W	PEP3	YCR088W	ABP1
YPL253C	VIK1	YLR315W	NKP2
YKL025C	PAN3	YBL088C	TEL1
YDR378C	LSM6	YGL163C	RAD54
YER144C	UBP5	YEL053C	MAK10
YDL077C	VAM6	YNL147W	LSM7
YDL192W	ARF1	YAL030W	SNC1
YBL102W	SFT2	YEL053C	MAK10
YGL033W	HOP2	YGL124C	MON1
YBR107C	IML3	YKL110C	KTI12
YMR198W	CIK1	YPL253C	VIK1
YBR030W	YBR030W	YHR081W	LRP1
YOR308C	SNU66	YLR182W	SWI6
YDR156W	RPA14	YJL006C	CTK2
YCL037C	SRO9	YJR033C	RAV1
YDL083C	RPS16B	YLR398C	SKI2
YDR484W	VPS52	YEL053C	MAK10
YFR010W	UBP6	YHR200W	RPN10
YDL239C	ADY3	YOL091W	SPO21
YOL091W	SPO21	YOR177C	MPC54
YMR092C	AIP1	YGR080W	TWF1
YHR160C	PEX18	YDR142C	PEX7
YIL036W	CST6	YFR036W	CDC26
YOR195W	SLK19	YJL095W	BCK1
YGR261C	APL6	YHL031C	GOS1
YOR080W	DIA2	YOR038C	HIR2
YHR060W	VMA22	YPL195W	APL5
YER116C	SLX8	YLR352W	YLR352W
YOR140W	SFL1	YMR311C	GLC8

Predictions at threshold level 0.75 continued...			
Gene1 of predicted pair		Gene2 of predicted pair	
Systematic Name	Standard Name	Systematic Name	Standard Name
YBR171W	SEC66	YNL238W	KEX2
YBL008W	HIR1	YEL050C	RML2
YHL020C	OPI1	YPL195W	APL5
YPR036W	VMA13	YGR188C	BUB1
YCR034W	FEN1	YOR039W	CKB2
YIL053W	RHR2	YBR030W	YBR030W
YCR065W	HCM1	YPL106C	SSE1
YGL133W	ITC1	YKL101W	HSL1
YML001W	YPT7	YJL183W	MNN11
YBR171W	SEC66	YMR055C	BUB2
YMR055C	BUB2	YPR120C	CLB5
YBR228W	SLX1	YJR140C	HIR3
YNL069C	RPL16B	YJR060W	CBF1
YHR157W	REC104	YPR119W	CLB2
YOR033C	EXO1	YOL030W	GAS5
YGR104C	SRB5	YKL160W	ELF1
YMR172W	HOT1	YKL048C	ELM1
YOR290C	SNF2	YNL238W	KEX2
YGL003C	CDH1	YER139C	YER139C
YCR086W	CSM1	YDR310C	SUM1
YNL201C	PSY2	YJR063W	RPA12
YNL199C	GCR2	YMR311C	GLC8
YKR099W	BAS1	YOL121C	RPS19A
YOR127W	RGA1	YBL087C	RPL23A
YDR448W	ADA2	YPL129W	TAF14
YGL158W	RCK1	YDR423C	CAD1
YOR337W	TEA1	YMR214W	SCJ1
YDR283C	GCN2	YFR009W	GCN20
YJL204C	RCY1	YHR012W	VPS29

Predictions at threshold level 0.75 continued...			
Gene1 of predicted pair		Gene2 of predicted pair	
Systematic Name	Standard Name	Systematic Name	Standard Name
YJL060W	BNA3	YPR131C	NAT3
YHR152W	SPO12	YHR115C	DMA1
YGR019W	UGA1	YML016C	PPZ1
YMR199W	CLN1	YDR422C	SIP1
YOR337W	TEA1	YGL115W	SNF4
YAL024C	LTE1	YDR245W	MNN10
YKL213C	DOA1	YDR440W	DOT1
YNL094W	APP1	YBR239C	YBR239C
YHR121W	LSM12	YPR119W	CLB2
YML001W	YPT7	YFR036W	CDC26
YPL145C	KES1	YKL188C	PXA2
YPR030W	CSR2	YOL081W	IRA2
YHR178W	STB5	YHR111W	UBA4
YGL200C	EMP24	YML067C	ERV41
YBL005W	PDR3	YPR119W	CLB2
YDR490C	PKH1	YGR086C	PIL1
YGL147C	RPL9A	YOR337W	TEA1
YPL164C	MLH3	YMR167W	MLH1
YOR033C	EXO1	YER116C	SLX8
YER144C	UBP5	YHR039C-A	VMA10
YGR270W	YTA7	YLR315W	NKP2
YKL110C	KTI12	YJR135C	MCM22
YPL022W	RAD1	YLR182W	SWI6
YOR140W	SFL1	YIL033C	BCY1
YMR198W	CIK1	YJR005W	APL1
YDR477W	SNF1	YEL036C	ANP1
YOR066W	MSA1	YEL060C	PRB1
YPR018W	RLF2	YDL020C	RPN4
YKL139W	CTK1	YFR036W	CDC26

Predictions at threshold level 0.75 continued...			
Gene1 of predicted pair		Gene2 of predicted pair	
Systematic Name	Standard Name	Systematic Name	Standard Name
YCL037C	SRO9	YLR350W	ORM2
YBR194W	SOY1	YNL236W	SIN4
YIL103W	DPH1	YNL107W	YAF9
YEL009C	GCN4	YJL176C	SWI3
YGL054C	ERV14	YER162C	RAD4
YFR010W	UBP6	YOR270C	VPH1
YNL218W	MGS1	YJL187C	SWE1
YBL058W	SHP1	YHR060W	VMA22
YLR337C	VRP1	YJR084W	CSN12
YKL032C	IXR1	YBR260C	RGD1
YBL008W	HIR1	YCR031C	RPS14A
YMR311C	GLC8	YDR207C	UME6
YIL076W	SEC28	YMR016C	SOK2
YDR477W	SNF1	YMR230W	RPS10B
YLR090W	XDJ1	YIL053W	RHR2
YPL024W	RMI1	YNL291C	MID1
YDR363W-A	SEM1	YDL006W	PTC1
YFR036W	CDC26	YER040W	GLN3
YDR183W	PLP1	YGL252C	RTG2
YBR217W	ATG12	YOR043W	WHI2
YML115C	VAN1	YHR081W	LRP1
YNL215W	IES2	YMR198W	CIK1
YIL148W	RPL40A	YPL246C	RBD2
YGR184C	UBR1	YOR014W	RTS1
YMR127C	SAS2	YKL048C	ELM1
YOR212W	STE4	YDR264C	AKR1
YKL048C	ELM1	YGL151W	NUT1
YDR004W	RAD57	YJR135C	MCM22
YJR056C	YJR056C	YKL048C	ELM1

Predictions at threshold level 0.75 continued...			
Gene1 of predicted pair		Gene2 of predicted pair	
Systematic Name	Standard Name	Systematic Name	Standard Name
YMR095C	SNO1	YDL192W	ARF1
YKR035W-A	DID2	YLR093C	NYV1
YDL013W	SLX5	YLR410W	VIP1
YGL043W	DST1	YBR111W-A	SUS1
YNL087W	TCB2	YLR370C	ARC18
YJR056C	YJR056C	YNL076W	MKS1
YGR133W	PEX4	YAL002W	VPS8
YIL159W	BNR1	YBR108W	YBR108W
YLR429W	CRN1	YPR046W	MCM16
YNL201C	PSY2	YML112W	CTK3
YBR084C-A	RPL19A	YGL031C	RPL24A
YGL078C	DBP3	YOL121C	RPS19A
YMR153W	NUP53	YGL212W	VAM7
YER044C	ERG28	YER040W	GLN3
YCL001W	RER1	YML001W	YPT7
YLR441C	RPS1A	YOL111C	MDY2
YLR441C	RPS1A	YDL192W	ARF1
YOR127W	RGA1	YKR020W	VPS51
YBL067C	UBP13	YJL095W	BCK1
YDR335W	MSN5	YFR036W	CDC26
YDR383C	NKP1	YIR005W	IST3
YLR441C	RPS1A	YPL037C	EGD1
YHR200W	RPN10	YMR152W	YIM1
YPR102C	RPL11A	YOR078W	BUD21
YKR020W	VPS51	YNR032W	PPG1
YHR012W	VPS29	YLR233C	EST1
YPL057C	SUR1	YDL013W	SLX5
YBR084C-A	RPL19A	YKL032C	IXR1
YMR202W	ERG2	YKL048C	ELM1

Predictions at threshold level 0.75 continued...			
Gene1 of predicted pair		Gene2 of predicted pair	
Systematic Name	Standard Name	Systematic Name	Standard Name
YLR360W	VPS38	YLR381W	CTF3
YGR217W	CCH1	YLR320W	MMS22
YHR149C	SKG6	YMR273C	ZDS1
YGR041W	BUD9	YOR301W	RAX1
YLR119W	SRN2	YPL161C	BEM4
YHR039C-A	VMA10	YCR086W	CSM1
YGR142W	BTN2	YBR264C	YPT10
YIR005W	IST3	YOR141C	ARP8
YMR133W	REC114	YCR053W	THR4
YDL223C	HBT1	YGL115W	SNF4
YOR270C	VPH1	YJL095W	BCK1
YER115C	SPR6	YKL068W	NUP100
YKR001C	VPS1	YEL051W	VMA8
YLR313C	SPH1	YOL090W	MSH2
YGL174W	BUD13	YOR308C	SNU66
YMR116C	ASC1	YJL187C	SWE1
YBR098W	MMS4	YGL100W	SEH1
YBR217W	ATG12	YPL057C	SUR1
YBL078C	ATG8	YOL018C	TLG2
YEL051W	VMA8	YNL051W	COG5
YMR142C	RPL13B	YNL032W	SIW14
YOR033C	EXO1	YGL031C	RPL24A
YHR114W	BZZ1	YDR310C	SUM1
YOR035C	SHE4	YDR323C	PEP7
YJR063W	RPA12	YJL140W	RPB4
YEL043W	YEL043W	YOL018C	TLG2
YNL058C	YNL058C	YOR327C	SNC2
YKL139W	CTK1	YKL160W	ELF1
YMR167W	MLH1	YCR031C	RPS14A

Predictions at threshold level 0.75 continued...			
Gene1 of predicted pair		Gene2 of predicted pair	
Systematic Name	Standard Name	Systematic Name	Standard Name
YPL195W	APL5	YLR398C	SKI2
YFL023W	BUD27	YDR261C	EXG2
YNL166C	BNI5	YHR114W	BZZ1
YHR073W	OSH3	YLR018C	POM34
YKL139W	CTK1	YPR030W	CSR2
YPL249C-A	RPL36B	YPR119W	CLB2
YIL053W	RHR2	YLR093C	NYV1
YGL033W	HOP2	YKL126W	YPK1
YLR360W	VPS38	YER110C	KAP123
YER162C	RAD4	YFR036W	CDC26
YDL116W	NUP84	YPL256C	CLN2
YDR174W	HMO1	YNL106C	INP52
YGR072W	UPF3	YHL020C	OPI1
YDR254W	CHL4	YFR030W	MET10
YOL111C	MDY2	YHR081W	LRP1
YDR293C	SSD1	YLR233C	EST1
YOR023C	AHC1	YBR107C	IML3
YDR507C	GIN4	YBR084C-A	RPL19A
YBR217W	ATG12	YDR261C	EXG2
YFR031C-A	RPL2A	YGL212W	VAM7
YCL001W	RER1	YFL033C	RIM15
YNL291C	MID1	YKR020W	VPS51
YBR009C	HHF1	YNL030W	HHF2
YKR099W	BAS1	YJL183W	MNN11
YNL139C	RLR1	YOR001W	RRP6
YIL053W	RHR2	YFL023W	BUD27
YDL020C	RPN4	YLR234W	TOP3
YJL053W	PEP8	YCL001W	RER1
YKR099W	BAS1	YLR371W	ROM2

Predictions at threshold level 0.75 continued...			
Gene1 of predicted pair		Gene2 of predicted pair	
Systematic Name	Standard Name	Systematic Name	Standard Name
YNL106C	INP52	YNL116W	DMA2
YNL297C	MON2	YPL105C	YPL105C
YGR261C	APL6	YKR030W	GMH1
YJL029C	VPS53	YDR323C	PEP7
YMR133W	REC114	YLR371W	ROM2
YEL060C	PRB1	YER122C	GLO3
YHL031C	GOS1	YNL297C	MON2
YPL253C	VIK1	YIL103W	DPH1
YNL106C	INP52	YJL006C	CTK2
YER122C	GLO3	YGR188C	BUB1
YLR320W	MMS22	YMR311C	GLC8
YDR293C	SSD1	YML016C	PPZ1
YPR120C	CLB5	YDR289C	RTT103
YNR072W	HXT17	YJR032W	CPR7
YMR095C	SNO1	YDR310C	SUM1
YPR029C	APL4	YJR058C	APS2
YFR010W	UBP6	YHR134W	WSS1
YBL078C	ATG8	YOR106W	VAM3
YOR275C	RIM20	YPR120C	CLB5
YOR195W	SLK19	YIL008W	URM1
YDR202C	RAV2	YHL020C	OPI1
YJL151C	SNA3	YOR284W	HUA2
YLR313C	SPH1	YBL079W	NUP170
YMR216C	SKY1	YPL105C	YPL105C
YPL115C	BEM3	YDR227W	SIR4
YBR108W	YBR108W	YBL051C	PIN4
YNR052C	POP2	YNL225C	CNM67
YEL053C	MAK10	YCR053W	THR4
YGL124C	MON1	YOL067C	RTG1

Predictions at threshold level 0.75 continued...			
Gene1 of predicted pair		Gene2 of predicted pair	
Systematic Name	Standard Name	Systematic Name	Standard Name
YDL101C	DUN1	YLR135W	SLX4
YDR443C	SSN2	YMR152W	YIM1
YNL291C	MID1	YMR055C	BUB2
YNL288W	CAF40	YHR178W	STB5
YOR033C	EXO1	YDR097C	MSH6
YGL147C	RPL9A	YBL058W	SHP1
YMR172W	HOT1	YMR055C	BUB2
YBR057C	MUM2	YDR254W	CHL4
YDR439W	LRS4	YJR135C	MCM22
YJL154C	VPS35	YOR036W	PEP12
YER068W	MOT2	YNL030W	HHF2
YJR099W	YUH1	YHR108W	GGA2
YNL041C	COG6	YGL003C	CDH1
YGL115W	SNF4	YML071C	COG8
YNL058C	YNL058C	YLR320W	MMS22
YDR293C	SSD1	YER122C	GLO3
YGL147C	RPL9A	YPR036W	VMA13
YLR386W	VAC14	YMR109W	MYO5
YKL126W	YPK1	YLR094C	GIS3
YHR060W	VMA22	YKL119C	VPH2
YER161C	SPT2	YDR073W	SNF11
YKL048C	ELM1	YOL121C	RPS19A
YNL291C	MID1	YGR188C	BUB1
YLR320W	MMS22	YOR066W	MSA1
YFR010W	UBP6	YPL137C	GIP3
YNL215W	IES2	YIL076W	SEC28
YLR079W	SIC1	YLR399C	BDF1
YDR385W	EFT2	YPR119W	CLB2
YPL253C	VIK1	YPR051W	MAK3

Predictions at threshold level 0.75 continued...			
Gene1 of predicted pair		Gene2 of predicted pair	
Systematic Name	Standard Name	Systematic Name	Standard Name
YJL095W	BCK1	YGL124C	MON1
YIL159W	BNR1	YBR036C	CSG2
YIL076W	SEC28	YDR378C	LSM6
YOR014W	RTS1	YLR032W	RAD5
YNR052C	POP2	YLR399C	BDF1
YPR040W	TIP41	YGL190C	CDC55
YGR014W	MSB2	YML097C	VPS9
YLR399C	BDF1	YNL307C	MCK1
YHR039C-A	VMA10	YPR046W	MCM16
YLR093C	NYV1	YGL003C	CDH1
YAL020C	ATS1	YHR111W	UBA4
YML095C	RAD10	YBL019W	APN2
YLR192C	HCR1	YHR013C	ARD1
YNL325C	FIG4	YGL115W	SNF4
YOR132W	VPS17	YCL001W	RER1
YLR360W	VPS38	YMR004W	MVP1
YLR216C	CPR6	YIL133C	RPL16A
YDL184C	RPL41A	YMR152W	YIM1
YMR216C	SKY1	YML001W	YPT7
YMR224C	MRE11	YDR484W	VPS52
YDR358W	GGA1	YML097C	VPS9
YDL184C	RPL41A	YDR310C	SUM1
YDR378C	LSM6	YHR111W	UBA4
YGL212W	VAM7	YHR200W	RPN10
YKL048C	ELM1	YKL101W	HSL1
YKR020W	VPS51	YLR309C	IMH1
YML067C	ERV41	YEL036C	ANP1
YLR371W	ROM2	YDR207C	UME6
YNL248C	RPA49	YPR046W	MCM16

Predictions at threshold level 0.75 continued...			
Gene1 of predicted pair		Gene2 of predicted pair	
Systematic Name	Standard Name	Systematic Name	Standard Name
YMR198W	CIK1	YBR200W	BEM1
YBR108W	YBR108W	YBR200W	BEM1
YAL024C	LTE1	YDR076W	RAD55
YLR006C	SSK1	YPL161C	BEM4
YLR144C	ACF2	YKR030W	GMH1
YJL138C	TIF2	YMR167W	MLH1
YOR112W	CEX1	YKR001C	VPS1
YIL053W	RHR2	YFR010W	UBP6
YMR198W	CIK1	YGR184C	UBR1
YML115C	VAN1	YDR385W	EFT2
YKL048C	ELM1	YNL116W	DMA2
YHR205W	SCH9	YLR200W	YKE2
YGL033W	HOP2	YOL121C	RPS19A
YLR360W	VPS38	YGL115W	SNF4
YGR104C	SRB5	YPR120C	CLB5
YMR142C	RPL13B	YBR108W	YBR108W
YBR215W	HPC2	YDR207C	UME6
YLR441C	RPS1A	YLR370C	ARC18
YHR111W	UBA4	YDR264C	AKR1
YMR109W	MYO5	YPR119W	CLB2
YNL248C	RPA49	YPL152W	RRD2
YFR036W	CDC26	YOR035C	SHE4
YBR188C	NTC20	YPR101W	SNT309
YKL139W	CTK1	YMR142C	RPL13B
YLR441C	RPS1A	YCR053W	THR4
YBR194W	SOY1	YPL022W	RAD1
YDR358W	GGA1	YBR036C	CSG2
YNL325C	FIG4	YER122C	GLO3
YLR386W	VAC14	YJR056C	YJR056C

Predictions at threshold level 0.75 continued...			
Gene1 of predicted pair		Gene2 of predicted pair	
Systematic Name	Standard Name	Systematic Name	Standard Name
YDR080W	VPS41	YDL226C	GCS1
YFL047W	RGD2	YML057W	CMP2
YJL124C	LSM1	YDL047W	SIT4
YML071C	COG8	YML097C	VPS9
YLR095C	IOC2	YNL032W	SIW14
YLR288C	MEC3	YKL114C	APN1
YNL218W	MGS1	YJR140C	HIR3
YLR435W	TSR2	YGL189C	RPS26A
YJL124C	LSM1	YPR046W	MCM16
YKR072C	SIS2	YMR116C	ASC1
YOR038C	HIR2	YOR141C	ARP8
YGR261C	APL6	YOR127W	RGA1
YDL006W	PTC1	YML071C	COG8
YJL154C	VPS35	YER114C	BOI2
YJR090C	GRR1	YLR352W	YLR352W
YLR362W	STE11	YPR119W	CLB2
YDR477W	SNF1	YMR214W	SCJ1
YLR360W	VPS38	YER122C	GLO3
YLR452C	SST2	YOL121C	RPS19A
YHR134W	WSS1	YOR127W	RGA1
YFR010W	UBP6	YDR440W	DOT1
YNL054W	VAC7	YJR063W	RPA12
YKL010C	UFD4	YNL079C	TPM1
YBL079W	NUP170	YPL246C	RBD2
YNL291C	MID1	YGR217W	CCH1
YLR170C	APS1	YDL006W	PTC1
YGR129W	SYF2	YIR009W	MSL1
YMR153W	NUP53	YOR066W	MSA1
YGR020C	VMA7	YHR060W	VMA22

Predictions at threshold level 0.75 continued...			
Gene1 of predicted pair		Gene2 of predicted pair	
Systematic Name	Standard Name	Systematic Name	Standard Name
YNL291C	MID1	YDR318W	MCM21
YDR179C	CSN9	YMR025W	CSI1
YFR010W	UBP6	YEL060C	PRB1
YMR142C	RPL13B	YDR261C	EXG2
YKL160W	ELF1	YLR350W	ORM2
YPL137C	GIP3	YMR152W	YIM1
YKL048C	ELM1	YDR028C	REG1
YDR225W	HTA1	YPL001W	HAT1
YGL100W	SEH1	YKL057C	NUP120
YLR452C	SST2	YLR370C	ARC18
YKL139W	CTK1	YBL025W	RRN10
YDR156W	RPA14	YPL161C	BEM4
YDL002C	NHP10	YGL148W	ARO2
YMR216C	SKY1	YJR033C	RAV1
YGR184C	UBR1	YML028W	TSA1
YDR004W	RAD57	YLR032W	RAD5
YMR026C	PEX12	YBL025W	RRN10
YLR148W	PEP3	YOR035C	SHE4
YKL048C	ELM1	YDR309C	GIC2
YEL053C	MAK10	YDR358W	GGA1
YGL033W	HOP2	YDR358W	GGA1
YGL223C	COG1	YOR036W	PEP12
YKR035W-A	DID2	YOL121C	RPS19A
YMR038C	CCS1	YLR182W	SWI6
YDR101C	ARX1	YBR031W	RPL4A
YBL058W	SHP1	YGR249W	MGA1
YDR216W	ADR1	YMR280C	CAT8
YDL002C	NHP10	YER122C	GLO3
YLR360W	VPS38	YOR023C	AHC1

Predictions at threshold level 0.75 continued...			
Gene1 of predicted pair		Gene2 of predicted pair	
Systematic Name	Standard Name	Systematic Name	Standard Name
YBR020W	GAL1	YML051W	GAL80
YDR146C	SWI5	YER049W	TPA1
YJL124C	LSM1	YPR101W	SNT309
YMR224C	MRE11	YLR018C	POM34
YMR224C	MRE11	YCR077C	PAT1
YDR260C	SWM1	YJL095W	BCK1
YGR200C	ELP2	YHR187W	IKI1
YNL087W	TCB2	YGL124C	MON1
YOR109W	INP53	YHL020C	OPI1
YPL037C	EGD1	YER162C	RAD4
YDL155W	CLB3	YJL183W	MNN11
YLR079W	SIC1	YGL163C	RAD54
YJR056C	YJR056C	YNR072W	HXT17
YGR261C	APL6	YOR106W	VAM3
YGR072W	UPF3	YKR030W	GMH1
YDR448W	ADA2	YBR089C-A	NHP6B
YIL159W	BNR1	YJL187C	SWE1
YJL004C	SYS1	YMR054W	STV1
YGL223C	COG1	YGL054C	ERV14
YPL256C	CLN2	YOR014W	RTS1
YOR358W	HAP5	YDR144C	MKC7
YMR142C	RPL13B	YPR036W	VMA13
YBR228W	SLX1	YLR313C	SPH1
YFR024C-A	LSB3	YMR192W	GYL1
YMR116C	ASC1	YPL096C-A	ERI1
YFR031C-A	RPL2A	YPR036W	VMA13
YDR309C	GIC2	YJL187C	SWE1
YLR093C	NYV1	YMR311C	GLC8
YDR477W	SNF1	YLR425W	TUS1

Predictions at threshold level 0.75 continued...			
Gene1 of predicted pair		Gene2 of predicted pair	
Systematic Name	Standard Name	Systematic Name	Standard Name
YPL253C	VIK1	YGL253W	HXK2
YBL058W	SHP1	YMR142C	RPL13B
YNL094W	APP1	YLR433C	CNA1
YDR144C	MKC7	YHR114W	BZZ1
YPR018W	RLF2	YGR184C	UBR1
YNL135C	FPR1	YGL124C	MON1
YCR086W	CSM1	YJL190C	RPS22A
YCR077C	PAT1	YDR289C	RTT103
YBR030W	YBR030W	YDL077C	VAM6
YDR174W	HMO1	YPR119W	CLB2
YGR261C	APL6	YNL107W	YAF9
YCR086W	CSM1	YGL252C	RTG2
YPR084W	YPR084W	YPL042C	SSN3
YNL107W	YAF9	YKL114C	APN1
YER162C	RAD4	YEL051W	VMA8
YMR153W	NUP53	YML057W	CMP2
YOR127W	RGA1	YKR057W	RPS21A
YIL008W	URM1	YDL006W	PTC1
YEL065W	SIT1	YLR119W	SRN2
YHR200W	RPN10	YPL161C	BEM4
YNR052C	POP2	YLR254C	NDL1
YFR031C-A	RPL2A	YML115C	VAN1
YER087C-B	SBH1	YHR200W	RPN10
YIL036W	CST6	YNL107W	YAF9
YHR200W	RPN10	YDR318W	MCM21
YFR031C-A	RPL2A	YJL095W	BCK1
YDR484W	VPS52	YPL057C	SUR1
YFL023W	BUD27	YLR180W	SAM1
YBR173C	UMP1	YHR056C	RSC30

Predictions at threshold level 0.75 continued...			
Gene1 of predicted pair		Gene2 of predicted pair	
Systematic Name	Standard Name	Systematic Name	Standard Name
YCL001W	RER1	YNL051W	COG5
YJL029C	VPS53	YOR327C	SNC2
YJL006C	CTK2	YLR370C	ARC18
YNL215W	IES2	YFR036W	CDC26
YML115C	VAN1	YMR152W	YIM1
YKR001C	VPS1	YDR358W	GGA1
YDL116W	NUP84	YPL022W	RAD1
YCR020C-A	MAK31	YGR072W	UPF3
YDR004W	RAD57	YOR014W	RTS1
YML115C	VAN1	YLR131C	ACE2
YLR079W	SIC1	YPR111W	DBF20
YOR358W	HAP5	YPL161C	BEM4
YPL137C	GIP3	YNL076W	MKS1
YJR040W	GEF1	YML071C	COG8
YLR386W	VAC14	YDR254W	CHL4
YDR146C	SWI5	YMR199W	CLN1
YDR379W	RGA2	YKL048C	ELM1
YKL048C	ELM1	YEL056W	HAT2
YOR127W	RGA1	YGR152C	RSR1
YBL008W	HIR1	YLR288C	MEC3
YCR066W	RAD18	YHR039C-A	VMA10
YLR135W	SLX4	YOL030W	GAS5
YBR200W	BEM1	YLR362W	STE11
YDR080W	VPS41	YCL037C	SRO9
YDL059C	RAD59	YLR032W	RAD5
YDR363W-A	SEM1	YMR038C	CCS1
YBR289W	SNF5	YER068W	MOT2
YNR052C	POP2	YDR254W	CHL4
YPR089W	YPR089W	YOR275C	RIM20

Predictions at threshold level 0.75 continued...			
Gene1 of predicted pair		Gene2 of predicted pair	
Systematic Name	Standard Name	Systematic Name	Standard Name
YJL183W	MNN11	YMR089C	YTA12
YCR053W	THR4	YOL081W	IRA2
YNL058C	YNL058C	YLR398C	SKI2
YMR032W	HOF1	YJR090C	GRR1
YKL025C	PAN3	YCR086W	CSM1
YGR249W	MGA1	YLR386W	VAC14
YNL238W	KEX2	YAR014C	BUD14
YML095C	RAD10	YBL047C	EDE1
YGL078C	DBP3	YDR261C	EXG2
YHR012W	VPS29	YGL253W	HXK2
YBL008W	HIR1	YKL109W	HAP4
YLR386W	VAC14	YLR182W	SWI6
YHL020C	OPI1	YOL108C	INO4
YIL076W	SEC28	YBR200W	BEM1
YNL069C	RPL16B	YGR261C	APL6
YMR116C	ASC1	YPL089C	RLM1
YMR016C	SOK2	YMR152W	YIM1
YDR443C	SSN2	YDL005C	MED2
YPL129W	TAF14	YOR141C	ARP8
YAR014C	BUD14	YAL002W	VPS8
YGL212W	VAM7	YHR039C-A	VMA10
YOR112W	CEX1	YJR033C	RAV1
YKL116C	PRR1	YBR200W	BEM1
YKL025C	PAN3	YDR144C	MKC7
YGL178W	MPT5	YBL016W	FUS3
YIL076W	SEC28	YMR142C	RPL13B
YAL024C	LTE1	YJL187C	SWE1
YLL039C	UBI4	YER001W	MNN1
YLR119W	SRN2	YHR200W	RPN10

Predictions at threshold level 0.75 continued...			
Gene1 of predicted pair		Gene2 of predicted pair	
Systematic Name	Standard Name	Systematic Name	Standard Name
YOL145C	CTR9	YPL248C	GAL4
YER162C	RAD4	YOL108C	INO4
YLR320W	MMS22	YDR207C	UME6
YDR176W	NGG1	YPL129W	TAF14
YPL152W	RRD2	YML112W	CTK3
YDR225W	HTA1	YOR039W	CKB2
YDR129C	SAC6	YLR370C	ARC18
YPL152W	RRD2	YLR315W	NKP2
YLR337C	VRP1	YMR198W	CIK1
YDR480W	DIG2	YPL256C	CLN2
YMR071C	TVP18	YAR014C	BUD14
YLR386W	VAC14	YMR127C	SAS2
YGR105W	VMA21	YNL051W	COG5
YDR310C	SUM1	YJR035W	RAD26
YLR386W	VAC14	YOL145C	CTR9
YDR004W	RAD57	YHR079C-A	SAE3
YML117W	NAB6	YMR205C	PFK2
YNL238W	KEX2	YPL137C	GIP3
YKL010C	UFD4	YKL032C	IXR1
YLR090W	XDJ1	YMR016C	SOK2
YML117W	NAB6	YJL117W	PHO86
YJL172W	CPS1	YER040W	GLN3
YMR153W	NUP53	YPR036W	VMA13
YKR020W	VPS51	YGR135W	PRE9
YCR034W	FEN1	YLR350W	ORM2
YOR185C	GSP2	YER110C	KAP123
YMR055C	BUB2	YNL107W	YAF9
YBR030W	YBR030W	YOL111C	MDY2
YBR217W	ATG12	YBL025W	RRN10

Predictions at threshold level 0.75 continued...			
Gene1 of predicted pair		Gene2 of predicted pair	
Systematic Name	Standard Name	Systematic Name	Standard Name
YPL024W	RMI1	YKR072C	SIS2
YFR031C-A	RPL2A	YHL020C	OPI1
YMR199W	CLN1	YHR012W	VPS29
YER144C	UBP5	YNL076W	MKS1
YHR156C	LIN1	YKL116C	PRR1
YDL106C	PHO2	YFR034C	PHO4
YBR127C	VMA2	YDL185W	TFP1
YCR002C	CDC10	YHR061C	GIC1
YHR156C	LIN1	YDR137W	RGP1
YPL024W	RMI1	YOR066W	MSA1
YNL116W	DMA2	YNL107W	YAF9
YOL121C	RPS19A	YDR310C	SUM1
YDL020C	RPN4	YJL183W	MNN11
YNL199C	GCR2	YJL095W	BCK1
YEL009C	GCN4	YOR038C	HIR2
YJL036W	SNX4	YGL223C	COG1
YKR099W	BAS1	YNL307C	MCK1
YMR198W	CIK1	YML028W	TSA1
YLR386W	VAC14	YBR114W	RAD16
YOR132W	VPS17	YJR135C	MCM22
YDR310C	SUM1	YNL107W	YAF9
YNL215W	IES2	YGL252C	RTG2
YHR119W	SET1	YLR330W	CHS5
YOR038C	HIR2	YBR036C	CSG2
YDR378C	LSM6	YAL002W	VPS8
YMR055C	BUB2	YLR410W	VIP1
YKL025C	PAN3	YDR310C	SUM1
YCR081W	SRB8	YPL248C	GAL4
YBL003C	HTA2	YNL030W	HHF2

Predictions at threshold level 0.75 continued...			
Gene1 of predicted pair		Gene2 of predicted pair	
Systematic Name	Standard Name	Systematic Name	Standard Name
YDR261C	EXG2	YPL137C	GIP3
YKR099W	BAS1	YER044C	ERG28
YHL031C	GOS1	YIR005W	IST3
YBR171W	SEC66	YML012W	ERV25
YPL161C	BEM4	YGR105W	VMA21
YNL238W	KEX2	YDR358W	GGA1
YIR002C	MPH1	YLR234W	TOP3
YNL201C	PSY2	YDR439W	LRS4
YJL151C	SNA3	YKR030W	GMH1
YGL212W	VAM7	YML071C	COG8
YPL240C	HSP82	YGL189C	RPS26A
YGR004W	PEX31	YMR204C	INP1
YLR429W	CRN1	YLR410W	VIP1
YJR063W	RPA12	YOR141C	ARP8
YPL050C	MNN9	YKR020W	VPS51
YOR008C	SLG1	YDR207C	UME6
YFL013C	IES1	YCR077C	PAT1
YJL036W	SNX4	YCL001W	RER1
YPL037C	EGD1	YLR309C	IMH1
YJL172W	CPS1	YER144C	UBP5
YDR378C	LSM6	YLR429W	CRN1
YBR108W	YBR108W	YOL121C	RPS19A
YGR261C	APL6	YGR020C	VMA7
YJR021C	REC107	YBR119W	MUD1
YLR119W	SRN2	YOR290C	SNF2
YLR453C	RIF2	YBR275C	RIF1
YLR386W	VAC14	YNL087W	TCB2
YOR066W	MSA1	YPR030W	CSR2
YLR240W	VPS34	YDR484W	VPS52

Predictions at threshold level 0.75 continued...			
Gene1 of predicted pair		Gene2 of predicted pair	
Systematic Name	Standard Name	Systematic Name	Standard Name
YFL013C	IES1	YOR080W	DIA2
YGL147C	RPL9A	YGL078C	DBP3
YLR083C	EMP70	YOL018C	TLG2
YPL024W	RMI1	YDR477W	SNF1
YFR031C-A	RPL2A	YJR084W	CSN12
YDR329C	PEX3	YPL112C	PEX25
YMR054W	STV1	YDR207C	UME6
YJR090C	GRR1	YGL253W	HXK2
YML115C	VAN1	YPL137C	GIP3
YHL020C	OPI1	YDR207C	UME6
YDR225W	HTA1	YDL013W	SLX5
YER122C	GLO3	YKL119C	VPH2
YLR313C	SPH1	YJL095W	BCK1
YLR435W	TSR2	YCR031C	RPS14A
YOR076C	SKI7	YHR134W	WSS1
YPL037C	EGD1	YJR033C	RAV1
YBR107C	IML3	YHR114W	BZZ1
YML115C	VAN1	YPL037C	EGD1
YPL022W	RAD1	YBR107C	IML3
YNL218W	MGS1	YCL037C	SRO9
YKL129C	MYO3	YCR088W	ABP1
YOL054W	PSH1	YAR014C	BUD14
YLR094C	GIS3	YDL192W	ARF1
YOR275C	RIM20	YKR020W	VPS51
YKR072C	SIS2	YML028W	TSA1
YBR200W	BEM1	YKR001C	VPS1
YNL288W	CAF40	YJL190C	RPS22A
YBR258C	SHG1	YDR440W	DOT1
YFL013C	IES1	YER161C	SPT2

Predictions at threshold level 0.75 continued...			
Gene1 of predicted pair		Gene2 of predicted pair	
Systematic Name	Standard Name	Systematic Name	Standard Name
YMR004W	MVP1	YCL037C	SRO9
YDR456W	NHX1	YBR260C	RGD1
YCR077C	PAT1	YBR036C	CSG2
YPR049C	ATG11	YPR185W	ATG13
YPR018W	RLF2	YNL291C	MID1
YLR090W	XDJ1	YGL033W	HOP2
YNL201C	PSY2	YDR156W	RPA14
YDR225W	HTA1	YDR254W	CHL4
YKL007W	CAP1	YPL161C	BEM4
YKR061W	KTR2	YKL048C	ELM1
YHR156C	LIN1	YHR200W	RPN10
YNL243W	SLA2	YJL115W	ASF1
YML115C	VAN1	YPL057C	SUR1
YHR079C	IRE1	YPL248C	GAL4
YML016C	PPZ1	YLR435W	TSR2
YPL161C	BEM4	YEL043W	YEL043W
YDL083C	RPS16B	YLR093C	NYV1
YLR094C	GIS3	YMR133W	REC114
YBL088C	TEL1	YKL048C	ELM1
YHR156C	LIN1	YLR371W	ROM2
YNR052C	POP2	YFR036W	CDC26
YEL050C	RML2	YMR179W	SPT21
YMR224C	MRE11	YJR063W	RPA12
YBR009C	HHF1	YPR119W	CLB2
YIL159W	BNR1	YCL027W	FUS1
YLR452C	SST2	YBL025W	RRN10
YPL096C-A	ERI1	YOR035C	SHE4
YNL243W	SLA2	YEL003W	GIM4
YKL160W	ELF1	YOR038C	HIR2

Predictions at threshold level 0.75 continued...			
Gene1 of predicted pair		Gene2 of predicted pair	
Systematic Name	Standard Name	Systematic Name	Standard Name
YLR320W	MMS22	YER040W	GLN3
YCR065W	HCM1	YOR195W	SLK19
YBL047C	EDE1	YNL116W	DMA2
YHR200W	RPN10	YEL053C	MAK10
YNL025C	SSN8	YDL005C	MED2
YNL106C	INP52	YIR009W	MSL1
YLR371W	ROM2	YDL192W	ARF1
YBL087C	RPL23A	YPR119W	CLB2
YGR184C	UBR1	YPR120C	CLB5
YLR292C	SEC72	YDR183W	PLP1
YGR217W	CCH1	YPL161C	BEM4
YLR381W	CTF3	YDR200C	VPS64
YLL039C	UBI4	YPL256C	CLN2
YDR137W	RGP1	YDR261C	EXG2
YDR335W	MSN5	YPL152W	RRD2
YNL248C	RPA49	YKL110C	KTI12
YHR111W	UBA4	YKL110C	KTI12
YOR140W	SFL1	YPL161C	BEM4
YKL006W	RPL14A	YML115C	VAN1
YPL024W	RMI1	YLR093C	NYV1
YLR315W	NKP2	YDR183W	PLP1
YML016C	PPZ1	YOR141C	ARP8
YOR332W	VMA4	YBR127C	VMA2
YNL107W	YAF9	YNL030W	HHF2
YPL042C	SSN3	YLR182W	SWI6
YLR371W	ROM2	YOL121C	RPS19A
YMR152W	YIM1	YOR035C	SHE4
YLR410W	VIP1	YDL155W	CLB3
YLR360W	VPS38	YJR060W	CBF1

Predictions at threshold level 0.75 continued...			
Gene1 of predicted pair		Gene2 of predicted pair	
Systematic Name	Standard Name	Systematic Name	Standard Name
YOL126C	MDH2	YNR065C	YNR065C
YGL054C	ERV14	YJL004C	SYS1
YGL016W	KAP122	YGR059W	SPR3
YNL206C	RTT106	YKL048C	ELM1
YPR017C	DSS4	YOR106W	VAM3
YPR029C	APL4	YOR109W	INP53
YKL007W	CAP1	YBR171W	SEC66
YLR386W	VAC14	YNL297C	MON2
YHL031C	GOS1	YDR108W	GSG1
YNL058C	YNL058C	YMR214W	SCJ1
YKL048C	ELM1	YGR135W	PRE9
YDL080C	THI3	YGL161C	YIP5
YKL116C	PRR1	YLR094C	GIS3
YDL101C	DUN1	YLR234W	TOP3
YML117W	NAB6	YBL101C	ECM21
YAR014C	BUD14	YNR032W	PPG1
YPL129W	TAF14	YFR034C	PHO4
YMR224C	MRE11	YKR061W	KTR2
YGR020C	VMA7	YKL080W	VMA5
YOR066W	MSA1	YER122C	GLO3
YJR056C	YJR056C	YLR370C	ARC18
YHR178W	STB5	YDR146C	SWI5
YER044C	ERG28	YHR039C-A	VMA10
YOR308C	SNU66	YHR167W	THP2
YNL293W	MSB3	YCR077C	PAT1
YLR381W	CTF3	YDR171W	HSP42
YJR056C	YJR056C	YFR036W	CDC26
YBL103C	RTG3	YLR180W	SAM1
YOR216C	RUD3	YEL022W	GEA2

Predictions at threshold level 0.75 continued...			
Gene1 of predicted pair		Gene2 of predicted pair	
Systematic Name	Standard Name	Systematic Name	Standard Name
YLR386W	VAC14	YOR275C	RIM20
YMR080C	NAM7	YOR191W	RIS1
YDL020C	RPN4	YBR200W	BEM1
YLR093C	NYV1	YLR370C	ARC18
YLR090W	XDJ1	YER044C	ERG28
YOR112W	CEX1	YJR040W	GEF1
YDR108W	GSG1	YDR318W	MCM21
YBL025W	RRN10	YMR311C	GLC8
YLR119W	SRN2	YMR214W	SCJ1
YIL053W	RHR2	YKL139W	CTK1
YDL192W	ARF1	YCR053W	THR4
YBR290W	BSD2	YLR079W	SIC1
YPR084W	YPR084W	YJL053W	PEP8
YJR005W	APL1	YNL107W	YAF9
YGL200C	EMP24	YDR440W	DOT1
YGR184C	UBR1	YHR200W	RPN10
YML115C	VAN1	YOR368W	RAD17
YPL024W	RMI1	YBR057C	MUM2
YKR035W-A	DID2	YPR173C	VPS4
YDR363W-A	SEM1	YDR310C	SUM1
YPL253C	VIK1	YGL151W	NUT1
YHL031C	GOS1	YNL107W	YAF9
YNL288W	CAF40	YJR090C	GRR1
YPL018W	CTF19	YOL081W	IRA2
YLR061W	RPL22A	YBR181C	RPS6B
YML115C	VAN1	YGR020C	VMA7
YDR378C	LSM6	YER139C	YER139C
YBR093C	PHO5	YNR052C	POP2
YGR072W	UPF3	YAL002W	VPS8

Predictions at threshold level 0.75 continued...			
Gene1 of predicted pair		Gene2 of predicted pair	
Systematic Name	Standard Name	Systematic Name	Standard Name
YGL115W	SNF4	YER040W	GLN3
YOR112W	CEX1	YCL037C	SRO9
YDR477W	SNF1	YDR245W	MNN10
YJR063W	RPA12	YHR115C	DMA1
YKR072C	SIS2	YPL256C	CLN2
YJL095W	BCK1	YNL076W	MKS1
YMR192W	GYL1	YHR016C	YSC84
YPR046W	MCM16	YBR019C	GAL10
YNL068C	FKH2	YJL095W	BCK1
YMR202W	ERG2	YDL047W	SIT4
YPR040W	TIP41	YNL289W	PCL1
YPR036W	VMA13	YDL223C	HBT1
YKL139W	CTK1	YPL057C	SUR1
YEL065W	SIT1	YJR056C	YJR056C
YLR119W	SRN2	YDR358W	GGA1
YKL010C	UFD4	YJL183W	MNN11
YNL069C	RPL16B	YMR004W	MVP1
YHR200W	RPN10	YPR119W	CLB2
YAL051W	OAF1	YDL127W	PCL2
YAL020C	ATS1	YPL161C	BEM4
YML038C	YMD8	YKR020W	VPS51
YGL066W	SGF73	YGR188C	BUB1
YGR270W	YTA7	YLR254C	NDL1
YNL087W	TCB2	YMR230W	RPS10B
YLR079W	SIC1	YOR001W	RRP6
YBR108W	YBR108W	YNL041C	COG6
YLR386W	VAC14	YNL166C	BNI5
YER116C	SLX8	YLR180W	SAM1
YMR198W	CIK1	YFR040W	SAP155

Predictions at threshold level 0.75 continued...			
Gene1 of predicted pair		Gene2 of predicted pair	
Systematic Name	Standard Name	Systematic Name	Standard Name
YML071C	COG8	YEL051W	VMA8
YLR386W	VAC14	YIL103W	DPH1
YMR224C	MRE11	YGR135W	PRE9
YGL087C	MMS2	YGL124C	MON1
YDR443C	SSN2	YPL137C	GIP3
YGL237C	HAP2	YNL297C	MON2
YHR200W	RPN10	YNL041C	COG6
YOR127W	RGA1	YHR061C	GIC1
YNL238W	KEX2	YKL080W	VMA5
YGL066W	SGF73	YOR368W	RAD17
YHL031C	GOS1	YNL044W	YIP3
YGR020C	VMA7	YJL004C	SYS1
YLR292C	SEC72	YIL008W	URM1
YPR036W	VMA13	YGL115W	SNF4
YBL078C	ATG8	YGL223C	COG1
YGL223C	COG1	YKR030W	GMH1
YGL033W	HOP2	YBR217W	ATG12
YNL325C	FIG4	YJL172W	CPS1
YLR288C	MEC3	YJL124C	LSM1
YIL036W	CST6	YLR315W	NKP2
YGL115W	SNF4	YEL051W	VMA8
YKL025C	PAN3	YMR055C	BUB2
YLR254C	NDL1	YGR188C	BUB1
YPL195W	APL5	YER139C	YER139C
YNL325C	FIG4	YLR094C	GIS3
YMR055C	BUB2	YNL116W	DMA2
YJL124C	LSM1	YLR182W	SWI6
YPR028W	YOP1	YBL106C	SRO77
YNL218W	MGS1	YGR071C	YGR071C

Predictions at threshold level 0.75 continued...			
Gene1 of predicted pair		Gene2 of predicted pair	
Systematic Name	Standard Name	Systematic Name	Standard Name
YMR199W	CLN1	YOR368W	RAD17
YIL076W	SEC28	YER044C	ERG28
YKR020W	VPS51	YJL187C	SWE1
YOR066W	MSA1	YGL003C	CDH1
YER044C	ERG28	YPR030W	CSR2
YDR261C	EXG2	YOL081W	IRA2
YDR448W	ADA2	YLR182W	SWI6
YNL288W	CAF40	YPR051W	MAK3
YML115C	VAN1	YLR452C	SST2
YGL135W	RPL1B	YDR385W	EFT2
YMR198W	CIK1	YOR001W	RRP6
YGL078C	DBP3	YLR320W	MMS22
YMR080C	NAM7	YDL020C	RPN4
YEL053C	MAK10	YLR093C	NYV1
YMR095C	SNO1	YOL111C	MDY2
YDL083C	RPS16B	YJL183W	MNN11
YMR224C	MRE11	YBR108W	YBR108W
YDL006W	PTC1	YNL079C	TPM1
YKL139W	CTK1	YPL195W	APL5
YEL065W	SIT1	YFL023W	BUD27
YHR134W	WSS1	YGR054W	YGR054W
YMR198W	CIK1	YPR120C	CLB5
YLR381W	CTF3	YLR370C	ARC18
YHR200W	RPN10	YJL095W	BCK1
YPR052C	NHP6A	YOR008C	SLG1
YLR447C	VMA6	YHR039C-A	VMA10
YEL044W	IES6	YFR036W	CDC26
YKL116C	PRR1	YML115C	VAN1
YPR017C	DSS4	YLR309C	IMH1

Predictions at threshold level 0.75 continued...			
Gene1 of predicted pair		Gene2 of predicted pair	
Systematic Name	Standard Name	Systematic Name	Standard Name
YFR010W	UBP6	YJR135C	MCM22
YKR061W	KTR2	YDR378C	LSM6
YNL201C	PSY2	YNL225C	CNM67
YPL161C	BEM4	YNL293W	MSB3
YLR090W	XDJ1	YJR056C	YJR056C
YML095C	RAD10	YDR171W	HSP42
YGL135W	RPL1B	YOR035C	SHE4
YNL032W	SIW14	YER144C	UBP5
YJL124C	LSM1	YAR014C	BUD14
YNL069C	RPL16B	YER120W	SCS2
YBR228W	SLX1	YDL013W	SLX5
YDL020C	RPN4	YGR071C	YGR071C
YPL024W	RMI1	YHR060W	VMA22
YOR270C	VPH1	YJL172W	CPS1
YMR016C	SOK2	YOR290C	SNF2
YDR443C	SSN2	YBL058W	SHP1
YDR477W	SNF1	YEL060C	PRB1
YMR026C	PEX12	YPL195W	APL5
YDL020C	RPN4	YNL288W	CAF40
YFL013C	IES1	YNL215W	IES2
YKL126W	YPK1	YJL095W	BCK1
YPL037C	EGD1	YKR020W	VPS51
YDR097C	MSH6	YNL307C	MCK1
YPL022W	RAD1	YDL047W	SIT4
YNR052C	POP2	YIR005W	IST3
YLR090W	XDJ1	YNL307C	MCK1
YBR108W	YBR108W	YOR327C	SNC2
YKR031C	SPO14	YFL031W	HAC1
YML016C	PPZ1	YDL226C	GCS1

Predictions at threshold level 0.75 continued...			
**Gene1 of predicted pair**		**Gene2 of predicted pair**	
**Systematic Name**	**Standard Name**	**Systematic Name**	**Standard Name**
YGL200C	EMP24	YJL004C	SYS1
YDR156W	RPA14	YBR082C	UBC4
YNL166C	BNI5	YPL161C	BEM4
YJR056C	YJR056C	YOL121C	RPS19A
YMR039C	SUB1	YOL081W	IRA2
YER044C	ERG28	YER122C	GLO3
YLR192C	HCR1	YPR036W	VMA13
YNL087W	TCB2	YPR036W	VMA13
YLR288C	MEC3	YGR188C	BUB1
YDR507C	GIN4	YHR115C	DMA1
YPL161C	BEM4	YHR115C	DMA1
YBR264C	YPT10	YGL198W	YIP4
YIR005W	IST3	YER139C	YER139C
YKL110C	KTI12	YJL006C	CTK2
YDR202C	RAV2	YPR051W	MAK3
YJL154C	VPS35	YOR008C	SLG1
YKL213C	DOA1	YLR350W	ORM2
YPL253C	VIK1	YDL226C	GCS1
YKR020W	VPS51	YDL077C	VAM6
YDR484W	VPS52	YFR036W	CDC26
YDL101C	DUN1	YBR082C	UBC4
YJL187C	SWE1	YJR035W	RAD26
YMR198W	CIK1	YGL200C	EMP24
YGR217W	CCH1	YOR275C	RIM20
YMR116C	ASC1	YLR371W	ROM2
YPR046W	MCM16	YBR280C	SAF1
YMR153W	NUP53	YFL047W	RGD2
YGR241C	YAP1802	YGL095C	VPS45
YBR173C	UMP1	YLR386W	VAC14

Predictions at threshold level 0.75 continued...			
Gene1 of predicted pair		Gene2 of predicted pair	
Systematic Name	Standard Name	Systematic Name	Standard Name
YMR152W	YIM1	YOL081W	IRA2
YHR178W	STB5	YLR233C	EST1
YPL267W	ACM1	YPL256C	CLN2
YGL147C	RPL9A	YLR094C	GIS3
YPR017C	DSS4	YAL002W	VPS8
YDR443C	SSN2	YBR036C	CSG2
YPL234C	TFP3	YOR035C	SHE4
YLL007C	YLL007C	YMR055C	BUB2
YBR173C	UMP1	YML071C	COG8
YBR173C	UMP1	YDR076W	RAD55
YOR127W	RGA1	YML016C	PPZ1
YKR001C	VPS1	YHR012W	VPS29
YBL072C	RPS8A	YLR425W	TUS1
YDL113C	ATG20	YPR119W	CLB2
YPR030W	CSR2	YJR084W	CSN12
YOR270C	VPH1	YDR484W	VPS52
YLR433C	CNA1	YPR101W	SNT309
YNL309W	STB1	YPL256C	CLN2
YIL076W	SEC28	YNL041C	COG6
YIL034C	CAP2	YGR108W	CLB1
YCR020W-B	HTL1	YLR357W	RSC2
YML115C	VAN1	YLR371W	ROM2
YNL201C	PSY2	YJL124C	LSM1
YJL176C	SWI3	YAL055W	PEX22
YBR127C	VMA2	YOR327C	SNC2
YPL050C	MNN9	YKR014C	YPT52
YCL037C	SRO9	YNL051W	COG5
YGR104C	SRB5	YNL199C	GCR2
YDR363W-A	SEM1	YER118C	SHO1

Predictions at threshold level 0.75 continued...			
Gene1 of predicted pair		Gene2 of predicted pair	
Systematic Name	Standard Name	Systematic Name	Standard Name
YLR234W	TOP3	YDR335W	MSN5
YOR127W	RGA1	YNL116W	DMA2
YNL087W	TCB2	YML001W	YPT7
YER118C	SHO1	YKL041W	VPS24
YLR288C	MEC3	YOL081W	IRA2
YKL139W	CTK1	YLR452C	SST2
YMR055C	BUB2	YAL015C	NTG1
YMR198W	CIK1	YBR215W	HPC2
YBL008W	HIR1	YDR448W	ADA2
YNL307C	MCK1	YKL101W	HSL1
YER144C	UBP5	YNR072W	HXT17
YEL065W	SIT1	YPR036W	VMA13
YDL116W	NUP84	YPL018W	CTF19
YKL213C	DOA1	YNR006W	VPS27
YLR192C	HCR1	YBR019C	GAL10
YBR278W	DPB3	YPL001W	HAT1
YER116C	SLX8	YOR014W	RTS1
YBL102W	SFT2	YNL041C	COG6
YOR069W	VPS5	YAR014C	BUD14
YMR095C	SNO1	YNL041C	COG6
YCR088W	ABP1	YMR231W	PEP5
YPL178W	CBC2	YKL117W	SBA1
YDR108W	GSG1	YML071C	COG8
YDR080W	VPS41	YER162C	RAD4
YLR360W	VPS38	YLR396C	VPS33
YGL147C	RPL9A	YJL095W	BCK1
YOR368W	RAD17	YLR370C	ARC18
YBL008W	HIR1	YJR135C	MCM22
YER162C	RAD4	YPL195W	APL5

Predictions at threshold level 0.75 continued...			
Gene1 of predicted pair		Gene2 of predicted pair	
Systematic Name	Standard Name	Systematic Name	Standard Name
YOR337W	TEA1	YBR108W	YBR108W
YLR119W	SRN2	YKL041W	VPS24
YBR194W	SOY1	YDL002C	NHP10
YKL213C	DOA1	YMR067C	UBX4
YLL007C	YLL007C	YGR188C	BUB1
YPL024W	RMI1	YNL087W	TCB2
YDL080C	THI3	YNL215W	IES2
YGR105W	VMA21	YJL095W	BCK1
YNL291C	MID1	YAL002W	VPS8
YOR043W	WHI2	YKL161C	YKL161C
YKL139W	CTK1	YOL121C	RPS19A
YMR095C	SNO1	YBR173C	UMP1
YKL025C	PAN3	YHR111W	UBA4
YLR192C	HCR1	YHR039C-A	VMA10
YOR270C	VPH1	YMR311C	GLC8
YFR010W	UBP6	YGR270W	YTA7
YMR198W	CIK1	YNL206C	RTT106
YNL288W	CAF40	YMR127C	SAS2
YNL087W	TCB2	YHR081W	LRP1
YDR507C	GIN4	YNL289W	PCL1
YDL002C	NHP10	YER161C	SPT2
YMR116C	ASC1	YGL031C	RPL24A
YJL053W	PEP8	YDR080W	VPS41
YKL116C	PRR1	YBL025W	RRN10
YGL019W	CKB1	YOR368W	RAD17
YDR156W	RPA14	YHR013C	ARD1
YCR020C-A	MAK31	YJL036W	SNX4
YNR052C	POP2	YNL054W	VAC7
YBL058W	SHP1	YJR056C	YJR056C

Predictions at threshold level 0.75 continued...			
**Gene1 of predicted pair**		**Gene2 of predicted pair**	
**Systematic Name**	**Standard Name**	**Systematic Name**	**Standard Name**
YDR080W	VPS41	YOL108C	INO4
YBL025W	RRN10	YDR207C	UME6
YKL116C	PRR1	YBR217W	ATG12
YPL120W	VPS30	YDR153C	ENT5
YHR160C	PEX18	YMR026C	PEX12
YOR033C	EXO1	YGR081C	SLX9
YNL016W	PUB1	YDL013W	SLX5
YJL154C	VPS35	YHR060W	VMA22
YOL121C	RPS19A	YLR398C	SKI2
YIR005W	IST3	YOL081W	IRA2
YDR477W	SNF1	YBR280C	SAF1
YGR252W	GCN5	YOR043W	WHI2
YDR254W	CHL4	YMR038C	CCS1
YAL051W	OAF1	YKL032C	IXR1
YBR171W	SEC66	YDR261C	EXG2
YHR098C	SFB3	YDR485C	VPS72
YLR360W	VPS38	YHR178W	STB5
YNL041C	COG6	YGL124C	MON1
YJL095W	BCK1	YBL025W	RRN10
YER118C	SHO1	YPR119W	CLB2
YML115C	VAN1	YNL079C	TPM1
YGR261C	APL6	YDR320C	SWA2
YGL212W	VAM7	YPL057C	SUR1
YDR179C	CSN9	YJR084W	CSN12
YLR095C	IOC2	YNL076W	MKS1
YER122C	GLO3	YNL107W	YAF9
YGL025C	PGD1	YGL115W	SNF4
YER044C	ERG28	YBR019C	GAL10
YMR026C	PEX12	YER144C	UBP5

Predictions at threshold level 0.75 continued...			
Gene1 of predicted pair		Gene2 of predicted pair	
Systematic Name	Standard Name	Systematic Name	Standard Name
YBL078C	ATG8	YLL042C	ATG10
YGR129W	SYF2	YGR006W	PRP18
YOR078W	BUD21	YER102W	RPS8B
YPR084W	YPR084W	YNL051W	COG5
YIL034C	CAP2	YLR386W	VAC14
YDL155W	CLB3	YFR036W	CDC26
YGR105W	VMA21	YDR153C	ENT5
YBR108W	YBR108W	YDR310C	SUM1
YHR200W	RPN10	YPL248C	GAL4
YER144C	UBP5	YJL183W	MNN11
YFR031C-A	RPL2A	YEL043W	YEL043W
YHR178W	STB5	YPR120C	CLB5
YGL033W	HOP2	YEL060C	PRB1
YKR035W-A	DID2	YDL083C	RPS16B
YDR260C	SWM1	YGR188C	BUB1
YBR111W-A	SUS1	YOR023C	AHC1
YDL013W	SLX5	YOR014W	RTS1
YLR083C	EMP70	YHR012W	VPS29
YGL087C	MMS2	YBL021C	HAP3
YJL053W	PEP8	YEL043W	YEL043W
YEL043W	YEL043W	YPR032W	SRO7
YBR126C	TPS1	YLR094C	GIS3
YIL034C	CAP2	YGR152C	RSR1
YOR337W	TEA1	YDL223C	HBT1
YPL106C	SSE1	YJR005W	APL1
YBL047C	EDE1	YBR260C	RGD1
YMR153W	NUP53	YOR327C	SNC2
YER089C	PTC2	YDL006W	PTC1
YJL095W	BCK1	YMR038C	CCS1

Predictions at threshold level 0.75 continued...			
**Gene1 of predicted pair**		**Gene2 of predicted pair**	
**Systematic Name**	**Standard Name**	**Systematic Name**	**Standard Name**
YBL025W	RRN10	YER122C	GLO3
YML095C	RAD10	YMR231W	PEP5
YJR056C	YJR056C	YNL058C	YNL058C
YLR079W	SIC1	YNL079C	TPM1
YDR293C	SSD1	YDR153C	ENT5
YOR132W	VPS17	YPL120W	VPS30
YDL059C	RAD59	YDR076W	RAD55
YNL243W	SLA2	YLR170C	APS1
YDR378C	LSM6	YHR013C	ARD1
YEL009C	GCN4	YPL248C	GAL4
YLR079W	SIC1	YKL068W	NUP100
YDR244W	PEX5	YGL205W	POX1
YNL166C	BNI5	YDR183W	PLP1
YNL076W	MKS1	YLR398C	SKI2
YHR200W	RPN10	YDR207C	UME6
YHL031C	GOS1	YKR030W	GMH1
YNL032W	SIW14	YDR261C	EXG2
YOR109W	INP53	YKR030W	GMH1
YDR097C	MSH6	YMR167W	MLH1
YPR036W	VMA13	YBR280C	SAF1
YNR052C	POP2	YLR398C	SKI2
YHR081W	LRP1	YBL025W	RRN10
YPL253C	VIK1	YJR032W	CPR7
YPL018W	CTF19	YOL067C	RTG1
YOR270C	VPH1	YOR290C	SNF2
YOR290C	SNF2	YDR073W	SNF11
YLR315W	NKP2	YFR030W	MET10
YML007W	YAP1	YKL068W	NUP100
YPR017C	DSS4	YGL198W	YIP4

Predictions at threshold level 0.75 continued...			
Gene1 of predicted pair		Gene2 of predicted pair	
Systematic Name	Standard Name	Systematic Name	Standard Name
YFR031C-A	RPL2A	YHR200W	RPN10
YNL243W	SLA2	YNL051W	COG5
YLR386W	VAC14	YNL058C	YNL058C
YIL159W	BNR1	YDR484W	VPS52
YOR124C	UBP2	YAL002W	VPS8
YMR311C	GLC8	YOL081W	IRA2
YBR278W	DPB3	YGL148W	ARO2
YGR184C	UBR1	YML051W	GAL80
YMR153W	NUP53	YBR019C	GAL10
YMR133W	REC114	YHR081W	LRP1
YDL116W	NUP84	YBR082C	UBC4
YMR008C	PLB1	YLL061W	MMP1
YNL243W	SLA2	YOR284W	HUA2
YAL024C	LTE1	YPL057C	SUR1
YCR066W	RAD18	YAL002W	VPS8
YNL069C	RPL16B	YGL115W	SNF4
YEL013W	VAC8	YNL107W	YAF9
YOR069W	VPS5	YDR207C	UME6
YIL008W	URM1	YBR114W	RAD16
YDL080C	THI3	YOR308C	SNU66
YGL133W	ITC1	YOR275C	RIM20
YDR293C	SSD1	YGL115W	SNF4
YBL103C	RTG3	YDR261C	EXG2
YBR215W	HPC2	YKL213C	DOA1
YOL145C	CTR9	YKR048C	NAP1
YMR224C	MRE11	YBR215W	HPC2
YDR358W	GGA1	YOR327C	SNC2
YOL145C	CTR9	YLR357W	RSC2
YGL115W	SNF4	YMR133W	REC114

Predictions at threshold level 0.75 continued...			
Gene1 of predicted pair		Gene2 of predicted pair	
Systematic Name	Standard Name	Systematic Name	Standard Name
YDR443C	SSN2	YOL111C	MDY2
YEL053C	MAK10	YLR398C	SKI2
YDR225W	HTA1	YOR014W	RTS1
YCR066W	RAD18	YEL043W	YEL043W
YJR040W	GEF1	YNL127W	FAR11
YER001W	MNN1	YDR245W	MNN10
YOL136C	PFK27	YLR309C	IMH1
YBR217W	ATG12	YDL192W	ARF1
YBR171W	SEC66	YGL003C	CDH1
YEL065W	SIT1	YMR224C	MRE11
YCR053W	THR4	YDR310C	SUM1
YJR033C	RAV1	YDR153C	ENT5
YNR032W	PPG1	YDR207C	UME6
YJR060W	CBF1	YKL114C	APN1
YHR156C	LIN1	YDL077C	VAM6
YNL106C	INP52	YOR243C	PUS7
YHR073W	OSH3	YIL156W	UBP7
YCR065W	HCM1	YBL027W	RPL19B
YPL195W	APL5	YER122C	GLO3
YNL117W	MLS1	YDR260C	SWM1
YLR399C	BDF1	YGL200C	EMP24
YDR254W	CHL4	YFR036W	CDC26
YMR116C	ASC1	YBL072C	RPS8A
YHL025W	SNF6	YKL109W	HAP4
YLR360W	VPS38	YHL020C	OPI1
YPL057C	SUR1	YOR066W	MSA1
YDR318W	MCM21	YOL091W	SPO21
YBL088C	TEL1	YPL022W	RAD1
YOR132W	VPS17	YKL135C	APL2

Predictions at threshold level 0.75 continued...			
Gene1 of predicted pair		Gene2 of predicted pair	
Systematic Name	Standard Name	Systematic Name	Standard Name
YLR234W	TOP3	YHR039C-A	VMA10
YLR441C	RPS1A	YJR056C	YJR056C
YGL033W	HOP2	YLR320W	MMS22
YLR095C	IOC2	YMR016C	SOK2
YHR073W	OSH3	YER110C	KAP123
YBR290W	BSD2	YGR270W	YTA7
YCR081W	SRB8	YNL199C	GCR2
YBR194W	SOY1	YKL139W	CTK1
YGL175C	SAE2	YGL031C	RPL24A
YDR174W	HMO1	YGR188C	BUB1
YJL053W	PEP8	YOR069W	VPS5
YIL002C	INP51	YDL192W	ARF1
YCR034W	FEN1	YLR288C	MEC3
YIL034C	CAP2	YOR035C	SHE4
YOR337W	TEA1	YJR032W	CPR7
YOR275C	RIM20	YNL116W	DMA2
YKL048C	ELM1	YLR254C	NDL1
YHR134W	WSS1	YBR073W	RDH54
YIL159W	BNR1	YDR477W	SNF1
YBR217W	ATG12	YBL103C	RTG3
YGR261C	APL6	YIL076W	SEC28
YNL199C	GCR2	YEL036C	ANP1
YHR134W	WSS1	YBR082C	UBC4
YDR202C	RAV2	YDR310C	SUM1
YDR477W	SNF1	YOR066W	MSA1
YPR084W	YPR084W	YER120W	SCS2
YHR031C	RRM3	YHR056C	RSC30
YER162C	RAD4	YMR214W	SCJ1
YPL042C	SSN3	YCL037C	SRO9

Predictions at threshold level 0.75 continued...			
Gene1 of predicted pair		Gene2 of predicted pair	
Systematic Name	Standard Name	Systematic Name	Standard Name
YGL087C	MMS2	YOL067C	RTG1
YBL051C	PIN4	YDR144C	MKC7
YDR101C	ARX1	YKL009W	MRT4
YHR079C	IRE1	YOR275C	RIM20
YNL139C	RLR1	YHR167W	THP2
YBR280C	SAF1	YMR214W	SCJ1
YPL024W	RMI1	YMR311C	GLC8
YNL325C	FIG4	YFR036W	CDC26
YOL121C	RPS19A	YOL081W	IRA2
YNL106C	INP52	YML112W	CTK3
YOR080W	DIA2	YKL213C	DOA1
YKL139W	CTK1	YGL003C	CDH1
YGL025C	PGD1	YDL192W	ARF1
YFL023W	BUD27	YNL041C	COG6
YBR200W	BEM1	YGL124C	MON1
YOR368W	RAD17	YLR182W	SWI6
YGL174W	BUD13	YLR315W	NKP2
YFR031C-A	RPL2A	YAR014C	BUD14
YMR224C	MRE11	YER139C	YER139C
YJR059W	PTK2	YOR358W	HAP5
YDL020C	RPN4	YDL047W	SIT4
YBR114W	RAD16	YAL002W	VPS8
YGR104C	SRB5	YOR140W	SFL1
YHR084W	STE12	YLR452C	SST2
YBL058W	SHP1	YKR035W-A	DID2
YBR112C	CYC8	YPL248C	GAL4
YEL009C	GCN4	YGR252W	GCN5
YOR358W	HAP5	YKL068W	NUP100
YDR129C	SAC6	YKR014C	YPT52

Predictions at threshold level 0.75 continued...			
**Gene1 of predicted pair**		**Gene2 of predicted pair**	
**Systematic Name**	**Standard Name**	**Systematic Name**	**Standard Name**
YBR030W	YBR030W	YOR035C	SHE4
YOR112W	CEX1	YOR069W	VPS5
YBL047C	EDE1	YBL067C	UBP13
YPR051W	MAK3	YJR033C	RAV1
YJL004C	SYS1	YPL195W	APL5
YOR337W	TEA1	YOR327C	SNC2
YNL094W	APP1	YBR133C	HSL7
YGL202W	ARO8	YBR043C	QDR3
YFR031C-A	RPL2A	YIL076W	SEC28
YDR174W	HMO1	YLR399C	BDF1
YER162C	RAD4	YML071C	COG8
YBR173C	UMP1	YIL076W	SEC28
YNR052C	POP2	YPL203W	TPK2
YML115C	VAN1	YPL213W	LEA1
YLR192C	HCR1	YEL053C	MAK10
YOR132W	VPS17	YAL002W	VPS8
YMR016C	SOK2	YDL192W	ARF1
YOL145C	CTR9	YLR182W	SWI6
YGL226C-A	OST5	YMR214W	SCJ1
YNL079C	TPM1	YMR089C	YTA12
YLR292C	SEC72	YJL183W	MNN11
YGR268C	HUA1	YPL246C	RBD2
YLR192C	HCR1	YPR046W	MCM16
YBR084C-A	RPL19A	YDL047W	SIT4
YMR116C	ASC1	YNL329C	PEX6
YJL140W	RPB4	YLR357W	RSC2
YDL184C	RPL41A	YMR311C	GLC8
YER110C	KAP123	YJL095W	BCK1
YDR320C	SWA2	YDR202C	RAV2

Predictions at threshold level 0.75 continued...			
Gene1 of predicted pair		Gene2 of predicted pair	
Systematic Name	Standard Name	Systematic Name	Standard Name
YNL236W	SIN4	YJR035W	RAD26
YNL025C	SSN8	YOR308C	SNU66
YLR381W	CTF3	YMR109W	MYO5
YPR120C	CLB5	YPL256C	CLN2
YKR061W	KTR2	YHR115C	DMA1
YLL007C	YLL007C	YBR107C	IML3
YBR171W	SEC66	YER139C	YER139C
YHR039C-A	VMA10	YDR310C	SUM1
YBL047C	EDE1	YJR084W	CSN12
YNL229C	URE2	YKR048C	NAP1
YBR200W	BEM1	YDR358W	GGA1
YBR194W	SOY1	YNL297C	MON2
YNL297C	MON2	YLR447C	VMA6
YMR004W	MVP1	YCR066W	RAD18
YBR112C	CYC8	YBL103C	RTG3
YPL042C	SSN3	YPL018W	CTF19
YML016C	PPZ1	YNL236W	SIN4
YDR443C	SSN2	YER040W	GLN3
YDR335W	MSN5	YJR135C	MCM22
YMR095C	SNO1	YMR214W	SCJ1
YDR439W	LRS4	YMR055C	BUB2
YCR088W	ABP1	YOR036W	PEP12
YMR205C	PFK2	YMR038C	CCS1
YPR084W	YPR084W	YHR167W	THP2
YER139C	YER139C	YBR036C	CSG2
YDR289C	RTT103	YAR014C	BUD14
YDL002C	NHP10	YIR005W	IST3
YBR200W	BEM1	YAL002W	VPS8
YOR195W	SLK19	YKL048C	ELM1

Predictions at threshold level 0.75 continued...			
Gene1 of predicted pair		Gene2 of predicted pair	
Systematic Name	Standard Name	Systematic Name	Standard Name
YOR191W	RIS1	YPL024W	RMI1
YML001W	YPT7	YKR014C	YPT52
YBR290W	BSD2	YKR024C	DBP7
YIL053W	RHR2	YLR180W	SAM1
YOR112W	CEX1	YAR014C	BUD14
YDR129C	SAC6	YBR260C	RGD1
YIL156W	UBP7	YMR153W	NUP53
YJR084W	CSN12	YMR311C	GLC8
YLR083C	EMP70	YPL161C	BEM4
YBR228W	SLX1	YPL057C	SUR1
YBR042C	CST26	YML115C	VAN1
YML115C	VAN1	YBR108W	YBR108W
YNL087W	TCB2	YNL058C	YNL058C
YLR447C	VMA6	YHR114W	BZZ1
YPL032C	SVL3	YLR429W	CRN1
YJR084W	CSN12	YBR082C	UBC4
YPL253C	VIK1	YEL053C	MAK10
YNL068C	FKH2	YPR120C	CLB5
YPL161C	BEM4	YLR452C	SST2
YIL036W	CST6	YER118C	SHO1
YOR112W	CEX1	YNL051W	COG5
YEL065W	SIT1	YNL076W	MKS1
YLR192C	HCR1	YMR152W	YIM1
YHR039C-A	VMA10	YOL081W	IRA2
YPL037C	EGD1	YLR093C	NYV1
YDR225W	HTA1	YGR188C	BUB1
YCR081W	SRB8	YDR456W	NHX1
YBL058W	SHP1	YLR452C	SST2
YFR031C-A	RPL2A	YJR056C	YJR056C

Predictions at threshold level 0.75 continued...			
Gene1 of predicted pair		Gene2 of predicted pair	
Systematic Name	Standard Name	Systematic Name	Standard Name
YNL325C	FIG4	YBR171W	SEC66
YBL103C	RTG3	YFR021W	ATG18
YPR084W	YPR084W	YNL215W	IES2
YBR108W	YBR108W	YBR239C	YBR239C
YLR119W	SRN2	YHR156C	LIN1
YKR061W	KTR2	YDR183W	PLP1
YER116C	SLX8	YPR120C	CLB5
YJR032W	CPR7	YMR230W	RPS10B
YDR155C	CPR1	YDR183W	PLP1
YMR133W	REC114	YLR093C	NYV1
YHR039C-A	VMA10	YBR019C	GAL10
YGR184C	UBR1	YLR150W	STM1
YNL069C	RPL16B	YAR014C	BUD14
YNL293W	MSB3	YAL030W	SNC1
YML016C	PPZ1	YLR233C	EST1
YFR031C-A	RPL2A	YBL102W	SFT2
YNL044W	YIP3	YMR054W	STV1
YPL042C	SSN3	YDR378C	LSM6
YHR134W	WSS1	YOR243C	PUS7
YEL022W	GEA2	YKR030W	GMH1
YGR105W	VMA21	YNL127W	FAR11
YEL043W	YEL043W	YNL238W	KEX2
YKL126W	YPK1	YDR310C	SUM1
YGL003C	CDH1	YHR115C	DMA1
YBR200W	BEM1	YJR032W	CPR7
YDR004W	RAD57	YDL002C	NHP10
YPL213W	LEA1	YLR370C	ARC18
YDR146C	SWI5	YPL248C	GAL4
YOR106W	VAM3	YJR032W	CPR7

Predictions at threshold level 0.75 continued...			
Gene1 of predicted pair		Gene2 of predicted pair	
Systematic Name	Standard Name	Systematic Name	Standard Name
YDR176W	NGG1	YDL005C	MED2
YBR131W	CCZ1	YDR137W	RGP1
YOR358W	HAP5	YNL199C	GCR2
YDR004W	RAD57	YLR394W	CST9
YOR375C	GDH1	YKL041W	VPS24
YMR116C	ASC1	YLR370C	ARC18
YMR133W	REC114	YJR084W	CSN12
YOR083W	WHI5	YJR060W	CBF1
YGR019W	UGA1	YAL002W	VPS8
YHL031C	GOS1	YDR153C	ENT5
YPR102C	RPL11A	YPL240C	HSP82
YLR095C	IOC2	YER116C	SLX8
YBR173C	UMP1	YPR046W	MCM16
YLR386W	VAC14	YML071C	COG8
YGL253W	HXK2	YHL002W	HSE1
YIL036W	CST6	YDR254W	CHL4
YLR079W	SIC1	YJL127C	SPT10
YJR140C	HIR3	YOR001W	RRP6
YNL079C	TPM1	YJL095W	BCK1
YMR216C	SKY1	YPL161C	BEM4
YLR399C	BDF1	YDR335W	MSN5
YOL018C	TLG2	YOL108C	INO4
YMR026C	PEX12	YBR171W	SEC66
YNL215W	IES2	YLR315W	NKP2
YNL215W	IES2	YIL103W	DPH1
YOR337W	TEA1	YJL172W	CPS1
YHR111W	UBA4	YLR410W	VIP1
YDL223C	HBT1	YMR152W	YIM1
YGR104C	SRB5	YHL002W	HSE1

Predictions at threshold level 0.75 continued...			
Gene1 of predicted pair		Gene2 of predicted pair	
Systematic Name	Standard Name	Systematic Name	Standard Name
YDR484W	VPS52	YLR396C	VPS33
YPL161C	BEM4	YJR135C	MCM22
YPR036W	VMA13	YPL195W	APL5
YDR335W	MSN5	YGL252C	RTG2
YHR205W	SCH9	YNL079C	TPM1
YBR171W	SEC66	YML001W	YPT7
YKL006W	RPL14A	YNL079C	TPM1
YER161C	SPT2	YEL013W	VAC8
YHR171W	ATG7	YMR159C	ATG16
YGR184C	UBR1	YPL057C	SUR1
YHR060W	VMA22	YMR054W	STV1
YHR161C	YAP1801	YKR020W	VPS51
YNL069C	RPL16B	YPL161C	BEM4
YNL054W	VAC7	YDR439W	LRS4
YLR095C	IOC2	YMR152W	YIM1
YFL023W	BUD27	YMR214W	SCJ1
YNL238W	KEX2	YOL018C	TLG2
YKL213C	DOA1	YJL048C	UBX6
YNL238W	KEX2	YAL002W	VPS8
YGR081C	SLX9	YER116C	SLX8
YOR080W	DIA2	YOR141C	ARP8
YML095C	RAD10	YDR264C	AKR1
YBR200W	BEM1	YOR023C	AHC1
YDR108W	GSG1	YEL053C	MAK10
YDR129C	SAC6	YLR360W	VPS38
YJL053W	PEP8	YNL297C	MON2
YGL223C	COG1	YMR054W	STV1
YDR176W	NGG1	YBR111W-A	SUS1
YDR245W	MNN10	YBL047C	EDE1

Predictions at threshold level 0.75 continued...			
**Gene1 of predicted pair**		**Gene2 of predicted pair**	
**Systematic Name**	**Standard Name**	**Systematic Name**	**Standard Name**
YIL159W	BNR1	YOR066W	MSA1
YLR452C	SST2	YLR180W	SAM1
YMR004W	MVP1	YLR447C	VMA6
YPL024W	RMI1	YNR072W	HXT17
YPR066W	UBA3	YLR306W	UBC12
YIL156W	UBP7	YJL095W	BCK1
YHR200W	RPN10	YOR035C	SHE4
YDL065C	PEX19	YNL214W	PEX17
YDR004W	RAD57	YMR179W	SPT21
YMR214W	SCJ1	YOL081W	IRA2
YDR443C	SSN2	YML115C	VAN1
YNL058C	YNL058C	YDL083C	RPS16B
YDR156W	RPA14	YGR270W	YTA7
YOL030W	GAS5	YGL175C	SAE2
YDL020C	RPN4	YDL160C	DHH1
YML095C	RAD10	YNL107W	YAF9
YGR104C	SRB5	YOR080W	DIA2
YHL031C	GOS1	YAR014C	BUD14
YMR127C	SAS2	YBR171W	SEC66
YDR179C	CSN9	YDL216C	RRI1
YNL044W	YIP3	YKR014C	YPT52
YIL008W	URM1	YPL256C	CLN2
YGL033W	HOP2	YMR311C	GLC8
YJL058C	BIT61	YGL003C	CDH1
YDL006W	PTC1	YPR119W	CLB2
YJL036W	SNX4	YEL053C	MAK10
YLR095C	IOC2	YPL195W	APL5
YGR261C	APL6	YGL054C	ERV14
YKL048C	ELM1	YDR207C	UME6

Predictions at threshold level 0.75 continued...			
Gene1 of predicted pair		Gene2 of predicted pair	
Systematic Name	Standard Name	Systematic Name	Standard Name
YLR119W	SRN2	YBL058W	SHP1
YCL037C	SRO9	YPR119W	CLB2
YCL037C	SRO9	YHR012W	VPS29
YGR261C	APL6	YDR080W	VPS41
YNL166C	BNI5	YCL024W	KCC4
YHL031C	GOS1	YLR315W	NKP2
YGL133W	ITC1	YLR386W	VAC14
YNL307C	MCK1	YOL081W	IRA2
YDR358W	GGA1	YPL259C	APM1
YDR293C	SSD1	YPL120W	VPS30
YDR395W	SXM1	YBR017C	KAP104
YHR206W	SKN7	YLR433C	CNA1
YMR095C	SNO1	YIL076W	SEC28
YDR192C	NUP42	YML109W	ZDS2
YNL206C	RTT106	YBR119W	MUD1
YGR104C	SRB5	YNL079C	TPM1
YMR127C	SAS2	YCR086W	CSM1
YGR241C	YAP1802	YHR108W	GGA2
YFR031C-A	RPL2A	YKL048C	ELM1
YMR172W	HOT1	YNL107W	YAF9
YNR072W	HXT17	YOL081W	IRA2
YDR156W	RPA14	YBR107C	IML3
YBL021C	HAP3	YEL056W	HAT2
YAR014C	BUD14	YHR115C	DMA1
YMR039C	SUB1	YDR289C	RTT103
YDR469W	SDC1	YPL018W	CTF19
YPL115C	BEM3	YDR318W	MCM21
YLR090W	XDJ1	YPR036W	VMA13
YMR153W	NUP53	YPR046W	MCM16

Predictions at threshold level 0.75 continued...			
Gene1 of predicted pair		Gene2 of predicted pair	
Systematic Name	Standard Name	Systematic Name	Standard Name
YLR313C	SPH1	YDL159W	STE7
YGL025C	PGD1	YHR081W	LRP1
YOL121C	RPS19A	YER040W	GLN3
YDR261C	EXG2	YGL124C	MON1
YLR410W	VIP1	YNL238W	KEX2
YBL008W	HIR1	YDR200C	VPS64
YBR036C	CSG2	YDR207C	UME6
YJL124C	LSM1	YBR082C	UBC4
YML115C	VAN1	YPL050C	MNN9
YFR031C-A	RPL2A	YMR152W	YIM1
YBR108W	YBR108W	YGL212W	VAM7
YLR381W	CTF3	YHR200W	RPN10
YGL054C	ERV14	YFL048C	EMP47
YLR093C	NYV1	YBR280C	SAF1
YKL006W	RPL14A	YDR378C	LSM6
YHR200W	RPN10	YLR320W	MMS22
YEL050C	RML2	YOL091W	SPO21
YMR026C	PEX12	YLR398C	SKI2
YPL137C	GIP3	YLR093C	NYV1
YHR178W	STB5	YBR171W	SEC66
YNR052C	POP2	YOR141C	ARP8
YLR386W	VAC14	YLR398C	SKI2
YNL021W	HDA1	YCR084C	TUP1
YPR084W	YPR084W	YJL036W	SNX4
YBR041W	FAT1	YKL094W	YJU3
YGL212W	VAM7	YNR072W	HXT17
YDL155W	CLB3	YDR144C	MKC7
YDR129C	SAC6	YHR161C	YAP1801
YJR083C	ACF4	YBR108W	YBR108W

Predictions at threshold level 0.75 continued...			
Gene1 of predicted pair		Gene2 of predicted pair	
Systematic Name	Standard Name	Systematic Name	Standard Name
YGL147C	RPL9A	YML071C	COG8
YHL020C	OPI1	YJL004C	SYS1
YGL212W	VAM7	YGR105W	VMA21
YMR127C	SAS2	YPR119W	CLB2
YMR127C	SAS2	YDR254W	CHL4
YER044C	ERG28	YBR280C	SAF1
YDR137W	RGP1	YBR019C	GAL10
YNL106C	INP52	YLR182W	SWI6
YCR065W	HCM1	YDL185W	TFP1
YER001W	MNN1	YKR020W	VPS51
YJL154C	VPS35	YMR311C	GLC8
YBR290W	BSD2	YPL256C	CLN2
YCR066W	RAD18	YAR014C	BUD14
YMR224C	MRE11	YHL002W	HSE1
YBL025W	RRN10	YLR370C	ARC18
YOR358W	HAP5	YHL002W	HSE1
YOR337W	TEA1	YML001W	YPT7
YKR057W	RPS21A	YHR152W	SPO12
YJR060W	CBF1	YOL091W	SPO21
YMR192W	GYL1	YLR144C	ACF2
YJL036W	SNX4	YNL051W	COG5
YPL105C	YPL105C	YPL195W	APL5
YBL008W	HIR1	YBR289W	SNF5
YLR079W	SIC1	YPL106C	SSE1
YNL218W	MGS1	YPL079W	RPL21B
YLR292C	SEC72	YDR254W	CHL4
YPL152W	RRD2	YDL047W	SIT4
YPL105C	YPL105C	YPR119W	CLB2
YDR137W	RGP1	YLR182W	SWI6

Predictions at threshold level 0.75 continued...			
Gene1 of predicted pair		Gene2 of predicted pair	
Systematic Name	Standard Name	Systematic Name	Standard Name
YMR116C	ASC1	YOL090W	MSH2
YER120W	SCS2	YNL127W	FAR11
YOR270C	VPH1	YER144C	UBP5
YLR234W	TOP3	YOL067C	RTG1
YLR207W	HRD3	YMR022W	UBC7
YLR447C	VMA6	YNL041C	COG6
YJR032W	CPR7	YMR167W	MLH1
YNL106C	INP52	YIL036W	CST6
YBL008W	HIR1	YLR399C	BDF1
YBR200W	BEM1	YKR035W-A	DID2
YBR200W	BEM1	YBR041W	FAT1
YMR016C	SOK2	YMR214W	SCJ1
YBR290W	BSD2	YBL079W	NUP170
YNL106C	INP52	YLR219W	MSC3
YLR381W	CTF3	YGR188C	BUB1
YML103C	NUP188	YJL006C	CTK2
YDR225W	HTA1	YML028W	TSA1
YDR129C	SAC6	YNL079C	TPM1
YCR066W	RAD18	YML001W	YPT7
YGR108W	CLB1	YNL236W	SIN4
YKL110C	KTI12	YDL047W	SIT4
YLR292C	SEC72	YNL288W	CAF40
YBL047C	EDE1	YDR207C	UME6
YAL024C	LTE1	YDL077C	VAM6
YPR017C	DSS4	YNL297C	MON2
YOR290C	SNF2	YBR009C	HHF1
YNL297C	MON2	YKR001C	VPS1
YOL111C	MDY2	YHR200W	RPN10
YKL048C	ELM1	YOL067C	RTG1

Predictions at threshold level 0.75 continued...			
Gene1 of predicted pair		Gene2 of predicted pair	
Systematic Name	Standard Name	Systematic Name	Standard Name
YLR090W	XDJ1	YDR261C	EXG2
YMR142C	RPL13B	YDL083C	RPS16B
YMR095C	SNO1	YHR156C	LIN1
YER110C	KAP123	YKL068W	NUP100
YGL033W	HOP2	YGL183C	MND1
YAL024C	LTE1	YBR200W	BEM1
YOR290C	SNF2	YOR066W	MSA1
YFR010W	UBP6	YHL030W	ECM29
YBR127C	VMA2	YPL045W	VPS16
YMR273C	ZDS1	YNL030W	HHF2
YAL024C	LTE1	YGR184C	UBR1
YPL129W	TAF14	YOL051W	GAL11
YGL087C	MMS2	YJL095W	BCK1
YDL020C	RPN4	YLR352W	YLR352W
YML103C	NUP188	YIL148W	RPL40A
YIL076W	SEC28	YOR127W	RGA1
YDR440W	DOT1	YLR350W	ORM2
YJR063W	RPA12	YDR289C	RTT103
YLR360W	VPS38	YOR127W	RGA1
YLR090W	XDJ1	YER139C	YER139C
YBR017C	KAP104	YKR082W	NUP133
YMR152W	YIM1	YER040W	GLN3
YNL288W	CAF40	YMR055C	BUB2
YOL108C	INO4	YAL002W	VPS8
YGL147C	RPL9A	YNL087W	TCB2
YDR245W	MNN10	YGR135W	PRE9
YJL036W	SNX4	YER120W	SCS2
YER116C	SLX8	YML095C	RAD10
YPR036W	VMA13	YDR358W	GGA1

Predictions at threshold level 0.75 continued...			
Gene1 of predicted pair		Gene2 of predicted pair	
Systematic Name	Standard Name	Systematic Name	Standard Name
YPL057C	SUR1	YPL079W	RPL21B
YBR270C	BIT2	YNL047C	SLM2
YIL053W	RHR2	YJR056C	YJR056C
YOR195W	SLK19	YHR013C	ARD1
YNL025C	SSN8	YBR194W	SOY1
YBR009C	HHF1	YKL068W	NUP100
YDL020C	RPN4	YML028W	TSA1
YNL044W	YIP3	YHR012W	VPS29
YPL178W	CBC2	YGL016W	KAP122
YPL018W	CTF19	YKL101W	HSL1
YBR182C	SMP1	YPL089C	RLM1
YDR156W	RPA14	YGL163C	RAD54
YGL038C	OCH1	YNL051W	COG5
YJL095W	BCK1	YGL019W	CKB1
YER114C	BOI2	YGR152C	RSR1
YML102W	CAC2	YML028W	TSA1
YDR443C	SSN2	YHR156C	LIN1
YDL155W	CLB3	YGR135W	PRE9
YOR112W	CEX1	YER162C	RAD4
YEL044W	IES6	YGL200C	EMP24
YBL103C	RTG3	YLR398C	SKI2
YJR024C	MDE1	YKL117W	SBA1
YBR108W	YBR108W	YDR358W	GGA1
YOR127W	RGA1	YPL195W	APL5
YNL297C	MON2	YJR032W	CPR7
YOR112W	CEX1	YKR020W	VPS51
YLR360W	VPS38	YBR128C	ATG14
YBL008W	HIR1	YER161C	SPT2
YFL023W	BUD27	YMR231W	PEP5

Predictions at threshold level 0.75 continued...			
Gene1 of predicted pair		Gene2 of predicted pair	
Systematic Name	Standard Name	Systematic Name	Standard Name
YDR448W	ADA2	YOR023C	AHC1
YGR159C	NSR1	YJL095W	BCK1
YNL297C	MON2	YFL033C	RIM15
YLR386W	VAC14	YBL103C	RTG3
YPL057C	SUR1	YBR215W	HPC2
YPR018W	RLF2	YBR009C	HHF1
YGR249W	MGA1	YPR030W	CSR2
YGL115W	SNF4	YGL003C	CDH1
YER115C	SPR6	YPL246C	RBD2
YPL129W	TAF14	YBR010W	HHT1
YIL036W	CST6	YBR200W	BEM1
YKL116C	PRR1	YHR081W	LRP1
YDR254W	CHL4	YJL190C	RPS22A
YER173W	RAD24	YHR079C-A	SAE3
YHR161C	YAP1801	YMR231W	PEP5
YKL213C	DOA1	YDR310C	SUM1
YJL154C	VPS35	YPR046W	MCM16
YPR084W	YPR084W	YIL076W	SEC28
YBL103C	RTG3	YPL137C	GIP3
YMR109W	MYO5	YMR032W	HOF1
YMR016C	SOK2	YER144C	UBP5
YOL145C	CTR9	YDL005C	MED2
YAL024C	LTE1	YLR288C	MEC3
YPL240C	HSP82	YGL095C	VPS45
YLR079W	SIC1	YMR311C	GLC8
YHR200W	RPN10	YLR094C	GIS3
YMR179W	SPT21	YOR001W	RRP6
YPL152W	RRD2	YDR289C	RTT103
YDL116W	NUP84	YLR399C	BDF1

Predictions at threshold level 0.75 continued...			
Gene1 of predicted pair		Gene2 of predicted pair	
Systematic Name	Standard Name	Systematic Name	Standard Name
YIL159W	BNR1	YIL076W	SEC28
YGR054W	YGR054W	YPL018W	CTF19
YPL161C	BEM4	YGL031C	RPL24A
YBL008W	HIR1	YBR009C	HHF1
YJL172W	CPS1	YGL025C	PGD1
YHR156C	LIN1	YMR152W	YIM1
YHR152W	SPO12	YGL163C	RAD54
YOR054C	VHS3	YDL047W	SIT4
YLR234W	TOP3	YDR144C	MKC7
YLR386W	VAC14	YOL054W	PSH1
YOL145C	CTR9	YDR363W-A	SEM1
YLR360W	VPS38	YDR320C	SWA2
YBL008W	HIR1	YPL024W	RMI1
YDR103W	STE5	YBR083W	TEC1
YFR036W	CDC26	YDR183W	PLP1
YBL079W	NUP170	YMR038C	CCS1
YGR019W	UGA1	YJR135C	MCM22
YDL223C	HBT1	YGL003C	CDH1
YDR225W	HTA1	YER116C	SLX8
YPL158C	YPL158C	YKL161C	YKL161C
YBL103C	RTG3	YHR060W	VMA22
YGL033W	HOP2	YML071C	COG8
YDR004W	RAD57	YMR167W	MLH1
YMR205C	PFK2	YLR320W	MMS22
YPL024W	RMI1	YJL172W	CPS1
YEL051W	VMA8	YDR310C	SUM1
YPL042C	SSN3	YLR429W	CRN1
YJL154C	VPS35	YDR323C	PEP7
YIL036W	CST6	YLR182W	SWI6

Predictions at threshold level 0.75 continued...			
Gene1 of predicted pair		Gene2 of predicted pair	
Systematic Name	Standard Name	Systematic Name	Standard Name
YBR200W	BEM1	YJL183W	MNN11
YMR238W	DFG5	YHR061C	GIC1
YBL051C	PIN4	YBR107C	IML3
YPL022W	RAD1	YDR076W	RAD55
YJL053W	PEP8	YAL002W	VPS8
YLR061W	RPL22A	YML097C	VPS9
YKL116C	PRR1	YKL139W	CTK1
YMR026C	PEX12	YLR386W	VAC14
YPL050C	MNN9	YML012W	ERV25
YBR195C	MSI1	YLR370C	ARC18
YLR320W	MMS22	YPL195W	APL5
YJL154C	VPS35	YPL105C	YPL105C
YDR320C	SWA2	YPL120W	VPS30
YAL024C	LTE1	YLR094C	GIS3
YDR484W	VPS52	YKL126W	YPK1
YHR178W	STB5	YOR308C	SNU66
YLR337C	VRP1	YHL027W	RIM101
YMR055C	BUB2	YJR090C	GRR1
YPL057C	SUR1	YMR230W	RPS10B
YIL103W	DPH1	YOR141C	ARP8
YBR258C	SHG1	YNL297C	MON2
YDR320C	SWA2	YPR032W	SRO7
YOR127W	RGA1	YGL163C	RAD54
YHR205W	SCH9	YDR245W	MNN10
YDR383C	NKP1	YNL225C	CNM67
YIL034C	CAP2	YGR054W	YGR054W
YPL161C	BEM4	YEL036C	ANP1
YPL037C	EGD1	YOR035C	SHE4
YHR178W	STB5	YBR195C	MSI1

Predictions at threshold level 0.75 continued...			
Gene1 of predicted pair		Gene2 of predicted pair	
Systematic Name	Standard Name	Systematic Name	Standard Name
YOR043W	WHI2	YHR108W	GGA2
YHR111W	UBA4	YDL155W	CLB3
YGR270W	YTA7	YGL124C	MON1
YGL147C	RPL9A	YFR036W	CDC26
YGL200C	EMP24	YOR375C	GDH1
YNL293W	MSB3	YPR028W	YOP1
YPR084W	YPR084W	YDR293C	SSD1
YGL115W	SNF4	YNL236W	SIN4
YKL139W	CTK1	YNL004W	HRB1
YGL212W	VAM7	YGL025C	PGD1
YBR083W	TEC1	YJR099W	YUH1
YNL225C	CNM67	YGL252C	RTG2
YNL106C	INP52	YKR020W	VPS51
YLR090W	XDJ1	YNL041C	COG6
YGR104C	SRB5	YOR001W	RRP6
YDL127W	PCL2	YDR310C	SUM1
YGL212W	VAM7	YEL051W	VMA8
YBR173C	UMP1	YNL325C	FIG4
YMR164C	MSS11	YBL079W	NUP170
YGL147C	RPL9A	YDL077C	VAM6
YNL187W	YNL187W	YDR515W	SLF1
YHR178W	STB5	YDR289C	RTT103
YFR010W	UBP6	YOR308C	SNU66
YJL176C	SWI3	YGR071C	YGR071C
YDR389W	SAC7	YLR120C	YPS1
YDR254W	CHL4	YPR046W	MCM16
YHR167W	THP2	YER063W	THO1
YEL065W	SIT1	YBR173C	UMP1
YLR234W	TOP3	YPR120C	CLB5

Predictions at threshold level 0.75 continued...			
Gene1 of predicted pair		Gene2 of predicted pair	
Systematic Name	Standard Name	Systematic Name	Standard Name
YDR443C	SSN2	YLR320W	MMS22
YGL223C	COG1	YER120W	SCS2
YPR018W	RLF2	YGL066W	SGF73
YDR484W	VPS52	YMR231W	PEP5
YGR019W	UGA1	YNL166C	BNI5
YLR182W	SWI6	YHR115C	DMA1
YML103C	NUP188	YJR063W	RPA12
YIL053W	RHR2	YPL161C	BEM4
YKR061W	KTR2	YJR063W	RPA12
YIL076W	SEC28	YGL054C	ERV14
YOR109W	INP53	YPR119W	CLB2
YHR010W	RPL27A	YIL133C	RPL16A
YKL048C	ELM1	YNL041C	COG6
YJR135C	MCM22	YOR308C	SNU66
YBR112C	CYC8	YLR176C	RFX1
YLR447C	VMA6	YHR012W	VPS29
YGL163C	RAD54	YDR264C	AKR1
YJL127C	SPT10	YOL091W	SPO21
YPR072W	NOT5	YOL148C	SPT20
YIL076W	SEC28	YOL121C	RPS19A
YGR019W	UGA1	YER110C	KAP123
YPL267W	ACM1	YDL155W	CLB3
YMR224C	MRE11	YDR440W	DOT1
YDR192C	NUP42	YKL068W	NUP100
YCL032W	STE50	YBL016W	FUS3
YPL024W	RMI1	YOR243C	PUS7
YLR018C	POM34	YLR234W	TOP3
YBR108W	YBR108W	YCR053W	THR4
YKR072C	SIS2	YMR231W	PEP5

Predictions at threshold level 0.75 continued...			
Gene1 of predicted pair		Gene2 of predicted pair	
Systematic Name	Standard Name	Systematic Name	Standard Name
YDR261C	EXG2	YLR093C	NYV1
YNL116W	DMA2	YOL054W	PSH1
YFR036W	CDC26	YMR230W	RPS10B
YDR318W	MCM21	YDR076W	RAD55
YMR116C	ASC1	YPL022W	RAD1
YOR132W	VPS17	YNL297C	MON2
YML001W	YPT7	YER040W	GLN3
YOR038C	HIR2	YBR010W	HHT1
YJR056C	YJR056C	YEL053C	MAK10
YDR156W	RPA14	YLR320W	MMS22
YML095C	RAD10	YHL002W	HSE1
YLR061W	RPL22A	YDR129C	SAC6
YKR020W	VPS51	YPL195W	APL5
YPL129W	TAF14	YEL013W	VAC8
YPL024W	RMI1	YPL037C	EGD1
YBR200W	BEM1	YMR205C	PFK2
YPR052C	NHP6A	YGL094C	PAN2
YDL184C	RPL41A	YLR370C	ARC18
YDR385W	EFT2	YGR105W	VMA21
YDR477W	SNF1	YBR030W	YBR030W
YGR019W	UGA1	YJL190C	RPS22A
YKR020W	VPS51	YJL183W	MNN11
YML028W	TSA1	YKR030W	GMH1
YHR079C	IRE1	YBR111W-A	SUS1
YFR040W	SAP155	YMR052W	FAR3
YHL031C	GOS1	YOR109W	INP53
YDL052C	SLC1	YKR046C	PET10
YPL125W	KAP120	YBR017C	KAP104
YOL111C	MDY2	YBL025W	RRN10

Predictions at threshold level 0.75 continued...			
Gene1 of predicted pair		Gene2 of predicted pair	
Systematic Name	Standard Name	Systematic Name	Standard Name
YDR443C	SSN2	YPR030W	CSR2
YJR135C	MCM22	YAL002W	VPS8
YPL106C	SSE1	YJR135C	MCM22
YDR142C	PEX7	YAR042W	SWH1
YCR081W	SRB8	YDR207C	UME6
YPL042C	SSN3	YNL215W	IES2
YGL175C	SAE2	YHR010W	RPL27A
YGL190C	CDC55	YKR082W	NUP133
YGR188C	BUB1	YLR032W	RAD5
YPR036W	VMA13	YDR310C	SUM1
YNL106C	INP52	YPL253C	VIK1
YMR216C	SKY1	YEL043W	YEL043W
YGL066W	SGF73	YCL010C	SGF29
YGL078C	DBP3	YJR032W	CPR7
YNL215W	IES2	YNL116W	DMA2
YCL001W	RER1	YOL018C	TLG2
YFR031C-A	RPL2A	YOR337W	TEA1
YNL166C	BNI5	YDR254W	CHL4
YMR142C	RPL13B	YGL025C	PGD1
YBL021C	HAP3	YJL095W	BCK1
YPL018W	CTF19	YJL190C	RPS22A
YOR337W	TEA1	YER122C	GLO3
YML095C	RAD10	YMR038C	CCS1
YKL006W	RPL14A	YDR129C	SAC6
YKL181W	PRS1	YER099C	PRS2
YBR084C-A	RPL19A	YNL229C	URE2
YOR112W	CEX1	YGL115W	SNF4
YPL256C	CLN2	YKR024C	DBP7
YOR080W	DIA2	YML016C	PPZ1

Predictions at threshold level 0.75 continued...			
Gene1 of predicted pair		Gene2 of predicted pair	
Systematic Name	Standard Name	Systematic Name	Standard Name
YDR318W	MCM21	YML028W	TSA1
YDL155W	CLB3	YDL047W	SIT4
YGR270W	YTA7	YPL152W	RRD2
YLR094C	GIS3	YOL121C	RPS19A
YKL007W	CAP1	YGR080W	TWF1
YER120W	SCS2	YER122C	GLO3
YDR293C	SSD1	YDL006W	PTC1
YER161C	SPT2	YPR120C	CLB5
YNL248C	RPA49	YLR410W	VIP1
YNL325C	FIG4	YJL183W	MNN11
YHR216W	IMD2	YPL240C	HSP82
YMR224C	MRE11	YLR452C	SST2
YGL174W	BUD13	YDR318W	MCM21
YDR265W	PEX10	YAL055W	PEX22
YNL297C	MON2	YEL042W	GDA1
YIL159W	BNR1	YPL057C	SUR1
YLR058C	SHM2	YLR381W	CTF3
YBL008W	HIR1	YGL151W	NUT1
YJL073W	JEM1	YOR085W	OST3
YGL212W	VAM7	YHR081W	LRP1
YLR234W	TOP3	YEL053C	MAK10
YPL161C	BEM4	YPR032W	SRO7
YCR002C	CDC10	YJL095W	BCK1
YOR106W	VAM3	YOL108C	INO4
YBL103C	RTG3	YML001W	YPT7
YNL307C	MCK1	YDR183W	PLP1
YKL010C	UFD4	YDR440W	DOT1
YOR212W	STE4	YLR362W	STE11
YIL034C	CAP2	YBL051C	PIN4

Predictions at threshold level 0.75 continued...			
Gene1 of predicted pair		Gene2 of predicted pair	
Systematic Name	Standard Name	Systematic Name	Standard Name
YFR031C-A	RPL2A	YKR001C	VPS1
YHR167W	THP2	YER122C	GLO3
YPL195W	APL5	YPR032W	SRO7
YHR134W	WSS1	YAL002W	VPS8
YJL183W	MNN11	YBR036C	CSG2
YOR332W	VMA4	YDL185W	TFP1
YBR065C	ECM2	YPR101W	SNT309
YOR358W	HAP5	YJR135C	MCM22
YLR216C	CPR6	YGL212W	VAM7
YDL116W	NUP84	YGR108W	CLB1
YPR084W	YPR084W	YBR194W	SOY1
YCR020C-A	MAK31	YOL108C	INO4
YPL129W	TAF14	YLR399C	BDF1
YBL102W	SFT2	YNL297C	MON2
YJR063W	RPA12	YLR182W	SWI6
YOR327C	SNC2	YPR032W	SRO7
YML001W	YPT7	YMR152W	YIM1
YCR066W	RAD18	YLR032W	RAD5
YLR337C	VRP1	YJR135C	MCM22
YBR217W	ATG12	YBR280C	SAF1
YHR079C	IRE1	YDR176W	NGG1
YIL159W	BNR1	YBR217W	ATG12
YPR052C	NHP6A	YOL148C	SPT20
YBR036C	CSG2	YGL148W	ARO2
YPL161C	BEM4	YNL238W	KEX2
YOR043W	WHI2	YML071C	COG8
YKL006W	RPL14A	YHL002W	HSE1
YCL037C	SRO9	YPL037C	EGD1
YGL066W	SGF73	YKR057W	RPS21A

Predictions at threshold level 0.75 continued...			
Gene1 of predicted pair		Gene2 of predicted pair	
Systematic Name	Standard Name	Systematic Name	Standard Name
YOR290C	SNF2	YHR200W	RPN10
YDL013W	SLX5	YGL175C	SAE2
YDR293C	SSD1	YGR054W	YGR054W
YLR102C	APC9	YBL087C	RPL23A
YDR477W	SNF1	YDR028C	REG1
YBR228W	SLX1	YMR284W	YKU70
YBR030W	YBR030W	YDL083C	RPS16B
YDR443C	SSN2	YLR441C	RPS1A
YML016C	PPZ1	YNL225C	CNM67
YEL056W	HAT2	YHL002W	HSE1
YHR200W	RPN10	YMR038C	CCS1
YOL121C	RPS19A	YDL077C	VAM6
YLR337C	VRP1	YLR425W	TUS1
YJL124C	LSM1	YLR313C	SPH1
YJR140C	HIR3	YHL031C	GOS1
YBR057C	MUM2	YPL096C-A	ERI1
YPL137C	GIP3	YER139C	YER139C
YHR200W	RPN10	YBL025W	RRN10
YPL024W	RMI1	YLR119W	SRN2
YER120W	SCS2	YHR012W	VPS29
YIL076W	SEC28	YMR216C	SKY1
YDR137W	RGP1	YHR200W	RPN10
YDR318W	MCM21	YDR155C	CPR1
YJR140C	HIR3	YJL138C	TIF2
YHL031C	GOS1	YPL037C	EGD1
YBL008W	HIR1	YLR410W	VIP1
YNL236W	SIN4	YJR060W	CBF1
YBR126C	TPS1	YMR261C	TPS3
YJL053W	PEP8	YMR216C	SKY1

Predictions at threshold level 0.75 continued...			
Gene1 of predicted pair		Gene2 of predicted pair	
Systematic Name	Standard Name	Systematic Name	Standard Name
YKL025C	PAN3	YHR134W	WSS1
YMR246W	FAA4	YMR152W	YIM1
YPL129W	TAF14	YDL005C	MED2
YLR410W	VIP1	YNL116W	DMA2
YNL031C	HHT2	YLR357W	RSC2
YGL161C	YIP5	YGL198W	YIP4
YEL053C	MAK10	YJR060W	CBF1
YJL154C	VPS35	YCL001W	RER1
YHR111W	UBA4	YGL253W	HXK2
YKR001C	VPS1	YDR323C	PEP7
YOR132W	VPS17	YLR447C	VMA6
YLR337C	VRP1	YLR386W	VAC14
YCL037C	SRO9	YOR124C	UBP2
YPR049C	ATG11	YLR423C	ATG17
YKL025C	PAN3	YMR109W	MYO5
YDR293C	SSD1	YER033C	ZRG8
YDR293C	SSD1	YJR060W	CBF1
YAL024C	LTE1	YPR036W	VMA13
YKL048C	ELM1	YPL161C	BEM4
YBR173C	UMP1	YFR036W	CDC26
YDR378C	LSM6	YHR167W	THP2
YHL031C	GOS1	YKR014C	YPT52
YJL176C	SWI3	YBR228W	SLX1
YDL155W	CLB3	YLR370C	ARC18
YLR148W	PEP3	YDL226C	GCS1
YDL223C	HBT1	YBL025W	RRN10
YDR318W	MCM21	YJR090C	GRR1
YLR083C	EMP70	YNL041C	COG6
YBL103C	RTG3	YJL095W	BCK1

Predictions at threshold level 0.75 continued...			
Gene1 of predicted pair		Gene2 of predicted pair	
Systematic Name	Standard Name	Systematic Name	Standard Name
YOR039W	CKB2	YML016C	PPZ1
YHR134W	WSS1	YLR410W	VIP1
YPL022W	RAD1	YMR202W	ERG2
YFR036W	CDC26	YDR076W	RAD55
YOL121C	RPS19A	YBL025W	RRN10
YLR429W	CRN1	YDL155W	CLB3
YDR439W	LRS4	YNL307C	MCK1
YNL016W	PUB1	YJL187C	SWE1
YBR200W	BEM1	YOL091W	SPO21
YBR200W	BEM1	YOR141C	ARP8
YDL223C	HBT1	YNL058C	YNL058C
YBR173C	UMP1	YEL053C	MAK10
YIL034C	CAP2	YOR023C	AHC1
YDR293C	SSD1	YCL001W	RER1
YNL297C	MON2	YDL077C	VAM6
YDR335W	MSN5	YER139C	YER139C
YMR055C	BUB2	YDR469W	SDC1
YLR148W	PEP3	YGR261C	APL6
YMR109W	MYO5	YLR452C	SST2
YOR008C	SLG1	YPL161C	BEM4
YKL139W	CTK1	YEL013W	VAC8
YPR120C	CLB5	YJR035W	RAD26
YMR172W	HOT1	YHR114W	BZZ1
YDR358W	GGA1	YEL022W	GEA2
YPL049C	DIG1	YPL256C	CLN2
YPR084W	YPR084W	YGR020C	VMA7
YLR399C	BDF1	YHR115C	DMA1
YDR245W	MNN10	YOR106W	VAM3
YLR079W	SIC1	YBR260C	RGD1

Predictions at threshold level 0.75 continued...			
Gene1 of predicted pair		Gene2 of predicted pair	
Systematic Name	Standard Name	Systematic Name	Standard Name
YIL148W	RPL40A	YMR199W	CLN1
YDR293C	SSD1	YGL124C	MON1
YDL116W	NUP84	YHR115C	DMA1
YOL018C	TLG2	YDL192W	ARF1
YIL002C	INP51	YHR108W	GGA2
YLR192C	HCR1	YOL121C	RPS19A
YML071C	COG8	YOL018C	TLG2
YBL051C	PIN4	YJR032W	CPR7
YNL297C	MON2	YKR020W	VPS51
YPR173C	VPS4	YKL041W	VPS24
YFR036W	CDC26	YDR358W	GGA1
YMR198W	CIK1	YNL107W	YAF9
YHR013C	ARD1	YPR119W	CLB2
YOR290C	SNF2	YOL121C	RPS19A
YLR150W	STM1	YDR318W	MCM21
YMR198W	CIK1	YML115C	VAN1
YBL058W	SHP1	YKL116C	PRR1
YDR484W	VPS52	YLR371W	ROM2
YNL243W	SLA2	YDR156W	RPA14
YIL053W	RHR2	YDL077C	VAM6
YML062C	MFT1	YKL160W	ELF1
YNL201C	PSY2	YHR134W	WSS1
YMR142C	RPL13B	YPL037C	EGD1
YDR363W-A	SEM1	YGL031C	RPL24A
YLR385C	SWC7	YJR082C	EAF6
YLR240W	VPS34	YPL045W	VPS16
YNL166C	BNI5	YBR107C	IML3
YCR066W	RAD18	YPL037C	EGD1
YGL178W	MPT5	YGL094C	PAN2

Predictions at threshold level 0.75 continued...			
Gene1 of predicted pair		Gene2 of predicted pair	
Systematic Name	Standard Name	Systematic Name	Standard Name
YGR054W	YGR054W	YGL151W	NUT1
YML103C	NUP188	YFR036W	CDC26
YKR035W-A	DID2	YMR133W	REC114
YDL056W	MBP1	YGL003C	CDH1
YML115C	VAN1	YDR310C	SUM1
YOR001W	RRP6	YDR254W	CHL4
YPR173C	VPS4	YDL192W	ARF1
YDR155C	CPR1	YAL002W	VPS8
YBL021C	HAP3	YCR086W	CSM1
YML095C	RAD10	YJR005W	APL1
YAL024C	LTE1	YDR174W	HMO1
YMR153W	NUP53	YLR371W	ROM2
YPL037C	EGD1	YOL108C	INO4
YHR156C	LIN1	YMR133W	REC114
YIL159W	BNR1	YDR207C	UME6
YOR043W	WHI2	YJR135C	MCM22
YEL050C	RML2	YPR046W	MCM16
YIL159W	BNR1	YNL041C	COG6
YHR060W	VMA22	YPL137C	GIP3
YIL038C	NOT3	YGR134W	CAF130
YMR199W	CLN1	YPL253C	VIK1
YPL115C	BEM3	YAL002W	VPS8
YOR290C	SNF2	YGL124C	MON1
YDL020C	RPN4	YOR191W	RIS1
YOR080W	DIA2	YOL091W	SPO21
YJL044C	GYP6	YDR456W	NHX1
YHL020C	OPI1	YJR060W	CBF1
YOR270C	VPH1	YBL025W	RRN10
YNL044W	YIP3	YNL051W	COG5

Predictions at threshold level 0.75 continued...			
Gene1 of predicted pair		Gene2 of predicted pair	
Systematic Name	Standard Name	Systematic Name	Standard Name
YLR090W	XDJ1	YLR371W	ROM2
YPR084W	YPR084W	YMR216C	SKY1
YDR443C	SSN2	YPR072W	NOT5
YKR101W	SIR1	YNL030W	HHF2
YOR195W	SLK19	YLR399C	BDF1
YBR194W	SOY1	YBR107C	IML3
YOL111C	MDY2	YNL041C	COG6
YBL027W	RPL19B	YGL031C	RPL24A
YFL047W	RGD2	YKL068W	NUP100
YJR060W	CBF1	YOR141C	ARP8
YNL264C	PDR17	YNL079C	TPM1
YER116C	SLX8	YHR039C-A	VMA10
YDL161W	ENT1	YFL026W	STE2
YNL229C	URE2	YGL031C	RPL24A
YDR004W	RAD57	YMR038C	CCS1
YKR095W	MLP1	YGL163C	RAD54
YOL121C	RPS19A	YOR327C	SNC2
YPL042C	SSN3	YGR270W	YTA7
YHR111W	UBA4	YDL047W	SIT4
YDR004W	RAD57	YHR010W	RPL27A
YHR060W	VMA22	YER044C	ERG28
YHR084W	STE12	YBR083W	TEC1
YLR083C	EMP70	YKR001C	VPS1
YLR170C	APS1	YDR358W	GGA1
YNL117W	MLS1	YKL101W	HSL1
YDL002C	NHP10	YDR137W	RGP1
YBR278W	DPB3	YBR195C	MSI1
YML001W	YPT7	YBR019C	GAL10
YPL137C	GIP3	YER040W	GLN3

Predictions at threshold level 0.75 continued...			
Gene1 of predicted pair		Gene2 of predicted pair	
Systematic Name	Standard Name	Systematic Name	Standard Name
YNL094W	APP1	YLR144C	ACF2
YBR171W	SEC66	YHL011C	PRS3
YGL033W	HOP2	YER144C	UBP5
YPL161C	BEM4	YPL137C	GIP3
YMR026C	PEX12	YGL212W	VAM7
YNR058W	BIO3	YML059C	NTE1
YNL215W	IES2	YOR368W	RAD17
YPL115C	BEM3	YNL288W	CAF40
YJL136C	RPS21B	YKL048C	ELM1
YDR146C	SWI5	YDR245W	MNN10
YGL175C	SAE2	YDR076W	RAD55
YGL066W	SGF73	YGR184C	UBR1
YHR039C-A	VMA10	YMR311C	GLC8
YPL161C	BEM4	YNR032W	PPG1
YER087C-B	SBH1	YNR049C	MSO1
YDL020C	RPN4	YOL067C	RTG1
YFR031C-A	RPL2A	YPL137C	GIP3
YLR061W	RPL22A	YML071C	COG8
YMR164C	MSS11	YER109C	FLO8
YNL058C	YNL058C	YNL041C	COG6
YPL256C	CLN2	YER049W	TPA1
YJL127C	SPT10	YMR202W	ERG2
YMR172W	HOT1	YJR032W	CPR7
YBR108W	YBR108W	YCR086W	CSM1
YLR452C	SST2	YOR066W	MSA1
YHR013C	ARD1	YOL081W	IRA2
YDR439W	LRS4	YHR115C	DMA1
YNL201C	PSY2	YDR335W	MSN5
YAL024C	LTE1	YJL124C	LSM1

Predictions at threshold level 0.75 continued...			
Gene1 of predicted pair		Gene2 of predicted pair	
Systematic Name	Standard Name	Systematic Name	Standard Name
YMR153W	NUP53	YOR270C	VPH1
YFL003C	MSH4	YOR358W	HAP5
YBR217W	ATG12	YHR171W	ATG7
YNR052C	POP2	YOR080W	DIA2
YCR020C-A	MAK31	YEL043W	YEL043W
YEL050C	RML2	YLR381W	CTF3
YNL243W	SLA2	YLR234W	TOP3
YPL161C	BEM4	YDR261C	EXG2
YML016C	PPZ1	YNL116W	DMA2
YMR153W	NUP53	YJL183W	MNN11
YOL126C	MDH2	YIL148W	RPL40A
YIL053W	RHR2	YMR026C	PEX12
YDL184C	RPL41A	YKL048C	ELM1
YFR030W	MET10	YNL107W	YAF9
YKR035W-A	DID2	YHR039C-A	VMA10
YGR072W	UPF3	YGL054C	ERV14
YOR140W	SFL1	YHR205W	SCH9
YDR129C	SAC6	YGL212W	VAM7
YDR261C	EXG2	YML071C	COG8
YLR090W	XDJ1	YDR443C	SSN2
YPL137C	GIP3	YEL060C	PRB1
YNL206C	RTT106	YDL047W	SIT4
YMR116C	ASC1	YOR312C	RPL20B
YDR383C	NKP1	YDR318W	MCM21
YNL069C	RPL16B	YGL246C	RAI1
YKL073W	LHS1	YJL159W	HSP150
YDR320C	SWA2	YDR108W	GSG1
YHL031C	GOS1	YLR350W	ORM2
YPR036W	VMA13	YPL057C	SUR1

Predictions at threshold level 0.75 continued...			
Gene1 of predicted pair		Gene2 of predicted pair	
Systematic Name	Standard Name	Systematic Name	Standard Name
YML097C	VPS9	YBR082C	UBC4
YOR290C	SNF2	YMR230W	RPS10B
YML016C	PPZ1	YML112W	CTK3
YPL161C	BEM4	YOR043W	WHI2
YHR119W	SET1	YAR003W	SWD1
YFL026W	STE2	YJR086W	STE18
YHR098C	SFB3	YCR053W	THR4
YDR477W	SNF1	YGL208W	SIP2
YGL174W	BUD13	YDR137W	RGP1
YMR004W	MVP1	YCL001W	RER1
YJL176C	SWI3	YPL213W	LEA1
YLR432W	IMD3	YHR216W	IMD2
YDR245W	MNN10	YJL117W	PHO86
YOR066W	MSA1	YNL238W	KEX2
YOR132W	VPS17	YKL048C	ELM1
YKR072C	SIS2	YMR199W	CLN1
YDR443C	SSN2	YPL248C	GAL4
YNL041C	COG6	YLR371W	ROM2
YOR327C	SNC2	YJL095W	BCK1
YJL124C	LSM1	YBR107C	IML3
YNL201C	PSY2	YHR013C	ARD1
YMR198W	CIK1	YPR046W	MCM16
YLR234W	TOP3	YPR119W	CLB2
YLR083C	EMP70	YDR108W	GSG1
YKR072C	SIS2	YDR289C	RTT103
YDR484W	VPS52	YGL095C	VPS45
YPL234C	TFP3	YNL127W	FAR11
YMR216C	SKY1	YML038C	YMD8
YGL025C	PGD1	YDR310C	SUM1

Predictions at threshold level 0.75 continued...			
Gene1 of predicted pair		Gene2 of predicted pair	
Systematic Name	Standard Name	Systematic Name	Standard Name
YLR386W	VAC14	YDL047W	SIT4
YGL153W	PEX14	YNL214W	PEX17
YOR270C	VPH1	YDR358W	GGA1
YGL003C	CDH1	YDR200C	VPS64
YPL042C	SSN3	YCR084C	TUP1
YNL087W	TCB2	YKL048C	ELM1
YBR217W	ATG12	YOL111C	MDY2
YBL008W	HIR1	YGL163C	RAD54
YEL053C	MAK10	YDR318W	MCM21
YBL008W	HIR1	YOR039W	CKB2
YDR156W	RPA14	YDR378C	LSM6
YPL161C	BEM4	YDR153C	ENT5
YBR030W	YBR030W	YNL325C	FIG4
YLR381W	CTF3	YDL155W	CLB3
YDR260C	SWM1	YPR046W	MCM16
YNL248C	RPA49	YHR115C	DMA1
YJL154C	VPS35	YHR178W	STB5
YOR127W	RGA1	YPL105C	YPL105C
YOR107W	RGS2	YJL106W	IME2
YNL106C	INP52	YNL107W	YAF9
YOR290C	SNF2	YER162C	RAD4
YNL166C	BNI5	YOL067C	RTG1
YAL010C	MDM10	YNL116W	DMA2
YKL048C	ELM1	YAL002W	VPS8
YOR332W	VMA4	YKR001C	VPS1
YDR103W	STE5	YPR046W	MCM16
YFR010W	UBP6	YJR056C	YJR056C
YFR031C-A	RPL2A	YER139C	YER139C
YKL110C	KTI12	YLR315W	NKP2

Predictions at threshold level 0.75 continued...			
Gene1 of predicted pair		Gene2 of predicted pair	
Systematic Name	Standard Name	Systematic Name	Standard Name
YMR004W	MVP1	YDL006W	PTC1
YJR063W	RPA12	YFR030W	MET10
YPR030W	CSR2	YLR093C	NYV1
YMR039C	SUB1	YER161C	SPT2
YOR101W	RAS1	YGR188C	BUB1
YGL133W	ITC1	YBR200W	BEM1
YEL050C	RML2	YML016C	PPZ1
YHR200W	RPN10	YLR398C	SKI2
YIL076W	SEC28	YJL183W	MNN11
YMR216C	SKY1	YKR030W	GMH1
YBR215W	HPC2	YER161C	SPT2
YHR081W	LRP1	YEL060C	PRB1
YGL223C	COG1	YNL297C	MON2
YMR164C	MSS11	YOR358W	HAP5
YLR447C	VMA6	YHR060W	VMA22
YOR132W	VPS17	YMR127C	SAS2
YNL291C	MID1	YOR112W	CEX1
YML103C	NUP188	YPL253C	VIK1
YPR030W	CSR2	YER122C	GLO3
YDR108W	GSG1	YOR106W	VAM3
YNL044W	YIP3	YDL077C	VAM6
YNL153C	GIM3	YGL190C	CDC55
YBR171W	SEC66	YML067C	ERV41
YKL135C	APL2	YHR161C	YAP1801
YBL025W	RRN10	YNL076W	MKS1
YPL037C	EGD1	YEL051W	VMA8
YDL116W	NUP84	YOR001W	RRP6
YEL065W	SIT1	YNL087W	TCB2
YPL253C	VIK1	YOL091W	SPO21

Predictions at threshold level 0.75 continued...			
Gene1 of predicted pair		Gene2 of predicted pair	
Systematic Name	Standard Name	Systematic Name	Standard Name
YDL223C	HBT1	YMR133W	REC114
YHR152W	SPO12	YBL087C	RPL23A
YMR172W	HOT1	YHR134W	WSS1
YOR076C	SKI7	YHR081W	LRP1
YGR129W	SYF2	YBR188C	NTC20
YLR371W	ROM2	YBR082C	UBC4
YJL058C	BIT61	YDR171W	HSP42
YDL020C	RPN4	YHR134W	WSS1
YDR225W	HTA1	YNL307C	MCK1
YCL037C	SRO9	YJR040W	GEF1
YFR032C-A	RPL29	YMR116C	ASC1
YMR311C	GLC8	YEL036C	ANP1
YHR156C	LIN1	YDR261C	EXG2
YPL195W	APL5	YJL095W	BCK1
YEL065W	SIT1	YER044C	ERG28
YJR063W	RPA12	YIR005W	IST3
YDR335W	MSN5	YFR034C	PHO4
YPL161C	BEM4	YDR218C	SPR28
YLR320W	MMS22	YDL083C	RPS16B
YGR020C	VMA7	YKR001C	VPS1
YLR079W	SIC1	YEL036C	ANP1
YMR153W	NUP53	YER044C	ERG28
YBL079W	NUP170	YLR410W	VIP1
YPR046W	MCM16	YOL054W	PSH1
YFR010W	UBP6	YER122C	GLO3
YGL060W	YBP2	YEL050C	RML2
YGL025C	PGD1	YLR093C	NYV1
YBR084C-A	RPL19A	YKR048C	NAP1
YDL161W	ENT1	YKR001C	VPS1

Predictions at threshold level 0.75 continued...			
Gene1 of predicted pair		Gene2 of predicted pair	
Systematic Name	Standard Name	Systematic Name	Standard Name
YKR035W-A	DID2	YDR358W	GGA1
YDR507C	GIN4	YKL101W	HSL1
YBR258C	SHG1	YDR469W	SDC1
YAL024C	LTE1	YDR260C	SWM1
YLR119W	SRN2	YOR270C	VPH1
YDR254W	CHL4	YNL107W	YAF9
YNL068C	FKH2	YBL047C	EDE1
YLR320W	MMS22	YGL124C	MON1
YBR239C	YBR239C	YPL246C	RBD2
YJL172W	CPS1	YEL053C	MAK10
YFL023W	BUD27	YHR060W	VMA22
YDL160C	DHH1	YER129W	SAK1
YJL080C	SCP160	YPR119W	CLB2
YCR084C	TUP1	YBR089C-A	NHP6B
YLR337C	VRP1	YLR360W	VPS38
YOR033C	EXO1	YFL023W	BUD27
YHL002W	HSE1	YNR006W	VPS27
YLR441C	RPS1A	YDL077C	VAM6
YLR452C	SST2	YHR081W	LRP1
YNL106C	INP52	YPR173C	VPS4
YOR193W	PEX27	YMR204C	INP1
YLR095C	IOC2	YBR217W	ATG12
YGL212W	VAM7	YJR125C	ENT3
YNL199C	GCR2	YKL213C	DOA1
YLR083C	EMP70	YAL002W	VPS8
YGL100W	SEH1	YMR167W	MLH1
YJL053W	PEP8	YCR088W	ABP1
YDR183W	PLP1	YGL253W	HXK2
YNL025C	SSN8	YGL174W	BUD13

Predictions at threshold level 0.75 continued...			
Gene1 of predicted pair		Gene2 of predicted pair	
Systematic Name	Standard Name	Systematic Name	Standard Name
YKL126W	YPK1	YMR214W	SCJ1
YDL116W	NUP84	YNL225C	CNM67
YDR480W	DIG2	YMR199W	CLN1
YKL007W	CAP1	YJL095W	BCK1
YGR108W	CLB1	YBR108W	YBR108W
YBR127C	VMA2	YHR039C-A	VMA10
YOR023C	AHC1	YOL067C	RTG1
YNL166C	BNI5	YHR111W	UBA4
YKL048C	ELM1	YER040W	GLN3
YDL020C	RPN4	YPL256C	CLN2
YGL066W	SGF73	YOR127W	RGA1
YGL151W	NUT1	YEL056W	HAT2
YHL031C	GOS1	YHR039C-A	VMA10
YER161C	SPT2	YGL151W	NUT1
YNL223W	ATG4	YNR007C	ATG3
YLR381W	CTF3	YLR233C	EST1
YMR004W	MVP1	YEL053C	MAK10
YLL039C	UBI4	YPL096C-A	ERI1
YBR108W	YBR108W	YOL067C	RTG1
YPL096C-A	ERI1	YER001W	MNN1
YMR199W	CLN1	YNL199C	GCR2
YKL139W	CTK1	YML112W	CTK3
YCL027W	FUS1	YMR109W	MYO5
YKR101W	SIR1	YOR038C	HIR2
YPR017C	DSS4	YAL030W	SNC1
YEL065W	SIT1	YLR093C	NYV1
YMR224C	MRE11	YPR120C	CLB5
YNL215W	IES2	YGL174W	BUD13
YJL124C	LSM1	YNL107W	YAF9

Predictions at threshold level 0.75 continued...			
Gene1 of predicted pair		Gene2 of predicted pair	
Systematic Name	Standard Name	Systematic Name	Standard Name
YOR324C	FRT1	YML057W	CMP2
YEL065W	SIT1	YKL048C	ELM1
YLR381W	CTF3	YCR086W	CSM1
YML038C	YMD8	YJR060W	CBF1
YMR054W	STV1	YDL192W	ARF1
YHL002W	HSE1	YAL002W	VPS8
YBL106C	SRO77	YNR049C	MSO1
YGL147C	RPL9A	YER040W	GLN3
YLR386W	VAC14	YML112W	CTK3
YDR443C	SSN2	YNL325C	FIG4
YDR363W-A	SEM1	YPL152W	RRD2
YPR131C	NAT3	YOR368W	RAD17
YER143W	DDI1	YMR017W	SPO20
YBL079W	NUP170	YBR082C	UBC4
YNL094W	APP1	YJL187C	SWE1
YMR284W	YKU70	YDL047W	SIT4
YPR030W	CSR2	YBR036C	CSG2
YNL106C	INP52	YFR010W	UBP6
YLR095C	IOC2	YER162C	RAD4
YEL050C	RML2	YJL124C	LSM1
YBL047C	EDE1	YGR252W	GCN5
YMR039C	SUB1	YBR274W	CHK1
YMR127C	SAS2	YGL151W	NUT1
YHR200W	RPN10	YOR066W	MSA1
YNL307C	MCK1	YML001W	YPT7
YBR200W	BEM1	YGL031C	RPL24A
YIR002C	MPH1	YLR320W	MMS22
YER116C	SLX8	YGR184C	UBR1
YOL030W	GAS5	YGL031C	RPL24A

Predictions at threshold level 0.75 continued...			
Gene1 of predicted pair		Gene2 of predicted pair	
Systematic Name	Standard Name	Systematic Name	Standard Name
YGL212W	VAM7	YBR280C	SAF1
YLR452C	SST2	YEL060C	PRB1
YFR032C-A	RPL29	YHR200W	RPN10
YLR090W	XDJ1	YDL192W	ARF1
YKL126W	YPK1	YBL072C	RPS8A
YOR195W	SLK19	YML028W	TSA1
YDR254W	CHL4	YHL002W	HSE1
YBR041W	FAT1	YLR371W	ROM2
YGL153W	PEX14	YPL161C	BEM4
YBL008W	HIR1	YDR073W	SNF11
YKR084C	HBS1	YJL191W	RPS14B
YMR152W	YIM1	YKL140W	TGL1
YOR109W	INP53	YJR040W	GEF1
YGL174W	BUD13	YKL213C	DOA1
YPL256C	CLN2	YDR076W	RAD55
YJR063W	RPA12	YJL006C	CTK2
YKL126W	YPK1	YMR152W	YIM1
YDR137W	RGP1	YHR039C-A	VMA10
YGR135W	PRE9	YLR357W	RSC2
YLR337C	VRP1	YDR144C	MKC7
YLR093C	NYV1	YNL076W	MKS1
YML038C	YMD8	YGL115W	SNF4
YCR034W	FEN1	YJR140C	HIR3
YLR119W	SRN2	YGL025C	PGD1
YCR086W	CSM1	YBR082C	UBC4
YPL050C	MNN9	YOR327C	SNC2
YBR042C	CST26	YLR131C	ACE2
YLR192C	HCR1	YBR217W	ATG12
YKL101W	HSL1	YLR350W	ORM2

Predictions at threshold level 0.75 continued...			
Gene1 of predicted pair		Gene2 of predicted pair	
Systematic Name	Standard Name	Systematic Name	Standard Name
YDL223C	HBT1	YMR230W	RPS10B
YMR152W	YIM1	YBR036C	CSG2
YIL148W	RPL40A	YKR024C	DBP7
YCR066W	RAD18	YMR054W	STV1
YKL139W	CTK1	YDR207C	UME6
YNL325C	FIG4	YDR310C	SUM1
YGL100W	SEH1	YOL090W	MSH2
YIL036W	CST6	YPL018W	CTF19
YDR146C	SWI5	YDR289C	RTT103
YGL078C	DBP3	YNL238W	KEX2
YMR095C	SNO1	YPL024W	RMI1
YBL008W	HIR1	YGL019W	CKB1
YPL137C	GIP3	YML071C	COG8
YBR228W	SLX1	YOL090W	MSH2
YPL234C	TFP3	YDL006W	PTC1
YLR381W	CTF3	YDL013W	SLX5
YKR069W	MET1	YMR109W	MYO5
YJL154C	VPS35	YGL115W	SNF4
YIL159W	BNR1	YGL025C	PGD1
YPL042C	SSN3	YGL174W	BUD13
YDR358W	GGA1	YEL060C	PRB1
YMR016C	SOK2	YER122C	GLO3
YLR337C	VRP1	YAL002W	VPS8
YOR032C	HMS1	YPR046W	MCM16
YLR192C	HCR1	YPR030W	CSR2
YML028W	TSA1	YDR207C	UME6
YER107C	GLE2	YDR334W	SWR1
YHR039C-A	VMA10	YML071C	COG8
YHR013C	ARD1	YLR254C	NDL1

Predictions at threshold level 0.75 continued...			
Gene1 of predicted pair		Gene2 of predicted pair	
Systematic Name	Standard Name	Systematic Name	Standard Name
YGR129W	SYF2	YIR005W	IST3
YDR318W	MCM21	YGL253W	HXK2
YGL033W	HOP2	YHR060W	VMA22
YDR443C	SSN2	YOR327C	SNC2
YDR254W	CHL4	YDR076W	RAD55
YNL215W	IES2	YKR061W	KTR2
YPR017C	DSS4	YJL036W	SNX4
YOR001W	RRP6	YLR410W	VIP1
YMR095C	SNO1	YCR053W	THR4
YNL107W	YAF9	YKR030W	GMH1
YLR090W	XDJ1	YBR173C	UMP1
YBR200W	BEM1	YPL152W	RRD2
YDR129C	SAC6	YGL200C	EMP24
YMR202W	ERG2	YBR215W	HPC2
YPR036W	VMA13	YNL307C	MCK1
YIL076W	SEC28	YJL004C	SYS1
YKL139W	CTK1	YDR289C	RTT103
YGL237C	HAP2	YDR469W	SDC1
YJL204C	RCY1	YDR131C	YDR131C
YDR202C	RAV2	YDR153C	ENT5
YDR245W	MNN10	YOR035C	SHE4
YHL031C	GOS1	YEL043W	YEL043W
YFR010W	UBP6	YLR452C	SST2
YDR383C	NKP1	YJL124C	LSM1
YGL033W	HOP2	YML001W	YPT7
YPL050C	MNN9	YML067C	ERV41
YDL013W	SLX5	YHR200W	RPN10
YPR017C	DSS4	YPL037C	EGD1
YPL161C	BEM4	YBR114W	RAD16

Predictions at threshold level 0.75 continued...			
Gene1 of predicted pair		Gene2 of predicted pair	
Systematic Name	Standard Name	Systematic Name	Standard Name
YPL234C	TFP3	YJL004C	SYS1
YLR292C	SEC72	YNL166C	BNI5
YLR410W	VIP1	YGR188C	BUB1
YLR102C	APC9	YKR057W	RPS21A
YPL057C	SUR1	YBR280C	SAF1
YNL325C	FIG4	YDR358W	GGA1
YGL033W	HOP2	YPR046W	MCM16
YAL024C	LTE1	YHR156C	LIN1
YGR249W	MGA1	YER044C	ERG28
YJR056C	YJR056C	YLR398C	SKI2
YJL124C	LSM1	YBR195C	MSI1
YOR080W	DIA2	YDR335W	MSN5
YML115C	VAN1	YKL101W	HSL1
YMR198W	CIK1	YLR429W	CRN1
YDR156W	RPA14	YHR111W	UBA4
YMR133W	REC114	YMR230W	RPS10B
YIL036W	CST6	YPL180W	TCO89
YLR192C	HCR1	YNL058C	YNL058C
YJL183W	MNN11	YDL192W	ARF1
YDR146C	SWI5	YKL161C	YKL161C
YDR137W	RGP1	YGL115W	SNF4
YLR441C	RPS1A	YJR084W	CSN12
YLR083C	EMP70	YHL031C	GOS1
YML103C	NUP188	YML016C	PPZ1
YIL148W	RPL40A	YML057W	CMP2
YLR360W	VPS38	YJL036W	SNX4
YOR039W	CKB2	YDR310C	SUM1
YLR079W	SIC1	YMR055C	BUB2
YNL054W	VAC7	YFR036W	CDC26

Predictions at threshold level 0.75 continued...			
Gene1 of predicted pair		Gene2 of predicted pair	
Systematic Name	Standard Name	Systematic Name	Standard Name
YIR005W	IST3	YAL002W	VPS8
YLR192C	HCR1	YOR043W	WHI2
YPL253C	VIK1	YDR310C	SUM1
YDR335W	MSN5	YER122C	GLO3
YEL022W	GEA2	YDL137W	ARF2
YHR156C	LIN1	YKR099W	BAS1
YGR020C	VMA7	YPL037C	EGD1
YMR199W	CLN1	YFR040W	SAP155
YJR060W	CBF1	YAR014C	BUD14
YBR108W	YBR108W	YOR358W	HAP5
YNL051W	COG5	YOL108C	INO4
YLR079W	SIC1	YDR310C	SUM1
YFR031C-A	RPL2A	YBR126C	TPS1
YMR199W	CLN1	YGL016W	KAP122
YPL129W	TAF14	YOR064C	YNG1
YLR095C	IOC2	YGL033W	HOP2
YDR443C	SSN2	YNL206C	RTT106
YER087C-B	SBH1	YPR141C	KAR3
YOR156C	NFI1	YPL161C	BEM4
YKL139W	CTK1	YMR152W	YIM1
YDL077C	VAM6	YCR053W	THR4
YDL184C	RPL41A	YGL115W	SNF4
YNL106C	INP52	YAR014C	BUD14
YMR198W	CIK1	YNL307C	MCK1
YDL020C	RPN4	YHR060W	VMA22
YGL223C	COG1	YBR171W	SEC66
YDR443C	SSN2	YPL203W	TPK2
YGR019W	UGA1	YJR032W	CPR7
YKL048C	ELM1	YBR280C	SAF1

Predictions at threshold level 0.75 continued...			
Gene1 of predicted pair		Gene2 of predicted pair	
Systematic Name	Standard Name	Systematic Name	Standard Name
YLR386W	VAC14	YGL124C	MON1
YMR055C	BUB2	YEL056W	HAT2
YLR421C	RPN13	YGR232W	NAS6
YDL116W	NUP84	YJL187C	SWE1
YJR063W	RPA12	YGL252C	RTG2
YDR477W	SNF1	YBR036C	CSG2
YJL136C	RPS21B	YNL229C	URE2
YCR086W	CSM1	YML028W	TSA1
YPL234C	TFP3	YNR032W	PPG1
YOL067C	RTG1	YGR188C	BUB1
YKR020W	VPS51	YJR084W	CSN12
YAL024C	LTE1	YIL036W	CST6
YLR452C	SST2	YMR311C	GLC8
YKL010C	UFD4	YJL006C	CTK2
YMR152W	YIM1	YDR207C	UME6
YCR086W	CSM1	YOR141C	ARP8
YDR293C	SSD1	YKL074C	MUD2
YMR142C	RPL13B	YER144C	UBP5
YJL138C	TIF2	YJR032W	CPR7
YBR171W	SEC66	YNL079C	TPM1
YIL053W	RHR2	YOL121C	RPS19A
YBR041W	FAT1	YGR192C	TDH3
YGL212W	VAM7	YNL107W	YAF9
YER110C	KAP123	YPL178W	CBC2
YKR059W	TIF1	YBR274W	CHK1
YNL201C	PSY2	YDR318W	MCM21
YLR234W	TOP3	YOR358W	HAP5
YGL124C	MON1	YMR214W	SCJ1
YBR200W	BEM1	YCR053W	THR4

Predictions at threshold level 0.75 continued...			
Gene1 of predicted pair		Gene2 of predicted pair	
Systematic Name	Standard Name	Systematic Name	Standard Name
YDR261C	EXG2	YMR311C	GLC8
YMR216C	SKY1	YER120W	SCS2
YNL291C	MID1	YDR076W	RAD55
YNL325C	FIG4	YFL023W	BUD27
YOR127W	RGA1	YNL166C	BNI5
YGL163C	RAD54	YDL047W	SIT4
YNL297C	MON2	YHR012W	VPS29
YJL006C	CTK2	YJR135C	MCM22
YLR320W	MMS22	YML001W	YPT7
YDR289C	RTT103	YGL252C	RTG2
YDR192C	NUP42	YNL049C	SFB2
YGL025C	PGD1	YPL195W	APL5
YML115C	VAN1	YNL041C	COG6
YPL253C	VIK1	YKL110C	KTI12
YHR216W	IMD2	YML056C	IMD4
YNL238W	KEX2	YOL081W	IRA2
YDL161W	ENT1	YDR358W	GGA1
YGR081C	SLX9	YML028W	TSA1
YMR095C	SNO1	YGL124C	MON1
YNL025C	SSN8	YNL215W	IES2
YGR071C	YGR071C	YBR036C	CSG2
YBR200W	BEM1	YML109W	ZDS2
YMR284W	YKU70	YHR056C	RSC30
YNL031C	HHT2	YLR182W	SWI6
YPL024W	RMI1	YBR274W	CHK1
YHR013C	ARD1	YJR063W	RPA12
YPL024W	RMI1	YIL076W	SEC28
YGR072W	UPF3	YML038C	YMD8
YHL011C	PRS3	YPR119W	CLB2

Predictions at threshold level 0.75 continued...			
Gene1 of predicted pair		Gene2 of predicted pair	
Systematic Name	Standard Name	Systematic Name	Standard Name
YOR106W	VAM3	YDR137W	RGP1
YJR032W	CPR7	YKL119C	VPH2
YDR439W	LRS4	YNL248C	RPA49
YML115C	VAN1	YNL087W	TCB2
YBR030W	YBR030W	YGL025C	PGD1
YOR066W	MSA1	YOL121C	RPS19A
YDL161W	ENT1	YBL047C	EDE1
YGL019W	CKB1	YDR310C	SUM1
YAL024C	LTE1	YOR213C	SAS5
YOR327C	SNC2	YAL002W	VPS8
YPR018W	RLF2	YJR090C	GRR1
YDR320C	SWA2	YDR080W	VPS41
YJR040W	GEF1	YKR030W	GMH1
YOR308C	SNU66	YEL013W	VAC8
YNL058C	YNL058C	YCR053W	THR4
YIL053W	RHR2	YER162C	RAD4
YNL215W	IES2	YNL307C	MCK1
YBL019W	APN2	YDL047W	SIT4
YLR090W	XDJ1	YJL095W	BCK1
YLR006C	SSK1	YBR200W	BEM1
YBR108W	YBR108W	YBR171W	SEC66
YDR507C	GIN4	YGL190C	CDC55
YHR119W	SET1	YJL168C	SET2
YBR042C	CST26	YDR385W	EFT2
YOR332W	VMA4	YLR447C	VMA6
YOR023C	AHC1	YNL107W	YAF9
YDR443C	SSN2	YNL087W	TCB2
YMR198W	CIK1	YKR014C	YPT52
YML016C	PPZ1	YHL011C	PRS3

Predictions at threshold level 0.75 continued...			
Gene1 of predicted pair		Gene2 of predicted pair	
Systematic Name	Standard Name	Systematic Name	Standard Name
YHL020C	OPI1	YPL120W	VPS30
YLR371W	ROM2	YJL183W	MNN11
YDR479C	PEX29	YBR168W	PEX32
YDR293C	SSD1	YGL054C	ERV14
YLR083C	EMP70	YDR207C	UME6
YDR320C	SWA2	YDR153C	ENT5
YGL153W	PEX14	YGR135W	PRE9
YMR224C	MRE11	YBR082C	UBC4
YLR090W	XDJ1	YGR249W	MGA1
YFR036W	CDC26	YDR207C	UME6
YDR174W	HMO1	YPL152W	RRD2
YLR441C	RPS1A	YFR036W	CDC26
YNL041C	COG6	YCR053W	THR4
YJR063W	RPA12	YGR188C	BUB1
YHL031C	GOS1	YBR171W	SEC66
YGR261C	APL6	YDL077C	VAM6
YOR083W	WHI5	YPL106C	SSE1
YBR173C	UMP1	YHR200W	RPN10
YOR275C	RIM20	YJL183W	MNN11
YGR249W	MGA1	YNL076W	MKS1
YCR077C	PAT1	YJL183W	MNN11
YJL124C	LSM1	YJR035W	RAD26
YMR095C	SNO1	YBR280C	SAF1
YOR337W	TEA1	YER044C	ERG28
YNL215W	IES2	YDR183W	PLP1
YKR001C	VPS1	YOR036W	PEP12
YLR396C	VPS33	YAL030W	SNC1
YLR095C	IOC2	YLR320W	MMS22
YMR152W	YIM1	YDR310C	SUM1

Predictions at threshold level 0.75 continued...			
Gene1 of predicted pair		Gene2 of predicted pair	
Systematic Name	Standard Name	Systematic Name	Standard Name
YMR080C	NAM7	YGR072W	UPF3
YPL018W	CTF19	YDR183W	PLP1
YLL007C	YLL007C	YLR360W	VPS38
YLR090W	XDJ1	YMR026C	PEX12
YMR201C	RAD14	YBR114W	RAD16
YLR452C	SST2	YOL018C	TLG2
YPL161C	BEM4	YOL081W	IRA2
YGR054W	YGR054W	YDR183W	PLP1
YLR381W	CTF3	YIL008W	URM1
YJL190C	RPS22A	YHR114W	BZZ1
YOL111C	MDY2	YPR030W	CSR2
YNL297C	MON2	YDR153C	ENT5
YBL101C	ECM21	YJL095W	BCK1
YLR337C	VRP1	YJR090C	GRR1
YOL126C	MDH2	YMR153W	NUP53
YGR249W	MGA1	YOR290C	SNF2
YPR131C	NAT3	YPL256C	CLN2
YFR032C-A	RPL29	YKR020W	VPS51
YDL059C	RAD59	YOR368W	RAD17
YOR047C	STD1	YDL194W	SNF3
YHR134W	WSS1	YPR046W	MCM16
YPL161C	BEM4	YJL095W	BCK1
YDR129C	SAC6	YFR036W	CDC26
YER120W	SCS2	YOL108C	INO4
YGL175C	SAE2	YMR038C	CCS1
YOL051W	GAL11	YLR182W	SWI6
YMR231W	PEP5	YAL002W	VPS8
YFR010W	UBP6	YNL307C	MCK1
YDR293C	SSD1	YLR447C	VMA6

Predictions at threshold level 0.75 continued...			
Gene1 of predicted pair		Gene2 of predicted pair	
Systematic Name	Standard Name	Systematic Name	Standard Name
YER139C	YER139C	YMR214W	SCJ1
YGR003W	CUL3	YDR139C	RUB1
YOR127W	RGA1	YMR004W	MVP1
YER044C	ERG28	YDR310C	SUM1
YGL147C	RPL9A	YMR153W	NUP53
YDR439W	LRS4	YFR030W	MET10
YMR164C	MSS11	YCR024C-A	PMP1
YLR233C	EST1	YDR310C	SUM1
YPL057C	SUR1	YKL213C	DOA1
YLL007C	YLL007C	YPR051W	MAK3
YDR146C	SWI5	YOR043W	WHI2
YMR216C	SKY1	YGR006W	PRP18
YOR371C	GPB1	YPR119W	CLB2
YHR152W	SPO12	YPR111W	DBF20
YDL101C	DUN1	YBR228W	SLX1
YNL041C	COG6	YLR093C	NYV1
YFL013C	IES1	YOR308C	SNU66
YHR111W	UBA4	YJL187C	SWE1
YOR106W	VAM3	YEL043W	YEL043W
YNL069C	RPL16B	YOR109W	INP53
YPR131C	NAT3	YDR076W	RAD55
YDR443C	SSN2	YLR360W	VPS38
YIL079C	AIR1	YGR152C	RSR1
YML109W	ZDS2	YNL293W	MSB3
YGL133W	ITC1	YOR195W	SLK19
YBR171W	SEC66	YGL115W	SNF4
YOR290C	SNF2	YLR452C	SST2
YOL051W	GAL11	YDL005C	MED2
YPR131C	NAT3	YBL079W	NUP170

Predictions at threshold level 0.75 continued...			
Gene1 of predicted pair		Gene2 of predicted pair	
Systematic Name	Standard Name	Systematic Name	Standard Name
YDR129C	SAC6	YML097C	VPS9
YPR036W	VMA13	YOR327C	SNC2
YBL051C	PIN4	YDR310C	SUM1
YDR137W	RGP1	YDR378C	LSM6
YDL056W	MBP1	YLR182W	SWI6
YER042W	MXR1	YLR093C	NYV1
YDR443C	SSN2	YDR261C	EXG2
YIL103W	DPH1	YDR289C	RTT103
YKR046C	PET10	YMR313C	TGL3
YPL240C	HSP82	YDR026C	YDR026C
YGR019W	UGA1	YBR107C	IML3
YCR066W	RAD18	YEL051W	VMA8
YNL166C	BNI5	YGR054W	YGR054W
YNL218W	MGS1	YKR072C	SIS2
YIL159W	BNR1	YML001W	YPT7
YGR020C	VMA7	YNL107W	YAF9
YOR014W	RTS1	YDR207C	UME6
YEL053C	MAK10	YJL183W	MNN11
YJL172W	CPS1	YDL083C	RPS16B
YOR066W	MSA1	YBL025W	RRN10
YIL038C	NOT3	YBR089C-A	NHP6B
YNL297C	MON2	YOL108C	INO4
YHR156C	LIN1	YBR200W	BEM1
YOR069W	VPS5	YHR039C-A	VMA10
YNL025C	SSN8	YDL002C	NHP10
YJR099W	YUH1	YDR139C	RUB1
YNL248C	RPA49	YML112W	CTK3
YIR005W	IST3	YDR289C	RTT103
YKL032C	IXR1	YPL213W	LEA1

Predictions at threshold level 0.75 continued...			
Gene1 of predicted pair		Gene2 of predicted pair	
Systematic Name	Standard Name	Systematic Name	Standard Name
YMR179W	SPT21	YGL163C	RAD54
YKL126W	YPK1	YPL161C	BEM4
YGR072W	UPF3	YNL238W	KEX2
YJL053W	PEP8	YGL054C	ERV14
YHR052W	CIC1	YPR154W	PIN3
YMR199W	CLN1	YKL032C	IXR1
YHR098C	SFB3	YMR048W	CSM3
YFR036W	CDC26	YBR280C	SAF1
YMR142C	RPL13B	YOR327C	SNC2
YLL007C	YLL007C	YBR108W	YBR108W
YGL250W	RMR1	YDR318W	MCM21
YLR090W	XDJ1	YLR095C	IOC2
YOR132W	VPS17	YDR144C	MKC7
YJR060W	CBF1	YOR327C	SNC2
YNL041C	COG6	YPR030W	CSR2
YNL248C	RPA49	YOL081W	IRA2
YFR031C-A	RPL2A	YIL053W	RHR2
YNL291C	MID1	YOR327C	SNC2
YOR270C	VPH1	YKL119C	VPH2
YOR112W	CEX1	YDR108W	GSG1
YDR183W	PLP1	YNL107W	YAF9
YDR225W	HTA1	YNL107W	YAF9
YEL042W	GDA1	YER122C	GLO3
YGL087C	MMS2	YMR172W	HOT1
YPL057C	SUR1	YML028W	TSA1
YER110C	KAP123	YDR254W	CHL4
YMR284W	YKU70	YGL175C	SAE2
YHR039C-A	VMA10	YML001W	YPT7
YKL048C	ELM1	YBR036C	CSG2

Predictions at threshold level 0.75 continued...			
Gene1 of predicted pair		Gene2 of predicted pair	
Systematic Name	Standard Name	Systematic Name	Standard Name
YGR135W	PRE9	YKR030W	GMH1
YOR043W	WHI2	YGR071C	YGR071C
YLR441C	RPS1A	YER102W	RPS8B
YJR056C	YJR056C	YPL057C	SUR1
YIL076W	SEC28	YMR133W	REC114
YJR140C	HIR3	YGL148W	ARO2
YPR051W	MAK3	YCL001W	RER1
YML115C	VAN1	YGR217W	CCH1
YGR241C	YAP1802	YDL226C	GCS1
YLR292C	SEC72	YMR127C	SAS2
YPR084W	YPR084W	YHR060W	VMA22
YEL051W	VMA8	YKR030W	GMH1
YEL009C	GCN4	YNL236W	SIN4
YJL127C	SPT10	YPL253C	VIK1
YNL243W	SLA2	YMR070W	MOT3
YPL057C	SUR1	YJL095W	BCK1
YJL172W	CPS1	YGL124C	MON1
YLR429W	CRN1	YNL107W	YAF9
YKL032C	IXR1	YGL153W	PEX14
YAL020C	ATS1	YJL095W	BCK1
YBR200W	BEM1	YER139C	YER139C
YHR073W	OSH3	YLR079W	SIC1
YML071C	COG8	YBR082C	UBC4
YNL215W	IES2	YIR005W	IST3
YBR041W	FAT1	YJL117W	PHO86
YCL037C	SRO9	YPL161C	BEM4
YKL110C	KTI12	YPL018W	CTF19
YBR194W	SOY1	YMR089C	YTA12
YGL115W	SNF4	YMR311C	GLC8

Predictions at threshold level 0.75 continued...			
Gene1 of predicted pair		Gene2 of predicted pair	
Systematic Name	Standard Name	Systematic Name	Standard Name
YBR126C	TPS1	YMR214W	SCJ1
YDR260C	SWM1	YJR135C	MCM22
YPL042C	SSN3	YNL236W	SIN4
YJL154C	VPS35	YAL002W	VPS8
YJL095W	BCK1	YLR180W	SAM1
YIR003W	YIR003W	YNL094W	APP1
YNL238W	KEX2	YNL076W	MKS1
YLR181C	VTA1	YKL041W	VPS24
YFR031C-A	RPL2A	YPL195W	APL5
YPL161C	BEM4	YNL289W	PCL1
YFR031C-A	RPL2A	YKL126W	YPK1
YOL051W	GAL11	YNL079C	TPM1
YPL042C	SSN3	YML062C	MFT1
YDR477W	SNF1	YBR019C	GAL10
YJR140C	HIR3	YBR036C	CSG2
YOL018C	TLG2	YJL164C	TPK1
YPR084W	YPR084W	YFL033C	RIM15
YAL024C	LTE1	YDR378C	LSM6
YKR057W	RPS21A	YGL163C	RAD54
YGL178W	MPT5	YPL161C	BEM4
YAL024C	LTE1	YBL025W	RRN10
YNL293W	MSB3	YGR152C	RSR1
YHR073W	OSH3	YGL016W	KAP122
YEL043W	YEL043W	YER122C	GLO3
YPL253C	VIK1	YHL002W	HSE1
YPL057C	SUR1	YLR370C	ARC18
YIL076W	SEC28	YGL223C	COG1
YML013W	UBX2	YHR012W	VPS29
YLR258W	GSY2	YPR119W	CLB2

Predictions at threshold level 0.75 continued...			
Gene1 of predicted pair		Gene2 of predicted pair	
Systematic Name	Standard Name	Systematic Name	Standard Name
YER044C	ERG28	YOL121C	RPS19A
YDR202C	RAV2	YNR032W	PPG1
YDR183W	PLP1	YGR188C	BUB1
YGL033W	HOP2	YMR152W	YIM1
YJL115W	ASF1	YHR056C	RSC30
YKL116C	PRR1	YML071C	COG8
YMR224C	MRE11	YMR133W	REC114
YER044C	ERG28	YOR327C	SNC2
YJL154C	VPS35	YER162C	RAD4
YLR083C	EMP70	YDR310C	SUM1
YMR127C	SAS2	YMR055C	BUB2
YMR224C	MRE11	YIR005W	IST3
YDR484W	VPS52	YGL198W	YIP4
YBR036C	CSG2	YGR188C	BUB1
YER044C	ERG28	YLR093C	NYV1
YBL003C	HTA2	YOR141C	ARP8
YOR043W	WHI2	YOR368W	RAD17
YMR216C	SKY1	YAL002W	VPS8
YDL184C	RPL41A	YGL025C	PGD1
YIR002C	MPH1	YPL022W	RAD1
YLR447C	VMA6	YCR086W	CSM1
YHR161C	YAP1801	YLR206W	ENT2
YDR183W	PLP1	YJR090C	GRR1
YKL126W	YPK1	YBR019C	GAL10
YMR026C	PEX12	YBR200W	BEM1
YJL154C	VPS35	YDR144C	MKC7
YLR381W	CTF3	YHR115C	DMA1
YPR017C	DSS4	YMR216C	SKY1
YFL023W	BUD27	YDL059C	RAD59

Predictions at threshold level 0.75 continued...			
Gene1 of predicted pair		Gene2 of predicted pair	
Systematic Name	Standard Name	Systematic Name	Standard Name
YHR039C-A	VMA10	YDL006W	PTC1
YAL014C	SYN8	YAL030W	SNC1
YMR152W	YIM1	YMR311C	GLC8
YHR039C-A	VMA10	YJR033C	RAV1
YNL236W	SIN4	YLR357W	RSC2
YOR080W	DIA2	YBL079W	NUP170
YLR109W	AHP1	YLR093C	NYV1
YDL002C	NHP10	YDR378C	LSM6
YLL007C	YLL007C	YMR127C	SAS2
YKL101W	HSL1	YHR115C	DMA1
YPR084W	YPR084W	YLR315W	NKP2
YKL116C	PRR1	YOR327C	SNC2
YHR178W	STB5	YJL190C	RPS22A
YHR178W	STB5	YGR054W	YGR054W
YDR443C	SSN2	YPR036W	VMA13
YKR001C	VPS1	YPL045W	VPS16
YHL031C	GOS1	YDL006W	PTC1
YGL178W	MPT5	YPL086C	ELP3
YPL057C	SUR1	YDR155C	CPR1
YPR120C	CLB5	YDR200C	VPS64
YPR051W	MAK3	YJR060W	CBF1
YOR290C	SNF2	YOR038C	HIR2
YDL184C	RPL41A	YJR084W	CSN12
YHL020C	OPI1	YPL248C	GAL4
YMR152W	YIM1	YLR398C	SKI2
YBR173C	UMP1	YMR016C	SOK2
YGL163C	RAD54	YLR254C	NDL1
YMR172W	HOT1	YGR270W	YTA7
YOR112W	CEX1	YHR012W	VPS29

Predictions at threshold level 0.75 continued...			
Gene1 of predicted pair		Gene2 of predicted pair	
Systematic Name	Standard Name	Systematic Name	Standard Name
YHR134W	WSS1	YEL053C	MAK10
YPR084W	YPR084W	YJR033C	RAV1
YGL200C	EMP24	YNL079C	TPM1
YMR167W	MLH1	YOL090W	MSH2
YGL115W	SNF4	YBR019C	GAL10
YER116C	SLX8	YJR135C	MCM22
YEL051W	VMA8	YJR033C	RAV1
YOR191W	RIS1	YGR072W	UPF3
YNL032W	SIW14	YLR371W	ROM2
YJL127C	SPT10	YDR254W	CHL4
YBR195C	MSI1	YCR086W	CSM1
YDR358W	GGA1	YLR370C	ARC18
YMR224C	MRE11	YPL037C	EGD1
YGR020C	VMA7	YAL002W	VPS8
YDR225W	HTA1	YOR195W	SLK19
YDR293C	SSD1	YNR052C	POP2
YBL051C	PIN4	YMR202W	ERG2
YDL059C	RAD59	YPL256C	CLN2
YEL053C	MAK10	YML071C	COG8
YGR188C	BUB1	YHR115C	DMA1
YPR036W	VMA13	YER162C	RAD4
YJR056C	YJR056C	YLR180W	SAM1
YLR447C	VMA6	YDR289C	RTT103
YOR001W	RRP6	YJL124C	LSM1
YNL238W	KEX2	YKR014C	YPT52
YCR077C	PAT1	YER139C	YER139C
YJR032W	CPR7	YBL025W	RRN10
YCL045C	YCL045C	YML051W	GAL80
YJL164C	TPK1	YDL047W	SIT4

Predictions at threshold level 0.75 continued...			
Gene1 of predicted pair		Gene2 of predicted pair	
Systematic Name	Standard Name	Systematic Name	Standard Name
YLR234W	TOP3	YJL006C	CTK2
YKL117W	SBA1	YGL016W	KAP122
YMR095C	SNO1	YOR290C	SNF2
YMR198W	CIK1	YDL127W	PCL2
YGL208W	SIP2	YJR090C	GRR1
YPL253C	VIK1	YCR086W	CSM1
YDR363W-A	SEM1	YKL048C	ELM1
YAL024C	LTE1	YML097C	VPS9
YPL018W	CTF19	YOR014W	RTS1
YCR053W	THR4	YMR230W	RPS10B
YGR249W	MGA1	YML001W	YPT7
YAL024C	LTE1	YGL147C	RPL9A
YMR198W	CIK1	YDL013W	SLX5
YDR004W	RAD57	YPR119W	CLB2
YGL025C	PGD1	YFR036W	CDC26
YPR120C	CLB5	YGL151W	NUT1
YOR212W	STE4	YPL112C	PEX25
YDR363W-A	SEM1	YPL256C	CLN2
YPL105C	YPL105C	YHR060W	VMA22
YBR217W	ATG12	YKR099W	BAS1
YNL087W	TCB2	YBR108W	YBR108W
YMR004W	MVP1	YNL297C	MON2
YLR234W	TOP3	YDL002C	NHP10
YLR381W	CTF3	YMR055C	BUB2
YDL020C	RPN4	YGL066W	SGF73
YMR004W	MVP1	YDR202C	RAV2
YGL033W	HOP2	YOR327C	SNC2
YIL034C	CAP2	YNL293W	MSB3
YMR202W	ERG2	YJR005W	APL1

Predictions at threshold level 0.75 continued...			
Gene1 of predicted pair		Gene2 of predicted pair	
Systematic Name	Standard Name	Systematic Name	Standard Name
YML038C	YMD8	YAL002W	VPS8
YOR127W	RGA1	YEL056W	HAT2
YGL135W	RPL1B	YJL095W	BCK1
YGR040W	KSS1	YLR362W	STE11
YGL087C	MMS2	YLR182W	SWI6
YMR016C	SOK2	YMR133W	REC114
YAR033W	MST28	YPL240C	HSP82
YPL256C	CLN2	YML028W	TSA1
YPL089C	RLM1	YKL161C	YKL161C
YNL218W	MGS1	YDL020C	RPN4
YER144C	UBP5	YDR261C	EXG2
YNL291C	MID1	YEL043W	YEL043W
YNL058C	YNL058C	YER162C	RAD4
YNL041C	COG6	YOR327C	SNC2
YKR030W	GMH1	YAL002W	VPS8
YDR004W	RAD57	YDL155W	CLB3
YDR448W	ADA2	YBR112C	CYC8
YIL053W	RHR2	YHR156C	LIN1
YPR084W	YPR084W	YDL006W	PTC1
YOR337W	TEA1	YLR093C	NYV1
YML097C	VPS9	YNL107W	YAF9
YOR109W	INP53	YLR309C	IMH1
YGR135W	PRE9	YDL047W	SIT4
YPL096C-A	ERI1	YGL200C	EMP24
YBR173C	UMP1	YBR200W	BEM1
YBR215W	HPC2	YOR124C	UBP2
YHR039C-A	VMA10	YJR084W	CSN12
YLR192C	HCR1	YER162C	RAD4
YGR135W	PRE9	YPR046W	MCM16

Predictions at threshold level 0.75 continued...			
Gene1 of predicted pair		Gene2 of predicted pair	
Systematic Name	Standard Name	Systematic Name	Standard Name
YNL044W	YIP3	YGL200C	EMP24
YNL297C	MON2	YEL051W	VMA8
YNL238W	KEX2	YMR054W	STV1
YFL013C	IES1	YDL002C	NHP10
YBL017C	PEP1	YDR484W	VPS52
YGL253W	HXK2	YGR188C	BUB1
YMR109W	MYO5	YNL107W	YAF9
YAL024C	LTE1	YLR093C	NYV1
YJL172W	CPS1	YBR280C	SAF1
YGR252W	GCN5	YHR158C	KEL1
YMR164C	MSS11	YBR083W	TEC1
YLR360W	VPS38	YJL190C	RPS22A
YNL243W	SLA2	YPL051W	ARL3
YAL024C	LTE1	YDR146C	SWI5
YDR097C	MSH6	YOL090W	MSH2
YPL198W	RPL7B	YOR078W	BUD21
YPR120C	CLB5	YML028W	TSA1
YMR152W	YIM1	YLR180W	SAM1
YIR003W	YIR003W	YCR088W	ABP1
YLR192C	HCR1	YHR200W	RPN10
YEL053C	MAK10	YPL234C	TFP3
YDR129C	SAC6	YBR082C	UBC4
YNL288W	CAF40	YPL018W	CTF19
YIL007C	NAS2	YLR371W	ROM2
YNL307C	MCK1	YDR318W	MCM21
YER114C	BOI2	YML109W	ZDS2
YLR079W	SIC1	YFR030W	MET10
YBR131W	CCZ1	YOR106W	VAM3
YBR057C	MUM2	YBL101C	ECM21

Predictions at threshold level 0.75 continued...			
Gene1 of predicted pair		Gene2 of predicted pair	
Systematic Name	Standard Name	Systematic Name	Standard Name
YPL256C	CLN2	YKL092C	BUD2
YLR234W	TOP3	YML028W	TSA1
YPL022W	RAD1	YDL059C	RAD59
YML016C	PPZ1	YOL091W	SPO21
YGL066W	SGF73	YFR036W	CDC26
YJL170C	ASG7	YLR452C	SST2
YNL032W	SIW14	YPR036W	VMA13
YJL124C	LSM1	YGR270W	YTA7
YGR209C	TRX2	YBR158W	AMN1
YLR288C	MEC3	YPL022W	RAD1
YML071C	COG8	YBL025W	RRN10
YNL031C	HHT2	YKL068W	NUP100
YPL178W	CBC2	YPL246C	RBD2
YJL036W	SNX4	YER122C	GLO3
YGR108W	CLB1	YGL253W	HXK2
YKL139W	CTK1	YDR439W	LRS4
YGR270W	YTA7	YJL190C	RPS22A
YKL160W	ELF1	YNL238W	KEX2
YJL172W	CPS1	YHR060W	VMA22
YMR095C	SNO1	YOR270C	VPH1
YIL156W	UBP7	YMR125W	STO1
YPR120C	CLB5	YOR141C	ARP8
YNL325C	FIG4	YLR180W	SAM1
YOR109W	INP53	YER122C	GLO3
YDR156W	RPA14	YIR005W	IST3
YDR378C	LSM6	YLR350W	ORM2
YDL020C	RPN4	YGR105W	VMA21
YDR443C	SSN2	YLR386W	VAC14
YDR103W	STE5	YJL187C	SWE1

Predictions at threshold level 0.75 continued...			
Gene1 of predicted pair		Gene2 of predicted pair	
Systematic Name	Standard Name	Systematic Name	Standard Name
YOR069W	VPS5	YDR153C	ENT5
YNR052C	POP2	YLR410W	VIP1
YPL137C	GIP3	YLR398C	SKI2
YGL115W	SNF4	YNL127W	FAR11
YDL006W	PTC1	YMR038C	CCS1
YJR060W	CBF1	YML071C	COG8
YGL212W	VAM7	YDL192W	ARF1
YER120W	SCS2	YNL107W	YAF9
YOR071C	THI71	YDR105C	TMS1
YHL031C	GOS1	YKR020W	VPS51
YML103C	NUP188	YPR120C	CLB5
YDR108W	GSG1	YNL107W	YAF9
YHR119W	SET1	YPL240C	HSP82
YLR135W	SLX4	YDR207C	UME6
YOR033C	EXO1	YCR086W	CSM1
YLR170C	APS1	YOR327C	SNC2
YBL103C	RTG3	YNL307C	MCK1
YBR057C	MUM2	YDL155W	CLB3
YNL036W	NCE103	YPL240C	HSP82
YIL076W	SEC28	YHR039C-A	VMA10
YEL043W	YEL043W	YDR207C	UME6
YJL127C	SPT10	YDL047W	SIT4
YIL053W	RHR2	YNR072W	HXT17
YPR017C	DSS4	YGL212W	VAM7
YNL027W	CRZ1	YIL033C	BCY1
YGL215W	CLG1	YGL200C	EMP24
YDL232W	OST4	YOR085W	OST3
YKL007W	CAP1	YOR367W	SCP1
YLR079W	SIC1	YDR363W-A	SEM1

Predictions at threshold level 0.75 continued...			
Gene1 of predicted pair		Gene2 of predicted pair	
Systematic Name	Standard Name	Systematic Name	Standard Name
YLR370C	ARC18	YLR398C	SKI2
YBL047C	EDE1	YHR013C	ARD1
YDL184C	RPL41A	YGL124C	MON1
YPL234C	TFP3	YNL238W	KEX2
YHR134W	WSS1	YEL056W	HAT2
YJR063W	RPA12	YNL107W	YAF9
YEL053C	MAK10	YLR452C	SST2
YDR477W	SNF1	YOL111C	MDY2
YPL256C	CLN2	YDR207C	UME6
YEL053C	MAK10	YBR036C	CSG2
YGR261C	APL6	YLR396C	VPS33
YLR447C	VMA6	YDL077C	VAM6
YKR020W	VPS51	YMR054W	STV1
YDR184C	ATC1	YPR119W	CLB2
YNL291C	MID1	YKR030W	GMH1
YPL096C-A	ERI1	YPL050C	MNN9
YOL111C	MDY2	YER162C	RAD4
YPL045W	VPS16	YLR093C	NYV1
YGL025C	PGD1	YGL151W	NUT1
YNL297C	MON2	YGL003C	CDH1
YNL106C	INP52	YDR254W	CHL4
YML099C	ARG81	YJR094C	IME1
YLR095C	IOC2	YBR036C	CSG2
YAL020C	ATS1	YHR115C	DMA1
YDR335W	MSN5	YDR440W	DOT1
YLR178C	TFS1	YOL081W	IRA2
YJL210W	PEX2	YNL214W	PEX17
YER096W	SHC1	YDR289C	RTT103
YFR036W	CDC26	YNL116W	DMA2

Predictions at threshold level 0.75 continued...			
Gene1 of predicted pair		Gene2 of predicted pair	
Systematic Name	Standard Name	Systematic Name	Standard Name
YGL087C	MMS2	YBR114W	RAD16
YNL215W	IES2	YLR350W	ORM2
YBL021C	HAP3	YBR114W	RAD16
YOR112W	CEX1	YML038C	YMD8
YGR072W	UPF3	YMR216C	SKY1
YHR152W	SPO12	YPR119W	CLB2
YOR195W	SLK19	YJR035W	RAD26
YBL051C	PIN4	YHR111W	UBA4
YLL007C	YLL007C	YIL034C	CAP2
YDR484W	VPS52	YDR323C	PEP7
YDR293C	SSD1	YLR381W	CTF3
YOR337W	TEA1	YHR060W	VMA22
YKL010C	UFD4	YPL213W	LEA1
YLR320W	MMS22	YNL076W	MKS1
YLR206W	ENT2	YBL106C	SRO77
YOR337W	TEA1	YOR035C	SHE4
YCR053W	THR4	YLR180W	SAM1
YNL068C	FKH2	YBR084C-A	RPL19A
YDR254W	CHL4	YGL115W	SNF4
YOL121C	RPS19A	YBR036C	CSG2
YCR024C-A	PMP1	YGR059W	SPR3
YOR106W	VAM3	YCL037C	SRO9
YPL120W	VPS30	YNR032W	PPG1
YDR363W-A	SEM1	YHR013C	ARD1
YNL069C	RPL16B	YAL002W	VPS8
YFR036W	CDC26	YDL192W	ARF1
YIL076W	SEC28	YPR051W	MAK3
YBR057C	MUM2	YAR014C	BUD14
YDR254W	CHL4	YDR183W	PLP1

Predictions at threshold level 0.75 continued...			
Gene1 of predicted pair		Gene2 of predicted pair	
Systematic Name	Standard Name	Systematic Name	Standard Name
YDR202C	RAV2	YML071C	COG8
YMR164C	MSS11	YKL117W	SBA1
YLR386W	VAC14	YHR081W	LRP1
YIL053W	RHR2	YGL003C	CDH1
YJR060W	CBF1	YIR005W	IST3
YLR452C	SST2	YDR358W	GGA1
YLL039C	UBI4	YLR128W	DCN1
YKL048C	ELM1	YKR048C	NAP1
YGR249W	MGA1	YBR030W	YBR030W
YER120W	SCS2	YDL006W	PTC1
YJR084W	CSN12	YGL124C	MON1
YBR215W	HPC2	YDR318W	MCM21
YOL100W	PKH2	YPL004C	LSP1
YFR031C-A	RPL2A	YMR016C	SOK2
YNL236W	SIN4	YPL106C	SSE1
YBL058W	SHP1	YBR173C	UMP1
YDR004W	RAD57	YGL249W	ZIP2
YJL124C	LSM1	YLR429W	CRN1
YGL066W	SGF73	YBR010W	HHT1
YBR215W	HPC2	YKL160W	ELF1
YHR039C-A	VMA10	YCR053W	THR4
YLR234W	TOP3	YGL151W	NUT1
YNL145W	MFA2	YPL220W	RPL1A
YNL307C	MCK1	YDR310C	SUM1
YDR202C	RAV2	YDL185W	TFP1
YJL058C	BIT61	YMR055C	BUB2
YAL020C	ATS1	YMR202W	ERG2
YJR040W	GEF1	YCR066W	RAD18
YDR174W	HMO1	YOR195W	SLK19

Predictions at threshold level 0.75 continued...			
Gene1 of predicted pair		Gene2 of predicted pair	
Systematic Name	Standard Name	Systematic Name	Standard Name
YOR358W	HAP5	YFL047W	RGD2
YER139C	YER139C	YJL095W	BCK1
YKL213C	DOA1	YIR005W	IST3
YFR010W	UBP6	YLR093C	NYV1
YFR031C-A	RPL2A	YOR327C	SNC2
YNL297C	MON2	YGL115W	SNF4
YNL288W	CAF40	YPL203W	TPK2
YMR116C	ASC1	YNL016W	PUB1
YOL030W	GAS5	YLR410W	VIP1
YBL051C	PIN4	YNL289W	PCL1
YDR443C	SSN2	YKL116C	PRR1
YOL136C	PFK27	YBR158W	AMN1
YLR240W	VPS34	YOL018C	TLG2
YDR174W	HMO1	YLR381W	CTF3
YOR109W	INP53	YDR153C	ENT5
YHR178W	STB5	YHL002W	HSE1
YPL037C	EGD1	YOL018C	TLG2
YLR119W	SRN2	YNL076W	MKS1
YHR200W	RPN10	YBR280C	SAF1
YDR448W	ADA2	YGR252W	GCN5
YNL199C	GCR2	YHR167W	THP2
YDR293C	SSD1	YCL037C	SRO9
YPR120C	CLB5	YAR014C	BUD14
YLR381W	CTF3	YGL163C	RAD54
YGR184C	UBR1	YPL018W	CTF19
YJL176C	SWI3	YGR135W	PRE9
YDR443C	SSN2	YMR095C	SNO1
YML058W	SML1	YML095C	RAD10
YJR135C	MCM22	YFR036W	CDC26

Predictions at threshold level 0.75 continued...			
Gene1 of predicted pair		Gene2 of predicted pair	
Systematic Name	Standard Name	Systematic Name	Standard Name
YFR030W	MET10	YJL129C	TRK1
YJR135C	MCM22	YDR200C	VPS64
YJL036W	SNX4	YCL037C	SRO9
YNL297C	MON2	YGL054C	ERV14
YKL139W	CTK1	YER144C	UBP5
YBR200W	BEM1	YLR315W	NKP2
YHR156C	LIN1	YCR053W	THR4
YPL158C	YPL158C	YHR108W	GGA2
YDR484W	VPS52	YLR441C	RPS1A
YDR244W	PEX5	YPL112C	PEX25
YMR095C	SNO1	YDL184C	RPL41A
YDR385W	EFT2	YKL041W	VPS24
YDR156W	RPA14	YIL036W	CST6
YLR337C	VRP1	YBL072C	RPS8A
YGR108W	CLB1	YHR134W	WSS1
YIL156W	UBP7	YER110C	KAP123
YLR360W	VPS38	YPL120W	VPS30
YOL121C	RPS19A	YEL060C	PRB1
YOR023C	AHC1	YML095C	RAD10
YMR116C	ASC1	YMR089C	YTA12
YGR104C	SRB5	YHR167W	THP2
YDR171W	HSP42	YLR370C	ARC18
YGL133W	ITC1	YML095C	RAD10
YNL215W	IES2	YJR060W	CBF1
YFR031C-A	RPL2A	YBR200W	BEM1
YKL126W	YPK1	YHR060W	VMA22
YDR129C	SAC6	YMR231W	PEP5
YKL032C	IXR1	YDR363W-A	SEM1
YHR086W	NAM8	YKL074C	MUD2

Predictions at threshold level 0.75 continued...			
Gene1 of predicted pair		Gene2 of predicted pair	
Systematic Name	Standard Name	Systematic Name	Standard Name
YCR034W	FEN1	YJR032W	CPR7
YPR120C	CLB5	YPR119W	CLB2
YBR215W	HPC2	YJR035W	RAD26
YKR057W	RPS21A	YML095C	RAD10
YBL021C	HAP3	YPL018W	CTF19
YEL053C	MAK10	YHR081W	LRP1
YPL037C	EGD1	YER120W	SCS2
YDR469W	SDC1	YEL056W	HAT2
YBR173C	UMP1	YOR270C	VPH1
YBR107C	IML3	YDR318W	MCM21
YPL161C	BEM4	YNL079C	TPM1
YJR035W	RAD26	YGL031C	RPL24A
YJR140C	HIR3	YGL250W	RMR1
YKL038W	RGT1	YBR112C	CYC8
YBR126C	TPS1	YGR249W	MGA1
YMR055C	BUB2	YMR038C	CCS1
YGL033W	HOP2	YNL307C	MCK1
YHR012W	VPS29	YOL108C	INO4
YJR056C	YJR056C	YBR280C	SAF1
YKL126W	YPK1	YLR425W	TUS1
YML095C	RAD10	YDR254W	CHL4
YPL037C	EGD1	YCR053W	THR4
YKL214C	YRA2	YHR167W	THP2
YMR054W	STV1	YJR033C	RAV1
YKL126W	YPK1	YNL041C	COG6
YLR090W	XDJ1	YDL184C	RPL41A
YGR261C	APL6	YEL051W	VMA8
YOR337W	TEA1	YOR043W	WHI2
YMR224C	MRE11	YOR308C	SNU66

Predictions at threshold level 0.75 continued...			
Gene1 of predicted pair		Gene2 of predicted pair	
Systematic Name	Standard Name	Systematic Name	Standard Name
YDL116W	NUP84	YEL050C	RML2
YBL047C	EDE1	YJL095W	BCK1
YIL033C	BCY1	YPL018W	CTF19
YFL013C	IES1	YJR140C	HIR3
YGR249W	MGA1	YOR043W	WHI2
YNL206C	RTT106	YOR038C	HIR2
YMR055C	BUB2	YJR035W	RAD26
YDR293C	SSD1	YPL256C	CLN2
YBL101C	ECM21	YHL002W	HSE1
YLR119W	SRN2	YER122C	GLO3
YGR072W	UPF3	YER122C	GLO3
YNL291C	MID1	YML071C	COG8
YDR156W	RPA14	YBL101C	ECM21
YNL041C	COG6	YDR469W	SDC1
YDR389W	SAC7	YOR035C	SHE4
YML016C	PPZ1	YNL107W	YAF9
YNL058C	YNL058C	YEL060C	PRB1
YHR013C	ARD1	YKL101W	HSL1
YFL033C	RIM15	YGL054C	ERV14
YLR292C	SEC72	YPL161C	BEM4
YOR043W	WHI2	YER161C	SPT2
YER144C	UBP5	YBR171W	SEC66
YML117W	NAB6	YHR200W	RPN10
YLR083C	EMP70	YNL291C	MID1
YDR004W	RAD57	YML095C	RAD10
YMR167W	MLH1	YOR368W	RAD17
YPL115C	BEM3	YDR183W	PLP1
YGR054W	YGR054W	YHR114W	BZZ1
YGL135W	RPL1B	YDR358W	GGA1

Predictions at threshold level 0.75 continued...			
Gene1 of predicted pair		Gene2 of predicted pair	
Systematic Name	Standard Name	Systematic Name	Standard Name
YBR108W	YBR108W	YEL053C	MAK10
YGL147C	RPL9A	YER162C	RAD4
YBL027W	RPL19B	YGR188C	BUB1
YLR288C	MEC3	YMR198W	CIK1
YPR131C	NAT3	YMR202W	ERG2
YOR106W	VAM3	YJR033C	RAV1
YOR145C	PNO1	YOR001W	RRP6
YKR035W-A	DID2	YNL076W	MKS1
YGR078C	PAC10	YEL022W	GEA2
YBR126C	TPS1	YBR280C	SAF1
YHR111W	UBA4	YKR048C	NAP1
YHL027W	RIM101	YER122C	GLO3
YDR174W	HMO1	YGL163C	RAD54
YJR056C	YJR056C	YHR081W	LRP1
YBL051C	PIN4	YLR381W	CTF3
YCR020C-A	MAK31	YCL037C	SRO9
YLR360W	VPS38	YAR014C	BUD14
YOR327C	SNC2	YNR049C	MSO1
YDR335W	MSN5	YGL003C	CDH1
YHR158C	KEL1	YOL081W	IRA2
YGL025C	PGD1	YNL236W	SIN4
YDR202C	RAV2	YML001W	YPT7
YCR088W	ABP1	YOR035C	SHE4
YML115C	VAN1	YOR140W	SFL1
YLR447C	VMA6	YHL002W	HSE1
YDL065C	PEX19	YOR193W	PEX27
YIL002C	INP51	YDR080W	VPS41
YGR184C	UBR1	YDR254W	CHL4
YNL041C	COG6	YKL119C	VPH2

Predictions at threshold level 0.75 continued...			
Gene1 of predicted pair		Gene2 of predicted pair	
Systematic Name	Standard Name	Systematic Name	Standard Name
YKL126W	YPK1	YDL192W	ARF1
YDR448W	ADA2	YNL031C	HHT2
YBL102W	SFT2	YNL107W	YAF9
YLR102C	APC9	YKL048C	ELM1
YPL161C	BEM4	YEL060C	PRB1
YPL022W	RAD1	YPL018W	CTF19
YLR234W	TOP3	YOR080W	DIA2
YNL215W	IES2	YNL225C	CNM67
YDL005C	MED2	YJL140W	RPB4
YOR039W	CKB2	YOR368W	RAD17
YKR072C	SIS2	YDL013W	SLX5
YAL024C	LTE1	YML112W	CTK3
YOR132W	VPS17	YJL204C	RCY1
YMR133W	REC114	YDR310C	SUM1
YKL025C	PAN3	YPR051W	MAK3
YBR217W	ATG12	YLR094C	GIS3
YMR152W	YIM1	YJL095W	BCK1
YOR243C	PUS7	YHR167W	THP2
YML099C	ARG81	YMR042W	ARG80
YDR245W	MNN10	YHL002W	HSE1
YKL048C	ELM1	YOR066W	MSA1
YLR093C	NYV1	YEL013W	VAC8
YNL215W	IES2	YGL148W	ARO2
YJL124C	LSM1	YOL091W	SPO21
YMR127C	SAS2	YHR114W	BZZ1
YJR140C	HIR3	YDR289C	RTT103
YIL159W	BNR1	YNL087W	TCB2
YOR290C	SNF2	YER068W	MOT2
YDR245W	MNN10	YOR043W	WHI2

Predictions at threshold level 0.75 continued...			
Gene1 of predicted pair		Gene2 of predicted pair	
Systematic Name	Standard Name	Systematic Name	Standard Name
YJL036W	SNX4	YER162C	RAD4
YLR058C	SHM2	YDR318W	MCM21
YHR200W	RPN10	YGL003C	CDH1
YKR020W	VPS51	YML071C	COG8
YOR039W	CKB2	YPR119W	CLB2
YDR439W	LRS4	YDR254W	CHL4
YEL065W	SIT1	YJL140W	RPB4
YOL111C	MDY2	YLR371W	ROM2
YAL024C	LTE1	YML071C	COG8
YDL020C	RPN4	YBR057C	MUM2
YNL106C	INP52	YLR381W	CTF3
YHL020C	OPI1	YAL002W	VPS8
YDR383C	NKP1	YDR378C	LSM6
YNL248C	RPA49	YAR014C	BUD14
YMR179W	SPT21	YDL013W	SLX5
YCR066W	RAD18	YDL047W	SIT4
YLR095C	IOC2	YML001W	YPT7
YGL054C	ERV14	YKR039W	GAP1
YLR170C	APS1	YPL259C	APM1
YOR368W	RAD17	YMR038C	CCS1
YBL021C	HAP3	YDR318W	MCM21
YOR337W	TEA1	YBR030W	YBR030W
YLR410W	VIP1	YOR141C	ARP8
YDR009W	GAL3	YML051W	GAL80
YOR106W	VAM3	YGL115W	SNF4
YKR001C	VPS1	YKL041W	VPS24
YPL024W	RMI1	YGL124C	MON1
YDR289C	RTT103	YOL054W	PSH1
YOR337W	TEA1	YBR173C	UMP1

Predictions at threshold level 0.75 continued...			
Gene1 of predicted pair		Gene2 of predicted pair	
Systematic Name	Standard Name	Systematic Name	Standard Name
YDR378C	LSM6	YPL220W	RPL1A
YGR261C	APL6	YDR207C	UME6
YEL053C	MAK10	YMR230W	RPS10B
YGL003C	CDH1	YOR368W	RAD17
YOR066W	MSA1	YHR081W	LRP1
YPR036W	VMA13	YJR032W	CPR7
YMR202W	ERG2	YML095C	RAD10
YPL246C	RBD2	YGR059W	SPR3
YJR060W	CBF1	YER120W	SCS2
YOR212W	STE4	YBL016W	FUS3
YCR020C-A	MAK31	YOR127W	RGA1
YBR171W	SEC66	YJR032W	CPR7
YFL023W	BUD27	YDL223C	HBT1
YML071C	COG8	YMR311C	GLC8
YDL149W	ATG9	YLR431C	ATG23
YGL212W	VAM7	YDL083C	RPS16B
YPL120W	VPS30	YNL127W	FAR11
YFR008W	FAR7	YMR052W	FAR3
YPL195W	APL5	YGL124C	MON1
YKL048C	ELM1	YER044C	ERG28
YGL253W	HXK2	YJL190C	RPS22A
YMR199W	CLN1	YLR425W	TUS1
YOR106W	VAM3	YPL120W	VPS30
YOR083W	WHI5	YPR046W	MCM16
YIL034C	CAP2	YJR032W	CPR7
YDL020C	RPN4	YDL155W	CLB3
YER110C	KAP123	YGL253W	HXK2
YIL076W	SEC28	YFR036W	CDC26
YBR057C	MUM2	YDR318W	MCM21

Predictions at threshold level 0.75 continued...			
**Gene1 of predicted pair**		**Gene2 of predicted pair**	
**Systematic Name**	**Standard Name**	**Systematic Name**	**Standard Name**
YML071C	COG8	YOL081W	IRA2
YBL102W	SFT2	YJL004C	SYS1
YFR010W	UBP6	YJR084W	CSN12
YOR033C	EXO1	YMR038C	CCS1
YDR320C	SWA2	YEL022W	GEA2
YGL147C	RPL9A	YNL307C	MCK1
YDR200C	VPS64	YOL091W	SPO21
YLR370C	ARC18	YMR230W	RPS10B
YDR378C	LSM6	YDR440W	DOT1
YBL079W	NUP170	YGL031C	RPL24A
YMR095C	SNO1	YKR035W-A	DID2
YNL248C	RPA49	YIR005W	IST3
YKL010C	UFD4	YDL155W	CLB3
YEL053C	MAK10	YDR183W	PLP1
YPL161C	BEM4	YMR230W	RPS10B
YOR275C	RIM20	YNL229C	URE2
YOR180C	DCI1	YLR284C	ECI1
YDR261C	EXG2	YLR371W	ROM2
YCR063W	BUD31	YIR009W	MSL1
YDR309C	GIC2	YDR310C	SUM1
YBR290W	BSD2	YMR164C	MSS11
YJL172W	CPS1	YNL076W	MKS1
YNL058C	YNL058C	YOL121C	RPS19A
YGL087C	MMS2	YMR127C	SAS2
YGL147C	RPL9A	YPL057C	SUR1
YMR205C	PFK2	YJL183W	MNN11
YLR386W	VAC14	YOR141C	ARP8
YBL103C	RTG3	YPL057C	SUR1
YNL069C	RPL16B	YGL054C	ERV14

Predictions at threshold level 0.75 continued...			
Gene1 of predicted pair		Gene2 of predicted pair	
Systematic Name	Standard Name	Systematic Name	Standard Name
YEL065W	SIT1	YMR311C	GLC8
YKL025C	PAN3	YBL051C	PIN4
YGR104C	SRB5	YJL095W	BCK1
YDL155W	CLB3	YBL072C	RPS8A
YHL031C	GOS1	YHR167W	THP2
YOR358W	HAP5	YPL248C	GAL4
YFL033C	RIM15	YEL053C	MAK10
YNL243W	SLA2	YDL192W	ARF1
YLR315W	NKP2	YML112W	CTK3
YHL020C	OPI1	YNL041C	COG6
YNR052C	POP2	YBR200W	BEM1
YMR116C	ASC1	YER001W	MNN1
YAL014C	SYN8	YEL013W	VAC8
YDL020C	RPN4	YBR082C	UBC4
YKL032C	IXR1	YKR020W	VPS51
YPL022W	RAD1	YBR274W	CHK1
YJR140C	HIR3	YDR146C	SWI5
YAR014C	BUD14	YGL252C	RTG2
YLR410W	VIP1	YJR060W	CBF1
YDR318W	MCM21	YOR014W	RTS1
YPR030W	CSR2	YGL003C	CDH1
YPL049C	DIG1	YDR480W	DIG2
YCL001W	RER1	YML038C	YMD8
YBR041W	FAT1	YHR200W	RPN10
YDR264C	AKR1	YPL256C	CLN2
YAL024C	LTE1	YDR183W	PLP1
YDL223C	HBT1	YKL041W	VPS24
YDL116W	NUP84	YIL148W	RPL40A
YCR088W	ABP1	YMR109W	MYO5

Predictions at threshold level 0.75 continued...			
**Gene1 of predicted pair**		**Gene2 of predicted pair**	
**Systematic Name**	**Standard Name**	**Systematic Name**	**Standard Name**
YHR039C-A	VMA10	YMR214W	SCJ1
YBL058W	SHP1	YFL023W	BUD27
YGR184C	UBR1	YMR038C	CCS1
YDR320C	SWA2	YDL006W	PTC1
YBR217W	ATG12	YHR039C-A	VMA10
YBR217W	ATG12	YNL032W	SIW14
YKL048C	ELM1	YLR371W	ROM2
YDR004W	RAD57	YBR278W	DPB3
YDR439W	LRS4	YML112W	CTK3
YOR127W	RGA1	YNL051W	COG5
YDR320C	SWA2	YOR327C	SNC2
YBR065C	ECM2	YDR163W	CWC15
YPR017C	DSS4	YIL076W	SEC28
YHR200W	RPN10	YBL087C	RPL23A
YLL039C	UBI4	YMR089C	YTA12
YBR279W	PAF1	YBR089C-A	NHP6B
YLR292C	SEC72	YCR086W	CSM1
YJL210W	PEX2	YMR026C	PEX12
YKR072C	SIS2	YBR274W	CHK1
YPL050C	MNN9	YJL095W	BCK1
YDR176W	NGG1	YNL031C	HHT2
YLR292C	SEC72	YGR054W	YGR054W
YLR240W	VPS34	YOR036W	PEP12
YGL163C	RAD54	YDR310C	SUM1
YNL218W	MGS1	YOR080W	DIA2
YFL033C	RIM15	YOL108C	INO4
YMR142C	RPL13B	YLR370C	ARC18
YHR156C	LIN1	YER139C	YER139C
YOR140W	SFL1	YHR012W	VPS29

Predictions at threshold level 0.75 continued...			
Gene1 of predicted pair		Gene2 of predicted pair	
Systematic Name	Standard Name	Systematic Name	Standard Name
YPR017C	DSS4	YOR069W	VPS5
YGL078C	DBP3	YCR053W	THR4
YER116C	SLX8	YBR200W	BEM1
YGR249W	MGA1	YPL037C	EGD1
YER110C	KAP123	YLR447C	VMA6
YER139C	YER139C	YOR124C	UBP2
YHR086W	NAM8	YNL206C	RTT106
YBR171W	SEC66	YGL124C	MON1
YML109W	ZDS2	YDR227W	SIR4
YLR095C	IOC2	YPL057C	SUR1
YKR061W	KTR2	YDR254W	CHL4
YIL053W	RHR2	YKR099W	BAS1
YML102W	CAC2	YGL175C	SAE2
YER120W	SCS2	YAR014C	BUD14
YKR035W-A	DID2	YPR046W	MCM16
YJL095W	BCK1	YBR280C	SAF1
YHR178W	STB5	YJL095W	BCK1
YMR004W	MVP1	YMR216C	SKY1
YBR108W	YBR108W	YOR035C	SHE4
YKL139W	CTK1	YFL023W	BUD27
YCR053W	THR4	YHL011C	PRS3
YBR019C	GAL10	YMR230W	RPS10B
YIR005W	IST3	YDR183W	PLP1
YHR124W	NDT80	YJR094C	IME1
YDR245W	MNN10	YOL081W	IRA2
YDR192C	NUP42	YGL100W	SEH1
YOR290C	SNF2	YJL183W	MNN11
YNR072W	HXT17	YLR452C	SST2
YFR031C-A	RPL2A	YLR119W	SRN2

Predictions at threshold level 0.75 continued...			
Gene1 of predicted pair		Gene2 of predicted pair	
Systematic Name	Standard Name	Systematic Name	Standard Name
YMR172W	HOT1	YDR310C	SUM1
YGL190C	CDC55	YKL057C	NUP120
YHR108W	GGA2	YER122C	GLO3
YLR360W	VPS38	YBL047C	EDE1
YBL008W	HIR1	YLR429W	CRN1
YDL116W	NUP84	YGR081C	SLX9
YPR084W	YPR084W	YDR108W	GSG1
YMR142C	RPL13B	YBR019C	GAL10
YGL078C	DBP3	YHR052W	CIC1
YHR156C	LIN1	YBR036C	CSG2
YLR381W	CTF3	YJL190C	RPS22A
YHR098C	SFB3	YGL223C	COG1
YHR161C	YAP1801	YIL033C	BCY1
YPL022W	RAD1	YMR038C	CCS1
YKR001C	VPS1	YJL183W	MNN11
YNL215W	IES2	YJR063W	RPA12
YKR101W	SIR1	YML102W	CAC2
YEL065W	SIT1	YMR026C	PEX12
YIL008W	URM1	YDL155W	CLB3
YLL007C	YLL007C	YOR127W	RGA1
YML013W	UBX2	YJR084W	CSN12
YIL156W	UBP7	YPL256C	CLN2
YKL048C	ELM1	YBL025W	RRN10
YJR058C	APS2	YDL192W	ARF1
YPL024W	RMI1	YKL116C	PRR1
YBR057C	MUM2	YBL025W	RRN10
YDR477W	SNF1	YOL121C	RPS19A
YDR004W	RAD57	YDR310C	SUM1
YCR065W	HCM1	YCR077C	PAT1

Predictions at threshold level 0.75 continued...			
Gene1 of predicted pair		Gene2 of predicted pair	
Systematic Name	Standard Name	Systematic Name	Standard Name
YHR156C	LIN1	YDL184C	RPL41A
YIL033C	BCY1	YGR188C	BUB1
YDL006W	PTC1	YGR135W	PRE9
YGR241C	YAP1802	YLR360W	VPS38
YDR383C	NKP1	YKR061W	KTR2
YPR036W	VMA13	YDR207C	UME6
YML095C	RAD10	YHR111W	UBA4
YPR084W	YPR084W	YNL199C	GCR2
YIL036W	CST6	YOL081W	IRA2
YDL184C	RPL41A	YPL037C	EGD1
YML115C	VAN1	YBL067C	UBP13
YOR106W	VAM3	YEL036C	ANP1
YCR002C	CDC10	YCL024W	KCC4
YOR127W	RGA1	YDL155W	CLB3
YPR030W	CSR2	YGL124C	MON1
YOL145C	CTR9	YDR469W	SDC1
YDR358W	GGA1	YDL137W	ARF2
YLR043C	TRX1	YEL013W	VAC8
YML115C	VAN1	YBR215W	HPC2
YDL020C	RPN4	YKR072C	SIS2
YLR119W	SRN2	YKL048C	ELM1
YFR010W	UBP6	YBR107C	IML3
YKL010C	UFD4	YJL095W	BCK1
YJR140C	HIR3	YKL160W	ELF1
YGL237C	HAP2	YHR115C	DMA1
YJR024C	MDE1	YOL126C	MDH2
YLR386W	VAC14	YNL107W	YAF9
YFL033C	RIM15	YER122C	GLO3
YER144C	UBP5	YBL025W	RRN10

Predictions at threshold level 0.75 continued...			
Gene1 of predicted pair		Gene2 of predicted pair	
Systematic Name	Standard Name	Systematic Name	Standard Name
YGR104C	SRB5	YDR146C	SWI5
YHR013C	ARD1	YKR020W	VPS51
YBL058W	SHP1	YNL087W	TCB2
YDR358W	GGA1	YDR207C	UME6
YOR132W	VPS17	YPR032W	SRO7
YFR031C-A	RPL2A	YCR053W	THR4
YMR116C	ASC1	YKL041W	VPS24
YBR171W	SEC66	YBR114W	RAD16
YMR224C	MRE11	YDR225W	HTA1
YPL240C	HSP82	YCR040W	MATALPHA1
YNL069C	RPL16B	YLR360W	VPS38
YMR216C	SKY1	YJR060W	CBF1
YCR077C	PAT1	YGL151W	NUT1
YEL022W	GEA2	YLR039C	RIC1
YLR288C	MEC3	YER116C	SLX8
YLR386W	VAC14	YKL110C	KTI12
YIL153W	RRD1	YGL190C	CDC55
YLR309C	IMH1	YAL002W	VPS8
YGL200C	EMP24	YGR105W	VMA21
YDL155W	CLB3	YDR289C	RTT103
YBR009C	HHF1	YLR357W	RSC2
YNL248C	RPA49	YPL018W	CTF19
YML103C	NUP188	YPL256C	CLN2
YGL147C	RPL9A	YBR030W	YBR030W
YGR241C	YAP1802	YDR323C	PEP7
YML012W	ERV25	YGL054C	ERV14
YMR284W	YKU70	YJL124C	LSM1
YIL034C	CAP2	YNL307C	MCK1
YNL238W	KEX2	YMR214W	SCJ1

Predictions at threshold level 0.75 continued...			
**Gene1 of predicted pair**		**Gene2 of predicted pair**	
**Systematic Name**	**Standard Name**	**Systematic Name**	**Standard Name**
YHR134W	WSS1	YOR358W	HAP5
YEL065W	SIT1	YLR410W	VIP1
YLR061W	RPL22A	YJR084W	CSN12
YDL006W	PTC1	YKL161C	YKL161C
YGR104C	SRB5	YEL036C	ANP1
YJL127C	SPT10	YMR106C	YKU80
YNL206C	RTT106	YLR399C	BDF1
YMR039C	SUB1	YMR055C	BUB2
YJL117W	PHO86	YJL095W	BCK1
YGL033W	HOP2	YJR056C	YJR056C
YEL053C	MAK10	YPR030W	CSR2
YPR120C	CLB5	YNL079C	TPM1
YDL116W	NUP84	YDR363W-A	SEM1
YKR061W	KTR2	YPL253C	VIK1
YFR030W	MET10	YOR141C	ARP8
YDR174W	HMO1	YPL253C	VIK1
YOR083W	WHI5	YPR131C	NAT3
YER049W	TPA1	YLR425W	TUS1
YLR135W	SLX4	YHR167W	THP2
YDR244W	PEX5	YLR284C	ECI1
YDR245W	MNN10	YGR270W	YTA7
YDR289C	RTT103	YGR188C	BUB1
YER116C	SLX8	YOL121C	RPS19A
YLL007C	YLL007C	YDR310C	SUM1
YDR129C	SAC6	YER001W	MNN1
YAL024C	LTE1	YLR006C	SSK1
YHR111W	UBA4	YDL006W	PTC1
YPR046W	MCM16	YEL060C	PRB1
YBL058W	SHP1	YNL325C	FIG4

Predictions at threshold level 0.75 continued...			
Gene1 of predicted pair		Gene2 of predicted pair	
Systematic Name	Standard Name	Systematic Name	Standard Name
YMR116C	ASC1	YJR035W	RAD26
YKL126W	YPK1	YEL053C	MAK10
YMR127C	SAS2	YGR270W	YTA7
YLR216C	CPR6	YDR260C	SWM1
YMR142C	RPL13B	YMR214W	SCJ1
YBR030W	YBR030W	YER122C	GLO3
YMR054W	STV1	YAL002W	VPS8
YOR080W	DIA2	YNL199C	GCR2
YBR108W	YBR108W	YDR318W	MCM21
YLR337C	VRP1	YOR358W	HAP5
YKR061W	KTR2	YPR120C	CLB5
YOR132W	VPS17	YDL155W	CLB3
YMR142C	RPL13B	YNL058C	YNL058C
YPL161C	BEM4	YDR309C	GIC2
YNR058W	BIO3	YLR094C	GIS3
YHL031C	GOS1	YLR182W	SWI6
YMR313C	TGL3	YKL094W	YJU3
YHR205W	SCH9	YJL095W	BCK1
YBL021C	HAP3	YJR135C	MCM22
YMR199W	CLN1	YGL031C	RPL24A
YIR003W	YIR003W	YPR171W	BSP1
YBR173C	UMP1	YJL183W	MNN11
YOR290C	SNF2	YFR036W	CDC26
YDR153C	ENT5	YNR032W	PPG1
YPL195W	APL5	YMR311C	GLC8
YDR200C	VPS64	YMR052W	FAR3
YBL058W	SHP1	YLR320W	MMS22
YHR056C	RSC30	YMR038C	CCS1
YMR095C	SNO1	YMR311C	GLC8

Predictions at threshold level 0.75 continued...			
Gene1 of predicted pair		Gene2 of predicted pair	
Systematic Name	Standard Name	Systematic Name	Standard Name
YPL256C	CLN2	YMR052W	FAR3
YCR077C	PAT1	YER122C	GLO3
YOL111C	MDY2	YDR207C	UME6
YGL115W	SNF4	YLR371W	ROM2
YEL053C	MAK10	YDR289C	RTT103
YMR016C	SOK2	YBL103C	RTG3
YJR040W	GEF1	YAR014C	BUD14
YPR131C	NAT3	YKR024C	DBP7
YOR109W	INP53	YNL041C	COG6
YNL325C	FIG4	YDR207C	UME6
YGL212W	VAM7	YLR180W	SAM1
YER019C-A	SBH2	YHR200W	RPN10
YBR108W	YBR108W	YLR093C	NYV1
YKR014C	YPT52	YBR082C	UBC4
YBR126C	TPS1	YFL023W	BUD27
YCR065W	HCM1	YLR452C	SST2
YKL048C	ELM1	YHR114W	BZZ1
YEL027W	CUP5	YER072W	VTC1
YIL007C	NAS2	YER105C	NUP157
YFR031C-A	RPL2A	YBL025W	RRN10
YOR275C	RIM20	YHR115C	DMA1
YNL106C	INP52	YLR135W	SLX4
YOR195W	SLK19	YOR141C	ARP8
YNL273W	TOF1	YHR056C	RSC30
YBL025W	RRN10	YCR053W	THR4
YDL155W	CLB3	YJL095W	BCK1
YGR019W	UGA1	YDR289C	RTT103
YLR064W	YLR064W	YOR284W	HUA2
YDR183W	PLP1	YJL190C	RPS22A

Predictions at threshold level 0.75 continued...			
Gene1 of predicted pair		Gene2 of predicted pair	
Systematic Name	Standard Name	Systematic Name	Standard Name
YER115C	SPR6	YGR059W	SPR3
YPL037C	EGD1	YNR032W	PPG1
YGR252W	GCN5	YHL011C	PRS3
YKL126W	YPK1	YHR081W	LRP1
YBR200W	BEM1	YBL085W	BOI1
YAL024C	LTE1	YGL124C	MON1
YGL060W	YBP2	YBR194W	SOY1
YFR031C-A	RPL2A	YMR095C	SNO1
YMR153W	NUP53	YGR059W	SPR3
YGL095C	VPS45	YOL018C	TLG2
YNR052C	POP2	YDL013W	SLX5
YEL043W	YEL043W	YKR030W	GMH1
YGL151W	NUT1	YHR114W	BZZ1
YBR082C	UBC4	YGL031C	RPL24A
YKR099W	BAS1	YJL095W	BCK1
YOR270C	VPH1	YMR214W	SCJ1
YBR108W	YBR108W	YPR030W	CSR2
YBL103C	RTG3	YOR035C	SHE4
YCR088W	ABP1	YLR429W	CRN1
YKR061W	KTR2	YNL307C	MCK1
YER115C	SPR6	YML057W	CMP2
YBR131W	CCZ1	YJR033C	RAV1
YPR120C	CLB5	YDL006W	PTC1
YDR245W	MNN10	YKL048C	ELM1
YDR183W	PLP1	YOR141C	ARP8
YER001W	MNN1	YBL072C	RPS8A
YNR052C	POP2	YGL031C	RPL24A
YER120W	SCS2	YJL004C	SYS1
YBR228W	SLX1	YCR086W	CSM1

Predictions at threshold level 0.75 continued...			
**Gene1 of predicted pair**		**Gene2 of predicted pair**	
**Systematic Name**	**Standard Name**	**Systematic Name**	**Standard Name**
YCL067C	HMLALPHA2	YKL181W	PRS1
YMR142C	RPL13B	YGL124C	MON1
YJR140C	HIR3	YOR038C	HIR2
YDL247W	MPH2	YPL240C	HSP82
YLR452C	SST2	YGL124C	MON1
YLR399C	BDF1	YGL163C	RAD54
YEL065W	SIT1	YLR306W	UBC12
YHR200W	RPN10	YLR306W	UBC12
YBR131W	CCZ1	YPR028W	YOP1
YOR109W	INP53	YGR105W	VMA21
YBR200W	BEM1	YJL190C	RPS22A
YDR309C	GIC2	YHR115C	DMA1
YLR337C	VRP1	YNL288W	CAF40
YLR441C	RPS1A	YBL025W	RRN10
YMR224C	MRE11	YHR039C-A	VMA10
YBL103C	RTG3	YKR099W	BAS1
YLR093C	NYV1	YBL025W	RRN10
YER116C	SLX8	YJR035W	RAD26
YLR288C	MEC3	YBR173C	UMP1
YNL325C	FIG4	YKR099W	BAS1
YPL195W	APL5	YOR035C	SHE4
YJL124C	LSM1	YLR315W	NKP2
YPL057C	SUR1	YDL223C	HBT1
YOL041C	NOP12	YNL175C	NOP13
YER139C	YER139C	YLR370C	ARC18
YGL087C	MMS2	YER116C	SLX8
YMR026C	PEX12	YMR153W	NUP53
YOR127W	RGA1	YER122C	GLO3
YMR095C	SNO1	YNL325C	FIG4

Predictions at threshold level 0.75 continued...			
Gene1 of predicted pair		Gene2 of predicted pair	
Systematic Name	Standard Name	Systematic Name	Standard Name
YFR031C-A	RPL2A	YCR020C-A	MAK31
YPR036W	VMA13	YNL041C	COG6
YPL240C	HSP82	YHR187W	IKI1
YDR363W-A	SEM1	YLR315W	NKP2
YLR399C	BDF1	YKL048C	ELM1
YBR228W	SLX1	YGR071C	YGR071C
YMR224C	MRE11	YOL030W	GAS5
YNL199C	GCR2	YER096W	SHC1
YKL126W	YPK1	YBR280C	SAF1
YEL053C	MAK10	YNL041C	COG6
YGL003C	CDH1	YLR032W	RAD5
YLR192C	HCR1	YNL238W	KEX2
YGR072W	UPF3	YCL001W	RER1
YGL066W	SGF73	YDR318W	MCM21
YKR099W	BAS1	YLR320W	MMS22
YGL066W	SGF73	YBR111W-A	SUS1
YCR066W	RAD18	YPL161C	BEM4
YKL126W	YPK1	YJL164C	TPK1
YJR059W	PTK2	YCR024C-A	PMP1
YJL136C	RPS21B	YDR363W-A	SEM1
YIL076W	SEC28	YHR060W	VMA22
YBR215W	HPC2	YLR315W	NKP2
YKL025C	PAN3	YDR155C	CPR1
YLR090W	XDJ1	YML115C	VAN1
YGL252C	RTG2	YOR208W	PTP2
YJL176C	SWI3	YHR013C	ARD1
YML001W	YPT7	YKR030W	GMH1
YLR135W	SLX4	YER116C	SLX8
YIL156W	UBP7	YPL246C	RBD2

Predictions at threshold level 0.75 continued...			
Gene1 of predicted pair		Gene2 of predicted pair	
Systematic Name	Standard Name	Systematic Name	Standard Name
YHL031C	GOS1	YLR447C	VMA6
YBR057C	MUM2	YJR135C	MCM22
YML115C	VAN1	YFL023W	BUD27
YBR173C	UMP1	YLR441C	RPS1A
YPR017C	DSS4	YDR153C	ENT5
YPR036W	VMA13	YPR030W	CSR2
YLR234W	TOP3	YNL147W	LSM7
YLL039C	UBI4	YDR358W	GGA1
YBL051C	PIN4	YKR048C	NAP1
YDL052C	SLC1	YMR246W	FAA4
YDR192C	NUP42	YML007W	YAP1
YFR010W	UBP6	YCR053W	THR4
YIL159W	BNR1	YNL325C	FIG4
YBL047C	EDE1	YBL101C	ECM21
YER144C	UBP5	YML071C	COG8
YBL058W	SHP1	YBR200W	BEM1
YPR120C	CLB5	YDL226C	GCS1
YDR440W	DOT1	YBL025W	RRN10
YLR441C	RPS1A	YDR310C	SUM1
YOR312C	RPL20B	YMR089C	YTA12
YJL176C	SWI3	YKL006W	RPL14A
YPR120C	CLB5	YBR158W	AMN1
YGR072W	UPF3	YCL037C	SRO9
YKL213C	DOA1	YEL013W	VAC8
YNL201C	PSY2	YJR060W	CBF1
YCR086W	CSM1	YOL081W	IRA2
YJR056C	YJR056C	YDR261C	EXG2
YNL041C	COG6	YMR230W	RPS10B
YEL056W	HAT2	YGL148W	ARO2

Predictions at threshold level 0.75 continued...			
Gene1 of predicted pair		Gene2 of predicted pair	
Systematic Name	Standard Name	Systematic Name	Standard Name
YPL057C	SUR1	YDL155W	CLB3
YER122C	GLO3	YER040W	GLN3
YIR009W	MSL1	YDR289C	RTT103
YJL095W	BCK1	YNL107W	YAF9
YDR443C	SSN2	YCR081W	SRB8
YMR224C	MRE11	YBR217W	ATG12
YFR032C-A	RPL29	YBL101C	ECM21
YPL022W	RAD1	YDR254W	CHL4
YNL218W	MGS1	YGL031C	RPL24A
YIL053W	RHR2	YGL212W	VAM7
YEL065W	SIT1	YCR053W	THR4
YER116C	SLX8	YJL187C	SWE1
YLR079W	SIC1	YNL199C	GCR2
YPR084W	YPR084W	YER139C	YER139C
YCR065W	HCM1	YFR010W	UBP6
YKR014C	YPT52	YLR093C	NYV1
YIL053W	RHR2	YDR261C	EXG2
YER054C	GIP2	YML016C	PPZ1
YJR032W	CPR7	YER162C	RAD4
YMR080C	NAM7	YML103C	NUP188
YPL259C	APM1	YDL192W	ARF1
YHR081W	LRP1	YJR035W	RAD26
YMR109W	MYO5	YBR260C	RGD1
YGL178W	MPT5	YJL127C	SPT10
YJL095W	BCK1	YGL031C	RPL24A
YJR060W	CBF1	YFR036W	CDC26
YPL115C	BEM3	YPL161C	BEM4
YPR018W	RLF2	YNL079C	TPM1
YLR216C	CPR6	YKR048C	NAP1

Predictions at threshold level 0.75 continued...			
Gene1 of predicted pair		Gene2 of predicted pair	
Systematic Name	Standard Name	Systematic Name	Standard Name
YBR065C	ECM2	YMR216C	SKY1
YDR129C	SAC6	YDR378C	LSM6
YHR052W	CIC1	YHR010W	RPL27A
YDR080W	VPS41	YPL105C	YPL105C
YNL076W	MKS1	YBR036C	CSG2
YNL199C	GCR2	YOR308C	SNU66
YJL172W	CPS1	YBR171W	SEC66
YGL133W	ITC1	YPL256C	CLN2
YEL009C	GCN4	YFR034C	PHO4
YDL083C	RPS16B	YMR214W	SCJ1
YOR127W	RGA1	YJL190C	RPS22A
YAL024C	LTE1	YGL033W	HOP2
YPL161C	BEM4	YOR035C	SHE4
YKL025C	PAN3	YDR254W	CHL4
YLR360W	VPS38	YML071C	COG8
YHR156C	LIN1	YPL037C	EGD1
YOR038C	HIR2	YDR289C	RTT103
YNL032W	SIW14	YKL048C	ELM1
YDR295C	HDA2	YOR195W	SLK19
YNL209W	SSB2	YDR477W	SNF1
YPL213W	LEA1	YHL002W	HSE1
YPR018W	RLF2	YML028W	TSA1
YNL307C	MCK1	YLR370C	ARC18
YLR360W	VPS38	YBR260C	RGD1
YMR202W	ERG2	YDR171W	HSP42
YOL145C	CTR9	YJR060W	CBF1
YKL126W	YPK1	YBL025W	RRN10
YJL095W	BCK1	YHL011C	PRS3
YDR293C	SSD1	YNL107W	YAF9

Predictions at threshold level 0.75 continued...			
Gene1 of predicted pair		Gene2 of predicted pair	
Systematic Name	Standard Name	Systematic Name	Standard Name
YGR108W	CLB1	YPR051W	MAK3
YML115C	VAN1	YOR008C	SLG1
YFR010W	UBP6	YBR173C	UMP1
YNL032W	SIW14	YLR320W	MMS22
YDR383C	NKP1	YDR335W	MSN5
YBR194W	SOY1	YDR171W	HSP42
YGR059W	SPR3	YML057W	CMP2
YDL020C	RPN4	YNL166C	BNI5
YPL161C	BEM4	YLR093C	NYV1
YJR059W	PTK2	YKR024C	DBP7
YJR060W	CBF1	YER162C	RAD4
YOR332W	VMA4	YPL234C	TFP3
YLR452C	SST2	YPL256C	CLN2
YLR240W	VPS34	YDL161W	ENT1
YOR106W	VAM3	YOR014W	RTS1
YBR217W	ATG12	YBR030W	YBR030W
YGL087C	MMS2	YHR013C	ARD1
YJR040W	GEF1	YML001W	YPT7
YHR060W	VMA22	YKL080W	VMA5
YDR129C	SAC6	YBL072C	RPS8A
YNL031C	HHT2	YPR072W	NOT5
YGL087C	MMS2	YDR318W	MCM21
YLR192C	HCR1	YMR016C	SOK2
YML013W	UBX2	YGL003C	CDH1
YBR107C	IML3	YLR254C	NDL1
YBL078C	ATG8	YPL057C	SUR1
YDR176W	NGG1	YGL221C	NIF3
YLR360W	VPS38	YHR114W	BZZ1
YEL065W	SIT1	YOR290C	SNF2

Predictions at threshold level 0.75 continued...			
Gene1 of predicted pair		Gene2 of predicted pair	
Systematic Name	Standard Name	Systematic Name	Standard Name
YGL133W	ITC1	YGL163C	RAD54
YGL115W	SNF4	YLR429W	CRN1
YKR035W-A	DID2	YMR311C	GLC8
YER116C	SLX8	YJL095W	BCK1
YLR094C	GIS3	YFR036W	CDC26
YLR381W	CTF3	YOL054W	PSH1
YBR126C	TPS1	YER116C	SLX8
YBL088C	TEL1	YNR052C	POP2
YDR293C	SSD1	YDL192W	ARF1
YDR318W	MCM21	YEL056W	HAT2
YDL116W	NUP84	YIL156W	UBP7
YBL021C	HAP3	YJL190C	RPS22A
YER044C	ERG28	YOL081W	IRA2
YKL139W	CTK1	YKR048C	NAP1
YBR173C	UMP1	YFL023W	BUD27
YDR443C	SSN2	YPL256C	CLN2
YLR090W	XDJ1	YMR152W	YIM1
YLR386W	VAC14	YLR370C	ARC18
YDL155W	CLB3	YBR114W	RAD16
YMR095C	SNO1	YFR010W	UBP6
YJL176C	SWI3	YDR358W	GGA1
YCR024C-A	PMP1	YNL199C	GCR2
YBR173C	UMP1	YDR358W	GGA1
YDL020C	RPN4	YDR254W	CHL4
YNL243W	SLA2	YLR064W	YLR064W
YIL076W	SEC28	YMR152W	YIM1
YOR358W	HAP5	YKR024C	DBP7
YHR081W	LRP1	YPR046W	MCM16
YPL161C	BEM4	YEL051W	VMA8

Predictions at threshold level 0.75 continued...			
Gene1 of predicted pair		Gene2 of predicted pair	
Systematic Name	Standard Name	Systematic Name	Standard Name
YOR023C	AHC1	YOR358W	HAP5
YPL024W	RMI1	YDR076W	RAD55
YPL024W	RMI1	YDR071C	PAA1
YGL078C	DBP3	YLR386W	VAC14
YBR030W	YBR030W	YLR180W	SAM1
YML058W	SML1	YBR274W	CHK1
YDR320C	SWA2	YPL037C	EGD1
YKR061W	KTR2	YLR182W	SWI6
YDR448W	ADA2	YLR399C	BDF1
YJL154C	VPS35	YDR293C	SSD1
YLR094C	GIS3	YBR280C	SAF1
YDL020C	RPN4	YKR001C	VPS1
YHR052W	CIC1	YKR024C	DBP7
YLR386W	VAC14	YGL025C	PGD1
YNL206C	RTT106	YHR200W	RPN10
YDL002C	NHP10	YOR043W	WHI2
YLR315W	NKP2	YFR036W	CDC26
YFR030W	MET10	YNL116W	DMA2
YPL024W	RMI1	YLR032W	RAD5
YGR072W	UPF3	YNL297C	MON2
YJL154C	VPS35	YHR114W	BZZ1
YKL139W	CTK1	YHR039C-A	VMA10
YHR171W	ATG7	YLR093C	NYV1
YOR058C	ASE1	YGL190C	CDC55
YIL008W	URM1	YNL107W	YAF9
YPL129W	TAF14	YJL124C	LSM1
YMR004W	MVP1	YDR310C	SUM1
YNL291C	MID1	YDL155W	CLB3
YMR142C	RPL13B	YBL025W	RRN10

Predictions at threshold level 0.75 continued...			
Gene1 of predicted pair		Gene2 of predicted pair	
Systematic Name	Standard Name	Systematic Name	Standard Name
YLR090W	XDJ1	YJR084W	CSN12
YHR200W	RPN10	YLR182W	SWI6
YDR293C	SSD1	YMR109W	MYO5
YPL253C	VIK1	YLR447C	VMA6
YLL007C	YLL007C	YPL115C	BEM3
YLR018C	POM34	YFL047W	RGD2
YBR188C	NTC20	YKL074C	MUD2
YLR381W	CTF3	YLR410W	VIP1
YPL195W	APL5	YOL081W	IRA2
YOL018C	TLG2	YER122C	GLO3
YJR056C	YJR056C	YDL223C	HBT1
YBR195C	MSI1	YPR120C	CLB5
YGL237C	HAP2	YBR107C	IML3
YER114C	BOI2	YER124C	DSE1
YBR194W	SOY1	YJR005W	APL1
YJR058C	APS2	YDL006W	PTC1
YOR337W	TEA1	YGL124C	MON1
YER161C	SPT2	YOR124C	UBP2
YBL079W	NUP170	YJR035W	RAD26
YGL197W	MDS3	YDL155W	CLB3
YHR039C-A	VMA10	YLR180W	SAM1
YNL238W	KEX2	YEL060C	PRB1
YGL033W	HOP2	YNL238W	KEX2
YGL087C	MMS2	YLR292C	SEC72
YDR451C	YHP1	YCR040W	MATALPHA1
YHR060W	VMA22	YNL107W	YAF9
YNL041C	COG6	YOL108C	INO4
YMR198W	CIK1	YKL114C	APN1
YMR167W	MLH1	YOL081W	IRA2

Predictions at threshold level 0.75 continued...			
Gene1 of predicted pair		Gene2 of predicted pair	
Systematic Name	Standard Name	Systematic Name	Standard Name
YPR018W	RLF2	YNL117W	MLS1
YDR378C	LSM6	YDR318W	MCM21
YOR324C	FRT1	YLR433C	CNA1
YML058W	SML1	YML006C	GIS4
YNL032W	SIW14	YDR358W	GGA1
YDL116W	NUP84	YOL030W	GAS5
YDR320C	SWA2	YDL077C	VAM6
YOR270C	VPH1	YDR202C	RAV2
YJL176C	SWI3	YJL183W	MNN11
YEL065W	SIT1	YMR230W	RPS10B
YEL053C	MAK10	YNL238W	KEX2
YGR020C	VMA7	YOR109W	INP53
YPL234C	TFP3	YGR105W	VMA21
YLR320W	MMS22	YOR368W	RAD17
YNL025C	SSN8	YCR077C	PAT1
YJR040W	GEF1	YPL161C	BEM4
YAL024C	LTE1	YDR207C	UME6
YNL041C	COG6	YPR046W	MCM16
YLR386W	VAC14	YMR199W	CLN1
YDR293C	SSD1	YKR028W	SAP190
YNL206C	RTT106	YDR379W	RGA2
YCL034W	LSB5	YPR119W	CLB2
YNL087W	TCB2	YHR200W	RPN10
YGL135W	RPL1B	YLR131C	ACE2
YDR335W	MSN5	YCR086W	CSM1
YPL024W	RMI1	YEL060C	PRB1
YNL068C	FKH2	YGL031C	RPL24A
YPL161C	BEM4	YJL187C	SWE1
YPL042C	SSN3	YDR245W	MNN10

Predictions at threshold level 0.75 continued...			
Gene1 of predicted pair		Gene2 of predicted pair	
Systematic Name	Standard Name	Systematic Name	Standard Name
YDL101C	DUN1	YOL043C	NTG2
YLR079W	SIC1	YLR315W	NKP2
YGL174W	BUD13	YER139C	YER139C
YPR051W	MAK3	YGL124C	MON1
YPR084W	YPR084W	YKL139W	CTK1
YDR469W	SDC1	YGL003C	CDH1
YDR156W	RPA14	YDR289C	RTT103
YJL124C	LSM1	YDR440W	DOT1
YCL027W	FUS1	YER032W	FIR1
YML095C	RAD10	YMR055C	BUB2
YGL087C	MMS2	YOR132W	VPS17
YPR131C	NAT3	YJR060W	CBF1
YJL204C	RCY1	YEL051W	VMA8
YBR173C	UMP1	YBR280C	SAF1
YDL161W	ENT1	YOR035C	SHE4
YPL115C	BEM3	YLR337C	VRP1
YML051W	GAL80	YPL248C	GAL4
YPL195W	APL5	YEL051W	VMA8
YDR379W	RGA2	YGR135W	PRE9
YPL050C	MNN9	YKL041W	VPS24
YCR020C-A	MAK31	YLR360W	VPS38
YDR156W	RPA14	YLR410W	VIP1
YGR108W	CLB1	YOR023C	AHC1
YNL229C	URE2	YKL101W	HSL1
YPR017C	DSS4	YHL020C	OPI1
YER068W	MOT2	YBR010W	HHT1
YLR315W	NKP2	YEL013W	VAC8
YJL029C	VPS53	YOR036W	PEP12
YMR055C	BUB2	YDR310C	SUM1

Predictions at threshold level 0.75 continued...			
Gene1 of predicted pair		Gene2 of predicted pair	
Systematic Name	Standard Name	Systematic Name	Standard Name
YGL115W	SNF4	YLR309C	IMH1
YER139C	YER139C	YNL076W	MKS1
YGL223C	COG1	YPL050C	MNN9
YHR081W	LRP1	YLR370C	ARC18
YGL078C	DBP3	YGL124C	MON1
YJL183W	MNN11	YMR038C	CCS1
YOR035C	SHE4	YLR398C	SKI2
YHR144C	DCD1	YPL240C	HSP82
YGR072W	UPF3	YGL223C	COG1
YLR234W	TOP3	YDR183W	PLP1
YDR153C	ENT5	YDR310C	SUM1
YMR026C	PEX12	YGL003C	CDH1
YLR058C	SHM2	YMR202W	ERG2
YBL079W	NUP170	YDR207C	UME6
YDR320C	SWA2	YDR207C	UME6
YHR013C	ARD1	YML071C	COG8
YPL105C	YPL105C	YJR033C	RAV1
YBR274W	CHK1	YJL124C	LSM1
YKL025C	PAN3	YNL107W	YAF9
YGL251C	HFM1	YDR378C	LSM6
YMR199W	CLN1	YFL047W	RGD2
YLR120C	YPS1	YHL031C	GOS1
YGR072W	UPF3	YJR040W	GEF1
YFL023W	BUD27	YJR090C	GRR1
YDL006W	PTC1	YHR108W	GGA2
YDR320C	SWA2	YCL037C	SRO9
YHL020C	OPI1	YPR032W	SRO7
YIL159W	BNR1	YLR180W	SAM1
YPL115C	BEM3	YDR144C	MKC7

Predictions at threshold level 0.75 continued...			
**Gene1 of predicted pair**		**Gene2 of predicted pair**	
**Systematic Name**	**Standard Name**	**Systematic Name**	**Standard Name**
YDL020C	RPN4	YIL007C	NAS2
YML016C	PPZ1	YLR263W	RED1
YJL187C	SWE1	YJR090C	GRR1
YPL096C-A	ERI1	YNL271C	BNI1
YCR020C-A	MAK31	YDL077C	VAM6
YJL124C	LSM1	YDR207C	UME6
YLR410W	VIP1	YJR135C	MCM22
YIL159W	BNR1	YBR171W	SEC66
YCR063W	BUD31	YPL213W	LEA1
YKL116C	PRR1	YPL057C	SUR1
YGR133W	PEX4	YDR265W	PEX10
YJL060W	BNA3	YBR194W	SOY1
YOL121C	RPS19A	YMR152W	YIM1
YIL053W	RHR2	YNL032W	SIW14
YMR055C	BUB2	YBR195C	MSI1
YLR119W	SRN2	YNL238W	KEX2
YDR443C	SSN2	YFR036W	CDC26
YDR289C	RTT103	YGL246C	RAI1
YFR031C-A	RPL2A	YJL172W	CPS1
YMR153W	NUP53	YBL025W	RRN10
YJR140C	HIR3	YBR195C	MSI1
YKR020W	VPS51	YKL119C	VPH2
YPL037C	EGD1	YMR152W	YIM1
YCR034W	FEN1	YKR059W	TIF1
YMR214W	SCJ1	YMR230W	RPS10B
YLR119W	SRN2	YOR035C	SHE4
YFR031C-A	RPL2A	YER116C	SLX8
YDL006W	PTC1	YMR054W	STV1
YOL111C	MDY2	YLR320W	MMS22

Predictions at threshold level 0.75 continued...			
Gene1 of predicted pair		Gene2 of predicted pair	
Systematic Name	Standard Name	Systematic Name	Standard Name
YMR026C	PEX12	YKL139W	CTK1
YOR069W	VPS5	YJL004C	SYS1
YBR215W	HPC2	YLR350W	ORM2
YFR032C-A	RPL29	YDR245W	MNN10
YLL007C	YLL007C	YHR114W	BZZ1
YDL006W	PTC1	YPL256C	CLN2
YNL307C	MCK1	YGL115W	SNF4
YNL243W	SLA2	YDL047W	SIT4
YLR095C	IOC2	YJL172W	CPS1
YHR073W	OSH3	YNL199C	GCR2
YJL172W	CPS1	YDL223C	HBT1
YLR234W	TOP3	YGL031C	RPL24A
YDR108W	GSG1	YKR020W	VPS51
YLR240W	VPS34	YJL154C	VPS35
YIL159W	BNR1	YMR109W	MYO5
YPL195W	APL5	YBR280C	SAF1
YPL057C	SUR1	YDR358W	GGA1
YPR052C	NHP6A	YNL030W	HHF2
YPR084W	YPR084W	YDR153C	ENT5
YDR174W	HMO1	YJR060W	CBF1
YIL076W	SEC28	YLR320W	MMS22
YIL159W	BNR1	YBR173C	UMP1
YCL037C	SRO9	YML001W	YPT7
YCR081W	SRB8	YLR360W	VPS38
YPL018W	CTF19	YDL047W	SIT4
YLR320W	MMS22	YJR032W	CPR7
YOR069W	VPS5	YNL044W	YIP3
YER044C	ERG28	YJL095W	BCK1
YFR036W	CDC26	YPL195W	APL5

Predictions at threshold level 0.75 continued...			
Gene1 of predicted pair		Gene2 of predicted pair	
Systematic Name	Standard Name	Systematic Name	Standard Name
YBR114W	RAD16	YAR014C	BUD14
YLR337C	VRP1	YGL200C	EMP24
YDR265W	PEX10	YGL153W	PEX14
YKR059W	TIF1	YOR039W	CKB2
YNL106C	INP52	YGR071C	YGR071C
YNL068C	FKH2	YKL032C	IXR1
YJL124C	LSM1	YNL236W	SIN4
YHR084W	STE12	YPL220W	RPL1A
YJL036W	SNX4	YDL006W	PTC1
YNL297C	MON2	YPL195W	APL5
YOR069W	VPS5	YGL115W	SNF4
YLR386W	VAC14	YNL054W	VAC7
YPL115C	BEM3	YIL008W	URM1
YJL053W	PEP8	YER122C	GLO3
YGL212W	VAM7	YLR371W	ROM2
YFR031C-A	RPL2A	YGL033W	HOP2
YOR080W	DIA2	YDR171W	HSP42
YML063W	RPS1B	YLR441C	RPS1A
YGR020C	VMA7	YLR309C	IMH1
YKL214C	YRA2	YCL011C	GBP2
YKR035W-A	DID2	YGL124C	MON1
YML103C	NUP188	YOL081W	IRA2
YLR090W	XDJ1	YNL032W	SIW14
YER139C	YER139C	YER122C	GLO3
YKL025C	PAN3	YOR023C	AHC1
YOR195W	SLK19	YNL225C	CNM67
YOR132W	VPS17	YML001W	YPT7
YPL129W	TAF14	YDR289C	RTT103
YOR035C	SHE4	YPL259C	APM1

Predictions at threshold level 0.75 continued...			
Gene1 of predicted pair		Gene2 of predicted pair	
Systematic Name	Standard Name	Systematic Name	Standard Name
YFR010W	UBP6	YDL083C	RPS16B
YDR245W	MNN10	YJL124C	LSM1
YGL147C	RPL9A	YGL246C	RAI1
YLR386W	VAC14	YDR130C	FIN1
YGR072W	UPF3	YPR051W	MAK3
YPR018W	RLF2	YFL023W	BUD27
YGL153W	PEX14	YBL072C	RPS8A
YER114C	BOI2	YMR055C	BUB2
YLR292C	SEC72	YPR046W	MCM16
YHR205W	SCH9	YHR012W	VPS29
YNL242W	ATG2	YDL149W	ATG9
YOR337W	TEA1	YGL033W	HOP2
YFL026W	STE2	YDR264C	AKR1
YJR024C	MDE1	YER115C	SPR6
YEL009C	GCN4	YNL107W	YAF9
YKL025C	PAN3	YJL154C	VPS35
YOL145C	CTR9	YGR135W	PRE9
YHR060W	VMA22	YLR370C	ARC18
YPL161C	BEM4	YPL089C	RLM1
YMR284W	YKU70	YDR076W	RAD55
YDL184C	RPL41A	YPL137C	GIP3
YML001W	YPT7	YOR035C	SHE4
YDR129C	SAC6	YOL081W	IRA2
YBR239C	YBR239C	YPR119W	CLB2
YDR293C	SSD1	YOR132W	VPS17
YOR332W	VMA4	YEL051W	VMA8
YDR174W	HMO1	YGR270W	YTA7
YOR195W	SLK19	YOL054W	PSH1
YGL174W	BUD13	YPL057C	SUR1

Predictions at threshold level 0.75 continued...			
Gene1 of predicted pair		Gene2 of predicted pair	
Systematic Name	Standard Name	Systematic Name	Standard Name
YBL021C	HAP3	YJL183W	MNN11
YNR052C	POP2	YBR107C	IML3
YLR135W	SLX4	YHR077C	NMD2
YLR447C	VMA6	YKL048C	ELM1
YDR439W	LRS4	YBR200W	BEM1
YDR225W	HTA1	YGL066W	SGF73
YNL106C	INP52	YGR184C	UBR1
YOR023C	AHC1	YBR171W	SEC66
YMR153W	NUP53	YBR280C	SAF1
YOR080W	DIA2	YPR046W	MCM16
YPR051W	MAK3	YNL166C	BNI5
YLR453C	RIF2	YCL073C	YCL073C
YIL159W	BNR1	YKL126W	YPK1
YGL025C	PGD1	YHR060W	VMA22
YBR228W	SLX1	YDR207C	UME6
YDR137W	RGP1	YDR318W	MCM21
YDR108W	GSG1	YDL077C	VAM6
YMR224C	MRE11	YMR198W	CIK1
YGL153W	PEX14	YDR440W	DOT1
YLR337C	VRP1	YNL215W	IES2
YIR009W	MSL1	YER096W	SHC1
YBR173C	UMP1	YDR477W	SNF1
YDL101C	DUN1	YMR284W	YKU70
YCR077C	PAT1	YDR440W	DOT1
YLR399C	BDF1	YBR289W	SNF5
YML115C	VAN1	YGL223C	COG1
YLR320W	MMS22	YLR371W	ROM2
YBR200W	BEM1	YGR188C	BUB1
YNL058C	YNL058C	YMR133W	REC114

Predictions at threshold level 0.75 continued...			
Gene1 of predicted pair		Gene2 of predicted pair	
Systematic Name	Standard Name	Systematic Name	Standard Name
YNL058C	YNL058C	YPL037C	EGD1
YCR081W	SRB8	YOR008C	SLG1
YLR381W	CTF3	YBR114W	RAD16
YDR245W	MNN10	YFR036W	CDC26
YNL054W	VAC7	YCR086W	CSM1
YHR111W	UBA4	YDR289C	RTT103
YLR119W	SRN2	YER162C	RAD4
YDR004W	RAD57	YDR318W	MCM21
YPR017C	DSS4	YJR060W	CBF1
YBR171W	SEC66	YMR152W	YIM1
YOR127W	RGA1	YJR033C	RAV1
YML016C	PPZ1	YLR399C	BDF1
YPR084W	YPR084W	YPR051W	MAK3
YER122C	GLO3	YOL108C	INO4
YOR109W	INP53	YDL226C	GCS1
YKL139W	CTK1	YCR053W	THR4
YLR360W	VPS38	YBR107C	IML3
YNL238W	KEX2	YLR180W	SAM1
YPR017C	DSS4	YNL044W	YIP3
YDR389W	SAC7	YOR109W	INP53
YBR108W	YBR108W	YGR270W	YTA7
YIL148W	RPL40A	YER110C	KAP123
YPL125W	KAP120	YER110C	KAP123
YBR107C	IML3	YNL225C	CNM67
YDL020C	RPN4	YDR144C	MKC7
YBR195C	MSI1	YDR310C	SUM1
YDL184C	RPL41A	YOR327C	SNC2
YLR094C	GIS3	YNL238W	KEX2
YBR171W	SEC66	YPL234C	TFP3

Predictions at threshold level 0.75 continued...			
Gene1 of predicted pair		Gene2 of predicted pair	
Systematic Name	Standard Name	Systematic Name	Standard Name
YPL137C	GIP3	YDR310C	SUM1
YHR171W	ATG7	YBL078C	ATG8
YHR060W	VMA22	YJL095W	BCK1
YMR199W	CLN1	YDL059C	RAD59
YOL111C	MDY2	YNL238W	KEX2
YFL047W	RGD2	YPL256C	CLN2
YOR290C	SNF2	YDR358W	GGA1
YBR010W	HHT1	YBL002W	HTB2
YLR216C	CPR6	YGL252C	RTG2
YMR039C	SUB1	YOR368W	RAD17
YJL124C	LSM1	YOR014W	RTS1
YER044C	ERG28	YGL124C	MON1
YJR140C	HIR3	YDR335W	MSN5
YMR246W	FAA4	YKR046C	PET10
YEL065W	SIT1	YLR370C	ARC18
YOR106W	VAM3	YNL107W	YAF9
YKL101W	HSL1	YOR014W	RTS1
YMR095C	SNO1	YER116C	SLX8
YKL007W	CAP1	YMR109W	MYO5
YLR315W	NKP2	YLR254C	NDL1
YPL161C	BEM4	YER139C	YER139C
YBR173C	UMP1	YKL116C	PRR1
YPL057C	SUR1	YLR150W	STM1
YGR178C	PBP1	YGL094C	PAN2
YMR017W	SPO20	YAL030W	SNC1
YPR030W	CSR2	YLR398C	SKI2
YCR034W	FEN1	YOR368W	RAD17
YBR158W	AMN1	YDL138W	RGT2
YLR234W	TOP3	YJL187C	SWE1

Predictions at threshold level 0.75 continued...			
Gene1 of predicted pair		Gene2 of predicted pair	
Systematic Name	Standard Name	Systematic Name	Standard Name
YDR318W	MCM21	YNL147W	LSM7
YLL007C	YLL007C	YNL288W	CAF40
YKR101W	SIR1	YDR227W	SIR4
YCR065W	HCM1	YKL048C	ELM1
YNL236W	SIN4	YMR038C	CCS1
YPL256C	CLN2	YML057W	CMP2
YOR023C	AHC1	YJL190C	RPS22A
YEL053C	MAK10	YDR207C	UME6
YLR337C	VRP1	YMR224C	MRE11
YDR484W	VPS52	YDL223C	HBT1
YDL101C	DUN1	YMR116C	ASC1
YDR293C	SSD1	YJR040W	GEF1
YPR017C	DSS4	YMR054W	STV1
YOR080W	DIA2	YDR207C	UME6
YGL033W	HOP2	YKR099W	BAS1
YMR091C	NPL6	YGR275W	RTT102
YKL068W	NUP100	YML057W	CMP2
YJL124C	LSM1	YDR422C	SIP1
YOR083W	WHI5	YLR381W	CTF3
YKR099W	BAS1	YPL037C	EGD1
YDR225W	HTA1	YDL042C	SIR2
YPR173C	VPS4	YDR486C	VPS60
YDR254W	CHL4	YHR111W	UBA4
YJR140C	HIR3	YBR278W	DPB3
YOR132W	VPS17	YPL105C	YPL105C
YGL215W	CLG1	YAL055W	PEX22
YBR108W	YBR108W	YHR039C-A	VMA10
YDL116W	NUP84	YLR352W	YLR352W
YBR260C	RGD1	YPL256C	CLN2

Predictions at threshold level 0.75 continued...			
Gene1 of predicted pair		Gene2 of predicted pair	
Systematic Name	Standard Name	Systematic Name	Standard Name
YML058W	SML1	YOR368W	RAD17
YGR108W	CLB1	YOR127W	RGA1
YOR127W	RGA1	YDR507C	GIN4
YCR086W	CSM1	YDR318W	MCM21
YFL013C	IES1	YBR215W	HPC2
YPL024W	RMI1	YMR167W	MLH1
YGR014W	MSB2	YER118C	SHO1
YLR337C	VRP1	YIL008W	URM1
YNL238W	KEX2	YML071C	COG8
YLL039C	UBI4	YJL095W	BCK1
YGR054W	YGR054W	YCR086W	CSM1
YAL024C	LTE1	YLR079W	SIC1
YMR153W	NUP53	YDR484W	VPS52
YNL041C	COG6	YJL183W	MNN11
YBR290W	BSD2	YMR125W	STO1
YGR054W	YGR054W	YDR318W	MCM21
YNL288W	CAF40	YJL095W	BCK1
YOR080W	DIA2	YGL003C	CDH1
YFR032C-A	RPL29	YOR275C	RIM20
YOR275C	RIM20	YLR425W	TUS1
YMR198W	CIK1	YGL115W	SNF4
YHR081W	LRP1	YNL076W	MKS1
YOR080W	DIA2	YAL002W	VPS8
YDR108W	GSG1	YPL195W	APL5
YEL053C	MAK10	YEL060C	PRB1
YMR172W	HOT1	YDL155W	CLB3
YNL094W	APP1	YMR253C	YMR253C
YML016C	PPZ1	YHR012W	VPS29
YDR083W	RRP8	YHR081W	LRP1

Predictions at threshold level 0.75 continued...			
Gene1 of predicted pair		Gene2 of predicted pair	
Systematic Name	Standard Name	Systematic Name	Standard Name
YHR012W	VPS29	YMR054W	STV1
YGL200C	EMP24	YBL101C	ECM21
YGL054C	ERV14	YOL018C	TLG2
YBR200W	BEM1	YBR082C	UBC4
YPL213W	LEA1	YJL095W	BCK1
YML038C	YMD8	YDR319C	YDR319C
YMR142C	RPL13B	YLR320W	MMS22
YLR320W	MMS22	YGR135W	PRE9
YPL161C	BEM4	YPL018W	CTF19
YJL006C	CTK2	YDR440W	DOT1
YDR378C	LSM6	YPL018W	CTF19
YDR293C	SSD1	YGR188C	BUB1
YLR337C	VRP1	YGL151W	NUT1
YMR153W	NUP53	YMR164C	MSS11
YDR318W	MCM21	YAL002W	VPS8
YDR225W	HTA1	YPR046W	MCM16
YGL237C	HAP2	YDR144C	MKC7
YIL076W	SEC28	YJR032W	CPR7
YLR447C	VMA6	YDL155W	CLB3
YGL223C	COG1	YOL108C	INO4
YBR173C	UMP1	YNL041C	COG6
YHR200W	RPN10	YER040W	GLN3
YLR441C	RPS1A	YPR046W	MCM16
YER122C	GLO3	YDR310C	SUM1
YDR484W	VPS52	YHR081W	LRP1
YDL127W	PCL2	YDR146C	SWI5
YOR213C	SAS5	YOR308C	SNU66
YBL103C	RTG3	YGL003C	CDH1
YMR133W	REC114	YER162C	RAD4

Predictions at threshold level 0.75 continued...			
Gene1 of predicted pair		Gene2 of predicted pair	
Systematic Name	Standard Name	Systematic Name	Standard Name
YJL176C	SWI3	YGL135W	RPL1B
YPR036W	VMA13	YHR200W	RPN10
YMR039C	SUB1	YPL001W	HAT1
YCR086W	CSM1	YBR114W	RAD16
YIL076W	SEC28	YOR035C	SHE4
YLR150W	STM1	YGR188C	BUB1
YLR337C	VRP1	YOR008C	SLG1
YLR360W	VPS38	YEL036C	ANP1
YBR030W	YBR030W	YEL053C	MAK10
YBR042C	CST26	YKL041W	VPS24
YAL024C	LTE1	YAR014C	BUD14
YFL013C	IES1	YEL013W	VAC8
YDR129C	SAC6	YDR484W	VPS52
YHR111W	UBA4	YGR188C	BUB1
YCL037C	SRO9	YLR315W	NKP2
YKL048C	ELM1	YPL195W	APL5
YNL068C	FKH2	YBR200W	BEM1
YDL077C	VAM6	YEL060C	PRB1
YDL116W	NUP84	YDL226C	GCS1
YCL001W	RER1	YDR153C	ENT5
YBR107C	IML3	YIL103W	DPH1
YFL026W	STE2	YLR206W	ENT2
YLR234W	TOP3	YGR081C	SLX9
YJL154C	VPS35	YGL095C	VPS45
YLR043C	TRX1	YBR158W	AMN1
YLR090W	XDJ1	YJR032W	CPR7
YMR202W	ERG2	YDR080W	VPS41
YOL126C	MDH2	YGL016W	KAP122
YDR320C	SWA2	YAL002W	VPS8

Predictions at threshold level 0.75 continued...			
Gene1 of predicted pair		Gene2 of predicted pair	
Systematic Name	Standard Name	Systematic Name	Standard Name
YIL103W	DPH1	YDR318W	MCM21
YOL111C	MDY2	YDR358W	GGA1
YBR215W	HPC2	YGR188C	BUB1
YKR001C	VPS1	YML071C	COG8
YDL116W	NUP84	YOL091W	SPO21
YGR249W	MGA1	YDR310C	SUM1
YMR202W	ERG2	YKR048C	NAP1
YGR249W	MGA1	YER116C	SLX8
YDL077C	VAM6	YNL051W	COG5
YOR290C	SNF2	YDR137W	RGP1
YLR320W	MMS22	YDR358W	GGA1
YMR068W	AVO2	YPL180W	TCO89
YPR046W	MCM16	YLR254C	NDL1
YFR031C-A	RPL2A	YKL139W	CTK1
YBR107C	IML3	YLR410W	VIP1
YER139C	YER139C	YNL107W	YAF9
YLR386W	VAC14	YJL095W	BCK1
YFR031C-A	RPL2A	YMR054W	STV1
YIL053W	RHR2	YJL172W	CPS1
YML103C	NUP188	YMR055C	BUB2
YIL156W	UBP7	YGR059W	SPR3
YBR127C	VMA2	YJL029C	VPS53
YLR370C	ARC18	YOL081W	IRA2
YKL048C	ELM1	YGL031C	RPL24A
YLR095C	IOC2	YJR084W	CSN12
YOR023C	AHC1	YKL048C	ELM1
YMR172W	HOT1	YDR144C	MKC7
YHR134W	WSS1	YJR032W	CPR7
YPR051W	MAK3	YML001W	YPT7

Predictions at threshold level 0.75 continued...			
Gene1 of predicted pair		Gene2 of predicted pair	
Systematic Name	Standard Name	Systematic Name	Standard Name
YFR010W	UBP6	YOR038C	HIR2
YLR320W	MMS22	YHR081W	LRP1
YNL058C	YNL058C	YHR200W	RPN10
YKR020W	VPS51	YKR014C	YPT52
YJL138C	TIF2	YGL250W	RMR1
YDR439W	LRS4	YLR381W	CTF3
YHR178W	STB5	YJR090C	GRR1
YGR249W	MGA1	YJR084W	CSN12
YEL065W	SIT1	YDL192W	ARF1
YGR054W	YGR054W	YDR144C	MKC7
YFR036W	CDC26	YPR046W	MCM16
YJR024C	MDE1	YBL079W	NUP170
YKL117W	SBA1	YJR032W	CPR7
YDR129C	SAC6	YBL047C	EDE1
YDR443C	SSN2	YJL095W	BCK1
YOR083W	WHI5	YNL236W	SIN4
YPL037C	EGD1	YPR032W	SRO7
YOR071C	THI71	YFL044C	OTU1
YNL094W	APP1	YGR268C	HUA1
YFR024C-A	LSB3	YCR088W	ABP1
YHR060W	VMA22	YKR035W-A	DID2
YMR224C	MRE11	YNL215W	IES2
YDR146C	SWI5	YPL001W	HAT1
YLR079W	SIC1	YDR477W	SNF1
YOR290C	SNF2	YPL037C	EGD1
YGL078C	DBP3	YGR283C	YGR283C
YMR116C	ASC1	YMR284W	YKU70
YKL213C	DOA1	YKL160W	ELF1
YOR001W	RRP6	YIR005W	IST3

Predictions at threshold level 0.75 continued...			
Gene1 of predicted pair		Gene2 of predicted pair	
Systematic Name	Standard Name	Systematic Name	Standard Name
YER144C	UBP5	YPL137C	GIP3
YPR046W	MCM16	YMR230W	RPS10B
YBR200W	BEM1	YKL126W	YPK1
YBR215W	HPC2	YHR039C-A	VMA10
YDR439W	LRS4	YLR399C	BDF1
YOR023C	AHC1	YGL124C	MON1
YFR031C-A	RPL2A	YDR153C	ENT5
YNL201C	PSY2	YHR115C	DMA1
YHR152W	SPO12	YGL031C	RPL24A
YOR001W	RRP6	YDR200C	VPS64
YAL024C	LTE1	YBL008W	HIR1
YML102W	CAC2	YKL160W	ELF1
YBR173C	UMP1	YDR261C	EXG2
YOL126C	MDH2	YPL178W	CBC2
YOR132W	VPS17	YMR055C	BUB2
YNL166C	BNI5	YNL107W	YAF9
YDL059C	RAD59	YBR073W	RDH54
YJR135C	MCM22	YGL253W	HXK2
YDR174W	HMO1	YOL081W	IRA2
YMR216C	SKY1	YEL051W	VMA8
YLR370C	ARC18	YEL036C	ANP1
YFR024C-A	LSB3	YJL151C	SNA3
YGL163C	RAD54	YDL155W	CLB3
YML016C	PPZ1	YGL163C	RAD54
YCR020C-A	MAK31	YDR080W	VPS41
YGR019W	UGA1	YJL095W	BCK1
YMR039C	SUB1	YOR043W	WHI2
YLR079W	SIC1	YDR439W	LRS4
YDL185W	TFP1	YPL234C	TFP3

Predictions at threshold level 0.75 continued...			
Gene1 of predicted pair		Gene2 of predicted pair	
Systematic Name	Standard Name	Systematic Name	Standard Name
YPL024W	RMI1	YGR188C	BUB1
YOR008C	SLG1	YMR311C	GLC8
YDR129C	SAC6	YDL192W	ARF1
YLR386W	VAC14	YLR315W	NKP2
YPL024W	RMI1	YML062C	MFT1
YNL293W	MSB3	YGL198W	YIP4
YBR131W	CCZ1	YLR447C	VMA6
YIL034C	CAP2	YGL124C	MON1
YPL115C	BEM3	YGR054W	YGR054W
YEL044W	IES6	YKL041W	VPS24
YFL023W	BUD27	YDR310C	SUM1
YLR381W	CTF3	YGR184C	UBR1
YHR012W	VPS29	YMR022W	UBC7
YLR079W	SIC1	YOR140W	SFL1
YOR141C	ARP8	YHR115C	DMA1
YDR440W	DOT1	YAL002W	VPS8
YGL038C	OCH1	YJL187C	SWE1
YOR132W	VPS17	YCL037C	SRO9
YDR261C	EXG2	YOR035C	SHE4
YJR135C	MCM22	YGR188C	BUB1
YKL160W	ELF1	YDR289C	RTT103
YDR477W	SNF1	YJR032W	CPR7
YNL201C	PSY2	YMR224C	MRE11
YJL006C	CTK2	YJR005W	APL1
YDL116W	NUP84	YOL054W	PSH1
YOR080W	DIA2	YPL253C	VIK1
YER107C	GLE2	YLR085C	ARP6
YKL116C	PRR1	YLR452C	SST2
YDL184C	RPL41A	YER162C	RAD4

Predictions at threshold level 0.75 continued...			
Gene1 of predicted pair		Gene2 of predicted pair	
Systematic Name	Standard Name	Systematic Name	Standard Name
YDL083C	RPS16B	YNL076W	MKS1
YOR195W	SLK19	YIL036W	CST6
YDL020C	RPN4	YAR014C	BUD14
YPR017C	DSS4	YNL069C	RPL16B
YDL006W	PTC1	YMR089C	YTA12
YPL057C	SUR1	YNR072W	HXT17
YPL161C	BEM4	YKR048C	NAP1
YHR119W	SET1	YGL058W	RAD6
YNL289W	PCL1	YLR425W	TUS1
YNL238W	KEX2	YJL004C	SYS1
YNL054W	VAC7	YML112W	CTK3
YDR264C	AKR1	YJL095W	BCK1
YMR224C	MRE11	YLR357W	RSC2
YJL036W	SNX4	YJR040W	GEF1
YGL147C	RPL9A	YPR030W	CSR2
YGL212W	VAM7	YPL234C	TFP3
YFL023W	BUD27	YCR053W	THR4
YJR032W	CPR7	YEL056W	HAT2
YMR055C	BUB2	YIR005W	IST3
YKL139W	CTK1	YGL174W	BUD13
YER110C	KAP123	YDR318W	MCM21
YGR252W	GCN5	YPL248C	GAL4
YHR073W	OSH3	YCR024C-A	PMP1
YPL022W	RAD1	YPR120C	CLB5
YMR055C	BUB2	YLR032W	RAD5
YBR010W	HHT1	YOR064C	YNG1
YCR066W	RAD18	YDL077C	VAM6
YLR452C	SST2	YPR030W	CSR2
YFR031C-A	RPL2A	YDR358W	GGA1

Predictions at threshold level 0.75 continued...			
Gene1 of predicted pair		Gene2 of predicted pair	
Systematic Name	Standard Name	Systematic Name	Standard Name
YML115C	VAN1	YKL041W	VPS24
YER139C	YER139C	YBL025W	RRN10
YGR072W	UPF3	YMR004W	MVP1
YMR224C	MRE11	YBL058W	SHP1
YPL024W	RMI1	YHR178W	STB5
YJL172W	CPS1	YHR039C-A	VMA10
YDR261C	EXG2	YPR030W	CSR2
YGR019W	UGA1	YJL183W	MNN11
YNL069C	RPL16B	YPL037C	EGD1
YER122C	GLO3	YLR350W	ORM2
YNR052C	POP2	YJR060W	CBF1
YLR288C	MEC3	YML102W	CAC2
YDR395W	SXM1	YDL116W	NUP84
YOR195W	SLK19	YOR275C	RIM20
YNL201C	PSY2	YNL116W	DMA2
YPL158C	YPL158C	YPL256C	CLN2
YJR140C	HIR3	YKL101W	HSL1
YAL024C	LTE1	YKL101W	HSL1
YPR084W	YPR084W	YDR378C	LSM6
YBL102W	SFT2	YML038C	YMD8
YHR178W	STB5	YML097C	VPS9
YOR127W	RGA1	YGL038C	OCH1
YBR200W	BEM1	YGL252C	RTG2
YLR109W	AHP1	YML028W	TSA1
YLR447C	VMA6	YDR254W	CHL4
YKL139W	CTK1	YJL183W	MNN11
YGL223C	COG1	YER122C	GLO3
YOR337W	TEA1	YGR249W	MGA1
YNL058C	YNL058C	YPL137C	GIP3

Predictions at threshold level 0.75 continued...			
Gene1 of predicted pair		Gene2 of predicted pair	
Systematic Name	Standard Name	Systematic Name	Standard Name
YGL190C	CDC55	YEL003W	GIM4
YDL020C	RPN4	YJL190C	RPS22A
YDL135C	RDI1	YIL007C	NAS2
YIL036W	CST6	YDR335W	MSN5
YNL106C	INP52	YCL037C	SRO9
YDL006W	PTC1	YLR425W	TUS1
YFR010W	UBP6	YOR243C	PUS7
YPL115C	BEM3	YJL157C	FAR1
YDR004W	RAD57	YBR036C	CSG2
YBL025W	RRN10	YKL041W	VPS24
YLR191W	PEX13	YPR119W	CLB2
YKL139W	CTK1	YMR230W	RPS10B
YLR090W	XDJ1	YAL024C	LTE1
YPL106C	SSE1	YPR119W	CLB2
YPR046W	MCM16	YKL114C	APN1
YOR195W	SLK19	YBR215W	HPC2
YLR119W	SRN2	YER116C	SLX8
YBL058W	SHP1	YIL076W	SEC28
YJL053W	PEP8	YDR153C	ENT5
YNL054W	VAC7	YPR046W	MCM16
YGL178W	MPT5	YGL058W	RAD6
YKL126W	YPK1	YER162C	RAD4
YEL065W	SIT1	YLR094C	GIS3
YLR386W	VAC14	YPL256C	CLN2
YDL101C	DUN1	YER089C	PTC2
YKR061W	KTR2	YNL116W	DMA2
YNL201C	PSY2	YMR055C	BUB2
YFR031C-A	RPL2A	YCR066W	RAD18
YPR051W	MAK3	YPL120W	VPS30

Predictions at threshold level 0.75 continued...			
Gene1 of predicted pair		Gene2 of predicted pair	
Systematic Name	Standard Name	Systematic Name	Standard Name
YGR275W	RTT102	YHL025W	SNF6
YPR084W	YPR084W	YOR001W	RRP6
YGL078C	DBP3	YFR036W	CDC26
YNL307C	MCK1	YDL083C	RPS16B
YER116C	SLX8	YOR035C	SHE4
YMR109W	MYO5	YIL008W	URM1
YGL025C	PGD1	YER044C	ERG28
YDL006W	PTC1	YDR207C	UME6
YKR001C	VPS1	YLR309C	IMH1
YGL250W	RMR1	YCR031C	RPS14A
YLR170C	APS1	YOL062C	APM4
YDR004W	RAD57	YBR173C	UMP1
YGL147C	RPL9A	YGL115W	SNF4
YBR171W	SEC66	YDL077C	VAM6
YIL053W	RHR2	YGL124C	MON1
YML038C	YMD8	YOL018C	TLG2
YDL013W	SLX5	YML028W	TSA1
YKL139W	CTK1	YML016C	PPZ1
YER044C	ERG28	YMR152W	YIM1
YGL223C	COG1	YNL238W	KEX2
YHR039C-A	VMA10	YKL119C	VPH2
YKL116C	PRR1	YDL223C	HBT1
YDL113C	ATG20	YJL036W	SNX4
YFR031C-A	RPL2A	YLR371W	ROM2
YMR142C	RPL13B	YLR093C	NYV1
YDR174W	HMO1	YKL048C	ELM1
YIL103W	DPH1	YNL116W	DMA2
YML038C	YMD8	YOR327C	SNC2
YFR031C-A	RPL2A	YBR019C	GAL10

Predictions at threshold level 0.75 continued...			
Gene1 of predicted pair		Gene2 of predicted pair	
Systematic Name	Standard Name	Systematic Name	Standard Name
YPL129W	TAF14	YJL140W	RPB4
YDL056W	MBP1	YPR119W	CLB2
YHR178W	STB5	YJR135C	MCM22
YMR153W	NUP53	YMR199W	CLN1
YOR195W	SLK19	YJR135C	MCM22
YDR137W	RGP1	YEL060C	PRB1
YER044C	ERG28	YEL060C	PRB1
YLR095C	IOC2	YKL048C	ELM1
YBL079W	NUP170	YFL047W	RGD2
YKR099W	BAS1	YDR261C	EXG2
YER114C	BOI2	YPL161C	BEM4
YDR076W	RAD55	YDR289C	RTT103
YPR131C	NAT3	YJR035W	RAD26
YEL065W	SIT1	YMR016C	SOK2
YLR371W	ROM2	YPL256C	CLN2
YHR156C	LIN1	YEL053C	MAK10
YGR261C	APL6	YGL223C	COG1
YPL042C	SSN3	YLR399C	BDF1
YDR477W	SNF1	YOR140W	SFL1
YHR111W	UBA4	YML112W	CTK3
YDL083C	RPS16B	YCR053W	THR4
YPL195W	APL5	YNL051W	COG5
YPL137C	GIP3	YCR053W	THR4
YMR153W	NUP53	YDL223C	HBT1
YEL009C	GCN4	YBR289W	SNF5
YDL160C	DHH1	YNL288W	CAF40
YLR018C	POM34	YML103C	NUP188
YLL039C	UBI4	YDL223C	HBT1
YPR046W	MCM16	YGL253W	HXK2

Predictions at threshold level 0.75 continued...			
Gene1 of predicted pair		Gene2 of predicted pair	
Systematic Name	Standard Name	Systematic Name	Standard Name
YPR046W	MCM16	YGR188C	BUB1
YML071C	COG8	YAL002W	VPS8
YNL166C	BNI5	YCR086W	CSM1
YDL161W	ENT1	YJR099W	YUH1
YPL161C	BEM4	YOL121C	RPS19A
YNL215W	IES2	YAR014C	BUD14
YPL024W	RMI1	YJL095W	BCK1
YDR383C	NKP1	YLR182W	SWI6
YBR217W	ATG12	YNL307C	MCK1
YPR052C	NHP6A	YNL236W	SIN4
YLR288C	MEC3	YML115C	VAN1
YBR127C	VMA2	YDL226C	GCS1
YMR109W	MYO5	YLR370C	ARC18
YDR477W	SNF1	YNL079C	TPM1
YHR200W	RPN10	YLR371W	ROM2
YHR134W	WSS1	YGR270W	YTA7
YLR360W	VPS38	YNL041C	COG6
YMR089C	YTA12	YKL041W	VPS24
YPL022W	RAD1	YDL013W	SLX5
YPR017C	DSS4	YGR261C	APL6
YHR084W	STE12	YJL128C	PBS2
YDL122W	UBP1	YIL148W	RPL40A
YNL058C	YNL058C	YPR030W	CSR2
YLR381W	CTF3	YAR014C	BUD14
YDR137W	RGP1	YEL053C	MAK10
YDR484W	VPS52	YOR290C	SNF2
YPR046W	MCM16	YOL081W	IRA2
YLR381W	CTF3	YOL067C	RTG1
YBR200W	BEM1	YHR205W	SCH9

Predictions at threshold level 0.75 continued...			
Gene1 of predicted pair		Gene2 of predicted pair	
Systematic Name	Standard Name	Systematic Name	Standard Name
YCL037C	SRO9	YNL041C	COG6
YPL158C	YPL158C	YNL079C	TPM1
YDL116W	NUP84	YMR273C	ZDS1
YDR293C	SSD1	YGL253W	HXK2
YKR001C	VPS1	YER122C	GLO3
YDR146C	SWI5	YPR120C	CLB5
YER179W	DMC1	YDR146C	SWI5
YGL212W	VAM7	YOR066W	MSA1
YNL199C	GCR2	YOR038C	HIR2
YDR103W	STE5	YLR452C	SST2
YOR195W	SLK19	YOL081W	IRA2
YHR178W	STB5	YLR386W	VAC14
YPR030W	CSR2	YOL121C	RPS19A
YNL041C	COG6	YLR357W	RSC2
YBR200W	BEM1	YNL107W	YAF9
YGL223C	COG1	YNR032W	PPG1
YOL018C	TLG2	YPR032W	SRO7
YGL087C	MMS2	YNL288W	CAF40
YPL137C	GIP3	YDL192W	ARF1
YBL078C	ATG8	YDR080W	VPS41
YLR360W	VPS38	YNL238W	KEX2
YDR156W	RPA14	YNL107W	YAF9
YBR290W	BSD2	YMR199W	CLN1
YDR320C	SWA2	YJR033C	RAV1
YMR284W	YKU70	YDR469W	SDC1
YBL101C	ECM21	YMR038C	CCS1
YDL006W	PTC1	YOL081W	IRA2
YGR104C	SRB5	YPR072W	NOT5
YNL031C	HHT2	YDR440W	DOT1

Predictions at threshold level 0.75 continued...			
Gene1 of predicted pair		Gene2 of predicted pair	
Systematic Name	Standard Name	Systematic Name	Standard Name
YER174C	GRX4	YGR262C	BUD32
YDL006W	PTC1	YNL127W	FAR11
YKL010C	UFD4	YDR245W	MNN10
YDR245W	MNN10	YLR371W	ROM2
YMR039C	SUB1	YJR140C	HIR3
YNL106C	INP52	YGL163C	RAD54
YMR224C	MRE11	YLR381W	CTF3
YMR016C	SOK2	YMR037C	MSN2
YDR156W	RPA14	YLR371W	ROM2
YDR156W	RPA14	YDR245W	MNN10
YGL124C	MON1	YAL002W	VPS8
YKR020W	VPS51	YGL124C	MON1
YKL048C	ELM1	YGL253W	HXK2
YAL024C	LTE1	YDR439W	LRS4
YBL021C	HAP3	YDL155W	CLB3
YIL036W	CST6	YNL248C	RPA49
YDL223C	HBT1	YBR280C	SAF1
YDR439W	LRS4	YCR086W	CSM1
YOR212W	STE4	YLR206W	ENT2
YJL138C	TIF2	YOR368W	RAD17
YCR088W	ABP1	YJL029C	VPS53
YGL060W	YBP2	YJR135C	MCM22
YDL059C	RAD59	YDR363W-A	SEM1
YMR311C	GLC8	YMR214W	SCJ1
YGL078C	DBP3	YLR192C	HCR1
YBR264C	YPT10	YML001W	YPT7
YBR274W	CHK1	YML016C	PPZ1
YOR127W	RGA1	YBR200W	BEM1
YLR360W	VPS38	YOL067C	RTG1

Predictions at threshold level 0.75 continued...			
Gene1 of predicted pair		Gene2 of predicted pair	
Systematic Name	Standard Name	Systematic Name	Standard Name
YNL166C	BNI5	YEL053C	MAK10
YGL033W	HOP2	YHR156C	LIN1
YOR290C	SNF2	YDL077C	VAM6
YLR095C	IOC2	YJL095W	BCK1
YOL067C	RTG1	YPR119W	CLB2
YPR017C	DSS4	YBL102W	SFT2
YNL307C	MCK1	YPR046W	MCM16
YNL139C	RLR1	YCL011C	GBP2
YJR059W	PTK2	YKL068W	NUP100
YBR020W	GAL1	YKL160W	ELF1
YIL156W	UBP7	YOR358W	HAP5
YDL006W	PTC1	YPL120W	VPS30
YJR084W	CSN12	YBL025W	RRN10
YEL053C	MAK10	YFR036W	CDC26
YML063W	RPS1B	YER102W	RPS8B
YML016C	PPZ1	YHR115C	DMA1
YPL042C	SSN3	YGL237C	HAP2
YLR080W	EMP46	YFL048C	EMP47
YBR215W	HPC2	YDR254W	CHL4
YLR386W	VAC14	YDR440W	DOT1
YLR234W	TOP3	YAL002W	VPS8
YKR001C	VPS1	YDR076W	RAD55
YNL041C	COG6	YDR310C	SUM1
YGL115W	SNF4	YPR120C	CLB5
YOR327C	SNC2	YDR207C	UME6
YDL116W	NUP84	YML012W	ERV25
YGL087C	MMS2	YPL057C	SUR1
YLR452C	SST2	YDL192W	ARF1
YJL176C	SWI3	YLL039C	UBI4

Predictions at threshold level 0.75 continued...			
Gene1 of predicted pair		Gene2 of predicted pair	
Systematic Name	Standard Name	Systematic Name	Standard Name
YNR058W	BIO3	YFR021W	ATG18
YOR195W	SLK19	YJL006C	CTK2
YDR477W	SNF1	YGR252W	GCN5
YEL044W	IES6	YJL095W	BCK1
YOR195W	SLK19	YDR183W	PLP1
YPL024W	RMI1	YHR013C	ARD1
YKL110C	KTI12	YGL163C	RAD54
YJR033C	RAV1	YNR032W	PPG1
YJL036W	SNX4	YGL115W	SNF4
YOR308C	SNU66	YAL002W	VPS8
YDR318W	MCM21	YDL047W	SIT4
YCR024C-A	PMP1	YER110C	KAP123
YGL025C	PGD1	YBL025W	RRN10
YOL111C	MDY2	YCR053W	THR4
YJL095W	BCK1	YBR181C	RPS6B
YJL060W	BNA3	YMR055C	BUB2
YBL058W	SHP1	YFR036W	CDC26
YHL031C	GOS1	YDR363W-A	SEM1
YNL087W	TCB2	YDR484W	VPS52
YIL156W	UBP7	YMR109W	MYO5
YPL024W	RMI1	YDL223C	HBT1
YNL032W	SIW14	YNL058C	YNL058C
YPL105C	YPL105C	YOL018C	TLG2
YLR337C	VRP1	YDL002C	NHP10
YJL183W	MNN11	YBR114W	RAD16
YNL291C	MID1	YJR040W	GEF1
YDR477W	SNF1	YHR081W	LRP1
YLR360W	VPS38	YNL127W	FAR11
YBL067C	UBP13	YLR370C	ARC18

Predictions at threshold level 0.75 continued...			
Gene1 of predicted pair		Gene2 of predicted pair	
Systematic Name	Standard Name	Systematic Name	Standard Name
YHR111W	UBA4	YCR086W	CSM1
YNL307C	MCK1	YER139C	YER139C
YIL053W	RHR2	YFR036W	CDC26
YJR040W	GEF1	YJR060W	CBF1
YMR116C	ASC1	YDL013W	SLX5
YOR275C	RIM20	YNL079C	TPM1
YOR066W	MSA1	YLR370C	ARC18
YIL148W	RPL40A	YFL047W	RGD2
YMR127C	SAS2	YBR010W	HHT1
YJR052W	RAD7	YER162C	RAD4
YBR030W	YBR030W	YMR142C	RPL13B
YOR080W	DIA2	YJR035W	RAD26
YPR120C	CLB5	YPR046W	MCM16
YDL101C	DUN1	YLR410W	VIP1
YGL060W	YBP2	YDR318W	MCM21
YJR140C	HIR3	YLR320W	MMS22
YPR120C	CLB5	YFR030W	MET10
YMR202W	ERG2	YPR040W	TIP41
YFR032C-A	RPL29	YGR135W	PRE9
YAL024C	LTE1	YNL116W	DMA2
YFL023W	BUD27	YGL163C	RAD54
YML117W	NAB6	YKR020W	VPS51
YFR031C-A	RPL2A	YIL159W	BNR1
YHR081W	LRP1	YOR327C	SNC2
YDR310C	SUM1	YDR207C	UME6
YOR337W	TEA1	YDR358W	GGA1
YEL056W	HAT2	YJL190C	RPS22A
YGR108W	CLB1	YPR120C	CLB5
YNL325C	FIG4	YNL032W	SIW14

Predictions at threshold level 0.75 continued...			
Gene1 of predicted pair		Gene2 of predicted pair	
Systematic Name	Standard Name	Systematic Name	Standard Name
YOR109W	INP53	YML001W	YPT7
YIL034C	CAP2	YLR381W	CTF3
YLR234W	TOP3	YLR435W	TSR2
YPL115C	BEM3	YER114C	BOI2
YDR009W	GAL3	YPL248C	GAL4
YER114C	BOI2	YCR002C	CDC10
YNL206C	RTT106	YLR452C	SST2
YDL190C	UFD2	YDR363W-A	SEM1
YGL237C	HAP2	YHR111W	UBA4
YIL159W	BNR1	YDL223C	HBT1
YPR036W	VMA13	YER040W	GLN3
YMR142C	RPL13B	YNL087W	TCB2
YMR152W	YIM1	YDL192W	ARF1
YEL053C	MAK10	YNR032W	PPG1
YIL053W	RHR2	YMR152W	YIM1
YNR052C	POP2	YGL252C	RTG2
YOR337W	TEA1	YBL025W	RRN10
YLR090W	XDJ1	YGL124C	MON1
YOR033C	EXO1	YBL079W	NUP170
YKL116C	PRR1	YKR099W	BAS1
YHR081W	LRP1	YDL077C	VAM6
YGL147C	RPL9A	YMR224C	MRE11
YPR036W	VMA13	YLR370C	ARC18
YFL026W	STE2	YDR183W	PLP1
YGR270W	YTA7	YER122C	GLO3
YOR106W	VAM3	YPL057C	SUR1
YLR386W	VAC14	YDR183W	PLP1
YER110C	KAP123	YMR273C	ZDS1
YOR337W	TEA1	YMR016C	SOK2

Predictions at threshold level 0.75 continued...			
Gene1 of predicted pair		Gene2 of predicted pair	
Systematic Name	Standard Name	Systematic Name	Standard Name
YNL291C	MID1	YDL013W	SLX5
YIR005W	IST3	YLR254C	NDL1
YHR060W	VMA22	YJR060W	CBF1
YGR261C	APL6	YPR032W	SRO7
YGR104C	SRB5	YBR010W	HHT1
YDR004W	RAD57	YPL253C	VIK1
YLR292C	SEC72	YHR012W	VPS29
YLR360W	VPS38	YKR001C	VPS1
YBR215W	HPC2	YDR310C	SUM1
YNR052C	POP2	YGL163C	RAD54
YEL065W	SIT1	YBR019C	GAL10
YOR195W	SLK19	YJL124C	LSM1
YBR108W	YBR108W	YDR289C	RTT103
YDR202C	RAV2	YEL051W	VMA8
YJL124C	LSM1	YNL248C	RPA49
YFR031C-A	RPL2A	YBR030W	YBR030W
YDL047W	SIT4	YNL051W	COG5
YDR129C	SAC6	YLR396C	VPS33
YBR173C	UMP1	YMR198W	CIK1
YLR360W	VPS38	YOL108C	INO4
YML016C	PPZ1	YHR158C	KEL1
YOR069W	VPS5	YNL041C	COG6
YGL175C	SAE2	YPL256C	CLN2
YIL034C	CAP2	YBR107C	IML3
YKR059W	TIF1	YJR032W	CPR7
YJL172W	CPS1	YBR036C	CSG2
YDR358W	GGA1	YAL030W	SNC1
YIL053W	RHR2	YJL183W	MNN11
YFR010W	UBP6	YFL023W	BUD27

Predictions at threshold level 0.75 continued...			
Gene1 of predicted pair		Gene2 of predicted pair	
Systematic Name	Standard Name	Systematic Name	Standard Name
YJR060W	CBF1	YOL054W	PSH1
YFL004W	VTC2	YER072W	VTC1
YBR290W	BSD2	YPR046W	MCM16
YBL016W	FUS3	YJL157C	FAR1
YDL006W	PTC1	YOR327C	SNC2
YNL199C	GCR2	YLR182W	SWI6
YER110C	KAP123	YMR109W	MYO5
YPL240C	HSP82	YIL033C	BCY1
YLR288C	MEC3	YOR043W	WHI2
YCR086W	CSM1	YOL067C	RTG1
YDR137W	RGP1	YPR046W	MCM16
YOR109W	INP53	YOR035C	SHE4
YPR036W	VMA13	YER072W	VTC1
YBL008W	HIR1	YBR195C	MSI1
YJL124C	LSM1	YGL148W	ARO2
YMR224C	MRE11	YOR141C	ARP8
YNL199C	GCR2	YAL002W	VPS8
YBR173C	UMP1	YBR036C	CSG2
YNL229C	URE2	YGR135W	PRE9
YHR056C	RSC30	YKL114C	APN1
YMR055C	BUB2	YOR141C	ARP8
YGR133W	PEX4	YBR082C	UBC4
YGL054C	ERV14	YPR119W	CLB2
YFL033C	RIM15	YNL127W	FAR11
YDL155W	CLB3	YDR076W	RAD55
YBL078C	ATG8	YAL030W	SNC1
YLR315W	NKP2	YLR182W	SWI6
YER075C	PTP3	YPR119W	CLB2
YIL053W	RHR2	YOR327C	SNC2

Predictions at threshold level 0.75 continued...			
Gene1 of predicted pair		Gene2 of predicted pair	
Systematic Name	Standard Name	Systematic Name	Standard Name
YDR335W	MSN5	YIR005W	IST3
YML115C	VAN1	YHR060W	VMA22
YMR216C	SKY1	YNL297C	MON2
YDR318W	MCM21	YML112W	CTK3
YGL115W	SNF4	YKR030W	GMH1
YML117W	NAB6	YMR116C	ASC1
YIL008W	URM1	YPR046W	MCM16
YDR320C	SWA2	YNL297C	MON2
YEL015W	EDC3	YNL147W	LSM7
YCR020C-A	MAK31	YPR017C	DSS4
YDR202C	RAV2	YOR327C	SNC2
YJR140C	HIR3	YER139C	YER139C
YKL048C	ELM1	YLR093C	NYV1
YKR057W	RPS21A	YKL101W	HSL1
YCL027W	FUS1	YKL129C	MYO3
YML058W	SML1	YPL022W	RAD1
YIL045W	PIG2	YLR258W	GSY2
YOR038C	HIR2	YOR124C	UBP2
YBL101C	ECM21	YBR082C	UBC4
YFR031C-A	RPL2A	YNL087W	TCB2
YDR004W	RAD57	YBR057C	MUM2
YBL103C	RTG3	YJR032W	CPR7
YGL033W	HOP2	YMR016C	SOK2
YKL110C	KTI12	YNL116W	DMA2
YMR152W	YIM1	YGL003C	CDH1
YLR315W	NKP2	YOR038C	HIR2
YDR174W	HMO1	YDR156W	RPA14
YPR046W	MCM16	YLR093C	NYV1
YML115C	VAN1	YLR441C	RPS1A

Predictions at threshold level 0.75 continued...			
Gene1 of predicted pair		Gene2 of predicted pair	
Systematic Name	Standard Name	Systematic Name	Standard Name
YOR132W	VPS17	YER162C	RAD4
YFR036W	CDC26	YOR141C	ARP8
YER044C	ERG28	YFR036W	CDC26
YNL025C	SSN8	YNR052C	POP2
YDL083C	RPS16B	YGL003C	CDH1
YMR127C	SAS2	YGL124C	MON1
YNL069C	RPL16B	YDR153C	ENT5
YLR095C	IOC2	YFR010W	UBP6
YDL020C	RPN4	YLR135W	SLX4
YFR031C-A	RPL2A	YOL111C	MDY2
YOR327C	SNC2	YNL051W	COG5
YDR176W	NGG1	YDR448W	ADA2
YMR054W	STV1	YKL080W	VMA5
YPR018W	RLF2	YPR046W	MCM16
YDR071C	PAA1	YNL030W	HHF2
YNL094W	APP1	YMR109W	MYO5
YFL023W	BUD27	YDR358W	GGA1
YAL024C	LTE1	YML115C	VAN1
YCR077C	PAT1	YPR119W	CLB2
YDR174W	HMO1	YJL006C	CTK2
YDL020C	RPN4	YPR120C	CLB5
YPR046W	MCM16	YKL101W	HSL1
YNL068C	FKH2	YHR200W	RPN10
YOR080W	DIA2	YLR058C	SHM2
YBL058W	SHP1	YML071C	COG8
YMR142C	RPL13B	YGL212W	VAM7
YEL050C	RML2	YLR079W	SIC1
YOR066W	MSA1	YER139C	YER139C
YBR200W	BEM1	YOL054W	PSH1

Predictions at threshold level 0.75 continued...			
Gene1 of predicted pair		Gene2 of predicted pair	
Systematic Name	Standard Name	Systematic Name	Standard Name
YJR060W	CBF1	YLR182W	SWI6
YNL238W	KEX2	YLR309C	IMH1
YLL007C	YLL007C	YIL008W	URM1
YDR320C	SWA2	YOR069W	VPS5
YMR039C	SUB1	YNL147W	LSM7
YFL033C	RIM15	YDR207C	UME6
YBR031W	RPL4A	YBL072C	RPS8A
YFR031C-A	RPL2A	YLR452C	SST2
YKR099W	BAS1	YKR035W-A	DID2
YDR174W	HMO1	YML112W	CTK3
YKL116C	PRR1	YNL041C	COG6
YKL126W	YPK1	YER139C	YER139C
YIL053W	RHR2	YNL238W	KEX2
YMR054W	STV1	YNL107W	YAF9
YFL023W	BUD27	YPL195W	APL5
YMR095C	SNO1	YMR142C	RPL13B
YDR156W	RPA14	YLR254C	NDL1
YNL069C	RPL16B	YNL051W	COG5
YOR132W	VPS17	YHR178W	STB5
YNL238W	KEX2	YDR310C	SUM1
YOR275C	RIM20	YPL158C	YPL158C
YMR109W	MYO5	YMR055C	BUB2
YOR127W	RGA1	YHR115C	DMA1
YDR363W-A	SEM1	YKR001C	VPS1
YJR005W	APL1	YDR318W	MCM21
YBR200W	BEM1	YOL111C	MDY2
YGR104C	SRB5	YGL178W	MPT5
YBR057C	MUM2	YDR076W	RAD55
YGR081C	SLX9	YMR231W	PEP5

Predictions at threshold level 0.75 continued...			
Gene1 of predicted pair		Gene2 of predicted pair	
Systematic Name	Standard Name	Systematic Name	Standard Name
YGR072W	UPF3	YEL053C	MAK10
YLR320W	MMS22	YBL025W	RRN10
YHR060W	VMA22	YOR327C	SNC2
YIL159W	BNR1	YNL238W	KEX2
YPL037C	EGD1	YDR207C	UME6
YFL023W	BUD27	YKR099W	BAS1
YCR081W	SRB8	YMR199W	CLN1
YMR202W	ERG2	YJR035W	RAD26
YMR133W	REC114	YFR036W	CDC26
YDL116W	NUP84	YLR288C	MEC3
YDL185W	TFP1	YGR105W	VMA21
YJR060W	CBF1	YNL116W	DMA2
YNL201C	PSY2	YNR052C	POP2
YLR447C	VMA6	YKR001C	VPS1
YBR217W	ATG12	YER162C	RAD4
YNL054W	VAC7	YKR061W	KTR2
YDR320C	SWA2	YML001W	YPT7
YOR337W	TEA1	YNL325C	FIG4
YBR173C	UMP1	YHR060W	VMA22
YLR093C	NYV1	YMR230W	RPS10B
YER116C	SLX8	YGL124C	MON1
YHR178W	STB5	YOR213C	SAS5
YDL155W	CLB3	YCR086W	CSM1
YLR090W	XDJ1	YGL003C	CDH1
YKL101W	HSL1	YJL095W	BCK1
YLR377C	FBP1	YOL126C	MDH2
YFR010W	UBP6	YNL076W	MKS1
YDR254W	CHL4	YKL110C	KTI12
YJL124C	LSM1	YMR055C	BUB2

Predictions at threshold level 0.75 continued...			
Gene1 of predicted pair		Gene2 of predicted pair	
Systematic Name	Standard Name	Systematic Name	Standard Name
YPL050C	MNN9	YBL047C	EDE1
YBR200W	BEM1	YJR056C	YJR056C
YLL007C	YLL007C	YPR046W	MCM16
YDR183W	PLP1	YNL116W	DMA2
YMR142C	RPL13B	YKR099W	BAS1
YIL053W	RHR2	YEL053C	MAK10
YKR072C	SIS2	YLR288C	MEC3
YDR479C	PEX29	YLR324W	PEX30
YKR048C	NAP1	YOR208W	PTP2
YEL009C	GCN4	YOR023C	AHC1
YGL223C	COG1	YOR109W	INP53
YKR035W-A	DID2	YDL192W	ARF1
YHL031C	GOS1	YGL054C	ERV14
YLR292C	SEC72	YHR111W	UBA4
YIL159W	BNR1	YLR452C	SST2
YDL116W	NUP84	YMR055C	BUB2
YDL002C	NHP10	YBR036C	CSG2
YCR066W	RAD18	YPL195W	APL5
YJL065C	DLS1	YOR304W	ISW2
YLR360W	VPS38	YJR033C	RAV1
YNL307C	MCK1	YHR081W	LRP1
YKR101W	SIR1	YLR399C	BDF1
YKR057W	RPS21A	YBR084C-A	RPL19A
YJR059W	PTK2	YPL246C	RBD2
YNL307C	MCK1	YJR032W	CPR7
YIL076W	SEC28	YPL037C	EGD1
YJR024C	MDE1	YKR024C	DBP7
YGR184C	UBR1	YHR052W	CIC1
YNL288W	CAF40	YGR188C	BUB1

Predictions at threshold level 0.75 continued...			
**Gene1 of predicted pair**		**Gene2 of predicted pair**	
**Systematic Name**	**Standard Name**	**Systematic Name**	**Standard Name**
YGL212W	VAM7	YAL002W	VPS8
YER001W	MNN1	YGL212W	VAM7
YDR245W	MNN10	YDR363W-A	SEM1
YBR290W	BSD2	YPL018W	CTF19
YOR270C	VPH1	YOL121C	RPS19A
YFR031C-A	RPL2A	YPL057C	SUR1
YJL172W	CPS1	YJR084W	CSN12
YLR119W	SRN2	YGL033W	HOP2
YHR178W	STB5	YDR183W	PLP1
YER096W	SHC1	YCR077C	PAT1
YDR477W	SNF1	YNL058C	YNL058C
YHR079C	IRE1	YBR010W	HHT1
YNL243W	SLA2	YDR358W	GGA1
YML103C	NUP188	YER115C	SPR6
YPL253C	VIK1	YPL256C	CLN2
YPL195W	APL5	YDR310C	SUM1
YPL024W	RMI1	YPL022W	RAD1
YLR320W	MMS22	YBR082C	UBC4
YNL291C	MID1	YEL051W	VMA8
YLR418C	CDC73	YGL094C	PAN2
YKL126W	YPK1	YFR036W	CDC26
YGR108W	CLB1	YHR178W	STB5
YPL105C	YPL105C	YHR012W	VPS29
YDL006W	PTC1	YOR035C	SHE4
YPR051W	MAK3	YLR447C	VMA6
YKR099W	BAS1	YCR053W	THR4
YLR386W	VAC14	YDL077C	VAM6
YDR108W	GSG1	YPL120W	VPS30
YDR174W	HMO1	YNR052C	POP2

Predictions at threshold level 0.75 continued...			
Gene1 of predicted pair		Gene2 of predicted pair	
Systematic Name	Standard Name	Systematic Name	Standard Name
YMR133W	REC114	YER139C	YER139C
YDR174W	HMO1	YHR115C	DMA1
YDR004W	RAD57	YPR120C	CLB5
YER115C	SPR6	YGL016W	KAP122
YFL013C	IES1	YLR350W	ORM2
YFL023W	BUD27	YBL025W	RRN10
YLR410W	VIP1	YLR254C	NDL1
YBR030W	YBR030W	YKR099W	BAS1
YJR060W	CBF1	YLR315W	NKP2
YLR234W	TOP3	YMR109W	MYO5
YFR031C-A	RPL2A	YPL037C	EGD1
YOR023C	AHC1	YDR254W	CHL4
YKL117W	SBA1	YMR273C	ZDS1
YDR101C	ARX1	YIL133C	RPL16A
YBR280C	SAF1	YER122C	GLO3
YDL101C	DUN1	YBL079W	NUP170
YDR383C	NKP1	YNL054W	VAC7
YEL065W	SIT1	YBL058W	SHP1
YDR144C	MKC7	YJL183W	MNN11
YBR126C	TPS1	YPL137C	GIP3
YER087C-B	SBH1	YPR028W	YOP1
YNL087W	TCB2	YLR320W	MMS22
YJL124C	LSM1	YGL252C	RTG2
YIL053W	RHR2	YDR207C	UME6
YGL033W	HOP2	YOR270C	VPH1
YCR081W	SRB8	YHR205W	SCH9
YNL307C	MCK1	YNL238W	KEX2
YCR020C-A	MAK31	YOL018C	TLG2
YDR448W	ADA2	YGL066W	SGF73

Predictions at threshold level 0.75 continued...			
Gene1 of predicted pair		Gene2 of predicted pair	
Systematic Name	Standard Name	Systematic Name	Standard Name
YLR079W	SIC1	YIL036W	CST6
YER122C	GLO3	YOL081W	IRA2
YOR001W	RRP6	YNL199C	GCR2
YML067C	ERV41	YFL048C	EMP47
YDR363W-A	SEM1	YKL160W	ELF1
YFR036W	CDC26	YLR254C	NDL1
YGL087C	MMS2	YJL183W	MNN11
YJL036W	SNX4	YHR060W	VMA22
YGL054C	ERV14	YDR153C	ENT5
YPR120C	CLB5	YNL116W	DMA2
YDR358W	GGA1	YCR053W	THR4
YKR001C	VPS1	YDR207C	UME6
YIL159W	BNR1	YMR095C	SNO1
YJL190C	RPS22A	YNL107W	YAF9
YBR200W	BEM1	YDL155W	CLB3
YDR108W	GSG1	YER122C	GLO3
YNL069C	RPL16B	YDR080W	VPS41
YPL137C	GIP3	YJR084W	CSN12
YHR111W	UBA4	YEL053C	MAK10
YDL116W	NUP84	YML095C	RAD10
YKL139W	CTK1	YLR441C	RPS1A
YGR104C	SRB5	YHR200W	RPN10
YMR016C	SOK2	YPL137C	GIP3
YKL110C	KTI12	YNL225C	CNM67
YML001W	YPT7	YJR032W	CPR7
YDL185W	TFP1	YJL006C	CTK2
YNL107W	YAF9	YGL252C	RTG2
YDR183W	PLP1	YJL183W	MNN11
YGR261C	APL6	YNL127W	FAR11

Predictions at threshold level 0.75 continued...			
Gene1 of predicted pair		Gene2 of predicted pair	
Systematic Name	Standard Name	Systematic Name	Standard Name
YPL057C	SUR1	YDR378C	LSM6
YJL183W	MNN11	YML097C	VPS9
YGL178W	MPT5	YDL225W	SHS1
YHR111W	UBA4	YGR135W	PRE9
YBL079W	NUP170	YPL256C	CLN2
YOR043W	WHI2	YLR371W	ROM2
YNL106C	INP52	YIL103W	DPH1
YER068W	MOT2	YJL140W	RPB4
YKL135C	APL2	YDL192W	ARF1
YPL037C	EGD1	YDR358W	GGA1
YOR127W	RGA1	YAR014C	BUD14
YLR094C	GIS3	YGL124C	MON1
YGL003C	CDH1	YPL195W	APL5
YEL044W	IES6	YLR370C	ARC18
YDR358W	GGA1	YLR131C	ACE2
YPL115C	BEM3	YOL067C	RTG1
YAL024C	LTE1	YDR309C	GIC2
YIL076W	SEC28	YKL213C	DOA1
YOR195W	SLK19	YDR379W	RGA2
YNL016W	PUB1	YOL090W	MSH2
YPR167C	MET16	YER042W	MXR1
YPR018W	RLF2	YER116C	SLX8
YDR320C	SWA2	YOR112W	CEX1
YPR131C	NAT3	YPR046W	MCM16
YDR080W	VPS41	YLR309C	IMH1
YBR173C	UMP1	YBL019W	APN2
YPR018W	RLF2	YDR076W	RAD55
YGL087C	MMS2	YDR092W	UBC13
YDR254W	CHL4	YGL252C	RTG2

Predictions at threshold level 0.75 continued...			
Gene1 of predicted pair		Gene2 of predicted pair	
Systematic Name	Standard Name	Systematic Name	Standard Name
YEL060C	PRB1	YMR214W	SCJ1
YNL201C	PSY2	YGR188C	BUB1
YDR507C	GIN4	YKR020W	VPS51
YDL059C	RAD59	YOR014W	RTS1
YJL060W	BNA3	YDR318W	MCM21
YJR056C	YJR056C	YBL025W	RRN10
YLR441C	RPS1A	YHR203C	RPS4B
YGR249W	MGA1	YIL076W	SEC28
YOR270C	VPH1	YBR200W	BEM1
YGL200C	EMP24	YFR036W	CDC26
YKR061W	KTR2	YGR270W	YTA7
YML103C	NUP188	YLR254C	NDL1
YPL105C	YPL105C	YDL077C	VAM6
YOL030W	GAS5	YDR207C	UME6
YOR270C	VPH1	YHR039C-A	VMA10
YBL102W	SFT2	YAR014C	BUD14
YOR140W	SFL1	YDR245W	MNN10
YBR057C	MUM2	YNL329C	PEX6
YDR202C	RAV2	YLR309C	IMH1
YGR249W	MGA1	YGL115W	SNF4
YML103C	NUP188	YDR335W	MSN5
YDR289C	RTT103	YNL107W	YAF9
YKR061W	KTR2	YNL225C	CNM67
YML103C	NUP188	YNL307C	MCK1
YDR335W	MSN5	YOR038C	HIR2
YNL087W	TCB2	YBR036C	CSG2
YKR001C	VPS1	YPL120W	VPS30
YMR127C	SAS2	YPL018W	CTF19
YFR031C-A	RPL2A	YMR153W	NUP53

Predictions at threshold level 0.75 continued...			
Gene1 of predicted pair		Gene2 of predicted pair	
Systematic Name	Standard Name	Systematic Name	Standard Name
YLR386W	VAC14	YOR327C	SNC2
YLR381W	CTF3	YAL002W	VPS8
YMR273C	ZDS1	YBL079W	NUP170
YIL156W	UBP7	YHR114W	BZZ1
YNL032W	SIW14	YHR039C-A	VMA10
YJL151C	SNA3	YHR114W	BZZ1
YKL048C	ELM1	YLR233C	EST1
YKR059W	TIF1	YHR178W	STB5
YDR144C	MKC7	YGR188C	BUB1
YGL133W	ITC1	YPL161C	BEM4
YGL212W	VAM7	YHL020C	OPI1
YPR120C	CLB5	YJL006C	CTK2
YML103C	NUP188	YCR086W	CSM1
YCR077C	PAT1	YLR425W	TUS1
YDL116W	NUP84	YDR383C	NKP1
YML115C	VAN1	YER096W	SHC1
YFR010W	UBP6	YBR057C	MUM2
YOL126C	MDH2	YOR358W	HAP5
YGL025C	PGD1	YHR039C-A	VMA10
YDR110W	FOB1	YHR152W	SPO12
YGL212W	VAM7	YER120W	SCS2
YJL004C	SYS1	YDL192W	ARF1
YPL050C	MNN9	YFL048C	EMP47
YLR452C	SST2	YBR036C	CSG2
YFR031C-A	RPL2A	YMR311C	GLC8
YIL053W	RHR2	YOR290C	SNF2
YIR009W	MSL1	YMR055C	BUB2
YBL058W	SHP1	YLR441C	RPS1A
YDR363W-A	SEM1	YOL091W	SPO21

Predictions at threshold level 0.75 continued...			
Gene1 of predicted pair		Gene2 of predicted pair	
Systematic Name	Standard Name	Systematic Name	Standard Name
YIL159W	BNR1	YOL111C	MDY2
YOR270C	VPH1	YGL212W	VAM7
YOR066W	MSA1	YML071C	COG8
YER110C	KAP123	YNL199C	GCR2
YNL297C	MON2	YML001W	YPT7
YOR069W	VPS5	YJR058C	APS2
YPL240C	HSP82	YHR079C-A	SAE3
YIL159W	BNR1	YHR060W	VMA22
YDR456W	NHX1	YHR012W	VPS29
YHR013C	ARD1	YNL116W	DMA2
YGL212W	VAM7	YLR093C	NYV1
YBR171W	SEC66	YLR370C	ARC18
YPL253C	VIK1	YGL252C	RTG2
YNL307C	MCK1	YKL048C	ELM1
YPL195W	APL5	YBL025W	RRN10
YPR018W	RLF2	YMR055C	BUB2
YBL051C	PIN4	YJL190C	RPS22A
YGL153W	PEX14	YLR425W	TUS1
YDR443C	SSN2	YNR052C	POP2
YAL014C	SYN8	YGL212W	VAM7
YNL291C	MID1	YOR069W	VPS5
YDR477W	SNF1	YDR146C	SWI5
YMR198W	CIK1	YJR084W	CSN12
YHR061C	GIC1	YGR152C	RSR1
YER099C	PRS2	YHL011C	PRS3
YOR127W	RGA1	YPR032W	SRO7
YDL020C	RPN4	YPL024W	RMI1
YDL223C	HBT1	YPL137C	GIP3
YPL115C	BEM3	YBR107C	IML3

Predictions at threshold level 0.75 continued...			
Gene1 of predicted pair		Gene2 of predicted pair	
Systematic Name	Standard Name	Systematic Name	Standard Name
YOR231W	MKK1	YDL159W	STE7
YLL007C	YLL007C	YCR086W	CSM1
YHR013C	ARD1	YML097C	VPS9
YNL041C	COG6	YEL051W	VMA8
YOR275C	RIM20	YBL101C	ECM21
YNR052C	POP2	YLR135W	SLX4
YDR137W	RGP1	YKR001C	VPS1
YOR033C	EXO1	YPR119W	CLB2
YGR209C	TRX2	YIL008W	URM1
YPL145C	KES1	YLR133W	CKI1
YLR192C	HCR1	YER139C	YER139C
YOR038C	HIR2	YHR167W	THP2
YBR217W	ATG12	YDR358W	GGA1
YLR079W	SIC1	YOL081W	IRA2
YDR456W	NHX1	YMR311C	GLC8
YJL006C	CTK2	YNL147W	LSM7
YDR216W	ADR1	YPL248C	GAL4
YNL076W	MKS1	YDR207C	UME6
YPL115C	BEM3	YDR310C	SUM1
YGR249W	MGA1	YMR153W	NUP53
YFR031C-A	RPL2A	YNL127W	FAR11
YMR172W	HOT1	YJL190C	RPS22A
YPL057C	SUR1	YNL041C	COG6
YPR084W	YPR084W	YPL105C	YPL105C
YJR135C	MCM22	YKL101W	HSL1
YGL237C	HAP2	YDL155W	CLB3
YKR035W-A	DID2	YGL003C	CDH1
YIL034C	CAP2	YGL237C	HAP2
YGL135W	RPL1B	YBL047C	EDE1

Predictions at threshold level 0.75 continued...			
Gene1 of predicted pair		Gene2 of predicted pair	
Systematic Name	Standard Name	Systematic Name	Standard Name
YER143W	DDI1	YHR200W	RPN10
YDR363W-A	SEM1	YDR289C	RTT103
YMR231W	PEP5	YGL124C	MON1
YBR195C	MSI1	YML102W	CAC2
YKL126W	YPK1	YGL115W	SNF4
YLR360W	VPS38	YGR133W	PEX4
YDR484W	VPS52	YER139C	YER139C
YGL212W	VAM7	YMR214W	SCJ1
YPR084W	YPR084W	YKR030W	GMH1
YGL115W	SNF4	YMR230W	RPS10B
YBR200W	BEM1	YPL018W	CTF19
YNL238W	KEX2	YGL124C	MON1
YPR051W	MAK3	YEL043W	YEL043W
YML071C	COG8	YNL127W	FAR11
YDR202C	RAV2	YAR014C	BUD14
YML103C	NUP188	YLR315W	NKP2
YAL024C	LTE1	YDR261C	EXG2
YPL024W	RMI1	YNL041C	COG6
YFR032C-A	RPL29	YML117W	NAB6
YDR484W	VPS52	YKR099W	BAS1
YJL095W	BCK1	YMR230W	RPS10B
YIL159W	BNR1	YPR119W	CLB2
YDR293C	SSD1	YBR108W	YBR108W
YFR010W	UBP6	YPR030W	CSR2
YNL041C	COG6	YLR370C	ARC18
YJL151C	SNA3	YNL238W	KEX2
YLR313C	SPH1	YJL187C	SWE1
YKL032C	IXR1	YPL256C	CLN2
YDR183W	PLP1	YOL081W	IRA2

Predictions at threshold level 0.75 continued...			
Gene1 of predicted pair		Gene2 of predicted pair	
Systematic Name	Standard Name	Systematic Name	Standard Name
YOR023C	AHC1	YHR012W	VPS29
YPR030W	CSR2	YBR019C	GAL10
YCR065W	HCM1	YDR171W	HSP42
YPL158C	YPL158C	YHL011C	PRS3
YBR217W	ATG12	YJR056C	YJR056C
YGR086C	PIL1	YOL100W	PKH2
YBR108W	YBR108W	YDL083C	RPS16B
YDR127W	ARO1	YPR119W	CLB2
YGL087C	MMS2	YOR014W	RTS1
YNL032W	SIW14	YOR066W	MSA1
YDR443C	SSN2	YLR371W	ROM2
YMR016C	SOK2	YDR484W	VPS52
YLR381W	CTF3	YMR127C	SAS2
YER162C	RAD4	YAR014C	BUD14
YHR178W	STB5	YGL124C	MON1
YGL153W	PEX14	YPL213W	LEA1
YOR080W	DIA2	YDL226C	GCS1
YGR072W	UPF3	YJL004C	SYS1
YFR024C-A	LSB3	YBR239C	YBR239C
YPL024W	RMI1	YDL184C	RPL41A
YLR381W	CTF3	YHR013C	ARD1
YDR080W	VPS41	YPL120W	VPS30
YLR381W	CTF3	YGR270W	YTA7
YLR399C	BDF1	YPR120C	CLB5
YKL135C	APL2	YKR001C	VPS1
YPR084W	YPR084W	YJL124C	LSM1
YDR318W	MCM21	YLR233C	EST1
YOR156C	NFI1	YGL003C	CDH1
YGL054C	ERV14	YNR032W	PPG1

Predictions at threshold level 0.75 continued...			
Gene1 of predicted pair		Gene2 of predicted pair	
Systematic Name	Standard Name	Systematic Name	Standard Name
YNR072W	HXT17	YGL003C	CDH1
YEL009C	GCN4	YCL010C	SGF29
YNL106C	INP52	YLR386W	VAC14
YER115C	SPR6	YKL117W	SBA1
YJR140C	HIR3	YLR315W	NKP2
YKL116C	PRR1	YBR030W	YBR030W
YFR010W	UBP6	YKR035W-A	DID2
YLR090W	XDJ1	YOR270C	VPH1
YBL047C	EDE1	YPL213W	LEA1
YHR012W	VPS29	YAL002W	VPS8
YBL058W	SHP1	YLR180W	SAM1
YPL241C	CIN2	YGL190C	CDC55
YKR020W	VPS51	YOL081W	IRA2
YJL058C	BIT61	YKL114C	APN1
YPL246C	RBD2	YLR144C	ACF2
YGL174W	BUD13	YMR038C	CCS1
YDR293C	SSD1	YHR012W	VPS29
YGL078C	DBP3	YEL053C	MAK10
YDL020C	RPN4	YKL080W	VMA5
YGL212W	VAM7	YOL018C	TLG2
YDR245W	MNN10	YMR311C	GLC8
YGL054C	ERV14	YPR032W	SRO7
YOR327C	SNC2	YLR398C	SKI2
YMR216C	SKY1	YGL223C	COG1
YOR132W	VPS17	YOR106W	VAM3
YKR014C	YPT52	YJR084W	CSN12
YER110C	KAP123	YFL047W	RGD2
YIL076W	SEC28	YGR105W	VMA21
YHR084W	STE12	YDR480W	DIG2

Predictions at threshold level 0.75 continued...			
**Gene1 of predicted pair**		**Gene2 of predicted pair**	
**Systematic Name**	**Standard Name**	**Systematic Name**	**Standard Name**
YNL297C	MON2	YER162C	RAD4
YDR183W	PLP1	YOL054W	PSH1
YMR055C	BUB2	YAR014C	BUD14
YDL020C	RPN4	YCL037C	SRO9
YGL003C	CDH1	YOL081W	IRA2
YPR017C	DSS4	YMR004W	MVP1
YDR439W	LRS4	YKL110C	KTI12
YNL031C	HHT2	YKR048C	NAP1
YMR216C	SKY1	YOR109W	INP53
YIL076W	SEC28	YGL115W	SNF4
YAL024C	LTE1	YDR484W	VPS52
YOR332W	VMA4	YKL080W	VMA5
YCR077C	PAT1	YPL256C	CLN2
YNL025C	SSN8	YAL002W	VPS8
YBL078C	ATG8	YOR327C	SNC2
YKR099W	BAS1	YOL081W	IRA2
YMR016C	SOK2	YOL121C	RPS19A
YPL022W	RAD1	YBR200W	BEM1
YGL025C	PGD1	YMR311C	GLC8
YHR060W	VMA22	YJR033C	RAV1
YDL149W	ATG9	YJR025C	BNA1
YOR368W	RAD17	YOL081W	IRA2
YFR010W	UBP6	YLR150W	STM1
YOR124C	UBP2	YMR038C	CCS1
YIL162W	SUC2	YGL226C-A	OST5
YBL103C	RTG3	YHR200W	RPN10
YDR264C	AKR1	YBL087C	RPL23A
YDR363W-A	SEM1	YKR048C	NAP1
YKL139W	CTK1	YOR327C	SNC2

Predictions at threshold level 0.75 continued...			
Gene1 of predicted pair		Gene2 of predicted pair	
Systematic Name	Standard Name	Systematic Name	Standard Name
YCR063W	BUD31	YBR065C	ECM2
YNL087W	TCB2	YLR398C	SKI2
YFL023W	BUD27	YOR043W	WHI2
YDL083C	RPS16B	YDR207C	UME6
YLR018C	POM34	YGL200C	EMP24
YLR119W	SRN2	YML001W	YPT7
YOR109W	INP53	YML038C	YMD8
YMR004W	MVP1	YGR105W	VMA21
YHR039C-A	VMA10	YJR032W	CPR7
YGR184C	UBR1	YPR046W	MCM16
YJL187C	SWE1	YHR115C	DMA1
YDR254W	CHL4	YDR144C	MKC7
YBR171W	SEC66	YDR254W	CHL4
YLR018C	POM34	YPL178W	CBC2
YDR289C	RTT103	YJL183W	MNN11
YLR399C	BDF1	YGR188C	BUB1
YMR216C	SKY1	YDR207C	UME6
YOR033C	EXO1	YBR228W	SLX1
YDL155W	CLB3	YGL003C	CDH1
YNL044W	YIP3	YNL238W	KEX2
YDR477W	SNF1	YPL161C	BEM4
YIL036W	CST6	YOR141C	ARP8
YGL153W	PEX14	YBL047C	EDE1
YHL020C	OPI1	YNL107W	YAF9
YPL161C	BEM4	YNL116W	DMA2
YGL253W	HXK2	YBR114W	RAD16
YAL024C	LTE1	YPR030W	CSR2
YKL025C	PAN3	YML095C	RAD10
YHR158C	KEL1	YLR425W	TUS1

Predictions at threshold level 0.75 continued...			
Gene1 of predicted pair		Gene2 of predicted pair	
Systematic Name	Standard Name	Systematic Name	Standard Name
YLR386W	VAC14	YKR048C	NAP1
YDR477W	SNF1	YKR099W	BAS1
YER116C	SLX8	YJL124C	LSM1
YMR153W	NUP53	YKR024C	DBP7
YMR152W	YIM1	YMR214W	SCJ1
YLR079W	SIC1	YJR135C	MCM22
YKL139W	CTK1	YJL172W	CPS1
YNR072W	HXT17	YER139C	YER139C
YPL037C	EGD1	YHR200W	RPN10
YNL291C	MID1	YPR032W	SRO7
YDR378C	LSM6	YFR036W	CDC26
YDR443C	SSN2	YIL038C	NOT3
YAL024C	LTE1	YPL115C	BEM3
YNL297C	MON2	YPL120W	VPS30
YOR270C	VPH1	YER044C	ERG28
YDR225W	HTA1	YGR252W	GCN5
YPR051W	MAK3	YPL234C	TFP3
YMR172W	HOT1	YPL018W	CTF19
YOR327C	SNC2	YMR230W	RPS10B
YJR060W	CBF1	YGL163C	RAD54
YOR337W	TEA1	YPL057C	SUR1
YNL106C	INP52	YBR200W	BEM1
YLR381W	CTF3	YLR399C	BDF1
YGR108W	CLB1	YHR114W	BZZ1
YBR108W	YBR108W	YJR084W	CSN12
YKR020W	VPS51	YER162C	RAD4
YIL076W	SEC28	YKR030W	GMH1
YDR477W	SNF1	YMR199W	CLN1
YHR178W	STB5	YDR318W	MCM21

Predictions at threshold level 0.75 continued...			
**Gene1 of predicted pair**		**Gene2 of predicted pair**	
**Systematic Name**	**Standard Name**	**Systematic Name**	**Standard Name**
YLR102C	APC9	YKL101W	HSL1
YOR112W	CEX1	YHR060W	VMA22
YPR017C	DSS4	YDR320C	SWA2
YDR202C	RAV2	YER120W	SCS2
YPL096C-A	ERI1	YBL047C	EDE1
YBR171W	SEC66	YNL058C	YNL058C
YKL139W	CTK1	YEL060C	PRB1
YGL025C	PGD1	YOR043W	WHI2
YER075C	PTP3	YDL006W	PTC1
YBR274W	CHK1	YGL148W	ARO2
YPL050C	MNN9	YKL204W	EAP1
YJL154C	VPS35	YMR199W	CLN1
YIL036W	CST6	YGL163C	RAD54
YDR335W	MSN5	YNL225C	CNM67
YMR016C	SOK2	YHR081W	LRP1
YEL053C	MAK10	YDR155C	CPR1
YDR108W	GSG1	YJR032W	CPR7
YJR090C	GRR1	YDR028C	REG1
YJR060W	CBF1	YJL004C	SYS1
YCR020C-A	MAK31	YML071C	COG8
YDR378C	LSM6	YCR086W	CSM1
YNL215W	IES2	YBR200W	BEM1
YLR399C	BDF1	YLR315W	NKP2
YHR039C-A	VMA10	YEL051W	VMA8
YFR031C-A	RPL2A	YOR069W	VPS5
YLR386W	VAC14	YDR439W	LRS4
YBR030W	YBR030W	YPL037C	EGD1
YLR090W	XDJ1	YBR019C	GAL10
YNL291C	MID1	YDR080W	VPS41

Predictions at threshold level 0.75 continued...			
Gene1 of predicted pair		Gene2 of predicted pair	
Systematic Name	Standard Name	Systematic Name	Standard Name
YBL047C	EDE1	YHR200W	RPN10
YDR225W	HTA1	YBR009C	HHF1
YDR358W	GGA1	YML071C	COG8
YML016C	PPZ1	YKL110C	KTI12
YLR192C	HCR1	YAL002W	VPS8
YHR216W	IMD2	YPR119W	CLB2
YJL204C	RCY1	YJR090C	GRR1
YGL066W	SGF73	YPL018W	CTF19
YPR017C	DSS4	YGL223C	COG1
YNL041C	COG6	YER162C	RAD4
YLR240W	VPS34	YPR173C	VPS4
YNL264C	PDR17	YML097C	VPS9
YHR073W	OSH3	YPL256C	CLN2
YJL151C	SNA3	YJL004C	SYS1
YLR441C	RPS1A	YPR036W	VMA13
YJR060W	CBF1	YDR207C	UME6
YOR127W	RGA1	YLR381W	CTF3
YBR171W	SEC66	YPR030W	CSR2
YPR036W	VMA13	YMR038C	CCS1
YNR023W	SNF12	YHL025W	SNF6
YKL213C	DOA1	YDR289C	RTT103
YDL020C	RPN4	YLR150W	STM1
YLR386W	VAC14	YDR207C	UME6
YGR019W	UGA1	YDR254W	CHL4
YNL307C	MCK1	YPL137C	GIP3
YMR198W	CIK1	YLR370C	ARC18
YEL065W	SIT1	YER122C	GLO3
YBR127C	VMA2	YMR055C	BUB2
YHR200W	RPN10	YOL081W	IRA2

Predictions at threshold level 0.75 continued...			
Gene1 of predicted pair		Gene2 of predicted pair	
Systematic Name	Standard Name	Systematic Name	Standard Name
YPR051W	MAK3	YER120W	SCS2
YKR001C	VPS1	YMR231W	PEP5
YMR243C	ZRC1	YDR423C	CAD1
YJR140C	HIR3	YJL124C	LSM1
YKL048C	ELM1	YHR081W	LRP1
YGL066W	SGF73	YDR363W-A	SEM1
YKL116C	PRR1	YLR386W	VAC14
YPR018W	RLF2	YHR013C	ARD1
YOL043C	NTG2	YMR167W	MLH1
YOR069W	VPS5	YOR035C	SHE4
YLR337C	VRP1	YER122C	GLO3
YKL048C	ELM1	YCR053W	THR4
YDL020C	RPN4	YGL124C	MON1
YIL156W	UBP7	YGL016W	KAP122
YLR447C	VMA6	YNL051W	COG5
YMR164C	MSS11	YML057W	CMP2
YLR090W	XDJ1	YMR133W	REC114
YLR083C	EMP70	YER120W	SCS2
YNL289W	PCL1	YJL187C	SWE1
YMR016C	SOK2	YNL058C	YNL058C
YNL297C	MON2	YCL001W	RER1
YNL076W	MKS1	YCR053W	THR4
YDL020C	RPN4	YDR363W-A	SEM1
YKL110C	KTI12	YAR014C	BUD14
YBL017C	PEP1	YNL238W	KEX2
YDL160C	DHH1	YPL203W	TPK2
YDR293C	SSD1	YDR080W	VPS41
YOR290C	SNF2	YLR094C	GIS3
YOR043W	WHI2	YJR084W	CSN12

Predictions at threshold level 0.75 continued...			
Gene1 of predicted pair		Gene2 of predicted pair	
Systematic Name	Standard Name	Systematic Name	Standard Name
YDR320C	SWA2	YHL020C	OPI1
YCL067C	HMLALPHA2	YGL025C	PGD1
YNL069C	RPL16B	YNL041C	COG6
YLR320W	MMS22	YPL137C	GIP3
YGL078C	DBP3	YBR173C	UMP1
YFR010W	UBP6	YMR276W	DSK2
YLR288C	MEC3	YGL250W	RMR1
YBR200W	BEM1	YGL212W	VAM7
YNL079C	TPM1	YHL011C	PRS3
YMR095C	SNO1	YLR370C	ARC18
YEL044W	IES6	YGR135W	PRE9
YNL206C	RTT106	YDR507C	GIN4
YIR009W	MSL1	YPL213W	LEA1
YBR030W	YBR030W	YLR452C	SST2
YNL297C	MON2	YDR469W	SDC1
YDL020C	RPN4	YHR178W	STB5
YCL001W	RER1	YAL007C	ERP2
YPL001W	HAT1	YBR215W	HPC2
YLR410W	VIP1	YDR318W	MCM21
YPR072W	NOT5	YBR010W	HHT1
YOR141C	ARP8	YDL047W	SIT4
YFR034C	PHO4	YPL248C	GAL4
YIL053W	RHR2	YIL076W	SEC28
YMR224C	MRE11	YDR245W	MNN10
YNL291C	MID1	YOR014W	RTS1
YDR137W	RGP1	YKL119C	VPH2
YJL172W	CPS1	YPR046W	MCM16
YDR192C	NUP42	YDR335W	MSN5
YNL218W	MGS1	YFR010W	UBP6

Predictions at threshold level 0.75 continued...			
Gene1 of predicted pair		Gene2 of predicted pair	
Systematic Name	Standard Name	Systematic Name	Standard Name
YIL076W	SEC28	YDL192W	ARF1
YMR026C	PEX12	YDL065C	PEX19
YML115C	VAN1	YEL053C	MAK10
YML016C	PPZ1	YDR254W	CHL4
YDR245W	MNN10	YEL036C	ANP1
YLR079W	SIC1	YPL018W	CTF19
YBR228W	SLX1	YBL079W	NUP170
YOR243C	PUS7	YPL079W	RPL21B
YML088W	UFO1	YJR090C	GRR1
YAL024C	LTE1	YNR072W	HXT17
YFR031C-A	RPL2A	YLR398C	SKI2
YKL139W	CTK1	YNL087W	TCB2
YGR270W	YTA7	YER161C	SPT2
YER047C	SAP1	YER161C	SPT2
YCR034W	FEN1	YMR055C	BUB2
YPR084W	YPR084W	YOR106W	VAM3
YJL095W	BCK1	YBR114W	RAD16
YNR052C	POP2	YJR135C	MCM22
YPL115C	BEM3	YOR023C	AHC1
YLR385C	SWC7	YNL107W	YAF9
YBR217W	ATG12	YFR036W	CDC26
YER116C	SLX8	YLR452C	SST2
YBR171W	SEC66	YDR309C	GIC2
YHR156C	LIN1	YNR072W	HXT17
YDR484W	VPS52	YBR280C	SAF1
YNL087W	TCB2	YFL023W	BUD27
YPR084W	YPR084W	YJL004C	SYS1
YGL151W	NUT1	YOR368W	RAD17
YPL253C	VIK1	YPL161C	BEM4

Predictions at threshold level 0.75 continued...			
Gene1 of predicted pair		Gene2 of predicted pair	
Systematic Name	Standard Name	Systematic Name	Standard Name
YBR030W	YBR030W	YPL161C	BEM4
YLR240W	VPS34	YHR161C	YAP1801
YFR010W	UBP6	YNL238W	KEX2
YAL031C	GIP4	YCL027W	FUS1
YPL161C	BEM4	YBR260C	RGD1
YDL059C	RAD59	YJR090C	GRR1
YDR469W	SDC1	YBR036C	CSG2
YKL139W	CTK1	YDR137W	RGP1
YCR088W	ABP1	YJL095W	BCK1
YHR171W	ATG7	YNR007C	ATG3
YDR171W	HSP42	YPL106C	SSE1
YDR156W	RPA14	YBR131W	CCZ1
YNL117W	MLS1	YMR038C	CCS1
YLR386W	VAC14	YBL025W	RRN10
YKL048C	ELM1	YNL107W	YAF9
YOR035C	SHE4	YBL025W	RRN10
YJL036W	SNX4	YDR310C	SUM1
YER096W	SHC1	YNL079C	TPM1
YGL053W	PRM8	YAR031W	PRM9
YPL129W	TAF14	YGL151W	NUT1
YOR337W	TEA1	YLR398C	SKI2
YLR090W	XDJ1	YKL048C	ELM1
YNL248C	RPA49	YIL103W	DPH1
YJR032W	CPR7	YOR327C	SNC2
YPL050C	MNN9	YLR093C	NYV1
YBR111W-A	SUS1	YPL181W	CTI6
YNL025C	SSN8	YPL057C	SUR1
YMR198W	CIK1	YLR410W	VIP1
YGR217W	CCH1	YBL101C	ECM21

Predictions at threshold level 0.75 continued...			
Gene1 of predicted pair		Gene2 of predicted pair	
Systematic Name	Standard Name	Systematic Name	Standard Name
YCL001W	RER1	YER122C	GLO3
YLR240W	VPS34	YBR128C	ATG14
YDR245W	MNN10	YDL155W	CLB3
YMR224C	MRE11	YLL039C	UBI4
YBL047C	EDE1	YIL008W	URM1
YDR507C	GIN4	YNL166C	BNI5
YDR293C	SSD1	YPL234C	TFP3
YNL215W	IES2	YNL107W	YAF9
YJL124C	LSM1	YKL160W	ELF1
YKR020W	VPS51	YPR032W	SRO7
YDR129C	SAC6	YBR057C	MUM2
YDR378C	LSM6	YAR014C	BUD14
YPL037C	EGD1	YER122C	GLO3
YGR063C	SPT4	YBR089C-A	NHP6B
YBR030W	YBR030W	YER144C	UBP5
YPR051W	MAK3	YJL004C	SYS1
YOL112W	MSB4	YAL030W	SNC1
YDR335W	MSN5	YLR315W	NKP2
YBR278W	DPB3	YDR289C	RTT103
YLR083C	EMP70	YIL076W	SEC28
YBR217W	ATG12	YMR159C	ATG16
YGR261C	APL6	YDR310C	SUM1
YMR172W	HOT1	YNL166C	BNI5
YGL212W	VAM7	YEL043W	YEL043W
YBL058W	SHP1	YLR093C	NYV1
YPL024W	RMI1	YDL154W	MSH5
YDL160C	DHH1	YPL022W	RAD1
YPR084W	YPR084W	YEL053C	MAK10
YGR104C	SRB5	YOR124C	UBP2

Predictions at threshold level 0.75 continued...			
Gene1 of predicted pair		Gene2 of predicted pair	
Systematic Name	Standard Name	Systematic Name	Standard Name
YDR225W	HTA1	YHR013C	ARD1
YLR262C	YPT6	YHR098C	SFB3
YLR337C	VRP1	YHL002W	HSE1
YIL034C	CAP2	YHR111W	UBA4
YLR381W	CTF3	YDL185W	TFP1
YBR173C	UMP1	YPR030W	CSR2
YFR031C-A	RPL2A	YER044C	ERG28
YDR320C	SWA2	YEL043W	YEL043W
YJL036W	SNX4	YPL120W	VPS30
YOR337W	TEA1	YDL083C	RPS16B
YOR043W	WHI2	YJL095W	BCK1
YDL089W	YDL089W	YCR086W	CSM1
YCR020C-A	MAK31	YER120W	SCS2
YGR249W	MGA1	YMR133W	REC114
YML071C	COG8	YPL195W	APL5
YKR028W	SAP190	YER033C	ZRG8
YDL052C	SLC1	YMR313C	TGL3
YKL007W	CAP1	YJL154C	VPS35
YJL204C	RCY1	YGL198W	YIP4
YCL032W	STE50	YMR273C	ZDS1
YPL115C	BEM3	YJL190C	RPS22A
YBR108W	YBR108W	YPL057C	SUR1
YBL103C	RTG3	YPR030W	CSR2
YGR108W	CLB1	YOR080W	DIA2
YDR004W	RAD57	YGL003C	CDH1
YFL013C	IES1	YIR005W	IST3
YDR137W	RGP1	YCR077C	PAT1
YLR441C	RPS1A	YPL137C	GIP3
YHR012W	VPS29	YPL120W	VPS30

Predictions at threshold level 0.75 continued...			
Gene1 of predicted pair		Gene2 of predicted pair	
Systematic Name	Standard Name	Systematic Name	Standard Name
YDR129C	SAC6	YDR207C	UME6
YML102W	CAC2	YPL256C	CLN2
YEL053C	MAK10	YPL195W	APL5
YPR051W	MAK3	YPL037C	EGD1
YOR132W	VPS17	YOR069W	VPS5
YKR061W	KTR2	YGL163C	RAD54
YBR200W	BEM1	YML095C	RAD10
YHR200W	RPN10	YHR115C	DMA1
YDR443C	SSN2	YBR030W	YBR030W
YLR399C	BDF1	YGR252W	GCN5
YDR477W	SNF1	YKL126W	YPK1
YPR036W	VMA13	YDL192W	ARF1
YKL139W	CTK1	YIL103W	DPH1
YJR056C	YJR056C	YJL095W	BCK1
YIL148W	RPL40A	YMR153W	NUP53
YNL106C	INP52	YNL230C	ELA1
YOR039W	CKB2	YJL095W	BCK1
YLR381W	CTF3	YPL018W	CTF19
YNL025C	SSN8	YER068W	MOT2
YLR447C	VMA6	YJR135C	MCM22
YCL037C	SRO9	YEL043W	YEL043W
YER162C	RAD4	YOR327C	SNC2
YPR052C	NHP6A	YGL071W	AFT1
YOR327C	SNC2	YDL192W	ARF1
YKR101W	SIR1	YML109W	ZDS2
YML103C	NUP188	YER110C	KAP123
YLR425W	TUS1	YKL041W	VPS24
YMR224C	MRE11	YLR313C	SPH1
YDR146C	SWI5	YLR131C	ACE2

Predictions at threshold level 0.75 continued...			
Gene1 of predicted pair		Gene2 of predicted pair	
Systematic Name	Standard Name	Systematic Name	Standard Name
YEL043W	YEL043W	YML001W	YPT7
YMR116C	ASC1	YPL161C	BEM4
YGL178W	MPT5	YDR448W	ADA2
YLR095C	IOC2	YLR180W	SAM1
YDR358W	GGA1	YKL041W	VPS24
YER116C	SLX8	YGL115W	SNF4
YPL042C	SSN3	YBR258C	SHG1
YPL024W	RMI1	YOR001W	RRP6
YBL103C	RTG3	YGL124C	MON1
YLR094C	GIS3	YDR261C	EXG2
YDL223C	HBT1	YPL161C	BEM4
YMR214W	SCJ1	YER040W	GLN3
YBR057C	MUM2	YBR107C	IML3
YBR108W	YBR108W	YML071C	COG8
YOR001W	RRP6	YHR167W	THP2
YDL083C	RPS16B	YLR370C	ARC18
YJR058C	APS2	YAL030W	SNC1
YML115C	VAN1	YGR135W	PRE9
YMR109W	MYO5	YPL018W	CTF19
YJR060W	CBF1	YJR035W	RAD26
YDR378C	LSM6	YFR030W	MET10
YLR360W	VPS38	YBR181C	RPS6B
YPL106C	SSE1	YJR035W	RAD26
YDL160C	DHH1	YJL164C	TPK1
YPL022W	RAD1	YML028W	TSA1
YOR132W	VPS17	YOL018C	TLG2
YNL307C	MCK1	YCR053W	THR4
YMR055C	BUB2	YOR368W	RAD17
YCL032W	STE50	YMR139W	RIM11

Predictions at threshold level 0.75 continued...			
Gene1 of predicted pair		Gene2 of predicted pair	
Systematic Name	Standard Name	Systematic Name	Standard Name
YIL036W	CST6	YCR086W	CSM1
YBR200W	BEM1	YDL192W	ARF1
YDR293C	SSD1	YNL041C	COG6
YBR194W	SOY1	YHR167W	THP2
YKR072C	SIS2	YOL030W	GAS5
YGL147C	RPL9A	YBR217W	ATG12
YDL161W	ENT1	YBR083W	TEC1
YMR055C	BUB2	YGL252C	RTG2
YBR217W	ATG12	YJL172W	CPS1
YIL076W	SEC28	YDR153C	ENT5
YHR156C	LIN1	YHR060W	VMA22
YML115C	VAN1	YPR036W	VMA13
YLR119W	SRN2	YDL184C	RPL41A
YCR039C	MATALPHA2	YMR042W	ARG80
YGR104C	SRB5	YLR357W	RSC2
YNL288W	CAF40	YHR111W	UBA4
YPL022W	RAD1	YLR058C	SHM2
YNR072W	HXT17	YLR398C	SKI2
YJL124C	LSM1	YOR308C	SNU66
YCR024C-A	PMP1	YMR199W	CLN1
YLR452C	SST2	YNL238W	KEX2
YEL065W	SIT1	YLR398C	SKI2
YPL115C	BEM3	YMR109W	MYO5
YNL325C	FIG4	YLR452C	SST2
YMR276W	DSK2	YOR124C	UBP2
YML115C	VAN1	YPR030W	CSR2
YLR150W	STM1	YMR055C	BUB2
YDL020C	RPN4	YCR086W	CSM1
YDR443C	SSN2	YIL053W	RHR2

Predictions at threshold level 0.75 continued...			
Gene1 of predicted pair		Gene2 of predicted pair	
Systematic Name	Standard Name	Systematic Name	Standard Name
YML103C	NUP188	YBR107C	IML3
YPL018W	CTF19	YLR370C	ARC18
YPL195W	APL5	YJR084W	CSN12
YLR102C	APC9	YOR275C	RIM20
YOL111C	MDY2	YMR230W	RPS10B
YER110C	KAP123	YKL117W	SBA1
YBR041W	FAT1	YKL140W	TGL1
YGR019W	UGA1	YHR111W	UBA4
YBL078C	ATG8	YLR093C	NYV1
YPR036W	VMA13	YGL025C	PGD1
YIL034C	CAP2	YDR289C	RTT103
YOR195W	SLK19	YGR135W	PRE9
YOL012C	HTZ1	YBR089C-A	NHP6B
YPL042C	SSN3	YJR140C	HIR3
YDL020C	RPN4	YHR013C	ARD1
YJL154C	VPS35	YJL190C	RPS22A
YLR018C	POM34	YAL055W	PEX22
YOR290C	SNF2	YBR289W	SNF5
YLR320W	MMS22	YGL003C	CDH1
YBL047C	EDE1	YPL158C	YPL158C
YMR172W	HOT1	YER110C	KAP123
YDR202C	RAV2	YGR020C	VMA7
YLR083C	EMP70	YOR132W	VPS17
YLL039C	UBI4	YOL081W	IRA2
YHR200W	RPN10	YOR213C	SAS5
YGL003C	CDH1	YOL091W	SPO21
YOL111C	MDY2	YJL095W	BCK1
YDL155W	CLB3	YAL002W	VPS8
YGL161C	YIP5	YDL002C	NHP10

Predictions at threshold level 0.75 continued...			
Gene1 of predicted pair		Gene2 of predicted pair	
Systematic Name	Standard Name	Systematic Name	Standard Name
YOR127W	RGA1	YGR014W	MSB2
YBR030W	YBR030W	YNL032W	SIW14
YNL025C	SSN8	YPL042C	SSN3
YCR020C-A	MAK31	YOR106W	VAM3
YML019W	OST6	YEL036C	ANP1
YML103C	NUP188	YLR381W	CTF3
YLR150W	STM1	YDR076W	RAD55
YPL045W	VPS16	YGL095C	VPS45
YDR293C	SSD1	YPL161C	BEM4
YNL307C	MCK1	YLR315W	NKP2
YBR200W	BEM1	YGL237C	HAP2
YHR205W	SCH9	YPL256C	CLN2
YLR094C	GIS3	YLR371W	ROM2
YDR358W	GGA1	YDR310C	SUM1
YJL124C	LSM1	YOR124C	UBP2
YAR014C	BUD14	YPR032W	SRO7
YNL215W	IES2	YHR115C	DMA1
YNL248C	RPA49	YDR254W	CHL4
YLR360W	VPS38	YNL291C	MID1
YOR038C	HIR2	YEL056W	HAT2
YGL161C	YIP5	YKL032C	IXR1
YJL117W	PHO86	YBR082C	UBC4
YGL087C	MMS2	YNL291C	MID1
YLL042C	ATG10	YMR159C	ATG16
YOR212W	STE4	YMR139W	RIM11
YOR337W	TEA1	YKR035W-A	DID2
YJL084C	ALY2	YIL050W	PCL7
YGL033W	HOP2	YLR386W	VAC14
YDR156W	RPA14	YOL081W	IRA2

Predictions at threshold level 0.75 continued...			
Gene1 of predicted pair		Gene2 of predicted pair	
Systematic Name	Standard Name	Systematic Name	Standard Name
YHR178W	STB5	YPR046W	MCM16
YDL002C	NHP10	YMR038C	CCS1
YFR010W	UBP6	YJL183W	MNN11
YDR129C	SAC6	YOR035C	SHE4
YNL288W	CAF40	YLR386W	VAC14
YJR056C	YJR056C	YPL161C	BEM4
YDR320C	SWA2	YNL041C	COG6
YER161C	SPT2	YBR010W	HHT1
YML058W	SML1	YDL059C	RAD59
YLR398C	SKI2	YEL060C	PRB1
YBR173C	UMP1	YGL212W	VAM7
YMR273C	ZDS1	YHR167W	THP2
YER161C	SPT2	YBR036C	CSG2
YPL089C	RLM1	YBR082C	UBC4
YJL176C	SWI3	YBR181C	RPS6B
YMR216C	SKY1	YAR014C	BUD14
YNL025C	SSN8	YDR448W	ADA2
YBL008W	HIR1	YOR001W	RRP6
YNL215W	IES2	YML097C	VPS9
YJL053W	PEP8	YOL108C	INO4
YER144C	UBP5	YMR214W	SCJ1
YPR051W	MAK3	YNL051W	COG5
YKL032C	IXR1	YDR378C	LSM6
YMR261C	TPS3	YFL033C	RIM15
YMR153W	NUP53	YLR320W	MMS22
YBL051C	PIN4	YDR289C	RTT103
YHR081W	LRP1	YDL192W	ARF1
YGL054C	ERV14	YHR039C-A	VMA10
YHL031C	GOS1	YBR131W	CCZ1

Predictions at threshold level 0.75 continued...			
Gene1 of predicted pair		Gene2 of predicted pair	
Systematic Name	Standard Name	Systematic Name	Standard Name
YOR140W	SFL1	YPL203W	TPK2
YOR213C	SAS5	YAL002W	VPS8
YOR231W	MKK1	YJL095W	BCK1
YJL157C	FAR1	YJR086W	STE18
YNL206C	RTT106	YGL031C	RPL24A
YHR060W	VMA22	YJL183W	MNN11
YBR171W	SEC66	YNL307C	MCK1
YMR004W	MVP1	YPL161C	BEM4
YGL147C	RPL9A	YLR452C	SST2
YDR202C	RAV2	YJR060W	CBF1
YNL135C	FPR1	YIL133C	RPL16A
YDL002C	NHP10	YMR055C	BUB2
YDR363W-A	SEM1	YHR111W	UBA4
YNL215W	IES2	YER139C	YER139C
YIL076W	SEC28	YDR202C	RAV2
YDR440W	DOT1	YLR370C	ARC18
YNL087W	TCB2	YNR072W	HXT17
YPR051W	MAK3	YJR032W	CPR7
YFR032C-A	RPL29	YJL095W	BCK1
YDL006W	PTC1	YJL183W	MNN11
YMR224C	MRE11	YJR032W	CPR7
YGR270W	YTA7	YAR014C	BUD14
YDL116W	NUP84	YNL215W	IES2
YDL077C	VAM6	YDR153C	ENT5
YPR072W	NOT5	YPL248C	GAL4
YDR245W	MNN10	YKL204W	EAP1
YIL034C	CAP2	YNL135C	FPR1
YDR202C	RAV2	YKR020W	VPS51
YKR059W	TIF1	YKL101W	HSL1

Predictions at threshold level 0.75 continued...			
Gene1 of predicted pair		Gene2 of predicted pair	
Systematic Name	Standard Name	Systematic Name	Standard Name
YOR191W	RIS1	YNL106C	INP52
YNL068C	FKH2	YHR111W	UBA4
YOR337W	TEA1	YGL003C	CDH1
YFR036W	CDC26	YOL081W	IRA2
YKL110C	KTI12	YIR005W	IST3
YJL004C	SYS1	YOL108C	INO4
YLR441C	RPS1A	YNL238W	KEX2
YDR245W	MNN10	YOR008C	SLG1
YGR072W	UPF3	YGL115W	SNF4
YEL044W	IES6	YKR020W	VPS51
YNL325C	FIG4	YNL041C	COG6
YPL037C	EGD1	YLR371W	ROM2
YAR014C	BUD14	YOL108C	INO4
YML001W	YPT7	YOR327C	SNC2
YJL029C	VPS53	YLR396C	VPS33
YDR245W	MNN10	YER161C	SPT2
YPL240C	HSP82	YHR072W-A	NOP10
YBL051C	PIN4	YBR114W	RAD16
YHR156C	LIN1	YMR153W	NUP53
YJL053W	PEP8	YDR323C	PEP7
YJL176C	SWI3	YBR042C	CST26
YHL031C	GOS1	YOR106W	VAM3
YER096W	SHC1	YER049W	TPA1
YLR148W	PEP3	YDR129C	SAC6
YDL116W	NUP84	YDR192C	NUP42
YML097C	VPS9	YLR398C	SKI2
YML115C	VAN1	YPL253C	VIK1
YOL090W	MSH2	YNL082W	PMS1
YER162C	RAD4	YDR207C	UME6

Predictions at threshold level 0.75 continued...			
Gene1 of predicted pair		Gene2 of predicted pair	
Systematic Name	Standard Name	Systematic Name	Standard Name
YNL058C	YNL058C	YOL111C	MDY2
YDR245W	MNN10	YBR195C	MSI1
YMR127C	SAS2	YNL107W	YAF9
YLL007C	YLL007C	YPL161C	BEM4
YGR105W	VMA21	YER162C	RAD4
YPL022W	RAD1	YPR046W	MCM16
YLR447C	VMA6	YKL119C	VPH2
YJR024C	MDE1	YML103C	NUP188
YER116C	SLX8	YLR371W	ROM2
YKL139W	CTK1	YER162C	RAD4
YLR425W	TUS1	YOL081W	IRA2
YBL058W	SHP1	YHR200W	RPN10
YNL106C	INP52	YDR389W	SAC7
YLR386W	VAC14	YKL126W	YPK1
YJL036W	SNX4	YGL054C	ERV14
YPL115C	BEM3	YDL020C	RPN4
YMR216C	SKY1	YGL054C	ERV14
YLR095C	IOC2	YDL192W	ARF1
YHL002W	HSE1	YKL041W	VPS24
YNL291C	MID1	YPL018W	CTF19
YJL053W	PEP8	YPR051W	MAK3
YIL034C	CAP2	YKL048C	ELM1
YOR069W	VPS5	YJL204C	RCY1
YDL116W	NUP84	YML057W	CMP2
YCR077C	PAT1	YHL002W	HSE1
YGR159C	NSR1	YHR052W	CIC1
YEL053C	MAK10	YOR035C	SHE4
YJL176C	SWI3	YKL139W	CTK1
YDL020C	RPN4	YDL013W	SLX5

Predictions at threshold level 0.75 continued...			
Gene1 of predicted pair		Gene2 of predicted pair	
Systematic Name	Standard Name	Systematic Name	Standard Name
YOR106W	VAM3	YNL199C	GCR2
YML058W	SML1	YLR032W	RAD5
YGL212W	VAM7	YOR043W	WHI2
YEL027W	CUP5	YMR054W	STV1
YKR099W	BAS1	YGL025C	PGD1
YNL087W	TCB2	YHR039C-A	VMA10
YFR030W	MET10	YDR318W	MCM21
YER143W	DDI1	YMR276W	DSK2
YDR439W	LRS4	YHR111W	UBA4
YHR060W	VMA22	YDL192W	ARF1
YJL124C	LSM1	YLR410W	VIP1
YKR035W-A	DID2	YER162C	RAD4
YEL009C	GCN4	YBR009C	HHF1
YDR137W	RGP1	YOR066W	MSA1
YOL051W	GAL11	YBR089C-A	NHP6B
YJR040W	GEF1	YDR310C	SUM1
YMR198W	CIK1	YJL058C	BIT61
YKL139W	CTK1	YJR060W	CBF1
YCR034W	FEN1	YCR031C	RPS14A
YGR072W	UPF3	YOR243C	PUS7
YLR417W	VPS36	YNL243W	SLA2
YPR052C	NHP6A	YPL129W	TAF14
YFL023W	BUD27	YDL192W	ARF1
YDR174W	HMO1	YLR315W	NKP2
YFR031C-A	RPL2A	YFR036W	CDC26
YDL006W	PTC1	YJL004C	SYS1
YNL069C	RPL16B	YOR112W	CEX1
YFR031C-A	RPL2A	YKR099W	BAS1
YOR327C	SNC2	YEL036C	ANP1

Predictions at threshold level 0.75 continued...			
Gene1 of predicted pair		Gene2 of predicted pair	
Systematic Name	Standard Name	Systematic Name	Standard Name
YGL100W	SEH1	YCL061C	MRC1
YHR156C	LIN1	YOR290C	SNF2
YDL116W	NUP84	YGL115W	SNF4
YIL034C	CAP2	YBL103C	RTG3
YLR095C	IOC2	YBR200W	BEM1
YHR134W	WSS1	YJL095W	BCK1
YKR072C	SIS2	YGL151W	NUT1
YOR023C	AHC1	YDR289C	RTT103
YML062C	MFT1	YPR046W	MCM16
YHR178W	STB5	YBR107C	IML3
YBR200W	BEM1	YLR254C	NDL1
YOR033C	EXO1	YPL024W	RMI1
YBL058W	SHP1	YER116C	SLX8
YNL291C	MID1	YDR260C	SWM1
YGL115W	SNF4	YER139C	YER139C
YLR288C	MEC3	YPL001W	HAT1
YOR270C	VPH1	YKL048C	ELM1
YGL043W	DST1	YBR089C-A	NHP6B
YPR036W	VMA13	YMR311C	GLC8
YAL010C	MDM10	YDR183W	PLP1
YCR020C-A	MAK31	YGL212W	VAM7
YLR248W	RCK2	YDL047W	SIT4
YDR443C	SSN2	YDL192W	ARF1
YML115C	VAN1	YBR131W	CCZ1
YKL092C	BUD2	YPR119W	CLB2
YFR010W	UBP6	YPL161C	BEM4
YHR134W	WSS1	YBR228W	SLX1
YER180C-A	SLO1	YPL051W	ARL3
YKL126W	YPK1	YNR072W	HXT17

Predictions at threshold level 0.75 continued...			
Gene1 of predicted pair		Gene2 of predicted pair	
Systematic Name	Standard Name	Systematic Name	Standard Name
YDL155W	CLB3	YLR233C	EST1
YGR270W	YTA7	YCR086W	CSM1
YHR156C	LIN1	YNL087W	TCB2
YHR060W	VMA22	YHR200W	RPN10
YNL238W	KEX2	YJL095W	BCK1
YFR036W	CDC26	YJL095W	BCK1
YBR231C	SWC5	YBR089C-A	NHP6B
YJR060W	CBF1	YDL047W	SIT4
YDR363W-A	SEM1	YER143W	DDI1
YJL006C	CTK2	YBL072C	RPS8A
YHL031C	GOS1	YKL160W	ELF1
YNL206C	RTT106	YDR254W	CHL4
YJL058C	BIT61	YNL107W	YAF9
YDL116W	NUP84	YDL020C	RPN4
YCR077C	PAT1	YOR124C	UBP2
YLR119W	SRN2	YML115C	VAN1
YPL234C	TFP3	YKL080W	VMA5
YDL077C	VAM6	YMR230W	RPS10B
YMR039C	SUB1	YBR036C	CSG2
YDL002C	NHP10	YBR215W	HPC2
YLR234W	TOP3	YDR207C	UME6
YMR068W	AVO2	YNL047C	SLM2
YLR309C	IMH1	YDR310C	SUM1
YOR069W	VPS5	YML038C	YMD8
YOR035C	SHE4	YBR019C	GAL10
YHR178W	STB5	YCR086W	CSM1
YLL039C	UBI4	YML115C	VAN1
YOR127W	RGA1	YDR207C	UME6
YGL087C	MMS2	YKL048C	ELM1

Predictions at threshold level 0.75 continued...			
Gene1 of predicted pair		Gene2 of predicted pair	
Systematic Name	Standard Name	Systematic Name	Standard Name
YDR443C	SSN2	YNL199C	GCR2
YPR018W	RLF2	YLR399C	BDF1
YLR018C	POM34	YOR080W	DIA2
YHR134W	WSS1	YML016C	PPZ1
YOR109W	INP53	YPR173C	VPS4
YDR484W	VPS52	YGL115W	SNF4
YMR142C	RPL13B	YLR094C	GIS3
YDL099W	BUG1	YKL050C	YKL050C
YBL103C	RTG3	YBR280C	SAF1
YGL178W	MPT5	YDL074C	BRE1
YDL184C	RPL41A	YNL076W	MKS1
YJL164C	TPK1	YNR049C	MSO1
YLL007C	YLL007C	YDR155C	CPR1
YMR055C	BUB2	YLR254C	NDL1
YBR126C	TPS1	YBR036C	CSG2
YBL058W	SHP1	YOR066W	MSA1
YOR127W	RGA1	YNL041C	COG6
YMR004W	MVP1	YGL223C	COG1
YPL024W	RMI1	YNL238W	KEX2
YPL253C	VIK1	YOR368W	RAD17
YJL190C	RPS22A	YOL067C	RTG1
YPR036W	VMA13	YLR447C	VMA6
YEL013W	VAC8	YLR350W	ORM2
YBL058W	SHP1	YHR039C-A	VMA10
YLR386W	VAC14	YER139C	YER139C
YLR234W	TOP3	YLR370C	ARC18
YDR004W	RAD57	YDR335W	MSN5
YKL010C	UFD4	YCR077C	PAT1
YIL053W	RHR2	YMR214W	SCJ1

Predictions at threshold level 0.75 continued...			
Gene1 of predicted pair		Gene2 of predicted pair	
Systematic Name	Standard Name	Systematic Name	Standard Name
YDR383C	NKP1	YPL018W	CTF19
YDR254W	CHL4	YBR114W	RAD16
YCL037C	SRO9	YPL105C	YPL105C
YDR225W	HTA1	YDR216W	ADR1
YLR079W	SIC1	YGL016W	KAP122
YKL048C	ELM1	YLR452C	SST2
YIL162W	SUC2	YML019W	OST6
YGL033W	HOP2	YER116C	SLX8
YMR095C	SNO1	YJL183W	MNN11
YBR215W	HPC2	YMR231W	PEP5
YMR224C	MRE11	YBL079W	NUP170
YGL025C	PGD1	YGL124C	MON1
YNL032W	SIW14	YJR032W	CPR7
YER110C	KAP123	YGR188C	BUB1
YBR217W	ATG12	YER144C	UBP5
YDR385W	EFT2	YDR358W	GGA1
YGL033W	HOP2	YOR043W	WHI2
YBL008W	HIR1	YFR036W	CDC26
YBR057C	MUM2	YOR014W	RTS1
YKR072C	SIS2	YHR039C-A	VMA10
YJL058C	BIT61	YNL236W	SIN4
YNL291C	MID1	YGR184C	UBR1
YBR200W	BEM1	YDL159W	STE7
YOR312C	RPL20B	YBL072C	RPS8A
YBR107C	IML3	YPL106C	SSE1
YDR146C	SWI5	YHR152W	SPO12
YJL154C	VPS35	YDR484W	VPS52
YMR016C	SOK2	YPL057C	SUR1
YOR275C	RIM20	YMR038C	CCS1

Predictions at threshold level 0.75 continued...			
Gene1 of predicted pair		Gene2 of predicted pair	
Systematic Name	Standard Name	Systematic Name	Standard Name
YDR363W-A	SEM1	YLR350W	ORM2
YHR098C	SFB3	YDR334W	SWR1
YDR443C	SSN2	YOL081W	IRA2
YLR292C	SEC72	YDR310C	SUM1
YGR072W	UPF3	YGR020C	VMA7
YGR020C	VMA7	YNL051W	COG5
YDR456W	NHX1	YEL036C	ANP1
YGL033W	HOP2	YBR030W	YBR030W
YJL060W	BNA3	YBR200W	BEM1
YBR036C	CSG2	YBR280C	SAF1
YLR441C	RPS1A	YEL053C	MAK10
YLR360W	VPS38	YER096W	SHC1
YCR002C	CDC10	YNL166C	BNI5
YJR040W	GEF1	YDR153C	ENT5
YBL103C	RTG3	YLR370C	ARC18
YDL223C	HBT1	YLR452C	SST2
YGR105W	VMA21	YPL195W	APL5
YMR205C	PFK2	YOR275C	RIM20
YDR443C	SSN2	YJR056C	YJR056C
YJL004C	SYS1	YOR284W	HUA2
YPL240C	HSP82	YGL094C	PAN2
YPR030W	CSR2	YOR327C	SNC2
YML103C	NUP188	YHR013C	ARD1
YBR107C	IML3	YNL107W	YAF9
YJL036W	SNX4	YEL043W	YEL043W
YGL250W	RMR1	YMR167W	MLH1
YLR083C	EMP70	YDR320C	SWA2
YLR093C	NYV1	YDL192W	ARF1
YOR112W	CEX1	YFL033C	RIM15

Predictions at threshold level 0.75 continued...			
Gene1 of predicted pair		Gene2 of predicted pair	
Systematic Name	Standard Name	Systematic Name	Standard Name
YAL024C	LTE1	YNL087W	TCB2
YOR195W	SLK19	YIR005W	IST3
YOR043W	WHI2	YNL238W	KEX2
YBR258C	SHG1	YNL107W	YAF9
YLR192C	HCR1	YPL137C	GIP3
YGL163C	RAD54	YKR048C	NAP1
YKL129C	MYO3	YMR032W	HOF1
YOR001W	RRP6	YLR381W	CTF3
YER144C	UBP5	YLR180W	SAM1
YGR020C	VMA7	YDR137W	RGP1
YLR090W	XDJ1	YPL161C	BEM4
YLR240W	VPS34	YKR001C	VPS1
YNL087W	TCB2	YLR180W	SAM1
YGL133W	ITC1	YJL065C	DLS1
YPL129W	TAF14	YDR216W	ADR1
YCR086W	CSM1	YLR182W	SWI6
YGL060W	YBP2	YLR370C	ARC18
YLR191W	PEX13	YGL153W	PEX14
YNL058C	YNL058C	YBR036C	CSG2
YLR083C	EMP70	YJL004C	SYS1
YPL037C	EGD1	YDL192W	ARF1
YOR270C	VPH1	YGL003C	CDH1
YNL069C	RPL16B	YEL051W	VMA8
YGR261C	APL6	YPL259C	APM1
YOR195W	SLK19	YMR199W	CLN1
YBR009C	HHF1	YNL107W	YAF9
YNL243W	SLA2	YER122C	GLO3
YGR072W	UPF3	YNL069C	RPL16B
YGL066W	SGF73	YPR119W	CLB2

Predictions at threshold level 0.75 continued...			
Gene1 of predicted pair		Gene2 of predicted pair	
Systematic Name	Standard Name	Systematic Name	Standard Name
YIL053W	RHR2	YER044C	ERG28
YOR033C	EXO1	YML095C	RAD10
YIL159W	BNR1	YCR053W	THR4
YNL025C	SSN8	YOR140W	SFL1
YLR234W	TOP3	YGL253W	HXK2
YPR046W	MCM16	YOR327C	SNC2
YGL078C	DBP3	YJL183W	MNN11
YLR386W	VAC14	YBL021C	HAP3
YFR034C	PHO4	YHR200W	RPN10
YDL020C	RPN4	YLR032W	RAD5
YPL240C	HSP82	YGL192W	IME4
YIL005W	EPS1	YKL117W	SBA1
YFL023W	BUD27	YLR371W	ROM2
YKR061W	KTR2	YCR086W	CSM1
YNL166C	BNI5	YMR109W	MYO5
YER001W	MNN1	YNL041C	COG6
YPL256C	CLN2	YGL016W	KAP122
YDR142C	PEX7	YGR077C	PEX8
YOR066W	MSA1	YOL081W	IRA2
YNL291C	MID1	YOL090W	MSH2
YJL053W	PEP8	YJL004C	SYS1
YNL297C	MON2	YOL018C	TLG2
YDL116W	NUP84	YGL016W	KAP122
YAL016W	TPD3	YPL152W	RRD2
YFR036W	CDC26	YDR289C	RTT103
YKL126W	YPK1	YKL048C	ELM1
YOR290C	SNF2	YER040W	GLN3
YBR200W	BEM1	YDL083C	RPS16B
YOR069W	VPS5	YPR051W	MAK3

Predictions at threshold level 0.75 continued...			
Gene1 of predicted pair		Gene2 of predicted pair	
Systematic Name	Standard Name	Systematic Name	Standard Name
YDR293C	SSD1	YDR108W	GSG1
YMR224C	MRE11	YPL137C	GIP3
YJR056C	YJR056C	YER162C	RAD4
YGR270W	YTA7	YFR030W	MET10
YAR014C	BUD14	YDR310C	SUM1
YDL020C	RPN4	YHR077C	NMD2
YJL137C	GLG2	YLR258W	GSY2
YPR051W	MAK3	YDR310C	SUM1
YOL121C	RPS19A	YBR019C	GAL10
YJR135C	MCM22	YPL018W	CTF19
YGL212W	VAM7	YGR135W	PRE9
YOR275C	RIM20	YKR035W-A	DID2
YCL063W	VAC17	YPR119W	CLB2
YHR205W	SCH9	YOR106W	VAM3
YJR140C	HIR3	YHR167W	THP2
YOR185C	GSP2	YLR131C	ACE2
YNL199C	GCR2	YKR024C	DBP7
YGR261C	APL6	YKR001C	VPS1
YHR205W	SCH9	YDR264C	AKR1
YPL024W	RMI1	YDR310C	SUM1
YLL039C	UBI4	YBR057C	MUM2
YAL014C	SYN8	YGL095C	VPS45
YKR001C	VPS1	YGL095C	VPS45
YBR126C	TPS1	YEL053C	MAK10
YDR383C	NKP1	YDR254W	CHL4
YLR090W	XDJ1	YBR280C	SAF1
YLR240W	VPS34	YGR241C	YAP1802
YDR477W	SNF1	YOR014W	RTS1
YJL053W	PEP8	YJL029C	VPS53

Predictions at threshold level 0.75 continued...			
Gene1 of predicted pair		Gene2 of predicted pair	
Systematic Name	Standard Name	Systematic Name	Standard Name
YIL076W	SEC28	YNL297C	MON2
YDR289C	RTT103	YJL095W	BCK1
YJR032W	CPR7	YDR155C	CPR1
YOR337W	TEA1	YBR217W	ATG12
YER044C	ERG28	YLR320W	MMS22
YCL067C	HMLALPHA2	YLR176C	RFX1
YPL129W	TAF14	YOR290C	SNF2
YDL042C	SIR2	YNL030W	HHF2
YLR288C	MEC3	YLR234W	TOP3
YBR108W	YBR108W	YDL155W	CLB3
YDR378C	LSM6	YBL072C	RPS8A
YAL056W	GPB2	YGL121C	GPG1
YPL105C	YPL105C	YNL107W	YAF9
YGL078C	DBP3	YGL212W	VAM7
YJL187C	SWE1	YER027C	GAL83
YDL232W	OST4	YML019W	OST6
YDR469W	SDC1	YGR135W	PRE9
YHR060W	VMA22	YEL060C	PRB1
YLR079W	SIC1	YML115C	VAN1
YGL071W	AFT1	YBR112C	CYC8
YHR156C	LIN1	YIL076W	SEC28
YLR337C	VRP1	YMR172W	HOT1
YGL066W	SGF73	YDR076W	RAD55
YDR335W	MSN5	YAR014C	BUD14
YDR144C	MKC7	YHL002W	HSE1
YMR095C	SNO1	YDR137W	RGP1
YOR106W	VAM3	YPL045W	VPS16
YFR031C-A	RPL2A	YOR127W	RGA1
YPR036W	VMA13	YNL058C	YNL058C

Predictions at threshold level 0.75 continued...			
Gene1 of predicted pair		Gene2 of predicted pair	
Systematic Name	Standard Name	Systematic Name	Standard Name
YPR036W	VMA13	YBR127C	VMA2
YKL139W	CTK1	YPL161C	BEM4
YMR198W	CIK1	YMR179W	SPT21
YDR477W	SNF1	YKL048C	ELM1
YLR381W	CTF3	YJL095W	BCK1
YJL127C	SPT10	YOR141C	ARP8
YHR134W	WSS1	YPL079W	RPL21B
YHR156C	LIN1	YBR030W	YBR030W
YER161C	SPT2	YGL163C	RAD54
YOR043W	WHI2	YLR093C	NYV1
YHR052W	CIC1	YJL190C	RPS22A
YNR052C	POP2	YJR063W	RPA12
YGL147C	RPL9A	YLR093C	NYV1
YDL223C	HBT1	YPR030W	CSR2
YMR080C	NAM7	YKL068W	NUP100
YOR080W	DIA2	YLR429W	CRN1
YNL236W	SIN4	YDL155W	CLB3
YMR224C	MRE11	YDR129C	SAC6
YGL212W	VAM7	YKR030W	GMH1
YKL025C	PAN3	YGL124C	MON1
YGR261C	APL6	YOL108C	INO4
YOR375C	GDH1	YMR089C	YTA12
YNL238W	KEX2	YPL120W	VPS30
YPL161C	BEM4	YGL115W	SNF4
YGL087C	MMS2	YML028W	TSA1
YMR109W	MYO5	YEL053C	MAK10
YPL256C	CLN2	YMR311C	GLC8
YNL238W	KEX2	YNL107W	YAF9
YDR174W	HMO1	YPR046W	MCM16

Predictions at threshold level 0.75 continued...			
Gene1 of predicted pair		Gene2 of predicted pair	
Systematic Name	Standard Name	Systematic Name	Standard Name
YHR052W	CIC1	YJL124C	LSM1
YOR038C	HIR2	YKL109W	HAP4
YLR292C	SEC72	YNL107W	YAF9
YPL161C	BEM4	YDL006W	PTC1
YOR195W	SLK19	YDR363W-A	SEM1
YGR104C	SRB5	YFL013C	IES1
YKL213C	DOA1	YLR315W	NKP2
YGL223C	COG1	YER162C	RAD4
YPL105C	YPL105C	YMR054W	STV1
YPL178W	CBC2	YNL199C	GCR2
YFR036W	CDC26	YAR014C	BUD14
YLR192C	HCR1	YOR327C	SNC2
YPL042C	SSN3	YBR010W	HHT1
YDL006W	PTC1	YJR033C	RAV1
YOR080W	DIA2	YDL155W	CLB3
YKL126W	YPK1	YHR039C-A	VMA10
YDL232W	OST4	YGL226C-A	OST5
YNL243W	SLA2	YPL161C	BEM4
YGR020C	VMA7	YGL115W	SNF4
YIL053W	RHR2	YMR016C	SOK2
YOR191W	RIS1	YHR134W	WSS1
YFR010W	UBP6	YGR188C	BUB1
YDR293C	SSD1	YNL051W	COG5
YLR337C	VRP1	YML016C	PPZ1
YDR439W	LRS4	YML016C	PPZ1
YBR131W	CCZ1	YGL124C	MON1
YNL201C	PSY2	YPL018W	CTF19
YHL031C	GOS1	YGL200C	EMP24
YDL184C	RPL41A	YDL223C	HBT1

Predictions at threshold level 0.75 continued...			
Gene1 of predicted pair		Gene2 of predicted pair	
Systematic Name	Standard Name	Systematic Name	Standard Name
YGL003C	CDH1	YDL192W	ARF1
YLR182W	SWI6	YKR030W	GMH1
YEL053C	MAK10	YOR327C	SNC2
YBR020W	GAL1	YNL107W	YAF9
YLR079W	SIC1	YKL110C	KTI12
YPR018W	RLF2	YNL107W	YAF9
YOR270C	VPH1	YOR043W	WHI2
YML115C	VAN1	YCR077C	PAT1
YGL223C	COG1	YJR033C	RAV1
YGL158W	RCK1	YNL065W	AQR1
YJL030W	MAD2	YHR205W	SCH9
YBR171W	SEC66	YDR144C	MKC7
YKL139W	CTK1	YLR094C	GIS3
YDR156W	RPA14	YDR318W	MCM21
YMR116C	ASC1	YDR378C	LSM6
YLR386W	VAC14	YDR144C	MKC7
YLR018C	POM34	YCR024C-A	PMP1
YMR016C	SOK2	YOR270C	VPH1
YOR127W	RGA1	YJR032W	CPR7
YPL115C	BEM3	YJL095W	BCK1
YMR026C	PEX12	YER116C	SLX8
YER129W	SAK1	YJR090C	GRR1
YIL034C	CAP2	YML095C	RAD10
YLR192C	HCR1	YOR270C	VPH1
YOR008C	SLG1	YLR371W	ROM2
YKL025C	PAN3	YIL008W	URM1
YML117W	NAB6	YML115C	VAN1
YNL041C	COG6	YBR019C	GAL10
YOL145C	CTR9	YOL051W	GAL11

Predictions at threshold level 0.75 continued...			
Gene1 of predicted pair		Gene2 of predicted pair	
Systematic Name	Standard Name	Systematic Name	Standard Name
YLR079W	SIC1	YML016C	PPZ1
YNL069C	RPL16B	YEL043W	YEL043W
YGL147C	RPL9A	YDR137W	RGP1
YLR095C	IOC2	YBL058W	SHP1
YDR318W	MCM21	YHR114W	BZZ1
YIL034C	CAP2	YEL056W	HAT2
YGL212W	VAM7	YDR137W	RGP1
YHR160C	PEX18	YDR244W	PEX5
YOL018C	TLG2	YJR033C	RAV1
YGR019W	UGA1	YDR318W	MCM21
YNL106C	INP52	YNL248C	RPA49
YML103C	NUP188	YER116C	SLX8
YNL068C	FKH2	YGR135W	PRE9
YNL289W	PCL1	YDR309C	GIC2
YPL115C	BEM3	YEL053C	MAK10
YGR249W	MGA1	YMR311C	GLC8
YOR038C	HIR2	YER122C	GLO3
YMR199W	CLN1	YIL008W	URM1
YLR234W	TOP3	YML102W	CAC2
YDR137W	RGP1	YLR447C	VMA6
YOR080W	DIA2	YLR131C	ACE2
YHL020C	OPI1	YLR309C	IMH1
YDL116W	NUP84	YOR368W	RAD17
YML095C	RAD10	YJL058C	BIT61
YPL024W	RMI1	YLR441C	RPS1A
YAL024C	LTE1	YOR270C	VPH1
YIL036W	CST6	YJR063W	RPA12
YPL112C	PEX25	YMR204C	INP1
YDR261C	EXG2	YJL095W	BCK1

Predictions at threshold level 0.75 continued...			
**Gene1 of predicted pair**		**Gene2 of predicted pair**	
**Systematic Name**	**Standard Name**	**Systematic Name**	**Standard Name**
YLR018C	POM34	YJR024C	MDE1
YBR010W	HHT1	YPL248C	GAL4
YDL020C	RPN4	YMR038C	CCS1
YBR260C	RGD1	YMR032W	HOF1
YNL325C	FIG4	YJL095W	BCK1
YEL060C	PRB1	YDR207C	UME6
YDL160C	DHH1	YPR119W	CLB2
YEL015W	EDC3	YER124C	DSE1
YMR032W	HOF1	YHR114W	BZZ1
YFR036W	CDC26	YGL252C	RTG2
YLR234W	TOP3	YGL175C	SAE2
YOR080W	DIA2	YML028W	TSA1
YNL079C	TPM1	YLR425W	TUS1
YPR036W	VMA13	YDL077C	VAM6
YHR161C	YAP1801	YNR006W	VPS27
YDR293C	SSD1	YJR033C	RAV1
YDR146C	SWI5	YBR195C	MSI1
YEL027W	CUP5	YKL080W	VMA5
YBR107C	IML3	YML112W	CTK3
YGR275W	RTT102	YNL041C	COG6
YLR371W	ROM2	YGL003C	CDH1
YLR006C	SSK1	YDR309C	GIC2
YGL060W	YBP2	YOR195W	SLK19
YDL155W	CLB3	YJL190C	RPS22A
YBR108W	YBR108W	YGL151W	NUT1
YHR178W	STB5	YHR012W	VPS29
YKR020W	VPS51	YPL234C	TFP3
YJR084W	CSN12	YER122C	GLO3
YDR245W	MNN10	YJL095W	BCK1

Predictions at threshold level 0.75 continued...			
Gene1 of predicted pair		Gene2 of predicted pair	
Systematic Name	Standard Name	Systematic Name	Standard Name
YPL042C	SSN3	YDL155W	CLB3
YBR195C	MSI1	YJR035W	RAD26
YLR083C	EMP70	YPL105C	YPL105C
YPL042C	SSN3	YBR200W	BEM1
YIL002C	INP51	YKR020W	VPS51
YKR061W	KTR2	YOL054W	PSH1
YHR013C	ARD1	YBL101C	ECM21
YLR234W	TOP3	YOL030W	GAS5
YBR131W	CCZ1	YJR032W	CPR7
YDR358W	GGA1	YJR084W	CSN12
YAL024C	LTE1	YOR038C	HIR2
YNL206C	RTT106	YDR318W	MCM21
YKR020W	VPS51	YDR440W	DOT1
YNL041C	COG6	YDL192W	ARF1
YJR140C	HIR3	YGL163C	RAD54
YLR094C	GIS3	YML071C	COG8
YLR315W	NKP2	YNL225C	CNM67
YLR095C	IOC2	YBR280C	SAF1
YHL031C	GOS1	YGL115W	SNF4
YOR344C	TYE7	YOL108C	INO4
YLR386W	VAC14	YDR363W-A	SEM1
YOR080W	DIA2	YIR005W	IST3
YDR261C	EXG2	YLR180W	SAM1
YER116C	SLX8	YMR214W	SCJ1
YBR131W	CCZ1	YLR396C	VPS33
YKR035W-A	DID2	YOL081W	IRA2
YDR448W	ADA2	YLR176C	RFX1
YDR097C	MSH6	YNL082W	PMS1
YBL047C	EDE1	YFR036W	CDC26

Predictions at threshold level 0.75 continued...			
Gene1 of predicted pair		Gene2 of predicted pair	
Systematic Name	Standard Name	Systematic Name	Standard Name
YOR308C	SNU66	YGR188C	BUB1
YML115C	VAN1	YOR080W	DIA2
YDL155W	CLB3	YDL226C	GCS1
YJR090C	GRR1	YML028W	TSA1
YLR386W	VAC14	YLR320W	MMS22
YLR447C	VMA6	YDR183W	PLP1
YNL054W	VAC7	YGR270W	YTA7
YHR160C	PEX18	YAR042W	SWH1
YBR217W	ATG12	YJL183W	MNN11
YML041C	VPS71	YBR089C-A	NHP6B
YMR153W	NUP53	YCR024C-A	PMP1
YOR127W	RGA1	YDR153C	ENT5
YGL237C	HAP2	YJR135C	MCM22
YMR224C	MRE11	YOR035C	SHE4
YKL126W	YPK1	YLR398C	SKI2
YPR051W	MAK3	YNL041C	COG6
YML103C	NUP188	YNL107W	YAF9
YOR290C	SNF2	YJL095W	BCK1
YMR199W	CLN1	YML016C	PPZ1
YJL095W	BCK1	YBL087C	RPL23A
YMR055C	BUB2	YDR076W	RAD55
YLR337C	VRP1	YMR109W	MYO5
YOR039W	CKB2	YKL101W	HSL1
YIL159W	BNR1	YHR039C-A	VMA10
YGL033W	HOP2	YER122C	GLO3
YCR066W	RAD18	YHR060W	VMA22
YLR265C	NEJ1	YGL090W	LIF1
YNL215W	IES2	YJR140C	HIR3
YPL129W	TAF14	YCR084C	TUP1

Predictions at threshold level 0.75 continued...			
Gene1 of predicted pair		Gene2 of predicted pair	
Systematic Name	Standard Name	Systematic Name	Standard Name
YNL325C	FIG4	YGL003C	CDH1
YDR245W	MNN10	YMR038C	CCS1
YKL048C	ELM1	YPR030W	CSR2
YBL051C	PIN4	YJR135C	MCM22
YLR058C	SHM2	YJR035W	RAD26
YBR108W	YBR108W	YKR030W	GMH1
YDR153C	ENT5	YAL002W	VPS8
YMR224C	MRE11	YNR052C	POP2
YGR105W	VMA21	YER072W	VTC1
YLR018C	POM34	YDR076W	RAD55
YJR060W	CBF1	YNL238W	KEX2
YLR386W	VAC14	YJR084W	CSN12
YPL024W	RMI1	YLR150W	STM1
YNR072W	HXT17	YER122C	GLO3
YCR024C-A	PMP1	YFL047W	RGD2
YLR234W	TOP3	YDR245W	MNN10
YJL060W	BNA3	YJR035W	RAD26
YPR046W	MCM16	YDL226C	GCS1
YNL229C	URE2	YPL256C	CLN2
YBR200W	BEM1	YOR066W	MSA1
YDR076W	RAD55	YEL056W	HAT2
YIL053W	RHR2	YPL037C	EGD1
YBR173C	UMP1	YGL124C	MON1
YOR076C	SKI7	YLR135W	SLX4
YBR171W	SEC66	YHL027W	RIM101
YOL006C	TOP1	YHR056C	RSC30
YFL023W	BUD27	YER139C	YER139C
YLR094C	GIS3	YPR030W	CSR2
YJL095W	BCK1	YHR115C	DMA1

Predictions at threshold level 0.75 continued...			
**Gene1 of predicted pair**		**Gene2 of predicted pair**	
**Systematic Name**	**Standard Name**	**Systematic Name**	**Standard Name**
YNL135C	FPR1	YKR048C	NAP1
YGL163C	RAD54	YHR010W	RPL27A
YEL065W	SIT1	YHR108W	GGA2
YGL033W	HOP2	YGL003C	CDH1
YBR065C	ECM2	YIR005W	IST3
YDR293C	SSD1	YFL033C	RIM15
YGR135W	PRE9	YNL116W	DMA2
YLR360W	VPS38	YDR484W	VPS52
YOR337W	TEA1	YDL192W	ARF1
YDR293C	SSD1	YHL002W	HSE1
YBR290W	BSD2	YBL051C	PIN4
YBR200W	BEM1	YHR111W	UBA4
YPL253C	VIK1	YLR233C	EST1
YHR200W	RPN10	YOL121C	RPS19A
YJR135C	MCM22	YEL056W	HAT2
YPL152W	RRD2	YNL225C	CNM67
YPL234C	TFP3	YML071C	COG8
YAL024C	LTE1	YNL206C	RTT106
YBL051C	PIN4	YAL002W	VPS8
YBR200W	BEM1	YLR452C	SST2
YMR125W	STO1	YGL016W	KAP122
YLR079W	SIC1	YPL253C	VIK1
YDR129C	SAC6	YPL213W	LEA1
YBR289W	SNF5	YDR073W	SNF11
YPR018W	RLF2	YDR225W	HTA1
YLR370C	ARC18	YDR207C	UME6
YNL288W	CAF40	YOR358W	HAP5
YDR192C	NUP42	YDL059C	RAD59
YHL020C	OPI1	YML001W	YPT7

Predictions at threshold level 0.75 continued...			
Gene1 of predicted pair		Gene2 of predicted pair	
Systematic Name	Standard Name	Systematic Name	Standard Name
YDL116W	NUP84	YOL126C	MDH2
YBR108W	YBR108W	YGL253W	HXK2
YHR013C	ARD1	YKL110C	KTI12
YIL159W	BNR1	YHR115C	DMA1
YDR320C	SWA2	YOR106W	VAM3
YLR094C	GIS3	YBR036C	CSG2
YNL288W	CAF40	YJR032W	CPR7
YDR320C	SWA2	YER122C	GLO3
YHR012W	VPS29	YNL127W	FAR11
YOR332W	VMA4	YDL161W	ENT1
YOR195W	SLK19	YDR507C	GIN4
YLR119W	SRN2	YJL183W	MNN11
YKR101W	SIR1	YDR310C	SUM1
YDR076W	RAD55	YOR014W	RTS1
YPL022W	RAD1	YFL023W	BUD27
YGL060W	YBP2	YDL226C	GCS1
YGR104C	SRB5	YDR335W	MSN5
YGR241C	YAP1802	YDR129C	SAC6
YDR477W	SNF1	YJL183W	MNN11
YGR270W	YTA7	YJR135C	MCM22
YBL102W	SFT2	YPL234C	TFP3
YBL051C	PIN4	YGL253W	HXK2
YGR249W	MGA1	YKL139W	CTK1
YER054C	GIP2	YLR263W	RED1
YGL249W	ZIP2	YLR394W	CST9
YDR383C	NKP1	YNL106C	INP52
YGL078C	DBP3	YDL192W	ARF1
YGL134W	PCL10	YER059W	PCL6
YDR440W	DOT1	YOR124C	UBP2

Predictions at threshold level 0.75 continued...			
Gene1 of predicted pair		Gene2 of predicted pair	
Systematic Name	Standard Name	Systematic Name	Standard Name
YBR200W	BEM1	YDR335W	MSN5
YHR160C	PEX18	YIL160C	POT1
YER068W	MOT2	YJL164C	TPK1
YDR378C	LSM6	YOR038C	HIR2
YBL008W	HIR1	YBR036C	CSG2
YPL057C	SUR1	YEL060C	PRB1
YOL148C	SPT20	YBR010W	HHT1
YOR043W	WHI2	YGL151W	NUT1
YEL053C	MAK10	YGL003C	CDH1
YOR327C	SNC2	YCR053W	THR4
YGL147C	RPL9A	YKL048C	ELM1
YNL291C	MID1	YHR013C	ARD1
YGL066W	SGF73	YKL139W	CTK1
YBR173C	UMP1	YNL087W	TCB2
YFR010W	UBP6	YOL054W	PSH1
YNL032W	SIW14	YER116C	SLX8
YLR371W	ROM2	YDR358W	GGA1
YJR090C	GRR1	YOL067C	RTG1
YFR031C-A	RPL2A	YOR106W	VAM3
YPR040W	TIP41	YHR111W	UBA4
YGL087C	MMS2	YOR312C	RPL20B
YEL043W	YEL043W	YMR054W	STV1
YPL112C	PEX25	YLR324W	PEX30
YOR243C	PUS7	YPL057C	SUR1
YLR135W	SLX4	YBL079W	NUP170
YGL147C	RPL9A	YLR398C	SKI2
YGR270W	YTA7	YDR440W	DOT1
YGR003W	CUL3	YLR128W	DCN1
YGR081C	SLX9	YPL256C	CLN2

Predictions at threshold level 0.75 continued...			
Gene1 of predicted pair		Gene2 of predicted pair	
Systematic Name	Standard Name	Systematic Name	Standard Name
YIL005W	EPS1	YJR032W	CPR7
YNL054W	VAC7	YLR254C	NDL1
YOR270C	VPH1	YEL051W	VMA8
YDR329C	PEX3	YGR239C	PEX21
YGR020C	VMA7	YPR032W	SRO7
YDR146C	SWI5	YHL011C	PRS3
YBR041W	FAT1	YPL089C	RLM1
YER107C	GLE2	YNL107W	YAF9
YGL087C	MMS2	YDL155W	CLB3
YKR099W	BAS1	YOR066W	MSA1
YMR095C	SNO1	YBR126C	TPS1
YKL139W	CTK1	YBR280C	SAF1
YDR378C	LSM6	YER161C	SPT2
YGL003C	CDH1	YMR230W	RPS10B
YPR084W	YPR084W	YPL120W	VPS30
YLR360W	VPS38	YHR111W	UBA4
YEL043W	YEL043W	YHR039C-A	VMA10
YJL210W	PEX2	YDR265W	PEX10
YPL057C	SUR1	YKR099W	BAS1
YDL116W	NUP84	YDR183W	PLP1
YOR195W	SLK19	YIL103W	DPH1
YHL027W	RIM101	YOR035C	SHE4
YGR261C	APL6	YPL120W	VPS30
YHR073W	OSH3	YJR059W	PTK2
YLL007C	YLL007C	YLR337C	VRP1
YIL076W	SEC28	YDR363W-A	SEM1
YJR135C	MCM22	YDR469W	SDC1
YBR019C	GAL10	YJR084W	CSN12
YNL106C	INP52	YLR315W	NKP2

Predictions at threshold level 0.75 continued...			
**Gene1 of predicted pair**		**Gene2 of predicted pair**	
**Systematic Name**	**Standard Name**	**Systematic Name**	**Standard Name**
YML115C	VAN1	YCR086W	CSM1
YOR132W	VPS17	YPR051W	MAK3
YDR254W	CHL4	YGL163C	RAD54
YLR079W	SIC1	YJL157C	FAR1
YDL002C	NHP10	YKL160W	ELF1
YEL056W	HAT2	YGR188C	BUB1
YKL048C	ELM1	YGL163C	RAD54
YJL154C	VPS35	YPL195W	APL5
YKL139W	CTK1	YDR484W	VPS52
YBR131W	CCZ1	YPL195W	APL5
YNL068C	FKH2	YPL256C	CLN2
YLR337C	VRP1	YDR129C	SAC6
YJR125C	ENT3	YDL137W	ARF2
YBR290W	BSD2	YBR200W	BEM1
YNL058C	YNL058C	YPL161C	BEM4
YHR012W	VPS29	YDR144C	MKC7
YAL024C	LTE1	YDL080C	THI3
YPL050C	MNN9	YLR370C	ARC18
YLR119W	SRN2	YDR207C	UME6
YPR017C	DSS4	YFL033C	RIM15
YNL106C	INP52	YJR140C	HIR3
YGR249W	MGA1	YMR016C	SOK2
YDR358W	GGA1	YJL095W	BCK1
YJR032W	CPR7	YBR280C	SAF1
YHL031C	GOS1	YCR077C	PAT1
YDR439W	LRS4	YLR410W	VIP1
YOL031C	SIL1	YMR214W	SCJ1
YDR443C	SSN2	YNL032W	SIW14
YPR036W	VMA13	YPL137C	GIP3

Predictions at threshold level 0.75 continued...			
Gene1 of predicted pair		Gene2 of predicted pair	
Systematic Name	Standard Name	Systematic Name	Standard Name
YGL200C	EMP24	YNL238W	KEX2
YNL116W	DMA2	YAR014C	BUD14
YLR093C	NYV1	YJR084W	CSN12
YKL048C	ELM1	YAR014C	BUD14
YLR360W	VPS38	YDR108W	GSG1
YDL047W	SIT4	YGL031C	RPL24A
YMR255W	GFD1	YMR273C	ZDS1
YJL053W	PEP8	YCL037C	SRO9
YCL032W	STE50	YHR061C	GIC1
YFR010W	UBP6	YER144C	UBP5
YGL135W	RPL1B	YJR084W	CSN12
YGL066W	SGF73	YHR152W	SPO12
YDL223C	HBT1	YDL077C	VAM6
YNL076W	MKS1	YBR280C	SAF1
YBR228W	SLX1	YNL248C	RPA49
YGL212W	VAM7	YGL003C	CDH1
YNL068C	FKH2	YDR363W-A	SEM1
YOR035C	SHE4	YMR311C	GLC8
YIL053W	RHR2	YBR019C	GAL10
YLL007C	YLL007C	YGR270W	YTA7
YHR200W	RPN10	YDR071C	PAA1
YDR254W	CHL4	YGL003C	CDH1
YGL033W	HOP2	YLR452C	SST2
YPL018W	CTF19	YLR032W	RAD5
YBL021C	HAP3	YDR183W	PLP1
YGR104C	SRB5	YJR060W	CBF1
YML071C	COG8	YLR370C	ARC18
YLR090W	XDJ1	YFR010W	UBP6
YDR335W	MSN5	YNL307C	MCK1

Predictions at threshold level 0.75 continued...			
Gene1 of predicted pair		Gene2 of predicted pair	
Systematic Name	Standard Name	Systematic Name	Standard Name
YOR127W	RGA1	YDR080W	VPS41
YNL094W	APP1	YBR108W	YBR108W
YNL085W	MKT1	YMR127C	SAS2
YGL078C	DBP3	YER040W	GLN3
YHL020C	OPI1	YDR310C	SUM1
YKL126W	YPK1	YMR311C	GLC8
YCR081W	SRB8	YBR200W	BEM1
YLR192C	HCR1	YDL083C	RPS16B
YHR081W	LRP1	YER162C	RAD4
YOL121C	RPS19A	YCR053W	THR4
YJL036W	SNX4	YFL033C	RIM15
YLR360W	VPS38	YEL053C	MAK10
YDL047W	SIT4	YDL226C	GCS1
YPR017C	DSS4	YKR030W	GMH1
YPR131C	NAT3	YJL058C	BIT61
YAL024C	LTE1	YMR095C	SNO1
YPL057C	SUR1	YLR182W	SWI6
YDR137W	RGP1	YKR099W	BAS1
YHR205W	SCH9	YDR207C	UME6
YER120W	SCS2	YKR030W	GMH1
YKL048C	ELM1	YER139C	YER139C
YAL024C	LTE1	YLR371W	ROM2
YOR080W	DIA2	YLR313C	SPH1
YGR217W	CCH1	YJL095W	BCK1
YDL184C	RPL41A	YLR398C	SKI2
YEL043W	YEL043W	YPL195W	APL5
YOL111C	MDY2	YER044C	ERG28
YPR051W	MAK3	YCR066W	RAD18
YBL051C	PIN4	YGL151W	NUT1

Predictions at threshold level 0.75 continued...			
Gene1 of predicted pair		Gene2 of predicted pair	
Systematic Name	Standard Name	Systematic Name	Standard Name
YPR072W	NOT5	YKL048C	ELM1
YPR131C	NAT3	YJR090C	GRR1
YDL020C	RPN4	YJL176C	SWI3
YHR205W	SCH9	YPL240C	HSP82
YDR440W	DOT1	YBL101C	ECM21
YPL161C	BEM4	YDL155W	CLB3
YOR043W	WHI2	YGR188C	BUB1
YLR292C	SEC72	YHR114W	BZZ1
YHR200W	RPN10	YDR358W	GGA1
YBR289W	SNF5	YDR071C	PAA1
YPR120C	CLB5	YFR036W	CDC26
YML016C	PPZ1	YDR245W	MNN10
YPR018W	RLF2	YOR213C	SAS5
YHR084W	STE12	YPR119W	CLB2
YPR072W	NOT5	YBR289W	SNF5
YCR066W	RAD18	YDR153C	ENT5
YDL116W	NUP84	YGL003C	CDH1
YDR183W	PLP1	YOL067C	RTG1
YDR245W	MNN10	YDR076W	RAD55
YDL042C	SIR2	YBR010W	HHT1
YGL033W	HOP2	YBR108W	YBR108W
YCR086W	CSM1	YNL107W	YAF9
YDL077C	VAM6	YKR030W	GMH1
YNL166C	BNI5	YBR114W	RAD16
YOR197W	MCA1	YPL050C	MNN9
YGR019W	UGA1	YMR055C	BUB2
YMR224C	MRE11	YBL072C	RPS8A
YHR081W	LRP1	YCR053W	THR4
YJR060W	CBF1	YGR188C	BUB1

Predictions at threshold level 0.75 continued...			
Gene1 of predicted pair		Gene2 of predicted pair	
Systematic Name	Standard Name	Systematic Name	Standard Name
YDR335W	MSN5	YHR115C	DMA1
YHR081W	LRP1	YLR180W	SAM1
YCR038C	BUD5	YGL054C	ERV14
YNL243W	SLA2	YOR327C	SNC2
YAL024C	LTE1	YMR152W	YIM1
YDL160C	DHH1	YPL042C	SSN3
YPL195W	APL5	YLR309C	IMH1
YJL154C	VPS35	YNL069C	RPL16B
YBR107C	IML3	YMR109W	MYO5
YOL111C	MDY2	YBR036C	CSG2
YDL160C	DHH1	YKL101W	HSL1
YMR199W	CLN1	YKR057W	RPS21A
YLR309C	IMH1	YDL138W	RGT2
YLR381W	CTF3	YDR076W	RAD55
YGL253W	HXK2	YNL107W	YAF9
YEL051W	VMA8	YOR327C	SNC2
YLR381W	CTF3	YJR035W	RAD26
YDL059C	RAD59	YBL079W	NUP170
YDR071C	PAA1	YOR064C	YNG1
YPL001W	HAT1	YGL151W	NUT1
YHR014W	SPO13	YOR109W	INP53
YPL115C	BEM3	YPL253C	VIK1
YNL054W	VAC7	YOR141C	ARP8
YLR315W	NKP2	YPR046W	MCM16
YKL139W	CTK1	YJL140W	RPB4
YMR109W	MYO5	YBR114W	RAD16
YDR156W	RPA14	YJR135C	MCM22
YLR386W	VAC14	YHR114W	BZZ1
YGR249W	MGA1	YBR173C	UMP1

Predictions at threshold level 0.75 continued...			
Gene1 of predicted pair		Gene2 of predicted pair	
Systematic Name	Standard Name	Systematic Name	Standard Name
YGL066W	SGF73	YDR335W	MSN5
YIL162W	SUC2	YBR283C	SSH1
YKR001C	VPS1	YFL033C	RIM15
YIL007C	NAS2	YPL112C	PEX25
YGL124C	MON1	YLR398C	SKI2
YGL006W	PMC1	YDR080W	VPS41
YJL154C	VPS35	YBR127C	VMA2
YBR084C-A	RPL19A	YGR135W	PRE9
YOR327C	SNC2	YNR032W	PPG1
YKL213C	DOA1	YJL095W	BCK1
YGL147C	RPL9A	YDR358W	GGA1
YCR024C-A	PMP1	YPL256C	CLN2
YGR135W	PRE9	YJL095W	BCK1
YJL004C	SYS1	YAR014C	BUD14
YNL325C	FIG4	YER044C	ERG28
YDR080W	VPS41	YDR153C	ENT5
YKR035W-A	DID2	YJL183W	MNN11
YGL087C	MMS2	YOR195W	SLK19
YDR245W	MNN10	YDL223C	HBT1
YOR039W	CKB2	YFR034C	PHO4
YHR156C	LIN1	YLR370C	ARC18
YDL192W	ARF1	YJR033C	RAV1
YNL106C	INP52	YHR013C	ARD1
YGR080W	TWF1	YLR429W	CRN1
YAL024C	LTE1	YER114C	BOI2
YBR194W	SOY1	YDR318W	MCM21
YCR024C-A	PMP1	YKL068W	NUP100
YLR371W	ROM2	YER139C	YER139C
YNL236W	SIN4	YPR046W	MCM16

Predictions at threshold level 0.75 continued...			
Gene1 of predicted pair		Gene2 of predicted pair	
Systematic Name	Standard Name	Systematic Name	Standard Name
YGR142W	BTN2	YKR014C	YPT52
YLR093C	NYV1	YGL124C	MON1
YOR270C	VPH1	YGR105W	VMA21
YOR124C	UBP2	YER122C	GLO3
YJL095W	BCK1	YML097C	VPS9
YKR061W	KTR2	YNL248C	RPA49
YGL078C	DBP3	YER139C	YER139C
YPL161C	BEM4	YEL053C	MAK10
YJL154C	VPS35	YOL018C	TLG2
YNL041C	COG6	YDL006W	PTC1
YPL152W	RRD2	YNL107W	YAF9
YMR198W	CIK1	YEL056W	HAT2
YOR112W	CEX1	YNL127W	FAR11
YLR119W	SRN2	YGR249W	MGA1
YIR003W	YIR003W	YJL095W	BCK1
YHR060W	VMA22	YLR094C	GIS3
YKL116C	PRR1	YHR200W	RPN10
YNL243W	SLA2	YJR075W	HOC1
YBR107C	IML3	YPL152W	RRD2
YDR289C	RTT103	YGL151W	NUT1
YIL159W	BNR1	YGL033W	HOP2
YGR188C	BUB1	YGL031C	RPL24A
YBL008W	HIR1	YDR146C	SWI5
YML001W	YPT7	YNL076W	MKS1
YNR072W	HXT17	YPL137C	GIP3
YIL076W	SEC28	YLR093C	NYV1
YDR153C	ENT5	YNR006W	VPS27
YDL155W	CLB3	YPL256C	CLN2
YDR028C	REG1	YPR119W	CLB2

Predictions at threshold level 0.75 continued...			
Gene1 of predicted pair		Gene2 of predicted pair	
Systematic Name	Standard Name	Systematic Name	Standard Name
YKR035W-A	DID2	YBL025W	RRN10
YDR146C	SWI5	YNL079C	TPM1
YGR020C	VMA7	YEL053C	MAK10
YBR194W	SOY1	YOR080W	DIA2
YLR288C	MEC3	YJR032W	CPR7
YGL212W	VAM7	YFR036W	CDC26
YNL199C	GCR2	YDR310C	SUM1
YBL051C	PIN4	YPL161C	BEM4
YLR150W	STM1	YDL155W	CLB3
YPR030W	CSR2	YEL060C	PRB1
YOR109W	INP53	YPL120W	VPS30
YIL053W	RHR2	YKL048C	ELM1
YHR039C-A	VMA10	YHR081W	LRP1
YNL243W	SLA2	YGR166W	KRE11
YFL023W	BUD27	YFR036W	CDC26
YDR174W	HMO1	YJR063W	RPA12
YGR184C	UBR1	YPL177C	CUP9
YBL047C	EDE1	YBR181C	RPS6B
YGR270W	YTA7	YEL056W	HAT2
YLR135W	SLX4	YOR243C	PUS7
YDR174W	HMO1	YIL036W	CST6
YEL009C	GCN4	YNR023W	SNF12
YKR020W	VPS51	YJL095W	BCK1
YJR040W	GEF1	YPL037C	EGD1
YDL184C	RPL41A	YOR270C	VPH1
YCR086W	CSM1	YJL095W	BCK1
YKR020W	VPS51	YJL006C	CTK2
YJL058C	BIT61	YJR005W	APL1
YLR371W	ROM2	YHR081W	LRP1

Predictions at threshold level 0.75 continued...			
Gene1 of predicted pair		Gene2 of predicted pair	
Systematic Name	Standard Name	Systematic Name	Standard Name
YPR017C	DSS4	YHR039C-A	VMA10
YNL309W	STB1	YFR040W	SAP155
YPL042C	SSN3	YML016C	PPZ1
YMR273C	ZDS1	YMR125W	STO1
YDR225W	HTA1	YCR084C	TUP1
YLR254C	NDL1	YPL018W	CTF19
YFR031C-A	RPL2A	YDR484W	VPS52
YBR200W	BEM1	YBL016W	FUS3
YMR039C	SUB1	YBR195C	MSI1
YMR004W	MVP1	YKR001C	VPS1
YJL053W	PEP8	YGR105W	VMA21
YGL087C	MMS2	YFR036W	CDC26
YKR020W	VPS51	YDL047W	SIT4
YMR004W	MVP1	YDL192W	ARF1
YBR107C	IML3	YGL151W	NUT1
YNL248C	RPA49	YCR086W	CSM1
YLR320W	MMS22	YER162C	RAD4
YFR032C-A	RPL29	YPL161C	BEM4
YLR292C	SEC72	YOR358W	HAP5
YBR173C	UMP1	YGL163C	RAD54
YPL253C	VIK1	YNL116W	DMA2
YFL023W	BUD27	YLR441C	RPS1A
YDL223C	HBT1	YCR053W	THR4
YNL032W	SIW14	YOR290C	SNF2
YHL020C	OPI1	YDL006W	PTC1
YOR290C	SNF2	YJR056C	YJR056C
YPR046W	MCM16	YOR141C	ARP8
YOR270C	VPH1	YLR398C	SKI2
YOR337W	TEA1	YHR081W	LRP1

Predictions at threshold level 0.75 continued...			
Gene1 of predicted pair		Gene2 of predicted pair	
Systematic Name	Standard Name	Systematic Name	Standard Name
YDR153C	ENT5	YAR014C	BUD14
YDR293C	SSD1	YER068W	MOT2
YNR052C	POP2	YJL187C	SWE1
YMR026C	PEX12	YLR093C	NYV1
YDL223C	HBT1	YLR371W	ROM2
YGL124C	MON1	YOR208W	PTP2
YKR020W	VPS51	YLR370C	ARC18
YKL160W	ELF1	YMR038C	CCS1
YKL129C	MYO3	YOR247W	SRL1
YGL212W	VAM7	YMR216C	SKY1
YNL288W	CAF40	YDR155C	CPR1
YHR013C	ARD1	YHR108W	GGA2
YDL160C	DHH1	YPL178W	CBC2
YPL024W	RMI1	YPR119W	CLB2
YLR441C	RPS1A	YJR032W	CPR7
YMR026C	PEX12	YGL025C	PGD1
YBR200W	BEM1	YNL116W	DMA2
YGL078C	DBP3	YBR036C	CSG2
YGR249W	MGA1	YNL087W	TCB2
YML115C	VAN1	YGL115W	SNF4
YJL006C	CTK2	YGR188C	BUB1
YBR131W	CCZ1	YKL119C	VPH2
YDR144C	MKC7	YJR032W	CPR7
YMR039C	SUB1	YHR039C-A	VMA10
YMR172W	HOT1	YLR360W	VPS38
YNL166C	BNI5	YDR144C	MKC7
YOR033C	EXO1	YLR288C	MEC3
YIL008W	URM1	YGL124C	MON1
YIR005W	IST3	YGR188C	BUB1

Predictions at threshold level 0.75 continued...			
Gene1 of predicted pair		Gene2 of predicted pair	
Systematic Name	Standard Name	Systematic Name	Standard Name
YLR094C	GIS3	YLR398C	SKI2
YBR108W	YBR108W	YHR016C	YSC84
YNR049C	MSO1	YAL030W	SNC1
YIL160C	POT1	YGL153W	PEX14
YDR174W	HMO1	YLR079W	SIC1
YMR116C	ASC1	YCR086W	CSM1
YDL116W	NUP84	YDR254W	CHL4
YDL047W	SIT4	YNL107W	YAF9
YMR216C	SKY1	YNL127W	FAR11
YNL199C	GCR2	YCR077C	PAT1
YGL078C	DBP3	YMR214W	SCJ1
YNL325C	FIG4	YJR084W	CSN12
YDR225W	HTA1	YLR150W	STM1
YDL002C	NHP10	YLR182W	SWI6
YEL009C	GCN4	YGR104C	SRB5
YEL053C	MAK10	YNL127W	FAR11
YLR216C	CPR6	YBR057C	MUM2
YML001W	YPT7	YER139C	YER139C
YLR148W	PEP3	YGL095C	VPS45
YPR084W	YPR084W	YFR010W	UBP6
YBL051C	PIN4	YGR188C	BUB1
YFL048C	EMP47	YER122C	GLO3
YFR036W	CDC26	YOR014W	RTS1
YBR200W	BEM1	YDR289C	RTT103
YKR061W	KTR2	YGR188C	BUB1
YDL077C	VAM6	YLR180W	SAM1
YMR055C	BUB2	YCR031C	RPS14A
YOR069W	VPS5	YEL043W	YEL043W
YML103C	NUP188	YDL059C	RAD59

Predictions at threshold level 0.75 continued...			
Gene1 of predicted pair		Gene2 of predicted pair	
Systematic Name	Standard Name	Systematic Name	Standard Name
YMR142C	RPL13B	YPL195W	APL5
YLL039C	UBI4	YNL079C	TPM1
YJL176C	SWI3	YNR023W	SNF12
YJL006C	CTK2	YJL095W	BCK1
YBR107C	IML3	YDR378C	LSM6
YBR274W	CHK1	YLR435W	TSR2
YLR216C	CPR6	YOR208W	PTP2
YPL240C	HSP82	YOR180C	DCI1
YKL213C	DOA1	YOR308C	SNU66
YNR052C	POP2	YHR013C	ARD1
YDR443C	SSN2	YHR205W	SCH9
YLR399C	BDF1	YCR084C	TUP1
YNL248C	RPA49	YJR135C	MCM22
YOR290C	SNF2	YMR311C	GLC8
YDL116W	NUP84	YMR231W	PEP5
YLR093C	NYV1	YML028W	TSA1
YMR080C	NAM7	YCL037C	SRO9
YNL032W	SIW14	YKR099W	BAS1
YKR020W	VPS51	YDR207C	UME6
YDR363W-A	SEM1	YGR270W	YTA7
YCR020C-A	MAK31	YPL120W	VPS30
YCL037C	SRO9	YER122C	GLO3
YLR119W	SRN2	YBL025W	RRN10
YLR150W	STM1	YOR014W	RTS1
YDR261C	EXG2	YMR230W	RPS10B
YJL154C	VPS35	YLR447C	VMA6
YCL067C	HMLALPHA2	YML099C	ARG81
YOR035C	SHE4	YDR310C	SUM1
YGR135W	PRE9	YPL018W	CTF19

Predictions at threshold level 0.75 continued...			
Gene1 of predicted pair		Gene2 of predicted pair	
Systematic Name	Standard Name	Systematic Name	Standard Name
YBR127C	VMA2	YOR035C	SHE4
YKR020W	VPS51	YDL155W	CLB3
YLR337C	VRP1	YKL129C	MYO3
YLR452C	SST2	YDL077C	VAM6
YLR234W	TOP3	YBR107C	IML3
YDL116W	NUP84	YOR141C	ARP8
YMR109W	MYO5	YJL095W	BCK1
YKL034W	TUL1	YBR082C	UBC4
YJR140C	HIR3	YDL002C	NHP10
YLR447C	VMA6	YDR318W	MCM21
YBR217W	ATG12	YMR214W	SCJ1
YNL206C	RTT106	YPL018W	CTF19
YNL215W	IES2	YJR135C	MCM22
YPL046C	ELC1	YDR422C	SIP1
YBR173C	UMP1	YKL139W	CTK1
YLR018C	POM34	YJR090C	GRR1
YGR104C	SRB5	YPL248C	GAL4
YPL037C	EGD1	YDL077C	VAM6
YML095C	RAD10	YOR275C	RIM20
YLR381W	CTF3	YDR254W	CHL4
YNL297C	MON2	YLR309C	IMH1
YFR010W	UBP6	YER116C	SLX8
YPL042C	SSN3	YPL129W	TAF14
YDL116W	NUP84	YGR059W	SPR3
YHR081W	LRP1	YMR230W	RPS10B
YBL058W	SHP1	YER122C	GLO3
YPL022W	RAD1	YHR010W	RPL27A
YOR106W	VAM3	YPL253C	VIK1
YGL066W	SGF73	YBR173C	UMP1

Predictions at threshold level 0.75 continued...			
Gene1 of predicted pair		Gene2 of predicted pair	
Systematic Name	Standard Name	Systematic Name	Standard Name
YDR146C	SWI5	YDR456W	NHX1
YNL238W	KEX2	YJR084W	CSN12
YDL127W	PCL2	YKR001C	VPS1
YKR061W	KTR2	YPL018W	CTF19
YOR076C	SKI7	YOR243C	PUS7
YDL065C	PEX19	YLR043C	TRX1
YLR337C	VRP1	YGL087C	MMS2
YJL172W	CPS1	YMR230W	RPS10B
YDR443C	SSN2	YMR214W	SCJ1
YJR135C	MCM22	YLR254C	NDL1
YNL087W	TCB2	YGL003C	CDH1
YPL024W	RMI1	YKL114C	APN1
YGR268C	HUA1	YLR064W	YLR064W
YOR096W	RPS7A	YBL071W-A	KTI11
YOR290C	SNF2	YER122C	GLO3
YML115C	VAN1	YDR358W	GGA1
YOR069W	VPS5	YMR216C	SKY1
YJL176C	SWI3	YKR020W	VPS51
YBR200W	BEM1	YML001W	YPT7
YBL021C	HAP3	YPR046W	MCM16
YEL065W	SIT1	YEL053C	MAK10
YER120W	SCS2	YER162C	RAD4
YDR179C	CSN9	YOL117W	RRI2
YAL020C	ATS1	YPR040W	TIP41
YJL154C	VPS35	YJR060W	CBF1
YGR261C	APL6	YML001W	YPT7
YAL024C	LTE1	YJR035W	RAD26
YDL116W	NUP84	YDL047W	SIT4
YKL025C	PAN3	YER110C	KAP123

Predictions at threshold level 0.75 continued...			
Gene1 of predicted pair		Gene2 of predicted pair	
Systematic Name	Standard Name	Systematic Name	Standard Name
YLL039C	UBI4	YFR036W	CDC26
YKL048C	ELM1	YOR141C	ARP8
YLR337C	VRP1	YDR378C	LSM6
YPL105C	YPL105C	YAL002W	VPS8
YKL139W	CTK1	YLR386W	VAC14
YOR304W	ISW2	YML099C	ARG81
YBR200W	BEM1	YOR008C	SLG1
YLR119W	SRN2	YNL041C	COG6
YHR060W	VMA22	YLR371W	ROM2
YDR155C	CPR1	YDR310C	SUM1
YGR020C	VMA7	YKR030W	GMH1
YER044C	ERG28	YMR214W	SCJ1
YIL053W	RHR2	YJL095W	BCK1
YDR477W	SNF1	YGL025C	PGD1
YDR329C	PEX3	YNL214W	PEX17
YDR293C	SSD1	YOR106W	VAM3
YAL024C	LTE1	YIL034C	CAP2
YER118C	SHO1	YMR089C	YTA12
YIL076W	SEC28	YGR020C	VMA7
YLR441C	RPS1A	YBR171W	SEC66
YDR293C	SSD1	YNL206C	RTT106
YLR386W	VAC14	YOR127W	RGA1
YMR153W	NUP53	YMR152W	YIM1
YCR031C	RPS14A	YLR350W	ORM2
YBR036C	CSG2	YEL060C	PRB1
YPL024W	RMI1	YGR072W	UPF3
YPR171W	BSP1	YER118C	SHO1
YKR072C	SIS2	YLR234W	TOP3
YKL116C	PRR1	YPR036W	VMA13

Predictions at threshold level 0.75 continued...			
Gene1 of predicted pair		Gene2 of predicted pair	
Systematic Name	Standard Name	Systematic Name	Standard Name
YOR127W	RGA1	YJL187C	SWE1
YMR198W	CIK1	YER118C	SHO1
YIL076W	SEC28	YOR290C	SNF2
YGL060W	YBP2	YLR058C	SHM2
YBR173C	UMP1	YCR053W	THR4
YGL153W	PEX14	YCR077C	PAT1
YGL190C	CDC55	YER016W	BIM1
YJL127C	SPT10	YPR046W	MCM16
YDR261C	EXG2	YLR398C	SKI2
YLR386W	VAC14	YBR019C	GAL10
YDL116W	NUP84	YGL252C	RTG2
YDL083C	RPS16B	YJR084W	CSN12
YNL236W	SIN4	YPL248C	GAL4
YBR173C	UMP1	YJL172W	CPS1
YDL223C	HBT1	YDR358W	GGA1
YJR140C	HIR3	YLR182W	SWI6
YKL139W	CTK1	YDL005C	MED2
YNL199C	GCR2	YPL161C	BEM4
YDR244W	PEX5	YDR265W	PEX10
YAR018C	KIN3	YBL106C	SRO77
YDR137W	RGP1	YPL137C	GIP3
YAL024C	LTE1	YBL058W	SHP1
YML001W	YPT7	YMR311C	GLC8
YBL047C	EDE1	YDL155W	CLB3
YMR153W	NUP53	YOR358W	HAP5
YDR318W	MCM21	YNL225C	CNM67
YIL002C	INP51	YOR035C	SHE4
YIL053W	RHR2	YMR095C	SNO1
YER162C	RAD4	YBR019C	GAL10

Predictions at threshold level 0.75 continued...			
Gene1 of predicted pair		Gene2 of predicted pair	
Systematic Name	Standard Name	Systematic Name	Standard Name
YMR164C	MSS11	YER110C	KAP123
YBR108W	YBR108W	YHR081W	LRP1
YMR095C	SNO1	YJR056C	YJR056C
YHR073W	OSH3	YKR024C	DBP7
YNL199C	GCR2	YER049W	TPA1
YGL147C	RPL9A	YGL124C	MON1
YMR055C	BUB2	YKL048C	ELM1
YER162C	RAD4	YLR032W	RAD5
YGL087C	MMS2	YOR023C	AHC1
YEL053C	MAK10	YJL095W	BCK1
YDR174W	HMO1	YDR335W	MSN5
YDL161W	ENT1	YOR036W	PEP12
YPL042C	SSN3	YOR141C	ARP8
YNL135C	FPR1	YNL248C	RPA49
YNL069C	RPL16B	YJR033C	RAV1
YBR217W	ATG12	YOR066W	MSA1
YBL008W	HIR1	YLR234W	TOP3
YHL020C	OPI1	YEL043W	YEL043W
YLR370C	ARC18	YCR053W	THR4
YFR010W	UBP6	YGL003C	CDH1
YJL029C	VPS53	YMR231W	PEP5
YMR198W	CIK1	YBR107C	IML3
YDR080W	VPS41	YJL004C	SYS1
YDR129C	SAC6	YJL095W	BCK1
YLR254C	NDL1	YNL107W	YAF9
YGR261C	APL6	YLR360W	VPS38
YPL037C	EGD1	YNL238W	KEX2
YMR198W	CIK1	YDL226C	GCS1
YGR040W	KSS1	YDL159W	STE7

Predictions at threshold level 0.75 continued...			
Gene1 of predicted pair		Gene2 of predicted pair	
Systematic Name	Standard Name	Systematic Name	Standard Name
YCR081W	SRB8	YHR012W	VPS29
YKR059W	TIF1	YOR368W	RAD17
YLR090W	XDJ1	YGL212W	VAM7
YGR178C	PBP1	YNL085W	MKT1
YBL008W	HIR1	YBR215W	HPC2
YBR107C	IML3	YHR115C	DMA1
YIL076W	SEC28	YDR310C	SUM1
YDR383C	NKP1	YNL307C	MCK1
YNL032W	SIW14	YLR094C	GIS3
YML016C	PPZ1	YHR013C	ARD1
YHR178W	STB5	YCR031C	RPS14A
YHR134W	WSS1	YPL022W	RAD1
YDL155W	CLB3	YGL151W	NUT1
YPL115C	BEM3	YJL154C	VPS35
YOR140W	SFL1	YLR371W	ROM2
YOR043W	WHI2	YER139C	YER139C
YDR335W	MSN5	YKL213C	DOA1
YBL008W	HIR1	YPL129W	TAF14
YER144C	UBP5	YDR137W	RGP1
YDR202C	RAV2	YPL120W	VPS30
YLR447C	VMA6	YDR310C	SUM1
YGR270W	YTA7	YOR308C	SNU66
YNL069C	RPL16B	YCL037C	SRO9
YOR275C	RIM20	YKL161C	YKL161C
YJL036W	SNX4	YMR004W	MVP1
YDR105C	TMS1	YKR093W	PTR2
YGL147C	RPL9A	YML001W	YPT7
YPR018W	RLF2	YJR060W	CBF1
YGL223C	COG1	YOL018C	TLG2

Predictions at threshold level 0.75 continued...			
Gene1 of predicted pair		Gene2 of predicted pair	
Systematic Name	Standard Name	Systematic Name	Standard Name
YOR270C	VPH1	YPR036W	VMA13
YJR056C	YJR056C	YER122C	GLO3
YLR095C	IOC2	YGL124C	MON1
YPL042C	SSN3	YBR089C-A	NHP6B
YMR142C	RPL13B	YHR060W	VMA22
YPL220W	RPL1A	YNL147W	LSM7
YML016C	PPZ1	YGL253W	HXK2
YBR215W	HPC2	YPL018W	CTF19
YML115C	VAN1	YBR200W	BEM1
YBL047C	EDE1	YKR014C	YPT52
YLR360W	VPS38	YCR066W	RAD18
YBR239C	YBR239C	YLR144C	ACF2
YMR133W	REC114	YBR019C	GAL10
YNL044W	YIP3	YGL198W	YIP4
YCR066W	RAD18	YNR032W	PPG1
YLR095C	IOC2	YIL076W	SEC28
YDL020C	RPN4	YGR019W	UGA1
YBR217W	ATG12	YGL212W	VAM7
YPL253C	VIK1	YHR012W	VPS29
YGL087C	MMS2	YPL253C	VIK1
YJR135C	MCM22	YLR182W	SWI6
YMR253C	YMR253C	YKR030W	GMH1
YNL106C	INP52	YIR005W	IST3
YOR195W	SLK19	YLR102C	APC9
YOL090W	MSH2	YDR207C	UME6
YDR378C	LSM6	YGL153W	PEX14
YLR083C	EMP70	YJL053W	PEP8
YLR337C	VRP1	YFR040W	SAP155
YPL022W	RAD1	YNL248C	RPA49

Predictions at threshold level 0.75 continued...			
Gene1 of predicted pair		Gene2 of predicted pair	
Systematic Name	Standard Name	Systematic Name	Standard Name
YPR101W	SNT309	YNL147W	LSM7
YGL133W	ITC1	YHR115C	DMA1
YBR217W	ATG12	YBL078C	ATG8
YDR004W	RAD57	YNL307C	MCK1
YDR477W	SNF1	YBR200W	BEM1
YOL147C	PEX11	YPR119W	CLB2
YBL102W	SFT2	YDL006W	PTC1
YOR066W	MSA1	YLR093C	NYV1
YGR020C	VMA7	YPL234C	TFP3
YBL103C	RTG3	YKR048C	NAP1
YJR032W	CPR7	YDR207C	UME6
YBR194W	SOY1	YCR086W	CSM1
YDL059C	RAD59	YLR352W	YLR352W
YMR202W	ERG2	YOL018C	TLG2
YDR443C	SSN2	YIL076W	SEC28
YCR065W	HCM1	YNL206C	RTT106
YKL048C	ELM1	YDR155C	CPR1
YHR152W	SPO12	YIL008W	URM1
YBR240C	THI2	YKL160W	ELF1
YLR095C	IOC2	YMR026C	PEX12
YLR399C	BDF1	YBR009C	HHF1
YHL031C	GOS1	YOL018C	TLG2
YDR477W	SNF1	YDR137W	RGP1
YGL133W	ITC1	YBL047C	EDE1
YGR019W	UGA1	YGL151W	NUT1
YBR274W	CHK1	YCR031C	RPS14A
YEL009C	GCN4	YLR357W	RSC2
YJL183W	MNN11	YAL002W	VPS8
YGR238C	KEL2	YHR158C	KEL1

Predictions at threshold level 0.75 continued...			
Gene1 of predicted pair		Gene2 of predicted pair	
Systematic Name	Standard Name	Systematic Name	Standard Name
YPL161C	BEM4	YAR014C	BUD14
YEL065W	SIT1	YGR249W	MGA1
YEL053C	MAK10	YOL067C	RTG1
YBR239C	YBR239C	YKR030W	GMH1
YCL037C	SRO9	YJL187C	SWE1
YPR084W	YPR084W	YGL115W	SNF4
YLR150W	STM1	YPR046W	MCM16
YBR119W	MUD1	YKL074C	MUD2
YHR152W	SPO12	YDR363W-A	SEM1
YGL054C	ERV14	YMR054W	STV1
YMR172W	HOT1	YJR090C	GRR1
YLR292C	SEC72	YGL253W	HXK2
YKL048C	ELM1	YOR035C	SHE4
YMR198W	CIK1	YJL124C	LSM1
YBR171W	SEC66	YNL076W	MKS1
YGL174W	BUD13	YLR350W	ORM2
YBL102W	SFT2	YNR032W	PPG1
YNL248C	RPA49	YDL013W	SLX5
YPL024W	RMI1	YPL195W	APL5
YNL106C	INP52	YDL192W	ARF1
YLR018C	POM34	YOL126C	MDH2
YML115C	VAN1	YGL124C	MON1
YDR129C	SAC6	YKL126W	YPK1
YCL037C	SRO9	YHR077C	NMD2
YBR108W	YBR108W	YEL056W	HAT2
YML103C	NUP188	YLR410W	VIP1
YLR191W	PEX13	YML035C	AMD1
YMR109W	MYO5	YAR014C	BUD14
YMR133W	REC114	YER122C	GLO3

Predictions at threshold level 0.75 continued...			
Gene1 of predicted pair		Gene2 of predicted pair	
Systematic Name	Standard Name	Systematic Name	Standard Name
YOR290C	SNF2	YDR207C	UME6
YDR448W	ADA2	YFL031W	HAC1
YOR001W	RRP6	YDL002C	NHP10
YDR329C	PEX3	YMR026C	PEX12
YOL067C	RTG1	YOL108C	INO4
YGR261C	APL6	YPR051W	MAK3
YGR072W	UPF3	YDL077C	VAM6
YDR477W	SNF1	YHR200W	RPN10
YER110C	KAP123	YBR017C	KAP104
YKR001C	VPS1	YLR324W	PEX30
YNL054W	VAC7	YDR318W	MCM21
YDR289C	RTT103	YBR036C	CSG2
YDR245W	MNN10	YDR335W	MSN5
YBL058W	SHP1	YDL184C	RPL41A
YML016C	PPZ1	YMR167W	MLH1
YIL076W	SEC28	YDR108W	GSG1
YLR095C	IOC2	YKL139W	CTK1
YOR032C	HMS1	YNL289W	PCL1
YPL105C	YPL105C	YNL127W	FAR11
YOL051W	GAL11	YGL151W	NUT1
YGL190C	CDC55	YMR048W	CSM3
YNL106C	INP52	YGR188C	BUB1
YOR327C	SNC2	YEL060C	PRB1
YGR104C	SRB5	YGL025C	PGD1
YOL126C	MDH2	YMR199W	CLN1
YEL044W	IES6	YNL079C	TPM1
YDR477W	SNF1	YLR452C	SST2
YDL223C	HBT1	YMR214W	SCJ1
YDL002C	NHP10	YGL163C	RAD54

Predictions at threshold level 0.75 continued...			
Gene1 of predicted pair		Gene2 of predicted pair	
Systematic Name	Standard Name	Systematic Name	Standard Name
YLR386W	VAC14	YBL051C	PIN4
YLR292C	SEC72	YEL056W	HAT2
YPL129W	TAF14	YPL248C	GAL4
YBL016W	FUS3	YPL256C	CLN2
YNL215W	IES2	YPL001W	HAT1
YJR032W	CPR7	YJR084W	CSN12
YBR217W	ATG12	YLR452C	SST2
YLR386W	VAC14	YLR371W	ROM2
YGR129W	SYF2	YMR216C	SKY1
YFR034C	PHO4	YGR233C	PHO81
YJL006C	CTK2	YAR014C	BUD14
YBR019C	GAL10	YER139C	YER139C
YGR108W	CLB1	YBR171W	SEC66
YCR039C	MATALPHA2	YCR040W	MATALPHA1
YMR164C	MSS11	YPL246C	RBD2
YDL020C	RPN4	YJL095W	BCK1
YAL051W	OAF1	YDR289C	RTT103
YBR200W	BEM1	YKR048C	NAP1
YMR095C	SNO1	YDL083C	RPS16B
YIL156W	UBP7	YNR006W	VPS27
YPL161C	BEM4	YER049W	TPA1
YMR198W	CIK1	YDR363W-A	SEM1
YLR447C	VMA6	YPL161C	BEM4
YNR052C	POP2	YNL031C	HHT2
YPL001W	HAT1	YOR043W	WHI2
YBR217W	ATG12	YEL053C	MAK10
YBL058W	SHP1	YMR311C	GLC8
YNL206C	RTT106	YOL081W	IRA2
YDR245W	MNN10	YPL050C	MNN9

Predictions at threshold level 0.75 continued...			
Gene1 of predicted pair		Gene2 of predicted pair	
Systematic Name	Standard Name	Systematic Name	Standard Name
YBL058W	SHP1	YJL172W	CPS1
YPR120C	CLB5	YGL163C	RAD54
YLR135W	SLX4	YJL187C	SWE1
YDR363W-A	SEM1	YLR429W	CRN1
YBL008W	HIR1	YNL215W	IES2
YNL248C	RPA49	YJR060W	CBF1
YDR484W	VPS52	YDR310C	SUM1
YPL115C	BEM3	YIL034C	CAP2
YPL024W	RMI1	YMR153W	NUP53
YDL020C	RPN4	YLR381W	CTF3
YPL037C	EGD1	YPL137C	GIP3
YGL153W	PEX14	YKL041W	VPS24
YPL195W	APL5	YNL127W	FAR11
YGL115W	SNF4	YBR036C	CSG2
YPL024W	RMI1	YMR142C	RPL13B
YLR360W	VPS38	YLR371W	ROM2
YNL206C	RTT106	YKR057W	RPS21A
YOR270C	VPH1	YJR032W	CPR7
YDL223C	HBT1	YER162C	RAD4
YBR200W	BEM1	YNL238W	KEX2
YNL325C	FIG4	YDL192W	ARF1
YCL067C	HMLALPHA2	YER167W	BCK2
YDR443C	SSN2	YDR448W	ADA2
YMR095C	SNO1	YMR133W	REC114
YNL325C	FIG4	YHR060W	VMA22
YDR253C	MET32	YLR254C	NDL1
YNL053W	MSG5	YPL240C	HSP82
YOR035C	SHE4	YGL124C	MON1
YML016C	PPZ1	YBR119W	MUD1

Predictions at threshold level 0.75 continued...			
Gene1 of predicted pair		Gene2 of predicted pair	
Systematic Name	Standard Name	Systematic Name	Standard Name
YMR224C	MRE11	YPL129W	TAF14
YFR010W	UBP6	YDR207C	UME6
YEL065W	SIT1	YIL076W	SEC28
YJL117W	PHO86	YLR320W	MMS22
YNL106C	INP52	YDR358W	GGA1
YKL010C	UFD4	YMR224C	MRE11
YDR320C	SWA2	YNL051W	COG5
YFL023W	BUD27	YER040W	GLN3
YFL013C	IES1	YER122C	GLO3
YGL033W	HOP2	YNL087W	TCB2
YBR057C	MUM2	YDR245W	MNN10
YBR278W	DPB3	YCR086W	CSM1
YJR032W	CPR7	YLR370C	ARC18
YJL095W	BCK1	YDL047W	SIT4
YIL076W	SEC28	YBR030W	YBR030W
YMR095C	SNO1	YBR200W	BEM1
YMR224C	MRE11	YNL032W	SIW14
YBR031W	RPL4A	YGR100W	MDR1
YGL078C	DBP3	YMR142C	RPL13B
YDR335W	MSN5	YDR254W	CHL4
YNL058C	YNL058C	YHR039C-A	VMA10
YOR069W	VPS5	YNL297C	MON2
YBR108W	YBR108W	YBR019C	GAL10
YCL037C	SRO9	YMR038C	CCS1
YPL234C	TFP3	YKR030W	GMH1
YKR099W	BAS1	YML001W	YPT7
YBR228W	SLX1	YJL187C	SWE1
YHR149C	SKG6	YML109W	ZDS2
YBR107C	IML3	YPL161C	BEM4

Predictions at threshold level 0.75 continued...			
Gene1 of predicted pair		Gene2 of predicted pair	
Systematic Name	Standard Name	Systematic Name	Standard Name
YLR083C	EMP70	YOR327C	SNC2
YER110C	KAP123	YIL008W	URM1
YCR088W	ABP1	YKR020W	VPS51
YMR224C	MRE11	YKL041W	VPS24
YIL159W	BNR1	YGL212W	VAM7
YDR496C	PUF6	YER002W	NOP16
YIL159W	BNR1	YBL103C	RTG3
YGL163C	RAD54	YML112W	CTK3
YIL148W	RPL40A	YKL068W	NUP100
YKR072C	SIS2	YGR081C	SLX9
YCR053W	THR4	YMR214W	SCJ1
YNL107W	YAF9	YLR350W	ORM2
YMR224C	MRE11	YPL213W	LEA1
YDL002C	NHP10	YOR038C	HIR2
YMR016C	SOK2	YLR094C	GIS3
YPR189W	SKI3	YNR052C	POP2
YIL076W	SEC28	YMR038C	CCS1
YKL025C	PAN3	YMR127C	SAS2
YDR202C	RAV2	YBR127C	VMA2
YOR337W	TEA1	YOR270C	VPH1
YDR289C	RTT103	YGL148W	ARO2
YDR443C	SSN2	YER144C	UBP5
YFR031C-A	RPL2A	YGR261C	APL6
YHR081W	LRP1	YJR084W	CSN12
YBR217W	ATG12	YLR371W	ROM2
YJR060W	CBF1	YML001W	YPT7
YDL116W	NUP84	YKR095W	MLP1
YPR036W	VMA13	YML028W	TSA1
YMR216C	SKY1	YNL051W	COG5

Predictions at threshold level 0.75 continued...			
Gene1 of predicted pair		Gene2 of predicted pair	
Systematic Name	Standard Name	Systematic Name	Standard Name
YBL008W	HIR1	YDL226C	GCS1
YDR174W	HMO1	YNL225C	CNM67
YCR020C-A	MAK31	YDR207C	UME6
YKR001C	VPS1	YEL056W	HAT2
YKL116C	PRR1	YDR477W	SNF1
YIL034C	CAP2	YCR086W	CSM1
YGR252W	GCN5	YFL031W	HAC1
YML115C	VAN1	YPL161C	BEM4
YML103C	NUP188	YBL079W	NUP170
YJL183W	MNN11	YDL077C	VAM6
YPL024W	RMI1	YBR200W	BEM1
YBL008W	HIR1	YJR140C	HIR3
YBR273C	UBX7	YKL213C	DOA1
YPR046W	MCM16	YMR311C	GLC8
YFR031C-A	RPL2A	YDR477W	SNF1
YAL024C	LTE1	YOR127W	RGA1
YLR090W	XDJ1	YPL195W	APL5
YPL024W	RMI1	YMR214W	SCJ1
YGL163C	RAD54	YDR318W	MCM21
YOL111C	MDY2	YBR280C	SAF1
YDL185W	TFP1	YDR289C	RTT103
YML095C	RAD10	YDR207C	UME6
YGR270W	YTA7	YJL183W	MNN11
YJL154C	VPS35	YPR051W	MAK3
YLR399C	BDF1	YLR357W	RSC2
YOR127W	RGA1	YPL161C	BEM4
YPL253C	VIK1	YIL008W	URM1
YHR134W	WSS1	YBL051C	PIN4
YEL053C	MAK10	YOL081W	IRA2

Predictions at threshold level 0.75 continued...			
Gene1 of predicted pair		Gene2 of predicted pair	
Systematic Name	Standard Name	Systematic Name	Standard Name
YFR031C-A	RPL2A	YCL001W	RER1
YDL077C	VAM6	YER122C	GLO3
YDL020C	RPN4	YBR171W	SEC66
YBL103C	RTG3	YJR056C	YJR056C
YPL180W	TCO89	YER118C	SHO1
YOR112W	CEX1	YPL161C	BEM4
YHL031C	GOS1	YGR105W	VMA21
YMR284W	YKU70	YML028W	TSA1
YGR249W	MGA1	YOL121C	RPS19A
YPR036W	VMA13	YEL027W	CUP5
YKL101W	HSL1	YCR031C	RPS14A
YGR104C	SRB5	YBR112C	CYC8
YBR215W	HPC2	YDR289C	RTT103
YOR332W	VMA4	YEL027W	CUP5
YOR043W	WHI2	YMR311C	GLC8
YPL049C	DIG1	YBL016W	FUS3
YJL176C	SWI3	YLR061W	RPL22A
YPL024W	RMI1	YJR035W	RAD26
YER139C	YER139C	YHR167W	THP2
YGL212W	VAM7	YGL124C	MON1
YKL110C	KTI12	YOL081W	IRA2
YFL023W	BUD27	YDR137W	RGP1
YKL007W	CAP1	YHR012W	VPS29
YDR137W	RGP1	YMR133W	REC114
YDR383C	NKP1	YLR410W	VIP1
YMR042W	ARG80	YJR094C	IME1
YBR171W	SEC66	YGR054W	YGR054W
YNL051W	COG5	YDR310C	SUM1
YFR040W	SAP155	YLR370C	ARC18

Predictions at threshold level 0.75 continued...			
Gene1 of predicted pair		Gene2 of predicted pair	
Systematic Name	Standard Name	Systematic Name	Standard Name
YJL058C	BIT61	YDL226C	GCS1
YHR156C	LIN1	YDR207C	UME6
YML059C	NTE1	YJR025C	BNA1
YER068W	MOT2	YOR064C	YNG1
YOR109W	INP53	YPL195W	APL5
YCR020C-A	MAK31	YDR310C	SUM1
YGL200C	EMP24	YDL006W	PTC1
YLR234W	TOP3	YBL019W	APN2
YGL223C	COG1	YOL112W	MSB4
YFR031C-A	RPL2A	YOL108C	INO4
YLR386W	VAC14	YHR111W	UBA4
YLR315W	NKP2	YCR086W	CSM1
YDR320C	SWA2	YJR060W	CBF1
YER116C	SLX8	YKL126W	YPK1
YDL192W	ARF1	YKL119C	VPH2
YOR043W	WHI2	YGL148W	ARO2
YGL212W	VAM7	YMR054W	STV1
YDR174W	HMO1	YIR005W	IST3
YLR381W	CTF3	YHL002W	HSE1
YKR020W	VPS51	YBL101C	ECM21
YER016W	BIM1	YER105C	NUP157
YDL192W	ARF1	YOR036W	PEP12
YDR443C	SSN2	YJL141C	YAK1
YPR046W	MCM16	YHR114W	BZZ1
YEL043W	YEL043W	YPL234C	TFP3
YPR052C	NHP6A	YNR052C	POP2
YPR084W	YPR084W	YNL107W	YAF9
YOR001W	RRP6	YJR060W	CBF1
YEL065W	SIT1	YMR152W	YIM1

Predictions at threshold level 0.75 continued...			
Gene1 of predicted pair		Gene2 of predicted pair	
Systematic Name	Standard Name	Systematic Name	Standard Name
YPR051W	MAK3	YJR040W	GEF1
YML115C	VAN1	YDL006W	PTC1
YHR156C	LIN1	YOR270C	VPH1
YKR059W	TIF1	YMR167W	MLH1
YOR106W	VAM3	YLR425W	TUS1
YDL002C	NHP10	YDR335W	MSN5
YPR040W	TIP41	YKR020W	VPS51
YEL065W	SIT1	YKL126W	YPK1
YHR119W	SET1	YLR418C	CDC73
YLR325C	RPL38	YHR010W	RPL27A
YDR490C	PKH1	YMR104C	YPK2
YLR360W	VPS38	YHL031C	GOS1
YML115C	VAN1	YDR207C	UME6
YNL206C	RTT106	YDL013W	SLX5
YKR001C	VPS1	YNL051W	COG5
YDR383C	NKP1	YGR270W	YTA7
YER120W	SCS2	YDR207C	UME6
YCL037C	SRO9	YLR309C	IMH1
YDL077C	VAM6	YMR214W	SCJ1
YOR327C	SNC2	YJR084W	CSN12
YJR058C	APS2	YJR005W	APL1
YNL051W	COG5	YAR014C	BUD14
YNL325C	FIG4	YOL111C	MDY2
YEL053C	MAK10	YGL124C	MON1
YDR443C	SSN2	YBR280C	SAF1
YKL007W	CAP1	YGL252C	RTG2
YLR079W	SIC1	YOR141C	ARP8
YDR076W	RAD55	YGR188C	BUB1
YOL111C	MDY2	YML071C	COG8

Predictions at threshold level 0.75 continued...			
Gene1 of predicted pair		Gene2 of predicted pair	
Systematic Name	Standard Name	Systematic Name	Standard Name
YCR066W	RAD18	YPR032W	SRO7
YMR216C	SKY1	YHR060W	VMA22
YJR024C	MDE1	YPL178W	CBC2
YPR120C	CLB5	YMR038C	CCS1
YER144C	UBP5	YHR081W	LRP1
YLR180W	SAM1	YOL081W	IRA2
YHL002W	HSE1	YNL107W	YAF9
YDR225W	HTA1	YDL056W	MBP1
YMR216C	SKY1	YFL033C	RIM15
YKL110C	KTI12	YLR410W	VIP1
YJL089W	SIP4	YDR477W	SNF1
YBL008W	HIR1	YPL042C	SSN3
YIL076W	SEC28	YBR108W	YBR108W
YMR199W	CLN1	YBR171W	SEC66
YDL184C	RPL41A	YGL003C	CDH1
YLR386W	VAC14	YOR106W	VAM3
YDR293C	SSD1	YDR320C	SWA2
YHR012W	VPS29	YHL002W	HSE1
YLR234W	TOP3	YDL185W	TFP1
YBL021C	HAP3	YJR032W	CPR7
YLR090W	XDJ1	YKL116C	PRR1
YGL115W	SNF4	YJL183W	MNN11
YGR072W	UPF3	YER120W	SCS2
YDL083C	RPS16B	YJL095W	BCK1
YGL115W	SNF4	YDR310C	SUM1
YLR410W	VIP1	YML112W	CTK3
YDR092W	UBC13	YCR066W	RAD18
YOR033C	EXO1	YKR072C	SIS2
YIL036W	CST6	YKL048C	ELM1

Predictions at threshold level 0.75 continued...			
Gene1 of predicted pair		Gene2 of predicted pair	
Systematic Name	Standard Name	Systematic Name	Standard Name
YJL036W	SNX4	YOR069W	VPS5
YPR120C	CLB5	YML112W	CTK3
YLR452C	SST2	YGL003C	CDH1
YHR013C	ARD1	YHR200W	RPN10
YOR001W	RRP6	YER161C	SPT2
YBR041W	FAT1	YOR059C	YOR059C
YGL147C	RPL9A	YNL076W	MKS1
YMR199W	CLN1	YPL246C	RBD2
YOL082W	ATG19	YGL156W	AMS1
YOR127W	RGA1	YJL004C	SYS1
YDR080W	VPS41	YEL051W	VMA8
YMR153W	NUP53	YPL178W	CBC2
YDR004W	RAD57	YBR107C	IML3
YBR280C	SAF1	YDR207C	UME6
YLL007C	YLL007C	YDR183W	PLP1
YPL024W	RMI1	YGL175C	SAE2
YLR079W	SIC1	YER115C	SPR6
YNL238W	KEX2	YGR105W	VMA21
YHR152W	SPO12	YPL256C	CLN2
YNL093W	YPT53	YPR119W	CLB2
YPR051W	MAK3	YDL077C	VAM6
YAL024C	LTE1	YDR379W	RGA2
YLR396C	VPS33	YPR028W	YOP1
YNL106C	INP52	YGL252C	RTG2
YNR072W	HXT17	YGL115W	SNF4
YPR018W	RLF2	YML115C	VAN1
YLR288C	MEC3	YHR152W	SPO12
YPL137C	GIP3	YPR046W	MCM16
YIL076W	SEC28	YLR386W	VAC14

Predictions at threshold level 0.75 continued...			
Gene1 of predicted pair		Gene2 of predicted pair	
Systematic Name	Standard Name	Systematic Name	Standard Name
YMR202W	ERG2	YNL289W	PCL1
YOR033C	EXO1	YPL022W	RAD1
YLR079W	SIC1	YNL307C	MCK1
YOR144C	ELG1	YHR098C	SFB3
YMR273C	ZDS1	YCR086W	CSM1
YNR052C	POP2	YNL116W	DMA2
YPL024W	RMI1	YNL325C	FIG4
YLR360W	VPS38	YJR040W	GEF1
YOR127W	RGA1	YML038C	YMD8
YMR199W	CLN1	YOR043W	WHI2
YAL024C	LTE1	YKL110C	KTI12
YMR095C	SNO1	YEL053C	MAK10
YAL024C	LTE1	YDR477W	SNF1
YDR378C	LSM6	YKL160W	ELF1
YLR006C	SSK1	YPL256C	CLN2
YJR032W	CPR7	YMR214W	SCJ1
YLR018C	POM34	YMR199W	CLN1
YPR018W	RLF2	YNL264C	PDR17
YIL053W	RHR2	YBR217W	ATG12
YKL139W	CTK1	YMR311C	GLC8
YDL083C	RPS16B	YNL238W	KEX2
YBL101C	ECM21	YLR425W	TUS1
YDR176W	NGG1	YLR399C	BDF1
YFR030W	MET10	YPR046W	MCM16
YJR084W	CSN12	YLR370C	ARC18
YFR021W	ATG18	YDL149W	ATG9
YMR026C	PEX12	YNL058C	YNL058C
YBR200W	BEM1	YOR358W	HAP5
YDL020C	RPN4	YMR179W	SPT21

Predictions at threshold level 0.75 continued...			
Gene1 of predicted pair		Gene2 of predicted pair	
Systematic Name	Standard Name	Systematic Name	Standard Name
YDR358W	GGA1	YMR230W	RPS10B
YGL115W	SNF4	YDL077C	VAM6
YLR182W	SWI6	YAL002W	VPS8
YLR093C	NYV1	YDR310C	SUM1
YIL034C	CAP2	YJL190C	RPS22A
YGR081C	SLX9	YMR199W	CLN1
YCR088W	ABP1	YDL226C	GCS1
YDR363W-A	SEM1	YOL054W	PSH1
YNL218W	MGS1	YHR077C	NMD2
YGL178W	MPT5	YER167W	BCK2
YMR284W	YKU70	YKL114C	APN1
YIL160C	POT1	YNL214W	PEX17
YKR014C	YPT52	YJL095W	BCK1
YDL113C	ATG20	YPR049C	ATG11
YGL161C	YIP5	YOR308C	SNU66
YPL042C	SSN3	YDR254W	CHL4
YLR061W	RPL22A	YKR020W	VPS51
YDR484W	VPS52	YER044C	ERG28
YER144C	UBP5	YMR311C	GLC8
YBL025W	RRN10	YLR180W	SAM1
YGL174W	BUD13	YHR167W	THP2
YNL297C	MON2	YNL107W	YAF9
YJL110C	GZF3	YKR034W	DAL80
YIL156W	UBP7	YHL002W	HSE1
YPL105C	YPL105C	YPL161C	BEM4
YLR386W	VAC14	YDL155W	CLB3
YLR288C	MEC3	YGL175C	SAE2
YFR031C-A	RPL2A	YMR224C	MRE11
YEL065W	SIT1	YDR261C	EXG2

Predictions at threshold level 0.75 continued...			
Gene1 of predicted pair		Gene2 of predicted pair	
Systematic Name	Standard Name	Systematic Name	Standard Name
YLR360W	VPS38	YGL124C	MON1
YER116C	SLX8	YNL016W	PUB1
YDR443C	SSN2	YNL041C	COG6
YKR001C	VPS1	YDL077C	VAM6
YDR004W	RAD57	YDL059C	RAD59
YDR293C	SSD1	YHR039C-A	VMA10
YFR036W	CDC26	YBL072C	RPS8A
YDL056W	MBP1	YPR120C	CLB5
YGR241C	YAP1802	YJR125C	ENT3
YOL121C	RPS19A	YLR180W	SAM1
YDL184C	RPL41A	YOR043W	WHI2
YBL079W	NUP170	YML028W	TSA1
YDR484W	VPS52	YJL183W	MNN11
YLR192C	HCR1	YBR181C	RPS6B
YDR335W	MSN5	YJL006C	CTK2
YMR202W	ERG2	YDR309C	GIC2
YDR359C	EAF1	YGL190C	CDC55
YNL238W	KEX2	YKR030W	GMH1
YBL058W	SHP1	YOL111C	MDY2
YMR116C	ASC1	YER116C	SLX8
YDR320C	SWA2	YJR032W	CPR7
YBL088C	TEL1	YJR021C	REC107
YGL161C	YIP5	YHR200W	RPN10
YMR095C	SNO1	YBR030W	YBR030W
YIL076W	SEC28	YLR398C	SKI2
YBR200W	BEM1	YBL101C	ECM21
YIL159W	BNR1	YNL289W	PCL1
YDR484W	VPS52	YMR311C	GLC8
YPL090C	RPS6A	YGL246C	RAI1

Predictions at threshold level 0.75 continued...			
Gene1 of predicted pair		Gene2 of predicted pair	
Systematic Name	Standard Name	Systematic Name	Standard Name
YML102W	CAC2	YHR010W	RPL27A
YKL025C	PAN3	YBR114W	RAD16
YPR120C	CLB5	YLR032W	RAD5
YPL022W	RAD1	YBR073W	RDH54
YDR378C	LSM6	YIL103W	DPH1
YCR063W	BUD31	YIR005W	IST3
YJR032W	CPR7	YCR086W	CSM1
YOR270C	VPH1	YBL103C	RTG3
YNL032W	SIW14	YDR137W	RGP1
YLR090W	XDJ1	YFL023W	BUD27
YMR224C	MRE11	YDR318W	MCM21
YNL243W	SLA2	YPL259C	APM1
YPL057C	SUR1	YPR120C	CLB5
YMR172W	HOT1	YDR254W	CHL4
YCL037C	SRO9	YCL001W	RER1
YOL148C	SPT20	YLR182W	SWI6
YLR381W	CTF3	YGL253W	HXK2
YOR213C	SAS5	YML097C	VPS9
YGR019W	UGA1	YLR233C	EST1
YLR234W	TOP3	YGL237C	HAP2
YOR127W	RGA1	YDR183W	PLP1
YDR440W	DOT1	YJL183W	MNN11
YMR153W	NUP53	YPL161C	BEM4
YLR381W	CTF3	YPR120C	CLB5
YNL264C	PDR17	YDL006W	PTC1
YGR019W	UGA1	YOR132W	VPS17
YDL002C	NHP10	YCR077C	PAT1
YDR108W	GSG1	YGL223C	COG1
YOR066W	MSA1	YMR152W	YIM1

Predictions at threshold level 0.75 continued...			
Gene1 of predicted pair		Gene2 of predicted pair	
Systematic Name	Standard Name	Systematic Name	Standard Name
YLR447C	VMA6	YJL190C	RPS22A
YHR156C	LIN1	YGL003C	CDH1
YML016C	PPZ1	YCR086W	CSM1
YBL051C	PIN4	YBR171W	SEC66
YLR447C	VMA6	YGL151W	NUT1
YKL048C	ELM1	YLR180W	SAM1
YKL139W	CTK1	YIL076W	SEC28
YHR134W	WSS1	YGL031C	RPL24A
YDR383C	NKP1	YKL110C	KTI12
YML001W	YPT7	YPR028W	YOP1
YHR200W	RPN10	YGR188C	BUB1
YGL078C	DBP3	YNR072W	HXT17
YMR026C	PEX12	YLR192C	HCR1
YDR293C	SSD1	YGR270W	YTA7
YGL115W	SNF4	YMR152W	YIM1
YOR358W	HAP5	YKL048C	ELM1
YJR074W	MOG1	YPR119W	CLB2
YDL116W	NUP84	YLR018C	POM34
YJR060W	CBF1	YGL252C	RTG2
YPR046W	MCM16	YJL190C	RPS22A
YGR081C	SLX9	YHR010W	RPL27A
YJR033C	RAV1	YOL108C	INO4
YEL065W	SIT1	YGL212W	VAM7
YGL115W	SNF4	YER120W	SCS2
YLR292C	SEC72	YBL051C	PIN4
YKL139W	CTK1	YNL032W	SIW14
YDL155W	CLB3	YJL006C	CTK2
YDR379W	RGA2	YDR363W-A	SEM1
YNL058C	YNL058C	YLR094C	GIS3

Predictions at threshold level 0.75 continued...			
Gene1 of predicted pair		Gene2 of predicted pair	
Systematic Name	Standard Name	Systematic Name	Standard Name
YLR371W	ROM2	YMR311C	GLC8
YNL215W	IES2	YDR439W	LRS4
YOR270C	VPH1	YDL077C	VAM6
YMR127C	SAS2	YPR046W	MCM16
YNL069C	RPL16B	YNL238W	KEX2
YJR060W	CBF1	YNL225C	CNM67
YMR224C	MRE11	YLR079W	SIC1
YIL076W	SEC28	YAR014C	BUD14
YOL051W	GAL11	YJL140W	RPB4
YBR126C	TPS1	YLR386W	VAC14
YLR371W	ROM2	YPR030W	CSR2
YLR095C	IOC2	YFL023W	BUD27
YGL212W	VAM7	YEL060C	PRB1
YDR080W	VPS41	YEL043W	YEL043W
YCR034W	FEN1	YBR274W	CHK1
YGL003C	CDH1	YER040W	GLN3
YBR200W	BEM1	YHL027W	RIM101
YNL238W	KEX2	YLR370C	ARC18
YGL115W	SNF4	YGL124C	MON1
YNL087W	TCB2	YMR311C	GLC8
YNL032W	SIW14	YOL111C	MDY2
YLL039C	UBI4	YCR077C	PAT1
YJL176C	SWI3	YLR135W	SLX4
YJL124C	LSM1	YDR137W	RGP1
YGL212W	VAM7	YER044C	ERG28
YIL159W	BNR1	YLR320W	MMS22
YGL212W	VAM7	YKL048C	ELM1
YDR378C	LSM6	YOL054W	PSH1
YLR288C	MEC3	YLR350W	ORM2

Predictions at threshold level 0.75 continued...			
Gene1 of predicted pair		Gene2 of predicted pair	
Systematic Name	Standard Name	Systematic Name	Standard Name
YHR200W	RPN10	YKL160W	ELF1
YNL325C	FIG4	YPR046W	MCM16
YOL111C	MDY2	YGL003C	CDH1
YGR261C	APL6	YML038C	YMD8
YNR052C	POP2	YOL054W	PSH1
YOR127W	RGA1	YIL008W	URM1
YBR215W	HPC2	YOR308C	SNU66
YDR225W	HTA1	YDR076W	RAD55
YBL102W	SFT2	YMR054W	STV1
YJL154C	VPS35	YGL124C	MON1
YKL116C	PRR1	YLR093C	NYV1
YFR031C-A	RPL2A	YBR280C	SAF1
YPR131C	NAT3	YPL022W	RAD1
YMR116C	ASC1	YHR013C	ARD1
YGR241C	YAP1802	YHR161C	YAP1801
YCR066W	RAD18	YGR105W	VMA21
YNL206C	RTT106	YGR184C	UBR1
YML016C	PPZ1	YJL190C	RPS22A
YDR108W	GSG1	YOL018C	TLG2
YKR035W-A	DID2	YDR310C	SUM1
YBR200W	BEM1	YHR012W	VPS29
YGL223C	COG1	YCL001W	RER1
YDR254W	CHL4	YNL225C	CNM67
YGL190C	CDC55	YDL194W	SNF3
YDR174W	HMO1	YPL018W	CTF19
YPL024W	RMI1	YCR077C	PAT1
YHR134W	WSS1	YMR116C	ASC1
YIL076W	SEC28	YLR315W	NKP2
YLR079W	SIC1	YCR024C-A	PMP1

Predictions at threshold level 0.75 continued...			
Gene1 of predicted pair		Gene2 of predicted pair	
Systematic Name	Standard Name	Systematic Name	Standard Name
YPL161C	BEM4	YNL041C	COG6
YPR084W	YPR084W	YCL001W	RER1
YOR069W	VPS5	YNL238W	KEX2
YER044C-A	MEI4	YJR021C	REC107
YML095C	RAD10	YJL183W	MNN11
YHR134W	WSS1	YPL253C	VIK1
YGL087C	MMS2	YBR057C	MUM2
YAL024C	LTE1	YCR077C	PAT1
YOR071C	THI71	YKR093W	PTR2
YLR386W	VAC14	YJL187C	SWE1
YOR243C	PUS7	YHR077C	NMD2
YGL212W	VAM7	YDL006W	PTC1
YBL058W	SHP1	YBL025W	RRN10
YNL215W	IES2	YBR107C	IML3
YMR026C	PEX12	YMR311C	GLC8
YKR001C	VPS1	YOR275C	RIM20
YML016C	PPZ1	YDL006W	PTC1
YKL048C	ELM1	YDL155W	CLB3
YKL110C	KTI12	YIL103W	DPH1
YDL184C	RPL41A	YPL057C	SUR1
YDR309C	GIC2	YCR077C	PAT1
YGL153W	PEX14	YJL183W	MNN11
YGR239C	PEX21	YDR244W	PEX5
YLR090W	XDJ1	YOR327C	SNC2
YNL094W	APP1	YPL246C	RBD2
YPR018W	RLF2	YMR224C	MRE11
YLR320W	MMS22	YLR398C	SKI2
YGL078C	DBP3	YMR152W	YIM1
YBR065C	ECM2	YJR050W	ISY1

Predictions at threshold level 0.75 continued...			
Gene1 of predicted pair		Gene2 of predicted pair	
Systematic Name	Standard Name	Systematic Name	Standard Name
YLR192C	HCR1	YHR156C	LIN1
YER110C	KAP123	YMR055C	BUB2
YJL172W	CPS1	YML071C	COG8
YML115C	VAN1	YDL223C	HBT1
YBR200W	BEM1	YDR318W	MCM21
YLR386W	VAC14	YLR093C	NYV1
YLR095C	IOC2	YMR153W	NUP53
YJR135C	MCM22	YGL124C	MON1
YMR198W	CIK1	YGL163C	RAD54
YNL215W	IES2	YBR194W	SOY1
YNL325C	FIG4	YHR200W	RPN10
YJL053W	PEP8	YCR066W	RAD18
YDR146C	SWI5	YJL124C	LSM1
YPR120C	CLB5	YHR039C-A	VMA10
YJL060W	BNA3	YPL106C	SSE1
YLR192C	HCR1	YBR108W	YBR108W
YGR249W	MGA1	YDL077C	VAM6
YER162C	RAD4	YPR046W	MCM16
YFL023W	BUD27	YBR019C	GAL10
YOR069W	VPS5	YOR036W	PEP12
YER120W	SCS2	YJR033C	RAV1
YBR171W	SEC66	YER040W	GLN3
YOR106W	VAM3	YER162C	RAD4
YKL048C	ELM1	YPR119W	CLB2
YML109W	ZDS2	YBR010W	HHT1
YNL041C	COG6	YDR318W	MCM21
YDR318W	MCM21	YKL114C	APN1
YBL058W	SHP1	YJR084W	CSN12
YJR032W	CPR7	YCR031C	RPS14A

Predictions at threshold level 0.75 continued...			
Gene1 of predicted pair		Gene2 of predicted pair	
Systematic Name	Standard Name	Systematic Name	Standard Name
YMR055C	BUB2	YPL018W	CTF19
YLR360W	VPS38	YOR327C	SNC2
YKL032C	IXR1	YOR275C	RIM20
YBR290W	BSD2	YML057W	CMP2
YNR072W	HXT17	YHR039C-A	VMA10
YDL223C	HBT1	YGL124C	MON1
YDR254W	CHL4	YGL253W	HXK2
YMR284W	YKU70	YEL056W	HAT2
YBR041W	FAT1	YPR119W	CLB2
YPL001W	HAT1	YOR038C	HIR2
YOR191W	RIS1	YFR010W	UBP6
YMR095C	SNO1	YBR108W	YBR108W
YFL013C	IES1	YNL199C	GCR2
YPL018W	CTF19	YGR188C	BUB1
YER001W	MNN1	YBL047C	EDE1
YER139C	YER139C	YDL077C	VAM6
YLR447C	VMA6	YEL053C	MAK10
YNL106C	INP52	YDR289C	RTT103
YLR090W	XDJ1	YML001W	YPT7
YNL079C	TPM1	YBL101C	ECM21
YDR146C	SWI5	YJL095W	BCK1
YOR023C	AHC1	YHR114W	BZZ1
YNL236W	SIN4	YDL047W	SIT4
YNL032W	SIW14	YDR484W	VPS52
YNL054W	VAC7	YFR030W	MET10
YDR174W	HMO1	YNL107W	YAF9
YER162C	RAD4	YNL107W	YAF9
YLL007C	YLL007C	YOR358W	HAP5
YGL078C	DBP3	YER162C	RAD4

Predictions at threshold level 0.75 continued...			
Gene1 of predicted pair		Gene2 of predicted pair	
Systematic Name	Standard Name	Systematic Name	Standard Name
YOR290C	SNF2	YOR035C	SHE4
YLR370C	ARC18	YBR280C	SAF1
YGL060W	YBP2	YDR200C	VPS64
YBR173C	UMP1	YOR195W	SLK19
YLR192C	HCR1	YML097C	VPS9
YJL154C	VPS35	YHR111W	UBA4
YJR140C	HIR3	YIR005W	IST3
YGR104C	SRB5	YGR089W	NNF2
YEL065W	SIT1	YGL115W	SNF4
YHL031C	GOS1	YML012W	ERV25
YGL135W	RPL1B	YGL200C	EMP24
YOR127W	RGA1	YOR023C	AHC1
YGR108W	CLB1	YDR183W	PLP1
YEL065W	SIT1	YLR371W	ROM2
YBR019C	GAL10	YDL077C	VAM6
YNL032W	SIW14	YJL172W	CPS1
YMR016C	SOK2	YCR053W	THR4
YOR312C	RPL20B	YGR135W	PRE9
YFR010W	UBP6	YOR001W	RRP6
YBR173C	UMP1	YOR066W	MSA1
YDR484W	VPS52	YKR001C	VPS1
YBR171W	SEC66	YEL060C	PRB1
YGR072W	UPF3	YML001W	YPT7
YDR363W-A	SEM1	YBL047C	EDE1
YFR010W	UBP6	YJR035W	RAD26
YJR024C	MDE1	YML057W	CMP2
YGR261C	APL6	YNL041C	COG6
YDR080W	VPS41	YNL238W	KEX2
YKL041W	VPS24	YOL081W	IRA2

Predictions at threshold level 0.75 continued...			
Gene1 of predicted pair		Gene2 of predicted pair	
Systematic Name	Standard Name	Systematic Name	Standard Name
YDR507C	GIN4	YJR090C	GRR1
YIL156W	UBP7	YMR273C	ZDS1
YPL024W	RMI1	YER161C	SPT2
YLR090W	XDJ1	YLR441C	RPS1A
YBR108W	YBR108W	YDR137W	RGP1
YPR046W	MCM16	YNL107W	YAF9
YNL087W	TCB2	YDL083C	RPS16B
YIL036W	CST6	YDR439W	LRS4
YGL153W	PEX14	YHL002W	HSE1
YGL078C	DBP3	YOR327C	SNC2
YLR292C	SEC72	YHR178W	STB5
YBR108W	YBR108W	YBR114W	RAD16
YML062C	MFT1	YCR077C	PAT1
YOR290C	SNF2	YDR310C	SUM1
YPL249C-A	RPL36B	YDR388W	RVS167
YOR127W	RGA1	YBL051C	PIN4
YHR012W	VPS29	YDL155W	CLB3
YLR371W	ROM2	YDR310C	SUM1
YJL176C	SWI3	YDR073W	SNF11
YMR127C	SAS2	YMR109W	MYO5
YBR030W	YBR030W	YDL223C	HBT1
YJR056C	YJR056C	YLR094C	GIS3
YBR200W	BEM1	YDR456W	NHX1
YMR026C	PEX12	YGL033W	HOP2
YHR111W	UBA4	YCR077C	PAT1
YNL058C	YNL058C	YDR310C	SUM1
YPR051W	MAK3	YOR109W	INP53
YPR017C	DSS4	YPL234C	TFP3
YNR052C	POP2	YOL090W	MSH2

Predictions at threshold level 0.75 continued...			
Gene1 of predicted pair		Gene2 of predicted pair	
Systematic Name	Standard Name	Systematic Name	Standard Name
YHL031C	GOS1	YER139C	YER139C
YIR005W	IST3	YFR030W	MET10
YMR224C	MRE11	YPL253C	VIK1
YGL087C	MMS2	YJL154C	VPS35
YNL069C	RPL16B	YGL212W	VAM7
YDR293C	SSD1	YOR208W	PTP2
YMR152W	YIM1	YEL060C	PRB1
YBL102W	SFT2	YOR109W	INP53
YGR261C	APL6	YHL020C	OPI1
YBL058W	SHP1	YNR072W	HXT17
YBL047C	EDE1	YNL079C	TPM1
YJL036W	SNX4	YHL020C	OPI1
YOR358W	HAP5	YPL256C	CLN2
YBR126C	TPS1	YDR477W	SNF1
YKR048C	NAP1	YGR135W	PRE9
YJR056C	YJR056C	YOL111C	MDY2
YKL032C	IXR1	YEL056W	HAT2
YNL297C	MON2	YDR310C	SUM1
YMR224C	MRE11	YOR327C	SNC2
YNL291C	MID1	YPL253C	VIK1
YKL139W	CTK1	YKL213C	DOA1
YCR034W	FEN1	YKL101W	HSL1
YKL139W	CTK1	YNR072W	HXT17
YBR274W	CHK1	YHR039C-A	VMA10
YDR028C	REG1	YDL229W	SSB1
YEL053C	MAK10	YLR094C	GIS3
YDR479C	PEX29	YMR204C	INP1
YMR055C	BUB2	YLR350W	ORM2
YPR084W	YPR084W	YOR308C	SNU66

Predictions at threshold level 0.75 continued...			
Gene1 of predicted pair		Gene2 of predicted pair	
Systematic Name	Standard Name	Systematic Name	Standard Name
YBR280C	SAF1	YMR230W	RPS10B
YOR140W	SFL1	YPL256C	CLN2
YGL185C	YGL185C	YPL240C	HSP82
YLR192C	HCR1	YDL077C	VAM6
YCR020C-A	MAK31	YHR060W	VMA22
YFR010W	UBP6	YPL018W	CTF19
YBR048W	RPS11B	YHR203C	RPS4B
YKL139W	CTK1	YBR030W	YBR030W
YHR013C	ARD1	YJL095W	BCK1
YBR131W	CCZ1	YHR200W	RPN10
YNL206C	RTT106	YGR135W	PRE9
YER087C-B	SBH1	YBR175W	SWD3
YDR146C	SWI5	YPL158C	YPL158C
YGR020C	VMA7	YKL041W	VPS24
YLR079W	SIC1	YOL054W	PSH1
YGR241C	YAP1802	YBR127C	VMA2
YNL069C	RPL16B	YMR054W	STV1
YOR337W	TEA1	YBR036C	CSG2
YBR171W	SEC66	YDR289C	RTT103
YHR156C	LIN1	YPR030W	CSR2
YDR410C	STE14	YOL081W	IRA2
YGL212W	VAM7	YPR032W	SRO7
YOR290C	SNF2	YHR060W	VMA22
YOR332W	VMA4	YPR036W	VMA13
YNL166C	BNI5	YKL048C	ELM1
YBR107C	IML3	YLR182W	SWI6
YDR335W	MSN5	YEL013W	VAC8
YNL307C	MCK1	YHR111W	UBA4
YLR288C	MEC3	YKL101W	HSL1

Predictions at threshold level 0.75 continued...			
Gene1 of predicted pair		Gene2 of predicted pair	
Systematic Name	Standard Name	Systematic Name	Standard Name
YDL184C	RPL41A	YLR093C	NYV1
YPL161C	BEM4	YLR320W	MMS22
YDR129C	SAC6	YPL045W	VPS16
YNL025C	SSN8	YCR084C	TUP1
YPR084W	YPR084W	YNL297C	MON2
YBR057C	MUM2	YKL041W	VPS24
YBR182C	SMP1	YKL161C	YKL161C
YLR234W	TOP3	YDR318W	MCM21
YLR386W	VAC14	YBR107C	IML3
YLR441C	RPS1A	YHR081W	LRP1
YCR020C-A	MAK31	YGL054C	ERV14
YDR363W-A	SEM1	YIR005W	IST3
YDL226C	GCS1	YPR119W	CLB2
YMR276W	DSK2	YNR006W	VPS27
YBR217W	ATG12	YBR019C	GAL10
YCL001W	RER1	YPR032W	SRO7
YEL053C	MAK10	YMR054W	STV1
YBR030W	YBR030W	YEL060C	PRB1
YDL042C	SIR2	YER110C	KAP123
YGL054C	ERV14	YOL108C	INO4
YKR048C	NAP1	YBL087C	RPL23A
YKR035W-A	DID2	YDR486C	VPS60
YER116C	SLX8	YGL175C	SAE2
YBR260C	RGD1	YEL036C	ANP1
YFR010W	UBP6	YBR036C	CSG2
YLR234W	TOP3	YHR010W	RPL27A
YLR216C	CPR6	YOL054W	PSH1
YEL053C	MAK10	YJR084W	CSN12
YEL065W	SIT1	YOL111C	MDY2

Predictions at threshold level 0.75 continued...			
Gene1 of predicted pair		Gene2 of predicted pair	
Systematic Name	Standard Name	Systematic Name	Standard Name
YDR320C	SWA2	YPL161C	BEM4
YIL036W	CST6	YMR055C	BUB2
YOR043W	WHI2	YLR370C	ARC18
YHR156C	LIN1	YMR016C	SOK2
YGR184C	UBR1	YMR055C	BUB2
YJR035W	RAD26	YKL114C	APN1
YBR127C	VMA2	YJL095W	BCK1
YGL223C	COG1	YNL051W	COG5
YBL078C	ATG8	YKR001C	VPS1
YGL115W	SNF4	YLR180W	SAM1
YLR192C	HCR1	YPL057C	SUR1
YKR057W	RPS21A	YPL256C	CLN2
YMR026C	PEX12	YOR066W	MSA1
YLR292C	SEC72	YBR108W	YBR108W
YPR040W	TIP41	YNR032W	PPG1
YDL005C	MED2	YNL107W	YAF9
YMR080C	NAM7	YPL057C	SUR1
YDL122W	UBP1	YMR276W	DSK2
YOR106W	VAM3	YEL042W	GDA1
YCL037C	SRO9	YDR335W	MSN5
YMR004W	MVP1	YML071C	COG8
YER044C	ERG28	YDR261C	EXG2
YHR111W	UBA4	YDR183W	PLP1
YNL291C	MID1	YNL297C	MON2
YDR142C	PEX7	YIL160C	POT1
YGR159C	NSR1	YFR036W	CDC26
YLR433C	CNA1	YDR184C	ATC1
YMR142C	RPL13B	YLR180W	SAM1
YDR137W	RGP1	YOL121C	RPS19A

Predictions at threshold level 0.75 continued...			
Gene1 of predicted pair		Gene2 of predicted pair	
Systematic Name	Standard Name	Systematic Name	Standard Name
YGR072W	UPF3	YJR140C	HIR3
YGR020C	VMA7	YGR105W	VMA21
YOR001W	RRP6	YPR046W	MCM16
YHR079C	IRE1	YOL148C	SPT20
YBR108W	YBR108W	YNL076W	MKS1
YGR249W	MGA1	YJL095W	BCK1
YOR127W	RGA1	YDR264C	AKR1
YPR084W	YPR084W	YDR335W	MSN5
YKR057W	RPS21A	YBL047C	EDE1
YBL058W	SHP1	YHR156C	LIN1
YDR293C	SSD1	YJL127C	SPT10
YNL201C	PSY2	YIL036W	CST6
YLR079W	SIC1	YIR005W	IST3
YGR104C	SRB5	YLR410W	VIP1
YLL039C	UBI4	YEL044W	IES6
YOR076C	SKI7	YBR228W	SLX1
YDR260C	SWM1	YHR013C	ARD1
YDR293C	SSD1	YHL020C	OPI1
YNL218W	MGS1	YGR072W	UPF3
YKR099W	BAS1	YHR060W	VMA22
YNL058C	YNL058C	YDR207C	UME6
YGL033W	HOP2	YER044C	ERG28
YLR441C	RPS1A	YLR093C	NYV1
YBL102W	SFT2	YKR001C	VPS1
YEL053C	MAK10	YJR033C	RAV1
YGR249W	MGA1	YJL183W	MNN11
YGR104C	SRB5	YOR106W	VAM3
YOR327C	SNC2	YNL107W	YAF9
YGL174W	BUD13	YGR188C	BUB1

Predictions at threshold level 0.75 continued...			
Gene1 of predicted pair		Gene2 of predicted pair	
Systematic Name	Standard Name	Systematic Name	Standard Name
YLR119W	SRN2	YLR180W	SAM1
YDR507C	GIN4	YLL054C	YLL054C
YIL159W	BNR1	YOL081W	IRA2
YNL238W	KEX2	YDL077C	VAM6
YJR056C	YJR056C	YER040W	GLN3
YDL223C	HBT1	YER044C	ERG28
YJR059W	PTK2	YPL256C	CLN2
YDR484W	VPS52	YBL025W	RRN10
YDR137W	RGP1	YNL058C	YNL058C
YKL139W	CTK1	YGR135W	PRE9
YMR153W	NUP53	YNL041C	COG6
YPL106C	SSE1	YNL107W	YAF9
YDR310C	SUM1	YOL108C	INO4
YPR030W	CSR2	YDL077C	VAM6
YCR034W	FEN1	YDR310C	SUM1
YMR125W	STO1	YKR024C	DBP7
YDL080C	THI3	YHR178W	STB5
YNL288W	CAF40	YDR144C	MKC7
YEL065W	SIT1	YNL238W	KEX2
YGL153W	PEX14	YOL081W	IRA2
YMR224C	MRE11	YLR371W	ROM2
YEL009C	GCN4	YDR073W	SNF11
YDL184C	RPL41A	YJR056C	YJR056C
YJL154C	VPS35	YOR109W	INP53
YGL178W	MPT5	YKL079W	SMY1
YAL024C	LTE1	YBR280C	SAF1
YLR079W	SIC1	YPL256C	CLN2
YDL101C	DUN1	YJL187C	SWE1
YOR069W	VPS5	YOR327C	SNC2

Predictions at threshold level 0.75 continued...			
Gene1 of predicted pair		Gene2 of predicted pair	
Systematic Name	Standard Name	Systematic Name	Standard Name
YPR135W	CTF4	YHR056C	RSC30
YIL036W	CST6	YIL103W	DPH1
YPL024W	RMI1	YDR484W	VPS52
YEL009C	GCN4	YDR216W	ADR1
YHR134W	WSS1	YNL166C	BNI5
YBR111W-A	SUS1	YGL252C	RTG2
YDR358W	GGA1	YBR019C	GAL10
YML016C	PPZ1	YJR063W	RPA12
YPL253C	VIK1	YOR008C	SLG1
YDL020C	RPN4	YPR131C	NAT3
YDL013W	SLX5	YLR032W	RAD5
YDR004W	RAD57	YOR043W	WHI2
YJL149W	YJL149W	YJL204C	RCY1
YLR292C	SEC72	YGR270W	YTA7
YDR144C	MKC7	YJL190C	RPS22A
YCR065W	HCM1	YNL147W	LSM7
YJR090C	GRR1	YAR014C	BUD14
YEL022W	GEA2	YPL051W	ARL3
YEL065W	SIT1	YBL025W	RRN10
YER044C	ERG28	YHR081W	LRP1
YDR477W	SNF1	YML001W	YPT7
YGL094C	PAN2	YDR485C	VPS72
YOL112W	MSB4	YOR327C	SNC2
YDR440W	DOT1	YBL072C	RPS8A
YJR135C	MCM22	YDL047W	SIT4
YCR086W	CSM1	YGL151W	NUT1
YDR108W	GSG1	YCL037C	SRO9
YHR081W	LRP1	YER139C	YER139C
YLR441C	RPS1A	YLR094C	GIS3

Predictions at threshold level 0.75 continued...			
Gene1 of predicted pair		Gene2 of predicted pair	
Systematic Name	Standard Name	Systematic Name	Standard Name
YLR447C	VMA6	YJR033C	RAV1
YDR378C	LSM6	YNL147W	LSM7
YLR095C	IOC2	YER040W	GLN3
YDR477W	SNF1	YLR320W	MMS22
YMR109W	MYO5	YEL056W	HAT2
YML073C	RPL6A	YPR119W	CLB2
YNL253W	TEX1	YML062C	MFT1
YPR084W	YPR084W	YER162C	RAD4
YCR020C-A	MAK31	YPL195W	APL5
YOL018C	TLG2	YDR310C	SUM1
YLR079W	SIC1	YKL114C	APN1
YMR152W	YIM1	YCR053W	THR4
YJL004C	SYS1	YML071C	COG8
YML115C	VAN1	YGL153W	PEX14
YLR192C	HCR1	YOR066W	MSA1
YLR083C	EMP70	YPL120W	VPS30
YNL025C	SSN8	YOR080W	DIA2
YEL036C	ANP1	YKL041W	VPS24
YKL139W	CTK1	YBR020W	GAL1
YOR076C	SKI7	YHL002W	HSE1
YDR076W	RAD55	YPL018W	CTF19
YBR108W	YBR108W	YER110C	KAP123
YDR174W	HMO1	YFR030W	MET10
YIR009W	MSL1	YDL001W	RMD1
YPR052C	NHP6A	YOL051W	GAL11
YDR378C	LSM6	YMR055C	BUB2
YOR112W	CEX1	YER120W	SCS2
YPL195W	APL5	YER040W	GLN3
YNL166C	BNI5	YGR270W	YTA7

Predictions at threshold level 0.75 continued...			
Gene1 of predicted pair		Gene2 of predicted pair	
Systematic Name	Standard Name	Systematic Name	Standard Name
YER001W	MNN1	YGL038C	OCH1
YJL053W	PEP8	YJL036W	SNX4
YJL154C	VPS35	YOR112W	CEX1
YNL325C	FIG4	YBR200W	BEM1
YDR156W	RPA14	YJL124C	LSM1
YJL053W	PEP8	YDR484W	VPS52
YPL105C	YPL105C	YNL238W	KEX2
YJR040W	GEF1	YEL043W	YEL043W
YNR052C	POP2	YBR082C	UBC4
YDL101C	DUN1	YJL124C	LSM1
YDR440W	DOT1	YOR308C	SNU66
YEL053C	MAK10	YHR114W	BZZ1
YKL094W	YJU3	YMR152W	YIM1
YJL124C	LSM1	YDL013W	SLX5
YJL124C	LSM1	YAL002W	VPS8
YOR290C	SNF2	YOR043W	WHI2
YLR399C	BDF1	YDR378C	LSM6
YNL307C	MCK1	YNL107W	YAF9
YGL253W	HXK2	YHR114W	BZZ1
YHR200W	RPN10	YLR180W	SAM1
YNR032C-A	HUB1	YDL223C	HBT1
YHR200W	RPN10	YDL155W	CLB3
YOR195W	SLK19	YOR014W	RTS1
YDR146C	SWI5	YHR158C	KEL1
YML103C	NUP188	YOL054W	PSH1
YJL127C	SPT10	YDR363W-A	SEM1
YLR386W	VAC14	YIL036W	CST6
YOR337W	TEA1	YCR053W	THR4
YGL200C	EMP24	YLR093C	NYV1

Predictions at threshold level 0.75 continued...			
Gene1 of predicted pair		Gene2 of predicted pair	
Systematic Name	Standard Name	Systematic Name	Standard Name
YBR107C	IML3	YJL006C	CTK2
YGL223C	COG1	YNL293W	MSB3
YNR052C	POP2	YPL018W	CTF19
YGL025C	PGD1	YCR084C	TUP1
YIL159W	BNR1	YLR119W	SRN2
YCR038C	BUD5	YPL240C	HSP82
YCR065W	HCM1	YJL058C	BIT61
YNL201C	PSY2	YKL048C	ELM1
YOR327C	SNC2	YDL077C	VAM6
YLR337C	VRP1	YER110C	KAP123
YKL007W	CAP1	YIL133C	RPL16A
YMR142C	RPL13B	YBR036C	CSG2
YGL178W	MPT5	YDR335W	MSN5
YLR079W	SIC1	YOR014W	RTS1
YNL291C	MID1	YMR054W	STV1
YJR031C	GEA1	YKR030W	GMH1
YML038C	YMD8	YNL041C	COG6
YOR106W	VAM3	YEL013W	VAC8
YGR249W	MGA1	YOL081W	IRA2
YHR156C	LIN1	YKL126W	YPK1
YLR371W	ROM2	YNL076W	MKS1
YPL045W	VPS16	YOR036W	PEP12
YGL223C	COG1	YHL020C	OPI1
YNL139C	RLR1	YNL253W	TEX1
YDR477W	SNF1	YDL192W	ARF1
YJR043C	POL32	YHR056C	RSC30
YHR111W	UBA4	YHR013C	ARD1
YGR020C	VMA7	YOL108C	INO4
YCL067C	HMLALPHA2	YCL066W	HMLALPHA1

Predictions at threshold level 0.75 continued...			
Gene1 of predicted pair		Gene2 of predicted pair	
Systematic Name	Standard Name	Systematic Name	Standard Name
YLR079W	SIC1	YLR058C	SHM2
YMR095C	SNO1	YDL077C	VAM6
YMR198W	CIK1	YBR181C	RPS6B
YFR036W	CDC26	YMR152W	YIM1
YLR386W	VAC14	YNL032W	SIW14
YNL248C	RPA49	YMR055C	BUB2
YDR363W-A	SEM1	YOR014W	RTS1
YHR200W	RPN10	YGL031C	RPL24A
YLR315W	NKP2	YOR014W	RTS1
YOL111C	MDY2	YLR094C	GIS3
YMR080C	NAM7	YFR010W	UBP6
YKL025C	PAN3	YDL155W	CLB3
YOR231W	MKK1	YER089C	PTC2
YOR076C	SKI7	YGL213C	SKI8
YDL077C	VAM6	YDR207C	UME6
YNL139C	RLR1	YER118C	SHO1
YDR363W-A	SEM1	YDR254W	CHL4
YDR261C	EXG2	YHR081W	LRP1
YLR386W	VAC14	YBR108W	YBR108W
YLR216C	CPR6	YGR135W	PRE9
YGL237C	HAP2	YOR358W	HAP5
YGR249W	MGA1	YNL058C	YNL058C
YGR249W	MGA1	YGL124C	MON1
YBR200W	BEM1	YER049W	TPA1
YOR033C	EXO1	YMR284W	YKU70
YJL029C	VPS53	YKR001C	VPS1
YJL154C	VPS35	YGR019W	UGA1
YMR199W	CLN1	YHR108W	GGA2
YCR024C-A	PMP1	YOR358W	HAP5

Predictions at threshold level 0.75 continued...			
Gene1 of predicted pair		Gene2 of predicted pair	
Systematic Name	Standard Name	Systematic Name	Standard Name
YDL020C	RPN4	YGR184C	UBR1
YPR131C	NAT3	YER116C	SLX8
YER144C	UBP5	YJR032W	CPR7
YLR083C	EMP70	YMR054W	STV1
YFL023W	BUD27	YGL212W	VAM7
YIL034C	CAP2	YML016C	PPZ1
YLR429W	CRN1	YGL003C	CDH1
YDR080W	VPS41	YPL045W	VPS16
YMR127C	SAS2	YIL008W	URM1
YNL288W	CAF40	YGR054W	YGR054W
YPL152W	RRD2	YLR254C	NDL1
YJR140C	HIR3	YKL213C	DOA1
YEL053C	MAK10	YJR090C	GRR1
YPR120C	CLB5	YHR108W	GGA2
YBR217W	ATG12	YLR093C	NYV1
YNL076W	MKS1	YER040W	GLN3
YDR318W	MCM21	YAR014C	BUD14
YGL003C	CDH1	YOR141C	ARP8
YOR043W	WHI2	YDR289C	RTT103
YMR224C	MRE11	YER144C	UBP5
YGR268C	HUA1	YJL004C	SYS1
YBL058W	SHP1	YGL033W	HOP2
YNL215W	IES2	YOL081W	IRA2
YKL101W	HSL1	YGL019W	CKB1
YLR441C	RPS1A	YBR280C	SAF1
YLR090W	XDJ1	YNL087W	TCB2
YBR030W	YBR030W	YJR056C	YJR056C
YGR104C	SRB5	YOR014W	RTS1
YBR108W	YBR108W	YER144C	UBP5

Predictions at threshold level 0.75 continued...			
Gene1 of predicted pair		Gene2 of predicted pair	
Systematic Name	Standard Name	Systematic Name	Standard Name
YMR246W	FAA4	YKL140W	TGL1
YHR060W	VMA22	YML071C	COG8
YOR069W	VPS5	YMR004W	MVP1
YBL025W	RRN10	YGL124C	MON1
YPR051W	MAK3	YLR309C	IMH1
YER143W	DDI1	YJL164C	TPK1
YNL307C	MCK1	YKL110C	KTI12
YML115C	VAN1	YOR043W	WHI2
YLR093C	NYV1	YOR036W	PEP12
YDR335W	MSN5	YNL199C	GCR2
YGL212W	VAM7	YJL183W	MNN11
YBL008W	HIR1	YOR141C	ARP8
YNL329C	PEX6	YHR013C	ARD1
YOR106W	VAM3	YPL161C	BEM4
YJL036W	SNX4	YCR066W	RAD18
YLR396C	VPS33	YML001W	YPT7
YKL116C	PRR1	YNL325C	FIG4
YHR114W	BZZ1	YBR114W	RAD16
YDR225W	HTA1	YOR038C	HIR2
YBR228W	SLX1	YOL030W	GAS5
YBL047C	EDE1	YML071C	COG8
YMR116C	ASC1	YJL124C	LSM1
YEL053C	MAK10	YKR030W	GMH1
YOR195W	SLK19	YFR030W	MET10
YKL048C	ELM1	YJL095W	BCK1
YJR060W	CBF1	YPL152W	RRD2
YDL006W	PTC1	YJL095W	BCK1
YML016C	PPZ1	YLR315W	NKP2
YLR216C	CPR6	YDR363W-A	SEM1

Predictions at threshold level 0.75 continued...			
Gene1 of predicted pair		Gene2 of predicted pair	
Systematic Name	Standard Name	Systematic Name	Standard Name
YPL024W	RMI1	YLR371W	ROM2
YBR171W	SEC66	YGL200C	EMP24
YDR335W	MSN5	YHR111W	UBA4
YER116C	SLX8	YML001W	YPT7
YGL033W	HOP2	YCR053W	THR4
YPL024W	RMI1	YIR002C	MPH1
YDR261C	EXG2	YJL183W	MNN11
YCL001W	RER1	YNL041C	COG6
YDR484W	VPS52	YMR230W	RPS10B
YLR292C	SEC72	YBR171W	SEC66
YNL044W	YIP3	YJL204C	RCY1
YDL020C	RPN4	YGL151W	NUT1
YLR360W	VPS38	YKR020W	VPS51
YLR441C	RPS1A	YMR230W	RPS10B
YBR041W	FAT1	YMR038C	CCS1
YBL103C	RTG3	YOR290C	SNF2
YNL206C	RTT106	YPL161C	BEM4
YOR290C	SNF2	YBR171W	SEC66
YNL058C	YNL058C	YJL095W	BCK1
YJL183W	MNN11	YJR084W	CSN12
YNL288W	CAF40	YIL038C	NOT3
YFL023W	BUD27	YPL037C	EGD1
YOR083W	WHI5	YBR215W	HPC2
YHR134W	WSS1	YJR090C	GRR1
YMR198W	CIK1	YNR052C	POP2
YOR069W	VPS5	YBR131W	CCZ1
YAL024C	LTE1	YBR030W	YBR030W
YEL053C	MAK10	YBR114W	RAD16
YFR010W	UBP6	YOL090W	MSH2

Predictions at threshold level 0.75 continued...			
**Gene1 of predicted pair**		**Gene2 of predicted pair**	
**Systematic Name**	**Standard Name**	**Systematic Name**	**Standard Name**
YHR060W	VMA22	YMR230W	RPS10B
YER144C	UBP5	YMR230W	RPS10B
YKR035W-A	DID2	YOR043W	WHI2
YIL008W	URM1	YJR090C	GRR1
YBR126C	TPS1	YJR056C	YJR056C
YAL010C	MDM10	YGL163C	RAD54
YKL007W	CAP1	YPL256C	CLN2
YHR134W	WSS1	YAR014C	BUD14
YPL253C	VIK1	YEL056W	HAT2
YML016C	PPZ1	YBR114W	RAD16
YDR289C	RTT103	YGL253W	HXK2
YOR275C	RIM20	YDR310C	SUM1
YPL256C	CLN2	YGL003C	CDH1
YEL053C	MAK10	YNL051W	COG5
YNL291C	MID1	YLR381W	CTF3
YDR383C	NKP1	YDR289C	RTT103
YNL291C	MID1	YNL238W	KEX2
YKR001C	VPS1	YLR396C	VPS33
YGL078C	DBP3	YGL115W	SNF4
YMR116C	ASC1	YNR052C	POP2
YMR080C	NAM7	YBR017C	KAP104
YBR171W	SEC66	YJR090C	GRR1
YPL240C	HSP82	YJR159W	SOR1
YIL159W	BNR1	YMR153W	NUP53
YIL008W	URM1	YKL101W	HSL1
YDR477W	SNF1	YHR060W	VMA22
YGR072W	UPF3	YGR261C	APL6
YIL036W	CST6	YJR135C	MCM22
YPR131C	NAT3	YDR200C	VPS64

Predictions at threshold level 0.75 continued...			
Gene1 of predicted pair		Gene2 of predicted pair	
Systematic Name	Standard Name	Systematic Name	Standard Name
YKL139W	CTK1	YPR046W	MCM16
YKR099W	BAS1	YMR230W	RPS10B
YKR061W	KTR2	YOL081W	IRA2
YPR173C	VPS4	YDL226C	GCS1
YDL223C	HBT1	YHR060W	VMA22
YPL022W	RAD1	YJR135C	MCM22
YPL195W	APL5	YEL060C	PRB1
YGL237C	HAP2	YDR318W	MCM21
YDR254W	CHL4	YDR289C	RTT103
YGR020C	VMA7	YNR032W	PPG1
YLR061W	RPL22A	YJL095W	BCK1
YIL076W	SEC28	YDL223C	HBT1
YPL234C	TFP3	YLR370C	ARC18
YGL087C	MMS2	YHR134W	WSS1
YER116C	SLX8	YNL041C	COG6
YML095C	RAD10	YJL095W	BCK1
YLL042C	ATG10	YNR007C	ATG3
YJL136C	RPS21B	YPL161C	BEM4
YIL008W	URM1	YDR264C	AKR1
YER110C	KAP123	YCR086W	CSM1
YDL020C	RPN4	YDL190C	UFD2
YPR131C	NAT3	YNL107W	YAF9
YPL022W	RAD1	YMR201C	RAD14
YGR104C	SRB5	YDR192C	NUP42
YPL147W	PXA1	YKL188C	PXA2
YKR001C	VPS1	YML038C	YMD8
YLR018C	POM34	YKR024C	DBP7
YML001W	YPT7	YDL192W	ARF1
YHR039C-A	VMA10	YJL183W	MNN11

Predictions at threshold level 0.75 continued...			
Gene1 of predicted pair		Gene2 of predicted pair	
Systematic Name	Standard Name	Systematic Name	Standard Name
YOR275C	RIM20	YMR022W	UBC7
YML103C	NUP188	YDR207C	UME6
YPL105C	YPL105C	YAR014C	BUD14
YGL174W	BUD13	YNL199C	GCR2
YPL161C	BEM4	YNL051W	COG5
YML095C	RAD10	YOR358W	HAP5
YBR194W	SOY1	YOR308C	SNU66
YBR107C	IML3	YNL236W	SIN4
YBL058W	SHP1	YPL161C	BEM4
YLR090W	XDJ1	YGL147C	RPL9A
YNR052C	POP2	YLR313C	SPH1
YIL159W	BNR1	YKL129C	MYO3
YER139C	YER139C	YER040W	GLN3
YOR132W	VPS17	YOL067C	RTG1
YPL037C	EGD1	YHR060W	VMA22
YOR358W	HAP5	YDR310C	SUM1
YNL288W	CAF40	YER110C	KAP123
YCL037C	SRO9	YPL234C	TFP3
YOR129C	YOR129C	YNL225C	CNM67
YOR066W	MSA1	YGL124C	MON1
YNL307C	MCK1	YDR254W	CHL4
YNL206C	RTT106	YMR038C	CCS1
YLR288C	MEC3	YJR090C	GRR1
YFL023W	BUD27	YNR072W	HXT17
YOL052C-A	DDR2	YPL240C	HSP82
YBL103C	RTG3	YER122C	GLO3
YGL212W	VAM7	YOR109W	INP53
YDR174W	HMO1	YNL307C	MCK1
YGR072W	UPF3	YJR033C	RAV1

Predictions at threshold level 0.75 continued...			
Gene1 of predicted pair		Gene2 of predicted pair	
Systematic Name	Standard Name	Systematic Name	Standard Name
YKL110C	KTI12	YHR187W	IKI1
YPL161C	BEM4	YDL047W	SIT4
YGR152C	RSR1	YNR049C	MSO1
YBR194W	SOY1	YPL106C	SSE1
YCR066W	RAD18	YNL041C	COG6
YOR085W	OST3	YEL036C	ANP1
YER116C	SLX8	YGL031C	RPL24A
YGR270W	YTA7	YKL213C	DOA1
YBR108W	YBR108W	YER044C	ERG28
YDL223C	HBT1	YKR035W-A	DID2
YJL154C	VPS35	YOL067C	RTG1
YGR081C	SLX9	YLR352W	YLR352W
YKL116C	PRR1	YLR441C	RPS1A
YGL087C	MMS2	YPR051W	MAK3
YGL223C	COG1	YPR032W	SRO7
YLR381W	CTF3	YJL058C	BIT61
YLR371W	ROM2	YBL101C	ECM21
YDR439W	LRS4	YDR183W	PLP1
YMR198W	CIK1	YOR195W	SLK19
YPL024W	RMI1	YJL176C	SWI3
YNL025C	SSN8	YJR140C	HIR3
YLR360W	VPS38	YNL199C	GCR2
YLR095C	IOC2	YMR224C	MRE11
YPR036W	VMA13	YHR039C-A	VMA10
YKR061W	KTR2	YJL124C	LSM1
YLR441C	RPS1A	YDR358W	GGA1
YBR194W	SOY1	YKL041W	VPS24
YOR195W	SLK19	YJL058C	BIT61
YOR008C	SLG1	YJL129C	TRK1

Predictions at threshold level 0.75 continued...			
Gene1 of predicted pair		Gene2 of predicted pair	
Systematic Name	Standard Name	Systematic Name	Standard Name
YBR290W	BSD2	YGL016W	KAP122
YDL116W	NUP84	YNL116W	DMA2
YPL042C	SSN3	YPR120C	CLB5
YBR215W	HPC2	YIR005W	IST3
YGL087C	MMS2	YPR120C	CLB5
YDR477W	SNF1	YER044C	ERG28
YNL307C	MCK1	YBL047C	EDE1
YHR156C	LIN1	YML001W	YPT7
YBR030W	YBR030W	YJL095W	BCK1
YBR036C	CSG2	YLR180W	SAM1
YBR171W	SEC66	YDR318W	MCM21
YDL227C	HO	YER143W	DDI1
YIL159W	BNR1	YDR507C	GIN4
YNL297C	MON2	YML071C	COG8
YMR055C	BUB2	YJL006C	CTK2
YGL212W	VAM7	YKR099W	BAS1
YDL223C	HBT1	YDL083C	RPS16B
YLR181C	VTA1	YOR275C	RIM20
YHR013C	ARD1	YGR188C	BUB1
YLR109W	AHP1	YGR209C	TRX2
YJL187C	SWE1	YMR038C	CCS1
YHL025W	SNF6	YDR073W	SNF11
YLR095C	IOC2	YLR192C	HCR1
YPL024W	RMI1	YGL033W	HOP2
YOR069W	VPS5	YOL108C	INO4
YKL135C	APL2	YOR327C	SNC2
YDR289C	RTT103	YHR167W	THP2
YER001W	MNN1	YGR135W	PRE9
YLR381W	CTF3	YNL225C	CNM67

Predictions at threshold level 0.75 continued...			
Gene1 of predicted pair		Gene2 of predicted pair	
Systematic Name	Standard Name	Systematic Name	Standard Name
YDR083W	RRP8	YOR145C	PNO1
YOR132W	VPS17	YOR109W	INP53
YER054C	GIP2	YAR014C	BUD14
YDL155W	CLB3	YDR200C	VPS64
YOR043W	WHI2	YOR327C	SNC2
YGL025C	PGD1	YOR035C	SHE4
YNL106C	INP52	YHR115C	DMA1
YER120W	SCS2	YOL018C	TLG2
YGR178C	PBP1	YPR119W	CLB2
YLR079W	SIC1	YDL155W	CLB3
YDR443C	SSN2	YJR032W	CPR7
YJL172W	CPS1	YOR327C	SNC2
YML016C	PPZ1	YLR371W	ROM2
YMR055C	BUB2	YJL190C	RPS22A
YMR153W	NUP53	YER144C	UBP5
YOR023C	AHC1	YNL166C	BNI5
YGL147C	RPL9A	YNL325C	FIG4
YIL156W	UBP7	YML057W	CMP2
YIL034C	CAP2	YDR310C	SUM1
YLR058C	SHM2	YJR005W	APL1
YDL020C	RPN4	YOR195W	SLK19
YLR337C	VRP1	YML095C	RAD10
YJL183W	MNN11	YGR188C	BUB1
YDR156W	RPA14	YMR116C	ASC1
YBR200W	BEM1	YJR135C	MCM22
YDL005C	MED2	YGL151W	NUT1
YGR241C	YAP1802	YBL017C	PEP1
YNL201C	PSY2	YNL107W	YAF9
YBR171W	SEC66	YHR081W	LRP1

Predictions at threshold level 0.75 continued...			
Gene1 of predicted pair		Gene2 of predicted pair	
Systematic Name	Standard Name	Systematic Name	Standard Name
YLR083C	EMP70	YCL037C	SRO9
YLR315W	NKP2	YOR141C	ARP8
YLR192C	HCR1	YMR153W	NUP53
YDR422C	SIP1	YDR028C	REG1
YLR234W	TOP3	YLR386W	VAC14
YNL264C	PDR17	YGL223C	COG1
YLR399C	BDF1	YGL019W	CKB1
YKR059W	TIF1	YMR055C	BUB2
YCR020C-A	MAK31	YML001W	YPT7
YLR148W	PEP3	YLR396C	VPS33
YGR261C	APL6	YAR014C	BUD14
YBR057C	MUM2	YPR046W	MCM16
YDL180W	YDL180W	YML038C	YMD8
YBR041W	FAT1	YMR152W	YIM1
YIL159W	BNR1	YER144C	UBP5
YPR051W	MAK3	YPL161C	BEM4
YGR072W	UPF3	YGR105W	VMA21
YGR104C	SRB5	YKL068W	NUP100
YOR080W	DIA2	YER116C	SLX8
YMR127C	SAS2	YDR144C	MKC7
YDR108W	GSG1	YGL212W	VAM7
YCL001W	RER1	YML012W	ERV25
YER110C	KAP123	YGR252W	GCN5
YDL160C	DHH1	YDR192C	NUP42
YHR200W	RPN10	YDR264C	AKR1
YJR140C	HIR3	YGR188C	BUB1
YGL212W	VAM7	YPR030W	CSR2
YHR129C	ARP1	YGL190C	CDC55
YER114C	BOI2	YCR077C	PAT1

Predictions at threshold level 0.75 continued...			
Gene1 of predicted pair		Gene2 of predicted pair	
Systematic Name	Standard Name	Systematic Name	Standard Name
YJR140C	HIR3	YMR167W	MLH1
YJL095W	BCK1	YKL041W	VPS24
YNL297C	MON2	YNL127W	FAR11
YBR274W	CHK1	YOR368W	RAD17
YLR192C	HCR1	YGL212W	VAM7
YLR102C	APC9	YPL161C	BEM4
YPR018W	RLF2	YML095C	RAD10
YNL291C	MID1	YMR167W	MLH1
YDR213W	UPC2	YBR127C	VMA2
YIL053W	RHR2	YBR280C	SAF1
YJL172W	CPS1	YGL212W	VAM7
YOR069W	VPS5	YGL223C	COG1
YOR127W	RGA1	YLR309C	IMH1
YBL072C	RPS8A	YDR207C	UME6
YGL178W	MPT5	YLR384C	IKI3
YNL058C	YNL058C	YOR043W	WHI2
YJR010C-A	SPC1	YJL128C	PBS2
YKR072C	SIS2	YLR410W	VIP1
YDL020C	RPN4	YDR318W	MCM21
YLR371W	ROM2	YER122C	GLO3
YLR452C	SST2	YLR094C	GIS3
YHR108W	GGA2	YKL161C	YKL161C
YMR095C	SNO1	YHR081W	LRP1
YLR119W	SRN2	YIL076W	SEC28
YPR018W	RLF2	YHR200W	RPN10
YGR261C	APL6	YKL135C	APL2
YJR090C	GRR1	YEL056W	HAT2
YML095C	RAD10	YKL032C	IXR1
YJL127C	SPT10	YMR179W	SPT21

Predictions at threshold level 0.75 continued...			
Gene1 of predicted pair		Gene2 of predicted pair	
Systematic Name	Standard Name	Systematic Name	Standard Name
YBR171W	SEC66	YOL081W	IRA2
YEL051W	VMA8	YDR153C	ENT5
YDL160C	DHH1	YGL208W	SIP2
YDR176W	NGG1	YGL013C	PDR1
YOR290C	SNF2	YPR030W	CSR2
YGL133W	ITC1	YBR084C-A	RPL19A
YLR234W	TOP3	YGL250W	RMR1
YOR337W	TEA1	YLR094C	GIS3
YLR058C	SHM2	YNL107W	YAF9
YPR171W	BSP1	YKL213C	DOA1
YMR026C	PEX12	YNL307C	MCK1
YBR200W	BEM1	YLR441C	RPS1A
YKL032C	IXR1	YJL095W	BCK1
YOR080W	DIA2	YGR270W	YTA7
YPL105C	YPL105C	YCL001W	RER1
YHR161C	YAP1801	YDL226C	GCS1
YBR108W	YBR108W	YMR109W	MYO5
YDR004W	RAD57	YDR207C	UME6
YBL078C	ATG8	YLR043C	TRX1
YBR171W	SEC66	YGL253W	HXK2
YDR293C	SSD1	YLR386W	VAC14
YGL147C	RPL9A	YBL103C	RTG3
YER120W	SCS2	YNL238W	KEX2
YOR191W	RIS1	YPL057C	SUR1
YHR056C	RSC30	YCL016C	DCC1
YHR060W	VMA22	YBR019C	GAL10
YGR252W	GCN5	YOR141C	ARP8
YDR363W-A	SEM1	YLR357W	RSC2
YJL183W	MNN11	YGL124C	MON1

Predictions at threshold level 0.75 continued...			
Gene1 of predicted pair		Gene2 of predicted pair	
Systematic Name	Standard Name	Systematic Name	Standard Name
YER016W	BIM1	YIL033C	BCY1
YOR132W	VPS17	YCR066W	RAD18
YLR234W	TOP3	YOR195W	SLK19
YLR337C	VRP1	YGL161C	YIP5
YCL037C	SRO9	YGL115W	SNF4
YNL243W	SLA2	YGR229C	SMI1
YJL149W	YJL149W	YJR090C	GRR1
YMR153W	NUP53	YKR099W	BAS1
YKL006W	RPL14A	YPL213W	LEA1
YPL024W	RMI1	YEL056W	HAT2
YKL116C	PRR1	YPL037C	EGD1
YGL135W	RPL1B	YKL041W	VPS24
YNL243W	SLA2	YNL084C	END3
YFR010W	UBP6	YLR421C	RPN13
YML001W	YPT7	YPL195W	APL5
YBR200W	BEM1	YHR115C	DMA1
YNL041C	COG6	YJR032W	CPR7
YMR125W	STO1	YDL047W	SIT4
YLR303W	MET17	YNR065C	YNR065C
YER120W	SCS2	YPL120W	VPS30
YDL116W	NUP84	YMR116C	ASC1
YGL212W	VAM7	YNL297C	MON2
YDL160C	DHH1	YDL047W	SIT4
YML112W	CTK3	YGR188C	BUB1
YPL001W	HAT1	YLL022C	HIF1
YNL041C	COG6	YAL002W	VPS8
YFR031C-A	RPL2A	YDR261C	EXG2
YNL032W	SIW14	YCR053W	THR4
YGL003C	CDH1	YJL095W	BCK1

Predictions at threshold level 0.75 continued...			
Gene1 of predicted pair		Gene2 of predicted pair	
Systematic Name	Standard Name	Systematic Name	Standard Name
YOL111C	MDY2	YLR452C	SST2
YEL065W	SIT1	YPL137C	GIP3
YKL110C	KTI12	YPR120C	CLB5
YML115C	VAN1	YDR378C	LSM6
YBR126C	TPS1	YCR053W	THR4
YIL076W	SEC28	YPL137C	GIP3
YNL032W	SIW14	YFL023W	BUD27
YKL126W	YPK1	YDR137W	RGP1
YLR337C	VRP1	YDR254W	CHL4
YML001W	YPT7	YJR084W	CSN12
YGR078C	PAC10	YGL190C	CDC55
YLR320W	MMS22	YJL095W	BCK1
YIL159W	BNR1	YAL020C	ATS1
YLR303W	MET17	YOL126C	MDH2
YLR192C	HCR1	YER040W	GLN3
YBR200W	BEM1	YOR275C	RIM20
YHR039C-A	VMA10	YFR036W	CDC26
YLR288C	MEC3	YDR310C	SUM1
YLR360W	VPS38	YGL212W	VAM7
YDR439W	LRS4	YPR046W	MCM16
YER001W	MNN1	YOR312C	RPL20B
YGR020C	VMA7	YPL161C	BEM4
YIL159W	BNR1	YMR202W	ERG2
YKL126W	YPK1	YER040W	GLN3
YHR013C	ARD1	YJR060W	CBF1
YOR054C	VHS3	YPL240C	HSP82
YCR086W	CSM1	YOR368W	RAD17
YJL176C	SWI3	YMR091C	NPL6
YIL034C	CAP2	YKR048C	NAP1

Predictions at threshold level 0.75 continued...			
Gene1 of predicted pair		Gene2 of predicted pair	
Systematic Name	Standard Name	Systematic Name	Standard Name
YKL025C	PAN3	YGR054W	YGR054W
YPL180W	TCO89	YKL041W	VPS24
YJR059W	PTK2	YFL047W	RGD2
YIL034C	CAP2	YBR171W	SEC66
YPL022W	RAD1	YDR071C	PAA1
YML115C	VAN1	YJR032W	CPR7
YBR173C	UMP1	YDR137W	RGP1
YBR200W	BEM1	YJL157C	FAR1
YOR035C	SHE4	YAL030W	SNC1
YBR200W	BEM1	YLR429W	CRN1
YNL243W	SLA2	YOR033C	EXO1
YFL023W	BUD27	YLR094C	GIS3
YFR031C-A	RPL2A	YNL041C	COG6
YKR099W	BAS1	YPL161C	BEM4
YDL020C	RPN4	YBR127C	VMA2
YJL136C	RPS21B	YNL116W	DMA2
YHR039C-A	VMA10	YOR035C	SHE4
YJR090C	GRR1	YHR114W	BZZ1
YDR440W	DOT1	YER139C	YER139C
YDR484W	VPS52	YDR261C	EXG2
YLR386W	VAC14	YBL047C	EDE1
YJL006C	CTK2	YDR200C	VPS64
YMR153W	NUP53	YPL057C	SUR1
YOR008C	SLG1	YNL031C	HHT2
YCL001W	RER1	YER120W	SCS2
YER116C	SLX8	YMR038C	CCS1
YPR120C	CLB5	YEL056W	HAT2
YIR003W	YIR003W	YDR310C	SUM1
YKL048C	ELM1	YNL079C	TPM1

Predictions at threshold level 0.75 continued...			
Gene1 of predicted pair		Gene2 of predicted pair	
Systematic Name	Standard Name	Systematic Name	Standard Name
YNL307C	MCK1	YLR371W	ROM2
YOR080W	DIA2	YOR124C	UBP2
YMR284W	YKU70	YJR035W	RAD26
YLR288C	MEC3	YBR215W	HPC2
YER162C	RAD4	YMR311C	GLC8
YPL195W	APL5	YKR030W	GMH1
YKL139W	CTK1	YJR056C	YJR056C
YPR131C	NAT3	YDR318W	MCM21
YDL083C	RPS16B	YBR036C	CSG2
YJL006C	CTK2	YBL101C	ECM21
YNL139C	RLR1	YML062C	MFT1
YJR060W	CBF1	YJL006C	CTK2
YCR086W	CSM1	YOL054W	PSH1
YMR109W	MYO5	YLR447C	VMA6
YHR060W	VMA22	YDR153C	ENT5
YNL090W	RHO2	YPR185W	ATG13
YFR010W	UBP6	YOL145C	CTR9
YJL036W	SNX4	YML001W	YPT7
YJL172W	CPS1	YNL058C	YNL058C
YIL008W	URM1	YDR155C	CPR1
YLR452C	SST2	YJL183W	MNN11
YDR225W	HTA1	YFR010W	UBP6
YOR109W	INP53	YGL054C	ERV14
YIL076W	SEC28	YML038C	YMD8
YDR293C	SSD1	YIL133C	RPL16A
YPL057C	SUR1	YNL058C	YNL058C
YAL024C	LTE1	YNL325C	FIG4
YJL124C	LSM1	YPL253C	VIK1
YLL007C	YLL007C	YLR386W	VAC14

Predictions at threshold level 0.75 continued...			
Gene1 of predicted pair		Gene2 of predicted pair	
Systematic Name	Standard Name	Systematic Name	Standard Name
YDR480W	DIG2	YBR083W	TEC1
YCR002C	CDC10	YPL161C	BEM4
YDR004W	RAD57	YNL206C	RTT106
YHR114W	BZZ1	YPR119W	CLB2
YHR039C-A	VMA10	YGL003C	CDH1
YGR104C	SRB5	YLR079W	SIC1
YLR095C	IOC2	YLR093C	NYV1
YDL077C	VAM6	YOL108C	INO4
YIL008W	URM1	YHR012W	VPS29
YOR195W	SLK19	YNL107W	YAF9
YBL058W	SHP1	YBR217W	ATG12
YOR337W	TEA1	YMR142C	RPL13B
YBR194W	SOY1	YPL180W	TCO89
YJL095W	BCK1	YER049W	TPA1
YBR200W	BEM1	YPR030W	CSR2
YER144C	UBP5	YPR036W	VMA13
YGR072W	UPF3	YPL161C	BEM4
YBR289W	SNF5	YHL025W	SNF6
YIL076W	SEC28	YLR180W	SAM1
YPL161C	BEM4	YBL072C	RPS8A
YAL024C	LTE1	YFL023W	BUD27
YJR033C	RAV1	YNL107W	YAF9
YML109W	ZDS2	YBL085W	BOI1
YJR135C	MCM22	YPR046W	MCM16
YLR234W	TOP3	YLR352W	YLR352W
YHR013C	ARD1	YGR270W	YTA7
YML115C	VAN1	YKL032C	IXR1
YJL004C	SYS1	YPR032W	SRO7
YHR134W	WSS1	YGL124C	MON1

Predictions at threshold level 0.75 continued...			
Gene1 of predicted pair		Gene2 of predicted pair	
Systematic Name	Standard Name	Systematic Name	Standard Name
YOR290C	SNF2	YLR182W	SWI6
YDR378C	LSM6	YKL213C	DOA1
YNL068C	FKH2	YML095C	RAD10
YPR017C	DSS4	YOR327C	SNC2
YBR274W	CHK1	YMR167W	MLH1
YLR234W	TOP3	YPL018W	CTF19
YNL032W	SIW14	YDL077C	VAM6
YEL009C	GCN4	YLR258W	GSY2
YNL025C	SSN8	YER161C	SPT2
YLR410W	VIP1	YML028W	TSA1
YGL223C	COG1	YPL161C	BEM4
YFR031C-A	RPL2A	YBR036C	CSG2
YMR080C	NAM7	YDR101C	ARX1
YKR059W	TIF1	YGR054W	YGR054W
YLR386W	VAC14	YHR060W	VMA22
YCL001W	RER1	YFL048C	EMP47
YPR119W	CLB2	YOL108C	INO4
YGL147C	RPL9A	YMR142C	RPL13B
YKR057W	RPS21A	YGL031C	RPL24A
YHR086W	NAM8	YDR110W	FOB1
YOR327C	SNC2	YJL164C	TPK1
YBL047C	EDE1	YMR089C	YTA12
YDR004W	RAD57	YGR184C	UBR1
YJR135C	MCM22	YNL225C	CNM67
YDR004W	RAD57	YFL023W	BUD27
YHR161C	YAP1801	YFL026W	STE2
YDR146C	SWI5	YGL151W	NUT1
YKL164C	PIR1	YKL114C	APN1
YMR201C	RAD14	YOL090W	MSH2

Predictions at threshold level 0.75 continued...			
Gene1 of predicted pair		Gene2 of predicted pair	
Systematic Name	Standard Name	Systematic Name	Standard Name
YKL126W	YPK1	YBR171W	SEC66
YKL048C	ELM1	YDR358W	GGA1
YFR010W	UBP6	YDR358W	GGA1
YBL103C	RTG3	YOR208W	PTP2
YDR137W	RGP1	YKL048C	ELM1
YBR131W	CCZ1	YKR020W	VPS51
YBR107C	IML3	YNL116W	DMA2
YGR014W	MSB2	YDR363W-A	SEM1
YBR009C	HHF1	YOR038C	HIR2
YOL126C	MDH2	YMR273C	ZDS1
YJR005W	APL1	YKL114C	APN1
YFL044C	OTU1	YPR149W	NCE102
YNR032W	PPG1	YOL108C	INO4
YPL057C	SUR1	YDR318W	MCM21
YMR133W	REC114	YDL077C	VAM6
YGL078C	DBP3	YMR230W	RPS10B
YDL116W	NUP84	YGL200C	EMP24
YBR279W	PAF1	YGL094C	PAN2
YHR012W	VPS29	YDR289C	RTT103
YPL161C	BEM4	YOL018C	TLG2
YKL116C	PRR1	YML001W	YPT7
YDR155C	CPR1	YEL056W	HAT2
YGR105W	VMA21	YDL077C	VAM6
YER161C	SPT2	YNL107W	YAF9
YPL234C	TFP3	YKL041W	VPS24
YER124C	DSE1	YBL085W	BOI1
YNL078W	NIS1	YBL085W	BOI1
YMR026C	PEX12	YKL126W	YPK1
YNL288W	CAF40	YPR046W	MCM16

Predictions at threshold level 0.75 continued...			
Gene1 of predicted pair		Gene2 of predicted pair	
Systematic Name	Standard Name	Systematic Name	Standard Name
YOR069W	VPS5	YML001W	YPT7
YDR293C	SSD1	YPR046W	MCM16
YDL155W	CLB3	YAR014C	BUD14
YJL183W	MNN11	YMR311C	GLC8
YNL127W	FAR11	YJR033C	RAV1
YOR023C	AHC1	YPR046W	MCM16
YKR061W	KTR2	YIL103W	DPH1
YBR030W	YBR030W	YOR066W	MSA1
YML115C	VAN1	YLR093C	NYV1
YKR059W	TIF1	YML115C	VAN1
YMR198W	CIK1	YDL155W	CLB3
YFR010W	UBP6	YLR315W	NKP2
YGR217W	CCH1	YLR371W	ROM2
YPL161C	BEM4	YGL253W	HXK2
YLR371W	ROM2	YOR327C	SNC2
YGL033W	HOP2	YEL053C	MAK10
YEL046C	GLY1	YPL249C	GYP5
YER179W	DMC1	YGR184C	UBR1
YBL025W	RRN10	YOL081W	IRA2
YGL115W	SNF4	YNL238W	KEX2
YIL159W	BNR1	YMR311C	GLC8
YIL159W	BNR1	YER122C	GLO3
YIL159W	BNR1	YLR370C	ARC18
YNL068C	FKH2	YKR048C	NAP1
YIL076W	SEC28	YBR036C	CSG2
YBR274W	CHK1	YGL151W	NUT1
YLR018C	POM34	YLR131C	ACE2
YMR080C	NAM7	YPL024W	RMI1
YPL213W	LEA1	YBL072C	RPS8A

Predictions at threshold level 0.75 continued...			
Gene1 of predicted pair		Gene2 of predicted pair	
Systematic Name	Standard Name	Systematic Name	Standard Name
YLR315W	NKP2	YER122C	GLO3
YKL139W	CTK1	YCL037C	SRO9
YNL288W	CAF40	YGL253W	HXK2
YLR234W	TOP3	YMR116C	ASC1
YFR036W	CDC26	YLR032W	RAD5
YDL161W	ENT1	YGL095C	VPS45
YOR033C	EXO1	YOR080W	DIA2
YDR156W	RPA14	YPL253C	VIK1
YOR270C	VPH1	YLR447C	VMA6
YPL161C	BEM4	YKR030W	GMH1
YBR041W	FAT1	YLR320W	MMS22
YPL129W	TAF14	YDL002C	NHP10
YGL147C	RPL9A	YGR249W	MGA1
YBR194W	SOY1	YJL124C	LSM1
YDR363W-A	SEM1	YPL018W	CTF19
YDL020C	RPN4	YOL051W	GAL11
YDL184C	RPL41A	YJL172W	CPS1
YNL199C	GCR2	YEL013W	VAC8
YDR383C	NKP1	YHR013C	ARD1
YPR018W	RLF2	YNL236W	SIN4
YPL022W	RAD1	YBR215W	HPC2
YOR132W	VPS17	YNL238W	KEX2
YIR003W	YIR003W	YKL204W	EAP1
YKL110C	KTI12	YML112W	CTK3
YDR484W	VPS52	YBR171W	SEC66
YNL076W	MKS1	YNL082W	PMS1
YBL102W	SFT2	YDR320C	SWA2
YMR224C	MRE11	YJL006C	CTK2
YDR004W	RAD57	YPR131C	NAT3

Predictions at threshold level 0.75 continued...			
Gene1 of predicted pair		Gene2 of predicted pair	
Systematic Name	Standard Name	Systematic Name	Standard Name
YKL048C	ELM1	YOR043W	WHI2
YLR386W	VAC14	YGR188C	BUB1
YBR030W	YBR030W	YDR484W	VPS52
YBR108W	YBR108W	YPR036W	VMA13
YGL087C	MMS2	YNL307C	MCK1
YKL010C	UFD4	YBR082C	UBC4
YPL001W	HAT1	YEL056W	HAT2
YEL043W	YEL043W	YGL054C	ERV14
YKL048C	ELM1	YJR063W	RPA12
YOR156C	NFI1	YDR440W	DOT1
YLR192C	HCR1	YNL076W	MKS1
YPL234C	TFP3	YDR310C	SUM1
YIR005W	IST3	YLR350W	ORM2
YJL004C	SYS1	YAL002W	VPS8
YMR224C	MRE11	YLR135W	SLX4
YLR234W	TOP3	YLR233C	EST1
YKL048C	ELM1	YMR038C	CCS1
YKL160W	ELF1	YHR108W	GGA2
YKL048C	ELM1	YGR054W	YGR054W
YBR108W	YBR108W	YER162C	RAD4
YLR371W	ROM2	YJR084W	CSN12
YJR040W	GEF1	YER120W	SCS2
YOR195W	SLK19	YNL229C	URE2
YDR129C	SAC6	YML016C	PPZ1
YFR031C-A	RPL2A	YHR012W	VPS29
YDR477W	SNF1	YPL037C	EGD1
YKL006W	RPL14A	YMR116C	ASC1
YOR043W	WHI2	YPL158C	YPL158C
YLR337C	VRP1	YGL237C	HAP2

Predictions at threshold level 0.75 continued...			
Gene1 of predicted pair		Gene2 of predicted pair	
Systematic Name	Standard Name	Systematic Name	Standard Name
YAL024C	LTE1	YMR224C	MRE11
YLR337C	VRP1	YLR233C	EST1
YML095C	RAD10	YPR120C	CLB5
YGR135W	PRE9	YDR440W	DOT1
YLR422W	YLR422W	YOR344C	TYE7
YJR063W	RPA12	YLR315W	NKP2
YJR063W	RPA12	YNL225C	CNM67
YDL184C	RPL41A	YML071C	COG8
YMR152W	YIM1	YLR370C	ARC18
YER019C-A	SBH2	YOR085W	OST3
YPL022W	RAD1	YBR114W	RAD16
YML095C	RAD10	YNL236W	SIN4
YCL037C	SRO9	YOR109W	INP53
YKR072C	SIS2	YBR228W	SLX1
YMR273C	ZDS1	YKL068W	NUP100
YBR030W	YBR030W	YDL192W	ARF1
YBR173C	UMP1	YLR370C	ARC18
YHR178W	STB5	YOR127W	RGA1
YDR156W	RPA14	YPR046W	MCM16
YLR095C	IOC2	YNL238W	KEX2
YDR137W	RGP1	YBR036C	CSG2
YLR320W	MMS22	YER139C	YER139C
YMR092C	AIP1	YLR429W	CRN1
YJL106W	IME2	YDR207C	UME6
YML016C	PPZ1	YDR310C	SUM1
YDR202C	RAV2	YGL223C	COG1
YNL032W	SIW14	YGL003C	CDH1
YPR017C	DSS4	YNL238W	KEX2
YGL200C	EMP24	YGL054C	ERV14

Predictions at threshold level 0.75 continued...			
Gene1 of predicted pair		Gene2 of predicted pair	
Systematic Name	Standard Name	Systematic Name	Standard Name
YIL076W	SEC28	YHL031C	GOS1
YNL031C	HHT2	YBR289W	SNF5
YDR490C	PKH1	YPL004C	LSP1
YLR083C	EMP70	YGR072W	UPF3
YGL212W	VAM7	YCR066W	RAD18
YKR035W-A	DID2	YBR280C	SAF1
YPL042C	SSN3	YIL038C	NOT3
YBL102W	SFT2	YKR030W	GMH1
YML001W	YPT7	YDR207C	UME6
YGR081C	SLX9	YKL068W	NUP100
YNL093W	YPT53	YKR014C	YPT52
YMR198W	CIK1	YGL124C	MON1
YBL058W	SHP1	YKL126W	YPK1
YCL037C	SRO9	YDR310C	SUM1
YKL025C	PAN3	YLR292C	SEC72
YDR293C	SSD1	YHR134W	WSS1
YHL031C	GOS1	YPL161C	BEM4
YGR071C	YGR071C	YJR135C	MCM22
YDR469W	SDC1	YLR357W	RSC2
YKL080W	VMA5	YKL119C	VPH2
YDR004W	RAD57	YGL066W	SGF73
YGR261C	APL6	YJR040W	GEF1
YDL223C	HBT1	YEL060C	PRB1
YJL060W	BNA3	YLR381W	CTF3
YIL159W	BNR1	YMR133W	REC114
YJL172W	CPS1	YER044C	ERG28
YBR217W	ATG12	YDR310C	SUM1
YOR096W	RPS7A	YHR203C	RPS4B
YDR484W	VPS52	YDL077C	VAM6

Predictions at threshold level 0.75 continued...			
**Gene1 of predicted pair**		**Gene2 of predicted pair**	
**Systematic Name**	**Standard Name**	**Systematic Name**	**Standard Name**
YCL037C	SRO9	YDL006W	PTC1
YOL043C	NTG2	YAL015C	NTG1
YLR386W	VAC14	YDR484W	VPS52
YGL087C	MMS2	YBL047C	EDE1
YLR360W	VPS38	YGR105W	VMA21
YDR155C	CPR1	YJL190C	RPS22A
YOL111C	MDY2	YER122C	GLO3
YMR153W	NUP53	YDL192W	ARF1
YNL051W	COG5	YLR309C	IMH1
YEL043W	YEL043W	YHR012W	VPS29
YMR095C	SNO1	YOR327C	SNC2
YLR429W	CRN1	YDR318W	MCM21
YMR125W	STO1	YGR059W	SPR3
YDR146C	SWI5	YDL002C	NHP10
YNL199C	GCR2	YER161C	SPT2
YNL288W	CAF40	YER068W	MOT2
YJL124C	LSM1	YHR167W	THP2
YBR200W	BEM1	YKL101W	HSL1
YGR003W	CUL3	YJR099W	YUH1
YER115C	SPR6	YER110C	KAP123
YEL053C	MAK10	YPR046W	MCM16
YOR106W	VAM3	YDR456W	NHX1
YNR052C	POP2	YJL006C	CTK2
YIL076W	SEC28	YPR030W	CSR2
YPR131C	NAT3	YGL175C	SAE2
YIL034C	CAP2	YMR109W	MYO5
YOR127W	RGA1	YHR200W	RPN10
YNL297C	MON2	YDL006W	PTC1
YEL053C	MAK10	YGR105W	VMA21

Predictions at threshold level 0.75 continued...			
Gene1 of predicted pair		Gene2 of predicted pair	
Systematic Name	Standard Name	Systematic Name	Standard Name
YDR171W	HSP42	YJL006C	CTK2
YIL076W	SEC28	YMR054W	STV1
YBL102W	SFT2	YCL001W	RER1
YOR106W	VAM3	YNL297C	MON2
YDR320C	SWA2	YJL053W	PEP8
YBL003C	HTA2	YLR357W	RSC2
YNL289W	PCL1	YPR119W	CLB2
YDR383C	NKP1	YJR135C	MCM22
YHR039C-A	VMA10	YLR371W	ROM2
YKR020W	VPS51	YLR371W	ROM2
YDR144C	MKC7	YDR155C	CPR1
YOR195W	SLK19	YJR060W	CBF1
YMR231W	PEP5	YMR038C	CCS1
YGL175C	SAE2	YLR032W	RAD5
YNL271C	BNI1	YEL022W	GEA2
YOR156C	NFI1	YKR095W	MLP1
YNL199C	GCR2	YML057W	CMP2
YGR184C	UBR1	YGR188C	BUB1
YDR289C	RTT103	YHL002W	HSE1
YML115C	VAN1	YNL325C	FIG4
YPL024W	RMI1	YMR026C	PEX12
YDL083C	RPS16B	YMR311C	GLC8
YKR099W	BAS1	YEL060C	PRB1
YDR389W	SAC7	YHL031C	GOS1
YNL044W	YIP3	YML012W	ERV25
YDL184C	RPL41A	YJL183W	MNN11
YPL161C	BEM4	YDR264C	AKR1
YMR224C	MRE11	YBR200W	BEM1
YPL161C	BEM4	YMR133W	REC114

Predictions at threshold level 0.75 continued...			
Gene1 of predicted pair		Gene2 of predicted pair	
Systematic Name	Standard Name	Systematic Name	Standard Name
YOR033C	EXO1	YMR167W	MLH1
YHR156C	LIN1	YBL103C	RTG3
YCR086W	CSM1	YJR035W	RAD26
YOR275C	RIM20	YGL031C	RPL24A
YMR216C	SKY1	YDL006W	PTC1
YLR095C	IOC2	YDR261C	EXG2
YNL069C	RPL16B	YKR020W	VPS51
YIL045W	PIG2	YAR014C	BUD14
YHR060W	VMA22	YKR030W	GMH1
YKL032C	IXR1	YHR200W	RPN10
YKL117W	SBA1	YPL246C	RBD2
YNL307C	MCK1	YLR320W	MMS22
YOR290C	SNF2	YKR099W	BAS1
YJL176C	SWI3	YGR133W	PEX4
YDR254W	CHL4	YDL155W	CLB3
YCR066W	RAD18	YDR310C	SUM1
YIL159W	BNR1	YLR398C	SKI2
YOR140W	SFL1	YDR456W	NHX1
YOR195W	SLK19	YKL032C	IXR1
YEL043W	YEL043W	YDR153C	ENT5
YDR137W	RGP1	YKL213C	DOA1
YFL023W	BUD27	YHR200W	RPN10
YHR103W	SBE22	YPR119W	CLB2
YJL036W	SNX4	YJR033C	RAV1
YOR127W	RGA1	YDR108W	GSG1
YMR153W	NUP53	YEL053C	MAK10
YDL116W	NUP84	YJR090C	GRR1
YNL293W	MSB3	YOR327C	SNC2
YFR036W	CDC26	YJR084W	CSN12

Predictions at threshold level 0.75 continued...			
Gene1 of predicted pair		Gene2 of predicted pair	
Systematic Name	Standard Name	Systematic Name	Standard Name
YKL048C	ELM1	YML001W	YPT7
YMR224C	MRE11	YGL124C	MON1
YFL033C	RIM15	YNR032W	PPG1
YKL007W	CAP1	YBR200W	BEM1
YLR093C	NYV1	YCR053W	THR4
YDL020C	RPN4	YJL154C	VPS35
YBR171W	SEC66	YDL155W	CLB3
YBL103C	RTG3	YGL025C	PGD1
YLR234W	TOP3	YEL056W	HAT2
YPL253C	VIK1	YGL124C	MON1
YOL121C	RPS19A	YDR207C	UME6
YLL007C	YLL007C	YMR109W	MYO5
YMR133W	REC114	YLR180W	SAM1
YMR055C	BUB2	YGL031C	RPL24A
YGL200C	EMP24	YKR020W	VPS51
YOR337W	TEA1	YLR180W	SAM1
YGL115W	SNF4	YPL120W	VPS30
YKR001C	VPS1	YDR153C	ENT5
YLR191W	PEX13	YAL055W	PEX22
YPR029C	APL4	YDR358W	GGA1
YJL176C	SWI3	YMR116C	ASC1
YMR198W	CIK1	YMR055C	BUB2
YLR452C	SST2	YBR019C	GAL10
YML115C	VAN1	YPL234C	TFP3
YBR126C	TPS1	YLR452C	SST2
YBR030W	YBR030W	YMR153W	NUP53
YLL039C	UBI4	YPL161C	BEM4
YPR131C	NAT3	YKR072C	SIS2
YGR261C	APL6	YMR054W	STV1

Predictions at threshold level 0.75 continued...			
Gene1 of predicted pair		Gene2 of predicted pair	
Systematic Name	Standard Name	Systematic Name	Standard Name
YNL329C	PEX6	YJL095W	BCK1
YBL051C	PIN4	YPL018W	CTF19
YER032W	FIR1	YHR152W	SPO12
YBR126C	TPS1	YML100W	TSL1
YCL001W	RER1	YJR060W	CBF1
YKL068W	NUP100	YBR010W	HHT1
YHL031C	GOS1	YER120W	SCS2
YPL158C	YPL158C	YLR425W	TUS1
YML016C	PPZ1	YAR014C	BUD14
YDR155C	CPR1	YJL183W	MNN11
YIL159W	BNR1	YPR036W	VMA13
YKR020W	VPS51	YDL226C	GCS1
YKL116C	PRR1	YOL121C	RPS19A
YDR383C	NKP1	YPR046W	MCM16
YPR120C	CLB5	YIR005W	IST3
YDR254W	CHL4	YIL103W	DPH1
YJR135C	MCM22	YOL054W	PSH1
YHR200W	RPN10	YBR036C	CSG2
YLR456W	YLR456W	YER032W	FIR1
YOR069W	VPS5	YKR001C	VPS1
YDL006W	PTC1	YPR032W	SRO7
YOL013C	HRD1	YLR207W	HRD3
YLR192C	HCR1	YJR056C	YJR056C
YFR010W	UBP6	YJL095W	BCK1
YFL013C	IES1	YDR335W	MSN5
YBR290W	BSD2	YNL107W	YAF9
YPL024W	RMI1	YOR270C	VPH1
YER116C	SLX8	YNR072W	HXT17
YOR066W	MSA1	YNL076W	MKS1

Predictions at threshold level 0.75 continued...			
Gene1 of predicted pair		Gene2 of predicted pair	
Systematic Name	Standard Name	Systematic Name	Standard Name
YMR055C	BUB2	YAL002W	VPS8
YGR100W	MDR1	YGL198W	YIP4
YHL031C	GOS1	YLR309C	IMH1
YNL076W	MKS1	YMR230W	RPS10B
YMR198W	CIK1	YPL022W	RAD1
YDR477W	SNF1	YOR327C	SNC2
YOR270C	VPH1	YFL004W	VTC2
YHR152W	SPO12	YHR111W	UBA4
YBL088C	TEL1	YPR120C	CLB5
YPL161C	BEM4	YOR141C	ARP8
YMR133W	REC114	YOR066W	MSA1
YBL051C	PIN4	YKR020W	VPS51
YIL148W	RPL40A	YER115C	SPR6
YAL024C	LTE1	YIL053W	RHR2
YDR484W	VPS52	YNL307C	MCK1
YLR381W	CTF3	YFR036W	CDC26
YDL020C	RPN4	YEL027W	CUP5
YFL023W	BUD27	YLR452C	SST2
YDR358W	GGA1	YPL195W	APL5
YNL325C	FIG4	YER162C	RAD4
YBR084C-A	RPL19A	YGL246C	RAI1
YOR127W	RGA1	YNL127W	FAR11
YJR060W	CBF1	YPL120W	VPS30
YGL200C	EMP24	YDL155W	CLB3
YMR116C	ASC1	YGR217W	CCH1
YLR399C	BDF1	YJR135C	MCM22
YAL024C	LTE1	YOR290C	SNF2
YOR109W	INP53	YNR032W	PPG1
YFR008W	FAR7	YDR200C	VPS64

Predictions at threshold level 0.75 continued...			
Gene1 of predicted pair		Gene2 of predicted pair	
Systematic Name	Standard Name	Systematic Name	Standard Name
YDR456W	NHX1	YER096W	SHC1
YPR030W	CSR2	YML071C	COG8
YPR036W	VMA13	YJR084W	CSN12
YHL002W	HSE1	YDR310C	SUM1
YML038C	YMD8	YNL238W	KEX2
YLR234W	TOP3	YOR001W	RRP6
YGL163C	RAD54	YKL068W	NUP100
YOR001W	RRP6	YAL002W	VPS8
YBR289W	SNF5	YJL140W	RPB4
YHR156C	LIN1	YLR094C	GIS3
YDR439W	LRS4	YGR270W	YTA7
YNL215W	IES2	YBR278W	DPB3
YBR290W	BSD2	YMR153W	NUP53
YHR152W	SPO12	YKL048C	ELM1
YNR032W	PPG1	YPR032W	SRO7
YJL053W	PEP8	YOR109W	INP53
YEL043W	YEL043W	YAL002W	VPS8
YOR337W	TEA1	YDL184C	RPL41A
YCL073C	YCL073C	YPL240C	HSP82
YLR061W	RPL22A	YOR035C	SHE4
YHL031C	GOS1	YOR308C	SNU66
YHR073W	OSH3	YML057W	CMP2
YOR270C	VPH1	YMR230W	RPS10B
YLR396C	VPS33	YLR093C	NYV1
YER044C	ERG28	YJR084W	CSN12
YGL215W	CLG1	YDR385W	EFT2
YOR185C	GSP2	YGL016W	KAP122
YLR381W	CTF3	YLR150W	STM1
YLR337C	VRP1	YLL039C	UBI4

Predictions at threshold level 0.75 continued...			
Gene1 of predicted pair		Gene2 of predicted pair	
Systematic Name	Standard Name	Systematic Name	Standard Name
YKL048C	ELM1	YBR082C	UBC4
YBL058W	SHP1	YER139C	YER139C
YOR033C	EXO1	YOR368W	RAD17
YNL044W	YIP3	YML071C	COG8
YIL008W	URM1	YKR048C	NAP1
YDR477W	SNF1	YOL145C	CTR9
YNL248C	RPA49	YDR318W	MCM21
YGL124C	MON1	YLR370C	ARC18
YLR234W	TOP3	YNL307C	MCK1
YNL215W	IES2	YNL054W	VAC7
YBR194W	SOY1	YDR378C	LSM6
YNL016W	PUB1	YML028W	TSA1
YBR200W	BEM1	YPR036W	VMA13
YNL218W	MGS1	YML028W	TSA1
YHR081W	LRP1	YDR358W	GGA1
YBL051C	PIN4	YOR327C	SNC2
YLR337C	VRP1	YJL006C	CTK2
YMR153W	NUP53	YJL172W	CPS1
YLR386W	VAC14	YNL229C	URE2
YDR335W	MSN5	YPL018W	CTF19
YPL106C	SSE1	YDL226C	GCS1
YJL151C	SNA3	YMR253C	YMR253C
YFL033C	RIM15	YAR014C	BUD14
YGL078C	DBP3	YBL103C	RTG3
YDR378C	LSM6	YJR063W	RPA12
YMR284W	YKU70	YMR038C	CCS1
YMR224C	MRE11	YKL068W	NUP100
YPL253C	VIK1	YOR141C	ARP8
YDR245W	MNN10	YGL174W	BUD13

Predictions at threshold level 0.75 continued...			
Gene1 of predicted pair		Gene2 of predicted pair	
Systematic Name	Standard Name	Systematic Name	Standard Name
YGL212W	VAM7	YBL072C	RPS8A
YCR039C	MATALPHA2	YDR451C	YHP1
YLR360W	VPS38	YEL043W	YEL043W
YPL042C	SSN3	YOR001W	RRP6
YBL051C	PIN4	YCR086W	CSM1
YDR318W	MCM21	YOR141C	ARP8
YPL024W	RMI1	YLR288C	MEC3
YIL159W	BNR1	YOR290C	SNF2
YPL115C	BEM3	YKL048C	ELM1
YML115C	VAN1	YBL072C	RPS8A
YLR447C	VMA6	YNL107W	YAF9
YDL101C	DUN1	YJR035W	RAD26
YBR200W	BEM1	YOR014W	RTS1
YHR171W	ATG7	YOR106W	VAM3
YML095C	RAD10	YPL256C	CLN2
YDR477W	SNF1	YOR290C	SNF2
YDL184C	RPL41A	YDR261C	EXG2
YDR129C	SAC6	YGL153W	PEX14
YOR001W	RRP6	YLR315W	NKP2
YDR320C	SWA2	YJL036W	SNX4
YGL033W	HOP2	YJR032W	CPR7
YMR095C	SNO1	YDR484W	VPS52
YDL020C	RPN4	YBR114W	RAD16
YLR292C	SEC72	YOL067C	RTG1
YNL297C	MON2	YOR327C	SNC2
YMR116C	ASC1	YOR035C	SHE4
YGL087C	MMS2	YML016C	PPZ1
YBL008W	HIR1	YJL138C	TIF2
YLR360W	VPS38	YOR014W	RTS1

Predictions at threshold level 0.75 continued...			
Gene1 of predicted pair		Gene2 of predicted pair	
Systematic Name	Standard Name	Systematic Name	Standard Name
YDR076W	RAD55	YOR368W	RAD17
YPL024W	RMI1	YOR032C	HMS1
YHR010W	RPL27A	YDR207C	UME6
YDL020C	RPN4	YFR010W	UBP6
YOR109W	INP53	YKR020W	VPS51
YJL154C	VPS35	YOR132W	VPS17
YMR039C	SUB1	YOR038C	HIR2
YML115C	VAN1	YER122C	GLO3
YBR274W	CHK1	YKL101W	HSL1
YPL213W	LEA1	YOL081W	IRA2
YNL243W	SLA2	YLR039C	RIC1
YJL154C	VPS35	YJL029C	VPS53
YEL053C	MAK10	YDL155W	CLB3
YLR090W	XDJ1	YLR452C	SST2
YJL204C	RCY1	YDR139C	RUB1
YMR224C	MRE11	YDL059C	RAD59
YLR095C	IOC2	YGL078C	DBP3
YJL136C	RPS21B	YHR115C	DMA1
YHL031C	GOS1	YDR207C	UME6
YBR126C	TPS1	YOR337W	TEA1
YPL018W	CTF19	YGL124C	MON1
YNL032W	SIW14	YLR452C	SST2
YML115C	VAN1	YDR146C	SWI5
YOL030W	GAS5	YDL013W	SLX5
YOL111C	MDY2	YDR310C	SUM1
YLR006C	SSK1	YBR171W	SEC66
YEL060C	PRB1	YER040W	GLN3
YNL127W	FAR11	YDR207C	UME6
YDR383C	NKP1	YPR120C	CLB5

Predictions at threshold level 0.75 continued...			
Gene1 of predicted pair		Gene2 of predicted pair	
Systematic Name	Standard Name	Systematic Name	Standard Name
YBR228W	SLX1	YMR038C	CCS1
YDL116W	NUP84	YPL125W	KAP120
YPL253C	VIK1	YDR144C	MKC7
YBL021C	HAP3	YIL008W	URM1
YBR274W	CHK1	YDL002C	NHP10
YPR036W	VMA13	YKR035W-A	DID2
YLR119W	SRN2	YBL103C	RTG3
YIL076W	SEC28	YML001W	YPT7
YPL042C	SSN3	YPL253C	VIK1
YNL025C	SSN8	YOL051W	GAL11
YGR249W	MGA1	YFL023W	BUD27
YJR060W	CBF1	YDR310C	SUM1
YLR386W	VAC14	YDR379W	RGA2
YKR020W	VPS51	YOR035C	SHE4
YBL008W	HIR1	YGL250W	RMR1
YIL008W	URM1	YJL183W	MNN11
YPL042C	SSN3	YGR108W	CLB1
YAL024C	LTE1	YBR215W	HPC2
YEL053C	MAK10	YER162C	RAD4
YDL078C	MDH3	YPR119W	CLB2
YNL236W	SIN4	YJL140W	RPB4
YIL076W	SEC28	YLR371W	ROM2
YDR108W	GSG1	YPL105C	YPL105C
YPL253C	VIK1	YDR254W	CHL4
YJL190C	RPS22A	YDR310C	SUM1
YBR200W	BEM1	YPL106C	SSE1
YJL172W	CPS1	YDR310C	SUM1
YGL115W	SNF4	YJL095W	BCK1
YBR030W	YBR030W	YPR036W	VMA13

Predictions at threshold level 0.75 continued...			
Gene1 of predicted pair		Gene2 of predicted pair	
Systematic Name	Standard Name	Systematic Name	Standard Name
YPR051W	MAK3	YKR001C	VPS1
YGL078C	DBP3	YNL058C	YNL058C
YOR312C	RPL20B	YLR370C	ARC18
YNL199C	GCR2	YPR119W	CLB2
YLR381W	CTF3	YLR452C	SST2
YBR173C	UMP1	YER122C	GLO3
YML115C	VAN1	YGR105W	VMA21
YMR224C	MRE11	YLR180W	SAM1
YBR195C	MSI1	YDR289C	RTT103
YBL008W	HIR1	YBR010W	HHT1
YLR119W	SRN2	YLR093C	NYV1
YER096W	SHC1	YLR371W	ROM2
YIR025W	MND2	YLR102C	APC9
YMR319C	FET4	YKR093W	PTR2
YOR227W	YOR227W	YPR119W	CLB2
YGR020C	VMA7	YDL006W	PTC1
YPL042C	SSN3	YDR440W	DOT1
YDR378C	LSM6	YJL006C	CTK2
YMR153W	NUP53	YJL095W	BCK1
YOR080W	DIA2	YLR381W	CTF3
YJL036W	SNX4	YDR202C	RAV2
YNR072W	HXT17	YLR093C	NYV1
YER162C	RAD4	YJR084W	CSN12
YDR156W	RPA14	YDR254W	CHL4
YCL010C	SGF29	YBR010W	HHT1
YKL025C	PAN3	YJR135C	MCM22
YMR054W	STV1	YPR032W	SRO7
YMR179W	SPT21	YDR207C	UME6
YPL152W	RRD2	YFR036W	CDC26

Predictions at threshold level 0.75 continued...			
Gene1 of predicted pair		Gene2 of predicted pair	
Systematic Name	Standard Name	Systematic Name	Standard Name
YPR131C	NAT3	YLR352W	YLR352W
YCL001W	RER1	YPL227C	ALG5
YDR225W	HTA1	YKR048C	NAP1
YKL010C	UFD4	YDR363W-A	SEM1
YOL111C	MDY2	YLR093C	NYV1
YLR288C	MEC3	YBR278W	DPB3
YLR337C	VRP1	YEL053C	MAK10
YBR200W	BEM1	YGR105W	VMA21
YOR127W	RGA1	YHR158C	KEL1
YBL058W	SHP1	YDR358W	GGA1
YDR383C	NKP1	YLR079W	SIC1
YPR017C	DSS4	YJR040W	GEF1
YLR360W	VPS38	YDR310C	SUM1
YNL076W	MKS1	YMR214W	SCJ1
YGL151W	NUT1	YNL107W	YAF9
YGL212W	VAM7	YEL053C	MAK10
YGL025C	PGD1	YNL041C	COG6
YPL042C	SSN3	YDR155C	CPR1
YOR080W	DIA2	YPL106C	SSE1
YNL333W	SNZ2	YAR014C	BUD14
YMR224C	MRE11	YDR363W-A	SEM1
YOR195W	SLK19	YAR014C	BUD14
YFR010W	UBP6	YPR046W	MCM16
YOR069W	VPS5	YDL077C	VAM6
YER059W	PCL6	YPR119W	CLB2
YBR274W	CHK1	YDR310C	SUM1
YBR041W	FAT1	YJL095W	BCK1
YDL227C	HO	YPR119W	CLB2
YKL139W	CTK1	YIL036W	CST6

Predictions at threshold level 0.75 continued...			
Gene1 of predicted pair		Gene2 of predicted pair	
Systematic Name	Standard Name	Systematic Name	Standard Name
YHR013C	ARD1	YML112W	CTK3
YKL010C	UFD4	YKR020W	VPS51
YGL087C	MMS2	YLR234W	TOP3
YLR309C	IMH1	YNL107W	YAF9
YKR099W	BAS1	YOL111C	MDY2
YOL018C	TLG2	YKR030W	GMH1
YGR020C	VMA7	YEL027W	CUP5
YMR199W	CLN1	YKL068W	NUP100
YDL116W	NUP84	YAR014C	BUD14
YKL214C	YRA2	YNL139C	RLR1
YFR010W	UBP6	YKL160W	ELF1
YPL024W	RMI1	YPL079W	RPL21B
YJL136C	RPS21B	YBL087C	RPL23A
YOR332W	VMA4	YOR270C	VPH1
YIL159W	BNR1	YPL137C	GIP3
YMR116C	ASC1	YOR275C	RIM20
YNL238W	KEX2	YER139C	YER139C
YKL010C	UFD4	YPL248C	GAL4
YKR020W	VPS51	YNL116W	DMA2
YDR383C	NKP1	YGL252C	RTG2
YDL185W	TFP1	YNL147W	LSM7
YER087C-B	SBH1	YLR226W	BUR2
YHR013C	ARD1	YOR312C	RPL20B
YNL201C	PSY2	YDR183W	PLP1
YLR360W	VPS38	YMR216C	SKY1
YGL212W	VAM7	YPL137C	GIP3
YLR090W	XDJ1	YKL139W	CTK1
YJR099W	YUH1	YML097C	VPS9
YOR358W	HAP5	YDR155C	CPR1

Predictions at threshold level 0.75 continued...			
Gene1 of predicted pair		Gene2 of predicted pair	
Systematic Name	Standard Name	Systematic Name	Standard Name
YBR171W	SEC66	YOL067C	RTG1
YMR026C	PEX12	YLR180W	SAM1
YAL024C	LTE1	YNL069C	RPL16B
YJL095W	BCK1	YDL192W	ARF1
YPL161C	BEM4	YNL107W	YAF9
YOR132W	VPS17	YPL259C	APM1
YGL180W	ATG1	YLR423C	ATG17
YLR422W	YLR422W	YOL108C	INO4
YBR173C	UMP1	YNL058C	YNL058C
YJR063W	RPA12	YPL018W	CTF19
YOR076C	SKI7	YPL079W	RPL21B
YER054C	GIP2	YOL091W	SPO21
YOR069W	VPS5	YNR032W	PPG1
YDR318W	MCM21	YHL002W	HSE1
YPL057C	SUR1	YBR171W	SEC66
YDR129C	SAC6	YPL234C	TFP3
YGL087C	MMS2	YML095C	RAD10
YBR173C	UMP1	YLR093C	NYV1
YCR063W	BUD31	YBR188C	NTC20
YER116C	SLX8	YML028W	TSA1
YER139C	YER139C	YAL002W	VPS8
YMR284W	YKU70	YJL187C	SWE1
YIL034C	CAP2	YHR134W	WSS1
YFR010W	UBP6	YER044C	ERG28
YMR054W	STV1	YDL077C	VAM6
YJL060W	BNA3	YBR107C	IML3
YER116C	SLX8	YGR188C	BUB1
YGL033W	HOP2	YLR094C	GIS3
YHR156C	LIN1	YMR142C	RPL13B

Predictions at threshold level 0.75 continued...			
Gene1 of predicted pair		Gene2 of predicted pair	
Systematic Name	Standard Name	Systematic Name	Standard Name
YLR337C	VRP1	YJL183W	MNN11
YGR104C	SRB5	YCR084C	TUP1
YIL034C	CAP2	YOR208W	PTP2
YOR038C	HIR2	YDR073W	SNF11
YNL291C	MID1	YML001W	YPT7
YLR090W	XDJ1	YER162C	RAD4
YGR108W	CLB1	YJR090C	GRR1
YBL058W	SHP1	YNL041C	COG6
YLR337C	VRP1	YOL081W	IRA2
YGL212W	VAM7	YBR036C	CSG2
YML115C	VAN1	YNL199C	GCR2
YDR507C	GIN4	YOR275C	RIM20
YEL013W	VAC8	YOR124C	UBP2
YGR072W	UPF3	YLR309C	IMH1
YMR109W	MYO5	YOL067C	RTG1
YKL079W	SMY1	YEL022W	GEA2
YDR443C	SSN2	YHR012W	VPS29
YOL126C	MDH2	YKR024C	DBP7
YDR363W-A	SEM1	YLR398C	SKI2
YBR200W	BEM1	YLR398C	SKI2
YBL102W	SFT2	YDL077C	VAM6
YIL053W	RHR2	YNL058C	YNL058C
YCR086W	CSM1	YJL183W	MNN11
YKL048C	ELM1	YKR020W	VPS51
YHR111W	UBA4	YGL163C	RAD54
YLR362W	STE11	YER118C	SHO1
YGL153W	PEX14	YAL055W	PEX22
YGR054W	YGR054W	YAL002W	VPS8
YMR095C	SNO1	YPL057C	SUR1

Predictions at threshold level 0.75 continued...			
Gene1 of predicted pair		Gene2 of predicted pair	
Systematic Name	Standard Name	Systematic Name	Standard Name
YBR084C-A	RPL19A	YOR275C	RIM20
YDL083C	RPS16B	YOL121C	RPS19A
YLR090W	XDJ1	YHR200W	RPN10
YDR137W	RGP1	YDR363W-A	SEM1
YGL066W	SGF73	YLR381W	CTF3
YLR360W	VPS38	YBR082C	UBC4
YPR084W	YPR084W	YGL054C	ERV14
YJR060W	CBF1	YPL195W	APL5
YJR024C	MDE1	YIL156W	UBP7
YGL063W	PUS2	YPL240C	HSP82
YDR335W	MSN5	YDL047W	SIT4
YOR069W	VPS5	YCL001W	RER1
YGL212W	VAM7	YER122C	GLO3
YCR034W	FEN1	YLR234W	TOP3
YMR153W	NUP53	YPR030W	CSR2
YOR140W	SFL1	YPL253C	VIK1
YMR016C	SOK2	YHR200W	RPN10
YDR293C	SSD1	YPL253C	VIK1
YPR051W	MAK3	YNR032W	PPG1
YOR033C	EXO1	YNL307C	MCK1
YDR443C	SSN2	YOR035C	SHE4
YLR234W	TOP3	YPL256C	CLN2
YMR224C	MRE11	YGL025C	PGD1
YJR021C	REC107	YKL074C	MUD2
YOR337W	TEA1	YLR452C	SST2
YFR010W	UBP6	YGR249W	MGA1
YOR043W	WHI2	YPL137C	GIP3
YNL215W	IES2	YNL248C	RPA49
YDR137W	RGP1	YLR350W	ORM2

Predictions at threshold level 0.75 continued...			
Gene1 of predicted pair		Gene2 of predicted pair	
Systematic Name	Standard Name	Systematic Name	Standard Name
YGR105W	VMA21	YAR014C	BUD14
YCL014W	BUD3	YLR219W	MSC3
YML001W	YPT7	YGL124C	MON1
YNL206C	RTT106	YDR207C	UME6
YCR086W	CSM1	YML112W	CTK3
YIR005W	IST3	YOL054W	PSH1
YNL058C	YNL058C	YBR280C	SAF1
YNL307C	MCK1	YHR115C	DMA1
YLR191W	PEX13	YGR077C	PEX8
YDR260C	SWM1	YFR036W	CDC26
YKL010C	UFD4	YLR337C	VRP1
YLR337C	VRP1	YDR207C	UME6
YFL023W	BUD27	YJL183W	MNN11
YIL033C	BCY1	YBL106C	SRO77
YGL087C	MMS2	YHR114W	BZZ1
YPL024W	RMI1	YOR327C	SNC2
YNL206C	RTT106	YPR036W	VMA13
YLR079W	SIC1	YNL225C	CNM67
YIL053W	RHR2	YNL076W	MKS1
YML115C	VAN1	YOR106W	VAM3
YOR066W	MSA1	YOR035C	SHE4
YPR040W	TIP41	YNL116W	DMA2
YOR367W	SCP1	YDL226C	GCS1
YOR358W	HAP5	YGR270W	YTA7
YLR234W	TOP3	YLR410W	VIP1
YER144C	UBP5	YER122C	GLO3
YLR234W	TOP3	YBL051C	PIN4
YDR004W	RAD57	YBR073W	RDH54
YBR217W	ATG12	YMR311C	GLC8

Predictions at threshold level 0.75 continued...			
Gene1 of predicted pair		Gene2 of predicted pair	
Systematic Name	Standard Name	Systematic Name	Standard Name
YLR360W	VPS38	YPR032W	SRO7
YIL159W	BNR1	YKL116C	PRR1
YJL172W	CPS1	YLR398C	SKI2
YEL053C	MAK10	YPR032W	SRO7
YKR020W	VPS51	YNL041C	COG6
YBR171W	SEC66	YGR152C	RSR1
YKL007W	CAP1	YNL135C	FPR1
YMR016C	SOK2	YER044C	ERG28
YMR172W	HOT1	YGL151W	NUT1
YER110C	KAP123	YEL053C	MAK10
YNL236W	SIN4	YOL091W	SPO21
YLR371W	ROM2	YNL090W	RHO2
YJR059W	PTK2	YBL079W	NUP170
YDL116W	NUP84	YBL008W	HIR1
YML071C	COG8	YBR280C	SAF1
YDR155C	CPR1	YBR114W	RAD16
YER139C	YER139C	YJL183W	MNN11
YHR200W	RPN10	YOL054W	PSH1
YMR016C	SOK2	YFL023W	BUD27
YGL223C	COG1	YPL195W	APL5
YKL025C	PAN3	YDR293C	SSD1
YGL078C	DBP3	YPR036W	VMA13
YOR132W	VPS17	YDR183W	PLP1
YMR199W	CLN1	YOR140W	SFL1
YML007W	YAP1	YNL031C	HHT2
YHR079C	IRE1	YER089C	PTC2
YDR363W-A	SEM1	YPL248C	GAL4
YKL126W	YPK1	YOR035C	SHE4
YAL024C	LTE1	YOR337W	TEA1

Predictions at threshold level 0.75 continued...			
Gene1 of predicted pair		Gene2 of predicted pair	
Systematic Name	Standard Name	Systematic Name	Standard Name
YDR080W	VPS41	YDR207C	UME6
YKL116C	PRR1	YDR261C	EXG2
YGL147C	RPL9A	YNR072W	HXT17
YPR089W	YPR089W	YDL155W	CLB3
YER116C	SLX8	YER144C	UBP5
YNL051W	COG5	YJR033C	RAV1
YCR053W	THR4	YOR036W	PEP12
YDL083C	RPS16B	YMR230W	RPS10B
YDR225W	HTA1	YBR057C	MUM2
YMR153W	NUP53	YMR311C	GLC8
YDL184C	RPL41A	YBL025W	RRN10
YMR224C	MRE11	YDR378C	LSM6
YIL008W	URM1	YDR310C	SUM1
YBR107C	IML3	YDR076W	RAD55
YGR270W	YTA7	YOL081W	IRA2
YLR144C	ACF2	YHR016C	YSC84
YEL050C	RML2	YLR410W	VIP1
YKR093W	PTR2	YFL044C	OTU1
YGL054C	ERV14	YNL127W	FAR11
YPR171W	BSP1	YNL094W	APP1
YHR150W	PEX28	YLR324W	PEX30
YPL042C	SSN3	YOR195W	SLK19
YOR112W	CEX1	YDL077C	VAM6
YDR004W	RAD57	YDR076W	RAD55
YPL042C	SSN3	YHR167W	THP2
YDR156W	RPA14	YKR061W	KTR2
YER115C	SPR6	YPL256C	CLN2
YAL020C	ATS1	YOR327C	SNC2
YDR378C	LSM6	YPL161C	BEM4

Predictions at threshold level 0.75 continued...			
Gene1 of predicted pair		Gene2 of predicted pair	
Systematic Name	Standard Name	Systematic Name	Standard Name
YMR224C	MRE11	YPL152W	RRD2
YER087C-B	SBH1	YGL198W	YIP4
YLR061W	RPL22A	YAL002W	VPS8
YKL025C	PAN3	YHR012W	VPS29
YMR142C	RPL13B	YNL307C	MCK1
YEL056W	HAT2	YLR233C	EST1
YDL192W	ARF1	YEL036C	ANP1
YOR275C	RIM20	YKL101W	HSL1
YOL051W	GAL11	YPR072W	NOT5
YPL050C	MNN9	YBL072C	RPS8A
YIL053W	RHR2	YCR053W	THR4
YER116C	SLX8	YDR137W	RGP1
YKL117W	SBA1	YOR358W	HAP5
YDR129C	SAC6	YMR109W	MYO5
YOL126C	MDH2	YPL246C	RBD2
YJR140C	HIR3	YHR077C	NMD2
YOR043W	WHI2	YPL195W	APL5
YBL103C	RTG3	YHR012W	VPS29
YKL213C	DOA1	YHR167W	THP2
YJR090C	GRR1	YDR310C	SUM1
YLR456W	YLR456W	YKR084C	HBS1
YLR102C	APC9	YHR152W	SPO12
YNL094W	APP1	YHR016C	YSC84
YJR040W	GEF1	YJL004C	SYS1
YGR108W	CLB1	YLR381W	CTF3
YPL115C	BEM3	YHR134W	WSS1
YGR217W	CCH1	YBR200W	BEM1
YHR013C	ARD1	YOR141C	ARP8
YNL325C	FIG4	YNL087W	TCB2

Predictions at threshold level 0.75 continued...			
Gene1 of predicted pair		Gene2 of predicted pair	
Systematic Name	Standard Name	Systematic Name	Standard Name
YFL023W	BUD27	YJR032W	CPR7
YDR484W	VPS52	YMR214W	SCJ1
YMR199W	CLN1	YNL116W	DMA2
YGL115W	SNF4	YOL081W	IRA2
YLR192C	HCR1	YFR036W	CDC26
YBR278W	DPB3	YMR055C	BUB2
YLL039C	UBI4	YDR245W	MNN10
YPL253C	VIK1	YGR188C	BUB1
YOR038C	HIR2	YMR038C	CCS1
YMR198W	CIK1	YJR060W	CBF1
YPR017C	DSS4	YPL105C	YPL105C
YMR198W	CIK1	YDL006W	PTC1
YOR337W	TEA1	YHR156C	LIN1
YLR399C	BDF1	YOR038C	HIR2
YIR009W	MSL1	YPL178W	CBC2
YOR275C	RIM20	YGR135W	PRE9
YBR217W	ATG12	YML115C	VAN1
YFL033C	RIM15	YJL004C	SYS1
YKL032C	IXR1	YGR135W	PRE9
YML095C	RAD10	YJR035W	RAD26
YOL121C	RPS19A	YNL076W	MKS1
YGR020C	VMA7	YKL119C	VPH2
YDR443C	SSN2	YLR370C	ARC18
YDR254W	CHL4	YDR171W	HSP42
YBR200W	BEM1	YHR061C	GIC1
YGR249W	MGA1	YHR081W	LRP1
YLR399C	BDF1	YJR063W	RPA12
YCL001W	RER1	YNR032W	PPG1
YDR108W	GSG1	YPL234C	TFP3

Predictions at threshold level 0.75 continued...			
Gene1 of predicted pair		Gene2 of predicted pair	
Systematic Name	Standard Name	Systematic Name	Standard Name
YOR270C	VPH1	YLR370C	ARC18
YDL020C	RPN4	YHL030W	ECM29
YBR131W	CCZ1	YNL238W	KEX2
YDR323C	PEP7	YPR119W	CLB2
YBR195C	MSI1	YOR368W	RAD17
YJL078C	PRY3	YKR093W	PTR2
YBR111W-A	SUS1	YOL012C	HTZ1
YLR018C	POM34	YMR164C	MSS11
YJR060W	CBF1	YML112W	CTK3
YDR080W	VPS41	YOR327C	SNC2
YGL033W	HOP2	YPL137C	GIP3
YMR026C	PEX12	YOL111C	MDY2
YFL047W	RGD2	YKR024C	DBP7
YLR381W	CTF3	YLR429W	CRN1
YPL057C	SUR1	YLR320W	MMS22
YER087C-B	SBH1	YLR113W	HOG1
YNL032W	SIW14	YKR035W-A	DID2
YER162C	RAD4	YEL060C	PRB1
YKR014C	YPT52	YGL198W	YIP4
YMR198W	CIK1	YOR368W	RAD17
YKR099W	BAS1	YMR133W	REC114
YBR260C	RGD1	YER049W	TPA1
YMR016C	SOK2	YLR398C	SKI2
YFR010W	UBP6	YDL155W	CLB3
YOR106W	VAM3	YJR125C	ENT3
YBR131W	CCZ1	YDL006W	PTC1
YGL115W	SNF4	YPR032W	SRO7
YMR224C	MRE11	YLR254C	NDL1
YNR072W	HXT17	YOR327C	SNC2

Predictions at threshold level 0.75 continued...			
Gene1 of predicted pair		Gene2 of predicted pair	
Systematic Name	Standard Name	Systematic Name	Standard Name
YIL076W	SEC28	YBR215W	HPC2
YGR019W	UGA1	YBR200W	BEM1
YKL135C	APL2	YPL259C	APM1
YKL135C	APL2	YJR125C	ENT3
YBR041W	FAT1	YOR275C	RIM20
YDR004W	RAD57	YLR150W	STM1
YLR102C	APC9	YDR507C	GIN4
YGR249W	MGA1	YKL048C	ELM1
YGL147C	RPL9A	YOR043W	WHI2
YEL003W	GIM4	YHR056C	RSC30
YDL223C	HBT1	YOR327C	SNC2
YPL213W	LEA1	YDR440W	DOT1
YJR060W	CBF1	YPL106C	SSE1
YLR090W	XDJ1	YNL058C	YNL058C
YDR004W	RAD57	YNL215W	IES2
YAL024C	LTE1	YBR057C	MUM2
YIL103W	DPH1	YNL225C	CNM67
YGR261C	APL6	YNR032W	PPG1
YNR072W	HXT17	YMR230W	RPS10B
YGL190C	CDC55	YHR191C	CTF8
YNL069C	RPL16B	YDR108W	GSG1
YDR289C	RTT103	YOR124C	UBP2
YLR043C	TRX1	YLR309C	IMH1
YHR156C	LIN1	YOR066W	MSA1
YMR164C	MSS11	YMR125W	STO1
YJL176C	SWI3	YLR018C	POM34
YLR090W	XDJ1	YDR484W	VPS52
YDR335W	MSN5	YJR063W	RPA12
YDR363W-A	SEM1	YER122C	GLO3

Predictions at threshold level 0.75 continued...			
Gene1 of predicted pair		Gene2 of predicted pair	
Systematic Name	Standard Name	Systematic Name	Standard Name
YJL172W	CPS1	YNL238W	KEX2
YKR059W	TIF1	YLR288C	MEC3
YMR224C	MRE11	YBL101C	ECM21
YMR095C	SNO1	YPR046W	MCM16
YLR337C	VRP1	YPL161C	BEM4
YBR108W	YBR108W	YAR014C	BUD14
YBL102W	SFT2	YPL161C	BEM4
YGL212W	VAM7	YOR327C	SNC2
YPR046W	MCM16	YHR115C	DMA1
YLR079W	SIC1	YGR188C	BUB1
YBL079W	NUP170	YOL090W	MSH2
YOR195W	SLK19	YBL031W	SHE1
YOR140W	SFL1	YBR260C	RGD1
YML038C	YMD8	YDL006W	PTC1
YNR072W	HXT17	YBR036C	CSG2
YDR448W	ADA2	YLR357W	RSC2
YOR127W	RGA1	YPL120W	VPS30
YIL076W	SEC28	YBR280C	SAF1
YGL212W	VAM7	YOR312C	RPL20B
YER044C	ERG28	YLR452C	SST2
YLR288C	MEC3	YGL151W	NUT1
YKL032C	IXR1	YBL087C	RPL23A
YGL087C	MMS2	YLR079W	SIC1
YNL325C	FIG4	YNL058C	YNL058C
YJR063W	RPA12	YOL054W	PSH1
YKL160W	ELF1	YGL019W	CKB1
YDL155W	CLB3	YDR310C	SUM1
YLR234W	TOP3	YKL101W	HSL1
YMR164C	MSS11	YKR024C	DBP7

Predictions at threshold level 0.75 continued...			
Gene1 of predicted pair		Gene2 of predicted pair	
Systematic Name	Standard Name	Systematic Name	Standard Name
YBR030W	YBR030W	YBL025W	RRN10
YPL137C	GIP3	YOR035C	SHE4
YIL103W	DPH1	YOL054W	PSH1
YOL145C	CTR9	YNL297C	MON2
YDR293C	SSD1	YKR048C	NAP1
YKL110C	KTI12	YOL018C	TLG2
YHR200W	RPN10	YNL076W	MKS1
YDR004W	RAD57	YNL082W	PMS1
YOR080W	DIA2	YOR001W	RRP6
YGR104C	SRB5	YDR207C	UME6
YML001W	YPT7	YOL081W	IRA2
YER161C	SPT2	YOR368W	RAD17
YER116C	SLX8	YOR270C	VPH1
YGL163C	RAD54	YBL087C	RPL23A
YOR132W	VPS17	YDR108W	GSG1
YMR224C	MRE11	YHR081W	LRP1
YNL215W	IES2	YGR188C	BUB1
YJL183W	MNN11	YBR280C	SAF1
YGL223C	COG1	YPL037C	EGD1
YDL059C	RAD59	YDR310C	SUM1
YKL139W	CTK1	YOR066W	MSA1
YDR293C	SSD1	YNL069C	RPL16B
YLR381W	CTF3	YDR335W	MSN5
YBR228W	SLX1	YOR243C	PUS7
YML115C	VAN1	YDL083C	RPS16B
YMR039C	SUB1	YBR278W	DPB3
YHR098C	SFB3	YOL006C	TOP1
YMR142C	RPL13B	YOR043W	WHI2
YFR031C-A	RPL2A	YER122C	GLO3

Predictions at threshold level 0.75 continued...			
**Gene1 of predicted pair**		**Gene2 of predicted pair**	
**Systematic Name**	**Standard Name**	**Systematic Name**	**Standard Name**
YOL054W	PSH1	YHR115C	DMA1
YBR131W	CCZ1	YDR080W	VPS41
YDL042C	SIR2	YNL031C	HHT2
YMR016C	SOK2	YPR036W	VMA13
YPL152W	RRD2	YAR014C	BUD14
YNR052C	POP2	YHR111W	UBA4
YOR083W	WHI5	YLR079W	SIC1
YLL039C	UBI4	YGL212W	VAM7
YBR108W	YBR108W	YOR290C	SNF2
YPL253C	VIK1	YLR429W	CRN1
YDR320C	SWA2	YER120W	SCS2
YOR001W	RRP6	YER122C	GLO3
YNL215W	IES2	YLR386W	VAC14
YMR172W	HOT1	YLR381W	CTF3
YLR370C	ARC18	YNL076W	MKS1
YER143W	DDI1	YGL095C	VPS45
YCR066W	RAD18	YJR033C	RAV1
YGR072W	UPF3	YDR207C	UME6
YGL174W	BUD13	YDR335W	MSN5
YBR108W	YBR108W	YJL172W	CPS1
YBL102W	SFT2	YPR032W	SRO7
YOR212W	STE4	YIL007C	NAS2
YHR052W	CIC1	YJL189W	RPL39
YDL077C	VAM6	YBR280C	SAF1
YDL002C	NHP10	YBR195C	MSI1
YMR199W	CLN1	YBL079W	NUP170
YAL024C	LTE1	YOR141C	ARP8
YHR039C-A	VMA10	YOL121C	RPS19A
YLR452C	SST2	YPL195W	APL5

Predictions at threshold level 0.75 continued...			
Gene1 of predicted pair		Gene2 of predicted pair	
Systematic Name	Standard Name	Systematic Name	Standard Name
YPL253C	VIK1	YDR183W	PLP1
YJR032W	CPR7	YHL002W	HSE1
YDL052C	SLC1	YKL140W	TGL1
YHL031C	GOS1	YPR051W	MAK3
YJL029C	VPS53	YOL018C	TLG2
YKL116C	PRR1	YDL192W	ARF1
YLR102C	APC9	YJL136C	RPS21B
YNL206C	RTT106	YHR152W	SPO12
YNL054W	VAC7	YHR013C	ARD1
YPL037C	EGD1	YAL002W	VPS8
YBL008W	HIR1	YHR178W	STB5
YBR128C	ATG14	YPR119W	CLB2
YMR116C	ASC1	YOL081W	IRA2
YBR030W	YBR030W	YBR108W	YBR108W
YDR156W	RPA14	YJL117W	PHO86
YOL090W	MSH2	YBR082C	UBC4
YGL161C	YIP5	YER139C	YER139C
YIL002C	INP51	YGL124C	MON1
YBR200W	BEM1	YPR046W	MCM16
YOR195W	SLK19	YDR318W	MCM21
YGL147C	RPL9A	YBL025W	RRN10
YIL076W	SEC28	YPR036W	VMA13
YDR254W	CHL4	YCR086W	CSM1
YBR260C	RGD1	YMR311C	GLC8
YDR225W	HTA1	YDR440W	DOT1
YDL020C	RPN4	YFR036W	CDC26
YMR202W	ERG2	YJL058C	BIT61
YER032W	FIR1	YOR363C	PIP2
YHR111W	UBA4	YOL054W	PSH1

Predictions at threshold level 0.75 continued...			
Gene1 of predicted pair		Gene2 of predicted pair	
Systematic Name	Standard Name	Systematic Name	Standard Name
YKR059W	TIF1	YLR350W	ORM2
YDR289C	RTT103	YER122C	GLO3
YMR026C	PEX12	YBR019C	GAL10
YER110C	KAP123	YBR010W	HHT1
YFR036W	CDC26	YCR053W	THR4
YDL020C	RPN4	YHR167W	THP2
YOR083W	WHI5	YOR080W	DIA2
YER116C	SLX8	YMR016C	SOK2
YNL025C	SSN8	YOR124C	UBP2
YIL053W	RHR2	YLR192C	HCR1
YLL007C	YLL007C	YPL018W	CTF19
YPL112C	PEX25	YDL240W	LRG1
YBR200W	BEM1	YBL047C	EDE1
YER044C	ERG28	YLR370C	ARC18
YLR192C	HCR1	YBL025W	RRN10
YDR320C	SWA2	YHR012W	VPS29
YJR060W	CBF1	YDR200C	VPS64
YBR278W	DPB3	YGL151W	NUT1
YIL159W	BNR1	YER044C	ERG28
YMR116C	ASC1	YHR200W	RPN10
YMR055C	BUB2	YLR435W	TSR2
YPL096C-A	ERI1	YNL079C	TPM1
YMR026C	PEX12	YNL076W	MKS1
YML001W	YPT7	YPR032W	SRO7
YBR200W	BEM1	YIL103W	DPH1
YCR084C	TUP1	YPL248C	GAL4
YLR090W	XDJ1	YDL083C	RPS16B
YPR036W	VMA13	YLR398C	SKI2
YCR066W	RAD18	YFL033C	RIM15

Predictions at threshold level 0.75 continued...			
Gene1 of predicted pair		Gene2 of predicted pair	
Systematic Name	Standard Name	Systematic Name	Standard Name
YCR066W	RAD18	YER120W	SCS2
YKL135C	APL2	YPR119W	CLB2
YER116C	SLX8	YFR036W	CDC26
YEL053C	MAK10	YER139C	YER139C
YDR335W	MSN5	YGR188C	BUB1
YMR198W	CIK1	YKL101W	HSL1
YKR072C	SIS2	YCR086W	CSM1
YDR004W	RAD57	YGL148W	ARO2
YLL007C	YLL007C	YGL151W	NUT1
YMR026C	PEX12	YHR081W	LRP1
YHR111W	UBA4	YGL031C	RPL24A
YNL243W	SLA2	YHR181W	SVP26
YIL034C	CAP2	YGR188C	BUB1
YMR091C	NPL6	YJR032W	CPR7
YNL297C	MON2	YHR060W	VMA22
YOR106W	VAM3	YGL003C	CDH1
YOR033C	EXO1	YHR010W	RPL27A
YNL218W	MGS1	YDL013W	SLX5
YLR429W	CRN1	YJR060W	CBF1
YLR381W	CTF3	YKL114C	APN1
YLL007C	YLL007C	YOR132W	VPS17
YJL058C	BIT61	YLR370C	ARC18
YKL101W	HSL1	YOR368W	RAD17
YOL148C	SPT20	YGL252C	RTG2
YGL223C	COG1	YGR105W	VMA21
YKR001C	VPS1	YKL119C	VPH2
YBR217W	ATG12	YER040W	GLN3
YLR396C	VPS33	YDL077C	VAM6
YPL047W	SGF11	YBR111W-A	SUS1

Predictions at threshold level 0.75 continued...			
Gene1 of predicted pair		Gene2 of predicted pair	
Systematic Name	Standard Name	Systematic Name	Standard Name
YCR020C-A	MAK31	YFL033C	RIM15
YMR273C	ZDS1	YDR227W	SIR4
YBR200W	BEM1	YDR379W	RGA2
YNL127W	FAR11	YPR032W	SRO7
YPL024W	RMI1	YKR059W	TIF1
YMR199W	CLN1	YKR024C	DBP7
YER116C	SLX8	YGL163C	RAD54
YHR200W	RPN10	YJL140W	RPB4
YER162C	RAD4	YBL025W	RRN10
YML016C	PPZ1	YDR289C	RTT103
YMR224C	MRE11	YML016C	PPZ1
YPL024W	RMI1	YML001W	YPT7
YIL034C	CAP2	YDR254W	CHL4
YBR108W	YBR108W	YDR261C	EXG2
YBR131W	CCZ1	YLR093C	NYV1
YHR111W	UBA4	YHR012W	VPS29
YJR140C	HIR3	YPL079W	RPL21B
YNL201C	PSY2	YDR378C	LSM6
YOR001W	RRP6	YBR215W	HPC2
YKR099W	BAS1	YML071C	COG8
YCL037C	SRO9	YDL077C	VAM6
YHR111W	UBA4	YFR030W	MET10
YBR200W	BEM1	YNL076W	MKS1
YKL048C	ELM1	YJR060W	CBF1
YMR116C	ASC1	YOR080W	DIA2
YGL147C	RPL9A	YBR280C	SAF1
YMR016C	SOK2	YPL195W	APL5
YNR052C	POP2	YFR030W	MET10
YGL033W	HOP2	YDR310C	SUM1

Predictions at threshold level 0.75 continued...			
Gene1 of predicted pair		Gene2 of predicted pair	
Systematic Name	Standard Name	Systematic Name	Standard Name
YGL163C	RAD54	YGR135W	PRE9
YOR109W	INP53	YEL051W	VMA8
YGL147C	RPL9A	YHR081W	LRP1
YKR035W-A	DID2	YDR207C	UME6
YLR441C	RPS1A	YGL212W	VAM7
YHR013C	ARD1	YCR086W	CSM1
YOR033C	EXO1	YDL020C	RPN4
YIL002C	INP51	YDL226C	GCS1
YBR126C	TPS1	YNL307C	MCK1
YPL115C	BEM3	YHR012W	VPS29
YDL006W	PTC1	YER162C	RAD4
YEL065W	SIT1	YLR441C	RPS1A
YCL037C	SRO9	YLR182W	SWI6
YML115C	VAN1	YNL117W	MLS1
YNL243W	SLA2	YBR098W	MMS4
YPL178W	CBC2	YMR125W	STO1
YPL234C	TFP3	YER120W	SCS2
YGL094C	PAN2	YBR089C-A	NHP6B
YOR080W	DIA2	YJL124C	LSM1
YNL288W	CAF40	YGR270W	YTA7
YDR156W	RPA14	YPL089C	RLM1
YPL253C	VIK1	YDR335W	MSN5
YLR095C	IOC2	YBR108W	YBR108W
YNL044W	YIP3	YNR049C	MSO1
YDR443C	SSN2	YJR084W	CSN12
YCR088W	ABP1	YLR370C	ARC18
YDR379W	RGA2	YHR152W	SPO12
YLR441C	RPS1A	YNL041C	COG6
YLR234W	TOP3	YDR254W	CHL4

Predictions at threshold level 0.75 continued...			
Gene1 of predicted pair		Gene2 of predicted pair	
Systematic Name	Standard Name	Systematic Name	Standard Name
YML062C	MFT1	YJR035W	RAD26
YGR252W	GCN5	YBR089C-A	NHP6B
YNL201C	PSY2	YJL006C	CTK2
YBR239C	YBR239C	YOR284W	HUA2
YPL001W	HAT1	YER161C	SPT2
YHR039C-A	VMA10	YMR054W	STV1
YNL094W	APP1	YLR064W	YLR064W
YBR217W	ATG12	YLR320W	MMS22
YLR102C	APC9	YDR264C	AKR1
YLR079W	SIC1	YKL101W	HSL1
YMR052W	FAR3	YNL127W	FAR11
YJL006C	CTK2	YIL103W	DPH1
YDR253C	MET32	YLR079W	SIC1
YOR290C	SNF2	YOR327C	SNC2
YPL037C	EGD1	YPL195W	APL5
YJL095W	BCK1	YBR036C	CSG2
YML028W	TSA1	YJR035W	RAD26
YPL253C	VIK1	YLR425W	TUS1
YOR127W	RGA1	YGR135W	PRE9
YBR114W	RAD16	YGR188C	BUB1
YDR129C	SAC6	YGR135W	PRE9
YLR133W	CKI1	YMR022W	UBC7
YLR093C	NYV1	YLR180W	SAM1
YGL100W	SEH1	YNL082W	PMS1
YDR318W	MCM21	YJL190C	RPS22A
YDR004W	RAD57	YMR199W	CLN1
YMR026C	PEX12	YHR156C	LIN1
YOR069W	VPS5	YML071C	COG8
YNL206C	RTT106	YPR120C	CLB5

Predictions at threshold level 0.75 continued...			
Gene1 of predicted pair		Gene2 of predicted pair	
Systematic Name	Standard Name	Systematic Name	Standard Name
YLR337C	VRP1	YDR310C	SUM1
YOR275C	RIM20	YHL011C	PRS3
YFR031C-A	RPL2A	YHR156C	LIN1
YDR293C	SSD1	YDR137W	RGP1
YDL223C	HBT1	YLR370C	ARC18
YDR363W-A	SEM1	YOR275C	RIM20
YNL236W	SIN4	YJR135C	MCM22
YPL253C	VIK1	YPL057C	SUR1
YPL240C	HSP82	YCL018W	LEU2
YER116C	SLX8	YDR254W	CHL4
YPL234C	TFP3	YER162C	RAD4
YIL076W	SEC28	YER116C	SLX8
YHR178W	STB5	YLR381W	CTF3
YMR167W	MLH1	YGL019W	CKB1
YLR192C	HCR1	YNL041C	COG6
YJR032W	CPR7	YJL095W	BCK1
YPR084W	YPR084W	YFR031C-A	RPL2A
YMR109W	MYO5	YJR135C	MCM22
YNL325C	FIG4	YML071C	COG8
YGL078C	DBP3	YBR030W	YBR030W
YLR064W	YLR064W	YHR016C	YSC84
YJL036W	SNX4	YGL212W	VAM7
YER087C-B	SBH1	YBR171W	SEC66
YBR126C	TPS1	YDL223C	HBT1
YPL158C	YPL158C	YDL006W	PTC1
YHR205W	SCH9	YNL199C	GCR2
YOR191W	RIS1	YHR167W	THP2
YNL215W	IES2	YKL110C	KTI12
YOR212W	STE4	YPL256C	CLN2

Predictions at threshold level 0.75 continued...			
Gene1 of predicted pair		Gene2 of predicted pair	
Systematic Name	Standard Name	Systematic Name	Standard Name
YLR119W	SRN2	YJR084W	CSN12
YEL013W	VAC8	YMR038C	CCS1
YBR133C	HSL7	YBL002W	HTB2
YGR104C	SRB5	YCL067C	HMLALPHA2
YNR032C-A	HUB1	YLR313C	SPH1
YBR108W	YBR108W	YOR023C	AHC1
YFR031C-A	RPL2A	YLR386W	VAC14
YHR200W	RPN10	YKR020W	VPS51
YDR363W-A	SEM1	YMR276W	DSK2
YNL166C	BNI5	YJR135C	MCM22
YBL103C	RTG3	YPL037C	EGD1
YGL087C	MMS2	YAL002W	VPS8
YDR108W	GSG1	YDR207C	UME6
YPR051W	MAK3	YER122C	GLO3
YNL307C	MCK1	YJL006C	CTK2
YAL024C	LTE1	YER161C	SPT2
YMR198W	CIK1	YDR318W	MCM21
YFR010W	UBP6	YDL002C	NHP10
YDR440W	DOT1	YDR289C	RTT103
YKL025C	PAN3	YEL056W	HAT2
YNL243W	SLA2	YBL047C	EDE1
YER116C	SLX8	YLR313C	SPH1
YER096W	SHC1	YGR188C	BUB1
YPL042C	SSN3	YER161C	SPT2
YBR126C	TPS1	YMR230W	RPS10B
YPL137C	GIP3	YLR371W	ROM2
YER122C	GLO3	YDL137W	ARF2
YER107C	GLE2	YNL271C	BNI1
YDR383C	NKP1	YBR200W	BEM1

Predictions at threshold level 0.75 continued...			
Gene1 of predicted pair		Gene2 of predicted pair	
Systematic Name	Standard Name	Systematic Name	Standard Name
YDL127W	PCL2	YDR076W	RAD55
YNL238W	KEX2	YDL192W	ARF1
YMR216C	SKY1	YHR039C-A	VMA10
YNL215W	IES2	YPR120C	CLB5
YLR119W	SRN2	YLR094C	GIS3
YDL083C	RPS16B	YOL081W	IRA2
YBR107C	IML3	YOR014W	RTS1
YBR171W	SEC66	YPL256C	CLN2
YGR249W	MGA1	YLR093C	NYV1
YOR001W	RRP6	YGL003C	CDH1
YMR224C	MRE11	YLR320W	MMS22
YIL034C	CAP2	YOR367W	SCP1
YBR200W	BEM1	YPL253C	VIK1
YIL076W	SEC28	YOR001W	RRP6
YER120W	SCS2	YNL041C	COG6
YMR179W	SPT21	YPR120C	CLB5
YGR249W	MGA1	YKR035W-A	DID2
YAL024C	LTE1	YGR249W	MGA1
YOR001W	RRP6	YDR318W	MCM21
YKR059W	TIF1	YLR435W	TSR2
YNL021W	HDA1	YDR295C	HDA2
YDR318W	MCM21	YER122C	GLO3
YBR200W	BEM1	YEL060C	PRB1
YDR439W	LRS4	YNL116W	DMA2
YNL087W	TCB2	YBR280C	SAF1
YNL248C	RPA49	YHR111W	UBA4
YDL192W	ARF1	YBR036C	CSG2
YPL045W	VPS16	YOR035C	SHE4
YMR080C	NAM7	YHR052W	CIC1

Predictions at threshold level 0.75 continued...			
Gene1 of predicted pair		Gene2 of predicted pair	
Systematic Name	Standard Name	Systematic Name	Standard Name
YJR060W	CBF1	YCR086W	CSM1
YDL192W	ARF1	YNL051W	COG5
YKR035W-A	DID2	YBR036C	CSG2
YGR135W	PRE9	YMR038C	CCS1
YDL056W	MBP1	YPL161C	BEM4
YMR153W	NUP53	YLR370C	ARC18
YDR385W	EFT2	YLR131C	ACE2
YPL115C	BEM3	YGR188C	BUB1
YGL135W	RPL1B	YGR105W	VMA21
YNL206C	RTT106	YJL006C	CTK2
YMR224C	MRE11	YLR192C	HCR1
YGR054W	YGR054W	YEL056W	HAT2
YDR484W	VPS52	YER122C	GLO3
YBR010W	HHT1	YJL140W	RPB4
YBL058W	SHP1	YLR398C	SKI2
YFR010W	UBP6	YDR363W-A	SEM1
YFR010W	UBP6	YML071C	COG8
YHL031C	GOS1	YDL192W	ARF1
YPR084W	YPR084W	YDR080W	VPS41
YHR134W	WSS1	YER116C	SLX8
YFR031C-A	RPL2A	YOL018C	TLG2
YNL201C	PSY2	YNL307C	MCK1
YBR084C-A	RPL19A	YDR264C	AKR1
YGL133W	ITC1	YJL136C	RPS21B
YBR108W	YBR108W	YFL023W	BUD27
YJL154C	VPS35	YLR381W	CTF3
YBR084C-A	RPL19A	YKL101W	HSL1
YGR261C	APL6	YER122C	GLO3
YDL020C	RPN4	YOR014W	RTS1

Predictions at threshold level 0.75 continued...			
Gene1 of predicted pair		Gene2 of predicted pair	
Systematic Name	Standard Name	Systematic Name	Standard Name
YMR273C	ZDS1	YIL033C	BCY1
YDR261C	EXG2	YNL238W	KEX2
YOR001W	RRP6	YML028W	TSA1
YOR033C	EXO1	YJR035W	RAD26
YLR079W	SIC1	YLR182W	SWI6
YIL033C	BCY1	YJL164C	TPK1
YBR217W	ATG12	YBR171W	SEC66
YBR194W	SOY1	YER161C	SPT2
YDR261C	EXG2	YNL041C	COG6
YER116C	SLX8	YPR030W	CSR2
YKL139W	CTK1	YGR188C	BUB1
YNL201C	PSY2	YOL054W	PSH1
YDR202C	RAV2	YAL002W	VPS8
YNL054W	VAC7	YAL010C	MDM10
YIL036W	CST6	YGR014W	MSB2
YPL129W	TAF14	YOR038C	HIR2
YNL106C	INP52	YDR335W	MSN5
YDR004W	RAD57	YHR200W	RPN10
YLR119W	SRN2	YER144C	UBP5
YNL025C	SSN8	YNL288W	CAF40
YJR024C	MDE1	YMR199W	CLN1
YML115C	VAN1	YKR035W-A	DID2
YNL201C	PSY2	YPR040W	TIP41
YIL053W	RHR2	YPR036W	VMA13
YPR018W	RLF2	YGL025C	PGD1
YBL051C	PIN4	YGR054W	YGR054W
YMR198W	CIK1	YHR200W	RPN10
YHR111W	UBA4	YNL107W	YAF9
YFR031C-A	RPL2A	YMR026C	PEX12

Predictions at threshold level 0.75 continued...			
Gene1 of predicted pair		Gene2 of predicted pair	
Systematic Name	Standard Name	Systematic Name	Standard Name
YHR060W	VMA22	YCR053W	THR4
YMR199W	CLN1	YBR215W	HPC2
YML095C	RAD10	YDL226C	GCS1
YDR174W	HMO1	YDR254W	CHL4
YBR019C	GAL10	YER040W	GLN3
YOR141C	ARP8	YGR188C	BUB1
YGR249W	MGA1	YLR441C	RPS1A
YPR030W	CSR2	YNL076W	MKS1
YLR429W	CRN1	YNL236W	SIN4
YMR231W	PEP5	YOR036W	PEP12
YBL058W	SHP1	YMR153W	NUP53
YNL264C	PDR17	YOL110W	SHR5
YDR108W	GSG1	YML038C	YMD8
YDL047W	SIT4	YHR115C	DMA1
YGR239C	PEX21	YGR077C	PEX8
YOR106W	VAM3	YBR036C	CSG2
YBL102W	SFT2	YOL108C	INO4
YER116C	SLX8	YBR019C	GAL10
YMR026C	PEX12	YKL116C	PRR1
YEL065W	SIT1	YPL037C	EGD1
YOR035C	SHE4	YER122C	GLO3
YBR215W	HPC2	YEL056W	HAT2
YMR224C	MRE11	YDL184C	RPL41A
YBR194W	SOY1	YGL174W	BUD13
YFL013C	IES1	YDR245W	MNN10
YBR274W	CHK1	YGL175C	SAE2
YML001W	YPT7	YGR105W	VMA21
YNL215W	IES2	YHR013C	ARD1
YHR200W	RPN10	YOR308C	SNU66

Predictions at threshold level 0.75 continued...			
Gene1 of predicted pair		Gene2 of predicted pair	
Systematic Name	Standard Name	Systematic Name	Standard Name
YHR178W	STB5	YGL151W	NUT1
YOR085W	OST3	YML019W	OST6
YPL239W	YAR1	YKL143W	LTV1
YDR083W	RRP8	YOR001W	RRP6
YDL059C	RAD59	YOR312C	RPL20B
YHR156C	LIN1	YGL124C	MON1
YNL032W	SIW14	YGL212W	VAM7
YKR101W	SIR1	YKL160W	ELF1
YGR104C	SRB5	YLR350W	ORM2
YOR290C	SNF2	YBR280C	SAF1
YMR080C	NAM7	YHR167W	THP2
YBR131W	CCZ1	YKL048C	ELM1
YMR311C	GLC8	YER122C	GLO3
YAL024C	LTE1	YJR084W	CSN12
YDL232W	OST4	YER019C-A	SBH2
YMR152W	YIM1	YBR019C	GAL10
YGR249W	MGA1	YNL032W	SIW14
YKL116C	PRR1	YPL137C	GIP3
YLR441C	RPS1A	YMR214W	SCJ1
YKL160W	ELF1	YLR315W	NKP2
YML115C	VAN1	YOR014W	RTS1
YPR040W	TIP41	YJL095W	BCK1
YLR292C	SEC72	YER110C	KAP123
YMR153W	NUP53	YKL068W	NUP100
YCL001W	RER1	YDL006W	PTC1
YBR228W	SLX1	YER116C	SLX8
YHR039C-A	VMA10	YGR105W	VMA21
YCR066W	RAD18	YOL108C	INO4
YHR013C	ARD1	YDR440W	DOT1

Predictions at threshold level 0.75 continued...			
Gene1 of predicted pair		Gene2 of predicted pair	
Systematic Name	Standard Name	Systematic Name	Standard Name
YLR370C	ARC18	YKL114C	APN1
YNL032W	SIW14	YPL195W	APL5
YLR058C	SHM2	YJR060W	CBF1
YKL139W	CTK1	YNL329C	PEX6
YLR386W	VAC14	YLR447C	VMA6
YGL003C	CDH1	YJR084W	CSN12
YML071C	COG8	YOL108C	INO4
YMR199W	CLN1	YJL187C	SWE1
YOR109W	INP53	YKR001C	VPS1
YDL184C	RPL41A	YBR108W	YBR108W
YKR001C	VPS1	YPL259C	APM1
YOL121C	RPS19A	YJL183W	MNN11
YOR327C	SNC2	YEL013W	VAC8
YMR091C	NPL6	YNL041C	COG6
YOR195W	SLK19	YLR254C	NDL1
YDR484W	VPS52	YDR358W	GGA1
YHL031C	GOS1	YCR066W	RAD18
YML115C	VAN1	YKR014C	YPT52
YOR106W	VAM3	YML001W	YPT7
YPR171W	BSP1	YNL106C	INP52
YJL154C	VPS35	YER049W	TPA1
YMR153W	NUP53	YNL087W	TCB2
YER129W	SAK1	YDR507C	GIN4
YMR116C	ASC1	YKR020W	VPS51
YOR337W	TEA1	YDR484W	VPS52
YDR245W	MNN10	YAL002W	VPS8
YOR039W	CKB2	YOL081W	IRA2
YMR311C	GLC8	YER049W	TPA1
YNL199C	GCR2	YLR425W	TUS1

Predictions at threshold level 0.75 continued...			
Gene1 of predicted pair		Gene2 of predicted pair	
Systematic Name	Standard Name	Systematic Name	Standard Name
YLR119W	SRN2	YDR310C	SUM1
YDR108W	GSG1	YNL041C	COG6
YCL037C	SRO9	YKR030W	GMH1
YMR224C	MRE11	YLR399C	BDF1
YBR131W	CCZ1	YDR202C	RAV2
YPL042C	SSN3	YDR469W	SDC1
YOL111C	MDY2	YPL161C	BEM4
YPL042C	SSN3	YHL031C	GOS1
YPL022W	RAD1	YGL190C	CDC55
YDR108W	GSG1	YDR202C	RAV2
YMR224C	MRE11	YDR183W	PLP1
YDL083C	RPS16B	YNL041C	COG6
YLR360W	VPS38	YPR046W	MCM16
YLR410W	VIP1	YOL090W	MSH2
YPL089C	RLM1	YMR038C	CCS1
YLR288C	MEC3	YMR167W	MLH1
YJL154C	VPS35	YNL044W	YIP3
YOR069W	VPS5	YAL002W	VPS8
YDL175C	AIR2	YJL124C	LSM1
YLR371W	ROM2	YGL124C	MON1
YIR009W	MSL1	YJR050W	ISY1
YOR106W	VAM3	YMR311C	GLC8
YJL141C	YAK1	YPR119W	CLB2
YLR370C	ARC18	YEL060C	PRB1
YJL124C	LSM1	YOR038C	HIR2
YPR030W	CSR2	YMR152W	YIM1
YNL031C	HHT2	YOR038C	HIR2
YDR484W	VPS52	YGL124C	MON1
YMR109W	MYO5	YPL234C	TFP3

Predictions at threshold level 0.75 continued...			
Gene1 of predicted pair		Gene2 of predicted pair	
Systematic Name	Standard Name	Systematic Name	Standard Name
YGR252W	GCN5	YCR084C	TUP1
YHL031C	GOS1	YDR202C	RAV2
YLR452C	SST2	YMR214W	SCJ1
YBR194W	SOY1	YDR335W	MSN5
YJL095W	BCK1	YLR032W	RAD5
YKL116C	PRR1	YJR056C	YJR056C
YLR192C	HCR1	YGL124C	MON1
YER161C	SPT2	YCR077C	PAT1
YNL069C	RPL16B	YNL297C	MON2
YMR038C	CCS1	YJR035W	RAD26
YDR293C	SSD1	YDR144C	MKC7
YJL047C	RTT101	YER105C	NUP157
YGL003C	CDH1	YBR036C	CSG2
YJL004C	SYS1	YER162C	RAD4
YPL024W	RMI1	YJL124C	LSM1
YNL291C	MID1	YPL057C	SUR1
YGL124C	MON1	YDR207C	UME6
YDR225W	HTA1	YHR200W	RPN10
YBR200W	BEM1	YBR036C	CSG2
YOR195W	SLK19	YNL236W	SIN4
YBR228W	SLX1	YNL016W	PUB1
YLR441C	RPS1A	YOR327C	SNC2
YGR054W	YGR054W	YJR032W	CPR7
YLR381W	CTF3	YNL107W	YAF9
YGL163C	RAD54	YLR352W	YLR352W
YGL161C	YIP5	YAL002W	VPS8
YGL054C	ERV14	YER120W	SCS2
YOR231W	MKK1	YPR119W	CLB2
YGL212W	VAM7	YPR051W	MAK3

Predictions at threshold level 0.75 continued...			
Gene1 of predicted pair		Gene2 of predicted pair	
Systematic Name	Standard Name	Systematic Name	Standard Name
YBL102W	SFT2	YNL051W	COG5
YPL161C	BEM4	YEL056W	HAT2
YNL325C	FIG4	YGL124C	MON1
YKL126W	YPK1	YGL003C	CDH1
YPR051W	MAK3	YHL020C	OPI1
YCR086W	CSM1	YKL101W	HSL1
YNL041C	COG6	YJL004C	SYS1
YGL178W	MPT5	YDR293C	SSD1
YLR435W	TSR2	YJR032W	CPR7
YML016C	PPZ1	YKR061W	KTR2
YLR352W	YLR352W	YOR368W	RAD17
YMR055C	BUB2	YMR167W	MLH1
YBR278W	DPB3	YGL163C	RAD54
YJL154C	VPS35	YMR055C	BUB2
YOR327C	SNC2	YNL116W	DMA2
YCR066W	RAD18	YEL053C	MAK10
YKL139W	CTK1	YPR036W	VMA13
YIL159W	BNR1	YER040W	GLN3
YLR410W	VIP1	YFR030W	MET10
YBR108W	YBR108W	YJR032W	CPR7
YOR080W	DIA2	YGL174W	BUD13
YGL087C	MMS2	YOR368W	RAD17
YBR127C	VMA2	YKL119C	VPH2
YNL041C	COG6	YOL081W	IRA2
YKL116C	PRR1	YEL060C	PRB1
YOR101W	RAS1	YNL236W	SIN4
YLR306W	UBC12	YMR038C	CCS1
YML001W	YPT7	YOL121C	RPS19A
YMR164C	MSS11	YGL016W	KAP122

Predictions at threshold level 0.75 continued...			
Gene1 of predicted pair		Gene2 of predicted pair	
Systematic Name	Standard Name	Systematic Name	Standard Name
YLR352W	YLR352W	YML028W	TSA1
YGL178W	MPT5	YGR200C	ELP2
YGL212W	VAM7	YPL195W	APL5
YEL009C	GCN4	YDR443C	SSN2
YDR363W	ESC2	YNL243W	SLA2
YNL325C	FIG4	YOL121C	RPS19A
YGL078C	DBP3	YLR180W	SAM1
YNL307C	MCK1	YLR094C	GIS3
YMR016C	SOK2	YLR452C	SST2
YFR040W	SAP155	YDR310C	SUM1
YJL184W	GON7	YGR262C	BUD32
YDL184C	RPL41A	YDL192W	ARF1
YCR088W	ABP1	YHR016C	YSC84
YOR270C	VPH1	YPR046W	MCM16
YJL124C	LSM1	YNL145W	MFA2
YLL007C	YLL007C	YNL107W	YAF9
YBR173C	UMP1	YNL307C	MCK1
YDR443C	SSN2	YKL126W	YPK1
YFR010W	UBP6	YBR215W	HPC2
YOR332W	VMA4	YBL047C	EDE1
YLR093C	NYV1	YLR398C	SKI2
YHR081W	LRP1	YPR030W	CSR2
YNL325C	FIG4	YMR214W	SCJ1
YMR284W	YKU70	YGL031C	RPL24A
YGL223C	COG1	YEL051W	VMA8
YHR178W	STB5	YGL253W	HXK2
YML001W	YPT7	YBR036C	CSG2
YHR079C	IRE1	YGR252W	GCN5
YML095C	RAD10	YDR289C	RTT103

Predictions at threshold level 0.75 continued...			
Gene1 of predicted pair		Gene2 of predicted pair	
Systematic Name	Standard Name	Systematic Name	Standard Name
YDR225W	HTA1	YFR034C	PHO4
YGL241W	KAP114	YGR252W	GCN5
YNL107W	YAF9	YOL108C	INO4
YLR288C	MEC3	YMR231W	PEP5
YMR016C	SOK2	YKL048C	ELM1
YPL253C	VIK1	YMR055C	BUB2
YBR019C	GAL10	YGL252C	RTG2
YLR192C	HCR1	YPL161C	BEM4
YBR200W	BEM1	YML071C	COG8
YGR229C	SMI1	YGL190C	CDC55
YDR490C	PKH1	YHR205W	SCH9
YDR293C	SSD1	YBR131W	CCZ1
YIL053W	RHR2	YOL081W	IRA2
YPL195W	APL5	YJL183W	MNN11
YDR004W	RAD57	YDR097C	MSH6
YDR335W	MSN5	YGL163C	RAD54
YPL057C	SUR1	YNL199C	GCR2
YBR200W	BEM1	YCR077C	PAT1
YFR010W	UBP6	YER040W	GLN3
YDR174W	HMO1	YMR055C	BUB2
YHL031C	GOS1	YML067C	ERV41
YOR008C	SLG1	YPL256C	CLN2
YLR192C	HCR1	YNL087W	TCB2
YDR244W	PEX5	YNL214W	PEX17
YGL163C	RAD54	YOL054W	PSH1
YJR040W	GEF1	YNL041C	COG6
YKR059W	TIF1	YGL250W	RMR1
YPL057C	SUR1	YJR135C	MCM22
YKL129C	MYO3	YJL095W	BCK1

Predictions at threshold level 0.75 continued...			
Gene1 of predicted pair		Gene2 of predicted pair	
Systematic Name	Standard Name	Systematic Name	Standard Name
YMR095C	SNO1	YER144C	UBP5
YJL176C	SWI3	YHR077C	NMD2
YMR127C	SAS2	YLR447C	VMA6
YBR215W	HPC2	YBR089C-A	NHP6B
YGL200C	EMP24	YEL036C	ANP1
YLR083C	EMP70	YEL053C	MAK10
YDR156W	RPA14	YCR086W	CSM1
YMR026C	PEX12	YER044C	ERG28
YDL116W	NUP84	YDL101C	DUN1
YKL139W	CTK1	YBL103C	RTG3
YDL116W	NUP84	YDR335W	MSN5
YPR036W	VMA13	YDL185W	TFP1
YBR084C-A	RPL19A	YBL047C	EDE1
YFR010W	UBP6	YLR381W	CTF3
YKR001C	VPS1	YJR032W	CPR7
YGR108W	CLB1	YJR032W	CPR7
YPL057C	SUR1	YMR133W	REC114
YDR202C	RAV2	YNL041C	COG6
YLR410W	VIP1	YOL091W	SPO21
YOR106W	VAM3	YCR066W	RAD18
YMR199W	CLN1	YDL006W	PTC1
YHR134W	WSS1	YDR155C	CPR1
YBR172C	SMY2	YPR119W	CLB2
YDR183W	PLP1	YBR114W	RAD16
YAL024C	LTE1	YFR010W	UBP6
YML109W	ZDS2	YPL161C	BEM4
YKL139W	CTK1	YLR371W	ROM2
YBR194W	SOY1	YKL160W	ELF1
YMR153W	NUP53	YOR035C	SHE4

Predictions at threshold level 0.75 continued...			
Gene1 of predicted pair		Gene2 of predicted pair	
Systematic Name	Standard Name	Systematic Name	Standard Name
YMR192W	GYL1	YPR154W	PIN3
YPL115C	BEM3	YBR200W	BEM1
YOR038C	HIR2	YAL002W	VPS8
YKL139W	CTK1	YNL031C	HHT2
YNL199C	GCR2	YOR124C	UBP2
YML117W	NAB6	YPL089C	RLM1
YMR127C	SAS2	YDR310C	SUM1
YIL103W	DPH1	YDR183W	PLP1
YLR386W	VAC14	YMR230W	RPS10B
YBL058W	SHP1	YPL057C	SUR1
YOR195W	SLK19	YGR184C	UBR1
YGL025C	PGD1	YMR133W	REC114
YPL137C	GIP3	YDR358W	GGA1
YOL030W	GAS5	YML028W	TSA1
YMR199W	CLN1	YPR120C	CLB5
YKL048C	ELM1	YMR230W	RPS10B
YDR245W	MNN10	YHR158C	KEL1
YNL307C	MCK1	YCR086W	CSM1
YML001W	YPT7	YDL006W	PTC1
YML013W	UBX2	YDL155W	CLB3
YDL006W	PTC1	YAR014C	BUD14
YEL043W	YEL043W	YDR310C	SUM1
YMR026C	PEX12	YOR327C	SNC2
YHR081W	LRP1	YOR035C	SHE4
YKL126W	YPK1	YPR036W	VMA13
YDR264C	AKR1	YKL101W	HSL1
YKR099W	BAS1	YGL124C	MON1
YMR224C	MRE11	YOR032C	HMS1
YHR111W	UBA4	YPL018W	CTF19

Predictions at threshold level 0.75 continued...			
Gene1 of predicted pair		Gene2 of predicted pair	
Systematic Name	Standard Name	Systematic Name	Standard Name
YDL184C	RPL41A	YMR133W	REC114
YMR133W	REC114	YML071C	COG8
YBL019W	APN2	YHR056C	RSC30
YDR261C	EXG2	YBR019C	GAL10
YDR129C	SAC6	YFR040W	SAP155
YGR072W	UPF3	YPL120W	VPS30
YOR112W	CEX1	YDR202C	RAV2
YOR358W	HAP5	YDR183W	PLP1
YIL034C	CAP2	YPR051W	MAK3
YML071C	COG8	YEL060C	PRB1
YBR089C-A	NHP6B	YLR357W	RSC2
YKR101W	SIR1	YNL107W	YAF9
YMR133W	REC114	YPL195W	APL5
YDL116W	NUP84	YIR005W	IST3
YDL059C	RAD59	YJR035W	RAD26
YBR108W	YBR108W	YMR214W	SCJ1
YER116C	SLX8	YMR055C	BUB2
YNL025C	SSN8	YDR335W	MSN5
YNL135C	FPR1	YGL212W	VAM7
YBL103C	RTG3	YLR094C	GIS3
YCR065W	HCM1	YJR060W	CBF1
YJL095W	BCK1	YLR233C	EST1
YOR212W	STE4	YPL161C	BEM4
YFR031C-A	RPL2A	YPL024W	RMI1
YGR252W	GCN5	YOR275C	RIM20
YBR200W	BEM1	YMR152W	YIM1
YMR216C	SKY1	YCL001W	RER1
YKL068W	NUP100	YGR059W	SPR3
YDR245W	MNN10	YLR315W	NKP2

Predictions at threshold level 0.75 continued...			
Gene1 of predicted pair		Gene2 of predicted pair	
Systematic Name	Standard Name	Systematic Name	Standard Name
YAL024C	LTE1	YGL025C	PGD1
YLR144C	ACF2	YPL249C	GYP5
YBR173C	UMP1	YML115C	VAN1
YDR293C	SSD1	YMR055C	BUB2
YAL024C	LTE1	YKL080W	VMA5
YHR156C	LIN1	YBR019C	GAL10
YDR439W	LRS4	YIL103W	DPH1
YDL006W	PTC1	YLR398C	SKI2
YOR076C	SKI7	YCL037C	SRO9
YDL042C	SIR2	YMR273C	ZDS1
YOR275C	RIM20	YKL002W	DID4
YLR447C	VMA6	YMR054W	STV1
YNL044W	YIP3	YOL018C	TLG2
YKL116C	PRR1	YER116C	SLX8
YKL048C	ELM1	YHL002W	HSE1
YGR004W	PEX31	YBR168W	PEX32
YMR273C	ZDS1	YDR309C	GIC2
YIL053W	RHR2	YLR441C	RPS1A
YGR166W	KRE11	YEL022W	GEA2
YMR205C	PFK2	YJL117W	PHO86
YDR477W	SNF1	YOR043W	WHI2
YML016C	PPZ1	YOR368W	RAD17
YMR116C	ASC1	YNL079C	TPM1
YDR254W	CHL4	YJR135C	MCM22
YKL116C	PRR1	YFL023W	BUD27
YMR016C	SOK2	YGL115W	SNF4
YDR254W	CHL4	YDL226C	GCS1
YIL034C	CAP2	YCR088W	ABP1
YLR386W	VAC14	YJL136C	RPS21B

Predictions at threshold level 0.75 continued...			
Gene1 of predicted pair		Gene2 of predicted pair	
Systematic Name	Standard Name	Systematic Name	Standard Name
YGL115W	SNF4	YJL004C	SYS1
YAL051W	OAF1	YGL161C	YIP5
YNL068C	FKH2	YKL101W	HSL1
YLR386W	VAC14	YGL031C	RPL24A
YKL074C	MUD2	YLR357W	RSC2
YAL024C	LTE1	YMR026C	PEX12
YLR079W	SIC1	YJR060W	CBF1
YOL111C	MDY2	YFR036W	CDC26
YKR001C	VPS1	YNL107W	YAF9
YNL291C	MID1	YNL117W	MLS1
YLR095C	IOC2	YFR031C-A	RPL2A
YDR443C	SSN2	YDL160C	DHH1
YLR095C	IOC2	YMR214W	SCJ1
YOL121C	RPS19A	YBR280C	SAF1
YOR308C	SNU66	YBR036C	CSG2
YPL024W	RMI1	YDR207C	UME6
YLR337C	VRP1	YLR381W	CTF3
YPR036W	VMA13	YPL037C	EGD1
YJR024C	MDE1	YGR059W	SPR3
YKL126W	YPK1	YNL079C	TPM1
YDR329C	PEX3	YGR077C	PEX8
YLR090W	XDJ1	YLR370C	ARC18
YGL054C	ERV14	YNL041C	COG6
YPR084W	YPR084W	YKR020W	VPS51
YMR198W	CIK1	YDR207C	UME6
YDR334W	SWR1	YGL094C	PAN2
YLR095C	IOC2	YDR484W	VPS52
YGR135W	PRE9	YBL087C	RPL23A
YKL116C	PRR1	YJR032W	CPR7

Predictions at threshold level 0.75 continued...			
Gene1 of predicted pair		Gene2 of predicted pair	
Systematic Name	Standard Name	Systematic Name	Standard Name
YNL201C	PSY2	YBL046W	PSY4
YLR429W	CRN1	YJR135C	MCM22
YKL010C	UFD4	YHL002W	HSE1
YJL053W	PEP8	YOR035C	SHE4
YER162C	RAD4	YNR032W	PPG1
YOR132W	VPS17	YNL044W	YIP3
YJL080C	SCP160	YOR198C	BFR1
YML016C	PPZ1	YOL054W	PSH1
YLR386W	VAC14	YLR441C	RPS1A
YNL041C	COG6	YER139C	YER139C
YJR083C	ACF4	YHR016C	YSC84
YNL068C	FKH2	YGL163C	RAD54
YPL112C	PEX25	YPR154W	PIN3
YPL057C	SUR1	YNL307C	MCK1
YKR061W	KTR2	YFR030W	MET10
YGL124C	MON1	YLR180W	SAM1
YPR171W	BSP1	YIL034C	CAP2
YKL048C	ELM1	YEL053C	MAK10
YLR083C	EMP70	YMR004W	MVP1
YLR337C	VRP1	YJR032W	CPR7
YDR443C	SSN2	YGL078C	DBP3
YOR083W	WHI5	YJL006C	CTK2
YOR290C	SNF2	YDR261C	EXG2
YML071C	COG8	YLR309C	IMH1
YDL116W	NUP84	YLR254C	NDL1
YLR018C	POM34	YDR192C	NUP42
YHL020C	OPI1	YPR119W	CLB2
YJL176C	SWI3	YOR038C	HIR2
YJR082C	EAF6	YNL107W	YAF9

Predictions at threshold level 0.75 continued...			
Gene1 of predicted pair		Gene2 of predicted pair	
Systematic Name	Standard Name	Systematic Name	Standard Name
YLR399C	BDF1	YDR318W	MCM21
YNL201C	PSY2	YDR174W	HMO1
YPR040W	TIP41	YJL187C	SWE1
YBL047C	EDE1	YOR014W	RTS1
YPL037C	EGD1	YGL003C	CDH1
YDR383C	NKP1	YBR107C	IML3
YBL058W	SHP1	YDR363W-A	SEM1
YDR320C	SWA2	YMR054W	STV1
YDR156W	RPA14	YFR030W	MET10
YKL126W	YPK1	YBL067C	UBP13
YNL147W	LSM7	YNL107W	YAF9
YKL025C	PAN3	YAL002W	VPS8
YBR173C	UMP1	YDR484W	VPS52
YER122C	GLO3	YNR032W	PPG1
YJR060W	CBF1	YDR289C	RTT103
YMR055C	BUB2	YIL103W	DPH1
YDL059C	RAD59	YGL163C	RAD54
YNL291C	MID1	YMR004W	MVP1
YKL116C	PRR1	YFR036W	CDC26
YBR111W-A	SUS1	YOR141C	ARP8
YPL050C	MNN9	YDR310C	SUM1
YBR194W	SOY1	YLR315W	NKP2
YNL238W	KEX2	YOR327C	SNC2
YDL005C	MED2	YBR010W	HHT1
YAR064W	YAR064W	YPL240C	HSP82
YDR080W	VPS41	YGL115W	SNF4
YNL307C	MCK1	YHR060W	VMA22
YOR018W	ROD1	YFR043C	IRC6
YLR386W	VAC14	YGR054W	YGR054W

Predictions at threshold level 0.75 continued...			
Gene1 of predicted pair		Gene2 of predicted pair	
Systematic Name	Standard Name	Systematic Name	Standard Name
YBR274W	CHK1	YLR350W	ORM2
YHR124W	NDT80	YJL106W	IME2
YNL238W	KEX2	YMR230W	RPS10B
YPL152W	RRD2	YFR030W	MET10
YMR055C	BUB2	YGR135W	PRE9
YFR034C	PHO4	YOL108C	INO4
YKR057W	RPS21A	YDR363W-A	SEM1
YBR107C	IML3	YPR046W	MCM16
YMR026C	PEX12	YBR036C	CSG2
YKL135C	APL2	YAL030W	SNC1
YEL056W	HAT2	YOR368W	RAD17
YDR254W	CHL4	YHR013C	ARD1
YMR055C	BUB2	YHR061C	GIC1
YKR035W-A	DID2	YNL238W	KEX2
YGL237C	HAP2	YDR310C	SUM1
YDR443C	SSN2	YLR180W	SAM1
YBL047C	EDE1	YPL161C	BEM4
YDR146C	SWI5	YGL025C	PGD1
YBR200W	BEM1	YGR054W	YGR054W
YDL077C	VAM6	YER040W	GLN3
YHR060W	VMA22	YMR133W	REC114
YOR080W	DIA2	YAL055W	PEX22
YLR386W	VAC14	YOR039W	CKB2
YOR106W	VAM3	YMR231W	PEP5
YHL011C	PRS3	YKL161C	YKL161C
YJL154C	VPS35	YHR012W	VPS29
YJL176C	SWI3	YML071C	COG8
YBR171W	SEC66	YFR040W	SAP155
YGL237C	HAP2	YLR233C	EST1

Predictions at threshold level 0.75 continued...			
Gene1 of predicted pair		Gene2 of predicted pair	
Systematic Name	Standard Name	Systematic Name	Standard Name
YOR270C	VPH1	YDR261C	EXG2
YMR026C	PEX12	YMR214W	SCJ1
YLR381W	CTF3	YOL081W	IRA2
YFR036W	CDC26	YDR318W	MCM21
YMR116C	ASC1	YBR131W	CCZ1
YER116C	SLX8	YKL048C	ELM1
YLR192C	HCR1	YIL071C	PCI8
YMR026C	PEX12	YNL087W	TCB2
YLR176C	RFX1	YGR252W	GCN5
YJR135C	MCM22	YDR144C	MKC7
YMR242C	RPL20A	YGL246C	RAI1
YNL094W	APP1	YJL004C	SYS1
YLR102C	APC9	YMR199W	CLN1
YIL008W	URM1	YHL002W	HSE1
YOR112W	CEX1	YGL054C	ERV14
YDR080W	VPS41	YLR396C	VPS33
YGL151W	NUT1	YGL253W	HXK2
YKL126W	YPK1	YLR320W	MMS22
YLR018C	POM34	YJR059W	PTK2
YFR010W	UBP6	YOR014W	RTS1
YBR171W	SEC66	YDR155C	CPR1
YMR172W	HOT1	YGR019W	UGA1
YOR270C	VPH1	YHR060W	VMA22
YNL076W	MKS1	YER122C	GLO3
YJL053W	PEP8	YDL006W	PTC1
YEL065W	SIT1	YML071C	COG8
YMR199W	CLN1	YDR363W-A	SEM1
YLL039C	UBI4	YDL192W	ARF1
YBL058W	SHP1	YMR026C	PEX12

Predictions at threshold level 0.75 continued...			
Gene1 of predicted pair		Gene2 of predicted pair	
Systematic Name	Standard Name	Systematic Name	Standard Name
YOR076C	SKI7	YDL020C	RPN4
YML103C	NUP188	YGL100W	SEH1
YEL050C	RML2	YDR363W-A	SEM1
YDR137W	RGP1	YOR327C	SNC2
YCL011C	GBP2	YHR167W	THP2
YJL136C	RPS21B	YPL256C	CLN2
YFL023W	BUD27	YJR084W	CSN12
YMR016C	SOK2	YNL238W	KEX2
YPL037C	EGD1	YOR043W	WHI2
YPL001W	HAT1	YPR120C	CLB5
YGR019W	UGA1	YLR234W	TOP3
YBL067C	UBP13	YBL072C	RPS8A
YER116C	SLX8	YDR261C	EXG2
YOR112W	CEX1	YJR060W	CBF1
YNL079C	TPM1	YHR108W	GGA2
YGR261C	APL6	YOL018C	TLG2
YKL032C	IXR1	YGL163C	RAD54
YOR337W	TEA1	YJR056C	YJR056C
YNL087W	TCB2	YBL025W	RRN10
YOR127W	RGA1	YPL234C	TFP3
YDL052C	SLC1	YMR152W	YIM1
YLR135W	SLX4	YLR410W	VIP1
YDL127W	PCL2	YKL032C	IXR1
YML016C	PPZ1	YDL185W	TFP1
YDR379W	RGA2	YPL256C	CLN2
YMR153W	NUP53	YER122C	GLO3
YJR140C	HIR3	YDR137W	RGP1
YBL058W	SHP1	YER044C	ERG28
YLR381W	CTF3	YLR182W	SWI6

Predictions at threshold level 0.75 continued...			
Gene1 of predicted pair		Gene2 of predicted pair	
Systematic Name	Standard Name	Systematic Name	Standard Name
YDR295C	HDA2	YLR131C	ACE2
YOR066W	MSA1	YER162C	RAD4
YBR171W	SEC66	YJR135C	MCM22
YBR057C	MUM2	YER116C	SLX8
YML115C	VAN1	YEL027W	CUP5
YBL003C	HTA2	YBR111W-A	SUS1
YJR140C	HIR3	YOR368W	RAD17
YDR245W	MNN10	YOR312C	RPL20B
YMR167W	MLH1	YDR310C	SUM1
YOR308C	SNU66	YMR038C	CCS1
YCL067C	HMLALPHA2	YAL040C	CLN3
YLR309C	IMH1	YPR032W	SRO7
YIL034C	CAP2	YOR127W	RGA1
YLR313C	SPH1	YCR086W	CSM1
YFL023W	BUD27	YOR368W	RAD17
YDR378C	LSM6	YIR005W	IST3
YOR327C	SNC2	YMR017W	SPO20
YDR254W	CHL4	YIL008W	URM1
YHR079C	IRE1	YDR225W	HTA1
YLR360W	VPS38	YMR055C	BUB2
YBR278W	DPB3	YPR120C	CLB5
YJL172W	CPS1	YJR032W	CPR7
YPL161C	BEM4	YPL213W	LEA1
YJR032W	CPR7	YLR093C	NYV1
YMR216C	SKY1	YPR051W	MAK3
YGL133W	ITC1	YDR379W	RGA2
YNL248C	RPA49	YFR030W	MET10
YDR129C	SAC6	YEL044W	IES6
YOR290C	SNF2	YNL031C	HHT2

Predictions at threshold level 0.75 continued...			
Gene1 of predicted pair		Gene2 of predicted pair	
Systematic Name	Standard Name	Systematic Name	Standard Name
YNL307C	MCK1	YGL163C	RAD54
YLR085C	ARP6	YBR089C-A	NHP6B
YLR360W	VPS38	YOL018C	TLG2
YMR095C	SNO1	YOR066W	MSA1
YML115C	VAN1	YDR261C	EXG2
YPL240C	HSP82	YNL220W	ADE12
YGR108W	CLB1	YLR410W	VIP1
YMR109W	MYO5	YDL155W	CLB3
YPR036W	VMA13	YMR152W	YIM1
YDR477W	SNF1	YLR398C	SKI2
YNR052C	POP2	YDR207C	UME6
YDR254W	CHL4	YEL053C	MAK10
YNL106C	INP52	YOL054W	PSH1
YER179W	DMC1	YCL014W	BUD3
YML117W	NAB6	YDL006W	PTC1
YJL190C	RPS22A	YHL002W	HSE1
YGL100W	SEH1	YNL076W	MKS1
YPR084W	YPR084W	YPR032W	SRO7
YLR315W	NKP2	YGL252C	RTG2
YDR507C	GIN4	YML095C	RAD10
YLR227C	ADY4	YOL091W	SPO21
YPL161C	BEM4	YPL195W	APL5
YHR156C	LIN1	YJR032W	CPR7
YAL024C	LTE1	YMR214W	SCJ1
YPR046W	MCM16	YMR152W	YIM1
YLR095C	IOC2	YPR030W	CSR2
YDL154W	MSH5	YLR394W	CST9
YPL024W	RMI1	YDR254W	CHL4
YBL103C	RTG3	YEL060C	PRB1

Predictions at threshold level 0.75 continued...			
Gene1 of predicted pair		Gene2 of predicted pair	
Systematic Name	Standard Name	Systematic Name	Standard Name
YDL184C	RPL41A	YNL058C	YNL058C
YNL307C	MCK1	YNL225C	CNM67
YLR360W	VPS38	YOR132W	VPS17
YPL042C	SSN3	YPL248C	GAL4
YDR448W	ADA2	YCL010C	SGF29
YBL103C	RTG3	YDR207C	UME6
YGR072W	UPF3	YPL037C	EGD1
YNL004W	HRB1	YHR167W	THP2
YOR132W	VPS17	YDR153C	ENT5
YGR054W	YGR054W	YJL183W	MNN11
YMR095C	SNO1	YKR099W	BAS1
YOR112W	CEX1	YLR309C	IMH1
YPR018W	RLF2	YBR107C	IML3
YGR270W	YTA7	YOR141C	ARP8
YBR127C	VMA2	YJR033C	RAV1
YKL073W	LHS1	YMR022W	UBC7
YLR090W	XDJ1	YBR171W	SEC66
YNL297C	MON2	YNL044W	YIP3
YKL110C	KTI12	YOR141C	ARP8
YFR036W	CDC26	YCR086W	CSM1
YMR224C	MRE11	YOR290C	SNF2
YDR156W	RPA14	YJR063W	RPA12
YJR040W	GEF1	YHL020C	OPI1
YLR254C	NDL1	YHR115C	DMA1
YNL032W	SIW14	YLR441C	RPS1A
YFR010W	UBP6	YER162C	RAD4
YHR156C	LIN1	YPL161C	BEM4
YMR133W	REC114	YLR370C	ARC18
YLR410W	VIP1	YIR005W	IST3

Predictions at threshold level 0.75 continued...			
Gene1 of predicted pair		Gene2 of predicted pair	
Systematic Name	Standard Name	Systematic Name	Standard Name
YLR386W	VAC14	YML001W	YPT7
YPL057C	SUR1	YER122C	GLO3
YIL008W	URM1	YCR086W	CSM1
YKR061W	KTR2	YNL107W	YAF9
YOR127W	RGA1	YNR032W	PPG1
YBL003C	HTA2	YGR252W	GCN5
YJL183W	MNN11	YDR310C	SUM1
YOR033C	EXO1	YPR046W	MCM16
YKL048C	ELM1	YHR111W	UBA4
YNL199C	GCR2	YBL079W	NUP170
YPR040W	TIP41	YKR048C	NAP1
YNL201C	PSY2	YKL110C	KTI12
YMR284W	YKU70	YBR082C	UBC4
YDR293C	SSD1	YLR234W	TOP3
YFR036W	CDC26	YGR188C	BUB1
YOR127W	RGA1	YOL067C	RTG1
YDR103W	STE5	YDR480W	DIG2
YBR194W	SOY1	YDR440W	DOT1
YOR290C	SNF2	YBR036C	CSG2
YOR358W	HAP5	YLR233C	EST1
YKL126W	YPK1	YDL077C	VAM6
YML115C	VAN1	YOR290C	SNF2
YNL307C	MCK1	YOR141C	ARP8
YGR249W	MGA1	YER040W	GLN3
YIR005W	IST3	YDR440W	DOT1
YDR245W	MNN10	YKL213C	DOA1
YIL007C	NAS2	YLR024C	UBR2
YDR320C	SWA2	YLR206W	ENT2
YLR109W	AHP1	YPR167C	MET16

Predictions at threshold level 0.75 continued...			
Gene1 of predicted pair		Gene2 of predicted pair	
Systematic Name	Standard Name	Systematic Name	Standard Name
YDL059C	RAD59	YDR207C	UME6
YKR061W	KTR2	YKL110C	KTI12
YKL139W	CTK1	YLR350W	ORM2
YDR261C	EXG2	YEL060C	PRB1
YBL003C	HTA2	YBR010W	HHT1
YGR241C	YAP1802	YDR484W	VPS52
YBR290W	BSD2	YBR108W	YBR108W
YOR069W	VPS5	YOR109W	INP53
YNL106C	INP52	YHR077C	NMD2
YOR083W	WHI5	YGL025C	PGD1
YNL215W	IES2	YHR178W	STB5
YGL087C	MMS2	YKL101W	HSL1
YDR137W	RGP1	YMR230W	RPS10B
YMR095C	SNO1	YNL076W	MKS1
YPL037C	EGD1	YBR280C	SAF1
YNL288W	CAF40	YAR014C	BUD14
YGR041W	BUD9	YLR353W	BUD8
YPR052C	NHP6A	YML062C	MFT1
YLR058C	SHM2	YKL114C	APN1
YDR108W	GSG1	YLR309C	IMH1
YPR131C	NAT3	YJL006C	CTK2
YIL076W	SEC28	YDR440W	DOT1

Predicted pairs at a threshold level of 0.80

Predictions at threshold level 0.80			
Gene1 of predicted pair		Gene2 of predicted pair	
Systematic Name	Standard Name	Systematic Name	Standard Name
YGL025C	PGD1	YJR032W	CPR7
YNL199C	GCR2	YGR059W	SPR3
YLR377C	FBP1	YDR028C	REG1
YDL116W	NUP84	YNL236W	SIN4
YNL054W	VAC7	YDR378C	LSM6
YKL038W	RGT1	YOR047C	STD1
YGR108W	CLB1	YDL155W	CLB3
YJL136C	RPS21B	YJL095W	BCK1
YMR095C	SNO1	YNL032W	SIW14
YLR435W	TSR2	YGL019W	CKB1
YHR111W	UBA4	YGR270W	YTA7
YBL102W	SFT2	YJR040W	GEF1
YBL008W	HIR1	YML115C	VAN1
YNR072W	HXT17	YLR180W	SAM1
YCR081W	SRB8	YMR311C	GLC8
YLR094C	GIS3	YPL137C	GIP3
YOR275C	RIM20	YLR320W	MMS22
YOR043W	WHI2	YDL077C	VAM6
YJL183W	MNN11	YKL119C	VPH2
YHR056C	RSC30	YPR119W	CLB2
YGR014W	MSB2	YKL041W	VPS24
YEL027W	CUP5	YHR039C-A	VMA10
YBR131W	CCZ1	YBR041W	FAT1
YFR031C-A	RPL2A	YHR081W	LRP1
YFR036W	CDC26	YNL107W	YAF9
YDR137W	RGP1	YOR038C	HIR2
YOR358W	HAP5	YAL002W	VPS8
YBL051C	PIN4	YDR183W	PLP1

Predictions at threshold level 0.80 continued...			
Gene1 of predicted pair		Gene2 of predicted pair	
Systematic Name	Standard Name	Systematic Name	Standard Name
YBL102W	SFT2	YNL291C	MID1
YMR109W	MYO5	YLR233C	EST1
YCR077C	PAT1	YGR188C	BUB1
YER161C	SPT2	YDR440W	DOT1
YMR080C	NAM7	YHR077C	NMD2
YLR043C	TRX1	YDR423C	CAD1
YDR456W	NHX1	YNL199C	GCR2
YPL057C	SUR1	YBR019C	GAL10
YDL161W	ENT1	YNR006W	VPS27
YLR288C	MEC3	YML016C	PPZ1
YLR018C	POM34	YDR207C	UME6
YGL003C	CDH1	YMR214W	SCJ1
YOR080W	DIA2	YDL047W	SIT4
YMR205C	PFK2	YKR020W	VPS51
YMR284W	YKU70	YER116C	SLX8
YKR001C	VPS1	YLR182W	SWI6
YHR108W	GGA2	YDL192W	ARF1
YBR009C	HHF1	YOR064C	YNG1
YOR270C	VPH1	YNL307C	MCK1
YNL054W	VAC7	YJR060W	CBF1
YPR072W	NOT5	YJR063W	RPA12
YLR292C	SEC72	YOR023C	AHC1
YDR443C	SSN2	YBR019C	GAL10
YDR507C	GIN4	YOR327C	SNC2
YJL187C	SWE1	YCR086W	CSM1
YLR360W	VPS38	YLR079W	SIC1
YJL154C	VPS35	YDL192W	ARF1
YGL163C	RAD54	YOR038C	HIR2
YLR234W	TOP3	YNL206C	RTT106

Predictions at threshold level 0.80 continued...			
Gene1 of predicted pair		Gene2 of predicted pair	
Systematic Name	Standard Name	Systematic Name	Standard Name
YBR258C	SHG1	YGL237C	HAP2
YPL178W	CBC2	YKL068W	NUP100
YGR071C	YGR071C	YGR188C	BUB1
YLR386W	VAC14	YER116C	SLX8
YBR042C	CST26	YDR358W	GGA1
YBL079W	NUP170	YKR024C	DBP7
YLR216C	CPR6	YFR036W	CDC26
YHR056C	RSC30	YKL113C	RAD27
YBR107C	IML3	YNL147W	LSM7
YPR120C	CLB5	YLR315W	NKP2
YBR173C	UMP1	YGL115W	SNF4
YBR042C	CST26	YDR137W	RGP1
YGR070W	ROM1	YOL081W	IRA2
YEL065W	SIT1	YEL060C	PRB1
YDR129C	SAC6	YDR310C	SUM1
YMR198W	CIK1	YPL180W	TCO89
YGL208W	SIP2	YJL187C	SWE1
YBL103C	RTG3	YER040W	GLN3
YOR270C	VPH1	YBR171W	SEC66
YGL033W	HOP2	YDL192W	ARF1
YOR337W	TEA1	YJL095W	BCK1
YJR063W	RPA12	YOR064C	YNG1
YGR261C	APL6	YJL053W	PEP8
YGL025C	PGD1	YOR327C	SNC2
YJL124C	LSM1	YBL047C	EDE1
YBR200W	BEM1	YBL021C	HAP3
YJL006C	CTK2	YLR182W	SWI6
YDR335W	MSN5	YDR318W	MCM21
YPL105C	YPL105C	YOR327C	SNC2

Predictions at threshold level 0.80 continued...			
Gene1 of predicted pair		Gene2 of predicted pair	
Systematic Name	Standard Name	Systematic Name	Standard Name
YOR080W	DIA2	YJR135C	MCM22
YDR320C	SWA2	YGR105W	VMA21
YDL020C	RPN4	YGR270W	YTA7
YPL024W	RMI1	YPR036W	VMA13
YBR274W	CHK1	YGR188C	BUB1
YFR031C-A	RPL2A	YAL002W	VPS8
YDR012W	RPL4B	YOR078W	BUD21
YOR127W	RGA1	YML001W	YPT7
YDR260C	SWM1	YGR135W	PRE9
YNL236W	SIN4	YER068W	MOT2
YKL126W	YPK1	YGL212W	VAM7
YOL018C	TLG2	YOR036W	PEP12
YDR383C	NKP1	YJL006C	CTK2
YNR052C	POP2	YLR315W	NKP2
YER116C	SLX8	YPL037C	EGD1
YLR079W	SIC1	YNL116W	DMA2
YER044C	ERG28	YOR035C	SHE4
YOR290C	SNF2	YBR019C	GAL10
YDL223C	HBT1	YML071C	COG8
YPR017C	DSS4	YKR001C	VPS1
YPL042C	SSN3	YOR038C	HIR2
YNR072W	HXT17	YDL192W	ARF1
YCR020C-A	MAK31	YNL238W	KEX2
YBR194W	SOY1	YJL183W	MNN11
YPL024W	RMI1	YDR358W	GGA1
YDL020C	RPN4	YAL002W	VPS8
YDR320C	SWA2	YCR066W	RAD18
YBR173C	UMP1	YOR368W	RAD17
YHL020C	OPI1	YNR032W	PPG1

Predictions at threshold level 0.80 continued...			
Gene1 of predicted pair		Gene2 of predicted pair	
Systematic Name	Standard Name	Systematic Name	Standard Name
YBR030W	YBR030W	YOL121C	RPS19A
YNL238W	KEX2	YHR108W	GGA2
YDL013W	SLX5	YDL155W	CLB3
YPL137C	GIP3	YER162C	RAD4
YPL050C	MNN9	YCR088W	ABP1
YER110C	KAP123	YPL246C	RBD2
YJL117W	PHO86	YPL089C	RLM1
YOL067C	RTG1	YAR014C	BUD14
YOR039W	CKB2	YCR031C	RPS14A
YML095C	RAD10	YDR318W	MCM21
YNL054W	VAC7	YNL248C	RPA49
YIL076W	SEC28	YDR320C	SWA2
YLR240W	VPS34	YMR202W	ERG2
YOR132W	VPS17	YPL234C	TFP3
YBR017C	KAP104	YKL057C	NUP120
YCL001W	RER1	YHR012W	VPS29
YDR310C	SUM1	YNR032W	PPG1
YMR224C	MRE11	YKR020W	VPS51
YNL087W	TCB2	YER040W	GLN3
YPL042C	SSN3	YPL057C	SUR1
YDL127W	PCL2	YDR289C	RTT103
YML103C	NUP188	YPL018W	CTF19
YIL053W	RHR2	YPL195W	APL5
YBL078C	ATG8	YBR131W	CCZ1
YDL065C	PEX19	YGL153W	PEX14
YOL051W	GAL11	YBR010W	HHT1
YIL036W	CST6	YLR399C	BDF1
YER161C	SPT2	YDR310C	SUM1
YMR199W	CLN1	YPR119W	CLB2

Predictions at threshold level 0.80 continued...			
Gene1 of predicted pair		Gene2 of predicted pair	
Systematic Name	Standard Name	Systematic Name	Standard Name
YMR167W	MLH1	YPR119W	CLB2
YNL054W	VAC7	YMR055C	BUB2
YNL087W	TCB2	YJR084W	CSN12
YOL121C	RPS19A	YOR035C	SHE4
YBR111W-A	SUS1	YLR399C	BDF1
YLR386W	VAC14	YGL212W	VAM7
YMR284W	YKU70	YLR313C	SPH1
YOR109W	INP53	YDR207C	UME6
YNL032W	SIW14	YMR016C	SOK2
YGL133W	ITC1	YLR102C	APC9
YMR224C	MRE11	YOR312C	RPL20B
YHR039C-A	VMA10	YDL083C	RPS16B
YPL105C	YPL105C	YNL051W	COG5
YDR378C	LSM6	YGL003C	CDH1
YMR273C	ZDS1	YML057W	CMP2
YMR172W	HOT1	YNL288W	CAF40
YKR020W	VPS51	YNL289W	PCL1
YJR032W	CPR7	YOL018C	TLG2
YKR099W	BAS1	YMR311C	GLC8
YGL163C	RAD54	YHR115C	DMA1
YDL155W	CLB3	YPL018W	CTF19
YML099C	ARG81	YDR207C	UME6
YGL212W	VAM7	YCL001W	RER1
YKL032C	IXR1	YNL116W	DMA2
YMR055C	BUB2	YPL152W	RRD2
YDR440W	DOT1	YGL003C	CDH1
YJR135C	MCM22	YER122C	GLO3
YNL032W	SIW14	YPL057C	SUR1
YDR225W	HTA1	YBL003C	HTA2

Predictions at threshold level 0.80 continued...			
Gene1 of predicted pair		Gene2 of predicted pair	
Systematic Name	Standard Name	Systematic Name	Standard Name
YKR035W-A	DID2	YEL060C	PRB1
YGL115W	SNF4	YOL091W	SPO21
YMR224C	MRE11	YKL126W	YPK1
YLR381W	CTF3	YNL166C	BNI5
YHR119W	SET1	YBR175W	SWD3
YBR030W	YBR030W	YMR152W	YIM1
YFR031C-A	RPL2A	YFR010W	UBP6
YER115C	SPR6	YMR273C	ZDS1
YCR020C-A	MAK31	YOR069W	VPS5
YLR337C	VRP1	YGL124C	MON1
YDL161W	ENT1	YJR125C	ENT3
YMR016C	SOK2	YLR370C	ARC18
YEL065W	SIT1	YFR036W	CDC26
YMR199W	CLN1	YBL087C	RPL23A
YMR116C	ASC1	YGL153W	PEX14
YDR080W	VPS41	YHR012W	VPS29
YDR156W	RPA14	YOR275C	RIM20
YDR004W	RAD57	YFR036W	CDC26
YML038C	YMD8	YDR153C	ENT5
YLR095C	IOC2	YDR310C	SUM1
YLR079W	SIC1	YOR195W	SLK19
YGR184C	UBR1	YDL013W	SLX5
YDR395W	SXM1	YKL068W	NUP100
YPR131C	NAT3	YDR192C	NUP42
YIL159W	BNR1	YOR337W	TEA1
YPL253C	VIK1	YHR115C	DMA1
YER114C	BOI2	YHR061C	GIC1
YDR320C	SWA2	YJR040W	GEF1
YLR094C	GIS3	YGL003C	CDH1

Predictions at threshold level 0.80 continued...			
Gene1 of predicted pair		Gene2 of predicted pair	
Systematic Name	Standard Name	Systematic Name	Standard Name
YFL047W	RGD2	YPL246C	RBD2
YHR039C-A	VMA10	YDR207C	UME6
YJL006C	CTK2	YOR141C	ARP8
YGL078C	DBP3	YEL060C	PRB1
YGR020C	VMA7	YER162C	RAD4
YFR030W	MET10	YJL006C	CTK2
YLR079W	SIC1	YMR199W	CLN1
YER116C	SLX8	YLR093C	NYV1
YJR056C	YJR056C	YGL003C	CDH1
YPL046C	ELC1	YNL230C	ELA1
YLR423C	ATG17	YPR119W	CLB2
YMR125W	STO1	YML057W	CMP2
YDL020C	RPN4	YOR358W	HAP5
YDL192W	ARF1	YEL042W	GDA1
YHR052W	CIC1	YLR325C	RPL38
YOR195W	SLK19	YMR055C	BUB2
YPL105C	YPL105C	YLR309C	IMH1
YLR119W	SRN2	YNL058C	YNL058C
YJL138C	TIF2	YDR310C	SUM1
YOR127W	RGA1	YDR144C	MKC7
YIL103W	DPH1	YGR188C	BUB1
YDL116W	NUP84	YLR313C	SPH1
YNR052C	POP2	YMR038C	CCS1
YBR036C	CSG2	YMR230W	RPS10B
YMR095C	SNO1	YER040W	GLN3
YPR030W	CSR2	YDR358W	GGA1
YMR224C	MRE11	YGL252C	RTG2
YBR171W	SEC66	YHR200W	RPN10
YLR381W	CTF3	YJR060W	CBF1

Predictions at threshold level 0.80 continued...			
Gene1 of predicted pair		Gene2 of predicted pair	
Systematic Name	Standard Name	Systematic Name	Standard Name
YBR164C	ARL1	YEL022W	GEA2
YDR477W	SNF1	YBR171W	SEC66
YLR399C	BDF1	YML112W	CTK3
YFR031C-A	RPL2A	YFL023W	BUD27
YDR363W-A	SEM1	YGR188C	BUB1
YCR066W	RAD18	YAL015C	NTG1
YOR127W	RGA1	YML095C	RAD10
YBR057C	MUM2	YEL036C	ANP1
YOR290C	SNF2	YKL109W	HAP4
YBL051C	PIN4	YLR447C	VMA6
YKL101W	HSL1	YOL081W	IRA2
YBL058W	SHP1	YMR133W	REC114
YOR112W	CEX1	YPL037C	EGD1
YBR030W	YBR030W	YHR039C-A	VMA10
YPR084W	YPR084W	YDR289C	RTT103
YOR039W	CKB2	YGR252W	GCN5
YPL147W	PXA1	YPL145C	KES1
YOR094W	ARF3	YCL034W	LSB5
YPR018W	RLF2	YCR086W	CSM1
YGL212W	VAM7	YIL133C	RPL16A
YLR381W	CTF3	YPL057C	SUR1
YPR018W	RLF2	YDR181C	SAS4
YMR055C	BUB2	YBR036C	CSG2
YDL226C	GCS1	YKL114C	APN1
YDL116W	NUP84	YDR076W	RAD55
YCL037C	SRO9	YNL297C	MON2
YDR443C	SSN2	YNL076W	MKS1
YOR059C	YOR059C	YKL094W	YJU3
YBL058W	SHP1	YNL307C	MCK1

Predictions at threshold level 0.80 continued...			
Gene1 of predicted pair		Gene2 of predicted pair	
Systematic Name	Standard Name	Systematic Name	Standard Name
YJR060W	CBF1	YDL226C	GCS1
YPL022W	RAD1	YML095C	RAD10
YOL111C	MDY2	YMR214W	SCJ1
YMR055C	BUB2	YOR014W	RTS1
YPR040W	TIP41	YOR327C	SNC2
YGL200C	EMP24	YML012W	ERV25
YML095C	RAD10	YJR135C	MCM22
YER116C	SLX8	YHR010W	RPL27A
YIL036W	CST6	YDL006W	PTC1
YOL111C	MDY2	YDR261C	EXG2
YBL101C	ECM21	YBL025W	RRN10
YJR084W	CSN12	YDR310C	SUM1
YGL124C	MON1	YCR053W	THR4
YDR477W	SNF1	YER096W	SHC1
YOR140W	SFL1	YDR207C	UME6
YDR358W	GGA1	YMR214W	SCJ1
YDL002C	NHP10	YEL013W	VAC8
YPR018W	RLF2	YOR033C	EXO1
YHR060W	VMA22	YNR032W	PPG1
YKL048C	ELM1	YHR115C	DMA1
YOL126C	MDH2	YKL117W	SBA1
YDR101C	ARX1	YBR267W	REI1
YNL215W	IES2	YDR254W	CHL4
YPL037C	EGD1	YOR327C	SNC2
YGL019W	CKB1	YLR350W	ORM2
YKL110C	KTI12	YLR182W	SWI6
YBL058W	SHP1	YOR035C	SHE4
YLR043C	TRX1	YER042W	MXR1
YNL307C	MCK1	YGR270W	YTA7

Predictions at threshold level 0.80 continued...			
Gene1 of predicted pair		Gene2 of predicted pair	
Systematic Name	Standard Name	Systematic Name	Standard Name
YKL048C	ELM1	YKR035W-A	DID2
YHR039C-A	VMA10	YOR327C	SNC2
YHR014W	SPO13	YIL002C	INP51
YKL048C	ELM1	YML071C	COG8
YPL024W	RMI1	YML115C	VAN1
YHL031C	GOS1	YDR137W	RGP1
YDL160C	DHH1	YKR095W	MLP1
YOR337W	TEA1	YBR171W	SEC66
YGR249W	MGA1	YLR452C	SST2
YNL307C	MCK1	YOR043W	WHI2
YBR107C	IML3	YGL252C	RTG2
YPL037C	EGD1	YBL025W	RRN10
YPR051W	MAK3	YKL048C	ELM1
YPL042C	SSN3	YDL002C	NHP10
YNR072W	HXT17	YER044C	ERG28
YNL031C	HHT2	YBL002W	HTB2
YDR254W	CHL4	YJR060W	CBF1
YKR048C	NAP1	YBR010W	HHT1
YOR066W	MSA1	YNL041C	COG6
YMR199W	CLN1	YPL161C	BEM4
YGR071C	YGR071C	YHR167W	THP2
YMR224C	MRE11	YJL124C	LSM1
YER122C	GLO3	YKR030W	GMH1
YGL178W	MPT5	YBR171W	SEC66
YLR386W	VAC14	YEL060C	PRB1
YMR116C	ASC1	YJL117W	PHO86
YJL127C	SPT10	YLR410W	VIP1
YBL047C	EDE1	YJL006C	CTK2
YGL223C	COG1	YEL043W	YEL043W

Predictions at threshold level 0.80 continued...			
Gene1 of predicted pair		Gene2 of predicted pair	
Systematic Name	Standard Name	Systematic Name	Standard Name
YDL006W	PTC1	YOL018C	TLG2
YGR105W	VMA21	YER122C	GLO3
YGR019W	UGA1	YBR171W	SEC66
YNL032W	SIW14	YER162C	RAD4
YBL008W	HIR1	YJL176C	SWI3
YDL020C	RPN4	YNL206C	RTT106
YDR484W	VPS52	YOR066W	MSA1
YCR065W	HCM1	YOL081W	IRA2
YNL106C	INP52	YML103C	NUP188
YOR132W	VPS17	YHL002W	HSE1
YNL087W	TCB2	YEL060C	PRB1
YOR043W	WHI2	YOL081W	IRA2
YAL024C	LTE1	YPL137C	GIP3
YLL039C	UBI4	YPL213W	LEA1
YBR173C	UMP1	YPL137C	GIP3
YJR140C	HIR3	YDR363W-A	SEM1
YBR114W	RAD16	YNL107W	YAF9
YNL069C	RPL16B	YJL036W	SNX4
YMR199W	CLN1	YKL161C	YKL161C
YAL024C	LTE1	YPL037C	EGD1
YNL180C	RHO5	YPL240C	HSP82
YDR289C	RTT103	YLR254C	NDL1
YLR094C	GIS3	YBR019C	GAL10
YMR055C	BUB2	YLR315W	NKP2
YPR029C	APL4	YDR153C	ENT5
YNL127W	FAR11	YDR153C	ENT5
YLR234W	TOP3	YGL163C	RAD54
YDL223C	HBT1	YER040W	GLN3
YKL139W	CTK1	YPL022W	RAD1

Predictions at threshold level 0.80 continued...			
Gene1 of predicted pair		Gene2 of predicted pair	
Systematic Name	Standard Name	Systematic Name	Standard Name
YIL076W	SEC28	YOR043W	WHI2
YAL030W	SNC1	YPR032W	SRO7
YNL325C	FIG4	YBL025W	RRN10
YJL176C	SWI3	YGL215W	CLG1
YPL042C	SSN3	YOL051W	GAL11
YPL152W	RRD2	YGL163C	RAD54
YPL105C	YPL105C	YJR060W	CBF1
YKR101W	SIR1	YDL042C	SIR2
YLR398C	SKI2	YMR214W	SCJ1
YHR178W	STB5	YNL107W	YAF9
YMR199W	CLN1	YNL229C	URE2
YOR337W	TEA1	YDR137W	RGP1
YBR264C	YPT10	YNL044W	YIP3
YDR310C	SUM1	YAL002W	VPS8
YPL046C	ELC1	YBR114W	RAD16
YPL129W	TAF14	YBR289W	SNF5
YGR184C	UBR1	YDR363W-A	SEM1
YAL024C	LTE1	YLR234W	TOP3
YNL117W	MLS1	YJL095W	BCK1
YPL161C	BEM4	YLR309C	IMH1
YMR224C	MRE11	YOL111C	MDY2
YMR109W	MYO5	YDR310C	SUM1
YLR441C	RPS1A	YPL161C	BEM4
YJL124C	LSM1	YNL016W	PUB1
YDL223C	HBT1	YDR261C	EXG2
YJR040W	GEF1	YNR032W	PPG1
YLR320W	MMS22	YMR152W	YIM1
YLR337C	VRP1	YDL155W	CLB3
YNL069C	RPL16B	YKR001C	VPS1

Predictions at threshold level 0.80 continued...			
Gene1 of predicted pair		Gene2 of predicted pair	
Systematic Name	Standard Name	Systematic Name	Standard Name
YGL178W	MPT5	YMR263W	SAP30
YPR131C	NAT3	YNL236W	SIN4
YKL139W	CTK1	YMR133W	REC114
YJL124C	LSM1	YCL037C	SRO9
YOR101W	RAS1	YNL079C	TPM1
YGL200C	EMP24	YFL048C	EMP47
YER162C	RAD4	YOL081W	IRA2
YGL094C	PAN2	YKL114C	APN1
YDR293C	SSD1	YJR032W	CPR7
YNL215W	IES2	YGL151W	NUT1
YMR054W	STV1	YEL051W	VMA8
YOR069W	VPS5	YHL020C	OPI1
YOL044W	PEX15	YGL153W	PEX14
YER144C	UBP5	YPL195W	APL5
YOR043W	WHI2	YGL115W	SNF4
YBR274W	CHK1	YGL250W	RMR1
YHR152W	SPO12	YKL032C	IXR1
YBR217W	ATG12	YCR053W	THR4
YDL077C	VAM6	YJR033C	RAV1
YOR124C	UBP2	YLR182W	SWI6
YKR014C	YPT52	YEL036C	ANP1
YBR010W	HHT1	YNL030W	HHF2
YCL001W	RER1	YEL053C	MAK10
YNR052C	POP2	YLR182W	SWI6
YOR132W	VPS17	YJR060W	CBF1
YDR174W	HMO1	YJR135C	MCM22
YMR095C	SNO1	YML115C	VAN1
YOR023C	AHC1	YDR310C	SUM1
YDR108W	GSG1	YKR001C	VPS1

Predictions at threshold level 0.80 continued...			
**Gene1 of predicted pair**		**Gene2 of predicted pair**	
**Systematic Name**	**Standard Name**	**Systematic Name**	**Standard Name**
YLR234W	TOP3	YBR200W	BEM1
YBR274W	CHK1	YNL234W	YNL234W
YEL065W	SIT1	YOR270C	VPH1
YFR040W	SAP155	YLR452C	SST2
YDR484W	VPS52	YDR207C	UME6
YLR093C	NYV1	YOR327C	SNC2
YDR477W	SNF1	YMR016C	SOK2
YOR043W	WHI2	YHR039C-A	VMA10
YBR228W	SLX1	YJL124C	LSM1
YKR035W-A	DID2	YLR452C	SST2
YGL212W	VAM7	YDL226C	GCS1
YDR477W	SNF1	YIL076W	SEC28
YNL307C	MCK1	YOR035C	SHE4
YDR320C	SWA2	YPL234C	TFP3
YLR309C	IMH1	YDR207C	UME6
YKR072C	SIS2	YJR090C	GRR1
YOR132W	VPS17	YMR004W	MVP1
YBL103C	RTG3	YBR171W	SEC66
YFL013C	IES1	YJL124C	LSM1
YPR030W	CSR2	YJL095W	BCK1
YKL126W	YPK1	YMR133W	REC114
YPL022W	RAD1	YJL006C	CTK2
YOR112W	CEX1	YOL108C	INO4
YLR090W	XDJ1	YFR031C-A	RPL2A
YGR054W	YGR054W	YOL067C	RTG1
YML115C	VAN1	YPR046W	MCM16
YKR072C	SIS2	YJR035W	RAD26
YJL154C	VPS35	YHL020C	OPI1
YDL184C	RPL41A	YOR035C	SHE4

Predictions at threshold level 0.80 continued...			
Gene1 of predicted pair		Gene2 of predicted pair	
Systematic Name	Standard Name	Systematic Name	Standard Name
YML115C	VAN1	YCR031C	RPS14A
YBR217W	ATG12	YJR084W	CSN12
YGL033W	HOP2	YER139C	YER139C
YMR216C	SKY1	YNL238W	KEX2
YDL020C	RPN4	YDL059C	RAD59
YJL124C	LSM1	YLR254C	NDL1
YNL325C	FIG4	YPR030W	CSR2
YNL087W	TCB2	YJL183W	MNN11
YDR108W	GSG1	YAR014C	BUD14
YOR132W	VPS17	YEL043W	YEL043W
YCR086W	CSM1	YGL031C	RPL24A
YER107C	GLE2	YMR223W	UBP8
YDR123C	INO2	YHL020C	OPI1
YOR096W	RPS7A	YOR078W	BUD21
YER110C	KAP123	YDR310C	SUM1
YOR127W	RGA1	YEL051W	VMA8
YPL152W	RRD2	YPL018W	CTF19
YLR360W	VPS38	YDR153C	ENT5
YLR447C	VMA6	YJL095W	BCK1
YMR224C	MRE11	YML103C	NUP188
YHR039C-A	VMA10	YPL137C	GIP3
YBL008W	HIR1	YHL025W	SNF6
YHR013C	ARD1	YBR181C	RPS6B
YDR137W	RGP1	YER044C	ERG28
YGR020C	VMA7	YPL195W	APL5
YHR111W	UBA4	YPL161C	BEM4
YLR006C	SSK1	YCR077C	PAT1
YDL020C	RPN4	YLR288C	MEC3
YNL117W	MLS1	YML028W	TSA1

Predictions at threshold level 0.80 continued...			
Gene1 of predicted pair		Gene2 of predicted pair	
Systematic Name	Standard Name	Systematic Name	Standard Name
YMR164C	MSS11	YPL178W	CBC2
YBL103C	RTG3	YNL076W	MKS1
YDR443C	SSN2	YBL025W	RRN10
YDR448W	ADA2	YFR034C	PHO4
YDR254W	CHL4	YOL081W	IRA2
YOR337W	TEA1	YPR036W	VMA13
YOR109W	INP53	YDR310C	SUM1
YGL133W	ITC1	YOR127W	RGA1
YBR200W	BEM1	YBR127C	VMA2
YGL175C	SAE2	YMR231W	PEP5
YGL115W	SNF4	YNL107W	YAF9
YDR245W	MNN10	YHR108W	GGA2
YLR399C	BDF1	YPR046W	MCM16
YNL054W	VAC7	YLR315W	NKP2
YOL086C	ADH1	YPR119W	CLB2
YJL172W	CPS1	YKR035W-A	DID2
YGL078C	DBP3	YPL195W	APL5
YFR036W	CDC26	YGL124C	MON1
YPL024W	RMI1	YCR053W	THR4
YPL253C	VIK1	YML112W	CTK3
YEL027W	CUP5	YNL238W	KEX2
YIL034C	CAP2	YLR429W	CRN1
YJL095W	BCK1	YLR398C	SKI2
YDR004W	RAD57	YKL068W	NUP100
YJR084W	CSN12	YCR053W	THR4
YJL154C	VPS35	YAR014C	BUD14
YOR290C	SNF2	YPL137C	GIP3
YMR026C	PEX12	YMR230W	RPS10B
YHR073W	OSH3	YJR024C	MDE1

Predictions at threshold level 0.80 continued...			
Gene1 of predicted pair		Gene2 of predicted pair	
Systematic Name	Standard Name	Systematic Name	Standard Name
YGL135W	RPL1B	YML071C	COG8
YDR108W	GSG1	YEL051W	VMA8
YDR171W	HSP42	YJR135C	MCM22
YPR072W	NOT5	YGL094C	PAN2
YNL325C	FIG4	YBR019C	GAL10
YGL223C	COG1	YJL029C	VPS53
YJL164C	TPK1	YAL030W	SNC1
YER116C	SLX8	YJL172W	CPS1
YMR016C	SOK2	YPR046W	MCM16
YJL053W	PEP8	YDL226C	GCS1
YJL187C	SWE1	YDL226C	GCS1
YMR095C	SNO1	YBL058W	SHP1
YJR060W	CBF1	YDR183W	PLP1
YLR288C	MEC3	YNL297C	MON2
YDL002C	NHP10	YLR350W	ORM2
YER162C	RAD4	YLR309C	IMH1
YGR249W	MGA1	YML071C	COG8
YPL024W	RMI1	YBR108W	YBR108W
YBL103C	RTG3	YPR046W	MCM16
YDR358W	GGA1	YGL003C	CDH1
YHR134W	WSS1	YDR144C	MKC7
YJL078C	PRY3	YMR319C	FET4
YDR277C	MTH1	YPR119W	CLB2
YGR217W	CCH1	YDL006W	PTC1
YHR111W	UBA4	YJR135C	MCM22
YBR173C	UMP1	YDL047W	SIT4
YHR178W	STB5	YEL056W	HAT2
YBL103C	RTG3	YDL083C	RPS16B
YKR059W	TIF1	YJR140C	HIR3

Predictions at threshold level 0.80 continued...			
**Gene1 of predicted pair**		**Gene2 of predicted pair**	
**Systematic Name**	**Standard Name**	**Systematic Name**	**Standard Name**
YPL161C	BEM4	YDR358W	GGA1
YPL115C	BEM3	YLR386W	VAC14
YML115C	VAN1	YPL195W	APL5
YKR061W	KTR2	YML112W	CTK3
YKL006W	RPL14A	YDR440W	DOT1
YCR024C-A	PMP1	YPL246C	RBD2
YLR234W	TOP3	YBR195C	MSI1
YMR016C	SOK2	YML001W	YPT7
YDR254W	CHL4	YLR254C	NDL1
YBL058W	SHP1	YJL183W	MNN11
YPR036W	VMA13	YGL163C	RAD54
YKL126W	YPK1	YLR180W	SAM1
YEL065W	SIT1	YBR200W	BEM1
YKL139W	CTK1	YPL137C	GIP3
YGL212W	VAM7	YNL238W	KEX2
YDR156W	RPA14	YKR020W	VPS51
YKL010C	UFD4	YMR116C	ASC1
YLR371W	ROM2	YBR019C	GAL10
YLR337C	VRP1	YDR245W	MNN10
YLR090W	XDJ1	YHR060W	VMA22
YPL234C	TFP3	YER122C	GLO3
YDL020C	RPN4	YML115C	VAN1
YHR178W	STB5	YJR140C	HIR3
YNL041C	COG6	YDL077C	VAM6
YMR116C	ASC1	YGL175C	SAE2
YDR448W	ADA2	YOL051W	GAL11
YML016C	PPZ1	YJL124C	LSM1
YLR360W	VPS38	YOR106W	VAM3
YNL058C	YNL058C	YOR035C	SHE4

Predictions at threshold level 0.80 continued...			
Gene1 of predicted pair		Gene2 of predicted pair	
Systematic Name	Standard Name	Systematic Name	Standard Name
YEL013W	VAC8	YER122C	GLO3
YOL030W	GAS5	YJL124C	LSM1
YJR060W	CBF1	YPL234C	TFP3
YMR055C	BUB2	YJR005W	APL1
YJL036W	SNX4	YPL161C	BEM4
YHR012W	VPS29	YEL036C	ANP1
YDR155C	CPR1	YJL095W	BCK1
YOR358W	HAP5	YGR059W	SPR3
YGR249W	MGA1	YEL060C	PRB1
YDR443C	SSN2	YER139C	YER139C
YGL175C	SAE2	YDR207C	UME6
YLR234W	TOP3	YHR111W	UBA4
YNL199C	GCR2	YHR012W	VPS29
YBR215W	HPC2	YNL107W	YAF9
YHR081W	LRP1	YML071C	COG8
YIL076W	SEC28	YPL105C	YPL105C
YLR337C	VRP1	YGR270W	YTA7
YPR189W	SKI3	YLR398C	SKI2
YML038C	YMD8	YNL107W	YAF9
YIL076W	SEC28	YBR217W	ATG12
YGL163C	RAD54	YGL148W	ARO2
YPL105C	YPL105C	YEL053C	MAK10
YNL199C	GCR2	YPL246C	RBD2
YBR089C-A	NHP6B	YPL248C	GAL4
YKR099W	BAS1	YNL058C	YNL058C
YMR313C	TGL3	YMR152W	YIM1
YDR129C	SAC6	YJL029C	VPS53
YKL126W	YPK1	YKR035W-A	DID2
YLR386W	VAC14	YLR254C	NDL1

Predictions at threshold level 0.80 continued...			
Gene1 of predicted pair		Gene2 of predicted pair	
Systematic Name	Standard Name	Systematic Name	Standard Name
YJL006C	CTK2	YML112W	CTK3
YDL083C	RPS16B	YEL060C	PRB1
YDR379W	RGA2	YOR275C	RIM20
YBL051C	PIN4	YOR023C	AHC1
YNR052C	POP2	YDL155W	CLB3
YEL053C	MAK10	YBR280C	SAF1
YIR005W	IST3	YPL018W	CTF19
YNL243W	SLA2	YCR009C	RVS161
YIL076W	SEC28	YOR308C	SNU66
YPL042C	SSN3	YDR363W-A	SEM1
YBR290W	BSD2	YJR032W	CPR7
YOR275C	RIM20	YHR200W	RPN10
YDL020C	RPN4	YLR024C	UBR2
YLR452C	SST2	YGL115W	SNF4
YJR056C	YJR056C	YGL124C	MON1
YML001W	YPT7	YOR066W	MSA1
YHR108W	GGA2	YLR306W	UBC12
YHR060W	VMA22	YOR043W	WHI2
YFR036W	CDC26	YBR019C	GAL10
YPR084W	YPR084W	YMR004W	MVP1
YPL057C	SUR1	YCL037C	SRO9
YMR224C	MRE11	YML112W	CTK3
YIL076W	SEC28	YER040W	GLN3
YML001W	YPT7	YMR054W	STV1
YDL184C	RPL41A	YHR039C-A	VMA10
YGL078C	DBP3	YGL025C	PGD1
YBR173C	UMP1	YBR030W	YBR030W
YBL101C	ECM21	YMR089C	YTA12
YBR019C	GAL10	YMR214W	SCJ1

Predictions at threshold level 0.80 continued...			
Gene1 of predicted pair		Gene2 of predicted pair	
Systematic Name	Standard Name	Systematic Name	Standard Name
YNL201C	PSY2	YBR200W	BEM1
YCL037C	SRO9	YEL013W	VAC8
YOR140W	SFL1	YER049W	TPA1
YMR116C	ASC1	YBR200W	BEM1
YLR386W	VAC14	YJL190C	RPS22A
YGL237C	HAP2	YPL161C	BEM4
YHR156C	LIN1	YER122C	GLO3
YPR120C	CLB5	YGL148W	ARO2
YOR043W	WHI2	YGL163C	RAD54
YOL111C	MDY2	YGL115W	SNF4
YER116C	SLX8	YML071C	COG8
YBR194W	SOY1	YOR038C	HIR2
YIL159W	BNR1	YBL058W	SHP1
YPL001W	HAT1	YGL148W	ARO2
YMR116C	ASC1	YEL044W	IES6
YMR055C	BUB2	YML112W	CTK3
YIL076W	SEC28	YOR080W	DIA2
YFR030W	MET10	YNL225C	CNM67
YGL078C	DBP3	YHR200W	RPN10
YJL210W	PEX2	YLR191W	PEX13
YPR052C	NHP6A	YNL031C	HHT2
YAL024C	LTE1	YHR178W	STB5
YHR013C	ARD1	YDL155W	CLB3
YNL166C	BNI5	YIL008W	URM1
YIL159W	BNR1	YJL183W	MNN11
YKR020W	VPS51	YPL045W	VPS16
YIL076W	SEC28	YMR311C	GLC8
YOR337W	TEA1	YMR153W	NUP53
YBR274W	CHK1	YMR055C	BUB2

Predictions at threshold level 0.80 continued...			
Gene1 of predicted pair		Gene2 of predicted pair	
Systematic Name	Standard Name	Systematic Name	Standard Name
YKL126W	YPK1	YOL111C	MDY2
YFL023W	BUD27	YBR036C	CSG2
YIL159W	BNR1	YGR249W	MGA1
YLR234W	TOP3	YMR055C	BUB2
YNL054W	VAC7	YJL006C	CTK2
YGL033W	HOP2	YFR010W	UBP6
YGL060W	YBP2	YGL003C	CDH1
YGL212W	VAM7	YPL120W	VPS30
YLR399C	BDF1	YDR183W	PLP1
YLR381W	CTF3	YNL116W	DMA2
YDR225W	HTA1	YPL018W	CTF19
YCR020C-A	MAK31	YHR012W	VPS29
YHR013C	ARD1	YML028W	TSA1
YNL288W	CAF40	YOR023C	AHC1
YLL039C	UBI4	YHL002W	HSE1
YMR284W	YKU70	YBL019W	APN2
YPR049C	ATG11	YKL103C	LAP4
YBR108W	YBR108W	YOR066W	MSA1
YMR153W	NUP53	YLR094C	GIS3
YDR378C	LSM6	YOL081W	IRA2
YBL047C	EDE1	YHR158C	KEL1
YKL139W	CTK1	YGL025C	PGD1
YLR386W	VAC14	YHR039C-A	VMA10
YCR020C-A	MAK31	YPL037C	EGD1
YML095C	RAD10	YGR188C	BUB1
YPL253C	VIK1	YJL190C	RPS22A
YKR061W	KTR2	YBR107C	IML3
YGL237C	HAP2	YPL018W	CTF19
YDR103W	STE5	YGR152C	RSR1

Predictions at threshold level 0.80 continued...			
Gene1 of predicted pair		Gene2 of predicted pair	
Systematic Name	Standard Name	Systematic Name	Standard Name
YEL065W	SIT1	YPL161C	BEM4
YNL069C	RPL16B	YGR105W	VMA21
YPL203W	TPK2	YIL033C	BCY1
YMR004W	MVP1	YPL105C	YPL105C
YGL147C	RPL9A	YGL003C	CDH1
YHL031C	GOS1	YDR335W	MSN5
YNL248C	RPA49	YDR335W	MSN5
YLR410W	VIP1	YPL018W	CTF19
YOR076C	SKI7	YGR071C	YGR071C
YBR057C	MUM2	YBL047C	EDE1
YLR386W	VAC14	YJR060W	CBF1
YGL087C	MMS2	YLR360W	VPS38
YJR140C	HIR3	YNL199C	GCR2
YLR398C	SKI2	YBR280C	SAF1
YJL078C	PRY3	YJR106W	ECM27
YJR056C	YJR056C	YHR200W	RPN10
YDR202C	RAV2	YER162C	RAD4
YJR032W	CPR7	YDR358W	GGA1
YNL032W	SIW14	YMR153W	NUP53
YGR152C	RSR1	YDR310C	SUM1
YKR095W	MLP1	YMR273C	ZDS1
YIL076W	SEC28	YJR033C	RAV1
YHR134W	WSS1	YDR310C	SUM1
YFR031C-A	RPL2A	YNL069C	RPL16B
YJR063W	RPA12	YLR254C	NDL1
YJL124C	LSM1	YHR111W	UBA4
YDR484W	VPS52	YDL192W	ARF1
YJL036W	SNX4	YNL107W	YAF9
YNL264C	PDR17	YDR363W-A	SEM1

Predictions at threshold level 0.80 continued...			
**Gene1 of predicted pair**		**Gene2 of predicted pair**	
**Systematic Name**	**Standard Name**	**Systematic Name**	**Standard Name**
YDR261C	EXG2	YBL025W	RRN10
YNL079C	TPM1	YHL002W	HSE1
YBR082C	UBC4	YAL002W	VPS8
YNL215W	IES2	YLR399C	BDF1
YNL201C	PSY2	YLR399C	BDF1
YJL176C	SWI3	YNL106C	INP52
YMR231W	PEP5	YGL095C	VPS45
YGR270W	YTA7	YGL253W	HXK2
YNL218W	MGS1	YOL090W	MSH2
YLR410W	VIP1	YBR082C	UBC4
YNL201C	PSY2	YLR079W	SIC1
YGL147C	RPL9A	YJR032W	CPR7
YDR137W	RGP1	YAL055W	PEX22
YNL094W	APP1	YBL106C	SRO77
YPR046W	MCM16	YOL067C	RTG1
YFR031C-A	RPL2A	YMR133W	REC114
YOR023C	AHC1	YDR318W	MCM21
YHR039C-A	VMA10	YLR309C	IMH1
YNL297C	MON2	YDR440W	DOT1
YPR029C	APL4	YKL135C	APL2
YIR005W	IST3	YEL013W	VAC8
YLR273C	PIG1	YLR258W	GSY2
YNL139C	RLR1	YKL139W	CTK1
YLR410W	VIP1	YPR046W	MCM16
YBL058W	SHP1	YBR019C	GAL10
YBL058W	SHP1	YLR192C	HCR1
YMR153W	NUP53	YMR230W	RPS10B
YPR036W	VMA13	YJL183W	MNN11
YLR410W	VIP1	YHR115C	DMA1

Predictions at threshold level 0.80 continued...			
Gene1 of predicted pair		Gene2 of predicted pair	
Systematic Name	Standard Name	Systematic Name	Standard Name
YMR004W	MVP1	YER122C	GLO3
YDR293C	SSD1	YCR077C	PAT1
YOR043W	WHI2	YGL124C	MON1
YDR202C	RAV2	YCL001W	RER1
YLR386W	VAC14	YOL067C	RTG1
YMR127C	SAS2	YHR012W	VPS29
YOL111C	MDY2	YJR032W	CPR7
YPL137C	GIP3	YOL081W	IRA2
YHR205W	SCH9	YHR030C	SLT2
YML016C	PPZ1	YDR378C	LSM6
YBR057C	MUM2	YPL253C	VIK1
YBR057C	MUM2	YHR013C	ARD1
YJL187C	SWE1	YGL031C	RPL24A
YNL031C	HHT2	YMR273C	ZDS1
YKR057W	RPS21A	YKL032C	IXR1
YHR111W	UBA4	YJL006C	CTK2
YBR065C	ECM2	YGR006W	PRP18
YER049W	TPA1	YDR207C	UME6
YPL256C	CLN2	YOR368W	RAD17
YLR313C	SPH1	YML028W	TSA1
YFR010W	UBP6	YLR370C	ARC18
YPR051W	MAK3	YGR105W	VMA21
YNR072W	HXT17	YLR320W	MMS22
YGL147C	RPL9A	YDR484W	VPS52
YGL135W	RPL1B	YAL055W	PEX22
YNL044W	YIP3	YJR033C	RAV1
YKR072C	SIS2	YBR195C	MSI1
YER139C	YER139C	YLR350W	ORM2
YDL160C	DHH1	YKL114C	APN1

Predictions at threshold level 0.80 continued...			
Gene1 of predicted pair		Gene2 of predicted pair	
Systematic Name	Standard Name	Systematic Name	Standard Name
YBR108W	YBR108W	YDL077C	VAM6
YMR153W	NUP53	YNR072W	HXT17
YOR001W	RRP6	YPL018W	CTF19
YBL102W	SFT2	YHL031C	GOS1
YLR315W	NKP2	YOR124C	UBP2
YLR119W	SRN2	YBR108W	YBR108W
YDR363W-A	SEM1	YFR036W	CDC26
YML097C	VPS9	YBR181C	RPS6B
YDR448W	ADA2	YDR216W	ADR1
YMR026C	PEX12	YGR249W	MGA1
YER096W	SHC1	YMR055C	BUB2
YBL102W	SFT2	YGL212W	VAM7
YBL021C	HAP3	YPL253C	VIK1
YDL184C	RPL41A	YPL195W	APL5
YJL060W	BNA3	YNL236W	SIN4
YDR137W	RGP1	YNL051W	COG5
YDR293C	SSD1	YIL038C	NOT3
YPL022W	RAD1	YPL106C	SSE1
YFR031C-A	RPL2A	YDL006W	PTC1
YCR086W	CSM1	YNL225C	CNM67
YLR090W	XDJ1	YOR035C	SHE4
YOR023C	AHC1	YCR086W	CSM1
YGL066W	SGF73	YLR102C	APC9
YDR129C	SAC6	YER122C	GLO3
YMR142C	RPL13B	YNL238W	KEX2
YJL176C	SWI3	YDR440W	DOT1
YDL013W	SLX5	YOR368W	RAD17
YKR001C	VPS1	YKR030W	GMH1
YJR033C	RAV1	YAL002W	VPS8

Predictions at threshold level 0.80 continued...			
Gene1 of predicted pair		Gene2 of predicted pair	
Systematic Name	Standard Name	Systematic Name	Standard Name
YML095C	RAD10	YJL006C	CTK2
YNL106C	INP52	YHR167W	THP2
YOR001W	RRP6	YDR289C	RTT103
YDR456W	NHX1	YNL079C	TPM1
YLR119W	SRN2	YOL111C	MDY2
YNL087W	TCB2	YOL121C	RPS19A
YJL136C	RPS21B	YGR135W	PRE9
YPL024W	RMI1	YLR386W	VAC14
YBR171W	SEC66	YLR371W	ROM2
YML103C	NUP188	YHR115C	DMA1
YML071C	COG8	YMR152W	YIM1
YBR041W	FAT1	YKL048C	ELM1
YAL024C	LTE1	YNL293W	MSB3
YNL087W	TCB2	YJL172W	CPS1
YNL229C	URE2	YNL116W	DMA2
YKR035W-A	DID2	YMR152W	YIM1
YDR318W	MCM21	YGL252C	RTG2
YJL172W	CPS1	YPL195W	APL5
YNR052C	POP2	YNL307C	MCK1
YDR080W	VPS41	YML071C	COG8
YDR363W-A	SEM1	YGL003C	CDH1
YLR441C	RPS1A	YER044C	ERG28
YLR381W	CTF3	YDR260C	SWM1
YML103C	NUP188	YIL103W	DPH1
YLR119W	SRN2	YNL307C	MCK1
YNL058C	YNL058C	YDR261C	EXG2
YMR004W	MVP1	YDR080W	VPS41
YDR171W	HSP42	YPL018W	CTF19
YHR178W	STB5	YER110C	KAP123

Predictions at threshold level 0.80 continued...			
Gene1 of predicted pair		Gene2 of predicted pair	
Systematic Name	Standard Name	Systematic Name	Standard Name
YLR452C	SST2	YLR398C	SKI2
YBR270C	BIT2	YIL105C	SLM1
YHR200W	RPN10	YPR046W	MCM16
YDR004W	RAD57	YER161C	SPT2
YNL069C	RPL16B	YPR051W	MAK3
YGR241C	YAP1802	YKR020W	VPS51
YKL101W	HSL1	YGL003C	CDH1
YBL058W	SHP1	YBL103C	RTG3
YJL006C	CTK2	YGL003C	CDH1
YEL065W	SIT1	YNL325C	FIG4
YKR059W	TIF1	YML016C	PPZ1
YDL116W	NUP84	YMR125W	STO1
YGL174W	BUD13	YNL107W	YAF9
YDR293C	SSD1	YHR086W	NAM8
YPL024W	RMI1	YOR035C	SHE4
YOR327C	SNC2	YAR014C	BUD14
YBR171W	SEC66	YPL050C	MNN9
YPR049C	ATG11	YPR119W	CLB2
YJL006C	CTK2	YJL183W	MNN11
YLR320W	MMS22	YML071C	COG8
YJL060W	BNA3	YLR079W	SIC1
YMR039C	SUB1	YBR107C	IML3
YBR020W	GAL1	YHR200W	RPN10
YBL103C	RTG3	YPR036W	VMA13
YPL161C	BEM4	YLR371W	ROM2
YGR249W	MGA1	YKR099W	BAS1
YDR293C	SSD1	YOR109W	INP53
YJR024C	MDE1	YIL148W	RPL40A
YPL057C	SUR1	YDR137W	RGP1

Predictions at threshold level 0.80 continued...			
Gene1 of predicted pair		Gene2 of predicted pair	
Systematic Name	Standard Name	Systematic Name	Standard Name
YDR192C	NUP42	YER110C	KAP123
YNL264C	PDR17	YNL236W	SIN4
YLR079W	SIC1	YLR381W	CTF3
YPL158C	YPL158C	YHR158C	KEL1
YJL124C	LSM1	YGL151W	NUT1
YGL025C	PGD1	YJL095W	BCK1
YNL307C	MCK1	YPL161C	BEM4
YGR019W	UGA1	YHR114W	BZZ1
YOR080W	DIA2	YCR086W	CSM1
YML115C	VAN1	YML097C	VPS9
YLR452C	SST2	YML001W	YPT7
YPR051W	MAK3	YDR289C	RTT103
YMR142C	RPL13B	YML001W	YPT7
YMR016C	SOK2	YGL124C	MON1
YER044C	ERG28	YBL025W	RRN10
YKL126W	YPK1	YDL083C	RPS16B
YNL068C	FKH2	YIL008W	URM1
YMR133W	REC114	YHL022C	SPO11
YBR082C	UBC4	YBR181C	RPS6B
YPR018W	RLF2	YLR381W	CTF3
YML007W	YAP1	YER110C	KAP123
YGL212W	VAM7	YNL051W	COG5
YMR224C	MRE11	YJR035W	RAD26
YDL059C	RAD59	YML028W	TSA1
YNR072W	HXT17	YGL124C	MON1
YPR084W	YPR084W	YKR001C	VPS1
YLR381W	CTF3	YGL115W	SNF4
YLR452C	SST2	YFR036W	CDC26
YJL154C	VPS35	YNL127W	FAR11

Predictions at threshold level 0.80 continued...			
**Gene1 of predicted pair**		**Gene2 of predicted pair**	
**Systematic Name**	**Standard Name**	**Systematic Name**	**Standard Name**
YJL210W	PEX2	YDR142C	PEX7
YHR013C	ARD1	YOR014W	RTS1
YER162C	RAD4	YPR032W	SRO7
YOR080W	DIA2	YNL236W	SIN4
YJL006C	CTK2	YHR115C	DMA1
YJL127C	SPT10	YJR060W	CBF1
YPL057C	SUR1	YNL297C	MON2
YJL172W	CPS1	YJR056C	YJR056C
YOR279C	RFM1	YDR310C	SUM1
YPL253C	VIK1	YHR013C	ARD1
YPR018W	RLF2	YDR260C	SWM1
YGL025C	PGD1	YDL077C	VAM6
YDL077C	VAM6	YBR036C	CSG2
YLR093C	NYV1	YPL195W	APL5
YJR056C	YJR056C	YJR032W	CPR7
YDR507C	GIN4	YDR335W	MSN5
YGL009C	LEU1	YPR119W	CLB2
YLR234W	TOP3	YOL090W	MSH2
YDR174W	HMO1	YKL110C	KTI12
YPL253C	VIK1	YMR038C	CCS1
YOL147C	PEX11	YDL078C	MDH3
YNL201C	PSY2	YKR061W	KTR2
YNR072W	HXT17	YMR133W	REC114
YKL006W	RPL14A	YBL101C	ECM21
YMR039C	SUB1	YPL024W	RMI1
YGR072W	UPF3	YPL195W	APL5
YGR081C	SLX9	YPL022W	RAD1
YHR039C-A	VMA10	YLR093C	NYV1
YHR171W	ATG7	YNL223W	ATG4

Predictions at threshold level 0.80 continued...			
Gene1 of predicted pair		Gene2 of predicted pair	
Systematic Name	Standard Name	Systematic Name	Standard Name
YKR039W	GAP1	YCL018W	LEU2
YBR195C	MSI1	YGL148W	ARO2
YLR234W	TOP3	YOR023C	AHC1
YBR173C	UMP1	YNL238W	KEX2
YIL034C	CAP2	YCR077C	PAT1
YDR174W	HMO1	YNL054W	VAC7
YMR224C	MRE11	YDL223C	HBT1
YHR012W	VPS29	YOR208W	PTP2
YKR099W	BAS1	YPL195W	APL5
YKR020W	VPS51	YBR082C	UBC4
YER002W	NOP16	YKL009W	MRT4
YHR178W	STB5	YDR155C	CPR1
YBL008W	HIR1	YBR274W	CHK1
YOR195W	SLK19	YNL116W	DMA2
YER027C	GAL83	YGL179C	TOS3
YMR004W	MVP1	YHL020C	OPI1
YNL297C	MON2	YDR207C	UME6
YHR156C	LIN1	YEL060C	PRB1
YDR443C	SSN2	YMR199W	CLN1
YGL115W	SNF4	YDR261C	EXG2
YKR072C	SIS2	YFL023W	BUD27
YGL178W	MPT5	YLR452C	SST2
YKL048C	ELM1	YGR188C	BUB1
YBR200W	BEM1	YHR152W	SPO12
YDR176W	NGG1	YGL252C	RTG2
YDR335W	MSN5	YLR410W	VIP1
YPR051W	MAK3	YHR114W	BZZ1
YPR036W	VMA13	YGL124C	MON1
YML071C	COG8	YNL051W	COG5

Predictions at threshold level 0.80 continued...			
Gene1 of predicted pair		Gene2 of predicted pair	
Systematic Name	Standard Name	Systematic Name	Standard Name
YML001W	YPT7	YPR046W	MCM16
YLR095C	IOC2	YEL053C	MAK10
YFR010W	UBP6	YNL199C	GCR2
YIL159W	BNR1	YLR441C	RPS1A
YGL078C	DBP3	YOR035C	SHE4
YLR386W	VAC14	YDL083C	RPS16B
YDR202C	RAV2	YJR040W	GEF1
YFR031C-A	RPL2A	YDR202C	RAV2
YDR439W	LRS4	YJR060W	CBF1
YGL215W	CLG1	YLR131C	ACE2
YER116C	SLX8	YNL238W	KEX2
YER001W	MNN1	YOR035C	SHE4
YGL124C	MON1	YDL192W	ARF1
YLR135W	SLX4	YLR313C	SPH1
YNL238W	KEX2	YER040W	GLN3
YEL053C	MAK10	YMR133W	REC114
YCR066W	RAD18	YML071C	COG8
YGL163C	RAD54	YLR182W	SWI6
YOR080W	DIA2	YER139C	YER139C
YBR057C	MUM2	YNL291C	MID1
YCR077C	PAT1	YHR167W	THP2
YDR383C	NKP1	YLR386W	VAC14
YLR192C	HCR1	YJR032W	CPR7
YAL024C	LTE1	YKR061W	KTR2
YEL065W	SIT1	YBR030W	YBR030W
YER116C	SLX8	YBL103C	RTG3
YEL050C	RML2	YMR202W	ERG2
YLL039C	UBI4	YBR083W	TEC1
YGL212W	VAM7	YER139C	YER139C

Predictions at threshold level 0.80 continued...			
Gene1 of predicted pair		Gene2 of predicted pair	
Systematic Name	Standard Name	Systematic Name	Standard Name
YOR106W	VAM3	YJL095W	BCK1
YLR094C	GIS3	YOR327C	SNC2
YJL151C	SNA3	YPL246C	RBD2
YML012W	ERV25	YFL048C	EMP47
YDR477W	SNF1	YNL238W	KEX2
YMR016C	SOK2	YBR019C	GAL10
YLR292C	SEC72	YJR135C	MCM22
YDR293C	SSD1	YGL212W	VAM7
YFR010W	UBP6	YMR142C	RPL13B
YCL001W	RER1	YOL108C	INO4
YGR275W	RTT102	YDR073W	SNF11
YLR095C	IOC2	YDL083C	RPS16B
YJL083W	TAX4	YIL002C	INP51
YIL053W	RHR2	YLR119W	SRN2
YKL139W	CTK1	YDR378C	LSM6
YLR191W	PEX13	YDL065C	PEX19
YDR378C	LSM6	YLR425W	TUS1
YKL114C	APN1	YLR032W	RAD5
YKL117W	SBA1	YBL079W	NUP170
YNL288W	CAF40	YHL002W	HSE1
YMR192W	GYL1	YNL094W	APP1
YBR173C	UMP1	YBR171W	SEC66
YOR195W	SLK19	YNL248C	RPA49
YLL007C	YLL007C	YHR012W	VPS29
YKL048C	ELM1	YNL229C	URE2
YBL047C	EDE1	YKR048C	NAP1
YMR224C	MRE11	YMR311C	GLC8
YBR195C	MSI1	YER161C	SPT2
YJL095W	BCK1	YOR014W	RTS1

Predictions at threshold level 0.80 continued...			
Gene1 of predicted pair		Gene2 of predicted pair	
Systematic Name	Standard Name	Systematic Name	Standard Name
YOR080W	DIA2	YKL114C	APN1
YPL042C	SSN3	YJR060W	CBF1
YLR396C	VPS33	YPL195W	APL5
YML001W	YPT7	YGL003C	CDH1
YJL036W	SNX4	YGR020C	VMA7
YDL020C	RPN4	YKL048C	ELM1
YDR363W-A	SEM1	YDR264C	AKR1
YBR030W	YBR030W	YLR370C	ARC18
YFR031C-A	RPL2A	YPL120W	VPS30
YGL174W	BUD13	YPR101W	SNT309
YNL025C	SSN8	YOR001W	RRP6
YMR153W	NUP53	YDR137W	RGP1
YGR040W	KSS1	YGL178W	MPT5
YLR119W	SRN2	YBR173C	UMP1
YPL057C	SUR1	YMR311C	GLC8
YJL060W	BNA3	YJL006C	CTK2
YMR153W	NUP53	YLR093C	NYV1
YDR192C	NUP42	YBL047C	EDE1
YDR123C	INO2	YOL108C	INO4
YBR030W	YBR030W	YDR207C	UME6
YKL006W	RPL14A	YBL047C	EDE1
YJL154C	VPS35	YER096W	SHC1
YLR090W	XDJ1	YFR036W	CDC26
YCR066W	RAD18	YOR368W	RAD17
YPL057C	SUR1	YDL077C	VAM6
YLR119W	SRN2	YKL139W	CTK1
YLR360W	VPS38	YPL256C	CLN2
YOR008C	SLG1	YNL079C	TPM1
YPL024W	RMI1	YHR081W	LRP1

Predictions at threshold level 0.80 continued...			
**Gene1 of predicted pair**		**Gene2 of predicted pair**	
**Systematic Name**	**Standard Name**	**Systematic Name**	**Standard Name**
YLR288C	MEC3	YBR195C	MSI1
YPL042C	SSN3	YLR350W	ORM2
YMR026C	PEX12	YOR043W	WHI2
YKR072C	SIS2	YNL215W	IES2
YMR004W	MVP1	YOR109W	INP53
YIL140W	AXL2	YCR038C	BUD5
YFR032C-A	RPL29	YLR371W	ROM2
YFL013C	IES1	YBR194W	SOY1
YHR156C	LIN1	YKR035W-A	DID2
YOR112W	CEX1	YJL004C	SYS1
YGR159C	NSR1	YOR375C	GDH1
YBL047C	EDE1	YHL011C	PRS3
YHR039C-A	VMA10	YNL051W	COG5
YLR381W	CTF3	YDR310C	SUM1
YML016C	PPZ1	YGR252W	GCN5
YGR019W	UGA1	YLR381W	CTF3
YCR081W	SRB8	YPL253C	VIK1
YHL031C	GOS1	YNL127W	FAR11
YML102W	CAC2	YLR352W	YLR352W
YLR386W	VAC14	YKR099W	BAS1
YLR315W	NKP2	YDR289C	RTT103
YGL212W	VAM7	YNL079C	TPM1
YKL139W	CTK1	YBR036C	CSG2
YDR477W	SNF1	YER122C	GLO3
YFR036W	CDC26	YOL121C	RPS19A
YJL060W	BNA3	YPL022W	RAD1
YMR224C	MRE11	YDR192C	NUP42
YIL036W	CST6	YDR378C	LSM6
YDR225W	HTA1	YOR141C	ARP8

Predictions at threshold level 0.80 continued...			
Gene1 of predicted pair		Gene2 of predicted pair	
Systematic Name	Standard Name	Systematic Name	Standard Name
YHR171W	ATG7	YNR049C	MSO1
YDR176W	NGG1	YOL145C	CTR9
YJL053W	PEP8	YJR040W	GEF1
YOR127W	RGA1	YNL297C	MON2
YMR139W	RIM11	YJL106W	IME2
YDR477W	SNF1	YHR205W	SCH9
YMR054W	STV1	YDR310C	SUM1
YML071C	COG8	YNR032W	PPG1
YGL151W	NUT1	YDR310C	SUM1
YLR119W	SRN2	YMR152W	YIM1
YLR410W	VIP1	YIL103W	DPH1
YOR127W	RGA1	YKR001C	VPS1
YDR254W	CHL4	YHR114W	BZZ1
YNL288W	CAF40	YOR127W	RGA1
YCL001W	RER1	YPL120W	VPS30
YGL071W	AFT1	YPL240C	HSP82
YGL115W	SNF4	YLR410W	VIP1
YDL160C	DHH1	YGL190C	CDC55
YOR066W	MSA1	YDL077C	VAM6
YOR080W	DIA2	YDR440W	DOT1
YFR010W	UBP6	YOR195W	SLK19
YJL095W	BCK1	YLR370C	ARC18
YOR043W	WHI2	YBL025W	RRN10
YNL166C	BNI5	YOR358W	HAP5
YOR035C	SHE4	YCR053W	THR4
YDR261C	EXG2	YDR358W	GGA1
YNL307C	MCK1	YDL013W	SLX5
YDL077C	VAM6	YMR311C	GLC8
YJL053W	PEP8	YJL204C	RCY1

Predictions at threshold level 0.80 continued...			
Gene1 of predicted pair		Gene2 of predicted pair	
Systematic Name	Standard Name	Systematic Name	Standard Name
YDR171W	HSP42	YDL226C	GCS1
YNL087W	TCB2	YDL192W	ARF1
YGL147C	RPL9A	YNL238W	KEX2
YPL050C	MNN9	YOR312C	RPL20B
YLR337C	VRP1	YNL107W	YAF9
YOR001W	RRP6	YOR308C	SNU66
YDR484W	VPS52	YDL226C	GCS1
YBR215W	HPC2	YGL163C	RAD54
YML028W	TSA1	YGR188C	BUB1
YKL048C	ELM1	YGL179C	TOS3
YJL151C	SNA3	YBR239C	YBR239C
YJR024C	MDE1	YLR079W	SIC1
YGR270W	YTA7	YLR410W	VIP1
YHL020C	OPI1	YOL018C	TLG2
YER115C	SPR6	YMR164C	MSS11
YPL161C	BEM4	YLR425W	TUS1
YGL163C	RAD54	YJL006C	CTK2
YIL076W	SEC28	YDR289C	RTT103
YKR035W-A	DID2	YEL053C	MAK10
YHR108W	GGA2	YDL137W	ARF2
YJR032W	CPR7	YDL077C	VAM6
YDL116W	NUP84	YGR270W	YTA7
YJL183W	MNN11	YHL002W	HSE1
YDR004W	RAD57	YHR039C-A	VMA10
YJL004C	SYS1	YDL077C	VAM6
YBR240C	THI2	YKL139W	CTK1
YOR270C	VPH1	YJR033C	RAV1
YDR477W	SNF1	YNL325C	FIG4
YLR441C	RPS1A	YPL195W	APL5

Predictions at threshold level 0.80 continued...			
Gene1 of predicted pair		Gene2 of predicted pair	
Systematic Name	Standard Name	Systematic Name	Standard Name
YHR156C	LIN1	YDR484W	VPS52
YLR410W	VIP1	YCR086W	CSM1
YDR484W	VPS52	YLR094C	GIS3
YDR004W	RAD57	YLR018C	POM34
YGR133W	PEX4	YHR013C	ARD1
YOR140W	SFL1	YNL199C	GCR2
YMR224C	MRE11	YMR231W	PEP5
YGL163C	RAD54	YGL031C	RPL24A
YOR270C	VPH1	YDR137W	RGP1
YMR026C	PEX12	YLR370C	ARC18
YBR127C	VMA2	YLR396C	VPS33
YGR261C	APL6	YAL002W	VPS8
YMR022W	UBC7	YNL051W	COG5
YJR024C	MDE1	YMR273C	ZDS1
YPL161C	BEM4	YOL067C	RTG1
YMR026C	PEX12	YDR477W	SNF1
YPL057C	SUR1	YIR005W	IST3
YJR140C	HIR3	YDR440W	DOT1
YOL121C	RPS19A	YPR046W	MCM16
YCR020C-A	MAK31	YGR261C	APL6
YNL288W	CAF40	YBR108W	YBR108W
YNL215W	IES2	YNL264C	PDR17
YDR225W	HTA1	YPR119W	CLB2
YHR200W	RPN10	YLR421C	RPN13
YDL020C	RPN4	YGR054W	YGR054W
YGR072W	UPF3	YOR127W	RGA1
YBR200W	BEM1	YDR155C	CPR1
YPL140C	MKK2	YOR208W	PTP2
YNL291C	MID1	YHR200W	RPN10

Predictions at threshold level 0.80 continued...			
Gene1 of predicted pair		Gene2 of predicted pair	
Systematic Name	Standard Name	Systematic Name	Standard Name
YGR249W	MGA1	YOR035C	SHE4
YDR004W	RAD57	YDL020C	RPN4
YGL133W	ITC1	YNL206C	RTT106
YMR311C	GLC8	YDR310C	SUM1
YKL126W	YPK1	YCR053W	THR4
YIL076W	SEC28	YDR261C	EXG2
YOR312C	RPL20B	YNL079C	TPM1
YBL058W	SHP1	YBR108W	YBR108W
YLR227C	ADY4	YNL225C	CNM67
YJR132W	NMD5	YPR119W	CLB2
YIL034C	CAP2	YGL252C	RTG2
YNL069C	RPL16B	YOR132W	VPS17
YKR072C	SIS2	YGL148W	ARO2
YMR004W	MVP1	YAL002W	VPS8
YOR327C	SNC2	YER122C	GLO3
YBR019C	GAL10	YJL095W	BCK1
YPL001W	HAT1	YKR001C	VPS1
YFR021W	ATG18	YJR025C	BNA1
YEL051W	VMA8	YAL002W	VPS8
YML102W	CAC2	YMR038C	CCS1
YOR112W	CEX1	YOR109W	INP53
YFR031C-A	RPL2A	YJR040W	GEF1
YDR439W	LRS4	YDR318W	MCM21
YPL161C	BEM4	YER122C	GLO3
YIL076W	SEC28	YEL013W	VAC8
YML016C	PPZ1	YHR111W	UBA4
YNL087W	TCB2	YER044C	ERG28
YLR064W	YLR064W	YMR253C	YMR253C
YIL053W	RHR2	YAR014C	BUD14

Predictions at threshold level 0.80 continued...			
Gene1 of predicted pair		Gene2 of predicted pair	
Systematic Name	Standard Name	Systematic Name	Standard Name
YNL297C	MON2	YDL192W	ARF1
YNL106C	INP52	YNL225C	CNM67
YBL102W	SFT2	YLR309C	IMH1
YBR108W	YBR108W	YGL124C	MON1
YNL291C	MID1	YNL107W	YAF9
YMR109W	MYO5	YPL161C	BEM4
YLR079W	SIC1	YFR036W	CDC26
YBR108W	YBR108W	YGR054W	YGR054W
YJR140C	HIR3	YGR270W	YTA7
YDL080C	THI3	YER139C	YER139C
YIL076W	SEC28	YCL001W	RER1
YDR477W	SNF1	YPR119W	CLB2
YIR025W	MND2	YDR260C	SWM1
YER161C	SPT2	YDR289C	RTT103
YHR108W	GGA2	YOL081W	IRA2
YIL156W	UBP7	YCR024C-A	PMP1
YLR119W	SRN2	YOR043W	WHI2
YGL161C	YIP5	YEL056W	HAT2
YBR041W	FAT1	YER044C	ERG28
YOR275C	RIM20	YPL089C	RLM1
YCR020C-A	MAK31	YKR001C	VPS1
YPL057C	SUR1	YER040W	GLN3
YDL184C	RPL41A	YGL212W	VAM7
YMR199W	CLN1	YMR052W	FAR3
YFR031C-A	RPL2A	YPR051W	MAK3
YGL078C	DBP3	YOR043W	WHI2
YKL101W	HSL1	YMR038C	CCS1
YDL020C	RPN4	YPR051W	MAK3
YDL184C	RPL41A	YDR484W	VPS52

Predictions at threshold level 0.80 continued...			
Gene1 of predicted pair		Gene2 of predicted pair	
Systematic Name	Standard Name	Systematic Name	Standard Name
YBL103C	RTG3	YOR066W	MSA1
YPR089W	YPR089W	YBR283C	SSH1
YJL127C	SPT10	YLR381W	CTF3
YMR055C	BUB2	YNL225C	CNM67
YKL025C	PAN3	YLR234W	TOP3
YDR245W	MNN10	YMR089C	YTA12
YER144C	UBP5	YBL103C	RTG3
YDR443C	SSN2	YMR224C	MRE11
YFR010W	UBP6	YKL116C	PRR1
YDR358W	GGA1	YDL192W	ARF1
YOR018W	ROD1	YMR243C	ZRC1
YMR202W	ERG2	YDR200C	VPS64
YDL013W	SLX5	YHR013C	ARD1
YDR484W	VPS52	YPR036W	VMA13
YLL007C	YLL007C	YDL155W	CLB3
YOR337W	TEA1	YPR046W	MCM16
YMR202W	ERG2	YDR254W	CHL4
YBR264C	YPT10	YER074W-A	YOS1
YMR224C	MRE11	YCR053W	THR4
YBR217W	ATG12	YER044C	ERG28
YNL166C	BNI5	YGL124C	MON1
YPR030W	CSR2	YMR311C	GLC8
YNL307C	MCK1	YNL116W	DMA2
YKL010C	UFD4	YGR135W	PRE9
YNL127W	FAR11	YKR030W	GMH1
YGR270W	YTA7	YKL110C	KTI12
YDL013W	SLX5	YOL090W	MSH2
YOR023C	AHC1	YGR252W	GCN5
YBL047C	EDE1	YNL229C	URE2

Predictions at threshold level 0.80 continued...			
Gene1 of predicted pair		Gene2 of predicted pair	
Systematic Name	Standard Name	Systematic Name	Standard Name
YER129W	SAK1	YPL256C	CLN2
YPR131C	NAT3	YPL018W	CTF19
YFR010W	UBP6	YFR036W	CDC26
YBR194W	SOY1	YKL213C	DOA1
YJL058C	BIT61	YDR318W	MCM21
YKR101W	SIR1	YBR009C	HHF1
YBL021C	HAP3	YEL053C	MAK10
YLR094C	GIS3	YOR035C	SHE4
YLR095C	IOC2	YBR173C	UMP1
YER107C	GLE2	YML103C	NUP188
YGL025C	PGD1	YDR207C	UME6
YHR039C-A	VMA10	YMR230W	RPS10B
YLR018C	POM34	YDR137W	RGP1
YLR090W	XDJ1	YDL223C	HBT1
YBL103C	RTG3	YMR152W	YIM1
YNL215W	IES2	YEL056W	HAT2
YBR126C	TPS1	YDL192W	ARF1
YER120W	SCS2	YMR054W	STV1
YDR448W	ADA2	YPL248C	GAL4
YPR120C	CLB5	YDR318W	MCM21
YHR205W	SCH9	YOR008C	SLG1
YLR410W	VIP1	YOL081W	IRA2
YDR439W	LRS4	YJL124C	LSM1
YJL004C	SYS1	YHR016C	YSC84
YBL008W	HIR1	YGR252W	GCN5
YBR131W	CCZ1	YMR231W	PEP5
YLR315W	NKP2	YCR077C	PAT1
YPL057C	SUR1	YMR055C	BUB2
YLR320W	MMS22	YFR036W	CDC26

Predictions at threshold level 0.80 continued...			
Gene1 of predicted pair		Gene2 of predicted pair	
Systematic Name	Standard Name	Systematic Name	Standard Name
YML103C	NUP188	YOL126C	MDH2
YPR051W	MAK3	YAR014C	BUD14
YLR292C	SEC72	YLR386W	VAC14
YLR094C	GIS3	YGL115W	SNF4
YGL178W	MPT5	YBR200W	BEM1
YDL065C	PEX19	YGR077C	PEX8
YJL095W	BCK1	YNL090W	RHO2
YMR016C	SOK2	YMR230W	RPS10B
YDL161W	ENT1	YDL226C	GCS1
YPR029C	APL4	YDL006W	PTC1
YLR170C	APS1	YOR069W	VPS5
YJR024C	MDE1	YNL199C	GCR2
YPR189W	SKI3	YPR119W	CLB2
YML071C	COG8	YEL042W	GDA1
YMR055C	BUB2	YJR135C	MCM22
YIL105C	SLM1	YJL058C	BIT61
YDR293C	SSD1	YNL288W	CAF40
YPL105C	YPL105C	YEL051W	VMA8
YLR018C	POM34	YBR042C	CST26
YCR086W	CSM1	YLR233C	EST1
YFR010W	UBP6	YGL025C	PGD1
YMR224C	MRE11	YAR014C	BUD14
YDR293C	SSD1	YJL190C	RPS22A
YDR254W	CHL4	YGL124C	MON1
YNL238W	KEX2	YJR033C	RAV1
YBR200W	BEM1	YPL234C	TFP3
YOR337W	TEA1	YPL137C	GIP3
YLR093C	NYV1	YFL048C	EMP47
YMR026C	PEX12	YER122C	GLO3

Predictions at threshold level 0.80 continued...			
**Gene1 of predicted pair**		**Gene2 of predicted pair**	
**Systematic Name**	**Standard Name**	**Systematic Name**	**Standard Name**
YBR200W	BEM1	YDR310C	SUM1
YHR134W	WSS1	YGL175C	SAE2
YMR054W	STV1	YDR153C	ENT5
YPL234C	TFP3	YJL183W	MNN11
YNL215W	IES2	YCL037C	SRO9
YDR174W	HMO1	YNL116W	DMA2
YFL023W	BUD27	YKL048C	ELM1
YIL159W	BNR1	YBL051C	PIN4
YHL020C	OPI1	YDL077C	VAM6
YLR288C	MEC3	YJL138C	TIF2
YLR254C	NDL1	YGL252C	RTG2
YLR360W	VPS38	YOR109W	INP53
YOL145C	CTR9	YGL025C	PGD1
YJR063W	RPA12	YER068W	MOT2
YDR156W	RPA14	YOR032C	HMS1
YIL034C	CAP2	YGL212W	VAM7
YGL135W	RPL1B	YBR042C	CST26
YJL210W	PEX2	YAL055W	PEX22
YJL136C	RPS21B	YDL047W	SIT4
YNL248C	RPA49	YKL048C	ELM1
YBR260C	RGD1	YLR425W	TUS1
YLR192C	HCR1	YDL184C	RPL41A
YDR477W	SNF1	YBR260C	RGD1
YGL147C	RPL9A	YIL159W	BNR1
YDR484W	VPS52	YKR035W-A	DID2
YKR072C	SIS2	YOR368W	RAD17
YDR443C	SSN2	YGL124C	MON1
YFR010W	UBP6	YBR194W	SOY1
YCL037C	SRO9	YOR308C	SNU66

Predictions at threshold level 0.80 continued...			
Gene1 of predicted pair		Gene2 of predicted pair	
Systematic Name	Standard Name	Systematic Name	Standard Name
YDR293C	SSD1	YJL004C	SYS1
YPL246C	RBD2	YKR030W	GMH1
YPL049C	DIG1	YBR083W	TEC1
YCR024C-A	PMP1	YMR125W	STO1
YMR095C	SNO1	YDR358W	GGA1
YBR107C	IML3	YIR005W	IST3
YJR056C	YJR056C	YNL041C	COG6
YOR270C	VPH1	YOR035C	SHE4
YBR173C	UMP1	YOL111C	MDY2
YIL002C	INP51	YDL161W	ENT1
YPL246C	RBD2	YOR284W	HUA2
YPR119W	CLB2	YLR032W	RAD5
YGL200C	EMP24	YLR131C	ACE2
YGR188C	BUB1	YNL225C	CNM67
YGL016W	KAP122	YKL068W	NUP100
YER162C	RAD4	YDR358W	GGA1
YDL223C	HBT1	YJL095W	BCK1
YDR137W	RGP1	YDL077C	VAM6
YML001W	YPT7	YBR280C	SAF1
YDL002C	NHP10	YHR167W	THP2
YBR194W	SOY1	YBR200W	BEM1
YBR173C	UMP1	YOR035C	SHE4
YDR477W	SNF1	YPR036W	VMA13
YLR360W	VPS38	YDR207C	UME6
YOR332W	VMA4	YKL119C	VPH2
YMR224C	MRE11	YDL155W	CLB3
YNL288W	CAF40	YEL053C	MAK10
YCR020C-A	MAK31	YOR132W	VPS17
YPL022W	RAD1	YGL094C	PAN2

Predictions at threshold level 0.80 continued...			
Gene1 of predicted pair		Gene2 of predicted pair	
Systematic Name	Standard Name	Systematic Name	Standard Name
YLR292C	SEC72	YAL002W	VPS8
YPL152W	RRD2	YPR119W	CLB2
YER139C	YER139C	YLR180W	SAM1
YLR292C	SEC72	YJL095W	BCK1
YBL102W	SFT2	YDR108W	GSG1
YBR171W	SEC66	YPL037C	EGD1
YJR056C	YJR056C	YHR039C-A	VMA10
YGR054W	YGR054W	YJR135C	MCM22
YGL237C	HAP2	YMR055C	BUB2
YMR311C	GLC8	YLR370C	ARC18
YHR111W	UBA4	YJR032W	CPR7
YPR018W	RLF2	YOL110W	SHR5
YGL250W	RMR1	YPR046W	MCM16
YJL176C	SWI3	YCR077C	PAT1
YGL115W	SNF4	YDL047W	SIT4
YNL215W	IES2	YHL031C	GOS1
YER139C	YER139C	YDL192W	ARF1
YOR033C	EXO1	YJL124C	LSM1
YML001W	YPT7	YMR230W	RPS10B
YDR477W	SNF1	YDL077C	VAM6
YDR383C	NKP1	YJR063W	RPA12
YKL006W	RPL14A	YBL072C	RPS8A
YFR031C-A	RPL2A	YER120W	SCS2
YLR410W	VIP1	YNL107W	YAF9
YER044C	ERG28	YEL053C	MAK10
YCR088W	ABP1	YOR284W	HUA2
YNL135C	FPR1	YDR155C	CPR1
YPL001W	HAT1	YDL002C	NHP10
YGL019W	CKB1	YPR119W	CLB2

Predictions at threshold level 0.80 continued...			
Gene1 of predicted pair		Gene2 of predicted pair	
Systematic Name	Standard Name	Systematic Name	Standard Name
YLL039C	UBI4	YDR440W	DOT1
YHL031C	GOS1	YBR215W	HPC2
YFR031C-A	RPL2A	YPR032W	SRO7
YFR010W	UBP6	YER161C	SPT2
YGR135W	PRE9	YOR035C	SHE4
YOR080W	DIA2	YGR105W	VMA21
YHR039C-A	VMA10	YKL080W	VMA5
YOR038C	HIR2	YLR350W	ORM2
YML103C	NUP188	YDR378C	LSM6
YGL212W	VAM7	YBL047C	EDE1
YIL033C	BCY1	YLR206W	ENT2
YOR001W	RRP6	YDR440W	DOT1
YPR084W	YPR084W	YOR080W	DIA2
YPL161C	BEM4	YJR032W	CPR7
YMR179W	SPT21	YGL003C	CDH1
YOR076C	SKI7	YPL024W	RMI1
YCR020C-A	MAK31	YOR109W	INP53
YDR156W	RPA14	YML117W	NAB6
YPL253C	VIK1	YOR014W	RTS1
YDL077C	VAM6	YKL119C	VPH2
YIR005W	IST3	YJR135C	MCM22
YNL041C	COG6	YLR309C	IMH1
YOR270C	VPH1	YER139C	YER139C
YLL039C	UBI4	YDR477W	SNF1
YMR172W	HOT1	YLR234W	TOP3
YHR134W	WSS1	YER110C	KAP123
YGL060W	YBP2	YJL058C	BIT61
YGL066W	SGF73	YBR107C	IML3
YEL065W	SIT1	YBR171W	SEC66

Predictions at threshold level 0.80 continued...			
Gene1 of predicted pair		Gene2 of predicted pair	
Systematic Name	Standard Name	Systematic Name	Standard Name
YCL037C	SRO9	YML071C	COG8
YLR094C	GIS3	YPR046W	MCM16
YDR443C	SSN2	YBR171W	SEC66
YML041C	VPS71	YGL094C	PAN2
YDR225W	HTA1	YLR399C	BDF1
YGR261C	APL6	YCL001W	RER1
YMR224C	MRE11	YOL090W	MSH2
YPR171W	BSP1	YHR016C	YSC84
YNL307C	MCK1	YKR035W-A	DID2
YJR040W	GEF1	YDL006W	PTC1
YPR131C	NAT3	YML095C	RAD10
YLR119W	SRN2	YNL087W	TCB2
YPL120W	VPS30	YNL107W	YAF9
YFR031C-A	RPL2A	YOR112W	CEX1
YML038C	YMD8	YEL051W	VMA8
YGL115W	SNF4	YJR135C	MCM22
YMR167W	MLH1	YNL082W	PMS1
YGR268C	HUA1	YKR030W	GMH1
YDR080W	VPS41	YEL053C	MAK10
YOR327C	SNC2	YDR310C	SUM1
YDR443C	SSN2	YMR016C	SOK2
YNL025C	SSN8	YNL199C	GCR2
YLR234W	TOP3	YDR155C	CPR1
YGL178W	MPT5	YER016W	BIM1
YLR079W	SIC1	YDR335W	MSN5
YGL208W	SIP2	YGL115W	SNF4
YDL192W	ARF1	YER040W	GLN3
YLR150W	STM1	YFR036W	CDC26
YPR131C	NAT3	YLR381W	CTF3

Predictions at threshold level 0.80 continued...			
Gene1 of predicted pair		Gene2 of predicted pair	
Systematic Name	Standard Name	Systematic Name	Standard Name
YML058W	SML1	YDR071C	PAA1
YMR139W	RIM11	YJR094C	IME1
YGR261C	APL6	YGL212W	VAM7
YNL058C	YNL058C	YOL081W	IRA2
YHR200W	RPN10	YLR093C	NYV1
YGR081C	SLX9	YJR090C	GRR1
YBL051C	PIN4	YDL155W	CLB3
YGL087C	MMS2	YFR010W	UBP6
YHR103W	SBE22	YFR021W	ATG18
YGL135W	RPL1B	YLR061W	RPL22A
YDR392W	SPT3	YER107C	GLE2
YJL036W	SNX4	YNL297C	MON2
YGL208W	SIP2	YDR028C	REG1
YLR337C	VRP1	YMR055C	BUB2
YOR080W	DIA2	YPL057C	SUR1
YJL176C	SWI3	YCL037C	SRO9
YOR043W	WHI2	YPL256C	CLN2
YLR119W	SRN2	YBR036C	CSG2
YER161C	SPT2	YGL148W	ARO2
YDR443C	SSN2	YKR035W-A	DID2
YBR274W	CHK1	YOL081W	IRA2
YNL166C	BNI5	YDR318W	MCM21
YDR295C	HDA2	YEL043W	YEL043W
YFR010W	UBP6	YLR180W	SAM1
YKL139W	CTK1	YLR399C	BDF1
YLR192C	HCR1	YIL076W	SEC28
YBR107C	IML3	YKL048C	ELM1
YER144C	UBP5	YHR200W	RPN10
YOR337W	TEA1	YLR441C	RPS1A

Predictions at threshold level 0.80 continued...			
**Gene1 of predicted pair**		**Gene2 of predicted pair**	
**Systematic Name**	**Standard Name**	**Systematic Name**	**Standard Name**
YPL057C	SUR1	YCR077C	PAT1
YLR234W	TOP3	YHR178W	STB5
YDL155W	CLB3	YPR046W	MCM16
YHR013C	ARD1	YKL041W	VPS24
YLR386W	VAC14	YKL101W	HSL1
YBR260C	RGD1	YNL079C	TPM1
YPR018W	RLF2	YML102W	CAC2
YGL115W	SNF4	YFR036W	CDC26
YDR254W	CHL4	YLR370C	ARC18
YLR018C	POM34	YER110C	KAP123
YGL124C	MON1	YHR115C	DMA1
YCR065W	HCM1	YGL124C	MON1
YLR337C	VRP1	YLR370C	ARC18
YKL048C	ELM1	YOR275C	RIM20
YKL116C	PRR1	YMR142C	RPL13B
YEL053C	MAK10	YHL020C	OPI1
YGL178W	MPT5	YLR182W	SWI6
YDR289C	RTT103	YJL190C	RPS22A
YOR290C	SNF2	YEL060C	PRB1
YMR224C	MRE11	YGL078C	DBP3
YPR051W	MAK3	YBR171W	SEC66
YPR018W	RLF2	YPL018W	CTF19
YHR111W	UBA4	YNL289W	PCL1
YIL159W	BNR1	YMR224C	MRE11
YOR195W	SLK19	YML016C	PPZ1
YJR135C	MCM22	YGL151W	NUT1
YMR199W	CLN1	YGL175C	SAE2
YDR225W	HTA1	YBL002W	HTB2
YLR083C	EMP70	YGR020C	VMA7

Predictions at threshold level 0.80 continued...			
Gene1 of predicted pair		Gene2 of predicted pair	
Systematic Name	Standard Name	Systematic Name	Standard Name
YBR131W	CCZ1	YOL018C	TLG2
YGL078C	DBP3	YKL048C	ELM1
YHR200W	RPN10	YGR135W	PRE9
YBR194W	SOY1	YLR350W	ORM2
YJL036W	SNX4	YOR106W	VAM3
YBR030W	YBR030W	YNL307C	MCK1
YOR109W	INP53	YNL127W	FAR11
YIL159W	BNR1	YJL095W	BCK1
YPL234C	TFP3	YLR309C	IMH1
YDR443C	SSN2	YER096W	SHC1
YLR360W	VPS38	YDR245W	MNN10
YNL166C	BNI5	YGL253W	HXK2
YDR379W	RGA2	YGR152C	RSR1
YKL048C	ELM1	YDL083C	RPS16B
YPL001W	HAT1	YCR086W	CSM1
YER087C-B	SBH1	YOR085W	OST3
YNL201C	PSY2	YGR270W	YTA7
YDR004W	RAD57	YDR254W	CHL4
YGL066W	SGF73	YPR036W	VMA13
YDR264C	AKR1	YNL116W	DMA2
YDL020C	RPN4	YBL051C	PIN4
YKL110C	KTI12	YOL054W	PSH1
YPL049C	DIG1	YHR084W	STE12
YLR079W	SIC1	YHR111W	UBA4
YJL177W	RPL17B	YPL240C	HSP82
YNL253W	TEX1	YCL011C	GBP2
YBR173C	UMP1	YHR156C	LIN1
YJL154C	VPS35	YNL238W	KEX2
YLL007C	YLL007C	YJR032W	CPR7

Predictions at threshold level 0.80 continued...			
Gene1 of predicted pair		Gene2 of predicted pair	
Systematic Name	Standard Name	Systematic Name	Standard Name
YGL054C	ERV14	YAL002W	VPS8
YDR335W	MSN5	YER161C	SPT2
YJL157C	FAR1	YGL003C	CDH1
YBR019C	GAL10	YMR311C	GLC8
YOR066W	MSA1	YDR358W	GGA1
YOR066W	MSA1	YFR036W	CDC26
YDL155W	CLB3	YCR077C	PAT1
YKR020W	VPS51	YDR309C	GIC2
YKR001C	VPS1	YPR032W	SRO7
YOR195W	SLK19	YNL054W	VAC7
YGL223C	COG1	YPL120W	VPS30
YDR245W	MNN10	YHR167W	THP2
YMR026C	PEX12	YKR099W	BAS1
YLR337C	VRP1	YBR082C	UBC4
YDR137W	RGP1	YER139C	YER139C
YBR057C	MUM2	YFR036W	CDC26
YBL047C	EDE1	YAL002W	VPS8
YLR102C	APC9	YJL095W	BCK1
YOR039W	CKB2	YJL138C	TIF2
YNL291C	MID1	YNL307C	MCK1
YNL307C	MCK1	YFR036W	CDC26
YPL253C	VIK1	YAL002W	VPS8
YPL253C	VIK1	YGR054W	YGR054W
YER096W	SHC1	YPL161C	BEM4
YBR030W	YBR030W	YML071C	COG8
YCR024C-A	PMP1	YBL079W	NUP170
YPL037C	EGD1	YJL095W	BCK1
YAL024C	LTE1	YMR055C	BUB2
YMR179W	SPT21	YML016C	PPZ1

Predictions at threshold level 0.80 continued...			
Gene1 of predicted pair		Gene2 of predicted pair	
Systematic Name	Standard Name	Systematic Name	Standard Name
YMR224C	MRE11	YBL103C	RTG3
YHR156C	LIN1	YBR108W	YBR108W
YKL139W	CTK1	YPL152W	RRD2
YFR032C-A	RPL29	YBR131W	CCZ1
YBR173C	UMP1	YLR032W	RAD5
YDR443C	SSN2	YPL253C	VIK1
YDR245W	MNN10	YOR141C	ARP8
YDR439W	LRS4	YPL018W	CTF19
YHL027W	RIM101	YLR370C	ARC18
YPL057C	SUR1	YGL124C	MON1
YLR079W	SIC1	YHR115C	DMA1
YFR036W	CDC26	YLR370C	ARC18
YML001W	YPT7	YEL060C	PRB1
YHR060W	VMA22	YOL121C	RPS19A
YKR001C	VPS1	YDR310C	SUM1
YPR120C	CLB5	YBR036C	CSG2
YLR234W	TOP3	YJR090C	GRR1
YDR484W	VPS52	YLR398C	SKI2
YNL032W	SIW14	YHL011C	PRS3
YLR381W	CTF3	YNL147W	LSM7
YPL024W	RMI1	YOL121C	RPS19A
YMR205C	PFK2	YLR371W	ROM2
YMR133W	REC114	YGL003C	CDH1
YNR052C	POP2	YJL124C	LSM1
YJL176C	SWI3	YDR137W	RGP1
YJL183W	MNN11	YGL253W	HXK2
YLR093C	NYV1	YNR007C	ATG3
YGL223C	COG1	YAR014C	BUD14
YDR289C	RTT103	YEL056W	HAT2

Predictions at threshold level 0.80 continued...			
Gene1 of predicted pair		Gene2 of predicted pair	
Systematic Name	Standard Name	Systematic Name	Standard Name
YDL116W	NUP84	YOR195W	SLK19
YGL147C	RPL9A	YBR108W	YBR108W
YBR200W	BEM1	YDR183W	PLP1
YBR107C	IML3	YBR114W	RAD16
YLL007C	YLL007C	YDR289C	RTT103
YNL238W	KEX2	YLR306W	UBC12
YDR245W	MNN10	YDR310C	SUM1
YGR270W	YTA7	YJL006C	CTK2
YPR171W	BSP1	YKL204W	EAP1
YJL157C	FAR1	YDL047W	SIT4
YDR254W	CHL4	YJL183W	MNN11
YGL223C	COG1	YDL192W	ARF1
YPL037C	EGD1	YPR046W	MCM16
YDL006W	PTC1	YOL108C	INO4
YOR109W	INP53	YHR039C-A	VMA10
YER044C	ERG28	YJL183W	MNN11
YJR040W	GEF1	YHR060W	VMA22
YHR013C	ARD1	YGL163C	RAD54
YKR072C	SIS2	YDR146C	SWI5
YMR172W	HOT1	YJR135C	MCM22
YDR261C	EXG2	YLR370C	ARC18
YPL256C	CLN2	YBL087C	RPL23A
YGR072W	UPF3	YGR071C	YGR071C
YMR224C	MRE11	YIR002C	MPH1
YDL116W	NUP84	YAL024C	LTE1
YDR265W	PEX10	YNL214W	PEX17
YHR060W	VMA22	YPL120W	VPS30
YIL159W	BNR1	YEL060C	PRB1
YPR046W	MCM16	YAL002W	VPS8

Predictions at threshold level 0.80 continued...			
Gene1 of predicted pair		Gene2 of predicted pair	
Systematic Name	Standard Name	Systematic Name	Standard Name
YGL033W	HOP2	YBL103C	RTG3
YJR056C	YJR056C	YDR358W	GGA1
YBL008W	HIR1	YDR318W	MCM21
YBL102W	SFT2	YCL037C	SRO9
YML007W	YAP1	YGL208W	SIP2
YNL117W	MLS1	YCR086W	CSM1
YLR360W	VPS38	YPR051W	MAK3
YBR171W	SEC66	YOR066W	MSA1
YJR060W	CBF1	YDR318W	MCM21
YDR137W	RGP1	YEL042W	GDA1
YLR337C	VRP1	YKR014C	YPT52
YDR129C	SAC6	YBL101C	ECM21
YOR197W	MCA1	YPL112C	PEX25
YBR108W	YBR108W	YLR381W	CTF3
YPL096C-A	ERI1	YLR370C	ARC18
YDL116W	NUP84	YNR052C	POP2
YLR447C	VMA6	YGR054W	YGR054W
YGL161C	YIP5	YOR213C	SAS5
YLR018C	POM34	YMR153W	NUP53
YFR031C-A	RPL2A	YLR094C	GIS3
YLR006C	SSK1	YDR379W	RGA2
YCR088W	ABP1	YKR001C	VPS1
YBR173C	UMP1	YOR043W	WHI2
YGR249W	MGA1	YDR137W	RGP1
YFR010W	UBP6	YBR228W	SLX1
YDL127W	PCL2	YFR034C	PHO4
YKL139W	CTK1	YDR363W-A	SEM1
YGL198W	YIP4	YNR049C	MSO1
YML071C	COG8	YJL095W	BCK1

Predictions at threshold level 0.80 continued...			
Gene1 of predicted pair		Gene2 of predicted pair	
Systematic Name	Standard Name	Systematic Name	Standard Name
YJR035W	RAD26	YDR207C	UME6
YOR043W	WHI2	YNL079C	TPM1
YKL048C	ELM1	YGL019W	CKB1
YPL137C	GIP3	YPR030W	CSR2
YBL103C	RTG3	YOL121C	RPS19A
YGR268C	HUA1	YHR016C	YSC84
YBR171W	SEC66	YNL107W	YAF9
YGR020C	VMA7	YPL120W	VPS30
YLR370C	ARC18	YDR310C	SUM1
YML016C	PPZ1	YMR109W	MYO5
YDR080W	VPS41	YJR040W	GEF1
YIL076W	SEC28	YLR094C	GIS3
YJL029C	VPS53	YGL198W	YIP4
YJL124C	LSM1	YEL056W	HAT2
YMR224C	MRE11	YIL036W	CST6
YFR010W	UBP6	YEL053C	MAK10
YPR051W	MAK3	YJL190C	RPS22A
YNL139C	RLR1	YBR111W-A	SUS1
YLR381W	CTF3	YNL248C	RPA49
YMR004W	MVP1	YJR060W	CBF1
YPL253C	VIK1	YDR363W-A	SEM1
YGL212W	VAM7	YDR261C	EXG2
YML115C	VAN1	YNL238W	KEX2
YPR131C	NAT3	YMR199W	CLN1
YGR108W	CLB1	YDR254W	CHL4
YPR018W	RLF2	YDL155W	CLB3
YML001W	YPT7	YNL051W	COG5
YLR337C	VRP1	YGL153W	PEX14
YNL297C	MON2	YML038C	YMD8

Predictions at threshold level 0.80 continued...			
Gene1 of predicted pair		Gene2 of predicted pair	
Systematic Name	Standard Name	Systematic Name	Standard Name
YKL116C	PRR1	YLR398C	SKI2
YHR060W	VMA22	YCL001W	RER1
YOR033C	EXO1	YDR207C	UME6
YOL126C	MDH2	YER115C	SPR6
YPL057C	SUR1	YGL163C	RAD54
YKL025C	PAN3	YPL115C	BEM3
YLR292C	SEC72	YDR318W	MCM21
YPR089W	YPR089W	YMR022W	UBC7
YNR072W	HXT17	YDR261C	EXG2
YHR134W	WSS1	YLR233C	EST1
YCL037C	SRO9	YIR005W	IST3
YOR312C	RPL20B	YEL036C	ANP1
YBR200W	BEM1	YDR261C	EXG2
YMR224C	MRE11	YGL153W	PEX14
YDL101C	DUN1	YML028W	TSA1
YDR146C	SWI5	YHR108W	GGA2
YPL024W	RMI1	YER144C	UBP5
YLR095C	IOC2	YGR249W	MGA1
YCR066W	RAD18	YER162C	RAD4
YDL020C	RPN4	YJR135C	MCM22
YDR320C	SWA2	YHR039C-A	VMA10
YKR001C	VPS1	YGL115W	SNF4
YNL032W	SIW14	YMR133W	REC114
YLR083C	EMP70	YOR127W	RGA1
YJR059W	PTK2	YMR125W	STO1
YLR093C	NYV1	YAL030W	SNC1
YNL044W	YIP3	YNL293W	MSB3
YDR293C	SSD1	YIL008W	URM1
YER114C	BOI2	YHR012W	VPS29

Predictions at threshold level 0.80 continued...			
Gene1 of predicted pair		Gene2 of predicted pair	
Systematic Name	Standard Name	Systematic Name	Standard Name
YDR200C	VPS64	YDL047W	SIT4
YBL103C	RTG3	YGL252C	RTG2
YLR192C	HCR1	YMR311C	GLC8
YKL025C	PAN3	YPL022W	RAD1
YPR051W	MAK3	YCR086W	CSM1
YJR032W	CPR7	YPR119W	CLB2
YMR214W	SCJ1	YDR207C	UME6
YBR173C	UMP1	YER139C	YER139C
YFL013C	IES1	YDR289C	RTT103
YGL066W	SGF73	YML095C	RAD10
YPL024W	RMI1	YDL077C	VAM6
YEL013W	VAC8	YHR167W	THP2
YLR288C	MEC3	YNL215W	IES2
YDR477W	SNF1	YNL087W	TCB2
YCR066W	RAD18	YMR055C	BUB2
YLR234W	TOP3	YPL161C	BEM4
YDR254W	CHL4	YHR200W	RPN10
YPL042C	SSN3	YPR072W	NOT5
YGR019W	UGA1	YOR127W	RGA1
YFL013C	IES1	YFR010W	UBP6
YCL037C	SRO9	YAR014C	BUD14
YGR270W	YTA7	YGR188C	BUB1
YMR109W	MYO5	YPR046W	MCM16
YGR184C	UBR1	YGL163C	RAD54
YGL212W	VAM7	YNR032W	PPG1
YMR198W	CIK1	YPL234C	TFP3
YIL008W	URM1	YGR135W	PRE9
YOR106W	VAM3	YFL033C	RIM15
YDR335W	MSN5	YDR363W-A	SEM1

Predictions at threshold level 0.80 continued...			
**Gene1 of predicted pair**		**Gene2 of predicted pair**	
**Systematic Name**	**Standard Name**	**Systematic Name**	**Standard Name**
YPR131C	NAT3	YLR370C	ARC18
YGR105W	VMA21	YPR032W	SRO7
YER144C	UBP5	YER162C	RAD4
YPR131C	NAT3	YML103C	NUP188
YBR111W-A	SUS1	YDR216W	ADR1
YDR004W	RAD57	YBL079W	NUP170
YMR116C	ASC1	YPL050C	MNN9
YFR036W	CDC26	YMR311C	GLC8
YNL201C	PSY2	YNL106C	INP52
YGR261C	APL6	YOR327C	SNC2
YPR084W	YPR084W	YJR060W	CBF1
YOR001W	RRP6	YCL037C	SRO9
YIL008W	URM1	YJR032W	CPR7
YNL206C	RTT106	YDR440W	DOT1
YJR106W	ECM27	YPR149W	NCE102
YPR089W	YPR089W	YDR310C	SUM1
YMR311C	GLC8	YLR425W	TUS1
YBL102W	SFT2	YML071C	COG8
YDR456W	NHX1	YLR371W	ROM2
YML115C	VAN1	YLR447C	VMA6
YMR142C	RPL13B	YPL161C	BEM4
YPL001W	HAT1	YKL032C	IXR1
YAL024C	LTE1	YML028W	TSA1
YDR245W	MNN10	YLR182W	SWI6
YBR200W	BEM1	YMR179W	SPT21
YKL010C	UFD4	YDR378C	LSM6
YLR381W	CTF3	YOL091W	SPO21
YKR001C	VPS1	YGR105W	VMA21
YCR020C-A	MAK31	YNL051W	COG5

Predictions at threshold level 0.80 continued...			
Gene1 of predicted pair		Gene2 of predicted pair	
Systematic Name	Standard Name	Systematic Name	Standard Name
YDR320C	SWA2	YEL051W	VMA8
YOR332W	VMA4	YJR033C	RAV1
YPL161C	BEM4	YBR280C	SAF1
YBR107C	IML3	YJL183W	MNN11
YOR195W	SLK19	YER116C	SLX8
YJR090C	GRR1	YJL190C	RPS22A
YER116C	SLX8	YKL068W	NUP100
YKL010C	UFD4	YOL081W	IRA2
YFR036W	CDC26	YPR119W	CLB2
YHR134W	WSS1	YML028W	TSA1
YNL069C	RPL16B	YPR032W	SRO7
YBR200W	BEM1	YHR200W	RPN10
YDR137W	RGP1	YOR043W	WHI2
YOR327C	SNC2	YBL025W	RRN10
YBR030W	YBR030W	YHR060W	VMA22
YNL041C	COG6	YNL238W	KEX2
YHL031C	GOS1	YPL234C	TFP3
YBL008W	HIR1	YDR254W	CHL4
YMR153W	NUP53	YML071C	COG8
YNL248C	RPA49	YGR188C	BUB1
YPL057C	SUR1	YOR308C	SNU66
YHR156C	LIN1	YNL325C	FIG4
YMR153W	NUP53	YKL048C	ELM1
YCR077C	PAT1	YBL072C	RPS8A
YPL045W	VPS16	YPL195W	APL5
YJR060W	CBF1	YNL107W	YAF9
YOR332W	VMA4	YGR105W	VMA21
YJL006C	CTK2	YCR077C	PAT1
YDL116W	NUP84	YGL031C	RPL24A

Predictions at threshold level 0.80 continued...			
Gene1 of predicted pair		Gene2 of predicted pair	
Systematic Name	Standard Name	Systematic Name	Standard Name
YER144C	UBP5	YLR371W	ROM2
YGR081C	SLX9	YBL079W	NUP170
YLR320W	MMS22	YDL192W	ARF1
YIL036W	CST6	YLR410W	VIP1
YKL032C	IXR1	YLR370C	ARC18
YOR290C	SNF2	YHR013C	ARD1
YOR290C	SNF2	YDL192W	ARF1
YHL031C	GOS1	YDL002C	NHP10
YFR036W	CDC26	YDL077C	VAM6
YCL032W	STE50	YDR309C	GIC2
YBR194W	SOY1	YDR245W	MNN10
YLR018C	POM34	YGR081C	SLX9
YHR073W	OSH3	YFL047W	RGD2
YDL002C	NHP10	YNL107W	YAF9
YOR023C	AHC1	YGL151W	NUT1
YPL001W	HAT1	YOR368W	RAD17
YHR012W	VPS29	YER162C	RAD4
YKR072C	SIS2	YJL187C	SWE1
YNL307C	MCK1	YOL090W	MSH2
YLR083C	EMP70	YDR080W	VPS41
YNL025C	SSN8	YLR350W	ORM2
YPL137C	GIP3	YER122C	GLO3
YLL007C	YLL007C	YBR114W	RAD16
YPL129W	TAF14	YMR127C	SAS2
YKL048C	ELM1	YDR289C	RTT103
YCL035C	GRX1	YML007W	YAP1
YMR224C	MRE11	YBL047C	EDE1
YDL059C	RAD59	YNL307C	MCK1
YDR080W	VPS41	YPL195W	APL5

Predictions at threshold level 0.80 continued...			
Gene1 of predicted pair		Gene2 of predicted pair	
Systematic Name	Standard Name	Systematic Name	Standard Name
YBL103C	RTG3	YNL238W	KEX2
YNL087W	TCB2	YBR019C	GAL10
YDR443C	SSN2	YPL195W	APL5
YGR261C	APL6	YNL291C	MID1
YNL051W	COG5	YDR207C	UME6
YJL190C	RPS22A	YBR114W	RAD16
YLR410W	VIP1	YDR207C	UME6
YCR077C	PAT1	YEL056W	HAT2
YOR043W	WHI2	YOL121C	RPS19A
YEL056W	HAT2	YBR036C	CSG2
YLR018C	POM34	YML057W	CMP2
YOL126C	MDH2	YNL199C	GCR2
YOL108C	INO4	YKR030W	GMH1
YOR083W	WHI5	YLR370C	ARC18
YGR104C	SRB5	YFR010W	UBP6
YOR212W	STE4	YDR309C	GIC2
YPL001W	HAT1	YBR009C	HHF1
YMR198W	CIK1	YKL041W	VPS24
YJL154C	VPS35	YDR320C	SWA2
YOR014W	RTS1	YPR119W	CLB2
YJR032W	CPR7	YDR310C	SUM1
YNL068C	FKH2	YNL206C	RTT106
YEL053C	MAK10	YAL002W	VPS8
YKL073W	LHS1	YMR214W	SCJ1
YGL054C	ERV14	YDL077C	VAM6
YGL003C	CDH1	YKL114C	APN1
YGL163C	RAD54	YPL256C	CLN2
YLR371W	ROM2	YMR230W	RPS10B
YPL042C	SSN3	YLR386W	VAC14

Predictions at threshold level 0.80 continued...			
Gene1 of predicted pair		Gene2 of predicted pair	
Systematic Name	Standard Name	Systematic Name	Standard Name
YLR102C	APC9	YHR200W	RPN10
YJR140C	HIR3	YML016C	PPZ1
YER001W	MNN1	YJL183W	MNN11
YMR127C	SAS2	YOR023C	AHC1
YPR036W	VMA13	YER044C	ERG28
YOR132W	VPS17	YPL161C	BEM4
YOR023C	AHC1	YMR055C	BUB2
YLR386W	VAC14	YAL010C	MDM10
YDR137W	RGP1	YER040W	GLN3
YDR293C	SSD1	YGR261C	APL6
YMR109W	MYO5	YHR012W	VPS29
YJR040W	GEF1	YKR020W	VPS51
YGL054C	ERV14	YOR327C	SNC2
YDR443C	SSN2	YDR456W	NHX1
YML115C	VAN1	YLR425W	TUS1
YDL116W	NUP84	YLR182W	SWI6
YLR083C	EMP70	YOR112W	CEX1
YDL161W	ENT1	YDR323C	PEP7
YPR028W	YOP1	YNR049C	MSO1
YLR090W	XDJ1	YEL053C	MAK10
YDR183W	PLP1	YDR310C	SUM1
YFR031C-A	RPL2A	YOR270C	VPH1
YOR106W	VAM3	YEL053C	MAK10
YDR320C	SWA2	YNL107W	YAF9
YOR337W	TEA1	YNL041C	COG6
YHR073W	OSH3	YKL068W	NUP100
YLR240W	VPS34	YPL120W	VPS30
YGL200C	EMP24	YNL049C	SFB2
YML115C	VAN1	YEL036C	ANP1

Predictions at threshold level 0.80 continued...			
Gene1 of predicted pair		Gene2 of predicted pair	
Systematic Name	Standard Name	Systematic Name	Standard Name
YBR228W	SLX1	YGL031C	RPL24A
YLR083C	EMP70	YML001W	YPT7
YDL149W	ATG9	YML059C	NTE1
YMR311C	GLC8	YER040W	GLN3
YJL172W	CPS1	YDR358W	GGA1
YOR080W	DIA2	YOR308C	SNU66
YMR319C	FET4	YOR071C	THI71
YPL253C	VIK1	YJL006C	CTK2
YHR134W	WSS1	YMR109W	MYO5
YLR320W	MMS22	YOR327C	SNC2
YBL021C	HAP3	YLR233C	EST1
YLR456W	YLR456W	YIR009W	MSL1
YDL042C	SIR2	YBR009C	HHF1
YBR173C	UMP1	YPL057C	SUR1
YMR054W	STV1	YML071C	COG8
YLR386W	VAC14	YML095C	RAD10
YIR005W	IST3	YGL252C	RTG2
YIR009W	MSL1	YPR101W	SNT309
YHR056C	RSC30	YHR191C	CTF8
YML095C	RAD10	YJR060W	CBF1
YBR278W	DPB3	YOR304W	ISW2
YLR079W	SIC1	YPL022W	RAD1
YJL004C	SYS1	YKR030W	GMH1
YGL237C	HAP2	YAL002W	VPS8
YBR200W	BEM1	YJL136C	RPS21B
YBL102W	SFT2	YDR202C	RAV2
YLR288C	MEC3	YMR199W	CLN1
YDR260C	SWM1	YDR254W	CHL4
YOR191W	RIS1	YBR228W	SLX1

Predictions at threshold level 0.80 continued...			
Gene1 of predicted pair		Gene2 of predicted pair	
Systematic Name	Standard Name	Systematic Name	Standard Name
YBR217W	ATG12	YNL087W	TCB2
YDR507C	GIN4	YDR363W-A	SEM1
YGL212W	VAM7	YMR230W	RPS10B
YDR293C	SSD1	YHR060W	VMA22
YPL024W	RMI1	YPL253C	VIK1
YGL237C	HAP2	YJR032W	CPR7
YGR241C	YAP1802	YPL045W	VPS16
YPR084W	YPR084W	YMR038C	CCS1
YGL147C	RPL9A	YDL223C	HBT1
YGL212W	VAM7	YGR020C	VMA7
YJL004C	SYS1	YDR310C	SUM1
YNL248C	RPA49	YLR254C	NDL1
YDL020C	RPN4	YPR046W	MCM16
YFR010W	UBP6	YHL031C	GOS1
YKR020W	VPS51	YNL079C	TPM1
YIL159W	BNR1	YKR048C	NAP1
YBL047C	EDE1	YBL072C	RPS8A
YBR280C	SAF1	YDR310C	SUM1
YHL002W	HSE1	YLR425W	TUS1
YGR249W	MGA1	YER162C	RAD4
YLR381W	CTF3	YGL237C	HAP2
YDR097C	MSH6	YNL076W	MKS1
YFR031C-A	RPL2A	YDL083C	RPS16B
YGL252C	RTG2	YOL081W	IRA2
YPL057C	SUR1	YHR039C-A	VMA10
YNL069C	RPL16B	YGL223C	COG1
YNL215W	IES2	YDL006W	PTC1
YOR132W	VPS17	YGL253W	HXK2
YMR004W	MVP1	YEL043W	YEL043W

Predictions at threshold level 0.80 continued...			
Gene1 of predicted pair		Gene2 of predicted pair	
Systematic Name	Standard Name	Systematic Name	Standard Name
YOL145C	CTR9	YPL057C	SUR1
YKL048C	ELM1	YHR039C-A	VMA10
YBR173C	UMP1	YHR039C-A	VMA10
YLR216C	CPR6	YDR293C	SSD1
YJR106W	ECM27	YDR105C	TMS1
YHR086W	NAM8	YDR378C	LSM6
YKR072C	SIS2	YNL016W	PUB1
YDL020C	RPN4	YDR207C	UME6
YLR090W	XDJ1	YLR386W	VAC14
YIL076W	SEC28	YOR270C	VPH1
YGL078C	DBP3	YLR398C	SKI2
YMR133W	REC114	YJL183W	MNN11
YHR200W	RPN10	YLR452C	SST2
YNL243W	SLA2	YJL187C	SWE1
YPL106C	SSE1	YJR032W	CPR7
YDR477W	SNF1	YLR094C	GIS3
YOR076C	SKI7	YPL057C	SUR1
YPL253C	VIK1	YDL013W	SLX5
YDR443C	SSN2	YKL139W	CTK1
YNL032W	SIW14	YNL307C	MCK1
YGL163C	RAD54	YPR046W	MCM16
YMR142C	RPL13B	YER044C	ERG28
YDR076W	RAD55	YLR032W	RAD5
YMR091C	NPL6	YLL026W	HSP104
YPR036W	VMA13	YPR119W	CLB2
YPR030W	CSR2	YMR230W	RPS10B
YJR063W	RPA12	YLR410W	VIP1
YNL307C	MCK1	YDR260C	SWM1
YFL023W	BUD27	YPR046W	MCM16

Predictions at threshold level 0.80 continued...			
Gene1 of predicted pair		Gene2 of predicted pair	
Systematic Name	Standard Name	Systematic Name	Standard Name
YGL060W	YBP2	YKL114C	APN1
YNL206C	RTT106	YNL147W	LSM7
YLR288C	MEC3	YDR146C	SWI5
YJL172W	CPS1	YMR311C	GLC8
YMR026C	PEX12	YPR046W	MCM16
YER110C	KAP123	YKL048C	ELM1
YLR216C	CPR6	YER114C	BOI2
YDR293C	SSD1	YDR202C	RAV2
YDL116W	NUP84	YMR284W	YKU70
YLR093C	NYV1	YJL183W	MNN11
YGL212W	VAM7	YJR084W	CSN12
YDR507C	GIN4	YPR040W	TIP41
YDR254W	CHL4	YPL018W	CTF19
YDR484W	VPS52	YCR053W	THR4
YLL039C	UBI4	YOL044W	PEX15
YLR191W	PEX13	YPR173C	VPS4
YJL124C	LSM1	YIR005W	IST3
YMR224C	MRE11	YLR093C	NYV1
YDR176W	NGG1	YPR101W	SNT309
YNL106C	INP52	YCL014W	BUD3
YDL184C	RPL41A	YLR371W	ROM2
YEL009C	GCN4	YDR146C	SWI5
YMR055C	BUB2	YKL110C	KTI12
YDR192C	NUP42	YKR095W	MLP1
YBL101C	ECM21	YLR370C	ARC18
YDL020C	RPN4	YLR292C	SEC72
YJL124C	LSM1	YPL220W	RPL1A
YDL052C	SLC1	YBR041W	FAT1
YLR396C	VPS33	YGL198W	YIP4

Predictions at threshold level 0.80 continued...			
Gene1 of predicted pair		Gene2 of predicted pair	
Systematic Name	Standard Name	Systematic Name	Standard Name
YMR153W	NUP53	YHR060W	VMA22
YNL307C	MCK1	YLR254C	NDL1
YBR274W	CHK1	YJL138C	TIF2
YHR119W	SET1	YLR015W	BRE2
YFR036W	CDC26	YML028W	TSA1
YDR146C	SWI5	YOR140W	SFL1
YPL024W	RMI1	YLR180W	SAM1
YOR033C	EXO1	YMR231W	PEP5
YBL008W	HIR1	YBR278W	DPB3
YLR410W	VIP1	YGL252C	RTG2
YDL184C	RPL41A	YPL220W	RPL1A
YGL060W	YBP2	YMR198W	CIK1
YER129W	SAK1	YDR028C	REG1
YLR386W	VAC14	YGL237C	HAP2
YDR443C	SSN2	YMR311C	GLC8
YPR046W	MCM16	YDR183W	PLP1
YKL048C	ELM1	YJR135C	MCM22
YHR079C	IRE1	YCR053W	THR4
YPL022W	RAD1	YPR119W	CLB2
YJL058C	BIT61	YJR035W	RAD26
YKL139W	CTK1	YKR035W-A	DID2
YMR199W	CLN1	YMR231W	PEP5
YBR108W	YBR108W	YGR188C	BUB1
YDR108W	GSG1	YGR071C	YGR071C
YNL201C	PSY2	YHR111W	UBA4
YIL076W	SEC28	YOR106W	VAM3
YHR073W	OSH3	YIL148W	RPL40A
YDR395W	SXM1	YML103C	NUP188
YHL031C	GOS1	YEL013W	VAC8

Predictions at threshold level 0.80 continued...			
Gene1 of predicted pair		Gene2 of predicted pair	
Systematic Name	Standard Name	Systematic Name	Standard Name
YGR108W	CLB1	YML016C	PPZ1
YGR014W	MSB2	YLR398C	SKI2
YOR304W	ISW2	YMR042W	ARG80
YMR216C	SKY1	YCL037C	SRO9
YLR234W	TOP3	YJR035W	RAD26
YCR084C	TUP1	YBR009C	HHF1
YIL076W	SEC28	YOR109W	INP53
YLR360W	VPS38	YPL234C	TFP3
YLR441C	RPS1A	YML071C	COG8
YLR079W	SIC1	YIL148W	RPL40A
YPL253C	VIK1	YHR114W	BZZ1
YKL139W	CTK1	YLR381W	CTF3
YER110C	KAP123	YMR125W	STO1
YDR223W	CRF1	YJL141C	YAK1
YBL047C	EDE1	YJL183W	MNN11
YOR358W	HAP5	YPR046W	MCM16
YBL051C	PIN4	YDR309C	GIC2
YJR032W	CPR7	YPR030W	CSR2
YAL024C	LTE1	YJR140C	HIR3
YDR261C	EXG2	YCR053W	THR4
YDR245W	MNN10	YER139C	YER139C
YGR252W	GCN5	YNL079C	TPM1
YKL025C	PAN3	YGR019W	UGA1
YPL137C	GIP3	YJL183W	MNN11
YKL048C	ELM1	YPR120C	CLB5
YNL201C	PSY2	YML016C	PPZ1
YJL124C	LSM1	YER122C	GLO3
YKL116C	PRR1	YER144C	UBP5
YJL172W	CPS1	YMR214W	SCJ1

Predictions at threshold level 0.80 continued...			
Gene1 of predicted pair		Gene2 of predicted pair	
Systematic Name	Standard Name	Systematic Name	Standard Name
YLR360W	VPS38	YJR032W	CPR7
YPL042C	SSN3	YPL161C	BEM4
YHR039C-A	VMA10	YER120W	SCS2
YPL024W	RMI1	YBR019C	GAL10
YLR441C	RPS1A	YER122C	GLO3
YER110C	KAP123	YNL031C	HHT2
YOR112W	CEX1	YNL107W	YAF9
YBR195C	MSI1	YDR076W	RAD55
YIL159W	BNR1	YLR094C	GIS3
YBR030W	YBR030W	YBR200W	BEM1
YKR082W	NUP133	YER105C	NUP157
YOR033C	EXO1	YDL013W	SLX5
YJR040W	GEF1	YOR327C	SNC2
YLR292C	SEC72	YOR132W	VPS17
YIL053W	RHR2	YBL103C	RTG3
YLR360W	VPS38	YDR456W	NHX1
YFR010W	UBP6	YPL079W	RPL21B
YLL007C	YLL007C	YLR381W	CTF3
YDR477W	SNF1	YDR422C	SIP1
YGL115W	SNF4	YBL025W	RRN10
YLR079W	SIC1	YJL006C	CTK2
YMR167W	MLH1	YKL101W	HSL1
YIL159W	BNR1	YFR010W	UBP6
YBR030W	YBR030W	YPR030W	CSR2
YLR150W	STM1	YNL307C	MCK1
YHR156C	LIN1	YBL025W	RRN10
YDL223C	HBT1	YNL238W	KEX2
YCL063W	VAC17	YFR021W	ATG18
YBR200W	BEM1	YOR035C	SHE4

Predictions at threshold level 0.80 continued...			
**Gene1 of predicted pair**		**Gene2 of predicted pair**	
**Systematic Name**	**Standard Name**	**Systematic Name**	**Standard Name**
YOR106W	VAM3	YPR032W	SRO7
YNL093W	YPT53	YNL044W	YIP3
YNL041C	COG6	YBL025W	RRN10
YMR311C	GLC8	YBR036C	CSG2
YPL256C	CLN2	YER027C	GAL83
YLR360W	VPS38	YOR140W	SFL1
YLR079W	SIC1	YOR312C	RPL20B
YER144C	UBP5	YLR093C	NYV1
YDL155W	CLB3	YDR183W	PLP1
YMR172W	HOT1	YDR318W	MCM21
YDL002C	NHP10	YNL199C	GCR2
YDR385W	EFT2	YGL200C	EMP24
YOL018C	TLG2	YNL051W	COG5
YGL033W	HOP2	YKL139W	CTK1
YLR043C	TRX1	YGL212W	VAM7
YML115C	VAN1	YBL101C	ECM21
YMR142C	RPL13B	YDR310C	SUM1
YGL115W	SNF4	YGR105W	VMA21
YCL001W	RER1	YPL161C	BEM4
YBR200W	BEM1	YJL124C	LSM1
YIL053W	RHR2	YPR030W	CSR2
YNL225C	CNM67	YHR115C	DMA1
YPL256C	CLN2	YPR119W	CLB2
YLR150W	STM1	YGL246C	RAI1
YOL067C	RTG1	YHL002W	HSE1
YNL218W	MGS1	YHR167W	THP2
YJL154C	VPS35	YJL053W	PEP8
YHR156C	LIN1	YKL048C	ELM1
YPL057C	SUR1	YJL183W	MNN11

Predictions at threshold level 0.80 continued...			
Gene1 of predicted pair		Gene2 of predicted pair	
Systematic Name	Standard Name	Systematic Name	Standard Name
YDR080W	VPS41	YGL223C	COG1
YPR036W	VMA13	YGL003C	CDH1
YNL041C	COG6	YBR036C	CSG2
YPL079W	RPL21B	YHR077C	NMD2
YPL024W	RMI1	YBL058W	SHP1
YMR224C	MRE11	YDL013W	SLX5
YNL068C	FKH2	YGL066W	SGF73
YJL183W	MNN11	YOR327C	SNC2
YKL160W	ELF1	YER139C	YER139C
YLR150W	STM1	YGL163C	RAD54
YBL058W	SHP1	YOR290C	SNF2
YMR153W	NUP53	YJR032W	CPR7
YER047C	SAP1	YPR119W	CLB2
YJR024C	MDE1	YMR125W	STO1
YFR040W	SAP155	YPL256C	CLN2
YNL068C	FKH2	YMR199W	CLN1
YDR108W	GSG1	YGL115W	SNF4
YDR245W	MNN10	YGL153W	PEX14
YMR224C	MRE11	YJL183W	MNN11
YPL022W	RAD1	YLR032W	RAD5
YPR051W	MAK3	YGL223C	COG1
YLR360W	VPS38	YJL004C	SYS1
YOR109W	INP53	YEL053C	MAK10
YNL069C	RPL16B	YMR055C	BUB2
YKR001C	VPS1	YPL195W	APL5
YMR198W	CIK1	YDL047W	SIT4
YKR061W	KTR2	YHR013C	ARD1
YNL215W	IES2	YOR038C	HIR2
YPL161C	BEM4	YNL229C	URE2

Predictions at threshold level 0.80 continued...			
Gene1 of predicted pair		Gene2 of predicted pair	
Systematic Name	Standard Name	Systematic Name	Standard Name
YOR140W	SFL1	YER096W	SHC1
YOR109W	INP53	YDL006W	PTC1
YJR059W	PTK2	YER115C	SPR6
YML016C	PPZ1	YIL008W	URM1
YJR140C	HIR3	YEL013W	VAC8
YPL037C	EGD1	YMR054W	STV1
YNL054W	VAC7	YJR135C	MCM22
YOL148C	SPT20	YBR089C-A	NHP6B
YBR173C	UMP1	YMR311C	GLC8
YMR004W	MVP1	YJR032W	CPR7
YKL129C	MYO3	YLR370C	ARC18
YBL103C	RTG3	YLR452C	SST2
YOR132W	VPS17	YGR054W	YGR054W
YPL234C	TFP3	YJR033C	RAV1
YGR072W	UPF3	YOR069W	VPS5
YOR127W	RGA1	YOR109W	INP53
YMR153W	NUP53	YLR441C	RPS1A
YGR135W	PRE9	YGR188C	BUB1
YLR083C	EMP70	YEL043W	YEL043W
YGR072W	UPF3	YJR060W	CBF1
YDR378C	LSM6	YDR363W-A	SEM1
YPR018W	RLF2	YMR199W	CLN1
YPR072W	NOT5	YJL164C	TPK1
YNR052C	POP2	YGR270W	YTA7
YNL054W	VAC7	YNL307C	MCK1
YGR239C	PEX21	YIL160C	POT1
YLR441C	RPS1A	YML001W	YPT7
YLR370C	ARC18	YKL041W	VPS24
YHR200W	RPN10	YDL077C	VAM6

Predictions at threshold level 0.80 continued...			
Gene1 of predicted pair		Gene2 of predicted pair	
Systematic Name	Standard Name	Systematic Name	Standard Name
YGR054W	YGR054W	YBR114W	RAD16
YDR443C	SSN2	YDR484W	VPS52
YLR192C	HCR1	YER044C	ERG28
YLR360W	VPS38	YGL054C	ERV14
YOR275C	RIM20	YGL163C	RAD54
YOR337W	TEA1	YKR099W	BAS1
YLR061W	RPL22A	YBR082C	UBC4
YKL006W	RPL14A	YKL032C	IXR1
YPL240C	HSP82	YJR086W	STE18
YJL183W	MNN11	YLR425W	TUS1
YBR084C-A	RPL19A	YDR379W	RGA2
YKL126W	YPK1	YNL076W	MKS1
YPR046W	MCM16	YCR053W	THR4
YML058W	SML1	YLR079W	SIC1
YPL253C	VIK1	YDR289C	RTT103
YMR142C	RPL13B	YFL023W	BUD27
YDR146C	SWI5	YBR200W	BEM1
YDR289C	RTT103	YMR038C	CCS1
YLR292C	SEC72	YHL002W	HSE1
YHR041C	SRB2	YBR089C-A	NHP6B
YMR016C	SOK2	YHR060W	VMA22
YNR052C	POP2	YHR115C	DMA1
YIL053W	RHR2	YDR477W	SNF1
YOR080W	DIA2	YGL175C	SAE2
YHR167W	THP2	YOR124C	UBP2
YIL053W	RHR2	YDR484W	VPS52
YOR043W	WHI2	YMR214W	SCJ1
YNL087W	TCB2	YDR261C	EXG2
YBL058W	SHP1	YGL003C	CDH1

Predictions at threshold level 0.80 continued...			
Gene1 of predicted pair		Gene2 of predicted pair	
Systematic Name	Standard Name	Systematic Name	Standard Name
YBL047C	EDE1	YLR370C	ARC18
YBR127C	VMA2	YPL234C	TFP3
YBL101C	ECM21	YKL041W	VPS24
YHR200W	RPN10	YNL107W	YAF9
YHR134W	WSS1	YJL183W	MNN11
YBR171W	SEC66	YER162C	RAD4
YOR106W	VAM3	YLR396C	VPS33
YDR076W	RAD55	YHR010W	RPL27A
YHR012W	VPS29	YKL119C	VPH2
YDR260C	SWM1	YGL003C	CDH1
YLR079W	SIC1	YDR171W	HSP42
YER044C	ERG28	YCR053W	THR4
YDL160C	DHH1	YDR448W	ADA2
YOR001W	RRP6	YPR120C	CLB5
YFR031C-A	RPL2A	YGL124C	MON1
YIL159W	BNR1	YPR040W	TIP41
YHR111W	UBA4	YIR005W	IST3
YLR418C	CDC73	YHR056C	RSC30
YER110C	KAP123	YDR183W	PLP1
YDR225W	HTA1	YBR010W	HHT1
YAL024C	LTE1	YGL252C	RTG2
YBL008W	HIR1	YPL253C	VIK1
YLR381W	CTF3	YDR144C	MKC7
YGL133W	ITC1	YNL229C	URE2
YNL243W	SLA2	YNL271C	BNI1
YPL105C	YPL105C	YDR207C	UME6
YJL053W	PEP8	YGL095C	VPS45
YDL160C	DHH1	YNL147W	LSM7
YDR080W	VPS41	YPL234C	TFP3

Predictions at threshold level 0.80 continued...			
Gene1 of predicted pair		Gene2 of predicted pair	
Systematic Name	Standard Name	Systematic Name	Standard Name
YPL129W	TAF14	YBR112C	CYC8
YLR370C	ARC18	YER040W	GLN3
YLR093C	NYV1	YJL095W	BCK1
YHL031C	GOS1	YDL077C	VAM6
YDR225W	HTA1	YBR289W	SNF5
YPL120W	VPS30	YPR032W	SRO7
YJR063W	RPA12	YFR036W	CDC26
YNL098C	RAS2	YIL033C	BCY1
YNL087W	TCB2	YNL041C	COG6
YIL076W	SEC28	YNL107W	YAF9
YER144C	UBP5	YML001W	YPT7
YBR108W	YBR108W	YHR114W	BZZ1
YDR146C	SWI5	YOR106W	VAM3
YML115C	VAN1	YMR038C	CCS1
YHR111W	UBA4	YIL008W	URM1
YLL039C	UBI4	YLR425W	TUS1
YLR094C	GIS3	YJR084W	CSN12
YML058W	SML1	YIL066C	RNR3
YPR036W	VMA13	YJL095W	BCK1
YHR167W	THP2	YAL002W	VPS8
YPL018W	CTF19	YMR038C	CCS1
YHL002W	HSE1	YAR014C	BUD14
YBR030W	YBR030W	YOR290C	SNF2
YOR001W	RRP6	YER041W	YEN1
YNL288W	CAF40	YHR134W	WSS1
YGR072W	UPF3	YDR202C	RAV2
YDR477W	SNF1	YNL032W	SIW14
YNR052C	POP2	YBL079W	NUP170
YDR378C	LSM6	YGL200C	EMP24

Predictions at threshold level 0.80 continued...			
Gene1 of predicted pair		Gene2 of predicted pair	
Systematic Name	Standard Name	Systematic Name	Standard Name
YMR055C	BUB2	YGR270W	YTA7
YDR443C	SSN2	YPL037C	EGD1
YKR072C	SIS2	YDR293C	SSD1
YGR135W	PRE9	YKL041W	VPS24
YER116C	SLX8	YKR095W	MLP1
YJL154C	VPS35	YER120W	SCS2
YBR171W	SEC66	YLR233C	EST1
YEL060C	PRB1	YLR180W	SAM1
YNL032W	SIW14	YER044C	ERG28
YDL160C	DHH1	YDR378C	LSM6
YGL253W	HXK2	YDR310C	SUM1
YJR056C	YJR056C	YDL192W	ARF1
YML038C	YMD8	YGL054C	ERV14
YFR010W	UBP6	YDR289C	RTT103
YBR030W	YBR030W	YDR310C	SUM1
YOR043W	WHI2	YDL192W	ARF1
YDR264C	AKR1	YDL047W	SIT4
YNL243W	SLA2	YAL030W	SNC1
YJR040W	GEF1	YPL105C	YPL105C
YDL160C	DHH1	YJL187C	SWE1
YHR079C	IRE1	YFL031W	HAC1
YKL101W	HSL1	YDR310C	SUM1
YER139C	YER139C	YCR053W	THR4
YGL212W	VAM7	YLR320W	MMS22
YPL137C	GIP3	YOL121C	RPS19A
YIL076W	SEC28	YER162C	RAD4
YMR095C	SNO1	YGL003C	CDH1
YAL024C	LTE1	YHR060W	VMA22
YBL058W	SHP1	YJR032W	CPR7

Predictions at threshold level 0.80 continued...			
Gene1 of predicted pair		Gene2 of predicted pair	
Systematic Name	Standard Name	Systematic Name	Standard Name
YGL115W	SNF4	YMR214W	SCJ1
YMR026C	PEX12	YGR077C	PEX8
YDR244W	PEX5	YAL055W	PEX22
YMR199W	CLN1	YLR352W	YLR352W
YGL190C	CDC55	YAL011W	SWC3
YDL020C	RPN4	YKL119C	VPH2
YMR016C	SOK2	YFR036W	CDC26
YOR145C	PNO1	YPL240C	HSP82
YOR035C	SHE4	YER139C	YER139C
YDR129C	SAC6	YML071C	COG8
YOR140W	SFL1	YJL095W	BCK1
YOR106W	VAM3	YGL124C	MON1
YJL060W	BNA3	YMR198W	CIK1
YPR018W	RLF2	YGR188C	BUB1
YMR055C	BUB2	YGL163C	RAD54
YMR199W	CLN1	YJR090C	GRR1
YDR245W	MNN10	YDL006W	PTC1
YPL037C	EGD1	YOR066W	MSA1
YGL066W	SGF73	YOR290C	SNF2
YJL176C	SWI3	YJR140C	HIR3
YEL065W	SIT1	YJL095W	BCK1
YPL161C	BEM4	YHR039C-A	VMA10
YOR195W	SLK19	YOR080W	DIA2
YNL236W	SIN4	YPL018W	CTF19
YJR021C	REC107	YGL090W	LIF1
YMR198W	CIK1	YPL106C	SSE1
YOR327C	SNC2	YMR311C	GLC8
YDR379W	RGA2	YIL008W	URM1
YER107C	GLE2	YDR485C	VPS72

Predictions at threshold level 0.80 continued...			
Gene1 of predicted pair		Gene2 of predicted pair	
Systematic Name	Standard Name	Systematic Name	Standard Name
YJL006C	CTK2	YPR119W	CLB2
YFR021W	ATG18	YML059C	NTE1
YBR057C	MUM2	YLR150W	STM1
YPL022W	RAD1	YNL107W	YAF9
YOR069W	VPS5	YER122C	GLO3
YDL006W	PTC1	YBL101C	ECM21
YLR381W	CTF3	YML112W	CTK3
YNR052C	POP2	YPR120C	CLB5
YDR004W	RAD57	YML028W	TSA1
YGR019W	UGA1	YLR292C	SEC72
YCR077C	PAT1	YNL079C	TPM1
YBL103C	RTG3	YOL081W	IRA2
YDR363W-A	SEM1	YDR071C	PAA1
YOR005C	DNL4	YLR265C	NEJ1
YGR184C	UBR1	YDL155W	CLB3
YMR139W	RIM11	YML099C	ARG81
YNL032W	SIW14	YOR327C	SNC2
YER087C-B	SBH1	YOR026W	BUB3
YOL111C	MDY2	YHR039C-A	VMA10
YNL264C	PDR17	YBR194W	SOY1
YPR017C	DSS4	YML001W	YPT7
YPL042C	SSN3	YJL124C	LSM1
YAL024C	LTE1	YGR152C	RSR1
YNL079C	TPM1	YER049W	TPA1
YKR035W-A	DID2	YER040W	GLN3
YDR329C	PEX3	YLR191W	PEX13
YMR224C	MRE11	YGR270W	YTA7
YBR084C-A	RPL19A	YGL163C	RAD54
YNL309W	STB1	YDL047W	SIT4

Predictions at threshold level 0.80 continued...			
Gene1 of predicted pair		Gene2 of predicted pair	
Systematic Name	Standard Name	Systematic Name	Standard Name
YPL213W	LEA1	YDL155W	CLB3
YML109W	ZDS2	YMR032W	HOF1
YMR016C	SOK2	YIL038C	NOT3
YPL037C	EGD1	YJR060W	CBF1
YMR133W	REC114	YMR311C	GLC8
YJL172W	CPS1	YPL037C	EGD1
YLR058C	SHM2	YBR200W	BEM1
YIL034C	CAP2	YIL133C	RPL16A
YOR033C	EXO1	YLR352W	YLR352W
YEL043W	YEL043W	YOL108C	INO4
YGL033W	HOP2	YNR072W	HXT17
YJL124C	LSM1	YBL079W	NUP170
YMR004W	MVP1	YEL051W	VMA8
YDL160C	DHH1	YDR507C	GIN4
YLR093C	NYV1	YER040W	GLN3
YNL069C	RPL16B	YJR040W	GEF1
YKL025C	PAN3	YGL094C	PAN2
YKR048C	NAP1	YKL101W	HSL1
YJR032W	CPR7	YJR033C	RAV1
YOR112W	CEX1	YPL120W	VPS30
YHR098C	SFB3	YNL233W	BNI4
YOR101W	RAS1	YGL025C	PGD1
YER068W	MOT2	YGL094C	PAN2
YHL006C	SHU1	YLR376C	PSY3
YDR156W	RPA14	YFR036W	CDC26
YBR258C	SHG1	YHR115C	DMA1
YFR031C-A	RPL2A	YEL053C	MAK10
YML103C	NUP188	YMR125W	STO1
YPR017C	DSS4	YHR012W	VPS29

Predictions at threshold level 0.80 continued...			
Gene1 of predicted pair		Gene2 of predicted pair	
Systematic Name	Standard Name	Systematic Name	Standard Name
YMR224C	MRE11	YKL101W	HSL1
YJL117W	PHO86	YMR038C	CCS1
YBL102W	SFT2	YOR327C	SNC2
YGR072W	UPF3	YNR032W	PPG1
YNL227C	JJJ1	YBR119W	MUD1
YGR019W	UGA1	YMR109W	MYO5
YAL024C	LTE1	YNL054W	VAC7
YPL115C	BEM3	YLR234W	TOP3
YNL069C	RPL16B	YHR039C-A	VMA10
YMR231W	PEP5	YOR368W	RAD17
YKL139W	CTK1	YOR308C	SNU66
YBR057C	MUM2	YPR120C	CLB5
YNL106C	INP52	YGL031C	RPL24A
YDL020C	RPN4	YGL163C	RAD54
YLR216C	CPR6	YAR014C	BUD14
YJL138C	TIF2	YOL081W	IRA2
YIL156W	UBP7	YKL129C	MYO3
YML115C	VAN1	YMR205C	PFK2
YHR178W	STB5	YDL002C	NHP10
YER116C	SLX8	YHR060W	VMA22
YML071C	COG8	YBR181C	RPS6B
YPR084W	YPR084W	YOL108C	INO4
YML115C	VAN1	YKL080W	VMA5
YMR172W	HOT1	YBR107C	IML3
YBR171W	SEC66	YKL048C	ELM1
YFR031C-A	RPL2A	YLR180W	SAM1
YMR198W	CIK1	YGR014W	MSB2
YPR051W	MAK3	YHR012W	VPS29
YGR252W	GCN5	YIL038C	NOT3

Predictions at threshold level 0.80 continued...			
Gene1 of predicted pair		Gene2 of predicted pair	
Systematic Name	Standard Name	Systematic Name	Standard Name
YOR106W	VAM3	YLR371W	ROM2
YLR095C	IOC2	YNR072W	HXT17
YMR109W	MYO5	YJL183W	MNN11
YMR199W	CLN1	YHR205W	SCH9
YEL065W	SIT1	YNL236W	SIN4
YNL215W	IES2	YOR001W	RRP6
YMR080C	NAM7	YGR071C	YGR071C
YLR337C	VRP1	YBR108W	YBR108W
YOR127W	RGA1	YPL253C	VIK1
YJL127C	SPT10	YPR120C	CLB5
YLR360W	VPS38	YCL001W	RER1
YKL025C	PAN3	YLR386W	VAC14
YLR135W	SLX4	YCL037C	SRO9
YOR127W	RGA1	YJL095W	BCK1
YJR032W	CPR7	YML071C	COG8
YMR080C	NAM7	YJL176C	SWI3
YBR131W	CCZ1	YER122C	GLO3
YIL076W	SEC28	YBL025W	RRN10
YPR036W	VMA13	YEL053C	MAK10
YLR090W	XDJ1	YML071C	COG8
YBL103C	RTG3	YOR043W	WHI2
YDL020C	RPN4	YER116C	SLX8
YOL051W	GAL11	YPL248C	GAL4
YGL237C	HAP2	YDR289C	RTT103
YER144C	UBP5	YNL238W	KEX2
YNL291C	MID1	YNR032W	PPG1
YOR080W	DIA2	YGL115W	SNF4
YBL103C	RTG3	YEL053C	MAK10
YBL021C	HAP3	YGL237C	HAP2

Predictions at threshold level 0.80 continued...			
Gene1 of predicted pair		Gene2 of predicted pair	
Systematic Name	Standard Name	Systematic Name	Standard Name
YFR031C-A	RPL2A	YJL183W	MNN11
YGL223C	COG1	YNR049C	MSO1
YJL095W	BCK1	YAL002W	VPS8
YPL042C	SSN3	YNL107W	YAF9
YBR126C	TPS1	YNR072W	HXT17
YLR441C	RPS1A	YOL121C	RPS19A
YNL325C	FIG4	YPL137C	GIP3
YMR224C	MRE11	YJR090C	GRR1
YMR153W	NUP53	YOL111C	MDY2
YMR224C	MRE11	YPL256C	CLN2
YPL183C	YPL183C	YBR061C	TRM7
YBL008W	HIR1	YDR363W-A	SEM1
YPL161C	BEM4	YGR188C	BUB1
YPR120C	CLB5	YOR038C	HIR2
YPR018W	RLF2	YOL051W	GAL11
YPL253C	VIK1	YLR399C	BDF1
YDR225W	HTA1	YBR111W-A	SUS1
YGL178W	MPT5	YGR252W	GCN5
YDL223C	HBT1	YNR072W	HXT17
YGL025C	PGD1	YNL058C	YNL058C
YMR202W	ERG2	YGL003C	CDH1
YEL043W	YEL043W	YNL107W	YAF9
YJL176C	SWI3	YOR243C	PUS7
YHR098C	SFB3	YGL054C	ERV14
YMR116C	ASC1	YEL036C	ANP1
YJL176C	SWI3	YDR385W	EFT2
YEL053C	MAK10	YEL051W	VMA8
YOR112W	CEX1	YCL001W	RER1
YER114C	BOI2	YHR115C	DMA1

Predictions at threshold level 0.80 continued...			
**Gene1 of predicted pair**		**Gene2 of predicted pair**	
**Systematic Name**	**Standard Name**	**Systematic Name**	**Standard Name**
YJR063W	RPA12	YPR120C	CLB5
YHR206W	SKN7	YOR208W	PTP2
YOR127W	RGA1	YNL107W	YAF9
YEL027W	CUP5	YEL051W	VMA8
YOR337W	TEA1	YBL103C	RTG3
YDR080W	VPS41	YLR093C	NYV1
YBL047C	EDE1	YOL081W	IRA2
YGL066W	SGF73	YKL048C	ELM1
YFR032C-A	RPL29	YPL089C	RLM1
YGR105W	VMA21	YLR131C	ACE2
YFR034C	PHO4	YNL289W	PCL1
YPL129W	TAF14	YNL236W	SIN4
YMR164C	MSS11	YMR273C	ZDS1
YML112W	CTK3	YAR014C	BUD14
YFL013C	IES1	YCL037C	SRO9
YDL185W	TFP1	YMR054W	STV1
YGL100W	SEH1	YOR368W	RAD17
YIL076W	SEC28	YDL077C	VAM6
YDR076W	RAD55	YKL114C	APN1
YPR131C	NAT3	YML102W	CAC2
YDR293C	SSD1	YGR105W	VMA21
YNL206C	RTT106	YKR048C	NAP1
YGR188C	BUB1	YNL107W	YAF9
YPL022W	RAD1	YLR370C	ARC18
YMR199W	CLN1	YMR273C	ZDS1
YBR030W	YBR030W	YML001W	YPT7
YFR010W	UBP6	YCL037C	SRO9
YDR477W	SNF1	YDL229W	SSB1
YNR031C	SSK2	YPR119W	CLB2

Predictions at threshold level 0.80 continued...			
Gene1 of predicted pair		Gene2 of predicted pair	
Systematic Name	Standard Name	Systematic Name	Standard Name
YNL087W	TCB2	YER162C	RAD4
YGL175C	SAE2	YBR082C	UBC4
YDR080W	VPS41	YJR060W	CBF1
YMR016C	SOK2	YJL172W	CPS1
YOR308C	SNU66	YML097C	VPS9
YOR066W	MSA1	YBR280C	SAF1
YMR038C	CCS1	YKL114C	APN1
YML103C	NUP188	YKL068W	NUP100
YLR254C	NDL1	YOL081W	IRA2
YBR009C	HHF1	YDR071C	PAA1
YLL039C	UBI4	YJL183W	MNN11
YLR148W	PEP3	YML001W	YPT7
YDL005C	MED2	YPL248C	GAL4
YOR043W	WHI2	YLR452C	SST2
YHR013C	ARD1	YJR135C	MCM22
YGR104C	SRB5	YOR038C	HIR2
YMR133W	REC114	YGL124C	MON1
YAL024C	LTE1	YPL024W	RMI1
YCR088W	ABP1	YGL095C	VPS45
YGR270W	YTA7	YPL018W	CTF19
YPL161C	BEM4	YBL101C	ECM21
YDR293C	SSD1	YEL053C	MAK10
YDR129C	SAC6	YPL050C	MNN9
YNL016W	PUB1	YGL175C	SAE2
YPL057C	SUR1	YDR335W	MSN5
YFR030W	MET10	YOL054W	PSH1
YER162C	RAD4	YCR053W	THR4
YNL215W	IES2	YOR080W	DIA2
YDR260C	SWM1	YHR200W	RPN10

Predictions at threshold level 0.80 continued...			
Gene1 of predicted pair		Gene2 of predicted pair	
Systematic Name	Standard Name	Systematic Name	Standard Name
YER139C	YER139C	YGL124C	MON1
YLR441C	RPS1A	YJL095W	BCK1
YCL037C	SRO9	YOL108C	INO4
YML115C	VAN1	YHR039C-A	VMA10
YOR069W	VPS5	YDR137W	RGP1
YNL243W	SLA2	YLR332W	MID2
YNL051W	COG5	YKR030W	GMH1
YLR079W	SIC1	YGR270W	YTA7
YOR106W	VAM3	YML028W	TSA1
YMR153W	NUP53	YER040W	GLN3
YML115C	VAN1	YMR311C	GLC8
YLR313C	SPH1	YGL031C	RPL24A
YNL032W	SIW14	YDL223C	HBT1
YOR023C	AHC1	YMR109W	MYO5
YPL018W	CTF19	YHR115C	DMA1
YDR110W	FOB1	YPR101W	SNT309
YML013W	UBX2	YDR310C	SUM1
YMR198W	CIK1	YKR001C	VPS1
YOL121C	RPS19A	YLR370C	ARC18
YDR484W	VPS52	YLR320W	MMS22
YBR112C	CYC8	YGL035C	MIG1
YBR228W	SLX1	YML028W	TSA1
YOR127W	RGA1	YOR069W	VPS5
YPR084W	YPR084W	YDL077C	VAM6
YLR094C	GIS3	YNL041C	COG6
YKR099W	BAS1	YPR046W	MCM16
YLR079W	SIC1	YJR059W	PTK2
YMR004W	MVP1	YML038C	YMD8
YOR080W	DIA2	YMR179W	SPT21

Predictions at threshold level 0.80 continued...			
Gene1 of predicted pair		Gene2 of predicted pair	
Systematic Name	Standard Name	Systematic Name	Standard Name
YDL013W	SLX5	YJR035W	RAD26
YIR009W	MSL1	YGL031C	RPL24A
YDR310C	SUM1	YLR182W	SWI6
YDR484W	VPS52	YGL212W	VAM7
YBR030W	YBR030W	YMR016C	SOK2
YGR072W	UPF3	YPL105C	YPL105C
YEL027W	CUP5	YGR105W	VMA21
YJR040W	GEF1	YAL002W	VPS8
YPL042C	SSN3	YOL145C	CTR9
YDR137W	RGP1	YLR131C	ACE2
YHL031C	GOS1	YNR032W	PPG1
YNL069C	RPL16B	YOR069W	VPS5
YMR205C	PFK2	YBR041W	FAT1
YLR083C	EMP70	YGR105W	VMA21
YHL020C	OPI1	YMR054W	STV1
YLR386W	VAC14	YKR061W	KTR2
YGR081C	SLX9	YML102W	CAC2
YDR443C	SSN2	YDR245W	MNN10
YHR052W	CIC1	YER002W	NOP16
YLR410W	VIP1	YGL175C	SAE2
YML016C	PPZ1	YBR260C	RGD1
YMR026C	PEX12	YOR035C	SHE4
YBR274W	CHK1	YLR032W	RAD5
YBR173C	UMP1	YOL121C	RPS19A
YCR077C	PAT1	YPR046W	MCM16
YFR031C-A	RPL2A	YNL076W	MKS1
YEL053C	MAK10	YLR320W	MMS22
YEL065W	SIT1	YJL172W	CPS1
YLR095C	IOC2	YLR370C	ARC18

Predictions at threshold level 0.80 continued...			
**Gene1 of predicted pair**		**Gene2 of predicted pair**	
**Systematic Name**	**Standard Name**	**Systematic Name**	**Standard Name**
YLR083C	EMP70	YMR216C	SKY1
YPL037C	EGD1	YHR039C-A	VMA10
YMR016C	SOK2	YDR310C	SUM1
YOR043W	WHI2	YDL083C	RPS16B
YBR173C	UMP1	YMR133W	REC114
YMR055C	BUB2	YOL081W	IRA2
YHR200W	RPN10	YOR043W	WHI2
YDR320C	SWA2	YOR109W	INP53
YMR198W	CIK1	YOL091W	SPO21
YIL053W	RHR2	YMR224C	MRE11
YHR081W	LRP1	YDR310C	SUM1
YHR156C	LIN1	YBR217W	ATG12
YEL056W	HAT2	YDR310C	SUM1
YKR020W	VPS51	YOL108C	INO4
YDL223C	HBT1	YGR135W	PRE9
YKR057W	RPS21A	YDR264C	AKR1
YFL023W	BUD27	YLR370C	ARC18
YKR001C	VPS1	YKL080W	VMA5
YFR031C-A	RPL2A	YJR060W	CBF1
YOR132W	VPS17	YDR080W	VPS41
YDR108W	GSG1	YMR054W	STV1
YPL022W	RAD1	YNL236W	SIN4
YFR010W	UBP6	YDR137W	RGP1
YNL201C	PSY2	YGL252C	RTG2
YBR215W	HPC2	YJR060W	CBF1
YLR216C	CPR6	YHR200W	RPN10
YML001W	YPT7	YDR358W	GGA1
YGL087C	MMS2	YMR055C	BUB2
YJL176C	SWI3	YJL006C	CTK2

Predictions at threshold level 0.80 continued...			
Gene1 of predicted pair		Gene2 of predicted pair	
Systematic Name	Standard Name	Systematic Name	Standard Name
YNL201C	PSY2	YAL024C	LTE1
YMR068W	AVO2	YIL105C	SLM1
YPR171W	BSP1	YDR310C	SUM1
YFR040W	SAP155	YOR035C	SHE4
YLL007C	YLL007C	YPL253C	VIK1
YOR290C	SNF2	YNL076W	MKS1
YGR252W	GCN5	YPR120C	CLB5
YJL036W	SNX4	YNL041C	COG6
YPR017C	DSS4	YPL161C	BEM4
YGL147C	RPL9A	YOR290C	SNF2
YKR020W	VPS51	YFR036W	CDC26
YHR178W	STB5	YJR032W	CPR7
YAL024C	LTE1	YCR053W	THR4
YLR234W	TOP3	YHR114W	BZZ1
YBL051C	PIN4	YPR051W	MAK3
YPL057C	SUR1	YGL115W	SNF4
YMR016C	SOK2	YER139C	YER139C
YDR507C	GIN4	YPL161C	BEM4
YPR018W	RLF2	YNL307C	MCK1
YLR119W	SRN2	YCR053W	THR4
YEL065W	SIT1	YGL147C	RPL9A
YHR039C-A	VMA10	YDR358W	GGA1
YIL103W	DPH1	YPL018W	CTF19
YNL215W	IES2	YCR077C	PAT1
YDR320C	SWA2	YEL053C	MAK10
YDL047W	SIT4	YMR038C	CCS1
YMR204C	INP1	YBR168W	PEX32
YOR106W	VAM3	YPL195W	APL5
YKR020W	VPS51	YEL036C	ANP1

Predictions at threshold level 0.80 continued...			
Gene1 of predicted pair		Gene2 of predicted pair	
Systematic Name	Standard Name	Systematic Name	Standard Name
YLR234W	TOP3	YBR108W	YBR108W
YKL006W	RPL14A	YKR020W	VPS51
YNL218W	MGS1	YJL176C	SWI3
YNL087W	TCB2	YGL013C	PDR1
YDL116W	NUP84	YMR179W	SPT21
YIL036W	CST6	YLR381W	CTF3
YML112W	CTK3	YLR182W	SWI6
YOR290C	SNF2	YLR399C	BDF1
YCR024C-A	PMP1	YPL178W	CBC2
YNL201C	PSY2	YIR005W	IST3
YBL021C	HAP3	YHR012W	VPS29
YMR153W	NUP53	YJR056C	YJR056C
YLR150W	STM1	YLR032W	RAD5
YNR072W	HXT17	YDR207C	UME6
YHR012W	VPS29	YNL238W	KEX2
YNL238W	KEX2	YLR093C	NYV1
YDR293C	SSD1	YDR289C	RTT103
YEL009C	GCN4	YGL025C	PGD1
YJR056C	YJR056C	YGL115W	SNF4
YGL212W	VAM7	YGL252C	RTG2
YLR452C	SST2	YMR052W	FAR3
YCR097W	HMRA1	YCL067C	HMLALPHA2
YBR200W	BEM1	YJR005W	APL1
YHR200W	RPN10	YLR357W	RSC2
YLR148W	PEP3	YOR106W	VAM3
YOR195W	SLK19	YPL057C	SUR1
YDR146C	SWI5	YOL081W	IRA2
YER116C	SLX8	YNL076W	MKS1
YMR199W	CLN1	YJL157C	FAR1

Predictions at threshold level 0.80 continued...			
Gene1 of predicted pair		Gene2 of predicted pair	
Systematic Name	Standard Name	Systematic Name	Standard Name
YBR108W	YBR108W	YNR072W	HXT17
YDL184C	RPL41A	YMR016C	SOK2
YBR107C	IML3	YGR270W	YTA7
YKR014C	YPT52	YML071C	COG8
YOR033C	EXO1	YLR135W	SLX4
YDL116W	NUP84	YKL110C	KTI12
YMR199W	CLN1	YDL047W	SIT4
YOR212W	STE4	YJR086W	STE18
YGR252W	GCN5	YDR227W	SIR4
YCR077C	PAT1	YOR368W	RAD17
YGL087C	MMS2	YER110C	KAP123
YDR383C	NKP1	YGL163C	RAD54
YPL240C	HSP82	YNL086W	YNL086W
YBR030W	YBR030W	YPL057C	SUR1
YDL155W	CLB3	YLR425W	TUS1
YBL058W	SHP1	YGL115W	SNF4
YMR153W	NUP53	YER162C	RAD4
YML115C	VAN1	YDR440W	DOT1
YKR101W	SIR1	YFR036W	CDC26
YML038C	YMD8	YAR014C	BUD14
YGL147C	RPL9A	YMR311C	GLC8
YGL066W	SGF73	YHR108W	GGA2
YLR398C	SKI2	YDR207C	UME6
YLR006C	SSK1	YNL293W	MSB3
YNL087W	TCB2	YNL307C	MCK1
YCR034W	FEN1	YBL008W	HIR1
YBR239C	YBR239C	YHR016C	YSC84
YPL256C	CLN2	YHR158C	KEL1
YLR079W	SIC1	YHR152W	SPO12

Predictions at threshold level 0.80 continued...			
Gene1 of predicted pair		Gene2 of predicted pair	
Systematic Name	Standard Name	Systematic Name	Standard Name
YDR156W	RPA14	YLR386W	VAC14
YBL103C	RTG3	YLR371W	ROM2
YNL079C	TPM1	YGR135W	PRE9
YGL078C	DBP3	YBR280C	SAF1
YPL129W	TAF14	YIR009W	MSL1
YLR090W	XDJ1	YGL115W	SNF4
YGL135W	RPL1B	YDR137W	RGP1
YDR137W	RGP1	YLR180W	SAM1
YNL248C	RPA49	YNL307C	MCK1
YLR337C	VRP1	YDR289C	RTT103
YNL289W	PCL1	YKR048C	NAP1
YDR004W	RAD57	YLR288C	MEC3
YNL293W	MSB3	YNR049C	MSO1
YIL036W	CST6	YPR120C	CLB5
YMR199W	CLN1	YOR106W	VAM3
YJR032W	CPR7	YGL003C	CDH1
YGL174W	BUD13	YJL124C	LSM1
YKR048C	NAP1	YGL124C	MON1
YIL076W	SEC28	YGR270W	YTA7
YHR134W	WSS1	YNL107W	YAF9
YPL019C	VTC3	YER072W	VTC1
YFR010W	UBP6	YOR368W	RAD17
YKR057W	RPS21A	YIL008W	URM1
YIL076W	SEC28	YDR335W	MSN5
YDL020C	RPN4	YMR199W	CLN1
YDR202C	RAV2	YKR030W	GMH1
YJL004C	SYS1	YDR207C	UME6
YGL212W	VAM7	YLR094C	GIS3
YLR119W	SRN2	YMR016C	SOK2

Predictions at threshold level 0.80 continued...			
Gene1 of predicted pair		Gene2 of predicted pair	
Systematic Name	Standard Name	Systematic Name	Standard Name
YDL116W	NUP84	YDR378C	LSM6
YKL025C	PAN3	YML058W	SML1
YMR016C	SOK2	YPR030W	CSR2
YPR131C	NAT3	YDL226C	GCS1
YKL116C	PRR1	YMR153W	NUP53
YBR057C	MUM2	YPL050C	MNN9
YAL024C	LTE1	YDR293C	SSD1
YOR270C	VPH1	YGL115W	SNF4
YKL116C	PRR1	YOR043W	WHI2
YBR009C	HHF1	YBR010W	HHT1
YMR016C	SOK2	YNL041C	COG6
YKL048C	ELM1	YLR320W	MMS22
YDR108W	GSG1	YHL020C	OPI1
YFR036W	CDC26	YDR310C	SUM1
YPL180W	TCO89	YGL200C	EMP24
YBR057C	MUM2	YNL307C	MCK1
YDL184C	RPL41A	YEL053C	MAK10
YGR133W	PEX4	YJL095W	BCK1
YOR290C	SNF2	YLR441C	RPS1A
YER162C	RAD4	YLR370C	ARC18
YAL010C	MDM10	YOL081W	IRA2
YBL051C	PIN4	YBL021C	HAP3
YJL176C	SWI3	YKL109W	HAP4
YAL020C	ATS1	YJL187C	SWE1
YHL031C	GOS1	YER161C	SPT2
YDR176W	NGG1	YOL051W	GAL11
YDR174W	HMO1	YDR183W	PLP1
YCR020C-A	MAK31	YDR108W	GSG1
YGL087C	MMS2	YGL163C	RAD54

Predictions at threshold level 0.80 continued...			
**Gene1 of predicted pair**		**Gene2 of predicted pair**	
**Systematic Name**	**Standard Name**	**Systematic Name**	**Standard Name**
YGR249W	MGA1	YPR046W	MCM16
YER044C	ERG28	YDR358W	GGA1
YKL139W	CTK1	YJL124C	LSM1
YNL288W	CAF40	YDL155W	CLB3
YDR004W	RAD57	YPL018W	CTF19
YGL190C	CDC55	YER027C	GAL83
YDR443C	SSN2	YBR260C	RGD1
YOR195W	SLK19	YPL106C	SSE1
YLR148W	PEP3	YBR131W	CCZ1
YGL066W	SGF73	YDL155W	CLB3
YFR010W	UBP6	YEL013W	VAC8
YHR200W	RPN10	YML001W	YPT7
YMR004W	MVP1	YML001W	YPT7
YKL139W	CTK1	YJL006C	CTK2
YDR245W	MNN10	YPL256C	CLN2
YGR100W	MDR1	YPR028W	YOP1
YNL079C	TPM1	YOL081W	IRA2
YMR243C	ZRC1	YPL058C	PDR12
YPR084W	YPR084W	YAL002W	VPS8
YGR133W	PEX4	YOR035C	SHE4
YPR051W	MAK3	YGL253W	HXK2
YJL006C	CTK2	YOL081W	IRA2
YJR032W	CPR7	YKL101W	HSL1
YKR035W-A	DID2	YLR094C	GIS3
YDR080W	VPS41	YAR014C	BUD14
YOR132W	VPS17	YCR086W	CSM1
YDR363W-A	SEM1	YNL107W	YAF9
YGR072W	UPF3	YNL291C	MID1
YDL184C	RPL41A	YMR230W	RPS10B

Predictions at threshold level 0.80 continued...			
Gene1 of predicted pair		Gene2 of predicted pair	
Systematic Name	Standard Name	Systematic Name	Standard Name
YDR379W	RGA2	YNL229C	URE2
YJR024C	MDE1	YKL068W	NUP100
YHR013C	ARD1	YBR082C	UBC4
YJR032W	CPR7	YER139C	YER139C
YHR156C	LIN1	YJR056C	YJR056C
YDR146C	SWI5	YGR089W	NNF2
YDR202C	RAV2	YNL051W	COG5
YPL240C	HSP82	YGR294W	PAU12
YNL135C	FPR1	YHR012W	VPS29
YIR005W	IST3	YHR167W	THP2
YDL184C	RPL41A	YER139C	YER139C
YGR249W	MGA1	YMR142C	RPL13B
YCL037C	SRO9	YHR167W	THP2
YNL289W	PCL1	YJL095W	BCK1
YNL025C	SSN8	YDR440W	DOT1
YFL013C	IES1	YKL139W	CTK1
YDR439W	LRS4	YDR335W	MSN5
YJL204C	RCY1	YAL030W	SNC1
YAL024C	LTE1	YHR115C	DMA1
YPL161C	BEM4	YCR077C	PAT1
YGL115W	SNF4	YNL041C	COG6
YPL024W	RMI1	YMR224C	MRE11
YMR198W	CIK1	YJR135C	MCM22
YBL071W-A	KTI11	YHR187W	IKI1
YDR440W	DOT1	YOR141C	ARP8
YEL044W	IES6	YBL047C	EDE1
YHL031C	GOS1	YJL124C	LSM1
YDR174W	HMO1	YGL252C	RTG2
YHR060W	VMA22	YGR105W	VMA21

Predictions at threshold level 0.80 continued...			
Gene1 of predicted pair		Gene2 of predicted pair	
Systematic Name	Standard Name	Systematic Name	Standard Name
YNL068C	FKH2	YPL161C	BEM4
YFR010W	UBP6	YKR099W	BAS1
YFR031C-A	RPL2A	YOR066W	MSA1
YBR200W	BEM1	YPL256C	CLN2
YMR153W	NUP53	YDR207C	UME6
YMR199W	CLN1	YPL178W	CBC2
YBR200W	BEM1	YFL023W	BUD27
YPL037C	EGD1	YER040W	GLN3
YLR150W	STM1	YKL101W	HSL1
YBR173C	UMP1	YOR327C	SNC2
YBR065C	ECM2	YPL213W	LEA1
YAL024C	LTE1	YMR133W	REC114
YBR107C	IML3	YDR144C	MKC7
YLR090W	XDJ1	YNR072W	HXT17
YBR289W	SNF5	YNL030W	HHF2
YDL065C	PEX19	YGR209C	TRX2
YJL117W	PHO86	YDL006W	PTC1
YMR224C	MRE11	YOL081W	IRA2
YNL127W	FAR11	YAR014C	BUD14
YDL083C	RPS16B	YPR046W	MCM16
YNL054W	VAC7	YIL103W	DPH1
YJL154C	VPS35	YMR127C	SAS2
YOR358W	HAP5	YBR114W	RAD16
YKR020W	VPS51	YLR396C	VPS33
YGL033W	HOP2	YKR035W-A	DID2
YPL024W	RMI1	YMR016C	SOK2
YPL178W	CBC2	YBL079W	NUP170
YDR293C	SSD1	YLR182W	SWI6
YML058W	SML1	YLR234W	TOP3

Predictions at threshold level 0.80 continued...			
Gene1 of predicted pair		Gene2 of predicted pair	
Systematic Name	Standard Name	Systematic Name	Standard Name
YLL007C	YLL007C	YDR144C	MKC7
YMR224C	MRE11	YOR270C	VPH1
YGL078C	DBP3	YKL116C	PRR1
YPR046W	MCM16	YDL192W	ARF1
YLR079W	SIC1	YML095C	RAD10
YER096W	SHC1	YGL031C	RPL24A
YML117W	NAB6	YBR200W	BEM1
YJL095W	BCK1	YOL067C	RTG1
YLR006C	SSK1	YMR052W	FAR3
YER144C	UBP5	YOR066W	MSA1
YDR477W	SNF1	YLR093C	NYV1
YFR010W	UBP6	YMR230W	RPS10B
YMR224C	MRE11	YDR137W	RGP1
YNL248C	RPA49	YGL252C	RTG2
YDL160C	DHH1	YMR224C	MRE11
YMR224C	MRE11	YPL161C	BEM4
YOL067C	RTG1	YHR114W	BZZ1
YDR195W	REF2	YOR179C	SYC1
YNR023W	SNF12	YPL240C	HSP82
YER139C	YER139C	YDR310C	SUM1
YIL148W	RPL40A	YGR059W	SPR3
YNL297C	MON2	YJR084W	CSN12
YKL139W	CTK1	YAR014C	BUD14
YDR129C	SAC6	YPL161C	BEM4
YBL058W	SHP1	YNL076W	MKS1
YIL008W	URM1	YBL087C	RPL23A
YMR179W	SPT21	YDR254W	CHL4
YLR234W	TOP3	YLR032W	RAD5
YNL032W	SIW14	YML071C	COG8

Predictions at threshold level 0.80 continued...			
**Gene1 of predicted pair**		**Gene2 of predicted pair**	
**Systematic Name**	**Standard Name**	**Systematic Name**	**Standard Name**
YOL090W	MSH2	YGR258C	RAD2
YOR106W	VAM3	YMR216C	SKY1
YDL020C	RPN4	YGR108W	CLB1
YDR156W	RPA14	YFR032C-A	RPL29
YHR200W	RPN10	YGR232W	NAS6
YKL010C	UFD4	YJL176C	SWI3
YOR112W	CEX1	YAL002W	VPS8
YHL031C	GOS1	YNL199C	GCR2
YPL115C	BEM3	YHR111W	UBA4
YHR111W	UBA4	YPR120C	CLB5
YOR308C	SNU66	YNL107W	YAF9
YBR274W	CHK1	YOR043W	WHI2
YMR139W	RIM11	YMR042W	ARG80
YOR358W	HAP5	YGL016W	KAP122
YPL213W	LEA1	YNL079C	TPM1
YJL036W	SNX4	YGR105W	VMA21
YER139C	YER139C	YDR207C	UME6
YMR284W	YKU70	YDR227W	SIR4
YLR381W	CTF3	YPL161C	BEM4
YOL148C	SPT20	YDR216W	ADR1
YPR120C	CLB5	YNL225C	CNM67
YNR023W	SNF12	YGL025C	PGD1
YDR174W	HMO1	YML016C	PPZ1
YDR146C	SWI5	YMR055C	BUB2
YPR017C	DSS4	YCR066W	RAD18
YKR099W	BAS1	YGL115W	SNF4
YNL069C	RPL16B	YHL020C	OPI1
YEL065W	SIT1	YFR010W	UBP6
YGR129W	SYF2	YPR101W	SNT309

Predictions at threshold level 0.80 continued...			
Gene1 of predicted pair		Gene2 of predicted pair	
Systematic Name	Standard Name	Systematic Name	Standard Name
YPL057C	SUR1	YER044C	ERG28
YAL024C	LTE1	YOL081W	IRA2
YJL183W	MNN11	YEL060C	PRB1
YDR261C	EXG2	YDL077C	VAM6
YMR039C	SUB1	YDR310C	SUM1
YOR112W	CEX1	YNL238W	KEX2
YDL020C	RPN4	YEL053C	MAK10
YKL126W	YPK1	YOL081W	IRA2
YKR099W	BAS1	YEL053C	MAK10
YDR108W	GSG1	YDR137W	RGP1
YDL161W	ENT1	YMR231W	PEP5
YGR249W	MGA1	YHR156C	LIN1
YJL058C	BIT61	YJR135C	MCM22
YGR249W	MGA1	YLR370C	ARC18
YLR119W	SRN2	YLR452C	SST2
YLR090W	XDJ1	YNL238W	KEX2
YPR018W	RLF2	YPR120C	CLB5
YFR010W	UBP6	YMR055C	BUB2
YMR199W	CLN1	YER049W	TPA1
YOR069W	VPS5	YHR060W	VMA22
YGL175C	SAE2	YLR352W	YLR352W
YML117W	NAB6	YOR275C	RIM20
YNL218W	MGS1	YHR134W	WSS1
YBL051C	PIN4	YNL116W	DMA2
YDR320C	SWA2	YGL212W	VAM7
YDR176W	NGG1	YNL107W	YAF9
YLR410W	VIP1	YDR289C	RTT103
YJR135C	MCM22	YHL002W	HSE1
YNL201C	PSY2	YFR036W	CDC26

Predictions at threshold level 0.80 continued...			
Gene1 of predicted pair		Gene2 of predicted pair	
Systematic Name	Standard Name	Systematic Name	Standard Name
YGR270W	YTA7	YPR120C	CLB5
YMR092C	AIP1	YDR129C	SAC6
YCL037C	SRO9	YCR066W	RAD18
YDR477W	SNF1	YNR072W	HXT17
YGL025C	PGD1	YLR370C	ARC18
YNL025C	SSN8	YCR081W	SRB8
YDR318W	MCM21	YDR289C	RTT103
YBL058W	SHP1	YLR370C	ARC18
YLR135W	SLX4	YOR080W	DIA2
YJL004C	SYS1	YNL107W	YAF9
YGL133W	ITC1	YDR507C	GIN4
YDR108W	GSG1	YHR060W	VMA22
YIL159W	BNR1	YJL172W	CPS1
YDL013W	SLX5	YCR086W	CSM1
YKR099W	BAS1	YBR280C	SAF1
YJL060W	BNA3	YBR215W	HPC2
YGR054W	YGR054W	YJR090C	GRR1
YOL018C	TLG2	YDR153C	ENT5
YPR084W	YPR084W	YGL212W	VAM7
YBR171W	SEC66	YBL025W	RRN10
YLR234W	TOP3	YNL117W	MLS1
YJL124C	LSM1	YML112W	CTK3
YLL007C	YLL007C	YJR135C	MCM22
YHR060W	VMA22	YBL025W	RRN10
YBR171W	SEC66	YEL036C	ANP1
YGL115W	SNF4	YBR280C	SAF1
YLR292C	SEC72	YEL053C	MAK10
YGL060W	YBP2	YBR107C	IML3
YLR381W	CTF3	YLR032W	RAD5

Predictions at threshold level 0.80 continued...			
Gene1 of predicted pair		Gene2 of predicted pair	
Systematic Name	Standard Name	Systematic Name	Standard Name
YKR014C	YPT52	YOR035C	SHE4
YPL256C	CLN2	YHR115C	DMA1
YLR386W	VAC14	YPL161C	BEM4
YML115C	VAN1	YJR084W	CSN12
YNL016W	PUB1	YJR035W	RAD26
YGL147C	RPL9A	YER116C	SLX8
YKL213C	DOA1	YMR038C	CCS1
YOR132W	VPS17	YNR032W	PPG1
YIR005W	IST3	YFR036W	CDC26
YPR040W	TIP41	YPL152W	RRD2
YNL215W	IES2	YHR039C-A	VMA10
YLR399C	BDF1	YKL110C	KTI12
YMR080C	NAM7	YOR076C	SKI7
YOR127W	RGA1	YOR275C	RIM20
YDL047W	SIT4	YKR030W	GMH1
YHR013C	ARD1	YMR038C	CCS1
YBL079W	NUP170	YML057W	CMP2
YDR383C	NKP1	YLR254C	NDL1
YIL076W	SEC28	YNL307C	MCK1
YDL083C	RPS16B	YDL077C	VAM6
YKR099W	BAS1	YBR036C	CSG2
YGL226C-A	OST5	YEL036C	ANP1
YDR260C	SWM1	YPL152W	RRD2
YDR443C	SSN2	YOR270C	VPH1
YAL024C	LTE1	YMR039C	SUB1
YGL025C	PGD1	YNL079C	TPM1
YLL007C	YLL007C	YGL124C	MON1
YJR024C	MDE1	YGL016W	KAP122
YBR126C	TPS1	YOR035C	SHE4

Predictions at threshold level 0.80 continued...			
Gene1 of predicted pair		Gene2 of predicted pair	
Systematic Name	Standard Name	Systematic Name	Standard Name
YGR104C	SRB5	YDR378C	LSM6
YDL116W	NUP84	YHR013C	ARD1
YLR381W	CTF3	YDR363W-A	SEM1
YGL147C	RPL9A	YOR270C	VPH1
YEL053C	MAK10	YLR370C	ARC18
YKR072C	SIS2	YGL031C	RPL24A
YDR254W	CHL4	YPL152W	RRD2
YLR095C	IOC2	YHR060W	VMA22
YMR172W	HOT1	YCR086W	CSM1
YHR205W	SCH9	YER096W	SHC1
YHR157W	REC104	YMR133W	REC114
YDR174W	HMO1	YOL054W	PSH1
YOR312C	RPL20B	YKL041W	VPS24
YNL264C	PDR17	YJR035W	RAD26
YHR134W	WSS1	YDR254W	CHL4
YMR055C	BUB2	YDL006W	PTC1
YJL154C	VPS35	YKL119C	VPH2
YDR293C	SSD1	YLR360W	VPS38
YNL215W	IES2	YDL002C	NHP10
YOR109W	INP53	YJR058C	APS2
YER110C	KAP123	YKR024C	DBP7
YML016C	PPZ1	YDR200C	VPS64
YPR131C	NAT3	YLR018C	POM34
YDR507C	GIN4	YCL024W	KCC4
YKL048C	ELM1	YGL124C	MON1
YOR337W	TEA1	YNL058C	YNL058C
YOR141C	ARP8	YOL081W	IRA2
YOR368W	RAD17	YGR188C	BUB1
YHR013C	ARD1	YJL006C	CTK2

Predictions at threshold level 0.80 continued...			
Gene1 of predicted pair		Gene2 of predicted pair	
Systematic Name	Standard Name	Systematic Name	Standard Name
YPL022W	RAD1	YDR207C	UME6
YPL046C	ELC1	YGL208W	SIP2
YGR249W	MGA1	YNL041C	COG6
YOR109W	INP53	YER120W	SCS2
YLR192C	HCR1	YBR171W	SEC66
YJL151C	SNA3	YLR144C	ACF2
YER179W	DMC1	YLR219W	MSC3
YPR120C	CLB5	YDR207C	UME6
YLR206W	ENT2	YNR006W	VPS27
YMR311C	GLC8	YEL060C	PRB1
YLR094C	GIS3	YMR311C	GLC8
YBL051C	PIN4	YNL107W	YAF9
YHR178W	STB5	YBR215W	HPC2
YJR040W	GEF1	YPR032W	SRO7
YDL192W	ARF1	YBR280C	SAF1
YBR057C	MUM2	YMR116C	ASC1
YLR240W	VPS34	YGL095C	VPS45
YGL163C	RAD54	YLR370C	ARC18
YPL024W	RMI1	YMR038C	CCS1
YNL069C	RPL16B	YER122C	GLO3
YPR036W	VMA13	YOR066W	MSA1
YKR048C	NAP1	YJL187C	SWE1
YLR093C	NYV1	YEL060C	PRB1
YPR018W	RLF2	YPL256C	CLN2
YDR245W	MNN10	YDR137W	RGP1
YKL116C	PRR1	YDR207C	UME6
YHR178W	STB5	YCR077C	PAT1
YGL147C	RPL9A	YMR214W	SCJ1
YHR108W	GGA2	YDR153C	ENT5

Predictions at threshold level 0.80 continued...			
**Gene1 of predicted pair**		**Gene2 of predicted pair**	
**Systematic Name**	**Standard Name**	**Systematic Name**	**Standard Name**
YJR084W	CSN12	YLR180W	SAM1
YHL006C	SHU1	YDR078C	SHU2
YGL212W	VAM7	YEL036C	ANP1
YMR116C	ASC1	YDR440W	DOT1
YLR292C	SEC72	YGR108W	CLB1
YLR079W	SIC1	YMR125W	STO1
YNL248C	RPA49	YHR013C	ARD1
YLR090W	XDJ1	YLR180W	SAM1
YDR309C	GIC2	YPL256C	CLN2
YGL163C	RAD54	YKL114C	APN1
YMR125W	STO1	YKL068W	NUP100
YPR084W	YPR084W	YEL043W	YEL043W
YDR293C	SSD1	YMR216C	SKY1
YLR234W	TOP3	YPR046W	MCM16
YDR137W	RGP1	YJL095W	BCK1
YBR217W	ATG12	YOR035C	SHE4
YDL080C	THI3	YML097C	VPS9
YLR006C	SSK1	YIL034C	CAP2
YBR278W	DPB3	YDL002C	NHP10
YLR292C	SEC72	YGL237C	HAP2
YGR014W	MSB2	YDL006W	PTC1
YDR004W	RAD57	YBR215W	HPC2
YJL124C	LSM1	YPL152W	RRD2
YFR040W	SAP155	YJR032W	CPR7
YOR124C	UBP2	YLR350W	ORM2
YLR384C	IKI3	YHR187W	IKI1
YML115C	VAN1	YMR133W	REC114
YDR293C	SSD1	YOR069W	VPS5
YDR293C	SSD1	YDR309C	GIC2

Predictions at threshold level 0.80 continued...			
Gene1 of predicted pair		Gene2 of predicted pair	
Systematic Name	Standard Name	Systematic Name	Standard Name
YFR010W	UBP6	YGR135W	PRE9
YKL116C	PRR1	YBR019C	GAL10
YPL253C	VIK1	YPR046W	MCM16
YOR127W	RGA1	YHL031C	GOS1
YBR030W	YBR030W	YLR386W	VAC14
YKR099W	BAS1	YDR310C	SUM1
YGL147C	RPL9A	YDL184C	RPL41A
YOL051W	GAL11	YLR399C	BDF1
YPL152W	RRD2	YIL103W	DPH1
YOR109W	INP53	YOL018C	TLG2
YML115C	VAN1	YCR053W	THR4
YDL154W	MSH5	YGL249W	ZIP2
YFL033C	RIM15	YDR310C	SUM1
YLR079W	SIC1	YER110C	KAP123
YOR195W	SLK19	YKL101W	HSL1
YPL042C	SSN3	YHR115C	DMA1
YGR159C	NSR1	YDR440W	DOT1
YLR360W	VPS38	YMR054W	STV1
YDL223C	HBT1	YJR032W	CPR7
YPL115C	BEM3	YJL183W	MNN11
YNL069C	RPL16B	YDR310C	SUM1
YDR245W	MNN10	YHR012W	VPS29
YDR080W	VPS41	YNL297C	MON2
YIL034C	CAP2	YBR200W	BEM1
YEL065W	SIT1	YER040W	GLN3
YLR360W	VPS38	YBR127C	VMA2
YML095C	RAD10	YDL059C	RAD59
YMR153W	NUP53	YCR053W	THR4
YKR020W	VPS51	YEL043W	YEL043W

Predictions at threshold level 0.80 continued...			
Gene1 of predicted pair		Gene2 of predicted pair	
Systematic Name	Standard Name	Systematic Name	Standard Name
YHR013C	ARD1	YNL107W	YAF9
YBL103C	RTG3	YPR119W	CLB2
YNL025C	SSN8	YCL037C	SRO9
YLR337C	VRP1	YML071C	COG8
YPL106C	SSE1	YLR370C	ARC18
YNL307C	MCK1	YGL100W	SEH1
YKL048C	ELM1	YJL006C	CTK2
YHL031C	GOS1	YDR378C	LSM6
YJL095W	BCK1	YBL072C	RPS8A
YNL291C	MID1	YHL031C	GOS1
YLR102C	APC9	YKL032C	IXR1
YJR056C	YJR056C	YDR310C	SUM1
YMR201C	RAD14	YGR258C	RAD2
YPL024W	RMI1	YJR140C	HIR3
YPL129W	TAF14	YBR111W-A	SUS1
YEL065W	SIT1	YOR327C	SNC2
YLR288C	MEC3	YER032W	FIR1
YMR224C	MRE11	YKL006W	RPL14A
YKL126W	YPK1	YDR358W	GGA1
YDR440W	DOT1	YHL002W	HSE1
YEL043W	YEL043W	YER120W	SCS2
YBR107C	IML3	YLR452C	SST2
YML016C	PPZ1	YDR318W	MCM21
YHR060W	VMA22	YOL081W	IRA2
YOR358W	HAP5	YHR111W	UBA4
YDR129C	SAC6	YKL041W	VPS24
YDR379W	RGA2	YDL047W	SIT4
YMR026C	PEX12	YHR039C-A	VMA10
YLR148W	PEP3	YGL198W	YIP4

Predictions at threshold level 0.80 continued...			
Gene1 of predicted pair		Gene2 of predicted pair	
Systematic Name	Standard Name	Systematic Name	Standard Name
YDR507C	GIN4	YJL136C	RPS21B
YIR005W	IST3	YOR038C	HIR2
YOR132W	VPS17	YDL006W	PTC1
YDR076W	RAD55	YLR352W	YLR352W
YOR127W	RGA1	YBR108W	YBR108W
YIL036W	CST6	YML112W	CTK3
YJR040W	GEF1	YGR105W	VMA21
YOL054W	PSH1	YGL252C	RTG2
YJL176C	SWI3	YOR035C	SHE4
YOR132W	VPS17	YDR289C	RTT103
YLR216C	CPR6	YNL135C	FPR1
YOR033C	EXO1	YPR120C	CLB5
YLR309C	IMH1	YDR153C	ENT5
YDR225W	HTA1	YMR055C	BUB2
YGL190C	CDC55	YPR135W	CTF4
YBR200W	BEM1	YHR060W	VMA22
YOR059C	YOR059C	YMR313C	TGL3
YNL201C	PSY2	YIL103W	DPH1
YCR081W	SRB8	YLR079W	SIC1
YOR290C	SNF2	YMR152W	YIM1
YKR020W	VPS51	YHL020C	OPI1
YMR152W	YIM1	YMR230W	RPS10B
YCR066W	RAD18	YPL234C	TFP3
YPR084W	YPR084W	YOL018C	TLG2
YLR320W	MMS22	YMR230W	RPS10B
YMR133W	REC114	YMR152W	YIM1
YOR270C	VPH1	YNR072W	HXT17
YKR020W	VPS51	YEL053C	MAK10
YMR109W	MYO5	YGR054W	YGR054W

Predictions at threshold level 0.80 continued...			
Gene1 of predicted pair		Gene2 of predicted pair	
Systematic Name	Standard Name	Systematic Name	Standard Name
YOR195W	SLK19	YPL152W	RRD2
YDL184C	RPL41A	YKL126W	YPK1
YNL236W	SIN4	YHR108W	GGA2
YHR134W	WSS1	YJL187C	SWE1
YHR178W	STB5	YGL148W	ARO2
YOR043W	WHI2	YNL041C	COG6
YDR245W	MNN10	YJR035W	RAD26
YOR001W	RRP6	YHL031C	GOS1
YKL139W	CTK1	YLR410W	VIP1
YGL208W	SIP2	YPL256C	CLN2
YOR270C	VPH1	YPR030W	CSR2
YBR107C	IML3	YDL226C	GCS1
YBL008W	HIR1	YGL115W	SNF4
YLR234W	TOP3	YBL079W	NUP170
YNR072W	HXT17	YOL121C	RPS19A
YOR080W	DIA2	YER122C	GLO3
YJL172W	CPS1	YHR081W	LRP1
YFR008W	FAR7	YMR029C	FAR8
YMR039C	SUB1	YDL002C	NHP10
YKR001C	VPS1	YEL053C	MAK10
YBL087C	RPL23A	YGL031C	RPL24A
YNL054W	VAC7	YGL252C	RTG2
YIR005W	IST3	YLR182W	SWI6
YLR083C	EMP70	YNR032W	PPG1
YEL044W	IES6	YDR245W	MNN10
YGL078C	DBP3	YBR171W	SEC66
YDR443C	SSN2	YNL288W	CAF40
YBR084C-A	RPL19A	YHR200W	RPN10
YGR019W	UGA1	YLR360W	VPS38

Predictions at threshold level 0.80 continued...			
Gene1 of predicted pair		Gene2 of predicted pair	
Systematic Name	Standard Name	Systematic Name	Standard Name
YFR031C-A	RPL2A	YOR035C	SHE4
YJL183W	MNN11	YCR053W	THR4
YNL264C	PDR17	YJL183W	MNN11
YNL025C	SSN8	YDR137W	RGP1
YBR107C	IML3	YMR055C	BUB2
YLR410W	VIP1	YJL006C	CTK2
YAL020C	ATS1	YBR200W	BEM1
YJR140C	HIR3	YOR124C	UBP2
YBR019C	GAL10	YGL124C	MON1
YDR245W	MNN10	YJL187C	SWE1
YBR173C	UMP1	YGR184C	UBR1
YNL079C	TPM1	YHR158C	KEL1
YGL178W	MPT5	YMR312W	ELP6
YMR133W	REC114	YBR036C	CSG2
YDL101C	DUN1	YHR134W	WSS1
YKL117W	SBA1	YKR024C	DBP7
YIL053W	RHR2	YDL184C	RPL41A
YDR108W	GSG1	YNL044W	YIP3
YPL161C	BEM4	YNL076W	MKS1
YMR142C	RPL13B	YBR171W	SEC66
YDR358W	GGA1	YER040W	GLN3
YMR179W	SPT21	YKL101W	HSL1
YLR018C	POM34	YOR358W	HAP5
YDL184C	RPL41A	YBR280C	SAF1
YNL243W	SLA2	YPR135W	CTF4
YDR293C	SSD1	YPR051W	MAK3
YOR231W	MKK1	YDL006W	PTC1
YML115C	VAN1	YDR456W	NHX1
YMR038C	CCS1	YDR207C	UME6

Predictions at threshold level 0.80 continued...			
Gene1 of predicted pair		Gene2 of predicted pair	
Systematic Name	Standard Name	Systematic Name	Standard Name
YLR360W	VPS38	YGR020C	VMA7
YPL037C	EGD1	YML001W	YPT7
YOR312C	RPL20B	YJR035W	RAD26
YER044C	ERG28	YOR066W	MSA1
YPL137C	GIP3	YJR032W	CPR7
YBR280C	SAF1	YOL081W	IRA2
YMR198W	CIK1	YLR386W	VAC14
YMR198W	CIK1	YKL032C	IXR1
YOR106W	VAM3	YDL192W	ARF1
YPR017C	DSS4	YOL112W	MSB4
YJL095W	BCK1	YER040W	GLN3
YBR082C	UBC4	YDR207C	UME6
YJL029C	VPS53	YJL164C	TPK1
YEL065W	SIT1	YDR207C	UME6
YJR032W	CPR7	YEL042W	GDA1
YLR447C	VMA6	YOL018C	TLG2
YMR038C	CCS1	YLR032W	RAD5
YPL024W	RMI1	YMR133W	REC114
YHR111W	UBA4	YDR309C	GIC2
YML115C	VAN1	YBL103C	RTG3
YBR107C	IML3	YJR060W	CBF1
YBR127C	VMA2	YGR105W	VMA21
YLR288C	MEC3	YMR038C	CCS1
YPR084W	YPR084W	YDR363W-A	SEM1
YHR111W	UBA4	YKL101W	HSL1
YLL007C	YLL007C	YBR171W	SEC66
YIL008W	URM1	YGR188C	BUB1
YPR046W	MCM16	YGL124C	MON1
YML103C	NUP188	YGL252C	RTG2

Predictions at threshold level 0.80 continued...			
Gene1 of predicted pair		Gene2 of predicted pair	
Systematic Name	Standard Name	Systematic Name	Standard Name
YDR103W	STE5	YLR362W	STE11
YMR192W	GYL1	YPL249C	GYP5
YMR172W	HOT1	YOR023C	AHC1
YGR105W	VMA21	YNL107W	YAF9
YKL126W	YPK1	YML071C	COG8
YFR010W	UBP6	YGL100W	SEH1
YPL018W	CTF19	YBR114W	RAD16
YLR447C	VMA6	YER122C	GLO3
YJL058C	BIT61	YPR046W	MCM16
YDR507C	GIN4	YHR111W	UBA4
YMR116C	ASC1	YJL006C	CTK2
YBR200W	BEM1	YLR447C	VMA6
YFR010W	UBP6	YNL325C	FIG4
YDR507C	GIN4	YCL032W	STE50
YML016C	PPZ1	YPL253C	VIK1
YHR081W	LRP1	YOL081W	IRA2
YEL065W	SIT1	YLR320W	MMS22
YDR477W	SNF1	YOR035C	SHE4
YJR035W	RAD26	YLR182W	SWI6
YGL178W	MPT5	YDR334W	SWR1
YPL057C	SUR1	YER161C	SPT2
YLR095C	IOC2	YJR056C	YJR056C
YDL006W	PTC1	YML097C	VPS9
YDR155C	CPR1	YLR233C	EST1
YDR254W	CHL4	YAL002W	VPS8
YBR010W	HHT1	YOR141C	ARP8
YPL042C	SSN3	YMR202W	ERG2
YFR010W	UBP6	YJR032W	CPR7
YBL058W	SHP1	YGL212W	VAM7

Predictions at threshold level 0.80 continued...			
**Gene1 of predicted pair**		**Gene2 of predicted pair**	
**Systematic Name**	**Standard Name**	**Systematic Name**	**Standard Name**
YGL115W	SNF4	YJR060W	CBF1
YPL024W	RMI1	YBL103C	RTG3
YGR054W	YGR054W	YDR289C	RTT103
YNL238W	KEX2	YBR280C	SAF1
YHR012W	VPS29	YER049W	TPA1
YKL116C	PRR1	YNL238W	KEX2
YMR172W	HOT1	YMR127C	SAS2
YGL190C	CDC55	YKL113C	RAD27
YDR335W	MSN5	YGR270W	YTA7
YAL024C	LTE1	YBR195C	MSI1
YLR386W	VAC14	YDR469W	SDC1
YOR132W	VPS17	YDL192W	ARF1
YHR111W	UBA4	YLR254C	NDL1
YER044C	ERG28	YPL161C	BEM4
YEL050C	RML2	YPL018W	CTF19
YPL057C	SUR1	YMR214W	SCJ1
YML115C	VAN1	YBR036C	CSG2
YLR234W	TOP3	YML115C	VAN1
YER144C	UBP5	YNL307C	MCK1
YJL172W	CPS1	YLR093C	NYV1
YBR194W	SOY1	YJR135C	MCM22
YML109W	ZDS2	YER124C	DSE1
YGL163C	RAD54	YFR036W	CDC26
YKR014C	YPT52	YBR181C	RPS6B
YFR031C-A	RPL2A	YCL037C	SRO9
YMR026C	PEX12	YFR036W	CDC26
YML115C	VAN1	YKR001C	VPS1
YMR095C	SNO1	YGL033W	HOP2
YBL058W	SHP1	YER040W	GLN3

Predictions at threshold level 0.80 continued...			
**Gene1 of predicted pair**		**Gene2 of predicted pair**	
**Systematic Name**	**Standard Name**	**Systematic Name**	**Standard Name**
YGR249W	MGA1	YPL161C	BEM4
YGL051W	MST27	YAR033W	MST28
YOR132W	VPS17	YMR216C	SKY1
YCL037C	SRO9	YOL018C	TLG2
YLR370C	ARC18	YER122C	GLO3
YEL009C	GCN4	YBR010W	HHT1
YHR134W	WSS1	YGL237C	HAP2
YHR200W	RPN10	YKR030W	GMH1
YNL032W	SIW14	YPL161C	BEM4
YML109W	ZDS2	YCR077C	PAT1
YNL058C	YNL058C	YJL183W	MNN11
YFR032C-A	RPL29	YMR205C	PFK2
YGR252W	GCN5	YDR216W	ADR1
YBR215W	HPC2	YGL175C	SAE2
YGR241C	YAP1802	YNL238W	KEX2
YLR292C	SEC72	YLR233C	EST1
YPL129W	TAF14	YGR275W	RTT102
YDR477W	SNF1	YNL076W	MKS1
YJL117W	PHO86	YJL183W	MNN11
YDL083C	RPS16B	YER040W	GLN3
YDR379W	RGA2	YNL116W	DMA2
YGL212W	VAM7	YDL077C	VAM6
YNL139C	RLR1	YER063W	THO1
YHR060W	VMA22	YGL054C	ERV14
YOR033C	EXO1	YMR055C	BUB2
YGL003C	CDH1	YBR019C	GAL10
YMR152W	YIM1	YBR280C	SAF1
YDR439W	LRS4	YGL252C	RTG2
YBR084C-A	RPL19A	YPL256C	CLN2

Predictions at threshold level 0.80 continued...			
Gene1 of predicted pair		Gene2 of predicted pair	
Systematic Name	Standard Name	Systematic Name	Standard Name
YNL106C	INP52	YGL212W	VAM7
YCL001W	RER1	YNL127W	FAR11
YHR039C-A	VMA10	YNL041C	COG6
YFL023W	BUD27	YKR035W-A	DID2
YML115C	VAN1	YNL307C	MCK1
YMR199W	CLN1	YKL101W	HSL1
YER001W	MNN1	YML071C	COG8
YGR249W	MGA1	YLR398C	SKI2
YFL033C	RIM15	YHL020C	OPI1
YML109W	ZDS2	YOR043W	WHI2
YPR084W	YPR084W	YAR014C	BUD14
YBL008W	HIR1	YJL124C	LSM1
YNR072W	HXT17	YNL076W	MKS1
YMR198W	CIK1	YLR032W	RAD5
YOR290C	SNF2	YPR046W	MCM16
YDL184C	RPL41A	YER144C	UBP5
YHR013C	ARD1	YLR032W	RAD5
YJR058C	APS2	YOR327C	SNC2
YMR230W	RPS10B	YOL081W	IRA2
YGR108W	CLB1	YIL008W	URM1
YFR040W	SAP155	YPL234C	TFP3
YHL031C	GOS1	YMR054W	STV1
YGR019W	UGA1	YGR054W	YGR054W
YNL206C	RTT106	YHR115C	DMA1
YBR107C	IML3	YJR090C	GRR1
YPR051W	MAK3	YJR090C	GRR1
YDR103W	STE5	YDL159W	STE7
YLR182W	SWI6	YNL225C	CNM67
YNR052C	POP2	YER116C	SLX8

Predictions at threshold level 0.80 continued...			
Gene1 of predicted pair		Gene2 of predicted pair	
Systematic Name	Standard Name	Systematic Name	Standard Name
YHR039C-A	VMA10	YER122C	GLO3
YLR090W	XDJ1	YBR200W	BEM1
YOL090W	MSH2	YJR035W	RAD26
YIL159W	BNR1	YML115C	VAN1
YOR069W	VPS5	YPL120W	VPS30
YAL014C	SYN8	YHL031C	GOS1
YKL048C	ELM1	YHR200W	RPN10
YEL053C	MAK10	YJR135C	MCM22
YGL212W	VAM7	YMR133W	REC114
YJL060W	BNA3	YPR046W	MCM16
YMR039C	SUB1	YCR077C	PAT1
YBR171W	SEC66	YHL002W	HSE1
YKL048C	ELM1	YJL190C	RPS22A
YKL032C	IXR1	YBL101C	ECM21
YMR224C	MRE11	YBR107C	IML3
YML112W	CTK3	YNL225C	CNM67
YKR057W	RPS21A	YNL116W	DMA2
YKR061W	KTR2	YLR399C	BDF1
YBR073W	RDH54	YBR107C	IML3
YPL019C	VTC3	YOR270C	VPH1
YOR066W	MSA1	YOR327C	SNC2
YBR200W	BEM1	YBL025W	RRN10
YDR004W	RAD57	YEL056W	HAT2
YJR010C-A	SPC1	YDL006W	PTC1
YLR083C	EMP70	YOL108C	INO4
YHL031C	GOS1	YOL108C	INO4
YBR133C	HSL7	YKL101W	HSL1
YGR108W	CLB1	YGL003C	CDH1
YCL037C	SRO9	YKL160W	ELF1

Predictions at threshold level 0.80 continued...			
Gene1 of predicted pair		Gene2 of predicted pair	
Systematic Name	Standard Name	Systematic Name	Standard Name
YIR005W	IST3	YNL225C	CNM67
YNL106C	INP52	YPL152W	RRD2
YJL176C	SWI3	YHL025W	SNF6
YOR035C	SHE4	YDL192W	ARF1
YMR055C	BUB2	YDR254W	CHL4
YDR108W	GSG1	YDR310C	SUM1
YPL240C	HSP82	YJR136C	YJR136C
YDR443C	SSN2	YNL236W	SIN4
YGR184C	UBR1	YNL230C	ELA1
YKL213C	DOA1	YAL002W	VPS8
YDR378C	LSM6	YBL101C	ECM21
YDR477W	SNF1	YDR310C	SUM1
YMR004W	MVP1	YPL120W	VPS30
YNL094W	APP1	YOR284W	HUA2
YPL158C	YPL158C	YOL081W	IRA2
YDR192C	NUP42	YHR052W	CIC1
YGR072W	UPF3	YHR039C-A	VMA10
YLR386W	VAC14	YHR152W	SPO12
YLR315W	NKP2	YAR014C	BUD14
YIL008W	URM1	YHR114W	BZZ1
YNL041C	COG6	YNL051W	COG5
YOR327C	SNC2	YDR153C	ENT5
YHR060W	VMA22	YDR261C	EXG2
YKL025C	PAN3	YGL087C	MMS2
YGR268C	HUA1	YLR144C	ACF2
YIL053W	RHR2	YER040W	GLN3
YPR036W	VMA13	YBR019C	GAL10
YER116C	SLX8	YGL212W	VAM7
YER116C	SLX8	YDL083C	RPS16B

Predictions at threshold level 0.80 continued...			
Gene1 of predicted pair		Gene2 of predicted pair	
Systematic Name	Standard Name	Systematic Name	Standard Name
YPL161C	BEM4	YJR033C	RAV1
YDR484W	VPS52	YGL003C	CDH1
YKL126W	YPK1	YPL137C	GIP3
YHR152W	SPO12	YKR048C	NAP1
YCR002C	CDC10	YNL078W	NIS1
YFL013C	IES1	YDR363W-A	SEM1
YDR358W	GGA1	YER139C	YER139C
YPL137C	GIP3	YJL095W	BCK1
YIL076W	SEC28	YJL095W	BCK1
YDR293C	SSD1	YPR072W	NOT5
YNL206C	RTT106	YML028W	TSA1
YNL288W	CAF40	YLR381W	CTF3
YCL067C	HMLALPHA2	YMR042W	ARG80
YJR060W	CBF1	YHR039C-A	VMA10
YFR030W	MET10	YAR014C	BUD14
YHR134W	WSS1	YBR171W	SEC66
YGL115W	SNF4	YEL043W	YEL043W
YNL032W	SIW14	YBR171W	SEC66
YDR484W	VPS52	YEL060C	PRB1
YOR076C	SKI7	YDR083W	RRP8
YNL229C	URE2	YBL087C	RPL23A
YHR060W	VMA22	YDL077C	VAM6
YIL076W	SEC28	YKL160W	ELF1
YDR477W	SNF1	YFR036W	CDC26
YOL030W	GAS5	YNL016W	PUB1
YHR200W	RPN10	YML028W	TSA1
YNL288W	CAF40	YEL056W	HAT2
YGL212W	VAM7	YOR036W	PEP12
YIL036W	CST6	YGR188C	BUB1

Predictions at threshold level 0.80 continued...			
Gene1 of predicted pair		Gene2 of predicted pair	
Systematic Name	Standard Name	Systematic Name	Standard Name
YDR156W	RPA14	YML062C	MFT1
YKL139W	CTK1	YDR183W	PLP1
YJL058C	BIT61	YPL018W	CTF19
YDR225W	HTA1	YBR133C	HSL7
YJR040W	GEF1	YOL018C	TLG2
YPR131C	NAT3	YMR231W	PEP5
YBR200W	BEM1	YNL289W	PCL1
YLR192C	HCR1	YER116C	SLX8
YJR056C	YJR056C	YOR035C	SHE4
YDL184C	RPL41A	YPL161C	BEM4
YML115C	VAN1	YHR205W	SCH9
YBR111W-A	SUS1	YNL031C	HHT2
YOR270C	VPH1	YER072W	VTC1
YIL076W	SEC28	YNL325C	FIG4
YFR021W	ATG18	YER162C	RAD4
YDR293C	SSD1	YJR090C	GRR1
YNL087W	TCB2	YKR099W	BAS1
YGR105W	VMA21	YNR032W	PPG1
YOL111C	MDY2	YEL060C	PRB1
YDR443C	SSN2	YDR137W	RGP1
YDR078C	SHU2	YLR376C	PSY3
YGL212W	VAM7	YAR014C	BUD14
YHR156C	LIN1	YDL083C	RPS16B
YDR477W	SNF1	YMR142C	RPL13B
YLR119W	SRN2	YDL192W	ARF1
YDR443C	SSN2	YGR104C	SRB5
YPL129W	TAF14	YDR146C	SWI5
YFR010W	UBP6	YOR080W	DIA2
YDL020C	RPN4	YLR233C	EST1

Predictions at threshold level 0.80 continued...			
Gene1 of predicted pair		Gene2 of predicted pair	
Systematic Name	Standard Name	Systematic Name	Standard Name
YOR270C	VPH1	YDR207C	UME6
YOR127W	RGA1	YGR270W	YTA7
YPR036W	VMA13	YKL101W	HSL1
YMR039C	SUB1	YJL006C	CTK2
YDL002C	NHP10	YML097C	VPS9
YJL172W	CPS1	YOL121C	RPS19A
YBL103C	RTG3	YKL048C	ELM1
YLR396C	VPS33	YOR036W	PEP12
YOR069W	VPS5	YLR447C	VMA6
YDR477W	SNF1	YPL253C	VIK1
YGL115W	SNF4	YOR066W	MSA1
YDR310C	SUM1	YOL081W	IRA2
YDL116W	NUP84	YNL054W	VAC7
YOR112W	CEX1	YGL212W	VAM7
YLR410W	VIP1	YJL140W	RPB4
YJR059W	PTK2	YNL199C	GCR2
YBL047C	EDE1	YGL163C	RAD54
YNL106C	INP52	YDL226C	GCS1
YDL020C	RPN4	YGR072W	UPF3
YLL039C	UBI4	YOR035C	SHE4
YDL013W	SLX5	YFR036W	CDC26
YGR270W	YTA7	YEL053C	MAK10
YNL215W	IES2	YKL048C	ELM1
YDL020C	RPN4	YGR188C	BUB1
YLR095C	IOC2	YDL184C	RPL41A
YHR098C	SFB3	YML041C	VPS71
YKR035W-A	DID2	YCR053W	THR4
YPR017C	DSS4	YDR080W	VPS41
YKL110C	KTI12	YGR188C	BUB1

Predictions at threshold level 0.80 continued...			
Gene1 of predicted pair		Gene2 of predicted pair	
Systematic Name	Standard Name	Systematic Name	Standard Name
YDR484W	VPS52	YNL041C	COG6
YBR017C	KAP104	YKL068W	NUP100
YBR200W	BEM1	YDR507C	GIN4
YLR234W	TOP3	YGL003C	CDH1
YGR249W	MGA1	YOR270C	VPH1
YER144C	UBP5	YDL083C	RPS16B
YOL112W	MSB4	YNR049C	MSO1
YPL129W	TAF14	YOL148C	SPT20
YHR206W	SKN7	YER118C	SHO1
YDR146C	SWI5	YPL161C	BEM4
YDR225W	HTA1	YJR135C	MCM22
YBL058W	SHP1	YOR043W	WHI2
YPR046W	MCM16	YNL225C	CNM67
YHL020C	OPI1	YHR012W	VPS29
YLR090W	XDJ1	YIL159W	BNR1
YDR076W	RAD55	YLR370C	ARC18
YPR017C	DSS4	YDR310C	SUM1
YGL223C	COG1	YJR035W	RAD26
YER075C	PTP3	YOR231W	MKK1
YLR216C	CPR6	YNL069C	RPL16B
YPL115C	BEM3	YGR019W	UGA1
YDR225W	HTA1	YER110C	KAP123
YPR084W	YPR084W	YER161C	SPT2
YLR119W	SRN2	YBR019C	GAL10
YGR014W	MSB2	YPL180W	TCO89
YMR004W	MVP1	YOR106W	VAM3
YBR200W	BEM1	YLR371W	ROM2
YML115C	VAN1	YBR082C	UBC4
YKL116C	PRR1	YBR280C	SAF1

Predictions at threshold level 0.80 continued...			
Gene1 of predicted pair		Gene2 of predicted pair	
Systematic Name	Standard Name	Systematic Name	Standard Name
YDR202C	RAV2	YOL108C	INO4
YER116C	SLX8	YBR171W	SEC66
YPL024W	RMI1	YBL025W	RRN10
YPL152W	RRD2	YNL116W	DMA2
YIL076W	SEC28	YLR182W	SWI6
YDR254W	CHL4	YJR032W	CPR7
YBR030W	YBR030W	YPL195W	APL5
YLR386W	VAC14	YBR258C	SHG1
YLR362W	STE11	YBL075C	SSA3
YDR156W	RPA14	YGR188C	BUB1
YEL043W	YEL043W	YDL006W	PTC1
YJR040W	GEF1	YKR001C	VPS1
YBR108W	YBR108W	YNL107W	YAF9
YOR083W	WHI5	YPL022W	RAD1
YMR054W	STV1	YER122C	GLO3
YJL006C	CTK2	YDR289C	RTT103
YBR084C-A	RPL19A	YBL087C	RPL23A
YGL147C	RPL9A	YNL041C	COG6
YHR200W	RPN10	YHR039C-A	VMA10
YGL237C	HAP2	YMR109W	MYO5
YOR270C	VPH1	YPR119W	CLB2
YDR171W	HSP42	YPR046W	MCM16
YGL147C	RPL9A	YDR207C	UME6
YLR079W	SIC1	YDR254W	CHL4
YEL009C	GCN4	YPR072W	NOT5
YML001W	YPT7	YML071C	COG8
YLR095C	IOC2	YOL121C	RPS19A
YNL106C	INP52	YDR080W	VPS41
YGL043W	DST1	YGL178W	MPT5

Predictions at threshold level 0.80 continued...			
Gene1 of predicted pair		Gene2 of predicted pair	
Systematic Name	Standard Name	Systematic Name	Standard Name
YOR023C	AHC1	YDR144C	MKC7
YGR067C	YGR067C	YPL240C	HSP82
YMR172W	HOT1	YPR046W	MCM16
YHR108W	GGA2	YOR124C	UBP2
YDL227C	HO	YML088W	UFO1
YJL058C	BIT61	YDR200C	VPS64
YPL234C	TFP3	YKL119C	VPH2
YCR081W	SRB8	YKL139W	CTK1
YAL014C	SYN8	YOR106W	VAM3
YGL006W	PMC1	YLR093C	NYV1
YOR327C	SNC2	YLR309C	IMH1
YDR289C	RTT103	YNL225C	CNM67
YOR327C	SNC2	YNL127W	FAR11
YBR278W	DPB3	YJL124C	LSM1
YNL329C	PEX6	YMR089C	YTA12
YMR311C	GLC8	YLR398C	SKI2
YGL078C	DBP3	YFL023W	BUD27
YPR046W	MCM16	YLR180W	SAM1
YDR443C	SSN2	YEL053C	MAK10
YLR240W	VPS34	YDR129C	SAC6
YGL178W	MPT5	YBL061C	SKT5
YHR098C	SFB3	YBL061C	SKT5
YCR020C-A	MAK31	YDR320C	SWA2
YPR036W	VMA13	YFR036W	CDC26
YOR035C	SHE4	YPR119W	CLB2
YDR443C	SSN2	YNL238W	KEX2
YDR156W	RPA14	YGR217W	CCH1
YNL135C	FPR1	YGL252C	RTG2
YIL159W	BNR1	YGL115W	SNF4

Predictions at threshold level 0.80 continued...			
Gene1 of predicted pair		Gene2 of predicted pair	
Systematic Name	Standard Name	Systematic Name	Standard Name
YLR292C	SEC72	YPL018W	CTF19
YDR245W	MNN10	YLR425W	TUS1
YER096W	SHC1	YJL095W	BCK1
YDR335W	MSN5	YHR013C	ARD1
YLR079W	SIC1	YHR205W	SCH9
YGR275W	RTT102	YLR357W	RSC2
YGR133W	PEX4	YBL047C	EDE1
YLR441C	RPS1A	YOR066W	MSA1
YER110C	KAP123	YHR114W	BZZ1
YIL053W	RHR2	YHR081W	LRP1
YNL291C	MID1	YER116C	SLX8
YPL042C	SSN3	YBR194W	SOY1
YHR039C-A	VMA10	YLR398C	SKI2
YLR095C	IOC2	YEL060C	PRB1
YOR023C	AHC1	YGR054W	YGR054W
YGR239C	PEX21	YLR191W	PEX13
YBL021C	HAP3	YKL109W	HAP4
YBR195C	MSI1	YHR039C-A	VMA10
YDR108W	GSG1	YPL161C	BEM4
YIL072W	HOP1	YOR351C	MEK1
YNL325C	FIG4	YOR270C	VPH1
YPR084W	YPR084W	YML038C	YMD8
YPR029C	APL4	YOR036W	PEP12
YPL129W	TAF14	YKL109W	HAP4
YHR012W	VPS29	YJR090C	GRR1
YJR040W	GEF1	YDR207C	UME6
YIL036W	CST6	YJL124C	LSM1
YCR088W	ABP1	YDR310C	SUM1
YJL172W	CPS1	YNR072W	HXT17

Predictions at threshold level 0.80 continued...			
Gene1 of predicted pair		Gene2 of predicted pair	
Systematic Name	Standard Name	Systematic Name	Standard Name
YLR386W	VAC14	YOL121C	RPS19A
YLR095C	IOC2	YBR019C	GAL10
YNL106C	INP52	YJR060W	CBF1
YER044C	ERG28	YMR133W	REC114
YDR254W	CHL4	YPL161C	BEM4
YPL024W	RMI1	YER122C	GLO3
YGR249W	MGA1	YBL025W	RRN10
YOR043W	WHI2	YOR035C	SHE4
YLR083C	EMP70	YKR030W	GMH1
YOR270C	VPH1	YLR180W	SAM1
YDR507C	GIN4	YDR309C	GIC2
YDR076W	RAD55	YPR119W	CLB2
YPL129W	TAF14	YGR252W	GCN5
YDR146C	SWI5	YHR205W	SCH9
YNL288W	CAF40	YBL051C	PIN4
YGR108W	CLB1	YDL226C	GCS1
YKL007W	CAP1	YPR171W	BSP1
YOR109W	INP53	YER162C	RAD4
YER001W	MNN1	YGL223C	COG1
YGL147C	RPL9A	YLR192C	HCR1
YLL039C	UBI4	YPL050C	MNN9
YBL102W	SFT2	YDR153C	ENT5
YDR174W	HMO1	YKR061W	KTR2
YIL053W	RHR2	YOR337W	TEA1
YGL087C	MMS2	YPL161C	BEM4
YGL066W	SGF73	YJL140W	RPB4
YER087C-B	SBH1	YDL232W	OST4
YLR386W	VAC14	YER044C	ERG28
YDR108W	GSG1	YNL297C	MON2

Predictions at threshold level 0.80 continued...			
Gene1 of predicted pair		Gene2 of predicted pair	
Systematic Name	Standard Name	Systematic Name	Standard Name
YCR031C	RPS14A	YGR188C	BUB1
YGR261C	APL6	YNL051W	COG5
YDR174W	HMO1	YLR135W	SLX4
YGL100W	SEH1	YPL240C	HSP82
YLR148W	PEP3	YBL078C	ATG8
YLR061W	RPL22A	YGR133W	PEX4
YIL034C	CAP2	YJR090C	GRR1
YGL033W	HOP2	YBR019C	GAL10
YPR046W	MCM16	YDR358W	GGA1
YNL238W	KEX2	YOL121C	RPS19A
YHR098C	SFB3	YER016W	BIM1
YDR139C	RUB1	YLR128W	DCN1
YMR224C	MRE11	YML058W	SML1
YDR443C	SSN2	YGL003C	CDH1
YLR273C	PIG1	YIL045W	PIG2
YOR001W	RRP6	YOR038C	HIR2
YLR079W	SIC1	YBR215W	HPC2
YEL009C	GCN4	YPL042C	SSN3
YMR038C	CCS1	YLR350W	ORM2
YDR144C	MKC7	YPR046W	MCM16
YLR095C	IOC2	YLR386W	VAC14
YPR036W	VMA13	YPL161C	BEM4
YNL054W	VAC7	YNL107W	YAF9
YML013W	UBX2	YPR089W	YPR089W
YGR081C	SLX9	YMR038C	CCS1
YGL135W	RPL1B	YMR198W	CIK1
YEL050C	RML2	YBR200W	BEM1
YML115C	VAN1	YMR016C	SOK2
YPL115C	BEM3	YJL187C	SWE1

Predictions at threshold level 0.80 continued...			
Gene1 of predicted pair		Gene2 of predicted pair	
Systematic Name	Standard Name	Systematic Name	Standard Name
YNL238W	KEX2	YMR152W	YIM1
YJL036W	SNX4	YHR039C-A	VMA10
YLR192C	HCR1	YKR014C	YPT52
YPL057C	SUR1	YDR261C	EXG2
YMR142C	RPL13B	YDL184C	RPL41A
YBR215W	HPC2	YGL003C	CDH1
YMR004W	MVP1	YNR032W	PPG1
YLR360W	VPS38	YML115C	VAN1
YGL147C	RPL9A	YPL195W	APL5
YOR080W	DIA2	YLR182W	SWI6
YPL161C	BEM4	YDR469W	SDC1
YBR215W	HPC2	YLR370C	ARC18
YMR153W	NUP53	YHR081W	LRP1
YNR052C	POP2	YML016C	PPZ1
YDL116W	NUP84	YJR035W	RAD26
YER116C	SLX8	YKR099W	BAS1
YMR133W	REC114	YOR327C	SNC2
YEL009C	GCN4	YLR176C	RFX1
YOR023C	AHC1	YLR233C	EST1
YNL087W	TCB2	YLR441C	RPS1A
YKL126W	YPK1	YOR043W	WHI2
YNR032W	PPG1	YKR030W	GMH1
YDR293C	SSD1	YGL151W	NUT1
YMR224C	MRE11	YPR036W	VMA13
YDR108W	GSG1	YJL004C	SYS1
YLR170C	APS1	YOR036W	PEP12
YBR173C	UMP1	YBR019C	GAL10
YBL047C	EDE1	YPR120C	CLB5
YPR018W	RLF2	YGR081C	SLX9

Predictions at threshold level 0.80 continued...			
Gene1 of predicted pair		Gene2 of predicted pair	
Systematic Name	Standard Name	Systematic Name	Standard Name
YML103C	NUP188	YPL246C	RBD2
YMR109W	MYO5	YHR016C	YSC84
YPL129W	TAF14	YER161C	SPT2
YJR040W	GEF1	YGL115W	SNF4
YNL297C	MON2	YNR032W	PPG1
YNL041C	COG6	YDR153C	ENT5
YNL291C	MID1	YPL105C	YPL105C
YPR036W	VMA13	YLR371W	ROM2
YDR108W	GSG1	YDL006W	PTC1
YHR060W	VMA22	YEL043W	YEL043W
YML103C	NUP188	YKL117W	SBA1
YEL065W	SIT1	YER116C	SLX8
YDR004W	RAD57	YDL154W	MSH5
YLR240W	VPS34	YDR323C	PEP7
YDR202C	RAV2	YPL037C	EGD1
YJR040W	GEF1	YMR054W	STV1
YHR111W	UBA4	YGR152C	RSR1
YDR108W	GSG1	YCR066W	RAD18
YJR140C	HIR3	YBR089C-A	NHP6B
YGL237C	HAP2	YBR114W	RAD16
YNR052C	POP2	YPL022W	RAD1
YHR171W	ATG7	YOR327C	SNC2
YML001W	YPT7	YLR180W	SAM1
YLR292C	SEC72	YLR381W	CTF3
YHL031C	GOS1	YAL030W	SNC1
YDR378C	LSM6	YJL095W	BCK1
YDR146C	SWI5	YDR310C	SUM1
YGL163C	RAD54	YLR315W	NKP2
YKL007W	CAP1	YNL243W	SLA2

Predictions at threshold level 0.80 continued...			
Gene1 of predicted pair		Gene2 of predicted pair	
Systematic Name	Standard Name	Systematic Name	Standard Name
YOR337W	TEA1	YER162C	RAD4
YEL027W	CUP5	YLR447C	VMA6
YHR081W	LRP1	YMR152W	YIM1
YGL003C	CDH1	YLR182W	SWI6
YDR192C	NUP42	YLR131C	ACE2
YNL229C	URE2	YDL047W	SIT4
YLR191W	PEX13	YDR265W	PEX10
YJL124C	LSM1	YJL006C	CTK2
YOR033C	EXO1	YML028W	TSA1
YPR084W	YPR084W	YJR140C	HIR3
YOR106W	VAM3	YNR032W	PPG1
YBR019C	GAL10	YBL025W	RRN10
YHR073W	OSH3	YMR153W	NUP53
YDL116W	NUP84	YHR134W	WSS1
YDR378C	LSM6	YML112W	CTK3
YLR240W	VPS34	YLR360W	VPS38
YDR443C	SSN2	YIL159W	BNR1
YEL009C	GCN4	YGL252C	RTG2
YBR173C	UMP1	YER044C	ERG28
YKR095W	MLP1	YDR076W	RAD55
YGL147C	RPL9A	YLR386W	VAC14
YER116C	SLX8	YER040W	GLN3
YCR077C	PAT1	YGR135W	PRE9
YGR249W	MGA1	YLR192C	HCR1
YMR224C	MRE11	YMR179W	SPT21
YGR020C	VMA7	YJL095W	BCK1
YKR048C	NAP1	YNL078W	NIS1
YGL087C	MMS2	YJR032W	CPR7
YMR055C	BUB2	YDL013W	SLX5

Predictions at threshold level 0.80 continued...			
Gene1 of predicted pair		Gene2 of predicted pair	
Systematic Name	Standard Name	Systematic Name	Standard Name
YHR178W	STB5	YGL013C	PDR1
YDR293C	SSD1	YJL095W	BCK1
YNL087W	TCB2	YCR053W	THR4
YNL236W	SIN4	YDL226C	GCS1
YLR240W	VPS34	YLR396C	VPS33
YBR108W	YBR108W	YMR152W	YIM1
YEL065W	SIT1	YDR358W	GGA1
YFR024C-A	LSB3	YJL004C	SYS1
YPR131C	NAT3	YBR215W	HPC2
YOR066W	MSA1	YLR371W	ROM2
YKR099W	BAS1	YOR035C	SHE4
YOR106W	VAM3	YHR115C	DMA1
YLR090W	XDJ1	YMR142C	RPL13B
YLR399C	BDF1	YBR107C	IML3
YER042W	MXR1	YGR209C	TRX2
YDR496C	PUF6	YKL130C	SHE2
YOR327C	SNC2	YDL047W	SIT4
YPR084W	YPR084W	YHL020C	OPI1
YML115C	VAN1	YDR260C	SWM1
YNL291C	MID1	YJR033C	RAV1
YLR337C	VRP1	YKL006W	RPL14A
YMR039C	SUB1	YBR215W	HPC2
YBR030W	YBR030W	YER116C	SLX8
YOR083W	WHI5	YPR119W	CLB2
YNL199C	GCR2	YDR363W-A	SEM1
YLR433C	CNA1	YGR006W	PRP18
YPR084W	YPR084W	YLR360W	VPS38
YLR292C	SEC72	YJR090C	GRR1
YDR171W	HSP42	YDR318W	MCM21

Predictions at threshold level 0.80 continued...			
Gene1 of predicted pair		Gene2 of predicted pair	
Systematic Name	Standard Name	Systematic Name	Standard Name
YLR234W	TOP3	YJL138C	TIF2
YNL307C	MCK1	YIR005W	IST3
YEL043W	YEL043W	YLR309C	IMH1
YMR224C	MRE11	YML071C	COG8
YNL041C	COG6	YKR014C	YPT52
YLR095C	IOC2	YHR081W	LRP1
YEL043W	YEL043W	YML071C	COG8
YDR079C-A	TFB5	YPL240C	HSP82
YKL139W	CTK1	YCL011C	GBP2
YKR099W	BAS1	YNL076W	MKS1
YOR080W	DIA2	YPL018W	CTF19
YOR127W	RGA1	YDL047W	SIT4
YMR205C	PFK2	YBR131W	CCZ1
YDL020C	RPN4	YJR035W	RAD26
YPL240C	HSP82	YGR296W	YRF1-3
YIL159W	BNR1	YKR099W	BAS1
YDL184C	RPL41A	YFR036W	CDC26
YCL027W	FUS1	YKL092C	BUD2
YLR337C	VRP1	YNL079C	TPM1
YLR441C	RPS1A	YER139C	YER139C
YNL218W	MGS1	YBR228W	SLX1
YKR020W	VPS51	YHR039C-A	VMA10
YOL126C	MDH2	YMR164C	MSS11
YMR284W	YKU70	YOL030W	GAS5
YHR205W	SCH9	YNL271C	BNI1
YOR243C	PUS7	YGR071C	YGR071C
YDR245W	MNN10	YBL072C	RPS8A
YCR020C-A	MAK31	YNL107W	YAF9
YLR360W	VPS38	YAL002W	VPS8

Predictions at threshold level 0.80 continued...			
Gene1 of predicted pair		Gene2 of predicted pair	
Systematic Name	Standard Name	Systematic Name	Standard Name
YBR030W	YBR030W	YJL183W	MNN11
YGR054W	YGR054W	YAR014C	BUD14
YBL003C	HTA2	YNL107W	YAF9
YGL054C	ERV14	YKR030W	GMH1
YJR032W	CPR7	YNL076W	MKS1
YCR077C	PAT1	YBL101C	ECM21
YDR146C	SWI5	YML016C	PPZ1
YKR001C	VPS1	YJR060W	CBF1
YKL139W	CTK1	YML001W	YPT7
YFR010W	UBP6	YDR477W	SNF1
YBR108W	YBR108W	YPL161C	BEM4
YLR452C	SST2	YJL006C	CTK2
YDL005C	MED2	YNL236W	SIN4
YDL116W	NUP84	YML103C	NUP188
YHR178W	STB5	YGL237C	HAP2
YJR060W	CBF1	YER122C	GLO3
YIL076W	SEC28	YPR046W	MCM16
YHR039C-A	VMA10	YDL192W	ARF1
YPL115C	BEM3	YDR293C	SSD1
YGL087C	MMS2	YGR184C	UBR1
YEL050C	RML2	YDR318W	MCM21
YNL044W	YIP3	YJR032W	CPR7
YOR127W	RGA1	YHR111W	UBA4
YCR020C-A	MAK31	YJR033C	RAV1
YKL116C	PRR1	YMR230W	RPS10B
YBR231C	SWC5	YGL094C	PAN2
YJR040W	GEF1	YPL120W	VPS30
YNL325C	FIG4	YDL077C	VAM6
YDR144C	MKC7	YDR310C	SUM1

Predictions at threshold level 0.80 continued...			
Gene1 of predicted pair		Gene2 of predicted pair	
Systematic Name	Standard Name	Systematic Name	Standard Name
YBR200W	BEM1	YGL115W	SNF4
YPL105C	YPL105C	YNR032W	PPG1
YGL025C	PGD1	YPL248C	GAL4
YFR031C-A	RPL2A	YJL004C	SYS1
YNL236W	SIN4	YNL107W	YAF9
YDL116W	NUP84	YKR061W	KTR2
YML016C	PPZ1	YDR335W	MSN5
YMR127C	SAS2	YNL166C	BNI5
YPR131C	NAT3	YDR171W	HSP42
YDR245W	MNN10	YKL041W	VPS24
YMR109W	MYO5	YOR247W	SRL1
YNL025C	SSN8	YKL213C	DOA1
YLR083C	EMP70	YFR031C-A	RPL2A
YHR111W	UBA4	YOR327C	SNC2
YOR112W	CEX1	YML071C	COG8
YKL139W	CTK1	YMR016C	SOK2
YOR132W	VPS17	YBR108W	YBR108W
YGR104C	SRB5	YGL151W	NUT1
YGL212W	VAM7	YGL200C	EMP24
YHR013C	ARD1	YKL160W	ELF1
YHL025W	SNF6	YLR357W	RSC2
YER052C	HOM3	YNL135C	FPR1
YLR398C	SKI2	YLR180W	SAM1
YLR315W	NKP2	YHR167W	THP2
YOR337W	TEA1	YMR026C	PEX12
YMR026C	PEX12	YOL121C	RPS19A
YNL199C	GCR2	YLR350W	ORM2
YHR013C	ARD1	YIR005W	IST3
YOR156C	NFI1	YDR469W	SDC1

Predictions at threshold level 0.80 continued...			
Gene1 of predicted pair		Gene2 of predicted pair	
Systematic Name	Standard Name	Systematic Name	Standard Name
YER120W	SCS2	YML071C	COG8
YPL253C	VIK1	YJR060W	CBF1
YEL053C	MAK10	YDL077C	VAM6
YML115C	VAN1	YKL119C	VPH2
YBR171W	SEC66	YBL047C	EDE1
YJL172W	CPS1	YDL192W	ARF1
YDR477W	SNF1	YOR106W	VAM3
YKR001C	VPS1	YMR204C	INP1
YLR456W	YLR456W	YPR119W	CLB2
YGR135W	PRE9	YAR014C	BUD14
YLR381W	CTF3	YJR063W	RPA12
YDR137W	RGP1	YOL111C	MDY2
YJL176C	SWI3	YHR134W	WSS1
YNL054W	VAC7	YDR335W	MSN5
YGL223C	COG1	YEL053C	MAK10
YLR095C	IOC2	YPL161C	BEM4
YEL065W	SIT1	YMR142C	RPL13B
YPR084W	YPR084W	YBL102W	SFT2
YMR142C	RPL13B	YDR207C	UME6
YCR065W	HCM1	YNL041C	COG6
YIL034C	CAP2	YGL087C	MMS2
YPL161C	BEM4	YCR086W	CSM1
YGR019W	UGA1	YPL018W	CTF19
YGR104C	SRB5	YGL066W	SGF73
YNL087W	TCB2	YPL161C	BEM4
YKL135C	APL2	YDR358W	GGA1
YKR099W	BAS1	YOR327C	SNC2
YBR107C	IML3	YOL054W	PSH1
YLR386W	VAC14	YDR335W	MSN5

Predictions at threshold level 0.80 continued...			
Gene1 of predicted pair		Gene2 of predicted pair	
Systematic Name	Standard Name	Systematic Name	Standard Name
YJL053W	PEP8	YPL037C	EGD1
YOR080W	DIA2	YDR363W-A	SEM1
YBR215W	HPC2	YCR077C	PAT1
YIL053W	RHR2	YNL325C	FIG4
YBR217W	ATG12	YNL325C	FIG4
YML112W	CTK3	YGL252C	RTG2
YGR105W	VMA21	YDR358W	GGA1
YEL065W	SIT1	YDL083C	RPS16B
YOR112W	CEX1	YNL041C	COG6
YHR013C	ARD1	YKR014C	YPT52
YER019C-A	SBH2	YML019W	OST6
YIL034C	CAP2	YDL155W	CLB3
YLR119W	SRN2	YMR153W	NUP53
YNL325C	FIG4	YNR072W	HXT17
YOR337W	TEA1	YBR019C	GAL10
YBR131W	CCZ1	YBL101C	ECM21
YPL037C	EGD1	YLR398C	SKI2
YNL032W	SIW14	YMR273C	ZDS1
YKR001C	VPS1	YML001W	YPT7
YJL183W	MNN11	YNL076W	MKS1
YOL051W	GAL11	YDR363W-A	SEM1
YNL243W	SLA2	YKL135C	APL2
YOR290C	SNF2	YGL212W	VAM7
YDR448W	ADA2	YPL042C	SSN3
YLR301W	YLR301W	YPL240C	HSP82
YJL176C	SWI3	YPL079W	RPL21B
YGL087C	MMS2	YHL002W	HSE1
YPL253C	VIK1	YGL115W	SNF4
YMR116C	ASC1	YKL032C	IXR1

Predictions at threshold level 0.80 continued...			
Gene1 of predicted pair		Gene2 of predicted pair	
Systematic Name	Standard Name	Systematic Name	Standard Name
YOR069W	VPS5	YPL037C	EGD1
YMR095C	SNO1	YOR035C	SHE4
YML001W	YPT7	YJL095W	BCK1
YLR371W	ROM2	YPL195W	APL5
YPR051W	MAK3	YHR060W	VMA22
YLR192C	HCR1	YKL116C	PRR1
YER161C	SPT2	YOR038C	HIR2
YHR206W	SKN7	YJR074W	MOG1
YEL065W	SIT1	YDR477W	SNF1
YDR155C	CPR1	YNL107W	YAF9
YDR171W	HSP42	YJR005W	APL1
YJL154C	VPS35	YHR134W	WSS1
YDL223C	HBT1	YNL076W	MKS1
YKL048C	ELM1	YEL060C	PRB1
YGR105W	VMA21	YKR030W	GMH1
YOR270C	VPH1	YKL080W	VMA5
YHR161C	YAP1801	YNR049C	MSO1
YLR360W	VPS38	YKL048C	ELM1
YDL223C	HBT1	YNL079C	TPM1
YGL200C	EMP24	YKL041W	VPS24
YDR469W	SDC1	YOR308C	SNU66
YIL159W	BNR1	YNL032W	SIW14
YJL089W	SIP4	YMR280C	CAT8
YPL115C	BEM3	YLR006C	SSK1
YDR129C	SAC6	YDR389W	SAC7
YKL025C	PAN3	YLR381W	CTF3
YBR195C	MSI1	YGL163C	RAD54
YPL022W	RAD1	YML102W	CAC2
YJL053W	PEP8	YKR001C	VPS1

Predictions at threshold level 0.80 continued...			
Gene1 of predicted pair		Gene2 of predicted pair	
Systematic Name	Standard Name	Systematic Name	Standard Name
YGL200C	EMP24	YBL072C	RPS8A
YBR200W	BEM1	YDR171W	HSP42
YPR018W	RLF2	YPL024W	RMI1
YLR234W	TOP3	YPR051W	MAK3
YGL212W	VAM7	YAL030W	SNC1
YAL010C	MDM10	YLR254C	NDL1
YEL009C	GCN4	YBR111W-A	SUS1
YHR111W	UBA4	YGR054W	YGR054W
YHR098C	SFB3	YJR075W	HOC1
YBR200W	BEM1	YML016C	PPZ1
YBL051C	PIN4	YER110C	KAP123
YPR036W	VMA13	YDL083C	RPS16B
YHL027W	RIM101	YDR310C	SUM1
YGL078C	DBP3	YDR477W	SNF1
YML117W	NAB6	YBR131W	CCZ1
YLR288C	MEC3	YDR076W	RAD55
YPR030W	CSR2	YJL183W	MNN11
YNL309W	STB1	YLR452C	SST2
YNL291C	MID1	YGL100W	SEH1
YPL018W	CTF19	YOL091W	SPO21
YLR234W	TOP3	YGR188C	BUB1
YPL253C	VIK1	YKL048C	ELM1
YLR441C	RPS1A	YLR452C	SST2
YOR195W	SLK19	YLR032W	RAD5
YPL001W	HAT1	YGL161C	YIP5
YER116C	SLX8	YOR327C	SNC2
YIL034C	CAP2	YBL021C	HAP3
YGR270W	YTA7	YNL107W	YAF9
YAL024C	LTE1	YDR137W	RGP1

Predictions at threshold level 0.80 continued...			
Gene1 of predicted pair		Gene2 of predicted pair	
Systematic Name	Standard Name	Systematic Name	Standard Name
YGR249W	MGA1	YJL172W	CPS1
YBR173C	UMP1	YJL095W	BCK1
YPR046W	MCM16	YGL151W	NUT1
YKL116C	PRR1	YJL172W	CPS1
YJL136C	RPS21B	YHR200W	RPN10
YOR035C	SHE4	YOR327C	SNC2
YGL253W	HXK2	YEL056W	HAT2
YKL048C	ELM1	YMR133W	REC114
YFR010W	UBP6	YOR327C	SNC2
YAL055W	PEX22	YKL041W	VPS24
YOR337W	TEA1	YBR280C	SAF1
YLR192C	HCR1	YMR142C	RPL13B
YMR216C	SKY1	YGR105W	VMA21
YNL201C	PSY2	YCR086W	CSM1
YNL307C	MCK1	YER122C	GLO3
YNL307C	MCK1	YJR084W	CSN12
YCR002C	CDC10	YKL101W	HSL1
YNL206C	RTT106	YML016C	PPZ1
YER107C	GLE2	YOL012C	HTZ1
YDR320C	SWA2	YEL042W	GDA1
YNL201C	PSY2	YDL116W	NUP84
YLR386W	VAC14	YOL081W	IRA2
YJR032W	CPR7	YPR046W	MCM16
YGL212W	VAM7	YGL115W	SNF4
YLR094C	GIS3	YMR230W	RPS10B
YOR066W	MSA1	YJR032W	CPR7
YBR200W	BEM1	YNR072W	HXT17
YDR137W	RGP1	YLR315W	NKP2
YLR090W	XDJ1	YLR119W	SRN2

Predictions at threshold level 0.80 continued...			
Gene1 of predicted pair		Gene2 of predicted pair	
Systematic Name	Standard Name	Systematic Name	Standard Name
YLR337C	VRP1	YPR046W	MCM16
YOL147C	PEX11	YDL065C	PEX19
YDR484W	VPS52	YLR180W	SAM1
YKL025C	PAN3	YNL166C	BNI5
YBR171W	SEC66	YPR120C	CLB5
YMR016C	SOK2	YBR171W	SEC66
YPL024W	RMI1	YML058W	SML1
YKL025C	PAN3	YPL253C	VIK1
YLL022C	HIF1	YBR009C	HHF1
YEL065W	SIT1	YIL053W	RHR2
YPL161C	BEM4	YNL090W	RHO2
YGL161C	YIP5	YDR289C	RTT103
YJL036W	SNX4	YHR012W	VPS29
YJL006C	CTK2	YGL252C	RTG2
YNL297C	MON2	YNL051W	COG5
YHR134W	WSS1	YBL079W	NUP170
YBL078C	ATG8	YLR396C	VPS33
YDR320C	SWA2	YOR127W	RGA1
YOR132W	VPS17	YDR207C	UME6
YMR179W	SPT21	YLR410W	VIP1
YDL185W	TFP1	YJR033C	RAV1
YPR018W	RLF2	YDL013W	SLX5
YNL291C	MID1	YNL041C	COG6
YOR358W	HAP5	YJL095W	BCK1
YOR001W	RRP6	YJR135C	MCM22
YIL036W	CST6	YAR014C	BUD14
YMR055C	BUB2	YER161C	SPT2
YMR224C	MRE11	YKL110C	KTI12
YDR245W	MNN10	YBR041W	FAT1

Predictions at threshold level 0.80 continued...			
Gene1 of predicted pair		Gene2 of predicted pair	
Systematic Name	Standard Name	Systematic Name	Standard Name
YDR443C	SSN2	YBR173C	UMP1
YDR192C	NUP42	YGL163C	RAD54
YLR370C	ARC18	YNL107W	YAF9
YIL156W	UBP7	YKL117W	SBA1
YCL067C	HMLALPHA2	YCR084C	TUP1
YOR195W	SLK19	YBL047C	EDE1
YFR036W	CDC26	YKL041W	VPS24
YER107C	GLE2	YKR082W	NUP133
YML094W	GIM5	YHR056C	RSC30
YJL183W	MNN11	YEL036C	ANP1
YOL044W	PEX15	YGR077C	PEX8
YMR016C	SOK2	YBR200W	BEM1
YER049W	TPA1	YOR014W	RTS1
YGL115W	SNF4	YPL195W	APL5
YMR142C	RPL13B	YOL121C	RPS19A
YMR273C	ZDS1	YCR053W	THR4
YDR137W	RGP1	YOR308C	SNU66
YER001W	MNN1	YLR370C	ARC18
YLL026W	HSP104	YLR357W	RSC2
YMR198W	CIK1	YKR020W	VPS51
YNL032W	SIW14	YNL087W	TCB2
YNL087W	TCB2	YDR207C	UME6
YGL223C	COG1	YJR040W	GEF1
YPL129W	TAF14	YBL003C	HTA2
YBR020W	GAL1	YGR135W	PRE9
YPR051W	MAK3	YMR055C	BUB2
YMR116C	ASC1	YDR245W	MNN10
YPL057C	SUR1	YOL111C	MDY2
YER144C	UBP5	YJL095W	BCK1

Predictions at threshold level 0.80 continued...			
Gene1 of predicted pair		Gene2 of predicted pair	
Systematic Name	Standard Name	Systematic Name	Standard Name
YEL053C	MAK10	YOL018C	TLG2
YBL025W	RRN10	YDR310C	SUM1
YNL215W	IES2	YGL200C	EMP24
YNL230C	ELA1	YCL014W	BUD3
YNL206C	RTT106	YDR363W-A	SEM1
YJR032W	CPR7	YJL183W	MNN11
YCR065W	HCM1	YDL047W	SIT4
YLR292C	SEC72	YMR109W	MYO5
YKL116C	PRR1	YMR133W	REC114
YJL053W	PEP8	YPL161C	BEM4
YLR095C	IOC2	YBL103C	RTG3
YGL003C	CDH1	YOR327C	SNC2
YML016C	PPZ1	YNL079C	TPM1
YKR057W	RPS21A	YNL229C	URE2
YBR171W	SEC66	YDR183W	PLP1
YLR441C	RPS1A	YHR060W	VMA22
YJL172W	CPS1	YPL137C	GIP3
YOR001W	RRP6	YMR038C	CCS1
YLR288C	MEC3	YDR207C	UME6
YDR335W	MSN5	YBL079W	NUP170
YMR224C	MRE11	YJL187C	SWE1
YOR332W	VMA4	YJL095W	BCK1
YPR018W	RLF2	YHR010W	RPL27A
YDR480W	DIG2	YBL016W	FUS3
YIL053W	RHR2	YPL137C	GIP3
YBR200W	BEM1	YJR060W	CBF1
YLR148W	PEP3	YPL195W	APL5
YDR225W	HTA1	YNL206C	RTT106
YMR224C	MRE11	YNL058C	YNL058C

Predictions at threshold level 0.80 continued...			
Gene1 of predicted pair		Gene2 of predicted pair	
Systematic Name	Standard Name	Systematic Name	Standard Name
YBR127C	VMA2	YEL051W	VMA8
YKR035W-A	DID2	YPL137C	GIP3
YPL161C	BEM4	YPL256C	CLN2
YGR020C	VMA7	YEL043W	YEL043W
YML012W	ERV25	YML067C	ERV41
YLR093C	NYV1	YOR035C	SHE4
YAL010C	MDM10	YOL054W	PSH1
YMR109W	MYO5	YKL204W	EAP1
YGL212W	VAM7	YLR370C	ARC18
YGR270W	YTA7	YJR032W	CPR7
YDR484W	VPS52	YLR093C	NYV1
YOR304W	ISW2	YJR094C	IME1
YOR127W	RGA1	YJL136C	RPS21B
YKL139W	CTK1	YHR013C	ARD1
YNL206C	RTT106	YBR200W	BEM1
YCR084C	TUP1	YPR068C	HOS1
YNL288W	CAF40	YCR086W	CSM1
YKL007W	CAP1	YKR048C	NAP1
YLR370C	ARC18	YHL002W	HSE1
YLR386W	VAC14	YMR016C	SOK2
YBL102W	SFT2	YIL076W	SEC28
YGL212W	VAM7	YEL013W	VAC8
YNR072W	HXT17	YHR060W	VMA22
YCR086W	CSM1	YAL002W	VPS8
YNL107W	YAF9	YPR032W	SRO7
YDR320C	SWA2	YNR032W	PPG1
YBR289W	SNF5	YLR357W	RSC2
YOR270C	VPH1	YLR093C	NYV1
YDR507C	GIN4	YGL163C	RAD54

Predictions at threshold level 0.80 continued...			
Gene1 of predicted pair		Gene2 of predicted pair	
Systematic Name	Standard Name	Systematic Name	Standard Name
YFL023W	BUD27	YPL057C	SUR1
YFR010W	UBP6	YHR077C	NMD2
YGL100W	SEH1	YKL113C	RAD27
YDL155W	CLB3	YML028W	TSA1
YNL243W	SLA2	YLR262C	YPT6
YNR052C	POP2	YLR381W	CTF3
YBL102W	SFT2	YML001W	YPT7
YDL161W	ENT1	YLR396C	VPS33
YOR038C	HIR2	YDR440W	DOT1
YDL013W	SLX5	YGL163C	RAD54
YEL053C	MAK10	YGL115W	SNF4
YBL103C	RTG3	YER044C	ERG28
YOR275C	RIM20	YHL027W	RIM101
YBR173C	UMP1	YEL060C	PRB1
YCL001W	RER1	YMR054W	STV1
YPL253C	VIK1	YEL036C	ANP1
YNL325C	FIG4	YOR035C	SHE4
YLR386W	VAC14	YMR152W	YIM1
YCR066W	RAD18	YCL001W	RER1
YEL050C	RML2	YOR080W	DIA2
YER116C	SLX8	YFL023W	BUD27
YDL116W	NUP84	YFR030W	MET10
YLR083C	EMP70	YDL077C	VAM6
YIL076W	SEC28	YOR038C	HIR2
YMR172W	HOT1	YAR014C	BUD14
YCR086W	CSM1	YGL124C	MON1
YOR026W	BUB3	YNL243W	SLA2
YDL080C	THI3	YHR200W	RPN10
YLR313C	SPH1	YMR038C	CCS1

Predictions at threshold level 0.80 continued...			
Gene1 of predicted pair		Gene2 of predicted pair	
Systematic Name	Standard Name	Systematic Name	Standard Name
YKL139W	CTK1	YNR052C	POP2
YOR358W	HAP5	YMR273C	ZDS1
YJL095W	BCK1	YBR082C	UBC4
YJR056C	YJR056C	YPL195W	APL5
YGR054W	YGR054W	YEL053C	MAK10
YML001W	YPT7	YLR309C	IMH1
YCR020C-A	MAK31	YPR032W	SRO7
YML016C	PPZ1	YDR144C	MKC7
YJR032W	CPR7	YAL002W	VPS8
YNL325C	FIG4	YLR398C	SKI2
YBR108W	YBR108W	YGL115W	SNF4
YLR292C	SEC72	YJL190C	RPS22A
YBR274W	CHK1	YOR038C	HIR2
YCR084C	TUP1	YBR010W	HHT1
YDR244W	PEX5	YDR142C	PEX7
YDR137W	RGP1	YOR035C	SHE4
YOR290C	SNF2	YDL223C	HBT1
YDR137W	RGP1	YLR094C	GIS3
YJL006C	CTK2	YGR135W	PRE9
YMR026C	PEX12	YCR053W	THR4
YDR108W	GSG1	YGL174W	BUD13
YIR005W	IST3	YJR050W	ISY1
YOR132W	VPS17	YGR105W	VMA21
YDR207C	UME6	YAL002W	VPS8
YEL050C	RML2	YJR060W	CBF1
YKR035W-A	DID2	YMR230W	RPS10B
YFR010W	UBP6	YKL101W	HSL1
YER144C	UBP5	YFL023W	BUD27
YGL033W	HOP2	YBR200W	BEM1

Predictions at threshold level 0.80 continued...			
Gene1 of predicted pair		Gene2 of predicted pair	
Systematic Name	Standard Name	Systematic Name	Standard Name
YKR099W	BAS1	YNR072W	HXT17
YOR231W	MKK1	YLR313C	SPH1
YJR032W	CPR7	YLR350W	ORM2
YPR030W	CSR2	YPL195W	APL5
YMR016C	SOK2	YEL060C	PRB1
YOR358W	HAP5	YML057W	CMP2
YLR399C	BDF1	YNL225C	CNM67
YBR215W	HPC2	YDL226C	GCS1
YNL291C	MID1	YER162C	RAD4
YOR304W	ISW2	YLR182W	SWI6
YGL163C	RAD54	YNL225C	CNM67
YPL115C	BEM3	YMR055C	BUB2
YOR312C	RPL20B	YDR440W	DOT1
YBR173C	UMP1	YNL076W	MKS1
YKL116C	PRR1	YOR035C	SHE4
YLR360W	VPS38	YHR013C	ARD1
YBR107C	IML3	YHR200W	RPN10
YLR337C	VRP1	YML115C	VAN1
YBR173C	UMP1	YOL081W	IRA2
YOR039W	CKB2	YDL006W	PTC1
YDR144C	MKC7	YGL253W	HXK2
YEL053C	MAK10	YDL083C	RPS16B
YLR452C	SST2	YPR119W	CLB2
YOR109W	INP53	YML071C	COG8
YDL065C	PEX19	YPL112C	PEX25
YBR200W	BEM1	YNL058C	YNL058C
YOR127W	RGA1	YGL115W	SNF4
YLR148W	PEP3	YDL077C	VAM6
YNL291C	MID1	YKL101W	HSL1

Predictions at threshold level 0.80 continued...			
Gene1 of predicted pair		Gene2 of predicted pair	
Systematic Name	Standard Name	Systematic Name	Standard Name
YOR337W	TEA1	YFL023W	BUD27
YER116C	SLX8	YPL256C	CLN2
YAL024C	LTE1	YBR107C	IML3
YGL200C	EMP24	YLR398C	SKI2
YCL032W	STE50	YLR362W	STE11
YDL006W	PTC1	YPL195W	APL5
YPR131C	NAT3	YDR254W	CHL4
YAL024C	LTE1	YNL032W	SIW14
YLR090W	XDJ1	YER122C	GLO3
YBL025W	RRN10	YEL060C	PRB1
YJL006C	CTK2	YNL225C	CNM67
YPR052C	NHP6A	YGL178W	MPT5
YDR101C	ARX1	YER002W	NOP16
YJL176C	SWI3	YBR289W	SNF5
YER144C	UBP5	YLR452C	SST2
YOR337W	TEA1	YKL139W	CTK1
YDR261C	EXG2	YDR310C	SUM1
YJL122W	ALB1	YHR052W	CIC1
YLR094C	GIS3	YER162C	RAD4
YCR086W	CSM1	YJR090C	GRR1
YJL124C	LSM1	YDL226C	GCS1
YLR102C	APC9	YDR363W-A	SEM1
YLR381W	CTF3	YBR107C	IML3
YNL307C	MCK1	YER162C	RAD4
YMR153W	NUP53	YOL081W	IRA2
YDR137W	RGP1	YDR335W	MSN5
YBR108W	YBR108W	YPR051W	MAK3
YGR192C	TDH3	YPR119W	CLB2
YMR142C	RPL13B	YHR039C-A	VMA10

Predictions at threshold level 0.80 continued...			
**Gene1 of predicted pair**		**Gene2 of predicted pair**	
**Systematic Name**	**Standard Name**	**Systematic Name**	**Standard Name**
YLR192C	HCR1	YML001W	YPT7
YLR095C	IOC2	YJL183W	MNN11
YKL032C	IXR1	YKR048C	NAP1
YML016C	PPZ1	YOR023C	AHC1
YBR215W	HPC2	YDR363W-A	SEM1
YPR046W	MCM16	YER040W	GLN3
YMR198W	CIK1	YML071C	COG8
YNL307C	MCK1	YJL183W	MNN11
YPR036W	VMA13	YDR137W	RGP1
YJL128C	PBS2	YDL006W	PTC1
YMR167W	MLH1	YLR350W	ORM2
YPL253C	VIK1	YLR410W	VIP1
YDR254W	CHL4	YJR090C	GRR1
YBR020W	GAL1	YCL011C	GBP2
YOL121C	RPS19A	YGL003C	CDH1
YDR245W	MNN10	YOR038C	HIR2
YBR217W	ATG12	YMR142C	RPL13B
YDR443C	SSN2	YPL161C	BEM4
YMR224C	MRE11	YMR038C	CCS1
YGL124C	MON1	YJL190C	RPS22A
YJR032W	CPR7	YFR036W	CDC26
YGR184C	UBR1	YDR207C	UME6
YPL024W	RMI1	YGL115W	SNF4
YHR060W	VMA22	YDR358W	GGA1
YHR200W	RPN10	YJR060W	CBF1
YDL020C	RPN4	YNL106C	INP52
YGL200C	EMP24	YPL161C	BEM4
YFR031C-A	RPL2A	YMR004W	MVP1
YER144C	UBP5	YOL121C	RPS19A

Predictions at threshold level 0.80 continued...			
Gene1 of predicted pair		Gene2 of predicted pair	
Systematic Name	Standard Name	Systematic Name	Standard Name
YGR108W	CLB1	YPR046W	MCM16
YPL195W	APL5	YLR180W	SAM1
YOR083W	WHI5	YNL107W	YAF9
YLR061W	RPL22A	YKR014C	YPT52
YGR020C	VMA7	YDL185W	TFP1
YDR335W	MSN5	YOR368W	RAD17
YPL253C	VIK1	YJR090C	GRR1
YFL013C	IES1	YHR167W	THP2
YNL206C	RTT106	YPL001W	HAT1
YOR066W	MSA1	YDR207C	UME6
YLR094C	GIS3	YHR081W	LRP1
YIL076W	SEC28	YML115C	VAN1
YPL022W	RAD1	YCR086W	CSM1
YKL006W	RPL14A	YCR077C	PAT1
YER143W	DDI1	YOR327C	SNC2
YBR228W	SLX1	YGL175C	SAE2
YLR079W	SIC1	YOL126C	MDH2
YOR312C	RPL20B	YPR120C	CLB5
YDL116W	NUP84	YJR060W	CBF1
YNL291C	MID1	YCR086W	CSM1
YPR018W	RLF2	YGL163C	RAD54
YNL032W	SIW14	YNL076W	MKS1
YKL007W	CAP1	YDR310C	SUM1
YBR108W	YBR108W	YDR254W	CHL4
YNL215W	IES2	YPL129W	TAF14
YDR108W	GSG1	YEL042W	GDA1
YOL151W	GRE2	YPR119W	CLB2
YNL054W	VAC7	YLR410W	VIP1
YKR061W	KTR2	YGL252C	RTG2

Predictions at threshold level 0.80 continued...			
Gene1 of predicted pair		Gene2 of predicted pair	
Systematic Name	Standard Name	Systematic Name	Standard Name
YER107C	GLE2	YKL057C	NUP120
YFL026W	STE2	YNR006W	VPS27
YPL024W	RMI1	YGR071C	YGR071C
YOR101W	RAS1	YIL033C	BCY1
YBL102W	SFT2	YEL043W	YEL043W
YGL153W	PEX14	YLR370C	ARC18
YOR023C	AHC1	YJL095W	BCK1
YNL079C	TPM1	YJL183W	MNN11
YFR031C-A	RPL2A	YMR142C	RPL13B
YHR200W	RPN10	YDR310C	SUM1
YLR399C	BDF1	YFR030W	MET10
YLR148W	PEP3	YKR020W	VPS51
YDR448W	ADA2	YGL025C	PGD1
YNL054W	VAC7	YLR381W	CTF3
YML016C	PPZ1	YJR032W	CPR7
YGR019W	UGA1	YLR386W	VAC14
YEL050C	RML2	YPL253C	VIK1
YJL006C	CTK2	YDL226C	GCS1
YHR012W	VPS29	YNL079C	TPM1
YDL020C	RPN4	YMR231W	PEP5
YOR083W	WHI5	YBR200W	BEM1
YLR234W	TOP3	YDR076W	RAD55
YBR173C	UMP1	YER144C	UBP5
YBR030W	YBR030W	YBL103C	RTG3
YHR084W	STE12	YLR441C	RPS1A
YDR156W	RPA14	YML115C	VAN1
YGL078C	DBP3	YLR370C	ARC18
YFL013C	IES1	YKL160W	ELF1
YLR386W	VAC14	YDR507C	GIN4

Predictions at threshold level 0.80 continued...			
Gene1 of predicted pair		Gene2 of predicted pair	
Systematic Name	Standard Name	Systematic Name	Standard Name
YBR258C	SHG1	YGL003C	CDH1
YOR127W	RGA1	YCR086W	CSM1
YMR224C	MRE11	YML102W	CAC2
YGR252W	GCN5	YNL107W	YAF9
YKR035W-A	DID2	YMR214W	SCJ1
YLR386W	VAC14	YPL057C	SUR1
YGL087C	MMS2	YBR107C	IML3
YNL215W	IES2	YER118C	SHO1
YOR109W	INP53	YGL124C	MON1
YHR013C	ARD1	YGL252C	RTG2
YDL020C	RPN4	YKR030W	GMH1
YDR254W	CHL4	YML112W	CTK3
YEL065W	SIT1	YGL066W	SGF73
YNL068C	FKH2	YOR195W	SLK19
YBR200W	BEM1	YDL077C	VAM6
YOR195W	SLK19	YMR179W	SPT21
YOR368W	RAD17	YLR350W	ORM2
YOL051W	GAL11	YNL031C	HHT2
YKL126W	YPK1	YLR370C	ARC18
YNL325C	FIG4	YLR320W	MMS22
YAL024C	LTE1	YFR031C-A	RPL2A
YPR046W	MCM16	YHL002W	HSE1
YKL048C	ELM1	YIL008W	URM1
YBL021C	HAP3	YMR055C	BUB2
YOR275C	RIM20	YHR108W	GGA2
YKL139W	CTK1	YNL058C	YNL058C
YHL031C	GOS1	YGR270W	YTA7
YDL020C	RPN4	YHR010W	RPL27A
YLR119W	SRN2	YGL115W	SNF4

Predictions at threshold level 0.80 continued...			
Gene1 of predicted pair		Gene2 of predicted pair	
Systematic Name	Standard Name	Systematic Name	Standard Name
YOR156C	NFI1	YNL297C	MON2
YCR088W	ABP1	YKL204W	EAP1
YIL076W	SEC28	YEL043W	YEL043W
YPR120C	CLB5	YIL103W	DPH1
YPL129W	TAF14	YDR073W	SNF11
YBR030W	YBR030W	YMR230W	RPS10B
YDL155W	CLB3	YOL081W	IRA2
YNL041C	COG6	YOR035C	SHE4
YJL058C	BIT61	YPR119W	CLB2
YMR152W	YIM1	YGL124C	MON1
YAL014C	SYN8	YDR080W	VPS41
YPR046W	MCM16	YGL003C	CDH1
YPL022W	RAD1	YOL030W	GAS5
YNL215W	IES2	YDR363W-A	SEM1
YJR040W	GEF1	YJR033C	RAV1
YGL163C	RAD54	YJR135C	MCM22
YLR109W	AHP1	YIL008W	URM1
YDR439W	LRS4	YLR182W	SWI6
YGR135W	PRE9	YPL089C	RLM1
YOR023C	AHC1	YDL155W	CLB3
YBL017C	PEP1	YJL029C	VPS53
YLR435W	TSR2	YJL138C	TIF2
YNL206C	RTT106	YGR188C	BUB1
YDR293C	SSD1	YKL048C	ELM1
YER044C	ERG28	YBR036C	CSG2
YBR108W	YBR108W	YDL223C	HBT1
YJL172W	CPS1	YPR036W	VMA13
YGL147C	RPL9A	YPL137C	GIP3
YPR018W	RLF2	YBR010W	HHT1

Predictions at threshold level 0.80 continued...			
Gene1 of predicted pair		Gene2 of predicted pair	
Systematic Name	Standard Name	Systematic Name	Standard Name
YKL006W	RPL14A	YGL200C	EMP24
YPL140C	MKK2	YPR119W	CLB2
YOR195W	SLK19	YPR046W	MCM16
YMR172W	HOT1	YPR051W	MAK3
YBR030W	YBR030W	YLR371W	ROM2
YGL115W	SNF4	YJR032W	CPR7
YHR178W	STB5	YLR447C	VMA6
YNL201C	PSY2	YDR289C	RTT103
YLR079W	SIC1	YOR080W	DIA2
YNL307C	MCK1	YER040W	GLN3
YDL116W	NUP84	YKR024C	DBP7
YPR040W	TIP41	YDR309C	GIC2
YGR239C	PEX21	YOL044W	PEX15
YFL023W	BUD27	YMR311C	GLC8
YGR209C	TRX2	YDR423C	CAD1
YJR056C	YJR056C	YDL083C	RPS16B
YPL115C	BEM3	YDR289C	RTT103
YEL053C	MAK10	YPL120W	VPS30
YDR318W	MCM21	YOL067C	RTG1
YEL065W	SIT1	YLR192C	HCR1
YPR052C	NHP6A	YIL038C	NOT3
YNL288W	CAF40	YPR072W	NOT5
YML103C	NUP188	YML057W	CMP2
YNL025C	SSN8	YGR104C	SRB5
YNL218W	MGS1	YNR052C	POP2
YLL039C	UBI4	YKR020W	VPS51
YPR084W	YPR084W	YML071C	COG8
YGR105W	VMA21	YOR327C	SNC2
YAR014C	BUD14	YGR188C	BUB1

Predictions at threshold level 0.80 continued...			
**Gene1 of predicted pair**		**Gene2 of predicted pair**	
**Systematic Name**	**Standard Name**	**Systematic Name**	**Standard Name**
YPR036W	VMA13	YOL111C	MDY2
YDL116W	NUP84	YMR199W	CLN1
YPL018W	CTF19	YJL183W	MNN11
YBR030W	YBR030W	YLR093C	NYV1
YMR199W	CLN1	YHR152W	SPO12
YLR288C	MEC3	YDL013W	SLX5
YOL111C	MDY2	YMR311C	GLC8
YBL058W	SHP1	YDR207C	UME6
YFR031C-A	RPL2A	YJR032W	CPR7
YNR052C	POP2	YGR134W	CAF130
YGL060W	YBP2	YGL115W	SNF4
YGL033W	HOP2	YNL076W	MKS1
YLL010C	PSR1	YOR043W	WHI2
YDR289C	RTT103	YHR115C	DMA1
YKL116C	PRR1	YNL307C	MCK1
YOR358W	HAP5	YCR086W	CSM1
YLR337C	VRP1	YJL176C	SWI3
YDR137W	RGP1	YIR005W	IST3
YDR245W	MNN10	YER049W	TPA1
YHR060W	VMA22	YMR214W	SCJ1
YJR040W	GEF1	YHR039C-A	VMA10
YGL212W	VAM7	YDR310C	SUM1
YBR030W	YBR030W	YNL041C	COG6
YBR019C	GAL10	YDR207C	UME6
YHR039C-A	VMA10	YJL004C	SYS1
YML115C	VAN1	YMR142C	RPL13B
YBR171W	SEC66	YER122C	GLO3
YDR264C	AKR1	YHR115C	DMA1
YMR224C	MRE11	YCR066W	RAD18

Predictions at threshold level 0.80 continued...			
Gene1 of predicted pair		Gene2 of predicted pair	
Systematic Name	Standard Name	Systematic Name	Standard Name
YDR146C	SWI5	YLR371W	ROM2
YLR441C	RPS1A	YER162C	RAD4
YNL032W	SIW14	YEL053C	MAK10
YML071C	COG8	YGL003C	CDH1
YPL213W	LEA1	YGR135W	PRE9
YBR194W	SOY1	YLR398C	SKI2
YNR031C	SSK2	YCR073C	SSK22
YIL076W	SEC28	YOR124C	UBP2
YNL054W	VAC7	YGL163C	RAD54
YMR202W	ERG2	YDL226C	GCS1
YDR202C	RAV2	YGR105W	VMA21
YPL057C	SUR1	YOR035C	SHE4
YDR073W	SNF11	YKL109W	HAP4
YDR378C	LSM6	YNL225C	CNM67
YLR234W	TOP3	YNL135C	FPR1
YBL051C	PIN4	YPR046W	MCM16
YHL031C	GOS1	YMR216C	SKY1
YDR171W	HSP42	YJR060W	CBF1
YLR119W	SRN2	YJR056C	YJR056C
YNL291C	MID1	YLR309C	IMH1
YLR234W	TOP3	YLR313C	SPH1
YFR010W	UBP6	YPR119W	CLB2
YKR057W	RPS21A	YDL047W	SIT4
YBL072C	RPS8A	YEL036C	ANP1
YBR126C	TPS1	YMR109W	MYO5
YER162C	RAD4	YAL002W	VPS8
YKL025C	PAN3	YJL095W	BCK1
YGR071C	YGR071C	YPL079W	RPL21B
YMR004W	MVP1	YAR014C	BUD14

Predictions at threshold level 0.80 continued...			
Gene1 of predicted pair		Gene2 of predicted pair	
Systematic Name	Standard Name	Systematic Name	Standard Name
YER144C	UBP5	YJR056C	YJR056C
YMR273C	ZDS1	YHL011C	PRS3
YOR337W	TEA1	YML071C	COG8
YLR061W	RPL22A	YML115C	VAN1
YMR231W	PEP5	YPL256C	CLN2
YNL291C	MID1	YKR001C	VPS1
YML016C	PPZ1	YGL237C	HAP2
YGL087C	MMS2	YJR090C	GRR1
YPL253C	VIK1	YIR005W	IST3
YNL243W	SLA2	YOR069W	VPS5
YMR055C	BUB2	YEL053C	MAK10
YOR109W	INP53	YOL108C	INO4
YKL126W	YPK1	YOR327C	SNC2
YHR134W	WSS1	YIL008W	URM1
YML103C	NUP188	YJL124C	LSM1
YGL223C	COG1	YCR066W	RAD18
YNL243W	SLA2	YPR046W	MCM16
YNL297C	MON2	YOR368W	RAD17
YPL024W	RMI1	YBR195C	MSI1
YML117W	NAB6	YDR245W	MNN10
YGL174W	BUD13	YIR005W	IST3
YDR484W	VPS52	YJR056C	YJR056C
YFR010W	UBP6	YML001W	YPT7
YJR060W	CBF1	YJR005W	APL1
YDR484W	VPS52	YPR030W	CSR2
YPL045W	VPS16	YDR323C	PEP7
YLL039C	UBI4	YEL036C	ANP1
YMR205C	PFK2	YBR082C	UBC4
YMR039C	SUB1	YCR086W	CSM1

Predictions at threshold level 0.80 continued...			
Gene1 of predicted pair		Gene2 of predicted pair	
Systematic Name	Standard Name	Systematic Name	Standard Name
YDR439W	LRS4	YBR107C	IML3
YNL236W	SIN4	YNL079C	TPM1
YAL024C	LTE1	YER040W	GLN3
YKL139W	CTK1	YMR153W	NUP53
YKL025C	PAN3	YLR447C	VMA6
YGL078C	DBP3	YML001W	YPT7
YFR031C-A	RPL2A	YMR216C	SKY1
YKR001C	VPS1	YNR032W	PPG1
YBR057C	MUM2	YHR200W	RPN10
YER110C	KAP123	YDR144C	MKC7
YAL024C	LTE1	YIL103W	DPH1
YER161C	SPT2	YOR308C	SNU66
YOR127W	RGA1	YER120W	SCS2
YMR201C	RAD14	YJR052W	RAD7
YGL078C	DBP3	YDR137W	RGP1
YDL160C	DHH1	YML109W	ZDS2
YNL058C	YNL058C	YLR452C	SST2
YOR337W	TEA1	YPL195W	APL5
YJL124C	LSM1	YPL018W	CTF19
YKR059W	TIF1	YCR031C	RPS14A
YPL253C	VIK1	YBR260C	RGD1
YDR363W-A	SEM1	YKL041W	VPS24
YAL024C	LTE1	YDL184C	RPL41A
YGR081C	SLX9	YDR076W	RAD55
YLR357W	RSC2	YOR064C	YNG1
YMR109W	MYO5	YOR035C	SHE4
YPL161C	BEM4	YGR135W	PRE9
YPL161C	BEM4	YHR114W	BZZ1
YER044C	ERG28	YNL041C	COG6

Predictions at threshold level 0.80 continued...			
Gene1 of predicted pair		Gene2 of predicted pair	
Systematic Name	Standard Name	Systematic Name	Standard Name
YLR095C	IOC2	YDL077C	VAM6
YLR398C	SKI2	YBR036C	CSG2
YPL057C	SUR1	YLR180W	SAM1
YOR080W	DIA2	YDR385W	EFT2
YKL117W	SBA1	YPL256C	CLN2
YDR443C	SSN2	YFL023W	BUD27
YMR133W	REC114	YDR207C	UME6
YDL020C	RPN4	YLR447C	VMA6
YIL034C	CAP2	YNL107W	YAF9
YCL001W	RER1	YAR014C	BUD14
YJL172W	CPS1	YPL057C	SUR1
YBR057C	MUM2	YMR089C	YTA12
YHR060W	VMA22	YER040W	GLN3
YGL178W	MPT5	YOL051W	GAL11
YLR303W	MET17	YLR377C	FBP1
YDL083C	RPS16B	YLR371W	ROM2
YKR099W	BAS1	YHR200W	RPN10
YPR029C	APL4	YPR119W	CLB2
YPL129W	TAF14	YKL129C	MYO3
YLR234W	TOP3	YNL107W	YAF9
YML038C	YMD8	YNL051W	COG5
YML103C	NUP188	YJR060W	CBF1
YBR130C	SHE3	YKL185W	ASH1
YPR036W	VMA13	YOL081W	IRA2
YGR241C	YAP1802	YOR035C	SHE4
YEL053C	MAK10	YDR310C	SUM1
YDL020C	RPN4	YKL041W	VPS24
YGL147C	RPL9A	YOR035C	SHE4
YJL154C	VPS35	YKR001C	VPS1

Predictions at threshold level 0.80 continued...			
Gene1 of predicted pair		Gene2 of predicted pair	
Systematic Name	Standard Name	Systematic Name	Standard Name
YGL151W	NUT1	YGL148W	ARO2
YLR148W	PEP3	YDR080W	VPS41
YDR202C	RAV2	YDL006W	PTC1
YMR142C	RPL13B	YER116C	SLX8
YLR386W	VAC14	YLR452C	SST2
YGL115W	SNF4	YOR035C	SHE4
YKL139W	CTK1	YLR398C	SKI2
YPR036W	VMA13	YER139C	YER139C
YJL210W	PEX2	YGL153W	PEX14
YFR024C-A	LSB3	YPL246C	RBD2
YKR101W	SIR1	YMR273C	ZDS1
YMR198W	CIK1	YDR200C	VPS64
YIL159W	BNR1	YBR126C	TPS1
YLR102C	APC9	YBR195C	MSI1
YHR156C	LIN1	YPR046W	MCM16
YER161C	SPT2	YCR086W	CSM1
YBR270C	BIT2	YJL058C	BIT61
YPR084W	YPR084W	YEL013W	VAC8
YBR228W	SLX1	YNR052C	POP2
YBR030W	YBR030W	YDL184C	RPL41A
YDL127W	PCL2	YJL124C	LSM1
YDR320C	SWA2	YPL195W	APL5
YLR360W	VPS38	YIL008W	URM1
YBL047C	EDE1	YDR440W	DOT1
YNL087W	TCB2	YBR200W	BEM1
YJL190C	RPS22A	YAR014C	BUD14
YHR178W	STB5	YML115C	VAN1
YDR425W	SNX41	YJL036W	SNX4
YDR137W	RGP1	YLR452C	SST2

Predictions at threshold level 0.80 continued...			
Gene1 of predicted pair		Gene2 of predicted pair	
Systematic Name	Standard Name	Systematic Name	Standard Name
YOL111C	MDY2	YER139C	YER139C
YLR288C	MEC3	YJR084W	CSN12
YKL139W	CTK1	YNL107W	YAF9
YNL079C	TPM1	YDR207C	UME6
YGL223C	COG1	YJR060W	CBF1
YMR016C	SOK2	YDR261C	EXG2
YHL031C	GOS1	YJR032W	CPR7
YGL066W	SGF73	YDR207C	UME6
YML115C	VAN1	YHL002W	HSE1
YDR379W	RGA2	YDR264C	AKR1
YNL054W	VAC7	YOL054W	PSH1
YJR033C	RAV1	YER122C	GLO3
YCR077C	PAT1	YOL081W	IRA2
YHR156C	LIN1	YDR310C	SUM1
YJL124C	LSM1	YCR077C	PAT1
YGR241C	YAP1802	YJL154C	VPS35
YDR490C	PKH1	YPR119W	CLB2
YBR200W	BEM1	YBR131W	CCZ1
YGR184C	UBR1	YOR368W	RAD17
YJR135C	MCM22	YML112W	CTK3
YLR441C	RPS1A	YLR371W	ROM2
YGL060W	YBP2	YNL107W	YAF9
YPR131C	NAT3	YMR055C	BUB2
YDR448W	ADA2	YIL038C	NOT3
YDL184C	RPL41A	YBR019C	GAL10
YPL037C	EGD1	YLR320W	MMS22
YDR320C	SWA2	YGR020C	VMA7
YOL082W	ATG19	YKL103C	LAP4
YEL022W	GEA2	YER122C	GLO3

Predictions at threshold level 0.80 continued...			
Gene1 of predicted pair		Gene2 of predicted pair	
Systematic Name	Standard Name	Systematic Name	Standard Name
YJL089W	SIP4	YER027C	GAL83
YAL010C	MDM10	YFR030W	MET10
YNR052C	POP2	YOR368W	RAD17
YKL139W	CTK1	YDL192W	ARF1
YNL291C	MID1	YOR195W	SLK19
YMR219W	ESC1	YPR119W	CLB2
YKL139W	CTK1	YHR167W	THP2
YCR077C	PAT1	YMR038C	CCS1
YBR200W	BEM1	YBL087C	RPL23A
YOR270C	VPH1	YOL111C	MDY2
YDR448W	ADA2	YJL140W	RPB4
YMR172W	HOT1	YGR188C	BUB1
YPL024W	RMI1	YGL148W	ARO2
YOR191W	RIS1	YJR140C	HIR3
YBR215W	HPC2	YGR270W	YTA7
YER162C	RAD4	YGL003C	CDH1
YHL022C	SPO11	YGL003C	CDH1
YNL243W	SLA2	YOL018C	TLG2
YNL291C	MID1	YGL054C	ERV14
YDR137W	RGP1	YPL195W	APL5
YMR179W	SPT21	YDR378C	LSM6
YBL027W	RPL19B	YMR055C	BUB2
YOR112W	CEX1	YDR207C	UME6
YMR026C	PEX12	YMR016C	SOK2
YDR363W-A	SEM1	YJR135C	MCM22
YOR132W	VPS17	YGL054C	ERV14
YIL076W	SEC28	YNR072W	HXT17
YLR079W	SIC1	YLR410W	VIP1
YHR056C	RSC30	YLR200W	YKE2

Predictions at threshold level 0.80 continued...			
Gene1 of predicted pair		Gene2 of predicted pair	
Systematic Name	Standard Name	Systematic Name	Standard Name
YML117W	NAB6	YBR041W	FAT1
YPL161C	BEM4	YHL020C	OPI1
YLR095C	IOC2	YJR032W	CPR7
YHR134W	WSS1	YBR108W	YBR108W
YER162C	RAD4	YJL183W	MNN11
YGL133W	ITC1	YMR199W	CLN1
YGR135W	PRE9	YBR082C	UBC4
YLR360W	VPS38	YPL045W	VPS16
YMR055C	BUB2	YJL095W	BCK1
YLR337C	VRP1	YIL130W	ASG1
YGL212W	VAM7	YBL025W	RRN10
YHR111W	UBA4	YPL152W	RRD2
YOR195W	SLK19	YDR335W	MSN5
YKL007W	CAP1	YIR003W	YIR003W
YDR484W	VPS52	YKR020W	VPS51
YNR072W	HXT17	YMR311C	GLC8
YIL053W	RHR2	YKR035W-A	DID2
YDR378C	LSM6	YDL047W	SIT4
YNL307C	MCK1	YDR363W-A	SEM1
YMR142C	RPL13B	YML071C	COG8
YPL022W	RAD1	YHR200W	RPN10
YDR261C	EXG2	YDL192W	ARF1
YDR383C	NKP1	YCR086W	CSM1
YCR065W	HCM1	YNL107W	YAF9
YNL297C	MON2	YML028W	TSA1
YGL133W	ITC1	YKR057W	RPS21A
YFL023W	BUD27	YBR215W	HPC2
YGR261C	APL6	YER120W	SCS2
YGL115W	SNF4	YOL121C	RPS19A

Predictions at threshold level 0.80 continued...			
**Gene1 of predicted pair**		**Gene2 of predicted pair**	
**Systematic Name**	**Standard Name**	**Systematic Name**	**Standard Name**
YMR095C	SNO1	YLR371W	ROM2
YPL195W	APL5	YJR033C	RAV1
YJR135C	MCM22	YBR114W	RAD16
YLR119W	SRN2	YPL195W	APL5
YLL007C	YLL007C	YLR292C	SEC72
YGL250W	RMR1	YOR368W	RAD17
YJR040W	GEF1	YOL108C	INO4
YFR031C-A	RPL2A	YNL238W	KEX2
YDR202C	RAV2	YCL037C	SRO9
YHR134W	WSS1	YJL190C	RPS22A
YPL240C	HSP82	YER074W-A	YOS1
YKL048C	ELM1	YBR114W	RAD16
YOR106W	VAM3	YKR001C	VPS1
YBR084C-A	RPL19A	YNL116W	DMA2
YFR010W	UBP6	YGL212W	VAM7
YOR275C	RIM20	YPR173C	VPS4
YER116C	SLX8	YOL090W	MSH2
YCR031C	RPS14A	YOR368W	RAD17
YFR010W	UBP6	YKL048C	ELM1
YJL124C	LSM1	YCR086W	CSM1
YFR010W	UBP6	YAR014C	BUD14
YBR030W	YBR030W	YBR171W	SEC66
YBL058W	SHP1	YML001W	YPT7
YJR140C	HIR3	YOR039W	CKB2
YPL057C	SUR1	YPL018W	CTF19
YJL060W	BNA3	YLR370C	ARC18
YGL003C	CDH1	YNL076W	MKS1
YPR173C	VPS4	YOL018C	TLG2
YGR108W	CLB1	YJL190C	RPS22A

Predictions at threshold level 0.80 continued...			
Gene1 of predicted pair		Gene2 of predicted pair	
Systematic Name	Standard Name	Systematic Name	Standard Name
YIL076W	SEC28	YCR066W	RAD18
YNL087W	TCB2	YPL137C	GIP3
YKR057W	RPS21A	YDR379W	RGA2
YBL025W	RRN10	YDL077C	VAM6
YDR245W	MNN10	YLR320W	MMS22
YDR080W	VPS41	YER122C	GLO3
YOR132W	VPS17	YPL253C	VIK1
YNL243W	SLA2	YGR200C	ELP2
YMR133W	REC114	YNL076W	MKS1
YMR216C	SKY1	YML071C	COG8
YPL057C	SUR1	YLR032W	RAD5
YOR270C	VPH1	YFR036W	CDC26
YJL004C	SYS1	YGL124C	MON1
YDL116W	NUP84	YBR215W	HPC2
YLR083C	EMP70	YCL001W	RER1
YLR234W	TOP3	YJL124C	LSM1
YDR335W	MSN5	YNL116W	DMA2
YPR036W	VMA13	YOR035C	SHE4
YGR040W	KSS1	YDR480W	DIG2
YMR026C	PEX12	YGL115W	SNF4
YPR131C	NAT3	YLR234W	TOP3
YFR032C-A	RPL29	YBR041W	FAT1
YLR119W	SRN2	YMR230W	RPS10B
YKL032C	IXR1	YDR289C	RTT103
YBL008W	HIR1	YPL001W	HAT1
YIR005W	IST3	YIL103W	DPH1
YPL234C	TFP3	YDR153C	ENT5
YER174C	GRX4	YFR030W	MET10
YEL044W	IES6	YEL036C	ANP1

Predictions at threshold level 0.80 continued...			
Gene1 of predicted pair		Gene2 of predicted pair	
Systematic Name	Standard Name	Systematic Name	Standard Name
YPL024W	RMI1	YGR249W	MGA1
YCL001W	RER1	YEL051W	VMA8
YMR092C	AIP1	YOR035C	SHE4
YHR193C	EGD2	YPL037C	EGD1
YDR245W	MNN10	YKL161C	YKL161C
YMR016C	SOK2	YDR358W	GGA1
YGR105W	VMA21	YDR207C	UME6
YPL057C	SUR1	YDR254W	CHL4
YNL206C	RTT106	YNL031C	HHT2
YOR270C	VPH1	YJR056C	YJR056C
YLR360W	VPS38	YPL253C	VIK1
YJR040W	GEF1	YML038C	YMD8
YDR477W	SNF1	YMR311C	GLC8
YMR198W	CIK1	YGL161C	YIP5
YEL044W	IES6	YOR312C	RPL20B
YGR241C	YAP1802	YMR231W	PEP5
YGR261C	APL6	YEL043W	YEL043W
YML115C	VAN1	YDR245W	MNN10
YJR032W	CPR7	YAR014C	BUD14
YBR200W	BEM1	YPR040W	TIP41
YHR178W	STB5	YMR109W	MYO5
YLR150W	STM1	YPL018W	CTF19
YDR244W	PEX5	YIL160C	POT1
YMR026C	PEX12	YOR290C	SNF2
YIL034C	CAP2	YLR337C	VRP1
YER102W	RPS8B	YBL072C	RPS8A
YLR248W	RCK2	YMR243C	ZRC1
YDR153C	ENT5	YKR030W	GMH1
YOL145C	CTR9	YGL003C	CDH1

Predictions at threshold level 0.80 continued...			
Gene1 of predicted pair		Gene2 of predicted pair	
Systematic Name	Standard Name	Systematic Name	Standard Name
YDR443C	SSN2	YPR046W	MCM16
YJL154C	VPS35	YHR205W	SCH9
YML028W	TSA1	YOR014W	RTS1
YLR095C	IOC2	YKR099W	BAS1
YFR031C-A	RPL2A	YML071C	COG8
YGL087C	MMS2	YDR254W	CHL4
YNL041C	COG6	YJR033C	RAV1
YLR090W	XDJ1	YMR095C	SNO1
YMR159C	ATG16	YPL149W	ATG5
YNL079C	TPM1	YMR311C	GLC8
YBR278W	DPB3	YEL056W	HAT2
YKR014C	YPT52	YAL002W	VPS8
YHR060W	VMA22	YMR152W	YIM1
YDL116W	NUP84	YDR439W	LRS4
YNL215W	IES2	YDR378C	LSM6
YBR107C	IML3	YFR036W	CDC26
YNL201C	PSY2	YOR195W	SLK19
YHR013C	ARD1	YPL152W	RRD2
YKL025C	PAN3	YJL190C	RPS22A
YPL161C	BEM4	YGL003C	CDH1
YKL139W	CTK1	YLR182W	SWI6
YBR030W	YBR030W	YLR094C	GIS3
YKL048C	ELM1	YJL183W	MNN11
YOR106W	VAM3	YPL256C	CLN2
YNL215W	IES2	YMR055C	BUB2
YMR055C	BUB2	YJL183W	MNN11
YJR059W	PTK2	YPL178W	CBC2
YDR439W	LRS4	YFR036W	CDC26
YKR072C	SIS2	YBL079W	NUP170

Predictions at threshold level 0.80 continued...			
Gene1 of predicted pair		Gene2 of predicted pair	
Systematic Name	Standard Name	Systematic Name	Standard Name
YMR016C	SOK2	YLR180W	SAM1
YER139C	YER139C	YML097C	VPS9
YJL154C	VPS35	YCR086W	CSM1
YPL024W	RMI1	YOR038C	HIR2
YOR127W	RGA1	YKL048C	ELM1
YOR112W	CEX1	YEL043W	YEL043W
YMR164C	MSS11	YGR059W	SPR3
YJR140C	HIR3	YBR274W	CHK1
YGL087C	MMS2	YCR086W	CSM1
YDL083C	RPS16B	YPL195W	APL5
YMR095C	SNO1	YER139C	YER139C
YPR046W	MCM16	YBR114W	RAD16
YIL159W	BNR1	YKR020W	VPS51
YLR398C	SKI2	YDR310C	SUM1
YMR224C	MRE11	YHR010W	RPL27A
YBR127C	VMA2	YAL030W	SNC1
YBL103C	RTG3	YMR133W	REC114
YMR133W	REC114	YPL137C	GIP3
YJL060W	BNA3	YJR005W	APL1
YEL065W	SIT1	YDL223C	HBT1
YKR035W-A	DID2	YGL115W	SNF4
YLR370C	ARC18	YMR214W	SCJ1
YOR115C	TRS33	YEL022W	GEA2
YJL154C	VPS35	YDR108W	GSG1
YOR127W	RGA1	YER162C	RAD4
YFR010W	UBP6	YDR310C	SUM1
YDR004W	RAD57	YOR368W	RAD17
YBR108W	YBR108W	YLR441C	RPS1A
YOR127W	RGA1	YGL031C	RPL24A

Predictions at threshold level 0.80 continued...			
Gene1 of predicted pair		Gene2 of predicted pair	
Systematic Name	Standard Name	Systematic Name	Standard Name
YDR293C	SSD1	YBR114W	RAD16
YER116C	SLX8	YER122C	GLO3
YPL240C	HSP82	YER180C-A	SLO1
YML103C	NUP188	YLR399C	BDF1
YNL293W	MSB3	YPL256C	CLN2
YBR171W	SEC66	YCL001W	RER1
YNL069C	RPL16B	YML038C	YMD8
YDL160C	DHH1	YMR273C	ZDS1
YPR018W	RLF2	YBR057C	MUM2
YBR217W	ATG12	YOL081W	IRA2
YJR058C	APS2	YOR036W	PEP12
YDL160C	DHH1	YDR071C	PAA1
YOR043W	WHI2	YDR310C	SUM1
YBR020W	GAL1	YPL248C	GAL4
YGR249W	MGA1	YHR200W	RPN10
YBR171W	SEC66	YHR108W	GGA2
YNL236W	SIN4	YBR089C-A	NHP6B
YKL048C	ELM1	YPL018W	CTF19
YBR217W	ATG12	YNL238W	KEX2
YKL139W	CTK1	YOR035C	SHE4
YOR023C	AHC1	YAL002W	VPS8
YEL051W	VMA8	YOL108C	INO4
YML001W	YPT7	YNR032W	PPG1
YDL116W	NUP84	YMR224C	MRE11
YOR337W	TEA1	YOL111C	MDY2
YBL051C	PIN4	YAR014C	BUD14
YOR032C	HMS1	YCR077C	PAT1
YPL018W	CTF19	YOL054W	PSH1
YPL256C	CLN2	YLR352W	YLR352W

Predictions at threshold level 0.80 continued...			
Gene1 of predicted pair		Gene2 of predicted pair	
Systematic Name	Standard Name	Systematic Name	Standard Name
YHR013C	ARD1	YNL236W	SIN4
YLR399C	BDF1	YIR005W	IST3
YDL184C	RPL41A	YNL238W	KEX2
YJR024C	MDE1	YCR024C-A	PMP1
YDR108W	GSG1	YER162C	RAD4
YKR001C	VPS1	YPL234C	TFP3
YJR063W	RPA12	YBR010W	HHT1
YGL212W	VAM7	YGL223C	COG1
YGR270W	YTA7	YNL225C	CNM67
YDR254W	CHL4	YPR120C	CLB5
YHR200W	RPN10	YJR035W	RAD26
YMR133W	REC114	YML001W	YPT7
YKL117W	SBA1	YFL047W	RGD2
YDR448W	ADA2	YBR289W	SNF5
YLR418C	CDC73	YBR089C-A	NHP6B
YPL037C	EGD1	YMR133W	REC114
YGL174W	BUD13	YLR182W	SWI6
YML013W	UBX2	YBR283C	SSH1
YLL039C	UBI4	YDL190C	UFD2
YOR127W	RGA1	YMR055C	BUB2
YDR183W	PLP1	YAR014C	BUD14
YIL076W	SEC28	YNL076W	MKS1
YGL147C	RPL9A	YEL060C	PRB1
YBR173C	UMP1	YCR066W	RAD18
YFR010W	UBP6	YGR184C	UBR1
YJR032W	CPR7	YDR318W	MCM21
YIL159W	BNR1	YDR358W	GGA1
YML095C	RAD10	YKL048C	ELM1
YBL103C	RTG3	YLR093C	NYV1

Predictions at threshold level 0.80 continued...			
Gene1 of predicted pair		Gene2 of predicted pair	
Systematic Name	Standard Name	Systematic Name	Standard Name
YHR134W	WSS1	YDR318W	MCM21
YOR023C	AHC1	YJR090C	GRR1
YCR066W	RAD18	YGL115W	SNF4
YHR200W	RPN10	YOR327C	SNC2
YOR195W	SLK19	YLR386W	VAC14
YJL154C	VPS35	YNL079C	TPM1
YDR171W	HSP42	YPR119W	CLB2
YBR057C	MUM2	YLR370C	ARC18
YBR107C	IML3	YLR370C	ARC18
YBR215W	HPC2	YPR046W	MCM16
YGL174W	BUD13	YAL002W	VPS8
YER120W	SCS2	YAL002W	VPS8
YLR320W	MMS22	YOL081W	IRA2
YPR131C	NAT3	YOR080W	DIA2
YFR024C-A	LSB3	YHR114W	BZZ1
YPR051W	MAK3	YGR054W	YGR054W
YLR135W	SLX4	YPL057C	SUR1
YDR137W	RGP1	YLR370C	ARC18
YBL047C	EDE1	YBR082C	UBC4
YGL163C	RAD54	YCR077C	PAT1
YJR135C	MCM22	YKL114C	APN1
YGR129W	SYF2	YPL213W	LEA1
YGL087C	MMS2	YPL018W	CTF19
YEL043W	YEL043W	YDL077C	VAM6
YDL020C	RPN4	YLR337C	VRP1
YNL218W	MGS1	YOL030W	GAS5
YLR386W	VAC14	YJL124C	LSM1
YCR081W	SRB8	YPL256C	CLN2
YDR156W	RPA14	YPL152W	RRD2

Predictions at threshold level 0.80 continued...			
Gene1 of predicted pair		Gene2 of predicted pair	
Systematic Name	Standard Name	Systematic Name	Standard Name
YJR135C	MCM22	YLR032W	RAD5
YDL083C	RPS16B	YBR019C	GAL10
YHR161C	YAP1801	YBL106C	SRO77
YNL087W	TCB2	YBR171W	SEC66
YER116C	SLX8	YJL183W	MNN11
YDR146C	SWI5	YNL199C	GCR2
YMR109W	MYO5	YGL124C	MON1
YDR174W	HMO1	YDR318W	MCM21
YHR200W	RPN10	YPL152W	RRD2
YDR202C	RAV2	YPL234C	TFP3
YOR270C	VPH1	YBR127C	VMA2
YGL163C	RAD54	YNL107W	YAF9
YPR141C	KAR3	YIL033C	BCY1
YDR378C	LSM6	YPL152W	RRD2
YNR072W	HXT17	YNL041C	COG6
YJL176C	SWI3	YDR245W	MNN10
YLR288C	MEC3	YCR066W	RAD18
YMR004W	MVP1	YPR051W	MAK3
YJR040W	GEF1	YLR309C	IMH1
YKR020W	VPS51	YNL127W	FAR11
YDR004W	RAD57	YNL076W	MKS1
YMR246W	FAA4	YKL094W	YJU3
YCR065W	HCM1	YGL003C	CDH1
YBR200W	BEM1	YEL027W	CUP5
YNL041C	COG6	YMR152W	YIM1
YKL135C	APL2	YNR006W	VPS27
YIL034C	CAP2	YDR293C	SSD1
YDR225W	HTA1	YNL031C	HHT2
YOR033C	EXO1	YPR131C	NAT3

Predictions at threshold level 0.80 continued...			
Gene1 of predicted pair		Gene2 of predicted pair	
Systematic Name	Standard Name	Systematic Name	Standard Name
YLR079W	SIC1	YPL246C	RBD2
YKR095W	MLP1	YAL009W	SPO7
YNL238W	KEX2	YEL051W	VMA8
YHR108W	GGA2	YLR425W	TUS1
YOR069W	VPS5	YDL192W	ARF1
YML095C	RAD10	YBR107C	IML3
YGL115W	SNF4	YCR053W	THR4
YIL053W	RHR2	YLR452C	SST2
YPL042C	SSN3	YIR005W	IST3
YLR441C	RPS1A	YGL115W	SNF4
YOR332W	VMA4	YIL002C	INP51
YMR055C	BUB2	YDR171W	HSP42
YOR080W	DIA2	YLR410W	VIP1
YNL218W	MGS1	YLR410W	VIP1
YKR020W	VPS51	YPR173C	VPS4
YLL039C	UBI4	YKL006W	RPL14A
YHR061C	GIC1	YBL085W	BOI1
YLR093C	NYV1	YOL018C	TLG2
YPR131C	NAT3	YGL163C	RAD54
YDR245W	MNN10	YDL226C	GCS1
YLR083C	EMP70	YCR066W	RAD18
YKL110C	KTI12	YFR036W	CDC26
YGR261C	APL6	YPL195W	APL5
YOR127W	RGA1	YEL043W	YEL043W
YPR036W	VMA13	YEL051W	VMA8
YNL215W	IES2	YLR182W	SWI6
YOR270C	VPH1	YHR081W	LRP1
YGL066W	SGF73	YBR200W	BEM1
YJR090C	GRR1	YOR368W	RAD17

Predictions at threshold level 0.80 continued...			
Gene1 of predicted pair		Gene2 of predicted pair	
Systematic Name	Standard Name	Systematic Name	Standard Name
YER144C	UBP5	YLR320W	MMS22
YMR224C	MRE11	YDR295C	HDA2
YKL048C	ELM1	YIL103W	DPH1
YNL166C	BNI5	YJR090C	GRR1
YDR469W	SDC1	YDR318W	MCM21
YDR146C	SWI5	YEL056W	HAT2
YBR173C	UMP1	YER040W	GLN3
YDR156W	RPA14	YNL215W	IES2
YOR195W	SLK19	YDR254W	CHL4
YLR337C	VRP1	YPL018W	CTF19
YGL087C	MMS2	YLR447C	VMA6
YFR010W	UBP6	YOR043W	WHI2
YMR116C	ASC1	YLR313C	SPH1
YLR079W	SIC1	YGR059W	SPR3
YFR010W	UBP6	YPL152W	RRD2
YMR125W	STO1	YFL047W	RGD2
YDR004W	RAD57	YPL001W	HAT1
YJL176C	SWI3	YGL200C	EMP24
YLR288C	MEC3	YLR032W	RAD5
YNL199C	GCR2	YDR207C	UME6
YKR020W	VPS51	YNL238W	KEX2
YHR134W	WSS1	YLR447C	VMA6
YKR099W	BAS1	YDR207C	UME6
YHR119W	SET1	YOL145C	CTR9
YOR066W	MSA1	YPL195W	APL5
YDR443C	SSN2	YGR134W	CAF130
YDR108W	GSG1	YGL054C	ERV14
YGR108W	CLB1	YBR114W	RAD16
YJL060W	BNA3	YPR119W	CLB2

Predictions at threshold level 0.80 continued...			
Gene1 of predicted pair		Gene2 of predicted pair	
Systematic Name	Standard Name	Systematic Name	Standard Name
YDR071C	PAA1	YDR469W	SDC1
YLR386W	VAC14	YAR014C	BUD14
YOR275C	RIM20	YLR371W	ROM2
YPL057C	SUR1	YFR036W	CDC26
YBR171W	SEC66	YLR180W	SAM1
YBL051C	PIN4	YJL095W	BCK1
YKR001C	VPS1	YAL002W	VPS8
YOL110W	SHR5	YLR246W	ERF2
YEL065W	SIT1	YPL195W	APL5
YPL024W	RMI1	YPR046W	MCM16
YMR198W	CIK1	YDR310C	SUM1
YHR060W	VMA22	YNL238W	KEX2
YCL037C	SRO9	YDR363W-A	SEM1
YGR184C	UBR1	YDR076W	RAD55
YPR036W	VMA13	YBR171W	SEC66
YLR452C	SST2	YMR152W	YIM1
YPR051W	MAK3	YDR254W	CHL4
YPR120C	CLB5	YPL018W	CTF19
YDR156W	RPA14	YKL110C	KTI12
YPL178W	CBC2	YMR273C	ZDS1
YMR004W	MVP1	YDR153C	ENT5
YNL325C	FIG4	YEL060C	PRB1
YDR320C	SWA2	YDL137W	ARF2
YER040W	GLN3	YMR230W	RPS10B
YDR261C	EXG2	YJR032W	CPR7
YDR443C	SSN2	YOR106W	VAM3
YLL007C	YLL007C	YBL051C	PIN4
YDR318W	MCM21	YDR183W	PLP1
YOR033C	EXO1	YNR052C	POP2

Predictions at threshold level 0.80 continued...			
Gene1 of predicted pair		Gene2 of predicted pair	
Systematic Name	Standard Name	Systematic Name	Standard Name
YGR019W	UGA1	YOR023C	AHC1
YIL036W	CST6	YGL200C	EMP24
YER139C	YER139C	YLR182W	SWI6
YDL083C	RPS16B	YER139C	YER139C
YKR072C	SIS2	YDR207C	UME6
YIL053W	RHR2	YPL024W	RMI1
YGL163C	RAD54	YGL252C	RTG2
YLR018C	POM34	YPL246C	RBD2
YNL238W	KEX2	YPR046W	MCM16
YML115C	VAN1	YKR020W	VPS51
YMR198W	CIK1	YOR035C	SHE4
YGL174W	BUD13	YNL041C	COG6
YLR119W	SRN2	YLR386W	VAC14
YPL045W	VPS16	YPR028W	YOP1
YBR171W	SEC66	YNL293W	MSB3
YLR320W	MMS22	YLR093C	NYV1
YGL147C	RPL9A	YJR084W	CSN12
YOR109W	INP53	YJL004C	SYS1
YIL034C	CAP2	YJL183W	MNN11
YMR276W	DSK2	YHR108W	GGA2
YBR200W	BEM1	YNL248C	RPA49
YER096W	SHC1	YMR311C	GLC8
YDR363W-A	SEM1	YJL095W	BCK1
YOR043W	WHI2	YEL056W	HAT2
YMR026C	PEX12	YDR265W	PEX10
YNL079C	TPM1	YBL072C	RPS8A
YGL066W	SGF73	YNL236W	SIN4
YOR033C	EXO1	YMR106C	YKU80
YDR363W-A	SEM1	YKL213C	DOA1

Predictions at threshold level 0.80 continued...			
Gene1 of predicted pair		Gene2 of predicted pair	
Systematic Name	Standard Name	Systematic Name	Standard Name
YDR443C	SSN2	YER068W	MOT2
YBL003C	HTA2	YBR009C	HHF1
YER143W	DDI1	YDL047W	SIT4
YJL183W	MNN11	YJL095W	BCK1
YDR245W	MNN10	YDR378C	LSM6
YKR099W	BAS1	YER122C	GLO3
YMR127C	SAS2	YML102W	CAC2
YKL048C	ELM1	YIL038C	NOT3
YDL002C	NHP10	YPL057C	SUR1
YLR079W	SIC1	YKR001C	VPS1
YBR111W-A	SUS1	YLR055C	SPT8
YNL106C	INP52	YJL004C	SYS1
YBL047C	EDE1	YKL101W	HSL1
YLR119W	SRN2	YFR036W	CDC26
YDR383C	NKP1	YIL103W	DPH1
YDR261C	EXG2	YOR327C	SNC2
YER162C	RAD4	YOL121C	RPS19A
YEL065W	SIT1	YDL184C	RPL41A
YLR324W	PEX30	YBR168W	PEX32
YMR202W	ERG2	YMR055C	BUB2
YOL111C	MDY2	YNL076W	MKS1
YNL325C	FIG4	YNL076W	MKS1
YOR080W	DIA2	YDR358W	GGA1
YDL184C	RPL41A	YCR053W	THR4
YFR031C-A	RPL2A	YOR290C	SNF2
YJR099W	YUH1	YNR006W	VPS27
YPL259C	APM1	YAL030W	SNC1
YHR134W	WSS1	YDR289C	RTT103
YLR083C	EMP70	YJL036W	SNX4

Predictions at threshold level 0.80 continued...			
Gene1 of predicted pair		Gene2 of predicted pair	
Systematic Name	Standard Name	Systematic Name	Standard Name
YPL086C	ELP3	YBR089C-A	NHP6B
YMR016C	SOK2	YML071C	COG8
YPL253C	VIK1	YDR207C	UME6
YPL115C	BEM3	YGL151W	NUT1
YMR153W	NUP53	YLR452C	SST2
YMR116C	ASC1	YML028W	TSA1
YLR452C	SST2	YLR182W	SWI6
YNL106C	INP52	YKL139W	CTK1
YBR171W	SEC66	YLR452C	SST2
YLR135W	SLX4	YJL124C	LSM1
YLL022C	HIF1	YEL056W	HAT2
YML028W	TSA1	YGL031C	RPL24A
YML016C	PPZ1	YGL019W	CKB1
YGR270W	YTA7	YML112W	CTK3
YNL288W	CAF40	YGL124C	MON1
YHR152W	SPO12	YGR135W	PRE9
YPR072W	NOT5	YPL203W	TPK2
YOR127W	RGA1	YLR447C	VMA6
YBL102W	SFT2	YOL018C	TLG2
YDL006W	PTC1	YPL259C	APM1
YLR102C	APC9	YBR200W	BEM1
YLR452C	SST2	YJL095W	BCK1
YOR059C	YOR059C	YKR046C	PET10
YAL055W	PEX22	YLR131C	ACE2
YLR410W	VIP1	YOL054W	PSH1
YOR008C	SLG1	YER096W	SHC1
YOR059C	YOR059C	YKL140W	TGL1
YDR293C	SSD1	YDR389W	SAC7
YJL138C	TIF2	YKL101W	HSL1

Predictions at threshold level 0.80 continued...			
Gene1 of predicted pair		Gene2 of predicted pair	
Systematic Name	Standard Name	Systematic Name	Standard Name
YPL042C	SSN3	YLR381W	CTF3
YIL034C	CAP2	YGR019W	UGA1
YDR217C	RAD9	YGL190C	CDC55
YOR368W	RAD17	YNL082W	PMS1
YNL054W	VAC7	YPL018W	CTF19
YJR056C	YJR056C	YKR035W-A	DID2
YLR371W	ROM2	YPL089C	RLM1
YMR312W	ELP6	YHR187W	IKI1
YFR010W	UBP6	YNL041C	COG6
YBR108W	YBR108W	YLR398C	SKI2
YMR198W	CIK1	YMR109W	MYO5
YOR312C	RPL20B	YFR036W	CDC26
YAL024C	LTE1	YBR171W	SEC66
YPR030W	CSR2	YBR280C	SAF1
YBR200W	BEM1	YKL041W	VPS24
YJL053W	PEP8	YER162C	RAD4
YDR244W	PEX5	YDL065C	PEX19
YNL199C	GCR2	YKL160W	ELF1
YLR292C	SEC72	YLR301W	YLR301W
YGL124C	MON1	YDL077C	VAM6
YDR202C	RAV2	YER122C	GLO3
YOL067C	RTG1	YAL002W	VPS8
YKL139W	CTK1	YER122C	GLO3
YBR200W	BEM1	YLR180W	SAM1
YJR060W	CBF1	YIL103W	DPH1
YER114C	BOI2	YNL307C	MCK1
YOR290C	SNF2	YLR410W	VIP1
YFR031C-A	RPL2A	YDL077C	VAM6
YMR039C	SUB1	YLR452C	SST2

Predictions at threshold level 0.80 continued...			
Gene1 of predicted pair		Gene2 of predicted pair	
Systematic Name	Standard Name	Systematic Name	Standard Name
YBL102W	SFT2	YGL115W	SNF4
YDR363W-A	SEM1	YDR440W	DOT1
YIL053W	RHR2	YHR060W	VMA22
YFR031C-A	RPL2A	YGR249W	MGA1
YDR293C	SSD1	YHR111W	UBA4
YNL054W	VAC7	YDR254W	CHL4
YDR385W	EFT2	YAL055W	PEX22
YPL120W	VPS30	YJR033C	RAV1
YAL024C	LTE1	YNL307C	MCK1
YBR107C	IML3	YGL124C	MON1
YMR224C	MRE11	YMR016C	SOK2
YML109W	ZDS2	YNL031C	HHT2
YOL043C	NTG2	YCR066W	RAD18
YHR060W	VMA22	YPR032W	SRO7
YIL076W	SEC28	YKL048C	ELM1
YLR386W	VAC14	YOR358W	HAP5
YJR060W	CBF1	YGL003C	CDH1
YJL004C	SYS1	YDL226C	GCS1
YNL032W	SIW14	YBR019C	GAL10
YBL008W	HIR1	YMR055C	BUB2
YKR072C	SIS2	YOR043W	WHI2
YGL190C	CDC55	YDR422C	SIP1
YEL065W	SIT1	YGL078C	DBP3
YBL088C	TEL1	YIL038C	NOT3
YOR275C	RIM20	YDL047W	SIT4
YOR290C	SNF2	YBR010W	HHT1
YOR023C	AHC1	YPL253C	VIK1
YML001W	YPT7	YNL041C	COG6
YMR224C	MRE11	YIL103W	DPH1

Predictions at threshold level 0.80 continued...			
**Gene1 of predicted pair**		**Gene2 of predicted pair**	
**Systematic Name**	**Standard Name**	**Systematic Name**	**Standard Name**
YJL036W	SNX4	YLR309C	IMH1
YLL007C	YLL007C	YEL056W	HAT2
YBL021C	HAP3	YMR109W	MYO5
YBR057C	MUM2	YBL072C	RPS8A
YBL103C	RTG3	YMR214W	SCJ1
YER144C	UBP5	YPR046W	MCM16
YOR080W	DIA2	YEL013W	VAC8
YPL161C	BEM4	YPR030W	CSR2
YHR013C	ARD1	YIL103W	DPH1
YPR052C	NHP6A	YKL160W	ELF1
YGR249W	MGA1	YJR056C	YJR056C
YAL024C	LTE1	YGL066W	SGF73
YPR036W	VMA13	YLR094C	GIS3
YPR120C	CLB5	YDL047W	SIT4
YJL124C	LSM1	YDR254W	CHL4
YCR084C	TUP1	YNL167C	SKO1
YBL079W	NUP170	YJL187C	SWE1
YHR161C	YAP1801	YDL161W	ENT1
YKL048C	ELM1	YPR046W	MCM16
YAL051W	OAF1	YDR310C	SUM1
YDR245W	MNN10	YLR370C	ARC18
YGR261C	APL6	YDR108W	GSG1
YOL145C	CTR9	YDR071C	PAA1
YOL111C	MDY2	YPL195W	APL5
YMR153W	NUP53	YKL126W	YPK1
YIL076W	SEC28	YDR207C	UME6
YMR199W	CLN1	YLR182W	SWI6
YLR386W	VAC14	YHL002W	HSE1
YBR173C	UMP1	YER162C	RAD4

Predictions at threshold level 0.80 continued...			
Gene1 of predicted pair		Gene2 of predicted pair	
Systematic Name	Standard Name	Systematic Name	Standard Name
YDR443C	SSN2	YDR207C	UME6
YBR200W	BEM1	YKL114C	APN1
YGR104C	SRB5	YDL005C	MED2
YKL139W	CTK1	YDL223C	HBT1
YFR010W	UBP6	YLR398C	SKI2
YLR309C	IMH1	YER122C	GLO3
YLR083C	EMP70	YNL051W	COG5
YNR072W	HXT17	YML071C	COG8
YHR012W	VPS29	YDL006W	PTC1
YOR109W	INP53	YGL115W	SNF4
YMR179W	SPT21	YLR381W	CTF3
YDL192W	ARF1	YDR207C	UME6
YHR200W	RPN10	YER139C	YER139C
YBL052C	SAS3	YBR089C-A	NHP6B
YDR448W	ADA2	YKL109W	HAP4
YOR109W	INP53	YAL030W	SNC1
YLR079W	SIC1	YMR153W	NUP53
YPL096C-A	ERI1	YMR089C	YTA12
YJL138C	TIF2	YCR031C	RPS14A
YKL139W	CTK1	YER139C	YER139C
YNL058C	YNL058C	YNL076W	MKS1
YDL020C	RPN4	YML016C	PPZ1
YMR284W	YKU70	YOL090W	MSH2
YDL155W	CLB3	YGR188C	BUB1
YDR448W	ADA2	YOL148C	SPT20
YGL003C	CDH1	YNL107W	YAF9
YKL025C	PAN3	YKL114C	APN1
YBL085W	BOI1	YER158C	YER158C
YJL124C	LSM1	YJL187C	SWE1

Predictions at threshold level 0.80 continued...			
Gene1 of predicted pair		Gene2 of predicted pair	
Systematic Name	Standard Name	Systematic Name	Standard Name
YBR200W	BEM1	YNL199C	GCR2
YHR152W	SPO12	YDR264C	AKR1
YPR046W	MCM16	YER139C	YER139C
YBR290W	BSD2	YLR360W	VPS38
YNR052C	POP2	YDR439W	LRS4
YOR127W	RGA1	YKL101W	HSL1
YPL096C-A	ERI1	YKL079W	SMY1
YJL029C	VPS53	YPL045W	VPS16
YIL008W	URM1	YEL056W	HAT2
YGR184C	UBR1	YLR219W	MSC3
YLR095C	IOC2	YLR441C	RPS1A
YPL057C	SUR1	YDR363W-A	SEM1
YOR109W	INP53	YPL259C	APM1
YDR156W	RPA14	YCR077C	PAT1
YLR006C	SSK1	YHR111W	UBA4
YMR198W	CIK1	YMR089C	YTA12
YDR245W	MNN10	YBR260C	RGD1
YBR111W-A	SUS1	YGR252W	GCN5
YAL024C	LTE1	YJR135C	MCM22
YGR135W	PRE9	YBL072C	RPS8A
YDR080W	VPS41	YOL018C	TLG2
YJL183W	MNN11	YNL107W	YAF9
YBR200W	BEM1	YPL037C	EGD1
YKL116C	PRR1	YNL087W	TCB2
YLR381W	CTF3	YPR051W	MAK3
YGL003C	CDH1	YOR035C	SHE4
YKL139W	CTK1	YNL041C	COG6
YOR290C	SNF2	YBL025W	RRN10
YFR021W	ATG18	YLR094C	GIS3

Predictions at threshold level 0.80 continued...			
Gene1 of predicted pair		Gene2 of predicted pair	
Systematic Name	Standard Name	Systematic Name	Standard Name
YDL127W	PCL2	YGL161C	YIP5
YDR156W	RPA14	YIL103W	DPH1
YFR031C-A	RPL2A	YOL121C	RPS19A
YKL101W	HSL1	YBL087C	RPL23A
YJL053W	PEP8	YGL115W	SNF4
YPR101W	SNT309	YDR163W	CWC15
YPR018W	RLF2	YDR004W	RAD57
YHR060W	VMA22	YOL018C	TLG2
YOR109W	INP53	YHR060W	VMA22
YOR140W	SFL1	YOR014W	RTS1
YER116C	SLX8	YER162C	RAD4
YDR439W	LRS4	YOL054W	PSH1
YDR293C	SSD1	YDR155C	CPR1
YML117W	NAB6	YPL161C	BEM4
YLR320W	MMS22	YBR036C	CSG2
YFR032C-A	RPL29	YMR038C	CCS1
YOR195W	SLK19	YMR038C	CCS1
YPL120W	VPS30	YDR310C	SUM1
YML062C	MFT1	YMR273C	ZDS1
YJL124C	LSM1	YNL307C	MCK1
YGR270W	YTA7	YHR114W	BZZ1
YAL024C	LTE1	YGL115W	SNF4
YHR108W	GGA2	YMR038C	CCS1
YMR153W	NUP53	YPL195W	APL5
YDR142C	PEX7	YGL205W	POX1
YER044C	ERG28	YNL238W	KEX2
YLR360W	VPS38	YML001W	YPT7
YDR129C	SAC6	YHL002W	HSE1
YGL078C	DBP3	YKL139W	CTK1

Predictions at threshold level 0.80 continued...			
Gene1 of predicted pair		Gene2 of predicted pair	
Systematic Name	Standard Name	Systematic Name	Standard Name
YML001W	YPT7	YHR081W	LRP1
YPR046W	MCM16	YJR090C	GRR1
YIR003W	YIR003W	YMR109W	MYO5
YNL325C	FIG4	YMR230W	RPS10B
YLR410W	VIP1	YGL003C	CDH1
YFR031C-A	RPL2A	YGL003C	CDH1
YJL136C	RPS21B	YGL031C	RPL24A
YLR083C	EMP70	YBL102W	SFT2
YHR134W	WSS1	YDR207C	UME6
YMR231W	PEP5	YDR207C	UME6
YHR073W	OSH3	YKL117W	SBA1
YLR216C	CPR6	YDR318W	MCM21
YBR278W	DPB3	YHR039C-A	VMA10
YDL223C	HBT1	YGL200C	EMP24
YCL037C	SRO9	YGR270W	YTA7
YIL071C	PCI8	YKR026C	GCN3
YMR095C	SNO1	YBL103C	RTG3
YBL003C	HTA2	YKR048C	NAP1
YDR383C	NKP1	YNL107W	YAF9
YDR363W-A	SEM1	YOR368W	RAD17
YJL053W	PEP8	YMR004W	MVP1
YDR378C	LSM6	YLR182W	SWI6
YDL232W	OST4	YJR010C-A	SPC1
YBL008W	HIR1	YJL140W	RPB4
YMR089C	YTA12	YDR207C	UME6
YBR194W	SOY1	YNL107W	YAF9
YKL048C	ELM1	YMR214W	SCJ1
YJL036W	SNX4	YPL234C	TFP3
YHL002W	HSE1	YOL081W	IRA2

Predictions at threshold level 0.80 continued...			
Gene1 of predicted pair		Gene2 of predicted pair	
Systematic Name	Standard Name	Systematic Name	Standard Name
YNL243W	SLA2	YMR224C	MRE11
YLR240W	VPS34	YMR231W	PEP5
YMR224C	MRE11	YNL016W	PUB1
YNL215W	IES2	YOR043W	WHI2
YDR469W	SDC1	YDR440W	DOT1
YLR320W	MMS22	YMR038C	CCS1
YLL007C	YLL007C	YAR014C	BUD14
YGL054C	ERV14	YJR033C	RAV1
YGL003C	CDH1	YEL060C	PRB1
YDR245W	MNN10	YGL163C	RAD54
YJL095W	BCK1	YHR158C	KEL1
YEL009C	GCN4	YCR084C	TUP1
YNL238W	KEX2	YER162C	RAD4
YGR108W	CLB1	YJR135C	MCM22
YPR036W	VMA13	YML071C	COG8
YPR018W	RLF2	YKL101W	HSL1
YDL155W	CLB3	YBR283C	SSH1
YDR293C	SSD1	YMR054W	STV1
YOR195W	SLK19	YBR107C	IML3
YHR156C	LIN1	YFR036W	CDC26
YGL115W	SNF4	YER122C	GLO3
YCR020W-B	HTL1	YPR120C	CLB5
YPL240C	HSP82	YML132W	COS3
YCR066W	RAD18	YJL004C	SYS1
YHR098C	SFB3	YJL168C	SET2
YMR153W	NUP53	YPL256C	CLN2
YDL116W	NUP84	YDR101C	ARX1
YIL159W	BNR1	YER139C	YER139C
YLR337C	VRP1	YBR200W	BEM1

Predictions at threshold level 0.80 continued...			
Gene1 of predicted pair		Gene2 of predicted pair	
Systematic Name	Standard Name	Systematic Name	Standard Name
YDR004W	RAD57	YGR081C	SLX9
YLR119W	SRN2	YER040W	GLN3
YOL051W	GAL11	YGR135W	PRE9
YDR293C	SSD1	YDR335W	MSN5
YML013W	UBX2	YMR022W	UBC7
YOR080W	DIA2	YBR215W	HPC2
YOL072W	THP1	YBR111W-A	SUS1
YBL047C	EDE1	YHR111W	UBA4
YLR095C	IOC2	YIL053W	RHR2
YML001W	YPT7	YER122C	GLO3
YBL079W	NUP170	YDR076W	RAD55
YMR055C	BUB2	YLR182W	SWI6
YML016C	PPZ1	YEL053C	MAK10
YNL068C	FKH2	YHR152W	SPO12
YNL016W	PUB1	YBR082C	UBC4
YGL174W	BUD13	YDR289C	RTT103
YIL053W	RHR2	YHR039C-A	VMA10
YDR202C	RAV2	YNL297C	MON2
YHR064C	SSZ1	YGR285C	ZUO1
YLR315W	NKP2	YJL006C	CTK2
YDR383C	NKP1	YNL215W	IES2
YKL126W	YPK1	YNL058C	YNL058C
YMR172W	HOT1	YHR012W	VPS29
YOR001W	RRP6	YCR086W	CSM1
YMR224C	MRE11	YER162C	RAD4
YMR004W	MVP1	YJR040W	GEF1
YAR014C	BUD14	YNL107W	YAF9
YDR192C	NUP42	YMR273C	ZDS1
YMR198W	CIK1	YKL048C	ELM1

Predictions at threshold level 0.80 continued...			
Gene1 of predicted pair		Gene2 of predicted pair	
Systematic Name	Standard Name	Systematic Name	Standard Name
YNL201C	PSY2	YNL248C	RPA49
YPL024W	RMI1	YBR217W	ATG12
YLR386W	VAC14	YJR032W	CPR7
YLR079W	SIC1	YDR183W	PLP1
YBL103C	RTG3	YFL023W	BUD27
YLR018C	POM34	YER115C	SPR6
YMR127C	SAS2	YJL190C	RPS22A
YHL002W	HSE1	YBR114W	RAD16
YOR069W	VPS5	YAL030W	SNC1
YFR031C-A	RPL2A	YLR192C	HCR1
YLR399C	BDF1	YLR182W	SWI6
YDR320C	SWA2	YGL115W	SNF4
YFR010W	UBP6	YAL002W	VPS8
YKL032C	IXR1	YNL229C	URE2
YGL178W	MPT5	YDR388W	RVS167
YGL178W	MPT5	YHR030C	SLT2
YLR148W	PEP3	YDR323C	PEP7
YKR028W	SAP190	YDL047W	SIT4
YPL096C-A	ERI1	YCR009C	RVS161
YBR200W	BEM1	YEL053C	MAK10
YCR066W	RAD18	YPR119W	CLB2
YPL137C	GIP3	YBL025W	RRN10
YPL042C	SSN3	YJR135C	MCM22
YDL113C	ATG20	YLR191W	PEX13
YAL024C	LTE1	YPL152W	RRD2
YNL079C	TPM1	YPL256C	CLN2
YNL243W	SLA2	YPR029C	APL4
YPR018W	RLF2	YDL116W	NUP84
YLR234W	TOP3	YOR038C	HIR2

Predictions at threshold level 0.80 continued...			
Gene1 of predicted pair		Gene2 of predicted pair	
Systematic Name	Standard Name	Systematic Name	Standard Name
YGR105W	VMA21	YJL004C	SYS1
YDL192W	ARF1	YLR180W	SAM1
YDL160C	DHH1	YMR199W	CLN1
YHR156C	LIN1	YDL223C	HBT1
YKL010C	UFD4	YKL041W	VPS24
YBR194W	SOY1	YAL002W	VPS8
YPL024W	RMI1	YML028W	TSA1
YLR381W	CTF3	YIR005W	IST3
YJR063W	RPA12	YIL103W	DPH1
YPR046W	MCM16	YDR076W	RAD55
YLR079W	SIC1	YDR318W	MCM21
YOR069W	VPS5	YEL042W	GDA1
YDR264C	AKR1	YGL031C	RPL24A
YHR134W	WSS1	YMR284W	YKU70
YBR200W	BEM1	YKR020W	VPS51
YPR072W	NOT5	YNL030W	HHF2
YLR234W	TOP3	YPL253C	VIK1
YOR270C	VPH1	YEL053C	MAK10
YPR036W	VMA13	YEL060C	PRB1
YLR410W	VIP1	YDL226C	GCS1
YBL051C	PIN4	YJR090C	GRR1
YKR020W	VPS51	YAL002W	VPS8
YLR119W	SRN2	YLR441C	RPS1A
YPL050C	MNN9	YGR135W	PRE9
YGR108W	CLB1	YPL161C	BEM4
YDR155C	CPR1	YGR188C	BUB1
YNL076W	MKS1	YGL252C	RTG2
YDR129C	SAC6	YOR008C	SLG1
YGL223C	COG1	YKR020W	VPS51

Predictions at threshold level 0.80 continued...			
Gene1 of predicted pair		Gene2 of predicted pair	
Systematic Name	Standard Name	Systematic Name	Standard Name
YBL051C	PIN4	YEL053C	MAK10
YOR035C	SHE4	YHR114W	BZZ1
YBR173C	UMP1	YDL192W	ARF1
YFR010W	UBP6	YPR036W	VMA13
YIL053W	RHR2	YDL223C	HBT1
YDR363W-A	SEM1	YOR038C	HIR2
YMR055C	BUB2	YHR039C-A	VMA10
YHR150W	PEX28	YBR168W	PEX32
YOR275C	RIM20	YDR264C	AKR1
YDR329C	PEX3	YGL153W	PEX14
YLR452C	SST2	YLR093C	NYV1
YDR225W	HTA1	YFR036W	CDC26
YBR200W	BEM1	YJL117W	PHO86
YLR079W	SIC1	YGL019W	CKB1
YOR132W	VPS17	YPL195W	APL5
YKL025C	PAN3	YNL288W	CAF40
YKR001C	VPS1	YEL043W	YEL043W
YHL031C	GOS1	YML071C	COG8
YOR083W	WHI5	YGR104C	SRB5
YOR001W	RRP6	YDL155W	CLB3
YBR127C	VMA2	YBL047C	EDE1
YKR001C	VPS1	YJL095W	BCK1
YLR381W	CTF3	YDR183W	PLP1
YCR077C	PAT1	YLR182W	SWI6
YBR194W	SOY1	YDR289C	RTT103
YNL107W	YAF9	YPR119W	CLB2
YLR192C	HCR1	YLR452C	SST2
YOR132W	VPS17	YOR036W	PEP12
YLR093C	NYV1	YJR125C	ENT3

Predictions at threshold level 0.80 continued...			
Gene1 of predicted pair		Gene2 of predicted pair	
Systematic Name	Standard Name	Systematic Name	Standard Name
YOR337W	TEA1	YKL116C	PRR1
YDR183W	PLP1	YJL095W	BCK1
YOR080W	DIA2	YJL006C	CTK2
YPL161C	BEM4	YOL108C	INO4
YKL048C	ELM1	YLR315W	NKP2
YNL307C	MCK1	YMR311C	GLC8
YJL154C	VPS35	YOL108C	INO4
YMR055C	BUB2	YDL226C	GCS1
YKR020W	VPS51	YMR022W	UBC7
YHR111W	UBA4	YKR020W	VPS51
YJR063W	RPA12	YNL030W	HHF2
YER044C	ERG28	YML001W	YPT7
YOR132W	VPS17	YOR035C	SHE4
YML001W	YPT7	YDL077C	VAM6
YMR055C	BUB2	YFR030W	MET10
YDR477W	SNF1	YER049W	TPA1
YGR054W	YGR054W	YJL190C	RPS22A
YPL115C	BEM3	YLR292C	SEC72
YJR056C	YJR056C	YER044C	ERG28
YLR083C	EMP70	YER162C	RAD4
YGR270W	YTA7	YJL095W	BCK1
YLR352W	YLR352W	YDR207C	UME6
YLR337C	VRP1	YGR188C	BUB1
YDL083C	RPS16B	YBR280C	SAF1
YHR073W	OSH3	YBL079W	NUP170
YBR200W	BEM1	YLR370C	ARC18
YGL174W	BUD13	YGR071C	YGR071C
YLR119W	SRN2	YLR192C	HCR1
YFR031C-A	RPL2A	YNL325C	FIG4

Predictions at threshold level 0.80 continued...			
Gene1 of predicted pair		Gene2 of predicted pair	
Systematic Name	Standard Name	Systematic Name	Standard Name
YMR095C	SNO1	YLR119W	SRN2
YMR016C	SOK2	YDR137W	RGP1
YPL115C	BEM3	YDR254W	CHL4
YNR058W	BIO3	YDL149W	ATG9
YCR065W	HCM1	YIR009W	MSL1
YBL078C	ATG8	YML001W	YPT7
YNR023W	SNF12	YDR073W	SNF11
YDR289C	RTT103	YEL013W	VAC8
YDR318W	MCM21	YMR038C	CCS1
YEL065W	SIT1	YOR035C	SHE4
YIL034C	CAP2	YDL226C	GCS1
YKR099W	BAS1	YBL025W	RRN10
YNL307C	MCK1	YLR182W	SWI6
YLR292C	SEC72	YBR114W	RAD16
YBL067C	UBP13	YDR207C	UME6
YNL054W	VAC7	YPL253C	VIK1
YLR441C	RPS1A	YLR180W	SAM1
YJL183W	MNN11	YLR180W	SAM1
YLR386W	VAC14	YPR036W	VMA13
YKL010C	UFD4	YBL072C	RPS8A
YLR399C	BDF1	YMR055C	BUB2
YNL248C	RPA49	YNL107W	YAF9
YAL024C	LTE1	YNL041C	COG6
YMR224C	MRE11	YLR370C	ARC18
YPL227C	ALG5	YMR214W	SCJ1
YLR234W	TOP3	YBR171W	SEC66
YIL159W	BNR1	YFL023W	BUD27
YML095C	RAD10	YNL229C	URE2
YDR174W	HMO1	YLR410W	VIP1

Predictions at threshold level 0.80 continued...			
Gene1 of predicted pair		Gene2 of predicted pair	
Systematic Name	Standard Name	Systematic Name	Standard Name
YPL256C	CLN2	YEL036C	ANP1
YFR031C-A	RPL2A	YBL103C	RTG3
YBL021C	HAP3	YDR155C	CPR1
YDL185W	TFP1	YEL051W	VMA8
YDR320C	SWA2	YGL054C	ERV14
YPR036W	VMA13	YGL212W	VAM7
YIL034C	CAP2	YHR178W	STB5
YKL007W	CAP1	YNL293W	MSB3
YJR032W	CPR7	YPL195W	APL5
YMR202W	ERG2	YPL018W	CTF19
YFR034C	PHO4	YDR363W-A	SEM1
YMR026C	PEX12	YJR032W	CPR7
YPL120W	VPS30	YOL018C	TLG2
YDR320C	SWA2	YJL004C	SYS1
YDL042C	SIR2	YML109W	ZDS2
YLL007C	YLL007C	YJL095W	BCK1
YJR135C	MCM22	YOR141C	ARP8
YKL117W	SBA1	YGR059W	SPR3
YHR200W	RPN10	YPL018W	CTF19
YKL025C	PAN3	YHL002W	HSE1
YCR086W	CSM1	YAR014C	BUD14
YGL078C	DBP3	YNL076W	MKS1
YPL018W	CTF19	YNL116W	DMA2
YKL116C	PRR1	YLR320W	MMS22
YPR084W	YPR084W	YOR327C	SNC2
YDL160C	DHH1	YLR079W	SIC1
YGL054C	ERV14	YDL006W	PTC1
YDR097C	MSH6	YFR010W	UBP6
YDR439W	LRS4	YPR120C	CLB5

Predictions at threshold level 0.80 continued...			
Gene1 of predicted pair		Gene2 of predicted pair	
Systematic Name	Standard Name	Systematic Name	Standard Name
YMR133W	REC114	YOL121C	RPS19A
YML117W	NAB6	YJL183W	MNN11
YOR069W	VPS5	YDR358W	GGA1
YMR095C	SNO1	YNL307C	MCK1
YER110C	KAP123	YGL151W	NUT1
YPR036W	VMA13	YMR230W	RPS10B
YOR195W	SLK19	YKR057W	RPS21A
YBR290W	BSD2	YMR109W	MYO5
YPR120C	CLB5	YDR310C	SUM1
YGL033W	HOP2	YOL111C	MDY2
YBL103C	RTG3	YNR072W	HXT17
YEL053C	MAK10	YER122C	GLO3
YLR447C	VMA6	YDR155C	CPR1
YER144C	UBP5	YNL058C	YNL058C
YCR077C	PAT1	YNL147W	LSM7
YBR171W	SEC66	YPL195W	APL5
YLR386W	VAC14	YMR214W	SCJ1
YHR205W	SCH9	YMR311C	GLC8
YPL057C	SUR1	YBR107C	IML3
YDR443C	SSN2	YLR095C	IOC2
YIL159W	BNR1	YPR046W	MCM16
YDR358W	GGA1	YMR152W	YIM1
YGL133W	ITC1	YDL047W	SIT4
YMR231W	PEP5	YLR352W	YLR352W
YGR249W	MGA1	YKL116C	PRR1
YPR029C	APL4	YHR108W	GGA2
YMR022W	UBC7	YDL047W	SIT4
YOR109W	INP53	YAL002W	VPS8
YDR174W	HMO1	YOR141C	ARP8

Predictions at threshold level 0.80 continued...			
Gene1 of predicted pair		Gene2 of predicted pair	
Systematic Name	Standard Name	Systematic Name	Standard Name
YDL154W	MSH5	YHR079C-A	SAE3
YHL020C	OPI1	YKR030W	GMH1
YOR290C	SNF2	YLR306W	UBC12
YDR443C	SSN2	YLR398C	SKI2
YOR033C	EXO1	YJL187C	SWE1
YNL199C	GCR2	YDR440W	DOT1
YML016C	PPZ1	YLR410W	VIP1
YDR108W	GSG1	YNL127W	FAR11
YBR112C	CYC8	YOR140W	SFL1
YER144C	UBP5	YLR441C	RPS1A
YMR116C	ASC1	YKL048C	ELM1
YDL020C	RPN4	YDR137W	RGP1
YCR077C	PAT1	YLR370C	ARC18
YDR004W	RAD57	YML103C	NUP188
YNL248C	RPA49	YJR063W	RPA12
YOR290C	SNF2	YLR093C	NYV1
YOR195W	SLK19	YJL136C	RPS21B
YMR172W	HOT1	YDR293C	SSD1
YDR137W	RGP1	YER122C	GLO3
YIL159W	BNR1	YDL083C	RPS16B
YGR019W	UGA1	YOL067C	RTG1
YHR134W	WSS1	YDL013W	SLX5
YPL234C	TFP3	YPL120W	VPS30
YPL161C	BEM4	YGR054W	YGR054W
YKR048C	NAP1	YDL047W	SIT4
YOR132W	VPS17	YHR114W	BZZ1
YLR119W	SRN2	YEL053C	MAK10
YPL018W	CTF19	YAR014C	BUD14
YPL161C	BEM4	YBR036C	CSG2

Predictions at threshold level 0.80 continued...			
Gene1 of predicted pair		Gene2 of predicted pair	
Systematic Name	Standard Name	Systematic Name	Standard Name
YAL010C	MDM10	YHR115C	DMA1
YLR452C	SST2	YPL137C	GIP3
YLR192C	HCR1	YDR358W	GGA1
YDL042C	SIR2	YDR440W	DOT1
YGR072W	UPF3	YER162C	RAD4
YAL024C	LTE1	YJL095W	BCK1
YGL115W	SNF4	YAL002W	VPS8
YJL136C	RPS21B	YBL047C	EDE1
YNL215W	IES2	YLR410W	VIP1
YLL039C	UBI4	YKL032C	IXR1
YGL054C	ERV14	YAR014C	BUD14
YOL111C	MDY2	YOR327C	SNC2
YHR178W	STB5	YER139C	YER139C
YDR202C	RAV2	YJR033C	RAV1
YJL127C	SPT10	YDL226C	GCS1
YNL229C	URE2	YGL163C	RAD54
YDR202C	RAV2	YMR054W	STV1
YER173W	RAD24	YGL100W	SEH1
YNL106C	INP52	YDR378C	LSM6
YOR270C	VPH1	YGL025C	PGD1
YGR097W	ASK10	YNL025C	SSN8
YDR289C	RTT103	YOR141C	ARP8
YDL077C	VAM6	YNL127W	FAR11
YDL020C	RPN4	YJR140C	HIR3
YLR079W	SIC1	YLR425W	TUS1
YPR120C	CLB5	YLR254C	NDL1
YBR173C	UMP1	YMR214W	SCJ1
YCL067C	HMLALPHA2	YBR112C	CYC8
YDR318W	MCM21	YLR370C	ARC18

Predictions at threshold level 0.80 continued...			
Gene1 of predicted pair		Gene2 of predicted pair	
Systematic Name	Standard Name	Systematic Name	Standard Name
YPL024W	RMI1	YNL032W	SIW14
YOR069W	VPS5	YER162C	RAD4
YDR137W	RGP1	YBR171W	SEC66
YDR174W	HMO1	YBR228W	SLX1
YKL126W	YPK1	YNL238W	KEX2
YMR055C	BUB2	YJR084W	CSN12
YKL007W	CAP1	YIL034C	CAP2
YHL022C	SPO11	YGL213C	SKI8
YPR046W	MCM16	YDR318W	MCM21
YHR156C	LIN1	YJL172W	CPS1
YDR443C	SSN2	YLR094C	GIS3
YDL184C	RPL41A	YML001W	YPT7
YFR010W	UBP6	YBR108W	YBR108W
YDR443C	SSN2	YDL223C	HBT1
YBR217W	ATG12	YPL037C	EGD1
YBR131W	CCZ1	YJL095W	BCK1
YGL237C	HAP2	YCR086W	CSM1
YER044C	ERG28	YPR046W	MCM16
YPL161C	BEM4	YMR054W	STV1
YPL024W	RMI1	YPR120C	CLB5
YLR337C	VRP1	YDR440W	DOT1
YDR477W	SNF1	YMR133W	REC114
YHR156C	LIN1	YNL076W	MKS1
YLR234W	TOP3	YNL291C	MID1
YMR142C	RPL13B	YLR371W	ROM2
YPL105C	YPL105C	YER122C	GLO3
YCR020C-A	MAK31	YOR327C	SNC2
YBL103C	RTG3	YJR084W	CSN12
YIL076W	SEC28	YOR327C	SNC2

Predictions at threshold level 0.80 continued...			
Gene1 of predicted pair		Gene2 of predicted pair	
Systematic Name	Standard Name	Systematic Name	Standard Name
YLL039C	UBI4	YJR125C	ENT3
YLR093C	NYV1	YBR019C	GAL10
YDR174W	HMO1	YDR378C	LSM6
YPR017C	DSS4	YHL031C	GOS1
YGL250W	RMR1	YGR188C	BUB1
YCL032W	STE50	YER118C	SHO1
YOR001W	RRP6	YBR009C	HHF1
YJL036W	SNX4	YEL051W	VMA8
YHR073W	OSH3	YGR059W	SPR3
YNL201C	PSY2	YML103C	NUP188
YGL223C	COG1	YGL200C	EMP24
YBR200W	BEM1	YDL223C	HBT1
YLR170C	APS1	YAL030W	SNC1
YOR080W	DIA2	YOL030W	GAS5
YMR109W	MYO5	YER122C	GLO3
YPL195W	APL5	YLR370C	ARC18
YLR292C	SEC72	YBR200W	BEM1
YML102W	CAC2	YDR076W	RAD55
YPR131C	NAT3	YKL068W	NUP100
YKL048C	ELM1	YDR318W	MCM21
YLR090W	XDJ1	YIL076W	SEC28
YLR386W	VAC14	YGR270W	YTA7
YGR105W	VMA21	YML071C	COG8
YLR192C	HCR1	YDR129C	SAC6
YPL045W	VPS16	YGL124C	MON1
YPR051W	MAK3	YEL053C	MAK10
YHR111W	UBA4	YLR182W	SWI6
YDR477W	SNF1	YER116C	SLX8
YBL058W	SHP1	YDR484W	VPS52

Predictions at threshold level 0.80 continued...			
Gene1 of predicted pair		Gene2 of predicted pair	
Systematic Name	Standard Name	Systematic Name	Standard Name
YJL183W	MNN11	YKL080W	VMA5
YDL116W	NUP84	YER115C	SPR6
YFL023W	BUD27	YMR133W	REC114
YJR140C	HIR3	YOL081W	IRA2
YMR153W	NUP53	YMR133W	REC114
YLR381W	CTF3	YML095C	RAD10
YJL172W	CPS1	YDR137W	RGP1
YLR083C	EMP70	YPR032W	SRO7
YER044C	ERG28	YMR230W	RPS10B
YLR192C	HCR1	YOR290C	SNF2
YJL004C	SYS1	YER122C	GLO3
YOR304W	ISW2	YMR139W	RIM11
YML013W	UBX2	YGR188C	BUB1
YDR289C	RTT103	YPL018W	CTF19
YGR270W	YTA7	YDR183W	PLP1
YJL053W	PEP8	YLR309C	IMH1
YHR134W	WSS1	YPR051W	MAK3
YDR363W-A	SEM1	YCR077C	PAT1
YOR290C	SNF2	YMR133W	REC114
YPL161C	BEM4	YDR183W	PLP1
YOR043W	WHI2	YLR180W	SAM1
YGL135W	RPL1B	YOR080W	DIA2
YNL087W	TCB2	YMR133W	REC114
YFR010W	UBP6	YDL192W	ARF1
YBL101C	ECM21	YOL081W	IRA2
YBR041W	FAT1	YJL052W	TDH1
YOR358W	HAP5	YDR289C	RTT103
YOR290C	SNF2	YDL083C	RPS16B
YMR231W	PEP5	YHR010W	RPL27A

Predictions at threshold level 0.80 continued...			
Gene1 of predicted pair		Gene2 of predicted pair	
Systematic Name	Standard Name	Systematic Name	Standard Name
YBR173C	UMP1	YMR055C	BUB2
YCR063W	BUD31	YPR101W	SNT309
YLR119W	SRN2	YDL223C	HBT1
YGR072W	UPF3	YGL212W	VAM7
YJL036W	SNX4	YNR032W	PPG1
YBR108W	YBR108W	YJR090C	GRR1
YPR018W	RLF2	YDR254W	CHL4
YML117W	NAB6	YLR371W	ROM2
YNL243W	SLA2	YNL094W	APP1
YML115C	VAN1	YOR270C	VPH1
YML103C	NUP188	YDR289C	RTT103
YLR090W	XDJ1	YPL024W	RMI1
YDR076W	RAD55	YDR207C	UME6
YDR293C	SSD1	YNL293W	MSB3
YPR189W	SKI3	YGL213C	SKI8
YIR002C	MPH1	YPR119W	CLB2
YDL155W	CLB3	YNL107W	YAF9
YBL058W	SHP1	YFR010W	UBP6
YPL253C	VIK1	YML028W	TSA1
YBR030W	YBR030W	YGL003C	CDH1
YDR137W	RGP1	YNL044W	YIP3
YMR224C	MRE11	YNL206C	RTT106
YBR019C	GAL10	YDL192W	ARF1
YPL042C	SSN3	YGL003C	CDH1
YGL190C	CDC55	YML094W	GIM5
YHR098C	SFB3	YLR039C	RIC1
YGL174W	BUD13	YEL013W	VAC8
YIL076W	SEC28	YML071C	COG8
YBR131W	CCZ1	YLR371W	ROM2

Predictions at threshold level 0.80 continued...			
Gene1 of predicted pair		Gene2 of predicted pair	
Systematic Name	Standard Name	Systematic Name	Standard Name
YHL031C	GOS1	YML038C	YMD8
YDR507C	GIN4	YDR422C	SIP1
YDR195W	REF2	YAR014C	BUD14
YBL047C	EDE1	YEL036C	ANP1
YMR039C	SUB1	YHR178W	STB5
YNL238W	KEX2	YKL119C	VPH2
YDR245W	MNN10	YPL158C	YPL158C
YDR358W	GGA1	YER122C	GLO3
YDR261C	EXG2	YBR036C	CSG2
YLR095C	IOC2	YLR371W	ROM2
YMR216C	SKY1	YER162C	RAD4
YLR090W	XDJ1	YBL025W	RRN10
YGR261C	APL6	YDR153C	ENT5
YDL160C	DHH1	YER027C	GAL83
YJL036W	SNX4	YOL018C	TLG2
YPL105C	YPL105C	YER162C	RAD4
YPL161C	BEM4	YIL008W	URM1
YGL087C	MMS2	YDR289C	RTT103
YLR119W	SRN2	YMR224C	MRE11
YJL030W	MAD2	YIL033C	BCY1
YAL024C	LTE1	YDR289C	RTT103
YLR083C	EMP70	YDL006W	PTC1
YHR111W	UBA4	YDR318W	MCM21
YLR441C	RPS1A	YLR398C	SKI2
YER087C-B	SBH1	YNL044W	YIP3
YAL024C	LTE1	YDR156W	RPA14
YBL047C	EDE1	YKR020W	VPS51
YPL024W	RMI1	YKL101W	HSL1
YOR127W	RGA1	YOR327C	SNC2

Predictions at threshold level 0.80 continued...			
Gene1 of predicted pair		Gene2 of predicted pair	
Systematic Name	Standard Name	Systematic Name	Standard Name
YBR217W	ATG12	YFL023W	BUD27
YOR127W	RGA1	YJR040W	GEF1
YOL126C	MDH2	YER110C	KAP123
YNR052C	POP2	YML028W	TSA1
YIL159W	BNR1	YPL195W	APL5
YNL243W	SLA2	YOR032C	HMS1
YDL184C	RPL41A	YNR072W	HXT17
YLR095C	IOC2	YHR200W	RPN10
YNL218W	MGS1	YNL106C	INP52
YMR039C	SUB1	YLR234W	TOP3
YOR195W	SLK19	YPL253C	VIK1
YAL024C	LTE1	YOR327C	SNC2
YDR293C	SSD1	YGR072W	UPF3
YEL050C	RML2	YPR120C	CLB5
YPL161C	BEM4	YDR207C	UME6
YPR051W	MAK3	YDL155W	CLB3
YKL129C	MYO3	YBR260C	RGD1
YOR141C	ARP8	YOL091W	SPO21
YOR035C	SHE4	YJL095W	BCK1
YJR140C	HIR3	YPR120C	CLB5
YMR026C	PEX12	YMR152W	YIM1
YJR135C	MCM22	YNL107W	YAF9
YMR172W	HOT1	YMR109W	MYO5
YBR173C	UMP1	YPR119W	CLB2
YOR275C	RIM20	YBR082C	UBC4
YBL021C	HAP3	YDR289C	RTT103
YGL033W	HOP2	YDL083C	RPS16B
YBR108W	YBR108W	YBL025W	RRN10
YHR111W	UBA4	YJL183W	MNN11

Predictions at threshold level 0.80 continued...			
Gene1 of predicted pair		Gene2 of predicted pair	
Systematic Name	Standard Name	Systematic Name	Standard Name
YNL199C	GCR2	YLR371W	ROM2
YOR080W	DIA2	YDR245W	MNN10
YDR183W	PLP1	YAL002W	VPS8
YKL032C	IXR1	YNL079C	TPM1
YLR234W	TOP3	YJL183W	MNN11
YOR033C	EXO1	YGL163C	RAD54
YFR031C-A	RPL2A	YKL116C	PRR1
YNL031C	HHT2	YJL140W	RPB4
YBR173C	UMP1	YPL037C	EGD1
YOR308C	SNU66	YER122C	GLO3
YPR017C	DSS4	YPR032W	SRO7
YHR081W	LRP1	YMR311C	GLC8
YLR399C	BDF1	YBR010W	HHT1
YDR289C	RTT103	YBR114W	RAD16
YGL147C	RPL9A	YJL183W	MNN11
YJL124C	LSM1	YDR335W	MSN5
YHL031C	GOS1	YJR033C	RAV1
YOR375C	GDH1	YPL161C	BEM4
YFR031C-A	RPL2A	YEL060C	PRB1
YPL001W	HAT1	YDR310C	SUM1
YKL007W	CAP1	YPR119W	CLB2
YKR014C	YPT52	YML097C	VPS9
YPL022W	RAD1	YJR005W	APL1
YMR004W	MVP1	YHR039C-A	VMA10
YDR443C	SSN2	YGL147C	RPL9A
YGR270W	YTA7	YGL163C	RAD54
YNL236W	SIN4	YJL006C	CTK2
YLR061W	RPL22A	YMR198W	CIK1
YLR447C	VMA6	YHR111W	UBA4

Predictions at threshold level 0.80 continued...			
Gene1 of predicted pair		Gene2 of predicted pair	
Systematic Name	Standard Name	Systematic Name	Standard Name
YFR031C-A	RPL2A	YGL147C	RPL9A
YER115C	SPR6	YKR024C	DBP7
YGL115W	SNF4	YPL234C	TFP3
YPL161C	BEM4	YBL025W	RRN10
YPR051W	MAK3	YOL108C	INO4
YJR063W	RPA12	YGR270W	YTA7
YDL160C	DHH1	YGL003C	CDH1
YHR156C	LIN1	YER144C	UBP5
YFR031C-A	RPL2A	YPL161C	BEM4
YKL116C	PRR1	YDR484W	VPS52
YKR072C	SIS2	YML095C	RAD10
YMR095C	SNO1	YOR043W	WHI2
YJL124C	LSM1	YNL225C	CNM67
YNL058C	YNL058C	YBR019C	GAL10
YKL110C	KTI12	YDR183W	PLP1
YKL048C	ELM1	YOR327C	SNC2
YBR131W	CCZ1	YJL183W	MNN11
YBL087C	RPL23A	YDL047W	SIT4
YNL206C	RTT106	YBR009C	HHF1
YOR039W	CKB2	YMR167W	MLH1
YNL218W	MGS1	YDL101C	DUN1
YMR016C	SOK2	YOL111C	MDY2
YHR010W	RPL27A	YMR038C	CCS1
YNL058C	YNL058C	YEL053C	MAK10
YIL036W	CST6	YLR398C	SKI2
YGR040W	KSS1	YBL016W	FUS3
YOR080W	DIA2	YCL037C	SRO9
YDR146C	SWI5	YPL256C	CLN2
YHL020C	OPI1	YGL054C	ERV14

Predictions at threshold level 0.80 continued...			
Gene1 of predicted pair		Gene2 of predicted pair	
Systematic Name	Standard Name	Systematic Name	Standard Name
YPR051W	MAK3	YER162C	RAD4
YGL178W	MPT5	YML041C	VPS71
YMR224C	MRE11	YDR358W	GGA1
YOR035C	SHE4	YER040W	GLN3
YOR043W	WHI2	YHR081W	LRP1
YOR337W	TEA1	YOR290C	SNF2
YPL047W	SGF11	YER107C	GLE2
YGR270W	YTA7	YOL067C	RTG1
YOR290C	SNF2	YEL053C	MAK10
YOL006C	TOP1	YGL095C	VPS45
YLR396C	VPS33	YDR323C	PEP7
YKL025C	PAN3	YDL020C	RPN4
YNR072W	HXT17	YPL161C	BEM4
YGL087C	MMS2	YGR054W	YGR054W
YKL025C	PAN3	YOL067C	RTG1
YCR020W-B	HTL1	YGR275W	RTT102
YKR059W	TIF1	YJL138C	TIF2
YDL116W	NUP84	YIL103W	DPH1
YPR017C	DSS4	YNL051W	COG5
YDR448W	ADA2	YOR038C	HIR2
YIL053W	RHR2	YBR108W	YBR108W
YJR060W	CBF1	YGR135W	PRE9
YMR127C	SAS2	YGR054W	YGR054W
YNL215W	IES2	YBR195C	MSI1
YIL076W	SEC28	YJR040W	GEF1
YOR132W	VPS17	YKL119C	VPH2
YPR084W	YPR084W	YJR040W	GEF1
YPR131C	NAT3	YDL059C	RAD59
YOL145C	CTR9	YNL031C	HHT2

Predictions at threshold level 0.80 continued...			
Gene1 of predicted pair		Gene2 of predicted pair	
Systematic Name	Standard Name	Systematic Name	Standard Name
YDL002C	NHP10	YDR310C	SUM1
YAL024C	LTE1	YBR108W	YBR108W
YDR395W	SXM1	YNL069C	RPL16B
YJR056C	YJR056C	YMR230W	RPS10B
YMR026C	PEX12	YGL078C	DBP3
YJL036W	SNX4	YAR014C	BUD14
YMR116C	ASC1	YPL213W	LEA1
YBR107C	IML3	YLR315W	NKP2
YFL023W	BUD27	YER162C	RAD4
YAL024C	LTE1	YPL001W	HAT1
YEL053C	MAK10	YOL121C	RPS19A
YOR337W	TEA1	YER139C	YER139C
YNL032W	SIW14	YPL037C	EGD1
YBR181C	RPS6B	YAL002W	VPS8
YMR172W	HOT1	YBL051C	PIN4
YDR484W	VPS52	YML071C	COG8
YHR060W	VMA22	YHL020C	OPI1
YBR260C	RGD1	YHR012W	VPS29
YKL160W	ELF1	YER122C	GLO3
YNL206C	RTT106	YNL116W	DMA2
YDR443C	SSN2	YLR079W	SIC1
YBR278W	DPB3	YDR310C	SUM1
YBR030W	YBR030W	YGL115W	SNF4
YBR228W	SLX1	YLR135W	SLX4
YPL018W	CTF19	YML112W	CTK3
YDL042C	SIR2	YNL206C	RTT106
YDL223C	HBT1	YBL067C	UBP13
YDR293C	SSD1	YPR032W	SRO7
YKL139W	CTK1	YOR195W	SLK19

Predictions at threshold level 0.80 continued...			
Gene1 of predicted pair		Gene2 of predicted pair	
Systematic Name	Standard Name	Systematic Name	Standard Name
YFR010W	UBP6	YNL058C	YNL058C
YHL031C	GOS1	YPL105C	YPL105C
YNL288W	CAF40	YLR233C	EST1
YNR072W	HXT17	YLR094C	GIS3
YPR036W	VMA13	YOR043W	WHI2
YJR135C	MCM22	YJR005W	APL1
YOR195W	SLK19	YPL022W	RAD1
YNL243W	SLA2	YLR268W	SEC22
YDR137W	RGP1	YMR152W	YIM1
YMR172W	HOT1	YEL053C	MAK10
YHR060W	VMA22	YOL108C	INO4
YKL139W	CTK1	YLR370C	ARC18
YLR386W	VAC14	YBR280C	SAF1
YNL116W	DMA2	YNL225C	CNM67
YIL034C	CAP2	YJL154C	VPS35
YPL042C	SSN3	YGL115W	SNF4
YFR031C-A	RPL2A	YDR137W	RGP1
YOR043W	WHI2	YJR032W	CPR7
YLR371W	ROM2	YLR398C	SKI2
YNL180C	RHO5	YFL047W	RGD2
YGL237C	HAP2	YGL253W	HXK2
YER087C-B	SBH1	YLR292C	SEC72
YML095C	RAD10	YDR155C	CPR1
YPL240C	HSP82	YER102W	RPS8B
YLR234W	TOP3	YDR310C	SUM1
YJR060W	CBF1	YNL051W	COG5
YNL087W	TCB2	YBL103C	RTG3
YNL025C	SSN8	YEL013W	VAC8
YGR261C	APL6	YBL102W	SFT2

Predictions at threshold level 0.80 continued...			
Gene1 of predicted pair		Gene2 of predicted pair	
Systematic Name	Standard Name	Systematic Name	Standard Name
YPL161C	BEM4	YML001W	YPT7
YDR293C	SSD1	YGL252C	RTG2
YLR288C	MEC3	YOR038C	HIR2
YBL088C	TEL1	YLR079W	SIC1
YGL006W	PMC1	YLR043C	TRX1
YGL115W	SNF4	YER162C	RAD4
YFL013C	IES1	YPR119W	CLB2
YGR217W	CCH1	YJL183W	MNN11
YJR140C	HIR3	YHR039C-A	VMA10
YNL025C	SSN8	YFL013C	IES1
YNL238W	KEX2	YLR371W	ROM2
YML103C	NUP188	YNR052C	POP2
YGR019W	UGA1	YDL155W	CLB3
YJL127C	SPT10	YDL155W	CLB3
YMR080C	NAM7	YOR243C	PUS7
YLR234W	TOP3	YJR032W	CPR7
YBL047C	EDE1	YCR077C	PAT1
YLR337C	VRP1	YBL101C	ECM21
YDR456W	NHX1	YPL161C	BEM4
YDR245W	MNN10	YHR200W	RPN10
YMR142C	RPL13B	YEL053C	MAK10
YJL124C	LSM1	YEL013W	VAC8
YLR095C	IOC2	YKL126W	YPK1
YDL160C	DHH1	YPR120C	CLB5
YNL215W	IES2	YML103C	NUP188
YLR381W	CTF3	YJR090C	GRR1
YJR135C	MCM22	YDR310C	SUM1
YOR033C	EXO1	YNL016W	PUB1
YKR020W	VPS51	YPL120W	VPS30

Predictions at threshold level 0.80 continued...			
Gene1 of predicted pair		Gene2 of predicted pair	
Systematic Name	Standard Name	Systematic Name	Standard Name
YPR036W	VMA13	YLR180W	SAM1
YNL069C	RPL16B	YNL127W	FAR11
YJR040W	GEF1	YGL054C	ERV14
YBR289W	SNF5	YBR009C	HHF1
YDR484W	VPS52	YER162C	RAD4
YML016C	PPZ1	YFR036W	CDC26
YDR225W	HTA1	YPL253C	VIK1
YDR004W	RAD57	YPR046W	MCM16
YOR312C	RPL20B	YOR014W	RTS1
YFL026W	STE2	YDR358W	GGA1
YJL136C	RPS21B	YDR264C	AKR1
YDR202C	RAV2	YPL161C	BEM4
YLR006C	SSK1	YJL187C	SWE1
YML095C	RAD10	YKL101W	HSL1
YNL264C	PDR17	YKL041W	VPS24
YGL078C	DBP3	YKL126W	YPK1
YKL032C	IXR1	YGL200C	EMP24
YIR005W	IST3	YER122C	GLO3
YGR184C	UBR1	YPL253C	VIK1
YDR137W	RGP1	YGR071C	YGR071C
YOR066W	MSA1	YJL183W	MNN11
YNL309W	STB1	YLR182W	SWI6
YMR198W	CIK1	YIL036W	CST6
YBR217W	ATG12	YML071C	COG8
YKL025C	PAN3	YLR360W	VPS38
YNL243W	SLA2	YJL030W	MAD2
YOR109W	INP53	YOR036W	PEP12
YML057W	CMP2	YPR119W	CLB2
YML071C	COG8	YLR398C	SKI2

Predictions at threshold level 0.80 continued...			
Gene1 of predicted pair		Gene2 of predicted pair	
Systematic Name	Standard Name	Systematic Name	Standard Name
YHR161C	YAP1801	YJL029C	VPS53
YKR099W	BAS1	YLR180W	SAM1
YMR198W	CIK1	YPR036W	VMA13
YLR234W	TOP3	YCR066W	RAD18
YLR429W	CRN1	YPR120C	CLB5
YDR378C	LSM6	YNL116W	DMA2
YLR292C	SEC72	YDR144C	MKC7
YNL069C	RPL16B	YHL031C	GOS1
YJR058C	APS2	YDR358W	GGA1
YJL127C	SPT10	YBR200W	BEM1
YJR090C	GRR1	YMR038C	CCS1
YER116C	SLX8	YCR053W	THR4
YDL002C	NHP10	YPR120C	CLB5
YJR063W	RPA12	YPL152W	RRD2
YNL238W	KEX2	YJL183W	MNN11
YML095C	RAD10	YDR379W	RGA2
YIL053W	RHR2	YDR310C	SUM1
YML071C	COG8	YKR030W	GMH1
YPL195W	APL5	YNL107W	YAF9
YDR289C	RTT103	YER139C	YER139C
YDR227W	SIR4	YBR010W	HHT1
YDR261C	EXG2	YDR207C	UME6
YNL031C	HHT2	YNL030W	HHF2
YNL291C	MID1	YAR014C	BUD14
YGL253W	HXK2	YAR014C	BUD14
YMR319C	FET4	YFL044C	OTU1
YPR017C	DSS4	YGR020C	VMA7
YPR120C	CLB5	YDR183W	PLP1
YPL253C	VIK1	YBR114W	RAD16

Predictions at threshold level 0.80 continued...			
**Gene1 of predicted pair**		**Gene2 of predicted pair**	
**Systematic Name**	**Standard Name**	**Systematic Name**	**Standard Name**
YNL106C	INP52	YFR030W	MET10
YDR484W	VPS52	YER144C	UBP5
YAL024C	LTE1	YLR452C	SST2
YJR060W	CBF1	YNR032W	PPG1
YLR148W	PEP3	YLR093C	NYV1
YGR019W	UGA1	YKL048C	ELM1
YFR040W	SAP155	YHL027W	RIM101
YBR010W	HHT1	YNL107W	YAF9
YPL253C	VIK1	YAL010C	MDM10
YPL037C	EGD1	YNL107W	YAF9
YMR198W	CIK1	YLR452C	SST2
YOR312C	RPL20B	YBL101C	ECM21
YDR144C	MKC7	YDR289C	RTT103
YGL087C	MMS2	YJR035W	RAD26
YPR084W	YPR084W	YDR320C	SWA2
YKR001C	VPS1	YDL006W	PTC1
YGL100W	SEH1	YNL273W	TOF1
YHR073W	OSH3	YMR164C	MSS11
YLR079W	SIC1	YNL107W	YAF9
YNL218W	MGS1	YDR207C	UME6
YLR337C	VRP1	YBL047C	EDE1
YBL058W	SHP1	YKR099W	BAS1
YNL223W	ATG4	YLR093C	NYV1
YNL106C	INP52	YPR120C	CLB5
YML038C	YMD8	YER162C	RAD4
YOL111C	MDY2	YMR133W	REC114
YDL184C	RPL41A	YER044C	ERG28
YBL047C	EDE1	YHR108W	GGA2
YOR127W	RGA1	YBR114W	RAD16

Predictions at threshold level 0.80 continued...			
Gene1 of predicted pair		Gene2 of predicted pair	
Systematic Name	Standard Name	Systematic Name	Standard Name
YDR456W	NHX1	YDR207C	UME6
YMR205C	PFK2	YPL089C	RLM1
YDR192C	NUP42	YJR090C	GRR1
YKR020W	VPS51	YJR060W	CBF1
YKL139W	CTK1	YJR135C	MCM22
YPL195W	APL5	YBR036C	CSG2
YNL307C	MCK1	YHR039C-A	VMA10
YBR200W	BEM1	YNL166C	BNI5
YBR107C	IML3	YJR135C	MCM22
YNL243W	SLA2	YKL079W	SMY1
YMR133W	REC114	YBR280C	SAF1
YDR137W	RGP1	YJR032W	CPR7
YNL243W	SLA2	YCR077C	PAT1
YKL110C	KTI12	YDR289C	RTT103
YDL116W	NUP84	YBR228W	SLX1
YJR010C-A	SPC1	YER019C-A	SBH2
YLR301W	YLR301W	YBR171W	SEC66
YEL065W	SIT1	YMR153W	NUP53
YBR108W	YBR108W	YDL192W	ARF1
YHR084W	STE12	YER109C	FLO8
YHR012W	VPS29	YOL067C	RTG1
YMR216C	SKY1	YGL115W	SNF4
YIL076W	SEC28	YNL127W	FAR11
YBR200W	BEM1	YLR381W	CTF3
YNL166C	BNI5	YAL002W	VPS8
YML095C	RAD10	YPR046W	MCM16
YDR155C	CPR1	YHR115C	DMA1
YBR108W	YBR108W	YHR012W	VPS29
YPL253C	VIK1	YJL095W	BCK1

Predictions at threshold level 0.80 continued...			
Gene1 of predicted pair		Gene2 of predicted pair	
Systematic Name	Standard Name	Systematic Name	Standard Name
YBR173C	UMP1	YKR099W	BAS1
YDL020C	RPN4	YPR036W	VMA13
YHR203C	RPS4B	YBL072C	RPS8A
YKL025C	PAN3	YDR183W	PLP1
YMR026C	PEX12	YPL057C	SUR1
YBL088C	TEL1	YGL090W	LIF1
YLR135W	SLX4	YGL175C	SAE2
YDR485C	VPS72	YBR089C-A	NHP6B
YDR200C	VPS64	YDL226C	GCS1
YDR335W	MSN5	YJR060W	CBF1
YOR033C	EXO1	YBR215W	HPC2
YKL126W	YPK1	YJL183W	MNN11
YPL024W	RMI1	YBR030W	YBR030W
YFR010W	UBP6	YML115C	VAN1
YBL025W	RRN10	YMR230W	RPS10B
YPR171W	BSP1	YJL095W	BCK1
YDR144C	MKC7	YGL124C	MON1
YGR104C	SRB5	YJL140W	RPB4
YCR081W	SRB8	YDR477W	SNF1
YLR170C	APS1	YOR035C	SHE4
YMR055C	BUB2	YGL148W	ARO2
YPR084W	YPR084W	YHL031C	GOS1
YNL199C	GCR2	YGL016W	KAP122
YML016C	PPZ1	YAL002W	VPS8
YOR197W	MCA1	YLL017W	YLL017W
YDR202C	RAV2	YLR447C	VMA6
YGR104C	SRB5	YCR081W	SRB8
YLR452C	SST2	YNL076W	MKS1
YJR063W	RPA12	YCR086W	CSM1

Predictions at threshold level 0.80 continued...			
Gene1 of predicted pair		Gene2 of predicted pair	
Systematic Name	Standard Name	Systematic Name	Standard Name
YBR107C	IML3	YGL253W	HXK2
YNL166C	BNI5	YNL078W	NIS1
YGR104C	SRB5	YDL106C	PHO2
YFR010W	UBP6	YNL082W	PMS1
YKR099W	BAS1	YDR358W	GGA1
YNL236W	SIN4	YGL003C	CDH1
YHR134W	WSS1	YPL161C	BEM4
YCL037C	SRO9	YOR327C	SNC2
YBR126C	TPS1	YDR261C	EXG2
YLR240W	VPS34	YOR035C	SHE4
YMR199W	CLN1	YOR275C	RIM20
YDR080W	VPS41	YNL107W	YAF9
YNL307C	MCK1	YBR036C	CSG2
YDR323C	PEP7	YDL226C	GCS1
YOR033C	EXO1	YMR116C	ASC1
YDR108W	GSG1	YLR447C	VMA6
YML016C	PPZ1	YPL152W	RRD2
YNL068C	FKH2	YOR275C	RIM20
YJR135C	MCM22	YMR038C	CCS1
YDR335W	MSN5	YDR076W	RAD55
YHR200W	RPN10	YKL101W	HSL1
YFL023W	BUD27	YOR327C	SNC2
YPL022W	RAD1	YBL079W	NUP170
YNL215W	IES2	YKL160W	ELF1
YMR224C	MRE11	YHR134W	WSS1
YLR095C	IOC2	YBL025W	RRN10
YLR079W	SIC1	YNL054W	VAC7
YJL095W	BCK1	YOL081W	IRA2
YDR484W	VPS52	YCR088W	ABP1

Predictions at threshold level 0.80 continued...			
Gene1 of predicted pair		Gene2 of predicted pair	
Systematic Name	Standard Name	Systematic Name	Standard Name
YHR060W	VMA22	YJR084W	CSN12
YPL105C	YPL105C	YGR105W	VMA21
YGR249W	MGA1	YNL307C	MCK1
YDR245W	MNN10	YPR120C	CLB5
YLR386W	VAC14	YOR290C	SNF2
YIL159W	BNR1	YBR280C	SAF1
YOR127W	RGA1	YKR048C	NAP1
YML016C	PPZ1	YPL158C	YPL158C
YNL243W	SLA2	YMR202W	ERG2
YML103C	NUP188	YNL225C	CNM67
YDL116W	NUP84	YPR120C	CLB5
YGL223C	COG1	YNL107W	YAF9
YOR358W	HAP5	YEL053C	MAK10
YBR030W	YBR030W	YML115C	VAN1
YKR001C	VPS1	YMR054W	STV1
YOR076C	SKI7	YNR052C	POP2
YKL007W	CAP1	YNL069C	RPL16B
YLR090W	XDJ1	YOR066W	MSA1
YER161C	SPT2	YAL002W	VPS8
YHR012W	VPS29	YPL195W	APL5
YKL007W	CAP1	YPL050C	MNN9
YKL191W	DPH2	YIL103W	DPH1
YDR146C	SWI5	YDL155W	CLB3
YPR046W	MCM16	YDL077C	VAM6
YJR063W	RPA12	YAR014C	BUD14
YDL013W	SLX5	YDR318W	MCM21
YBR009C	HHF1	YEL056W	HAT2
YKR072C	SIS2	YPL022W	RAD1
YPR084W	YPR084W	YGL174W	BUD13

Predictions at threshold level 0.80 continued...			
Gene1 of predicted pair		Gene2 of predicted pair	
Systematic Name	Standard Name	Systematic Name	Standard Name
YNL166C	BNI5	YHL002W	HSE1
YLR288C	MEC3	YBR036C	CSG2
YBR042C	CST26	YAL055W	PEX22
YJL053W	PEP8	YPR032W	SRO7
YDL223C	HBT1	YMR089C	YTA12
YKL139W	CTK1	YNL199C	GCR2
YMR032W	HOF1	YER118C	SHO1
YGL178W	MPT5	YLL021W	SPA2
YDR484W	VPS52	YGL025C	PGD1
YDR174W	HMO1	YML103C	NUP188
YDR289C	RTT103	YAL002W	VPS8
YHR178W	STB5	YNL166C	BNI5
YPL057C	SUR1	YHR081W	LRP1
YKR059W	TIF1	YOL045W	PSK2
YDL013W	SLX5	YBL079W	NUP170
YBR171W	SEC66	YDL223C	HBT1
YBR112C	CYC8	YGR252W	GCN5
YML028W	TSA1	YKL114C	APN1
YMR091C	NPL6	YHL025W	SNF6
YDR080W	VPS41	YNR032W	PPG1
YIL159W	BNR1	YJR084W	CSN12
YNL093W	YPT53	YBR264C	YPT10
YHR039C-A	VMA10	YNL107W	YAF9
YBR215W	HPC2	YJL006C	CTK2
YDR108W	GSG1	YEL043W	YEL043W
YPR120C	CLB5	YJL157C	FAR1
YDR293C	SSD1	YNL135C	FPR1
YBR274W	CHK1	YEL056W	HAT2
YGR072W	UPF3	YPL079W	RPL21B

Predictions at threshold level 0.80 continued...			
Gene1 of predicted pair		Gene2 of predicted pair	
Systematic Name	Standard Name	Systematic Name	Standard Name
YGL078C	DBP3	YBR019C	GAL10
YLR079W	SIC1	YNR052C	POP2
YER116C	SLX8	YDR207C	UME6
YPL057C	SUR1	YKL160W	ELF1
YDR004W	RAD57	YDR192C	NUP42
YLR094C	GIS3	YDR207C	UME6
YGR071C	YGR071C	YER122C	GLO3
YFL023W	BUD27	YML028W	TSA1
YDR245W	MNN10	YOR014W	RTS1
YER116C	SLX8	YNL248C	RPA49
YHR086W	NAM8	YMR133W	REC114
YEL065W	SIT1	YPL057C	SUR1
YDR443C	SSN2	YDL184C	RPL41A
YPL161C	BEM4	YJL117W	PHO86
YJL176C	SWI3	YHL002W	HSE1
YDR293C	SSD1	YBR107C	IML3
YMR004W	MVP1	YHR060W	VMA22
YDR183W	PLP1	YHL002W	HSE1
YMR104C	YPK2	YOL100W	PKH2
YGL025C	PGD1	YKL048C	ELM1
YDR146C	SWI5	YOR008C	SLG1
YGR239C	PEX21	YDL065C	PEX19
YHL031C	GOS1	YPL057C	SUR1
YDR484W	VPS52	YNR072W	HXT17
YMR052W	FAR3	YDL047W	SIT4
YGL133W	ITC1	YGL031C	RPL24A
YDL116W	NUP84	YOR080W	DIA2
YHR039C-A	VMA10	YPL120W	VPS30
YOR033C	EXO1	YDR156W	RPA14

Predictions at threshold level 0.80 continued...			
Gene1 of predicted pair		Gene2 of predicted pair	
Systematic Name	Standard Name	Systematic Name	Standard Name
YLR240W	VPS34	YKR020W	VPS51
YBL008W	HIR1	YNL031C	HHT2
YDR335W	MSN5	YLR350W	ORM2
YOL018C	TLG2	YEL042W	GDA1
YDL160C	DHH1	YGR054W	YGR054W
YBR030W	YBR030W	YPR046W	MCM16
YGL163C	RAD54	YPL018W	CTF19
YIL076W	SEC28	YFL023W	BUD27
YCR086W	CSM1	YHR115C	DMA1
YNL206C	RTT106	YBL047C	EDE1
YOR038C	HIR2	YNL107W	YAF9
YHR134W	WSS1	YOL067C	RTG1
YNR052C	POP2	YPL253C	VIK1
YJR135C	MCM22	YJR035W	RAD26
YOR358W	HAP5	YJL190C	RPS22A
YDR155C	CPR1	YHL002W	HSE1
YNL166C	BNI5	YEL056W	HAT2
YPL137C	GIP3	YFR036W	CDC26
YGL066W	SGF73	YLR410W	VIP1
YDR335W	MSN5	YAL002W	VPS8
YBR280C	SAF1	YEL060C	PRB1
YOR069W	VPS5	YGR020C	VMA7
YLR018C	POM34	YML115C	VAN1
YDL077C	VAM6	YOL081W	IRA2
YLR447C	VMA6	YJR032W	CPR7
YDR484W	VPS52	YMR133W	REC114
YPL024W	RMI1	YBR036C	CSG2
YNL307C	MCK1	YNL076W	MKS1
YOR039W	CKB2	YGL019W	CKB1

Predictions at threshold level 0.80 continued...			
Gene1 of predicted pair		Gene2 of predicted pair	
Systematic Name	Standard Name	Systematic Name	Standard Name
YPL129W	TAF14	YDR477W	SNF1
YOR290C	SNF2	YJR063W	RPA12
YAL014C	SYN8	YLR093C	NYV1
YPR131C	NAT3	YML028W	TSA1
YLR018C	POM34	YGR059W	SPR3
YOL111C	MDY2	YDL083C	RPS16B
YJL176C	SWI3	YBL025W	RRN10
YJR135C	MCM22	YHR115C	DMA1
YDR443C	SSN2	YKL048C	ELM1
YPR131C	NAT3	YBR107C	IML3
YOR127W	RGA1	YDR318W	MCM21
YDL223C	HBT1	YPL195W	APL5
YOR032C	HMS1	YML062C	MFT1
YGR019W	UGA1	YGL124C	MON1
YLR292C	SEC72	YLR234W	TOP3
YDL116W	NUP84	YJR135C	MCM22
YOR054C	VHS3	YKL113C	RAD27
YLR079W	SIC1	YBR194W	SOY1
YOR043W	WHI2	YML001W	YPT7
YGR184C	UBR1	YPL248C	GAL4
YBR084C-A	RPL19A	YHR115C	DMA1
YJL117W	PHO86	YBL101C	ECM21
YMR224C	MRE11	YGL212W	VAM7
YCR020C-A	MAK31	YMR216C	SKY1
YLR337C	VRP1	YBR107C	IML3
YOR132W	VPS17	YLR309C	IMH1
YDL047W	SIT4	YOL091W	SPO21
YHR178W	STB5	YOL081W	IRA2
YNL054W	VAC7	YKL048C	ELM1

Predictions at threshold level 0.80 continued...			
Gene1 of predicted pair		Gene2 of predicted pair	
Systematic Name	Standard Name	Systematic Name	Standard Name
YDR440W	DOT1	YOL081W	IRA2
YER144C	UBP5	YGL124C	MON1
YOR368W	RAD17	YLR032W	RAD5
YDR293C	SSD1	YLR309C	IMH1
YER139C	YER139C	YMR038C	CCS1
YBR171W	SEC66	YPL018W	CTF19
YDR004W	RAD57	YDR289C	RTT103
YOR351C	MEK1	YLR263W	RED1
YEL065W	SIT1	YBL103C	RTG3
YLR360W	VPS38	YHR134W	WSS1
YML095C	RAD10	YML028W	TSA1
YJL136C	RPS21B	YHR152W	SPO12
YLR090W	XDJ1	YOL081W	IRA2
YDL223C	HBT1	YER122C	GLO3
YDL059C	RAD59	YKR001C	VPS1
YJL183W	MNN11	YEL056W	HAT2
YKR057W	RPS21A	YHR111W	UBA4
YGR135W	PRE9	YHL002W	HSE1
YDR293C	SSD1	YCR066W	RAD18
YKR099W	BAS1	YDL083C	RPS16B
YOR337W	TEA1	YLR386W	VAC14
YIL159W	BNR1	YNL076W	MKS1
YLR452C	SST2	YMR133W	REC114
YNR052C	POP2	YOL030W	GAS5
YDR289C	RTT103	YGL031C	RPL24A
YML016C	PPZ1	YDR363W-A	SEM1
YDR265W	PEX10	YPR119W	CLB2
YNR072W	HXT17	YDL083C	RPS16B
YFR010W	UBP6	YOL051W	GAL11

Predictions at threshold level 0.80 continued...			
Gene1 of predicted pair		Gene2 of predicted pair	
Systematic Name	Standard Name	Systematic Name	Standard Name
YHR073W	OSH3	YER115C	SPR6
YDR146C	SWI5	YBR036C	CSG2
YAL020C	ATS1	YDR309C	GIC2
YLR360W	VPS38	YPL105C	YPL105C
YHR156C	LIN1	YKL139W	CTK1
YLR381W	CTF3	YPL106C	SSE1
YIL034C	CAP2	YJL187C	SWE1
YAL024C	LTE1	YLR399C	BDF1
YLR386W	VAC14	YLR381W	CTF3
YMR116C	ASC1	YLR425W	TUS1
YLR386W	VAC14	YLR399C	BDF1
YOL030W	GAS5	YBL079W	NUP170
YIL053W	RHR2	YBL058W	SHP1
YNL215W	IES2	YOR195W	SLK19
YLR094C	GIS3	YER122C	GLO3
YGR056W	RSC1	YBR089C-A	NHP6B
YDR335W	MSN5	YOR308C	SNU66
YMR039C	SUB1	YNL215W	IES2
YOR290C	SNF2	YNR023W	SNF12
YJR056C	YJR056C	YDL077C	VAM6
YPR017C	DSS4	YJL004C	SYS1
YHR111W	UBA4	YPL256C	CLN2
YER162C	RAD4	YER139C	YER139C
YLR058C	SHM2	YPL018W	CTF19
YOR270C	VPH1	YPL037C	EGD1
YAL024C	LTE1	YLR320W	MMS22
YOR327C	SNC2	YHR115C	DMA1
YBR084C-A	RPL19A	YKL048C	ELM1
YOR035C	SHE4	YBR082C	UBC4

Predictions at threshold level 0.80 continued...			
Gene1 of predicted pair		Gene2 of predicted pair	
Systematic Name	Standard Name	Systematic Name	Standard Name
YKR020W	VPS51	YOR312C	RPL20B
YPL022W	RAD1	YBR082C	UBC4
YBR057C	MUM2	YOR195W	SLK19
YIL034C	CAP2	YMR172W	HOT1
YPR036W	VMA13	YML001W	YPT7
YKL139W	CTK1	YJR084W	CSN12
YCL001W	RER1	YML071C	COG8
YPR052C	NHP6A	YOR039W	CKB2
YML016C	PPZ1	YGL250W	RMR1
YGL200C	EMP24	YJL006C	CTK2
YHR111W	UBA4	YNL293W	MSB3
YNL291C	MID1	YDR202C	RAV2
YMR039C	SUB1	YEL056W	HAT2
YMR231W	PEP5	YPL045W	VPS16
YBR171W	SEC66	YER044C	ERG28
YPL042C	SSN3	YKL213C	DOA1
YBR173C	UMP1	YOR290C	SNF2
YDL101C	DUN1	YLR313C	SPH1
YOR290C	SNF2	YKL048C	ELM1
YGR019W	UGA1	YGL237C	HAP2
YHR039C-A	VMA10	YKR030W	GMH1
YDR293C	SSD1	YBL088C	TEL1
YHR200W	RPN10	YBR082C	UBC4
YBR217W	ATG12	YHR081W	LRP1
YLR090W	XDJ1	YGL078C	DBP3
YGL078C	DBP3	YNL307C	MCK1
YPR017C	DSS4	YDR293C	SSD1
YBR188C	NTC20	YIR009W	MSL1
YPR018W	RLF2	YOR195W	SLK19

Predictions at threshold level 0.80 continued...			
Gene1 of predicted pair		Gene2 of predicted pair	
Systematic Name	Standard Name	Systematic Name	Standard Name
YPL018W	CTF19	YPR119W	CLB2
YKR001C	VPS1	YJR035W	RAD26
YGR072W	UPF3	YDR320C	SWA2
YBL051C	PIN4	YLR233C	EST1
YOR023C	AHC1	YDR155C	CPR1
YCR086W	CSM1	YGL148W	ARO2
YPR029C	APL4	YAL030W	SNC1
YAL024C	LTE1	YBR173C	UMP1
YOR195W	SLK19	YPL256C	CLN2
YGL190C	CDC55	YLR200W	YKE2
YPL256C	CLN2	YLR131C	ACE2
YPL037C	EGD1	YMR311C	GLC8
YDL116W	NUP84	YDR174W	HMO1
YMR198W	CIK1	YHR012W	VPS29
YLR386W	VAC14	YER110C	KAP123
YIL103W	DPH1	YFR036W	CDC26
YOR337W	TEA1	YML115C	VAN1
YPR084W	YPR084W	YOR124C	UBP2
YFR030W	MET10	YML112W	CTK3
YLL039C	UBI4	YOR312C	RPL20B
YDR439W	LRS4	YGR188C	BUB1
YKL139W	CTK1	YOR124C	UBP2
YBR057C	MUM2	YMR055C	BUB2
YJL154C	VPS35	YBL051C	PIN4
YDR439W	LRS4	YJL006C	CTK2
YDR244W	PEX5	YGR077C	PEX8
YGL178W	MPT5	YPR119W	CLB2
YOR195W	SLK19	YJL127C	SPT10
YCR086W	CSM1	YLR032W	RAD5

Predictions at threshold level 0.80 continued...			
Gene1 of predicted pair		Gene2 of predicted pair	
Systematic Name	Standard Name	Systematic Name	Standard Name
YBR131W	CCZ1	YDL077C	VAM6
YDL006W	PTC1	YNL051W	COG5
YNL032W	SIW14	YGL124C	MON1
YNL044W	YIP3	YCL001W	RER1
YPR051W	MAK3	YGR020C	VMA7
YKL139W	CTK1	YLR315W	NKP2
YHR012W	VPS29	YDR310C	SUM1
YNL068C	FKH2	YJL136C	RPS21B
YPL253C	VIK1	YPR120C	CLB5
YMR054W	STV1	YAR014C	BUD14
YMR095C	SNO1	YLR320W	MMS22
YLR452C	SST2	YNL041C	COG6
YBR036C	CSG2	YER122C	GLO3
YDL083C	RPS16B	YPL137C	GIP3
YGL115W	SNF4	YDR422C	SIP1
YKL116C	PRR1	YHR039C-A	VMA10
YDR363W-A	SEM1	YIL008W	URM1
YDR137W	RGP1	YJR033C	RAV1
YDR289C	RTT103	YJR090C	GRR1
YOR080W	DIA2	YBR082C	UBC4
YIL148W	RPL40A	YOR358W	HAP5
YMR095C	SNO1	YBR019C	GAL10
YGR184C	UBR1	YPR036W	VMA13
YLR410W	VIP1	YGL163C	RAD54
YBR215W	HPC2	YGR252W	GCN5
YHL031C	GOS1	YAL002W	VPS8
YDR385W	EFT2	YDR137W	RGP1
YEL005C	VAB2	YEL013W	VAC8
YLR119W	SRN2	YHR039C-A	VMA10

Predictions at threshold level 0.80 continued...			
Gene1 of predicted pair		Gene2 of predicted pair	
Systematic Name	Standard Name	Systematic Name	Standard Name
YHR178W	STB5	YHR114W	BZZ1
YLR233C	EST1	YAL002W	VPS8
YHR098C	SFB3	YOL012C	HTZ1
YDR155C	CPR1	YGL003C	CDH1
YDL226C	GCS1	YDL137W	ARF2
YLR079W	SIC1	YBL079W	NUP170
YDL184C	RPL41A	YJL095W	BCK1
YBR200W	BEM1	YMR038C	CCS1
YNL307C	MCK1	YGL003C	CDH1
YDL116W	NUP84	YHR073W	OSH3
YPL024W	RMI1	YLR234W	TOP3
YMR198W	CIK1	YJL095W	BCK1
YCL037C	SRO9	YEL060C	PRB1
YGR133W	PEX4	YKR014C	YPT52
YLR148W	PEP3	YHR161C	YAP1801
YDR477W	SNF1	YLR386W	VAC14
YCR077C	PAT1	YGL148W	ARO2
YBL021C	HAP3	YDR254W	CHL4
YOR132W	VPS17	YHR111W	UBA4
YPL037C	EGD1	YER139C	YER139C
YPR028W	YOP1	YGL198W	YIP4
YPR051W	MAK3	YGR188C	BUB1
YCR024C-A	PMP1	YGL016W	KAP122
YOR195W	SLK19	YDR264C	AKR1
YKR072C	SIS2	YMR055C	BUB2
YPR051W	MAK3	YCL037C	SRO9
YHR152W	SPO12	YHR200W	RPN10
YFR030W	MET10	YGR262C	BUD32
YBR200W	BEM1	YBR215W	HPC2

Predictions at threshold level 0.80 continued...			
Gene1 of predicted pair		Gene2 of predicted pair	
Systematic Name	Standard Name	Systematic Name	Standard Name
YIL053W	RHR2	YLR370C	ARC18
YJR040W	GEF1	YNL238W	KEX2
YDR261C	EXG2	YJR084W	CSN12
YOL126C	MDH2	YPL256C	CLN2
YMR026C	PEX12	YDL223C	HBT1
YFL023W	BUD27	YNL076W	MKS1
YOR270C	VPH1	YDL223C	HBT1
YHR156C	LIN1	YJL183W	MNN11
YKL032C	IXR1	YLR425W	TUS1
YNL238W	KEX2	YNL127W	FAR11
YDR378C	LSM6	YLR410W	VIP1
YJR032W	CPR7	YCR053W	THR4
YOR106W	VAM3	YPL234C	TFP3
YMR198W	CIK1	YMR038C	CCS1
YHR060W	VMA22	YDR207C	UME6
YDL155W	CLB3	YHR114W	BZZ1
YDR318W	MCM21	YNL116W	DMA2
YDL065C	PEX19	YOL044W	PEX15
YPR040W	TIP41	YLR425W	TUS1
YMR109W	MYO5	YJR090C	GRR1
YOR043W	WHI2	YLR398C	SKI2
YER075C	PTP3	YER089C	PTC2
YLR090W	XDJ1	YPL057C	SUR1
YOR327C	SNC2	YKR030W	GMH1
YMR004W	MVP1	YNL127W	FAR11
YMR116C	ASC1	YDL223C	HBT1
YOR033C	EXO1	YNL076W	MKS1
YLR381W	CTF3	YGR135W	PRE9
YMR152W	YIM1	YDL077C	VAM6

Predictions at threshold level 0.80 continued...			
Gene1 of predicted pair		Gene2 of predicted pair	
Systematic Name	Standard Name	Systematic Name	Standard Name
YBR200W	BEM1	YNL229C	URE2
YFR031C-A	RPL2A	YLR370C	ARC18
YLR018C	POM34	YGL215W	CLG1
YMR224C	MRE11	YNL291C	MID1
YFR024C-A	LSB3	YPR119W	CLB2
YKR093W	PTR2	YPR149W	NCE102
YLR135W	SLX4	YBR073W	RDH54
YHR039C-A	VMA10	YPR032W	SRO7
YNL199C	GCR2	YOR014W	RTS1
YPR084W	YPR084W	YNL291C	MID1
YJR032W	CPR7	YNL051W	COG5
YDL116W	NUP84	YKL139W	CTK1
YGL163C	RAD54	YOR014W	RTS1
YIL076W	SEC28	YKR020W	VPS51
YLR079W	SIC1	YLR371W	ROM2
YGL212W	VAM7	YPL037C	EGD1
YGR249W	MGA1	YHR060W	VMA22
YFR031C-A	RPL2A	YNL307C	MCK1
YNL054W	VAC7	YJL124C	LSM1
YNR052C	POP2	YML112W	CTK3
YMR080C	NAM7	YNL106C	INP52
YJR052W	RAD7	YBR114W	RAD16
YJL176C	SWI3	YKL032C	IXR1
YDR174W	HMO1	YFR036W	CDC26
YJR135C	MCM22	YDR318W	MCM21
YDL020C	RPN4	YPL234C	TFP3
YCR002C	CDC10	YDR309C	GIC2
YPL042C	SSN3	YOR308C	SNU66
YHR156C	LIN1	YLR452C	SST2

Predictions at threshold level 0.80 continued...			
Gene1 of predicted pair		Gene2 of predicted pair	
Systematic Name	Standard Name	Systematic Name	Standard Name
YBR194W	SOY1	YKL114C	APN1
YPL057C	SUR1	YHR167W	THP2
YLR360W	VPS38	YBL051C	PIN4
YML115C	VAN1	YLR180W	SAM1
YDL080C	THI3	YNL107W	YAF9
YNL087W	TCB2	YKL126W	YPK1
YPL137C	GIP3	YLR180W	SAM1
YER116C	SLX8	YPL253C	VIK1
YPL253C	VIK1	YDR200C	VPS64
YHR039C-A	VMA10	YNL076W	MKS1
YML071C	COG8	YDR207C	UME6
YLR192C	HCR1	YLR093C	NYV1
YDR129C	SAC6	YDL161W	ENT1
YOR018W	ROD1	YLR248W	RCK2
YPR084W	YPR084W	YLR182W	SWI6
YOR106W	VAM3	YKR030W	GMH1
YLR313C	SPH1	YDL013W	SLX5
YER110C	KAP123	YBR107C	IML3
YKR099W	BAS1	YNL041C	COG6
YDR443C	SSN2	YER044C	ERG28
YDL013W	SLX5	YBR082C	UBC4
YMR216C	SKY1	YEL053C	MAK10
YPR018W	RLF2	YOR101W	RAS1
YIL076W	SEC28	YHR167W	THP2
YMR070W	MOT3	YOR035C	SHE4
YGL115W	SNF4	YLR320W	MMS22
YDR080W	VPS41	YER120W	SCS2
YAL024C	LTE1	YDR363W-A	SEM1
YFL003C	MSH4	YPR119W	CLB2

Predictions at threshold level 0.80 continued...			
Gene1 of predicted pair		Gene2 of predicted pair	
Systematic Name	Standard Name	Systematic Name	Standard Name
YBR228W	SLX1	YMR116C	ASC1
YMR054W	STV1	YNR032W	PPG1
YOR106W	VAM3	YAR014C	BUD14
YLR386W	VAC14	YGL253W	HXK2
YKL139W	CTK1	YPL253C	VIK1
YNL291C	MID1	YNL051W	COG5
YLR360W	VPS38	YBR200W	BEM1
YJL172W	CPS1	YOR290C	SNF2
YLR381W	CTF3	YML028W	TSA1
YOL067C	RTG1	YNL107W	YAF9
YGL175C	SAE2	YOL090W	MSH2
YGL087C	MMS2	YDL013W	SLX5
YIL156W	UBP7	YKL068W	NUP100
YML109W	ZDS2	YDR309C	GIC2
YMR004W	MVP1	YNL238W	KEX2
YGR104C	SRB5	YNR052C	POP2
YDR363W-A	SEM1	YJL183W	MNN11
YDR254W	CHL4	YGL151W	NUT1
YEL050C	RML2	YNL107W	YAF9
YGR072W	UPF3	YFR010W	UBP6
YGR071C	YGR071C	YNL041C	COG6
YDR484W	VPS52	YJL172W	CPS1
YGL087C	MMS2	YDR076W	RAD55
YPL178W	CBC2	YFL047W	RGD2
YDR261C	EXG2	YNL076W	MKS1
YGR105W	VMA21	YAL055W	PEX22
YHR081W	LRP1	YMR214W	SCJ1
YKL214C	YRA2	YBR111W-A	SUS1
YDR496C	PUF6	YHR052W	CIC1

Predictions at threshold level 0.80 continued...			
Gene1 of predicted pair		Gene2 of predicted pair	
Systematic Name	Standard Name	Systematic Name	Standard Name
YBL058W	SHP1	YKL139W	CTK1
YMR142C	RPL13B	YJR084W	CSN12
YLR381W	CTF3	YOR023C	AHC1
YFR031C-A	RPL2A	YPL105C	YPL105C
YGL066W	SGF73	YBL047C	EDE1
YMR231W	PEP5	YDL226C	GCS1
YDR156W	RPA14	YDR335W	MSN5
YPL057C	SUR1	YGR188C	BUB1
YJR032W	CPR7	YOL081W	IRA2
YJL154C	VPS35	YJR040W	GEF1
YPL161C	BEM4	YER162C	RAD4
YIL076W	SEC28	YNL032W	SIW14
YOR290C	SNF2	YGL003C	CDH1
YLR288C	MEC3	YBR274W	CHK1
YFL023W	BUD27	YHR081W	LRP1
YER116C	SLX8	YLR381W	CTF3
YML016C	PPZ1	YNL147W	LSM7
YKR020W	VPS51	YNL051W	COG5
YLR292C	SEC72	YGL151W	NUT1
YLR119W	SRN2	YPL137C	GIP3
YML071C	COG8	YPR032W	SRO7
YBL025W	RRN10	YLR398C	SKI2
YML121W	GTR1	YKR007W	MEH1
YPL253C	VIK1	YJR063W	RPA12
YNL215W	IES2	YPL018W	CTF19
YGR105W	VMA21	YJR033C	RAV1
YGL066W	SGF73	YOR275C	RIM20
YKR061W	KTR2	YMR055C	BUB2
YLR119W	SRN2	YNR072W	HXT17

Predictions at threshold level 0.80 continued...			
Gene1 of predicted pair		Gene2 of predicted pair	
Systematic Name	Standard Name	Systematic Name	Standard Name
YJR056C	YJR056C	YDR137W	RGP1
YDR443C	SSN2	YMR230W	RPS10B
YHR060W	VMA22	YEL053C	MAK10
YJR140C	HIR3	YOR308C	SNU66
YDR484W	VPS52	YOR043W	WHI2
YPL115C	BEM3	YLR447C	VMA6
YDR378C	LSM6	YDR183W	PLP1
YMR016C	SOK2	YBR280C	SAF1
YER044C	ERG28	YJR032W	CPR7
YNL288W	CAF40	YGR108W	CLB1
YBR030W	YBR030W	YJL172W	CPS1
YDR245W	MNN10	YDR207C	UME6
YLR093C	NYV1	YMR214W	SCJ1
YKL116C	PRR1	YOR290C	SNF2
YML038C	YMD8	YNR032W	PPG1
YKL213C	DOA1	YER122C	GLO3
YLR337C	VRP1	YGR108W	CLB1
YGL237C	HAP2	YDR183W	PLP1
YDR334W	SWR1	YGL190C	CDC55
YDR293C	SSD1	YDR310C	SUM1
YJL154C	VPS35	YNL291C	MID1
YJL124C	LSM1	YHR115C	DMA1
YJL137C	GLG2	YIL045W	PIG2
YNL236W	SIN4	YLR370C	ARC18
YDR004W	RAD57	YMR224C	MRE11
YDR080W	VPS41	YOR036W	PEP12
YIL008W	URM1	YAL002W	VPS8
YGL115W	SNF4	YNL051W	COG5
YBR127C	VMA2	YKR020W	VPS51

Predictions at threshold level 0.80 continued...			
Gene1 of predicted pair		Gene2 of predicted pair	
Systematic Name	Standard Name	Systematic Name	Standard Name
YPL024W	RMI1	YBR215W	HPC2
YDR378C	LSM6	YJR060W	CBF1
YDR363W-A	SEM1	YOR312C	RPL20B
YGL174W	BUD13	YDR378C	LSM6
YDL184C	RPL41A	YBR171W	SEC66
YPL057C	SUR1	YOR043W	WHI2
YGR071C	YGR071C	YHR077C	NMD2
YOR043W	WHI2	YJL183W	MNN11
YNL032W	SIW14	YMR214W	SCJ1
YBR108W	YBR108W	YPL037C	EGD1
YLR119W	SRN2	YOR066W	MSA1
YFR031C-A	RPL2A	YKR020W	VPS51
YJR070C	LIA1	YEL022W	GEA2
YBR274W	CHK1	YPR120C	CLB5
YDR183W	PLP1	YML112W	CTK3
YPR029C	APL4	YOR327C	SNC2
YER001W	MNN1	YNL079C	TPM1
YGL087C	MMS2	YLR150W	STM1
YPR131C	NAT3	YJR135C	MCM22
YGL078C	DBP3	YLR371W	ROM2
YNL069C	RPL16B	YIL076W	SEC28
YMR026C	PEX12	YBR217W	ATG12
YOR132W	VPS17	YHR060W	VMA22
YBL058W	SHP1	YGL124C	MON1
YER001W	MNN1	YFR036W	CDC26
YPL018W	CTF19	YNL225C	CNM67
YJL176C	SWI3	YDR129C	SAC6
YER116C	SLX8	YMR133W	REC114
YOR290C	SNF2	YLR398C	SKI2

Predictions at threshold level 0.80 continued...			
Gene1 of predicted pair		Gene2 of predicted pair	
Systematic Name	Standard Name	Systematic Name	Standard Name
YGL033W	HOP2	YLR180W	SAM1
YJL127C	SPT10	YPL018W	CTF19
YMR039C	SUB1	YDR254W	CHL4
YBR171W	SEC66	YDL006W	PTC1
YGR217W	CCH1	YBR041W	FAT1
YBR030W	YBR030W	YNR072W	HXT17
YGL078C	DBP3	YOR290C	SNF2
YPR032W	SRO7	YAL002W	VPS8
YLR386W	VAC14	YER122C	GLO3
YMR039C	SUB1	YGL163C	RAD54
YDR293C	SSD1	YML001W	YPT7
YKL119C	VPH2	YKL041W	VPS24
YCL037C	SRO9	YER162C	RAD4
YIL036W	CST6	YDR289C	RTT103
YDR137W	RGP1	YHR167W	THP2
YDR225W	HTA1	YPL129W	TAF14
YNL201C	PSY2	YAR014C	BUD14
YDR477W	SNF1	YPR030W	CSR2
YLR079W	SIC1	YDR245W	MNN10
YIL034C	CAP2	YDR183W	PLP1
YDR358W	GGA1	YBL025W	RRN10
YGL200C	EMP24	YOR141C	ARP8
YGR135W	PRE9	YHR115C	DMA1
YDL006W	PTC1	YDR310C	SUM1
YER116C	SLX8	YBR215W	HPC2
YIL034C	CAP2	YMR055C	BUB2
YBR215W	HPC2	YNL236W	SIN4
YPL024W	RMI1	YOR014W	RTS1
YJR040W	GEF1	YPL234C	TFP3

Predictions at threshold level 0.80 continued...			
Gene1 of predicted pair		Gene2 of predicted pair	
Systematic Name	Standard Name	Systematic Name	Standard Name
YMR109W	MYO5	YER118C	SHO1
YPR051W	MAK3	YKR030W	GMH1
YCL037C	SRO9	YOR038C	HIR2
YER040W	GLN3	YOL081W	IRA2
YER139C	YER139C	YOL081W	IRA2
YDL116W	NUP84	YER116C	SLX8
YJL172W	CPS1	YDL077C	VAM6
YGL078C	DBP3	YHR060W	VMA22
YOL111C	MDY2	YGL124C	MON1
YAL024C	LTE1	YGL003C	CDH1
YAL024C	LTE1	YDR358W	GGA1
YCR039C	MATALPHA2	YPL240C	HSP82
YGR108W	CLB1	YOR141C	ARP8
YGR249W	MGA1	YBR217W	ATG12
YDR318W	MCM21	YHR115C	DMA1
YHR079C	IRE1	YOL031C	SIL1
YJR140C	HIR3	YML115C	VAN1
YIL008W	URM1	YNL079C	TPM1
YPL024W	RMI1	YKR099W	BAS1
YBR258C	SHG1	YDR155C	CPR1
YPR030W	CSR2	YDL192W	ARF1
YDL116W	NUP84	YPR131C	NAT3
YMR080C	NAM7	YJR140C	HIR3
YBL103C	RTG3	YJL183W	MNN11
YOL091W	SPO21	YNL107W	YAF9
YLR234W	TOP3	YGL148W	ARO2
YLR119W	SRN2	YPL057C	SUR1
YDR378C	LSM6	YPL213W	LEA1
YGL033W	HOP2	YPL037C	EGD1

Predictions at threshold level 0.80 continued...			
Gene1 of predicted pair		Gene2 of predicted pair	
Systematic Name	Standard Name	Systematic Name	Standard Name
YNL325C	FIG4	YOR066W	MSA1
YER044C	ERG28	YKR035W-A	DID2
YDR320C	SWA2	YPR119W	CLB2
YPR017C	DSS4	YPL120W	VPS30
YGR133W	PEX4	YJR084W	CSN12
YIL159W	BNR1	YGL124C	MON1
YNL206C	RTT106	YMR179W	SPT21
YJL124C	LSM1	YBR036C	CSG2
YLR102C	APC9	YNL229C	URE2
YJR140C	HIR3	YGL174W	BUD13
YOR195W	SLK19	YOR101W	RAS1
YLL007C	YLL007C	YJL190C	RPS22A
YGR217W	CCH1	YMR038C	CCS1
YOR270C	VPH1	YPL161C	BEM4
YLR360W	VPS38	YBR171W	SEC66
YBL102W	SFT2	YHR012W	VPS29
YFR031C-A	RPL2A	YMR214W	SCJ1
YLR386W	VAC14	YPL152W	RRD2
YHR200W	RPN10	YGL124C	MON1
YKL139W	CTK1	YLR093C	NYV1
YGR019W	UGA1	YGR188C	BUB1
YMR139W	RIM11	YDR207C	UME6
YDR395W	SXM1	YER110C	KAP123
YMR004W	MVP1	YLR309C	IMH1
YPL001W	HAT1	YBR195C	MSI1
YBL058W	SHP1	YML115C	VAN1
YPL161C	BEM4	YDR318W	MCM21
YML103C	NUP188	YAR014C	BUD14
YML115C	VAN1	YER044C	ERG28

Predictions at threshold level 0.80 continued...			
Gene1 of predicted pair		Gene2 of predicted pair	
Systematic Name	Standard Name	Systematic Name	Standard Name
YIL156W	UBP7	YML103C	NUP188
YNL289W	PCL1	YOR327C	SNC2
YMR224C	MRE11	YNR072W	HXT17
YLR360W	VPS38	YKR030W	GMH1
YOR033C	EXO1	YHR134W	WSS1
YOR276W	CAF20	YOL045W	PSK2
YBR030W	YBR030W	YER040W	GLN3
YPR131C	NAT3	YKL114C	APN1
YLR360W	VPS38	YGL253W	HXK2
YHR152W	SPO12	YPL161C	BEM4
YNL307C	MCK1	YBR019C	GAL10
YPL253C	VIK1	YFR030W	MET10
YPR017C	DSS4	YDR202C	RAV2
YDR156W	RPA14	YML103C	NUP188
YKL126W	YPK1	YBL047C	EDE1
YKR072C	SIS2	YOR038C	HIR2
YPL046C	ELC1	YGL115W	SNF4
YHR167W	THP2	YNL107W	YAF9
YKL213C	DOA1	YHL002W	HSE1
YJL176C	SWI3	YKR014C	YPT52
YPR102C	RPL11A	YGR100W	MDR1
YFL023W	BUD27	YPR030W	CSR2
YOL145C	CTR9	YDR155C	CPR1
YKL139W	CTK1	YOR080W	DIA2
YGL054C	ERV14	YEL051W	VMA8
YFR031C-A	RPL2A	YDR320C	SWA2
YMR273C	ZDS1	YGL016W	KAP122
YHR160C	PEX18	YNL214W	PEX17
YNL201C	PSY2	YLR386W	VAC14

Predictions at threshold level 0.80 continued...			
Gene1 of predicted pair		Gene2 of predicted pair	
Systematic Name	Standard Name	Systematic Name	Standard Name
YHR156C	LIN1	YJR084W	CSN12
YFL023W	BUD27	YBR280C	SAF1
YDR383C	NKP1	YJR060W	CBF1
YNL076W	MKS1	YEL060C	PRB1
YIR005W	IST3	YNL107W	YAF9
YDL223C	HBT1	YHR081W	LRP1
YMR198W	CIK1	YPL001W	HAT1
YBL008W	HIR1	YCR077C	PAT1
YER179W	DMC1	YNL106C	INP52
YKL116C	PRR1	YJR084W	CSN12
YOR132W	VPS17	YJR090C	GRR1
YLR360W	VPS38	YDR080W	VPS41
YMR039C	SUB1	YNL236W	SIN4
YNL236W	SIN4	YDR318W	MCM21
YEL065W	SIT1	YLR452C	SST2
YDR181C	SAS4	YMR127C	SAS2
YML103C	NUP188	YGR270W	YTA7
YBR217W	ATG12	YPL195W	APL5
YLR119W	SRN2	YDL077C	VAM6
YHR156C	LIN1	YNL041C	COG6
YBL058W	SHP1	YCR053W	THR4
YBR107C	IML3	YJR005W	APL1
YLR337C	VRP1	YHR013C	ARD1
YMR142C	RPL13B	YKR035W-A	DID2
YDR108W	GSG1	YDL192W	ARF1
YJL176C	SWI3	YLR360W	VPS38
YHR178W	STB5	YBR200W	BEM1
YPL161C	BEM4	YGL163C	RAD54
YBR057C	MUM2	YLR381W	CTF3

Predictions at threshold level 0.80 continued...			
Gene1 of predicted pair		Gene2 of predicted pair	
Systematic Name	Standard Name	Systematic Name	Standard Name
YDL047W	SIT4	YKL114C	APN1
YGL115W	SNF4	YDL192W	ARF1
YPL024W	RMI1	YKL139W	CTK1
YBR171W	SEC66	YJL183W	MNN11
YPL240C	HSP82	YBR089C-A	NHP6B
YPR120C	CLB5	YOR368W	RAD17
YLR192C	HCR1	YOL081W	IRA2
YDR383C	NKP1	YPL253C	VIK1
YKL010C	UFD4	YML115C	VAN1
YDR129C	SAC6	YLR371W	ROM2
YMR004W	MVP1	YJL004C	SYS1
YDR477W	SNF1	YGL003C	CDH1
YDL020C	RPN4	YJR032W	CPR7
YPR018W	RLF2	YLR102C	APC9
YGL163C	RAD54	YOR368W	RAD17
YPR084W	YPR084W	YDR207C	UME6
YBL008W	HIR1	YDL042C	SIR2
YLR192C	HCR1	YMR198W	CIK1
YLR083C	EMP70	YHL020C	OPI1
YJR090C	GRR1	YAL002W	VPS8
YCL067C	HMLALPHA2	YCR039C	MATALPHA2
YDR379W	RGA2	YJL095W	BCK1
YDL013W	SLX5	YDR207C	UME6
YPL024W	RMI1	YJL183W	MNN11
YBL058W	SHP1	YPR030W	CSR2
YLR102C	APC9	YIL008W	URM1
YPR084W	YPR084W	YOR112W	CEX1
YKL068W	NUP100	YDR076W	RAD55
YMR199W	CLN1	YER027C	GAL83

Predictions at threshold level 0.80 continued...			
Gene1 of predicted pair		Gene2 of predicted pair	
Systematic Name	Standard Name	Systematic Name	Standard Name
YJL006C	CTK2	YKL041W	VPS24
YMR179W	SPT21	YPL253C	VIK1
YNL201C	PSY2	YLR254C	NDL1
YOR080W	DIA2	YDL002C	NHP10
YJL154C	VPS35	YCR088W	ABP1
YIL076W	SEC28	YMR214W	SCJ1
YNL079C	TPM1	YGL019W	CKB1
YDL116W	NUP84	YPL042C	SSN3
YJL183W	MNN11	YMR230W	RPS10B
YLR381W	CTF3	YDR155C	CPR1
YBR173C	UMP1	YPL022W	RAD1
YER116C	SLX8	YNL087W	TCB2
YMR004W	MVP1	YOL018C	TLG2
YOR109W	INP53	YJR033C	RAV1
YKL139W	CTK1	YLR180W	SAM1
YMR202W	ERG2	YGL115W	SNF4
YGL078C	DBP3	YDL184C	RPL41A
YPL046C	ELC1	YER162C	RAD4
YDR254W	CHL4	YPR119W	CLB2
YBL017C	PEP1	YJR125C	ENT3
YDL184C	RPL41A	YKR099W	BAS1
YDR443C	SSN2	YER162C	RAD4
YJR032W	CPR7	YLR398C	SKI2
YMR142C	RPL13B	YGL115W	SNF4
YJL004C	SYS1	YNL127W	FAR11
YLR234W	TOP3	YBR036C	CSG2
YER179W	DMC1	YBR073W	RDH54
YPL024W	RMI1	YER139C	YER139C
YGL025C	PGD1	YPL161C	BEM4

Predictions at threshold level 0.80 continued...			
Gene1 of predicted pair		Gene2 of predicted pair	
Systematic Name	Standard Name	Systematic Name	Standard Name
YBR289W	SNF5	YKL109W	HAP4
YKL139W	CTK1	YHR200W	RPN10
YMR153W	NUP53	YGL003C	CDH1
YKL126W	YPK1	YER122C	GLO3
YEL053C	MAK10	YDR261C	EXG2
YOR127W	RGA1	YGR054W	YGR054W
YBR127C	VMA2	YJL183W	MNN11
YLR095C	IOC2	YKR035W-A	DID2
YEL053C	MAK10	YJR032W	CPR7
YPL046C	ELC1	YOR028C	CIN5
YEL027W	CUP5	YKR001C	VPS1
YPL037C	EGD1	YKR035W-A	DID2
YOR127W	RGA1	YCL037C	SRO9
YOR106W	VAM3	YGL198W	YIP4
YLR315W	NKP2	YDR440W	DOT1
YNL051W	COG5	YNL107W	YAF9
YKL139W	CTK1	YOR001W	RRP6
YGR184C	UBR1	YCL014W	BUD3
YIL159W	BNR1	YNL058C	YNL058C
YJL036W	SNX4	YOR109W	INP53
YOR195W	SLK19	YPR120C	CLB5
YNL289W	PCL1	YNL116W	DMA2
YER144C	UBP5	YDR207C	UME6
YDL155W	CLB3	YOL067C	RTG1
YGR252W	GCN5	YHR200W	RPN10
YOR083W	WHI5	YJR005W	APL1
YBR009C	HHF1	YOR141C	ARP8
YOR001W	RRP6	YER139C	YER139C
YDR320C	SWA2	YJR031C	GEA1

Predictions at threshold level 0.80 continued...			
Gene1 of predicted pair		Gene2 of predicted pair	
Systematic Name	Standard Name	Systematic Name	Standard Name
YDR176W	NGG1	YJL140W	RPB4
YLR337C	VRP1	YOR308C	SNU66
YOR035C	SHE4	YDR207C	UME6
YJL176C	SWI3	YER161C	SPT2
YBL008W	HIR1	YDL002C	NHP10
YML016C	PPZ1	YBR107C	IML3
YPL253C	VIK1	YPL152W	RRD2
YLR386W	VAC14	YER162C	RAD4
YFL023W	BUD27	YOR035C	SHE4
YMR116C	ASC1	YMR205C	PFK2
YBR030W	YBR030W	YKR035W-A	DID2
YOR083W	WHI5	YPR120C	CLB5
YLR360W	VPS38	YGL200C	EMP24
YDL184C	RPL41A	YER040W	GLN3
YKR020W	VPS51	YNL107W	YAF9
YDL006W	PTC1	YGR105W	VMA21
YLR093C	NYV1	YOL081W	IRA2
YDL020C	RPN4	YMR224C	MRE11
YNL147W	LSM7	YOL081W	IRA2
YDR176W	NGG1	YLR182W	SWI6
YGL200C	EMP24	YOR035C	SHE4
YHR178W	STB5	YBR274W	CHK1
YPL246C	RBD2	YKL068W	NUP100
YOR001W	RRP6	YKL213C	DOA1
YGR104C	SRB5	YBR089C-A	NHP6B
YNL218W	MGS1	YMR284W	YKU70
YHR060W	VMA22	YAL002W	VPS8
YPL046C	ELC1	YER059W	PCL6
YOR106W	VAM3	YOL018C	TLG2

Predictions at threshold level 0.80 continued...			
Gene1 of predicted pair		Gene2 of predicted pair	
Systematic Name	Standard Name	Systematic Name	Standard Name
YMR273C	ZDS1	YBR009C	HHF1
YDR383C	NKP1	YNL248C	RPA49
YBR200W	BEM1	YJL187C	SWE1
YGR019W	UGA1	YHR012W	VPS29
YOR337W	TEA1	YDR477W	SNF1
YGL212W	VAM7	YER040W	GLN3
YKR035W-A	DID2	YPL195W	APL5
YPL024W	RMI1	YBR278W	DPB3
YDL155W	CLB3	YGL124C	MON1
YNL325C	FIG4	YGL212W	VAM7
YEL065W	SIT1	YFR031C-A	RPL2A
YDR137W	RGP1	YGL124C	MON1
YER116C	SLX8	YDR076W	RAD55
YBR127C	VMA2	YKL080W	VMA5
YDL116W	NUP84	YML016C	PPZ1
YLR083C	EMP70	YDR202C	RAV2
YGL054C	ERV14	YJR060W	CBF1
YDR363W-A	SEM1	YDL226C	GCS1
YLR095C	IOC2	YOR337W	TEA1
YNL325C	FIG4	YDL184C	RPL41A
YIL053W	RHR2	YOR043W	WHI2
YPL152W	RRD2	YPR046W	MCM16
YOR080W	DIA2	YLR350W	ORM2
YMR284W	YKU70	YPL022W	RAD1
YBR215W	HPC2	YBR195C	MSI1
YDL116W	NUP84	YLR135W	SLX4
YLR386W	VAC14	YDR289C	RTT103
YNL087W	TCB2	YPR046W	MCM16
YGL078C	DBP3	YOR270C	VPH1

Predictions at threshold level 0.80 continued...			
Gene1 of predicted pair		Gene2 of predicted pair	
Systematic Name	Standard Name	Systematic Name	Standard Name
YER115C	SPR6	YNL199C	GCR2
YER144C	UBP5	YFR036W	CDC26
YLR018C	POM34	YLR079W	SIC1
YKR001C	VPS1	YPL161C	BEM4
YKL032C	IXR1	YOL081W	IRA2
YGR104C	SRB5	YLR399C	BDF1
YLR386W	VAC14	YJL172W	CPS1
YPL195W	APL5	YNR032W	PPG1
YJR059W	PTK2	YMR153W	NUP53
YGL175C	SAE2	YJR090C	GRR1
YMR004W	MVP1	YER120W	SCS2
YPL022W	RAD1	YDR363W-A	SEM1
YIL034C	CAP2	YDR318W	MCM21
YJR063W	RPA12	YBR289W	SNF5
YPL042C	SSN3	YFR010W	UBP6
YEL044W	IES6	YDL223C	HBT1
YIL008W	URM1	YOL067C	RTG1
YML115C	VAN1	YLR032W	RAD5
YBL103C	RTG3	YOL108C	INO4
YHR012W	VPS29	YKR030W	GMH1
YML095C	RAD10	YPR119W	CLB2
YGR104C	SRB5	YPL042C	SSN3
YDR202C	RAV2	YPL195W	APL5
YPL022W	RAD1	YJL058C	BIT61
YLR006C	SSK1	YNR031C	SSK2
YLR447C	VMA6	YER072W	VTC1
YFR010W	UBP6	YNL087W	TCB2
YLR441C	RPS1A	YDL083C	RPS16B
YER096W	SHC1	YPL256C	CLN2

Predictions at threshold level 0.80 continued...			
Gene1 of predicted pair		Gene2 of predicted pair	
Systematic Name	Standard Name	Systematic Name	Standard Name
YDL184C	RPL41A	YNL041C	COG6
YKL116C	PRR1	YGL115W	SNF4
YOR066W	MSA1	YMR230W	RPS10B
YHR115C	DMA1	YGL252C	RTG2
YJR056C	YJR056C	YMR133W	REC114
YER114C	BOI2	YBL085W	BOI1
YPR018W	RLF2	YLR032W	RAD5
YLR360W	VPS38	YHR060W	VMA22
YJL154C	VPS35	YDR153C	ENT5
YBR195C	MSI1	YNL183C	NPR1
YNL291C	MID1	YDR153C	ENT5
YEL065W	SIT1	YBR036C	CSG2
YOR145C	PNO1	YHR081W	LRP1
YIL076W	SEC28	YNL087W	TCB2
YER161C	SPT2	YMR038C	CCS1
YIL034C	CAP2	YKL213C	DOA1
YLR119W	SRN2	YNR006W	VPS27
YJL060W	BNA3	YGL003C	CDH1
YMR224C	MRE11	YGL003C	CDH1
YLR386W	VAC14	YJR135C	MCM22
YPR051W	MAK3	YMR054W	STV1
YHR200W	RPN10	YFL007W	BLM10
YJL187C	SWE1	YOL090W	MSH2
YLR288C	MEC3	YOR039W	CKB2
YOR001W	RRP6	YLR350W	ORM2
YKL032C	IXR1	YBL072C	RPS8A
YAL024C	LTE1	YNL107W	YAF9
YNL307C	MCK1	YER044C	ERG28
YDR202C	RAV2	YKR001C	VPS1

Predictions at threshold level 0.80 continued...			
Gene1 of predicted pair		Gene2 of predicted pair	
Systematic Name	Standard Name	Systematic Name	Standard Name
YML071C	COG8	YER040W	GLN3
YKL010C	UFD4	YGL200C	EMP24
YNL087W	TCB2	YNL076W	MKS1
YGL163C	RAD54	YIL103W	DPH1
YDL185W	TFP1	YKL080W	VMA5
YLR150W	STM1	YPR120C	CLB5
YGR135W	PRE9	YGL031C	RPL24A
YER116C	SLX8	YMR231W	PEP5
YDR137W	RGP1	YPR030W	CSR2
YDR225W	HTA1	YKL101W	HSL1
YLR148W	PEP3	YOR036W	PEP12
YNL236W	SIN4	YOR141C	ARP8
YBL008W	HIR1	YPR046W	MCM16
YER162C	RAD4	YMR230W	RPS10B
YJR106W	ECM27	YOR071C	THI71
YPL115C	BEM3	YPL256C	CLN2
YDR146C	SWI5	YOR038C	HIR2
YKL048C	ELM1	YGL252C	RTG2
YPL057C	SUR1	YDL083C	RPS16B
YMR172W	HOT1	YOR132W	VPS17
YPR101W	SNT309	YBR114W	RAD16
YPL115C	BEM3	YGL253W	HXK2
YIL159W	BNR1	YFR036W	CDC26
YPL045W	VPS16	YAL002W	VPS8
YDR137W	RGP1	YDL223C	HBT1
YLR288C	MEC3	YDL002C	NHP10
YGL025C	PGD1	YHR200W	RPN10
YDL155W	CLB3	YDR155C	CPR1
YBR041W	FAT1	YJL183W	MNN11

Predictions at threshold level 0.80 continued...			
Gene1 of predicted pair		Gene2 of predicted pair	
Systematic Name	Standard Name	Systematic Name	Standard Name
YDR507C	GIN4	YBL087C	RPL23A
YNL243W	SLA2	YIL002C	INP51
YER144C	UBP5	YOL111C	MDY2
YBR194W	SOY1	YEL013W	VAC8
YKL010C	UFD4	YLR425W	TUS1
YDR443C	SSN2	YOR014W	RTS1
YML016C	PPZ1	YHR108W	GGA2
YLR119W	SRN2	YKR035W-A	DID2
YBR200W	BEM1	YOR212W	STE4
YNL020C	ARK1	YCR088W	ABP1
YJL190C	RPS22A	YAL002W	VPS8
YML038C	YMD8	YEL053C	MAK10
YLR386W	VAC14	YDR264C	AKR1
YMR142C	RPL13B	YDL192W	ARF1
YPL115C	BEM3	YJR135C	MCM22
YFR031C-A	RPL2A	YHL031C	GOS1
YOR112W	CEX1	YDL006W	PTC1
YPL253C	VIK1	YLR182W	SWI6
YLR386W	VAC14	YOR043W	WHI2
YKR061W	KTR2	YDR289C	RTT103
YNL068C	FKH2	YHR115C	DMA1
YGL078C	DBP3	YOL111C	MDY2
YDR469W	SDC1	YNL107W	YAF9
YBR217W	ATG12	YBR036C	CSG2
YPL234C	TFP3	YNL051W	COG5
YPL246C	RBD2	YML057W	CMP2
YDR261C	EXG2	YER122C	GLO3
YDR108W	GSG1	YMR216C	SKY1
YDL223C	HBT1	YOR312C	RPL20B

Predictions at threshold level 0.80 continued...			
**Gene1 of predicted pair**		**Gene2 of predicted pair**	
**Systematic Name**	**Standard Name**	**Systematic Name**	**Standard Name**
YJL053W	PEP8	YHR060W	VMA22
YKL032C	IXR1	YHR111W	UBA4
YGR217W	CCH1	YBR131W	CCZ1
YHR134W	WSS1	YLR386W	VAC14
YPL057C	SUR1	YDR310C	SUM1
YDL184C	RPL41A	YLR441C	RPS1A
YDL077C	VAM6	YAL002W	VPS8
YPL256C	CLN2	YJL187C	SWE1
YJL172W	CPS1	YEL060C	PRB1
YNL041C	COG6	YHR081W	LRP1
YBR171W	SEC66	YOR358W	HAP5
YBR112C	CYC8	YNL167C	SKO1
YLL007C	YLL007C	YGR108W	CLB1
YNL069C	RPL16B	YJL004C	SYS1
YOL090W	MSH2	YOR368W	RAD17
YLR079W	SIC1	YGL252C	RTG2
YLR337C	VRP1	YPL234C	TFP3
YFR036W	CDC26	YBL025W	RRN10
YMR116C	ASC1	YOL030W	GAS5
YKR061W	KTR2	YLR254C	NDL1
YJL129C	TRK1	YGR262C	BUD32
YFR031C-A	RPL2A	YDL184C	RPL41A
YKR001C	VPS1	YOL018C	TLG2
YJR135C	MCM22	YDR183W	PLP1
YJR063W	RPA12	YJR135C	MCM22
YBR217W	ATG12	YDR207C	UME6
YIL034C	CAP2	YDL020C	RPN4
YHR200W	RPN10	YIL008W	URM1
YMR224C	MRE11	YOR368W	RAD17

Predictions at threshold level 0.80 continued...			
Gene1 of predicted pair		Gene2 of predicted pair	
Systematic Name	Standard Name	Systematic Name	Standard Name
YKR001C	VPS1	YKR020W	VPS51
YPR017C	DSS4	YER120W	SCS2
YGL078C	DBP3	YOR066W	MSA1
YJL124C	LSM1	YKL048C	ELM1
YHR156C	LIN1	YML115C	VAN1
YKL048C	ELM1	YER162C	RAD4
YNR072W	HXT17	YPL195W	APL5
YDR439W	LRS4	YKL048C	ELM1
YFR010W	UBP6	YMR153W	NUP53
YHR156C	LIN1	YOR035C	SHE4
YMR224C	MRE11	YER044C	ERG28
YOR106W	VAM3	YDR207C	UME6
YHR200W	RPN10	YMR311C	GLC8
YKL032C	IXR1	YKR001C	VPS1
YDL059C	RAD59	YHR010W	RPL27A
YMR055C	BUB2	YJR060W	CBF1
YOR127W	RGA1	YPL256C	CLN2
YLR386W	VAC14	YNL079C	TPM1
YER116C	SLX8	YOR043W	WHI2
YML103C	NUP188	YML112W	CTK3
YDR076W	RAD55	YML028W	TSA1
YOR195W	SLK19	YGL163C	RAD54
YDR383C	NKP1	YKL048C	ELM1
YLR371W	ROM2	YER040W	GLN3
YOR106W	VAM3	YGL054C	ERV14
YKR057W	RPS21A	YOR275C	RIM20
YPR084W	YPR084W	YLR309C	IMH1
YGL078C	DBP3	YML115C	VAN1
YDR507C	GIN4	YDR379W	RGA2

Predictions at threshold level 0.80 continued...			
Gene1 of predicted pair		Gene2 of predicted pair	
Systematic Name	Standard Name	Systematic Name	Standard Name
YGL054C	ERV14	YLR309C	IMH1
YMR179W	SPT21	YJL124C	LSM1
YLR018C	POM34	YER116C	SLX8
YPR084W	YPR084W	YFL013C	IES1
YML016C	PPZ1	YPL018W	CTF19
YGR159C	NSR1	YPL161C	BEM4
YDR477W	SNF1	YJL095W	BCK1
YER016W	BIM1	YGL094C	PAN2
YKR101W	SIR1	YNL031C	HHT2
YIL053W	RHR2	YDR137W	RGP1
YCR020C-A	MAK31	YOR112W	CEX1
YFL023W	BUD27	YER122C	GLO3
YNR052C	POP2	YDR289C	RTT103
YOR140W	SFL1	YEL036C	ANP1
YNL079C	TPM1	YOL110W	SHR5
YER116C	SLX8	YCR086W	CSM1
YDR310C	SUM1	YPR032W	SRO7
YPR018W	RLF2	YFR010W	UBP6
YOR001W	RRP6	YDL047W	SIT4
YML115C	VAN1	YNL032W	SIW14
YOR080W	DIA2	YMR038C	CCS1
YDL020C	RPN4	YBR108W	YBR108W
YNR052C	POP2	YJL141C	YAK1
YOR127W	RGA1	YJR060W	CBF1
YBR107C	IML3	YHR013C	ARD1
YDL006W	PTC1	YHR158C	KEL1
YBR173C	UMP1	YLR452C	SST2
YDL160C	DHH1	YJL124C	LSM1
YNL032W	SIW14	YHR081W	LRP1

Predictions at threshold level 0.80 continued...			
Gene1 of predicted pair		Gene2 of predicted pair	
Systematic Name	Standard Name	Systematic Name	Standard Name
YLR386W	VAC14	YNL041C	COG6
YDR383C	NKP1	YOR195W	SLK19
YBL079W	NUP170	YJR090C	GRR1
YDR156W	RPA14	YGL252C	RTG2
YML062C	MFT1	YOL145C	CTR9
YPL234C	TFP3	YML001W	YPT7
YDR439W	LRS4	YAR014C	BUD14
YHR203C	RPS4B	YER102W	RPS8B
YBR289W	SNF5	YNL236W	SIN4
YLR234W	TOP3	YJR140C	HIR3
YDR477W	SNF1	YCR084C	TUP1
YER144C	UBP5	YER044C	ERG28
YPR032W	SRO7	YDR207C	UME6
YGR217W	CCH1	YJL117W	PHO86
YBR173C	UMP1	YLR320W	MMS22
YLR288C	MEC3	YJR140C	HIR3
YKL116C	PRR1	YBR108W	YBR108W
YLR381W	CTF3	YBL021C	HAP3
YBR108W	YBR108W	YOR284W	HUA2
YLR360W	VPS38	YGL095C	VPS45
YDL184C	RPL41A	YEL060C	PRB1
YER144C	UBP5	YDR310C	SUM1
YDR192C	NUP42	YML103C	NUP188
YDR108W	GSG1	YJR040W	GEF1
YGL003C	CDH1	YLR180W	SAM1
YHR012W	VPS29	YDR207C	UME6
YLR148W	PEP3	YKR001C	VPS1
YEL051W	VMA8	YNL127W	FAR11
YMR224C	MRE11	YBR173C	UMP1

Predictions at threshold level 0.80 continued...			
Gene1 of predicted pair		Gene2 of predicted pair	
Systematic Name	Standard Name	Systematic Name	Standard Name
YER122C	GLO3	YLR180W	SAM1
YDL223C	HBT1	YJL183W	MNN11
YDR456W	NHX1	YER049W	TPA1
YKL025C	PAN3	YLL007C	YLL007C
YDL160C	DHH1	YKR059W	TIF1
YGL115W	SNF4	YJR084W	CSN12
YGL147C	RPL9A	YMR133W	REC114
YGR188C	BUB1	YDR310C	SUM1
YER162C	RAD4	YNL127W	FAR11
YBR108W	YBR108W	YML001W	YPT7
YHR039C-A	VMA10	YPL195W	APL5
YHR013C	ARD1	YAL002W	VPS8
YBL051C	PIN4	YML095C	RAD10
YER116C	SLX8	YPL195W	APL5
YBR131W	CCZ1	YOR275C	RIM20
YJL095W	BCK1	YEL060C	PRB1
YGR104C	SRB5	YFR034C	PHO4
YOR039W	CKB2	YBR200W	BEM1
YOR270C	VPH1	YJR084W	CSN12
YDR490C	PKH1	YKL126W	YPK1
YPR017C	DSS4	YOR132W	VPS17
YIL076W	SEC28	YCR077C	PAT1
YOL054W	PSH1	YLR182W	SWI6
YDR225W	HTA1	YBR107C	IML3
YMR052W	FAR3	YPR119W	CLB2
YNL068C	FKH2	YDR264C	AKR1
YHR081W	LRP1	YBR036C	CSG2
YEL065W	SIT1	YLR386W	VAC14
YGL163C	RAD54	YDR183W	PLP1

Predictions at threshold level 0.80 continued...			
Gene1 of predicted pair		Gene2 of predicted pair	
Systematic Name	Standard Name	Systematic Name	Standard Name
YDR129C	SAC6	YLR425W	TUS1
YNL127W	FAR11	YNL107W	YAF9
YBR127C	VMA2	YDR323C	PEP7
YLR148W	PEP3	YGR241C	YAP1802
YDR439W	LRS4	YLR315W	NKP2
YOR195W	SLK19	YDL013W	SLX5
YGL025C	PGD1	YJL183W	MNN11
YNL068C	FKH2	YOR127W	RGA1
YMR133W	REC114	YOL081W	IRA2
YDR225W	HTA1	YDR448W	ADA2
YGR019W	UGA1	YEL056W	HAT2
YGR270W	YTA7	YDL155W	CLB3
YGR249W	MGA1	YPR036W	VMA13
YKL010C	UFD4	YKL006W	RPL14A
YPL161C	BEM4	YML071C	COG8
YDR156W	RPA14	YOR195W	SLK19
YBR108W	YBR108W	YPL018W	CTF19
YPR084W	YPR084W	YDR310C	SUM1
YGL147C	RPL9A	YOL111C	MDY2
YER122C	GLO3	YMR214W	SCJ1
YLR170C	APS1	YJR058C	APS2
YIL159W	BNR1	YMR230W	RPS10B
YNL215W	IES2	YPL057C	SUR1
YPL152W	RRD2	YHR115C	DMA1
YOR337W	TEA1	YMR133W	REC114
YDL083C	RPS16B	YDR310C	SUM1
YLR386W	VAC14	YBR171W	SEC66
YIL148W	RPL40A	YPL256C	CLN2
YPR051W	MAK3	YOL067C	RTG1

Predictions at threshold level 0.80 continued...			
Gene1 of predicted pair		Gene2 of predicted pair	
Systematic Name	Standard Name	Systematic Name	Standard Name
YLR360W	VPS38	YIL076W	SEC28
YMR026C	PEX12	YJL095W	BCK1
YMR224C	MRE11	YNL087W	TCB2
YNL291C	MID1	YDL006W	PTC1
YJR084W	CSN12	YBR181C	RPS6B
YDR153C	ENT5	YER122C	GLO3
YER116C	SLX8	YBL079W	NUP170
YMR055C	BUB2	YLR370C	ARC18
YGR108W	CLB1	YEL050C	RML2
YEL056W	HAT2	YHR114W	BZZ1
YDL160C	DHH1	YPL024W	RMI1
YDR443C	SSN2	YHR200W	RPN10
YLR079W	SIC1	YJL095W	BCK1
YJR060W	CBF1	YOL081W	IRA2
YPL057C	SUR1	YOR014W	RTS1
YNL031C	HHT2	YOR141C	ARP8
YKL160W	ELF1	YJR060W	CBF1
YPL024W	RMI1	YHR156C	LIN1
YLR288C	MEC3	YHR039C-A	VMA10
YML001W	YPT7	YBL025W	RRN10
YKL057C	NUP120	YER105C	NUP157
YGR261C	APL6	YOR112W	CEX1
YJR060W	CBF1	YJR135C	MCM22
YDR202C	RAV2	YGL054C	ERV14
YJL154C	VPS35	YLR360W	VPS38
YDR318W	MCM21	YBR114W	RAD16
YML012W	ERV25	YJL004C	SYS1
YEL009C	GCN4	YOL051W	GAL11
YNL201C	PSY2	YKL139W	CTK1

Predictions at threshold level 0.80 continued...			
**Gene1 of predicted pair**		**Gene2 of predicted pair**	
**Systematic Name**	**Standard Name**	**Systematic Name**	**Standard Name**
YGL147C	RPL9A	YDR477W	SNF1
YPR052C	NHP6A	YKL139W	CTK1
YMR224C	MRE11	YDR076W	RAD55
YLR090W	XDJ1	YMR311C	GLC8
YKL181W	PRS1	YOR043W	WHI2
YJL183W	MNN11	YER040W	GLN3
YLR191W	PEX13	YAR042W	SWH1
YGL215W	CLG1	YDR137W	RGP1
YHR156C	LIN1	YLR441C	RPS1A
YMR153W	NUP53	YEL060C	PRB1
YJL127C	SPT10	YDR200C	VPS64
YJL124C	LSM1	YPL057C	SUR1
YHR016C	YSC84	YKR030W	GMH1
YLR441C	RPS1A	YDR137W	RGP1
YDL185W	TFP1	YOL081W	IRA2
YDR363W-A	SEM1	YJL140W	RPB4
YPL253C	VIK1	YNL248C	RPA49
YMR127C	SAS2	YJR032W	CPR7
YBR258C	SHG1	YPL161C	BEM4
YMR016C	SOK2	YMR311C	GLC8
YFR010W	UBP6	YLR032W	RAD5
YLR441C	RPS1A	YJL183W	MNN11
YBR200W	BEM1	YGL025C	PGD1
YOR312C	RPL20B	YBL025W	RRN10
YNL307C	MCK1	YPL195W	APL5
YML071C	COG8	YOR035C	SHE4
YNL330C	RPD3	YBR089C-A	NHP6B
YLR095C	IOC2	YLR094C	GIS3
YJR060W	CBF1	YFR030W	MET10

Predictions at threshold level 0.80 continued...			
Gene1 of predicted pair		Gene2 of predicted pair	
Systematic Name	Standard Name	Systematic Name	Standard Name
YEL027W	CUP5	YDL185W	TFP1
YPR171W	BSP1	YPL050C	MNN9
YDR293C	SSD1	YAL002W	VPS8
YBR200W	BEM1	YEL036C	ANP1
YDR310C	SUM1	YLR350W	ORM2
YOR337W	TEA1	YDR310C	SUM1
YOR001W	RRP6	YDR245W	MNN10
YNL215W	IES2	YGL161C	YIP5
YMR199W	CLN1	YLR452C	SST2
YJL006C	CTK2	YCR086W	CSM1
YEL065W	SIT1	YER139C	YER139C
YDR507C	GIN4	YKL032C	IXR1
YGL174W	BUD13	YBR036C	CSG2
YNL069C	RPL16B	YHR060W	VMA22
YLR288C	MEC3	YCR077C	PAT1
YBR173C	UMP1	YMR153W	NUP53
YDL020C	RPN4	YMR172W	HOT1
YAL024C	LTE1	YMR142C	RPL13B
YFR010W	UBP6	YDL077C	VAM6
YDR443C	SSN2	YPL057C	SUR1
YEL053C	MAK10	YOL108C	INO4
YPL137C	GIP3	YBR280C	SAF1
YDL155W	CLB3	YOR014W	RTS1
YBL058W	SHP1	YDL192W	ARF1
YBR264C	YPT10	YKR014C	YPT52
YGR252W	GCN5	YOR038C	HIR2
YBL021C	HAP3	YGL253W	HXK2
YER116C	SLX8	YOL111C	MDY2
YLL039C	UBI4	YGR135W	PRE9

Predictions at threshold level 0.80 continued...			
Gene1 of predicted pair		Gene2 of predicted pair	
Systematic Name	Standard Name	Systematic Name	Standard Name
YHR178W	STB5	YER161C	SPT2
YML016C	PPZ1	YGL252C	RTG2
YOR069W	VPS5	YCL037C	SRO9
YGL178W	MPT5	YER068W	MOT2
YBR126C	TPS1	YKL126W	YPK1
YNR072W	HXT17	YKL048C	ELM1
YMR224C	MRE11	YPR030W	CSR2
YLR315W	NKP2	YGR188C	BUB1
YBR274W	CHK1	YDL059C	RAD59
YNL054W	VAC7	YPR120C	CLB5
YPR018W	RLF2	YGL087C	MMS2
YNL297C	MON2	YJR033C	RAV1
YBR030W	YBR030W	YNL076W	MKS1
YHR060W	VMA22	YGL003C	CDH1
YLR452C	SST2	YER139C	YER139C
YGR135W	PRE9	YFR036W	CDC26
YNR072W	HXT17	YDR310C	SUM1
YDL185W	TFP1	YPR119W	CLB2
YLR386W	VAC14	YOL111C	MDY2
YMR224C	MRE11	YMR153W	NUP53
YPL057C	SUR1	YGL237C	HAP2
YIL053W	RHR2	YMR142C	RPL13B
YDR335W	MSN5	YKL068W	NUP100
YOR039W	CKB2	YNL030W	HHF2
YBR111W-A	SUS1	YAR002W	NUP60
YMR198W	CIK1	YDR076W	RAD55
YPL001W	HAT1	YDR289C	RTT103
YJL006C	CTK2	YDR183W	PLP1
YEL053C	MAK10	YDL192W	ARF1

Predictions at threshold level 0.80 continued...			
Gene1 of predicted pair		Gene2 of predicted pair	
Systematic Name	Standard Name	Systematic Name	Standard Name
YJL172W	CPS1	YML001W	YPT7
YKR057W	RPS21A	YKR048C	NAP1
YDL184C	RPL41A	YOL121C	RPS19A
YLR192C	HCR1	YDR310C	SUM1
YJL124C	LSM1	YNL116W	DMA2
YJL176C	SWI3	YGR105W	VMA21
YDR080W	VPS41	YHR039C-A	VMA10
YHR073W	OSH3	YML103C	NUP188
YML112W	CTK3	YNL116W	DMA2
YNL243W	SLA2	YOR106W	VAM3
YIL156W	UBP7	YLR079W	SIC1
YJL124C	LSM1	YHR013C	ARD1
YDR484W	VPS52	YHR039C-A	VMA10
YBR093C	PHO5	YMR198W	CIK1
YBR020W	GAL1	YJR060W	CBF1
YDR443C	SSN2	YDR477W	SNF1
YOR132W	VPS17	YIL008W	URM1
YLR288C	MEC3	YML013W	UBX2
YKL139W	CTK1	YHR060W	VMA22
YJL151C	SNA3	YNL094W	APP1
YDR363W-A	SEM1	YPR046W	MCM16
YMR198W	CIK1	YNL236W	SIN4
YLR018C	POM34	YKL041W	VPS24
YOR039W	CKB2	YLR399C	BDF1
YOR066W	MSA1	YCR053W	THR4
YLR192C	HCR1	YGL025C	PGD1
YDR378C	LSM6	YHR115C	DMA1
YHR012W	VPS29	YPR032W	SRO7
YMR070W	MOT3	YBR127C	VMA2

Predictions at threshold level 0.80 continued...			
Gene1 of predicted pair		Gene2 of predicted pair	
Systematic Name	Standard Name	Systematic Name	Standard Name
YIL008W	URM1	YDR318W	MCM21
YLL007C	YLL007C	YGR019W	UGA1
YLR337C	VRP1	YLR061W	RPL22A
YLR090W	XDJ1	YEL060C	PRB1
YMR172W	HOT1	YLR292C	SEC72
YDR137W	RGP1	YKL080W	VMA5
YGR054W	YGR054W	YDR310C	SUM1
YLR119W	SRN2	YBR171W	SEC66
YDR443C	SSN2	YER049W	TPA1
YMR142C	RPL13B	YKL048C	ELM1
YIL076W	SEC28	YOL108C	INO4
YLR119W	SRN2	YOL081W	IRA2
YBR200W	BEM1	YMR214W	SCJ1
YGL066W	SGF73	YMR199W	CLN1
YMR095C	SNO1	YER122C	GLO3
YGL163C	RAD54	YDR207C	UME6
YJR140C	HIR3	YER161C	SPT2
YMR224C	MRE11	YMR230W	RPS10B
YMR224C	MRE11	YPL057C	SUR1
YDR443C	SSN2	YFR031C-A	RPL2A
YDR192C	NUP42	YER116C	SLX8
YGR072W	UPF3	YDR080W	VPS41
YPR017C	DSS4	YNL127W	FAR11
YHR060W	VMA22	YLR452C	SST2
YCL029C	BIK1	YGL190C	CDC55
YOR312C	RPL20B	YOR368W	RAD17
YNL069C	RPL16B	YPL120W	VPS30
YOR008C	SLG1	YER049W	TPA1
YPR120C	CLB5	YPL152W	RRD2

Predictions at threshold level 0.80 continued...			
Gene1 of predicted pair		Gene2 of predicted pair	
Systematic Name	Standard Name	Systematic Name	Standard Name
YPR051W	MAK3	YJL095W	BCK1
YLR192C	HCR1	YGR133W	PEX4
YFR030W	MET10	YJR135C	MCM22
YBR030W	YBR030W	YER044C	ERG28
YKL007W	CAP1	YOR208W	PTP2
YGL087C	MMS2	YMR038C	CCS1
YHR156C	LIN1	YBR171W	SEC66
YJL154C	VPS35	YPL037C	EGD1
YOR270C	VPH1	YPL057C	SUR1
YML117W	NAB6	YKL048C	ELM1
YBL102W	SFT2	YEL051W	VMA8
YBR290W	BSD2	YGL124C	MON1
YKL139W	CTK1	YBR200W	BEM1
YJR056C	YJR056C	YPR030W	CSR2
YNL201C	PSY2	YPR046W	MCM16
YPL161C	BEM4	YKL080W	VMA5
YIL053W	RHR2	YER116C	SLX8
YOR033C	EXO1	YLR313C	SPH1
YGR019W	UGA1	YDR155C	CPR1
YOR332W	VMA4	YBR200W	BEM1
YLR240W	VPS34	YJL053W	PEP8
YPL129W	TAF14	YGL246C	RAI1
YMR116C	ASC1	YGL212W	VAM7
YOR023C	AHC1	YDR183W	PLP1
YAL024C	LTE1	YAL002W	VPS8
YER110C	KAP123	YPL256C	CLN2
YMR224C	MRE11	YHR178W	STB5
YLR090W	XDJ1	YHR081W	LRP1
YPR018W	RLF2	YLR288C	MEC3

Predictions at threshold level 0.80 continued...			
Gene1 of predicted pair		Gene2 of predicted pair	
Systematic Name	Standard Name	Systematic Name	Standard Name
YHR060W	VMA22	YNL051W	COG5
YNL032W	SIW14	YPR030W	CSR2
YMR055C	BUB2	YHL002W	HSE1
YDR004W	RAD57	YJR140C	HIR3
YDR171W	HSP42	YNL236W	SIN4
YDR080W	VPS41	YDL077C	VAM6
YHR013C	ARD1	YHR115C	DMA1
YLR399C	BDF1	YIL103W	DPH1
YDR080W	VPS41	YMR054W	STV1
YML038C	YMD8	YPR032W	SRO7
YHR012W	VPS29	YER122C	GLO3
YOL126C	MDH2	YCR024C-A	PMP1
YPR084W	YPR084W	YOR127W	RGA1
YDR496C	PUF6	YDR101C	ARX1
YOR195W	SLK19	YIL033C	BCY1
YGL147C	RPL9A	YLR119W	SRN2
YLR079W	SIC1	YHR013C	ARD1
YGL147C	RPL9A	YKL126W	YPK1
YPL037C	EGD1	YDR261C	EXG2
YBL051C	PIN4	YDR254W	CHL4
YGR249W	MGA1	YDL192W	ARF1
YJL124C	LSM1	YGR188C	BUB1
YER044C	ERG28	YGL115W	SNF4
YFL013C	IES1	YNL107W	YAF9
YDL006W	PTC1	YKR030W	GMH1
YDR318W	MCM21	YBR036C	CSG2
YBR171W	SEC66	YPR046W	MCM16
YGL078C	DBP3	YGR159C	NSR1
YDR507C	GIN4	YKR057W	RPS21A

Predictions at threshold level 0.80 continued...			
Gene1 of predicted pair		Gene2 of predicted pair	
Systematic Name	Standard Name	Systematic Name	Standard Name
YPL024W	RMI1	YLR452C	SST2
YNL025C	SSN8	YNL107W	YAF9
YJL154C	VPS35	YLR386W	VAC14
YIL034C	CAP2	YIL008W	URM1
YBL021C	HAP3	YBR107C	IML3
YMR109W	MYO5	YGL253W	HXK2
YDR293C	SSD1	YGR108W	CLB1
YOL111C	MDY2	YEL053C	MAK10
YLR410W	VIP1	YJL187C	SWE1
YEL051W	VMA8	YKL080W	VMA5
YNL291C	MID1	YLR032W	RAD5
YNL058C	YNL058C	YMR230W	RPS10B
YNL206C	RTT106	YBR010W	HHT1
YLL007C	YLL007C	YMR172W	HOT1
YDR293C	SSD1	YPL180W	TCO89
YNL199C	GCR2	YBR260C	RGD1
YMR199W	CLN1	YHR010W	RPL27A
YEL053C	MAK10	YNL107W	YAF9
YMR055C	BUB2	YER162C	RAD4
YER144C	UBP5	YGL115W	SNF4
YPL115C	BEM3	YGL124C	MON1
YOR043W	WHI2	YDR318W	MCM21
YNL293W	MSB3	YDR310C	SUM1
YNL215W	IES2	YDR289C	RTT103
YDR129C	SAC6	YOR036W	PEP12
YKL025C	PAN3	YJL183W	MNN11
YMR198W	CIK1	YDR171W	HSP42
YIR002C	MPH1	YGL163C	RAD54
YPL152W	RRD2	YJL187C	SWE1

Predictions at threshold level 0.80 continued...			
Gene1 of predicted pair		Gene2 of predicted pair	
Systematic Name	Standard Name	Systematic Name	Standard Name
YNL238W	KEX2	YOR035C	SHE4
YCR020C-A	MAK31	YNL291C	MID1
YDL160C	DHH1	YGR252W	GCN5
YDR171W	HSP42	YGL003C	CDH1
YLR058C	SHM2	YMR055C	BUB2
YPL037C	EGD1	YKL048C	ELM1
YLR148W	PEP3	YLR240W	VPS34
YHR039C-A	VMA10	YLR370C	ARC18
YBR017C	KAP104	YDR335W	MSN5
YMR224C	MRE11	YAL002W	VPS8
YDR080W	VPS41	YMR231W	PEP5
YDL116W	NUP84	YKR072C	SIS2
YFR010W	UBP6	YBR280C	SAF1
YLR320W	MMS22	YBR019C	GAL10
YDR225W	HTA1	YPL248C	GAL4
YOR033C	EXO1	YFR010W	UBP6
YIL053W	RHR2	YLR386W	VAC14
YDR293C	SSD1	YKL119C	VPH2
YHR200W	RPN10	YGL163C	RAD54
YPR046W	MCM16	YML028W	TSA1
YOR290C	SNF2	YGL115W	SNF4
YPL024W	RMI1	YLR370C	ARC18
YNL307C	MCK1	YOR327C	SNC2
YCR081W	SRB8	YNL079C	TPM1
YGR108W	CLB1	YPL018W	CTF19
YPL045W	VPS16	YDL226C	GCS1
YHR156C	LIN1	YOL111C	MDY2
YBR083W	TEC1	YDL159W	STE7
YGL115W	SNF4	YPL106C	SSE1

Predictions at threshold level 0.80 continued...			
Gene1 of predicted pair		Gene2 of predicted pair	
Systematic Name	Standard Name	Systematic Name	Standard Name
YBR200W	BEM1	YGR134W	CAF130
YHR200W	RPN10	YMR214W	SCJ1
YPL024W	RMI1	YOR043W	WHI2
YLR423C	ATG17	YPR185W	ATG13
YGR081C	SLX9	YGL175C	SAE2
YGL060W	YBP2	YPL022W	RAD1
YBR215W	HPC2	YKL114C	APN1
YOR043W	WHI2	YPR120C	CLB5
YBR217W	ATG12	YMR230W	RPS10B
YER144C	UBP5	YOR327C	SNC2
YLR094C	GIS3	YER040W	GLN3
YOR195W	SLK19	YDL047W	SIT4
YIL076W	SEC28	YGL003C	CDH1
YOR270C	VPH1	YER162C	RAD4
YDR245W	MNN10	YPL253C	VIK1
YLR320W	MMS22	YOL121C	RPS19A
YML115C	VAN1	YJL095W	BCK1
YDR108W	GSG1	YGR188C	BUB1
YMR199W	CLN1	YJL095W	BCK1
YPL137C	GIP3	YOR327C	SNC2
YLR234W	TOP3	YJR084W	CSN12
YDR378C	LSM6	YKR020W	VPS51
YBR173C	UMP1	YJR056C	YJR056C
YFL023W	BUD27	YML001W	YPT7
YBR173C	UMP1	YDL184C	RPL41A
YMR142C	RPL13B	YLR441C	RPS1A
YAL042W	ERV46	YPR119W	CLB2
YDR456W	NHX1	YOR014W	RTS1
YBR108W	YBR108W	YER122C	GLO3

Predictions at threshold level 0.80 continued...			
Gene1 of predicted pair		Gene2 of predicted pair	
Systematic Name	Standard Name	Systematic Name	Standard Name
YPR036W	VMA13	YKR099W	BAS1
YIL103W	DPH1	YLR182W	SWI6
YDL116W	NUP84	YMR038C	CCS1
YLR079W	SIC1	YOR008C	SLG1
YGR209C	TRX2	YML028W	TSA1
YMR070W	MOT3	YDL161W	ENT1
YPL057C	SUR1	YEL013W	VAC8
YER161C	SPT2	YHR167W	THP2
YOR109W	INP53	YEL043W	YEL043W
YER001W	MNN1	YGL200C	EMP24
YBL102W	SFT2	YPL195W	APL5
YGL078C	DBP3	YER122C	GLO3
YHR012W	VPS29	YPR046W	MCM16
YGR097W	ASK10	YPL129W	TAF14
YBL058W	SHP1	YOR327C	SNC2
YNL309W	STB1	YMR052W	FAR3
YNL069C	RPL16B	YER114C	BOI2
YCR077C	PAT1	YNL107W	YAF9
YHR200W	RPN10	YPR120C	CLB5
YCR020C-A	MAK31	YJR040W	GEF1
YOR358W	HAP5	YJR032W	CPR7
YML038C	YMD8	YJL004C	SYS1
YGR262C	BUD32	YML056C	IMD4
YGL078C	DBP3	YDR358W	GGA1
YKL048C	ELM1	YCR086W	CSM1
YOR069W	VPS5	YNL051W	COG5
YPR046W	MCM16	YML071C	COG8
YBR030W	YBR030W	YCR053W	THR4
YIL034C	CAP2	YLR360W	VPS38

Predictions at threshold level 0.80 continued...			
Gene1 of predicted pair		Gene2 of predicted pair	
Systematic Name	Standard Name	Systematic Name	Standard Name
YBR173C	UMP1	YMR142C	RPL13B
YJL176C	SWI3	YOR312C	RPL20B
YDL059C	RAD59	YBL047C	EDE1
YBR108W	YBR108W	YJL190C	RPS22A
YJL124C	LSM1	YJR135C	MCM22
YPL256C	CLN2	YGR059W	SPR3
YNL016W	PUB1	YLR410W	VIP1
YGL095C	VPS45	YDL226C	GCS1
YEL013W	VAC8	YLR182W	SWI6
YBL008W	HIR1	YMR179W	SPT21
YHR156C	LIN1	YOL121C	RPS19A
YKL116C	PRR1	YCR053W	THR4
YLR102C	APC9	YDR260C	SWM1
YLR094C	GIS3	YER139C	YER139C
YBL021C	HAP3	YHR187W	IKI1
YLL039C	UBI4	YMR116C	ASC1
YJL154C	VPS35	YKR030W	GMH1
YGL066W	SGF73	YHR200W	RPN10
YMR142C	RPL13B	YJL095W	BCK1
YNL166C	BNI5	YJL095W	BCK1
YNL041C	COG6	YAR014C	BUD14
YPL137C	GIP3	YMR311C	GLC8
YLR337C	VRP1	YGR133W	PEX4
YGL115W	SNF4	YPL018W	CTF19
YKL007W	CAP1	YCR077C	PAT1
YHL031C	GOS1	YGL223C	COG1
YLR386W	VAC14	YER144C	UBP5
YDR245W	MNN10	YLR350W	ORM2
YKL048C	ELM1	YNL238W	KEX2

Predictions at threshold level 0.80 continued...			
**Gene1 of predicted pair**		**Gene2 of predicted pair**	
**Systematic Name**	**Standard Name**	**Systematic Name**	**Standard Name**
YLR119W	SRN2	YLR370C	ARC18
YLR058C	SHM2	YML095C	RAD10
YCL001W	RER1	YHR039C-A	VMA10
YGR184C	UBR1	YFR036W	CDC26
YPL253C	VIK1	YNL199C	GCR2
YOL051W	GAL11	YBR289W	SNF5
YOR112W	CEX1	YHL031C	GOS1
YDR443C	SSN2	YGL033W	HOP2
YLR381W	CTF3	YJR005W	APL1
YJR032W	CPR7	YDR289C	RTT103
YBL088C	TEL1	YDR253C	MET32
YNL058C	YNL058C	YLR370C	ARC18
YMR153W	NUP53	YBR171W	SEC66
YGL213C	SKI8	YLR398C	SKI2
YDR443C	SSN2	YMR026C	PEX12
YBL051C	PIN4	YPR040W	TIP41
YAL009W	SPO7	YMR165C	PAH1
YLR360W	VPS38	YNL079C	TPM1
YNL243W	SLA2	YDR245W	MNN10
YLR386W	VAC14	YLR094C	GIS3
YPL042C	SSN3	YPL106C	SSE1
YMR004W	MVP1	YDR207C	UME6
YPL057C	SUR1	YLR093C	NYV1
YLR360W	VPS38	YER049W	TPA1
YCR063W	BUD31	YJR050W	ISY1
YDR477W	SNF1	YDL223C	HBT1
YBR111W-A	SUS1	YHR167W	THP2
YMR172W	HOT1	YBR114W	RAD16
YMR202W	ERG2	YBR107C	IML3

Predictions at threshold level 0.80 continued...			
Gene1 of predicted pair		Gene2 of predicted pair	
Systematic Name	Standard Name	Systematic Name	Standard Name
YLR234W	TOP3	YMR198W	CIK1
YBL003C	HTA2	YGL241W	KAP114
YDR137W	RGP1	YGR105W	VMA21
YPL057C	SUR1	YPR046W	MCM16
YMR109W	MYO5	YGR270W	YTA7
YBL051C	PIN4	YMR109W	MYO5
YMR055C	BUB2	YML028W	TSA1
YBR112C	CYC8	YHL027W	RIM101
YJL137C	GLG2	YLR273C	PIG1
YMR273C	ZDS1	YGR059W	SPR3
YPL115C	BEM3	YGL038C	OCH1
YHL031C	GOS1	YEL053C	MAK10
YDR108W	GSG1	YNR032W	PPG1
YKL116C	PRR1	YJL183W	MNN11
YCL037C	SRO9	YER161C	SPT2
YOL090W	MSH2	YMR038C	CCS1
YDR254W	CHL4	YJL095W	BCK1
YKR072C	SIS2	YBR036C	CSG2
YOR195W	SLK19	YPL018W	CTF19
YML112W	CTK3	YHR115C	DMA1
YLR452C	SST2	YOR035C	SHE4
YFL047W	RGD2	YGR059W	SPR3
YGR239C	PEX21	YAR042W	SWH1
YPR084W	YPR084W	YHR012W	VPS29
YDR363W-A	SEM1	YNL229C	URE2
YBR107C	IML3	YDL013W	SLX5
YGR105W	VMA21	YOL018C	TLG2
YIL034C	CAP2	YHR114W	BZZ1
YNR052C	POP2	YGL175C	SAE2

Predictions at threshold level 0.80 continued...			
Gene1 of predicted pair		Gene2 of predicted pair	
Systematic Name	Standard Name	Systematic Name	Standard Name
YMR224C	MRE11	YGR184C	UBR1
YMR116C	ASC1	YLR320W	MMS22
YER116C	SLX8	YBR280C	SAF1
YHR156C	LIN1	YMR311C	GLC8
YER019C-A	SBH2	YGL226C-A	OST5
YGR081C	SLX9	YOR368W	RAD17
YDR004W	RAD57	YNL291C	MID1
YCR086W	CSM1	YDR207C	UME6
YKL126W	YPK1	YNL307C	MCK1
YBL051C	PIN4	YHR115C	DMA1
YIL034C	CAP2	YMR127C	SAS2
YDR318W	MCM21	YOL054W	PSH1
YDL116W	NUP84	YLR410W	VIP1
YPR017C	DSS4	YGL054C	ERV14
YPL024W	RMI1	YJR032W	CPR7
YGR261C	APL6	YJL004C	SYS1
YHR081W	LRP1	YJL183W	MNN11
YPL050C	MNN9	YPR154W	PIN3
YDR137W	RGP1	YGR270W	YTA7
YNL215W	IES2	YER122C	GLO3
YOR066W	MSA1	YDR310C	SUM1
YBR107C	IML3	YOR358W	HAP5
YIR005W	IST3	YHR115C	DMA1
YLR192C	HCR1	YMR133W	REC114
YNL054W	VAC7	YIR005W	IST3
YOR290C	SNF2	YCR053W	THR4
YML115C	VAN1	YGR188C	BUB1
YBR200W	BEM1	YPL195W	APL5
YBR073W	RDH54	YDR254W	CHL4

Predictions at threshold level 0.80 continued...			
Gene1 of predicted pair		Gene2 of predicted pair	
Systematic Name	Standard Name	Systematic Name	Standard Name
YHR013C	ARD1	YOL054W	PSH1
YKR035W-A	DID2	YOR327C	SNC2
YBR131W	CCZ1	YMR038C	CCS1
YNL087W	TCB2	YDL077C	VAM6
YML001W	YPT7	YOL018C	TLG2
YDL013W	SLX5	YJR135C	MCM22
YBR171W	SEC66	YJL095W	BCK1
YDR443C	SSN2	YAL024C	LTE1
YNL058C	YNL058C	YGL003C	CDH1
YHR060W	VMA22	YER122C	GLO3
YDL116W	NUP84	YLR079W	SIC1
YIL034C	CAP2	YDR144C	MKC7
YOR332W	VMA4	YPR119W	CLB2
YPR051W	MAK3	YBR107C	IML3
YJL172W	CPS1	YJL095W	BCK1
YOR112W	CEX1	YNR032W	PPG1
YJR031C	GEA1	YER122C	GLO3
YOR327C	SNC2	YBR280C	SAF1
YMR224C	MRE11	YML115C	VAN1
YJL029C	VPS53	YDL047W	SIT4
YGR108W	CLB1	YNL107W	YAF9
YGR249W	MGA1	YBR200W	BEM1
YKR001C	VPS1	YJR033C	RAV1
YLR371W	ROM2	YBR036C	CSG2
YLR381W	CTF3	YPL253C	VIK1
YPL057C	SUR1	YNL107W	YAF9
YGL215W	CLG1	YDR358W	GGA1
YMR198W	CIK1	YHR013C	ARD1
YML115C	VAN1	YJR056C	YJR056C

Predictions at threshold level 0.80 continued...			
Gene1 of predicted pair		Gene2 of predicted pair	
Systematic Name	Standard Name	Systematic Name	Standard Name
YNL087W	TCB2	YPL037C	EGD1
YOR275C	RIM20	YPL256C	CLN2
YOR132W	VPS17	YML038C	YMD8
YBR108W	YBR108W	YMR055C	BUB2
YPL161C	BEM4	YLR182W	SWI6
YOL126C	MDH2	YKL068W	NUP100
YDR383C	NKP1	YGR188C	BUB1
YGL147C	RPL9A	YMR095C	SNO1
YKR072C	SIS2	YDL059C	RAD59
YOR337W	TEA1	YER040W	GLN3
YPL195W	APL5	YBR019C	GAL10
YDR245W	MNN10	YCL037C	SRO9
YPL161C	BEM4	YOR014W	RTS1
YDR443C	SSN2	YEL060C	PRB1
YHR156C	LIN1	YPR036W	VMA13
YDR254W	CHL4	YOR014W	RTS1
YKL181W	PRS1	YHR030C	SLT2
YMR179W	SPT21	YDL155W	CLB3
YJL154C	VPS35	YDR207C	UME6
YNL236W	SIN4	YPR119W	CLB2
YOR106W	VAM3	YDR440W	DOT1
YLR102C	APC9	YGR135W	PRE9
YGR081C	SLX9	YDR207C	UME6
YIL159W	BNR1	YHR081W	LRP1
YER162C	RAD4	YOL018C	TLG2
YPL129W	TAF14	YMR201C	RAD14
YNR052C	POP2	YKL048C	ELM1
YBR194W	SOY1	YIR005W	IST3
YHR134W	WSS1	YNR052C	POP2

Predictions at threshold level 0.80 continued...			
Gene1 of predicted pair		Gene2 of predicted pair	
Systematic Name	Standard Name	Systematic Name	Standard Name
YPL001W	HAT1	YJL124C	LSM1
YFR010W	UBP6	YJL124C	LSM1
YBL047C	EDE1	YDL006W	PTC1
YNL209W	SSB2	YPL240C	HSP82
YNL106C	INP52	YLR254C	NDL1
YDR378C	LSM6	YBL047C	EDE1
YLR381W	CTF3	YLR254C	NDL1
YDR477W	SNF1	YER040W	GLN3
YJL127C	SPT10	YDR318W	MCM21
YJL172W	CPS1	YMR133W	REC114
YDR254W	CHL4	YJR005W	APL1
YBR274W	CHK1	YCR077C	PAT1
YAL024C	LTE1	YPL256C	CLN2
YLR090W	XDJ1	YHR156C	LIN1
YDR378C	LSM6	YER122C	GLO3
YDR443C	SSN2	YMR133W	REC114
YGL033W	HOP2	YPL057C	SUR1
YNL218W	MGS1	YJL124C	LSM1
YJL176C	SWI3	YJL095W	BCK1
YJR056C	YJR056C	YOR327C	SNC2
YGR072W	UPF3	YPL057C	SUR1
YBL102W	SFT2	YJL053W	PEP8
YLR320W	MMS22	YNL238W	KEX2
YGL133W	ITC1	YDR264C	AKR1
YER110C	KAP123	YML057W	CMP2
YLR386W	VAC14	YMR311C	GLC8
YMR153W	NUP53	YHR039C-A	VMA10
YBR009C	HHF1	YKR048C	NAP1
YKL007W	CAP1	YER114C	BOI2

Predictions at threshold level 0.80 continued...			
Gene1 of predicted pair		Gene2 of predicted pair	
Systematic Name	Standard Name	Systematic Name	Standard Name
YDR156W	RPA14	YLR381W	CTF3
YNL291C	MID1	YER122C	GLO3
YDL155W	CLB3	YMR022W	UBC7
YBL102W	SFT2	YHR060W	VMA22
YJL172W	CPS1	YKR099W	BAS1
YJR092W	BUD4	YPR122W	AXL1
YEL065W	SIT1	YNR072W	HXT17
YJL172W	CPS1	YBL025W	RRN10
YOL054W	PSH1	YNL225C	CNM67
YBR057C	MUM2	YEL044W	IES6
YNL297C	MON2	YHR039C-A	VMA10
YGL147C	RPL9A	YBR019C	GAL10
YEL009C	GCN4	YDR448W	ADA2
YGR261C	APL6	YLR093C	NYV1
YER143W	DDI1	YPR032W	SRO7
YOR270C	VPH1	YLR441C	RPS1A
YDR254W	CHL4	YLR315W	NKP2
YGL115W	SNF4	YNR032W	PPG1
YFR031C-A	RPL2A	YNL051W	COG5
YKL116C	PRR1	YLR370C	ARC18
YOR106W	VAM3	YDR202C	RAV2
YGL200C	EMP24	YPL213W	LEA1
YDL155W	CLB3	YOR141C	ARP8
YNL106C	INP52	YPL079W	RPL21B
YGL175C	SAE2	YJL187C	SWE1
YHL031C	GOS1	YKL213C	DOA1
YDR443C	SSN2	YHR081W	LRP1
YGR054W	YGR054W	YDL155W	CLB3
YJL140W	RPB4	YOR064C	YNG1

Predictions at threshold level 0.80 continued...			
Gene1 of predicted pair		Gene2 of predicted pair	
Systematic Name	Standard Name	Systematic Name	Standard Name
YDR309C	GIC2	YJL095W	BCK1
YOR127W	RGA1	YPL018W	CTF19
YDR439W	LRS4	YNL225C	CNM67
YLR337C	VRP1	YML097C	VPS9
YDL184C	RPL41A	YKR035W-A	DID2
YDR176W	NGG1	YKR048C	NAP1
YER116C	SLX8	YPR046W	MCM16
YAL024C	LTE1	YEL056W	HAT2
YIL053W	RHR2	YOR270C	VPH1
YEL009C	GCN4	YOL148C	SPT20
YAL024C	LTE1	YLR119W	SRN2
YLR360W	VPS38	YDR477W	SNF1
YMR202W	ERG2	YJR060W	CBF1
YOR270C	VPH1	YEL027W	CUP5
YMR224C	MRE11	YGL175C	SAE2
YMR127C	SAS2	YJL095W	BCK1
YKR001C	VPS1	YDL226C	GCS1
YML115C	VAN1	YDL184C	RPL41A
YDR335W	MSN5	YPL161C	BEM4
YMR205C	PFK2	YBL101C	ECM21
YHR039C-A	VMA10	YDR261C	EXG2
YJL176C	SWI3	YNL329C	PEX6
YFR010W	UBP6	YPL248C	GAL4
YJR063W	RPA12	YBR009C	HHF1
YNL087W	TCB2	YER139C	YER139C
YDL155W	CLB3	YNL079C	TPM1
YGL147C	RPL9A	YNL032W	SIW14
YLR386W	VAC14	YMR133W	REC114
YLR447C	VMA6	YNL238W	KEX2

Predictions at threshold level 0.80 continued...			
Gene1 of predicted pair		Gene2 of predicted pair	
Systematic Name	Standard Name	Systematic Name	Standard Name
YEL059C-A	SOM1	YMR035W	IMP2
YDL020C	RPN4	YOR023C	AHC1
YGL078C	DBP3	YPL057C	SUR1
YLR079W	SIC1	YMR273C	ZDS1
YDL013W	SLX5	YPL018W	CTF19
YBR200W	BEM1	YKL032C	IXR1
YML016C	PPZ1	YDL155W	CLB3
YKL126W	YPK1	YLR093C	NYV1
YBR171W	SEC66	YLR093C	NYV1
YLR447C	VMA6	YDR144C	MKC7
YDR245W	MNN10	YOR367W	SCP1
YDR245W	MNN10	YBL067C	UBP13
YDR071C	PAA1	YER068W	MOT2
YPL022W	RAD1	YJR090C	GRR1
YDR174W	HMO1	YBR073W	RDH54
YFR010W	UBP6	YMR167W	MLH1
YJL060W	BNA3	YJR135C	MCM22
YDL125C	HNT1	YDL047W	SIT4
YDR363W-A	SEM1	YGR232W	NAS6
YMR224C	MRE11	YGR275W	RTT102
YMR216C	SKY1	YPL120W	VPS30
YBL058W	SHP1	YPL195W	APL5
YIL159W	BNR1	YLR093C	NYV1
YLR337C	VRP1	YGL212W	VAM7
YHR200W	RPN10	YER162C	RAD4
YKL048C	ELM1	YMR311C	GLC8
YBR171W	SEC66	YMR214W	SCJ1
YBL103C	RTG3	YFR036W	CDC26
YKL160W	ELF1	YGR135W	PRE9

Predictions at threshold level 0.80 continued...			
Gene1 of predicted pair		Gene2 of predicted pair	
Systematic Name	Standard Name	Systematic Name	Standard Name
YGL078C	DBP3	YGL033W	HOP2
YKL025C	PAN3	YBR274W	CHK1
YLR018C	POM34	YGL016W	KAP122
YJR060W	CBF1	YPR046W	MCM16
YER161C	SPT2	YLR315W	NKP2
YOR132W	VPS17	YPR046W	MCM16
YLR095C	IOC2	YMR142C	RPL13B
YJL183W	MNN11	YER122C	GLO3
YBR173C	UMP1	YDR335W	MSN5
YIL076W	SEC28	YOR069W	VPS5
YDR477W	SNF1	YBR108W	YBR108W
YIL076W	SEC28	YLR309C	IMH1
YPL152W	RRD2	YOL081W	IRA2
YDR443C	SSN2	YLR093C	NYV1
YLR441C	RPS1A	YGL003C	CDH1
YNL058C	YNL058C	YML001W	YPT7
YBR133C	HSL7	YJL187C	SWE1
YLR192C	HCR1	YGL115W	SNF4
YGR184C	UBR1	YJR135C	MCM22
YLR337C	VRP1	YKL032C	IXR1
YKR072C	SIS2	YBR278W	DPB3
YJL127C	SPT10	YDR335W	MSN5
YOR038C	HIR2	YFR036W	CDC26
YML016C	PPZ1	YIL103W	DPH1
YNL041C	COG6	YMR311C	GLC8
YOR083W	WHI5	YOR195W	SLK19
YNL032W	SIW14	YER040W	GLN3
YDR320C	SWA2	YNL238W	KEX2
YNL229C	URE2	YJL095W	BCK1

Predictions at threshold level 0.80 continued...			
Gene1 of predicted pair		Gene2 of predicted pair	
Systematic Name	Standard Name	Systematic Name	Standard Name
YNL264C	PDR17	YGL200C	EMP24
YJR056C	YJR056C	YEL060C	PRB1
YJL053W	PEP8	YHL020C	OPI1
YLR206W	ENT2	YJR125C	ENT3
YNL236W	SIN4	YGL094C	PAN2
YOR270C	VPH1	YMR054W	STV1
YNR052C	POP2	YNL248C	RPA49
YPR051W	MAK3	YAL002W	VPS8
YDL006W	PTC1	YDL077C	VAM6
YBR128C	ATG14	YPL120W	VPS30
YCR020C-A	MAK31	YPR051W	MAK3
YDL232W	OST4	YIL162W	SUC2
YML103C	NUP188	YBR200W	BEM1
YPL161C	BEM4	YDL240W	LRG1
YGL200C	EMP24	YLR425W	TUS1
YPL050C	MNN9	YGL200C	EMP24
YDR174W	HMO1	YHR111W	UBA4
YEL043W	YEL043W	YAR014C	BUD14
YPL161C	BEM4	YOR327C	SNC2
YBR173C	UMP1	YPL253C	VIK1
YHR108W	GGA2	YHL011C	PRS3
YLR337C	VRP1	YGL135W	RPL1B
YKL139W	CTK1	YIR005W	IST3
YHR012W	VPS29	YMR311C	GLC8
YOR195W	SLK19	YLR410W	VIP1
YDL160C	DHH1	YCR077C	PAT1
YBR173C	UMP1	YER116C	SLX8
YBR274W	CHK1	YBR036C	CSG2
YDL020C	RPN4	YER110C	KAP123

Predictions at threshold level 0.80 continued...			
Gene1 of predicted pair		Gene2 of predicted pair	
Systematic Name	Standard Name	Systematic Name	Standard Name
YML062C	MFT1	YHR081W	LRP1
YMR026C	PEX12	YDR261C	EXG2
YOR195W	SLK19	YDR171W	HSP42
YFR036W	CDC26	YLR182W	SWI6
YPL018W	CTF19	YJR090C	GRR1
YAL024C	LTE1	YNL106C	INP52
YPL161C	BEM4	YJR060W	CBF1
YMR194W	RPL36A	YGL246C	RAI1
YMR026C	PEX12	YFR010W	UBP6
YMR109W	MYO5	YJL190C	RPS22A
YMR224C	MRE11	YNL238W	KEX2
YLR381W	CTF3	YOR014W	RTS1
YCL001W	RER1	YJL004C	SYS1
YDR080W	VPS41	YML001W	YPT7
YBR107C	IML3	YML028W	TSA1
YGR019W	UGA1	YNL107W	YAF9
YFL013C	IES1	YMR038C	CCS1
YNL135C	FPR1	YNL023C	FAP1
YKL117W	SBA1	YKL068W	NUP100
YKR001C	VPS1	YDR289C	RTT103
YFL033C	RIM15	YLR309C	IMH1
YDL232W	OST4	YEL036C	ANP1
YMR016C	SOK2	YDL083C	RPS16B
YBR171W	SEC66	YGR270W	YTA7
YPL018W	CTF19	YGL253W	HXK2
YHR134W	WSS1	YHR077C	NMD2
YDR108W	GSG1	YKR030W	GMH1
YHR039C-A	VMA10	YOL108C	INO4
YBR030W	YBR030W	YOL081W	IRA2

Predictions at threshold level 0.80 continued...			
Gene1 of predicted pair		Gene2 of predicted pair	
Systematic Name	Standard Name	Systematic Name	Standard Name
YLR216C	CPR6	YKL117W	SBA1
YML115C	VAN1	YBR171W	SEC66
YDR137W	RGP1	YML001W	YPT7
YDR176W	NGG1	YDR469W	SDC1
YKL135C	APL2	YOR036W	PEP12
YBR111W-A	SUS1	YOR001W	RRP6
YBR126C	TPS1	YPL037C	EGD1
YML038C	YMD8	YPL234C	TFP3
YBR171W	SEC66	YKL161C	YKL161C
YCR065W	HCM1	YOR308C	SNU66
YHR012W	VPS29	YBR114W	RAD16
YOR001W	RRP6	YOR141C	ARP8
YBR290W	BSD2	YMR127C	SAS2
YIL036W	CST6	YIR005W	IST3
YJR060W	CBF1	YJR033C	RAV1
YGL223C	COG1	YAL002W	VPS8
YJL176C	SWI3	YLR131C	ACE2
YGL060W	YBP2	YPL018W	CTF19
YER120W	SCS2	YNL051W	COG5
YOR112W	CEX1	YHR039C-A	VMA10
YBR030W	YBR030W	YLR320W	MMS22
YLR094C	GIS3	YLR370C	ARC18
YLR079W	SIC1	YOR368W	RAD17
YHR060W	VMA22	YLR309C	IMH1
YGL066W	SGF73	YKR048C	NAP1
YBR200W	BEM1	YOL121C	RPS19A
YBR030W	YBR030W	YGL124C	MON1
YBR030W	YBR030W	YOR270C	VPH1
YDL116W	NUP84	YBL079W	NUP170

Predictions at threshold level 0.80 continued...			
Gene1 of predicted pair		Gene2 of predicted pair	
Systematic Name	Standard Name	Systematic Name	Standard Name
YIL076W	SEC28	YER139C	YER139C
YMR198W	CIK1	YER114C	BOI2
YLR095C	IOC2	YMR230W	RPS10B
YGL147C	RPL9A	YMR016C	SOK2
YOR127W	RGA1	YNL229C	URE2
YDL116W	NUP84	YFL047W	RGD2
YDR477W	SNF1	YLR371W	ROM2
YLR452C	SST2	YDR261C	EXG2
YLR386W	VAC14	YGL019W	CKB1
YIL005W	EPS1	YPL106C	SSE1
YHR134W	WSS1	YMR038C	CCS1
YGR261C	APL6	YBL078C	ATG8
YDR195W	REF2	YER032W	FIR1
YCR088W	ABP1	YDR323C	PEP7
YBL058W	SHP1	YPL137C	GIP3
YLR360W	VPS38	YGL223C	COG1
YLR432W	IMD3	YML056C	IMD4
YGR108W	CLB1	YCR086W	CSM1
YPL057C	SUR1	YPL037C	EGD1
YMR198W	CIK1	YJL183W	MNN11
YOR083W	WHI5	YDL226C	GCS1
YML062C	MFT1	YER063W	THO1
YPL253C	VIK1	YNL307C	MCK1
YLR192C	HCR1	YDL192W	ARF1
YKL094W	YJU3	YKL140W	TGL1
YMR055C	BUB2	YLR447C	VMA6
YPL042C	SSN3	YDR477W	SNF1
YPL037C	EGD1	YHL020C	OPI1
YDL192W	ARF1	YNL076W	MKS1

Predictions at threshold level 0.80 continued...			
Gene1 of predicted pair		Gene2 of predicted pair	
Systematic Name	Standard Name	Systematic Name	Standard Name
YLR192C	HCR1	YBL047C	EDE1
YLR292C	SEC72	YKL048C	ELM1
YLL026W	HSP104	YGR275W	RTT102
YML071C	COG8	YOR327C	SNC2
YJR058C	APS2	YPL259C	APM1
YNL041C	COG6	YOL121C	RPS19A
YDR080W	VPS41	YDL192W	ARF1
YOR191W	RIS1	YGR071C	YGR071C
YOL018C	TLG2	YAR014C	BUD14
YBR195C	MSI1	YFR036W	CDC26
YBR057C	MUM2	YNL079C	TPM1
YPL024W	RMI1	YDL059C	RAD59
YMR127C	SAS2	YOR213C	SAS5
YMR026C	PEX12	YJL183W	MNN11
YKR072C	SIS2	YPR120C	CLB5
YAL010C	MDM10	YLR315W	NKP2
YDR363W-A	SEM1	YDL047W	SIT4
YDR335W	MSN5	YKL048C	ELM1
YDL116W	NUP84	YDR289C	RTT103
YNL325C	FIG4	YER040W	GLN3
YIL053W	RHR2	YIL159W	BNR1
YIL148W	RPL40A	YPL178W	CBC2
YER161C	SPT2	YLR182W	SWI6
YBL103C	RTG3	YBR036C	CSG2
YDR439W	LRS4	YKR061W	KTR2
YDL006W	PTC1	YNR032W	PPG1
YDL184C	RPL41A	YLR320W	MMS22
YGR072W	UPF3	YHL031C	GOS1
YML095C	RAD10	YHR115C	DMA1

Predictions at threshold level 0.80 continued...			
Gene1 of predicted pair		Gene2 of predicted pair	
Systematic Name	Standard Name	Systematic Name	Standard Name
YLR452C	SST2	YPL018W	CTF19
YMR153W	NUP53	YNL199C	GCR2
YGL078C	DBP3	YOL081W	IRA2
YNR052C	POP2	YOL081W	IRA2
YDR101C	ARX1	YOR312C	RPL20B
YJR059W	PTK2	YML057W	CMP2
YLR386W	VAC14	YNL116W	DMA2
YOR275C	RIM20	YDL155W	CLB3
YOL091W	SPO21	YDL226C	GCS1
YNR052C	POP2	YPR046W	MCM16
YLR386W	VAC14	YNL238W	KEX2
YPL042C	SSN3	YLR079W	SIC1
YPR046W	MCM16	YPL195W	APL5
YDR378C	LSM6	YDL155W	CLB3
YGL161C	YIP5	YPR119W	CLB2
YBR017C	KAP104	YBL079W	NUP170
YJL095W	BCK1	YGR188C	BUB1
YGL237C	HAP2	YGL003C	CDH1
YHR178W	STB5	YDR144C	MKC7
YDR108W	GSG1	YDR469W	SDC1
YLR192C	HCR1	YDR207C	UME6
YDL020C	RPN4	YDR076W	RAD55
YKL139W	CTK1	YNL236W	SIN4
YPR036W	VMA13	YKL080W	VMA5
YLR094C	GIS3	YML059C	NTE1
YDR097C	MSH6	YOR368W	RAD17
YBR107C	IML3	YDR289C	RTT103
YLR093C	NYV1	YMR152W	YIM1
YOR358W	HAP5	YDR254W	CHL4

Predictions at threshold level 0.80 continued...			
Gene1 of predicted pair		Gene2 of predicted pair	
Systematic Name	Standard Name	Systematic Name	Standard Name
YNL229C	URE2	YHR115C	DMA1
YML012W	ERV25	YNL041C	COG6
YBR108W	YBR108W	YPR046W	MCM16
YDR261C	EXG2	YMR152W	YIM1
YIL159W	BNR1	YER162C	RAD4
YDL047W	SIT4	YNL116W	DMA2
YHR079C	IRE1	YOR036W	PEP12
YFL033C	RIM15	YNL238W	KEX2
YML103C	NUP188	YLR386W	VAC14
YDR108W	GSG1	YNL051W	COG5
YOR358W	HAP5	YJL183W	MNN11
YKR099W	BAS1	YFR036W	CDC26
YMR224C	MRE11	YNL107W	YAF9
YKL006W	RPL14A	YLR425W	TUS1
YNL291C	MID1	YGR105W	VMA21
YMR016C	SOK2	YNL307C	MCK1
YNL201C	PSY2	YPL253C	VIK1
YDL101C	DUN1	YMR224C	MRE11
YJL176C	SWI3	YPL129W	TAF14
YER044C	ERG28	YER139C	YER139C
YGL078C	DBP3	YNL087W	TCB2
YHR205W	SCH9	YER111C	SWI4
YLR043C	TRX1	YML028W	TSA1
YLR234W	TOP3	YCR086W	CSM1
YDR004W	RAD57	YAL024C	LTE1
YKL139W	CTK1	YJR032W	CPR7
YFR010W	UBP6	YLR094C	GIS3
YLR452C	SST2	YHR039C-A	VMA10
YJL187C	SWE1	YGL019W	CKB1

Predictions at threshold level 0.80 continued...			
Gene1 of predicted pair		Gene2 of predicted pair	
Systematic Name	Standard Name	Systematic Name	Standard Name
YJL172W	CPS1	YER122C	GLO3
YMR095C	SNO1	YLR398C	SKI2
YML115C	VAN1	YDR484W	VPS52
YJR060W	CBF1	YAL002W	VPS8
YDR358W	GGA1	YGL124C	MON1
YBL103C	RTG3	YOR327C	SNC2
YLR441C	RPS1A	YER040W	GLN3
YNL069C	RPL16B	YJL053W	PEP8
YFL023W	BUD27	YOL081W	IRA2
YPL161C	BEM4	YML038C	YMD8
YBR173C	UMP1	YLR180W	SAM1
YDL184C	RPL41A	YDR137W	RGP1
YNL058C	YNL058C	YLR093C	NYV1
YGR261C	APL6	YOR069W	VPS5
YMR224C	MRE11	YBL025W	RRN10
YLR095C	IOC2	YNL325C	FIG4
YBR217W	ATG12	YNR007C	ATG3
YDR137W	RGP1	YGL003C	CDH1
YGL115W	SNF4	YHR012W	VPS29
YDR443C	SSN2	YOR140W	SFL1
YIL053W	RHR2	YPR046W	MCM16
YER116C	SLX8	YMR199W	CLN1
YOR001W	RRP6	YBR010W	HHT1
YMR142C	RPL13B	YPR046W	MCM16
YHR081W	LRP1	YBR280C	SAF1
YBR030W	YBR030W	YHR200W	RPN10
YJL176C	SWI3	YLR370C	ARC18
YJR056C	YJR056C	YJR084W	CSN12
YNL025C	SSN8	YLR315W	NKP2

Predictions at threshold level 0.80 continued...			
Gene1 of predicted pair		Gene2 of predicted pair	
Systematic Name	Standard Name	Systematic Name	Standard Name
YLR043C	TRX1	YPL195W	APL5
YER059W	PCL6	YOR028C	CIN5
YKL048C	ELM1	YMR152W	YIM1
YKR001C	VPS1	YNL238W	KEX2
YMR142C	RPL13B	YKL126W	YPK1
YIL156W	UBP7	YMR199W	CLN1
YPL057C	SUR1	YER139C	YER139C
YMR026C	PEX12	YDR358W	GGA1
YMR205C	PFK2	YJL095W	BCK1
YJL176C	SWI3	YLR192C	HCR1
YMR224C	MRE11	YDR477W	SNF1
YOR112W	CEX1	YDR080W	VPS41
YML103C	NUP188	YNL248C	RPA49
YDR318W	MCM21	YLR254C	NDL1
YLR396C	VPS33	YDL226C	GCS1
YLR360W	VPS38	YLR309C	IMH1
YDR202C	RAV2	YOR109W	INP53
YKL139W	CTK1	YNL248C	RPA49
YMR224C	MRE11	YMR026C	PEX12
YML095C	RAD10	YPL018W	CTF19
YDR335W	MSN5	YNL107W	YAF9
YMR199W	CLN1	YBR260C	RGD1
YNL218W	MGS1	YER116C	SLX8
YML016C	PPZ1	YKL161C	YKL161C
YEL022W	GEA2	YDL226C	GCS1
YLR452C	SST2	YLR371W	ROM2
YML097C	VPS9	YAL002W	VPS8
YNL025C	SSN8	YGR270W	YTA7
YGR159C	NSR1	YDR245W	MNN10

Predictions at threshold level 0.80 continued...			
Gene1 of predicted pair		Gene2 of predicted pair	
Systematic Name	Standard Name	Systematic Name	Standard Name
YJR056C	YJR056C	YLR371W	ROM2
YKL101W	HSL1	YNL116W	DMA2
YMR109W	MYO5	YDR318W	MCM21
YDR378C	LSM6	YLR315W	NKP2
YKL117W	SBA1	YMR125W	STO1
YBL072C	RPS8A	YKL041W	VPS24
YOR078W	BUD21	YHR203C	RPS4B
YNL288W	CAF40	YHR114W	BZZ1
YBR173C	UMP1	YMR152W	YIM1
YNL068C	FKH2	YLR102C	APC9
YBR173C	UMP1	YMR038C	CCS1
YJR140C	HIR3	YMR038C	CCS1
YIR005W	IST3	YOR308C	SNU66
YHL031C	GOS1	YER122C	GLO3
YDR129C	SAC6	YBL067C	UBP13
YOR078W	BUD21	YGR100W	MDR1
YLR090W	XDJ1	YLR094C	GIS3
YHL020C	OPI1	YGR105W	VMA21
YOR112W	CEX1	YEL051W	VMA8
YBR200W	BEM1	YKR099W	BAS1
YLR090W	XDJ1	YBR108W	YBR108W
YLR079W	SIC1	YDR456W	NHX1
YGL078C	DBP3	YLR094C	GIS3
YNL325C	FIG4	YER139C	YER139C
YDR264C	AKR1	YKL178C	STE3
YPL024W	RMI1	YGR184C	UBR1
YGL200C	EMP24	YCR077C	PAT1
YKL139W	CTK1	YBR171W	SEC66
YOR195W	SLK19	YKL110C	KTI12

Predictions at threshold level 0.80 continued...			
Gene1 of predicted pair		Gene2 of predicted pair	
Systematic Name	Standard Name	Systematic Name	Standard Name
YDR293C	SSD1	YLR120C	YPS1
YER116C	SLX8	YBR107C	IML3
YNL215W	IES2	YOR308C	SNU66
YNL214W	PEX17	YGR077C	PEX8
YDR293C	SSD1	YAR014C	BUD14
YMR142C	RPL13B	YMR230W	RPS10B
YBR041W	FAT1	YKR020W	VPS51
YDR293C	SSD1	YGL223C	COG1
YLR192C	HCR1	YBR200W	BEM1
YLR095C	IOC2	YOL111C	MDY2
YFR031C-A	RPL2A	YGR105W	VMA21
YEL065W	SIT1	YPL024W	RMI1
YJR056C	YJR056C	YER139C	YER139C
YJR032W	CPR7	YPL018W	CTF19
YBR009C	HHF1	YJL140W	RPB4
YKL139W	CTK1	YER044C	ERG28
YOR178C	GAC1	YLR258W	GSY2
YDR097C	MSH6	YNL291C	MID1
YEL015W	EDC3	YDR378C	LSM6
YEL013W	VAC8	YGR262C	BUD32
YGL133W	ITC1	YKR048C	NAP1
YGR249W	MGA1	YHR039C-A	VMA10
YCR077C	PAT1	YGL031C	RPL24A
YML016C	PPZ1	YJL138C	TIF2
YCL037C	SRO9	YDR153C	ENT5
YBL025W	RRN10	YDL192W	ARF1
YNL307C	MCK1	YDL155W	CLB3
YGL066W	SGF73	YDR507C	GIN4
YLR386W	VAC14	YML016C	PPZ1

Predictions at threshold level 0.80 continued...			
**Gene1 of predicted pair**		**Gene2 of predicted pair**	
**Systematic Name**	**Standard Name**	**Systematic Name**	**Standard Name**
YER161C	SPT2	YEL056W	HAT2
YHR084W	STE12	YPL042C	SSN3
YDL184C	RPL41A	YLR452C	SST2
YHR161C	YAP1801	YOR036W	PEP12
YEL065W	SIT1	YIL159W	BNR1
YDL020C	RPN4	YJR090C	GRR1
YDL116W	NUP84	YDR318W	MCM21
YLR435W	TSR2	YGL250W	RMR1
YNL106C	INP52	YPL057C	SUR1
YOR109W	INP53	YHR012W	VPS29
YNL106C	INP52	YLR410W	VIP1
YGL124C	MON1	YBR280C	SAF1
YFR031C-A	RPL2A	YDR207C	UME6
YML100W	TSL1	YMR261C	TPS3
YLR109W	AHP1	YHR111W	UBA4
YCR020C-A	MAK31	YNL069C	RPL16B
YEL009C	GCN4	YKL109W	HAP4
YAL010C	MDM10	YHR111W	UBA4
YBL003C	HTA2	YNL031C	HHT2
YNL206C	RTT106	YBR084C-A	RPL19A
YPL234C	TFP3	YAL002W	VPS8
YLR234W	TOP3	YLR150W	STM1
YNL215W	IES2	YCR086W	CSM1
YJL124C	LSM1	YGL003C	CDH1
YMR199W	CLN1	YKL117W	SBA1
YDR439W	LRS4	YNL107W	YAF9
YNR072W	HXT17	YPR030W	CSR2
YBR200W	BEM1	YLR410W	VIP1
YOR195W	SLK19	YML112W	CTK3

Predictions at threshold level 0.80 continued...			
Gene1 of predicted pair		Gene2 of predicted pair	
Systematic Name	Standard Name	Systematic Name	Standard Name
YNL032W	SIW14	YEL060C	PRB1
YOR375C	GDH1	YFR036W	CDC26
YFL023W	BUD27	YMR038C	CCS1
YDR293C	SSD1	YDR318W	MCM21
YGL066W	SGF73	YOL148C	SPT20
YPR120C	CLB5	YHL011C	PRS3
YGL025C	PGD1	YDR335W	MSN5
YHR178W	STB5	YGR270W	YTA7
YOL145C	CTR9	YOR106W	VAM3
YDR254W	CHL4	YGR188C	BUB1
YML001W	YPT7	YPL137C	GIP3
YIL053W	RHR2	YLR398C	SKI2
YLR381W	CTF3	YGL151W	NUT1
YER116C	SLX8	YDR484W	VPS52
YJL095W	BCK1	YMR311C	GLC8
YML001W	YPT7	YGL198W	YIP4
YJR140C	HIR3	YGL151W	NUT1
YDR156W	RPA14	YLR315W	NKP2
YBL025W	RRN10	YBR280C	SAF1
YHR134W	WSS1	YBR107C	IML3
YHR060W	VMA22	YER162C	RAD4
YNL309W	STB1	YMR199W	CLN1
YDR129C	SAC6	YKL048C	ELM1
YLR102C	APC9	YBR084C-A	RPL19A
YNL199C	GCR2	YER139C	YER139C
YNL307C	MCK1	YJR060W	CBF1
YCR020W-B	HTL1	YHR056C	RSC30
YLR090W	XDJ1	YPL137C	GIP3
YBL102W	SFT2	YMR216C	SKY1

Predictions at threshold level 0.80 continued...			
Gene1 of predicted pair		Gene2 of predicted pair	
Systematic Name	Standard Name	Systematic Name	Standard Name
YBL008W	HIR1	YOL081W	IRA2
YGL033W	HOP2	YOL081W	IRA2
YGL058W	RAD6	YHR056C	RSC30
YLR381W	CTF3	YKL101W	HSL1
YDL020C	RPN4	YDR310C	SUM1
YNL068C	FKH2	YKL048C	ELM1
YMR224C	MRE11	YKR035W-A	DID2
YLR288C	MEC3	YEL056W	HAT2
YKR014C	YPT52	YER074W-A	YOS1
YGR135W	PRE9	YNL107W	YAF9
YBR036C	CSG2	YBR161W	CSH1
YKL139W	CTK1	YLR432W	IMD3
YBL078C	ATG8	YPL195W	APL5
YIL159W	BNR1	YMR214W	SCJ1
YJL172W	CPS1	YHR200W	RPN10
YIL002C	INP51	YJL004C	SYS1
YCL001W	RER1	YML067C	ERV41
YBL102W	SFT2	YDR080W	VPS41
YJR135C	MCM22	YOL067C	RTG1
YMR026C	PEX12	YDR310C	SUM1
YGR270W	YTA7	YOR038C	HIR2
YOR080W	DIA2	YJR005W	APL1
YGR105W	VMA21	YKL041W	VPS24
YAL024C	LTE1	YLR192C	HCR1
YGR108W	CLB1	YDR378C	LSM6
YNL076W	MKS1	YLR180W	SAM1
YDR004W	RAD57	YLR381W	CTF3
YLR240W	VPS34	YOR106W	VAM3
YMR026C	PEX12	YDL184C	RPL41A

Predictions at threshold level 0.80 continued...			
Gene1 of predicted pair		Gene2 of predicted pair	
Systematic Name	Standard Name	Systematic Name	Standard Name
YNL032W	SIW14	YJL183W	MNN11
YOR080W	DIA2	YJL058C	BIT61
YMR311C	GLC8	YLR180W	SAM1
YGR072W	UPF3	YLR360W	VPS38
YDR253C	MET32	YML095C	RAD10
YMR142C	RPL13B	YOR066W	MSA1
YNL243W	SLA2	YOR109W	INP53
YIL053W	RHR2	YER144C	UBP5
YDR080W	VPS41	YPL161C	BEM4
YNL215W	IES2	YJL006C	CTK2
YHL011C	PRS3	YLR425W	TUS1
YIL159W	BNR1	YPR030W	CSR2
YLR386W	VAC14	YKR057W	RPS21A
YJL136C	RPS21B	YIL008W	URM1
YPR084W	YPR084W	YNL238W	KEX2
YGR249W	MGA1	YNR072W	HXT17
YOR112W	CEX1	YMR054W	STV1
YPL037C	EGD1	YCL001W	RER1
YOR132W	VPS17	YHL020C	OPI1
YLR216C	CPR6	YLR381W	CTF3
YLR234W	TOP3	YKR095W	MLP1
YDR293C	SSD1	YGR020C	VMA7
YGR019W	UGA1	YLR447C	VMA6
YLR371W	ROM2	YLR180W	SAM1
YNL291C	MID1	YOR132W	VPS17
YPL018W	CTF19	YHL002W	HSE1
YGR104C	SRB5	YBR215W	HPC2
YBL102W	SFT2	YHL020C	OPI1
YMR026C	PEX12	YLR371W	ROM2

Predictions at threshold level 0.80 continued...			
Gene1 of predicted pair		Gene2 of predicted pair	
Systematic Name	Standard Name	Systematic Name	Standard Name
YMR016C	SOK2	YDL077C	VAM6
YLR441C	RPS1A	YMR311C	GLC8
YML095C	RAD10	YGL175C	SAE2
YLR119W	SRN2	YNL325C	FIG4
YNL106C	INP52	YCR077C	PAT1
YBR131W	CCZ1	YKR001C	VPS1
YMR231W	PEP5	YML028W	TSA1
YCL010C	SGF29	YBR111W-A	SUS1
YKR072C	SIS2	YOL090W	MSH2
YHR052W	CIC1	YOL041C	NOP12
YCR081W	SRB8	YER068W	MOT2
YIL076W	SEC28	YHL020C	OPI1
YDR202C	RAV2	YMR216C	SKY1
YNL288W	CAF40	YBR200W	BEM1
YKL048C	ELM1	YOL081W	IRA2
YBR194W	SOY1	YDR137W	RGP1
YFR010W	UBP6	YMR038C	CCS1
YMR127C	SAS2	YOR038C	HIR2
YLL007C	YLL007C	YNL166C	BNI5
YLR381W	CTF3	YDR289C	RTT103
YDR174W	HMO1	YKL139W	CTK1
YLR079W	SIC1	YOR358W	HAP5
YOR038C	HIR2	YGL151W	NUT1
YDL185W	TFP1	YDR318W	MCM21
YPL253C	VIK1	YDR076W	RAD55
YEL065W	SIT1	YHR200W	RPN10
YJL060W	BNA3	YNL107W	YAF9
YGL223C	COG1	YML012W	ERV25
YFR010W	UBP6	YNL107W	YAF9

Predictions at threshold level 0.80 continued...			
Gene1 of predicted pair		Gene2 of predicted pair	
Systematic Name	Standard Name	Systematic Name	Standard Name
YBL047C	EDE1	YOR312C	RPL20B
YMR039C	SUB1	YGL151W	NUT1
YOR332W	VMA4	YKL041W	VPS24
YPL161C	BEM4	YNL127W	FAR11
YML115C	VAN1	YOR312C	RPL20B
YKR057W	RPS21A	YHR200W	RPN10
YGL033W	HOP2	YDR261C	EXG2
YDL223C	HBT1	YOL111C	MDY2
YKL007W	CAP1	YDR309C	GIC2
YBR200W	BEM1	YJL095W	BCK1
YML103C	NUP188	YNL054W	VAC7
YNL291C	MID1	YNL127W	FAR11
YDR192C	NUP42	YDR101C	ARX1
YPL024W	RMI1	YHR077C	NMD2
YFR031C-A	RPL2A	YOR109W	INP53
YLR192C	HCR1	YNL032W	SIW14
YNL069C	RPL16B	YMR216C	SKY1
YOL145C	CTR9	YBR010W	HHT1
YIL076W	SEC28	YDR358W	GGA1
YBR131W	CCZ1	YJL117W	PHO86
YDR080W	VPS41	YKR020W	VPS51
YAL024C	LTE1	YGL161C	YIP5
YDR378C	LSM6	YGR188C	BUB1
YLR192C	HCR1	YNL325C	FIG4
YNL325C	FIG4	YBL103C	RTG3
YMR224C	MRE11	YOR195W	SLK19
YBR108W	YBR108W	YPL137C	GIP3
YGR040W	KSS1	YHR084W	STE12
YGL115W	SNF4	YPR030W	CSR2

Predictions at threshold level 0.80 continued...			
Gene1 of predicted pair		Gene2 of predicted pair	
Systematic Name	Standard Name	Systematic Name	Standard Name
YEL051W	VMA8	YOL018C	TLG2
YNL041C	COG6	YER122C	GLO3
YER116C	SLX8	YJR084W	CSN12
YDR379W	RGA2	YKL032C	IXR1
YBL058W	SHP1	YLR306W	UBC12
YCR024C-A	PMP1	YKL117W	SBA1
YDR320C	SWA2	YMR216C	SKY1
YOR332W	VMA4	YHR039C-A	VMA10
YJL053W	PEP8	YDR108W	GSG1
YKL025C	PAN3	YHR114W	BZZ1
YOR033C	EXO1	YNL082W	PMS1
YBR200W	BEM1	YBR107C	IML3
YMR016C	SOK2	YKR035W-A	DID2
YGR108W	CLB1	YDR318W	MCM21
YER122C	GLO3	YAL002W	VPS8
YFR010W	UBP6	YJR060W	CBF1
YOR141C	ARP8	YDL226C	GCS1
YPR120C	CLB5	YKL101W	HSL1
YJR106W	ECM27	YFL044C	OTU1
YIL076W	SEC28	YJR140C	HIR3
YBR131W	CCZ1	YNL041C	COG6
YLR441C	RPS1A	YKR099W	BAS1
YHR134W	WSS1	YHR012W	VPS29
YOR156C	NFI1	YDR155C	CPR1
YHR160C	PEX18	YDL065C	PEX19
YPL106C	SSE1	YPR046W	MCM16
YMR198W	CIK1	YBR082C	UBC4
YNL127W	FAR11	YER122C	GLO3
YFR032C-A	RPL29	YDL006W	PTC1

Predictions at threshold level 0.80 continued...			
Gene1 of predicted pair		Gene2 of predicted pair	
Systematic Name	Standard Name	Systematic Name	Standard Name
YOR096W	RPS7A	YER102W	RPS8B
YOR083W	WHI5	YDR335W	MSN5
YNL032W	SIW14	YML001W	YPT7
YPL129W	TAF14	YMR109W	MYO5
YPL024W	RMI1	YDR318W	MCM21
YGL135W	RPL1B	YBR181C	RPS6B
YPL024W	RMI1	YHR200W	RPN10
YBR195C	MSI1	YCR077C	PAT1
YMR026C	PEX12	YPL137C	GIP3
YLR337C	VRP1	YBL051C	PIN4
YJL136C	RPS21B	YGL163C	RAD54
YBL102W	SFT2	YOR127W	RGA1
YML115C	VAN1	YER162C	RAD4
YML103C	NUP188	YFR030W	MET10
YNL166C	BNI5	YDR289C	RTT103
YLR058C	SHM2	YNL236W	SIN4
YJL128C	PBS2	YER118C	SHO1
YLR337C	VRP1	YBR057C	MUM2
YER122C	GLO3	YLR182W	SWI6
YGR072W	UPF3	YML071C	COG8
YDR443C	SSN2	YNL058C	YNL058C
YOR083W	WHI5	YGL003C	CDH1
YKR072C	SIS2	YJR140C	HIR3
YBR041W	FAT1	YDL006W	PTC1
YFR031C-A	RPL2A	YOL081W	IRA2
YDR289C	RTT103	YOR308C	SNU66
YJL172W	CPS1	YOL111C	MDY2
YHR152W	SPO12	YNL229C	URE2
YDL116W	NUP84	YER110C	KAP123

Predictions at threshold level 0.80 continued...			
Gene1 of predicted pair		Gene2 of predicted pair	
Systematic Name	Standard Name	Systematic Name	Standard Name
YAL024C	LTE1	YLR182W	SWI6
YPR120C	CLB5	YJR060W	CBF1
YMR231W	PEP5	YDR076W	RAD55
YPL050C	MNN9	YMR089C	YTA12
YNL253W	TEX1	YNL004W	HRB1
YFR032C-A	RPL29	YGR217W	CCH1
YMR055C	BUB2	YPL106C	SSE1
YGL003C	CDH1	YJR035W	RAD26
YML001W	YPT7	YAL002W	VPS8
YDR225W	HTA1	YLR032W	RAD5
YCR020C-A	MAK31	YJL004C	SYS1
YDR137W	RGP1	YHR081W	LRP1
YLR337C	VRP1	YPL213W	LEA1
YCR086W	CSM1	YGL253W	HXK2
YOL030W	GAS5	YJL187C	SWE1
YMR231W	PEP5	YGL198W	YIP4
YDR101C	ARX1	YOL041C	NOP12
YJL176C	SWI3	YBL101C	ECM21
YNL238W	KEX2	YCR053W	THR4
YER107C	GLE2	YLR055C	SPT8
YEL050C	RML2	YDL226C	GCS1
YBR200W	BEM1	YDR264C	AKR1
YJR135C	MCM22	YGL252C	RTG2
YML071C	COG8	YBR036C	CSG2
YLR090W	XDJ1	YOL111C	MDY2
YGR020C	VMA7	YGL054C	ERV14
YJL210W	PEX2	YDL065C	PEX19
YNL238W	KEX2	YLR398C	SKI2
YNL041C	COG6	YNR032W	PPG1

Predictions at threshold level 0.80 continued...			
Gene1 of predicted pair		Gene2 of predicted pair	
Systematic Name	Standard Name	Systematic Name	Standard Name
YLR135W	SLX4	YOL090W	MSH2
YOL054W	PSH1	YOL081W	IRA2
YCR053W	THR4	YDR207C	UME6
YMR255W	GFD1	YBR017C	KAP104
YKL048C	ELM1	YOL111C	MDY2
YPR017C	DSS4	YNR032W	PPG1
YOR275C	RIM20	YIL008W	URM1
YPL037C	EGD1	YDR153C	ENT5
YFR031C-A	RPL2A	YBR171W	SEC66
YLR131C	ACE2	YKL041W	VPS24
YCL037C	SRO9	YOL045W	PSK2
YLR441C	RPS1A	YHR039C-A	VMA10
YFL023W	BUD27	YGL175C	SAE2
YIL159W	BNR1	YMR152W	YIM1
YPL161C	BEM4	YLR370C	ARC18
YLR360W	VPS38	YNR032W	PPG1
YDR496C	PUF6	YKL185W	ASH1
YJL154C	VPS35	YJL036W	SNX4
YLR079W	SIC1	YDR289C	RTT103
YLR148W	PEP3	YLR043C	TRX1
YMR216C	SKY1	YJR040W	GEF1
YLR095C	IOC2	YHR039C-A	VMA10
YOR270C	VPH1	YNL058C	YNL058C
YER162C	RAD4	YOR035C	SHE4
YBL027W	RPL19B	YER096W	SHC1
YBR200W	BEM1	YDR207C	UME6
YBR194W	SOY1	YML028W	TSA1
YNR052C	POP2	YNL016W	PUB1
YDR225W	HTA1	YPL057C	SUR1

Predictions at threshold level 0.80 continued...			
**Gene1 of predicted pair**		**Gene2 of predicted pair**	
**Systematic Name**	**Standard Name**	**Systematic Name**	**Standard Name**
YKR020W	VPS51	YDR323C	PEP7
YOR038C	HIR2	YDR310C	SUM1
YNL288W	CAF40	YBR171W	SEC66
YOR043W	WHI2	YEL060C	PRB1
YBR200W	BEM1	YOL081W	IRA2
YPR030W	CSR2	YER040W	GLN3
YLR447C	VMA6	YGL124C	MON1
YHR156C	LIN1	YML071C	COG8
YLR441C	RPS1A	YNR072W	HXT17
YJL151C	SNA3	YGR268C	HUA1
YPL105C	YPL105C	YDL006W	PTC1
YLR370C	ARC18	YJR035W	RAD26
YPL115C	BEM3	YMR172W	HOT1
YHR012W	VPS29	YDR153C	ENT5
YDL002C	NHP10	YDR440W	DOT1
YDR137W	RGP1	YNL041C	COG6
YER161C	SPT2	YER139C	YER139C
YBR171W	SEC66	YHR012W	VPS29
YER116C	SLX8	YNL058C	YNL058C
YDL005C	MED2	YNL079C	TPM1
YDR335W	MSN5	YHR167W	THP2
YDR142C	PEX7	YNL214W	PEX17
YMR016C	SOK2	YOR327C	SNC2
YNL314W	DAL82	YIR023W	DAL81
YBL008W	HIR1	YGL148W	ARO2
YLR386W	VAC14	YKL032C	IXR1
YOR001W	RRP6	YDR378C	LSM6
YKR020W	VPS51	YDL192W	ARF1
YGL135W	RPL1B	YML097C	VPS9

Predictions at threshold level 0.80 continued...			
Gene1 of predicted pair		Gene2 of predicted pair	
Systematic Name	Standard Name	Systematic Name	Standard Name
YBR126C	TPS1	YHR156C	LIN1
YPR084W	YPR084W	YML001W	YPT7
YOR275C	RIM20	YBR283C	SSH1
YER110C	KAP123	YJR135C	MCM22
YKL160W	ELF1	YOR308C	SNU66
YHR178W	STB5	YGR188C	BUB1
YDR156W	RPA14	YLR079W	SIC1
YAL020C	ATS1	YBL051C	PIN4
YLR381W	CTF3	YFR030W	MET10
YEL065W	SIT1	YHR013C	ARD1
YIL053W	RHR2	YDR358W	GGA1
YMR026C	PEX12	YDL192W	ARF1
YAL024C	LTE1	YLR180W	SAM1
YMR224C	MRE11	YJL095W	BCK1
YNL243W	SLA2	YBR200W	BEM1
YLR337C	VRP1	YPL253C	VIK1
YMR080C	NAM7	YLR135W	SLX4
YDR261C	EXG2	YER139C	YER139C
YBR041W	FAT1	YGR135W	PRE9
YPL180W	TCO89	YLR398C	SKI2
YOR076C	SKI7	YOR191W	RIS1
YMR179W	SPT21	YGR188C	BUB1
YJR060W	CBF1	YOL108C	INO4
YDR137W	RGP1	YNL297C	MON2
YDR358W	GGA1	YAL055W	PEX22
YHR111W	UBA4	YGL124C	MON1
YDR108W	GSG1	YOR109W	INP53
YPL001W	HAT1	YMR055C	BUB2
YNL041C	COG6	YMR214W	SCJ1

Predictions at threshold level 0.80 continued...			
Gene1 of predicted pair		Gene2 of predicted pair	
Systematic Name	Standard Name	Systematic Name	Standard Name
YOR106W	VAM3	YPR051W	MAK3
YPL057C	SUR1	YML001W	YPT7
YAL024C	LTE1	YHR111W	UBA4
YPR084W	YPR084W	YJL154C	VPS35
YJR040W	GEF1	YEL053C	MAK10
YDR439W	LRS4	YPL152W	RRD2
YLR386W	VAC14	YGL151W	NUT1
YNL297C	MON2	YOR109W	INP53
YOR039W	CKB2	YNL206C	RTT106
YPL106C	SSE1	YDL047W	SIT4
YGR072W	UPF3	YKR001C	VPS1
YMR216C	SKY1	YPL037C	EGD1
YNL058C	YNL058C	YMR152W	YIM1
YKL160W	ELF1	YLR410W	VIP1
YML001W	YPT7	YJR033C	RAV1
YBR084C-A	RPL19A	YJL095W	BCK1
YNL243W	SLA2	YLR330W	CHS5
YJR059W	PTK2	YER110C	KAP123
YOR290C	SNF2	YDR469W	SDC1
YPR017C	DSS4	YEL043W	YEL043W
YLR090W	XDJ1	YKL126W	YPK1
YOR080W	DIA2	YDR318W	MCM21
YBL008W	HIR1	YPR120C	CLB5
YBR171W	SEC66	YCR053W	THR4
YER120W	SCS2	YPR032W	SRO7
YLR102C	APC9	YGL031C	RPL24A
YKL025C	PAN3	YML016C	PPZ1
YIL076W	SEC28	YPL161C	BEM4
YBR215W	HPC2	YMR055C	BUB2

Predictions at threshold level 0.80 continued...			
Gene1 of predicted pair		Gene2 of predicted pair	
Systematic Name	Standard Name	Systematic Name	Standard Name
YNL297C	MON2	YPL234C	TFP3
YDR477W	SNF1	YML071C	COG8
YLR315W	NKP2	YLR350W	ORM2
YDL013W	SLX5	YGL031C	RPL24A
YPL042C	SSN3	YMR037C	MSN2
YHR060W	VMA22	YBR280C	SAF1
YMR092C	AIP1	YDR389W	SAC7
YLR083C	EMP70	YPR051W	MAK3
YBR171W	SEC66	YHR114W	BZZ1
YMR202W	ERG2	YJL095W	BCK1
YMR199W	CLN1	YBL047C	EDE1
YOR191W	RIS1	YOR243C	PUS7
YGL175C	SAE2	YML028W	TSA1
YEL043W	YEL043W	YOR327C	SNC2
YDL059C	RAD59	YGL175C	SAE2
YOR127W	RGA1	YPR051W	MAK3
YAL024C	LTE1	YFR030W	MET10
YML095C	RAD10	YBR084C-A	RPL19A
YBR290W	BSD2	YMR273C	ZDS1
YDR076W	RAD55	YJR035W	RAD26
YFL023W	BUD27	YEL053C	MAK10
YNL069C	RPL16B	YDL077C	VAM6
YDR245W	MNN10	YJL006C	CTK2
YGL087C	MMS2	YLR381W	CTF3
YKR099W	BAS1	YLR093C	NYV1
YBR127C	VMA2	YNL238W	KEX2
YPL042C	SSN3	YGR252W	GCN5
YBL058W	SHP1	YHR081W	LRP1
YML095C	RAD10	YMR201C	RAD14

Predictions at threshold level 0.80 continued...			
Gene1 of predicted pair		Gene2 of predicted pair	
Systematic Name	Standard Name	Systematic Name	Standard Name
YPL195W	APL5	YMR230W	RPS10B
YMR224C	MRE11	YMR214W	SCJ1
YJR056C	YJR056C	YML001W	YPT7
YDR108W	GSG1	YJR060W	CBF1
YMR199W	CLN1	YFL023W	BUD27
YGL066W	SGF73	YPR046W	MCM16
YGR239C	PEX21	YNL214W	PEX17
YOR132W	VPS17	YER122C	GLO3
YBL088C	TEL1	YML095C	RAD10
YLR083C	EMP70	YJR060W	CBF1
YJL095W	BCK1	YDL077C	VAM6
YMR095C	SNO1	YMR230W	RPS10B
YBR010W	HHT1	YLR357W	RSC2
YGR104C	SRB5	YGR270W	YTA7
YJL154C	VPS35	YCL037C	SRO9
YMR224C	MRE11	YML062C	MFT1
YDL002C	NHP10	YOR213C	SAS5
YGL054C	ERV14	YHR012W	VPS29
YJR140C	HIR3	YPL057C	SUR1
YDR260C	SWM1	YCR086W	CSM1
YER096W	SHC1	YOR014W	RTS1
YDL161W	ENT1	YCR088W	ABP1
YPR030W	CSR2	YDR207C	UME6
YDR383C	NKP1	YMR224C	MRE11
YDR137W	RGP1	YJL183W	MNN11
YJL006C	CTK2	YNL079C	TPM1
YNL069C	RPL16B	YHR012W	VPS29
YJL124C	LSM1	YOR141C	ARP8
YDR507C	GIN4	YHR200W	RPN10

Predictions at threshold level 0.80 continued...			
Gene1 of predicted pair		Gene2 of predicted pair	
Systematic Name	Standard Name	Systematic Name	Standard Name
YDL116W	NUP84	YGL175C	SAE2
YOR358W	HAP5	YKL109W	HAP4
YKR001C	VPS1	YDL192W	ARF1
YLR095C	IOC2	YBR171W	SEC66
YBR019C	GAL10	YBR280C	SAF1
YDL020C	RPN4	YML095C	RAD10
YEL065W	SIT1	YLR095C	IOC2
YJR032W	CPR7	YMR152W	YIM1
YDL101C	DUN1	YGL175C	SAE2
YEL065W	SIT1	YML001W	YPT7
YHR157W	REC104	YJR021C	REC107
YIL002C	INP51	YPR173C	VPS4
YML115C	VAN1	YBL025W	RRN10
YPL024W	RMI1	YLR320W	MMS22
YMR153W	NUP53	YKL117W	SBA1
YML001W	YPT7	YMR231W	PEP5
YDR174W	HMO1	YBR200W	BEM1
YML095C	RAD10	YPL161C	BEM4
YEL044W	IES6	YPL050C	MNN9
YPR084W	YPR084W	YMR054W	STV1
YKL160W	ELF1	YNL107W	YAF9
YMR026C	PEX12	YBR173C	UMP1
YPL240C	HSP82	YPR118W	MRI1
YDR477W	SNF1	YNL199C	GCR2
YGL066W	SGF73	YLR032W	RAD5
YGR133W	PEX4	YML097C	VPS9
YLR083C	EMP70	YDR153C	ENT5
YJL006C	CTK2	YFR036W	CDC26
YJR032W	CPR7	YMR311C	GLC8

Predictions at threshold level 0.80 continued...			
**Gene1 of predicted pair**		**Gene2 of predicted pair**	
**Systematic Name**	**Standard Name**	**Systematic Name**	**Standard Name**
YER116C	SLX8	YGL003C	CDH1
YFR031C-A	RPL2A	YNL058C	YNL058C
YGL066W	SGF73	YOR014W	RTS1
YHR115C	DMA1	YGL031C	RPL24A
YNL058C	YNL058C	YJR084W	CSN12
YDL020C	RPN4	YGL253W	HXK2
YBR107C	IML3	YGL163C	RAD54
YNL291C	MID1	YDL077C	VAM6
YLR234W	TOP3	YDL059C	RAD59
YLL039C	UBI4	YER143W	DDI1
YJR140C	HIR3	YPL001W	HAT1
YMR224C	MRE11	YGL031C	RPL24A
YGL033W	HOP2	YFL023W	BUD27
YBL047C	EDE1	YLR425W	TUS1
YDR108W	GSG1	YOR308C	SNU66
YER179W	DMC1	YPL121C	MEI5
YER116C	SLX8	YBR073W	RDH54
YJL095W	BCK1	YKL161C	YKL161C
YOR038C	HIR2	YOR308C	SNU66
YPL037C	EGD1	YAR014C	BUD14
YJR135C	MCM22	YOL091W	SPO21
YER110C	KAP123	YJR032W	CPR7
YOR083W	WHI5	YJR035W	RAD26
YHR081W	LRP1	YGL124C	MON1
YDR245W	MNN10	YKR020W	VPS51
YBR107C	IML3	YDR260C	SWM1
YOR033C	EXO1	YML062C	MFT1
YCR066W	RAD18	YKL114C	APN1
YNL238W	KEX2	YER122C	GLO3

Predictions at threshold level 0.80 continued...			
Gene1 of predicted pair		Gene2 of predicted pair	
Systematic Name	Standard Name	Systematic Name	Standard Name
YGL147C	RPL9A	YPL024W	RMI1
YLR360W	VPS38	YDL161W	ENT1
YNL031C	HHT2	YJR063W	RPA12
YJL128C	PBS2	YCR073C	SSK22
YDR176W	NGG1	YKL139W	CTK1
YMR054W	STV1	YJR032W	CPR7
YAL024C	LTE1	YGL212W	VAM7
YLR083C	EMP70	YPL195W	APL5
YGL060W	YBP2	YMR202W	ERG2
YKL139W	CTK1	YLR306W	UBC12
YPL105C	YPL105C	YML038C	YMD8
YPR051W	MAK3	YPR032W	SRO7
YHR111W	UBA4	YBR114W	RAD16
YKR020W	VPS51	YGL054C	ERV14
YJL036W	SNX4	YPL195W	APL5
YPR040W	TIP41	YDL047W	SIT4
YKR072C	SIS2	YCR077C	PAT1
YNL051W	COG5	YPR032W	SRO7
YPL096C-A	ERI1	YGR135W	PRE9
YGL212W	VAM7	YOL121C	RPS19A
YLR192C	HCR1	YJL095W	BCK1
YPR017C	DSS4	YNL291C	MID1
YPR017C	DSS4	YGR105W	VMA21
YLR018C	POM34	YNL199C	GCR2
YDR146C	SWI5	YOR275C	RIM20
YAR014C	BUD14	YLR182W	SWI6
YMR142C	RPL13B	YNR072W	HXT17
YKL139W	CTK1	YGL124C	MON1
YOR312C	RPL20B	YLR182W	SWI6

Predictions at threshold level 0.80 continued...			
**Gene1 of predicted pair**		**Gene2 of predicted pair**	
**Systematic Name**	**Standard Name**	**Systematic Name**	**Standard Name**
YIL159W	BNR1	YNR072W	HXT17
YHR178W	STB5	YDL155W	CLB3
YHR200W	RPN10	YML071C	COG8
YDL160C	DHH1	YML058W	SML1
YLR410W	VIP1	YJR035W	RAD26
YGL212W	VAM7	YJR040W	GEF1
YPR051W	MAK3	YBR114W	RAD16
YGL237C	HAP2	YKL048C	ELM1
YPL024W	RMI1	YKL048C	ELM1
YMR224C	MRE11	YDR310C	SUM1
YKR020W	VPS51	YPL089C	RLM1
YKL126W	YPK1	YLR371W	ROM2
YMR133W	REC114	YPR030W	CSR2
YHR052W	CIC1	YDR312W	SSF2
YEL053C	MAK10	YER040W	GLN3
YPL024W	RMI1	YLR094C	GIS3
YJR056C	YJR056C	YLR320W	MMS22
YDR137W	RGP1	YDL083C	RPS16B
YNL218W	MGS1	YBL079W	NUP170
YKL048C	ELM1	YFR030W	MET10
YAL024C	LTE1	YKR035W-A	DID2
YIL053W	RHR2	YGL147C	RPL9A
YDR202C	RAV2	YEL053C	MAK10
YBR290W	BSD2	YIL156W	UBP7
YDR254W	CHL4	YJR063W	RPA12
YLR079W	SIC1	YLR254C	NDL1
YMR216C	SKY1	YDR153C	ENT5
YNL229C	URE2	YER040W	GLN3
YLR095C	IOC2	YER044C	ERG28

Predictions at threshold level 0.80 continued...			
Gene1 of predicted pair		Gene2 of predicted pair	
Systematic Name	Standard Name	Systematic Name	Standard Name
YDL216C	RRI1	YOL117W	RRI2
YDR392W	SPT3	YBR111W-A	SUS1
YFR010W	UBP6	YDR318W	MCM21
YGR184C	UBR1	YNL307C	MCK1
YMR127C	SAS2	YBR107C	IML3
YKL139W	CTK1	YOL111C	MDY2
YNR049C	MSO1	YPR032W	SRO7
YDL020C	RPN4	YPL018W	CTF19
YKR035W-A	DID2	YJL095W	BCK1
YJL154C	VPS35	YDL161W	ENT1
YLR337C	VRP1	YHR200W	RPN10
YIL103W	DPH1	YCR086W	CSM1
YOR358W	HAP5	YGR188C	BUB1
YDR378C	LSM6	YPR046W	MCM16
YFR010W	UBP6	YPL037C	EGD1
YOR026W	BUB3	YHR205W	SCH9
YKL048C	ELM1	YDL194W	SNF3
YNL025C	SSN8	YJL124C	LSM1
YGR270W	YTA7	YHR167W	THP2
YAL024C	LTE1	YER144C	UBP5
YAL015C	NTG1	YKL114C	APN1
YDR245W	MNN10	YBR215W	HPC2
YOR337W	TEA1	YMR139W	RIM11
YLR288C	MEC3	YOR368W	RAD17
YNL087W	TCB2	YOR043W	WHI2
YBR194W	SOY1	YJR060W	CBF1
YML001W	YPT7	YLR371W	ROM2
YNL218W	MGS1	YOR243C	PUS7
YDR329C	PEX3	YJL210W	PEX2

Predictions at threshold level 0.80 continued...			
Gene1 of predicted pair		Gene2 of predicted pair	
Systematic Name	Standard Name	Systematic Name	Standard Name
YOL062C	APM4	YPL259C	APM1
YJR059W	PTK2	YGR059W	SPR3
YLR181C	VTA1	YPR173C	VPS4
YJL060W	BNA3	YML095C	RAD10
YMR199W	CLN1	YGR135W	PRE9
YLR337C	VRP1	YLR447C	VMA6
YDR507C	GIN4	YGL208W	SIP2
YAL024C	LTE1	YER139C	YER139C
YOR083W	WHI5	YMR198W	CIK1
YDR245W	MNN10	YEL013W	VAC8
YGR072W	UPF3	YMR054W	STV1
YGR104C	SRB5	YBL075C	SSA3
YKR020W	VPS51	YOL018C	TLG2
YAL024C	LTE1	YHR039C-A	VMA10
YPR036W	VMA13	YNR072W	HXT17
YNL032W	SIW14	YGL115W	SNF4
YNR023W	SNF12	YER161C	SPT2
YAL024C	LTE1	YDL223C	HBT1
YOR112W	CEX1	YMR216C	SKY1
YHL031C	GOS1	YOR036W	PEP12
YLR234W	TOP3	YJR135C	MCM22
YDR363W-A	SEM1	YDR469W	SDC1
YBL058W	SHP1	YJL095W	BCK1
YHR052W	CIC1	YKR057W	RPS21A
YOR106W	VAM3	YER042W	MXR1
YMR224C	MRE11	YGR081C	SLX9
YER116C	SLX8	YLR398C	SKI2
YKL006W	RPL14A	YJL006C	CTK2
YDL077C	VAM6	YNR032W	PPG1

Predictions at threshold level 0.80 continued...			
Gene1 of predicted pair		Gene2 of predicted pair	
Systematic Name	Standard Name	Systematic Name	Standard Name
YBR200W	BEM1	YKL129C	MYO3
YMR127C	SAS2	YHR111W	UBA4
YNL288W	CAF40	YOL067C	RTG1
YDR477W	SNF1	YBR217W	ATG12
YPL240C	HSP82	YER105C	NUP157
YAL051W	OAF1	YMR198W	CIK1
YHR178W	STB5	YOL067C	RTG1
YEL050C	RML2	YOR001W	RRP6
YHR079C	IRE1	YGL223C	COG1
YMR172W	HOT1	YJL154C	VPS35
YNL054W	VAC7	YNL116W	DMA2
YJL053W	PEP8	YDR310C	SUM1
YHR081W	LRP1	YBR019C	GAL10
YPR131C	NAT3	YJR005W	APL1
YMR224C	MRE11	YHR156C	LIN1
YDR440W	DOT1	YEL013W	VAC8
YOR290C	SNF2	YOR064C	YNG1
YML058W	SML1	YGL115W	SNF4
YFR031C-A	RPL2A	YNR032W	PPG1
YBR126C	TPS1	YFL033C	RIM15
YJL036W	SNX4	YMR054W	STV1
YDL020C	RPN4	YHR012W	VPS29
YNL243W	SLA2	YNL153C	GIM3
YBR215W	HPC2	YPL256C	CLN2
YGL178W	MPT5	YLR418C	CDC73
YNR052C	POP2	YJL164C	TPK1
YPR052C	NHP6A	YDL160C	DHH1
YDR477W	SNF1	YCR053W	THR4
YOL090W	MSH2	YNL076W	MKS1

Predictions at threshold level 0.80 continued...			
Gene1 of predicted pair		Gene2 of predicted pair	
Systematic Name	Standard Name	Systematic Name	Standard Name
YHL031C	GOS1	YCL037C	SRO9
YMR039C	SUB1	YML016C	PPZ1
YLR094C	GIS3	YBL025W	RRN10
YKL032C	IXR1	YDR264C	AKR1
YMR016C	SOK2	YNL076W	MKS1
YMR198W	CIK1	YGR133W	PEX4
YKR001C	VPS1	YER120W	SCS2
YHR081W	LRP1	YPL195W	APL5
YBR107C	IML3	YJL095W	BCK1
YDR202C	RAV2	YNL127W	FAR11
YLR386W	VAC14	YPR030W	CSR2
YJL095W	BCK1	YEL036C	ANP1
YAL024C	LTE1	YKL139W	CTK1
YNL107W	YAF9	YNL225C	CNM67
YJL006C	CTK2	YNL107W	YAF9
YOR195W	SLK19	YDR207C	UME6
YPR084W	YPR084W	YPL195W	APL5
YNL264C	PDR17	YGR014W	MSB2
YOR127W	RGA1	YML071C	COG8
YIR003W	YIR003W	YDR245W	MNN10
YPR036W	VMA13	YCR053W	THR4
YFR010W	UBP6	YMR016C	SOK2
YMR172W	HOT1	YGL253W	HXK2
YLR079W	SIC1	YMR164C	MSS11
YOR033C	EXO1	YLR234W	TOP3
YPL115C	BEM3	YOR127W	RGA1
YDR261C	EXG2	YMR214W	SCJ1
YNR072W	HXT17	YHR081W	LRP1
YMR116C	ASC1	YLR410W	VIP1

Predictions at threshold level 0.80 continued...			
**Gene1 of predicted pair**		**Gene2 of predicted pair**	
**Systematic Name**	**Standard Name**	**Systematic Name**	**Standard Name**
YLR093C	NYV1	YDR207C	UME6
YFR036W	CDC26	YNL225C	CNM67
YNL297C	MON2	YEL053C	MAK10
YBL102W	SFT2	YER162C	RAD4
YFL023W	BUD27	YML102W	CAC2
YCR088W	ABP1	YPL045W	VPS16
YMR054W	STV1	YOL108C	INO4
YGL246C	RAI1	YIL133C	RPL16A
YBL071W-A	KTI11	YOL121C	RPS19A
YPR171W	BSP1	YMR109W	MYO5
YDR245W	MNN10	YDL002C	NHP10
YLR425W	TUS1	YDR207C	UME6
YLR452C	SST2	YJR032W	CPR7
YBR171W	SEC66	YBR019C	GAL10
YOR290C	SNF2	YLR180W	SAM1
YBR171W	SEC66	YDR358W	GGA1
YDR137W	RGP1	YAL002W	VPS8
YIL053W	RHR2	YGL078C	DBP3
YHR060W	VMA22	YER139C	YER139C
YML115C	VAN1	YLR094C	GIS3
YDR448W	ADA2	YBL002W	HTB2
YML115C	VAN1	YER144C	UBP5
YJR063W	RPA12	YDR318W	MCM21
YOR083W	WHI5	YBR107C	IML3
YDR254W	CHL4	YLR429W	CRN1
YDR129C	SAC6	YJR032W	CPR7
YKR020W	VPS51	YER120W	SCS2
YNL218W	MGS1	YOR191W	RIS1
YDR207C	UME6	YKR030W	GMH1

Predictions at threshold level 0.80 continued...			
Gene1 of predicted pair		Gene2 of predicted pair	
Systematic Name	Standard Name	Systematic Name	Standard Name
YPL106C	SSE1	YDR318W	MCM21
YLR370C	ARC18	YLR425W	TUS1
YIL156W	UBP7	YBL079W	NUP170
YFL023W	BUD27	YLR398C	SKI2
YBR217W	ATG12	YDR137W	RGP1
YPR084W	YPR084W	YDL002C	NHP10
YOR290C	SNF2	YLR370C	ARC18
YGR249W	MGA1	YOR066W	MSA1
YNL201C	PSY2	YLR410W	VIP1
YGR249W	MGA1	YBR108W	YBR108W
YMR246W	FAA4	YMR313C	TGL3
YMR273C	ZDS1	YPL161C	BEM4
YDL083C	RPS16B	YER122C	GLO3
YPL115C	BEM3	YEL056W	HAT2
YOR156C	NFI1	YGL237C	HAP2
YGL025C	PGD1	YML071C	COG8
YIL008W	URM1	YJL190C	RPS22A
YOR191W	RIS1	YHR077C	NMD2
YML103C	NUP188	YNL199C	GCR2
YGR020C	VMA7	YAR014C	BUD14
YDR484W	VPS52	YOL018C	TLG2
YIL034C	CAP2	YDR155C	CPR1
YJL124C	LSM1	YML028W	TSA1
YLR234W	TOP3	YOR039W	CKB2
YPL195W	APL5	YOR327C	SNC2
YOL121C	RPS19A	YMR214W	SCJ1
YJL183W	MNN11	YLR398C	SKI2
YOL117W	RRI2	YJR084W	CSN12
YMR198W	CIK1	YBR194W	SOY1

Predictions at threshold level 0.80 continued...			
Gene1 of predicted pair		Gene2 of predicted pair	
Systematic Name	Standard Name	Systematic Name	Standard Name
YML115C	VAN1	YKR099W	BAS1
YNL243W	SLA2	YML094W	GIM5
YGR261C	APL6	YNL238W	KEX2
YJR135C	MCM22	YDL226C	GCS1
YFR010W	UBP6	YHR013C	ARD1
YNL243W	SLA2	YJL148W	RPA34
YPL024W	RMI1	YML071C	COG8
YML058W	SML1	YPR120C	CLB5
YDR443C	SSN2	YOR008C	SLG1
YLR234W	TOP3	YML013W	UBX2
YHR161C	YAP1801	YOR035C	SHE4
YDR443C	SSN2	YMR142C	RPL13B
YMR004W	MVP1	YGR020C	VMA7
YJR106W	ECM27	YMR319C	FET4
YKL116C	PRR1	YMR311C	GLC8
YKL080W	VMA5	YJL095W	BCK1
YLR083C	EMP70	YNL069C	RPL16B
YKR095W	MLP1	YKL068W	NUP100
YHR200W	RPN10	YKR048C	NAP1
YDL184C	RPL41A	YMR153W	NUP53
YHL020C	OPI1	YER120W	SCS2
YNL291C	MID1	YJR060W	CBF1
YOR083W	WHI5	YDR254W	CHL4
YGR184C	UBR1	YLR032W	RAD5
YHR039C-A	VMA10	YER040W	GLN3
YBL008W	HIR1	YLR320W	MMS22
YFL013C	IES1	YLR315W	NKP2
YNL079C	TPM1	YBL067C	UBP13
YJR059W	PTK2	YIL148W	RPL40A

Predictions at threshold level 0.80 continued...			
Gene1 of predicted pair		Gene2 of predicted pair	
Systematic Name	Standard Name	Systematic Name	Standard Name
YGL250W	RMR1	YLR350W	ORM2
YOR023C	AHC1	YEL056W	HAT2
YGR072W	UPF3	YPR032W	SRO7
YJL060W	BNA3	YLR058C	SHM2
YNR072W	HXT17	YLR371W	ROM2
YOR132W	VPS17	YHR012W	VPS29
YLR192C	HCR1	YMR214W	SCJ1
YKL126W	YPK1	YPR046W	MCM16
YKR035W-A	DID2	YBR019C	GAL10
YLR135W	SLX4	YGR071C	YGR071C
YNL325C	FIG4	YLR370C	ARC18
YBL058W	SHP1	YBR030W	YBR030W
YMR224C	MRE11	YER116C	SLX8
YMR153W	NUP53	YDL077C	VAM6
YIL076W	SEC28	YDL184C	RPL41A
YMR038C	CCS1	YOR014W	RTS1
YLR234W	TOP3	YMR199W	CLN1
YDR439W	LRS4	YJR063W	RPA12
YDR110W	FOB1	YBR119W	MUD1
YGR054W	YGR054W	YGR188C	BUB1
YML001W	YPT7	YDR261C	EXG2
YMR224C	MRE11	YMR284W	YKU70
YMR152W	YIM1	YOR327C	SNC2
YDL192W	ARF1	YDL077C	VAM6
YLR234W	TOP3	YBL021C	HAP3
YDR137W	RGP1	YOL081W	IRA2
YEL027W	CUP5	YJL095W	BCK1
YNL325C	FIG4	YKR035W-A	DID2
YDR004W	RAD57	YLR234W	TOP3

Predictions at threshold level 0.80 continued...			
Gene1 of predicted pair		Gene2 of predicted pair	
Systematic Name	Standard Name	Systematic Name	Standard Name
YDR310C	SUM1	YKR030W	GMH1
YER116C	SLX8	YPL137C	GIP3
YJL127C	SPT10	YDR378C	LSM6
YBR107C	IML3	YDR183W	PLP1
YOR080W	DIA2	YBR200W	BEM1
YLR386W	VAC14	YNL307C	MCK1
YPL178W	CBC2	YKR024C	DBP7
YPL042C	SSN3	YLR315W	NKP2
YKL139W	CTK1	YHR108W	GGA2
YDR137W	RGP1	YHR060W	VMA22
YJL060W	BNA3	YDR254W	CHL4
YDR293C	SSD1	YGL237C	HAP2
YAL051W	OAF1	YDR076W	RAD55
YBR200W	BEM1	YDR254W	CHL4
YAL024C	LTE1	YKL126W	YPK1
YPL057C	SUR1	YAL002W	VPS8
YJL172W	CPS1	YDR261C	EXG2
YNL307C	MCK1	YBR280C	SAF1
YNL248C	RPA49	YOL054W	PSH1
YNL288W	CAF40	YDR183W	PLP1
YHL031C	GOS1	YOR038C	HIR2
YDR363W-A	SEM1	YDR318W	MCM21
YJL036W	SNX4	YDR153C	ENT5
YFR010W	UBP6	YDL047W	SIT4
YLL007C	YLL007C	YDR293C	SSD1
YOR368W	RAD17	YJR035W	RAD26
YFR010W	UBP6	YBL103C	RTG3
YPL213W	LEA1	YJL006C	CTK2
YOL051W	GAL11	YCR084C	TUP1

Predictions at threshold level 0.80 continued...			
Gene1 of predicted pair		Gene2 of predicted pair	
Systematic Name	Standard Name	Systematic Name	Standard Name
YPL057C	SUR1	YNL238W	KEX2
YGL124C	MON1	YDL226C	GCS1
YPL037C	EGD1	YJR084W	CSN12
YBR200W	BEM1	YGL253W	HXK2
YKL048C	ELM1	YDR310C	SUM1
YJL183W	MNN11	YBL072C	RPS8A
YDR254W	CHL4	YOR141C	ARP8
YNL107W	YAF9	YHR115C	DMA1
YGR142W	BTN2	YER074W-A	YOS1
YKR001C	VPS1	YBR283C	SSH1
YPL234C	TFP3	YNL107W	YAF9
YBL102W	SFT2	YCR066W	RAD18
YDL223C	HBT1	YKL048C	ELM1
YOR127W	RGA1	YDR289C	RTT103
YOR195W	SLK19	YKR061W	KTR2
YIL053W	RHR2	YGL115W	SNF4
YBR215W	HPC2	YLR320W	MMS22
YLR083C	EMP70	YOR106W	VAM3
YPL129W	TAF14	YEL044W	IES6
YBR173C	UMP1	YPL195W	APL5
YJL154C	VPS35	YEL036C	ANP1
YDR293C	SSD1	YER162C	RAD4
YGR055W	MUP1	YPL240C	HSP82
YDR137W	RGP1	YPL161C	BEM4
YGR249W	MGA1	YMR230W	RPS10B
YIL034C	CAP2	YLR292C	SEC72
YPR040W	TIP41	YPL161C	BEM4
YBR111W-A	SUS1	YPL248C	GAL4
YBL021C	HAP3	YOR358W	HAP5

Predictions at threshold level 0.80 continued...			
Gene1 of predicted pair		Gene2 of predicted pair	
Systematic Name	Standard Name	Systematic Name	Standard Name
YAL024C	LTE1	YDL002C	NHP10
YCL037C	SRO9	YPL195W	APL5
YOR023C	AHC1	YPR051W	MAK3
YIL159W	BNR1	YLR386W	VAC14
YNL297C	MON2	YNL238W	KEX2
YOR275C	RIM20	YJL095W	BCK1
YER044C	ERG28	YDL192W	ARF1
YBR030W	YBR030W	YMR133W	REC114
YJR084W	CSN12	YML097C	VPS9
YDR335W	MSN5	YER105C	NUP157
YJL157C	FAR1	YJL187C	SWE1
YDL184C	RPL41A	YBR036C	CSG2
YDR293C	SSD1	YDL077C	VAM6
YDR144C	MKC7	YJL095W	BCK1
YPL178W	CBC2	YOR358W	HAP5
YLR192C	HCR1	YDR261C	EXG2
YMR224C	MRE11	YDL077C	VAM6
YJR140C	HIR3	YNL107W	YAF9
YMR153W	NUP53	YER139C	YER139C
YGL200C	EMP24	YJL095W	BCK1
YEL009C	GCN4	YBL008W	HIR1
YDL020C	RPN4	YDR293C	SSD1
YGL163C	RAD54	YDR289C	RTT103
YFR010W	UBP6	YNR072W	HXT17
YLR441C	RPS1A	YDR261C	EXG2
YLR386W	VAC14	YML115C	VAN1
YPL121C	MEI5	YHR079C-A	SAE3
YMR199W	CLN1	YOR008C	SLG1
YGL163C	RAD54	YBR036C	CSG2

Predictions at threshold level 0.80 continued...			
Gene1 of predicted pair		Gene2 of predicted pair	
Systematic Name	Standard Name	Systematic Name	Standard Name
YOR327C	SNC2	YOL081W	IRA2
YKL010C	UFD4	YBL047C	EDE1
YGL115W	SNF4	YDL006W	PTC1
YMR039C	SUB1	YPR120C	CLB5
YPR084W	YPR084W	YDR245W	MNN10
YER116C	SLX8	YHR081W	LRP1
YGL215W	CLG1	YKL041W	VPS24
YGL147C	RPL9A	YPL037C	EGD1
YNL069C	RPL16B	YOR327C	SNC2
YOR112W	CEX1	YGL223C	COG1
YKR057W	RPS21A	YJL095W	BCK1
YGL148W	ARO2	YDR310C	SUM1
YML095C	RAD10	YHR200W	RPN10
YLR292C	SEC72	YOR127W	RGA1
YPL253C	VIK1	YMR109W	MYO5
YKL126W	YPK1	YPL057C	SUR1
YGL078C	DBP3	YNL032W	SIW14
YBR108W	YBR108W	YJR135C	MCM22
YPL253C	VIK1	YDR378C	LSM6
YLR371W	ROM2	YMR214W	SCJ1
YKR061W	KTR2	YPL152W	RRD2
YDL184C	RPL41A	YBL103C	RTG3
YDR378C	LSM6	YGR135W	PRE9
YKR001C	VPS1	YOR014W	RTS1
YJL058C	BIT61	YBR215W	HPC2
YGL025C	PGD1	YPR046W	MCM16
YMR179W	SPT21	YLR429W	CRN1
YBR107C	IML3	YJR032W	CPR7
YJR125C	ENT3	YNR006W	VPS27

Predictions at threshold level 0.80 continued...			
Gene1 of predicted pair		Gene2 of predicted pair	
Systematic Name	Standard Name	Systematic Name	Standard Name
YLR360W	VPS38	YDL077C	VAM6
YDR156W	RPA14	YNL106C	INP52
YNL243W	SLA2	YIL034C	CAP2
YOR043W	WHI2	YEL053C	MAK10
YMR172W	HOT1	YHR178W	STB5
YDR477W	SNF1	YPL195W	APL5
YLR288C	MEC3	YLR435W	TSR2
YLR381W	CTF3	YGL003C	CDH1
YNL106C	INP52	YJR063W	RPA12
YHR178W	STB5	YIL008W	URM1
YGR108W	CLB1	YDR310C	SUM1
YLR254C	NDL1	YML112W	CTK3
YOR038C	HIR2	YOR368W	RAD17
YOR337W	TEA1	YMR230W	RPS10B
YLR292C	SEC72	YGR188C	BUB1
YFR043C	IRC6	YLR248W	RCK2
YGL212W	VAM7	YLR398C	SKI2
YFL003C	MSH4	YDL154W	MSH5
YPR046W	MCM16	YLR032W	RAD5
YBL019W	APN2	YMR038C	CCS1
YLL026W	HSP104	YNL041C	COG6
YBR131W	CCZ1	YMR004W	MVP1
YGL151W	NUT1	YGL124C	MON1
YIL159W	BNR1	YBR030W	YBR030W
YNL032W	SIW14	YDR310C	SUM1
YMR142C	RPL13B	YER162C	RAD4
YDL101C	DUN1	YNL016W	PUB1
YKL139W	CTK1	YDR318W	MCM21
YNL054W	VAC7	YLR182W	SWI6

Predictions at threshold level 0.80 continued...			
Gene1 of predicted pair		Gene2 of predicted pair	
Systematic Name	Standard Name	Systematic Name	Standard Name
YNL087W	TCB2	YGL115W	SNF4
YML103C	NUP188	YJR059W	PTK2
YPL001W	HAT1	YDR076W	RAD55
YGL147C	RPL9A	YER139C	YER139C
YPL120W	VPS30	YER122C	GLO3
YDR202C	RAV2	YNL107W	YAF9
YBL102W	SFT2	YOR106W	VAM3
YDR507C	GIN4	YHR152W	SPO12
YHR012W	VPS29	YBR283C	SSH1
YPL115C	BEM3	YGL087C	MMS2
YBR194W	SOY1	YHL031C	GOS1
YDR146C	SWI5	YOL051W	GAL11
YJL124C	LSM1	YOR368W	RAD17
YPL137C	GIP3	YDL077C	VAM6
YBL047C	EDE1	YKR001C	VPS1
YNL041C	COG6	YOL018C	TLG2
YNL206C	RTT106	YKL101W	HSL1
YBR173C	UMP1	YGL003C	CDH1
YJR058C	APS2	YOR035C	SHE4
YHR081W	LRP1	YJL095W	BCK1
YGL087C	MMS2	YGR270W	YTA7
YIL034C	CAP2	YBR114W	RAD16
YLR234W	TOP3	YJL190C	RPS22A
YBR057C	MUM2	YOR035C	SHE4
YJL176C	SWI3	YOL081W	IRA2
YMR142C	RPL13B	YBR280C	SAF1
YCR084C	TUP1	YKL181W	PRS1
YMR016C	SOK2	YOR066W	MSA1
YLR429W	CRN1	YPL018W	CTF19

Predictions at threshold level 0.80 continued...			
Gene1 of predicted pair		Gene2 of predicted pair	
Systematic Name	Standard Name	Systematic Name	Standard Name
YNL201C	PSY2	YNL215W	IES2
YBR228W	SLX1	YPL079W	RPL21B
YDR477W	SNF1	YML062C	MFT1
YDL020C	RPN4	YNL291C	MID1
YDR192C	NUP42	YBL079W	NUP170
YCR020C-A	MAK31	YMR004W	MVP1
YDR320C	SWA2	YOL018C	TLG2
YMR199W	CLN1	YOR358W	HAP5
YHL031C	GOS1	YJR060W	CBF1
YDR507C	GIN4	YBR267W	REI1
YLR292C	SEC72	YLR360W	VPS38
YKR072C	SIS2	YBR082C	UBC4
YGL147C	RPL9A	YHR060W	VMA22
YPR018W	RLF2	YDR227W	SIR4
YBL067C	UBP13	YLR425W	TUS1
YDR146C	SWI5	YOR014W	RTS1
YBL051C	PIN4	YMR127C	SAS2
YPL161C	BEM4	YJR090C	GRR1
YDL223C	HBT1	YBL047C	EDE1
YER087C-B	SBH1	YLR055C	SPT8
YMR202W	ERG2	YJL006C	CTK2
YBR228W	SLX1	YBR082C	UBC4
YOR080W	DIA2	YNL107W	YAF9
YEL042W	GDA1	YDR310C	SUM1
YBL079W	NUP170	YKL068W	NUP100
YDR335W	MSN5	YFR030W	MET10
YMR026C	PEX12	YDL083C	RPS16B
YNL307C	MCK1	YNL041C	COG6
YLR119W	SRN2	YLR320W	MMS22

Predictions at threshold level 0.80 continued...			
Gene1 of predicted pair		Gene2 of predicted pair	
Systematic Name	Standard Name	Systematic Name	Standard Name
YDR507C	GIN4	YER027C	GAL83
YBR274W	CHK1	YBR195C	MSI1
YMR004W	MVP1	YNL051W	COG5
YOR195W	SLK19	YDL226C	GCS1
YBR108W	YBR108W	YLR447C	VMA6
YPL253C	VIK1	YNL225C	CNM67
YNL325C	FIG4	YOR290C	SNF2
YLR360W	VPS38	YNL051W	COG5
YDR080W	VPS41	YDR323C	PEP7
YBR173C	UMP1	YBL103C	RTG3
YBL102W	SFT2	YNL238W	KEX2
YDL184C	RPL41A	YDL077C	VAM6
YDR378C	LSM6	YNL107W	YAF9
YJR035W	RAD26	YOR014W	RTS1
YKL160W	ELF1	YNL236W	SIN4
YLR170C	APS1	YPR029C	APL4
YAL024C	LTE1	YGR270W	YTA7
YDR440W	DOT1	YNL107W	YAF9
YHR039C-A	VMA10	YNL238W	KEX2
YBR036C	CSG2	YMR214W	SCJ1
YDL005C	MED2	YKR048C	NAP1
YER158C	YER158C	YAR014C	BUD14
YBR030W	YBR030W	YER162C	RAD4
YEL050C	RML2	YDR200C	VPS64
YML115C	VAN1	YDL192W	ARF1
YCR034W	FEN1	YMR167W	MLH1
YPL046C	ELC1	YJR052W	RAD7
YMR311C	GLC8	YOR014W	RTS1
YBL021C	HAP3	YKL048C	ELM1

Predictions at threshold level 0.80 continued...			
Gene1 of predicted pair		Gene2 of predicted pair	
Systematic Name	Standard Name	Systematic Name	Standard Name
YDR137W	RGP1	YKL160W	ELF1
YKR020W	VPS51	YBL072C	RPS8A
YGL087C	MMS2	YJR135C	MCM22
YPR036W	VMA13	YHR081W	LRP1
YGL003C	CDH1	YDL226C	GCS1
YPL042C	SSN3	YEL050C	RML2
YPL057C	SUR1	YHR200W	RPN10
YJR056C	YJR056C	YNL238W	KEX2
YDR156W	RPA14	YNL307C	MCK1
YPR051W	MAK3	YEL056W	HAT2
YMR127C	SAS2	YJR135C	MCM22
YPR017C	DSS4	YCL037C	SRO9
YKL116C	PRR1	YER139C	YER139C
YNL297C	MON2	YMR054W	STV1
YKR099W	BAS1	YLR452C	SST2
YPL022W	RAD1	YJL124C	LSM1
YGL147C	RPL9A	YJL172W	CPS1
YNL329C	PEX6	YBL101C	ECM21
YDR469W	SDC1	YJL140W	RPB4
YLR090W	XDJ1	YER144C	UBP5
YGR270W	YTA7	YIR005W	IST3
YDR153C	ENT5	YDL137W	ARF2
YML028W	TSA1	YBR082C	UBC4
YER144C	UBP5	YMR133W	REC114
YER116C	SLX8	YPL057C	SUR1
YJL183W	MNN11	YMR214W	SCJ1
YEL027W	CUP5	YJL183W	MNN11
YDL002C	NHP10	YGL151W	NUT1
YCR020C-A	MAK31	YKR020W	VPS51

Predictions at threshold level 0.80 continued...			
Gene1 of predicted pair		Gene2 of predicted pair	
Systematic Name	Standard Name	Systematic Name	Standard Name
YPL105C	YPL105C	YJL004C	SYS1
YBL088C	TEL1	YJL106W	IME2
YGL174W	BUD13	YDR469W	SDC1
YHR061C	GIC1	YCR086W	CSM1
YGR270W	YTA7	YFR036W	CDC26
YLR079W	SIC1	YJL124C	LSM1
YKL025C	PAN3	YBR200W	BEM1
YCR020C-A	MAK31	YBL102W	SFT2
YLR079W	SIC1	YKL139W	CTK1
YLR386W	VAC14	YDR155C	CPR1
YML102W	CAC2	YGL163C	RAD54
YCR020C-A	MAK31	YEL051W	VMA8
YDL184C	RPL41A	YDR358W	GGA1
YOR337W	TEA1	YEL060C	PRB1
YIL076W	SEC28	YHR200W	RPN10
YLR324W	PEX30	YMR204C	INP1
YLR381W	CTF3	YJL183W	MNN11
YBR108W	YBR108W	YLR370C	ARC18
YMR205C	PFK2	YDR245W	MNN10
YHR012W	VPS29	YCR086W	CSM1
YBR131W	CCZ1	YPL161C	BEM4
YER143W	DDI1	YOL018C	TLG2
YLR094C	GIS3	YLR093C	NYV1
YOL030W	GAS5	YJR035W	RAD26
YBR173C	UMP1	YMR230W	RPS10B
YDR329C	PEX3	YDR265W	PEX10
YKL116C	PRR1	YGL003C	CDH1
YLR370C	ARC18	YDL226C	GCS1
YMR198W	CIK1	YDR254W	CHL4

Predictions at threshold level 0.80 continued...			
Gene1 of predicted pair		Gene2 of predicted pair	
Systematic Name	Standard Name	Systematic Name	Standard Name
YGL060W	YBP2	YPL106C	SSE1
YOR043W	WHI2	YDL006W	PTC1
YEL044W	IES6	YMR089C	YTA12
YKR001C	VPS1	YHR039C-A	VMA10
YOL111C	MDY2	YDL077C	VAM6
YBL103C	RTG3	YIL133C	RPL16A
YBR057C	MUM2	YPL057C	SUR1
YML095C	RAD10	YBL087C	RPL23A
YPR084W	YPR084W	YNL025C	SSN8
YDR137W	RGP1	YDR207C	UME6
YBR019C	GAL10	YER122C	GLO3
YBL008W	HIR1	YDR225W	HTA1
YLR399C	BDF1	YDR289C	RTT103
YLR095C	IOC2	YDR137W	RGP1
YOR080W	DIA2	YML095C	RAD10
YNL025C	SSN8	YER139C	YER139C
YPL057C	SUR1	YBR036C	CSG2
YDL223C	HBT1	YNL307C	MCK1
YPL022W	RAD1	YLR381W	CTF3
YJL176C	SWI3	YHR167W	THP2
YBR107C	IML3	YCR086W	CSM1
YLR452C	SST2	YOL081W	IRA2
YLR371W	ROM2	YOR014W	RTS1
YFR031C-A	RPL2A	YHR060W	VMA22
YLR234W	TOP3	YER162C	RAD4
YDL052C	SLC1	YOR059C	YOR059C
YOR083W	WHI5	YML095C	RAD10
YDL006W	PTC1	YDL192W	ARF1
YML012W	ERV25	YKR014C	YPT52

Predictions at threshold level 0.80 continued...			
Gene1 of predicted pair		Gene2 of predicted pair	
Systematic Name	Standard Name	Systematic Name	Standard Name
YPR131C	NAT3	YGL003C	CDH1
YGR188C	BUB1	YPR119W	CLB2
YOR195W	SLK19	YDR260C	SWM1
YLL039C	UBI4	YJL006C	CTK2
YGL135W	RPL1B	YKR014C	YPT52
YDR448W	ADA2	YNL107W	YAF9
YKL048C	ELM1	YDR254W	CHL4
YIL151C	YIL151C	YPL240C	HSP82
YPL024W	RMI1	YDL155W	CLB3
YJL149W	YJL149W	YML088W	UFO1
YMR055C	BUB2	YGL253W	HXK2
YNL297C	MON2	YPL161C	BEM4
YIL053W	RHR2	YMR153W	NUP53
YKL002W	DID4	YKL041W	VPS24
YLR090W	XDJ1	YLR093C	NYV1
YNL016W	PUB1	YCR086W	CSM1
YMR004W	MVP1	YDL077C	VAM6
YLR399C	BDF1	YOL054W	PSH1
YPL213W	LEA1	YJL183W	MNN11
YDR004W	RAD57	YDR146C	SWI5
YMR192W	GYL1	YBR108W	YBR108W
YKL116C	PRR1	YPR046W	MCM16
YKL032C	IXR1	YIL008W	URM1
YMR198W	CIK1	YOR141C	ARP8
YDR137W	RGP1	YNL307C	MCK1
YLR119W	SRN2	YER139C	YER139C
YKL117W	SBA1	YML057W	CMP2
YMR026C	PEX12	YMR142C	RPL13B
YFR031C-A	RPL2A	YOR132W	VPS17

Predictions at threshold level 0.80 continued...			
Gene1 of predicted pair		Gene2 of predicted pair	
Systematic Name	Standard Name	Systematic Name	Standard Name
YLR337C	VRP1	YAR014C	BUD14
YHR081W	LRP1	YOL121C	RPS19A
YLR410W	VIP1	YLR306W	UBC12
YIL148W	RPL40A	YNL199C	GCR2
YGR188C	BUB1	YDR207C	UME6
YLR090W	XDJ1	YOL121C	RPS19A
YER144C	UBP5	YCR053W	THR4
YJL004C	SYS1	YNL051W	COG5
YMR224C	MRE11	YHR200W	RPN10
YBR200W	BEM1	YMR199W	CLN1
YFR010W	UBP6	YPL253C	VIK1
YMR038C	CCS1	YML028W	TSA1
YDR004W	RAD57	YMR039C	SUB1
YDR176W	NGG1	YPL248C	GAL4
YGL212W	VAM7	YJR033C	RAV1
YER040W	GLN3	YDR207C	UME6
YNL215W	IES2	YOR213C	SAS5
YJL154C	VPS35	YOR035C	SHE4
YOR069W	VPS5	YPL161C	BEM4
YJL124C	LSM1	YOR043W	WHI2
YPR051W	MAK3	YDR153C	ENT5
YLR381W	CTF3	YOR358W	HAP5
YBR108W	YBR108W	YKL126W	YPK1
YLR447C	VMA6	YKR020W	VPS51
YPR036W	VMA13	YKL048C	ELM1
YKL010C	UFD4	YPL161C	BEM4
YMR199W	CLN1	YGR059W	SPR3
YLR337C	VRP1	YER139C	YER139C
YJL183W	MNN11	YBL025W	RRN10

Predictions at threshold level 0.80 continued...			
Gene1 of predicted pair		Gene2 of predicted pair	
Systematic Name	Standard Name	Systematic Name	Standard Name
YLR234W	TOP3	YMR167W	MLH1
YBR112C	CYC8	YCR084C	TUP1
YOR195W	SLK19	YBL087C	RPL23A
YGR261C	APL6	YHR039C-A	VMA10
YJL036W	SNX4	YOL108C	INO4
YNL025C	SSN8	YER122C	GLO3
YMR198W	CIK1	YBL047C	EDE1
YER161C	SPT2	YER122C	GLO3
YGR105W	VMA21	YPL120W	VPS30
YOR080W	DIA2	YJL187C	SWE1
YDR146C	SWI5	YBR260C	RGD1
YBR200W	BEM1	YER044C	ERG28
YBL079W	NUP170	YGL016W	KAP122
YHR157W	REC104	YHL022C	SPO11
YJR084W	CSN12	YJL095W	BCK1
YPR120C	CLB5	YGL252C	RTG2
YDR293C	SSD1	YNL044W	YIP3
YMR202W	ERG2	YDR318W	MCM21
YPL253C	VIK1	YLR371W	ROM2
YLL007C	YLL007C	YER110C	KAP123
YOR076C	SKI7	YLR398C	SKI2
YBR107C	IML3	YAR014C	BUD14
YPL115C	BEM3	YBR171W	SEC66
YER044C	ERG28	YLR180W	SAM1
YNR052C	POP2	YKL110C	KTI12
YKR020W	VPS51	YHR115C	DMA1
YPR036W	VMA13	YLR320W	MMS22
YMR133W	REC114	YDL192W	ARF1
YLR337C	VRP1	YEL036C	ANP1

Predictions at threshold level 0.80 continued...			
Gene1 of predicted pair		Gene2 of predicted pair	
Systematic Name	Standard Name	Systematic Name	Standard Name
YMR016C	SOK2	YNR072W	HXT17
YDR245W	MNN10	YPL089C	RLM1
YNL201C	PSY2	YLR381W	CTF3
YIL034C	CAP2	YDR389W	SAC7
YML115C	VAN1	YFR036W	CDC26
YJR059W	PTK2	YMR273C	ZDS1
YLR386W	VAC14	YOR035C	SHE4
YHR084W	STE12	YBL016W	FUS3
YDL101C	DUN1	YBL088C	TEL1
YGL250W	RMR1	YGL019W	CKB1
YCR024C-A	PMP1	YMR273C	ZDS1
YLR119W	SRN2	YMR133W	REC114
YDR378C	LSM6	YJL183W	MNN11
YMR109W	MYO5	YCR086W	CSM1
YMR198W	CIK1	YJL006C	CTK2
YHR134W	WSS1	YGL253W	HXK2
YJR033C	RAV1	YDR207C	UME6
YOR213C	SAS5	YER139C	YER139C
YPL220W	RPL1A	YKR026C	GCN3
YDR363W-A	SEM1	YNL236W	SIN4
YHR039C-A	VMA10	YGL124C	MON1
YLR288C	MEC3	YGR081C	SLX9
YFR010W	UBP6	YLR182W	SWI6
YCL037C	SRO9	YEL053C	MAK10
YLR192C	HCR1	YHR081W	LRP1
YGR020C	VMA7	YJR060W	CBF1
YBR171W	SEC66	YCR086W	CSM1
YJR140C	HIR3	YJR032W	CPR7
YKL032C	IXR1	YKL041W	VPS24

Predictions at threshold level 0.80 continued...			
Gene1 of predicted pair		Gene2 of predicted pair	
Systematic Name	Standard Name	Systematic Name	Standard Name
YCR024C-A	PMP1	YML057W	CMP2
YBR171W	SEC66	YNL041C	COG6
YBR108W	YBR108W	YHR200W	RPN10
YPL234C	TFP3	YNL041C	COG6
YLR170C	APS1	YHL019C	APM2
YMR224C	MRE11	YER122C	GLO3
YGL237C	HAP2	YEL053C	MAK10
YHR134W	WSS1	YOL030W	GAS5
YCR020C-A	MAK31	YJL053W	PEP8
YKL048C	ELM1	YER122C	GLO3
YLR288C	MEC3	YGL148W	ARO2
YBR200W	BEM1	YMR311C	GLC8
YLR079W	SIC1	YKR061W	KTR2
YKR059W	TIF1	YGL019W	CKB1
YKR099W	BAS1	YER040W	GLN3
YBR131W	CCZ1	YGR135W	PRE9
YER114C	BOI2	YDR309C	GIC2
YOR112W	CEX1	YCR066W	RAD18
YPL024W	RMI1	YNL076W	MKS1
YMR142C	RPL13B	YHR200W	RPN10
YKR099W	BAS1	YDL192W	ARF1
YAR014C	BUD14	YDR207C	UME6
YML115C	VAN1	YEL060C	PRB1
YCL037C	SRO9	YER120W	SCS2
YGR275W	RTT102	YOR290C	SNF2
YOL018C	TLG2	YDR207C	UME6
YJL154C	VPS35	YGR261C	APL6
YFR036W	CDC26	YML071C	COG8
YDR329C	PEX3	YDR142C	PEX7

Predictions at threshold level 0.80 continued...			
Gene1 of predicted pair		Gene2 of predicted pair	
Systematic Name	Standard Name	Systematic Name	Standard Name
YMR224C	MRE11	YKR095W	MLP1
YDR080W	VPS41	YGL124C	MON1
YHR156C	LIN1	YER044C	ERG28
YBR278W	DPB3	YBR274W	CHK1
YLR337C	VRP1	YKL126W	YPK1
YFR031C-A	RPL2A	YPL234C	TFP3
YKL101W	HSL1	YGL031C	RPL24A
YHR013C	ARD1	YPR046W	MCM16
YHR156C	LIN1	YPL195W	APL5
YKL139W	CTK1	YNL225C	CNM67
YNL248C	RPA49	YGR270W	YTA7
YJL154C	VPS35	YBR260C	RGD1
YDR004W	RAD57	YJR090C	GRR1
YLR192C	HCR1	YLR398C	SKI2
YMR016C	SOK2	YPL161C	BEM4
YOR106W	VAM3	YER096W	SHC1
YMR095C	SNO1	YPL037C	EGD1
YLR064W	YLR064W	YHR114W	BZZ1
YHR111W	UBA4	YNL079C	TPM1
YHR060W	VMA22	YNL076W	MKS1
YGL066W	SGF73	YJR135C	MCM22
YBR290W	BSD2	YAL002W	VPS8
YJL124C	LSM1	YOL090W	MSH2
YHR178W	STB5	YPL253C	VIK1
YBL058W	SHP1	YDR477W	SNF1
YNL058C	YNL058C	YOR066W	MSA1
YDR174W	HMO1	YHR013C	ARD1
YDR254W	CHL4	YDR318W	MCM21
YGL060W	YBP2	YPR131C	NAT3

Predictions at threshold level 0.80 continued...			
Gene1 of predicted pair		Gene2 of predicted pair	
Systematic Name	Standard Name	Systematic Name	Standard Name
YPR084W	YPR084W	YPL037C	EGD1
YOL111C	MDY2	YPL137C	GIP3
YGL115W	SNF4	YOR327C	SNC2
YEL065W	SIT1	YOR337W	TEA1
YBR173C	UMP1	YKR035W-A	DID2
YJR056C	YJR056C	YBL085W	BOI1
YBR108W	YBR108W	YMR230W	RPS10B
YNL215W	IES2	YPL253C	VIK1
YGL212W	VAM7	YNL076W	MKS1
YOR358W	HAP5	YPL246C	RBD2
YMR026C	PEX12	YEL053C	MAK10
YJR056C	YJR056C	YMR152W	YIM1
YBL088C	TEL1	YPR072W	NOT5
YDR443C	SSN2	YKR099W	BAS1
YPR120C	CLB5	YLR425W	TUS1
YFR031C-A	RPL2A	YEL051W	VMA8
YLR313C	SPH1	YLR362W	STE11
YER162C	RAD4	YGL124C	MON1
YOR195W	SLK19	YMR202W	ERG2
YMR205C	PFK2	YPL161C	BEM4
YDR309C	GIC2	YGR152C	RSR1
YAR014C	BUD14	YKR030W	GMH1
YHR012W	VPS29	YLR371W	ROM2
YDR108W	GSG1	YHR012W	VPS29
YGL133W	ITC1	YDR363W-A	SEM1
YOR080W	DIA2	YGL031C	RPL24A
YPL120W	VPS30	YNL051W	COG5
YGL178W	MPT5	YNL271C	BNI1
YPR017C	DSS4	YEL053C	MAK10

Predictions at threshold level 0.80 continued...			
Gene1 of predicted pair		Gene2 of predicted pair	
Systematic Name	Standard Name	Systematic Name	Standard Name
YDR137W	RGP1	YDL192W	ARF1
YNL218W	MGS1	YLR313C	SPH1
YGR081C	SLX9	YFL023W	BUD27
YNL243W	SLA2	YLR200W	YKE2
YJL124C	LSM1	YDR076W	RAD55
YBR200W	BEM1	YLR399C	BDF1
YJR005W	APL1	YDL226C	GCS1
YNL069C	RPL16B	YOR106W	VAM3
YGR105W	VMA21	YLR309C	IMH1
YDR176W	NGG1	YOR023C	AHC1
YBL051C	PIN4	YIL008W	URM1
YGR104C	SRB5	YBR200W	BEM1
YDR335W	MSN5	YNL236W	SIN4
YBR031W	RPL4A	YOR078W	BUD21
YKR035W-A	DID2	YLR180W	SAM1
YOR327C	SNC2	YGL124C	MON1
YMR016C	SOK2	YLR093C	NYV1
YBR260C	RGD1	YHR114W	BZZ1
YER087C-B	SBH1	YJR010C-A	SPC1
YOL111C	MDY2	YLR180W	SAM1
YJR135C	MCM22	YCR086W	CSM1
YMR055C	BUB2	YJL187C	SWE1
YER115C	SPR6	YCR024C-A	PMP1
YBL047C	EDE1	YOR043W	WHI2
YMR319C	FET4	YDR105C	TMS1
YCL037C	SRO9	YHL020C	OPI1
YLR095C	IOC2	YNL058C	YNL058C
YNR072W	HXT17	YDL077C	VAM6
YOR270C	VPH1	YGL124C	MON1

Predictions at threshold level 0.80 continued...			
Gene1 of predicted pair		Gene2 of predicted pair	
Systematic Name	Standard Name	Systematic Name	Standard Name
YFL026W	STE2	YJR125C	ENT3
YLR135W	SLX4	YMR038C	CCS1
YJL151C	SNA3	YBR108W	YBR108W
YLR083C	EMP70	YCR020C-A	MAK31
YGL174W	BUD13	YER161C	SPT2
YOR327C	SNC2	YLR370C	ARC18
YDR358W	GGA1	YDL077C	VAM6
YKL110C	KTI12	YCR086W	CSM1
YHR111W	UBA4	YDR155C	CPR1
YLR216C	CPR6	YBL103C	RTG3
YMR054W	STV1	YKL119C	VPH2
YOR195W	SLK19	YLR381W	CTF3
YNL058C	YNL058C	YPL195W	APL5
YER162C	RAD4	YNL076W	MKS1
YDR129C	SAC6	YDR323C	PEP7
YGR270W	YTA7	YDR144C	MKC7
YDR363W-A	SEM1	YDL155W	CLB3
YCR066W	RAD18	YPL120W	VPS30
YGR108W	CLB1	YOL091W	SPO21
YOR096W	RPS7A	YBR189W	RPS9B
YPL018W	CTF19	YOR141C	ARP8
YGL156W	AMS1	YKL103C	LAP4
YBR290W	BSD2	YER115C	SPR6
YHR161C	YAP1801	YKR001C	VPS1
YKL116C	PRR1	YMR152W	YIM1
YKL214C	YRA2	YOR001W	RRP6
YIL076W	SEC28	YDL083C	RPS16B
YOR008C	SLG1	YJL095W	BCK1
YDR129C	SAC6	YEL036C	ANP1

Predictions at threshold level 0.80 continued...			
Gene1 of predicted pair		Gene2 of predicted pair	
Systematic Name	Standard Name	Systematic Name	Standard Name
YER011W	TIR1	YPL178W	CBC2
YHR200W	RPN10	YLR032W	RAD5
YOR112W	CEX1	YHL020C	OPI1
YAL024C	LTE1	YFR036W	CDC26
YLR180W	SAM1	YDR207C	UME6
YAL024C	LTE1	YDR383C	NKP1
YPR017C	DSS4	YDL077C	VAM6
YDL160C	DHH1	YOR368W	RAD17
YER143W	DDI1	YNR049C	MSO1
YGR104C	SRB5	YNL215W	IES2
YOR101W	RAS1	YDL005C	MED2
YJL036W	SNX4	YDR080W	VPS41
YLR043C	TRX1	YIL008W	URM1
YJL187C	SWE1	YJL095W	BCK1
YAL024C	LTE1	YMR311C	GLC8
YOR337W	TEA1	YJR084W	CSN12
YPL120W	VPS30	YOR327C	SNC2
YJR063W	RPA12	YOL081W	IRA2
YDR443C	SSN2	YML071C	COG8
YNL041C	COG6	YPL120W	VPS30
YGL212W	VAM7	YLR452C	SST2
YCR053W	THR4	YBR280C	SAF1
YBR171W	SEC66	YOR035C	SHE4
YJL124C	LSM1	YKR001C	VPS1
YBL051C	PIN4	YKL048C	ELM1
YMR055C	BUB2	YJL138C	TIF2
YMR311C	GLC8	YMR230W	RPS10B
YFR010W	UBP6	YOR035C	SHE4
YGR249W	MGA1	YDR261C	EXG2

Predictions at threshold level 0.80 continued...			
Gene1 of predicted pair		Gene2 of predicted pair	
Systematic Name	Standard Name	Systematic Name	Standard Name
YHR098C	SFB3	YOR036W	PEP12
YKR035W-A	DID2	YJR032W	CPR7
YGR252W	GCN5	YNL236W	SIN4
YGL250W	RMR1	YPL018W	CTF19
YJR063W	RPA12	YDR183W	PLP1
YAL024C	LTE1	YPL253C	VIK1
YJL172W	CPS1	YER139C	YER139C
YGL033W	HOP2	YDR207C	UME6
YEL065W	SIT1	YBR108W	YBR108W
YGR072W	UPF3	YOL108C	INO4
YIL034C	CAP2	YLR233C	EST1
YBL058W	SHP1	YDL077C	VAM6
YLR254C	NDL1	YNL116W	DMA2
YJR024C	MDE1	YMR164C	MSS11
YCR065W	HCM1	YOR080W	DIA2
YOR023C	AHC1	YHL002W	HSE1
YCL001W	RER1	YAL002W	VPS8
YLR192C	HCR1	YBR036C	CSG2
YAR014C	BUD14	YPR119W	CLB2
YKR020W	VPS51	YJR032W	CPR7
YGL135W	RPL1B	YGR133W	PEX4
YLR386W	VAC14	YCR086W	CSM1
YIL076W	SEC28	YPL234C	TFP3
YMR116C	ASC1	YML115C	VAN1
YJL095W	BCK1	YJL190C	RPS22A
YOR039W	CKB2	YGL250W	RMR1
YIL103W	DPH1	YAR014C	BUD14
YGL147C	RPL9A	YIL076W	SEC28
YKL116C	PRR1	YGL212W	VAM7

Predictions at threshold level 0.80 continued...			
Gene1 of predicted pair		Gene2 of predicted pair	
Systematic Name	Standard Name	Systematic Name	Standard Name
YDL192W	ARF1	YLR370C	ARC18
YKR072C	SIS2	YLR313C	SPH1
YBR194W	SOY1	YOR001W	RRP6
YPL253C	VIK1	YDR318W	MCM21
YOR080W	DIA2	YDR378C	LSM6
YGL087C	MMS2	YGL253W	HXK2
YKL048C	ELM1	YGR270W	YTA7
YDR176W	NGG1	YLR357W	RSC2
YBR171W	SEC66	YLR447C	VMA6
YHR086W	NAM8	YBR119W	MUD1
YIL159W	BNR1	YDL192W	ARF1
YCR081W	SRB8	YOR014W	RTS1
YGL095C	VPS45	YOR327C	SNC2
YDR320C	SWA2	YHL031C	GOS1
YNL325C	FIG4	YDR137W	RGP1
YOR066W	MSA1	YMR311C	GLC8
YKL032C	IXR1	YJL183W	MNN11
YOR066W	MSA1	YDR261C	EXG2
YLR360W	VPS38	YNL107W	YAF9
YMR142C	RPL13B	YDR358W	GGA1
YLR119W	SRN2	YKR099W	BAS1
YBR217W	ATG12	YMR016C	SOK2
YLR150W	STM1	YJR135C	MCM22
YOR106W	VAM3	YHR060W	VMA22
YJR005W	APL1	YPR046W	MCM16
YLR135W	SLX4	YDL013W	SLX5
YLR119W	SRN2	YPR036W	VMA13
YHR111W	UBA4	YHR114W	BZZ1
YFR043C	IRC6	YMR243C	ZRC1

Predictions at threshold level 0.80 continued...			
Gene1 of predicted pair		Gene2 of predicted pair	
Systematic Name	Standard Name	Systematic Name	Standard Name
YJL095W	BCK1	YDR310C	SUM1
YLR234W	TOP3	YOR127W	RGA1
YPR072W	NOT5	YLR357W	RSC2
YBL008W	HIR1	YCR086W	CSM1
YNL215W	IES2	YML112W	CTK3
YLR119W	SRN2	YMR311C	GLC8
YJL020C	BBC1	YGL095C	VPS45
YJR063W	RPA12	YNL116W	DMA2
YGL066W	SGF73	YDL013W	SLX5
YOR033C	EXO1	YCR077C	PAT1
YPL057C	SUR1	YPL195W	APL5
YBR173C	UMP1	YGL025C	PGD1
YDR484W	VPS52	YBL103C	RTG3
YBR217W	ATG12	YDL083C	RPS16B
YGL087C	MMS2	YJL124C	LSM1
YLL007C	YLL007C	YJL154C	VPS35
YLR109W	AHP1	YER042W	MXR1
YJL183W	MNN11	YJL190C	RPS22A
YLR095C	IOC2	YML115C	VAN1
YCL067C	HMLALPHA2	YPL240C	HSP82
YLR090W	XDJ1	YBL103C	RTG3
YML103C	NUP188	YKL048C	ELM1
YMR106C	YKU80	YML028W	TSA1
YFR031C-A	RPL2A	YPR046W	MCM16
YDR469W	SDC1	YDR155C	CPR1
YPR046W	MCM16	YDR155C	CPR1
YFR010W	UBP6	YML028W	TSA1
YCR034W	FEN1	YGL250W	RMR1
YDR477W	SNF1	YPL256C	CLN2

Predictions at threshold level 0.80 continued...			
Gene1 of predicted pair		Gene2 of predicted pair	
Systematic Name	Standard Name	Systematic Name	Standard Name
YOR127W	RGA1	YDL006W	PTC1
YJL029C	VPS53	YGL095C	VPS45
YHL031C	GOS1	YNL041C	COG6
YHR013C	ARD1	YDR183W	PLP1
YER116C	SLX8	YLR410W	VIP1
YNL307C	MCK1	YML071C	COG8
YPL240C	HSP82	YER100W	UBC6
YDR225W	HTA1	YNR072W	HXT17
YNL206C	RTT106	YEL056W	HAT2
YPL161C	BEM4	YDL192W	ARF1
YBR260C	RGD1	YJL095W	BCK1
YHR052W	CIC1	YLR150W	STM1
YKL110C	KTI12	YBL071W-A	KTI11
YHR160C	PEX18	YLR191W	PEX13
YIL159W	BNR1	YDR137W	RGP1
YPL129W	TAF14	YNL031C	HHT2
YPL050C	MNN9	YOR035C	SHE4
YHL031C	GOS1	YJL004C	SYS1
YDR004W	RAD57	YMR231W	PEP5
YDR318W	MCM21	YGL003C	CDH1
YIL036W	CST6	YNL307C	MCK1
YEL053C	MAK10	YEL056W	HAT2
YBL008W	HIR1	YMR167W	MLH1
YGR249W	MGA1	YMR214W	SCJ1
YPR072W	NOT5	YDR071C	PAA1
YNL307C	MCK1	YML028W	TSA1
YDR245W	MNN10	YDR456W	NHX1
YHR039C-A	VMA10	YOR038C	HIR2
YBR200W	BEM1	YBL103C	RTG3

Predictions at threshold level 0.80 continued...			
Gene1 of predicted pair		Gene2 of predicted pair	
Systematic Name	Standard Name	Systematic Name	Standard Name
YLR292C	SEC72	YMR055C	BUB2
YDR293C	SSD1	YOR127W	RGA1
YLR192C	HCR1	YLR370C	ARC18
YNL307C	MCK1	YOL111C	MDY2
YGL178W	MPT5	YCR009C	RVS161
YLR093C	NYV1	YML071C	COG8
YPL253C	VIK1	YGR270W	YTA7
YPR120C	CLB5	YKL161C	YKL161C
YOR132W	VPS17	YPL037C	EGD1
YOL044W	PEX15	YAL055W	PEX22
YPR029C	APL4	YJR125C	ENT3
YGL212W	VAM7	YCL037C	SRO9
YNL291C	MID1	YPR120C	CLB5
YDR225W	HTA1	YLR381W	CTF3
YFR031C-A	RPL2A	YGL223C	COG1
YMR080C	NAM7	YNL218W	MGS1
YDR309C	GIC2	YOR327C	SNC2
YJR135C	MCM22	YJR032W	CPR7
YJL183W	MNN11	YLR233C	EST1
YCR077C	PAT1	YLR350W	ORM2
YMR224C	MRE11	YBR228W	SLX1
YER116C	SLX8	YHR013C	ARD1
YLR337C	VRP1	YPL050C	MNN9
YDL184C	RPL41A	YBR200W	BEM1
YNR072W	HXT17	YOR043W	WHI2
YBL071W-A	KTI11	YIL103W	DPH1
YOL111C	MDY2	YKR035W-A	DID2
YOR043W	WHI2	YDR469W	SDC1
YJL127C	SPT10	YJL124C	LSM1

Predictions at threshold level 0.80 continued...			
Gene1 of predicted pair		Gene2 of predicted pair	
Systematic Name	Standard Name	Systematic Name	Standard Name
YAL024C	LTE1	YDR335W	MSN5
YPL018W	CTF19	YKL114C	APN1
YLR119W	SRN2	YPR046W	MCM16
YDR080W	VPS41	YHR060W	VMA22
YBR107C	IML3	YDR254W	CHL4
YIL156W	UBP7	YNL199C	GCR2
YJR060W	CBF1	YHR012W	VPS29
YDL083C	RPS16B	YHR081W	LRP1
YHR156C	LIN1	YLR398C	SKI2
YBR042C	CST26	YGL215W	CLG1
YLR254C	NDL1	YAR014C	BUD14
YER114C	BOI2	YBR200W	BEM1
YJL172W	CPS1	YNL041C	COG6
YOR043W	WHI2	YHR158C	KEL1
YJL176C	SWI3	YGR275W	RTT102
YBR194W	SOY1	YPL018W	CTF19
YDR443C	SSN2	YPL042C	SSN3
YOR043W	WHI2	YBR280C	SAF1
YLR079W	SIC1	YPR046W	MCM16
YKL025C	PAN3	YBL021C	HAP3
YNL054W	VAC7	YGR188C	BUB1
YDR295C	HDA2	YDR318W	MCM21
YPL024W	RMI1	YMR055C	BUB2
YOR290C	SNF2	YML001W	YPT7
YLL007C	YLL007C	YOL067C	RTG1
YKR099W	BAS1	YLR370C	ARC18
YBR127C	VMA2	YKL041W	VPS24
YIL053W	RHR2	YML071C	COG8
YDR379W	RGA2	YHR111W	UBA4

Predictions at threshold level 0.80 continued...			
**Gene1 of predicted pair**		**Gene2 of predicted pair**	
**Systematic Name**	**Standard Name**	**Systematic Name**	**Standard Name**
YFR036W	CDC26	YGL003C	CDH1
YCR086W	CSM1	YEL056W	HAT2
YER144C	UBP5	YOR035C	SHE4
YMR224C	MRE11	YMR199W	CLN1
YGL033W	HOP2	YMR153W	NUP53
YNL069C	RPL16B	YKR030W	GMH1
YLR094C	GIS3	YDR358W	GGA1
YDR245W	MNN10	YGL200C	EMP24
YKL025C	PAN3	YPL161C	BEM4
YKL139W	CTK1	YKR099W	BAS1
YFR010W	UBP6	YDR261C	EXG2
YPL057C	SUR1	YHR077C	NMD2
YMR080C	NAM7	YBR228W	SLX1
YJL154C	VPS35	YEL043W	YEL043W
YFR010W	UBP6	YGL163C	RAD54
YDR108W	GSG1	YBR131W	CCZ1
YPR046W	MCM16	YMR214W	SCJ1
YFR010W	UBP6	YBR020W	GAL1
YOR014W	RTS1	YLR182W	SWI6
YKL139W	CTK1	YBR107C	IML3
YOR023C	AHC1	YJR032W	CPR7
YGL133W	ITC1	YKL048C	ELM1
YGL174W	BUD13	YGR270W	YTA7
YNL289W	PCL1	YHR115C	DMA1
YHL027W	RIM101	YPR173C	VPS4
YPL137C	GIP3	YBR019C	GAL10
YPL024W	RMI1	YER044C	ERG28
YLR234W	TOP3	YBR114W	RAD16
YNL325C	FIG4	YNL238W	KEX2

Predictions at threshold level 0.80 continued...			
Gene1 of predicted pair		Gene2 of predicted pair	
Systematic Name	Standard Name	Systematic Name	Standard Name
YLR288C	MEC3	YER161C	SPT2
YJL060W	BNA3	YJR060W	CBF1
YGL147C	RPL9A	YLR320W	MMS22
YDR378C	LSM6	YKL110C	KTI12
YGR108W	CLB1	YHL002W	HSE1
YDR318W	MCM21	YNL107W	YAF9
YBR107C	IML3	YHL002W	HSE1
YDR264C	AKR1	YJR086W	STE18
YNL215W	IES2	YAL002W	VPS8
YGR104C	SRB5	YDR477W	SNF1
YDL020C	RPN4	YGR081C	SLX9
YJL053W	PEP8	YAR014C	BUD14
YEL053C	MAK10	YLR180W	SAM1
YBR194W	SOY1	YMR038C	CCS1
YNL031C	HHT2	YOR064C	YNG1
YGL095C	VPS45	YOR036W	PEP12
YPL240C	HSP82	YJL184W	GON7
YDR108W	GSG1	YDR153C	ENT5
YOR112W	CEX1	YPL234C	TFP3
YOR043W	WHI2	YNL076W	MKS1
YDL020C	RPN4	YNL307C	MCK1
YOR043W	WHI2	YBR019C	GAL10
YGR019W	UGA1	YDR293C	SSD1
YDL116W	NUP84	YGL163C	RAD54
YHL027W	RIM101	YJL095W	BCK1
YMR284W	YKU70	YOR080W	DIA2
YML097C	VPS9	YMR089C	YTA12
YPL057C	SUR1	YGL003C	CDH1
YDR213W	UPC2	YMR070W	MOT3

Predictions at threshold level 0.80 continued...			
Gene1 of predicted pair		Gene2 of predicted pair	
Systematic Name	Standard Name	Systematic Name	Standard Name
YDR358W	GGA1	YOL081W	IRA2
YML117W	NAB6	YBR082C	UBC4
YJL136C	RPS21B	YKR048C	NAP1
YDR484W	VPS52	YML001W	YPT7
YMR224C	MRE11	YDR260C	SWM1
YNL041C	COG6	YLR398C	SKI2
YDL042C	SIR2	YLR399C	BDF1
YGR270W	YTA7	YLR350W	ORM2
YDR080W	VPS41	YCR066W	RAD18
YGR054W	YGR054W	YGL253W	HXK2
YGL025C	PGD1	YBR171W	SEC66
YKL129C	MYO3	YOR035C	SHE4
YMR026C	PEX12	YBR280C	SAF1
YDL116W	NUP84	YHR111W	UBA4
YPR017C	DSS4	YCL001W	RER1
YPR036W	VMA13	YOL121C	RPS19A
YBR173C	UMP1	YDL223C	HBT1
YBL102W	SFT2	YLR360W	VPS38
YLR386W	VAC14	YFR036W	CDC26
YHR156C	LIN1	YMR214W	SCJ1
YDR080W	VPS41	YPR032W	SRO7
YOR038C	HIR2	YER139C	YER139C
YLR192C	HCR1	YBR082C	UBC4
YOR290C	SNF2	YKR035W-A	DID2
YNL243W	SLA2	YDL006W	PTC1
YHL031C	GOS1	YEL036C	ANP1
YLR192C	HCR1	YBR280C	SAF1
YNL307C	MCK1	YLR032W	RAD5
YHR134W	WSS1	YHR167W	THP2

Predictions at threshold level 0.80 continued...			
Gene1 of predicted pair		Gene2 of predicted pair	
Systematic Name	Standard Name	Systematic Name	Standard Name
YDR329C	PEX3	YDL065C	PEX19
YER161C	SPT2	YHR039C-A	VMA10
YLR315W	NKP2	YNL107W	YAF9
YNR072W	HXT17	YJL183W	MNN11
YLR309C	IMH1	YNR032W	PPG1
YJR056C	YJR056C	YOR043W	WHI2
YLR095C	IOC2	YER144C	UBP5
YLR337C	VRP1	YLR292C	SEC72
YOR337W	TEA1	YPR030W	CSR2
YGL124C	MON1	YER122C	GLO3
YML038C	YMD8	YHR012W	VPS29
YLR353W	BUD8	YOR301W	RAX1
YLR119W	SRN2	YGL078C	DBP3
YNL087W	TCB2	YOR327C	SNC2
YDR363W-A	SEM1	YHR167W	THP2
YNL079C	TPM1	YPR119W	CLB2
YIL053W	RHR2	YBR173C	UMP1
YJL176C	SWI3	YBR082C	UBC4
YFR036W	CDC26	YNL076W	MKS1
YNL215W	IES2	YDR335W	MSN5
YMR224C	MRE11	YOR213C	SAS5
YHR073W	OSH3	YPL178W	CBC2
YLR320W	MMS22	YDR261C	EXG2
YDR108W	GSG1	YKL119C	VPH2
YER116C	SLX8	YDR318W	MCM21
YMR152W	YIM1	YNL076W	MKS1
YML038C	YMD8	YJR033C	RAV1
YDR378C	LSM6	YKR026C	GCN3
YJL124C	LSM1	YBR215W	HPC2

Predictions at threshold level 0.80 continued...			
Gene1 of predicted pair		Gene2 of predicted pair	
Systematic Name	Standard Name	Systematic Name	Standard Name
YNL218W	MGS1	YOR076C	SKI7
YER096W	SHC1	YLR425W	TUS1
YKL160W	ELF1	YLR306W	UBC12
YMR038C	CCS1	YGL031C	RPL24A
YJL176C	SWI3	YGR072W	UPF3
YKL025C	PAN3	YJR032W	CPR7
YGL147C	RPL9A	YLR370C	ARC18
YGR108W	CLB1	YLR429W	CRN1
YIL053W	RHR2	YEL060C	PRB1
YDR443C	SSN2	YOR066W	MSA1
YDL077C	VAM6	YLR309C	IMH1
YPL158C	YPL158C	YJL095W	BCK1
YGL087C	MMS2	YLR032W	RAD5
YBL051C	PIN4	YGR270W	YTA7
YDR071C	PAA1	YNL107W	YAF9
YPR084W	YPR084W	YCR077C	PAT1
YEL053C	MAK10	YEL043W	YEL043W
YDR080W	VPS41	YKR001C	VPS1
YBR200W	BEM1	YPL057C	SUR1
YDR443C	SSN2	YOL051W	GAL11
YML095C	RAD10	YJL190C	RPS22A
YGL060W	YBP2	YLR381W	CTF3
YLR150W	STM1	YHR200W	RPN10
YOR109W	INP53	YPR032W	SRO7
YJR059W	PTK2	YMR199W	CLN1
YDL101C	DUN1	YCR086W	CSM1
YDR245W	MNN10	YOR275C	RIM20
YLR094C	GIS3	YJR032W	CPR7
YPL195W	APL5	YPL120W	VPS30

Predictions at threshold level 0.80 continued...			
Gene1 of predicted pair		Gene2 of predicted pair	
Systematic Name	Standard Name	Systematic Name	Standard Name
YMR095C	SNO1	YHR060W	VMA22
YMR109W	MYO5	YHL027W	RIM101
YOL126C	MDH2	YML057W	CMP2
YNR052C	POP2	YDR378C	LSM6
YKL048C	ELM1	YDR144C	MKC7
YLR094C	GIS3	YOR066W	MSA1
YNL051W	COG5	YAL002W	VPS8
YMR055C	BUB2	YKL101W	HSL1
YIL050W	PCL7	YPL219W	PCL8
YLR381W	CTF3	YKR061W	KTR2
YPL024W	RMI1	YBR194W	SOY1
YBR260C	RGD1	YOR035C	SHE4
YNL031C	HHT2	YEL056W	HAT2
YKL048C	ELM1	YLR410W	VIP1
YFL023W	BUD27	YER044C	ERG28
YPL022W	RAD1	YER116C	SLX8
YMR224C	MRE11	YDL192W	ARF1
YKL139W	CTK1	YNL116W	DMA2
YBR111W-A	SUS1	YOL148C	SPT20
YGL066W	SGF73	YPL161C	BEM4
YBR107C	IML3	YJL190C	RPS22A
YGR104C	SRB5	YKL139W	CTK1
YNL325C	FIG4	YDR484W	VPS52
YJR032W	CPR7	YDR183W	PLP1
YNL215W	IES2	YBR215W	HPC2
YOR145C	PNO1	YKL143W	LTV1
YDL116W	NUP84	YOR358W	HAP5
YMR224C	MRE11	YFR036W	CDC26
YPR131C	NAT3	YDR207C	UME6

Predictions at threshold level 0.80 continued...			
**Gene1 of predicted pair**		**Gene2 of predicted pair**	
**Systematic Name**	**Standard Name**	**Systematic Name**	**Standard Name**
YBR200W	BEM1	YER162C	RAD4
YML103C	NUP188	YBR017C	KAP104
YKR069W	MET1	YKL129C	MYO3
YNL291C	MID1	YPR051W	MAK3
YOR253W	NAT5	YHR013C	ARD1
YBR057C	MUM2	YGL212W	VAM7
YGL066W	SGF73	YGR252W	GCN5
YBR041W	FAT1	YBL101C	ECM21
YDR146C	SWI5	YFR034C	PHO4
YDR108W	GSG1	YAL002W	VPS8
YLR095C	IOC2	YGL115W	SNF4
YDL116W	NUP84	YNL307C	MCK1
YEL053C	MAK10	YDL006W	PTC1
YEL050C	RML2	YDR378C	LSM6
YIL076W	SEC28	YDR080W	VPS41
YHR156C	LIN1	YOL081W	IRA2
YDR156W	RPA14	YKL048C	ELM1
YDR137W	RGP1	YPL234C	TFP3
YNL307C	MCK1	YOR312C	RPL20B
YDL122W	UBP1	YJR099W	YUH1
YBL008W	HIR1	YGR108W	CLB1
YGL147C	RPL9A	YMR230W	RPS10B
YMR116C	ASC1	YBL047C	EDE1
YLR083C	EMP70	YLR309C	IMH1
YDR320C	SWA2	YKR001C	VPS1
YNL288W	CAF40	YDR310C	SUM1
YKL032C	IXR1	YCR077C	PAT1
YGL161C	YIP5	YML097C	VPS9
YOR141C	ARP8	YLR182W	SWI6

Predictions at threshold level 0.80 continued...			
Gene1 of predicted pair		Gene2 of predicted pair	
Systematic Name	Standard Name	Systematic Name	Standard Name
YPL256C	CLN2	YJR090C	GRR1
YML095C	RAD10	YKR048C	NAP1
YNL243W	SLA2	YBL061C	SKT5
YKR035W-A	DID2	YJR084W	CSN12
YER179W	DMC1	YNL230C	ELA1
YDR293C	SSD1	YPL037C	EGD1
YML115C	VAN1	YGL200C	EMP24
YLR288C	MEC3	YDR289C	RTT103
YHR052W	CIC1	YGL246C	RAI1
YBL051C	PIN4	YMR055C	BUB2
YDR484W	VPS52	YHR060W	VMA22
YIL159W	BNR1	YDL184C	RPL41A
YCR065W	HCM1	YPR119W	CLB2
YMR224C	MRE11	YNL248C	RPA49
YGR252W	GCN5	YHR108W	GGA2
YDR137W	RGP1	YPL037C	EGD1
YOR127W	RGA1	YNL206C	RTT106
YBL058W	SHP1	YBR280C	SAF1
YLR192C	HCR1	YJL183W	MNN11
YIL034C	CAP2	YBR108W	YBR108W
YOR083W	WHI5	YMR055C	BUB2
YJL053W	PEP8	YDL161W	ENT1
YER162C	RAD4	YJL095W	BCK1
YJR060W	CBF1	YMR054W	STV1
YLR135W	SLX4	YML028W	TSA1
YLR315W	NKP2	YER139C	YER139C
YHR060W	VMA22	YHR012W	VPS29
YML016C	PPZ1	YJR060W	CBF1
YKL139W	CTK1	YMR214W	SCJ1

Predictions at threshold level 0.80 continued...			
Gene1 of predicted pair		Gene2 of predicted pair	
Systematic Name	Standard Name	Systematic Name	Standard Name
YLR090W	XDJ1	YLR398C	SKI2
YDR443C	SSN2	YPL024W	RMI1
YJR056C	YJR056C	YPR046W	MCM16
YDR004W	RAD57	YMR055C	BUB2
YPL140C	MKK2	YJL095W	BCK1
YHR108W	GGA2	YHR158C	KEL1
YLR119W	SRN2	YKL116C	PRR1
YNL206C	RTT106	YGR252W	GCN5
YDR443C	SSN2	YNL307C	MCK1
YPR036W	VMA13	YDR261C	EXG2
YLR148W	PEP3	YAL002W	VPS8
YJL053W	PEP8	YNR032W	PPG1
YDR484W	VPS52	YOR327C	SNC2
YDR071C	PAA1	YGR135W	PRE9
YMR127C	SAS2	YGR188C	BUB1
YIL053W	RHR2	YNL087W	TCB2
YDR176W	NGG1	YNL236W	SIN4
YNL215W	IES2	YNL199C	GCR2
YPL253C	VIK1	YBR107C	IML3
YFR010W	UBP6	YNL032W	SIW14
YBR108W	YBR108W	YKR035W-A	DID2
YGL163C	RAD54	YJR035W	RAD26
YOR141C	ARP8	YNL107W	YAF9
YNL032W	SIW14	YKL126W	YPK1
YGR105W	VMA21	YJR032W	CPR7
YHR134W	WSS1	YJR135C	MCM22
YDR477W	SNF1	YBR010W	HHT1
YKR020W	VPS51	YCR077C	PAT1
YMR127C	SAS2	YPR051W	MAK3

Predictions at threshold level 0.80 continued...			
Gene1 of predicted pair		Gene2 of predicted pair	
Systematic Name	Standard Name	Systematic Name	Standard Name
YCL001W	RER1	YDL077C	VAM6
YNL032W	SIW14	YMR152W	YIM1
YDR293C	SSD1	YPL018W	CTF19
YGL054C	ERV14	YNL107W	YAF9
YDR392W	SPT3	YGL178W	MPT5
YKR072C	SIS2	YEL056W	HAT2
YOR127W	RGA1	YGL054C	ERV14
YOR083W	WHI5	YDR171W	HSP42
YNL206C	RTT106	YKL074C	MUD2
YDR137W	RGP1	YDR440W	DOT1
YDR379W	RGA2	YKR048C	NAP1
YGL124C	MON1	YMR311C	GLC8
YBR217W	ATG12	YLL042C	ATG10
YGR252W	GCN5	YKL160W	ELF1
YDR293C	SSD1	YJL183W	MNN11
YDR202C	RAV2	YPL105C	YPL105C
YOR069W	VPS5	YNL127W	FAR11
YHR060W	VMA22	YEL051W	VMA8
YPL037C	EGD1	YEL043W	YEL043W
YNL307C	MCK1	YNL082W	PMS1
YDR335W	MSN5	YDR183W	PLP1
YMR224C	MRE11	YGL115W	SNF4
YDR320C	SWA2	YGL223C	COG1
YGL033W	HOP2	YLR371W	ROM2
YDL116W	NUP84	YNL199C	GCR2
YGL175C	SAE2	YGL163C	RAD54
YIL159W	BNR1	YGL003C	CDH1
YHR111W	UBA4	YDR310C	SUM1
YDR383C	NKP1	YDR183W	PLP1

Predictions at threshold level 0.80 continued...			
Gene1 of predicted pair		Gene2 of predicted pair	
Systematic Name	Standard Name	Systematic Name	Standard Name
YMR142C	RPL13B	YBR200W	BEM1
YGL200C	EMP24	YBL047C	EDE1
YDR477W	SNF1	YGL212W	VAM7
YGR204W	ADE3	YEL046C	GLY1
YKL161C	YKL161C	YHR158C	KEL1
YBR020W	GAL1	YNL004W	HRB1
YGL060W	YBP2	YOR080W	DIA2
YMR224C	MRE11	YGL033W	HOP2
YKL116C	PRR1	YER122C	GLO3
YJL154C	VPS35	YBL102W	SFT2
YGR019W	UGA1	YEL053C	MAK10
YHR060W	VMA22	YNL127W	FAR11
YDR144C	MKC7	YNL107W	YAF9
YGL147C	RPL9A	YDL192W	ARF1
YML103C	NUP188	YKR095W	MLP1
YDR363W-A	SEM1	YGL115W	SNF4
YJL095W	BCK1	YCR053W	THR4
YKL126W	YPK1	YJR032W	CPR7
YOR140W	SFL1	YLR425W	TUS1
YOR109W	INP53	YPL161C	BEM4
YBL103C	RTG3	YPL161C	BEM4
YGL153W	PEX14	YJL006C	CTK2
YJR032W	CPR7	YBR036C	CSG2
YBR108W	YBR108W	YJR056C	YJR056C
YDR477W	SNF1	YFL023W	BUD27
YHR167W	THP2	YHR077C	NMD2
YBL008W	HIR1	YOL091W	SPO21
YAL024C	LTE1	YHR158C	KEL1
YPL096C-A	ERI1	YDL223C	HBT1

Predictions at threshold level 0.80 continued...			
Gene1 of predicted pair		Gene2 of predicted pair	
Systematic Name	Standard Name	Systematic Name	Standard Name
YLR216C	CPR6	YNL307C	MCK1
YEL043W	YEL043W	YPL120W	VPS30
YCR084C	TUP1	YNL236W	SIN4
YBR173C	UMP1	YKL048C	ELM1
YDR443C	SSN2	YML001W	YPT7
YDR383C	NKP1	YNL116W	DMA2
YAL024C	LTE1	YGL163C	RAD54
YGL223C	COG1	YHR060W	VMA22
YOR337W	TEA1	YBR200W	BEM1
YMR095C	SNO1	YDR477W	SNF1
YPL057C	SUR1	YKR035W-A	DID2
YER116C	SLX8	YKL101W	HSL1
YDL002C	NHP10	YAL002W	VPS8
YLR371W	ROM2	YER162C	RAD4
YKL204W	EAP1	YDR310C	SUM1
YPR120C	CLB5	YGL003C	CDH1
YER116C	SLX8	YJR090C	GRR1
YLR381W	CTF3	YNL307C	MCK1
YKR072C	SIS2	YNR052C	POP2
YLR119W	SRN2	YOR327C	SNC2
YBR194W	SOY1	YLR381W	CTF3
YPL024W	RMI1	YER040W	GLN3
YDR080W	VPS41	YDL006W	PTC1
YDR245W	MNN10	YGL212W	VAM7
YIL076W	SEC28	YEL051W	VMA8
YNL288W	CAF40	YBR107C	IML3
YJL154C	VPS35	YPL161C	BEM4
YOR001W	RRP6	YDR335W	MSN5
YLR381W	CTF3	YGL252C	RTG2

Predictions at threshold level 0.80 continued...			
Gene1 of predicted pair		Gene2 of predicted pair	
Systematic Name	Standard Name	Systematic Name	Standard Name
YDL020C	RPN4	YBR215W	HPC2
YML016C	PPZ1	YJR135C	MCM22
YDL116W	NUP84	YMR164C	MSS11
YBR108W	YBR108W	YNL166C	BNI5
YAL024C	LTE1	YJL183W	MNN11
YJR084W	CSN12	YLR032W	RAD5
YIL034C	CAP2	YAR014C	BUD14
YDR289C	RTT103	YOL081W	IRA2
YGR241C	YAP1802	YNR006W	VPS27
YDR254W	CHL4	YLR182W	SWI6
YGL174W	BUD13	YBR215W	HPC2
YLR192C	HCR1	YKR020W	VPS51
YNL032W	SIW14	YOL121C	RPS19A
YLR386W	VAC14	YDL184C	RPL41A
YGL054C	ERV14	YPL234C	TFP3
YBL102W	SFT2	YKR020W	VPS51
YDR484W	VPS52	YPL195W	APL5
YEL053C	MAK10	YGL253W	HXK2
YGL066W	SGF73	YNL307C	MCK1
YDR378C	LSM6	YDL226C	GCS1
YNL297C	MON2	YJR040W	GEF1
YBR050C	REG2	YER027C	GAL83
YGL133W	ITC1	YBL087C	RPL23A
YHL031C	GOS1	YPL050C	MNN9
YOR290C	SNF2	YKL126W	YPK1
YGL124C	MON1	YBR036C	CSG2
YOR290C	SNF2	YPL161C	BEM4
YIL034C	CAP2	YPL253C	VIK1
YOR270C	VPH1	YPL137C	GIP3

Predictions at threshold level 0.80 continued...			
Gene1 of predicted pair		Gene2 of predicted pair	
Systematic Name	Standard Name	Systematic Name	Standard Name
YAL024C	LTE1	YBR036C	CSG2
YMR224C	MRE11	YOR043W	WHI2
YBR171W	SEC66	YFR036W	CDC26
YHR200W	RPN10	YCR053W	THR4
YNL199C	GCR2	YMR125W	STO1
YGL115W	SNF4	YDL083C	RPS16B
YBR217W	ATG12	YPL161C	BEM4
YGL212W	VAM7	YPR046W	MCM16
YLR337C	VRP1	YGR135W	PRE9
YIL034C	CAP2	YPL018W	CTF19
YPL253C	VIK1	YER049W	TPA1
YFR031C-A	RPL2A	YOR043W	WHI2
YFR031C-A	RPL2A	YJL154C	VPS35
YOR337W	TEA1	YNL076W	MKS1
YGR270W	YTA7	YDR155C	CPR1
YDR439W	LRS4	YHR013C	ARD1
YHL031C	GOS1	YMR038C	CCS1
YMR273C	ZDS1	YFL047W	RGD2
YJL053W	PEP8	YKR030W	GMH1
YNL238W	KEX2	YJL140W	RPB4
YDR448W	ADA2	YDR469W	SDC1
YML071C	COG8	YMR214W	SCJ1
YOR132W	VPS17	YGL115W	SNF4
YLR064W	YLR064W	YPL246C	RBD2
YDR363W-A	SEM1	YER139C	YER139C
YDR108W	GSG1	YER120W	SCS2
YOL067C	RTG1	YBR114W	RAD16
YDR293C	SSD1	YKR020W	VPS51
YDR335W	MSN5	YPR046W	MCM16

Predictions at threshold level 0.80 continued...			
**Gene1 of predicted pair**		**Gene2 of predicted pair**	
**Systematic Name**	**Standard Name**	**Systematic Name**	**Standard Name**
YLR386W	VAC14	YOR066W	MSA1
YGR108W	CLB1	YDL047W	SIT4
YPL057C	SUR1	YDR289C	RTT103
YKR048C	NAP1	YLR182W	SWI6
YOR033C	EXO1	YMR199W	CLN1
YFR010W	UBP6	YKL126W	YPK1
YGR270W	YTA7	YAL002W	VPS8
YOL126C	MDH2	YBL079W	NUP170
YLL007C	YLL007C	YML095C	RAD10
YBR108W	YBR108W	YDR484W	VPS52
YLR018C	POM34	YBL079W	NUP170
YFR031C-A	RPL2A	YDL192W	ARF1
YHR144C	DCD1	YMR201C	RAD14
YDR363W-A	SEM1	YBL087C	RPL23A
YJR063W	RPA12	YDR071C	PAA1
YLR396C	VPS33	YPL045W	VPS16
YJR090C	GRR1	YGL252C	RTG2
YLR093C	NYV1	YBR036C	CSG2
YGL095C	VPS45	YDR323C	PEP7
YPL256C	CLN2	YGL031C	RPL24A
YGR020C	VMA7	YDR153C	ENT5
YBR042C	CST26	YGR105W	VMA21
YDR363W-A	SEM1	YDR260C	SWM1
YOR076C	SKI7	YOR145C	PNO1
YBR194W	SOY1	YDL226C	GCS1
YHR013C	ARD1	YNL238W	KEX2
YML071C	COG8	YCR053W	THR4
YOR023C	AHC1	YPL161C	BEM4
YHR134W	WSS1	YGL151W	NUT1

Predictions at threshold level 0.80 continued...			
Gene1 of predicted pair		Gene2 of predicted pair	
Systematic Name	Standard Name	Systematic Name	Standard Name
YDL184C	RPL41A	YPR030W	CSR2
YJL036W	SNX4	YJR060W	CBF1
YNL107W	YAF9	YOL081W	IRA2
YBR215W	HPC2	YDR335W	MSN5
YEL060C	PRB1	YMR230W	RPS10B
YBR131W	CCZ1	YGR105W	VMA21
YLR079W	SIC1	YML057W	CMP2
YNL032W	SIW14	YDL192W	ARF1
YGL178W	MPT5	YDL056W	MBP1
YPL253C	VIK1	YAR014C	BUD14
YLR234W	TOP3	YML103C	NUP188
YMR127C	SAS2	YFR036W	CDC26
YBR278W	DPB3	YBR036C	CSG2
YML103C	NUP188	YDR254W	CHL4
YHR205W	SCH9	YPL161C	BEM4
YOR035C	SHE4	YJR084W	CSN12
YLR371W	ROM2	YEL036C	ANP1
YBR107C	IML3	YFR030W	MET10
YDL101C	DUN1	YCR066W	RAD18
YPR018W	RLF2	YPR131C	NAT3
YOR127W	RGA1	YOR112W	CEX1
YJL136C	RPS21B	YML095C	RAD10
YOR035C	SHE4	YBR181C	RPS6B
YOR337W	TEA1	YER116C	SLX8
YMR216C	SKY1	YDL077C	VAM6
YDL184C	RPL41A	YOL111C	MDY2
YPL037C	EGD1	YPL105C	YPL105C
YER001W	MNN1	YMR089C	YTA12
YDR379W	RGA2	YGL038C	OCH1

Predictions at threshold level 0.80 continued...			
Gene1 of predicted pair		Gene2 of predicted pair	
Systematic Name	Standard Name	Systematic Name	Standard Name
YKL006W	RPL14A	YJL095W	BCK1
YJR032W	CPR7	YJL190C	RPS22A
YNR064C	YNR064C	YOR344C	TYE7
YBR111W-A	SUS1	YMR273C	ZDS1
YHR156C	LIN1	YMR230W	RPS10B
YKL135C	APL2	YJR005W	APL1
YKR048C	NAP1	YPL256C	CLN2
YMR142C	RPL13B	YJL172W	CPS1
YDR174W	HMO1	YCR086W	CSM1
YLR435W	TSR2	YKL101W	HSL1
YPL137C	GIP3	YMR214W	SCJ1
YMR026C	PEX12	YNL032W	SIW14
YMR216C	SKY1	YOL108C	INO4
YOR069W	VPS5	YKR030W	GMH1
YOR112W	CEX1	YDR153C	ENT5
YLR234W	TOP3	YBR194W	SOY1
YJL124C	LSM1	YFR030W	MET10
YLR079W	SIC1	YPL152W	RRD2
YFR010W	UBP6	YLR386W	VAC14
YCR065W	HCM1	YNL236W	SIN4
YNL116W	DMA2	YLR182W	SWI6
YKL126W	YPK1	YHR200W	RPN10
YKL139W	CTK1	YDR261C	EXG2
YLR381W	CTF3	YJR135C	MCM22
YGR241C	YAP1802	YLR206W	ENT2
YBR274W	CHK1	YCR086W	CSM1
YNL291C	MID1	YGL163C	RAD54
YGL124C	MON1	YNL076W	MKS1
YMR133W	REC114	YNL238W	KEX2

Predictions at threshold level 0.80 continued...			
Gene1 of predicted pair		Gene2 of predicted pair	
Systematic Name	Standard Name	Systematic Name	Standard Name
YNL215W	IES2	YPL152W	RRD2
YNL325C	FIG4	YPR036W	VMA13
YML103C	NUP188	YOR358W	HAP5
YDR260C	SWM1	YLR032W	RAD5
YGL212W	VAM7	YOL108C	INO4
YLR441C	RPS1A	YNL076W	MKS1
YML115C	VAN1	YGL025C	PGD1
YGL178W	MPT5	YBR231C	SWC5
YDR477W	SNF1	YDL184C	RPL41A
YJR056C	YJR056C	YBR019C	GAL10
YPL057C	SUR1	YDR207C	UME6
YGL060W	YBP2	YJR035W	RAD26
YMR198W	CIK1	YML095C	RAD10
YJL172W	CPS1	YOR066W	MSA1
YPR017C	DSS4	YPR051W	MAK3
YPL018W	CTF19	YHR114W	BZZ1
YLR386W	VAC14	YFL023W	BUD27
YNL069C	RPL16B	YOL108C	INO4
YBR057C	MUM2	YGL200C	EMP24
YDR137W	RGP1	YCL037C	SRO9
YNL032W	SIW14	YDL083C	RPS16B
YGR019W	UGA1	YDR310C	SUM1
YKR035W-A	DID2	YDL077C	VAM6
YOR001W	RRP6	YER063W	THO1
YGL133W	ITC1	YHR200W	RPN10
YFL013C	IES1	YOR001W	RRP6
YEL050C	RML2	YDR254W	CHL4
YBR057C	MUM2	YER001W	MNN1
YBL058W	SHP1	YPR046W	MCM16

Predictions at threshold level 0.80 continued...			
Gene1 of predicted pair		Gene2 of predicted pair	
Systematic Name	Standard Name	Systematic Name	Standard Name
YOR337W	TEA1	YFR010W	UBP6
YHR013C	ARD1	YLR410W	VIP1
YJR140C	HIR3	YCR031C	RPS14A
YIL159W	BNR1	YLR371W	ROM2
YPL022W	RAD1	YOL090W	MSH2
YMR216C	SKY1	YHL020C	OPI1
YHR039C-A	VMA10	YBR036C	CSG2
YLR090W	XDJ1	YNL076W	MKS1
YLR292C	SEC72	YPL253C	VIK1
YJR063W	RPA12	YLR357W	RSC2
YER144C	UBP5	YER139C	YER139C
YPL152W	RRD2	YJL006C	CTK2
YDL020C	RPN4	YGR135W	PRE9
YMR004W	MVP1	YJR033C	RAV1
YBL058W	SHP1	YEL060C	PRB1
YCR086W	CSM1	YMR038C	CCS1
YMR026C	PEX12	YAL055W	PEX22
YLR192C	HCR1	YLR180W	SAM1
YML071C	COG8	YLR180W	SAM1
YJL154C	VPS35	YLR371W	ROM2
YLR090W	XDJ1	YOR043W	WHI2
YDL083C	RPS16B	YOR066W	MSA1
YBR131W	CCZ1	YMR054W	STV1
YLR337C	VRP1	YGR019W	UGA1
YER110C	KAP123	YPL018W	CTF19
YJR135C	MCM22	YJL183W	MNN11
YML115C	VAN1	YPR119W	CLB2
YGR054W	YGR054W	YJL095W	BCK1
YMR198W	CIK1	YOR080W	DIA2

Predictions at threshold level 0.80 continued...			
Gene1 of predicted pair		Gene2 of predicted pair	
Systematic Name	Standard Name	Systematic Name	Standard Name
YML109W	ZDS2	YHR061C	GIC1
YBR030W	YBR030W	YDR261C	EXG2
YDR004W	RAD57	YFL003C	MSH4
YOR069W	VPS5	YER120W	SCS2
YMR004W	MVP1	YGL054C	ERV14
YPR084W	YPR084W	YPL234C	TFP3
YJR135C	MCM22	YIL103W	DPH1
YPR141C	KAR3	YHR056C	RSC30
YOR005C	DNL4	YGL090W	LIF1
YDR146C	SWI5	YER096W	SHC1
YLR102C	APC9	YGL163C	RAD54
YOR127W	RGA1	YMR199W	CLN1
YPL057C	SUR1	YML071C	COG8
YJL154C	VPS35	YPR032W	SRO7
YDR507C	GIN4	YDR264C	AKR1
YOL090W	MSH2	YML028W	TSA1
YBR108W	YBR108W	YJL095W	BCK1
YEL053C	MAK10	YCR086W	CSM1
YGL212W	VAM7	YMR152W	YIM1
YJL172W	CPS1	YGL003C	CDH1
YAL021C	CCR4	YHR056C	RSC30
YLR386W	VAC14	YDR137W	RGP1
YDR335W	MSN5	YKL160W	ELF1
YJL030W	MAD2	YGL190C	CDC55
YBL103C	RTG3	YDL223C	HBT1
YOL121C	RPS19A	YGL124C	MON1
YJL124C	LSM1	YLR362W	STE11
YML103C	NUP188	YGR188C	BUB1
YNL248C	RPA49	YFR036W	CDC26

Predictions at threshold level 0.80 continued...			
Gene1 of predicted pair		Gene2 of predicted pair	
Systematic Name	Standard Name	Systematic Name	Standard Name
YDR378C	LSM6	YGL115W	SNF4
YCL063W	VAC17	YEL013W	VAC8
YKR009C	FOX2	YOL147C	PEX11
YDR293C	SSD1	YIL076W	SEC28
YLR292C	SEC72	YDL155W	CLB3
YNL307C	MCK1	YMR230W	RPS10B
YKR020W	VPS51	YGL115W	SNF4
YAL024C	LTE1	YDR318W	MCM21
YMR004W	MVP1	YHR012W	VPS29
YJR056C	YJR056C	YDR207C	UME6
YLR018C	POM34	YDR358W	GGA1
YLR292C	SEC72	YPR051W	MAK3
YER116C	SLX8	YOL030W	GAS5
YHR171W	ATG7	YLL042C	ATG10
YPL195W	APL5	YOL108C	INO4
YMR153W	NUP53	YBL079W	NUP170
YOR127W	RGA1	YBR171W	SEC66
YLR399C	BDF1	YFR036W	CDC26
YIL053W	RHR2	YLR371W	ROM2
YPL057C	SUR1	YDR076W	RAD55
YKL117W	SBA1	YNL199C	GCR2
YJL029C	VPS53	YAL030W	SNC1
YGL087C	MMS2	YKR001C	VPS1
YGR135W	PRE9	YOL054W	PSH1
YMR313C	TGL3	YKL140W	TGL1
YDL020C	RPN4	YDR225W	HTA1
YGR020C	VMA7	YJR040W	GEF1
YNR072W	HXT17	YCR053W	THR4
YNL307C	MCK1	YPL037C	EGD1

Predictions at threshold level 0.80 continued...			
Gene1 of predicted pair		Gene2 of predicted pair	
Systematic Name	Standard Name	Systematic Name	Standard Name
YJL172W	CPS1	YLR094C	GIS3
YJL154C	VPS35	YDR183W	PLP1
YHL031C	GOS1	YPL120W	VPS30
YKR061W	KTR2	YFR036W	CDC26
YIR009W	MSL1	YPR119W	CLB2
YHR012W	VPS29	YLR309C	IMH1
YLR119W	SRN2	YHR060W	VMA22
YLR452C	SST2	YDR207C	UME6
YHR134W	WSS1	YDL155W	CLB3
YGL226C-A	OST5	YML019W	OST6
YOR023C	AHC1	YGL237C	HAP2
YPL246C	RBD2	YJL004C	SYS1
YBR131W	CCZ1	YLR320W	MMS22
YDR443C	SSN2	YDR146C	SWI5
YGL147C	RPL9A	YPR046W	MCM16
YBR200W	BEM1	YGL003C	CDH1
YGR072W	UPF3	YEL043W	YEL043W
YOR069W	VPS5	YHR012W	VPS29
YDL116W	NUP84	YGL100W	SEH1
YEL065W	SIT1	YHR039C-A	VMA10
YNL288W	CAF40	YBL021C	HAP3
YFL023W	BUD27	YJR056C	YJR056C
YKR020W	VPS51	YMR231W	PEP5
YGR108W	CLB1	YNL166C	BNI5
YLR006C	SSK1	YOR127W	RGA1
YBR200W	BEM1	YDR245W	MNN10
YMR016C	SOK2	YLR371W	ROM2
YLR182W	SWI6	YLR350W	ORM2
YLR192C	HCR1	YGL003C	CDH1

Predictions at threshold level 0.80 continued...			
Gene1 of predicted pair		Gene2 of predicted pair	
Systematic Name	Standard Name	Systematic Name	Standard Name
YKR099W	BAS1	YJR032W	CPR7
YLR386W	VAC14	YGL115W	SNF4
YLR234W	TOP3	YGR270W	YTA7
YNL106C	INP52	YER096W	SHC1
YLR234W	TOP3	YOR132W	VPS17
YML103C	NUP188	YMR153W	NUP53
YHR200W	RPN10	YDL192W	ARF1
YGL174W	BUD13	YKL160W	ELF1
YBR057C	MUM2	YGR188C	BUB1
YDL059C	RAD59	YBR215W	HPC2
YGL060W	YBP2	YLR079W	SIC1
YBL103C	RTG3	YDR358W	GGA1
YGL134W	PCL10	YPL219W	PCL8
YGL124C	MON1	YGL253W	HXK2
YLR083C	EMP70	YNL127W	FAR11
YHR073W	OSH3	YMR125W	STO1
YDR383C	NKP1	YDR439W	LRS4
YML103C	NUP188	YPL178W	CBC2
YML071C	COG8	YDR153C	ENT5
YGR249W	MGA1	YBR171W	SEC66
YPL042C	SSN3	YDL226C	GCS1
YCR081W	SRB8	YJL154C	VPS35
YIL148W	RPL40A	YCR024C-A	PMP1
YHL031C	GOS1	YEL042W	GDA1
YHR052W	CIC1	YKL009W	MRT4
YNL041C	COG6	YOR308C	SNU66
YNL044W	YIP3	YNL041C	COG6
YMR224C	MRE11	YBR280C	SAF1
YDR004W	RAD57	YPL022W	RAD1

Predictions at threshold level 0.80 continued...			
Gene1 of predicted pair		Gene2 of predicted pair	
Systematic Name	Standard Name	Systematic Name	Standard Name
YBR108W	YBR108W	YHR111W	UBA4
YOR132W	VPS17	YJR040W	GEF1
YGL115W	SNF4	YEL060C	PRB1
YJL172W	CPS1	YMR152W	YIM1
YMR284W	YKU70	YNL016W	PUB1
YDR135C	YCF1	YGL181W	GTS1
YDR443C	SSN2	YHR039C-A	VMA10
YCR088W	ABP1	YJL187C	SWE1
YOR156C	NFI1	YNL078W	NIS1
YER115C	SPR6	YMR125W	STO1
YPL024W	RMI1	YOR368W	RAD17
YGR104C	SRB5	YDR289C	RTT103
YJR056C	YJR056C	YPR036W	VMA13
YIL034C	CAP2	YLR234W	TOP3
YDR156W	RPA14	YMR205C	PFK2
YLR090W	XDJ1	YKR099W	BAS1
YMR179W	SPT21	YJR060W	CBF1
YBR200W	BEM1	YHR081W	LRP1
YDR378C	LSM6	YCR077C	PAT1
YOR270C	VPH1	YLR094C	GIS3
YMR224C	MRE11	YNL106C	INP52
YPR120C	CLB5	YNL107W	YAF9
YPR030W	CSR2	YER162C	RAD4
YOR043W	WHI2	YBR195C	MSI1
YML115C	VAN1	YML028W	TSA1
YER116C	SLX8	YMR311C	GLC8
YDR293C	SSD1	YBL103C	RTG3
YMR199W	CLN1	YHL011C	PRS3
YLR386W	VAC14	YDL192W	ARF1

Predictions at threshold level 0.80 continued...			
Gene1 of predicted pair		Gene2 of predicted pair	
Systematic Name	Standard Name	Systematic Name	Standard Name
YDR156W	RPA14	YGR135W	PRE9
YPL115C	BEM3	YPR051W	MAK3
YDR440W	DOT1	YHR115C	DMA1
YNL199C	GCR2	YER122C	GLO3
YDR146C	SWI5	YOR290C	SNF2
YKR020W	VPS51	YML001W	YPT7
YHR060W	VMA22	YOL111C	MDY2
YDR137W	RGP1	YEL027W	CUP5
YKL007W	CAP1	YKL213C	DOA1
YNL051W	COG5	YKL119C	VPH2
YNR052C	POP2	YNL107W	YAF9
YGL078C	DBP3	YBR200W	BEM1
YDL020C	RPN4	YKL139W	CTK1
YIR025W	MND2	YGL003C	CDH1
YJL176C	SWI3	YDR378C	LSM6
YCL001W	RER1	YNL107W	YAF9
YDL116W	NUP84	YKL048C	ELM1
YBR290W	BSD2	YHR114W	BZZ1
YGR135W	PRE9	YBL101C	ECM21
YCR031C	RPS14A	YGL019W	CKB1
YLR360W	VPS38	YDR318W	MCM21
YPL042C	SSN3	YNL297C	MON2
YER068W	MOT2	YLR357W	RSC2
YLR192C	HCR1	YLR371W	ROM2
YMR004W	MVP1	YKR030W	GMH1
YFR036W	CDC26	YKL101W	HSL1
YDL184C	RPL41A	YJR032W	CPR7
YGR003W	CUL3	YLR306W	UBC12
YGR135W	PRE9	YKL101W	HSL1

Predictions at threshold level 0.80 continued...			
**Gene1 of predicted pair**		**Gene2 of predicted pair**	
**Systematic Name**	**Standard Name**	**Systematic Name**	**Standard Name**
YJL053W	PEP8	YJR060W	CBF1
YLR360W	VPS38	YMR311C	GLC8
YBR126C	TPS1	YKL139W	CTK1
YBR200W	BEM1	YLR320W	MMS22
YDL116W	NUP84	YBR200W	BEM1
YPL137C	GIP3	YHR081W	LRP1
YAL024C	LTE1	YOR035C	SHE4
YNL087W	TCB2	YHR060W	VMA22
YNL166C	BNI5	YPR046W	MCM16
YGL003C	CDH1	YMR311C	GLC8
YPR154W	PIN3	YHR016C	YSC84
YHR129C	ARP1	YHR098C	SFB3
YLR119W	SRN2	YMR026C	PEX12
YLL007C	YLL007C	YOR023C	AHC1
YKL139W	CTK1	YOL051W	GAL11
YBR009C	HHF1	YER068W	MOT2
YOR112W	CEX1	YDR310C	SUM1
YCR020C-A	MAK31	YJL154C	VPS35
YDR318W	MCM21	YPR119W	CLB2
YDR130C	FIN1	YCR086W	CSM1
YLR337C	VRP1	YOR213C	SAS5
YJL154C	VPS35	YMR004W	MVP1
YDR183W	PLP1	YNL225C	CNM67
YMR142C	RPL13B	YOR035C	SHE4
YML038C	YMD8	YPL120W	VPS30
YNL264C	PDR17	YPL180W	TCO89
YLR362W	STE11	YJL128C	PBS2
YOR035C	SHE4	YAL002W	VPS8
YLR386W	VAC14	YIR005W	IST3

Predictions at threshold level 0.80 continued...			
Gene1 of predicted pair		Gene2 of predicted pair	
Systematic Name	Standard Name	Systematic Name	Standard Name
YPL161C	BEM4	YMR311C	GLC8
YER016W	BIM1	YHR056C	RSC30
YDR477W	SNF1	YLR180W	SAM1
YDR129C	SAC6	YOR312C	RPL20B
YBR131W	CCZ1	YNL044W	YIP3
YMR224C	MRE11	YDR289C	RTT103
YDR484W	VPS52	YPL037C	EGD1
YOR069W	VPS5	YPL234C	TFP3
YGL115W	SNF4	YDL155W	CLB3
YLR370C	ARC18	YBR036C	CSG2
YDL116W	NUP84	YCR086W	CSM1
YLR386W	VAC14	YPL137C	GIP3
YER110C	KAP123	YGR270W	YTA7
YGL066W	SGF73	YKL101W	HSL1
YBR200W	BEM1	YGR270W	YTA7
YBR200W	BEM1	YEL056W	HAT2
YGR249W	MGA1	YDR207C	UME6
YHR060W	VMA22	YJL004C	SYS1
YNL094W	APP1	YKL129C	MYO3
YOR001W	RRP6	YKL160W	ELF1
YGR217W	CCH1	YBR082C	UBC4
YOR290C	SNF2	YJL140W	RPB4
YEL065W	SIT1	YDR443C	SSN2
YEL051W	VMA8	YDL077C	VAM6
YBR200W	BEM1	YDL006W	PTC1
YBR200W	BEM1	YHR013C	ARD1
YLR234W	TOP3	YCR077C	PAT1
YJR060W	CBF1	YGR105W	VMA21
YDL020C	RPN4	YBR107C	IML3

Predictions at threshold level 0.80 continued...			
Gene1 of predicted pair		Gene2 of predicted pair	
Systematic Name	Standard Name	Systematic Name	Standard Name
YDR137W	RGP1	YBL025W	RRN10
YER116C	SLX8	YDL184C	RPL41A
YDL020C	RPN4	YMR127C	SAS2
YLR083C	EMP70	YOR109W	INP53
YPL161C	BEM4	YOR066W	MSA1
YDL013W	SLX5	YDR076W	RAD55
YNL288W	CAF40	YLR447C	VMA6
YKL139W	CTK1	YGL115W	SNF4
YOL051W	GAL11	YHR200W	RPN10
YMR026C	PEX12	YNL325C	FIG4
YBL102W	SFT2	YPL105C	YPL105C
YNL032W	SIW14	YER122C	GLO3
YGL178W	MPT5	YGR229C	SMI1
YDL083C	RPS16B	YMR152W	YIM1
YBR108W	YBR108W	YPL253C	VIK1
YPL240C	HSP82	YDR545W	YRF1-1
YDL116W	NUP84	YNL248C	RPA49
YLR337C	VRP1	YHR134W	WSS1
YDL116W	NUP84	YPL253C	VIK1
YLR216C	CPR6	YOR195W	SLK19
YPR018W	RLF2	YLR150W	STM1
YAL020C	ATS1	YKR020W	VPS51
YLR102C	APC9	YBL047C	EDE1
YDR202C	RAV2	YGL115W	SNF4
YPR036W	VMA13	YGR105W	VMA21
YPL022W	RAD1	YLR320W	MMS22
YNL291C	MID1	YOR127W	RGA1
YFR030W	MET10	YLR182W	SWI6
YIL159W	BNR1	YBR200W	BEM1

Predictions at threshold level 0.80 continued...			
Gene1 of predicted pair		Gene2 of predicted pair	
Systematic Name	Standard Name	Systematic Name	Standard Name
YDR320C	SWA2	YKR020W	VPS51
YGR249W	MGA1	YBR280C	SAF1
YMR127C	SAS2	YAL002W	VPS8
YMR224C	MRE11	YMR152W	YIM1
YBR030W	YBR030W	YFL023W	BUD27
YPL253C	VIK1	YOL054W	PSH1
YDR253C	MET32	YPR120C	CLB5
YBR019C	GAL10	YBR036C	CSG2
YCR097W	HMRA1	YPL240C	HSP82
YLR452C	SST2	YCR053W	THR4
YHL025W	SNF6	YOR038C	HIR2
YDR383C	NKP1	YPL152W	RRD2
YGL212W	VAM7	YKL041W	VPS24
YPR036W	VMA13	YLR452C	SST2
YDR507C	GIN4	YBL047C	EDE1
YDR260C	SWM1	YDR318W	MCM21
YLR410W	VIP1	YDR200C	VPS64
YBL058W	SHP1	YER144C	UBP5
YML062C	MFT1	YCL011C	GBP2
YCL010C	SGF29	YPL248C	GAL4
YDR137W	RGP1	YML071C	COG8
YPR089W	YPR089W	YKR001C	VPS1
YOR156C	NFI1	YOL145C	CTR9
YGR105W	VMA21	YDR310C	SUM1
YBR019C	GAL10	YCR053W	THR4
YMR039C	SUB1	YJR060W	CBF1
YLR441C	RPS1A	YGL025C	PGD1
YBR173C	UMP1	YJR084W	CSN12
YHR178W	STB5	YPL001W	HAT1

Predictions at threshold level 0.80 continued...			
Gene1 of predicted pair		Gene2 of predicted pair	
Systematic Name	Standard Name	Systematic Name	Standard Name
YDR103W	STE5	YJL157C	FAR1
YLR386W	VAC14	YOR156C	NFI1
YKL048C	ELM1	YOL054W	PSH1
YBR171W	SEC66	YDR310C	SUM1
YLR447C	VMA6	YGR270W	YTA7
YPL234C	TFP3	YJL095W	BCK1
YPR052C	NHP6A	YBR240C	THI2
YGL054C	ERV14	YER122C	GLO3
YMR153W	NUP53	YGL016W	KAP122
YGL175C	SAE2	YOR368W	RAD17
YLR090W	XDJ1	YOR290C	SNF2
YDR260C	SWM1	YML028W	TSA1
YCR086W	CSM1	YLR254C	NDL1
YIL159W	BNR1	YDR309C	GIC2
YPR120C	CLB5	YHR158C	KEL1
YOR290C	SNF2	YMR214W	SCJ1
YLR288C	MEC3	YNL291C	MID1
YGR249W	MGA1	YPL137C	GIP3
YNL297C	MON2	YEL043W	YEL043W
YHL031C	GOS1	YDR440W	DOT1
YBR215W	HPC2	YHR167W	THP2
YJL124C	LSM1	YHR039C-A	VMA10
YDL223C	HBT1	YLR093C	NYV1
YOR124C	UBP2	YNL107W	YAF9
YIL053W	RHR2	YPL057C	SUR1
YBL047C	EDE1	YHR115C	DMA1
YCR031C	RPS14A	YDR310C	SUM1
YFR031C-A	RPL2A	YBR217W	ATG12
YLR381W	CTF3	YLR447C	VMA6

Predictions at threshold level 0.80 continued...			
Gene1 of predicted pair		Gene2 of predicted pair	
Systematic Name	Standard Name	Systematic Name	Standard Name
YER116C	SLX8	YLR320W	MMS22
YDR254W	CHL4	YOL054W	PSH1
YKR020W	VPS51	YKR030W	GMH1
YDR254W	CHL4	YPL106C	SSE1
YDR363W-A	SEM1	YJR060W	CBF1
YJL053W	PEP8	YPL234C	TFP3
YLR337C	VRP1	YMR116C	ASC1
YJL154C	VPS35	YPL234C	TFP3
YPL106C	SSE1	YGL003C	CDH1
YLR399C	BDF1	YJL006C	CTK2
YBR215W	HPC2	YJR090C	GRR1
YJR032W	CPR7	YLR180W	SAM1
YHR178W	STB5	YDR310C	SUM1
YER110C	KAP123	YOL067C	RTG1
YPL024W	RMI1	YLR398C	SKI2
YHR114W	BZZ1	YKR030W	GMH1
YBL051C	PIN4	YGL237C	HAP2
YGR104C	SRB5	YCL037C	SRO9
YOR327C	SNC2	YJR033C	RAV1
YBR126C	TPS1	YPL161C	BEM4
YDR146C	SWI5	YGR252W	GCN5
YGL115W	SNF4	YNL230C	ELA1
YML115C	VAN1	YOR327C	SNC2
YDR146C	SWI5	YMR311C	GLC8
YOR195W	SLK19	YLR182W	SWI6
YDR137W	RGP1	YJR135C	MCM22
YFL023W	BUD27	YNL238W	KEX2
YLR360W	VPS38	YDL006W	PTC1
YBR107C	IML3	YGL003C	CDH1

Predictions at threshold level 0.80 continued...			
Gene1 of predicted pair		Gene2 of predicted pair	
Systematic Name	Standard Name	Systematic Name	Standard Name
YIL008W	URM1	YDL047W	SIT4
YPR051W	MAK3	YNL238W	KEX2
YDR071C	PAA1	YOR368W	RAD17
YLR381W	CTF3	YLR315W	NKP2
YBL021C	HAP3	YDR144C	MKC7
YDR156W	RPA14	YLR399C	BDF1
YNL325C	FIG4	YMR133W	REC114
YDR183W	PLP1	YGL124C	MON1
YDL042C	SIR2	YDR110W	FOB1
YDR293C	SSD1	YOL018C	TLG2
YLR441C	RPS1A	YBR019C	GAL10
YML067C	ERV41	YLR093C	NYV1
YOR337W	TEA1	YKL048C	ELM1
YKR001C	VPS1	YNL041C	COG6
YCR020C-A	MAK31	YHR039C-A	VMA10
YCL037C	SRO9	YNL107W	YAF9
YMR116C	ASC1	YCR077C	PAT1
YFR010W	UBP6	YLR234W	TOP3
YBR215W	HPC2	YGL151W	NUT1
YER116C	SLX8	YBR082C	UBC4
YKL139W	CTK1	YOR038C	HIR2
YDR080W	VPS41	YOR109W	INP53
YGL087C	MMS2	YGL237C	HAP2
YHR134W	WSS1	YLR135W	SLX4
YPR017C	DSS4	YER162C	RAD4
YAL024C	LTE1	YIL159W	BNR1
YHR039C-A	VMA10	YAL002W	VPS8
YBR057C	MUM2	YKR020W	VPS51
YJL176C	SWI3	YPL057C	SUR1

Predictions at threshold level 0.80 continued...			
Gene1 of predicted pair		Gene2 of predicted pair	
Systematic Name	Standard Name	Systematic Name	Standard Name
YNR052C	POP2	YLR386W	VAC14
YOR106W	VAM3	YGR105W	VMA21
YPL213W	LEA1	YIR005W	IST3
YPL120W	VPS30	YAR014C	BUD14
YNL307C	MCK1	YAR014C	BUD14
YML115C	VAN1	YMR214W	SCJ1
YPL137C	GIP3	YGL003C	CDH1
YLR398C	SKI2	YKL041W	VPS24
YPL161C	BEM4	YJL183W	MNN11
YOR106W	VAM3	YGL212W	VAM7
YPL203W	TPK2	YER068W	MOT2
YDR439W	LRS4	YIR005W	IST3
YAL024C	LTE1	YOR308C	SNU66
YDR293C	SSD1	YHR114W	BZZ1
YLR119W	SRN2	YHR081W	LRP1
YGL033W	HOP2	YPL161C	BEM4
YPR046W	MCM16	YDR310C	SUM1
YGR104C	SRB5	YIR005W	IST3
YDL020C	RPN4	YDR289C	RTT103
YFR036W	CDC26	YML112W	CTK3
YLR083C	EMP70	YEL051W	VMA8
YJL036W	SNX4	YPL037C	EGD1
YMR095C	SNO1	YKL139W	CTK1
YDL116W	NUP84	YNL218W	MGS1
YJL172W	CPS1	YJL183W	MNN11
YDL223C	HBT1	YPL050C	MNN9
YBR200W	BEM1	YDR378C	LSM6
YDL020C	RPN4	YFL023W	BUD27
YER118C	SHO1	YLR398C	SKI2

Predictions at threshold level 0.80 continued...			
Gene1 of predicted pair		Gene2 of predicted pair	
Systematic Name	Standard Name	Systematic Name	Standard Name
YOR275C	RIM20	YPL161C	BEM4
YNL032W	SIW14	YBL103C	RTG3
YMR133W	REC114	YPR046W	MCM16
YKL116C	PRR1	YIL076W	SEC28
YIL034C	CAP2	YEL053C	MAK10
YDR225W	HTA1	YJR060W	CBF1
YBR200W	BEM1	YKL119C	VPH2
YKR035W-A	DID2	YML001W	YPT7
YDR200C	VPS64	YPR119W	CLB2
YLR148W	PEP3	YBR127C	VMA2
YNL325C	FIG4	YLR371W	ROM2
YML103C	NUP188	YGR059W	SPR3
YMR224C	MRE11	YLR094C	GIS3
YOR106W	VAM3	YOR008C	SLG1
YMR142C	RPL13B	YBL103C	RTG3
YBR217W	ATG12	YHR060W	VMA22
YHL020C	OPI1	YHR039C-A	VMA10
YML016C	PPZ1	YBR171W	SEC66
YGL135W	RPL1B	YHR013C	ARD1
YGL087C	MMS2	YOR127W	RGA1
YLR043C	TRX1	YKR001C	VPS1
YLR254C	NDL1	YNL225C	CNM67
YDR004W	RAD57	YGL151W	NUT1
YDR507C	GIN4	YGL031C	RPL24A
YHL011C	PRS3	YHR158C	KEL1
YPR032W	SRO7	YKR030W	GMH1
YPR046W	MCM16	YLR233C	EST1
YDR443C	SSN2	YDR310C	SUM1
YGR249W	MGA1	YLR371W	ROM2

Predictions at threshold level 0.80 continued...			
Gene1 of predicted pair		Gene2 of predicted pair	
Systematic Name	Standard Name	Systematic Name	Standard Name
YER044C	ERG28	YHR200W	RPN10
YIL159W	BNR1	YHR111W	UBA4
YOR043W	WHI2	YPR030W	CSR2
YML012W	ERV25	YNL049C	SFB2
YLR337C	VRP1	YBR171W	SEC66
YNL106C	INP52	YDR318W	MCM21
YFL023W	BUD27	YOL111C	MDY2
YOR337W	TEA1	YGL078C	DBP3
YPR084W	YPR084W	YGR105W	VMA21
YDR448W	ADA2	YNR052C	POP2
YLR083C	EMP70	YHR039C-A	VMA10
YLR292C	SEC72	YDR155C	CPR1
YGL153W	PEX14	YKR020W	VPS51
YML115C	VAN1	YHR013C	ARD1
YCR020C-A	MAK31	YJR060W	CBF1
YBR280C	SAF1	YLR180W	SAM1
YDL239C	ADY3	YOR177C	MPC54
YHR171W	ATG7	YPL149W	ATG5
YDL002C	NHP10	YER139C	YER139C
YBL051C	PIN4	YEL056W	HAT2
YLR079W	SIC1	YKL117W	SBA1
YIL159W	BNR1	YKL139W	CTK1
YDR103W	STE5	YBL016W	FUS3
YHR039C-A	VMA10	YEL056W	HAT2
YEL065W	SIT1	YGL124C	MON1
YMR153W	NUP53	YDR358W	GGA1
YGL163C	RAD54	YMR231W	PEP5
YGL250W	RMR1	YJR032W	CPR7
YFR010W	UBP6	YOL111C	MDY2

Predictions at threshold level 0.80 continued...			
Gene1 of predicted pair		Gene2 of predicted pair	
Systematic Name	Standard Name	Systematic Name	Standard Name
YEL065W	SIT1	YKR035W-A	DID2
YOL054W	PSH1	YNL107W	YAF9
YMR095C	SNO1	YLR386W	VAC14
YBR200W	BEM1	YPL161C	BEM4
YLR138W	NHA1	YML132W	COS3
YMR055C	BUB2	YNL079C	TPM1
YPR029C	APL4	YPL259C	APM1
YDR144C	MKC7	YJR090C	GRR1
YGL147C	RPL9A	YKR099W	BAS1
YPR084W	YPR084W	YER122C	GLO3
YLR083C	EMP70	YLR360W	VPS38
YBR107C	IML3	YOR141C	ARP8
YOR043W	WHI2	YMR230W	RPS10B
YGL250W	RMR1	YJR135C	MCM22
YML001W	YPT7	YDR153C	ENT5
YIL159W	BNR1	YOR035C	SHE4
YPL256C	CLN2	YLR182W	SWI6
YJR140C	HIR3	YOR080W	DIA2
YBL103C	RTG3	YBL025W	RRN10
YER116C	SLX8	YER139C	YER139C
YPL161C	BEM4	YCR053W	THR4
YML109W	ZDS2	YKR095W	MLP1
YML115C	VAN1	YJL006C	CTK2
YER122C	GLO3	YPR032W	SRO7
YOL111C	MDY2	YLR398C	SKI2
YLL039C	UBI4	YDR378C	LSM6
YAL024C	LTE1	YPL018W	CTF19
YBR082C	UBC4	YJR035W	RAD26
YFL023W	BUD27	YJL095W	BCK1

Predictions at threshold level 0.80 continued...			
**Gene1 of predicted pair**		**Gene2 of predicted pair**	
**Systematic Name**	**Standard Name**	**Systematic Name**	**Standard Name**
YGR249W	MGA1	YML115C	VAN1
YMR004W	MVP1	YMR054W	STV1
YDR076W	RAD55	YDR310C	SUM1
YPR084W	YPR084W	YIR005W	IST3
YDR129C	SAC6	YGR133W	PEX4
YJL124C	LSM1	YPR119W	CLB2
YHR039C-A	VMA10	YGL148W	ARO2
YNL206C	RTT106	YNL229C	URE2
YAR014C	BUD14	YNL225C	CNM67
YLR371W	ROM2	YMR152W	YIM1
YNL069C	RPL16B	YDR202C	RAV2
YBL106C	SRO77	YNL090W	RHO2
YBL008W	HIR1	YNL206C	RTT106
YDR382W	RPP2B	YNL145W	MFA2
YLR320W	MMS22	YBL101C	ECM21
YGL147C	RPL9A	YLR371W	ROM2
YNL206C	RTT106	YML102W	CAC2
YLR360W	VPS38	YDL226C	GCS1
YKL126W	YPK1	YDR261C	EXG2
YKL139W	CTK1	YBL101C	ECM21
YMR199W	CLN1	YHR200W	RPN10
YPL129W	TAF14	YOR189W	IES4
YLR410W	VIP1	YMR038C	CCS1
YPL115C	BEM3	YPR046W	MCM16
YLR093C	NYV1	YDL077C	VAM6
YEL053C	MAK10	YGL054C	ERV14
YDR254W	CHL4	YJL006C	CTK2
YOR375C	GDH1	YJL095W	BCK1
YGR249W	MGA1	YDR358W	GGA1

Predictions at threshold level 0.80 continued...			
Gene1 of predicted pair		Gene2 of predicted pair	
Systematic Name	Standard Name	Systematic Name	Standard Name
YLR102C	APC9	YGL003C	CDH1
YOR069W	VPS5	YKL119C	VPH2
YDL127W	PCL2	YGR233C	PHO81
YLR441C	RPS1A	YPL057C	SUR1
YDR335W	MSN5	YJL157C	FAR1
YLR192C	HCR1	YJL172W	CPS1
YLR079W	SIC1	YMR198W	CIK1
YGL135W	RPL1B	YBR082C	UBC4
YKR099W	BAS1	YOR043W	WHI2
YPL137C	GIP3	YBR036C	CSG2
YMR224C	MRE11	YLR394W	CST9
YDR378C	LSM6	YOR320C	GNT1
YBR274W	CHK1	YBR215W	HPC2
YJR099W	YUH1	YOR124C	UBP2
YFL023W	BUD27	YKL126W	YPK1
YLR083C	EMP70	YGL054C	ERV14
YDL006W	PTC1	YAL002W	VPS8
YFR031C-A	RPL2A	YER040W	GLN3
YNL106C	INP52	YFR036W	CDC26
YNL201C	PSY2	YOR141C	ARP8
YPL022W	RAD1	YJR060W	CBF1
YNR052C	POP2	YJR035W	RAD26
YLL007C	YLL007C	YAL002W	VPS8
YGL033W	HOP2	YNL325C	FIG4
YDL020C	RPN4	YGL175C	SAE2
YBR107C	IML3	YGR188C	BUB1
YGR104C	SRB5	YCR077C	PAT1
YBR171W	SEC66	YGL151W	NUT1
YLR320W	MMS22	YDR310C	SUM1

Predictions at threshold level 0.80 continued...			
Gene1 of predicted pair		Gene2 of predicted pair	
Systematic Name	Standard Name	Systematic Name	Standard Name
YMR004W	MVP1	YNL044W	YIP3
YKR048C	NAP1	YOR327C	SNC2
YPR018W	RLF2	YDR207C	UME6
YLR292C	SEC72	YBR107C	IML3
YCR081W	SRB8	YBR260C	RGD1
YHR200W	RPN10	YLR370C	ARC18
YDR443C	SSN2	YGR249W	MGA1
YGL054C	ERV14	YPL120W	VPS30
YHR111W	UBA4	YJR060W	CBF1
YKL126W	YPK1	YDL223C	HBT1
YHR152W	SPO12	YJL095W	BCK1
YLR337C	VRP1	YMR127C	SAS2
YLR288C	MEC3	YPR120C	CLB5
YOR308C	SNU66	YOR124C	UBP2
YIL053W	RHR2	YER122C	GLO3
YDR108W	GSG1	YCL001W	RER1
YIL053W	RHR2	YJR032W	CPR7
YBR216C	YBP1	YML007W	YAP1
YMR017W	SPO20	YNR049C	MSO1
YOR290C	SNF2	YHR108W	GGA2
YDR320C	SWA2	YDR137W	RGP1
YGL223C	COG1	YNL041C	COG6
YMR095C	SNO1	YLR180W	SAM1
YOR195W	SLK19	YKL114C	APN1
YLR018C	POM34	YDL059C	RAD59
YKL116C	PRR1	YER040W	GLN3
YOR106W	VAM3	YLR309C	IMH1
YFR010W	UBP6	YIL076W	SEC28
YLR399C	BDF1	YPR072W	NOT5

Predictions at threshold level 0.80 continued...			
Gene1 of predicted pair		Gene2 of predicted pair	
Systematic Name	Standard Name	Systematic Name	Standard Name
YMR199W	CLN1	YKR048C	NAP1
YKR061W	KTR2	YIR005W	IST3
YNR072W	HXT17	YBR019C	GAL10
YNL307C	MCK1	YGR188C	BUB1
YDR080W	VPS41	YCL001W	RER1
YHR098C	SFB3	YGR229C	SMI1
YOR076C	SKI7	YJR140C	HIR3
YDR443C	SSN2	YER122C	GLO3
YDR289C	RTT103	YHR114W	BZZ1
YKL006W	RPL14A	YKL041W	VPS24
YPR051W	MAK3	YDR318W	MCM21
YJR056C	YJR056C	YOR066W	MSA1
YFR040W	SAP155	YJL095W	BCK1
YPL115C	BEM3	YMR273C	ZDS1
YNL297C	MON2	YLR032W	RAD5
YLR090W	XDJ1	YDR477W	SNF1
YJR135C	MCM22	YJL190C	RPS22A
YHL031C	GOS1	YER162C	RAD4
YGL212W	VAM7	YLR309C	IMH1
YGL087C	MMS2	YEL056W	HAT2
YCR020C-A	MAK31	YCR066W	RAD18
YDL184C	RPL41A	YOR290C	SNF2
YNL307C	MCK1	YPL018W	CTF19
YOR076C	SKI7	YHR167W	THP2
YPL137C	GIP3	YMR230W	RPS10B
YOR127W	RGA1	YBL021C	HAP3
YBR108W	YBR108W	YKR099W	BAS1
YDR310C	SUM1	YMR230W	RPS10B
YBL078C	ATG8	YHL031C	GOS1

Predictions at threshold level 0.80 continued...			
Gene1 of predicted pair		Gene2 of predicted pair	
Systematic Name	Standard Name	Systematic Name	Standard Name
YKR020W	VPS51	YJL004C	SYS1
YLR337C	VRP1	YFR036W	CDC26
YPR066W	UBA3	YDR139C	RUB1
YHL031C	GOS1	YJL036W	SNX4
YPR131C	NAT3	YHR010W	RPL27A
YMR054W	STV1	YNL051W	COG5
YGL200C	EMP24	YKR014C	YPT52
YEL013W	VAC8	YAL002W	VPS8
YOR035C	SHE4	YJL183W	MNN11
YOR069W	VPS5	YJR040W	GEF1
YNL325C	FIG4	YBR280C	SAF1
YMR199W	CLN1	YGL190C	CDC55
YER116C	SLX8	YMR153W	NUP53
YPL249C-A	RPL36B	YCR009C	RVS161
YBL008W	HIR1	YNL236W	SIN4
YOR043W	WHI2	YMR152W	YIM1
YDL185W	TFP1	YNL107W	YAF9
YMR026C	PEX12	YDR142C	PEX7
YPL037C	EGD1	YFL033C	RIM15
YPR046W	MCM16	YJR035W	RAD26
YML058W	SML1	YGL094C	PAN2
YNL215W	IES2	YGR270W	YTA7
YPL253C	VIK1	YDR155C	CPR1
YDR358W	GGA1	YLR398C	SKI2
YLR320W	MMS22	YGR188C	BUB1
YBR171W	SEC66	YDL083C	RPS16B
YPL022W	RAD1	YOR368W	RAD17
YKL116C	PRR1	YBR171W	SEC66
YMR172W	HOT1	YGR054W	YGR054W

Predictions at threshold level 0.80 continued...			
Gene1 of predicted pair		Gene2 of predicted pair	
Systematic Name	Standard Name	Systematic Name	Standard Name
YPL096C-A	ERI1	YER111C	SWI4
YKL139W	CTK1	YKL110C	KTI12
YNL087W	TCB2	YFR036W	CDC26
YDR004W	RAD57	YPL178W	CBC2
YDL184C	RPL41A	YDL083C	RPS16B
YPL022W	RAD1	YGL163C	RAD54
YHR178W	STB5	YAL002W	VPS8
YOR127W	RGA1	YJR135C	MCM22
YOL051W	GAL11	YER068W	MOT2
YPL001W	HAT1	YCR077C	PAT1
YOR290C	SNF2	YLR371W	ROM2
YOR076C	SKI7	YHR077C	NMD2
YAL024C	LTE1	YNL076W	MKS1
YOR039W	CKB2	YNL079C	TPM1
YJL036W	SNX4	YKR030W	GMH1
YNL248C	RPA49	YPR120C	CLB5
YMR224C	MRE11	YDL083C	RPS16B
YNL106C	INP52	YLR079W	SIC1
YHR178W	STB5	YBL021C	HAP3
YDL083C	RPS16B	YGL124C	MON1
YFR036W	CDC26	YOL054W	PSH1
YDL082W	RPL13A	YGL246C	RAI1
YDR004W	RAD57	YKL101W	HSL1
YDR378C	LSM6	YNL079C	TPM1
YBL071W-A	KTI11	YKL191W	DPH2
YML071C	COG8	YPL120W	VPS30
YML016C	PPZ1	YKL101W	HSL1
YDL116W	NUP84	YDL059C	RAD59
YBL047C	EDE1	YHL002W	HSE1

Predictions at threshold level 0.80 continued...			
Gene1 of predicted pair		Gene2 of predicted pair	
Systematic Name	Standard Name	Systematic Name	Standard Name
YJL036W	SNX4	YDL077C	VAM6
YDR254W	CHL4	YNL236W	SIN4
YGL078C	DBP3	YIL076W	SEC28
YNL058C	YNL058C	YER122C	GLO3
YOR243C	PUS7	YCL037C	SRO9
YML095C	RAD10	YML102W	CAC2
YJL138C	TIF2	YGL019W	CKB1
YDR137W	RGP1	YFR036W	CDC26
YNL090W	RHO2	YDL240W	LRG1
YER115C	SPR6	YMR199W	CLN1
YLR337C	VRP1	YPL096C-A	ERI1
YOR035C	SHE4	YLR180W	SAM1
YIL156W	UBP7	YOL126C	MDH2
YKL048C	ELM1	YDL077C	VAM6
YLR079W	SIC1	YKL213C	DOA1
YLR309C	IMH1	YAR014C	BUD14
YER144C	UBP5	YEL060C	PRB1
YAL051W	OAF1	YEL056W	HAT2
YKR057W	RPS21A	YKL048C	ELM1
YKL048C	ELM1	YLR370C	ARC18
YLR234W	TOP3	YNL297C	MON2
YDL116W	NUP84	YDL013W	SLX5
YER118C	SHO1	YJL183W	MNN11
YDR383C	NKP1	YOL054W	PSH1
YOR109W	INP53	YCR066W	RAD18
YDR137W	RGP1	YJR084W	CSN12
YNL044W	YIP3	YKR020W	VPS51
YGR108W	CLB1	YBL051C	PIN4
YPR051W	MAK3	YKR020W	VPS51

Predictions at threshold level 0.80 continued...			
Gene1 of predicted pair		Gene2 of predicted pair	
Systematic Name	Standard Name	Systematic Name	Standard Name
YLR095C	IOC2	YMR311C	GLC8
YNL032W	SIW14	YLR370C	ARC18
YKR048C	NAP1	YGL031C	RPL24A
YBR108W	YBR108W	YJL183W	MNN11
YJR032W	CPR7	YHR081W	LRP1
YGR200C	ELP2	YBL071W-A	KTI11
YNL215W	IES2	YER161C	SPT2
YNL291C	MID1	YJR135C	MCM22
YKL116C	PRR1	YNL058C	YNL058C
YDR477W	SNF1	YDR358W	GGA1
YOR337W	TEA1	YPL161C	BEM4
YPL253C	VIK1	YNL107W	YAF9
YEL065W	SIT1	YHR060W	VMA22
YCR097W	HMRA1	YCR039C	MATALPHA2
YIL076W	SEC28	YKR099W	BAS1
YMR152W	YIM1	YER139C	YER139C
YPL001W	HAT1	YBR274W	CHK1
YPR120C	CLB5	YOL091W	SPO21
YNL106C	INP52	YML016C	PPZ1
YGL153W	PEX14	YJL095W	BCK1
YML117W	NAB6	YMR038C	CCS1
YPL115C	BEM3	YCR086W	CSM1
YOR066W	MSA1	YBR019C	GAL10
YCL001W	RER1	YNL238W	KEX2
YOR197W	MCA1	YBL106C	SRO77
YIL076W	SEC28	YEL053C	MAK10
YLR360W	VPS38	YMR199W	CLN1
YDR363W-A	SEM1	YGR252W	GCN5
YPR017C	DSS4	YER122C	GLO3

Predictions at threshold level 0.80 continued...			
Gene1 of predicted pair		Gene2 of predicted pair	
Systematic Name	Standard Name	Systematic Name	Standard Name
YDR443C	SSN2	YGL115W	SNF4
YLR360W	VPS38	YER162C	RAD4
YMR199W	CLN1	YEL036C	ANP1
YGL147C	RPL9A	YER122C	GLO3
YNL068C	FKH2	YNL229C	URE2
YLR135W	SLX4	YCR086W	CSM1
YDR245W	MNN10	YPL213W	LEA1
YGL133W	ITC1	YIL008W	URM1
YER068W	MOT2	YGR134W	CAF130
YER110C	KAP123	YBR171W	SEC66
YGL212W	VAM7	YDR080W	VPS41
YMR055C	BUB2	YPR046W	MCM16
YNL325C	FIG4	YNL307C	MCK1
YNL297C	MON2	YGR105W	VMA21
YMR224C	MRE11	YHR115C	DMA1
YGR020C	VMA7	YOL018C	TLG2
YKL032C	IXR1	YDL155W	CLB3
YGL078C	DBP3	YDL077C	VAM6
YDR146C	SWI5	YDR207C	UME6
YNL107W	YAF9	YLR182W	SWI6
YOR270C	VPH1	YMR152W	YIM1
YOL121C	RPS19A	YMR311C	GLC8
YPL042C	SSN3	YOR080W	DIA2
YMR164C	MSS11	YKL068W	NUP100
YMR231W	PEP5	YLR093C	NYV1
YOR127W	RGA1	YJL183W	MNN11
YHR012W	VPS29	YJL095W	BCK1
YBL072C	RPS8A	YOL081W	IRA2
YER144C	UBP5	YJR084W	CSN12

Predictions at threshold level 0.80 continued...			
Gene1 of predicted pair		Gene2 of predicted pair	
Systematic Name	Standard Name	Systematic Name	Standard Name
YDR103W	STE5	YCL032W	STE50
YLR386W	VAC14	YBR200W	BEM1
YFL048C	EMP47	YEL036C	ANP1
YLR234W	TOP3	YNL016W	PUB1
YBL103C	RTG3	YOL067C	RTG1
YJL078C	PRY3	YFL044C	OTU1
YKR072C	SIS2	YPL001W	HAT1
YMR199W	CLN1	YMR311C	GLC8
YOR001W	RRP6	YLR182W	SWI6
YIL159W	BNR1	YPL037C	EGD1
YBR108W	YBR108W	YER139C	YER139C
YHR161C	YAP1801	YAL030W	SNC1
YLR234W	TOP3	YMR038C	CCS1
YKL139W	CTK1	YNL325C	FIG4
YOR275C	RIM20	YKR048C	NAP1
YPL253C	VIK1	YOL067C	RTG1
YDL116W	NUP84	YMR198W	CIK1
YMR224C	MRE11	YLR441C	RPS1A
YER044C	ERG28	YML071C	COG8
YLR079W	SIC1	YJR063W	RPA12
YAL014C	SYN8	YOR036W	PEP12
YNL106C	INP52	YPL018W	CTF19
YMR224C	MRE11	YMR055C	BUB2
YPL253C	VIK1	YHR111W	UBA4
YBL008W	HIR1	YGL003C	CDH1
YDL006W	PTC1	YBR082C	UBC4
YLR006C	SSK1	YGL038C	OCH1
YPR072W	NOT5	YOR064C	YNG1
YFR036W	CDC26	YLR398C	SKI2

Predictions at threshold level 0.80 continued...			
**Gene1 of predicted pair**		**Gene2 of predicted pair**	
**Systematic Name**	**Standard Name**	**Systematic Name**	**Standard Name**
YLR360W	VPS38	YOR035C	SHE4
YHR178W	STB5	YMR055C	BUB2
YHR013C	ARD1	YBL025W	RRN10
YDR071C	PAA1	YKL114C	APN1
YMR224C	MRE11	YHR111W	UBA4
YIL036W	CST6	YGR270W	YTA7
YKL048C	ELM1	YJL117W	PHO86
YCL037C	SRO9	YKR020W	VPS51
YBR108W	YBR108W	YHL002W	HSE1
YML112W	CTK3	YOL054W	PSH1
YPR072W	NOT5	YGR134W	CAF130
YBL051C	PIN4	YOL067C	RTG1
YGR104C	SRB5	YBR289W	SNF5
YPR084W	YPR084W	YOR069W	VPS5
YOR035C	SHE4	YMR089C	YTA12
YKL007W	CAP1	YCR088W	ABP1
YOR112W	CEX1	YPL105C	YPL105C
YDR225W	HTA1	YDR227W	SIR4
YFR031C-A	RPL2A	YDR080W	VPS41
YOR144C	ELG1	YHR056C	RSC30
YBR065C	ECM2	YBR188C	NTC20
YPL115C	BEM3	YLR381W	CTF3
YBR194W	SOY1	YMR202W	ERG2
YNL054W	VAC7	YOL081W	IRA2
YDL101C	DUN1	YER116C	SLX8
YOR066W	MSA1	YJR084W	CSN12
YGR105W	VMA21	YDL192W	ARF1
YLR182W	SWI6	YOL081W	IRA2
YLR357W	RSC2	YNL107W	YAF9

Predictions at threshold level 0.80 continued...			
Gene1 of predicted pair		Gene2 of predicted pair	
Systematic Name	Standard Name	Systematic Name	Standard Name
YDL065C	PEX19	YAL055W	PEX22
YKL007W	CAP1	YLR006C	SSK1
YHR111W	UBA4	YJR063W	RPA12
YOR043W	WHI2	YLR094C	GIS3
YLR447C	VMA6	YEL051W	VMA8
YIL159W	BNR1	YOR043W	WHI2
YMR095C	SNO1	YNL333W	SNZ2
YHL027W	RIM101	YMR032W	HOF1
YOR106W	VAM3	YAL002W	VPS8
YJL006C	CTK2	YJR035W	RAD26
YNL297C	MON2	YKL119C	VPH2
YMR133W	REC114	YJL095W	BCK1
YOR375C	GDH1	YOR141C	ARP8
YDR440W	DOT1	YHR167W	THP2
YOL018C	TLG2	YNL107W	YAF9
YPL253C	VIK1	YLR032W	RAD5
YKR095W	MLP1	YJR090C	GRR1
YDL059C	RAD59	YMR231W	PEP5
YKR059W	TIF1	YOL081W	IRA2
YNL032W	SIW14	YBR200W	BEM1
YOL062C	APM4	YJR005W	APL1
YBL051C	PIN4	YDR507C	GIN4
YCR034W	FEN1	YGL019W	CKB1
YGR072W	UPF3	YEL051W	VMA8
YIL159W	BNR1	YDR261C	EXG2
YOR290C	SNF2	YGL025C	PGD1
YOR043W	WHI2	YMR133W	REC114
YLR325C	RPL38	YIL133C	RPL16A
YPR131C	NAT3	YFL023W	BUD27

Predictions at threshold level 0.80 continued...			
Gene1 of predicted pair		Gene2 of predicted pair	
Systematic Name	Standard Name	Systematic Name	Standard Name
YGR241C	YAP1802	YJR035W	RAD26
YJL154C	VPS35	YJL004C	SYS1
YMR142C	RPL13B	YMR311C	GLC8
YBR173C	UMP1	YPL161C	BEM4
YPL161C	BEM4	YKL101W	HSL1
YDL116W	NUP84	YJL127C	SPT10
YLR095C	IOC2	YPL024W	RMI1
YDR004W	RAD57	YDL013W	SLX5
YFR036W	CDC26	YLR093C	NYV1
YLR452C	SST2	YOR327C	SNC2
YER110C	KAP123	YAL002W	VPS8
YMR016C	SOK2	YJR032W	CPR7
YDR318W	MCM21	YGL124C	MON1
YEL051W	VMA8	YPL120W	VPS30
YER087C-B	SBH1	YGL244W	RTF1
YEL050C	RML2	YGL115W	SNF4
YBR173C	UMP1	YBR108W	YBR108W
YLR399C	BDF1	YGR270W	YTA7
YJR090C	GRR1	YDR139C	RUB1
YNL291C	MID1	YHR060W	VMA22
YNL325C	FIG4	YHR039C-A	VMA10
YDR293C	SSD1	YKR030W	GMH1
YOR327C	SNC2	YNL076W	MKS1
YDR004W	RAD57	YOR038C	HIR2
YMR029C	FAR8	YMR052W	FAR3
YDR245W	MNN10	YHL011C	PRS3
YDR225W	HTA1	YOL148C	SPT20
YNL041C	COG6	YPR032W	SRO7
YGL163C	RAD54	YFR030W	MET10

Predictions at threshold level 0.80 continued...			
Gene1 of predicted pair		Gene2 of predicted pair	
Systematic Name	Standard Name	Systematic Name	Standard Name
YLR119W	SRN2	YBR280C	SAF1
YDR108W	GSG1	YMR004W	MVP1
YOR195W	SLK19	YDR378C	LSM6
YKL048C	ELM1	YDL047W	SIT4
YOR001W	RRP6	YNL236W	SIN4
YGR019W	UGA1	YPL253C	VIK1
YBR200W	BEM1	YNL041C	COG6
YBR108W	YBR108W	YKL048C	ELM1
YDR174W	HMO1	YDR439W	LRS4
YCL037C	SRO9	YJR060W	CBF1
YPL137C	GIP3	YLR370C	ARC18
YCL037C	SRO9	YDR378C	LSM6
YGL212W	VAM7	YJR032W	CPR7
YNL236W	SIN4	YGL151W	NUT1
YLR181C	VTA1	YKL002W	DID4
YMR026C	PEX12	YLR320W	MMS22
YIL159W	BNR1	YJR032W	CPR7
YGL200C	EMP24	YLR370C	ARC18
YEL053C	MAK10	YJL190C	RPS22A
YMR055C	BUB2	YDR289C	RTT103
YNL297C	MON2	YNL041C	COG6
YCR020C-A	MAK31	YAR014C	BUD14
YJR099W	YUH1	YLR128W	DCN1
YHR039C-A	VMA10	YGL163C	RAD54
YDR156W	RPA14	YMR038C	CCS1
YNR072W	HXT17	YMR214W	SCJ1
YIL034C	CAP2	YHR012W	VPS29
YML103C	NUP188	YLR079W	SIC1
YER162C	RAD4	YLR398C	SKI2

Predictions at threshold level 0.80 continued...			
Gene1 of predicted pair		Gene2 of predicted pair	
Systematic Name	Standard Name	Systematic Name	Standard Name
YOR337W	TEA1	YNL238W	KEX2
YER116C	SLX8	YMR230W	RPS10B
YMR142C	RPL13B	YPL057C	SUR1
YDR225W	HTA1	YDR176W	NGG1
YJR024C	MDE1	YPL256C	CLN2
YMR026C	PEX12	YOL081W	IRA2
YER096W	SHC1	YBR260C	RGD1
YCR034W	FEN1	YLR435W	TSR2
YDR137W	RGP1	YCR053W	THR4
YHR119W	SET1	YDR469W	SDC1
YKR001C	VPS1	YOR035C	SHE4
YGL237C	HAP2	YKL109W	HAP4
YGL163C	RAD54	YAR014C	BUD14
YKL126W	YPK1	YEL060C	PRB1
YML112W	CTK3	YOL081W	IRA2
YPL037C	EGD1	YHR081W	LRP1
YIL053W	RHR2	YNL041C	COG6
YJR140C	HIR3	YDR245W	MNN10
YKL048C	ELM1	YDR264C	AKR1
YMR127C	SAS2	YDL155W	CLB3
YNL214W	PEX17	YAL055W	PEX22
YDR174W	HMO1	YLR254C	NDL1
YOR080W	DIA2	YDR137W	RGP1
YER116C	SLX8	YLR094C	GIS3
YHR134W	WSS1	YPL018W	CTF19
YEL053C	MAK10	YHR039C-A	VMA10
YOL067C	RTG1	YDR310C	SUM1
YLR386W	VAC14	YNR072W	HXT17
YKR099W	BAS1	YPR030W	CSR2

Predictions at threshold level 0.80 continued...			
Gene1 of predicted pair		Gene2 of predicted pair	
Systematic Name	Standard Name	Systematic Name	Standard Name
YLR315W	NKP2	YOL054W	PSH1
YOR039W	CKB2	YBR009C	HHF1
YHR200W	RPN10	YNL238W	KEX2
YNR052C	POP2	YGR188C	BUB1
YLR119W	SRN2	YER044C	ERG28
YLR234W	TOP3	YLR350W	ORM2
YLR357W	RSC2	YNL030W	HHF2
YJR032W	CPR7	YOL121C	RPS19A
YBR042C	CST26	YGL200C	EMP24
YJL095W	BCK1	YGL253W	HXK2
YFR010W	UBP6	YHR039C-A	VMA10
YPL161C	BEM4	YDR155C	CPR1
YPL234C	TFP3	YOL108C	INO4
YKR061W	KTR2	YJR060W	CBF1
YLR386W	VAC14	YGL252C	RTG2
YNL127W	FAR11	YNL051W	COG5
YBR217W	ATG12	YER139C	YER139C
YHL011C	PRS3	YOL081W	IRA2
YDL192W	ARF1	YMR311C	GLC8
YLR288C	MEC3	YOR363C	PIP2
YGL134W	PCL10	YPL240C	HSP82
YGL060W	YBP2	YOR083W	WHI5
YLR090W	XDJ1	YJL183W	MNN11
YDR153C	ENT5	YOL108C	INO4
YOR035C	SHE4	YDL077C	VAM6
YNL225C	CNM67	YOL081W	IRA2
YPR084W	YPR084W	YCR020C-A	MAK31
YJL060W	BNA3	YDR171W	HSP42
YIL159W	BNR1	YDL077C	VAM6

Predictions at threshold level 0.80 continued...			
Gene1 of predicted pair		Gene2 of predicted pair	
Systematic Name	Standard Name	Systematic Name	Standard Name
YDR080W	VPS41	YDR310C	SUM1
YKL048C	ELM1	YJR032W	CPR7
YFR010W	UBP6	YJR140C	HIR3
YMR095C	SNO1	YPL137C	GIP3
YNL032W	SIW14	YFR036W	CDC26
YIL034C	CAP2	YJL095W	BCK1
YOR109W	INP53	YNR006W	VPS27
YDR137W	RGP1	YBR280C	SAF1
YDR456W	NHX1	YJL095W	BCK1
YMR294W	JNM1	YGL190C	CDC55
YGR105W	VMA21	YKL119C	VPH2
YHR178W	STB5	YDR254W	CHL4
YDR004W	RAD57	YBL008W	HIR1
YMR026C	PEX12	YNL041C	COG6
YMR276W	DSK2	YJR099W	YUH1
YMR142C	RPL13B	YPR030W	CSR2
YHR012W	VPS29	YJL004C	SYS1
YHR200W	RPN10	YMR230W	RPS10B
YBR258C	SHG1	YPL057C	SUR1
YDL155W	CLB3	YBL101C	ECM21
YFR040W	SAP155	YER122C	GLO3
YMR216C	SKY1	YPR032W	SRO7
YBR171W	SEC66	YPL161C	BEM4
YEL009C	GCN4	YLR399C	BDF1
YNL201C	PSY2	YOL081W	IRA2
YBR215W	HPC2	YBR036C	CSG2
YNL291C	MID1	YML028W	TSA1
YMR026C	PEX12	YER162C	RAD4
YFR010W	UBP6	YER139C	YER139C

Predictions at threshold level 0.80 continued...			
Gene1 of predicted pair		Gene2 of predicted pair	
Systematic Name	Standard Name	Systematic Name	Standard Name
YLR254C	NDL1	YLR182W	SWI6
YPL234C	TFP3	YAR014C	BUD14
YIL008W	URM1	YAR014C	BUD14
YOR270C	VPH1	YOL081W	IRA2
YAL024C	LTE1	YGR135W	PRE9
YOR275C	RIM20	YJL117W	PHO86
YMR109W	MYO5	YDR254W	CHL4
YLR381W	CTF3	YGL124C	MON1
YMR224C	MRE11	YBR019C	GAL10
YOR195W	SLK19	YDR076W	RAD55
YDR379W	RGA2	YPL161C	BEM4
YML016C	PPZ1	YOL081W	IRA2
YNL307C	MCK1	YOL121C	RPS19A
YIL036W	CST6	YPL152W	RRD2
YGR133W	PEX4	YAL055W	PEX22
YOR270C	VPH1	YLR371W	ROM2
YPR046W	MCM16	YJR084W	CSN12
YJR135C	MCM22	YPR119W	CLB2
YLR095C	IOC2	YOR043W	WHI2
YKR001C	VPS1	YJR058C	APS2
YOL121C	RPS19A	YJR084W	CSN12
YDL020C	RPN4	YNL238W	KEX2
YPL253C	VIK1	YGL003C	CDH1
YJL176C	SWI3	YBL047C	EDE1
YDL135C	RDI1	YDL240W	LRG1
YHR119W	SET1	YLR288C	MEC3
YDR202C	RAV2	YNL238W	KEX2
YIL159W	BNR1	YNL307C	MCK1
YAL024C	LTE1	YKR072C	SIS2

Predictions at threshold level 0.80 continued...			
Gene1 of predicted pair		Gene2 of predicted pair	
Systematic Name	Standard Name	Systematic Name	Standard Name
YOR127W	RGA1	YDR155C	CPR1
YJL124C	LSM1	YDR318W	MCM21
YJR032W	CPR7	YGL151W	NUT1
YLR234W	TOP3	YIL008W	URM1
YHR206W	SKN7	YNL027W	CRZ1
YFL023W	BUD27	YDL083C	RPS16B
YNL106C	INP52	YMR055C	BUB2
YER110C	KAP123	YJL190C	RPS22A
YPL042C	SSN3	YBR107C	IML3
YPL096C-A	ERI1	YHR030C	SLT2
YHR039C-A	VMA10	YAR014C	BUD14
YNL215W	IES2	YKL139W	CTK1
YER001W	MNN1	YNL051W	COG5
YMR224C	MRE11	YGL161C	YIP5
YOR275C	RIM20	YHR012W	VPS29
YGR135W	PRE9	YEL036C	ANP1
YLR429W	CRN1	YDL226C	GCS1
YMR153W	NUP53	YPL137C	GIP3
YDL006W	PTC1	YOR036W	PEP12
YMR016C	SOK2	YLR441C	RPS1A
YNL206C	RTT106	YHR111W	UBA4
YDR379W	RGA2	YGL163C	RAD54
YDR443C	SSN2	YMR153W	NUP53
YDR323C	PEP7	YOR036W	PEP12
YJL124C	LSM1	YDR310C	SUM1
YFL023W	BUD27	YNL307C	MCK1
YKL080W	VMA5	YKL041W	VPS24
YNL307C	MCK1	YJR135C	MCM22
YDL101C	DUN1	YMR055C	BUB2

Predictions at threshold level 0.80 continued...			
Gene1 of predicted pair		Gene2 of predicted pair	
Systematic Name	Standard Name	Systematic Name	Standard Name
YPL105C	YPL105C	YML001W	YPT7
YBR171W	SEC66	YIL008W	URM1
YHR039C-A	VMA10	YPR030W	CSR2
YDL116W	NUP84	YLR381W	CTF3
YDL020C	RPN4	YPL079W	RPL21B
YOR127W	RGA1	YBR084C-A	RPL19A
YDR254W	CHL4	YNL147W	LSM7
YBR274W	CHK1	YLR320W	MMS22
YGR159C	NSR1	YOR141C	ARP8
YPL037C	EGD1	YER044C	ERG28
YGR104C	SRB5	YDL002C	NHP10
YGR104C	SRB5	YDR363W-A	SEM1
YDR129C	SAC6	YKR020W	VPS51
YBR217W	ATG12	YMR152W	YIM1
YPL057C	SUR1	YOR124C	UBP2
YHR010W	RPL27A	YOR368W	RAD17
YOR001W	RRP6	YOR124C	UBP2
YGR020C	VMA7	YML001W	YPT7
YDR156W	RPA14	YJL095W	BCK1
YLR192C	HCR1	YLR386W	VAC14
YNL238W	KEX2	YPR030W	CSR2
YDR439W	LRS4	YGL163C	RAD54
YLR094C	GIS3	YCR053W	THR4
YDL161W	ENT1	YJL029C	VPS53
YJL172W	CPS1	YBR019C	GAL10
YOR001W	RRP6	YPL178W	CBC2
YAL024C	LTE1	YHR081W	LRP1
YJL124C	LSM1	YDL155W	CLB3
YKL048C	ELM1	YIR005W	IST3

Predictions at threshold level 0.80 continued...			
Gene1 of predicted pair		Gene2 of predicted pair	
Systematic Name	Standard Name	Systematic Name	Standard Name
YGL163C	RAD54	YJL095W	BCK1
YFL031W	HAC1	YDR207C	UME6
YML088W	UFO1	YDR139C	RUB1
YGL147C	RPL9A	YGL212W	VAM7
YER139C	YER139C	YJR084W	CSN12
YPL253C	VIK1	YJL183W	MNN11
YMR038C	CCS1	YNL107W	YAF9
YBR108W	YBR108W	YMR016C	SOK2
YMR167W	MLH1	YNL076W	MKS1
YGR108W	CLB1	YGR054W	YGR054W
YLR337C	VRP1	YKR020W	VPS51
YMR016C	SOK2	YGL003C	CDH1
YPL161C	BEM4	YJR084W	CSN12
YDR334W	SWR1	YBR089C-A	NHP6B
YMR231W	PEP5	YDL077C	VAM6
YLR234W	TOP3	YOL081W	IRA2
YDR137W	RGP1	YMR054W	STV1
YCR066W	RAD18	YER122C	GLO3
YNL068C	FKH2	YDL047W	SIT4
YPL018W	CTF19	YEL056W	HAT2
YGL212W	VAM7	YDR153C	ENT5
YML016C	PPZ1	YLR429W	CRN1
YDL006W	PTC1	YDR153C	ENT5
YJL136C	RPS21B	YDR379W	RGA2
YCR065W	HCM1	YER114C	BOI2
YIL159W	BNR1	YBR019C	GAL10
YBL008W	HIR1	YKR072C	SIS2
YHR060W	VMA22	YML001W	YPT7
YNL307C	MCK1	YPR120C	CLB5

Predictions at threshold level 0.80 continued...			
**Gene1 of predicted pair**		**Gene2 of predicted pair**	
**Systematic Name**	**Standard Name**	**Systematic Name**	**Standard Name**
YOR290C	SNF2	YNR072W	HXT17
YLR386W	VAC14	YNL225C	CNM67
YNL288W	CAF40	YGR134W	CAF130
YML016C	PPZ1	YOR358W	HAP5
YCR077C	PAT1	YAL002W	VPS8
YJL176C	SWI3	YBR057C	MUM2
YDR477W	SNF1	YLR370C	ARC18
YML001W	YPT7	YCR053W	THR4
YMR224C	MRE11	YDR439W	LRS4
YOR132W	VPS17	YBR171W	SEC66
YLR288C	MEC3	YER162C	RAD4
YLR234W	TOP3	YGR184C	UBR1
YDR422C	SIP1	YJR090C	GRR1
YOR275C	RIM20	YKL041W	VPS24
YKL116C	PRR1	YBL103C	RTG3
YER110C	KAP123	YGR054W	YGR054W
YBL008W	HIR1	YJR032W	CPR7
YER116C	SLX8	YOR290C	SNF2
YJL210W	PEX2	YGR077C	PEX8
YMR273C	ZDS1	YPL256C	CLN2
YOR035C	SHE4	YLR370C	ARC18
YCR081W	SRB8	YLR371W	ROM2
YBR171W	SEC66	YLR398C	SKI2
YML094W	GIM5	YEL022W	GEA2
YBL102W	SFT2	YOR069W	VPS5
YEL027W	CUP5	YHR060W	VMA22
YHR134W	WSS1	YHR111W	UBA4
YMR026C	PEX12	YBL103C	RTG3
YKR020W	VPS51	YER122C	GLO3

Predictions at threshold level 0.80 continued...			
Gene1 of predicted pair		Gene2 of predicted pair	
Systematic Name	Standard Name	Systematic Name	Standard Name
YPL234C	TFP3	YPR032W	SRO7
YDR108W	GSG1	YPR051W	MAK3
YIL162W	SUC2	YEL036C	ANP1
YGR249W	MGA1	YNL238W	KEX2
YDR103W	STE5	YDR309C	GIC2
YMR199W	CLN1	YPL158C	YPL158C
YLR094C	GIS3	YMR214W	SCJ1
YPR131C	NAT3	YPL106C	SSE1
YLR386W	VAC14	YOR270C	VPH1
YNL025C	SSN8	YBL075C	SSA3
YMR142C	RPL13B	YFR036W	CDC26
YGL095C	VPS45	YDL047W	SIT4
YGL087C	MMS2	YJL190C	RPS22A
YMR253C	YMR253C	YHR114W	BZZ1
YDR448W	ADA2	YBL003C	HTA2
YBR171W	SEC66	YPL137C	GIP3
YKL204W	EAP1	YKL213C	DOA1
YOR106W	VAM3	YGL223C	COG1
YBR200W	BEM1	YJL058C	BIT61
YJL053W	PEP8	YPL195W	APL5
YCR053W	THR4	YEL060C	PRB1
YMR142C	RPL13B	YOL081W	IRA2
YDL116W	NUP84	YNL016W	PUB1
YMR199W	CLN1	YJL136C	RPS21B
YBR127C	VMA2	YGL095C	VPS45
YOR043W	WHI2	YER122C	GLO3
YDL002C	NHP10	YCR086W	CSM1
YML115C	VAN1	YDR137W	RGP1
YOR141C	ARP8	YAR014C	BUD14

Predictions at threshold level 0.80 continued...			
Gene1 of predicted pair		Gene2 of predicted pair	
Systematic Name	Standard Name	Systematic Name	Standard Name
YPR018W	RLF2	YKL160W	ELF1
YPR120C	CLB5	YPL158C	YPL158C
YOR312C	RPL20B	YDR310C	SUM1
YER129W	SAK1	YMR199W	CLN1
YML117W	NAB6	YLR320W	MMS22
YDR245W	MNN10	YMR109W	MYO5
YBR200W	BEM1	YDL226C	GCS1
YNL288W	CAF40	YBR114W	RAD16
YKL139W	CTK1	YAL002W	VPS8
YLR360W	VPS38	YJL053W	PEP8
YMR153W	NUP53	YGL124C	MON1
YNL106C	INP52	YNL215W	IES2
YHR086W	NAM8	YPR101W	SNT309
YDR443C	SSN2	YOR290C	SNF2
YNL307C	MCK1	YMR167W	MLH1
YIL076W	SEC28	YGL124C	MON1
YJL124C	LSM1	YOL081W	IRA2
YPL161C	BEM4	YPL234C	TFP3
YFL031W	HAC1	YCR053W	THR4
YKR020W	VPS51	YMR038C	CCS1
YML103C	NUP188	YCR024C-A	PMP1
YOR332W	VMA4	YML115C	VAN1
YOR066W	MSA1	YJL095W	BCK1
YMR172W	HOT1	YHR111W	UBA4
YHR060W	VMA22	YNL041C	COG6
YPL037C	EGD1	YOL111C	MDY2
YLR386W	VAC14	YDL006W	PTC1
YML102W	CAC2	YJR090C	GRR1
YMR199W	CLN1	YHR158C	KEL1

Predictions at threshold level 0.80 continued...			
Gene1 of predicted pair		Gene2 of predicted pair	
Systematic Name	Standard Name	Systematic Name	Standard Name
YOR132W	VPS17	YDR310C	SUM1
YMR133W	REC114	YEL060C	PRB1
YDR108W	GSG1	YPR032W	SRO7
YOR127W	RGA1	YDR363W-A	SEM1
YPL022W	RAD1	YMR199W	CLN1
YHR200W	RPN10	YMR276W	DSK2
YNL307C	MCK1	YOR014W	RTS1
YBR200W	BEM1	YOR001W	RRP6
YML016C	PPZ1	YNL307C	MCK1
YIL034C	CAP2	YPL050C	MNN9
YLR191W	PEX13	YIL160C	POT1
YNL238W	KEX2	YMR038C	CCS1
YOR195W	SLK19	YKR048C	NAP1
YGL115W	SNF4	YOL108C	INO4
YNL025C	SSN8	YDR245W	MNN10
YMR127C	SAS2	YPL253C	VIK1
YNL201C	PSY2	YPR120C	CLB5
YOR132W	VPS17	YOR112W	CEX1
YOR337W	TEA1	YNL307C	MCK1
YDR260C	SWM1	YPR119W	CLB2
YHR134W	WSS1	YGR188C	BUB1
YLR119W	SRN2	YGL003C	CDH1
YDR137W	RGP1	YDR310C	SUM1
YNL069C	RPL16B	YGR020C	VMA7
YOR332W	VMA4	YMR070W	MOT3
YKL025C	PAN3	YPL018W	CTF19
YOR023C	AHC1	YGL253W	HXK2
YHR200W	RPN10	YAR014C	BUD14
YKR014C	YPT52	YPR028W	YOP1

Predictions at threshold level 0.80 continued...			
Gene1 of predicted pair		Gene2 of predicted pair	
Systematic Name	Standard Name	Systematic Name	Standard Name
YLR337C	VRP1	YCR086W	CSM1
YMR224C	MRE11	YLR150W	STM1
YIL148W	RPL40A	YMR125W	STO1
YFR031C-A	RPL2A	YML038C	YMD8
YOR290C	SNF2	YNL041C	COG6
YMR172W	HOT1	YJL095W	BCK1
YOL126C	MDH2	YFL047W	RGD2
YDR146C	SWI5	YLR425W	TUS1
YFR010W	UBP6	YBR030W	YBR030W
YMR224C	MRE11	YLR410W	VIP1
YFL013C	IES1	YIL076W	SEC28
YKL032C	IXR1	YPL161C	BEM4
YER044C	ERG28	YOR043W	WHI2
YPL115C	BEM3	YJR032W	CPR7
YHL020C	OPI1	YEL051W	VMA8
YER110C	KAP123	YNL166C	BNI5
YBR030W	YBR030W	YBR280C	SAF1
YNL215W	IES2	YJL183W	MNN11
YBR194W	SOY1	YDR363W-A	SEM1
YLR386W	VAC14	YMR142C	RPL13B
YFR010W	UBP6	YKR030W	GMH1
YJR056C	YJR056C	YMR214W	SCJ1
YNL297C	MON2	YHR115C	DMA1
YGR104C	SRB5	YGR135W	PRE9
YHR039C-A	VMA10	YJL095W	BCK1
YDR477W	SNF1	YDR207C	UME6
YDL020C	RPN4	YNL107W	YAF9
YBR108W	YBR108W	YNL307C	MCK1
YMR216C	SKY1	YKR001C	VPS1

Predictions at threshold level 0.80 continued...			
Gene1 of predicted pair		Gene2 of predicted pair	
Systematic Name	Standard Name	Systematic Name	Standard Name
YNL236W	SIN4	YJR005W	APL1
YNL087W	TCB2	YDR358W	GGA1
YIR005W	IST3	YOR124C	UBP2
YJR084W	CSN12	YGR188C	BUB1
YPL256C	CLN2	YHL011C	PRS3
YOR064C	YNG1	YNL030W	HHF2
YHR156C	LIN1	YLR320W	MMS22
YPL024W	RMI1	YEL053C	MAK10
YDL160C	DHH1	YPL256C	CLN2
YPL115C	BEM3	YJR090C	GRR1
YNL201C	PSY2	YNL054W	VAC7
YHR111W	UBA4	YJL190C	RPS22A
YGR072W	UPF3	YNL107W	YAF9
YPR051W	MAK3	YGL115W	SNF4
YML095C	RAD10	YGR135W	PRE9
YPR017C	DSS4	YML038C	YMD8
YNL307C	MCK1	YMR133W	REC114
YKL126W	YPK1	YPR030W	CSR2
YHR156C	LIN1	YER162C	RAD4
YER162C	RAD4	YDL077C	VAM6
YDR477W	SNF1	YPL248C	GAL4
YBR173C	UMP1	YLR398C	SKI2
YML016C	PPZ1	YGL115W	SNF4
YHR200W	RPN10	YDR469W	SDC1
YDR254W	CHL4	YHR115C	DMA1
YBR057C	MUM2	YGL163C	RAD54
YDR443C	SSN2	YDR358W	GGA1
YDR004W	RAD57	YPL057C	SUR1
YLR095C	IOC2	YPL137C	GIP3

Predictions at threshold level 0.80 continued...			
Gene1 of predicted pair		Gene2 of predicted pair	
Systematic Name	Standard Name	Systematic Name	Standard Name
YFR010W	UBP6	YMR214W	SCJ1
YBR290W	BSD2	YER110C	KAP123
YIL076W	SEC28	YNR032W	PPG1
YKR099W	BAS1	YLR398C	SKI2
YNL291C	MID1	YGL115W	SNF4
YMR224C	MRE11	YOR014W	RTS1
YMR026C	PEX12	YBR108W	YBR108W
YOR270C	VPH1	YKL126W	YPK1
YPL057C	SUR1	YKL048C	ELM1
YLR234W	TOP3	YPL057C	SUR1
YDL005C	MED2	YLR182W	SWI6
YMR026C	PEX12	YOL044W	PEX15
YKL007W	CAP1	YAL024C	LTE1
YCL001W	RER1	YER162C	RAD4
YMR142C	RPL13B	YDR484W	VPS52
YNL291C	MID1	YOR109W	INP53
YEL060C	PRB1	YDR310C	SUM1
YOR023C	AHC1	YBL021C	HAP3
YLR381W	CTF3	YEL056W	HAT2
YDR156W	RPA14	YMR224C	MRE11
YGR252W	GCN5	YOL148C	SPT20
YNL307C	MCK1	YKR001C	VPS1
YDR192C	NUP42	YBR017C	KAP104
YDL101C	DUN1	YOL090W	MSH2
YNL288W	CAF40	YNR052C	POP2
YMR016C	SOK2	YLR320W	MMS22
YKL116C	PRR1	YER044C	ERG28
YPR051W	MAK3	YML038C	YMD8
YNL041C	COG6	YJL095W	BCK1

Predictions at threshold level 0.80 continued...			
Gene1 of predicted pair		Gene2 of predicted pair	
Systematic Name	Standard Name	Systematic Name	Standard Name
YOR112W	CEX1	YEL053C	MAK10
YOL030W	GAS5	YBR082C	UBC4
YDR329C	PEX3	YOL147C	PEX11
YPL024W	RMI1	YOL111C	MDY2
YHR060W	VMA22	YDL083C	RPS16B
YDR108W	GSG1	YJL036W	SNX4
YOR001W	RRP6	YCR077C	PAT1
YHR134W	WSS1	YNL016W	PUB1
YFR010W	UBP6	YOR124C	UBP2
YHL020C	OPI1	YNL051W	COG5
YBR217W	ATG12	YLR386W	VAC14
YNL116W	DMA2	YGL031C	RPL24A
YMR055C	BUB2	YCR086W	CSM1
YNL238W	KEX2	YBR036C	CSG2
YLR090W	XDJ1	YLR320W	MMS22
YOR368W	RAD17	YNL107W	YAF9
YBL008W	HIR1	YLR079W	SIC1
YNL032W	SIW14	YER139C	YER139C
YMR198W	CIK1	YPR119W	CLB2
YNL087W	TCB2	YJL095W	BCK1
YER115C	SPR6	YPL178W	CBC2
YIR009W	MSL1	YDR184C	ATC1
YFL013C	IES1	YOR124C	UBP2
YBL008W	HIR1	YNL107W	YAF9
YBL058W	SHP1	YBR036C	CSG2
YDR484W	VPS52	YOL111C	MDY2
YMR198W	CIK1	YLR058C	SHM2
YFL023W	BUD27	YPL256C	CLN2
YEL053C	MAK10	YGR188C	BUB1

Predictions at threshold level 0.80 continued...			
Gene1 of predicted pair		Gene2 of predicted pair	
Systematic Name	Standard Name	Systematic Name	Standard Name
YLR377C	FBP1	YNR065C	YNR065C
YOR069W	VPS5	YKR020W	VPS51
YLR079W	SIC1	YGL115W	SNF4
YDL083C	RPS16B	YFR036W	CDC26
YLR119W	SRN2	YMR142C	RPL13B
YEL065W	SIT1	YOL121C	RPS19A
YBR171W	SEC66	YPL227C	ALG5
YFR010W	UBP6	YLR371W	ROM2
YHR084W	STE12	YDL159W	STE7
YJR135C	MCM22	YLR370C	ARC18
YLR452C	SST2	YDR289C	RTT103
YHR056C	RSC30	YCL061C	MRC1
YOR127W	RGA1	YCL001W	RER1
YMR055C	BUB2	YPR119W	CLB2
YDR289C	RTT103	YML112W	CTK3
YGR188C	BUB1	YAL002W	VPS8
YGR252W	GCN5	YEL056W	HAT2
YBR200W	BEM1	YFR036W	CDC26
YFL013C	IES1	YAL002W	VPS8
YJR135C	MCM22	YJL095W	BCK1
YOL111C	MDY2	YJL183W	MNN11
YDR335W	MSN5	YCR077C	PAT1
YDR225W	HTA1	YDL155W	CLB3
YDR320C	SWA2	YOL108C	INO4
YPL234C	TFP3	YMR054W	STV1
YGL078C	DBP3	YJL172W	CPS1
YHR156C	LIN1	YOR327C	SNC2
YJL136C	RPS21B	YOR275C	RIM20
YOR033C	EXO1	YDR076W	RAD55

Predictions at threshold level 0.80 continued...			
Gene1 of predicted pair		Gene2 of predicted pair	
Systematic Name	Standard Name	Systematic Name	Standard Name
YNL106C	INP52	YCR088W	ABP1
YLR399C	BDF1	YCR086W	CSM1
YLR119W	SRN2	YKL126W	YPK1
YML115C	VAN1	YMR230W	RPS10B
YFR010W	UBP6	YGR232W	NAS6
YHR012W	VPS29	YJL183W	MNN11
YGR105W	VMA21	YJL183W	MNN11
YPL024W	RMI1	YOR337W	TEA1
YLR058C	SHM2	YDL226C	GCS1
YLR148W	PEP3	YDL161W	ENT1
YMR224C	MRE11	YDL002C	NHP10
YPL086C	ELP3	YHR187W	IKI1
YPR018W	RLF2	YLR352W	YLR352W
YEL053C	MAK10	YHR012W	VPS29
YDR245W	MNN10	YPL161C	BEM4
YPR051W	MAK3	YNL107W	YAF9
YJL183W	MNN11	YDR207C	UME6
YLR360W	VPS38	YJR084W	CSN12
YMR153W	NUP53	YBR036C	CSG2
YBL102W	SFT2	YOR112W	CEX1
YOR270C	VPH1	YBR036C	CSG2
YPL195W	APL5	YNL076W	MKS1
YLR095C	IOC2	YDR477W	SNF1
YCR077C	PAT1	YEL013W	VAC8
YBL058W	SHP1	YDL083C	RPS16B
YPR046W	MCM16	YLR370C	ARC18
YCL037C	SRO9	YKL213C	DOA1
YHL020C	OPI1	YML071C	COG8
YLR191W	PEX13	YKL041W	VPS24

Predictions at threshold level 0.80 continued...			
Gene1 of predicted pair		Gene2 of predicted pair	
Systematic Name	Standard Name	Systematic Name	Standard Name
YIL002C	INP51	YMR070W	MOT3
YOR026W	BUB3	YGL190C	CDC55
YOR023C	AHC1	YPL018W	CTF19
YBR030W	YBR030W	YDR137W	RGP1
YKL126W	YPK1	YER044C	ERG28
YDL192W	ARF1	YLR398C	SKI2
YBR030W	YBR030W	YLR441C	RPS1A
YIL053W	RHR2	YBL025W	RRN10
YLR452C	SST2	YBR280C	SAF1
YDR443C	SSN2	YFL028C	CAF16
YLR447C	VMA6	YGR188C	BUB1
YOR106W	VAM3	YDR310C	SUM1
YEL027W	CUP5	YPL234C	TFP3
YGR105W	VMA21	YEL051W	VMA8
YLR360W	VPS38	YOR112W	CEX1
YMR224C	MRE11	YNL225C	CNM67
YNL032W	SIW14	YNL238W	KEX2
YLR094C	GIS3	YHR039C-A	VMA10
YLR102C	APC9	YFR036W	CDC26
YNL106C	INP52	YKL048C	ELM1
YJL140W	RPB4	YNL030W	HHF2
YNR052C	POP2	YDR318W	MCM21
YNL199C	GCR2	YGR270W	YTA7
YBR108W	YBR108W	YLR180W	SAM1
YDL101C	DUN1	YNR052C	POP2
YPL194W	DDC1	YGL100W	SEH1
YKR046C	PET10	YMR152W	YIM1
YDR443C	SSN2	YCR053W	THR4
YLR381W	CTF3	YPR046W	MCM16

Predictions at threshold level 0.80 continued...			
Gene1 of predicted pair		Gene2 of predicted pair	
Systematic Name	Standard Name	Systematic Name	Standard Name
YEL056W	HAT2	YAL002W	VPS8
YDR200C	VPS64	YJR035W	RAD26
YHR200W	RPN10	YMR133W	REC114
YIL076W	SEC28	YER144C	UBP5
YLR398C	SKI2	YMR230W	RPS10B
YML013W	UBX2	YLR032W	RAD5
YOR358W	HAP5	YHR012W	VPS29
YDR129C	SAC6	YDR440W	DOT1
YOR140W	SFL1	YNL236W	SIN4
YLR095C	IOC2	YDR358W	GGA1
YAL024C	LTE1	YJL006C	CTK2
YKL139W	CTK1	YDL002C	NHP10
YIR005W	IST3	YDR318W	MCM21
YLR429W	CRN1	YOL091W	SPO21
YPR051W	MAK3	YHR039C-A	VMA10
YKL101W	HSL1	YDL047W	SIT4
YOL126C	MDH2	YMR125W	STO1
YER144C	UBP5	YGL003C	CDH1
YLR090W	XDJ1	YJL172W	CPS1
YLR234W	TOP3	YKL114C	APN1
YDR477W	SNF1	YDR261C	EXG2
YPL024W	RMI1	YPL018W	CTF19
YLR399C	BDF1	YPL018W	CTF19
YPR046W	MCM16	YDR200C	VPS64
YNL106C	INP52	YOL081W	IRA2
YPL024W	RMI1	YKR035W-A	DID2
YAL024C	LTE1	YNL215W	IES2
YPR131C	NAT3	YBR200W	BEM1
YLR191W	PEX13	YOL044W	PEX15

Predictions at threshold level 0.80 continued...			
Gene1 of predicted pair		Gene2 of predicted pair	
Systematic Name	Standard Name	Systematic Name	Standard Name
YPL042C	SSN3	YCR077C	PAT1
YLR095C	IOC2	YER139C	YER139C
YPL042C	SSN3	YMR198W	CIK1
YKL048C	ELM1	YDR130C	FIN1
YDL185W	TFP1	YLR452C	SST2
YIL008W	URM1	YJR135C	MCM22
YJR032W	CPR7	YER040W	GLN3
YLR452C	SST2	YER162C	RAD4
YKR046C	PET10	YKL140W	TGL1
YDR254W	CHL4	YGR270W	YTA7
YDR383C	NKP1	YOL081W	IRA2
YAR042W	SWH1	YHR195W	NVJ1
YGL163C	RAD54	YEL056W	HAT2
YJR024C	MDE1	YER110C	KAP123
YLR093C	NYV1	YDR358W	GGA1
YPR072W	NOT5	YBR009C	HHF1
YFR030W	MET10	YDR289C	RTT103
YBR200W	BEM1	YNL236W	SIN4
YDR245W	MNN10	YER096W	SHC1
YER110C	KAP123	YDL155W	CLB3
YGL147C	RPL9A	YJR056C	YJR056C
YOR332W	VMA4	YNL238W	KEX2
YDR484W	VPS52	YBR036C	CSG2
YNL087W	TCB2	YLR093C	NYV1
YOR290C	SNF2	YOL081W	IRA2
YDR379W	RGA2	YGL031C	RPL24A
YIL076W	SEC28	YDR245W	MNN10
YPL024W	RMI1	YGL087C	MMS2
YKL126W	YPK1	YML001W	YPT7

Predictions at threshold level 0.80 continued...			
Gene1 of predicted pair		Gene2 of predicted pair	
Systematic Name	Standard Name	Systematic Name	Standard Name
YDR137W	RGP1	YNR072W	HXT17
YHL031C	GOS1	YNL051W	COG5
YDR261C	EXG2	YER162C	RAD4
YGR019W	UGA1	YIL008W	URM1
YML095C	RAD10	YOL090W	MSH2
YBL019W	APN2	YCR066W	RAD18
YPR120C	CLB5	YOR014W	RTS1
YKL025C	PAN3	YOR358W	HAP5
YCR086W	CSM1	YPL018W	CTF19
YLR381W	CTF3	YER110C	KAP123
YLR386W	VAC14	YGL163C	RAD54
YDR383C	NKP1	YAR014C	BUD14
YBR019C	GAL10	YDR310C	SUM1
YLR288C	MEC3	YGL003C	CDH1
YLR006C	SSK1	YCR073C	SSK22
YEL065W	SIT1	YDR484W	VPS52
YBR030W	YBR030W	YFR036W	CDC26
YBR057C	MUM2	YDR363W-A	SEM1
YLR182W	SWI6	YGL252C	RTG2
YJR140C	HIR3	YLR435W	TSR2
YNL248C	RPA49	YDR183W	PLP1
YJL095W	BCK1	YDR207C	UME6
YKL048C	ELM1	YLR398C	SKI2
YBR173C	UMP1	YDR207C	UME6
YHR134W	WSS1	YOL090W	MSH2
YCL001W	RER1	YKR030W	GMH1
YKL116C	PRR1	YDL184C	RPL41A
YER144C	UBP5	YLR398C	SKI2
YJL154C	VPS35	YGL054C	ERV14

Predictions at threshold level 0.80 continued...			
Gene1 of predicted pair		Gene2 of predicted pair	
Systematic Name	Standard Name	Systematic Name	Standard Name
YOL111C	MDY2	YOL081W	IRA2
YKL139W	CTK1	YNL253W	TEX1
YEL009C	GCN4	YDR176W	NGG1
YEL053C	MAK10	YMR311C	GLC8
YGR104C	SRB5	YGR252W	GCN5
YDL155W	CLB3	YKL041W	VPS24
YBL103C	RTG3	YDL077C	VAM6
YNL021W	HDA1	YPR119W	CLB2
YMR026C	PEX12	YDR137W	RGP1
YDR137W	RGP1	YNL238W	KEX2
YPL037C	EGD1	YPL120W	VPS30
YDR080W	VPS41	YAL002W	VPS8
YMR284W	YKU70	YLR410W	VIP1
YCR081W	SRB8	YJL095W	BCK1
YGL174W	BUD13	YCR077C	PAT1
YPL180W	TCO89	YJL183W	MNN11
YBR171W	SEC66	YKR014C	YPT52
YGL223C	COG1	YML071C	COG8
YLR441C	RPS1A	YLR320W	MMS22
YJR005W	APL1	YDR200C	VPS64
YLR447C	VMA6	YPL018W	CTF19
YOR035C	SHE4	YMR230W	RPS10B
YHL031C	GOS1	YJR040W	GEF1
YDR245W	MNN10	YOR375C	GDH1
YHR156C	LIN1	YHR039C-A	VMA10
YHL031C	GOS1	YDR310C	SUM1
YFL023W	BUD27	YML071C	COG8
YNL054W	VAC7	YBR107C	IML3
YJR056C	YJR056C	YML071C	COG8

Predictions at threshold level 0.80 continued...			
Gene1 of predicted pair		Gene2 of predicted pair	
Systematic Name	Standard Name	Systematic Name	Standard Name
YEL065W	SIT1	YDR137W	RGP1
YHR114W	BZZ1	YAR014C	BUD14
YLR435W	TSR2	YMR167W	MLH1
YGL115W	SNF4	YLR398C	SKI2
YKR099W	BAS1	YBR019C	GAL10
YDR378C	LSM6	YGL252C	RTG2
YKL160W	ELF1	YAL002W	VPS8
YMR224C	MRE11	YLR315W	NKP2
YCR077C	PAT1	YKL041W	VPS24
YDL116W	NUP84	YIL036W	CST6
YBR200W	BEM1	YPR120C	CLB5
YDR202C	RAV2	YGL212W	VAM7
YLR119W	SRN2	YOR337W	TEA1
YKR020W	VPS51	YKL041W	VPS24
YER139C	YER139C	YMR311C	GLC8
YKL032C	IXR1	YHR115C	DMA1
YGR252W	GCN5	YBL002W	HTB2
YOR043W	WHI2	YDR358W	GGA1
YNL069C	RPL16B	YCR066W	RAD18
YDL020C	RPN4	YLR386W	VAC14
YMR224C	MRE11	YKR072C	SIS2
YBR171W	SEC66	YBR107C	IML3
YML016C	PPZ1	YOR275C	RIM20
YKL126W	YPK1	YOR066W	MSA1
YKR072C	SIS2	YGL163C	RAD54
YPR007C	REC8	YPR119W	CLB2
YER162C	RAD4	YBR036C	CSG2
YGL135W	RPL1B	YLR018C	POM34
YML016C	PPZ1	YMR055C	BUB2

Predictions at threshold level 0.80 continued...			
Gene1 of predicted pair		Gene2 of predicted pair	
Systematic Name	Standard Name	Systematic Name	Standard Name
YML088W	UFO1	YJL204C	RCY1
YJL004C	SYS1	YNR032W	PPG1
YAL024C	LTE1	YGL151W	NUT1
YJL124C	LSM1	YKL213C	DOA1
YMR116C	ASC1	YGL200C	EMP24
YML071C	COG8	YDL192W	ARF1
YER116C	SLX8	YML013W	UBX2
YOR106W	VAM3	YER120W	SCS2
YGL115W	SNF4	YHR039C-A	VMA10
YMR142C	RPL13B	YJL183W	MNN11
YPL256C	CLN2	YOL081W	IRA2
YMR142C	RPL13B	YNL076W	MKS1
YKL116C	PRR1	YJL095W	BCK1
YMR202W	ERG2	YER044C	ERG28
YMR116C	ASC1	YMR038C	CCS1
YGL178W	MPT5	YDR485C	VPS72
YLR094C	GIS3	YDL149W	ATG9
YHR161C	YAP1801	YLR396C	VPS33
YIL034C	CAP2	YKL204W	EAP1
YHL020C	OPI1	YER162C	RAD4
YDR174W	HMO1	YDR289C	RTT103
YLR018C	POM34	YMR273C	ZDS1
YDR484W	VPS52	YJL204C	RCY1
YMR198W	CIK1	YJR035W	RAD26
YDR080W	VPS41	YPL037C	EGD1
YPL024W	RMI1	YDR261C	EXG2
YBL058W	SHP1	YOL121C	RPS19A
YGR078C	PAC10	YHR056C	RSC30
YPR036W	VMA13	YMR214W	SCJ1

Predictions at threshold level 0.80 continued...			
Gene1 of predicted pair		Gene2 of predicted pair	
Systematic Name	Standard Name	Systematic Name	Standard Name
YMR153W	NUP53	YBR108W	YBR108W
YJR040W	GEF1	YER122C	GLO3
YNL087W	TCB2	YOL111C	MDY2
YPR017C	DSS4	YGR072W	UPF3
YBR126C	TPS1	YBR030W	YBR030W
YDL155W	CLB3	YDR440W	DOT1
YBR107C	IML3	YDR171W	HSP42
YLR337C	VRP1	YOR127W	RGA1
YMR016C	SOK2	YJR084W	CSN12
YJL154C	VPS35	YGL253W	HXK2
YPR046W	MCM16	YJL183W	MNN11
YCR020C-A	MAK31	YAL002W	VPS8
YPL253C	VIK1	YLR254C	NDL1
YNL288W	CAF40	YPL042C	SSN3
YDR382W	RPP2B	YPL220W	RPL1A
YJR135C	MCM22	YAR014C	BUD14
YOR001W	RRP6	YDR137W	RGP1
YDR129C	SAC6	YAL002W	VPS8
YDL116W	NUP84	YCR024C-A	PMP1
YMR142C	RPL13B	YER040W	GLN3
YJL176C	SWI3	YJR084W	CSN12
YJL060W	BNA3	YDL226C	GCS1
YMR055C	BUB2	YFR036W	CDC26
YER120W	SCS2	YLR309C	IMH1
YBR260C	RGD1	YNL090W	RHO2
YBR215W	HPC2	YCL037C	SRO9
YJL029C	VPS53	YDL226C	GCS1
YGR020C	VMA7	YDR310C	SUM1
YBL102W	SFT2	YHR039C-A	VMA10

Predictions at threshold level 0.80 continued...			
**Gene1 of predicted pair**		**Gene2 of predicted pair**	
**Systematic Name**	**Standard Name**	**Systematic Name**	**Standard Name**
YBL078C	ATG8	YNL223W	ATG4
YNL288W	CAF40	YAL002W	VPS8
YEL050C	RML2	YPL106C	SSE1
YDR245W	MNN10	YFL048C	EMP47
YJL154C	VPS35	YPL253C	VIK1
YDR293C	SSD1	YNL166C	BNI5
YNL238W	KEX2	YGL003C	CDH1
YIL159W	BNR1	YDR310C	SUM1
YOL145C	CTR9	YDR440W	DOT1
YJL172W	CPS1	YER162C	RAD4
YDR004W	RAD57	YMR198W	CIK1
YJL154C	VPS35	YNL297C	MON2
YNL031C	HHT2	YDR227W	SIR4
YOR195W	SLK19	YNL307C	MCK1
YPR030W	CSR2	YER139C	YER139C
YKL139W	CTK1	YJR140C	HIR3
YBR171W	SEC66	YML071C	COG8
YPR052C	NHP6A	YBR215W	HPC2
YPL057C	SUR1	YLR371W	ROM2
YER162C	RAD4	YMR152W	YIM1
YLR385C	SWC7	YLR399C	BDF1
YNL215W	IES2	YBR036C	CSG2
YHR060W	VMA22	YKL041W	VPS24
YOR076C	SKI7	YPR189W	SKI3
YIR005W	IST3	YNL116W	DMA2
YKL048C	ELM1	YGL115W	SNF4
YBR107C	IML3	YEL053C	MAK10
YDL160C	DHH1	YJR090C	GRR1
YKR061W	KTR2	YDR335W	MSN5

Predictions at threshold level 0.80 continued...			
Gene1 of predicted pair		Gene2 of predicted pair	
Systematic Name	Standard Name	Systematic Name	Standard Name
YJL172W	CPS1	YLR180W	SAM1
YBR019C	GAL10	YLR370C	ARC18
YNL079C	TPM1	YLR371W	ROM2
YBR200W	BEM1	YCR086W	CSM1
YEL065W	SIT1	YOL081W	IRA2
YDR484W	VPS52	YKL048C	ELM1
YCR020C-A	MAK31	YDL006W	PTC1
YGL133W	ITC1	YGR135W	PRE9
YOR375C	GDH1	YDR440W	DOT1
YKL160W	ELF1	YLR182W	SWI6
YLR337C	VRP1	YOL067C	RTG1
YPR051W	MAK3	YHL002W	HSE1
YDR289C	RTT103	YLR350W	ORM2
YCR092C	MSH3	YOL090W	MSH2
YKL006W	RPL14A	YGR135W	PRE9
YGL212W	VAM7	YKR048C	NAP1
YKL139W	CTK1	YOR270C	VPH1
YJR060W	CBF1	YLR309C	IMH1
YMR055C	BUB2	YDR260C	SWM1
YOR275C	RIM20	YBL087C	RPL23A
YBR126C	TPS1	YMR224C	MRE11
YGL003C	CDH1	YDL047W	SIT4
YPL105C	YPL105C	YPL120W	VPS30
YLR234W	TOP3	YBR057C	MUM2
YNL243W	SLA2	YLR337C	VRP1
YML115C	VAN1	YOR275C	RIM20
YDR363W-A	SEM1	YLR421C	RPN13
YDR358W	GGA1	YMR311C	GLC8
YDR507C	GIN4	YHR061C	GIC1

Predictions at threshold level 0.80 continued...			
Gene1 of predicted pair		Gene2 of predicted pair	
Systematic Name	Standard Name	Systematic Name	Standard Name
YKL025C	PAN3	YGL253W	HXK2
YPR018W	RLF2	YDL059C	RAD59
YOR290C	SNF2	YKL160W	ELF1
YMR273C	ZDS1	YKR024C	DBP7
YMR004W	MVP1	YNL107W	YAF9
YDR477W	SNF1	YOL081W	IRA2
YMR080C	NAM7	YHR134W	WSS1
YLR452C	SST2	YJR084W	CSN12
YML013W	UBX2	YMR055C	BUB2
YOR358W	HAP5	YBL079W	NUP170
YNL199C	GCR2	YNL107W	YAF9
YPL161C	BEM4	YHR061C	GIC1
YKL139W	CTK1	YDR254W	CHL4
YOL111C	MDY2	YER040W	GLN3
YDR320C	SWA2	YDR310C	SUM1
YPL022W	RAD1	YNL016W	PUB1
YJL176C	SWI3	YGL153W	PEX14
YBR171W	SEC66	YHR111W	UBA4
YDR320C	SWA2	YMR004W	MVP1
YFR010W	UBP6	YPL195W	APL5
YML001W	YPT7	YLR093C	NYV1
YKL116C	PRR1	YPR030W	CSR2
YGL078C	DBP3	YMR153W	NUP53
YKR099W	BAS1	YHR039C-A	VMA10
YKR001C	VPS1	YOR312C	RPL20B
YHR060W	VMA22	YPR046W	MCM16
YKL006W	RPL14A	YPL161C	BEM4
YOR127W	RGA1	YHR060W	VMA22
YGL174W	BUD13	YOR043W	WHI2

Predictions at threshold level 0.80 continued...			
Gene1 of predicted pair		Gene2 of predicted pair	
Systematic Name	Standard Name	Systematic Name	Standard Name
YPL259C	APM1	YOR327C	SNC2
YHR098C	SFB3	YPR135W	CTF4
YNL058C	YNL058C	YHR081W	LRP1
YGL038C	OCH1	YMR052W	FAR3
YDL020C	RPN4	YHR039C-A	VMA10
YPL240C	HSP82	YDL246C	SOR2
YMR202W	ERG2	YJL187C	SWE1
YIL034C	CAP2	YPR046W	MCM16
YGL078C	DBP3	YMR016C	SOK2
YMR038C	CCS1	YER122C	GLO3
YKL139W	CTK1	YMR038C	CCS1
YNL199C	GCR2	YDR289C	RTT103
YLR429W	CRN1	YOR141C	ARP8
YEL044W	IES6	YOR035C	SHE4
YKR099W	BAS1	YMR152W	YIM1
YHL031C	GOS1	YGL212W	VAM7
YOR106W	VAM3	YKR020W	VPS51
YLR083C	EMP70	YNL107W	YAF9
YOR023C	AHC1	YHR111W	UBA4
YLR360W	VPS38	YCR086W	CSM1
YJR060W	CBF1	YEL051W	VMA8
YPL119C	DBP1	YPR119W	CLB2
YLR423C	ATG17	YNL090W	RHO2
YIL133C	RPL16A	YGL252C	RTG2
YMR198W	CIK1	YPL018W	CTF19
YGR252W	GCN5	YGL252C	RTG2
YHR200W	RPN10	YML097C	VPS9
YPL024W	RMI1	YDL192W	ARF1
YMR142C	RPL13B	YPL137C	GIP3

Predictions at threshold level 0.80 continued...			
Gene1 of predicted pair		Gene2 of predicted pair	
Systematic Name	Standard Name	Systematic Name	Standard Name
YGL174W	BUD13	YDL002C	NHP10
YIL034C	CAP2	YOL067C	RTG1
YOR109W	INP53	YPL105C	YPL105C
YDR507C	GIN4	YJL187C	SWE1
YML016C	PPZ1	YLR425W	TUS1
YOL145C	CTR9	YJL140W	RPB4
YJL154C	VPS35	YDR080W	VPS41
YBR200W	BEM1	YKL080W	VMA5
YDR103W	STE5	YOR212W	STE4
YDR363W-A	SEM1	YGL163C	RAD54
YPL037C	EGD1	YNL041C	COG6
YPL024W	RMI1	YFL023W	BUD27
YMR152W	YIM1	YJR084W	CSN12
YLR094C	GIS3	YPL195W	APL5
YIL008W	URM1	YGL253W	HXK2
YJR043C	POL32	YGL100W	SEH1
YDR137W	RGP1	YGL025C	PGD1
YLR386W	VAC14	YBL087C	RPL23A
YMR055C	BUB2	YGR054W	YGR054W
YJL117W	PHO86	YLR371W	ROM2
YLR083C	EMP70	YGR261C	APL6
YBR200W	BEM1	YDL047W	SIT4
YDR192C	NUP42	YGR081C	SLX9
YIL034C	CAP2	YOR132W	VPS17
YFR032C-A	RPL29	YKL048C	ELM1
YLR371W	ROM2	YLR093C	NYV1
YBR084C-A	RPL19A	YHR152W	SPO12
YBR030W	YBR030W	YLR398C	SKI2
YGR249W	MGA1	YGL003C	CDH1

Predictions at threshold level 0.80 continued...			
Gene1 of predicted pair		Gene2 of predicted pair	
Systematic Name	Standard Name	Systematic Name	Standard Name
YGL066W	SGF73	YGR135W	PRE9
YER107C	GLE2	YML041C	VPS71
YLR119W	SRN2	YLR371W	ROM2
YMR224C	MRE11	YLR032W	RAD5
YJL124C	LSM1	YFR036W	CDC26
YNR072W	HXT17	YFR036W	CDC26
YNL068C	FKH2	YKR057W	RPS21A
YPR141C	KAR3	YGL190C	CDC55
YCR081W	SRB8	YLR425W	TUS1
YLR360W	VPS38	YJL029C	VPS53
YGR217W	CCH1	YMR205C	PFK2
YHR156C	LIN1	YNL058C	YNL058C
YLR234W	TOP3	YHR013C	ARD1
YLR410W	VIP1	YHR108W	GGA2
YLR240W	VPS34	YJL029C	VPS53
YBR171W	SEC66	YDL056W	MBP1
YAL024C	LTE1	YHL022C	SPO11
YDR335W	MSN5	YIL103W	DPH1
YNL069C	RPL16B	YER162C	RAD4
YBL058W	SHP1	YNL058C	YNL058C
YNL325C	FIG4	YOR327C	SNC2
YDR383C	NKP1	YLR381W	CTF3
YLR079W	SIC1	YDR378C	LSM6
YHR060W	VMA22	YGL115W	SNF4
YPL042C	SSN3	YBR215W	HPC2
YER116C	SLX8	YEL060C	PRB1
YJL187C	SWE1	YML028W	TSA1
YNL307C	MCK1	YGL124C	MON1
YDR261C	EXG2	YBR280C	SAF1

Predictions at threshold level 0.80 continued...			
**Gene1 of predicted pair**		**Gene2 of predicted pair**	
**Systematic Name**	**Standard Name**	**Systematic Name**	**Standard Name**
YPL152W	RRD2	YDR318W	MCM21
YGR217W	CCH1	YPL089C	RLM1
YBR107C	IML3	YDR155C	CPR1
YLR386W	VAC14	YHR013C	ARD1
YLR090W	XDJ1	YMR214W	SCJ1
YER110C	KAP123	YGR059W	SPR3
YMR025W	CSI1	YJR084W	CSN12
YLR313C	SPH1	YLR410W	VIP1
YKR001C	VPS1	YER162C	RAD4
YDR477W	SNF1	YPR046W	MCM16
YLR337C	VRP1	YDR183W	PLP1
YDR245W	MNN10	YCR077C	PAT1
YPL213W	LEA1	YCR077C	PAT1
YHL031C	GOS1	YMR004W	MVP1
YNR072W	HXT17	YNL238W	KEX2
YDR477W	SNF1	YMR153W	NUP53
YHR012W	VPS29	YAR014C	BUD14
YIL076W	SEC28	YMR004W	MVP1
YBL071W-A	KTI11	YPL101W	ELP4
YGR270W	YTA7	YGL252C	RTG2
YMR055C	BUB2	YHR013C	ARD1
YGR135W	PRE9	YPL256C	CLN2
YMR026C	PEX12	YIL076W	SEC28
YOL051W	GAL11	YLR357W	RSC2
YGR019W	UGA1	YBL051C	PIN4
YLR410W	VIP1	YAR014C	BUD14
YLR399C	BDF1	YFR034C	PHO4
YBL047C	EDE1	YKL048C	ELM1
YML115C	VAN1	YMR199W	CLN1

Predictions at threshold level 0.80 continued...			
Gene1 of predicted pair		Gene2 of predicted pair	
Systematic Name	Standard Name	Systematic Name	Standard Name
YIR003W	YIR003W	YPL050C	MNN9
YHR081W	LRP1	YER040W	GLN3
YDL226C	GCS1	YOR036W	PEP12
YFR031C-A	RPL2A	YNL107W	YAF9
YKL068W	NUP100	YJR090C	GRR1
YJR090C	GRR1	YNL107W	YAF9
YDR254W	CHL4	YJR035W	RAD26
YOR039W	CKB2	YJL187C	SWE1
YPR029C	APL4	YKR001C	VPS1
YER116C	SLX8	YOR066W	MSA1
YBR107C	IML3	YDR310C	SUM1
YPR036W	VMA13	YNL076W	MKS1
YIL053W	RHR2	YGR249W	MGA1
YGL078C	DBP3	YDR207C	UME6
YMR116C	ASC1	YKL139W	CTK1
YDR156W	RPA14	YHR200W	RPN10
YDR137W	RGP1	YOL018C	TLG2
YOR106W	VAM3	YML071C	COG8
YMR109W	MYO5	YDR289C	RTT103
YAL024C	LTE1	YDL083C	RPS16B
YNL032W	SIW14	YOR270C	VPH1
YBR194W	SOY1	YER122C	GLO3
YOR230W	WTM1	YOR229W	WTM2
YPR046W	MCM16	YNL076W	MKS1
YBL102W	SFT2	YMR004W	MVP1
YGR173W	RBG2	YDR152W	GIR2
YLR381W	CTF3	YGR054W	YGR054W
YNL238W	KEX2	YMR311C	GLC8
YNL069C	RPL16B	YCL001W	RER1

Predictions at threshold level 0.80 continued...			
Gene1 of predicted pair		Gene2 of predicted pair	
Systematic Name	Standard Name	Systematic Name	Standard Name
YDR245W	MNN10	YOR124C	UBP2
YLR288C	MEC3	YHR010W	RPL27A
YHR200W	RPN10	YEL060C	PRB1
YJL172W	CPS1	YCR053W	THR4
YML115C	VAN1	YMR153W	NUP53
YLL007C	YLL007C	YGL253W	HXK2
YPL022W	RAD1	YPL256C	CLN2
YCL001W	RER1	YPL234C	TFP3
YIL053W	RHR2	YBR126C	TPS1
YML117W	NAB6	YGR135W	PRE9
YKR099W	BAS1	YLR094C	GIS3
YNL079C	TPM1	YDR440W	DOT1
YJL172W	CPS1	YFL023W	BUD27
YDR137W	RGP1	YLR371W	ROM2
YGL212W	VAM7	YDR207C	UME6
YBR290W	BSD2	YCR086W	CSM1
YOR109W	INP53	YNL238W	KEX2
YLL039C	UBI4	YBL072C	RPS8A
YBL058W	SHP1	YLR386W	VAC14
YBR274W	CHK1	YKL114C	APN1
YJR035W	RAD26	YKL054C	DEF1
YPL213W	LEA1	YLR425W	TUS1
YNL041C	COG6	YDR358W	GGA1
YMR026C	PEX12	YGL124C	MON1
YEL050C	RML2	YJR135C	MCM22
YKL139W	CTK1	YDL077C	VAM6
YPR120C	CLB5	YCR077C	PAT1
YKL139W	CTK1	YGL252C	RTG2
YLR090W	XDJ1	YER040W	GLN3

Predictions at threshold level 0.80 continued...			
Gene1 of predicted pair		Gene2 of predicted pair	
Systematic Name	Standard Name	Systematic Name	Standard Name
YEL053C	MAK10	YBR019C	GAL10
YGR072W	UPF3	YDL006W	PTC1
YER116C	SLX8	YEL053C	MAK10
YBL103C	RTG3	YDL192W	ARF1
YBL008W	HIR1	YLR350W	ORM2
YEL013W	VAC8	YPR185W	ATG13
YLR320W	MMS22	YDL006W	PTC1
YLR386W	VAC14	YPL253C	VIK1
YNL058C	YNL058C	YDL077C	VAM6
YDR477W	SNF1	YBL103C	RTG3
YHR039C-A	VMA10	YOR066W	MSA1
YHR167W	THP2	YLR350W	ORM2
YNL215W	IES2	YFR030W	MET10
YOL044W	PEX15	YNL214W	PEX17
YBR200W	BEM1	YML112W	CTK3
YGL163C	RAD54	YIR005W	IST3
YFR010W	UBP6	YKL139W	CTK1
YDR477W	SNF1	YMR152W	YIM1
YBR108W	YBR108W	YBL103C	RTG3
YPR036W	VMA13	YER005W	YND1
YDR293C	SSD1	YEL056W	HAT2
YGR270W	YTA7	YOL054W	PSH1
YCR066W	RAD18	YHL020C	OPI1
YMR109W	MYO5	YDR155C	CPR1
YPL057C	SUR1	YNL076W	MKS1
YOR080W	DIA2	YPR119W	CLB2
YMR142C	RPL13B	YOR270C	VPH1
YBR215W	HPC2	YOL081W	IRA2
YBL021C	HAP3	YDR310C	SUM1

Predictions at threshold level 0.80 continued...			
Gene1 of predicted pair		Gene2 of predicted pair	
Systematic Name	Standard Name	Systematic Name	Standard Name
YMR199W	CLN1	YDR379W	RGA2
YGR081C	SLX9	YBR215W	HPC2
YBR030W	YBR030W	YNL238W	KEX2
YGL025C	PGD1	YML001W	YPT7
YML016C	PPZ1	YGL003C	CDH1
YLR447C	VMA6	YAL002W	VPS8
YOR106W	VAM3	YDR469W	SDC1
YBR215W	HPC2	YDR171W	HSP42
YAL024C	LTE1	YMR198W	CIK1
YJL004C	SYS1	YJR033C	RAV1
YCR088W	ABP1	YKL213C	DOA1
YDR484W	VPS52	YJL029C	VPS53
YNL206C	RTT106	YBR107C	IML3
YOR096W	RPS7A	YLR441C	RPS1A
YPL050C	MNN9	YPL112C	PEX25
YLL007C	YLL007C	YHR111W	UBA4
YNL025C	SSN8	YKL139W	CTK1
YKR101W	SIR1	YBR010W	HHT1
YDR129C	SAC6	YJR084W	CSN12
YNL069C	RPL16B	YLR309C	IMH1
YML016C	PPZ1	YDR183W	PLP1
YFL047W	RGD2	YGL016W	KAP122
YFR036W	CDC26	YOR368W	RAD17
YHR013C	ARD1	YDR289C	RTT103
YNL058C	YNL058C	YPR046W	MCM16
YBR057C	MUM2	YGR135W	PRE9
YPL037C	EGD1	YHR012W	VPS29
YHR134W	WSS1	YOR023C	AHC1
YLR083C	EMP70	YHR060W	VMA22

Predictions at threshold level 0.80 continued...			
Gene1 of predicted pair		Gene2 of predicted pair	
Systematic Name	Standard Name	Systematic Name	Standard Name
YDR363W-A	SEM1	YPL161C	BEM4
YNL297C	MON2	YHL020C	OPI1
YDR156W	RPA14	YPL018W	CTF19
YML038C	YMD8	YER120W	SCS2
YLR109W	AHP1	YOL151W	GRE2
YGR270W	YTA7	YDR310C	SUM1
YLR447C	VMA6	YOL067C	RTG1
YGL237C	HAP2	YNL107W	YAF9
YKR099W	BAS1	YMR214W	SCJ1
YOR083W	WHI5	YBR194W	SOY1
YGR261C	APL6	YDL006W	PTC1
YEL051W	VMA8	YNL107W	YAF9
YBR084C-A	RPL19A	YHR111W	UBA4
YHR200W	RPN10	YDL047W	SIT4
YER144C	UBP5	YDL077C	VAM6
YGR159C	NSR1	YLR410W	VIP1
YFR010W	UBP6	YDR484W	VPS52
YCL037C	SRO9	YML038C	YMD8
YML016C	PPZ1	YPR120C	CLB5
YPL042C	SSN3	YKL160W	ELF1
YGR108W	CLB1	YDR289C	RTT103
YLR148W	PEP3	YMR231W	PEP5
YNL041C	COG6	YER040W	GLN3
YMR224C	MRE11	YLR182W	SWI6
YNL127W	FAR11	YAL002W	VPS8
YCR086W	CSM1	YDR183W	PLP1
YJL053W	PEP8	YHR161C	YAP1801
YNL032W	SIW14	YLR180W	SAM1
YGR020C	VMA7	YER122C	GLO3

Predictions at threshold level 0.80 continued...			
Gene1 of predicted pair		Gene2 of predicted pair	
Systematic Name	Standard Name	Systematic Name	Standard Name
YPR030W	CSR2	YCR053W	THR4
YNL106C	INP52	YHR111W	UBA4
YLR447C	VMA6	YKL080W	VMA5
YOR132W	VPS17	YNL127W	FAR11
YOR337W	TEA1	YKL126W	YPK1
YDR378C	LSM6	YOL091W	SPO21
YHR111W	UBA4	YBL087C	RPL23A
YMR224C	MRE11	YPL195W	APL5
YGL078C	DBP3	YLR452C	SST2
YNL076W	MKS1	YDR310C	SUM1
YNL032W	SIW14	YNL041C	COG6
YIR025W	MND2	YFR036W	CDC26
YAL024C	LTE1	YBR278W	DPB3
YHR156C	LIN1	YER116C	SLX8
YDL006W	PTC1	YPL089C	RLM1
YOR195W	SLK19	YOR127W	RGA1
YGL006W	PMC1	YBL078C	ATG8
YNL041C	COG6	YEL036C	ANP1
YDR176W	NGG1	YCL010C	SGF29
YIL159W	BNR1	YLR425W	TUS1
YGL250W	RMR1	YKL101W	HSL1
YKR095W	MLP1	YBL079W	NUP170
YNL325C	FIG4	YKL126W	YPK1
YNL058C	YNL058C	YNL238W	KEX2
YER144C	UBP5	YPL161C	BEM4
YDR310C	SUM1	YOR014W	RTS1
YDR004W	RAD57	YKR072C	SIS2
YDR484W	VPS52	YNL058C	YNL058C
YMR199W	CLN1	YDR076W	RAD55

Predictions at threshold level 0.80 continued...			
Gene1 of predicted pair		Gene2 of predicted pair	
Systematic Name	Standard Name	Systematic Name	Standard Name
YGL178W	MPT5	YPL101W	ELP4
YLR396C	VPS33	YAL002W	VPS8
YDR484W	VPS52	YLR370C	ARC18
YGL066W	SGF73	YPL248C	GAL4
YNL117W	MLS1	YLR032W	RAD5
YGR108W	CLB1	YBR200W	BEM1
YER116C	SLX8	YLR370C	ARC18
YLR170C	APS1	YOR132W	VPS17
YBR194W	SOY1	YOR124C	UBP2
YLR119W	SRN2	YDL083C	RPS16B
YMR026C	PEX12	YKL048C	ELM1
YMR216C	SKY1	YCR066W	RAD18
YDL155W	CLB3	YGL253W	HXK2
YPR017C	DSS4	YOR112W	CEX1
YMR284W	YKU70	YLR135W	SLX4
YML038C	YMD8	YML001W	YPT7
YPL256C	CLN2	YLR425W	TUS1
YHR157W	REC104	YER044C-A	MEI4
YLR043C	TRX1	YDR080W	VPS41
YOR270C	VPH1	YHR200W	RPN10
YPL037C	EGD1	YEL060C	PRB1
YNR052C	POP2	YBR089C-A	NHP6B
YCR086W	CSM1	YHR114W	BZZ1
YPL018W	CTF19	YGL151W	NUT1
YJL154C	VPS35	YER122C	GLO3
YHR205W	SCH9	YPL253C	VIK1
YGR270W	YTA7	YGR054W	YGR054W
YNL106C	INP52	YOR195W	SLK19
YPL253C	VIK1	YHR200W	RPN10

Predictions at threshold level 0.80 continued...			
Gene1 of predicted pair		Gene2 of predicted pair	
Systematic Name	Standard Name	Systematic Name	Standard Name
YDR146C	SWI5	YBL047C	EDE1
YDL083C	RPS16B	YML071C	COG8
YBR200W	BEM1	YJR035W	RAD26
YFR031C-A	RPL2A	YMR230W	RPS10B
YDL239C	ADY3	YNL225C	CNM67
YBR200W	BEM1	YER040W	GLN3
YMR224C	MRE11	YMR142C	RPL13B
YBR278W	DPB3	YOR043W	WHI2
YJL029C	VPS53	YER143W	DDI1
YGL066W	SGF73	YKL160W	ELF1
YML115C	VAN1	YLR398C	SKI2
YGR089W	NNF2	YHL010C	YHL010C
YNL264C	PDR17	YLR398C	SKI2
YHR156C	LIN1	YGL025C	PGD1
YGL025C	PGD1	YLR398C	SKI2
YHR073W	OSH3	YOL126C	MDH2
YBR108W	YBR108W	YLR452C	SST2
YIL159W	BNR1	YPL161C	BEM4
YLR095C	IOC2	YLR119W	SRN2
YML062C	MFT1	YNL004W	HRB1
YHR200W	RPN10	YPL256C	CLN2
YGR261C	APL6	YGL115W	SNF4
YML095C	RAD10	YGL163C	RAD54
YOR270C	VPH1	YNL041C	COG6
YML016C	PPZ1	YFR030W	MET10
YOR035C	SHE4	YML097C	VPS9
YDR443C	SSN2	YLR192C	HCR1
YBR215W	HPC2	YBR107C	IML3
YDL127W	PCL2	YEL056W	HAT2

Predictions at threshold level 0.80 continued...			
Gene1 of predicted pair		Gene2 of predicted pair	
Systematic Name	Standard Name	Systematic Name	Standard Name
YNL297C	MON2	YAR014C	BUD14
YBR215W	HPC2	YDR378C	LSM6
YGL066W	SGF73	YPR120C	CLB5
YPR046W	MCM16	YOL091W	SPO21
YDL160C	DHH1	YMR125W	STO1
YAL024C	LTE1	YCR086W	CSM1
YOR212W	STE4	YCL032W	STE50
YER144C	UBP5	YDL192W	ARF1
YIL076W	SEC28	YAL002W	VPS8
YLR447C	VMA6	YKL041W	VPS24
YER107C	GLE2	YBR231C	SWC5
YNL068C	FKH2	YLR386W	VAC14
YKL110C	KTI12	YLR254C	NDL1
YGR020C	VMA7	YHL020C	OPI1
YNL218W	MGS1	YLR234W	TOP3
YDR484W	VPS52	YDR137W	RGP1
YOR043W	WHI2	YLR425W	TUS1
YML117W	NAB6	YJL095W	BCK1
YHR060W	VMA22	YHR039C-A	VMA10
YPL096C-A	ERI1	YJL148W	RPA34
YBR200W	BEM1	YNL225C	CNM67
YDR484W	VPS52	YMR152W	YIM1
YKR020W	VPS51	YDL006W	PTC1
YDR146C	SWI5	YDL006W	PTC1
YAL024C	LTE1	YBL103C	RTG3
YGR249W	MGA1	YBL103C	RTG3
YBR108W	YBR108W	YOR270C	VPH1
YLR381W	CTF3	YPR119W	CLB2
YJR024C	MDE1	YMR153W	NUP53

Predictions at threshold level 0.80 continued...			
Gene1 of predicted pair		Gene2 of predicted pair	
Systematic Name	Standard Name	Systematic Name	Standard Name
YDR318W	MCM21	YJR035W	RAD26
YLR119W	SRN2	YLR398C	SKI2
YCR081W	SRB8	YDR146C	SWI5
YMR172W	HOT1	YIL008W	URM1
YPL042C	SSN3	YNR052C	POP2
YNL058C	YNL058C	YDL192W	ARF1
YGR241C	YAP1802	YFL026W	STE2
YGL095C	VPS45	YOR035C	SHE4
YDR004W	RAD57	YER162C	RAD4
YHR178W	STB5	YMR127C	SAS2
YDR004W	RAD57	YOL090W	MSH2
YBR111W-A	SUS1	YBR009C	HHF1
YGL078C	DBP3	YPL161C	BEM4
YBR131W	CCZ1	YNL051W	COG5
YOR106W	VAM3	YDL077C	VAM6
YBR200W	BEM1	YFR030W	MET10
YHR134W	WSS1	YHR178W	STB5
YMR055C	BUB2	YGR188C	BUB1
YJL124C	LSM1	YPR120C	CLB5
YCR081W	SRB8	YOL051W	GAL11
YDR080W	VPS41	YKR030W	GMH1
YCL037C	SRO9	YPR032W	SRO7
YMR216C	SKY1	YNR032W	PPG1
YNR072W	HXT17	YJR084W	CSN12
YPL037C	EGD1	YDR252W	BTT1
YJR084W	CSN12	YEL060C	PRB1
YDR507C	GIN4	YJL095W	BCK1
YNL041C	COG6	YNL076W	MKS1
YDR293C	SSD1	YJL053W	PEP8

Predictions at threshold level 0.80 continued...			
Gene1 of predicted pair		Gene2 of predicted pair	
Systematic Name	Standard Name	Systematic Name	Standard Name
YKL025C	PAN3	YOR127W	RGA1
YPR017C	DSS4	YML071C	COG8
YER016W	BIM1	YBR089C-A	NHP6B
YPL042C	SSN3	YMR038C	CCS1
YDR202C	RAV2	YJR032W	CPR7
YHR013C	ARD1	YJR084W	CSN12
YGR239C	PEX21	YDR142C	PEX7
YLR254C	NDL1	YOR141C	ARP8
YNL206C	RTT106	YMR199W	CLN1
YDL047W	SIT4	YLR182W	SWI6
YCR020C-A	MAK31	YNL297C	MON2
YJR032W	CPR7	YBR114W	RAD16
YLR381W	CTF3	YJL006C	CTK2
YEL065W	SIT1	YLR180W	SAM1
YKL139W	CTK1	YNL054W	VAC7
YEL043W	YEL043W	YGR105W	VMA21
YGR072W	UPF3	YOR109W	INP53
YOR023C	AHC1	YEL053C	MAK10
YBL079W	NUP170	YGL163C	RAD54
YNL230C	ELA1	YLR219W	MSC3
YKL048C	ELM1	YLR094C	GIS3
YHL031C	GOS1	YHL020C	OPI1
YBR112C	CYC8	YGL025C	PGD1
YBR200W	BEM1	YOR290C	SNF2
YKR072C	SIS2	YHR178W	STB5
YJL138C	TIF2	YPR119W	CLB2
YPL022W	RAD1	YGR135W	PRE9
YDR293C	SSD1	YPL195W	APL5
YNL307C	MCK1	YLR180W	SAM1

Predictions at threshold level 0.80 continued...			
Gene1 of predicted pair		Gene2 of predicted pair	
Systematic Name	Standard Name	Systematic Name	Standard Name
YLR381W	CTF3	YHR114W	BZZ1
YKR020W	VPS51	YBL025W	RRN10
YGL237C	HAP2	YDR440W	DOT1
YLR360W	VPS38	YHR012W	VPS29
YCL001W	RER1	YGL115W	SNF4
YDL184C	RPL41A	YLR094C	GIS3
YDR309C	GIC2	YLR425W	TUS1
YMR095C	SNO1	YBR171W	SEC66
YCR034W	FEN1	YJL138C	TIF2
YDR293C	SSD1	YBR171W	SEC66
YLR094C	GIS3	YLR320W	MMS22
YLR371W	ROM2	YML071C	COG8
YKL048C	ELM1	YBL101C	ECM21
YFR031C-A	RPL2A	YLR360W	VPS38
YIL036W	CST6	YJL183W	MNN11
YDL020C	RPN4	YGR020C	VMA7
YIL053W	RHR2	YML001W	YPT7
YDL101C	DUN1	YOR080W	DIA2
YBR084C-A	RPL19A	YIL008W	URM1
YIL036W	CST6	YJR060W	CBF1
YER087C-B	SBH1	YLR418C	CDC73
YBL008W	HIR1	YBR200W	BEM1
YBL008W	HIR1	YOR368W	RAD17
YML103C	NUP188	YOR195W	SLK19
YLR396C	VPS33	YGL095C	VPS45
YML006C	GIS4	YGL115W	SNF4
YLR090W	XDJ1	YMR153W	NUP53
YPR030W	CSR2	YLR370C	ARC18
YOR112W	CEX1	YML001W	YPT7

Predictions at threshold level 0.80 continued...			
Gene1 of predicted pair		Gene2 of predicted pair	
Systematic Name	Standard Name	Systematic Name	Standard Name
YPR036W	VMA13	YER122C	GLO3
YEL065W	SIT1	YOR043W	WHI2
YBL102W	SFT2	YDR310C	SUM1
YOR195W	SLK19	YNL206C	RTT106
YLR288C	MEC3	YCR031C	RPS14A
YJL154C	VPS35	YLR292C	SEC72
YNL206C	RTT106	YIL008W	URM1
YLR061W	RPL22A	YHR013C	ARD1
YDR507C	GIN4	YNL116W	DMA2
YLR238W	FAR10	YMR052W	FAR3
YDR507C	GIN4	YNL078W	NIS1
YJR140C	HIR3	YOR243C	PUS7
YGR261C	APL6	YJL036W	SNX4
YDL013W	SLX5	YPR046W	MCM16
YMR055C	BUB2	YJR032W	CPR7
YPL161C	BEM4	YLR180W	SAM1
YMR080C	NAM7	YPL079W	RPL21B
YNL307C	MCK1	YBL025W	RRN10
YFR036W	CDC26	YLR180W	SAM1
YKL025C	PAN3	YBR107C	IML3
YNL069C	RPL16B	YNR032W	PPG1
YDR484W	VPS52	YLR452C	SST2
YDL223C	HBT1	YOL121C	RPS19A
YML103C	NUP188	YFL047W	RGD2
YIR005W	IST3	YAR014C	BUD14
YBR107C	IML3	YPL018W	CTF19
YJL127C	SPT10	YNL107W	YAF9
YNL325C	FIG4	YCR053W	THR4
YMR026C	PEX12	YFL023W	BUD27

Predictions at threshold level 0.80 continued...			
Gene1 of predicted pair		Gene2 of predicted pair	
Systematic Name	Standard Name	Systematic Name	Standard Name
YML016C	PPZ1	YLR182W	SWI6
YMR095C	SNO1	YDR207C	UME6
YLR425W	TUS1	YEL036C	ANP1
YHL020C	OPI1	YER122C	GLO3
YML115C	VAN1	YER049W	TPA1
YDR174W	HMO1	YPR120C	CLB5
YPR120C	CLB5	YOL054W	PSH1
YBL103C	RTG3	YGL115W	SNF4
YHR134W	WSS1	YMR055C	BUB2
YBL058W	SHP1	YLR094C	GIS3
YHR134W	WSS1	YMR127C	SAS2
YML067C	ERV41	YKR014C	YPT52
YFR010W	UBP6	YMR198W	CIK1
YDL042C	SIR2	YGR252W	GCN5
YMR026C	PEX12	YLR191W	PEX13
YLR288C	MEC3	YGL019W	CKB1
YNL054W	VAC7	YBR200W	BEM1
YDL116W	NUP84	YLR429W	CRN1
YLR337C	VRP1	YOR332W	VMA4
YDR207C	UME6	YMR230W	RPS10B
YKL126W	YPK1	YBR036C	CSG2
YDR477W	SNF1	YER144C	UBP5
YFR032C-A	RPL29	YML115C	VAN1
YMR142C	RPL13B	YLR398C	SKI2
YDR289C	RTT103	YNL147W	LSM7
YIL034C	CAP2	YJR135C	MCM22
YNL264C	PDR17	YER118C	SHO1
YDL223C	HBT1	YMR311C	GLC8
YEL065W	SIT1	YKL139W	CTK1

Predictions at threshold level 0.80 continued...			
Gene1 of predicted pair		Gene2 of predicted pair	
Systematic Name	Standard Name	Systematic Name	Standard Name
YBR107C	IML3	YMR038C	CCS1
YPL234C	TFP3	YHR039C-A	VMA10
YJR056C	YJR056C	YLR452C	SST2
YBR217W	ATG12	YLR398C	SKI2
YPL161C	BEM4	YKL041W	VPS24
YKL025C	PAN3	YBR171W	SEC66
YHR013C	ARD1	YLR306W	UBC12
YDR443C	SSN2	YGL025C	PGD1
YHR114W	BZZ1	YGR188C	BUB1
YAL024C	LTE1	YOR043W	WHI2
YPL240C	HSP82	YLR419W	YLR419W
YKL032C	IXR1	YGL031C	RPL24A
YNL215W	IES2	YLR234W	TOP3
YNL243W	SLA2	YLR206W	ENT2
YLR337C	VRP1	YJL095W	BCK1
YMR172W	HOT1	YLR386W	VAC14
YDR202C	RAV2	YHR039C-A	VMA10
YIL076W	SEC28	YJR056C	YJR056C
YFR032C-A	RPL29	YJL183W	MNN11
YHR079C	IRE1	YHR098C	SFB3
YDR507C	GIN4	YJL157C	FAR1
YER001W	MNN1	YEL036C	ANP1
YLR254C	NDL1	YDR183W	PLP1
YKL007W	CAP1	YNL307C	MCK1
YCR077C	PAT1	YJL095W	BCK1
YCR066W	RAD18	YNL051W	COG5
YAL024C	LTE1	YLR216C	CPR6
YMR224C	MRE11	YFL023W	BUD27
YNL025C	SSN8	YIL076W	SEC28

Predictions at threshold level 0.80 continued...			
Gene1 of predicted pair		Gene2 of predicted pair	
Systematic Name	Standard Name	Systematic Name	Standard Name
YOR195W	SLK19	YJR063W	RPA12
YLR386W	VAC14	YNL325C	FIG4
YPL256C	CLN2	YDR310C	SUM1
YNL243W	SLA2	YMR022W	UBC7
YLL007C	YLL007C	YDR318W	MCM21
YKR061W	KTR2	YLR410W	VIP1
YKL139W	CTK1	YKL041W	VPS24
YML115C	VAN1	YKL126W	YPK1
YKL160W	ELF1	YIR005W	IST3
YGR108W	CLB1	YLR079W	SIC1
YMR224C	MRE11	YFR030W	MET10
YPR046W	MCM16	YJL095W	BCK1
YOR132W	VPS17	YLR386W	VAC14
YOL111C	MDY2	YMR152W	YIM1
YJL058C	BIT61	YDR254W	CHL4
YHL031C	GOS1	YEL051W	VMA8
YBR108W	YBR108W	YOL081W	IRA2
YMR004W	MVP1	YGL212W	VAM7
YNL166C	BNI5	YDR310C	SUM1
YHR010W	RPL27A	YJR090C	GRR1
YLL039C	UBI4	YMR276W	DSK2
YMR039C	SUB1	YOL145C	CTR9
YCR077C	PAT1	YCR086W	CSM1
YOR290C	SNF2	YNL058C	YNL058C
YBR215W	HPC2	YOR368W	RAD17
YHR052W	CIC1	YGL189C	RPS26A
YPR018W	RLF2	YDL042C	SIR2
YGL237C	HAP2	YPR046W	MCM16
YBR215W	HPC2	YDR076W	RAD55

Predictions at threshold level 0.80 continued...			
Gene1 of predicted pair		Gene2 of predicted pair	
Systematic Name	Standard Name	Systematic Name	Standard Name
YMR039C	SUB1	YJL124C	LSM1
YKL048C	ELM1	YBR019C	GAL10
YHR013C	ARD1	YGR135W	PRE9
YKL048C	ELM1	YNL076W	MKS1
YDL155W	CLB3	YMR038C	CCS1
YOR080W	DIA2	YOL090W	MSH2
YJL124C	LSM1	YNL147W	LSM7
YLR381W	CTF3	YIL103W	DPH1
YML095C	RAD10	YDL047W	SIT4
YLR386W	VAC14	YJR063W	RPA12
YLR119W	SRN2	YGL212W	VAM7
YGL163C	RAD54	YMR038C	CCS1
YMR199W	CLN1	YER110C	KAP123
YMR092C	AIP1	YIL034C	CAP2
YGR004W	PEX31	YLR324W	PEX30
YJR084W	CSN12	YMR230W	RPS10B
YNL206C	RTT106	YDR289C	RTT103
YBR093C	PHO5	YNL107W	YAF9
YPR046W	MCM16	YEL056W	HAT2
YHR152W	SPO12	YBL047C	EDE1
YNL166C	BNI5	YAR014C	BUD14
YIR003W	YIR003W	YIL034C	CAP2
YGL178W	MPT5	YBR089C-A	NHP6B
YJR060W	CBF1	YHR115C	DMA1
YNR072W	HXT17	YER162C	RAD4
YNL087W	TCB2	YNL238W	KEX2
YDR335W	MSN5	YMR038C	CCS1
YLR079W	SIC1	YOR039W	CKB2
YGR261C	APL6	YHR060W	VMA22

Predictions at threshold level 0.80 continued...			
Gene1 of predicted pair		Gene2 of predicted pair	
Systematic Name	Standard Name	Systematic Name	Standard Name
YGR071C	YGR071C	YDR318W	MCM21
YLR360W	VPS38	YHR161C	YAP1801
YAL024C	LTE1	YLR381W	CTF3
YIL034C	CAP2	YLR447C	VMA6
YGR014W	MSB2	YJL183W	MNN11
YGR249W	MGA1	YBR019C	GAL10
YLL007C	YLL007C	YHR178W	STB5
YGL174W	BUD13	YKL074C	MUD2
YGR072W	UPF3	YPL234C	TFP3
YMR055C	BUB2	YDR144C	MKC7
YKL213C	DOA1	YLR182W	SWI6
YHR205W	SCH9	YOR027W	STI1
YPL024W	RMI1	YGL003C	CDH1
YKL048C	ELM1	YDL192W	ARF1
YMR179W	SPT21	YMR038C	CCS1
YBL003C	HTA2	YPL248C	GAL4
YBR131W	CCZ1	YML071C	COG8
YHR134W	WSS1	YBL021C	HAP3
YCR077C	PAT1	YOR308C	SNU66
YKL139W	CTK1	YHR111W	UBA4
YKR020W	VPS51	YDR153C	ENT5
YER116C	SLX8	YBR036C	CSG2
YPR102C	RPL11A	YER102W	RPS8B
YHR134W	WSS1	YCL037C	SRO9
YJL036W	SNX4	YMR216C	SKY1
YMR038C	CCS1	YAL002W	VPS8
YKL139W	CTK1	YMR055C	BUB2
YLR371W	ROM2	YLR370C	ARC18
YKL007W	CAP1	YHR111W	UBA4

Predictions at threshold level 0.80 continued...			
Gene1 of predicted pair		Gene2 of predicted pair	
Systematic Name	Standard Name	Systematic Name	Standard Name
YPR046W	MCM16	YDR207C	UME6
YGL087C	MMS2	YDR155C	CPR1
YKR020W	VPS51	YJR033C	RAV1
YMR224C	MRE11	YLR425W	TUS1
YNL215W	IES2	YNR052C	POP2
YMR055C	BUB2	YHR200W	RPN10
YGL190C	CDC55	YNL250W	RAD50
YNL051W	COG5	YNR032W	PPG1
YDL101C	DUN1	YDR207C	UME6
YAL024C	LTE1	YLR410W	VIP1
YPR046W	MCM16	YLR398C	SKI2
YNL025C	SSN8	YIR005W	IST3
YMR199W	CLN1	YLR371W	ROM2
YNL264C	PDR17	YNL107W	YAF9
YJL176C	SWI3	YFR010W	UBP6
YLR079W	SIC1	YLR370C	ARC18
YLR192C	HCR1	YPL195W	APL5
YAL024C	LTE1	YDL192W	ARF1
YGL124C	MON1	YHR114W	BZZ1
YKL032C	IXR1	YPR046W	MCM16
YML095C	RAD10	YJR032W	CPR7
YEL050C	RML2	YDL047W	SIT4
YGL200C	EMP24	YML097C	VPS9
YDR295C	HDA2	YPR179C	HDA3
YLR386W	VAC14	YGR135W	PRE9
YDR363W-A	SEM1	YPR120C	CLB5
YNL041C	COG6	YJR084W	CSN12
YDR477W	SNF1	YML115C	VAN1
YGL215W	CLG1	YOR080W	DIA2

Predictions at threshold level 0.80 continued...			
Gene1 of predicted pair		Gene2 of predicted pair	
Systematic Name	Standard Name	Systematic Name	Standard Name
YOR066W	MSA1	YPR046W	MCM16
YDR105C	TMS1	YPR149W	NCE102
YNL032W	SIW14	YJR056C	YJR056C
YOR106W	VAM3	YER049W	TPA1
YHR187W	IKI1	YDL047W	SIT4
YBR215W	HPC2	YLR182W	SWI6
YGL200C	EMP24	YCL001W	RER1
YOR066W	MSA1	YMR214W	SCJ1
YDR293C	SSD1	YOR358W	HAP5
YGR070W	ROM1	YLR425W	TUS1
YGR054W	YGR054W	YHR012W	VPS29
YMR026C	PEX12	YPL161C	BEM4
YLR337C	VRP1	YJL190C	RPS22A
YDR293C	SSD1	YOR023C	AHC1
YNL025C	SSN8	YHR167W	THP2
YMR055C	BUB2	YCR077C	PAT1
YER044C-A	MEI4	YMR133W	REC114
YNL201C	PSY2	YJR135C	MCM22
YHL020C	OPI1	YJR033C	RAV1
YPL024W	RMI1	YJR084W	CSN12
YGR108W	CLB1	YGL115W	SNF4
YLR386W	VAC14	YPR051W	MAK3
YDR142C	PEX7	YGL153W	PEX14
YPL024W	RMI1	YCL037C	SRO9
YLR083C	EMP70	YPL234C	TFP3
YBR111W-A	SUS1	YBR010W	HHT1
YDR103W	STE5	YDR335W	MSN5
YHR156C	LIN1	YNL032W	SIW14
YJL124C	LSM1	YJR060W	CBF1

Predictions at threshold level 0.80 continued...			
Gene1 of predicted pair		Gene2 of predicted pair	
Systematic Name	Standard Name	Systematic Name	Standard Name
YIL159W	BNR1	YMR016C	SOK2
YNL307C	MCK1	YOR066W	MSA1
YCR020C-A	MAK31	YGR020C	VMA7
YBR107C	IML3	YKL114C	APN1
YGR241C	YAP1802	YDR358W	GGA1
YPL256C	CLN2	YJL095W	BCK1
YMR224C	MRE11	YGR249W	MGA1
YDR137W	RGP1	YER161C	SPT2
YGR129W	SYF2	YCR063W	BUD31
YDR477W	SNF1	YNL041C	COG6
YDR383C	NKP1	YKL139W	CTK1
YGL124C	MON1	YNL116W	DMA2
YLR192C	HCR1	YML071C	COG8
YGR081C	SLX9	YGL163C	RAD54
YGL025C	PGD1	YKR048C	NAP1
YLR248W	RCK2	YGL158W	RCK1
YMR243C	ZRC1	YGL158W	RCK1
YGL078C	DBP3	YFR010W	UBP6
YGL178W	MPT5	YLR330W	CHS5
YGL078C	DBP3	YHR039C-A	VMA10
YPL161C	BEM4	YER040W	GLN3
YOR043W	WHI2	YOR038C	HIR2
YOL018C	TLG2	YDL047W	SIT4
YOR069W	VPS5	YPL105C	YPL105C
YKL116C	PRR1	YDR310C	SUM1
YDR335W	MSN5	YOR141C	ARP8
YLR234W	TOP3	YOR014W	RTS1
YML071C	COG8	YAR014C	BUD14
YKL116C	PRR1	YNL032W	SIW14

Predictions at threshold level 0.80 continued...			
Gene1 of predicted pair		Gene2 of predicted pair	
Systematic Name	Standard Name	Systematic Name	Standard Name
YHL031C	GOS1	YKR001C	VPS1
YGR261C	APL6	YCR066W	RAD18
YDR363W-A	SEM1	YOR141C	ARP8
YDR108W	GSG1	YNL238W	KEX2
YDL184C	RPL41A	YHR081W	LRP1
YGL025C	PGD1	YER162C	RAD4
YLR192C	HCR1	YNL307C	MCK1
YEL009C	GCN4	YOR290C	SNF2
YOR290C	SNF2	YNL236W	SIN4
YPR017C	DSS4	YDR207C	UME6
YBR045C	GIP1	YDR103W	STE5
YHR160C	PEX18	YOL044W	PEX15
YLR360W	VPS38	YDR144C	MKC7
YDR440W	DOT1	YLR182W	SWI6
YBR215W	HPC2	YJR005W	APL1
YNL087W	TCB2	YOR270C	VPH1
YNL044W	YIP3	YER122C	GLO3
YDR146C	SWI5	YPL253C	VIK1
YDR293C	SSD1	YBL102W	SFT2
YPL037C	EGD1	YLR370C	ARC18
YFL013C	IES1	YGL174W	BUD13
YNL106C	INP52	YBL027W	RPL19B
YKL139W	CTK1	YER068W	MOT2
YIL008W	URM1	YJL187C	SWE1
YDR261C	EXG2	YOL121C	RPS19A
YGR270W	YTA7	YGL151W	NUT1
YGR020C	VMA7	YLR447C	VMA6
YER001W	MNN1	YFL048C	EMP47
YOR112W	CEX1	YMR004W	MVP1

Predictions at threshold level 0.80 continued...			
Gene1 of predicted pair		Gene2 of predicted pair	
Systematic Name	Standard Name	Systematic Name	Standard Name
YHR073W	OSH3	YOR358W	HAP5
YNL229C	URE2	YDR264C	AKR1
YKR020W	VPS51	YEL051W	VMA8
YML095C	RAD10	YDR076W	RAD55
YCL037C	SRO9	YHR039C-A	VMA10
YDR293C	SSD1	YER120W	SCS2
YBR171W	SEC66	YPL158C	YPL158C
YMR224C	MRE11	YKL116C	PRR1
YHR012W	VPS29	YNR032W	PPG1
YJL006C	CTK2	YPL018W	CTF19
YNL041C	COG6	YEL060C	PRB1
YJR033C	RAV1	YDR310C	SUM1
YGR105W	VMA21	YMR054W	STV1
YDL013W	SLX5	YPR120C	CLB5
YDL116W	NUP84	YFL023W	BUD27
YAL024C	LTE1	YBR274W	CHK1
YGR129W	SYF2	YGL174W	BUD13
YMR199W	CLN1	YGL208W	SIP2
YLL039C	UBI4	YBL101C	ECM21
YLR079W	SIC1	YKR024C	DBP7
YGR104C	SRB5	YBR009C	HHF1
YMR095C	SNO1	YPL161C	BEM4
YOR033C	EXO1	YOL090W	MSH2
YGL013C	PDR1	YPR119W	CLB2
YOL111C	MDY2	YPR046W	MCM16
YOR109W	INP53	YAR014C	BUD14
YJL172W	CPS1	YLR441C	RPS1A
YFR031C-A	RPL2A	YER162C	RAD4
YER139C	YER139C	YMR230W	RPS10B

Predictions at threshold level 0.80 continued...			
Gene1 of predicted pair		Gene2 of predicted pair	
Systematic Name	Standard Name	Systematic Name	Standard Name
YBL103C	RTG3	YPL195W	APL5
YDR383C	NKP1	YNR052C	POP2
YIL076W	SEC28	YOR112W	CEX1
YJL176C	SWI3	YNL079C	TPM1
YDR156W	RPA14	YDR439W	LRS4
YHR039C-A	VMA10	YOR368W	RAD17
YLR452C	SST2	YDR310C	SUM1
YPR120C	CLB5	YGR188C	BUB1
YNL025C	SSN8	YDR378C	LSM6
YNL047C	SLM2	YJL058C	BIT61
YEL065W	SIT1	YGL003C	CDH1
YBL051C	PIN4	YJL187C	SWE1
YMR133W	REC114	YDR358W	GGA1
YDL077C	VAM6	YEL042W	GDA1
YCL001W	RER1	YGL054C	ERV14
YGR020C	VMA7	YNL297C	MON2
YNL051W	COG5	YDR153C	ENT5
YLR410W	VIP1	YDL047W	SIT4
YKL139W	CTK1	YCR086W	CSM1
YBR194W	SOY1	YIL076W	SEC28
YDR156W	RPA14	YOR141C	ARP8
YNL025C	SSN8	YKL160W	ELF1
YNL106C	INP52	YDR439W	LRS4
YIL008W	URM1	YDR289C	RTT103
YHR010W	RPL27A	YGL246C	RAI1
YLR447C	VMA6	YGR105W	VMA21
YBR215W	HPC2	YER139C	YER139C
YKR035W-A	DID2	YOR035C	SHE4
YMR116C	ASC1	YBL025W	RRN10

Predictions at threshold level 0.80 continued...			
Gene1 of predicted pair		Gene2 of predicted pair	
Systematic Name	Standard Name	Systematic Name	Standard Name
YLR337C	VRP1	YOR023C	AHC1
YDR293C	SSD1	YER110C	KAP123
YJR040W	GEF1	YCL001W	RER1
YLR452C	SST2	YDL083C	RPS16B
YDR245W	MNN10	YOR308C	SNU66
YLR192C	HCR1	YMR230W	RPS10B
YOR195W	SLK19	YDR439W	LRS4
YEL027W	CUP5	YKL119C	VPH2
YDR129C	SAC6	YDL155W	CLB3
YMR039C	SUB1	YKR072C	SIS2
YJR140C	HIR3	YCL037C	SRO9
YKL048C	ELM1	YNL225C	CNM67
YMR039C	SUB1	YNL107W	YAF9
YGL178W	MPT5	YPL031C	PHO85
YBR073W	RDH54	YPR046W	MCM16
YGL087C	MMS2	YHR012W	VPS29
YPL240C	HSP82	YMR165C	PAH1
YPR072W	NOT5	YNL236W	SIN4
YML115C	VAN1	YOR035C	SHE4
YMR246W	FAA4	YOR059C	YOR059C
YML038C	YMD8	YEL043W	YEL043W
YMR224C	MRE11	YIL076W	SEC28
YOR195W	SLK19	YGR188C	BUB1
YKL139W	CTK1	YPR120C	CLB5
YPR084W	YPR084W	YEL051W	VMA8
YMR039C	SUB1	YDR146C	SWI5
YGR241C	YAP1802	YDL161W	ENT1
YMR026C	PEX12	YLR441C	RPS1A
YDR225W	HTA1	YOR368W	RAD17

Predictions at threshold level 0.80 continued...			
Gene1 of predicted pair		Gene2 of predicted pair	
Systematic Name	Standard Name	Systematic Name	Standard Name
YDL116W	NUP84	YNL107W	YAF9
YKL032C	IXR1	YHL002W	HSE1
YML115C	VAN1	YER139C	YER139C
YML115C	VAN1	YNL076W	MKS1
YDR477W	SNF1	YBL025W	RRN10
YML001W	YPT7	YEL051W	VMA8
YLR441C	RPS1A	YEL060C	PRB1
YGL161C	YIP5	YDR076W	RAD55
YPR120C	CLB5	YGL175C	SAE2
YDR477W	SNF1	YJL172W	CPS1
YOR141C	ARP8	YNL225C	CNM67
YHR060W	VMA22	YGL124C	MON1
YPL105C	YPL105C	YPR032W	SRO7
YBL058W	SHP1	YEL053C	MAK10
YCR020C-A	MAK31	YHL020C	OPI1
YLR006C	SSK1	YGR152C	RSR1
YNL238W	KEX2	YJR032W	CPR7
YDR443C	SSN2	YOR043W	WHI2
YGR108W	CLB1	YOL067C	RTG1
YGR135W	PRE9	YLR370C	ARC18
YPL050C	MNN9	YFR036W	CDC26
YNL025C	SSN8	YOR038C	HIR2
YMR055C	BUB2	YHR115C	DMA1
YML103C	NUP188	YIL036W	CST6
YIR009W	MSL1	YKR099W	BAS1
YHR156C	LIN1	YFL023W	BUD27
YJL124C	LSM1	YLR399C	BDF1
YDR507C	GIN4	YLR425W	TUS1
YKL010C	UFD4	YLL039C	UBI4

Predictions at threshold level 0.80 continued...			
Gene1 of predicted pair		Gene2 of predicted pair	
Systematic Name	Standard Name	Systematic Name	Standard Name
YPL042C	SSN3	YLR410W	VIP1
YHR134W	WSS1	YHR114W	BZZ1
YMR164C	MSS11	YMR199W	CLN1
YER087C-B	SBH1	YDR392W	SPT3
YHL031C	GOS1	YOR327C	SNC2
YPR017C	DSS4	YDR108W	GSG1
YLR386W	VAC14	YER040W	GLN3
YLR441C	RPS1A	YNL307C	MCK1
YNL329C	PEX6	YDR440W	DOT1
YAL024C	LTE1	YML016C	PPZ1
YDL006W	PTC1	YDR358W	GGA1
YMR216C	SKY1	YJL004C	SYS1
YIL103W	DPH1	YML112W	CTK3
YKL116C	PRR1	YPL161C	BEM4
YNR072W	HXT17	YMR152W	YIM1
YKR001C	VPS1	YCL001W	RER1
YDR202C	RAV2	YEL043W	YEL043W
YFL003C	MSH4	YGL249W	ZIP2
YJL154C	VPS35	YML038C	YMD8
YMR172W	HOT1	YGR108W	CLB1
YPR017C	DSS4	YJR033C	RAV1
YNL243W	SLA2	YJR058C	APS2
YML012W	ERV25	YLR093C	NYV1
YER110C	KAP123	YBR009C	HHF1
YLR095C	IOC2	YOR290C	SNF2
YCL037C	SRO9	YPL079W	RPL21B
YJL210W	PEX2	YDR244W	PEX5
YAL024C	LTE1	YER162C	RAD4
YGL003C	CDH1	YER122C	GLO3

Predictions at threshold level 0.80 continued...			
Gene1 of predicted pair		Gene2 of predicted pair	
Systematic Name	Standard Name	Systematic Name	Standard Name
YDR422C	SIP1	YGL179C	TOS3
YPL106C	SSE1	YJL006C	CTK2
YCR081W	SRB8	YER096W	SHC1
YLR399C	BDF1	YNL031C	HHT2
YNL238W	KEX2	YPL195W	APL5
YPR046W	MCM16	YER122C	GLO3
YER144C	UBP5	YBR280C	SAF1
YHR205W	SCH9	YBR260C	RGD1
YDR004W	RAD57	YHR013C	ARD1
YDL020C	RPN4	YBR228W	SLX1
YNL243W	SLA2	YPL031C	PHO85
YHR098C	SFB3	YFL031W	HAC1
YIL036W	CST6	YGL252C	RTG2
YLR386W	VAC14	YLR102C	APC9
YDL020C	RPN4	YGL237C	HAP2
YLR095C	IOC2	YOR066W	MSA1
YMR224C	MRE11	YGL163C	RAD54
YFR024C-A	LSB3	YMR253C	YMR253C
YEL051W	VMA8	YLR309C	IMH1
YIL076W	SEC28	YBR171W	SEC66
YGL178W	MPT5	YPR072W	NOT5
YEL053C	MAK10	YAR014C	BUD14
YLR095C	IOC2	YOL081W	IRA2
YLR399C	BDF1	YNL236W	SIN4
YDR378C	LSM6	YDR254W	CHL4
YMR004W	MVP1	YDR137W	RGP1
YOR033C	EXO1	YKL114C	APN1
YLR320W	MMS22	YBR280C	SAF1
YOR035C	SHE4	YOR036W	PEP12

Predictions at threshold level 0.80 continued...			
Gene1 of predicted pair		Gene2 of predicted pair	
Systematic Name	Standard Name	Systematic Name	Standard Name
YPR084W	YPR084W	YGL223C	COG1
YDR174W	HMO1	YJL124C	LSM1
YML103C	NUP188	YOR141C	ARP8
YHR056C	RSC30	YKR082W	NUP133
YDR293C	SSD1	YMR127C	SAS2
YPR051W	MAK3	YDL006W	PTC1
YNL054W	VAC7	YAR014C	BUD14
YPL037C	EGD1	YJL004C	SYS1
YHR079C	IRE1	YNR049C	MSO1
YPL115C	BEM3	YAR014C	BUD14
YMR199W	CLN1	YNL079C	TPM1
YDR245W	MNN10	YNL107W	YAF9
YFR032C-A	RPL29	YLR320W	MMS22
YGR261C	APL6	YJR033C	RAV1
YOR332W	VMA4	YJL183W	MNN11
YGL124C	MON1	YNL107W	YAF9
YOR109W	INP53	YOR327C	SNC2
YCR081W	SRB8	YPL161C	BEM4
YOR132W	VPS17	YDR358W	GGA1
YMR026C	PEX12	YMR133W	REC114
YDR254W	CHL4	YDR310C	SUM1
YOR035C	SHE4	YMR214W	SCJ1
YGL025C	PGD1	YPR120C	CLB5
YGR019W	UGA1	YPR046W	MCM16
YOR132W	VPS17	YAR014C	BUD14
YGR159C	NSR1	YKL041W	VPS24
YBR057C	MUM2	YKL139W	CTK1
YLR452C	SST2	YML071C	COG8
YLR386W	VAC14	YLR180W	SAM1

Predictions at threshold level 0.80 continued...			
**Gene1 of predicted pair**		**Gene2 of predicted pair**	
**Systematic Name**	**Standard Name**	**Systematic Name**	**Standard Name**
YOR035C	SHE4	YOL081W	IRA2
YDR320C	SWA2	YPR051W	MAK3
YDL080C	THI3	YLR337C	VRP1
YKL139W	CTK1	YML115C	VAN1
YBR042C	CST26	YOR080W	DIA2
YMR109W	MYO5	YHL002W	HSE1
YKL032C	IXR1	YKL101W	HSL1
YGL115W	SNF4	YPR046W	MCM16
YBR171W	SEC66	YAL002W	VPS8
YPR051W	MAK3	YML071C	COG8
YDR137W	RGP1	YLR398C	SKI2
YDR293C	SSD1	YHL031C	GOS1
YML001W	YPT7	YMR214W	SCJ1
YKL032C	IXR1	YDR076W	RAD55
YFR010W	UBP6	YMR133W	REC114
YIL053W	RHR2	YBR200W	BEM1
YOR332W	VMA4	YMR054W	STV1
YPL024W	RMI1	YCR031C	RPS14A
YGL078C	DBP3	YER116C	SLX8
YMR133W	REC114	YNL041C	COG6
YLL007C	YLL007C	YEL053C	MAK10
YJL176C	SWI3	YLR357W	RSC2
YGL066W	SGF73	YNL229C	URE2
YLR150W	STM1	YBR107C	IML3
YJL154C	VPS35	YDL006W	PTC1
YHR161C	YAP1801	YPL045W	VPS16
YBL008W	HIR1	YDL047W	SIT4
YLR320W	MMS22	YDL077C	VAM6
YLR234W	TOP3	YAR014C	BUD14

Predictions at threshold level 0.80 continued...			
**Gene1 of predicted pair**		**Gene2 of predicted pair**	
**Systematic Name**	**Standard Name**	**Systematic Name**	**Standard Name**
YBR270C	BIT2	YPR119W	CLB2
YBL008W	HIR1	YBR089C-A	NHP6B
YBR073W	RDH54	YNL248C	RPA49
YOR039W	CKB2	YKL048C	ELM1
YHR200W	RPN10	YER122C	GLO3
YDR080W	VPS41	YEL013W	VAC8
YPL115C	BEM3	YOR358W	HAP5
YDR477W	SNF1	YHR039C-A	VMA10
YLR102C	APC9	YDR379W	RGA2
YGL153W	PEX14	YBL101C	ECM21
YDR254W	CHL4	YDL047W	SIT4
YMR319C	FET4	YPR149W	NCE102
YKR020W	VPS51	YOR036W	PEP12
YMR029C	FAR8	YPR119W	CLB2
YLR360W	VPS38	YGR188C	BUB1
YJR140C	HIR3	YDR310C	SUM1
YJL136C	RPS21B	YBR084C-A	RPL19A
YMR198W	CIK1	YML016C	PPZ1
YFR036W	CDC26	YPL018W	CTF19
YDR378C	LSM6	YJR135C	MCM22
YDL005C	MED2	YNL031C	HHT2
YFR010W	UBP6	YJL172W	CPS1
YKL010C	UFD4	YGL153W	PEX14
YBR173C	UMP1	YLR371W	ROM2
YDL116W	NUP84	YLR315W	NKP2
YKL092C	BUD2	YGR152C	RSR1
YEL056W	HAT2	YOL067C	RTG1
YLR018C	POM34	YKL068W	NUP100
YMR004W	MVP1	YPL234C	TFP3

Predictions at threshold level 0.80 continued...			
Gene1 of predicted pair		Gene2 of predicted pair	
Systematic Name	Standard Name	Systematic Name	Standard Name
YLR371W	ROM2	YPR046W	MCM16
YGL033W	HOP2	YLR093C	NYV1
YLR083C	EMP70	YGL223C	COG1
YHR012W	VPS29	YDR183W	PLP1
YNL325C	FIG4	YBR108W	YBR108W
YPL213W	LEA1	YKL041W	VPS24
YLR083C	EMP70	YGL212W	VAM7
YDR153C	ENT5	YPR032W	SRO7
YLL039C	UBI4	YGL153W	PEX14
YLR337C	VRP1	YMR032W	HOF1
YOL121C	RPS19A	YDR358W	GGA1
YML058W	SML1	YKL139W	CTK1
YAL051W	OAF1	YOR363C	PIP2
YIL002C	INP51	YDR358W	GGA1
YNL291C	MID1	YEL053C	MAK10
YPL024W	RMI1	YLR381W	CTF3
YML071C	COG8	YBR019C	GAL10
YIL076W	SEC28	YNL058C	YNL058C
YGR270W	YTA7	YPR046W	MCM16
YER116C	SLX8	YLR441C	RPS1A
YDL161W	ENT1	YKR020W	VPS51
YLR371W	ROM2	YFR036W	CDC26
YBR030W	YBR030W	YMR311C	GLC8
YML095C	RAD10	YNL116W	DMA2
YGL178W	MPT5	YNL236W	SIN4
YBR107C	IML3	YGL115W	SNF4
YNL215W	IES2	YOL054W	PSH1
YKL139W	CTK1	YGL163C	RAD54
YML095C	RAD10	YDR363W-A	SEM1

Predictions at threshold level 0.80 continued...			
Gene1 of predicted pair		Gene2 of predicted pair	
Systematic Name	Standard Name	Systematic Name	Standard Name
YDR080W	VPS41	YNL127W	FAR11
YEL043W	YEL043W	YNL051W	COG5
YML001W	YPT7	YJL004C	SYS1
YDR283C	GCN2	YKR026C	GCN3
YDR129C	SAC6	YBR181C	RPS6B
YHR156C	LIN1	YBR280C	SAF1
YLR095C	IOC2	YER122C	GLO3
YPL240C	HSP82	YHL050C	YHL050C
YFR010W	UBP6	YIR005W	IST3
YDR137W	RGP1	YKL041W	VPS24
YML071C	COG8	YKL119C	VPH2
YNL218W	MGS1	YBR082C	UBC4
YHR178W	STB5	YBL051C	PIN4
YPL234C	TFP3	YDL077C	VAM6
YNL025C	SSN8	YPL248C	GAL4
YBR131W	CCZ1	YML001W	YPT7
YDR192C	NUP42	YDR076W	RAD55
YMR224C	MRE11	YEL060C	PRB1
YML001W	YPT7	YPL045W	VPS16
YOR195W	SLK19	YGL031C	RPL24A
YGL237C	HAP2	YBR171W	SEC66
YER044C	ERG28	YPL137C	GIP3
YHR111W	UBA4	YPR046W	MCM16
YHR205W	SCH9	YEL036C	ANP1
YJR135C	MCM22	YNL116W	DMA2
YKL139W	CTK1	YOL081W	IRA2
YBL102W	SFT2	YJL036W	SNX4
YNL307C	MCK1	YLR398C	SKI2
YMR116C	ASC1	YLR135W	SLX4

Predictions at threshold level 0.80 continued...			
Gene1 of predicted pair		Gene2 of predicted pair	
Systematic Name	Standard Name	Systematic Name	Standard Name
YPR032W	SRO7	YOL108C	INO4
YOR069W	VPS5	YPL259C	APM1
YOR127W	RGA1	YAL002W	VPS8
YNL069C	RPL16B	YPL105C	YPL105C
YMR095C	SNO1	YMR153W	NUP53
YDR146C	SWI5	YER161C	SPT2
YLR191W	PEX13	YDR142C	PEX7
YKR024C	DBP7	YML057W	CMP2
YBR200W	BEM1	YBL051C	PIN4
YPL042C	SSN3	YPR046W	MCM16
YER116C	SLX8	YDL059C	RAD59
YER116C	SLX8	YDL155W	CLB3
YNL087W	TCB2	YOR035C	SHE4
YNL069C	RPL16B	YOL018C	TLG2
YPR131C	NAT3	YMR224C	MRE11
YBR194W	SOY1	YGR014W	MSB2
YNL291C	MID1	YPL195W	APL5
YGL212W	VAM7	YNL307C	MCK1
YGL054C	ERV14	YML001W	YPT7
YNL243W	SLA2	YLL021W	SPA2
YMR224C	MRE11	YGR188C	BUB1
YER110C	KAP123	YGL016W	KAP122
YPR040W	TIP41	YKL048C	ELM1
YGL163C	RAD54	YNL116W	DMA2
YOR080W	DIA2	YPR120C	CLB5
YJR056C	YJR056C	YJL183W	MNN11
YBL051C	PIN4	YOR358W	HAP5
YLR396C	VPS33	YMR231W	PEP5
YDR004W	RAD57	YKR095W	MLP1

Predictions at threshold level 0.80 continued...			
Gene1 of predicted pair		Gene2 of predicted pair	
Systematic Name	Standard Name	Systematic Name	Standard Name
YDR383C	NKP1	YFR030W	MET10
YDR484W	VPS52	YOR036W	PEP12
YMR095C	SNO1	YBL025W	RRN10
YAL020C	ATS1	YDR507C	GIN4
YNL041C	COG6	YKR030W	GMH1
YDR264C	AKR1	YKR048C	NAP1
YDR245W	MNN10	YJL183W	MNN11
YGL237C	HAP2	YGR188C	BUB1
YLR018C	POM34	YIL148W	RPL40A
YBR107C	IML3	YDR363W-A	SEM1
YHR134W	WSS1	YKL048C	ELM1
YLR337C	VRP1	YER001W	MNN1
YCR066W	RAD18	YJR060W	CBF1
YGL066W	SGF73	YDR264C	AKR1
YMR055C	BUB2	YOR038C	HIR2
YHL020C	OPI1	YPL234C	TFP3
YBR200W	BEM1	YAR014C	BUD14
YBR289W	SNF5	YBR010W	HHT1
YLR433C	CNA1	YIR009W	MSL1
YMR055C	BUB2	YLR233C	EST1
YDR422C	SIP1	YPL256C	CLN2
YDR245W	MNN10	YOR368W	RAD17
YNL243W	SLA2	YBR164C	ARL1
YKL135C	APL2	YDL006W	PTC1
YGL163C	RAD54	YGL151W	NUT1
YNL041C	COG6	YBR280C	SAF1
YGL115W	SNF4	YER027C	GAL83
YPR167C	MET16	YLR093C	NYV1
YOR035C	SHE4	YBR036C	CSG2

Predictions at threshold level 0.80 continued...			
Gene1 of predicted pair		Gene2 of predicted pair	
Systematic Name	Standard Name	Systematic Name	Standard Name
YJL124C	LSM1	YML088W	UFO1
YDR289C	RTT103	YOL067C	RTG1
YJL140W	RPB4	YNL107W	YAF9
YDR129C	SAC6	YKL032C	IXR1
YDR484W	VPS52	YFL023W	BUD27
YMR055C	BUB2	YDL155W	CLB3
YDL020C	RPN4	YHR111W	UBA4
YDR202C	RAV2	YCR066W	RAD18
YMR199W	CLN1	YML028W	TSA1
YIR005W	IST3	YML112W	CTK3
YCR081W	SRB8	YEL036C	ANP1
YNL069C	RPL16B	YCR077C	PAT1
YLR386W	VAC14	YMR153W	NUP53
YOR112W	CEX1	YNL297C	MON2
YHR167W	THP2	YLR182W	SWI6
YGL200C	EMP24	YER118C	SHO1
YIL053W	RHR2	YHR200W	RPN10
YIL034C	CAP2	YPL161C	BEM4
YPL057C	SUR1	YBL025W	RRN10
YPR036W	VMA13	YNL238W	KEX2
YBL047C	EDE1	YOR368W	RAD17
YLR102C	APC9	YNL206C	RTT106
YFR010W	UBP6	YLR320W	MMS22
YOR080W	DIA2	YHR167W	THP2
YJL172W	CPS1	YFR036W	CDC26
YDR004W	RAD57	YGR188C	BUB1
YPL024W	RMI1	YDR245W	MNN10
YEL015W	EDC3	YJR140C	HIR3
YPL180W	TCO89	YML097C	VPS9

Predictions at threshold level 0.80 continued...			
Gene1 of predicted pair		Gene2 of predicted pair	
Systematic Name	Standard Name	Systematic Name	Standard Name
YGL078C	DBP3	YJL095W	BCK1
YPL042C	SSN3	YOR156C	NFI1
YGR184C	UBR1	YBR107C	IML3
YOR368W	RAD17	YBR036C	CSG2
YLL007C	YLL007C	YKL048C	ELM1
YPL024W	RMI1	YPL161C	BEM4
YLR386W	VAC14	YDR310C	SUM1
YKL139W	CTK1	YOR141C	ARP8
YPL129W	TAF14	YNR023W	SNF12
YMR004W	MVP1	YKR020W	VPS51
YNL058C	YNL058C	YFR036W	CDC26
YBR107C	IML3	YHR111W	UBA4
YBR274W	CHK1	YGL163C	RAD54
YOR270C	VPH1	YLR452C	SST2
YPL253C	VIK1	YOL081W	IRA2
YGL054C	ERV14	YDR207C	UME6
YBL051C	PIN4	YPL253C	VIK1
YGR020C	VMA7	YCL001W	RER1
YPR018W	RLF2	YJR135C	MCM22
YPL057C	SUR1	YGR071C	YGR071C
YKR035W-A	DID2	YER122C	GLO3
YPL024W	RMI1	YKL126W	YPK1
YGR241C	YAP1802	YCR088W	ABP1
YML038C	YMD8	YLR309C	IMH1
YDR484W	VPS52	YBR019C	GAL10
YDL101C	DUN1	YML095C	RAD10
YDR144C	MKC7	YAR014C	BUD14
YJR032W	CPR7	YNL107W	YAF9
YPR017C	DSS4	YNL107W	YAF9

Predictions at threshold level 0.80 continued...			
Gene1 of predicted pair		Gene2 of predicted pair	
Systematic Name	Standard Name	Systematic Name	Standard Name
YHR205W	SCH9	YOR014W	RTS1
YDL116W	NUP84	YPL246C	RBD2
YBL047C	EDE1	YKL161C	YKL161C
YGR261C	APL6	YPL105C	YPL105C
YML071C	COG8	YNL107W	YAF9
YCL037C	SRO9	YDR207C	UME6
YPL001W	HAT1	YNL031C	HHT2
YBR041W	FAT1	YPL161C	BEM4
YOR071C	THI71	YPR149W	NCE102
YNL288W	CAF40	YDR254W	CHL4
YLR095C	IOC2	YGL147C	RPL9A
YKR020W	VPS51	YPL213W	LEA1
YNL032W	SIW14	YMR311C	GLC8
YOR358W	HAP5	YDR318W	MCM21
YGR249W	MGA1	YPL195W	APL5
YBR274W	CHK1	YJR032W	CPR7
YLR240W	VPS34	YCR088W	ABP1
YJL106W	IME2	YJR094C	IME1
YDR378C	LSM6	YMR038C	CCS1
YGL223C	COG1	YDL006W	PTC1
YOR080W	DIA2	YJR060W	CBF1
YHR156C	LIN1	YLR180W	SAM1
YLR386W	VAC14	YDR358W	GGA1
YOR038C	HIR2	YGL148W	ARO2
YGL033W	HOP2	YNL041C	COG6
YER162C	RAD4	YER122C	GLO3
YJL006C	CTK2	YNL116W	DMA2
YOR080W	DIA2	YLR370C	ARC18
YGR019W	UGA1	YBR108W	YBR108W

Predictions at threshold level 0.80 continued...			
Gene1 of predicted pair		Gene2 of predicted pair	
Systematic Name	Standard Name	Systematic Name	Standard Name
YHR081W	LRP1	YER122C	GLO3
YOR005C	DNL4	YCR014C	POL4
YNL087W	TCB2	YOR290C	SNF2
YPR084W	YPR084W	YCL037C	SRO9
YLR079W	SIC1	YML112W	CTK3
YBL102W	SFT2	YAL002W	VPS8
YDR129C	SAC6	YBR171W	SEC66
YLR079W	SIC1	YMR179W	SPT21
YNL206C	RTT106	YOR275C	RIM20
YDL244W	THI13	YPL240C	HSP82
YML095C	RAD10	YHR010W	RPL27A
YLR090W	XDJ1	YBR030W	YBR030W
YML115C	VAN1	YER040W	GLN3
YLR360W	VPS38	YDR254W	CHL4
YNL094W	APP1	YKR030W	GMH1
YDR363W-A	SEM1	YAL002W	VPS8
YBR131W	CCZ1	YPL089C	RLM1
YKR061W	KTR2	YJR135C	MCM22
YFR024C-A	LSB3	YKR030W	GMH1
YPL024W	RMI1	YGL163C	RAD54
YER019C-A	SBH2	YDL006W	PTC1
YMR172W	HOT1	YOL067C	RTG1
YJL176C	SWI3	YBL072C	RPS8A
YMR179W	SPT21	YJR135C	MCM22
YBR107C	IML3	YAL002W	VPS8
YLR386W	VAC14	YAL002W	VPS8
YGL147C	RPL9A	YER044C	ERG28
YEL044W	IES6	YBL072C	RPS8A
YNL058C	YNL058C	YER139C	YER139C

Predictions at threshold level 0.80 continued...			
Gene1 of predicted pair		Gene2 of predicted pair	
Systematic Name	Standard Name	Systematic Name	Standard Name
YPL022W	RAD1	YLR410W	VIP1
YJR024C	MDE1	YJR059W	PTK2
YGL078C	DBP3	YMR133W	REC114
YLR320W	MMS22	YPR030W	CSR2
YDR004W	RAD57	YJL124C	LSM1
YMR224C	MRE11	YNL307C	MCK1
YLR079W	SIC1	YDL047W	SIT4
YBR217W	ATG12	YER122C	GLO3
YNL166C	BNI5	YPL018W	CTF19
YBR200W	BEM1	YMR230W	RPS10B
YMR054W	STV1	YPL120W	VPS30
YGL212W	VAM7	YER162C	RAD4
YGR249W	MGA1	YDR477W	SNF1
YDR477W	SNF1	YEL053C	MAK10
YDR395W	SXM1	YBL079W	NUP170
YGL161C	YIP5	YDR310C	SUM1
YDR254W	CHL4	YOL067C	RTG1
YBR131W	CCZ1	YDL192W	ARF1
YMR202W	ERG2	YDR507C	GIN4
YDR289C	RTT103	YDR155C	CPR1
YLR090W	XDJ1	YBR217W	ATG12
YNL116W	DMA2	YGL252C	RTG2
YDR137W	RGP1	YEL013W	VAC8
YGL135W	RPL1B	YLR360W	VPS38
YLR410W	VIP1	YFR036W	CDC26
YML115C	VAN1	YBR041W	FAT1
YBR171W	SEC66	YEL053C	MAK10
YGL060W	YBP2	YJL006C	CTK2
YPL042C	SSN3	YJL089W	SIP4

Predictions at threshold level 0.80 continued...			
Gene1 of predicted pair		Gene2 of predicted pair	
Systematic Name	Standard Name	Systematic Name	Standard Name
YLR399C	BDF1	YOR141C	ARP8
YGL250W	RMR1	YDR310C	SUM1
YDL184C	RPL41A	YPR036W	VMA13
YOR035C	SHE4	YBL072C	RPS8A
YOR337W	TEA1	YDR261C	EXG2
YGR105W	VMA21	YKL080W	VMA5
YDL020C	RPN4	YIL008W	URM1
YNL307C	MCK1	YGR135W	PRE9
YBR200W	BEM1	YLR425W	TUS1
YLR447C	VMA6	YPL234C	TFP3
YDR309C	GIC2	YNL116W	DMA2
YNL243W	SLA2	YNL020C	ARK1
YGR072W	UPF3	YDR153C	ENT5
YKL116C	PRR1	YER162C	RAD4
YDL116W	NUP84	YKL117W	SBA1
YEL009C	GCN4	YOR298C-A	MBF1
YOR127W	RGA1	YOL018C	TLG2
YOR337W	TEA1	YLR370C	ARC18
YLR370C	ARC18	YPR119W	CLB2
YBR215W	HPC2	YDR440W	DOT1
YPL045W	VPS16	YGL198W	YIP4
YKR072C	SIS2	YOR080W	DIA2
YGR108W	CLB1	YEL056W	HAT2
YOR132W	VPS17	YJR058C	APS2
YDR440W	DOT1	YDR155C	CPR1
YOR358W	HAP5	YGL253W	HXK2
YPR046W	MCM16	YOR035C	SHE4
YDL116W	NUP84	YJL006C	CTK2
YOR069W	VPS5	YDL006W	PTC1

Predictions at threshold level 0.80 continued...			
Gene1 of predicted pair		Gene2 of predicted pair	
Systematic Name	Standard Name	Systematic Name	Standard Name
YGL223C	COG1	YML001W	YPT7
YBR217W	ATG12	YPL137C	GIP3
YPL057C	SUR1	YHR013C	ARD1
YNL199C	GCR2	YKL068W	NUP100
YGR217W	CCH1	YGR135W	PRE9
YFR010W	UBP6	YNL117W	MLS1
YPL042C	SSN3	YDR289C	RTT103
YGR270W	YTA7	YHR115C	DMA1
YOR270C	VPH1	YDL192W	ARF1
YFL023W	BUD27	YPR036W	VMA13
YGL241W	KAP114	YBR010W	HHT1
YKL101W	HSL1	YGR188C	BUB1
YBL087C	RPL23A	YHR115C	DMA1
YAR014C	BUD14	YER122C	GLO3
YPL161C	BEM4	YDR289C	RTT103
YNL259C	ATX1	YDR270W	CCC2
YML095C	RAD10	YJR090C	GRR1
YOR001W	RRP6	YGL174W	BUD13
YPL253C	VIK1	YPL018W	CTF19
YBR217W	ATG12	YNL076W	MKS1
YER110C	KAP123	YPR051W	MAK3
YMR055C	BUB2	YOR043W	WHI2
YMR224C	MRE11	YHR013C	ARD1
YBL058W	SHP1	YER162C	RAD4
YCR065W	HCM1	YGL031C	RPL24A
YBR108W	YBR108W	YOL111C	MDY2
YER120W	SCS2	YDR310C	SUM1
YLR320W	MMS22	YHR039C-A	VMA10
YGR135W	PRE9	YDR318W	MCM21

Predictions at threshold level 0.80 continued...			
Gene1 of predicted pair		Gene2 of predicted pair	
Systematic Name	Standard Name	Systematic Name	Standard Name
YML001W	YPT7	YNL107W	YAF9
YIL076W	SEC28	YLR441C	RPS1A
YLR337C	VRP1	YLR234W	TOP3
YBR228W	SLX1	YCL037C	SRO9
YGR249W	MGA1	YER139C	YER139C
YOR033C	EXO1	YGL175C	SAE2
YIL053W	RHR2	YMR133W	REC114
YNL307C	MCK1	YFR030W	MET10
YCR086W	CSM1	YBR036C	CSG2
YLR337C	VRP1	YBR114W	RAD16
YPL057C	SUR1	YOR038C	HIR2
YML115C	VAN1	YOL081W	IRA2
YGL095C	VPS45	YAL030W	SNC1
YBR057C	MUM2	YLR032W	RAD5
YJL187C	SWE1	YDR207C	UME6
YGR072W	UPF3	YLR135W	SLX4
YGR270W	YTA7	YCR077C	PAT1
YOR127W	RGA1	YMR054W	STV1
YDR080W	VPS41	YJR033C	RAV1
YIL159W	BNR1	YJR056C	YJR056C
YGL078C	DBP3	YJR056C	YJR056C
YOR145C	PNO1	YOR078W	BUD21
YKR001C	VPS1	YOR368W	RAD17
YDL116W	NUP84	YDR156W	RPA14
YLR399C	BDF1	YNL248C	RPA49
YML103C	NUP188	YKL110C	KTI12
YMR198W	CIK1	YAL002W	VPS8
YPR101W	SNT309	YJR050W	ISY1
YKR072C	SIS2	YMR038C	CCS1

Predictions at threshold level 0.80 continued...			
Gene1 of predicted pair		Gene2 of predicted pair	
Systematic Name	Standard Name	Systematic Name	Standard Name
YDR225W	HTA1	YMR153W	NUP53
YLR234W	TOP3	YML016C	PPZ1
YIL008W	URM1	YGL163C	RAD54
YOR069W	VPS5	YDR310C	SUM1
YNL291C	MID1	YPL161C	BEM4
YMR224C	MRE11	YKR001C	VPS1
YLR452C	SST2	YER122C	GLO3
YDR176W	NGG1	YBL005W	PDR3
YGL147C	RPL9A	YDL083C	RPS16B
YFR010W	UBP6	YBR200W	BEM1
YBL019W	APN2	YMR055C	BUB2
YGR261C	APL6	YER162C	RAD4
YDR484W	VPS52	YJR084W	CSN12
YDR097C	MSH6	YGL100W	SEH1
YMR224C	MRE11	YMR116C	ASC1
YBR030W	YBR030W	YJR084W	CSN12
YNL329C	PEX6	YKL041W	VPS24
YPL105C	YPL105C	YKR030W	GMH1
YGR261C	APL6	YLR309C	IMH1
YDR335W	MSN5	YMR055C	BUB2
YAL020C	ATS1	YKL048C	ELM1
YJL154C	VPS35	YIL008W	URM1
YLR094C	GIS3	YJL095W	BCK1
YDR202C	RAV2	YDL192W	ARF1
YPL105C	YPL105C	YKR020W	VPS51
YKL139W	CTK1	YDR310C	SUM1
YKR072C	SIS2	YHR010W	RPL27A
YNL016W	PUB1	YMR038C	CCS1
YNL025C	SSN8	YNL236W	SIN4

Predictions at threshold level 0.80 continued...			
Gene1 of predicted pair		Gene2 of predicted pair	
Systematic Name	Standard Name	Systematic Name	Standard Name
YKL032C	IXR1	YDR310C	SUM1
YFL023W	BUD27	YGL124C	MON1
YGR129W	SYF2	YKL074C	MUD2
YKL007W	CAP1	YLR216C	CPR6
YNL248C	RPA49	YDR289C	RTT103
YGL253W	HXK2	YAL002W	VPS8
YDR477W	SNF1	YDR456W	NHX1
YPL050C	MNN9	YNL041C	COG6
YJR135C	MCM22	YOR014W	RTS1
YGR072W	UPF3	YBL102W	SFT2
YOR069W	VPS5	YCR066W	RAD18
YMR142C	RPL13B	YHR081W	LRP1
YJL124C	LSM1	YDR289C	RTT103
YNL135C	FPR1	YBL103C	RTG3
YOR156C	NFI1	YPL057C	SUR1
YML095C	RAD10	YGL031C	RPL24A
YMR153W	NUP53	YBL103C	RTG3
YNL307C	MCK1	YJL095W	BCK1
YBL102W	SFT2	YDR207C	UME6
YLR234W	TOP3	YGL019W	CKB1
YPL246C	RBD2	YHR016C	YSC84
YLR399C	BDF1	YNL107W	YAF9
YNL215W	IES2	YGL163C	RAD54
YCR034W	FEN1	YML016C	PPZ1
YBR173C	UMP1	YHR081W	LRP1
YPL050C	MNN9	YMR109W	MYO5
YML071C	COG8	YER139C	YER139C
YDR165W	TRM82	YDL201W	TRM8
YGL135W	RPL1B	YML115C	VAN1

Predictions at threshold level 0.80 continued...			
Gene1 of predicted pair		Gene2 of predicted pair	
Systematic Name	Standard Name	Systematic Name	Standard Name
YHR060W	VMA22	YPL234C	TFP3
YDR137W	RGP1	YOR124C	UBP2
YMR224C	MRE11	YLR288C	MEC3
YKL025C	PAN3	YEL053C	MAK10
YGR072W	UPF3	YOR327C	SNC2
YKR099W	BAS1	YKL048C	ELM1
YLR192C	HCR1	YCR053W	THR4
YCR020C-A	MAK31	YMR054W	STV1
YER162C	RAD4	YLR180W	SAM1
YJR135C	MCM22	YHR114W	BZZ1
YLR119W	SRN2	YNL032W	SIW14
YDR309C	GIC2	YDR227W	SIR4
YLR083C	EMP70	YER122C	GLO3
YMR142C	RPL13B	YDL077C	VAM6
YPL240C	HSP82	YJL103C	GSM1
YBL087C	RPL23A	YNL116W	DMA2
YJR140C	HIR3	YCR077C	PAT1
YKL130C	SHE2	YKL185W	ASH1
YDL213C	NOP6	YOR078W	BUD21
YBR200W	BEM1	YMR109W	MYO5
YML016C	PPZ1	YPL256C	CLN2
YKL048C	ELM1	YHR060W	VMA22
YDR155C	CPR1	YPL018W	CTF19
YPL129W	TAF14	YLR176C	RFX1
YDR320C	SWA2	YDL226C	GCS1
YLR095C	IOC2	YOR270C	VPH1
YGR270W	YTA7	YKL160W	ELF1
YIL159W	BNR1	YEL053C	MAK10
YJR059W	PTK2	YMR164C	MSS11

Predictions at threshold level 0.80 continued...			
Gene1 of predicted pair		Gene2 of predicted pair	
Systematic Name	Standard Name	Systematic Name	Standard Name
YLR386W	VAC14	YKR035W-A	DID2
YDR080W	VPS41	YGR105W	VMA21
YGL054C	ERV14	YGR105W	VMA21
YDR260C	SWM1	YPL018W	CTF19
YBL007C	SLA1	YGL095C	VPS45
YML095C	RAD10	YLR352W	YLR352W
YBR200W	BEM1	YBR171W	SEC66
YGL087C	MMS2	YNL166C	BNI5
YFL023W	BUD27	YPL137C	GIP3
YLR313C	SPH1	YBR082C	UBC4
YBR138C	YBR138C	YGR223C	HSV2
YKR048C	NAP1	YLR425W	TUS1
YCR077C	PAT1	YGR152C	RSR1
YBR127C	VMA2	YMR054W	STV1
YPL256C	CLN2	YKL101W	HSL1
YAL024C	LTE1	YHR200W	RPN10
YOR290C	SNF2	YML071C	COG8
YMR133W	REC114	YBL025W	RRN10
YJR135C	MCM22	YDR289C	RTT103
YNL032W	SIW14	YHR200W	RPN10
YBR215W	HPC2	YCR086W	CSM1
YPR018W	RLF2	YMR038C	CCS1
YDR202C	RAV2	YDR207C	UME6
YER162C	RAD4	YLR093C	NYV1
YGL223C	COG1	YEL036C	ANP1
YHR013C	ARD1	YAR014C	BUD14
YKL126W	YPK1	YOL121C	RPS19A
YNL307C	MCK1	YEL053C	MAK10
YAL024C	LTE1	YOL111C	MDY2

Predictions at threshold level 0.80 continued...			
Gene1 of predicted pair		Gene2 of predicted pair	
Systematic Name	Standard Name	Systematic Name	Standard Name
YGL087C	MMS2	YAR014C	BUD14
YLR386W	VAC14	YNL076W	MKS1
YGL066W	SGF73	YDR254W	CHL4
YGL115W	SNF4	YHR081W	LRP1
YLR386W	VAC14	YDR261C	EXG2
YDR358W	GGA1	YOR035C	SHE4
YEL053C	MAK10	YLR309C	IMH1
YLR090W	XDJ1	YDR358W	GGA1
YDR320C	SWA2	YML038C	YMD8
YHR134W	WSS1	YOR080W	DIA2
YNL004W	HRB1	YCL011C	GBP2
YDR363W-A	SEM1	YJR035W	RAD26
YBR107C	IML3	YDL155W	CLB3
YLR090W	XDJ1	YDR137W	RGP1
YOR368W	RAD17	YKL114C	APN1
YDR004W	RAD57	YBR195C	MSI1
YKL025C	PAN3	YIL034C	CAP2
YJR135C	MCM22	YGR135W	PRE9
YPR051W	MAK3	YJR135C	MCM22
YOR069W	VPS5	YOR106W	VAM3
YOR195W	SLK19	YJR005W	APL1
YOL111C	MDY2	YJR084W	CSN12
YGL237C	HAP2	YJL190C	RPS22A
YJR140C	HIR3	YMR055C	BUB2
YNL206C	RTT106	YOR141C	ARP8
YKL160W	ELF1	YHR167W	THP2
YER116C	SLX8	YAL009W	SPO7
YJL036W	SNX4	YPR119W	CLB2
YGL161C	YIP5	YNL107W	YAF9

Predictions at threshold level 0.80 continued...			
Gene1 of predicted pair		Gene2 of predicted pair	
Systematic Name	Standard Name	Systematic Name	Standard Name
YGL033W	HOP2	YER040W	GLN3
YEL053C	MAK10	YJL004C	SYS1
YHR152W	SPO12	YDL047W	SIT4
YGR239C	PEX21	YGL153W	PEX14
YDR293C	SSD1	YPR120C	CLB5
YOR337W	TEA1	YHR039C-A	VMA10
YHR200W	RPN10	YPL137C	GIP3
YLR452C	SST2	YPR046W	MCM16
YBL051C	PIN4	YML016C	PPZ1
YLR192C	HCR1	YLR441C	RPS1A
YGL066W	SGF73	YDR379W	RGA2
YIL053W	RHR2	YMR230W	RPS10B
YMR116C	ASC1	YBL079W	NUP170
YPR084W	YPR084W	YPL057C	SUR1
YMR127C	SAS2	YDR183W	PLP1
YLR095C	IOC2	YNL307C	MCK1
YMR224C	MRE11	YEL053C	MAK10
YPL024W	RMI1	YPL001W	HAT1
YOR106W	VAM3	YDL006W	PTC1
YHL031C	GOS1	YDR080W	VPS41
YOR270C	VPH1	YML071C	COG8
YDR260C	SWM1	YKL101W	HSL1
YGR133W	PEX4	YML071C	COG8
YGL151W	NUT1	YAL002W	VPS8
YLR119W	SRN2	YEL060C	PRB1
YGL147C	RPL9A	YBR171W	SEC66
YDR293C	SSD1	YNL238W	KEX2
YPL137C	GIP3	YDR207C	UME6
YIL034C	CAP2	YAL002W	VPS8

Predictions at threshold level 0.80 continued...			
Gene1 of predicted pair		Gene2 of predicted pair	
Systematic Name	Standard Name	Systematic Name	Standard Name
YFR036W	CDC26	YER122C	GLO3
YGL087C	MMS2	YMR109W	MYO5
YCL037C	SRO9	YNR032W	PPG1
YBR127C	VMA2	YEL027W	CUP5
YLR386W	VAC14	YOR023C	AHC1
YGR108W	CLB1	YDR363W-A	SEM1
YNL054W	VAC7	YHR115C	DMA1
YHR200W	RPN10	YJR084W	CSN12
YML001W	YPT7	YHR012W	VPS29
YGL124C	MON1	YGR188C	BUB1
YLL042C	ATG10	YPL149W	ATG5
YBL047C	EDE1	YDL047W	SIT4
YBR260C	RGD1	YOR014W	RTS1
YLR192C	HCR1	YKL139W	CTK1
YIR009W	MSL1	YGR188C	BUB1
YKR057W	RPS21A	YBL087C	RPL23A
YNL243W	SLA2	YMR294W	JNM1
YNL288W	CAF40	YIL008W	URM1
YCR086W	CSM1	YDR289C	RTT103
YLR102C	APC9	YKR048C	NAP1
YPL120W	VPS30	YKR030W	GMH1
YJR032W	CPR7	YGR188C	BUB1
YKL181W	PRS1	YPR119W	CLB2
YDR225W	HTA1	YGL163C	RAD54
YPR135W	CTF4	YBR089C-A	NHP6B
YEL044W	IES6	YGL212W	VAM7
YFR010W	UBP6	YDR076W	RAD55
YNL297C	MON2	YAL002W	VPS8
YLR360W	VPS38	YER120W	SCS2

Predictions at threshold level 0.80 continued...			
Gene1 of predicted pair		Gene2 of predicted pair	
Systematic Name	Standard Name	Systematic Name	Standard Name
YNL058C	YNL058C	YER040W	GLN3
YIL053W	RHR2	YLR320W	MMS22
YMR026C	PEX12	YOR270C	VPH1
YGL087C	MMS2	YDR310C	SUM1
YOR106W	VAM3	YHR039C-A	VMA10
YLR150W	STM1	YHR013C	ARD1
YPL022W	RAD1	YDR318W	MCM21
YLR095C	IOC2	YDL223C	HBT1
YPL195W	APL5	YCR053W	THR4
YIR005W	IST3	YPR046W	MCM16
YJL036W	SNX4	YAL030W	SNC1
YPL042C	SSN3	YOL091W	SPO21
YMR109W	MYO5	YKL213C	DOA1
YDR092W	UBC13	YLR032W	RAD5
YMR153W	NUP53	YNL238W	KEX2
YKL161C	YKL161C	YLR425W	TUS1
YJL124C	LSM1	YOR312C	RPL20B
YER001W	MNN1	YKL041W	VPS24
YIL159W	BNR1	YMR026C	PEX12
YDR379W	RGA2	YBL087C	RPL23A
YLR119W	SRN2	YPL037C	EGD1
YCR020C-A	MAK31	YPL161C	BEM4
YMR273C	ZDS1	YHR061C	GIC1
YFR036W	CDC26	YOR327C	SNC2
YGL153W	PEX14	YGL200C	EMP24
YCL037C	SRO9	YEL051W	VMA8
YBL047C	EDE1	YLR182W	SWI6
YMR153W	NUP53	YNL076W	MKS1
YPL234C	TFP3	YOR327C	SNC2

Predictions at threshold level 0.80 continued...			
Gene1 of predicted pair		Gene2 of predicted pair	
Systematic Name	Standard Name	Systematic Name	Standard Name
YDL184C	RPL41A	YPR046W	MCM16
YHR178W	STB5	YOR038C	HIR2
YGL163C	RAD54	YDR076W	RAD55
YKR048C	NAP1	YNL116W	DMA2
YAL024C	LTE1	YOR368W	RAD17
YBR030W	YBR030W	YOR327C	SNC2
YGR159C	NSR1	YGL200C	EMP24
YNL031C	HHT2	YDR071C	PAA1
YBL103C	RTG3	YNL058C	YNL058C
YPL161C	BEM4	YBL087C	RPL23A
YER116C	SLX8	YKR035W-A	DID2
YPR017C	DSS4	YEL051W	VMA8
YAL024C	LTE1	YEL060C	PRB1
YDR144C	MKC7	YBR114W	RAD16
YER087C-B	SBH1	YBR279W	PAF1
YER044C	ERG28	YMR311C	GLC8
YBR107C	IML3	YOL081W	IRA2
YGL124C	MON1	YEL060C	PRB1
YAL024C	LTE1	YKL048C	ELM1
YPL037C	EGD1	YNR072W	HXT17
YNL243W	SLA2	YPL024W	RMI1
YDR123C	INO2	YPL248C	GAL4
YAL024C	LTE1	YJR063W	RPA12
YBL008W	HIR1	YDR378C	LSM6
YPL050C	MNN9	YNL079C	TPM1
YDR142C	PEX7	YDL065C	PEX19
YLR095C	IOC2	YDR207C	UME6
YGL175C	SAE2	YJR035W	RAD26
YER110C	KAP123	YGL124C	MON1

Predictions at threshold level 0.80 continued...			
Gene1 of predicted pair		Gene2 of predicted pair	
Systematic Name	Standard Name	Systematic Name	Standard Name
YKL139W	CTK1	YLR320W	MMS22
YJR035W	RAD26	YNL107W	YAF9
YOR112W	CEX1	YOL018C	TLG2
YKL025C	PAN3	YOR132W	VPS17
YLR119W	SRN2	YJR032W	CPR7
YAL024C	LTE1	YPL195W	APL5
YBR278W	DPB3	YOR038C	HIR2
YDR244W	PEX5	YOL044W	PEX15
YIL034C	CAP2	YHL002W	HSE1
YBR171W	SEC66	YDR207C	UME6
YCL010C	SGF29	YGR252W	GCN5
YMR055C	BUB2	YDR183W	PLP1
YNR072W	HXT17	YDR358W	GGA1
YGR249W	MGA1	YCR053W	THR4
YPL022W	RAD1	YGL031C	RPL24A
YDR440W	DOT1	YKL041W	VPS24
YDL155W	CLB3	YLR131C	ACE2
YHR161C	YAP1801	YOR327C	SNC2
YPL256C	CLN2	YNL116W	DMA2
YPL042C	SSN3	YDR318W	MCM21
YDR289C	RTT103	YOR368W	RAD17
YDL116W	NUP84	YML112W	CTK3
YBR200W	BEM1	YMR032W	HOF1
YDR254W	CHL4	YLR032W	RAD5
YJR084W	CSN12	YOR368W	RAD17
YDL116W	NUP84	YPL178W	CBC2
YPL161C	BEM4	YHL002W	HSE1
YGR105W	VMA21	YAL002W	VPS8
YDR101C	ARX1	YKL068W	NUP100

Predictions at threshold level 0.80 continued...			
**Gene1 of predicted pair**		**Gene2 of predicted pair**	
**Systematic Name**	**Standard Name**	**Systematic Name**	**Standard Name**
YDL160C	DHH1	YML095C	RAD10
YPL018W	CTF19	YJR035W	RAD26
YIL008W	URM1	YPL018W	CTF19
YBR171W	SEC66	YGR188C	BUB1
YBR215W	HPC2	YDR200C	VPS64
YPR017C	DSS4	YOL018C	TLG2
YDR105C	TMS1	YFL044C	OTU1
YKL048C	ELM1	YJL157C	FAR1
YPL115C	BEM3	YDL155W	CLB3
YHR185C	PFS1	YIL007C	NAS2
YDL005C	MED2	YCR084C	TUP1
YDR293C	SSD1	YDR183W	PLP1
YLR360W	VPS38	YOR036W	PEP12
YPR084W	YPR084W	YKL213C	DOA1
YJR084W	CSN12	YMR214W	SCJ1
YER116C	SLX8	YLR150W	STM1
YJL176C	SWI3	YKL041W	VPS24
YJR090C	GRR1	YER027C	GAL83
YDR254W	CHL4	YEL056W	HAT2
YFL003C	MSH4	YLR394W	CST9
YOL121C	RPS19A	YLR093C	NYV1
YGR019W	UGA1	YHR178W	STB5
YOR132W	VPS17	YKR030W	GMH1
YFR031C-A	RPL2A	YPR017C	DSS4
YOR001W	RRP6	YLR429W	CRN1
YCR086W	CSM1	YGR188C	BUB1
YPL022W	RAD1	YKR030W	GMH1
YMR224C	MRE11	YNL116W	DMA2
YNL116W	DMA2	YHR115C	DMA1

Predictions at threshold level 0.80 continued...			
Gene1 of predicted pair		Gene2 of predicted pair	
Systematic Name	Standard Name	Systematic Name	Standard Name
YJR024C	MDE1	YOR358W	HAP5
YOR106W	VAM3	YNL079C	TPM1
YPL195W	APL5	YMR214W	SCJ1
YOL090W	MSH2	YGL031C	RPL24A
YER087C-B	SBH1	YBR200W	BEM1
YDL184C	RPL41A	YNL307C	MCK1
YER116C	SLX8	YPL161C	BEM4
YCR002C	CDC10	YDR507C	GIN4
YMR004W	MVP1	YER162C	RAD4
YDL160C	DHH1	YDR253C	MET32
YER044C	ERG28	YDL077C	VAM6
YMR153W	NUP53	YHR200W	RPN10
YGL124C	MON1	YMR230W	RPS10B
YPL120W	VPS30	YDR207C	UME6
YLR292C	SEC72	YML095C	RAD10
YJR084W	CSN12	YDL077C	VAM6
YHR060W	VMA22	YER120W	SCS2
YOL147C	PEX11	YNL117W	MLS1
YDL223C	HBT1	YOR035C	SHE4
YDR245W	MNN10	YKL126W	YPK1
YDR358W	GGA1	YBR280C	SAF1
YGL078C	DBP3	YGL003C	CDH1
YLR181C	VTA1	YKR035W-A	DID2
YNL032W	SIW14	YLR398C	SKI2
YHR205W	SCH9	YDR388W	RVS167
YPR167C	MET16	YML028W	TSA1
YGR217W	CCH1	YKR020W	VPS51
YOR035C	SHE4	YKL041W	VPS24
YJL190C	RPS22A	YGR188C	BUB1

Predictions at threshold level 0.80 continued...			
Gene1 of predicted pair		Gene2 of predicted pair	
Systematic Name	Standard Name	Systematic Name	Standard Name
YKL032C	IXR1	YDR440W	DOT1
YDR156W	RPA14	YNR052C	POP2
YMR224C	MRE11	YJL172W	CPS1
YLR079W	SIC1	YBL047C	EDE1
YDR153C	ENT5	YDR207C	UME6
YDL006W	PTC1	YEL051W	VMA8
YJL127C	SPT10	YLR429W	CRN1
YPL152W	RRD2	YJR135C	MCM22
YDL116W	NUP84	YJR063W	RPA12
YMR153W	NUP53	YMR016C	SOK2
YOR290C	SNF2	YJR032W	CPR7
YOR083W	WHI5	YJR135C	MCM22
YNL087W	TCB2	YDR137W	RGP1
YJR106W	ECM27	YKR093W	PTR2
YGL190C	CDC55	YJL187C	SWE1
YBR200W	BEM1	YNL307C	MCK1
YKL139W	CTK1	YGL212W	VAM7
YBR093C	PHO5	YOR368W	RAD17
YGL212W	VAM7	YCR053W	THR4
YLR399C	BDF1	YPL152W	RRD2
YNL291C	MID1	YJL095W	BCK1
YCR086W	CSM1	YNL116W	DMA2
YMR016C	SOK2	YJL183W	MNN11
YLR309C	IMH1	YKR030W	GMH1
YHR111W	UBA4	YOR275C	RIM20
YMR172W	HOT1	YAL002W	VPS8
YCL037C	SRO9	YJL004C	SYS1
YER120W	SCS2	YEL051W	VMA8
YFR010W	UBP6	YDR245W	MNN10

Predictions at threshold level 0.80 continued...			
Gene1 of predicted pair		Gene2 of predicted pair	
Systematic Name	Standard Name	Systematic Name	Standard Name
YGL060W	YBP2	YML095C	RAD10
YEL043W	YEL043W	YEL051W	VMA8
YLR079W	SIC1	YAR014C	BUD14
YLR337C	VRP1	YPR051W	MAK3
YJL006C	CTK2	YLR425W	TUS1
YGR104C	SRB5	YJL124C	LSM1
YLR191W	PEX13	YDR244W	PEX5
YGL060W	YBP2	YJR005W	APL1
YBL067C	UBP13	YOR035C	SHE4
YOR023C	AHC1	YJR135C	MCM22
YMR133W	REC114	YDL083C	RPS16B
YPL129W	TAF14	YHL025W	SNF6
YDR378C	LSM6	YKL048C	ELM1
YMR311C	GLC8	YCR053W	THR4
YJR005W	APL1	YPL018W	CTF19
YMR055C	BUB2	YGL019W	CKB1
YEL065W	SIT1	YJR032W	CPR7
YMR153W	NUP53	YOR043W	WHI2
YBR215W	HPC2	YML102W	CAC2
YGR241C	YAP1802	YBL047C	EDE1
YFL023W	BUD27	YDR207C	UME6
YNR052C	POP2	YIL036W	CST6
YHR060W	VMA22	YDL006W	PTC1
YGL087C	MMS2	YDR363W-A	SEM1
YOL145C	CTR9	YNL107W	YAF9
YNL243W	SLA2	YNL098C	RAS2
YKL006W	RPL14A	YOL081W	IRA2
YDR335W	MSN5	YOL054W	PSH1
YGR252W	GCN5	YDR469W	SDC1

Predictions at threshold level 0.80 continued...			
Gene1 of predicted pair		Gene2 of predicted pair	
Systematic Name	Standard Name	Systematic Name	Standard Name
YPR018W	RLF2	YDL005C	MED2
YMR198W	CIK1	YML097C	VPS9
YGL078C	DBP3	YPL137C	GIP3
YDR004W	RAD57	YPR036W	VMA13
YKL025C	PAN3	YDR318W	MCM21
YOR337W	TEA1	YGL212W	VAM7
YBR200W	BEM1	YBR114W	RAD16
YDR156W	RPA14	YNL054W	VAC7
YOR043W	WHI2	YFR036W	CDC26
YBR108W	YBR108W	YIL008W	URM1
YHR111W	UBA4	YJR090C	GRR1
YKR061W	KTR2	YJL006C	CTK2
YKL135C	APL2	YJR058C	APS2
YNR064C	YNR064C	YOL108C	INO4
YPL022W	RAD1	YMR231W	PEP5
YBR171W	SEC66	YDR335W	MSN5
YER044C	ERG28	YLR398C	SKI2
YNL087W	TCB2	YKR035W-A	DID2
YIL156W	UBP7	YMR164C	MSS11
YLR234W	TOP3	YAL015C	NTG1
YMR153W	NUP53	YDL083C	RPS16B
YNL058C	YNL058C	YML071C	COG8
YGL178W	MPT5	YAL040C	CLN3
YDR363W-A	SEM1	YLR410W	VIP1
YGL161C	YIP5	YKR001C	VPS1
YOR080W	DIA2	YDR289C	RTT103
YPL253C	VIK1	YKL101W	HSL1
YLR410W	VIP1	YGL031C	RPL24A
YKL032C	IXR1	YJL006C	CTK2

Predictions at threshold level 0.80 continued...			
Gene1 of predicted pair		Gene2 of predicted pair	
Systematic Name	Standard Name	Systematic Name	Standard Name
YMR026C	PEX12	YHR200W	RPN10
YBL008W	HIR1	YOR080W	DIA2
YHL031C	GOS1	YGR020C	VMA7
YDL002C	NHP10	YOR308C	SNU66
YLR360W	VPS38	YPL161C	BEM4
YMR224C	MRE11	YNL117W	MLS1
YGL133W	ITC1	YHR152W	SPO12
YOR001W	RRP6	YMR125W	STO1
YDR156W	RPA14	YMR055C	BUB2
YNL041C	COG6	YML071C	COG8
YPL037C	EGD1	YKR030W	GMH1
YLR337C	VRP1	YDR155C	CPR1
YMR153W	NUP53	YLR180W	SAM1
YPL115C	BEM3	YPL018W	CTF19
YDR379W	RGA2	YJL187C	SWE1
YEL043W	YEL043W	YJL004C	SYS1
YLR371W	ROM2	YER049W	TPA1
YKR020W	VPS51	YAR014C	BUD14
YFL023W	BUD27	YDL077C	VAM6
YNL325C	FIG4	YKL048C	ELM1
YDR174W	HMO1	YEL027W	CUP5
YOR080W	DIA2	YNL016W	PUB1
YGR072W	UPF3	YNL041C	COG6
YPL161C	BEM4	YMR038C	CCS1
YIR009W	MSL1	YCR077C	PAT1
YER110C	KAP123	YHR111W	UBA4
YOR290C	SNF2	YER044C	ERG28
YPL240C	HSP82	YGL096W	TOS8
YDL101C	DUN1	YOL030W	GAS5

Predictions at threshold level 0.80 continued...			
Gene1 of predicted pair		Gene2 of predicted pair	
Systematic Name	Standard Name	Systematic Name	Standard Name
YJL154C	VPS35	YOR140W	SFL1
YKL160W	ELF1	YCR077C	PAT1
YGL200C	EMP24	YOL081W	IRA2
YHR200W	RPN10	YGL115W	SNF4
YFL013C	IES1	YHL031C	GOS1
YDR363W-A	SEM1	YKL101W	HSL1
YHR114W	BZZ1	YHL002W	HSE1
YPL024W	RMI1	YDR137W	RGP1
YLR362W	STE11	YDL159W	STE7
YHL031C	GOS1	YDR245W	MNN10
YDR004W	RAD57	YML013W	UBX2
YPR120C	CLB5	YDL155W	CLB3
YMR198W	CIK1	YHL027W	RIM101
YER110C	KAP123	YLR399C	BDF1
YOR038C	HIR2	YLR182W	SWI6
YLR094C	GIS3	YNL076W	MKS1
YKL048C	ELM1	YNL289W	PCL1
YIL076W	SEC28	YER161C	SPT2
YKL139W	CTK1	YBR215W	HPC2
YLR381W	CTF3	YEL053C	MAK10
YNL291C	MID1	YPL037C	EGD1
YMR091C	NPL6	YLR357W	RSC2
YOL013C	HRD1	YMR022W	UBC7
YLR234W	TOP3	YOR043W	WHI2
YPR131C	NAT3	YOR195W	SLK19
YKL006W	RPL14A	YJL183W	MNN11
YOR212W	STE4	YDR183W	PLP1
YOR337W	TEA1	YLR192C	HCR1
YJR021C	REC107	YMR133W	REC114

Predictions at threshold level 0.80 continued...			
Gene1 of predicted pair		Gene2 of predicted pair	
Systematic Name	Standard Name	Systematic Name	Standard Name
YDL020C	RPN4	YJR060W	CBF1
YOR106W	VAM3	YJR060W	CBF1
YDL002C	NHP10	YOR124C	UBP2
YIL034C	CAP2	YOR358W	HAP5
YMR153W	NUP53	YER115C	SPR6
YMR172W	HOT1	YBR108W	YBR108W
YLR234W	TOP3	YMR231W	PEP5
YGR133W	PEX4	YKR020W	VPS51
YNL243W	SLA2	YNL250W	RAD50
YHR156C	LIN1	YDL192W	ARF1
YJL078C	PRY3	YPR149W	NCE102
YLR090W	XDJ1	YPR030W	CSR2
YJR056C	YJR056C	YPL137C	GIP3
YPL022W	RAD1	YDL226C	GCS1
YMR109W	MYO5	YGL151W	NUT1
YFR010W	UBP6	YDR335W	MSN5
YCL037C	SRO9	YPL120W	VPS30
YOL061W	PRS5	YOR043W	WHI2
YML013W	UBX2	YER162C	RAD4
YMR179W	SPT21	YDL047W	SIT4
YEL065W	SIT1	YPR046W	MCM16
YGL003C	CDH1	YLR370C	ARC18
YBR194W	SOY1	YCR077C	PAT1
YLR320W	MMS22	YJL183W	MNN11
YPL105C	YPL105C	YEL043W	YEL043W
YLR192C	HCR1	YKL126W	YPK1
YFR021W	ATG18	YPR119W	CLB2
YPL096C-A	ERI1	YEL036C	ANP1
YMR109W	MYO5	YAL002W	VPS8

Predictions at threshold level 0.80 continued...			
Gene1 of predicted pair		Gene2 of predicted pair	
Systematic Name	Standard Name	Systematic Name	Standard Name
YHR039C-A	VMA10	YDR153C	ENT5
YLR360W	VPS38	YPL018W	CTF19
YLR360W	VPS38	YOR008C	SLG1
YDR261C	EXG2	YFR036W	CDC26
YML071C	COG8	YJR084W	CSN12
YBL103C	RTG3	YER139C	YER139C
YNL106C	INP52	YNL054W	VAC7
YIL156W	UBP7	YJR059W	PTK2
YHR178W	STB5	YKL048C	ELM1
YGR054W	YGR054W	YHL002W	HSE1
YOR080W	DIA2	YCR077C	PAT1
YFR036W	CDC26	YDR440W	DOT1
YCL001W	RER1	YLR309C	IMH1
YML103C	NUP188	YGL163C	RAD54
YGL025C	PGD1	YNL307C	MCK1
YOR368W	RAD17	YDR207C	UME6
YJL187C	SWE1	YBR082C	UBC4
YOR127W	RGA1	YEL053C	MAK10
YGL223C	COG1	YML067C	ERV41
YLL007C	YLL007C	YLR234W	TOP3
YBR107C	IML3	YIL008W	URM1
YLR192C	HCR1	YDR137W	RGP1
YDR225W	HTA1	YNL030W	HHF2
YBL078C	ATG8	YNR007C	ATG3
YDR477W	SNF1	YJR056C	YJR056C
YLR221C	RSA3	YKR024C	DBP7
YMR202W	ERG2	YJR135C	MCM22
YGR108W	CLB1	YMR109W	MYO5
YMR199W	CLN1	YDR264C	AKR1

Predictions at threshold level 0.80 continued...			
Gene1 of predicted pair		Gene2 of predicted pair	
Systematic Name	Standard Name	Systematic Name	Standard Name
YDR108W	GSG1	YJR135C	MCM22
YML117W	NAB6	YGR217W	CCH1
YJL124C	LSM1	YIL103W	DPH1
YJL138C	TIF2	YLR350W	ORM2
YFR024C-A	LSB3	YGR268C	HUA1
YMR016C	SOK2	YDR207C	UME6
YER087C-B	SBH1	YGL226C-A	OST5
YFR031C-A	RPL2A	YNR072W	HXT17
YMR016C	SOK2	YKR099W	BAS1
YPL018W	CTF19	YNL147W	LSM7
YNL206C	RTT106	YDR264C	AKR1
YNL106C	INP52	YHR108W	GGA2
YDR156W	RPA14	YML016C	PPZ1
YKL025C	PAN3	YGL237C	HAP2
YDR192C	NUP42	YDR207C	UME6
YDR484W	VPS52	YJR032W	CPR7
YMR142C	RPL13B	YDR137W	RGP1
YML038C	YMD8	YPL195W	APL5
YKL116C	PRR1	YLR371W	ROM2
YBR200W	BEM1	YIL008W	URM1
YPL022W	RAD1	YJL187C	SWE1
YDR392W	SPT3	YBR089C-A	NHP6B
YGL212W	VAM7	YKR020W	VPS51
YOR001W	RRP6	YML016C	PPZ1
YNL288W	CAF40	YMR109W	MYO5
YLR170C	APS1	YKL135C	APL2
YML103C	NUP188	YLR182W	SWI6
YBR171W	SEC66	YEL056W	HAT2
YMR199W	CLN1	YGL163C	RAD54

Predictions at threshold level 0.80 continued...			
Gene1 of predicted pair		Gene2 of predicted pair	
Systematic Name	Standard Name	Systematic Name	Standard Name
YLR386W	VAC14	YBR084C-A	RPL19A
YKL025C	PAN3	YPR046W	MCM16
YDL042C	SIR2	YDR227W	SIR4
YER116C	SLX8	YER044C	ERG28
YEL065W	SIT1	YBR280C	SAF1
YPL057C	SUR1	YKL101W	HSL1
YNL288W	CAF40	YPL253C	VIK1
YOR106W	VAM3	YJR040W	GEF1
YLR398C	SKI2	YER040W	GLN3
YDR146C	SWI5	YBR215W	HPC2
YNL206C	RTT106	YDL185W	TFP1
YLR360W	VPS38	YOR069W	VPS5
YPL018W	CTF19	YGL252C	RTG2
YLR381W	CTF3	YNL236W	SIN4
YLR292C	SEC72	YHR134W	WSS1
YMR224C	MRE11	YJR084W	CSN12
YDL223C	HBT1	YNL041C	COG6
YFR010W	UBP6	YOR066W	MSA1
YLR441C	RPS1A	YOR035C	SHE4
YNL215W	IES2	YLR398C	SKI2
YJL154C	VPS35	YNL199C	GCR2
YJL172W	CPS1	YPL161C	BEM4
YDR137W	RGP1	YNL107W	YAF9
YHR111W	UBA4	YAL002W	VPS8
YLR144C	ACF2	YOR284W	HUA2
YJL172W	CPS1	YGL115W	SNF4
YOR140W	SFL1	YNL079C	TPM1
YDR130C	FIN1	YLR254C	NDL1
YOR290C	SNF2	YNL107W	YAF9

Predictions at threshold level 0.80 continued...			
Gene1 of predicted pair		Gene2 of predicted pair	
Systematic Name	Standard Name	Systematic Name	Standard Name
YDR439W	LRS4	YDR378C	LSM6
YJR031C	GEA1	YDL137W	ARF2
YGL087C	MMS2	YHR178W	STB5
YPL161C	BEM4	YHR012W	VPS29
YER144C	UBP5	YPR030W	CSR2
YMR026C	PEX12	YJR056C	YJR056C
YHR134W	WSS1	YBR200W	BEM1
YGL087C	MMS2	YGR188C	BUB1
YER110C	KAP123	YPL253C	VIK1
YMR224C	MRE11	YML095C	RAD10
YLR360W	VPS38	YMR231W	PEP5
YLR150W	STM1	YDR254W	CHL4
YOR106W	VAM3	YHL020C	OPI1
YDL223C	HBT1	YLR320W	MMS22
YOL111C	MDY2	YBR019C	GAL10
YOR337W	TEA1	YLR371W	ROM2
YPL018W	CTF19	YDL226C	GCS1
YDR484W	VPS52	YNL238W	KEX2
YHR200W	RPN10	YDL006W	PTC1
YNL166C	BNI5	YGR188C	BUB1
YBL103C	RTG3	YCR053W	THR4
YBR112C	CYC8	YPR068C	HOS1
YMR224C	MRE11	YPL022W	RAD1
YDR378C	LSM6	YPR120C	CLB5
YDL155W	CLB3	YKL101W	HSL1
YFR010W	UBP6	YCR086W	CSM1
YBR217W	ATG12	YLR180W	SAM1
YDL116W	NUP84	YPL152W	RRD2
YOR270C	VPH1	YKR099W	BAS1

Predictions at threshold level 0.80 continued...			
**Gene1 of predicted pair**		**Gene2 of predicted pair**	
**Systematic Name**	**Standard Name**	**Systematic Name**	**Standard Name**
YGL043W	DST1	YHR056C	RSC30
YBL058W	SHP1	YOL081W	IRA2
YNL253W	TEX1	YHR167W	THP2
YNL333W	SNZ2	YMR096W	SNZ1
YDL125C	HNT1	YNL236W	SIN4
YDR080W	VPS41	YPR173C	VPS4
YKR035W-A	DID2	YLR371W	ROM2
YEL050C	RML2	YGL003C	CDH1
YGL033W	HOP2	YDR484W	VPS52
YDL002C	NHP10	YHR200W	RPN10
YDL077C	VAM6	YLR398C	SKI2
YNL199C	GCR2	YMR038C	CCS1
YML038C	YMD8	YKR030W	GMH1
YDR285W	ZIP1	YLR394W	CST9
YKL139W	CTK1	YLR254C	NDL1
YDR245W	MNN10	YDR289C	RTT103
YLR381W	CTF3	YKL048C	ELM1
YDR484W	VPS52	YHR200W	RPN10
YOR035C	SHE4	YBR280C	SAF1
YLR058C	SHM2	YGL003C	CDH1
YIL053W	RHR2	YGL033W	HOP2
YDR137W	RGP1	YGL200C	EMP24
YAL024C	LTE1	YNR052C	POP2
YKL116C	PRR1	YNL076W	MKS1
YHR128W	FUR1	YPL240C	HSP82
YDL192W	ARF1	YDR310C	SUM1
YLR083C	EMP70	YPL037C	EGD1
YJR135C	MCM22	YGL003C	CDH1
YPL057C	SUR1	YDR440W	DOT1

Predictions at threshold level 0.80 continued...			
Gene1 of predicted pair		Gene2 of predicted pair	
Systematic Name	Standard Name	Systematic Name	Standard Name
YNL215W	IES2	YGR014W	MSB2
YMR255W	GFD1	YIL033C	BCY1
YER114C	BOI2	YNL116W	DMA2
YMR199W	CLN1	YDR245W	MNN10
YER001W	MNN1	YEL044W	IES6
YKR001C	VPS1	YPL105C	YPL105C
YLL007C	YLL007C	YHR134W	WSS1
YPR017C	DSS4	YKR020W	VPS51
YHR152W	SPO12	YNL116W	DMA2
YHR084W	STE12	YLR362W	STE11
YHR060W	VMA22	YLR320W	MMS22
YBR215W	HPC2	YPL106C	SSE1
YHR039C-A	VMA10	YBL025W	RRN10
YNL058C	YNL058C	YNR072W	HXT17
YNL243W	SLA2	YNR051C	BRE5
YDR202C	RAV2	YNL044W	YIP3
YDR363W-A	SEM1	YLR182W	SWI6
YDR183W	PLP1	YLR182W	SWI6
YFR010W	UBP6	YLR192C	HCR1
YAL020C	ATS1	YLR425W	TUS1
YBR200W	BEM1	YBR084C-A	RPL19A
YMR153W	NUP53	YNL058C	YNL058C
YJL154C	VPS35	YML001W	YPT7
YBR200W	BEM1	YOR140W	SFL1
YPR017C	DSS4	YOR127W	RGA1
YOR127W	RGA1	YGR105W	VMA21
YML103C	NUP188	YMR273C	ZDS1
YDR335W	MSN5	YML112W	CTK3
YPL161C	BEM4	YBL085W	BOI1

Predictions at threshold level 0.80 continued...			
Gene1 of predicted pair		Gene2 of predicted pair	
Systematic Name	Standard Name	Systematic Name	Standard Name
YLR083C	EMP70	YGL115W	SNF4
YBR030W	YBR030W	YNL087W	TCB2
YMR224C	MRE11	YOR337W	TEA1
YBL047C	EDE1	YDR264C	AKR1
YMR198W	CIK1	YBR171W	SEC66
YHR056C	RSC30	YKL057C	NUP120
YMR089C	YTA12	YEL036C	ANP1
YHR178W	STB5	YOR358W	HAP5
YCL001W	RER1	YDR310C	SUM1
YLR090W	XDJ1	YMR224C	MRE11
YEL053C	MAK10	YLR371W	ROM2
YPL042C	SSN3	YEL013W	VAC8
YFR010W	UBP6	YBR171W	SEC66
YCR020C-A	MAK31	YDR293C	SSD1
YCR024C-A	PMP1	YKR024C	DBP7
YJL183W	MNN11	YKL041W	VPS24
YMR224C	MRE11	YGL200C	EMP24
YOR106W	VAM3	YER122C	GLO3
YFR031C-A	RPL2A	YJR033C	RAV1
YDR507C	GIN4	YGR135W	PRE9
YFL023W	BUD27	YML095C	RAD10
YLR320W	MMS22	YPL089C	RLM1
YNL218W	MGS1	YMR116C	ASC1
YGR104C	SRB5	YBR020W	GAL1
YML112W	CTK3	YNL107W	YAF9
YHL031C	GOS1	YML001W	YPT7
YJR140C	HIR3	YEL056W	HAT2
YOR106W	VAM3	YEL051W	VMA8
YDR439W	LRS4	YOL081W	IRA2

Predictions at threshold level 0.80 continued...			
Gene1 of predicted pair		Gene2 of predicted pair	
Systematic Name	Standard Name	Systematic Name	Standard Name
YER144C	UBP5	YBR019C	GAL10
YPL195W	APL5	YDR207C	UME6
YDR358W	GGA1	YLR206W	ENT2
YJL122W	ALB1	YDR101C	ARX1
YJL176C	SWI3	YML097C	VPS9
YBL008W	HIR1	YML016C	PPZ1
YBR215W	HPC2	YEL013W	VAC8
YLR360W	VPS38	YDL155W	CLB3
YER116C	SLX8	YBR108W	YBR108W
YJL154C	VPS35	YBR108W	YBR108W
YPR017C	DSS4	YNR049C	MSO1
YGL147C	RPL9A	YOL121C	RPS19A
YNL069C	RPL16B	YBL102W	SFT2
YNL238W	KEX2	YHR081W	LRP1
YDR146C	SWI5	YEL036C	ANP1
YGL078C	DBP3	YDR484W	VPS52
YPL195W	APL5	YDR153C	ENT5
YML115C	VAN1	YAL002W	VPS8
YBL047C	EDE1	YPL256C	CLN2
YKR059W	TIF1	YGR188C	BUB1
YIL133C	RPL16A	YOR208W	PTP2
YNL041C	COG6	YNL127W	FAR11
YLL039C	UBI4	YGL200C	EMP24
YIL148W	RPL40A	YGL016W	KAP122
YNL238W	KEX2	YDR207C	UME6
YFR031C-A	RPL2A	YER144C	UBP5
YMR231W	PEP5	YOR035C	SHE4
YLR441C	RPS1A	YKL048C	ELM1
YNL215W	IES2	YIL036W	CST6

Predictions at threshold level 0.80 continued...			
Gene1 of predicted pair		Gene2 of predicted pair	
Systematic Name	Standard Name	Systematic Name	Standard Name
YLR360W	VPS38	YMR127C	SAS2
YFL013C	IES1	YDR137W	RGP1
YFR031C-A	RPL2A	YNL032W	SIW14
YJR094C	IME1	YDR207C	UME6
YDR225W	HTA1	YNL215W	IES2
YBL102W	SFT2	YGL223C	COG1
YAL024C	LTE1	YGL038C	OCH1
YMR004W	MVP1	YOR327C	SNC2
YER044C	ERG28	YGL003C	CDH1
YGL066W	SGF73	YPL256C	CLN2
YLR006C	SSK1	YDR293C	SSD1
YDL020C	RPN4	YMR198W	CIK1
YER116C	SLX8	YLR032W	RAD5
YOR043W	WHI2	YER040W	GLN3
YER120W	SCS2	YML001W	YPT7
YLR090W	XDJ1	YKR035W-A	DID2
YDR129C	SAC6	YCR077C	PAT1
YDR225W	HTA1	YDR318W	MCM21
YOR008C	SLG1	YEL036C	ANP1
YDR477W	SNF1	YJR084W	CSN12
YLR233C	EST1	YGR188C	BUB1
YGL135W	RPL1B	YDR129C	SAC6
YPL022W	RAD1	YLR352W	YLR352W
YCR077C	PAT1	YOR038C	HIR2
YHR156C	LIN1	YNL307C	MCK1
YKR035W-A	DID2	YER139C	YER139C
YKL139W	CTK1	YDL083C	RPS16B
YNL107W	YAF9	YAL002W	VPS8
YMR214W	SCJ1	YLR180W	SAM1

Predictions at threshold level 0.80 continued...			
Gene1 of predicted pair		Gene2 of predicted pair	
Systematic Name	Standard Name	Systematic Name	Standard Name
YHR010W	RPL27A	YML028W	TSA1
YGR019W	UGA1	YMR127C	SAS2
YPR036W	VMA13	YBL025W	RRN10
YGL163C	RAD54	YCR086W	CSM1
YDL006W	PTC1	YER118C	SHO1
YKL025C	PAN3	YGL151W	NUT1
YDR363W-A	SEM1	YER161C	SPT2
YIL132C	CSM2	YHL006C	SHU1
YOR270C	VPH1	YGR020C	VMA7
YHR108W	GGA2	YJL095W	BCK1
YML115C	VAN1	YAL055W	PEX22
YNL087W	TCB2	YMR214W	SCJ1
YHR161C	YAP1801	YGL095C	VPS45
YBR173C	UMP1	YKL126W	YPK1
YLR288C	MEC3	YLR320W	MMS22
YNL297C	MON2	YMR038C	CCS1
YDL020C	RPN4	YOR243C	PUS7
YBR194W	SOY1	YDL006W	PTC1
YOR327C	SNC2	YMR214W	SCJ1
YNL291C	MID1	YPR046W	MCM16
YGL100W	SEH1	YMR048W	CSM3
YGR108W	CLB1	YAR014C	BUD14
YJL058C	BIT61	YJL006C	CTK2
YGL212W	VAM7	YBR171W	SEC66
YOR127W	RGA1	YDR379W	RGA2
YGL212W	VAM7	YJL004C	SYS1
YJR084W	CSN12	YLR398C	SKI2
YKR020W	VPS51	YJL117W	PHO86
YCL027W	FUS1	YPR119W	CLB2

Predictions at threshold level 0.80 continued...			
Gene1 of predicted pair		Gene2 of predicted pair	
Systematic Name	Standard Name	Systematic Name	Standard Name
YOL121C	RPS19A	YML071C	COG8
YKL213C	DOA1	YOR124C	UBP2
YGR104C	SRB5	YPL129W	TAF14
YDR108W	GSG1	YOR327C	SNC2
YHR039C-A	VMA10	YKL041W	VPS24
YPR030W	CSR2	YPR046W	MCM16
YNL307C	MCK1	YDL192W	ARF1
YPL057C	SUR1	YJR032W	CPR7
YBR108W	YBR108W	YEL060C	PRB1
YER139C	YER139C	YOR327C	SNC2
YMR042W	ARG80	YDR207C	UME6
YKR048C	NAP1	YHR115C	DMA1
YPR051W	MAK3	YPR046W	MCM16
YCR053W	THR4	YBR036C	CSG2
YGR019W	UGA1	YBR114W	RAD16
YMR109W	MYO5	YKL048C	ELM1
YLR381W	CTF3	YHR111W	UBA4
YMR224C	MRE11	YFR010W	UBP6
YHR205W	SCH9	YER049W	TPA1
YML115C	VAN1	YDL077C	VAM6
YPR120C	CLB5	YKL114C	APN1
YER087C-B	SBH1	YGL058W	RAD6
YGR270W	YTA7	YIL103W	DPH1
YMR016C	SOK2	YER040W	GLN3
YLR399C	BDF1	YAR014C	BUD14
YLR337C	VRP1	YNL166C	BNI5
YGL078C	DBP3	YER144C	UBP5
YOR358W	HAP5	YMR055C	BUB2
YBR200W	BEM1	YLR094C	GIS3

Predictions at threshold level 0.80 continued...			
Gene1 of predicted pair		Gene2 of predicted pair	
Systematic Name	Standard Name	Systematic Name	Standard Name
YJL053W	PEP8	YOR036W	PEP12
YJR140C	HIR3	YGR071C	YGR071C
YLR386W	VAC14	YKL048C	ELM1
YGR019W	UGA1	YBL021C	HAP3
YGR270W	YTA7	YMR038C	CCS1
YDR004W	RAD57	YPL024W	RMI1
YMR284W	YKU70	YDL013W	SLX5
YLR083C	EMP70	YJR033C	RAV1
YCL037C	SRO9	YGR071C	YGR071C
YML115C	VAN1	YPL089C	RLM1
YNR072W	HXT17	YEL053C	MAK10
YBR200W	BEM1	YFR040W	SAP155
YLR441C	RPS1A	YOR043W	WHI2
YOR132W	VPS17	YOL108C	INO4
YBL058W	SHP1	YDR310C	SUM1
YMR026C	PEX12	YDR207C	UME6
YDL006W	PTC1	YAL030W	SNC1
YNL106C	INP52	YGR072W	UPF3
YKL139W	CTK1	YKL048C	ELM1
YLR043C	TRX1	YOR106W	VAM3
YDR335W	MSN5	YDR378C	LSM6
YGL078C	DBP3	YML071C	COG8
YKR001C	VPS1	YJL004C	SYS1
YIL076W	SEC28	YLR452C	SST2
YFR010W	UBP6	YGL115W	SNF4
YKR095W	MLP1	YDR207C	UME6
YOR195W	SLK19	YCR086W	CSM1
YHR178W	STB5	YGL163C	RAD54
YJL078C	PRY3	YOR071C	THI71

Predictions at threshold level 0.80 continued...			
Gene1 of predicted pair		Gene2 of predicted pair	
Systematic Name	Standard Name	Systematic Name	Standard Name
YDR137W	RGP1	YBR127C	VMA2
YJR056C	YJR056C	YKR099W	BAS1
YLR386W	VAC14	YGL003C	CDH1
YOR008C	SLG1	YNL030W	HHF2
YLR090W	XDJ1	YER116C	SLX8
YDR080W	VPS41	YGL054C	ERV14
YLR095C	IOC2	YFR036W	CDC26
YDL020C	RPN4	YEL056W	HAT2
YOR337W	TEA1	YJR094C	IME1
YML115C	VAN1	YBL047C	EDE1
YLR079W	SIC1	YER049W	TPA1
YML115C	VAN1	YNR072W	HXT17
YDR225W	HTA1	YMR038C	CCS1
YDR379W	RGA2	YHR200W	RPN10
YMR026C	PEX12	YNL214W	PEX17
YNL153C	GIM3	YHR056C	RSC30
YBR200W	BEM1	YBR260C	RGD1
YKL032C	IXR1	YDL047W	SIT4
YBL051C	PIN4	YHR114W	BZZ1
YNL206C	RTT106	YGL163C	RAD54
YJL060W	BNA3	YOR080W	DIA2
YGL212W	VAM7	YDL223C	HBT1
YOR001W	RRP6	YNL107W	YAF9
YDR383C	NKP1	YDR156W	RPA14
YDR156W	RPA14	YBR200W	BEM1
YCR020C-A	MAK31	YCL001W	RER1
YGR108W	CLB1	YOR001W	RRP6
YPR040W	TIP41	YHR115C	DMA1
YPR171W	BSP1	YDR245W	MNN10

Predictions at threshold level 0.80 continued...			
Gene1 of predicted pair		Gene2 of predicted pair	
Systematic Name	Standard Name	Systematic Name	Standard Name
YLR148W	PEP3	YJL029C	VPS53
YMR205C	PFK2	YDL006W	PTC1
YPL195W	APL5	YDL192W	ARF1
YER096W	SHC1	YEL036C	ANP1
YDR245W	MNN10	YCR088W	ABP1
YDR245W	MNN10	YBR131W	CCZ1
YHR013C	ARD1	YFR030W	MET10
YML038C	YMD8	YML071C	COG8
YDR156W	RPA14	YKL139W	CTK1
YML038C	YMD8	YDL077C	VAM6
YPL240C	HSP82	YBL071W-A	KTI11
YLR320W	MMS22	YJR084W	CSN12
YFR031C-A	RPL2A	YGL054C	ERV14
YAL024C	LTE1	YLR095C	IOC2
YOR033C	EXO1	YML102W	CAC2
YHR156C	LIN1	YDR358W	GGA1
YBR107C	IML3	YLR032W	RAD5
YOR275C	RIM20	YDL006W	PTC1
YMR026C	PEX12	YLR094C	GIS3
YML095C	RAD10	YLR447C	VMA6
YER161C	SPT2	YIR005W	IST3
YBR188C	NTC20	YPL213W	LEA1
YDL116W	NUP84	YFR036W	CDC26
YPL161C	BEM4	YGR152C	RSR1
YLR192C	HCR1	YLR094C	GIS3
YBL019W	APN2	YOL043C	NTG2
YPL001W	HAT1	YBR036C	CSG2
YKL048C	ELM1	YHR013C	ARD1
YPL161C	BEM4	YDR310C	SUM1

Predictions at threshold level 0.80 continued...			
Gene1 of predicted pair		Gene2 of predicted pair	
Systematic Name	Standard Name	Systematic Name	Standard Name
YDR335W	MSN5	YLR254C	NDL1
YMR199W	CLN1	YER096W	SHC1
YKR057W	RPS21A	YHR115C	DMA1
YAL024C	LTE1	YIL076W	SEC28
YDL002C	NHP10	YEL056W	HAT2
YML001W	YPT7	YNL127W	FAR11
YFR031C-A	RPL2A	YML001W	YPT7
YJR033C	RAV1	YLR309C	IMH1
YKL139W	CTK1	YDR440W	DOT1
YIL076W	SEC28	YER122C	GLO3
YCR088W	ABP1	YLR396C	VPS33
YDL020C	RPN4	YKL101W	HSL1
YNL248C	RPA49	YDR378C	LSM6
YMR224C	MRE11	YBR171W	SEC66
YKL213C	DOA1	YNL107W	YAF9
YJL095W	BCK1	YEL056W	HAT2
YER144C	UBP5	YKR035W-A	DID2
YPL024W	RMI1	YMR230W	RPS10B
YBR107C	IML3	YNL307C	MCK1
YNL288W	CAF40	YDR318W	MCM21
YDL190C	UFD2	YMR276W	DSK2
YOR358W	HAP5	YEL056W	HAT2
YLR090W	XDJ1	YDR310C	SUM1
YPL024W	RMI1	YCR086W	CSM1
YGL147C	RPL9A	YOR327C	SNC2
YMR133W	REC114	YDR261C	EXG2
YGL212W	VAM7	YGL054C	ERV14
YKL007W	CAP1	YGL124C	MON1
YOR195W	SLK19	YDR200C	VPS64

Predictions at threshold level 0.80 continued...			
Gene1 of predicted pair		Gene2 of predicted pair	
Systematic Name	Standard Name	Systematic Name	Standard Name
YDR389W	SAC7	YLR429W	CRN1
YDR484W	VPS52	YJL095W	BCK1
YDR004W	RAD57	YNL297C	MON2
YML095C	RAD10	YOR368W	RAD17
YDL083C	RPS16B	YOR327C	SNC2
YLR386W	VAC14	YJL183W	MNN11
YDR146C	SWI5	YPR111W	DBF20
YNR032W	PPG1	YAL002W	VPS8
YHR039C-A	VMA10	YEL060C	PRB1
YKL139W	CTK1	YOL054W	PSH1
YGL163C	RAD54	YGR188C	BUB1
YBR194W	SOY1	YPR046W	MCM16
YMR172W	HOT1	YDR183W	PLP1
YPL024W	RMI1	YMR152W	YIM1
YKL139W	CTK1	YML006C	GIS4
YLR192C	HCR1	YNR072W	HXT17
YMR026C	PEX12	YPL037C	EGD1
YNL243W	SLA2	YKL113C	RAD27
YDR507C	GIN4	YNL229C	URE2
YDL116W	NUP84	YJL124C	LSM1
YFR040W	SAP155	YMR109W	MYO5
YOR327C	SNC2	YER040W	GLN3
YNR052C	POP2	YPL152W	RRD2
YNL079C	TPM1	YOR014W	RTS1
YCL001W	RER1	YKR020W	VPS51
YLR429W	CRN1	YDL047W	SIT4
YGL200C	EMP24	YNL041C	COG6
YER116C	SLX8	YDR335W	MSN5
YGL147C	RPL9A	YMR152W	YIM1

Predictions at threshold level 0.80 continued...			
Gene1 of predicted pair		Gene2 of predicted pair	
Systematic Name	Standard Name	Systematic Name	Standard Name
YPR120C	CLB5	YJL095W	BCK1
YJL183W	MNN11	YFL048C	EMP47
YML013W	UBX2	YKR001C	VPS1
YER122C	GLO3	YMR230W	RPS10B
YBR057C	MUM2	YOL054W	PSH1
YLR079W	SIC1	YPL178W	CBC2
YER115C	SPR6	YFL047W	RGD2
YOR270C	VPH1	YNL076W	MKS1
YNL307C	MCK1	YML112W	CTK3
YPL115C	BEM3	YGR108W	CLB1
YCR066W	RAD18	YLR309C	IMH1
YGL003C	CDH1	YBR280C	SAF1
YDR293C	SSD1	YDL056W	MBP1
YMR068W	AVO2	YBR270C	BIT2
YDR144C	MKC7	YDR318W	MCM21
YCR038C	BUD5	YPR122W	AXL1
YJL124C	LSM1	YGL115W	SNF4
YHR178W	STB5	YJL124C	LSM1
YIL103W	DPH1	YGL252C	RTG2
YDR293C	SSD1	YJL036W	SNX4
YOL018C	TLG2	YAL002W	VPS8
YJL172W	CPS1	YLR370C	ARC18
YJR090C	GRR1	YDR207C	UME6
YIL113W	SDP1	YOR208W	PTP2
YBR273C	UBX7	YPR119W	CLB2
YPR036W	VMA13	YMR133W	REC114
YPL024W	RMI1	YPL137C	GIP3
YMR199W	CLN1	YOR014W	RTS1
YGR108W	CLB1	YMR198W	CIK1

Predictions at threshold level 0.80 continued...			
Gene1 of predicted pair		Gene2 of predicted pair	
Systematic Name	Standard Name	Systematic Name	Standard Name
YDR176W	NGG1	YGR252W	GCN5
YDR293C	SSD1	YDR254W	CHL4
YLR386W	VAC14	YEL056W	HAT2
YOR043W	WHI2	YDR207C	UME6
YPL129W	TAF14	YBR009C	HHF1
YCL037C	SRO9	YCR077C	PAT1
YLR119W	SRN2	YFR010W	UBP6
YDR156W	RPA14	YLR234W	TOP3
YDR245W	MNN10	YBL101C	ECM21
YPL234C	TFP3	YPL195W	APL5
YDR293C	SSD1	YKR001C	VPS1
YNL264C	PDR17	YMR089C	YTA12
YHR178W	STB5	YBR278W	DPB3
YBL047C	EDE1	YGR135W	PRE9
YLR192C	HCR1	YEL060C	PRB1
YAL024C	LTE1	YPL161C	BEM4
YPR018W	RLF2	YPL253C	VIK1
YPR131C	NAT3	YLR288C	MEC3
YJR140C	HIR3	YLR135W	SLX4
YNL288W	CAF40	YJR135C	MCM22
YPR052C	NHP6A	YGR252W	GCN5
YGR184C	UBR1	YKL101W	HSL1
YCL037C	SRO9	YDL192W	ARF1
YCR086W	CSM1	YOL090W	MSH2
YGL115W	SNF4	YOL018C	TLG2
YMR055C	BUB2	YOL067C	RTG1
YLR360W	VPS38	YCL037C	SRO9
YDL155W	CLB3	YLR032W	RAD5
YKL006W	RPL14A	YDL155W	CLB3

Predictions at threshold level 0.80 continued...			
Gene1 of predicted pair		Gene2 of predicted pair	
Systematic Name	Standard Name	Systematic Name	Standard Name
YGR054W	YGR054W	YPR046W	MCM16
YLR371W	ROM2	YBR280C	SAF1
YLR035C	MLH2	YMR167W	MLH1
YGL078C	DBP3	YMR311C	GLC8
YNL145W	MFA2	YCR077C	PAT1
YPR030W	CSR2	YDR310C	SUM1
YJR040W	GEF1	YER162C	RAD4
YMR039C	SUB1	YDL185W	TFP1
YPR017C	DSS4	YAR014C	BUD14
YDL192W	ARF1	YMR214W	SCJ1
YLR337C	VRP1	YDR318W	MCM21
YLR095C	IOC2	YMR133W	REC114
YDR101C	ARX1	YHR052W	CIC1
YML109W	ZDS2	YPR119W	CLB2
YHR039C-A	VMA10	YOL018C	TLG2
YBL021C	HAP3	YAL002W	VPS8
YJR033C	RAV1	YPR032W	SRO7
YDR108W	GSG1	YOL108C	INO4
YNL078W	NIS1	YCL024W	KCC4
YIL036W	CST6	YKL110C	KTI12
YFR031C-A	RPL2A	YHR039C-A	VMA10
YFR040W	SAP155	YJL183W	MNN11
YCL037C	SRO9	YER139C	YER139C
YLR371W	ROM2	YCR053W	THR4
YLR090W	XDJ1	YPR046W	MCM16
YIR005W	IST3	YMR038C	CCS1
YFL033C	RIM15	YKR030W	GMH1
YML006C	GIS4	YJR090C	GRR1
YKL116C	PRR1	YDR358W	GGA1

Predictions at threshold level 0.80 continued...			
Gene1 of predicted pair		Gene2 of predicted pair	
Systematic Name	Standard Name	Systematic Name	Standard Name
YBR171W	SEC66	YJL190C	RPS22A
YDR443C	SSN2	YDL077C	VAM6
YOR066W	MSA1	YDL192W	ARF1
YHR156C	LIN1	YJL095W	BCK1
YNL297C	MON2	YJL004C	SYS1
YKR072C	SIS2	YER161C	SPT2
YBR108W	YBR108W	YER040W	GLN3
YDR004W	RAD57	YPL256C	CLN2
YLR083C	EMP70	YDR293C	SSD1
YGL147C	RPL9A	YCR053W	THR4
YIL076W	SEC28	YDR484W	VPS52
YMR172W	HOT1	YGL124C	MON1
YGL200C	EMP24	YOR312C	RPL20B
YFR024C-A	LSB3	YHR016C	YSC84
YLR306W	UBC12	YDR139C	RUB1
YLR216C	CPR6	YMR055C	BUB2
YNL297C	MON2	YKR030W	GMH1
YGL033W	HOP2	YHR200W	RPN10
YNL215W	IES2	YKL213C	DOA1
YKL101W	HSL1	YLR032W	RAD5
YMR216C	SKY1	YPL195W	APL5
YIL036W	CST6	YDR318W	MCM21
YDR176W	NGG1	YGL066W	SGF73
YNL025C	SSN8	YDR289C	RTT103
YOR023C	AHC1	YAR014C	BUD14
YDL059C	RAD59	YLR182W	SWI6
YML115C	VAN1	YOR066W	MSA1
YPL253C	VIK1	YNL236W	SIN4
YNL206C	RTT106	YLR381W	CTF3

Predictions at threshold level 0.80 continued...			
Gene1 of predicted pair		Gene2 of predicted pair	
Systematic Name	Standard Name	Systematic Name	Standard Name
YFL023W	BUD27	YHR010W	RPL27A
YMR116C	ASC1	YDL006W	PTC1
YNL291C	MID1	YDR254W	CHL4
YNL243W	SLA2	YDR388W	RVS167
YLR090W	XDJ1	YHR039C-A	VMA10
YLR360W	VPS38	YNL297C	MON2
YNL206C	RTT106	YML095C	RAD10
YDL184C	RPL41A	YMR214W	SCJ1
YPL129W	TAF14	YPR072W	NOT5
YOR308C	SNU66	YLR350W	ORM2
YFL013C	IES1	YPL057C	SUR1
YJR063W	RPA12	YKL110C	KTI12
YNL307C	MCK1	YGL252C	RTG2
YBR200W	BEM1	YIR005W	IST3
YAL024C	LTE1	YKR099W	BAS1
YMR055C	BUB2	YHR111W	UBA4
YEL065W	SIT1	YHR081W	LRP1
YOL030W	GAS5	YCR086W	CSM1
YLR119W	SRN2	YDR477W	SNF1
YER044C-A	MEI4	YHR086W	NAM8
YGL215W	CLG1	YGR105W	VMA21
YOR106W	VAM3	YBR260C	RGD1
YGR217W	CCH1	YDR245W	MNN10
YNL054W	VAC7	YPL152W	RRD2
YER096W	SHC1	YDR207C	UME6
YGL223C	COG1	YPL234C	TFP3
YPL161C	BEM4	YPR046W	MCM16
YOR132W	VPS17	YDR320C	SWA2
YPR101W	SNT309	YDR378C	LSM6

Predictions at threshold level 0.80 continued...			
Gene1 of predicted pair		Gene2 of predicted pair	
Systematic Name	Standard Name	Systematic Name	Standard Name
YJL172W	CPS1	YKL048C	ELM1
YGL060W	YBP2	YJL127C	SPT10
YDR318W	MCM21	YKL101W	HSL1
YBL047C	EDE1	YGL031C	RPL24A
YPL234C	TFP3	YEL051W	VMA8
YGR040W	KSS1	YDR103W	STE5
YML103C	NUP188	YDR183W	PLP1
YMR142C	RPL13B	YJR032W	CPR7
YGR135W	PRE9	YOL081W	IRA2
YLR410W	VIP1	YPL152W	RRD2
YPL246C	RBD2	YGL016W	KAP122
YNL068C	FKH2	YDR379W	RGA2
YNL307C	MCK1	YPL152W	RRD2
YAL024C	LTE1	YGL078C	DBP3
YPR046W	MCM16	YBR036C	CSG2
YFR010W	UBP6	YLR350W	ORM2
YPR131C	NAT3	YBR194W	SOY1
YDR146C	SWI5	YBR274W	CHK1
YLR234W	TOP3	YLR320W	MMS22
YMR224C	MRE11	YCR086W	CSM1
YDR156W	RPA14	YML112W	CTK3
YGL237C	HAP2	YJL095W	BCK1
YHR134W	WSS1	YBR114W	RAD16
YKR099W	BAS1	YER162C	RAD4
YMR172W	HOT1	YPL253C	VIK1
YLR192C	HCR1	YDR484W	VPS52
YMR026C	PEX12	YPR030W	CSR2
YML102W	CAC2	YDR207C	UME6
YDR469W	SDC1	YGR188C	BUB1

Predictions at threshold level 0.80 continued...			
Gene1 of predicted pair		Gene2 of predicted pair	
Systematic Name	Standard Name	Systematic Name	Standard Name
YLR320W	MMS22	YEL060C	PRB1
YMR142C	RPL13B	YLR452C	SST2
YER118C	SHO1	YML097C	VPS9
YOR290C	SNF2	YDR071C	PAA1
YPL161C	BEM4	YAL002W	VPS8
YOR337W	TEA1	YMR311C	GLC8
YPR046W	MCM16	YML112W	CTK3
YHR108W	GGA2	YJL140W	RPB4
YML016C	PPZ1	YDL047W	SIT4
YPR017C	DSS4	YNL041C	COG6
YFR031C-A	RPL2A	YBL058W	SHP1
YLR192C	HCR1	YBR030W	YBR030W
YOR127W	RGA1	YJR090C	GRR1
YMR172W	HOT1	YLR447C	VMA6
YNL218W	MGS1	YJR035W	RAD26
YIR003W	YIR003W	YKL213C	DOA1
YPL057C	SUR1	YJR084W	CSN12
YDR146C	SWI5	YBR171W	SEC66
YNL248C	RPA49	YBR107C	IML3
YHR161C	YAP1801	YHR108W	GGA2
YDL083C	RPS16B	YER162C	RAD4
YDL184C	RPL41A	YHR200W	RPN10
YBR188C	NTC20	YGL174W	BUD13
YOR290C	SNF2	YNL030W	HHF2
YNL107W	YAF9	YDR207C	UME6
YJR063W	RPA12	YML112W	CTK3
YOR106W	VAM3	YMR054W	STV1
YBR217W	ATG12	YER116C	SLX8
YLR079W	SIC1	YDL226C	GCS1

Predictions at threshold level 0.80 continued...			
Gene1 of predicted pair		Gene2 of predicted pair	
Systematic Name	Standard Name	Systematic Name	Standard Name
YBL058W	SHP1	YDR137W	RGP1
YIL076W	SEC28	YBR019C	GAL10
YNL069C	RPL16B	YNL291C	MID1
YHL027W	RIM101	YPL234C	TFP3
YMR311C	GLC8	YNL076W	MKS1
YGL078C	DBP3	YBL025W	RRN10
YGR081C	SLX9	YDL059C	RAD59
YMR016C	SOK2	YKL126W	YPK1
YDR293C	SSD1	YOL108C	INO4
YBL017C	PEP1	YHR161C	YAP1801
YDL059C	RAD59	YMR038C	CCS1
YGR072W	UPF3	YOR112W	CEX1
YMR039C	SUB1	YBL008W	HIR1
YHR081W	LRP1	YGL003C	CDH1
YAL024C	LTE1	YHR013C	ARD1
YDR225W	HTA1	YMR284W	YKU70
YLR386W	VAC14	YHR012W	VPS29
YCR020C-A	MAK31	YML038C	YMD8
YNL238W	KEX2	YPR032W	SRO7
YNL329C	PEX6	YKR020W	VPS51
YBR171W	SEC66	YAR014C	BUD14
YNL206C	RTT106	YJL095W	BCK1
YIL159W	BNR1	YGL078C	DBP3
YML095C	RAD10	YGR270W	YTA7
YFL013C	IES1	YOR038C	HIR2
YFL023W	BUD27	YGL003C	CDH1
YMR216C	SKY1	YMR054W	STV1
YLR079W	SIC1	YLR386W	VAC14
YLR090W	XDJ1	YLR192C	HCR1

Predictions at threshold level 0.80 continued...			
Gene1 of predicted pair		Gene2 of predicted pair	
Systematic Name	Standard Name	Systematic Name	Standard Name
YNL215W	IES2	YPL180W	TCO89
YOR290C	SNF2	YFL023W	BUD27
YOR080W	DIA2	YMR202W	ERG2
YOL018C	TLG2	YKL119C	VPH2
YDR363W-A	SEM1	YAR014C	BUD14
YNL044W	YIP3	YGR105W	VMA21
YJR056C	YJR056C	YCR053W	THR4
YHR013C	ARD1	YMR089C	YTA12
YPL195W	APL5	YOL018C	TLG2
YIL076W	SEC28	YGL174W	BUD13
YML001W	YPT7	YER162C	RAD4
YLL039C	UBI4	YNR006W	VPS27
YLR447C	VMA6	YIL008W	URM1
YOR270C	VPH1	YKR035W-A	DID2
YGL033W	HOP2	YBR036C	CSG2
YPL024W	RMI1	YNL307C	MCK1
YDR146C	SWI5	YOR368W	RAD17
YPL042C	SSN3	YOR124C	UBP2
YNL054W	VAC7	YNL225C	CNM67
YAL020C	ATS1	YNL116W	DMA2
YLR216C	CPR6	YDR254W	CHL4
YGL033W	HOP2	YDL223C	HBT1
YFR024C-A	LSB3	YLR144C	ACF2
YPL024W	RMI1	YJR135C	MCM22
YOR109W	INP53	YPL234C	TFP3
YML103C	NUP188	YDR318W	MCM21
YMR026C	PEX12	YML001W	YPT7
YNL297C	MON2	YJR060W	CBF1
YMR026C	PEX12	YNR072W	HXT17

Predictions at threshold level 0.80 continued...			
Gene1 of predicted pair		Gene2 of predicted pair	
Systematic Name	Standard Name	Systematic Name	Standard Name
YMR055C	BUB2	YGL151W	NUT1
YPR017C	DSS4	YNL293W	MSB3
YDR129C	SAC6	YJL006C	CTK2
YML115C	VAN1	YBR280C	SAF1
YPL096C-A	ERI1	YBL007C	SLA1
YMR153W	NUP53	YPL037C	EGD1
YMR224C	MRE11	YGL066W	SGF73
YMR153W	NUP53	YGL025C	PGD1
YPL024W	RMI1	YDR289C	RTT103
YDL155W	CLB3	YDR318W	MCM21
YPL253C	VIK1	YBR171W	SEC66
YML071C	COG8	YDR310C	SUM1
YGL087C	MMS2	YBL051C	PIN4
YLR064W	YLR064W	YKR030W	GMH1
YDR108W	GSG1	YBR036C	CSG2
YDL116W	NUP84	YDL155W	CLB3
YDR004W	RAD57	YCR077C	PAT1
YDL020C	RPN4	YOR368W	RAD17
YAL024C	LTE1	YLR386W	VAC14
YMR216C	SKY1	YLR309C	IMH1
YMR109W	MYO5	YOR358W	HAP5
YIL036W	CST6	YBR107C	IML3
YLR337C	VRP1	YDR293C	SSD1
YDR004W	RAD57	YBR274W	CHK1
YDR293C	SSD1	YGL003C	CDH1
YDR171W	HSP42	YJR035W	RAD26
YBL078C	ATG8	YGL212W	VAM7
YOR358W	HAP5	YPL018W	CTF19
YKR072C	SIS2	YDR076W	RAD55

Predictions at threshold level 0.80 continued...			
Gene1 of predicted pair		Gene2 of predicted pair	
Systematic Name	Standard Name	Systematic Name	Standard Name
YPL042C	SSN3	YDL005C	MED2
YCR031C	RPS14A	YOL081W	IRA2
YML103C	NUP188	YMR164C	MSS11
YHR012W	VPS29	YHR114W	BZZ1
YEL065W	SIT1	YHR156C	LIN1
YGL133W	ITC1	YJL095W	BCK1
YLR170C	APS1	YOR109W	INP53
YLR441C	RPS1A	YDR207C	UME6
YPL129W	TAF14	YJR140C	HIR3
YFR030W	MET10	YLR254C	NDL1
YBR057C	MUM2	YDL223C	HBT1
YNL166C	BNI5	YJL190C	RPS22A
YJR084W	CSN12	YNL076W	MKS1
YNL199C	GCR2	YPL256C	CLN2
YMR284W	YKU70	YCR066W	RAD18
YPL240C	HSP82	YEL022W	GEA2
YJL154C	VPS35	YDR245W	MNN10
YNL069C	RPL16B	YDR207C	UME6
YMR198W	CIK1	YDR378C	LSM6
YBR073W	RDH54	YDL013W	SLX5
YDR129C	SAC6	YMR198W	CIK1
YPR051W	MAK3	YPL018W	CTF19
YLR360W	VPS38	YBR108W	YBR108W
YJL151C	SNA3	YLR064W	YLR064W
YOR270C	VPH1	YPL234C	TFP3
YER144C	UBP5	YER040W	GLN3
YGR054W	YGR054W	YPR163C	TIF3
YNL307C	MCK1	YLR452C	SST2
YLR102C	APC9	YPL256C	CLN2

Predictions at threshold level 0.80 continued...			
Gene1 of predicted pair		Gene2 of predicted pair	
Systematic Name	Standard Name	Systematic Name	Standard Name
YPL037C	EGD1	YDL083C	RPS16B
YIL076W	SEC28	YOR066W	MSA1
YPR052C	NHP6A	YLR357W	RSC2
YBR215W	HPC2	YAL002W	VPS8
YDR293C	SSD1	YHR178W	STB5
YOR043W	WHI2	YCR086W	CSM1
YGL223C	COG1	YDR153C	ENT5
YLR119W	SRN2	YFL023W	BUD27
YDL155W	CLB3	YHL002W	HSE1
YBL102W	SFT2	YPL120W	VPS30
YFR032C-A	RPL29	YJL117W	PHO86
YDL077C	VAM6	YNL076W	MKS1
YNL069C	RPL16B	YML001W	YPT7
YLR386W	VAC14	YMR055C	BUB2
YKL025C	PAN3	YBR108W	YBR108W
YPR017C	DSS4	YDL006W	PTC1
YOR275C	RIM20	YHR158C	KEL1
YFL023W	BUD27	YEL060C	PRB1
YLR313C	SPH1	YJR035W	RAD26
YHR161C	YAP1801	YJR125C	ENT3
YDR174W	HMO1	YIL103W	DPH1
YGR261C	APL6	YNL297C	MON2
YGL212W	VAM7	YJL095W	BCK1
YML016C	PPZ1	YJL006C	CTK2
YPL057C	SUR1	YDL192W	ARF1
YJL172W	CPS1	YLR452C	SST2
YBR009C	HHF1	YDR440W	DOT1
YLR234W	TOP3	YBR215W	HPC2
YBR200W	BEM1	YKL110C	KTI12

Predictions at threshold level 0.80 continued...			
Gene1 of predicted pair		Gene2 of predicted pair	
Systematic Name	Standard Name	Systematic Name	Standard Name
YOL111C	MDY2	YDL192W	ARF1
YPL253C	VIK1	YNL079C	TPM1
YML071C	COG8	YDL077C	VAM6
YDR443C	SSN2	YOR337W	TEA1
YKR072C	SIS2	YLR352W	YLR352W
YBR171W	SEC66	YOL111C	MDY2
YMR026C	PEX12	YBR030W	YBR030W
YHR149C	SKG6	YPL240C	HSP82
YFR031C-A	RPL2A	YGR020C	VMA7
YDR293C	SSD1	YDL155W	CLB3
YKL126W	YPK1	YJR056C	YJR056C
YDR363W-A	SEM1	YML097C	VPS9
YPR084W	YPR084W	YPL161C	BEM4
YKR035W-A	DID2	YPL161C	BEM4
YPL161C	BEM4	YKR020W	VPS51
YFL023W	BUD27	YGL115W	SNF4
YKL006W	RPL14A	YDR245W	MNN10
YJL095W	BCK1	YNL116W	DMA2
YLR371W	ROM2	YBL025W	RRN10
YML115C	VAN1	YJL117W	PHO86
YPR120C	CLB5	YCR086W	CSM1
YNL248C	RPA49	YLR182W	SWI6
YLR360W	VPS38	YMR109W	MYO5
YJR056C	YJR056C	YNL307C	MCK1
YNL041C	COG6	YPL137C	GIP3
YDL155W	CLB3	YJR090C	GRR1
YNL044W	YIP3	YPR028W	YOP1
YNL248C	RPA49	YGL163C	RAD54
YLR371W	ROM2	YDL077C	VAM6

Predictions at threshold level 0.80 continued...			
Gene1 of predicted pair		Gene2 of predicted pair	
Systematic Name	Standard Name	Systematic Name	Standard Name
YNR052C	POP2	YDR335W	MSN5
YER120W	SCS2	YPL195W	APL5
YPL024W	RMI1	YOR195W	SLK19
YJL183W	MNN11	YOL081W	IRA2
YBR200W	BEM1	YNL293W	MSB3
YGL237C	HAP2	YDR155C	CPR1
YLL039C	UBI4	YKL041W	VPS24
YJR084W	CSN12	YOL081W	IRA2
YMR116C	ASC1	YBL101C	ECM21
YPL037C	EGD1	YDR310C	SUM1
YPR036W	VMA13	YMR054W	STV1
YGR108W	CLB1	YEL053C	MAK10
YDR156W	RPA14	YNL116W	DMA2
YBR215W	HPC2	YJR135C	MCM22
YLR095C	IOC2	YKL116C	PRR1
YKR072C	SIS2	YER116C	SLX8
YGL115W	SNF4	YML001W	YPT7
YPR030W	CSR2	YOR035C	SHE4
YBR107C	IML3	YPR120C	CLB5
YNL186W	UBP10	YPR119W	CLB2
YDR144C	MKC7	YEL056W	HAT2
YBR171W	SEC66	YOR043W	WHI2
YGL066W	SGF73	YMR055C	BUB2
YDL223C	HBT1	YML001W	YPT7
YGL124C	MON1	YOL081W	IRA2
YDL020C	RPN4	YMR055C	BUB2
YDR137W	RGP1	YGR188C	BUB1
YNL215W	IES2	YDR146C	SWI5
YJL036W	SNX4	YPR051W	MAK3

Predictions at threshold level 0.80 continued...			
Gene1 of predicted pair		Gene2 of predicted pair	
Systematic Name	Standard Name	Systematic Name	Standard Name
YOR080W	DIA2	YDR254W	CHL4
YDR378C	LSM6	YDR289C	RTT103
YLL039C	UBI4	YDL155W	CLB3
YLR360W	VPS38	YCR088W	ABP1
YNL094W	APP1	YCR088W	ABP1
YNL215W	IES2	YKL041W	VPS24
YER116C	SLX8	YOR368W	RAD17
YDL013W	SLX5	YJL187C	SWE1
YKR072C	SIS2	YLR135W	SLX4
YGR249W	MGA1	YOR327C	SNC2
YJL095W	BCK1	YMR089C	YTA12
YNL243W	SLA2	YML062C	MFT1
YMR109W	MYO5	YDR183W	PLP1
YDR265W	PEX10	YOL044W	PEX15
YMR202W	ERG2	YOR327C	SNC2
YLR441C	RPS1A	YOL081W	IRA2
YDR156W	RPA14	YNL225C	CNM67
YNL025C	SSN8	YDR477W	SNF1
YER087C-B	SBH1	YML019W	OST6
YLR288C	MEC3	YPL256C	CLN2
YOR033C	EXO1	YGL100W	SEH1
YBL058W	SHP1	YMR152W	YIM1
YCR066W	RAD18	YKR020W	VPS51
YOR290C	SNF2	YHR039C-A	VMA10
YCR086W	CSM1	YDR155C	CPR1
YLR360W	VPS38	YDR323C	PEP7
YOR106W	VAM3	YGR020C	VMA7
YGL003C	CDH1	YGL124C	MON1
YJR140C	HIR3	YOR043W	WHI2

Predictions at threshold level 0.80 continued...			
Gene1 of predicted pair		Gene2 of predicted pair	
Systematic Name	Standard Name	Systematic Name	Standard Name
YLR135W	SLX4	YPL079W	RPL21B
YOR112W	CEX1	YJL036W	SNX4
YNL238W	KEX2	YNL051W	COG5
YHR178W	STB5	YOR368W	RAD17
YKL139W	CTK1	YKR030W	GMH1
YNL106C	INP52	YNL307C	MCK1
YLR386W	VAC14	YNL248C	RPA49
YDR383C	NKP1	YML103C	NUP188
YBR215W	HPC2	YMR038C	CCS1
YBL103C	RTG3	YHR081W	LRP1
YEL053C	MAK10	YER120W	SCS2
YLR079W	SIC1	YKL048C	ELM1
YCL037C	SRO9	YDR440W	DOT1
YBR289W	SNF5	YOR038C	HIR2
YPR051W	MAK3	YMR109W	MYO5
YLR399C	BDF1	YDR254W	CHL4
YFR031C-A	RPL2A	YJL053W	PEP8
YER144C	UBP5	YHR060W	VMA22
YBL102W	SFT2	YGR020C	VMA7
YJL095W	BCK1	YMR214W	SCJ1
YJR040W	GEF1	YEL051W	VMA8
YLR119W	SRN2	YBR200W	BEM1
YPR030W	CSR2	YBL025W	RRN10
YGR108W	CLB1	YAL002W	VPS8
YMR055C	BUB2	YDR363W-A	SEM1
YLL007C	YLL007C	YLR447C	VMA6
YOR358W	HAP5	YGR054W	YGR054W
YBR108W	YBR108W	YNL058C	YNL058C
YBR171W	SEC66	YNL166C	BNI5

Predictions at threshold level 0.80 continued...			
**Gene1 of predicted pair**		**Gene2 of predicted pair**	
**Systematic Name**	**Standard Name**	**Systematic Name**	**Standard Name**
YBR057C	MUM2	YML028W	TSA1
YCR066W	RAD18	YGL054C	ERV14
YOR083W	WHI5	YPL018W	CTF19
YOR195W	SLK19	YNR052C	POP2
YLR090W	XDJ1	YNL325C	FIG4
YLR135W	SLX4	YGL031C	RPL24A
YMR224C	MRE11	YKL032C	IXR1
YLR234W	TOP3	YHL002W	HSE1
YMR116C	ASC1	YGR135W	PRE9
YPL180W	TCO89	YDR363W-A	SEM1
YML071C	COG8	YMR230W	RPS10B
YOR127W	RGA1	YNL238W	KEX2
YDR484W	VPS52	YOL121C	RPS19A
YDL020C	RPN4	YGL003C	CDH1
YHL020C	OPI1	YDR153C	ENT5
YDR225W	HTA1	YNL291C	MID1
YJR084W	CSN12	YBR280C	SAF1
YLL007C	YLL007C	YJL183W	MNN11
YKL007W	CAP1	YGR152C	RSR1
YNL248C	RPA49	YNL225C	CNM67
YJL154C	VPS35	YEL053C	MAK10
YKL010C	UFD4	YBL101C	ECM21
YDR443C	SSN2	YNL079C	TPM1
YLR094C	GIS3	YJL183W	MNN11
YBR041W	FAT1	YKR046C	PET10
YLR381W	CTF3	YBR215W	HPC2
YBR107C	IML3	YOL067C	RTG1
YMR199W	CLN1	YOL081W	IRA2
YKL007W	CAP1	YKL204W	EAP1

Predictions at threshold level 0.80 continued...			
**Gene1 of predicted pair**		**Gene2 of predicted pair**	
**Systematic Name**	**Standard Name**	**Systematic Name**	**Standard Name**
YNR058W	BIO3	YJR025C	BNA1
YMR199W	CLN1	YMR038C	CCS1
YBR171W	SEC66	YBR280C	SAF1
YDL002C	NHP10	YOR141C	ARP8
YPL105C	YPL105C	YML071C	COG8
YER110C	KAP123	YNL107W	YAF9
YEL065W	SIT1	YDL077C	VAM6
YLR410W	VIP1	YLR315W	NKP2
YBR107C	IML3	YGR135W	PRE9
YBL008W	HIR1	YKL101W	HSL1
YGL033W	HOP2	YJL172W	CPS1
YCL037C	SRO9	YDR289C	RTT103
YDL020C	RPN4	YML102W	CAC2
YBR273C	UBX7	YJL048C	UBX6
YLL039C	UBI4	YBL047C	EDE1
YLR119W	SRN2	YBR030W	YBR030W
YER116C	SLX8	YDL192W	ARF1
YJL176C	SWI3	YOR290C	SNF2
YFL023W	BUD27	YDR076W	RAD55
YER087C-B	SBH1	YAR003W	SWD1
YNL215W	IES2	YDR245W	MNN10
YIL076W	SEC28	YPL057C	SUR1
YIL076W	SEC28	YOL018C	TLG2
YMR133W	REC114	YER040W	GLN3
YNL058C	YNL058C	YMR311C	GLC8
YMR153W	NUP53	YDR310C	SUM1
YDL154W	MSH5	YMR167W	MLH1
YHR056C	RSC30	YLR357W	RSC2
YKL110C	KTI12	YFR030W	MET10

Predictions at threshold level 0.80 continued...			
Gene1 of predicted pair		Gene2 of predicted pair	
Systematic Name	Standard Name	Systematic Name	Standard Name
YFR031C-A	RPL2A	YLR441C	RPS1A
YKR099W	BAS1	YHR081W	LRP1
YDR289C	RTT103	YNL116W	DMA2
YDL020C	RPN4	YBL021C	HAP3
YBR171W	SEC66	YKR035W-A	DID2
YLR381W	CTF3	YJL124C	LSM1
YLR337C	VRP1	YEL056W	HAT2
YMR224C	MRE11	YML001W	YPT7
YMR142C	RPL13B	YGL003C	CDH1
YOL121C	RPS19A	YDL192W	ARF1
YPL253C	VIK1	YGL237C	HAP2
YDL020C	RPN4	YPL057C	SUR1
YGL100W	SEH1	YKL068W	NUP100
YGL124C	MON1	YDR310C	SUM1
YKL032C	IXR1	YBL047C	EDE1
YOR001W	RRP6	YGR270W	YTA7
YAL024C	LTE1	YER116C	SLX8
YPR084W	YPR084W	YNR032W	PPG1
YNL307C	MCK1	YNR072W	HXT17
YHR178W	STB5	YBR108W	YBR108W
YNL025C	SSN8	YDR363W-A	SEM1
YDR142C	PEX7	YLL061W	MMP1
YMR216C	SKY1	YHR012W	VPS29
YBR057C	MUM2	YGR184C	UBR1
YDR254W	CHL4	YGR135W	PRE9
YDR080W	VPS41	YNL051W	COG5
YJL124C	LSM1	YDL059C	RAD59
YNL051W	COG5	YEL042W	GDA1
YMR095C	SNO1	YNR072W	HXT17

Predictions at threshold level 0.80 continued...			
Gene1 of predicted pair		Gene2 of predicted pair	
Systematic Name	Standard Name	Systematic Name	Standard Name
YBR278W	DPB3	YCR077C	PAT1
YPL161C	BEM4	YDL083C	RPS16B
YNR072W	HXT17	YMR038C	CCS1
YNL288W	CAF40	YPL161C	BEM4
YGR217W	CCH1	YKL048C	ELM1
YMR198W	CIK1	YLR398C	SKI2
YMR055C	BUB2	YPL161C	BEM4
YPL089C	RLM1	YJL183W	MNN11
YFR010W	UBP6	YOL081W	IRA2
YBR278W	DPB3	YBR215W	HPC2
YEL036C	ANP1	YDR207C	UME6
YDL223C	HBT1	YBR019C	GAL10
YLR058C	SHM2	YPL106C	SSE1
YJR056C	YJR056C	YBR171W	SEC66
YBL051C	PIN4	YJL183W	MNN11
YJL176C	SWI3	YPL161C	BEM4
YHR073W	OSH3	YMR273C	ZDS1
YBL058W	SHP1	YBR171W	SEC66
YHR060W	VMA22	YBR036C	CSG2
YDR146C	SWI5	YCR086W	CSM1
YGL178W	MPT5	YGL003C	CDH1
YPR084W	YPR084W	YPR017C	DSS4
YNL215W	IES2	YLR079W	SIC1
YGR054W	YGR054W	YNL107W	YAF9
YLR234W	TOP3	YHR200W	RPN10
YGL060W	YBP2	YNL236W	SIN4
YPL037C	EGD1	YDL006W	PTC1
YDR443C	SSN2	YBR108W	YBR108W
YER161C	SPT2	YBR009C	HHF1

Predictions at threshold level 0.80 continued...			
Gene1 of predicted pair		Gene2 of predicted pair	
Systematic Name	Standard Name	Systematic Name	Standard Name
YGL019W	CKB1	YOL081W	IRA2
YFR030W	MET10	YPL018W	CTF19
YIL156W	UBP7	YPL178W	CBC2
YDR389W	SAC7	YBR200W	BEM1
YDL080C	THI3	YMR224C	MRE11
YKL126W	YPK1	YDR207C	UME6
YGL190C	CDC55	YNL271C	BNI1
YLR386W	VAC14	YDL223C	HBT1
YGL190C	CDC55	YGL208W	SIP2
YOL111C	MDY2	YOR035C	SHE4
YDR129C	SAC6	YHL027W	RIM101
YDR085C	AFR1	YBL085W	BOI1
YCR081W	SRB8	YGL025C	PGD1
YMR125W	STO1	YBL079W	NUP170
YPR017C	DSS4	YOL108C	INO4
YGL147C	RPL9A	YLR180W	SAM1
YJL154C	VPS35	YOR069W	VPS5
YBR127C	VMA2	YHR060W	VMA22
YJL060W	BNA3	YPL018W	CTF19
YOR337W	TEA1	YJL183W	MNN11
YNL041C	COG6	YJR135C	MCM22
YPL024W	RMI1	YBR173C	UMP1
YMR179W	SPT21	YGL115W	SNF4
YLR083C	EMP70	YJR040W	GEF1
YLL007C	YLL007C	YDL020C	RPN4
YJL124C	LSM1	YDR363W-A	SEM1
YIL076W	SEC28	YDL002C	NHP10
YMR095C	SNO1	YGL115W	SNF4
YKR099W	BAS1	YDL223C	HBT1

Predictions at threshold level 0.80 continued...			
Gene1 of predicted pair		Gene2 of predicted pair	
Systematic Name	Standard Name	Systematic Name	Standard Name
YPL115C	BEM3	YBL051C	PIN4
YBR111W-A	SUS1	YDR159W	SAC3
YMR116C	ASC1	YBR041W	FAT1
YLR094C	GIS3	YDL077C	VAM6
YDR293C	SSD1	YML071C	COG8
YGR249W	MGA1	YPL203W	TPK2
YJL154C	VPS35	YMR216C	SKY1
YGR072W	UPF3	YOR106W	VAM3
YDL223C	HBT1	YDL192W	ARF1
YLR337C	VRP1	YLR192C	HCR1
YJL154C	VPS35	YLR309C	IMH1
YKL025C	PAN3	YLR337C	VRP1
YOR127W	RGA1	YKR030W	GMH1
YCL001W	RER1	YPL195W	APL5
YBL051C	PIN4	YHR012W	VPS29
YLR095C	IOC2	YOR035C	SHE4
YFR010W	UBP6	YCR077C	PAT1
YKL139W	CTK1	YFR030W	MET10
YMR172W	HOT1	YPL161C	BEM4
YMR224C	MRE11	YBR030W	YBR030W
YML115C	VAN1	YER116C	SLX8
YLR119W	SRN2	YJL172W	CPS1
YER115C	SPR6	YBL079W	NUP170
YDR261C	EXG2	YGL003C	CDH1
YJR060W	CBF1	YPR032W	SRO7
YOR337W	TEA1	YNL032W	SIW14
YMR273C	ZDS1	YPL246C	RBD2
YLR371W	ROM2	YEL060C	PRB1
YFR024C-A	LSB3	YOR284W	HUA2

Predictions at threshold level 0.80 continued...			
Gene1 of predicted pair		Gene2 of predicted pair	
Systematic Name	Standard Name	Systematic Name	Standard Name
YHR086W	NAM8	YJR021C	REC107
YIL076W	SEC28	YPR032W	SRO7
YPL195W	APL5	YDL077C	VAM6
YFL066C	YFL066C	YNL147W	LSM7
YDR202C	RAV2	YOL018C	TLG2
YGL003C	CDH1	YCR053W	THR4
YOR156C	NFI1	YNL107W	YAF9
YDR293C	SSD1	YJR135C	MCM22
YHR013C	ARD1	YLR315W	NKP2
YMR125W	STO1	YPL256C	CLN2
YGR275W	RTT102	YOR156C	NFI1
YOR080W	DIA2	YGL200C	EMP24
YJL172W	CPS1	YOL081W	IRA2
YOR270C	VPH1	YDR310C	SUM1
YDR440W	DOT1	YBR010W	HHT1
YDR293C	SSD1	YBL051C	PIN4
YNL054W	VAC7	YKL110C	KTI12
YHR156C	LIN1	YGL115W	SNF4
YPL115C	BEM3	YMR127C	SAS2
YNL069C	RPL16B	YDL006W	PTC1
YLR119W	SRN2	YGL124C	MON1
YLR398C	SKI2	YOL081W	IRA2
YLR381W	CTF3	YGL250W	RMR1
YER044C	ERG28	YLR371W	ROM2
YPR018W	RLF2	YLR234W	TOP3
YBR258C	SHG1	YOL145C	CTR9
YHR039C-A	VMA10	YBR280C	SAF1
YAL024C	LTE1	YPR046W	MCM16
YMR199W	CLN1	YML095C	RAD10

Predictions at threshold level 0.80 continued...			
Gene1 of predicted pair		Gene2 of predicted pair	
Systematic Name	Standard Name	Systematic Name	Standard Name
YHR119W	SET1	YBR258C	SHG1
YML016C	PPZ1	YLR350W	ORM2
YGL115W	SNF4	YGL054C	ERV14
YHR200W	RPN10	YJR135C	MCM22
YGL200C	EMP24	YAL055W	PEX22
YKR072C	SIS2	YGL175C	SAE2
YLR093C	NYV1	YER139C	YER139C
YHR156C	LIN1	YGL212W	VAM7
YGL246C	RAI1	YDR025W	RPS11A
YER116C	SLX8	YDR310C	SUM1
YDR260C	SWM1	YOL054W	PSH1
YOR195W	SLK19	YLR315W	NKP2
YNL199C	GCR2	YIR005W	IST3
YPL161C	BEM4	YBR082C	UBC4
YLL007C	YLL007C	YJR090C	GRR1
YIL053W	RHR2	YBR036C	CSG2
YNL054W	VAC7	YML016C	PPZ1
YHR098C	SFB3	YBR231C	SWC5
YBL046W	PSY4	YPR040W	TIP41
YDR174W	HMO1	YLR182W	SWI6
YDL116W	NUP84	YJR059W	PTK2
YGL003C	CDH1	YLR398C	SKI2
YER162C	RAD4	YPL120W	VPS30
YJL053W	PEP8	YML001W	YPT7
YBR030W	YBR030W	YPL137C	GIP3
YDR309C	GIC2	YKR048C	NAP1
YBR019C	GAL10	YNL076W	MKS1
YHR134W	WSS1	YOR132W	VPS17
YHR205W	SCH9	YGR063C	SPT4

Predictions at threshold level 0.80 continued...			
Gene1 of predicted pair		Gene2 of predicted pair	
Systematic Name	Standard Name	Systematic Name	Standard Name
YLR313C	SPH1	YGL175C	SAE2
YBR200W	BEM1	YBR019C	GAL10
YDL013W	SLX5	YGR188C	BUB1
YDR439W	LRS4	YPL253C	VIK1
YOR106W	VAM3	YGL237C	HAP2
YDR507C	GIN4	YDL047W	SIT4
YOR033C	EXO1	YJR090C	GRR1
YCR020C-A	MAK31	YIL076W	SEC28
YML115C	VAN1	YER001W	MNN1
YDR477W	SNF1	YPL057C	SUR1
YDR202C	RAV2	YDL077C	VAM6
YGL078C	DBP3	YGR249W	MGA1
YLR150W	STM1	YMR038C	CCS1
YML013W	UBX2	YNL297C	MON2
YJL176C	SWI3	YLR425W	TUS1
YDR156W	RPA14	YDR183W	PLP1
YPR131C	NAT3	YGR081C	SLX9
YOR127W	RGA1	YKL032C	IXR1
YLR234W	TOP3	YFL023W	BUD27
YBR173C	UMP1	YLR192C	HCR1
YBR108W	YBR108W	YDR183W	PLP1
YIL159W	BNR1	YPL024W	RMI1
YIL076W	SEC28	YJR060W	CBF1
YGR020C	VMA7	YDL077C	VAM6
YLR360W	VPS38	YDR183W	PLP1
YIL159W	BNR1	YML071C	COG8
YNL288W	CAF40	YML016C	PPZ1
YGL180W	ATG1	YPR185W	ATG13
YML016C	PPZ1	YGR188C	BUB1

Predictions at threshold level 0.80 continued...			
Gene1 of predicted pair		Gene2 of predicted pair	
Systematic Name	Standard Name	Systematic Name	Standard Name
YIL076W	SEC28	YDR137W	RGP1
YML038C	YMD8	YNL127W	FAR11
YJL154C	VPS35	YDR456W	NHX1
YHR013C	ARD1	YPR120C	CLB5
YER120W	SCS2	YNR032W	PPG1
YGL147C	RPL9A	YDR310C	SUM1
YBR173C	UMP1	YLR234W	TOP3
YOR132W	VPS17	YKR001C	VPS1
YHR060W	VMA22	YPL161C	BEM4
YOR001W	RRP6	YGL115W	SNF4
YMR054W	STV1	YER162C	RAD4
YBR258C	SHG1	YOR156C	NFI1
YEL043W	YEL043W	YNR032W	PPG1
YDR202C	RAV2	YDR080W	VPS41
YJR040W	GEF1	YHR012W	VPS29
YMR133W	REC114	YJR032W	CPR7
YBR290W	BSD2	YDL155W	CLB3
YHR200W	RPN10	YNL229C	URE2
YLR441C	RPS1A	YMR133W	REC114
YKL048C	ELM1	YDL006W	PTC1
YBR138C	YBR138C	YMR022W	UBC7
YOR109W	INP53	YPL037C	EGD1
YML001W	YPT7	YPR030W	CSR2
YKL135C	APL2	YOR069W	VPS5
YER115C	SPR6	YML109W	ZDS2
YMR133W	REC114	YMR214W	SCJ1
YMR055C	BUB2	YHR114W	BZZ1
YBR036C	CSG2	YDR310C	SUM1
YML103C	NUP188	YMR199W	CLN1

Predictions at threshold level 0.80 continued...			
Gene1 of predicted pair		Gene2 of predicted pair	
Systematic Name	Standard Name	Systematic Name	Standard Name
YML103C	NUP188	YJR090C	GRR1
YDL184C	RPL41A	YFL023W	BUD27
YLR320W	MMS22	YOR035C	SHE4
YML058W	SML1	YBL088C	TEL1
YBL103C	RTG3	YHR039C-A	VMA10
YPR049C	ATG11	YGL180W	ATG1
YOR290C	SNF2	YPL057C	SUR1
YJR135C	MCM22	YBR036C	CSG2
YOR069W	VPS5	YJR060W	CBF1
YGL223C	COG1	YOR327C	SNC2
YDL184C	RPL41A	YHR060W	VMA22
YER114C	BOI2	YGL124C	MON1
YDL116W	NUP84	YLR386W	VAC14
YLR315W	NKP2	YAL002W	VPS8
YHR200W	RPN10	YHR081W	LRP1
YNL307C	MCK1	YMR055C	BUB2
YJL060W	BNA3	YJL058C	BIT61
YGL115W	SNF4	YDL226C	GCS1
YPR046W	MCM16	YDL047W	SIT4
YER116C	SLX8	YNL297C	MON2
YFL013C	IES1	YPL129W	TAF14
YDL227C	HO	YLL013C	PUF3
YNL297C	MON2	YER120W	SCS2
YGR135W	PRE9	YJL183W	MNN11
YML058W	SML1	YKL114C	APN1
YNL325C	FIG4	YOR043W	WHI2
YLR386W	VAC14	YDR378C	LSM6
YEL051W	VMA8	YER122C	GLO3
YPL161C	BEM4	YER120W	SCS2

Predictions at threshold level 0.80 continued...			
Gene1 of predicted pair		Gene2 of predicted pair	
Systematic Name	Standard Name	Systematic Name	Standard Name
YFR010W	UBP6	YDR378C	LSM6
YEL065W	SIT1	YMR214W	SCJ1
YLR315W	NKP2	YMR038C	CCS1
YJR010C-A	SPC1	YML019W	OST6
YDR004W	RAD57	YCR086W	CSM1
YBR030W	YBR030W	YNL058C	YNL058C
YFL031W	HAC1	YOR036W	PEP12
YGR019W	UGA1	YPR051W	MAK3
YGR072W	UPF3	YAR014C	BUD14
YPL105C	YPL105C	YGL054C	ERV14
YMR198W	CIK1	YGR188C	BUB1
YOR112W	CEX1	YOR106W	VAM3
YBL102W	SFT2	YER122C	GLO3
YBR171W	SEC66	YNR072W	HXT17
YJR032W	CPR7	YEL060C	PRB1
YBR215W	HPC2	YER122C	GLO3
YEL065W	SIT1	YNL307C	MCK1
YBR215W	HPC2	YGL148W	ARO2
YNL215W	IES2	YHR167W	THP2
YGL031C	RPL24A	YDR207C	UME6
YPR101W	SNT309	YPL213W	LEA1
YOR195W	SLK19	YBR200W	BEM1
YDR004W	RAD57	YML102W	CAC2
YJR140C	HIR3	YER122C	GLO3
YOR270C	VPH1	YCR053W	THR4
YML103C	NUP188	YDR439W	LRS4
YJL127C	SPT10	YPL161C	BEM4
YHR134W	WSS1	YPL057C	SUR1
YNL166C	BNI5	YMR055C	BUB2

Predictions at threshold level 0.80 continued...			
Gene1 of predicted pair		Gene2 of predicted pair	
Systematic Name	Standard Name	Systematic Name	Standard Name
YJR140C	HIR3	YLR350W	ORM2
YMR198W	CIK1	YMR202W	ERG2
YFR010W	UBP6	YPR120C	CLB5
YDL020C	RPN4	YPL161C	BEM4
YMR026C	PEX12	YNL238W	KEX2
YDR137W	RGP1	YMR311C	GLC8
YGL161C	YIP5	YJL124C	LSM1
YDR363W-A	SEM1	YOR308C	SNU66
YDR293C	SSD1	YGR152C	RSR1
YBR158W	AMN1	YLR309C	IMH1
YDR144C	MKC7	YOL067C	RTG1
YGL212W	VAM7	YMR311C	GLC8
YKL110C	KTI12	YGL252C	RTG2
YHR119W	SET1	YGR166W	KRE11
YPR120C	CLB5	YOL081W	IRA2
YPL115C	BEM3	YDR379W	RGA2
YGR135W	PRE9	YLR425W	TUS1
YLR386W	VAC14	YLR233C	EST1
YBL102W	SFT2	YER120W	SCS2
YMR224C	MRE11	YOR066W	MSA1
YLR018C	POM34	YKR095W	MLP1
YJR084W	CSN12	YDR207C	UME6
YDR004W	RAD57	YJR084W	CSN12
YNL069C	RPL16B	YNL107W	YAF9
YNL199C	GCR2	YNL079C	TPM1
YDR071C	PAA1	YBR010W	HHT1
YGR108W	CLB1	YMR055C	BUB2
YML103C	NUP188	YKL139W	CTK1
YOR033C	EXO1	YMR224C	MRE11

Predictions at threshold level 0.80 continued...			
Gene1 of predicted pair		Gene2 of predicted pair	
Systematic Name	Standard Name	Systematic Name	Standard Name
YOR127W	RGA1	YLR233C	EST1
YHR111W	UBA4	YEL056W	HAT2
YBR107C	IML3	YGR054W	YGR054W
YKR035W-A	DID2	YDR261C	EXG2
YIL005W	EPS1	YHR200W	RPN10
YJL004C	SYS1	YOR327C	SNC2
YLR447C	VMA6	YPR046W	MCM16
YNL325C	FIG4	YML001W	YPT7
YMR224C	MRE11	YOL054W	PSH1
YPL024W	RMI1	YBR107C	IML3
YGL253W	HXK2	YLR233C	EST1
YER139C	YER139C	YLR398C	SKI2
YER059W	PCL6	YPL219W	PCL8
YJL176C	SWI3	YAL002W	VPS8
YGR184C	UBR1	YDR335W	MSN5
YLR148W	PEP3	YLR360W	VPS38
YML095C	RAD10	YIL008W	URM1
YHR205W	SCH9	YGR078C	PAC10
YPR131C	NAT3	YLR079W	SIC1
YFR010W	UBP6	YHR156C	LIN1
YNL031C	HHT2	YBR009C	HHF1
YOR109W	INP53	YDL077C	VAM6
YNL291C	MID1	YDR320C	SWA2
YML016C	PPZ1	YGR270W	YTA7
YJR031C	GEA1	YDL226C	GCS1
YGL174W	BUD13	YDR440W	DOT1
YLR234W	TOP3	YLR381W	CTF3
YGR108W	CLB1	YPL253C	VIK1
YBR200W	BEM1	YDR137W	RGP1

Predictions at threshold level 0.80 continued...			
Gene1 of predicted pair		Gene2 of predicted pair	
Systematic Name	Standard Name	Systematic Name	Standard Name
YGL147C	RPL9A	YFR010W	UBP6
YAL024C	LTE1	YML001W	YPT7
YDR176W	NGG1	YBL003C	HTA2
YBR041W	FAT1	YBR082C	UBC4
YGR072W	UPF3	YDR108W	GSG1
YER162C	RAD4	YNL051W	COG5
YOR337W	TEA1	YFR036W	CDC26
YFL023W	BUD27	YMR230W	RPS10B
YKL048C	ELM1	YPL152W	RRD2
YGR184C	UBR1	YHR013C	ARD1
YOL062C	APM4	YJR058C	APS2
YDR254W	CHL4	YIR005W	IST3
YGL115W	SNF4	YDR153C	ENT5
YJL117W	PHO86	YGR135W	PRE9
YBR057C	MUM2	YML115C	VAN1
YOR132W	VPS17	YBR114W	RAD16
YBR057C	MUM2	YDR260C	SWM1
YBR108W	YBR108W	YMR127C	SAS2
[truncated: 371,743 more chars]
